# Software-aided approach to investigate peptide structure and metabolic susceptibility of amide bonds in peptide drugs based on high resolution mass spectrometry

**DOI:** 10.1371/journal.pone.0186461

**Published:** 2017-11-01

**Authors:** Tatiana Radchenko, Andreas Brink, Yves Siegrist, Christopher Kochansky, Alison Bateman, Fabien Fontaine, Luca Morettoni, Ismael Zamora

**Affiliations:** 1 Pompeu Fabra University, Barcelona, Spain; 2 Lead Molecular Design, S.L, Sant Cugat del Vallés, Spain; 3 Pharma Research and Early Development, Roche Innovation Center Basel, F. Hoffmann-La Roche Ltd., Basel, Switzerland; 4 Pharmacokinetics, Pharmacodynamics, and Drug Metabolism, Merck & Co., Inc., West Point, Pennsylvania, United States of America; 5 Molecular Discovery Ltd, London, United Kingdom; National Research Council of Italy, ITALY

## Abstract

Interest in using peptide molecules as therapeutic agents due to high selectivity and efficacy is increasing within the pharmaceutical industry. However, most peptide-derived drugs cannot be administered orally because of low bioavailability and instability in the gastrointestinal tract due to protease activity. Therefore, structural modifications peptides are required to improve their stability. For this purpose, several *in-silico* software tools have been developed such as PeptideCutter or PoPS, which aim to predict peptide cleavage sites for different proteases. Moreover, several databases exist where this information is collected and stored from public sources such as MEROPS and ExPASy ENZYME databases. These tools can help design a peptide drug with increased stability against proteolysis, though they are limited to natural amino acids or cannot process cyclic peptides, for example. We worked to develop a new methodology to analyze peptide structure and amide bond metabolic stability based on the peptide structure (linear/cyclic, natural/unnatural amino acids). This approach used liquid chromatography / high resolution, mass spectrometry to obtain the analytical data from *in vitro* incubations. We collected experimental data for a set (linear/cyclic, natural/unnatural amino acids) of fourteen peptide drugs and four substrate peptides incubated with different proteolytic media: trypsin, chymotrypsin, pepsin, pancreatic elastase, dipeptidyl peptidase-4 and neprilysin. Mass spectrometry data was analyzed to find metabolites and determine their structures, then all the results were stored in a chemically aware manner, which allows us to compute the peptide bond susceptibility by using a frequency analysis of the metabolic-liable bonds. In total 132 metabolites were found from the various *in vitro* conditions tested resulting in 77 distinct cleavage sites. The most frequent observed cleavage sites agreed with those reported in the literature. The main advantages of the developed approach are the abilities to elucidate metabolite structure of cyclic peptides and those containing unnatural amino acids, store processed information in a searchable format within a database leading to frequency analysis of the labile sites for the analyzed peptides. The presented algorithm may be useful to optimize peptide drug properties with regards to cleavage sites, stability, metabolism and degradation products in drug discovery.

## Introduction

During the last two decades, interest in peptide therapeutics has increased in pharmaceutical research and development. Peptides are generally thought to be well-suited for diseases where the target is a protein-protein interaction [1, 2]. They have great potential as new drugs due to high specificity to certain protein targets and higher selectivity, for example the peptide drug G-protein-coupled receptors (GPCR) [3–5]. Usually peptides also have a good safety profile and tolerability with high efficacy [1, 3–7].

Today, peptides represent only 2% of the worldwide drug market with approximately 140 peptide-based drugs-available and around 500 peptides in clinical development and preclinical drug discovery stages [3, 4]. Limited development of therapeutic peptides occurred in the past due to insufficient absorption, distribution, metabolism and elimination or excretion (ADME) properties: short half-life time, low permeability, low solubility, limited residence time in tissues. On one hand, low cell permeability is often related to structural factors such as high hydrogen bonding capacity and low lipophilicity [1]. On the other hand, low oral bioavailability is more frequently related to physiological processes, like low absorption and fast extraction through proteolysis, and pH dependent hydrolysis in blood, gastrointestinal tract, and liver [1, 3, 7, 8]. Therefore, peptides are usually administered through injection or delivered via non-oral routes such as transbuccal, nasal, inhaled or transdermal [1, 3, 4, 6–8]. Future successful drug peptides should have appropriate ADME properties.

Traditional structure-based peptide design methodologies include substitution of amino acids and the building of structure-activity relations (SAR) via experiments such as an alanine scan and estimation of the half maximal effective concentration (EC50). To achieve better ADME properties the following chemical modifications are typically applied: substitution of the common L-amino acids to D-amino acids or other unnatural amino acids, backbone N-methylation, alpha-methylation of amino acids, salt-bridge formation, lactam bridge formation, cyclization of the peptide, deamination, oxidation, isomerization and others [3, 7]. These changes are applied during the design-make-test drug discovery cycle, with hopes of improving the physicochemical and pharmacokinetics properties of the compound of interest. Therefore, it is crucial to evaluate these properties rapidly in early development.

Several analytical techniques are used when examining ADME properties including immunoassays, bioassays and high-performance liquid chromatography (HPLC) linked to mass spectrometry (MS). MS-based approaches are capable of efficient and reliable quantitative and qualitative analyses: peptide-parent loss over time and metabolite formation and identification. MS techniques combined with HPLC are the methods of choice for drug metabolism studies, when metabolites should be separated and identified with a high degree of certainty in complex biological matrices [9].

Since the task of metabolite characterization of peptides from MS data is very time-consuming, several semi-automated tools were developed for full scan/data-dependent MS/MS peptide data interpretation. These approaches include four main groups: database searching (SEQUEST, MASCOT, etc.), de novo peptide sequencing (PEAKS, PepNovo, etc.), peptide sequence tagging (GutenTag) and consensus of multiple search engines (Scaffold) [10]. These MS-based proteomics approaches have difficulties with sequencing cyclic peptides without prior linearization and they are limited to the 20 standard amino acids [6, 14, 15].

Recently, information was collected for about 500–600 human proteases in total and about 300–400 that are functional in the human body [14, 16, 17]. High-resolution crystallographic structures are publicly available for over 150 distinct human proteases. MEROPS [11], CutDB [12] and ExPASy ENZYME database [13] integrate available information about proteolytic sites and, consequently, about proteases, their cleavage sites, substrates and inhibitors. This information can be used to identify possible labile residues in the candidate peptide for individual proteases. Most of the information in these databases is limited to 20 standard amino acids [14]. Several computational bioinformatics sequence-based and matrix pattern approaches were developed to identify the most possible protease and/or the labile residues and the most likely cleavage site for the studied protein/peptide. However, these tools (SitePrediciting [18], PROSPER [19], PoPS [20], PeptideCutter [21]) are based on available literature or rely on the MEROPS database and therefore are limited to the 20 standard amino acids [14] and thus do not properly cover unnatural amino acids and cyclic peptides.

This article presents a new approach that uses LC-MS data from peptide metabolic stability experiments to determine the specific metabolic cleavage sites and then store the results in a chemically aware database, where chemical structure based searches can be performed by structure and/or fragments. This approach includes a new search algorithm applied for the mentioned database. Finally, this methodology can be used to perform frequency analysis to discover the most frequent metabolically labile amide bonds within this experimentally derived database, enabling the match of chemical peptide structures.

## Materials and methods

### Experimental data

#### Dataset

Metabolite identification was performed using two different peptides sets and experimental conditions. The first set (dataset-1) included ten commercially available peptides (secretin, calcitonin, oxytocin, octreotide, deslorelin, histrelin, goserelin, buserelin, leuprolide and gonadorelin) and four marker substrate peptides with known cleavage sites as positive controls for each of the selected proteases—trypsin, chymotrypsin, pancreatic elastase and pepsin Table 1. Five out of the ten compounds had unnatural amino acids and three of them were cyclic peptides. Moreover, to investigate the effect of small chemical/monomer changes in the peptide structure with respect to the proteases catalyzed reactions, the set was selected to also contain five synthetic analogues for the same peptide series, the luteinizing-hormone releasing hormone (LHRH). All test compounds were prepared as a stock at a concentration of 10 mM in dimethyl sulfoxide (DMSO).

**Table 1:** Peptide-substrates structures and other characteristics.

Drug Name	Provider	Molecular weight	Structure	Sequence
**Dataset 1**				
**Linear peptides**				
***Gonadotropin releasing hormone and analogues***				
**Gonadorelin**	BioNet HS2014	1182.29	linear	H-Pyr-His-Trp-Ser-Tyr-Gly-Leu-Arg-Pro-Gly-NH2
**Deslorelin**	BioNet HS2009	1282.45	linear	H-Pyr-His-Trp-Ser-Tyr-D-Trp-Leu-Arg-Pro-NHEt
**Goserelin**	Sigma G4919	1269.41	linear	Glp-His-Trp-Ser-Tyr-Ser-tBu-Leu-Arg-Pro-NHNHCONH2
**Buserelin**	Sigma B3303	1238.66	linear	Glp-His-Trp-Ser-Tyr-Ser-tBu-Leu-Arg-Pro-NHEt
**Histrelin**	Sigma L2761	1323.5	linear	Glp-His-Trp-Ser-Tyr-HisBzl-Leu-Arg-Pro-NHEt
**Leuprolide**	Sigma L0399	1209.4	linear	Glp-His-Trp-Ser-Tyr-D-Leu-Leu-Arg-Pro-NHEt
***Other peptides***				
**Secretin human**	Sigma S7147	3039.41	linear	H-His-Ser-Asp-Gly-Thr-Phe-Thr-Ser-Glu-Leu-Ser-Arg-Leu-Arg-Glu-Gly-Ala-Arg-Leu-Gln-Arg-Leu-Leu-Gln-Gly-Leu-Val-NH2
**Cyclic peptides**				
**Octreotide**	Sigma O1014	1019.24	cyclic	H-D-Phe-Cys(1)-Phe-D-Trp-Lys-Thr-Cys(1)-Thr-ol
**Oxytocin**	Sigma O6379	1007.19	cyclic	H-Cys(1)-Tyr-Ile-Gln-Asn-Cys (1)-Pro-Leu-Gly-NH2
**Calcitonin**	Sigma T3660	3429.71	cyclic	H-Cys(1)-Ser-Asn-Leu-Ser-Thr-Cys (1)-Val-Leu-Gly-Lys-Leu-Ser-Gln-Glu-Leu-His-Lys-Leu-Gln-Thr-Tyr-Pro-Arg-Thr-Asn-Thr-Gly-Ser-Gly-Thr-Pro-NH2
**Control substrate peptides**				
**Ala-Ala-Phe-7-amido-4-methylcoumarin**	Sigma A3401	464.52	linear	H-DL-Ala-DL-Ala-DL-Phe-AMC
**N-methoxysuccinyl-Ala-Ala-Pro-Val-7-amido-4-methylcoumarin**	Sigma M9771	627.69	linear	MeoSuc-Ala-Ala-Pro-Val-AMC
**N-Benzoyl-L-isoleucyl-L-glutamyl-glycyl-L-arginine-4-nitroanilide**	Sigma 87528	697.74	linear	Bz-Ile-Glu-Gly-Arg-pNA
**Phe-Ala-Ala-Phe(4-NO2)-Phe-Val-Leu(4-pyridylmethyl) esther**	Sigma 77431	950.09	linear	Phe-Ala-Ala-Phe(4-NO2)-Phe-Val-Leu(4-pyridylmethyl) esther
**Dataset 2**				
**Linear peptides**				
***Glucagon like protein-1 and analogues***				
**Glucagon-Like 1 protein**	Sigma G9416	3297.68	linear	H2N-His-Ala-Glu-Gly-Thr-Phe-Thr-Ser-Asp-Val-Ser-Ser-Tyr-Leu-Glu-Gly-Gln-Ala-Ala-Lys-Glu-Phe-Ile-Ala-Trp-Leu-Val-Lys-Gly-Arg- Gly-OH
**Liraglutide**	Bachem AG H-6724	3749.95	linear	H-His-Ala-Glu-Gly-Thr-Phe-Thr-Ser-Asp-Val-Ser-Ser-Tyr-Leu-Glu-Gly-Gln-Ala-Ala-Lys(γ-Glu-palmitoyl)-Glu-Phe-Ile-Ala-Trp-Leu-Val-Arg-Gly-Arg-Gly-OH
**Taspoglutide**	Pharmten Chemical Ltd. PTN3367	3338.71	linear	H-His-Aib-Glu-Gly-Thr-Phe-Thr-Ser-Asp-Val-Ser-Ser-Tyr-Leu-Glu-Gly-Gln-Ala-Ala-Lys-Glu-Phe-Ile-Ala-Trp-Leu-Val-Lys-Aib-Arg-NH_2_
**Exenatide**	Chemie Brunschwig E957300	4184.0	linear	H-His-Gly-Glu-Gly-Thr-Phe-Thr-Ser-Asp-Leu-Ser-Lys-Gln-Met-Glu-Glu-Glu-Ala-Val-Arg-Leu-Phe-Ile-Glu-Trp-Leu-Lys-Asn-Gly-Gly-Pro-Ser-Ser-Gly-Ala-Pro-Pro-Pro-Ser-NH_2_

Metabolite identification following incubation with select proteases, was performed for a second set of peptides (dataset-2) which consisted of four commercially available peptides: glucagon-like peptide-1 (GLP-1) and three synthetic analogues of GLP-1—taspoglutide, exenatide and liraglutide (Table 1). Taspoglutide peptide had non-natural amino acids and liraglutide had C-16 fatty acid side chain (palmitic acid). GLP-1, taspoglutide and exenatide were prepared as a stock in concentration of 10 mM and liraglutide was prepared in concentration of 5 mM in DMSO.

#### Incubations

Incubations with dataset-1 were performed using isolated enzymes. We investigated serine proteases like trypsin, chymotrypsin, and pancreatic elastase (E 3.4.21.1, E 3.4.21.4, E 3.4.21.36) at a concentration of 500 μg/mL in simulated intestinal fluid (SIF) and the aspartic protease pepsin (E 3.4.23.1) at a concentration of 500 μg/mL in simulated gastric fluid (SGF). Proteases were purchased from Sigma Aldrich and SIF and SGF were purchased from RICCA Chemical Company (Arlington, TX, USA). For dataset-2 incubations proteases with different catalytic mechanisms were employed. The serine protease dipeptidyl peptidase-4 (DPP-4) (E 3.4.14.5) was incubated at 2 μg/mL concentration and the zinc-dependent metalloprotease like neprilysin (NEP) (E 3.4.24.11) was also incubated at 2 μg/mL concentration in Hank's buffered salt solution. All incubations were conducted at 37°C. More detailed information regarding incubation conditions is provided in S1 Table.

The dataset-1 incubations were performed at Merck Research Laboratories (Merck & Co, West Point, PA, USA). Negative control samples were prepared under the same conditions of the incubation (see above), containing each enzyme but without adding probe compounds. Probe compounds were added to 96-well plates (Micronic 0.75 mL V-bottom) using a Hewlett Packard HPD300e. To give a 10-μM concentration for a 50 μL incubation per well, 50 nL of each compound was dispensed. Each well was a time point, with five timepoints (0, 5, 15, 45 and 120 min) per compound per protease. Incubations were carried out using a Hamilton MicroLab STAR Plus liquid-handler to initiate, conduct and stop incubation reactions within a single automated method. The incubation plates were placed in heated shakers present on the deck of the liquid-handler and operated at 37°C and 400 rpm shaking speed. Reactions were started with the addition of the enzyme at the appropriate time to allow for all reactions to be quenched at a single time. Incubation quenching was again carried out by the Hamilton STAR Plus with the addition of 100 μL of acetonitrile with 1% formic acid (FA) or 1% ammonium hydroxide (pepsin-only) and the internal standard (melanotan) at a concentration of 1 μM. Following reaction quenching, the samples were vortexed for approximately 2 min and the time zero sample was generated by aliquoting 50 nL of the appropriate stock compound into quenched control samples. The samples were then centrifuged in a Beckman Allegra 25R at 6000 rcf for 20 min at 10°C. Resulting supernatant was transferred to Eppendorf LoBind 96-well plates and 1 μL was injected onto a Waters Acquity BEH-C18 1 x 50 mm, 1.7 μm column via a Waters Acquity M-Class ultra-performance liquid chromatography (UPLC) autosampler. All time points were analyzed using a data-dependent MS/MS method. Full scan/data-dependent MS/MS experimental settings are provided in S2 Table.

The dataset-2 peptide incubations were performed at Roche Innovation Center Basel F. Hoffmann-La Roche Ltd., (Hoffmann-La Roche, Basel, Switzerland) using isolated enzymes. For both DPP-4 and NEP incubations, a solution of the respective enzyme in Hank’s buffered salt solution was pre-incubated at 37°C for 3 to 5 min, in 96-well plate Nunc (Thermo Scientific, 163320) before the addition of 30 μL of test substance to give final concentrations of 2 μg/mL DPP-4 or 2 μg/mL NEP. Negative control samples were prepared under the same conditions (see above), containing each enzyme but without adding test compound. Samples for taspoglutide, liraglutide and exenatide were incubated at 37°C for 2, 4, 8, and 24 hr and for GLP-1 for 0, 5, 15, 30 and 60 min. The incubation plates were placed in heated shakers present on the deck of the liquid-handler and operated at 37°C and 600 rpm shaking speed. Reactions were started with the addition of the enzyme and then quenched at each time point with 75 μL cold acetonitrile containing 1% FA followed by thorough mixing. The samples were centrifuged at 4°C for 15 min at 14,000g, and the supernatant was collected. The metabolite profile was immediately analyzed. Full scan/data-dependent MS/MS experimental settings are provided in S2 Table.

#### UPLC-MS/MS

For dataset-1, chromatographic separation of metabolites was performed using the ACQUITY UPLC system (Waters, Milford, MA, USA). The ACQUITY UPLC BEH C18 column (1.0 × 50 mm, 1.7 μm) was heated to 60°C. The mobile phase consisted of 0.1% FA in water (eluent A) and 0.1% formic acid in acetonitrile (eluent B) at a flow rate of 75 μL/min. The initial condition was 10% eluent B, which was maintained for 0.1 min. Eluent B was then increased via a linear gradient to 40% until 3.1 min and further increased to 90% via linear gradient to 3.6 min. Eluent B was then held at 90% until 4.0 min when it was ramped down to 10% by 4.05 min and held until the end of the run at 6 min. ACQUITY UPLC system experimental settings are provided in S3 Table. Full scan/data-dependent MS/MS analyses were run on a Thermo Scientific Q-Exactive Plus mass spectrometer operated in positive electrospray ionization (ESI) mode. The MS and MS/MS acquisition scans were operated with a resolution setting of 17,500 for both, with an automatic gain control (AGC) setting of 1E6 and maximum injection time of 100 ms for MS and AGC of 2E5 and max injection time of 50 ms for full scan/data-dependent MS/MS. Lock masses were employed using background phthalate ions with exact masses of 391.2848, 419.3161, and 447.3474. Full scan MS/MS was a data dependent acquisition (DDA) using peptide-specific inclusion lists containing amide hydrolysis ions of multiple charge states (z = 1 to 3). These lists were generated using a MOL file for each peptide and the software Mass-MetaSite 5.1.5. The DDA method settings employed a chromatographic peak width of 4 s with an apex trigger between 1.5–5 s, under fill ratio of 3.0%, charge exclusion of 6–8 and > 8, peptide matching preferred, isotopes excluded, dynamic exclusion of 4 s.

To generate dataset-2, a Thermo Scientific Dionex UltiMate 3000 RS UPLC system in combination with a Pal autosampler (CTC Analytics AG, Zwingen, Switzerland) was used. Chromatographic separation was performed using an Acquity CSHT Phenyl-Hexyl Column (1.7 μm, 2.1 x 100 mm). The mobile phases consisted of A: H_2_O/0.1% FA and B: methanol/0.1% FA. Start condition was 5% B at a flow rate of 0.4 mL/min, the LC gradient started at 1 min and was increased at 10 min to 100%. ACQUITY UPLC system experimental settings are provided in S4 Table. Full scan/data-dependent MS/MS analyses were run on a Q Exactive^™^ Hybrid Quadrupole-Orbitrap Mass Spectrometer (Thermo Fisher Scientific) operated in positive ESI mode. Spray voltage was 3.5 kV, the collision energy was set to 25 V. Full scan MS/MS was a DDA using peptide specific inclusion lists containing amide hydrolysis ions of multiple charge states (z = 1 to 5). These lists were generated using a MOL file for each peptide and the software Mass-MetaSite 5.1.5. The post-acquisition data analyses were performed using the peptide specific mode integrated in Mass-MetaSite 5.1.5 Mass 3.2.3 software (Molecular Discovery Ltd, Middlesex, UK). Minimal dataset containing raw files for all the incubations is available on the BioStudies repository (https://www.ebi.ac.uk/biostudies/). Submission number is S-BSST33.

### Data processing

**Fig 1:**
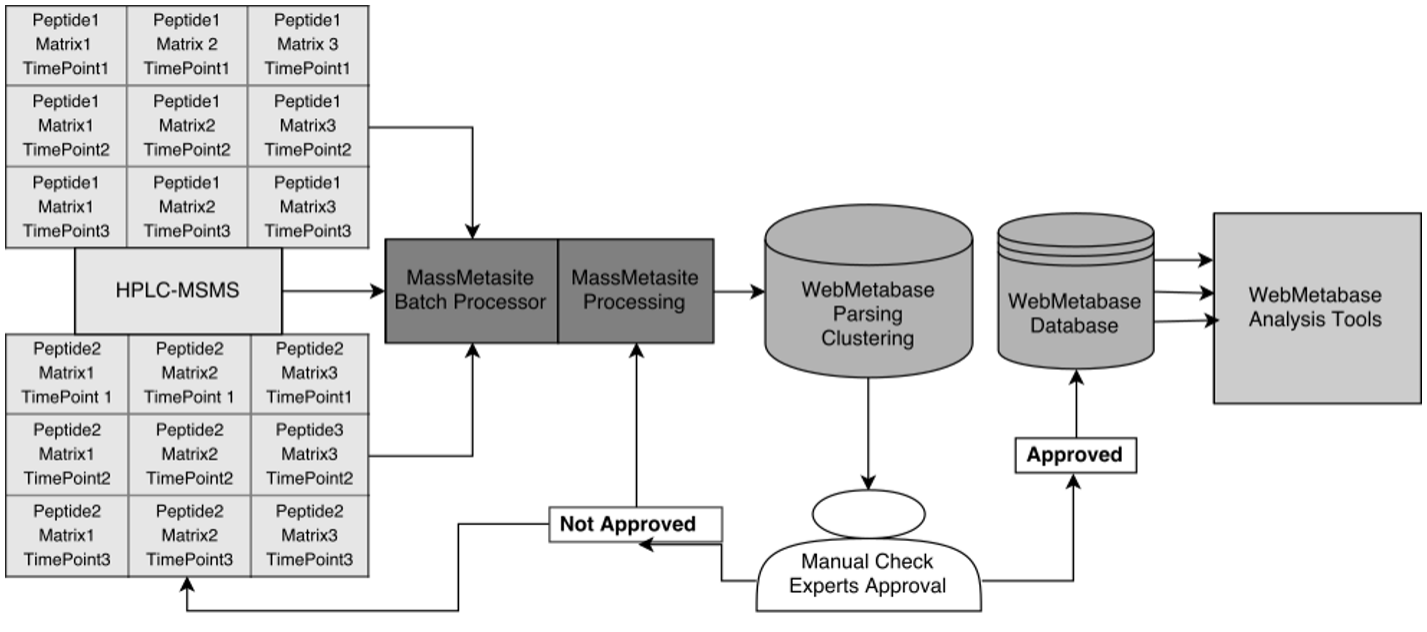
Mass-Metasite/WebMetabase workflow from experimental data to searchable information manageable by *in silico* analysis tools.

All data acquired from the LC/MS system were processed using MetaSite 5.1.5. The MetaSite-Batch Processor was used to process data without supervision. The produced output was automatically uploaded into the web application “WebMetabase 3.2.7” (Molecular Discovery Ltd, Middlesex, UK), where all the samples from the same experiment were clustered together for further analysis and interpretation. In WebMetabase the detected chromatographic peaks were displayed together with the structural elucidation data for parent and metabolites. The Mass-MetaSite/WebMetabase workflow used is shown in Fig 1.

The Mass-MetaSite settings used for the MetaSite-Batch Processor and required to reproduce the results are reported in S5 Table. The sample list used for the batch was generated in WebMetabase mirroring the experimental design (enzymes, time points and instrument) and defined as a WebMetabase protocol. Settings used for the WebMetabase protocol are given in S6 Table.

#### Mass-MetaSite

The application of Mass-MetaSite for the interpretation of small molecules metabolic stability data has been described previously [22–28]. Here, we will point out the main processing parameters differences for the case of peptides with respect to the previously described small molecule parameters.

Mass-MetaSite uses as inputs the 2D structure of the compound together with control and treated sample data files (Fig 1). The data can be processed sample-by-sample manually or in a batch mode with an automatic processing of a set of sample files. The data processing consists of two steps. Step-1 consists of automatic detection of the chromatographic peaks related to the parent compound, i.e. metabolites. The methodology for peptides does not differ from the one described for small molecules [25]. Step-2 consists of structure elucidation of the potential metabolites based on the fragmentation pattern for each peak detected. Once the list of potential chromatographic peaks has been selected (step-1), Mass-MetaSite compares the *m/z* associated with each peak to all the possible theoretical metabolites based on a list of included biotransformation reactions [25]. In this study, the only transformation of interest selected was the hydrolysis of amide bonds. Mass-Metasite then generates all possible metabolites based on a predefined list of metabolic biotransformation reactions.

The overall principle for the structural elucidation of metabolites is a comparison of fragment ions obtained from the parent (assigned from the incubation time t = 0 sample) and the ones from the metabolites (t = incubation time) and then identifying mass shifts corresponding to the mass of the metabolite or common neutral losses [24]. In addition, for peptides, an extra bond/breaking rule was added and carbon-carbon bonds having the same hybridization are not broken, mainly to optimize the computational speed since the number of fragments increases exponentially with the number of broken bonds. The maximum number of bonds broken was set to 2 to also help reduce the computational time.

In addition to the above comparative fragmentation analysis, the fragmentation of the metabolite without comparison to the parent molecule is considered. This fragmentation strategy is most advantageous in the case of cyclic peptides where the metabolite could be a linear peptide (the amide hydrolysis is occurring in and opening the cycle) and fragmentation can be significantly different compared to parent. Fragmenting all the metabolite structures to the same extent as the substrate takes a prohibitive amount of computational time; therefore, the number of bonds that can be broken to generate metabolite fragments has been limited to 1.

A score is assigned to each peptide metabolite based on the number of matches/mismatches between the theoretical fragment *m/z* value and the *m/z* value observed in the MSMS spectrum as it has been described for small molecules [28]. Once Mass-MetaSite results have been uploaded into the WebMetabase, they should be manually checked and approved by the expert (Fig 1).

#### WebMetabase

All WebMetabase experimental settings are reported in S6 Table. Each experiment consisted of a set of samples, i.e. one sample per incubation time point per protease. Mass-MetaSite processes every sample file as a separate unit and thus generated three main pieces of information for each sample: metabolic scheme, spectrometry data (structural fragment assignment) and chromatogram (retention time, MS area, MS relative area and ppm) for each structure, both substrate and metabolites. Afterwards, WebMetabase consolidates all these data from the individual files in a single interpretation for the entire experiment (time/protease) and analyze which metabolite peaks from each sample can be grouped based on the retention time and *m/z*. Retention time tolerance and *m/z* tolerance are shown in S6 Table. These consolidated substrates and metabolites are used for the following analysis.

**Fig 2:**
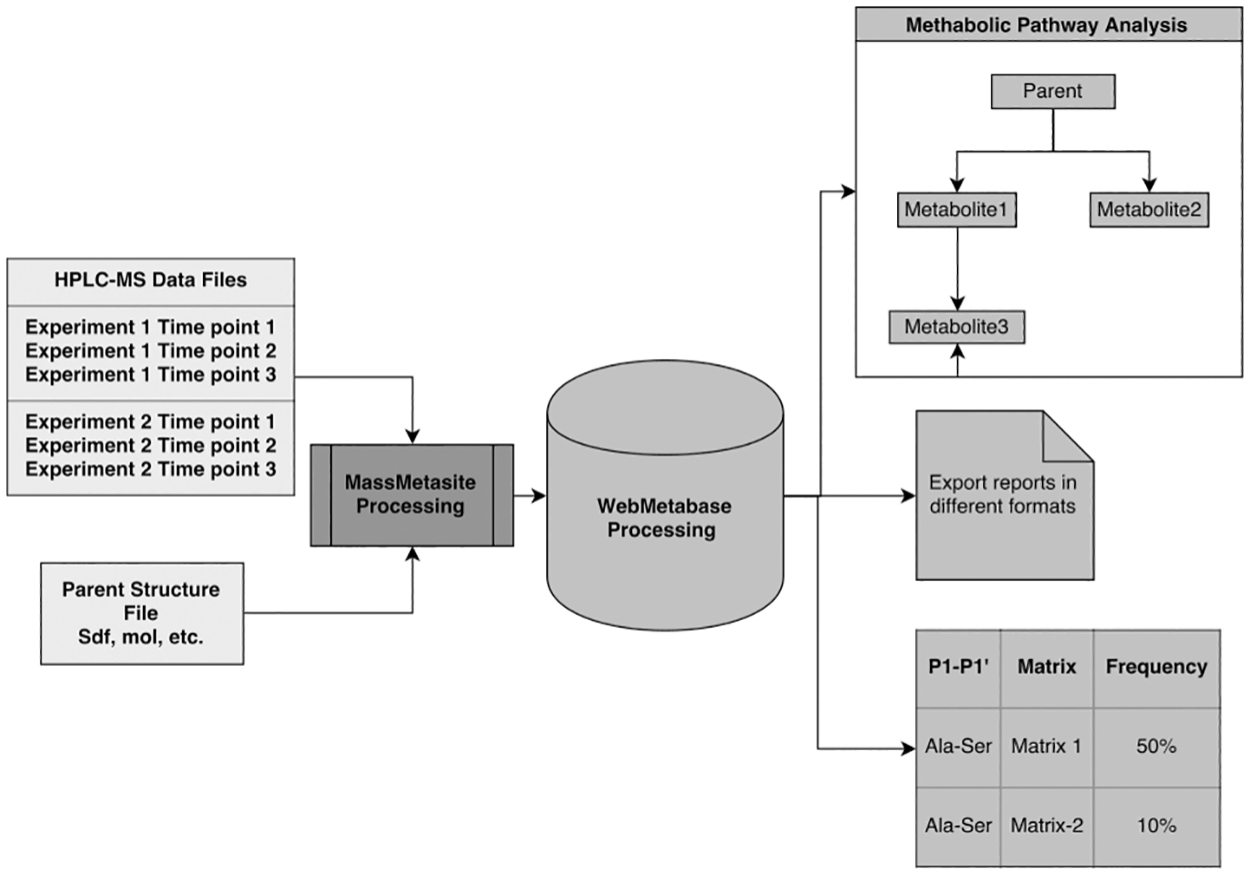
The Mass-MetaSite/WebMetabase workflow with *in silico* analysis tools available for the peptides data processing.

A new algorithm was introduced into WebMetabase to store information about peptide in a chemically aware searchable format, including a system to perform searches based on matches of chemical structure. Also, this approach can perform frequency analysis to understand the most metabolically labile amide bonds in peptides towards the specific proteases/specific media (Fig 2).

#### Peptide database and search algorithm

**Fig 3:**
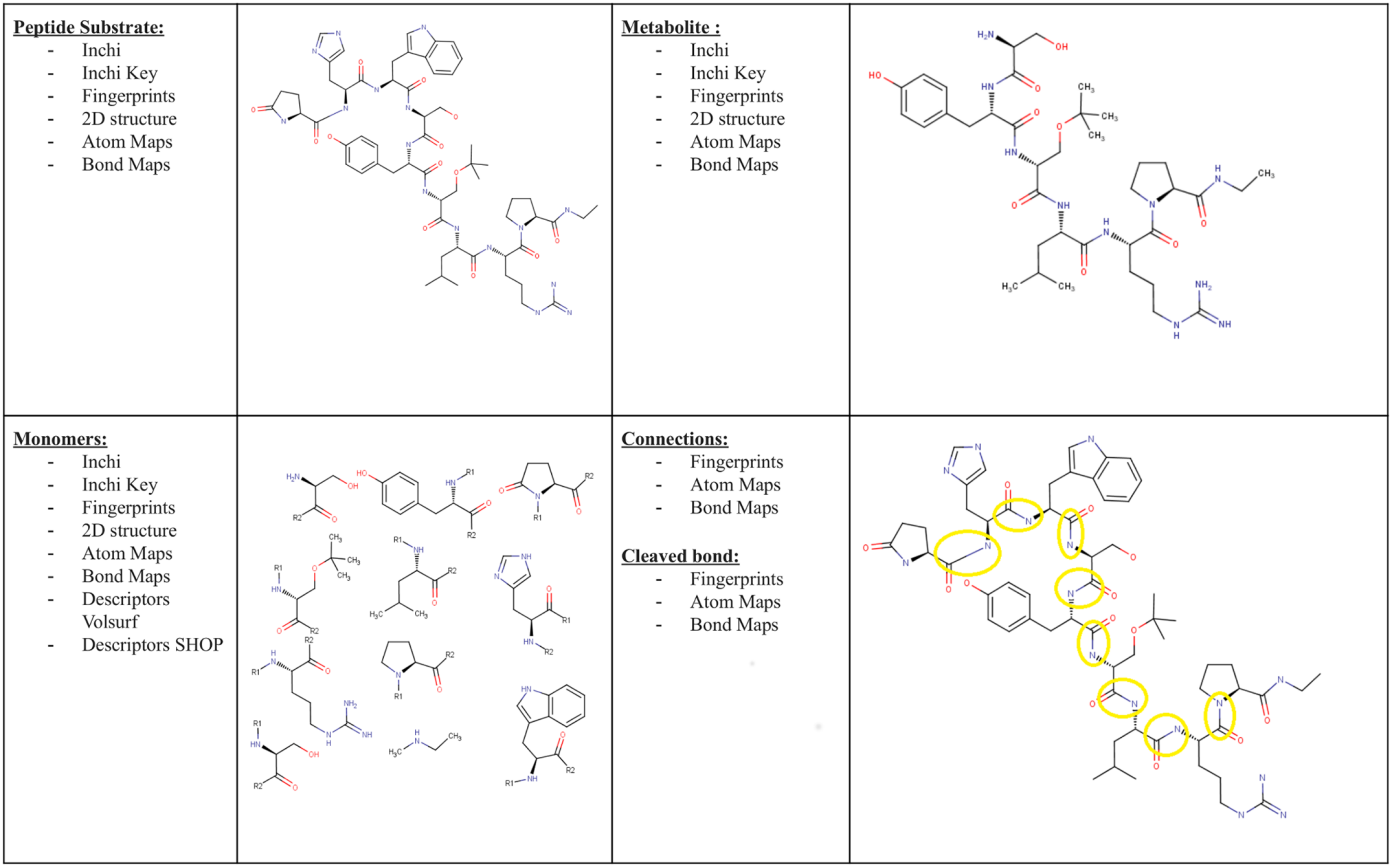
Peptide’s parent and metabolite structure annotation in the database.

Once the experimental results are interpreted and approved in WebMetabase, the peptide and metabolite structures are stored in the database. Each peptide structure is annotated by the structural blocks (SB) between amide bonds and their connectivity. Only the amide bonds are assumed to be broken to form SB and in this way a SB can contain more than one amino acid, for example cyclic peptides where amino acids can be connected through disulfide bridges, in peptides-conjugates, or with other bond types between amino acids. SBs are interpreted as different structures depending on chirality (L, D or undefined) and position in the peptide (N-terminal, C-terminal, in the middle). For each SB, additional information is stored in the database such as InchiKey and Inchi, 2D structure, atom mapping and connectivity, physico-chemical properties and pharmacophoric properties described through molecular descriptors calculated by VolSurf [29] and SHOP software [30] (Fig 3). Each connection of SBs is annotated by two connected structural blocks, bond type and two connected atoms which help to identify the direction of the connection. Moreover, in the case of the metabolites additional information is computed related to the cleaved bonds and incubation protease. The annotation of the peptide information in this manner enables doing a chemically aware substructure search inside the database and is not limited to any type of peptides (cyclic/linear, natural/synthetic). It is important to highlight that the developed search algorithm does not consider the theoretical mass spectrum or even sequence alignment, but rather performs an exact chemically aware search of parent’s and its metabolites SB structure.

Searches can be performed for exact sequence of the SBs. This type of search is treated as a chemical structure and therefore the target sequence should be used as an input with specific chirality and specified connection points. The algorithm provides an output of a list of experiments that contains the chemical substructure (searched sequence) in the parent compound. Also, the methodology can be used to perform a structural search of cleaved bonds. In this way, the developed system can identify experiments where a certain bond of interest was involved in a metabolic reaction. In this case, the searched sequence and marked bond should be used as an input. The output of this type of search is a list of experiments where the searched bond between two monomers was cleaved.

#### Peptide frequency analysis

A peptide frequency analysis of the cleaved amide bonds can be performed for the entire database or for the selected set of the approved experiments in WebMetabase. The algorithm collects information about all cleaved peptide bonds that were involved in the metabolic reactions of interest. Herein site of cleavage (SoC) considers the two SBs involved in the amide cleavage containing the C-terminal and N-terminal of the SBs involved. We define a potential SoC (pSoC) as the pair of structural blocks that may and may not be involved in the catalysis. The frequency analysis refers to the number of times that a pSoC is observed in the parent structure and how many times this is an actual SoC.

There are a number of ways to count the different SoCs, and so the frequency calculation follows certain rules: a) if the same pSoC was cleaved to generate different metabolites for the same parent peptide (i.e. if a metabolite is further metabolized) it will be counted as one. b) if the same actual SoC was identified in the same parent/protease from different experimental occasions (i.e. experimental replication) it is calculated as one. c) if the pSoC was found in a parent peptide at more than one location, for each actual SoC found the frequency is computed separately and finally added up.

The output of the frequency analysis is done by protease and provides two tables. The first table contains the number of times that the pSoC was found and the number of times it was an actual SoC. The second table contains the number of times that the pSoC was found and if it was cleaved once, twice or more than twice. A frequency analysis of the actual SoC depending on the protease can be done to create a set of empirically derived rules that later could be used to predict the metabolic liability of different amide bonds in a new non-tested peptide.

## Results and discussion

Here, we present experimental results of applying our approach and algorithm for the analysis of the two peptide datasets. First, Mass-MetaSite processes the DDA HRMS data and finds chromatographic peaks related to the parent and then elucidates the metabolites structure based on this HRMS data. Second, these results were uploaded to WebMetabase followed by consolidation of all these data (cluster metabolites from different experimental conditions, i.e. incubation times from the same experiment). This consolidated data was used for further analysis, i.e. evaluation of the kinetics of the parent peptide and metabolites. After approval of the experiments, parent and metabolite structures were stored in the database. Finally, the methodology can be used to perform an analysis on the information stored including frequency analysis of the metabolized bonds. The frequency analysis results were used to identify the most metabolically labile SoC in the studied peptide sets and protease.

Metabolite identification was performed on the fourteen commercial peptide compounds and the four positive substrates for the selected proteases that were incubated with digestive serine, aspartic proteases and a metalloprotease. The compounds were structurally diverse due to linear and cyclic structure, containing natural and unnatural amino acids and a wide range of molecular weights (Table 1). The peptide mode of Mass-MetaSite was used to process UPLC-HRMS and UPLC-MS/MS data to detect chromatographic peaks and to assign the most probable metabolite structures of the tested peptides. As an example, we will discuss results of the metabolite identification for linear buserelin and cyclic oxytocin incubated in chymotrypsin for 120 minutes. Further results are presented in supporting information listed in SFiles.

### SFiles. Metabolite identification reports exported from WebMetabase for each of the tested peptides in each protease

**Fig 4:**
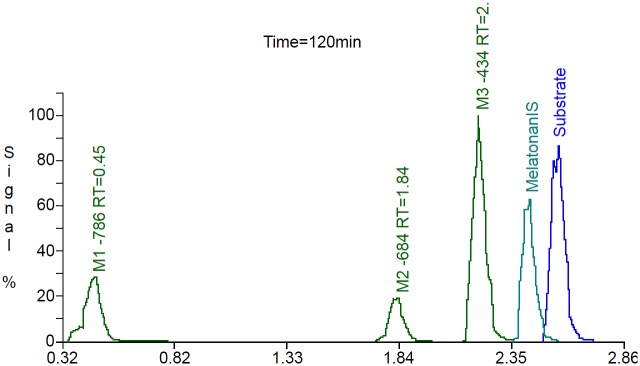
Extracted ion chromatogram of buserelin after 120 minutes of incubation with chymotrypsin. Blue peak—parent peptide compound;Green peaks—first generation of metabolites;Brown peaks- second generation or higher;Marine peak—internal standard;Red—structure was not elucidated automatically or peak was not found automatically.

**Fig 5:**
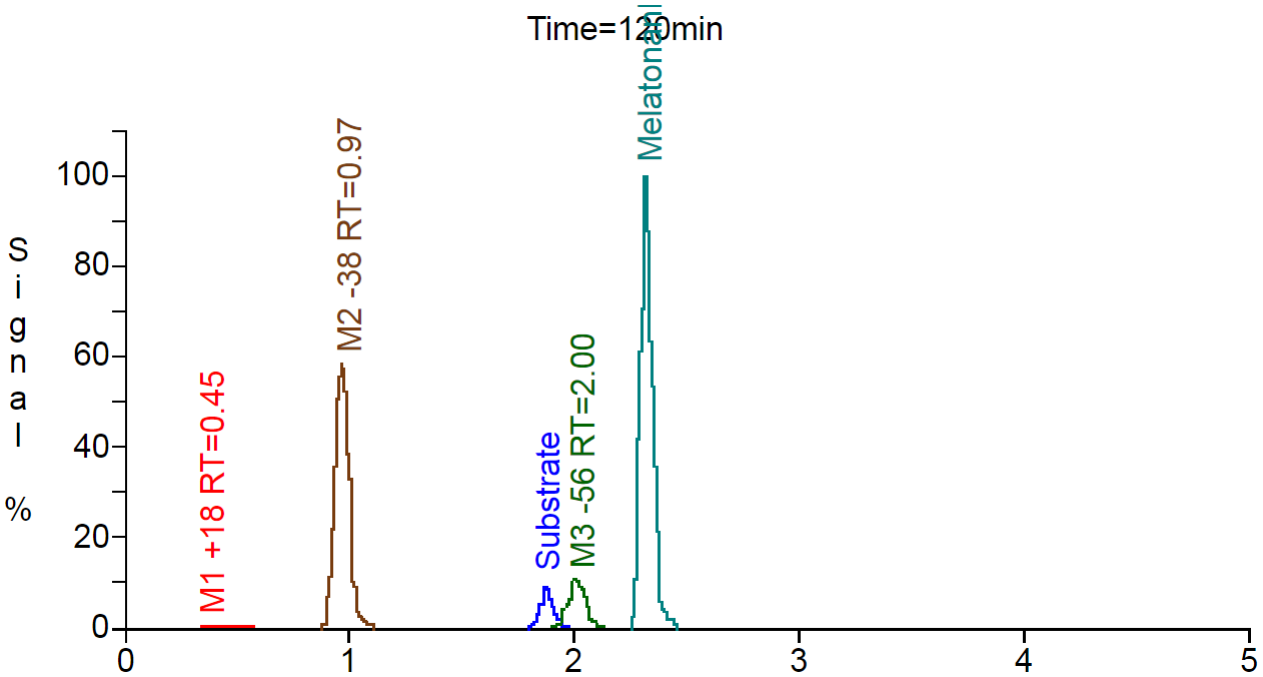
Extracted ion chromatogram of oxytocin after 120 minutes of incubation with chymotrypsin. Blue peak—parent peptide compound;Green peaks—first generation of metabolites;Brown peaks- second generation or higher;Marine peak—internal standard;Red—structure was not elucidated automatically or peak was not found automatically.

The results of the first step of peak detection using the Mass-MetaSite algorithm for the 120-minute chymotrypsin incubations with buserelin and oxytocin are shown in Figs 4 and 5. The metabolites listed in the figure are named by a shift in *m/z* (such as -786 or +18) with respect to the parent. For this example, buserelin yielded three metabolites peaks (M1-786, M2-684 and M3-434) with a retention time of 0.45, 1.84 and 2.12 minutes, respectively. For oxytocin, three metabolites were also identified, M-38 with retention time 0.97, M-56 with retention time 2.00 and M+18 with retention time 0.45 min. The computed *m/z* values of the identified metabolites agreed with the predicted values. Four of the metabolites (three from buserelin and one from oxytocin) correspond to first-generation products (from a single reaction) and are indicated by the green color of the peak. The one brown colored metabolite is indicative of multiple enzymatic reactions (2 or more) needed to reach the observed *m/z*. Though detected at earlier time points, one of the oxytocin metabolites was not automatically retrieved from the 120-min sample. In this case a manual extracted ion chromatogram was performed within WebMetabase to collect the peak information.

**Fig 6:**
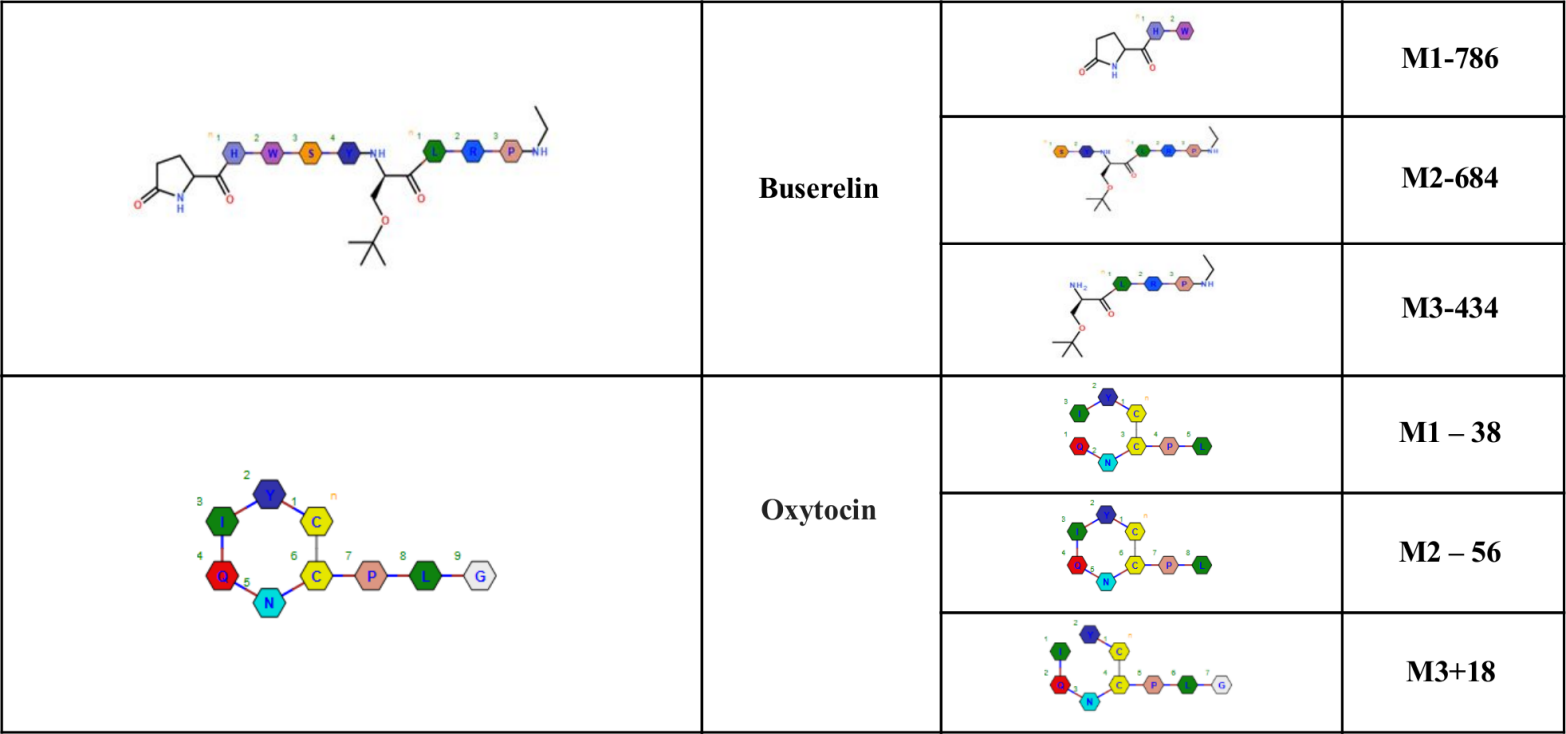
Proposed metabolites of buserelin and oxytocin found in 120 min incubations with chymotrypsin.

The second step of our algorithm assigns chemical structures to the identified metabolites. All buserelin and oxytocin metabolites have a similar fragmentation pattern as compared to the substrate fragmentation. The software predicts the theoretical fragments for the parent compound and metabolites, and compares them with experimentally obtained MS and MS/MS results. The metabolite fragment ions can have the same mass as a parent fragment or an expected mass shift, conserved and shifted, respectively. The assigned structures of the previously found metabolites for buserelin and oxytocin are presented in Fig 6.

**Fig 7:**
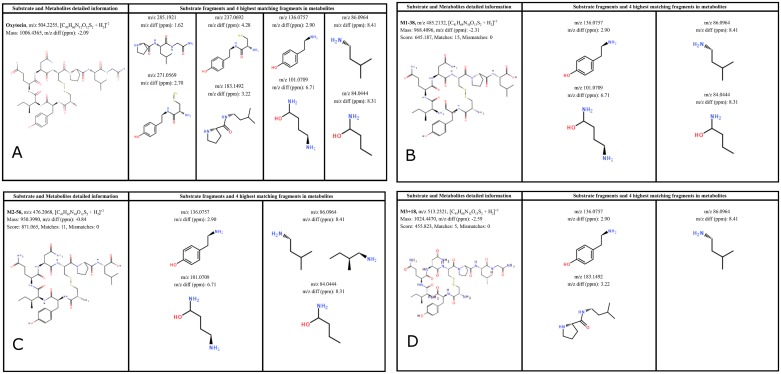
Full scan/data-dependent MS/MS fragment analysis of oxytocin substrate and metabolites. a) Oxytocin substrateb) Metabolite M1-38c) Metabolite M2-56d) Metabolite M3+18.

The structural assignments for oxytocin metabolites are shown in greater detail in Fig 7a–7d and are based on the fragment ions (ppm<10) that were detected in the substrate and metabolite spectra. In this figure, nine fragment ions were found that are compatible with the shown structure for oxytocin. For the M1-38 and M2-56 metabolites, four of the matching metabolite fragment ions with the highest full scan/data-dependent MS/MS signal intensity are shown. A score is calculated and reported for each metabolite. For the metabolites M1-38 and M2-56, the score was 645 with 15 matching fragments and 871 with 11 matching fragments, respectively. The structural assignment is believed to be reliable because of the high score, several matching fragments, no mismatching fragments, and a low m/z difference between the observed and computed values (<3 ppm). Other results are in supporting information listed in SFiles.

**Fig 8:**
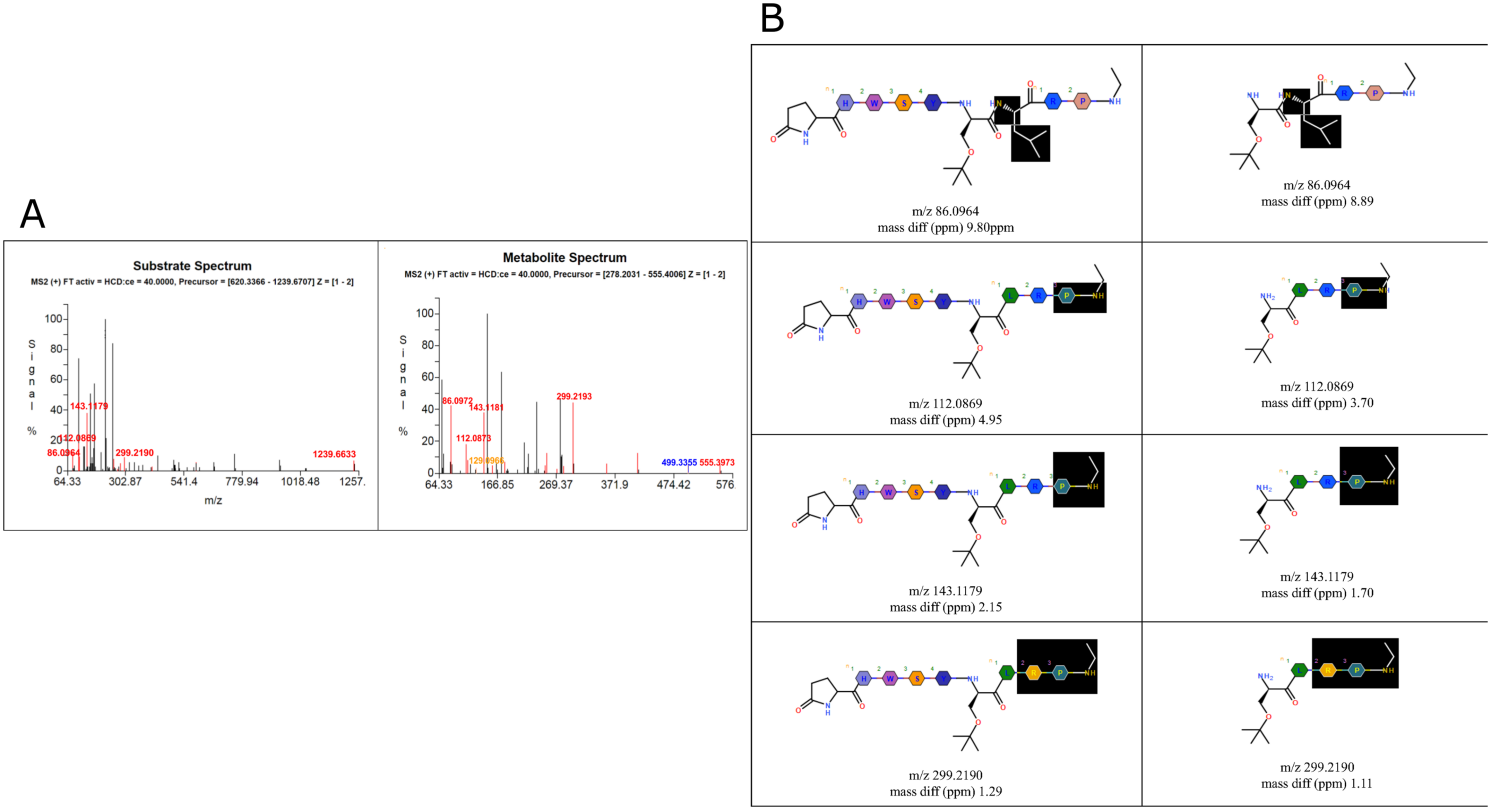
The fragmentation pattern for the M2-684 metabolite of buserelin in incubations with chymotrypsin 120 min. a) Full scan/data-dependent MS/MS spectra for the buserelin and M2-684b) Fragments structures
Red peaks—correlated with fragments that match between metabolite and fragment;Blue peaks—describe peaks correlated only with parent;Orange peaks—correlate only with metabolite.

Results from structure assignment of the buserelin metabolite M2-684 peak which elutes at 2.12 minutes are additionally shown in Fig 8a and 8b. Here, the full scan/data-dependent MS/MS spectra for the substrate buserelin and the metabolite M2-684 (score: 953, matches: 25, mismatches: 1) are shown along with the SB structures of the four highest matching fragments from substrate and metabolite, one metabolite fragment and one mismatching fragment. This metabolite structural assignment is reliable because the mass score is greater than 500, there are several matching fragments with only one mismatch, and the difference between the observed and theoretical or exact m/z was less than 3 ppm.

The previous algorithm processes one sample (protease or time point) at a time. However, an experiment typically has multiple time points and/or matrices. To be able to compare the multiple samples of an experiment, the metabolites from each sample should be grouped together with the metabolites from the other samples. We have termed this type of grouping “analysis clustering”.

**Fig 9:**
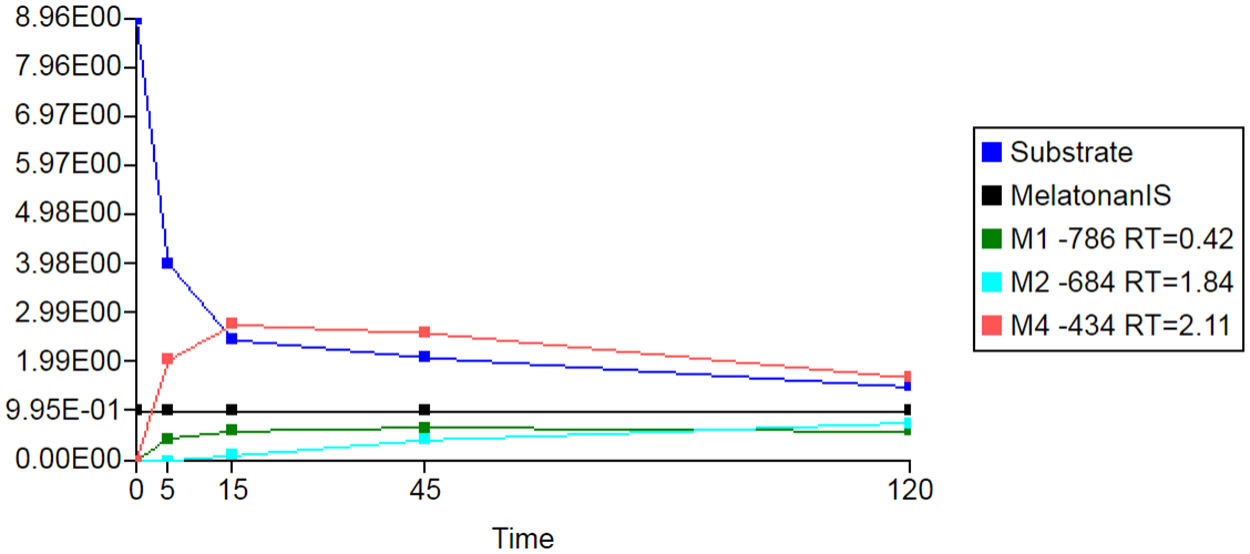
Peak area over time for buserelin, its metabolites and internal standard with chymotrypsin. X axis—MS Area, Y axis—Time (hr).

**Fig 10:**
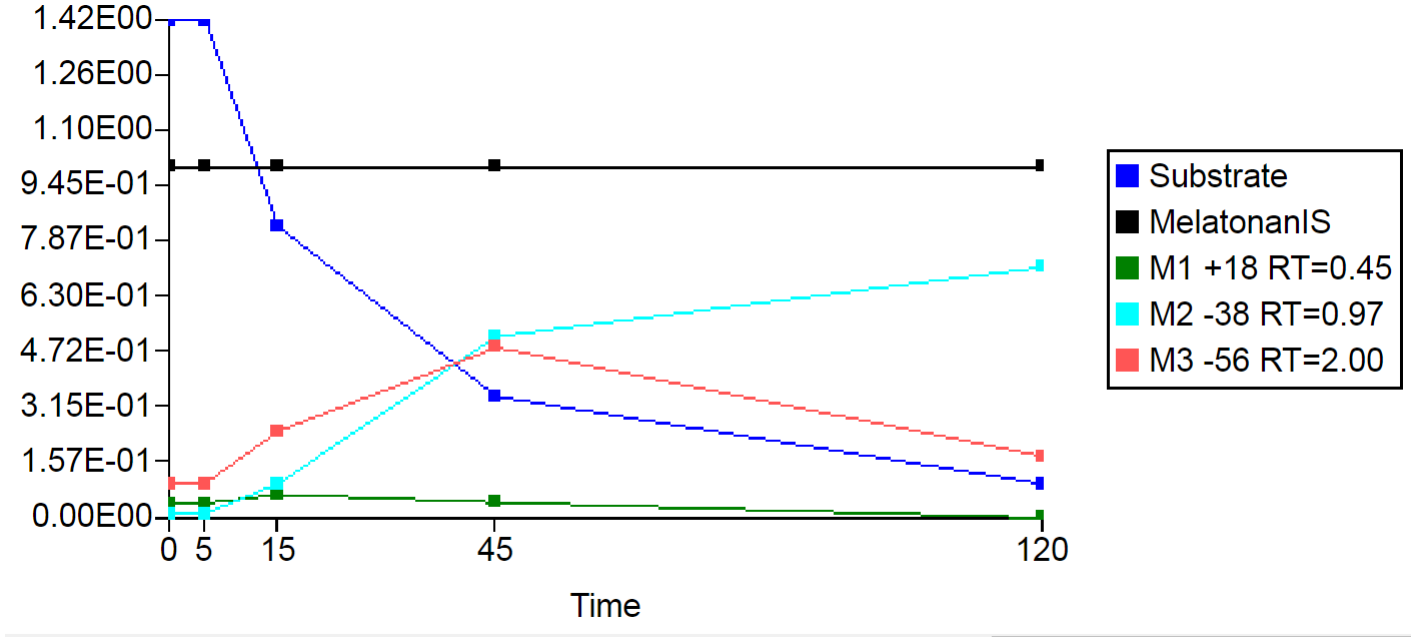
Peak area over time for oxytocin, its metabolites and internal standard with chymotrypsin. X axis—MS Area, Y axis—Time (hr).

When metabolic stability is an issue for obtaining good pharmacokinetics, understanding the disappearance of the parent compound over time (that determines the clearance) and the appearance of metabolites can be used in the drug discovery process. The information about the structure of the first-formed metabolite may help to understand the major metabolic clearance pathway and aid in designing a new compound that is hopefully more metabolically stable. This is similarly done in the soft-spot analysis by Mass-MetaSite/WebMetabase for small molecule, but in that case the rate of formation of the metabolite is reported back as a color intensity in the parent molecule [25, 35]. To show an example in the peptide arena, the metabolite time profile for the major metabolites of buserelin and oxytocin incubated with chymotrypsin for 120 minutes is shown in Figs 9 and 10, respectively. To minimize injection-to-injection differences we used an internal standard during all incubations and so the peak area ratios of parent or metabolites to internal standard are shown in Figs 9 and 10. It is worth mentioning that the concentration of the metabolite cannot be directly correlated with the signal shown if a calibration line is not computed with an authentic standard of the metabolite. We did not have authentic standards of the metabolites and so these curves were evaluated qualitatively. The first generated metabolite usually has an exponential shape when the metabolites are starting to be formed, for example M1-786 and M4-434 in Fig 9 and M1+18 and M3-56 in Fig 10. If the metabolites are further metabolized, the signal of the metabolite will decrease since the metabolite has been consumed to generate a second generation one. Typically, the second-generation metabolite has a sigmoidal shape since it needs the first-generation metabolite to form and then be further metabolized, example being M2-684 in Fig 9 and M2-38 in Fig 10. In some cases, metabolites could be detected in the sample labeled as t = 0, for example M1+18 and M3-56 in Fig 10. This is potentially due to insufficient mixing of the quench solution and t = 0 incubation sample prior to addition of the peptide substrate. Other results are in supporting information listed in SFiles.

**Fig 11:**
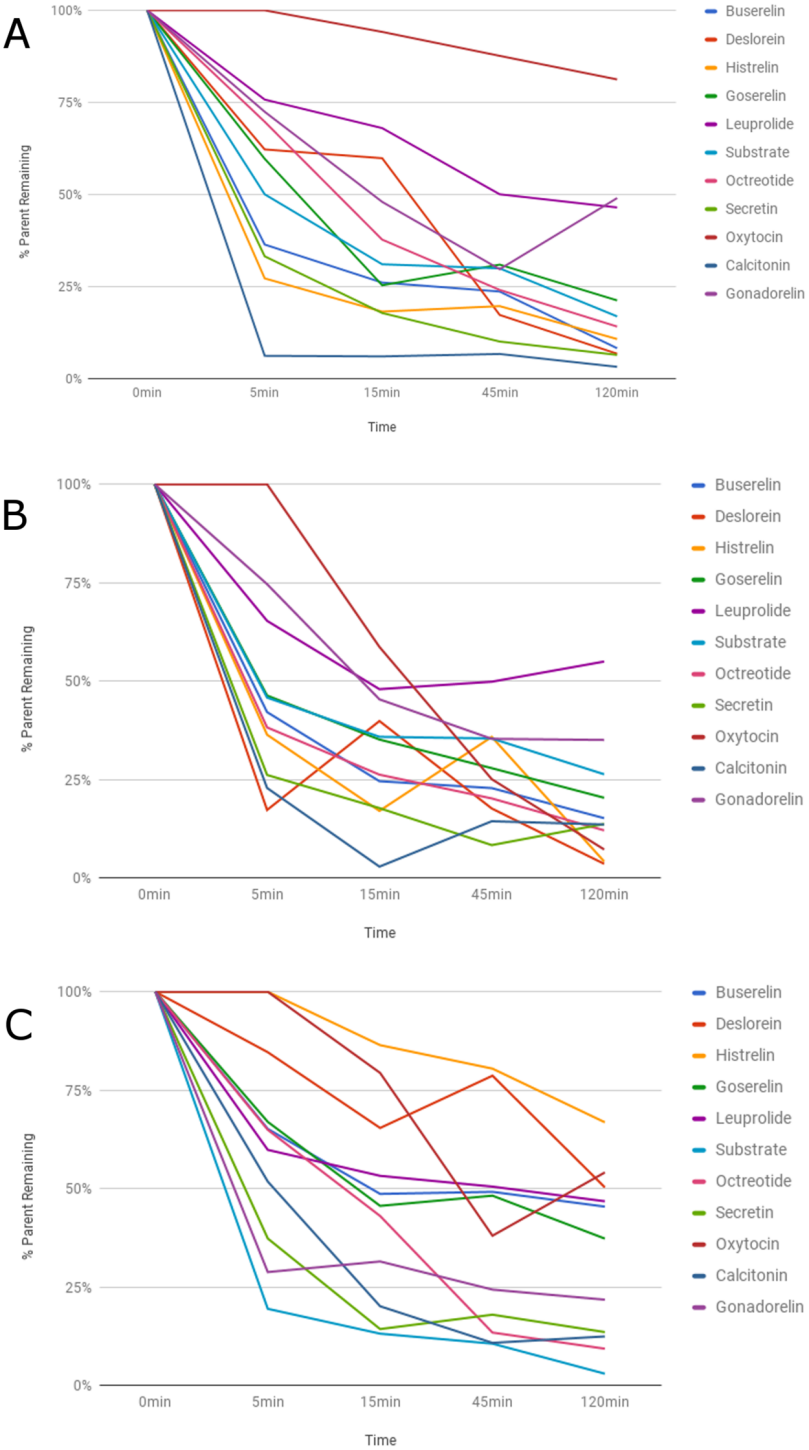
Metabolic stability of peptides from dataset-1 with serine proteases. a) Trypsinb) Chymotrypsinc) Elastase.

**Fig 12:**
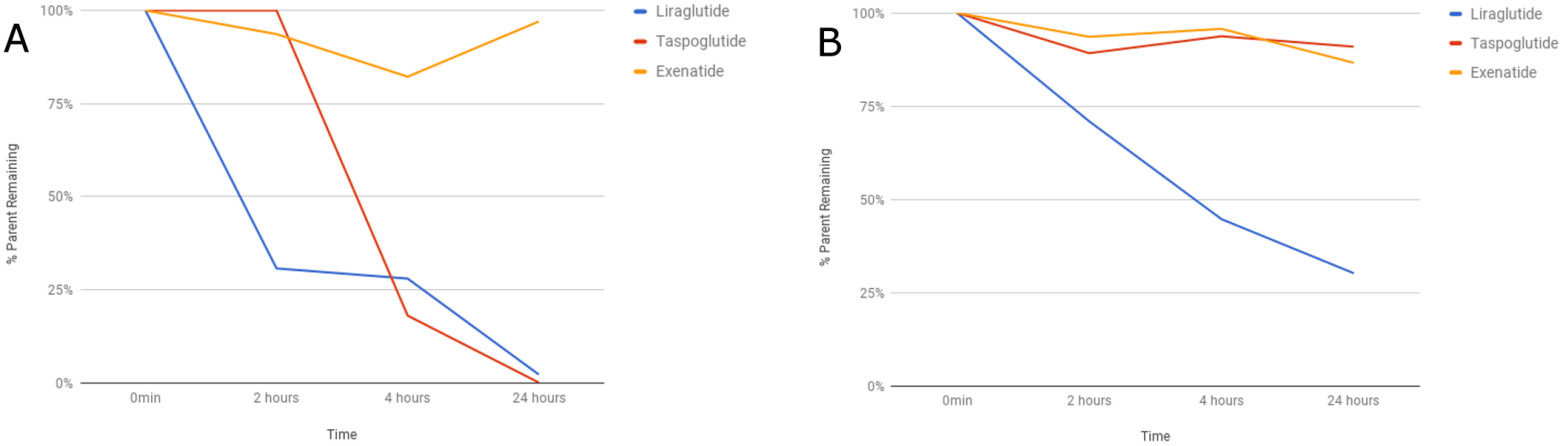
Metabolic stability of peptides in dataset-2 with a) DPP-4 and b) NEP.

We evaluated the metabolism of dataset-1 that contained ten peptide drugs and four peptide substrates in SIF with chymotrypsin, trypsin or pancreatic elastase and from the second dataset that contained four peptide drugs in DPP-4 and NEP. We compared the percent parent peptide remaining with respect to time for all investigated peptides. We used percent parent peptide remaining for the proteases marker substrates for serine proteases in dataset-1 and for both proteases in dataset-2 as a reference for the comparison (Figs 11a–11c, 12a and 12b).

All compounds from dataset-1 were hydrolyzed by chymotrypsin and trypsin with the same velocity compared to the proteases marker substrates. Pancreatic elastase acted slower than the other serine proteases. All LHRH analogues were digested with the similar speed by chymotrypsin and trypsin except leuprolide, which was hydrolyzed slower (Fig 11a and 11b). Also, histrelin clearance by the elastase was slower than for leuprolide (Fig 11c). In addition, oxytocin (the smallest peptide, MW 1007.187) was digested slower than calcitonin (the biggest peptide, MW 3429.713) by the chymotrypsin and trypsin (Fig 11a and 11b). Also, oxytocin was hydrolyzed slower than all other peptides in the trypsin incubations (Fig 11a). For the elastase protease, the smaller peptide octreotide (MW 1019.239) was digested significantly faster than the larger secretin and calcitonin, but it was slower than oxytocin (Fig 11c).

We next set out to examine the effect of small chemical changes in peptide structures for a set of 5 synthetic analogues for the luteinizing-hormone releasing hormone along with natural gonadorelin, and they were analyzed with respect to the protease-catalyzed reactions (Table 1). All LHRH analogues were digested at similar rates by both chymotrypsin and trypsin except for leuprolide, which was hydrolyzed slower. This may be due to the replacement of Gly6 in gonadorelin with the non-natural amino acid, D-Leu to form leuprolide. Our approach here revealed not only the rates of metabolism but also the site of catalysis. We found that elastase cleaved the Ser-Tyr bond and trypsin cleaved the Arg-Pro bond, except when D-Ser(tBu) was positioned in buserelin and goserelin on P2-prime, and also when the C-terminal Pro was modified to Pro-NHNHCONH2 in goserelin instead of Pro-NHet (Fig 11a–11c).

The effect of small chemical changes in peptide structures was also analyzed in dataset-2 with respect to the proteases DPP-4 and NEP (Table 1). DPP-4 and NEP hydrolyzed exenatide slower as compared to liraglutide and taspoglutide. This may be related to the substitution of Lys34 for arginine in exenatide and the addition of a C16 fatty acid at the ɛ-amino group of Lys26 using a γ-glutamic acid spacer in liraglutide. Liraglutide was digested faster than the other compounds in both matrices. For liraglutide, NEP acted slower as compared to DPP-4. Our approach revealed that liraglutide and GLP-1 were cleaved at the Ala-Glu linkage. On one hand, exenatide was not cleaved at the same site due to the amino acid change in the parent where Ala8 was modified to Gly. On the other hand, taspoglutide was cleaved despite the modification Ala8 to α-aminoisobutyric acid (Aib) (Fig 12a and 12b).

The analysis of dataset-1 resulted in 104 metabolites, while dataset-2 gave a total of 28 metabolites that were annotated in the database. All the metabolites identified were produced by amide hydrolysis. All these metabolite structural assignments were checked manually and were considered as reliable because the fragmentation was adequate, isotope pattern was as expected, the *m/z* small differences between the *m/z* of observed and theoretical (<3 ppm), and the mass score was high. For three positive control substrates (for trypsin, elastase and pepsin) the expected metabolites, coming from losing the leaving group, were detected (7-amino-4-methylcoumarin or 4-nitroanilide, depending on substrate). In the case of chymotrypsin, we observed the loss of parent signal over time, but no metabolites were identified automatically or manually. We hypothesize that this may be related to fast degradation of the metabolites.

The approach to identify metabolite products is validated by literature and the experimental evidence collected (i.e. fragmentation). As an example, for buserelin main metabolites M1-786 and M3-434 were produced by the amide hydrolysis of the Trp-Ser bond and M2-684 from amide hydrolysis of the Tyr-D-Ser(tBu) bond. The identified SoC agreed with the expected actual SoC for chymotrypsin from the literature such as Tyr-|-Xaa, Trp-|-Xaa, Phe-|-Xaa, Leu-|-Xaa, and Met-|-Xaa, where Xaa is any amino acids [7, 31]. Our observations of SoC for oxytocin are also in line with these literature reports. We found the main metabolites produced by chymotrypsin were generated through the cleavage of Leu-Gly (M1-38 and M2-56) and the Tyr-Ile (M1-38, cycle opening). GLP-1 and liraglutide were mainly metabolized by DPP-4 at the SoC Ala-Glu [32, 33]. While in the case of taspoglutide the Aib—Glu SoC was cleaved as reported in the literature [34]. All the other identified SoC in the tested incubations with investigated proteases are shown in supporting information listed in SFiles.

We have used the new developed algorithm to perform a frequency analysis of the metabolized chemical moieties for each protease. The frequency analysis considered 55 and 22 metabolized bonds from the first and second datasets respectively.

The frequency analysis performed on dataset-1 revealed that the most frequent amide bond to be cleaved was the Trp-Ser and was so for all four proteases. Moreover, several bonds were selectively cleaved by each of the proteases: 1) for the pancreatic elastase—Ser-Tyr; 2) for the trypsin—Lys-Leu, Leu-Ser; 3) for the chymotrypsin—Leu-Ser, Phe-Thr, and Gly-Leu; and 4) for the pepsin—Pro-Trp, Glu-Leu, and Gly-Leu. Therefore, to improve stability in chymotrypsin, elastase, trypsin or pepsin incubations, the design team should focus at starting point on the common SoC: Trp-Ser.

The frequency analysis performed on dataset-2 revealed that for both proteases (DPP-4 and NEP), the most frequent actual SoC was between the Ala-Glu including the cleavage of taspoglutide where Ala was modified to α-aminoisobutyric acid. Moreover, several SoCs were selective for each of the proteases: 1) DPP-4—Tyr-Leu, Ala-Ala, Ala-Lys and His-Ala and 2) NEP—Tyr-Leu and Ser-Tyr. Therefore, to improve the stability in this family of peptides, the design should consider the Ala-Glu modification, although the other more specific cleavage sites might also be considered.

The results from the SoC frequency analysis were compared with literature results and specificity models described in MEROPS and ExPASy ENZYME databases [11, 13] in Table 2.

**Table 2:** Frequency analysis results performed and compared with MEROPS and ExPASy ENZYME databases.

Protease	The most frequent SoC		MEROPS		ExPASy ENZYME	
	P1	P1’	P1	P1’	P1	P1’
**Dataset-1**						
Trypsin	Trp	Ser	Lys, Arg	Leu, Ala	Arg-|-Xaa, Lys-|-Xaa	
	***Lys***	**Leu**				
	Lue	Ser				
Chymotrypsin	*Trp*	**Ser**	Tyr, Phe, Leu	Lys, Leu, Ser	Tyr-|-Xaa, Trp-|-Xaa, Phe-|-Xaa, Leu-|-Xaa, Met-|-Xaa, His-|-Xaa	
	***Leu***	**Ser**				
	***Phe***	Thr				
	Gly	**Leu**				
Pepsin	*Trp*	Ser	Phe, Leu	Leu, Ala	Hydrophobic, preferably aromatic, residues in P1 and P1' position	
	Pro	*Trp*				
	Glu	**Leu**				
	Gly	**Leu**				
Pancreatic elastase	Trp	**Ser**	Ala, Val	Ser, Leu, Val	Ala-|-Xaa*	
	Ser	Tyr				
**Dataset-2**						
DPP-4	Tyr	**Leu**	Pro	Leu, Val, Tyr	Release of an N-terminal dipeptide, Xaa-Yaa-|-Zaa-, from a polypeptide, preferentially when Yaa is Pro, provided Zaa is neither Pro nor hydroxyproline	
	Ala	Ala				
	Ala	Lys				
	His	Ala				
Neprilysin	*Tyr*	**Leu**	Gly, Pro, Arg	Phe, Leu	Preferential cleavage of polypeptides between hydrophobic residues, particularly with Phe or Tyr at P1'	
	**Ser**	**Tyr**				

**Bold**—P1 or P1´ of the most frequent bond coincide with protease catalytic activity indicated in ExPASy ENZYME database;

*Italic*—P1 or P1´ of the most frequent bond coincide with protease specificity rules indicated in MEROPS database;

***Bold*** and ***italic***- P1 or P1´ of the most frequent bond coincide with protease specificity rules indicated in ExPASy ENZYME and MEROPS databases;

*Xaa—unknown amino acid;

The results of the frequency analysis were compared with the occurrence matrices in the MEROPS database. The frequency is a percentage defined as how many times a pair of amino acids at the P1-P1’ positions is registered as cleaved in the database divided by the total number of times the same pair is registered in the database. In the MEROPS database, we can find an occurrence of each of the natural amino acids on the positions from P4 to P4´ in the active site. The MEROPS-derived occurrence is a number of times an amino acid is found at a certain position from P4 to P4’ in the cleavage site of the investigated peptides, independently of what are at the amino acids at the other positions. This occurrence calculation is based on the available literature data and publicly available experimental information and it cannot be customized to the set of peptides of interest in a drug design project. It is important to mention that the MEROPS-derived occurrence is not considering combinations of the amino acids in the site of cleavage (only looks for amino acid in the pockets (P1, P2, P1’, P2’, etc) independently from each other). Moreover, we compared our results with the catalyzed reaction appearance rules described for each of the investigated proteases in the ExPASy ENZYME database where the search of the protease was performed using EC number. It was found that for serine proteases, only occurrence of specific amino acids in P1 position was considered to generate the catalysis reaction appearance rules. For pepsin and neprilysin reactions, catalysis rules considered amino acids’ properties at position P1 and P1´ such as hydrophobicity and aromaticity of the side chain. These rules are based on the recommendations of the Nomenclature Committee of the International Union of Biochemistry and Molecular Biology (IUBMB) and additional data extracted from the available literature. Therefore, the conclusions from these databases would represent the general rules for a peptide that may or may not be applicable to a concrete series of compounds. In contrast, our method used information coming from the database of the experiments and this database can be enriched with new experimental data. Moreover, the frequency analysis considers appearance of the pair of the monomers at the SoC.

The catalytic activity matrix from MEROPS was compared to the frequency analysis proposed in our work. In this comparison, the most frequent SoC found in our analysis matched the amino acids in P1 with highest occurrence in the catalytic matrix from MEROPS for trypsin and chymotrypsin cases. Nevertheless, in the case of pancreatic elastase, pepsin, DPP4 and neprilysin the most frequent SoC found did not match with information extracted from the MEROPS database. This may be related to the fact that the SoC (pair of amino acids) frequency analysis is compared to an amino acid occurrence and therefore it is more specific, because the SoC frequency method considers the amino acids at P1 and P1’ pocket at the same time as compared to the occurrence in MEROPS that only consider them independently.

During this comparison with ExPASy ENZYME database we revealed several matches for trypsin, chymotrypsin, pepsin, and neprilysin frequency analysis results matched with the reaction catalysis rules described in ExPASy ENZYME database. No matches were found for the pancreatic elastase and DPP4, possibly due to the high specificity of these rules generated from limited data.

Though the current number of peptide analysis does not allow us to define a statistical significance for predicting the SoC, as the datasets grow, so will the statistical significance. We did find that the most frequently cleaved bonds were comparable with the literature, demonstrating the usability of the developed methods and approach. Nevertheless, the proposed methodology compared to existing databases (i.e. ExPASy) can be applied in the case of non-natural amino acid and/or cyclic peptides. This approach can be used to derive cleavage site appearance rules for the specific peptide family (i.e. GLP-1 and analogues) or for specific experimental condition (i.e. individual protease or complex matrix as plasma). Moreover, since the system used to derive the cleavage site appearance rules (frequency analysis), could be linked to the software assisted metabolite structure elucidation based on MS data, the database could be enriched by the new experiments. Rules can then be refined to tune the system for the experimental conditions and/or peptide families of interest.

## Conclusions

We have developed an approach based on Mass-Metasite and WebMetabase to process high resolution mass spectrometry data from *in vitro* incubation samples, to predict specific metabolic cleavage sites of peptides, to store the results in a database and to analyse on this stored information. The first step involves reading peptide MSMS data, finding the chromatographic peaks related to the parent compound and elucidating the structure of the metabolites. The second step is storing of the peptide information and its metabolites within a database system. The developed approach stores peptide and metabolite structures in a database, annotating structural blocks of amino acids between amide bonds and their connectivity. In this way, the database is chemically aware and can be used to search for chemistry/structural-based algorithms, which are independent of the peptide structure (natural or non-natural amino acids, linear or cyclic structure etc.) and substructures of any size. The advantage of this new approach is that the database is not restricted to the more traditional approaches like the theoretical mass spectrum or sequence alignment. Finally, frequency analysis—the third step—reveals the most metabolically labile SoC in the studied peptides for the specific protease tested.

Using this approach, we processed high-resolution, mass-spectrometry data from incubation of fourteen peptide drugs incubated with different proteolytic media: trypsin, chymotrypsin, pancreatic elastase, DPP4 and NEP. The results were then stored in a database and available for SoC frequency analyses within WebMetabase. The advantages of this new approach are that the database is not restricted to the more traditional approaches like the theoretical mass spectrum or sequence alignment. The database is chemically aware and suited for chemistry/structural based searching algorithms which are independent of the peptide structure (natural or non-natural amino acids, linear or cyclic structure etc.) and substructures of any size. As we have shown with our selected fourteen peptide drugs, the database can be enriched with new experimental data and subsequently customized for peptides of interest.

Finally, we compared results of our database with the results available for investigated proteases in the MEROPS and ExPASy ENZYME databases. Generally, the results agreed with the publicly available data from MEROPS and/or ExPASy ENZYME databases. In conclusion, the presented approach could be useful to optimize properties of peptide drugs with regards to cleavage sites, stability, metabolism and degradation products in drug discovery.

## Supporting information

S1 Table Incubation conditions and proteases characteristics. (PDF)

S2 Table Full scan/data-dependent MS/MS experimental settings. (PDF)

S3 Table ACQUITY UPLC system experimental settings for dataset 1. (PDF)

S4 Table ACQUITY UPLC system experimental settings for dataset 2. (PDF)

S5 Table Mass-Metasite settings. (PDF)

S6 Table WebMetabase settings with experimental details. (PDF)

S1 File Metabolite identification reports exported from WebMetabase for each of the tested peptides in each protease. (ZIP)
