# Correction: Software-aided approach to investigate peptide structure and metabolic susceptibility of amide bonds in peptide drugs based on high resolution mass spectrometry

**DOI:** 10.1371/journal.pone.0200772

**Published:** 2018-07-11

**Authors:** Tatiana Radchenko, Andreas Brink, Yves Siegrist, Christopher Kochansky, Alison Bateman, Fabien Fontaine, Luca Morettoni, Ismael Zamora

In Fig 6 the images for buserelin metabolites M2 and M3 are incorrectly switched. Please see the corrected Fig 6 here.

**Fig 6:**
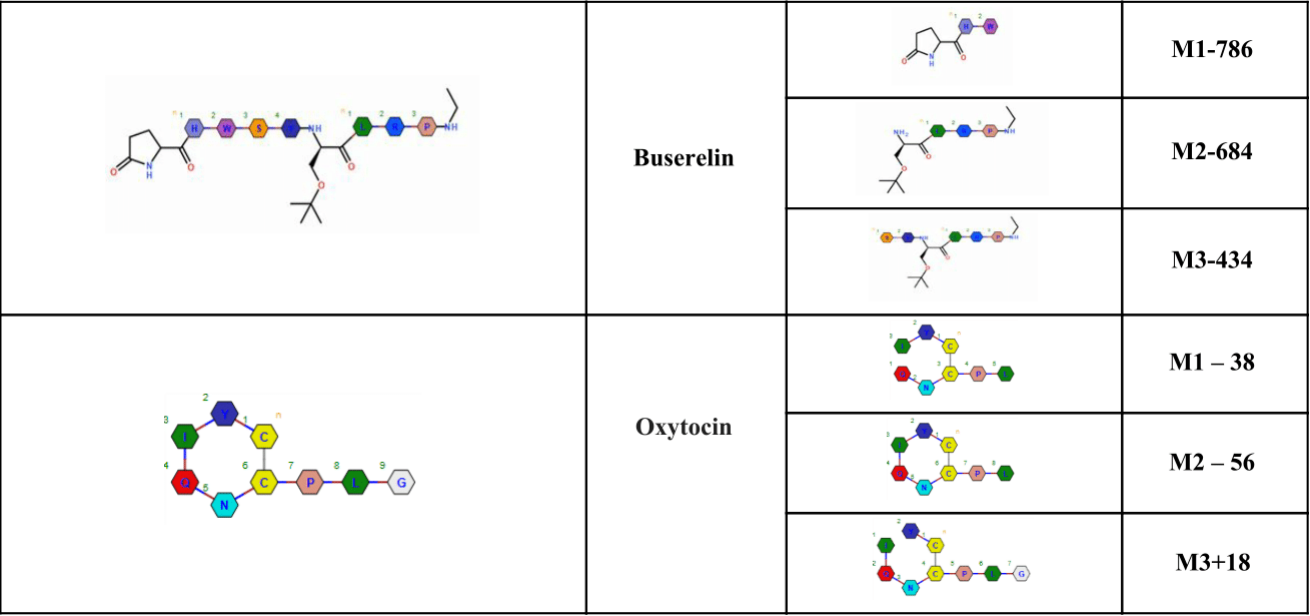
Proposed metabolites of buserelin and oxytocin found in 120 min incubations with chymotrypsin.
