# Supplementary material for: Software-aided approach to investigate peptide structure and metabolic susceptibility of amide bonds in peptide drugs based on high resolution mass spectrometry
Source: PLoS One. 2017 Nov 1;12(11):e0186461. doi: 10.1371/journal.pone.0186461 (PMC5665424; doi:10.1371/journal.pone.0186461)
Supplement: S1 Table — (PDF) [file pone.0186461.s001.pdf]

Supporting Table 1: Incubation conditions and proteases characteristics

<table>
<tr><th colspan="5">Dataset 1</th></tr>
<tr><th></th><th>Chymotrypsin</th><th>Trypsin</th><th>Elastase</th><th>Pepsin</th></tr>
<tr><td>Protease</td><td>αChymotrypsin from bovine pancreas, Sigma C3142</td><td>Trypsin from bovine pancreas, Sigma T1426</td><td>Elastase from porcine pancreas, Sigma E0258</td><td>Pepsin from porcine gastric mucosa, Sigma P6887</td></tr>
<tr><td>Matrix</td><td colspan="3">Simulated Intestinal Fluid (without Pancreatin), USP XXII Formulation, Ricca Chemical, pH (25°C) 7.4-7.6</td><td>Simulated Gastric Fluid (without Pepsin) 0.2% (w/v) Sodium Chloride in 0.7% (v/v) Hydrochloric Acid, Ricca Chemical CAT#7108-32 pH(25°C) 1.0-1.4</td></tr>
<tr><td>Internal standard</td><td colspan="4">Melanotan II acetate salt, Sigma M8693</td></tr>
<tr><td>Mobile Phase A:</td><td colspan="4">Water +0.1% formic acid</td></tr>
<tr><td>Mobile Phase B:</td><td colspan="4">Acetonitrile+0.1% formic acid</td></tr>
<tr><td>Instrument</td><td colspan="4">Thermo Scientific Q-Exactive Plus with Waters Acquity M-Class UPLC</td></tr>
<tr><td>Injection Volume</td><td colspan="4">1.00uL</td></tr>
<tr><th colspan="5">Dataset 2</th></tr>
<tr><th></th><th colspan="2">Dipeptidyl peptidase-4</th><th colspan="2">Neprilysin</th></tr>
<tr><td>Protease</td><td colspan="2">Dipeptidyl Peptidase IV human, recombinant, expressed in Sf9 cells, Sigma D3446</td><td colspan="2">Recombinant neprilysin, Creative Biomart, MME-265H</td></tr>
<tr><td>Matrix</td><td colspan="4">Hank's buffered salt solution</td></tr>
<tr><td>Mobile Phase A:</td><td colspan="4">Water +0.1% formic acid</td></tr>
<tr><td>Mobile Phase B:</td><td colspan="4">Methanol+0.1% formic acid</td></tr>
<tr><td>Instrument</td><td colspan="4">Thermo Scientific Dionex UltiMate 3000 RS HPLC and Q-Exactive™ Hybrid Quadrupole-Orbitrap Mass Spectrometer</td></tr>
<tr><td>Injection Volume</td><td colspan="4">10 μL</td></tr>
</table>
