# Supplementary material for: Software-aided approach to investigate peptide structure and metabolic susceptibility of amide bonds in peptide drugs based on high resolution mass spectrometry
Source: PLoS One. 2017 Nov 1;12(11):e0186461. doi: 10.1371/journal.pone.0186461 (PMC5665424; doi:10.1371/journal.pone.0186461)
Supplement: S2 Table — (PDF) [file pone.0186461.s002.pdf]

Supporting Table 2: MS/MS experimental settings

Setting name	Dataset 1	Dataset 2
**Parameter types are source and gas**	CUR=25 (Curtain Gas)	CUR = 25 (Curtain Gas)
	GS1=45 (Ion Source Gas 1)	GS1 = 45 (Ion Source Gas 1)
	GS2=60 (Ion Source Gas 2)	GS2 = 60 (Ion Source Gas 2)
	ISVF=5500 V (Ion Spray Voltage Floating)	ISVF = 5500 V (Ion Spray Voltage Floating)
	TEM=500°C (Temperature)	TEM = 500°C (Temperature)
**The settings for TOF MS**	CE=10 eV (Collision Energy)	CE = 10 eV (Collision Energy)
	DP=80 eV (De-clustering Potential)	DP = 80 eV (De-clustering Potential)
	Mass Range 130-1200 m/z	Mass Range 130-1200 m/z
	Accumulation Time 80 ms	Accumulation Time 80 ms
**The IDA (Information Dependent Acquisition) criteria**	Intensity is greater than 450 cps	Intensity threshold 1000 cps
	Spectra is 10, excluded for 4 seconds	TopN 5 dynamic exclusion 2.5 seconds
	Mass Tolerance is 25 ppm	Mass Tolerance is 25 ppm
	Excluded after 3 occurrences	Excluded after 3 occurrences
	Excluded isotope is 4 Da	Excluded isotope is 4 Da
**The settings for the MS/MS**	CE=40 eV	CE = 40 eV
	CES=20 eV (Collision Energy Spread)	CES = 20 eV (Collision Energy Spread)
	DP=80 eV	DP = 80 eV
	Mass Range m/z 50-1200	Mass Range m/z 50-1200
	Accumulation Time 50.0 ms	Accumulation Time 50.0 ms
