# Supplementary material for: Software-aided approach to investigate peptide structure and metabolic susceptibility of amide bonds in peptide drugs based on high resolution mass spectrometry
Source: PLoS One. 2017 Nov 1;12(11):e0186461. doi: 10.1371/journal.pone.0186461 (PMC5665424; doi:10.1371/journal.pone.0186461)
Supplement: S3 Table — (PDF) [file pone.0186461.s003.pdf]

Supporting Table 3: ACQUITY UPLC system experimental settings for dataset 1

Time (min)	Flow (uL/min)/(mL/min)	% Eluent A	% Eluent B
Initial	75.0/0.5	90	10
0.10	75.0/0.5	90	10
3.10	75.0/0.5	60	40
3.60	75.0/0.5	10	90
4.00	75.0/0.5	10	90
4.05	75.0/0.5	90	10
5.00	75.0/0.5	90	10
