# Supplementary material for: Software-aided approach to investigate peptide structure and metabolic susceptibility of amide bonds in peptide drugs based on high resolution mass spectrometry
Source: PLoS One. 2017 Nov 1;12(11):e0186461. doi: 10.1371/journal.pone.0186461 (PMC5665424; doi:10.1371/journal.pone.0186461)
Supplement: S4 Table — (PDF) [file pone.0186461.s004.pdf]

Supporting Table 4: ACQUITY HPLC system experimental settings for dataset 2

Time (min)	Flow (mL/min)	% Eluent A	% Eluent B
Initial	0.4	95	5
1	0.4	95	5
7.50	0.4	60	40
11.0	0.4	0	100
