# Supplementary material for: Software-aided approach to investigate peptide structure and metabolic susceptibility of amide bonds in peptide drugs based on high resolution mass spectrometry
Source: PLoS One. 2017 Nov 1;12(11):e0186461. doi: 10.1371/journal.pone.0186461 (PMC5665424; doi:10.1371/journal.pone.0186461)
Supplement: S5 Table — (PDF) [file pone.0186461.s005.pdf]

Supporting Table 5: Mass-MetaSite settings

Mass-MetaSite setting category	Mass-MetaSite setting name	Mass-MetaSite setting value
Import	Protonation policy	pH=7
	Maximum number of conformers	20
Metabolite generation	Minimum mass	50
	Metabolite stereochemistry and redundant metabolites	ignored
	MIM (the percentage of the monoisotopic mass of the parent)	30%
	Common cytochrome P450 reaction mechanisms	none
Mass settings, experiment	Amide Hydrolysis	true
	Retention time range (min)	not used
	GSH mode	deactivated
Mass settings, MS peaks	Maximum metabolite count limit	20
	Peak area threshold (%)	0.50%
	Peak area threshold (absolute)	0
	Peak detection smoothing	level 1
Expected metabolites	Rescue computed DRM peaks	not used
	Split computed DRM peaks	not used
	Adducts	not used
	Dimeric Ions	
	Unexpected metabolites	excluded
	Break metabolites	used
Mass settings, Met ID	Number of metabolite generations	2
	Compound fragmenting, bond breaking limit	2
	Even electron	MS and MS/MS
	Odd electron	MS and MS/MS
	N-Oxide	MS
Mass settings, DD-MS/MS algorithms, thresholds	Mass spectrometer	Thermo Orbitrap_DDS
	Same peak tolerance (amu)	0.01
	Chromatogram automatic filtering threshold	0.97
	MS automatic filtering threshold	0.98
	MS/MS automatic filtering threshold	0.95
	Ionization mode	positive $[M+H]^+$
	Spectra comparisons for “Maximum MS/MS level"	2
	Signal filtering	automatic
	Scan filtering	automatic
