# Supplementary material for: Software-aided approach to investigate peptide structure and metabolic susceptibility of amide bonds in peptide drugs based on high resolution mass spectrometry
Source: PLoS One. 2017 Nov 1;12(11):e0186461. doi: 10.1371/journal.pone.0186461 (PMC5665424; doi:10.1371/journal.pone.0186461)
Supplement: S6 Table — (PDF) [file pone.0186461.s006.pdf]

Supporting Table 6: WebMetabase settings with experimental details

<table>
<tr><th>Settings/Variables</th><th>Setting/Variable name</th><th>Dataset 1</th><th colspan="2">Dataset 2</th></tr>
<tr><td rowspan="4">User Experimental Setting</td><td>M/Z tolerance</td><td>0.025</td><td colspan="2">0.025</td></tr>
<tr><td>Retention time tolerance</td><td>0.2</td><td colspan="2">0.2</td></tr>
<tr><td>Retention time for calibration experiments</td><td>0.6</td><td colspan="2">0.6</td></tr>
<tr><td>Number for the important metabolites to show</td><td>4</td><td colspan="2">4</td></tr>
<tr><td rowspan="3">Protocol Variables</td><td rowspan="2">Time (min)</td><td rowspan="2">0, 5, 15, 45,120</td><td>GLP-1 analogues</td><td>0, 120, 240, 1440</td></tr>
<tr><td>GLP-1</td><td>0, 5, 15, 30,60</td></tr>
<tr><td>Matrix</td><td>Trypsin, Chymotrypsin, Elastase, Pepsin</td><td colspan="2">DPP-4, NEP</td></tr>
</table>
