# Supplementary material for: Software-aided approach to investigate peptide structure and metabolic susceptibility of amide bonds in peptide drugs based on high resolution mass spectrometry
Source: PLoS One. 2017 Nov 1;12(11):e0186461. doi: 10.1371/journal.pone.0186461 (PMC5665424; doi:10.1371/journal.pone.0186461)
Supplement: S1 File — (ZIP) [file pone.0186461.s007.zip › SFiles/S10_File.pdf]

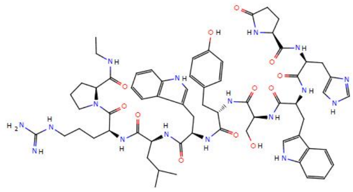

# Deslorelin

| Property name | Property value |
|---|---|
| Time | 0min, 5min, 15min, 45min, 120min |
| Instrument | ThermoQAPlus |
| Matrix | trypsin |
| Acquisition Mode | ddMS2 |

## Chromatograms

Time=0min

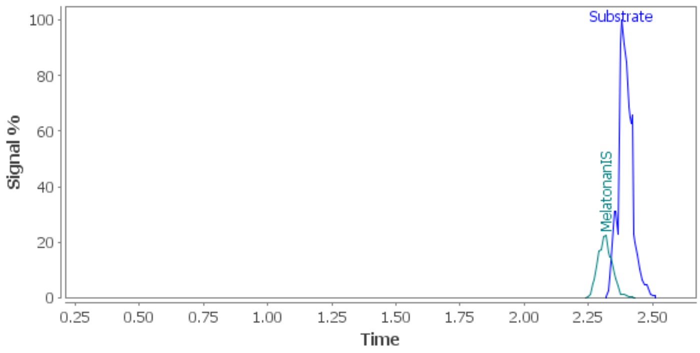


Time=5min

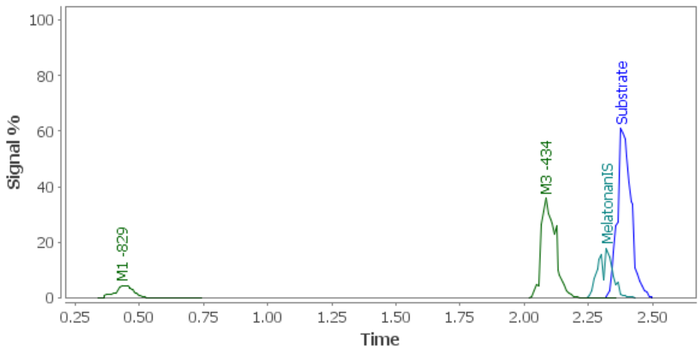


Time=15min

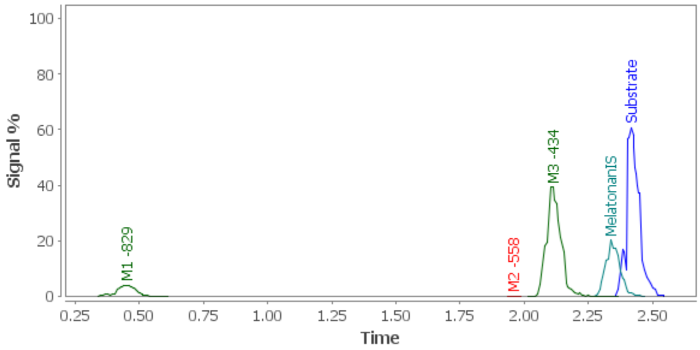


Time=45min

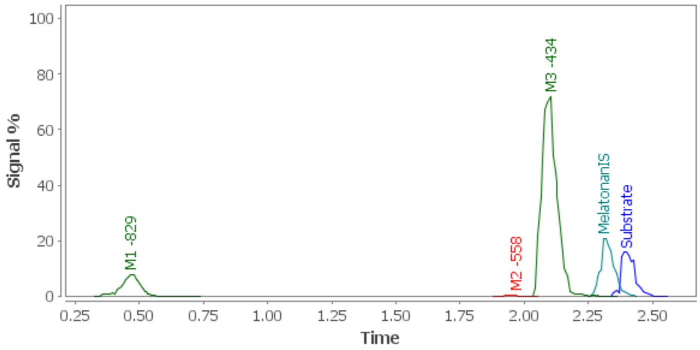


Time=120min

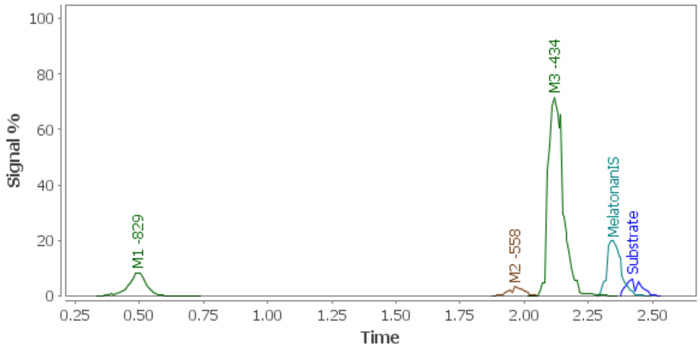

# Custom Charts

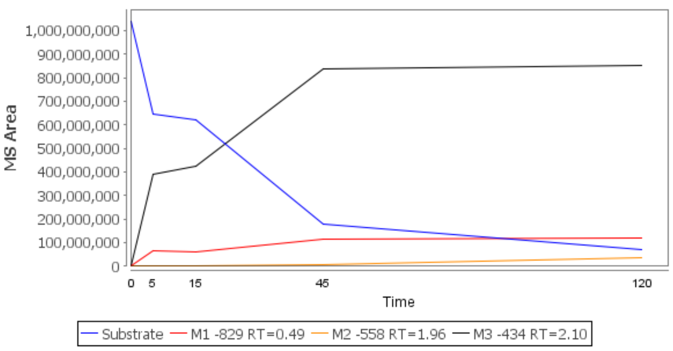

# Fragmentation

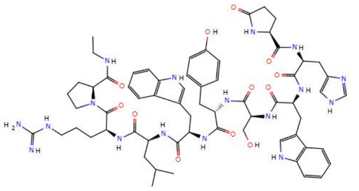

Deslorelin

MS (+) FT

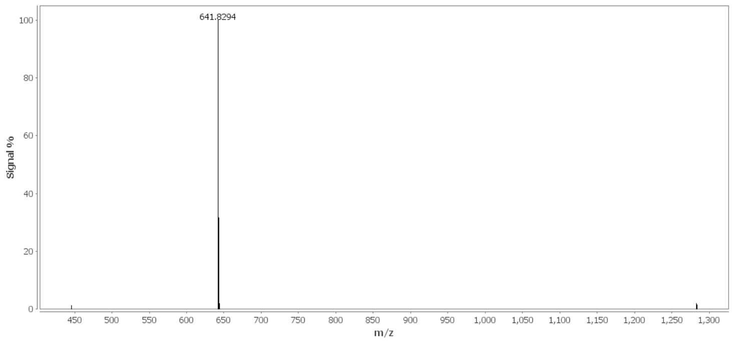


MS (+) FT

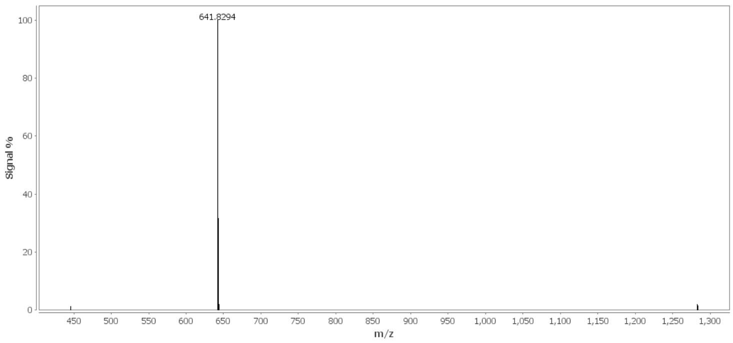


MS2 (+) FT activ = HCD:ce =

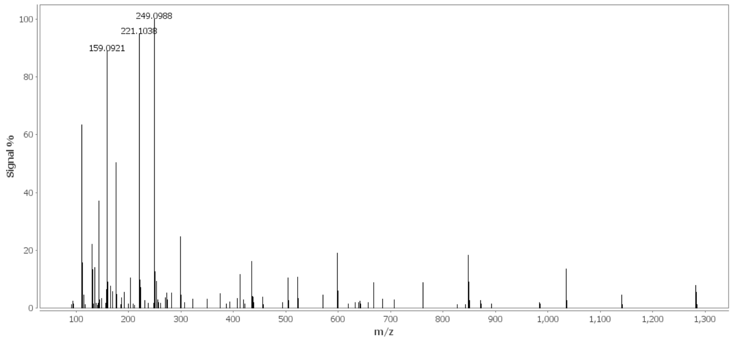


MS2 (+) FT activ = HCD:ce =

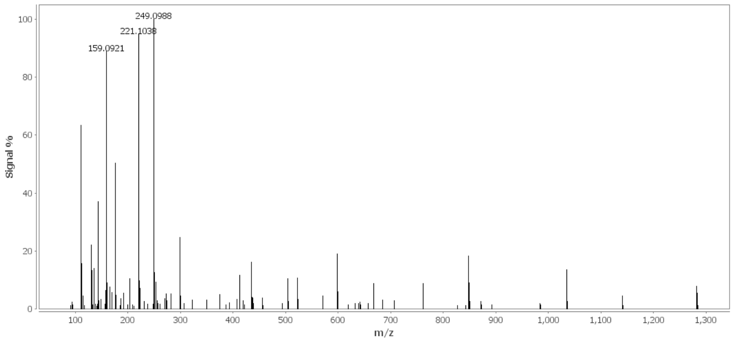


Metabolite: Substrate

| Type | score | sub. m/z observed | sub. m/z calculated | sub ppm | | met. m/z observed | met. m/z calculated | met. ppm |
|---|---|---|---|---|---|---|---|---|
| MATCH | 18.6 | 1282.6521 | 1282.6480 | -3.21 | 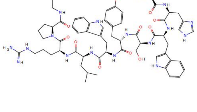 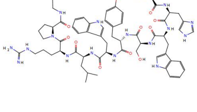 | 1282.6521 | 1282.6480 | -3.21 |
| MATCH | 101.9 | 1282.6492 | 1282.6480 | -0.94 | 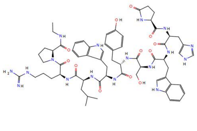 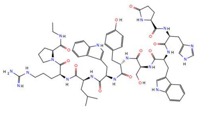 | 1282.6492 | 1282.6480 | -0.94 |
| MATCH | 15.5 | 1034.5590 | 1034.5570 | -1.87 | 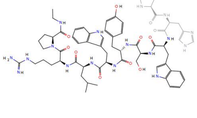 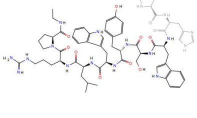 | 1034.5590 | 1034.5570 | -1.87 |

Metabolite: Substrate

| Type | score | sub. m/z observed | sub. m/z calculated | sub ppm | | | met. m/z observed | met. m/z calculated | met. ppm |
|---|---|---|---|---|---|---|---|---|---|
| MATCH | 4.4 | 871.3543 | 871.3522 | -2.43 | 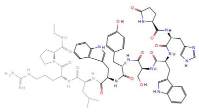 | 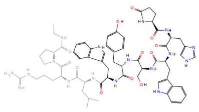 | 871.3543 | 871.3522 | -2.43 |
| MATCH | 8.5 | 685.2731 | 685.2729 | -0.31 | 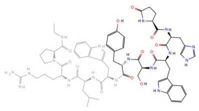 | 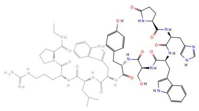 | 685.2731 | 685.2729 | -0.31 |
| MATCH | 101.9 | 657.2795 | 657.2780 | -2.33 | 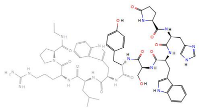 | 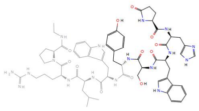 | 657.2795 | 657.2780 | -2.33 |
| MATCH | 200.0 | 641.8294 | 641.8276 | -2.72 | 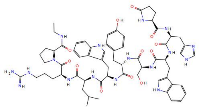 | 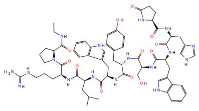 | 641.8294 | 641.8276 | -2.72 |
| MATCH | 13.0 | 641.8274 | 641.8276 | 0.35 | 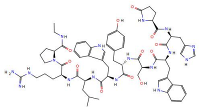 | 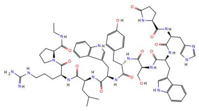 | 641.8274 | 641.8276 | 0.35 |
| MATCH | 58.4 | 598.3833 | 598.3824 | -1.62 | 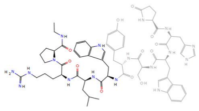 | 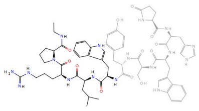 | 598.3833 | 598.3824 | -1.62 |
| MATCH | 12.8 | 504.1999 | 504.1990 | -1.72 | 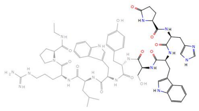 | 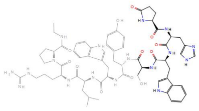 | 504.1999 | 504.1990 | -1.72 |
| MATCH | 25.4 | 494.2152 | 494.2146 | -1.07 | 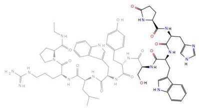 | 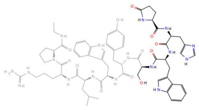 | 494.2152 | 494.2146 | -1.07 |
| MATCH | 12.3 | 456.2727 | 456.2718 | -1.99 | 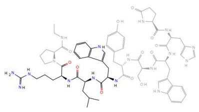 | 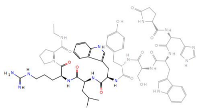 | 456.2727 | 456.2718 | -1.99 |

Metabolite: Substrate

| Type | score | sub. m/z observed | sub. m/z calculated | sub ppm | | | met. m/z observed | met. m/z calculated | met. ppm |
|---|---|---|---|---|---|---|---|---|---|
| MATCH | 4.5 | 439.2443 | 439.2452 | 1.99 | 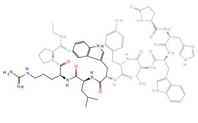 | 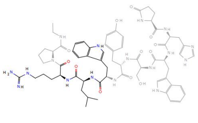 | 439.2443 | 439.2452 | 1.99 |
| MATCH | 32.6 | 412.3042 | 412.3031 | -2.73 | 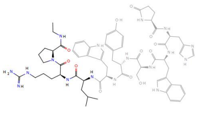 | 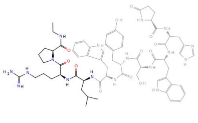 | 412.3042 | 412.3031 | -2.73 |
| MATCH | 3.0 | 300.1708 | 300.1707 | -0.47 | 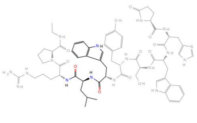 | 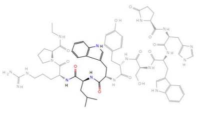 | 300.1708 | 300.1707 | -0.47 |
| MATCH | 3.0 | 300.1708 | 300.1707 | -0.47 | 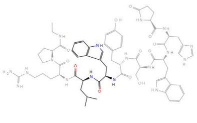 | 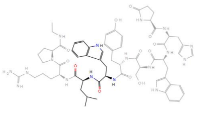 | 300.1708 | 300.1707 | -0.47 |
| MATCH | 67.6 | 299.2197 | 299.2190 | -2.43 | 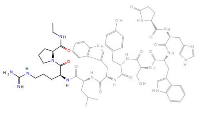 | 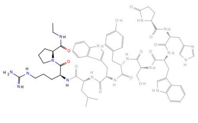 | 299.2197 | 299.2190 | -2.43 |
| MATCH | 13.9 | 282.1924 | 282.1925 | 0.03 | 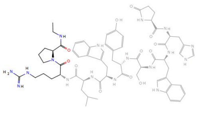 | 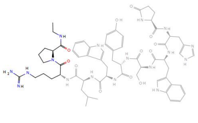 | 282.1924 | 282.1925 | 0.03 |
| MATCH | 12.5 | 272.1761 | 272.1757 | -1.47 | 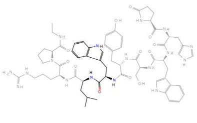 | 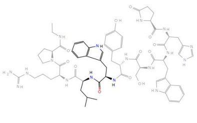 | 272.1761 | 272.1757 | -1.47 |
| MATCH | 9.2 | 270.1931 | 270.1925 | -2.56 | 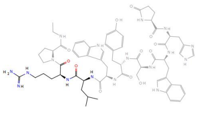 | 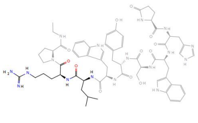 | 270.1931 | 270.1925 | -2.56 |
| MATCH | 21.4 | 261.1133 | 261.1164 | 11.73 | 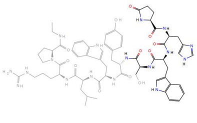 | 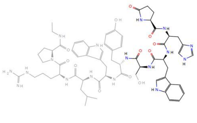 | 261.1133 | 261.1164 | 11.73 |

Metabolite: Substrate

| Type | score | sub. m/z observed | sub. m/z calculated | sub ppm | | | met. m/z observed | met. m/z calculated | met. ppm |
|---|---|---|---|---|---|---|---|---|---|
| MATCH | 8.6 | 255.1495 | 255.1492 | -1.16 | 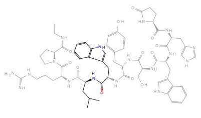 | 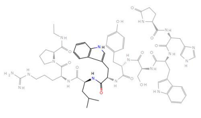 | 255.1495 | 255.1492 | -1.16 |
| MATCH | 27.6 | 253.1663 | 253.1659 | -1.66 | 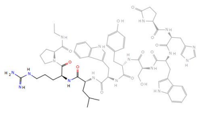 | 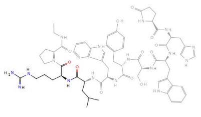 | 253.1663 | 253.1659 | -1.66 |
| MATCH | 176.6 | 249.0988 | 249.0982 | -2.16 | 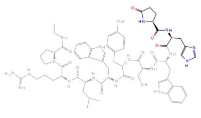 | 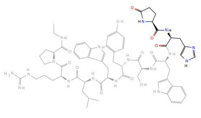 | 249.0988 | 249.0982 | -2.16 |
| MATCH | 4.1 | 237.1359 | 237.1346 | -5.52 | 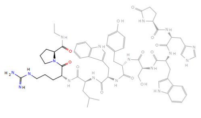 | 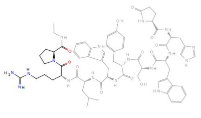 | 237.1359 | 237.1346 | -5.52 |
| MATCH | 179.3 | 221.1038 | 221.1033 | -2.28 | 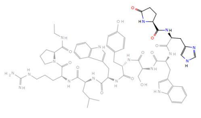 | 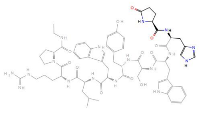 | 221.1038 | 221.1033 | -2.28 |
| MATCH | 7.9 | 187.0869 | 187.0866 | -1.83 | 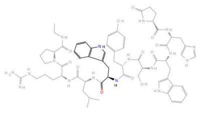 | 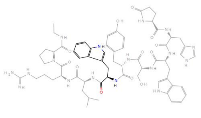 | 187.0869 | 187.0866 | -1.83 |
| MATCH | 7.9 | 187.0869 | 187.0866 | -1.83 | 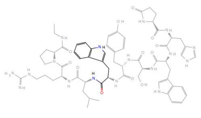 | 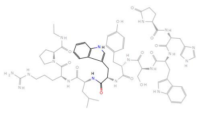 | 187.0869 | 187.0866 | -1.83 |
| MATCH | 4.0 | 185.1042 | 185.1033 | -5.06 | 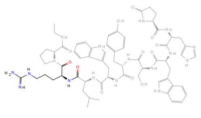 | 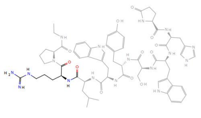 | 185.1042 | 185.1033 | -5.06 |
| MATCH | 21.6 | 170.0604 | 170.0600 | -2.21 | 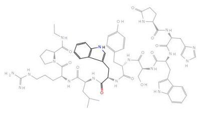 | 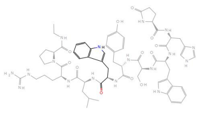 | 170.0604 | 170.0600 | -2.21 |

Metabolite: Substrate

| Type | score | sub. m/z observed | sub. m/z calculated | sub ppm | | met. m/z observed | met. m/z calculated | met. ppm |
|---|---|---|---|---|---|---|---|---|
| MATCH | 15.9 | 166.0614 | 166.0611 | -1.93 | 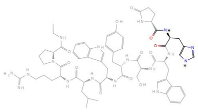 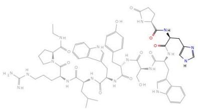 | 166.0614 | 166.0611 | -1.93 |
| MATCH | 179.7 | 159.0921 | 159.0917 | -2.53 | 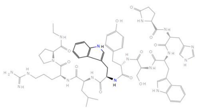 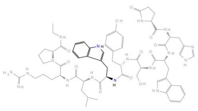 | 159.0921 | 159.0917 | -2.53 |
| MATCH | 102.0 | 159.0921 | 159.0917 | -2.53 | 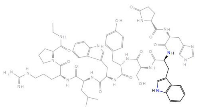 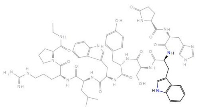 | 159.0921 | 159.0917 | -2.53 |
| MATCH | 13.2 | 157.1087 | 157.1084 | -1.85 | 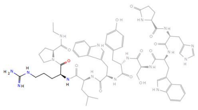 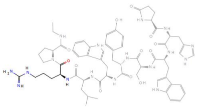 | 157.1087 | 157.1084 | -1.85 |
| MATCH | 5.1 | 144.0812 | 144.0808 | -2.88 | 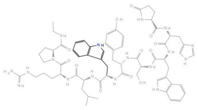 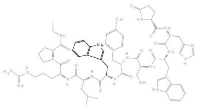 | 144.0812 | 144.0808 | -2.88 |
| MATCH | 75.5 | 143.1183 | 143.1179 | -2.90 | 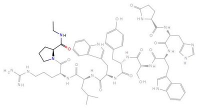 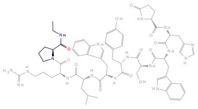 | 143.1183 | 143.1179 | -2.90 |
| MATCH | 3.2 | 142.0658 | 142.0757 | 69.33 | 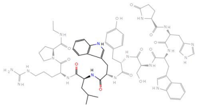 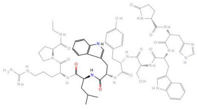 | 142.0658 | 142.0757 | 69.33 |
| MATCH | 2.1 | 140.0824 | 140.0818 | -4.18 | 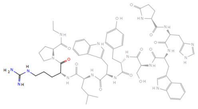 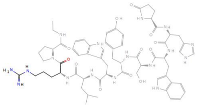 | 140.0824 | 140.0818 | -4.18 |
| MATCH | 83.2 | 136.0761 | 136.0757 | -3.21 | 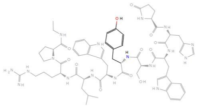 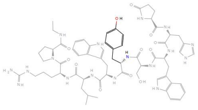 | 136.0761 | 136.0757 | -3.21 |

Metabolite: Substrate

| Type | score | sub. m/z observed | sub. m/z calculated | sub ppm | | | met. m/z observed | met. m/z calculated | met. ppm |
|---|---|---|---|---|---|---|---|---|---|
| MATCH | 9.9 | 115.0871 | 115.0866 | -4.82 | 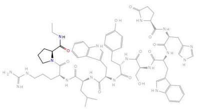 | 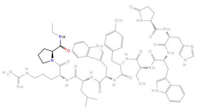 | 115.0871 | 115.0866 | -4.82 |
| MATCH | 34.7 | 112.0876 | 112.0869 | -5.85 | 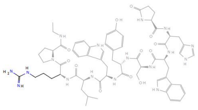 | 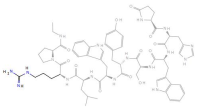 | 112.0876 | 112.0869 | -5.85 |
| MATCH | 163.4 | 110.0719 | 110.0713 | -6.01 | 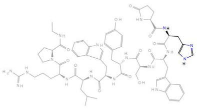 | 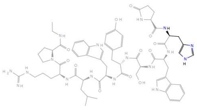 | 110.0719 | 110.0713 | -6.01 |
| MATCH | 4.3 | 95.0613 | 95.0604 | -9.26 | 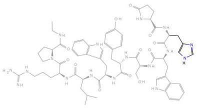 | 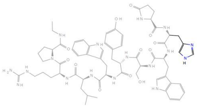 | 95.0613 | 95.0604 | -9.26 |
| MATCH | 6.0 | 91.0549 | 91.0522 | -29.6 | 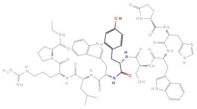 | 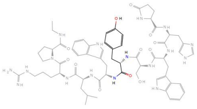 | 91.0549 | 91.0522 | -29.6 |

MS (+) FT

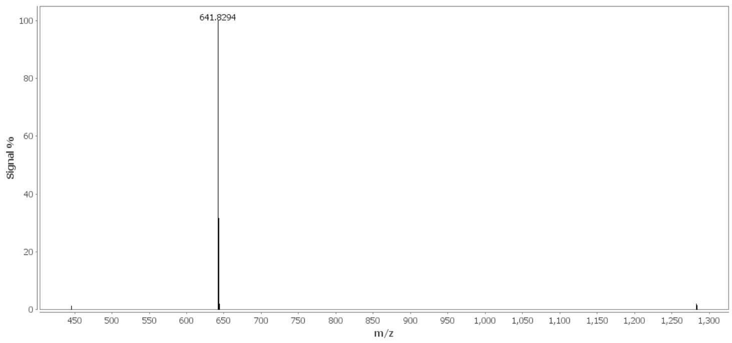


MS (+) FT

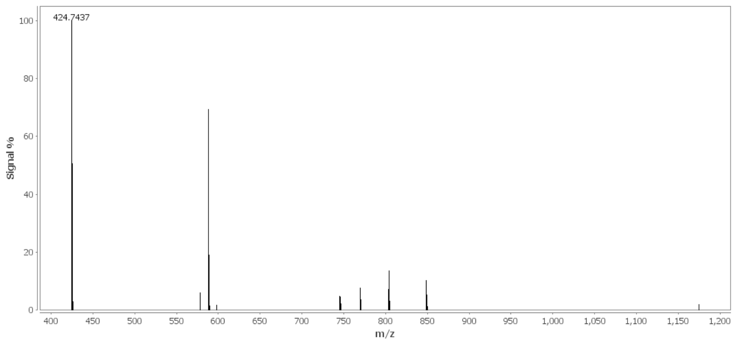


MS2 (+) FT activ = HCD:ce =

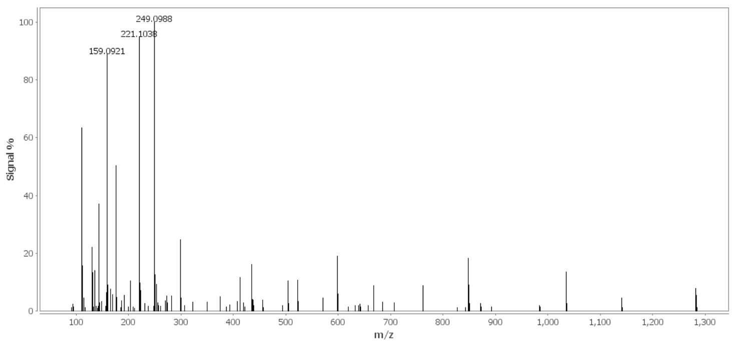


MS2 (+) FT activ = HCD:ce =

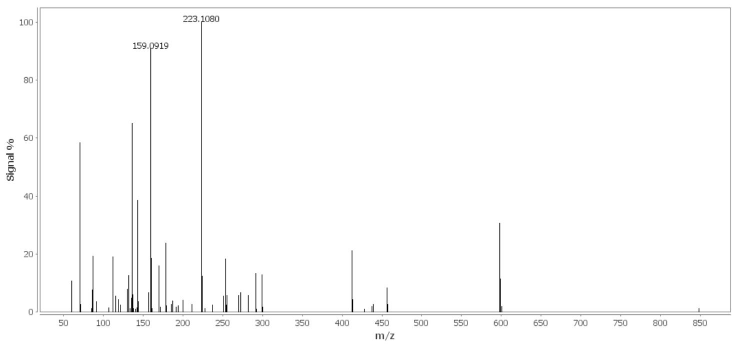

Metabolite: M3 -434 RT=2.10

| Type | score | sub. m/z observed | sub. m/z calculated | sub ppm | | met. m/z observed | met. m/z calculated | met. ppm |
|---|---|---|---|---|---|---|---|---|
| MATCH | 200.0 | 641.8294 | 641.8276 | -2.72 | 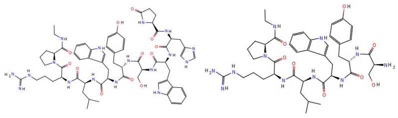 | 424.7437 | 424.7425 | -2.89 |
| MATCH | 200.0 | 641.8294 | 641.8276 | -2.72 | 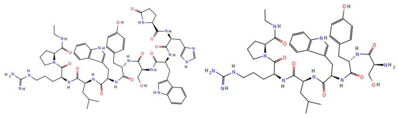 | 424.7437 | 424.7425 | -2.89 |
| MATCH | 110.3 | 641.8294 | 641.8276 | -2.72 | 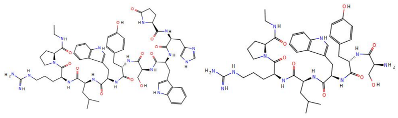 | 848.4798 | 848.4777 | -2.48 |
| MATCH | 110.3 | 641.8294 | 641.8276 | -2.72 | 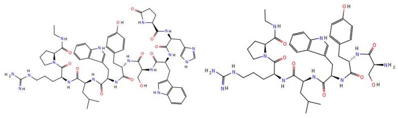 | 848.4798 | 848.4777 | -2.48 |
| MATCH | 101.9 | 1282.6492 | 1282.6480 | -0.94 | 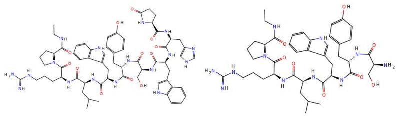 | 424.7437 | 424.7425 | -2.89 |
| MATCH | 101.9 | 1282.6492 | 1282.6480 | -0.94 | 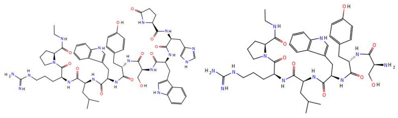 | 424.7437 | 424.7425 | -2.89 |
| MATCH | 12.1 | 1282.6492 | 1282.6480 | -0.94 | 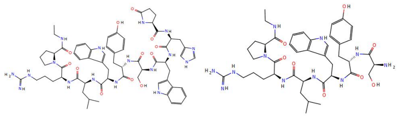 | 848.4798 | 848.4777 | -2.48 |
| MATCH | 12.1 | 1282.6492 | 1282.6480 | -0.94 | 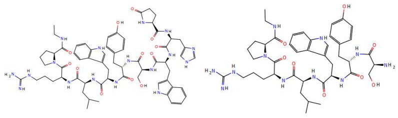 | 848.4798 | 848.4777 | -2.48 |
| MATCH | 4.8 | 91.0549 | 91.0522 | -29.6 | 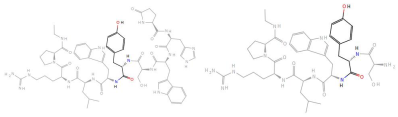 | 91.0549 | 91.0522 | -29.4 |

Metabolite: M3 -434 RT=2.10

| Type | score | sub. m/z observed | sub. m/z calculated | sub ppm | | met. m/z observed | met. m/z calculated | met. ppm |
|---|---|---|---|---|---|---|---|---|
| MATCH | 34.7 | 112.0876 | 112.0869 | -5.85 | 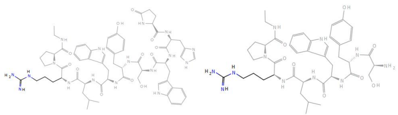 | 112.0874 | 112.0869 | -4.66 |
| MATCH | 9.9 | 115.0871 | 115.0866 | -4.82 | 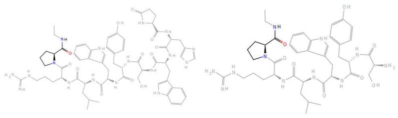 | 115.0872 | 115.0866 | -5.12 |
| MATCH | 78.8 | 136.0761 | 136.0757 | -3.21 | 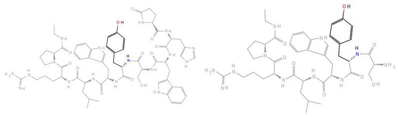 | 136.0760 | 136.0757 | -2.24 |
| MATCH | 2.1 | 140.0824 | 140.0818 | -4.18 | 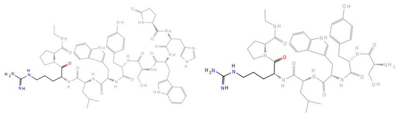 | 140.0824 | 140.0818 | -3.68 |
| MATCH | 3.2 | 142.0658 | 142.0757 | 69.33 | 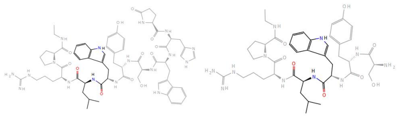 | 142.0653 | 142.0757 | 73.36 |
| MATCH | 75.5 | 143.1183 | 143.1179 | -2.90 | 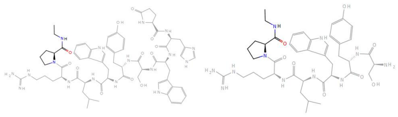 | 143.1182 | 143.1179 | -2.12 |
| MATCH | 4.7 | 144.0812 | 144.0808 | -2.88 | 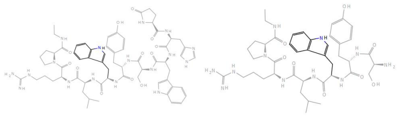 | 144.0808 | 144.0808 | -0.07 |
| MATCH | 13.2 | 157.1087 | 157.1084 | -1.85 | 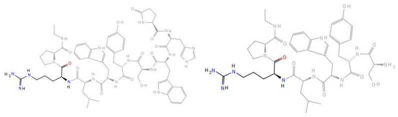 | 157.1085 | 157.1084 | -0.97 |
| MATCH | 179.7 | 159.0921 | 159.0917 | -2.53 | 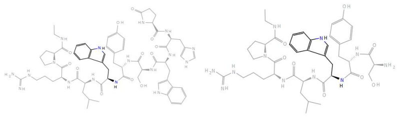 | 159.0919 | 159.0917 | -1.43 |

Metabolite: M3 -434 RT=2.10

| Type | score | sub. m/z observed | sub. m/z calculated | sub ppm | | met. m/z observed | met. m/z calculated | met. ppm |
|---|---|---|---|---|---|---|---|---|
| MATCH | 21.6 | 170.0604 | 170.0600 | -2.21 | 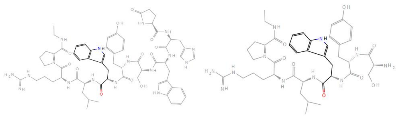 | 170.0603 | 170.0600 | -1.42 |
| MATCH | 4.0 | 185.1042 | 185.1033 | -5.06 | 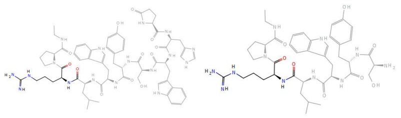 | 185.1034 | 185.1033 | -0.31 |
| MATCH | 7.5 | 187.0869 | 187.0866 | -1.83 | 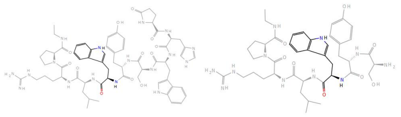 | 187.0869 | 187.0866 | -1.56 |
| MATCH | 7.5 | 187.0869 | 187.0866 | -1.83 | 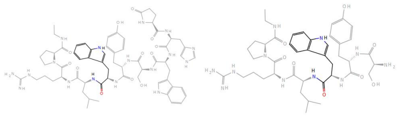 | 187.0869 | 187.0866 | -1.56 |
| MATCH | 4.1 | 237.1359 | 237.1346 | -5.52 | 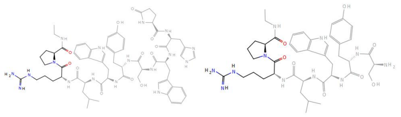 | 237.1357 | 237.1346 | -4.52 |
| MATCH | 27.6 | 253.1663 | 253.1659 | -1.66 | 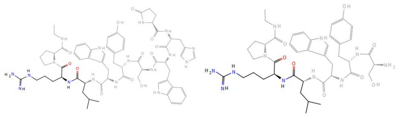 | 253.1660 | 253.1659 | -0.48 |
| MATCH | 8.6 | 255.1495 | 255.1492 | -1.16 | 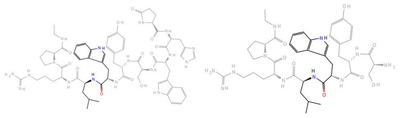 | 255.1491 | 255.1492 | 0.42 |
| MATCH | 20.8 | 261.1133 | 261.1164 | 11.73 | 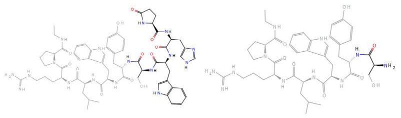 | 87.0561 | 87.0553 | -8.90 |
| MATCH | 9.2 | 270.1931 | 270.1925 | -2.56 | 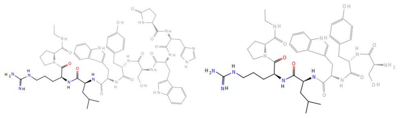 | 270.1932 | 270.1925 | -2.74 |

Metabolite: M3 -434 RT=2.10

| Type | score | sub. m/z observed | sub. m/z calculated | sub ppm | | met. m/z observed | met. m/z calculated | met. ppm |
|---|---|---|---|---|---|---|---|---|
| MATCH | 11.8 | 272.1761 | 272.1757 | -1.47 | 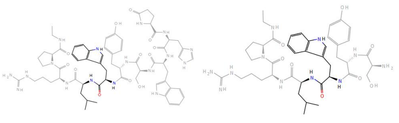 | 272.1757 | 272.1757 | 0.18 |
| MATCH | 11.0 | 282.1924 | 282.1925 | 0.03 | 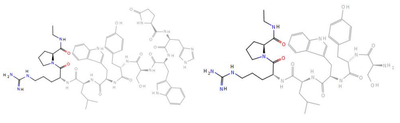 | 282.1925 | 282.1925 | -0.19 |
| MATCH | 37.6 | 299.2197 | 299.2190 | -2.43 | 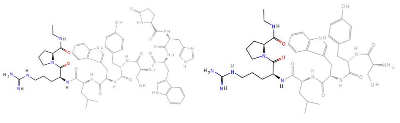 | 299.2192 | 299.2190 | -0.66 |
| MATCH | 3.0 | 300.1708 | 300.1707 | -0.47 | 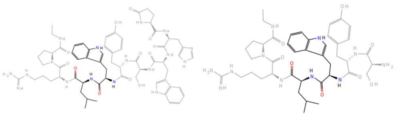 | 300.1706 | 300.1707 | 0.24 |
| MATCH | 3.0 | 300.1708 | 300.1707 | -0.47 | 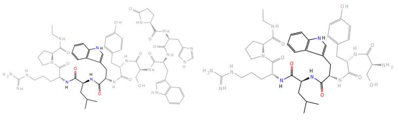 | 300.1706 | 300.1707 | 0.24 |
| MATCH | 32.6 | 412.3042 | 412.3031 | -2.73 | 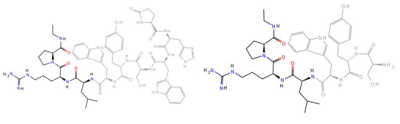 | 412.3035 | 412.3031 | -1.08 |
| MATCH | 4.5 | 439.2443 | 439.2452 | 1.99 | 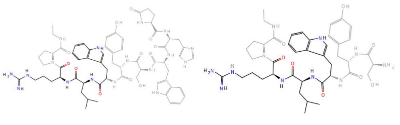 | 439.2457 | 439.2452 | -1.14 |
| MATCH | 12.3 | 456.2727 | 456.2718 | -1.99 | 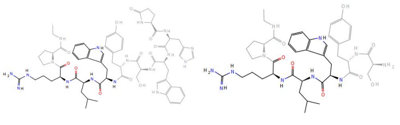 | 456.2718 | 456.2718 | -0.12 |
| MATCH | 12.6 | 494.2152 | 494.2146 | -1.07 | 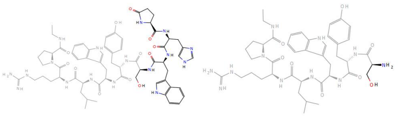 | 60.0454 | 60.0444 | -16.2 |

Metabolite: M3 -434 RT=2.10

| Type | score | sub. m/z observed | sub. m/z calculated | sub ppm | | met. m/z observed | met. m/z calculated | met. ppm |
|---|---|---|---|---|---|---|---|---|
| MATCH | 12.5 | 504.1999 | 504.1990 | -1.72 | 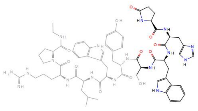 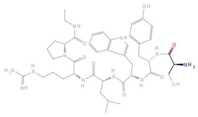 | 70.0296 | 70.0287 | -12.2 |
| MATCH | 49.6 | 598.3833 | 598.3824 | -1.62 | 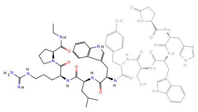 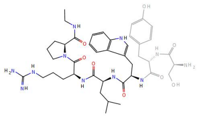 | 598.3827 | 598.3824 | -0.52 |
| MATCH | 3.5 | 641.8274 | 641.8276 | 0.35 | 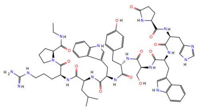 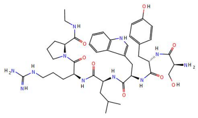 | 848.4789 | 848.4777 | -1.43 |
| | | | | | 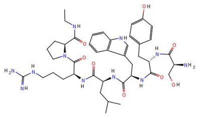 | 848.4789 | 848.4777 | -1.43 |
| MATCH | 101.9 | 657.2795 | 657.2780 | -2.33 | 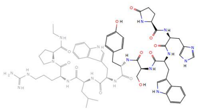 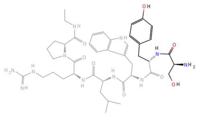 | 223.1080 | 223.1077 | -1.08 |
| MATCH | 8.5 | 685.2731 | 685.2729 | -0.31 | 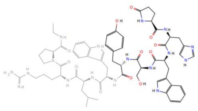 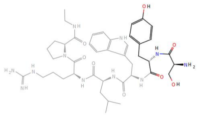 | 251.1030 | 251.1026 | -1.28 |
| MATCH | 4.4 | 871.3543 | 871.3522 | -2.43 | 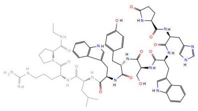 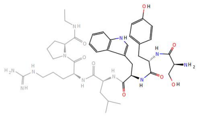 | 437.1815 | 437.1819 | 1.08 |
| MATCH | 9.1 | 1282.6521 | 1282.6480 | -3.21 | 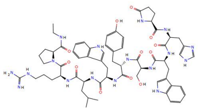 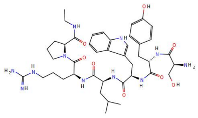 | 848.4789 | 848.4777 | -1.43 |
| | | | | | 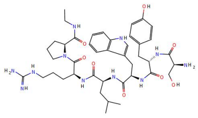 | 848.4789 | 848.4777 | -1.43 |

Metabolite: M3 -434 RT=2.10

| Type | score | sub. m/z observed | sub. m/z calculated | sub ppm | | met. m/z observed | met. m/z calculated | met. ppm |
|---|---|---|---|---|---|---|---|---|
| MET_MATCH | | | | | 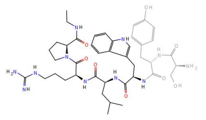 | 598.3835 | 598.3824 | -1.83 |
| MET_MATCH | | | | | 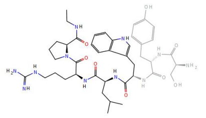 | 291.1818 | 291.1816 | -0.82 |

MS (+) FT

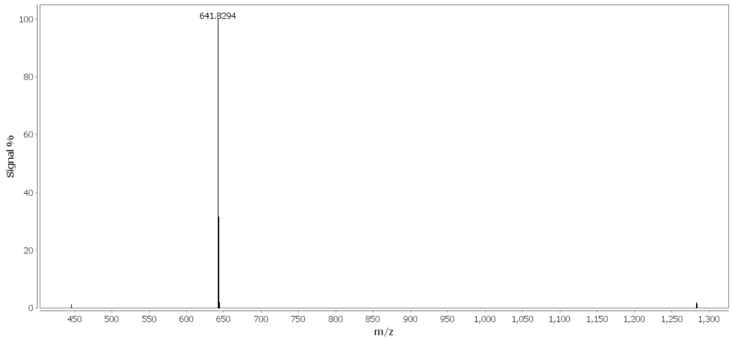


MS (+) FT

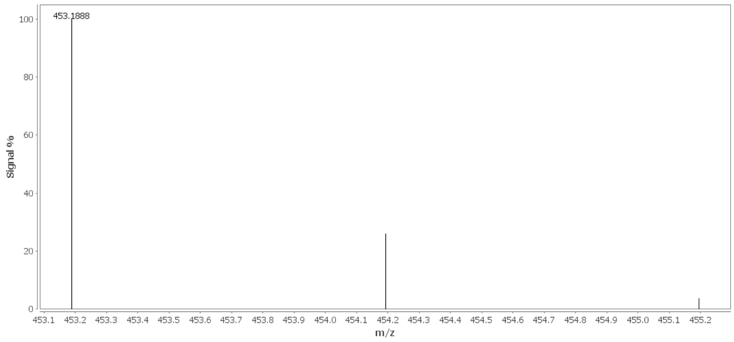


MS2 (+) FT activ = HCD:ce =

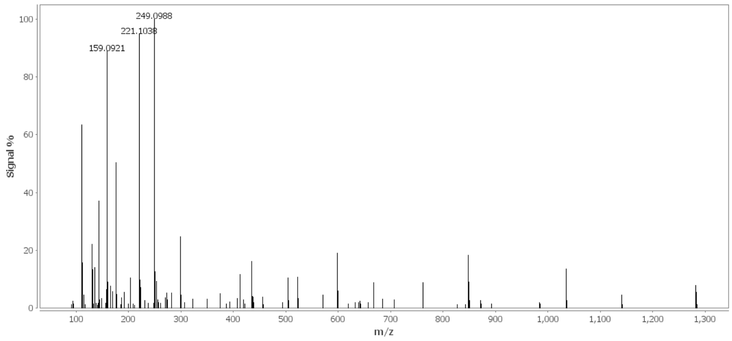


MS2 (+) FT activ = HCD:ce =

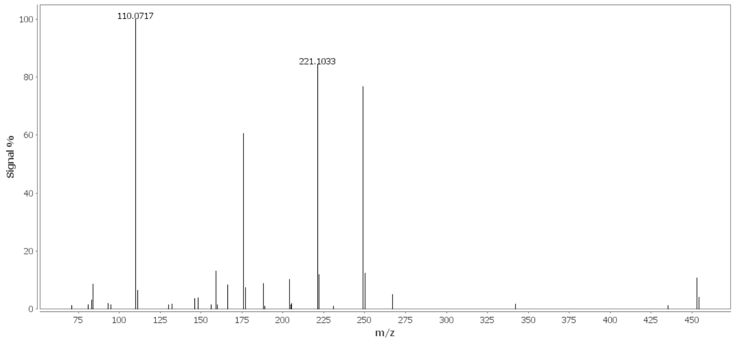


Metabolite: M1 -829 RT=0.49

| Type | score | sub. m/z observed | sub. m/z calculated | sub ppm | | | met. m/z observed | met. m/z calculated | met. ppm |
|---|---|---|---|---|---|---|---|---|---|
| MATCH | 200.0 | 641.8294 | 641.8276 | -2.72 | 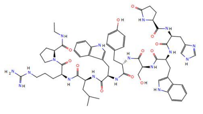 | 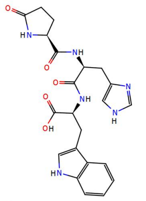 | 453.1888 | 453.1881 | -1.62 |
| | | | | | | 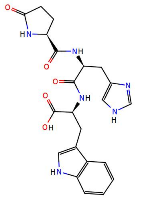 | 453.1888 | 453.1881 | -1.62 |

Metabolite: M1 -829 RT=0.49

| Type | score | sub. m/z observed | sub. m/z calculated | sub ppm | | | met. m/z observed | met. m/z calculated | met. ppm |
|---|---|---|---|---|---|---|---|---|---|
| MATCH | 101.9 | 1282.6492 | 1282.6480 | -0.94 | 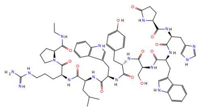 | 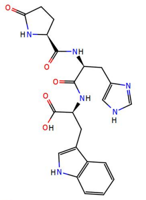 | 453.1888 | 453.1881 | -1.62 |
| | | | | | | 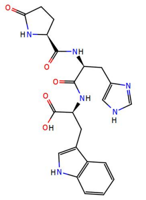 | 453.1888 | 453.1881 | -1.62 |
| MATCH | 4.3 | 93.0456 | 93.0447 | -9.59 | 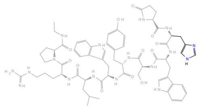 | 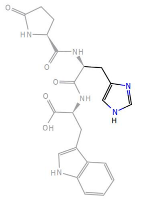 | 93.0454 | 93.0447 | -6.73 |
| MATCH | 3.1 | 95.0613 | 95.0604 | -9.26 | 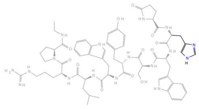 | 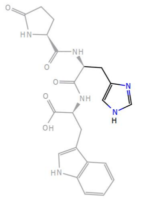 | 95.0609 | 95.0604 | -5.80 |
| MATCH | 163.4 | 110.0719 | 110.0713 | -6.01 | 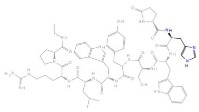 | 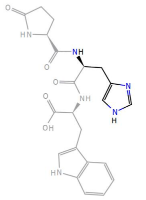 | 110.0717 | 110.0713 | -4.06 |
| MATCH | 102.0 | 159.0921 | 159.0917 | -2.53 | 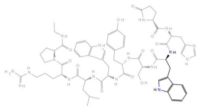 | 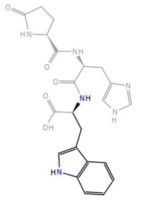 | 159.0917 | 159.0917 | -0.25 |
| MATCH | 15.9 | 166.0614 | 166.0611 | -1.93 | 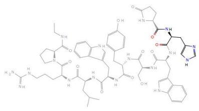 | 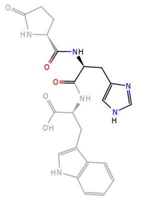 | 166.0612 | 166.0611 | -0.36 |
| MATCH | 179.3 | 221.1038 | 221.1033 | -2.28 | 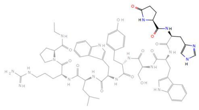 | 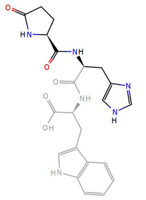 | 221.1033 | 221.1033 | -0.18 |
| MATCH | 176.6 | 249.0988 | 249.0982 | -2.16 | 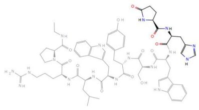 | 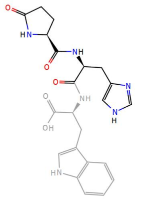 | 249.0982 | 249.0982 | -0.12 |

Metabolite: M1 -829 RT=0.49

| Type | score | sub. m/z observed | sub. m/z calculated | sub ppm | | | met. m/z observed | met. m/z calculated | met. ppm |
|---|---|---|---|---|---|---|---|---|---|
| MATCH | 13.0 | 641.8274 | 641.8276 | 0.35 | 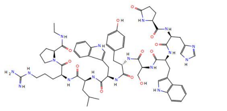 | 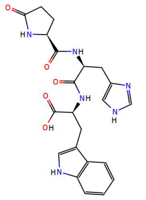 | 453.1880 | 453.1881 | 0.29 |
| | | | | | | 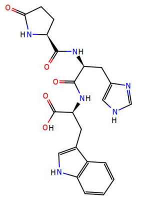 | 453.1880 | 453.1881 | 0.29 |
| MATCH | 15.5 | 1034.5590 | 1034.5570 | -1.87 | 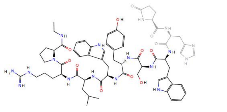 | 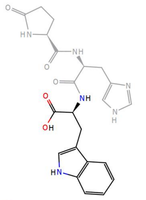 | 205.0976 | 205.0972 | -2.13 |
| MATCH | 18.6 | 1282.6521 | 1282.6480 | -3.21 | 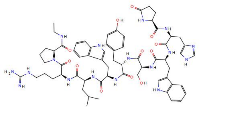 | 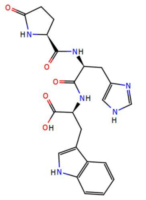 | 453.1880 | 453.1881 | 0.29 |
| | | | | | | 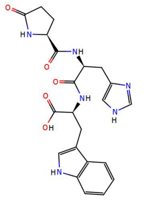 | 453.1880 | 453.1881 | 0.29 |
| MET_MATCH | | | | | | 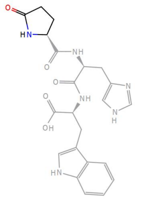 | 84.0451 | 84.0444 | -8.84 |
| MET_MATCH | | | | | | 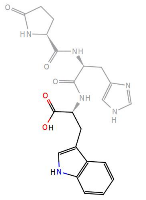 | 188.0706 | 188.0706 | 0.16 |
| MET_MATCH | | | | | | 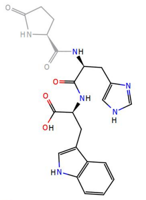 | 342.1561 | 342.1561 | -0.11 |
| MET_MATCH | | | | | | 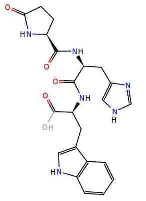 | 435.1764 | 435.1775 | 2.54 |

MS (+) FT

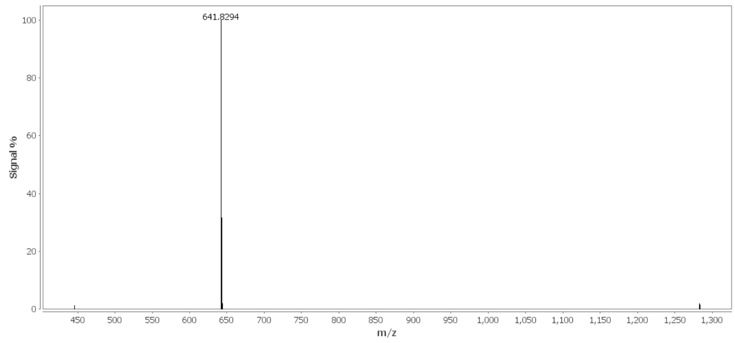


MS (+) FT

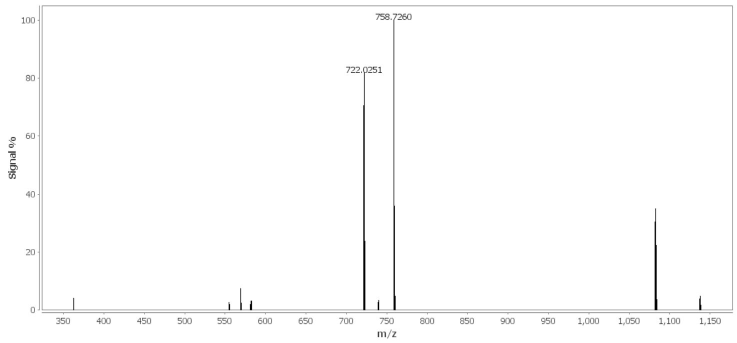


MS2 (+) FT activ = HCD:ce =

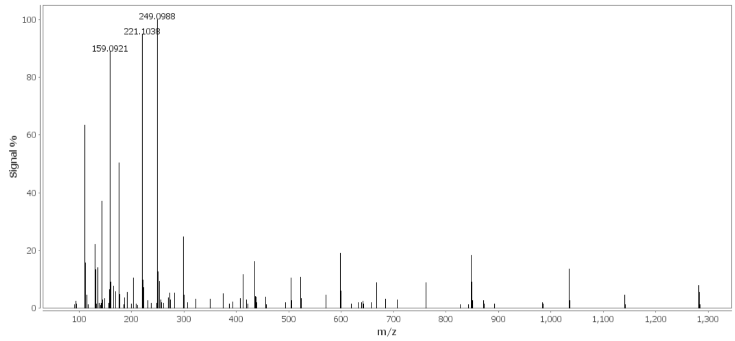


MS2 (+) FT activ = HCD:ce =

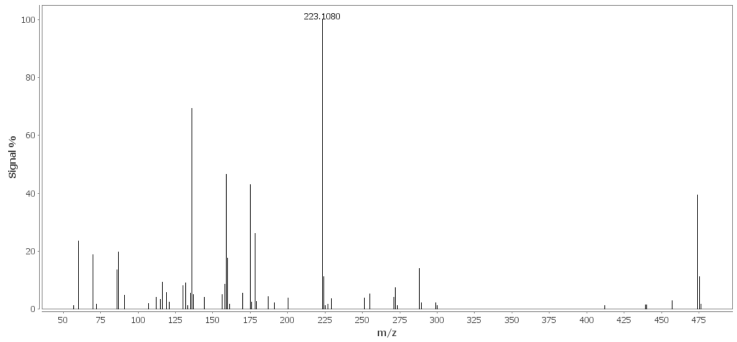


Metabolite: M2 -558 RT=1.96

| Type | score | sub. m/z observed | sub. m/z calculated | sub ppm | | | met. m/z observed | met. m/z calculated | met. ppm |
|---|---|---|---|---|---|---|---|---|---|
| MATCH | 104.1 | 641.8294 | 641.8276 | -2.72 | 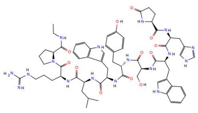 | 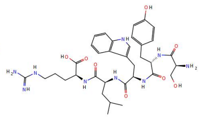 | 362.6928 | 362.6925 | -0.77 |
| | | | | | | 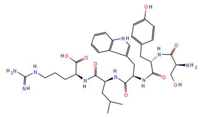 | 362.6928 | 362.6925 | -0.77 |
| MATCH | 6.0 | 1282.6492 | 1282.6480 | -0.94 | 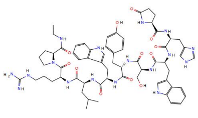 | 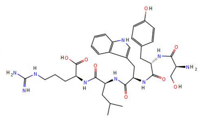 | 362.6928 | 362.6925 | -0.77 |
| | | | | | | 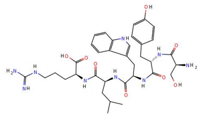 | 362.6928 | 362.6925 | -0.77 |
| MATCH | 6.0 | 91.0549 | 91.0522 | -29.6 | 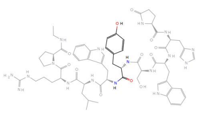 | 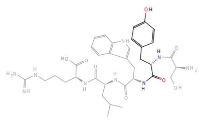 | 91.0550 | 91.0522 | -30.9 |

Metabolite: M2 -558 RT=1.96

| Type | score | sub. m/z observed | sub. m/z calculated | sub ppm | | met. m/z observed | met. m/z calculated | met. ppm |
|---|---|---|---|---|---|---|---|---|
| MATCH | 19.8 | 112.0876 | 112.0869 | -5.85 | 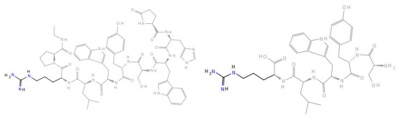 | 112.0875 | 112.0869 | -5.48 |
| MATCH | 83.2 | 136.0761 | 136.0757 | -3.21 | 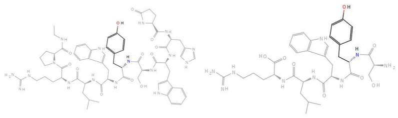 | 136.0761 | 136.0757 | -2.67 |
| MATCH | 5.1 | 144.0812 | 144.0808 | -2.88 | 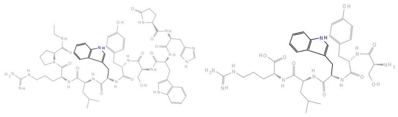 | 144.0811 | 144.0808 | -2.08 |
| MATCH | 135.5 | 159.0921 | 159.0917 | -2.53 | 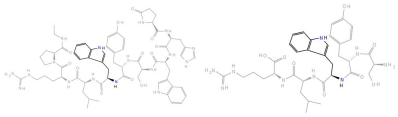 | 159.0919 | 159.0917 | -1.65 |
| MATCH | 11.0 | 170.0604 | 170.0600 | -2.21 | 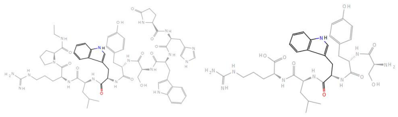 | 170.0604 | 170.0600 | -2.15 |
| MATCH | 7.9 | 187.0869 | 187.0866 | -1.83 | 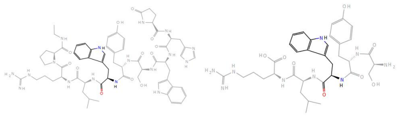 | 187.0870 | 187.0866 | -2.45 |
| MATCH | 7.9 | 187.0869 | 187.0866 | -1.83 | 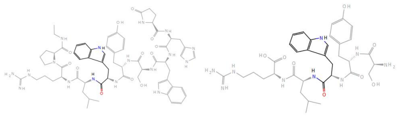 | 187.0870 | 187.0866 | -2.45 |
| MATCH | 8.2 | 255.1495 | 255.1492 | -1.16 | 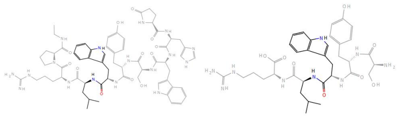 | 255.1502 | 255.1492 | -4.03 |
| MATCH | 21.4 | 261.1133 | 261.1164 | 11.73 | 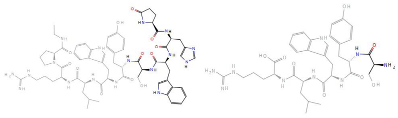 | 87.0561 | 87.0553 | -9.19 |

Metabolite: M2 -558 RT=1.96

| Type | score | sub. m/z observed | sub. m/z calculated | sub ppm | | met. m/z observed | met. m/z calculated | met. ppm |
|---|---|---|---|---|---|---|---|---|
| MATCH | 12.5 | 272.1761 | 272.1757 | -1.47 | 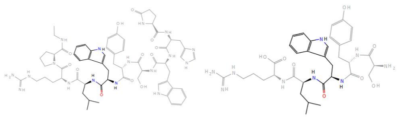 | 272.1760 | 272.1757 | -1.13 |
| MATCH | 13.9 | 282.1924 | 282.1925 | 0.03 | 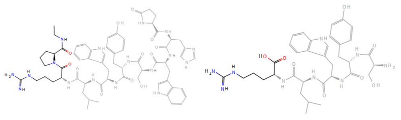 | 158.0928 | 158.0924 | -2.39 |
| MATCH | 67.6 | 299.2197 | 299.2190 | -2.43 | 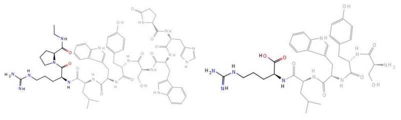 | 175.1193 | 175.1190 | -1.78 |
| MATCH | 25.6 | 412.3042 | 412.3031 | -2.73 | 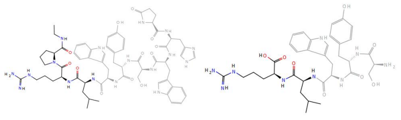 | 288.2031 | 288.2030 | -0.16 |
| MATCH | 25.4 | 494.2152 | 494.2146 | -1.07 | 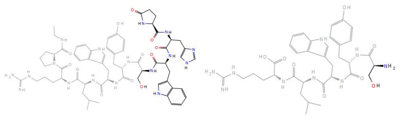 | 60.0454 | 60.0444 | -16.7 |
| MATCH | 12.8 | 504.1999 | 504.1990 | -1.72 | 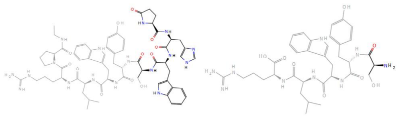 | 70.0296 | 70.0287 | -12.4 |
| MATCH | 58.4 | 598.3833 | 598.3824 | -1.62 | 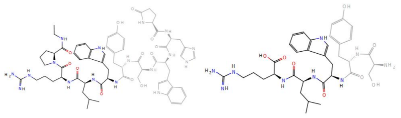 | 474.2829 | 474.2823 | -1.23 |
| MATCH | 101.9 | 657.2795 | 657.2780 | -2.33 | 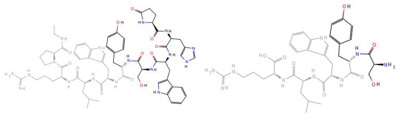 | 223.1080 | 223.1077 | -1.38 |
| MATCH | 6.8 | 685.2731 | 685.2729 | -0.31 | 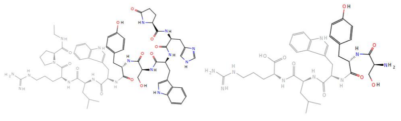 | 251.1025 | 251.1026 | 0.37 |

Metabolite: M2 -558 RT=1.96

| Type | score | sub. m/z observed | sub. m/z calculated | sub ppm | | met. m/z observed | met. m/z calculated | met. ppm |
|---|---|---|---|---|---|---|---|---|
| MET_MATCH | | | | | 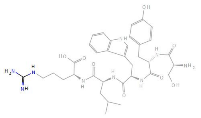 | 60.0566 | 60.0556 | -16.7 |
| MET_MATCH | | | | | 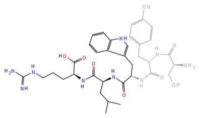 | 229.1318 | 229.1315 | -1.12 |
| MET_MATCH | | | | | 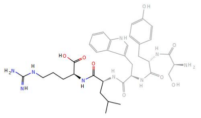 | 271.1767 | 271.1765 | -0.94 |
| MET_MATCH | | | | | 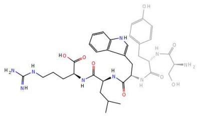 | 457.2563 | 457.2558 | -1.24 |
