# Supplementary material for: Software-aided approach to investigate peptide structure and metabolic susceptibility of amide bonds in peptide drugs based on high resolution mass spectrometry
Source: PLoS One. 2017 Nov 1;12(11):e0186461. doi: 10.1371/journal.pone.0186461 (PMC5665424; doi:10.1371/journal.pone.0186461)
Supplement: S1 File — (ZIP) [file pone.0186461.s007.zip › SFiles/S11_File.pdf]

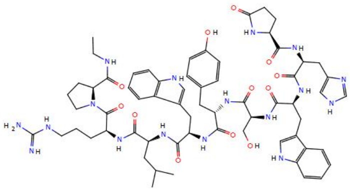

Deslorelin

Property name	Property value
Time	0min, 5min, 15min, 45min, 120min
Instrument	ThermoQAPlus
Matrix	elastase
Acquisition Mode	ddMS2

Chromatograms

Time=0min

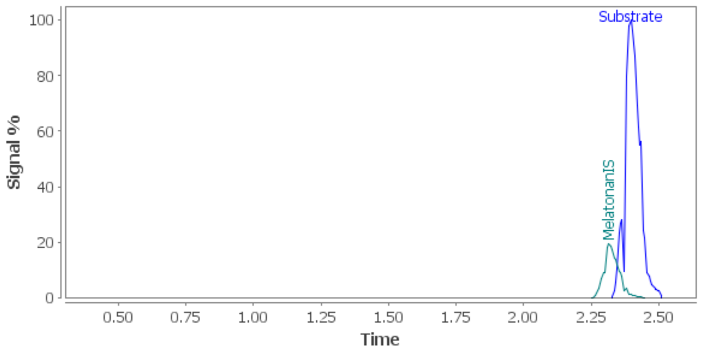

Time=5min

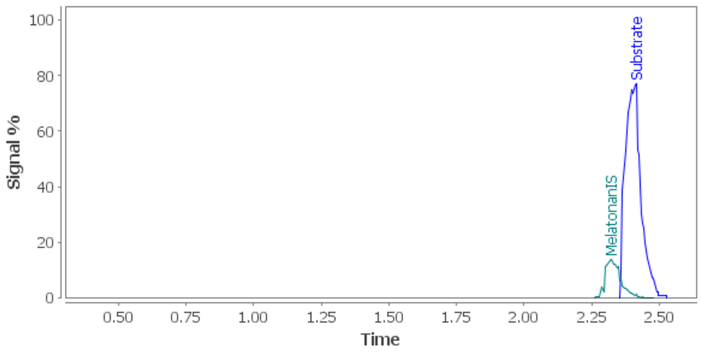

Time=15min

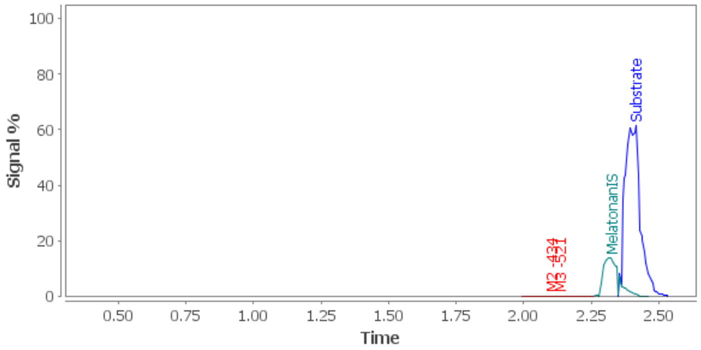

Time=45min

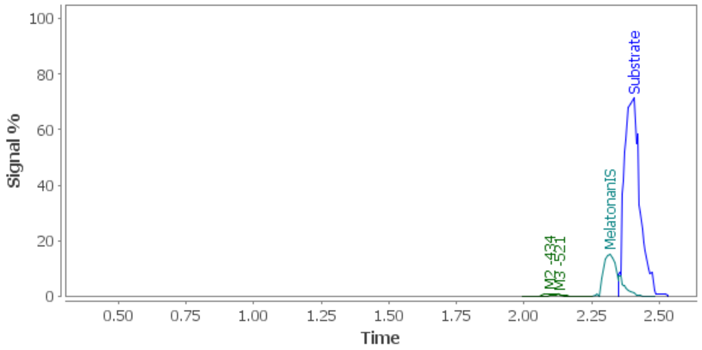

Time=120min

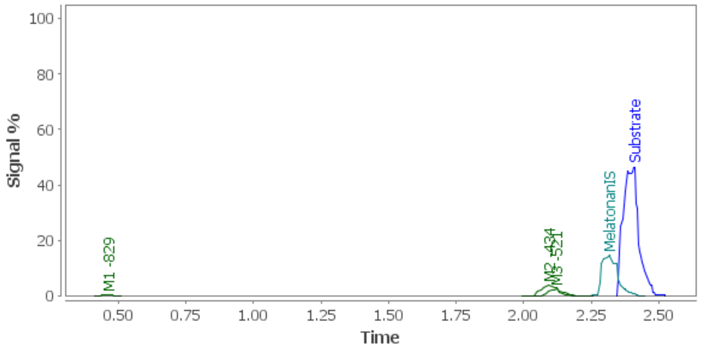

Custom Charts

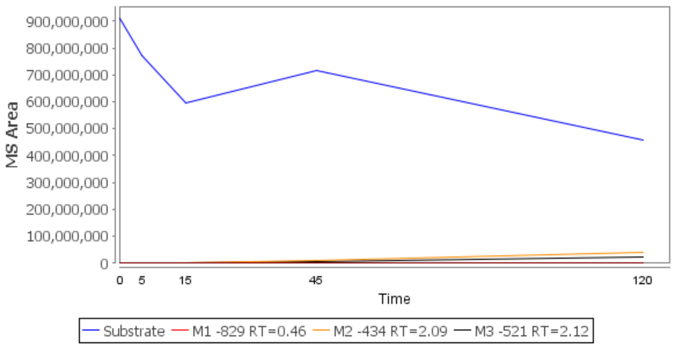

Fragmentation

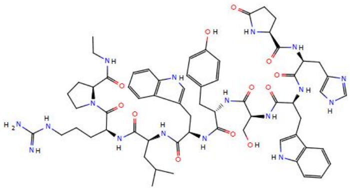

Deslorelin

MS (+) FT

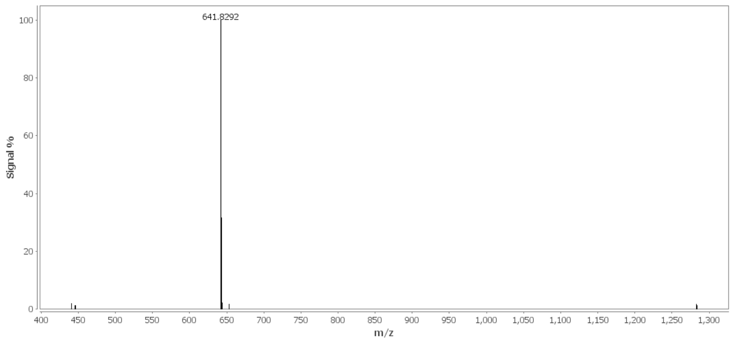

MS (+) FT

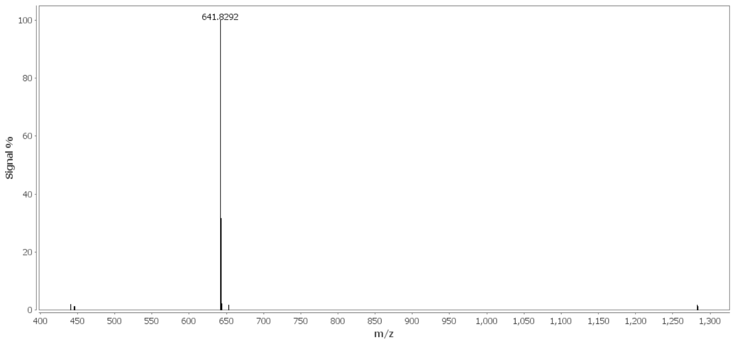

MS2 (+) FT activ = HCD:ce =

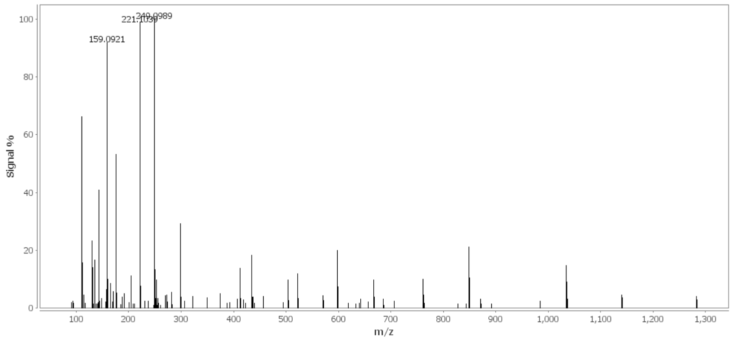

MS2 (+) FT activ = HCD:ce =

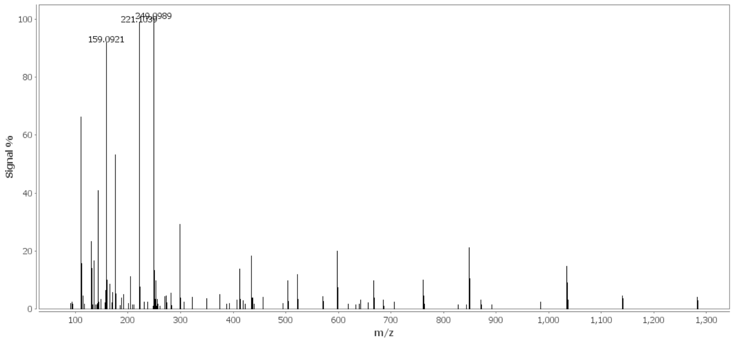

Metabolite: Substrate

Type	score	sub. m/z observed	sub. m/z calculated	sub ppm		met. m/z observed	met. m/z calculated	met. ppm
MATCH	14.6	1282.6502	1282.6480	-1.69	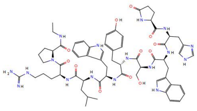 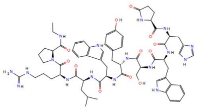	1282.6502	1282.6480	-1.69
MATCH	101.2	1282.6483	1282.6480	-0.25	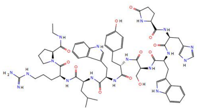 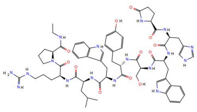	1282.6483	1282.6480	-0.25
MATCH	5.2	871.3520	871.3522	0.26	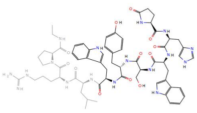 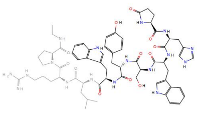	871.3520	871.3522	0.26

Metabolite: Substrate

Type	score	sub. m/z observed	sub. m/z calculated	sub ppm		met. m/z observed	met. m/z calculated	met. ppm
MATCH	10.1	843.3578	843.3573	-0.60	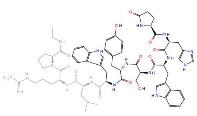 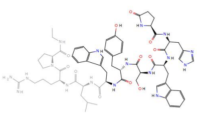	843.3578	843.3573	-0.60
MATCH	8.3	685.2723	685.2729	0.84	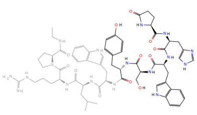 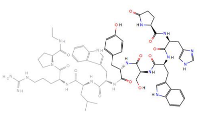	685.2723	685.2729	0.84
MATCH	101.6	657.2783	657.2780	-0.48	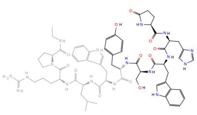 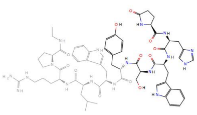	657.2783	657.2780	-0.48
MATCH	200.0	641.8294	641.8276	-2.73	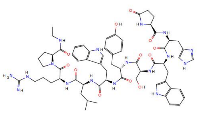 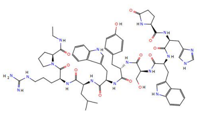	641.8294	641.8276	-2.73
MATCH	15.5	641.8272	641.8276	0.62	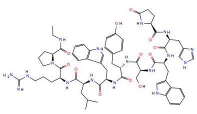 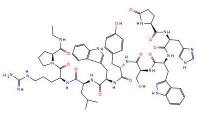	641.8272	641.8276	0.62
MATCH	49.6	598.3828	598.3824	-0.67	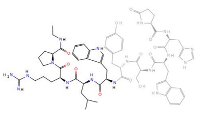 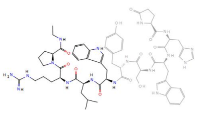	598.3828	598.3824	-0.67
MATCH	11.1	504.1992	504.1990	-0.48	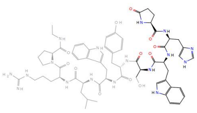 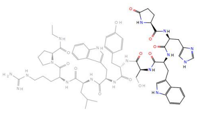	504.1992	504.1990	-0.48
MATCH	11.6	494.2143	494.2146	0.63	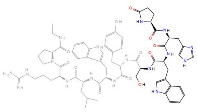 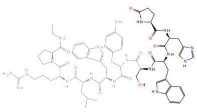	494.2143	494.2146	0.63
MATCH	11.5	456.2718	456.2718	-0.06	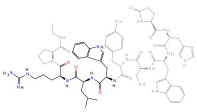 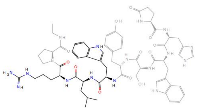	456.2718	456.2718	-0.06

Metabolite: Substrate

Type	score	sub. m/z observed	sub. m/z calculated	sub ppm			met. m/z observed	met. m/z calculated	met. ppm
MATCH	101.1	428.2216	428.2208	-1.79	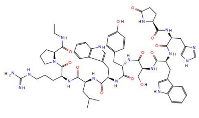	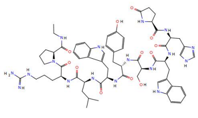	428.2216	428.2208	-1.79
MATCH	40.5	412.3032	412.3031	-0.42	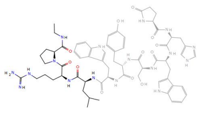	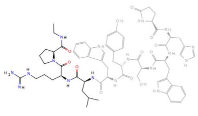	412.3032	412.3031	-0.42
MATCH	5.6	350.1502	350.1499	-0.75	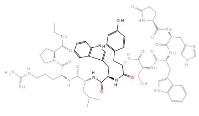	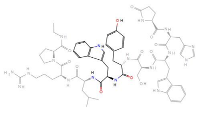	350.1502	350.1499	-0.75
MATCH	39.6	299.2193	299.2190	-1.00	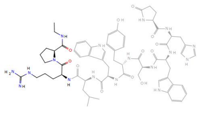	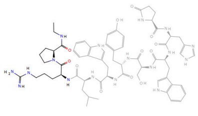	299.2193	299.2190	-1.00
MATCH	9.4	282.1923	282.1925	0.43	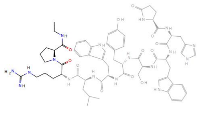	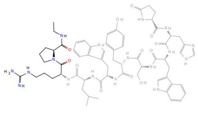	282.1923	282.1925	0.43
MATCH	10.9	272.1757	272.1757	-0.02	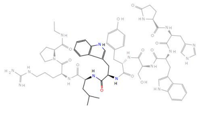	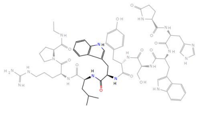	272.1757	272.1757	-0.02
MATCH	13.0	270.1927	270.1925	-0.82	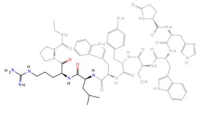	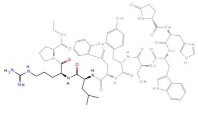	270.1927	270.1925	-0.82
MATCH	18.2	261.1136	261.1164	10.63	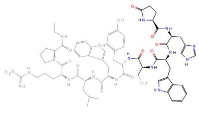	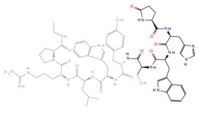	261.1136	261.1164	10.63
MATCH	7.6	255.1491	255.1492	0.37	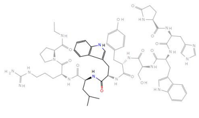	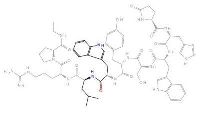	255.1491	255.1492	0.37

Metabolite: Substrate

Type	score	sub. m/z observed	sub. m/z calculated	sub ppm			met. m/z observed	met. m/z calculated	met. ppm
MATCH	31.2	253.1660	253.1659	-0.45	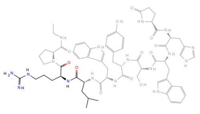	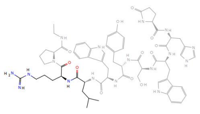	253.1660	253.1659	-0.45
MATCH	181.4	249.0984	249.0982	-0.71	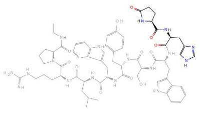	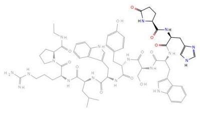	249.0984	249.0982	-0.71
MATCH	3.8	237.1350	237.1346	-1.73	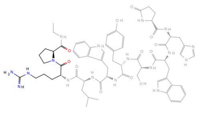	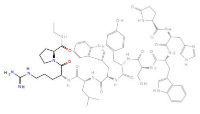	237.1350	237.1346	-1.73
MATCH	179.9	221.1035	221.1033	-1.09	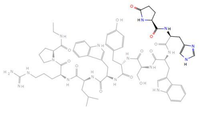	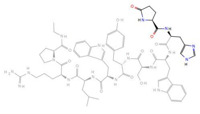	221.1035	221.1033	-1.09
MISMATCH	-33.6	191.0819	191.0871	27.50	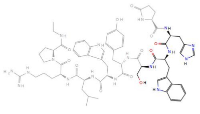	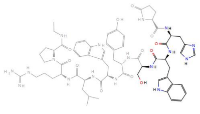	191.0819	191.0871	27.50
MATCH	8.6	187.0867	187.0866	-0.51	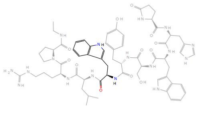	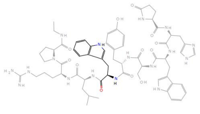	187.0867	187.0866	-0.51
MATCH	8.6	187.0867	187.0866	-0.51	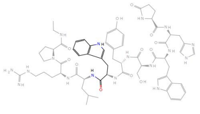	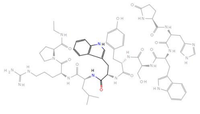	187.0867	187.0866	-0.51
MATCH	23.6	170.0603	170.0600	-1.65	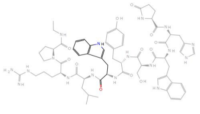	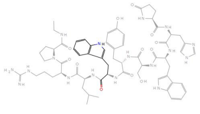	170.0603	170.0600	-1.65
MISMATCH	-3.4	169.0761	169.0846	50.26	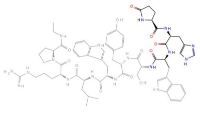	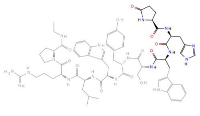	169.0761	169.0846	50.26

Metabolite: Substrate

Type	score	sub. m/z observed	sub. m/z calculated	sub ppm			met. m/z observed	met. m/z calculated	met. ppm
MATCH	13.7	166.0613	166.0611	-1.35	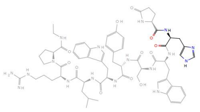	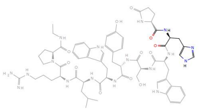	166.0613	166.0611	-1.35
MATCH	172.3	159.0919	159.0917	-1.26	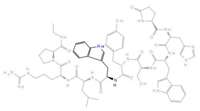	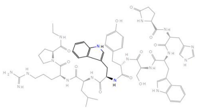	159.0919	159.0917	-1.26
MATCH	110.1	159.0919	159.0917	-1.26	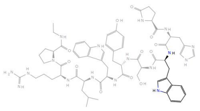	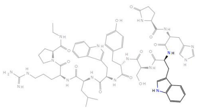	159.0919	159.0917	-1.26
MATCH	13.4	157.1086	157.1084	-1.67	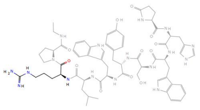	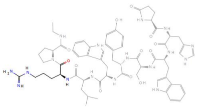	157.1086	157.1084	-1.67
MATCH	4.4	144.0809	144.0808	-0.87	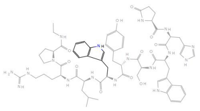	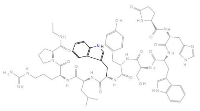	144.0809	144.0808	-0.87
MATCH	76.8	143.1181	143.1179	-1.81	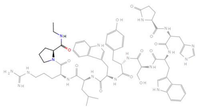	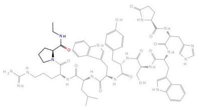	143.1181	143.1179	-1.81
MATCH	2.9	140.0820	140.0818	-1.21	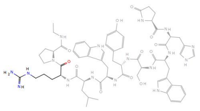	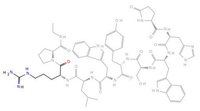	140.0820	140.0818	-1.21
MISMATCH	77.7	136.0760	136.0757	-2.20	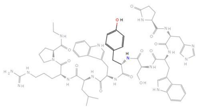	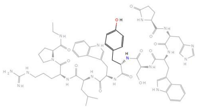	136.0760	136.0757	-2.20
MATCH	10.3	115.0871	115.0866	-4.15	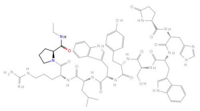	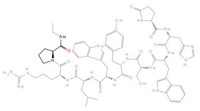	115.0871	115.0866	-4.15

Metabolite: Substrate

Type	score	sub. m/z observed	sub. m/z calculated	sub ppm		met. m/z observed	met. m/z calculated	met. ppm
MATCH	35.3	112.0874	112.0869	-4.61	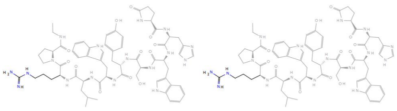	112.0874	112.0869	-4.61
MATCH	172.0	110.0718	110.0713	-4.82	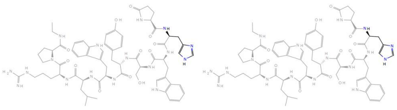	110.0718	110.0713	-4.82
MATCH	6.3	91.0550	91.0522	-31.0	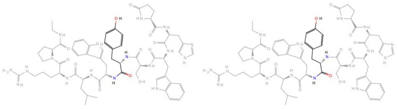	91.0550	91.0522	-31.0

MS (+) FT

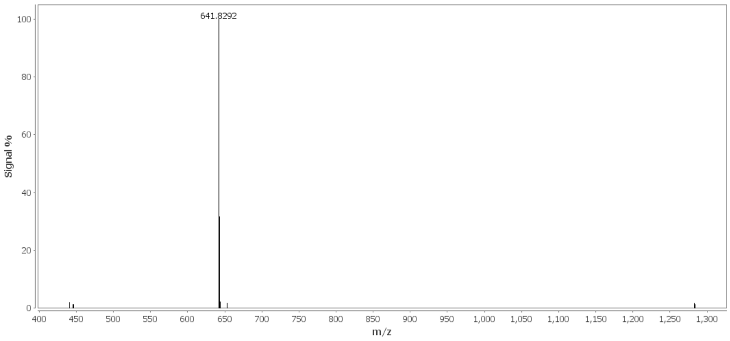

MS (+) FT

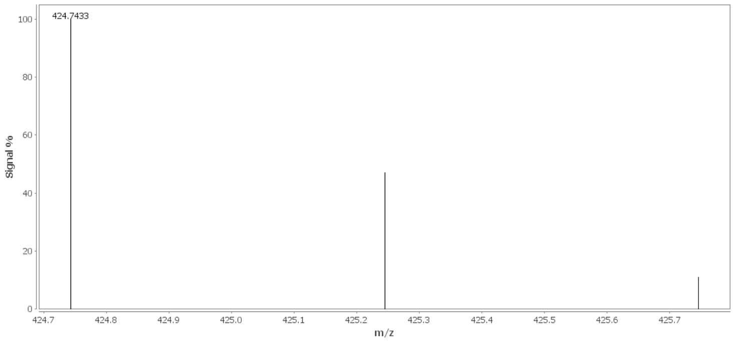

MS2 (+) FT activ = HCD:ce =

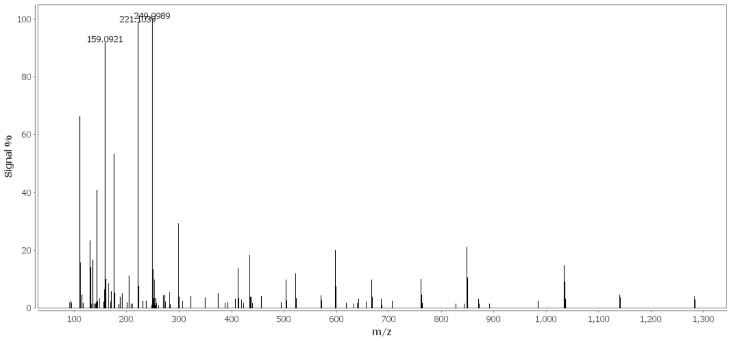

MS2 (+) FT activ = HCD:ce =

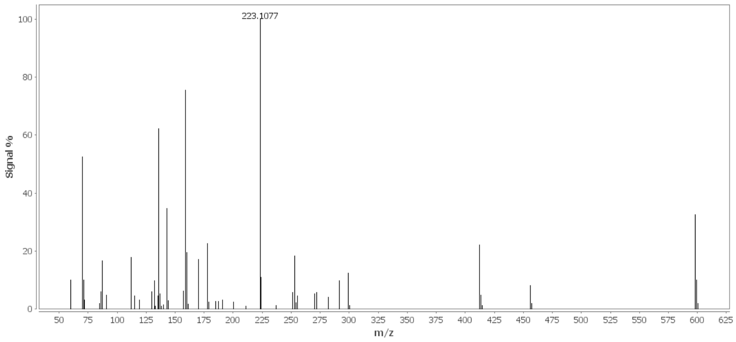

Metabolite: M2 -434 RT=2.09

Type	score	sub. m/z observed	sub. m/z calculated	sub ppm		met. m/z observed	met. m/z calculated	met. ppm
MATCH	101.1	428.2216	428.2208	-1.79	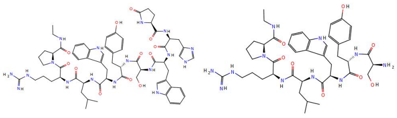	424.7433	424.7425	-1.77

Metabolite: M2 -434 RT=2.09

Type	score	sub. m/z observed	sub. m/z calculated	sub ppm			met. m/z observed	met. m/z calculated	met. ppm
						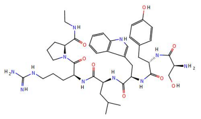	424.7433	424.7425	-1.77
						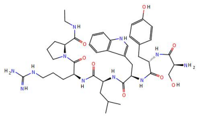	424.7433	424.7425	-1.77
MATCH	200.0	641.8294	641.8276	-2.73	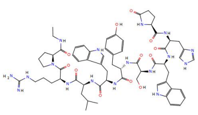	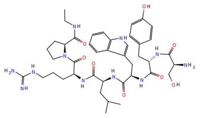	424.7433	424.7425	-1.77
						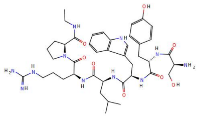	424.7433	424.7425	-1.77
						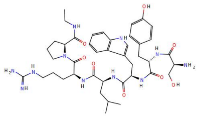	424.7433	424.7425	-1.77
MATCH	101.2	1282.6483	1282.6480	-0.25	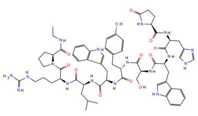	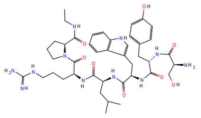	424.7433	424.7425	-1.77
						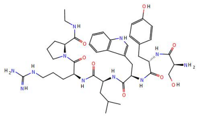	424.7433	424.7425	-1.77
						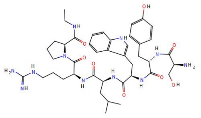	424.7433	424.7425	-1.77
MATCH	6.3	91.0550	91.0522	-31.0	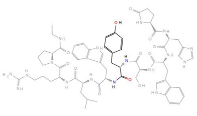	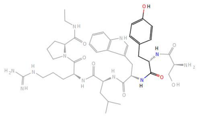	91.0548	91.0522	-28.3

Metabolite: M2 -434 RT=2.09

Type	score	sub. m/z observed	sub. m/z calculated	sub ppm		met. m/z observed	met. m/z calculated	met. ppm
MATCH	35.3	112.0874	112.0869	-4.61	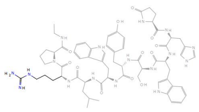 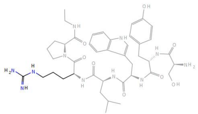	112.0874	112.0869	-3.97
MATCH	10.3	115.0871	115.0866	-4.15	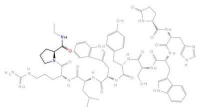 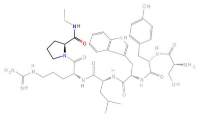	115.0872	115.0866	-5.07
MATCH	77.7	136.0760	136.0757	-2.20	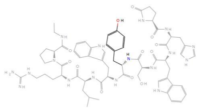 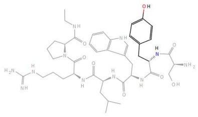	136.0759	136.0757	-1.37
MATCH	2.9	140.0820	140.0818	-1.21	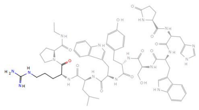 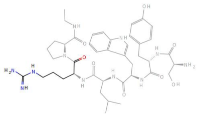	140.0770	140.0818	34.70
MATCH	76.8	143.1181	143.1179	-1.81	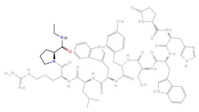 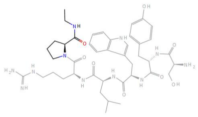	143.1181	143.1179	-1.20
MATCH	4.4	144.0809	144.0808	-0.87	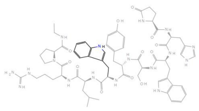 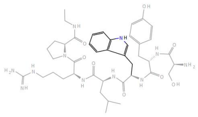	144.0809	144.0808	-0.56
MATCH	13.4	157.1086	157.1084	-1.67	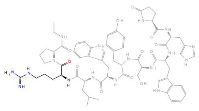 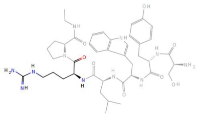	157.1084	157.1084	0.22
MATCH	172.3	159.0919	159.0917	-1.26	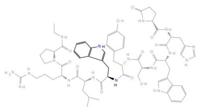 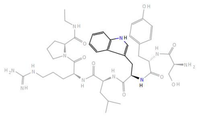	159.0917	159.0917	-0.39
MATCH	23.6	170.0603	170.0600	-1.65	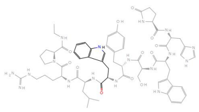 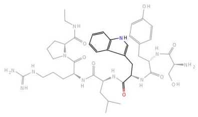	170.0600	170.0600	0.28

Metabolite: M2 -434 RT=2.09

Type	score	sub. m/z observed	sub. m/z calculated	sub ppm		met. m/z observed	met. m/z calculated	met. ppm
MATCH	6.5	187.0867	187.0866	-0.51	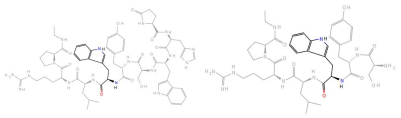	187.0867	187.0866	-0.84
MATCH	6.5	187.0867	187.0866	-0.51	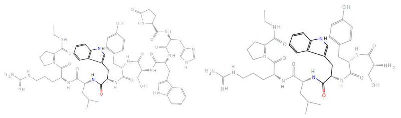	187.0867	187.0866	-0.84
MATCH	3.5	237.1350	237.1346	-1.73	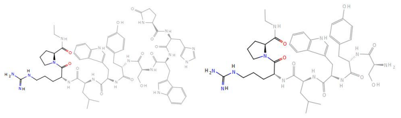	237.1346	237.1346	-0.11
MATCH	28.3	253.1660	253.1659	-0.45	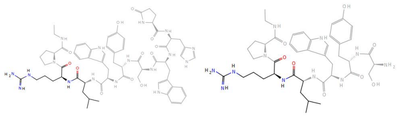	253.1658	253.1659	0.38
MATCH	7.6	255.1491	255.1492	0.37	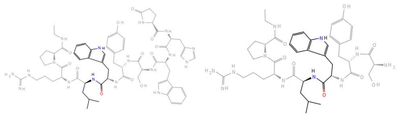	255.1493	255.1492	-0.52
MATCH	18.2	261.1136	261.1164	10.63	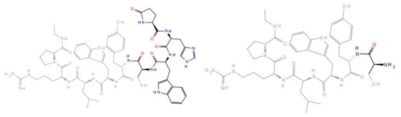	87.0559	87.0553	-7.03
MATCH	9.0	270.1927	270.1925	-0.82	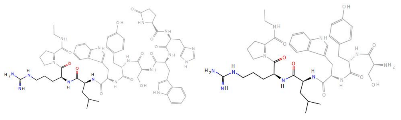	270.1930	270.1925	-1.89
MATCH	10.9	272.1757	272.1757	-0.02	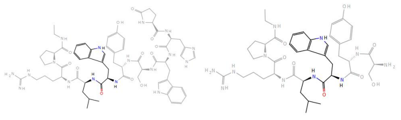	272.1750	272.1757	2.65
MATCH	9.4	282.1923	282.1925	0.43	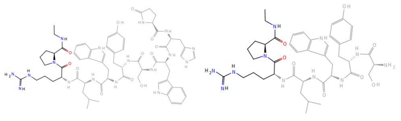	282.1922	282.1925	1.00

Metabolite: M2 -434 RT=2.09

Type	score	sub. m/z observed	sub. m/z calculated	sub ppm			met. m/z observed	met. m/z calculated	met. ppm
MATCH	39.6	299.2193	299.2190	-1.00	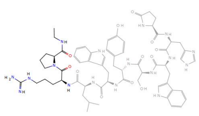	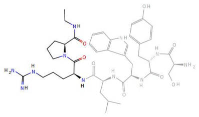	299.2190	299.2190	0.06
MATCH	35.2	412.3032	412.3031	-0.42	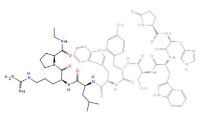	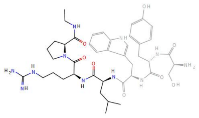	412.3026	412.3031	1.04
MATCH	11.5	456.2718	456.2718	-0.06	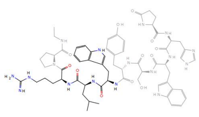	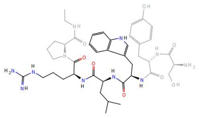	456.2723	456.2718	-1.19
MATCH	11.6	494.2143	494.2146	0.63	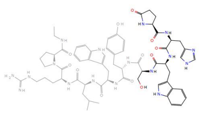	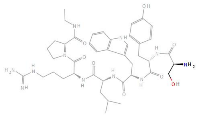	60.0453	60.0444	-15.6
MATCH	11.1	504.1992	504.1990	-0.48	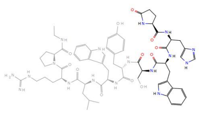	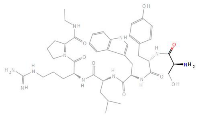	70.0295	70.0287	-11.2
MATCH	49.6	598.3828	598.3824	-0.67	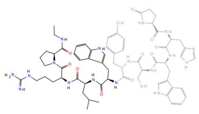	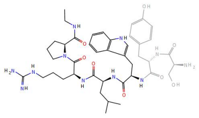	598.3817	598.3824	1.19
MATCH	101.6	657.2783	657.2780	-0.48	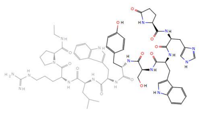	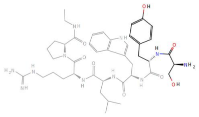	223.1077	223.1077	-0.05
MATCH	8.3	685.2723	685.2729	0.84	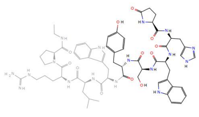	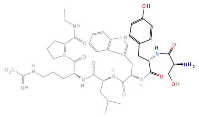	251.1029	251.1026	-0.90
MET_MATCH						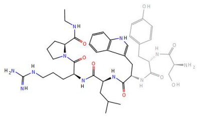	291.1818	291.1816	-1.00

MS (+) FT

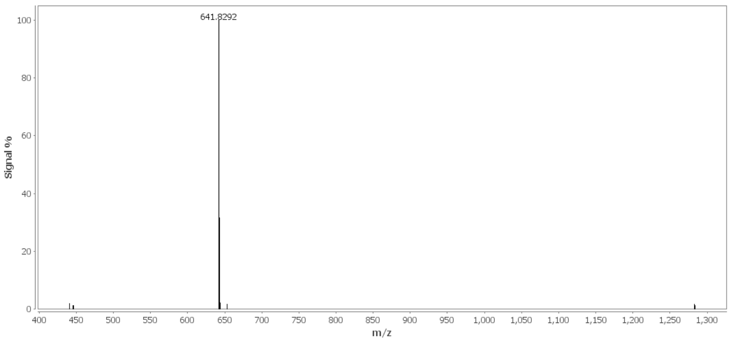

MS (+) FT

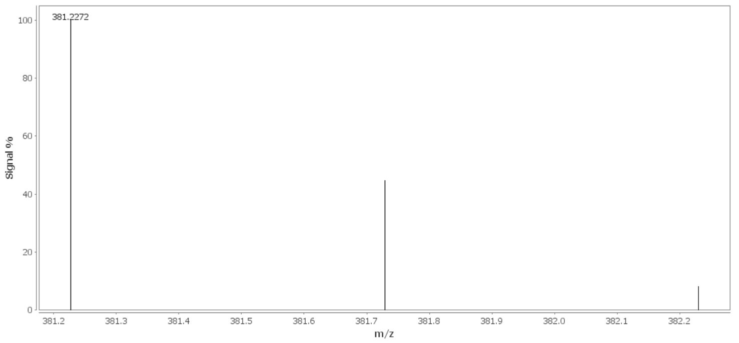

MS2 (+) FT activ = HCD:ce =

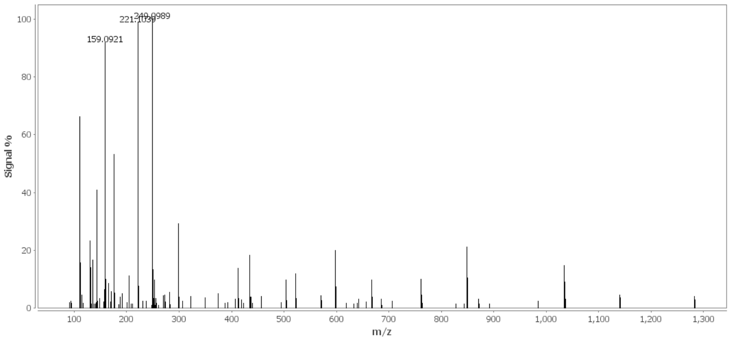

MS2 (+) FT activ = HCD:ce =

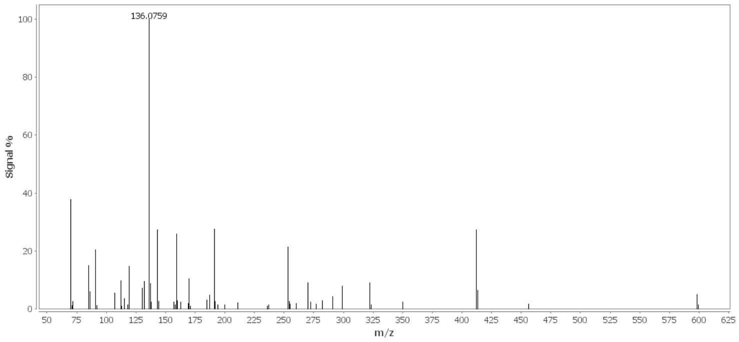

Metabolite: M3 -521 RT=2.12

Type	score	sub. m/z observed	sub. m/z calculated	sub ppm		met. m/z observed	met. m/z calculated	met. ppm
MATCH	101.1	428.2216	428.2208	-1.79	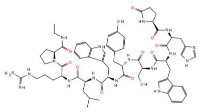 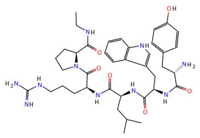	381.2272	381.2265	-1.84
					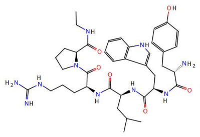	381.2272	381.2265	-1.84
					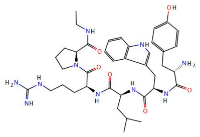	381.2272	381.2265	-1.84
MATCH	200.0	641.8294	641.8276	-2.73	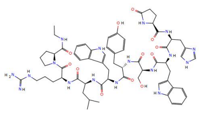 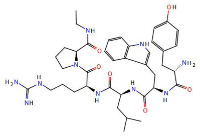	381.2272	381.2265	-1.84
					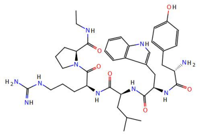	381.2272	381.2265	-1.84

Metabolite: M3 -521 RT=2.12

Type	score	sub. m/z observed	sub. m/z calculated	sub ppm			met. m/z observed	met. m/z calculated	met. ppm
						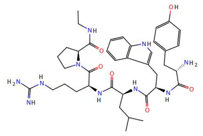	381.2272	381.2265	-1.84
MATCH	101.2	1282.6483	1282.6480	-0.25	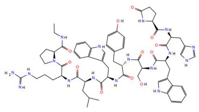	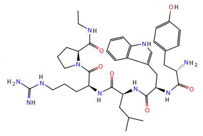	381.2272	381.2265	-1.84
						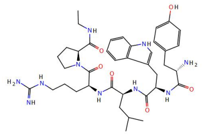	381.2272	381.2265	-1.84
						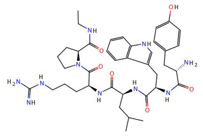	381.2272	381.2265	-1.84
MATCH	27.4	112.0874	112.0869	-4.61	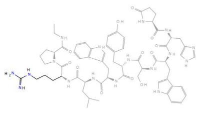	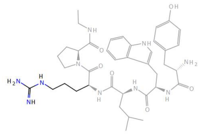	112.0874	112.0869	-4.10
MATCH	9.3	115.0871	115.0866	-4.15	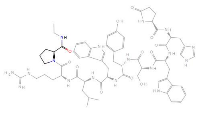	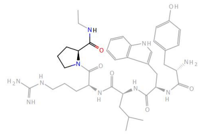	115.0871	115.0866	-4.50
MATCH	69.4	143.1181	143.1179	-1.81	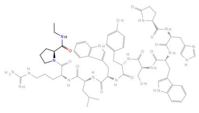	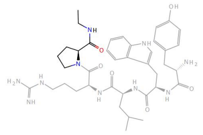	143.1181	143.1179	-1.61
MATCH	4.3	144.0809	144.0808	-0.87	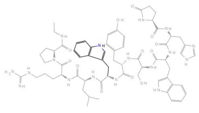	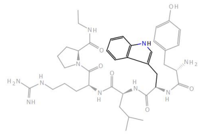	144.0810	144.0808	-1.43
MATCH	9.5	157.1086	157.1084	-1.67	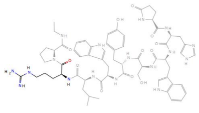	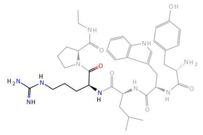	157.1084	157.1084	-0.18

Metabolite: M3 -521 RT=2.12

Type	score	sub. m/z observed	sub. m/z calculated	sub ppm		met. m/z observed	met. m/z calculated	met. ppm
MATCH	122.7	159.0919	159.0917	-1.26	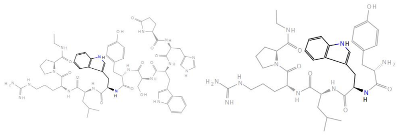	159.0919	159.0917	-1.18
MATCH	16.9	170.0603	170.0600	-1.65	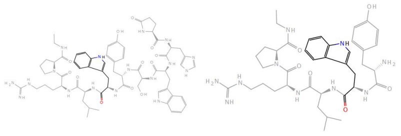	170.0603	170.0600	-1.41
MATCH	8.6	187.0867	187.0866	-0.51	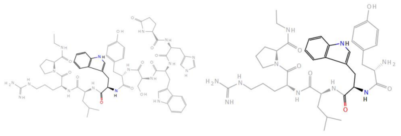	187.0869	187.0866	-1.56
MATCH	8.6	187.0867	187.0866	-0.51	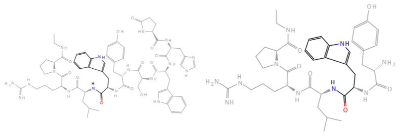	187.0869	187.0866	-1.56
MATCH	3.8	237.1350	237.1346	-1.73	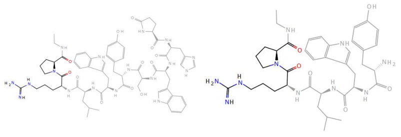	237.1349	237.1346	-1.46
MATCH	31.2	253.1660	253.1659	-0.45	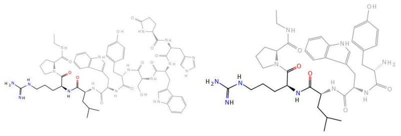	253.1660	253.1659	-0.55
MATCH	4.8	255.1491	255.1492	0.37	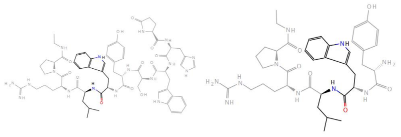	255.1498	255.1492	-2.53
MATCH	13.0	270.1927	270.1925	-0.82	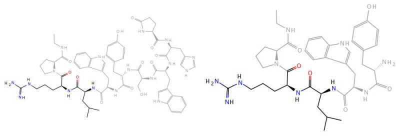	270.1927	270.1925	-0.88
MATCH	7.8	272.1757	272.1757	-0.02	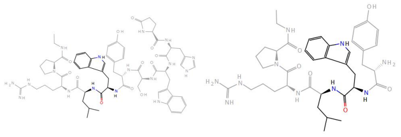	272.1768	272.1757	-3.74

Metabolite: M3 -521 RT=2.12

Type	score	sub. m/z observed	sub. m/z calculated	sub ppm			met. m/z observed	met. m/z calculated	met. ppm
MATCH	8.4	282.1923	282.1925	0.43	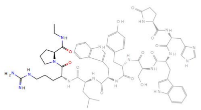	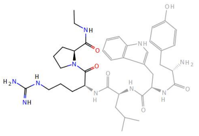	282.1927	282.1925	-0.83
MATCH	35.0	299.2193	299.2190	-1.00	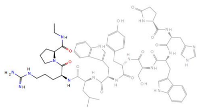	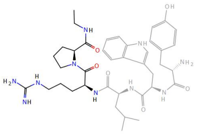	299.2190	299.2190	-0.16
MATCH	5.6	350.1502	350.1499	-0.75	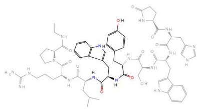	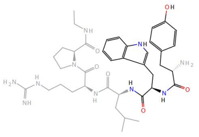	350.1493	350.1499	1.85
						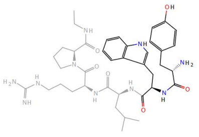	350.1493	350.1499	1.85
MATCH	40.5	412.3032	412.3031	-0.42	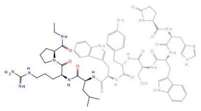	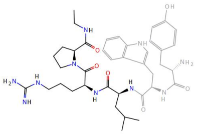	412.3032	412.3031	-0.36
MATCH	5.1	456.2718	456.2718	-0.06	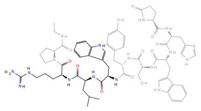	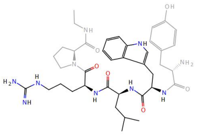	456.2725	456.2718	-1.55
MATCH	21.9	598.3828	598.3824	-0.67	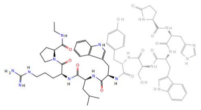	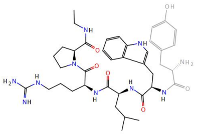	598.3822	598.3824	0.23
MATCH	101.6	657.2783	657.2780	-0.48	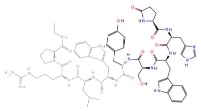	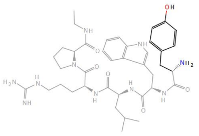	136.0759	136.0757	-1.79
MATCH	10.1	843.3578	843.3573	-0.60	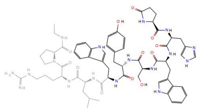	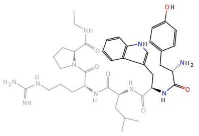	322.1550	322.1550	-0.11

Metabolite: M3 -521 RT=2.12

Type	score	sub. m/z observed	sub. m/z calculated	sub ppm		met. m/z observed	met. m/z calculated	met. ppm
MATCH	5.2	871.3520	871.3522	0.26	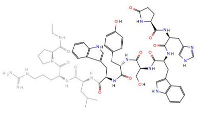 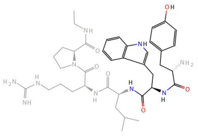	350.1493	350.1499	1.85
					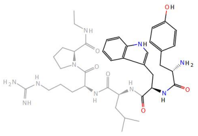	350.1493	350.1499	1.85
MISMATCH	-3.4	169.0761	169.0846	50.26	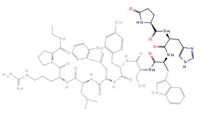	169.0764	169.0764	0.00
MISMATCH	-33.6	191.0819	191.0871	27.50	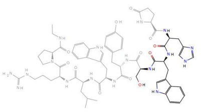	191.0817	191.0817	0.00
MET_MATCH					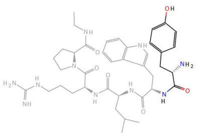	91.0549	91.0522	-29.8
MET_MATCH					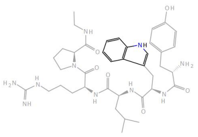	118.0658	118.0651	-5.35
MET_MATCH					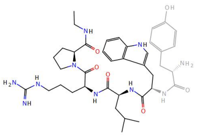	291.1821	291.1816	-1.72

MS (+) FT

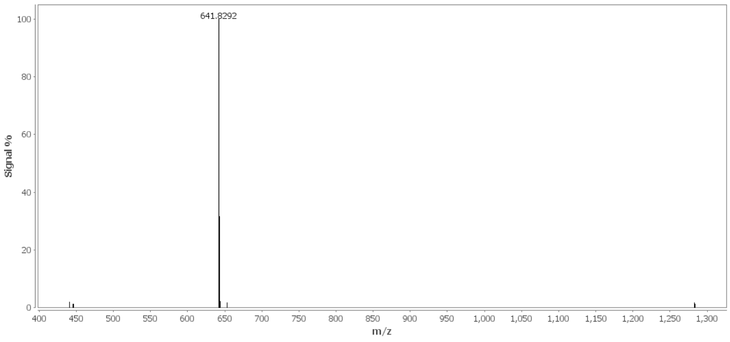

MS (+) FT

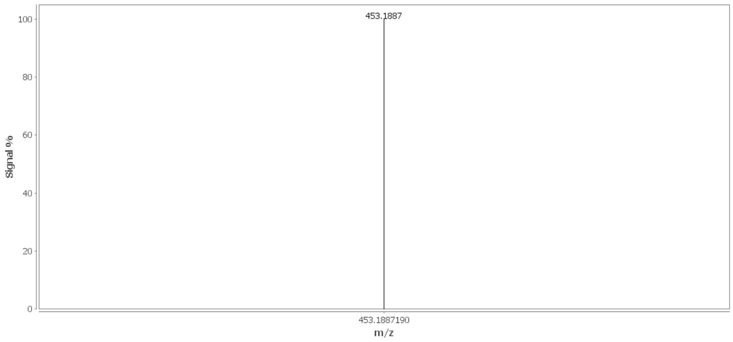

MS2 (+) FT activ = HCD:ce =

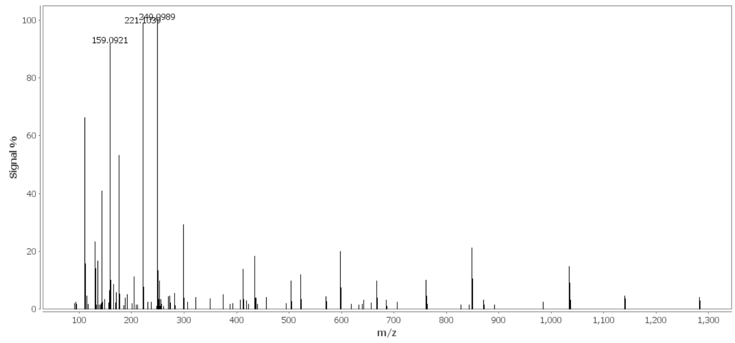

MS2 (+) FT activ = HCD:ce =

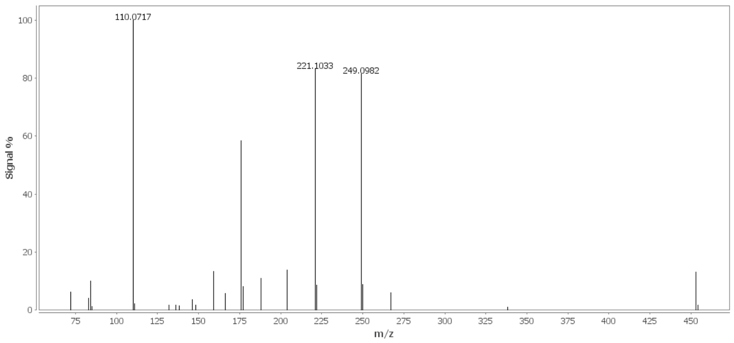

Metabolite: M1 -829 RT=0.46

Type	score	sub. m/z observed	sub. m/z calculated	sub ppm			met. m/z observed	met. m/z calculated	met. ppm
MATCH	101.1	428.2216	428.2208	-1.79	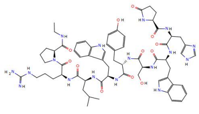	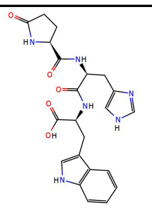	453.1887	453.1881	-1.38
						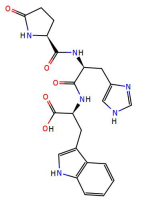	453.1887	453.1881	-1.38
						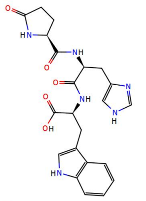	453.1887	453.1881	-1.38
MATCH	200.0	641.8294	641.8276	-2.73	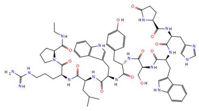	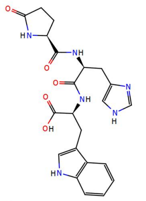	453.1887	453.1881	-1.38
						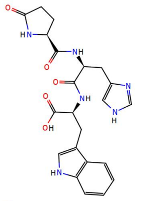	453.1887	453.1881	-1.38
						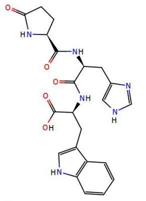	453.1887	453.1881	-1.38
MATCH	101.2	1282.6483	1282.6480	-0.25	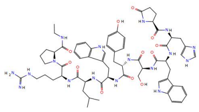	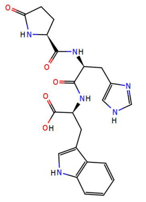	453.1887	453.1881	-1.38

Metabolite: M1 -829 RT=0.46

Type	score	sub. m/z observed	sub. m/z calculated	sub ppm			met. m/z observed	met. m/z calculated	met. ppm
						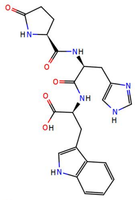	453.1887	453.1881	-1.38
						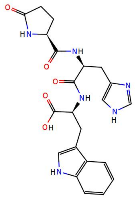	453.1887	453.1881	-1.38
MATCH	172.0	110.0718	110.0713	-4.82	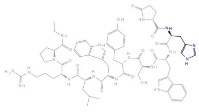	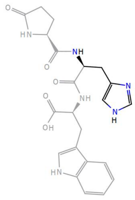	110.0717	110.0713	-3.81
MATCH	110.1	159.0919	159.0917	-1.26	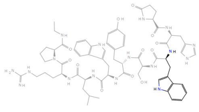	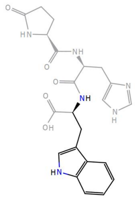	159.0915	159.0917	0.87
MATCH	13.7	166.0613	166.0611	-1.35	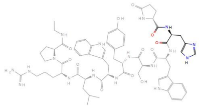	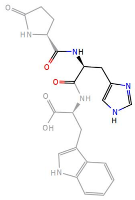	166.0610	166.0611	0.32
MATCH	179.9	221.1035	221.1033	-1.09	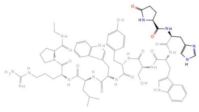	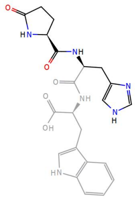	221.1033	221.1033	0.00
MATCH	181.4	249.0984	249.0982	-0.71	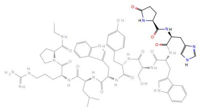	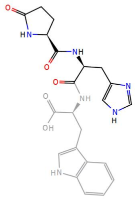	249.0982	249.0982	0.17
MATCH	15.5	641.8272	641.8276	0.62	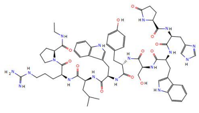	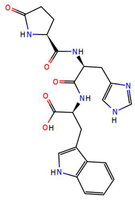	453.1878	453.1881	0.70
						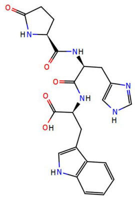	453.1878	453.1881	0.70

Metabolite: M1 -829 RT=0.46

Type	score	sub. m/z observed	sub. m/z calculated	sub ppm			met. m/z observed	met. m/z calculated	met. ppm
MATCH	14.6	1282.6502	1282.6480	-1.69	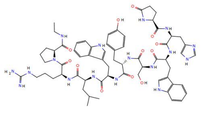	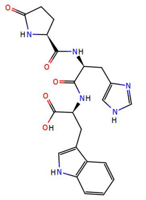	453.1878	453.1881	0.70
						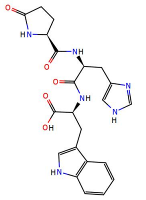	453.1878	453.1881	0.70
MISMATCH	-17.2	136.0760	136.0757	-2.20	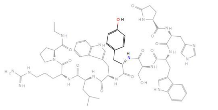		136.0758	136.0758	0.00
MET_MATCH						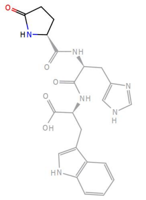	84.0451	84.0444	-8.69
MET_MATCH						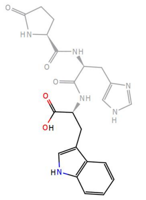	188.0703	188.0706	1.64
