# Supplementary material for: Software-aided approach to investigate peptide structure and metabolic susceptibility of amide bonds in peptide drugs based on high resolution mass spectrometry
Source: PLoS One. 2017 Nov 1;12(11):e0186461. doi: 10.1371/journal.pone.0186461 (PMC5665424; doi:10.1371/journal.pone.0186461)
Supplement: S1 File — (ZIP) [file pone.0186461.s007.zip › SFiles/S12_File.pdf]

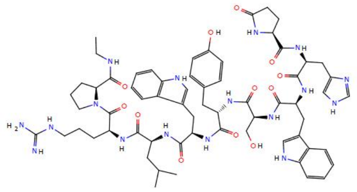

Deslorelin

Property name	Property value
Time	0min, 5min, 15min, 45min, 120min
Instrument	ThermoQAPlus
Acquisition Mode	ddMS2
Matrix	pepsin

Chromatograms

Time=0min

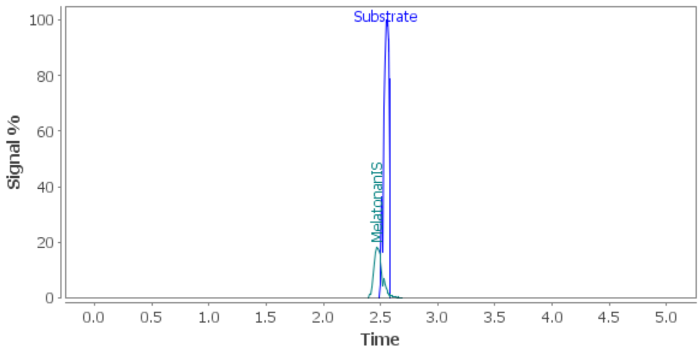

Time=5min

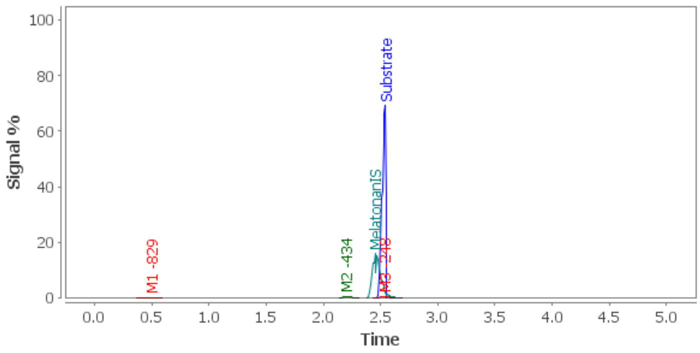

Time=15min

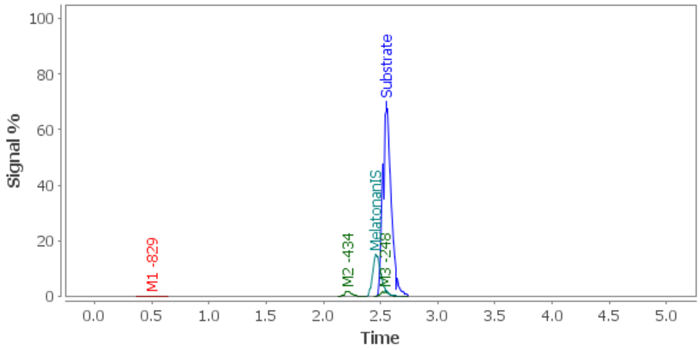

Time=45min

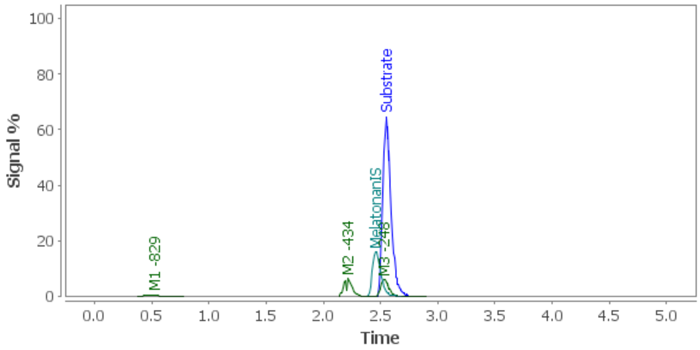

Time=120min

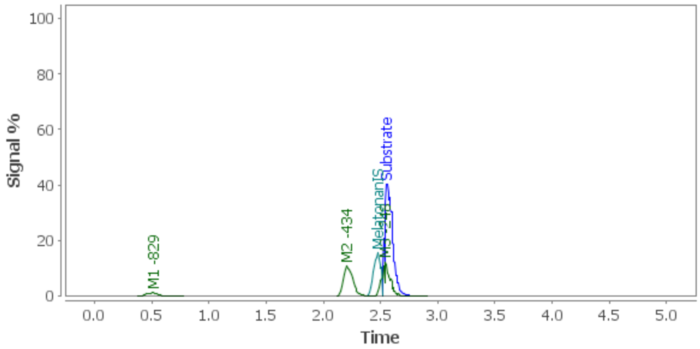

Custom Charts

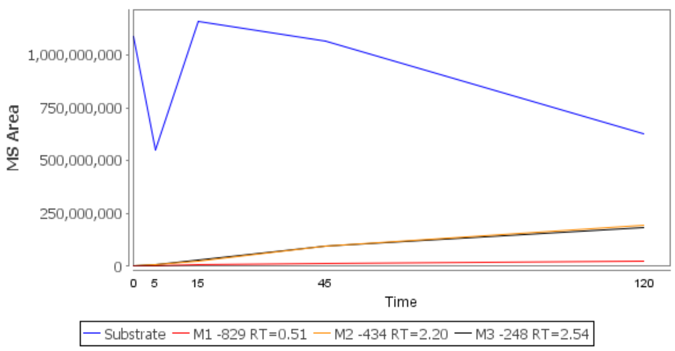

Fragmentation

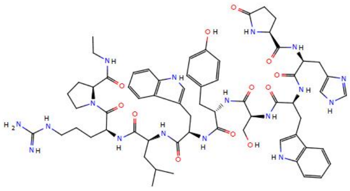

Deslorelin

MS (+) FT

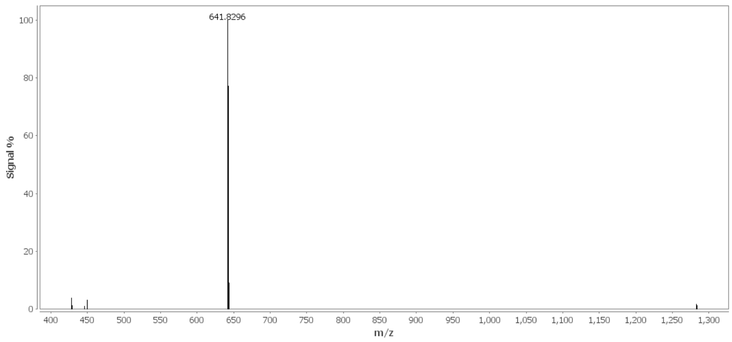

MS (+) FT

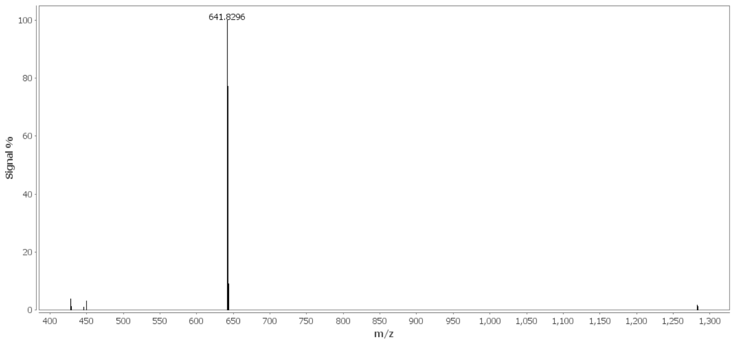

MS2 (+) FT activ = HCD:ce =

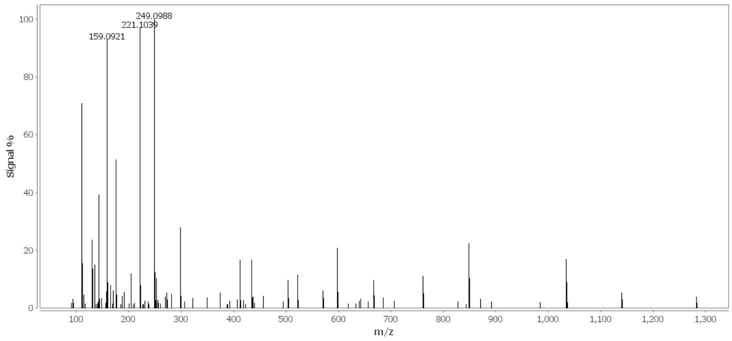

MS2 (+) FT activ = HCD:ce =

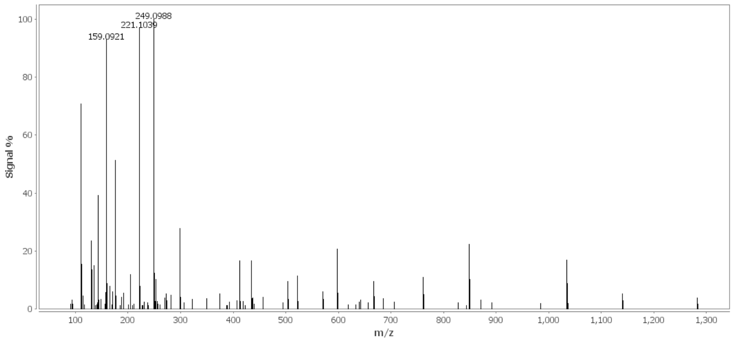

Metabolite: Substrate

Type	score	sub. m/z observed	sub. m/z calculated	sub ppm		met. m/z observed	met. m/z calculated	met. ppm
MATCH	16.9	1282.6527	1282.6480	-3.71	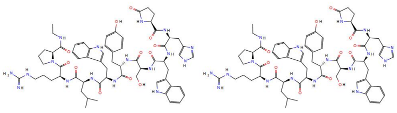	1282.6527	1282.6480	-3.71
MISMATCH	101.6	1282.6517	1282.6480	-2.87	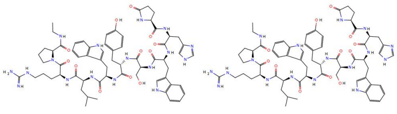	1282.6517	1282.6480	-2.87
MATCH	33.1	848.4802	848.4777	-2.96	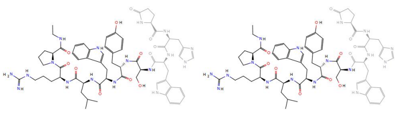	848.4802	848.4777	-2.96

Metabolite: Substrate

Type	score	sub. m/z observed	sub. m/z calculated	sub ppm		met. m/z observed	met. m/z calculated	met. ppm
MATCH	21.6	761.4484	761.4457	-3.48	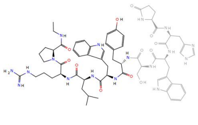 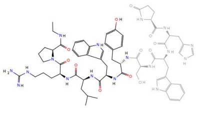	761.4484	761.4457	-3.48
MATCH	9.0	685.2719	685.2729	1.49	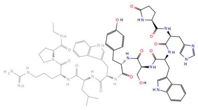 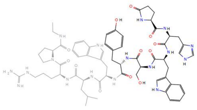	685.2719	685.2729	1.49
MATCH	13.2	667.2625	667.2623	-0.33	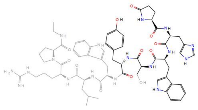 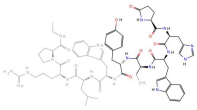	667.2625	667.2623	-0.33
MATCH	102.1	657.2751	657.2780	4.32	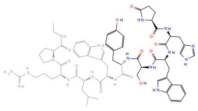 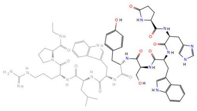	657.2751	657.2780	4.32
MISMATCH	200.0	641.8296	641.8276	-3.10	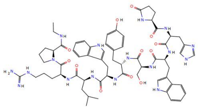 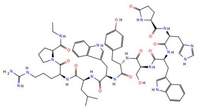	641.8296	641.8276	-3.10
MATCH	16.4	641.8288	641.8276	-1.74	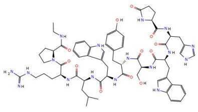 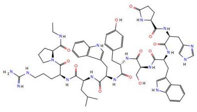	641.8288	641.8276	-1.74
MATCH	53.9	598.3840	598.3824	-2.76	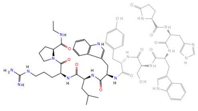 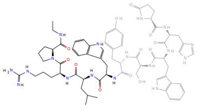	598.3840	598.3824	-2.76
MATCH	15.3	522.2109	522.2096	-2.58	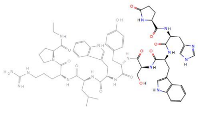 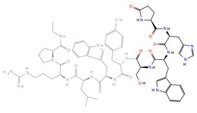	522.2109	522.2096	-2.58
MATCH	14.0	504.2007	504.1990	-3.40	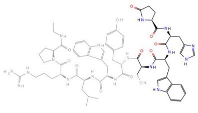 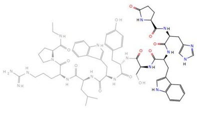	504.2007	504.1990	-3.40

Metabolite: Substrate

Type	score	sub. m/z observed	sub. m/z calculated	sub ppm			met. m/z observed	met. m/z calculated	met. ppm
MATCH	13.8	494.2130	494.2146	3.33	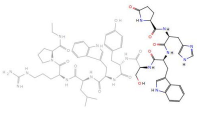	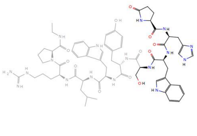	494.2130	494.2146	3.33
MATCH	12.6	456.2740	456.2718	-4.81	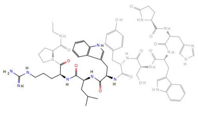	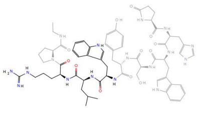	456.2740	456.2718	-4.81
MATCH	4.1	439.2452	439.2452	-0.07	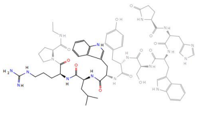	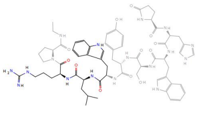	439.2452	439.2452	-0.07
MATCH	21.6	435.1786	435.1775	-2.41	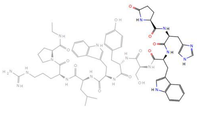	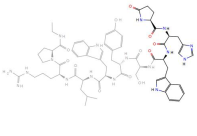	435.1786	435.1775	-2.41
MISMATCH	103.9	428.2220	428.2208	-2.62	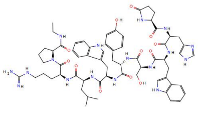	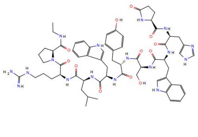	428.2220	428.2208	-2.62
MATCH	38.0	412.3042	412.3031	-2.70	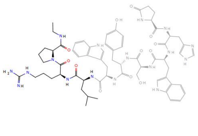	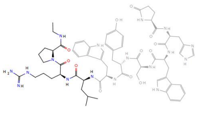	412.3042	412.3031	-2.70
MATCH	102.9	407.1849	407.1826	-5.51	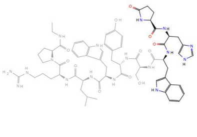	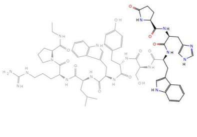	407.1849	407.1826	-5.51
MATCH	5.5	350.1511	350.1499	-3.25	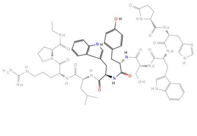	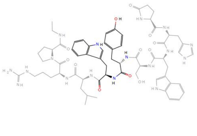	350.1511	350.1499	-3.25
MATCH	5.5	350.1511	350.1499	-3.25	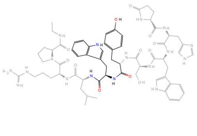	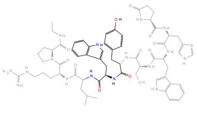	350.1511	350.1499	-3.25

Metabolite: Substrate

Type	score	sub. m/z observed	sub. m/z calculated	sub ppm		met. m/z observed	met. m/z calculated	met. ppm
MATCH	6.7	322.1557	322.1550	-2.31	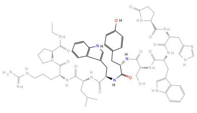 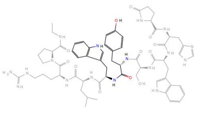	322.1557	322.1550	-2.31
MATCH	3.3	300.1710	300.1707	-1.27	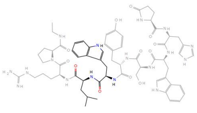 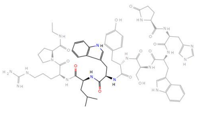	300.1710	300.1707	-1.27
MATCH	3.3	300.1710	300.1707	-1.27	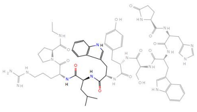 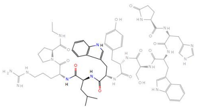	300.1710	300.1707	-1.27
MATCH	41.8	299.2199	299.2190	-2.91	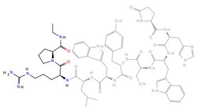 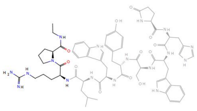	299.2199	299.2190	-2.91
MATCH	11.4	282.1925	282.1925	-0.27	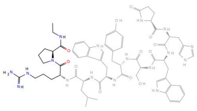 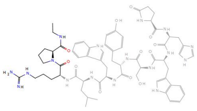	282.1925	282.1925	-0.27
MATCH	6.9	274.1194	274.1186	-2.78	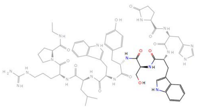 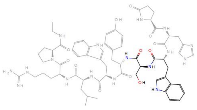	274.1194	274.1186	-2.78
MATCH	11.5	272.1760	272.1757	-0.81	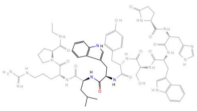 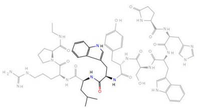	272.1760	272.1757	-0.81
MATCH	10.4	270.1928	270.1925	-1.17	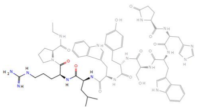 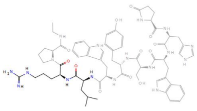	270.1928	270.1925	-1.17
MATCH	21.6	261.1145	261.1164	7.47	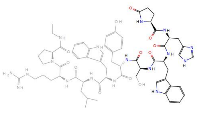 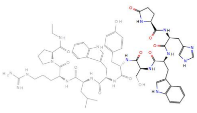	261.1145	261.1164	7.47

Metabolite: Substrate

Type	score	sub. m/z observed	sub. m/z calculated	sub ppm			met. m/z observed	met. m/z calculated	met. ppm
MATCH	8.0	255.1490	255.1492	0.80	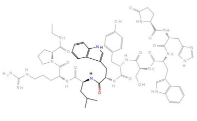	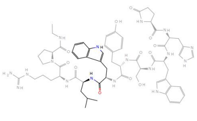	255.1490	255.1492	0.80
MATCH	29.4	253.1663	253.1659	-1.65	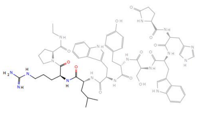	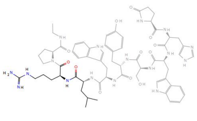	253.1663	253.1659	-1.65
MATCH	179.4	249.0988	249.0982	-2.32	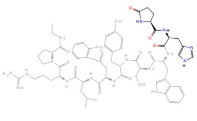	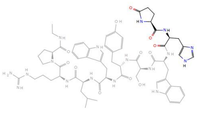	249.0988	249.0982	-2.32
MATCH	4.5	237.1351	237.1346	-2.24	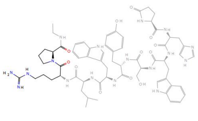	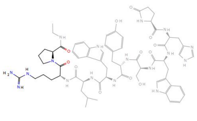	237.1351	237.1346	-2.24
MATCH	178.0	221.1039	221.1033	-2.69	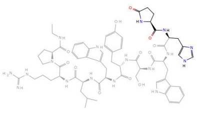	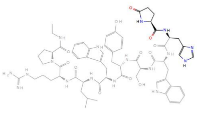	221.1039	221.1033	-2.69
MATCH	9.0	187.0868	187.0866	-1.34	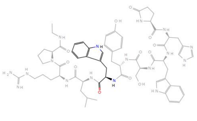	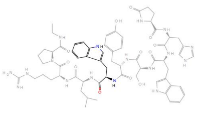	187.0868	187.0866	-1.34
MATCH	9.0	187.0868	187.0866	-1.34	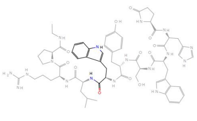	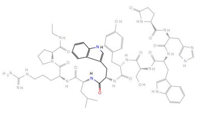	187.0868	187.0866	-1.34
MATCH	9.0	187.0868	187.0866	-1.34	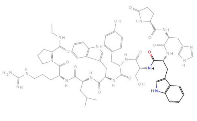	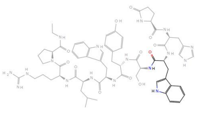	187.0868	187.0866	-1.34
MATCH	27.8	170.0604	170.0600	-2.24	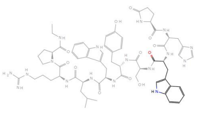	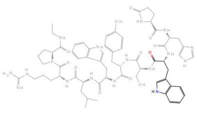	170.0604	170.0600	-2.24

Metabolite: Substrate

Type	score	sub. m/z observed	sub. m/z calculated	sub ppm			met. m/z observed	met. m/z calculated	met. ppm
MATCH	27.8	170.0604	170.0600	-2.24	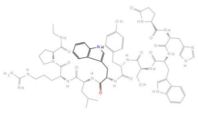	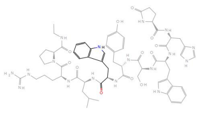	170.0604	170.0600	-2.24
MISMATCH	-3.0	169.0766	169.0846	47.41	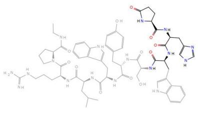	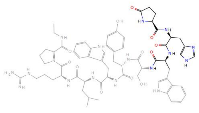	169.0766	169.0846	47.41
MATCH	14.3	166.0615	166.0611	-2.26	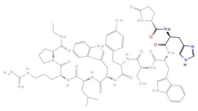	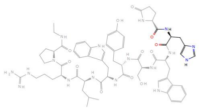	166.0615	166.0611	-2.26
MATCH	104.6	159.0921	159.0917	-2.73	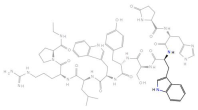	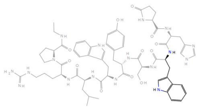	159.0921	159.0917	-2.73
MATCH	192.9	159.0921	159.0917	-2.73	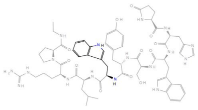	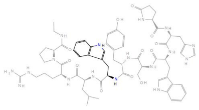	159.0921	159.0917	-2.73
MATCH	11.3	157.1087	157.1084	-2.22	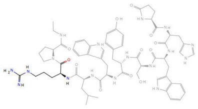	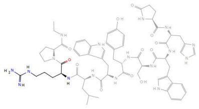	157.1087	157.1084	-2.22
MATCH	4.3	144.0811	144.0808	-2.41	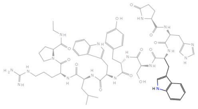	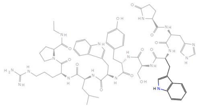	144.0811	144.0808	-2.41
MATCH	4.6	144.0811	144.0808	-2.41	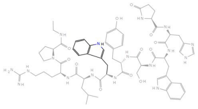	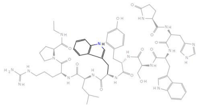	144.0811	144.0808	-2.41
MATCH	76.8	143.1184	143.1179	-3.57	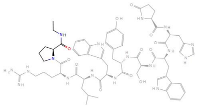	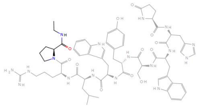	143.1184	143.1179	-3.57

Metabolite: Substrate

Type	score	sub. m/z observed	sub. m/z calculated	sub ppm			met. m/z observed	met. m/z calculated	met. ppm
MATCH	7.8	142.0657	142.0757	70.02	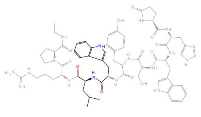	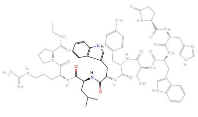	142.0657	142.0757	70.02
MATCH	2.9	140.0823	140.0818	-3.53	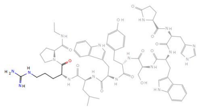	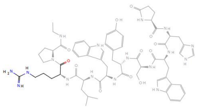	140.0823	140.0818	-3.53
MATCH	81.8	136.0761	136.0757	-3.37	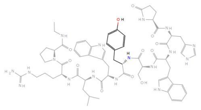	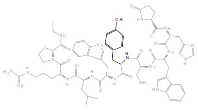	136.0761	136.0757	-3.37
MATCH	77.4	130.0656	130.0575	-62.1	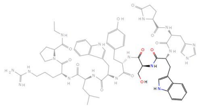	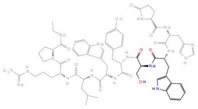	130.0656	130.0575	-62.1
MATCH	9.8	115.0873	115.0866	-5.79	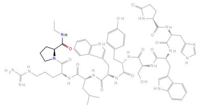	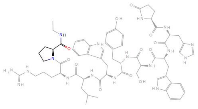	115.0873	115.0866	-5.79
MATCH	4.3	115.0551	115.0522	-25.1	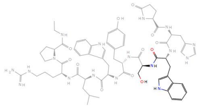	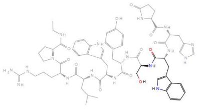	115.0551	115.0522	-25.1
MATCH	34.1	112.0876	112.0869	-5.97	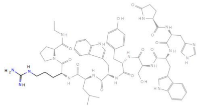	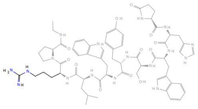	112.0876	112.0869	-5.97
MATCH	170.7	110.0720	110.0713	-6.29	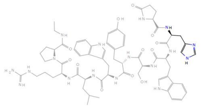	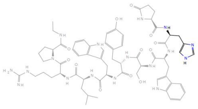	110.0720	110.0713	-6.29
MATCH	6.6	91.0553	91.0522	-33.7	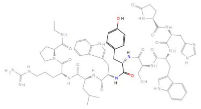	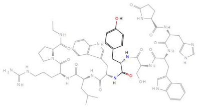	91.0553	91.0522	-33.7

MS (+) FT

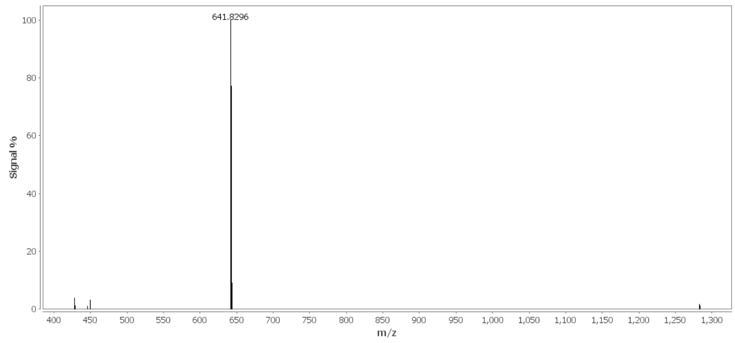

MS (+) FT

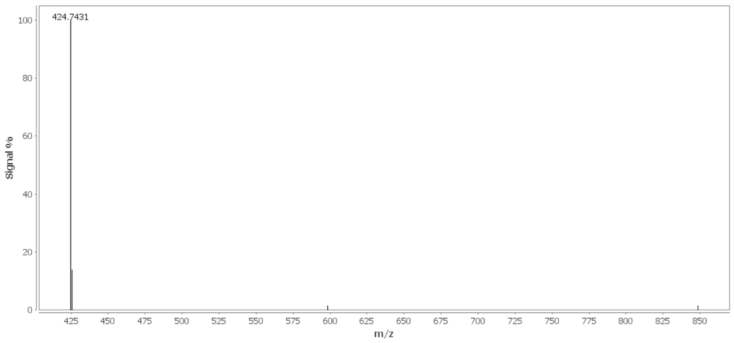

MS2 (+) FT activ = HCD:ce =

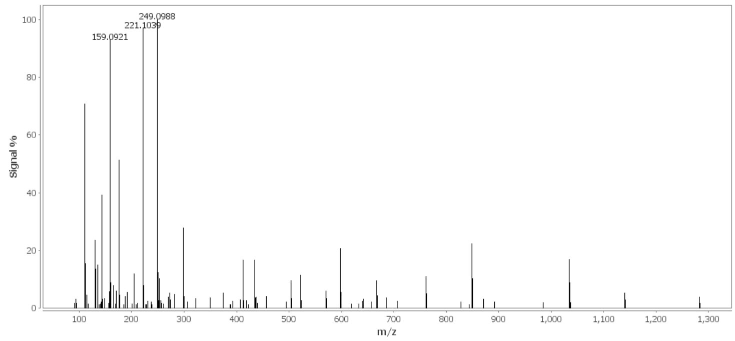

MS2 (+) FT activ = HCD:ce =

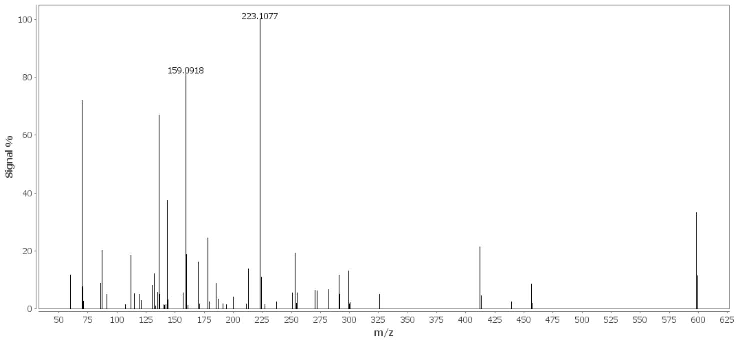

Metabolite: M2 -434 RT=2.20

Type	score	sub. m/z observed	sub. m/z calculated	sub ppm		met. m/z observed	met. m/z calculated	met. ppm
MATCH	103.9	428.2220	428.2208	-2.62	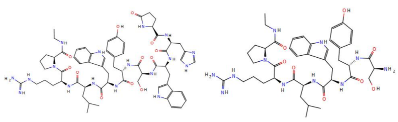	424.7431	424.7425	-1.41
MATCH	103.9	428.2220	428.2208	-2.62	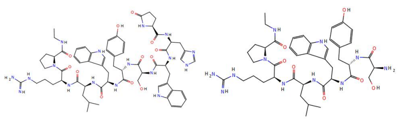	424.7431	424.7425	-1.41
					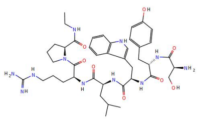	424.7431	424.7425	-1.41
MATCH	5.3	428.2220	428.2208	-2.62	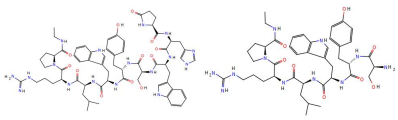	848.4783	848.4777	-0.64
MATCH	5.3	428.2220	428.2208	-2.62	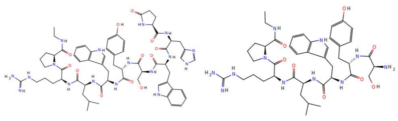	848.4783	848.4777	-0.64

Metabolite: M2 -434 RT=2.20

Type	score	sub. m/z observed	sub. m/z calculated	sub ppm			met. m/z observed	met. m/z calculated	met. ppm
						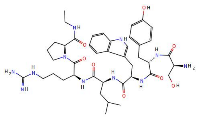	848.4783	848.4777	-0.64
MATCH	200.0	641.8296	641.8276	-3.10	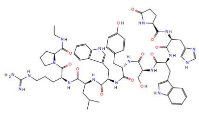	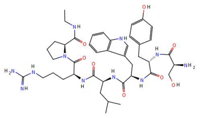	424.7431	424.7425	-1.41
MATCH	200.0	641.8296	641.8276	-3.10	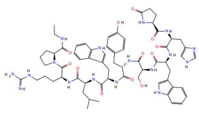	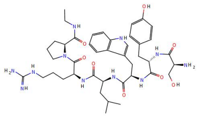	424.7431	424.7425	-1.41
						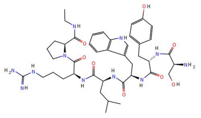	424.7431	424.7425	-1.41
MATCH	101.3	641.8296	641.8276	-3.10	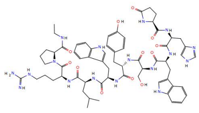	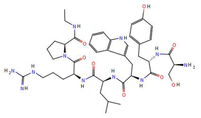	848.4783	848.4777	-0.64
MATCH	101.3	641.8296	641.8276	-3.10	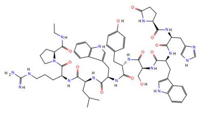	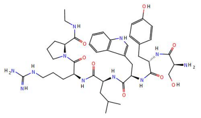	848.4783	848.4777	-0.64
						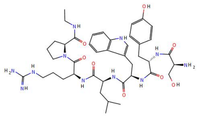	848.4783	848.4777	-0.64
MATCH	101.6	1282.6517	1282.6480	-2.87	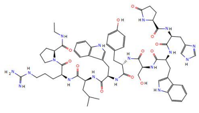	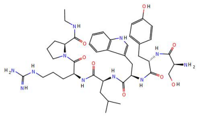	424.7431	424.7425	-1.41
MATCH	101.6	1282.6517	1282.6480	-2.87	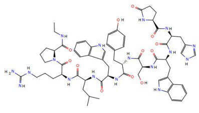	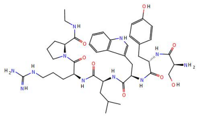	424.7431	424.7425	-1.41

Metabolite: M2 -434 RT=2.20

Type	score	sub. m/z observed	sub. m/z calculated	sub ppm			met. m/z observed	met. m/z calculated	met. ppm
						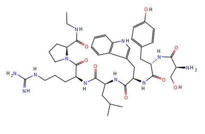	424.7431	424.7425	-1.41
MATCH	2.9	1282.6517	1282.6480	-2.87	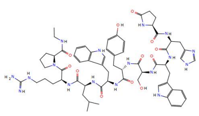	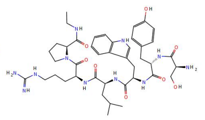	848.4783	848.4777	-0.64
MATCH	2.9	1282.6517	1282.6480	-2.87	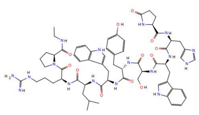	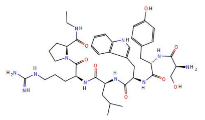	848.4783	848.4777	-0.64
						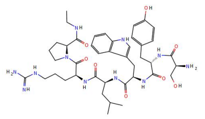	848.4783	848.4777	-0.64
MATCH	6.6	91.0553	91.0522	-33.7	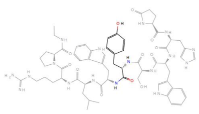	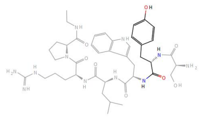	91.0549	91.0522	-29.2
MATCH	34.1	112.0876	112.0869	-5.97	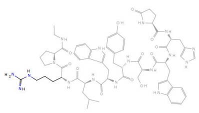	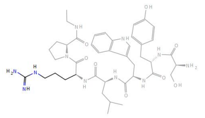	112.0873	112.0869	-3.73
MATCH	9.8	115.0873	115.0866	-5.79	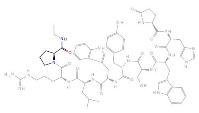	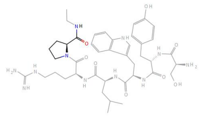	115.0870	115.0866	-3.81
MATCH	81.8	136.0761	136.0757	-3.37	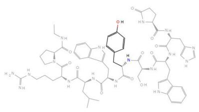	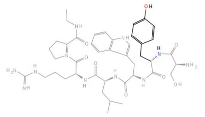	136.0759	136.0757	-1.32
MATCH	2.9	140.0823	140.0818	-3.53	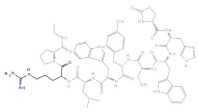	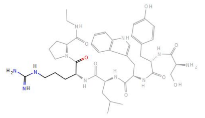	140.0821	140.0818	-1.75

Metabolite: M2 -434 RT=2.20

Type	score	sub. m/z observed	sub. m/z calculated	sub ppm		met. m/z observed	met. m/z calculated	met. ppm
MATCH	3.6	142.0657	142.0757	70.02	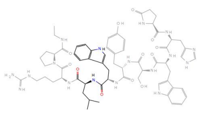 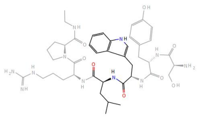	142.0654	142.0757	72.71
MATCH	76.8	143.1184	143.1179	-3.57	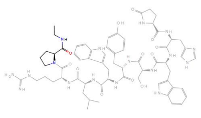 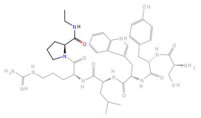	143.1181	143.1179	-1.21
MATCH	4.6	144.0811	144.0808	-2.41	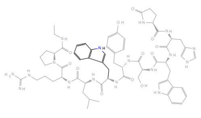 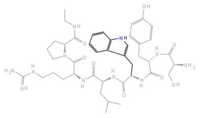	144.0809	144.0808	-0.74
MATCH	11.3	157.1087	157.1084	-2.22	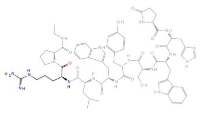 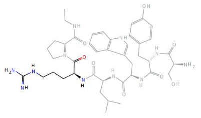	157.1085	157.1084	-0.56
MATCH	174.2	159.0921	159.0917	-2.73	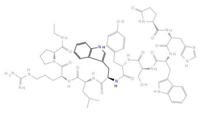 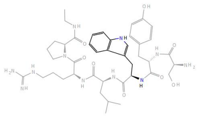	159.0918	159.0917	-0.67
MATCH	22.2	170.0604	170.0600	-2.24	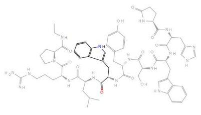 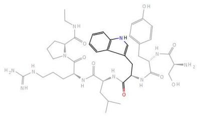	170.0601	170.0600	-0.07
MATCH	7.5	187.0868	187.0866	-1.34	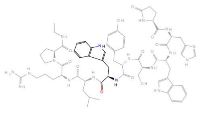 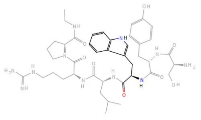	187.0867	187.0866	-0.34
MATCH	7.5	187.0868	187.0866	-1.34	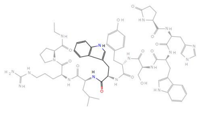 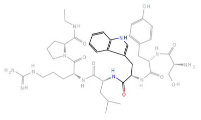	187.0867	187.0866	-0.34
MATCH	4.5	237.1351	237.1346	-2.24	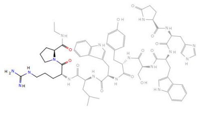 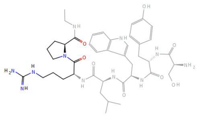	237.1344	237.1346	1.00

Metabolite: M2 -434 RT=2.20

Type	score	sub. m/z observed	sub. m/z calculated	sub ppm		met. m/z observed	met. m/z calculated	met. ppm
MATCH	29.4	253.1663	253.1659	-1.65	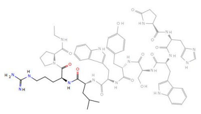 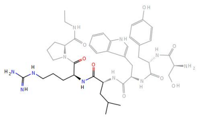	253.1660	253.1659	-0.28
MATCH	8.0	255.1490	255.1492	0.80	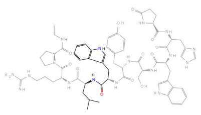 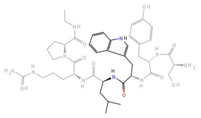	255.1491	255.1492	0.47
MATCH	21.6	261.1145	261.1164	7.47	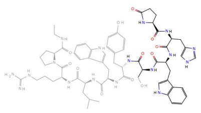 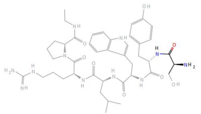	87.0560	87.0553	-8.17
MATCH	10.4	270.1928	270.1925	-1.17	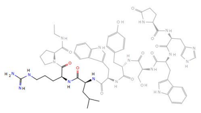 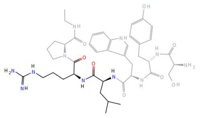	270.1927	270.1925	-0.97
MATCH	11.5	272.1760	272.1757	-0.81	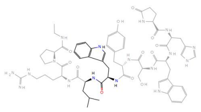 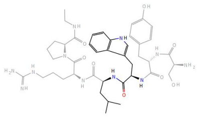	272.1758	272.1757	-0.11
MATCH	11.4	282.1925	282.1925	-0.27	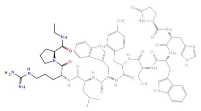 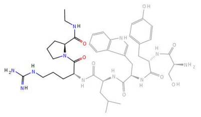	282.1915	282.1925	3.49
MATCH	40.8	299.2199	299.2190	-2.91	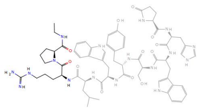 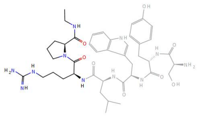	299.2192	299.2190	-0.65
MATCH	3.3	300.1710	300.1707	-1.27	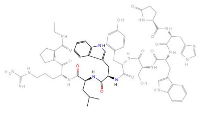 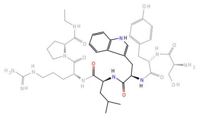	300.1710	300.1707	-1.05
MATCH	3.3	300.1710	300.1707	-1.27	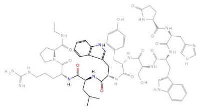 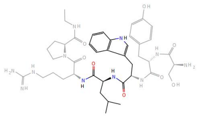	300.1710	300.1707	-1.05

Metabolite: M2 -434 RT=2.20

Type	score	sub. m/z observed	sub. m/z calculated	sub ppm		met. m/z observed	met. m/z calculated	met. ppm
MATCH	38.0	412.3042	412.3031	-2.70	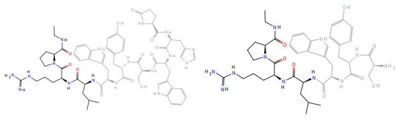	412.3031	412.3031	-0.18
MATCH	4.1	439.2452	439.2452	-0.07	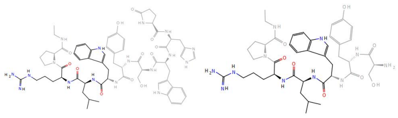	439.2450	439.2452	0.44
MATCH	12.6	456.2740	456.2718	-4.81	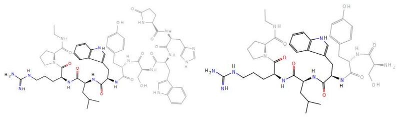	456.2716	456.2718	0.45
MATCH	13.8	494.2130	494.2146	3.33	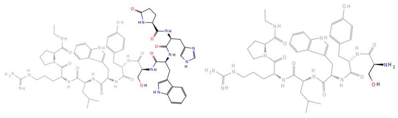	60.0453	60.0444	-15.4
MATCH	11.9	504.2007	504.1990	-3.40	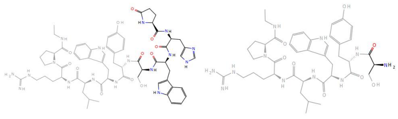	70.0297	70.0287	-13.4
MATCH	53.9	598.3840	598.3824	-2.76	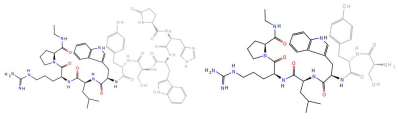	598.3829	598.3824	-0.87
MATCH	102.1	657.2751	657.2780	4.32	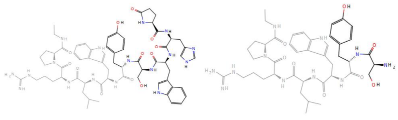	223.1077	223.1077	-0.09
MATCH	9.0	685.2719	685.2729	1.49	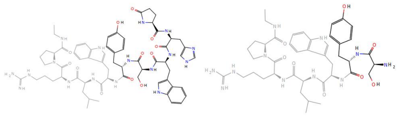	251.1027	251.1026	-0.19
MET_MATCH					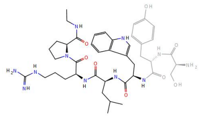	598.3834	598.3824	-1.63

Metabolite: M2 -434 RT=2.20

Type	score	sub. m/z observed	sub. m/z calculated	sub ppm		met. m/z observed	met. m/z calculated	met. ppm
MET_MATCH					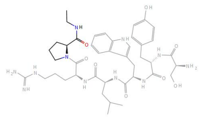	141.1025	141.1022	-1.99
MET_MATCH					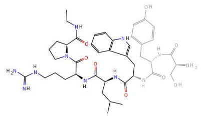	291.1817	291.1816	-0.55

MS (+) FT

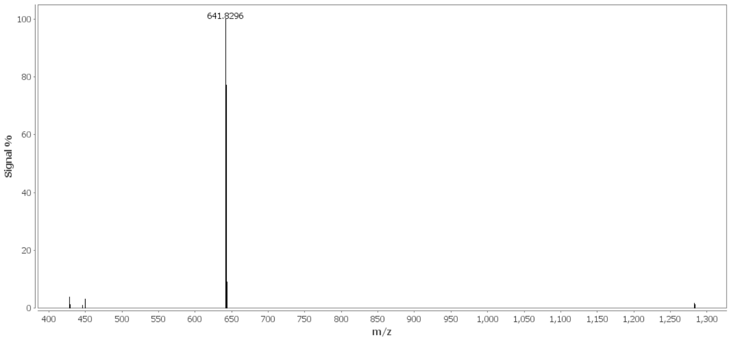

MS (+) FT

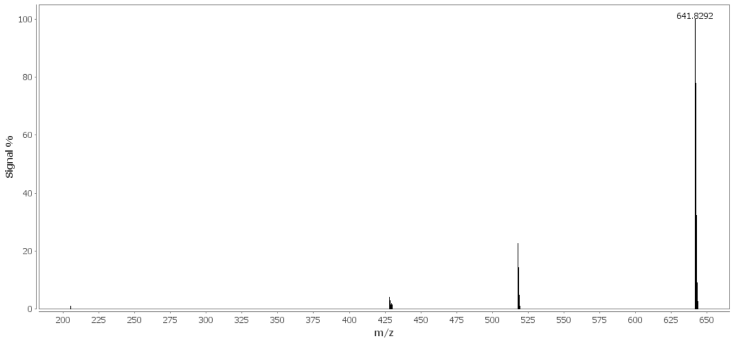

MS2 (+) FT activ = HCD:ce =

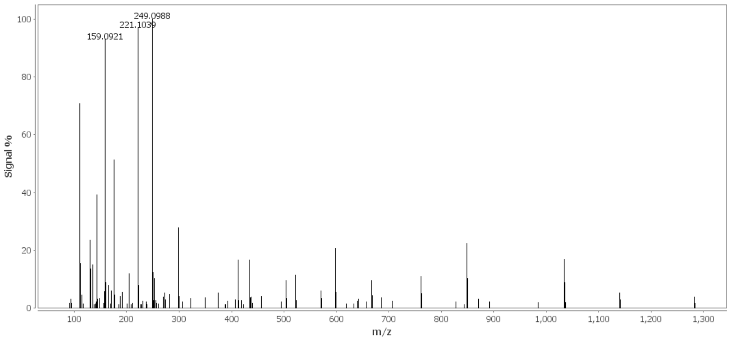

MS2 (+) FT activ = HCD:ce =

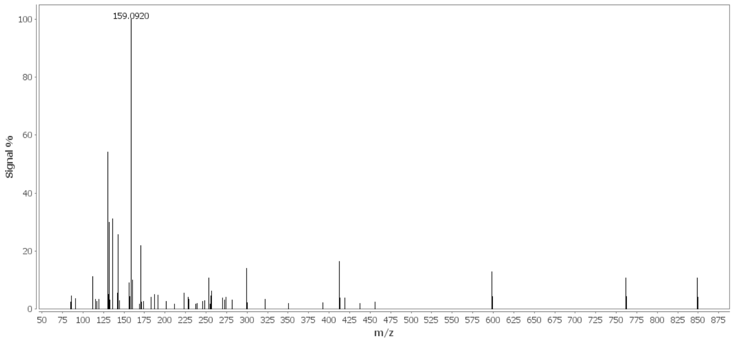

Metabolite: M3 -248 RT=2.54

Type	score	sub. m/z observed	sub. m/z calculated	sub ppm		met. m/z observed	met. m/z calculated	met. ppm
MATCH	26.6	428.2220	428.2208	-2.62	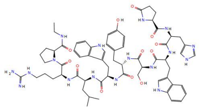 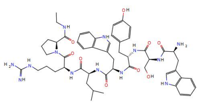	517.7837	517.7822	-2.93
					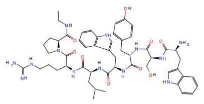	517.7837	517.7822	-2.93

Metabolite: M3 -248 RT=2.54

Type	score	sub. m/z observed	sub. m/z calculated	sub ppm			met. m/z observed	met. m/z calculated	met. ppm
						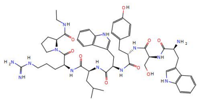	517.7837	517.7822	-2.93
MATCH	122.6	641.8296	641.8276	-3.10	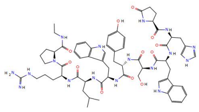	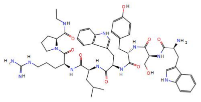	517.7837	517.7822	-2.93
						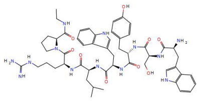	517.7837	517.7822	-2.93
						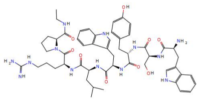	517.7837	517.7822	-2.93
MATCH	24.2	1282.6517	1282.6480	-2.87	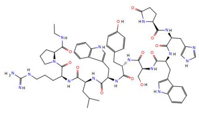	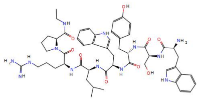	517.7837	517.7822	-2.93
						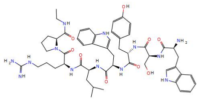	517.7837	517.7822	-2.93
						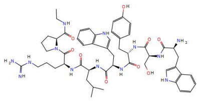	517.7837	517.7822	-2.93
MATCH	5.2	91.0553	91.0522	-33.7	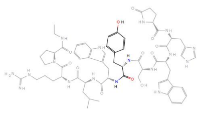	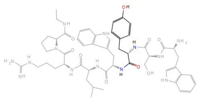	91.0549	91.0522	-29.7
MATCH	26.8	112.0876	112.0869	-5.97	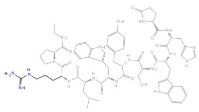	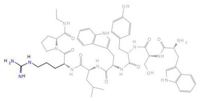	112.0875	112.0869	-5.57

Metabolite: M3 -248 RT=2.54

Type	score	sub. m/z observed	sub. m/z calculated	sub ppm		met. m/z observed	met. m/z calculated	met. ppm
MATCH	4.3	115.0551	115.0522	-25.1	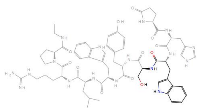 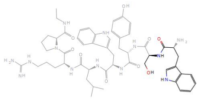	115.0546	115.0522	-20.3
MATCH	7.9	115.0873	115.0866	-5.79	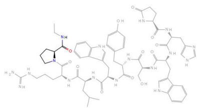 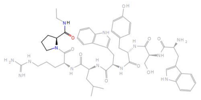	115.0870	115.0866	-3.17
MATCH	4.2	117.0580	117.0679	84.63	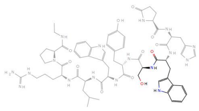 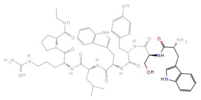	117.0578	117.0679	86.31
MATCH	77.4	130.0656	130.0575	-62.1	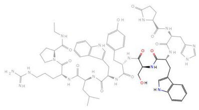 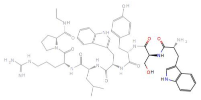	130.0655	130.0575	-61.1
MATCH	46.0	136.0761	136.0757	-3.37	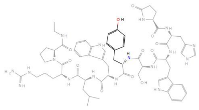 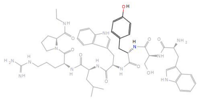	136.0760	136.0757	-2.65
MATCH	7.8	142.0657	142.0757	70.02	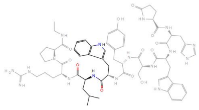 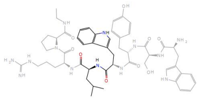	142.0655	142.0757	71.90
MATCH	64.9	143.1184	143.1179	-3.57	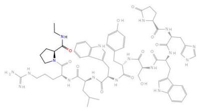 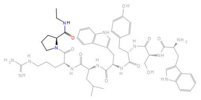	143.1183	143.1179	-2.92
MATCH	4.3	144.0811	144.0808	-2.41	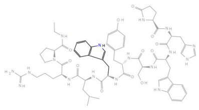 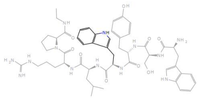	144.0812	144.0808	-2.90
MATCH	4.3	144.0811	144.0808	-2.41	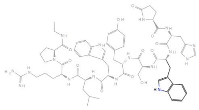 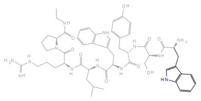	144.0812	144.0808	-2.90

Metabolite: M3 -248 RT=2.54

Type	score	sub. m/z observed	sub. m/z calculated	sub ppm	sub. structure	met. structure	met. m/z observed	met. m/z calculated	met. ppm
MATCH	10.1	157.1087	157.1084	-2.22	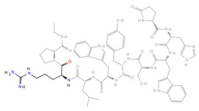	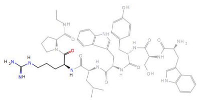	157.1085	157.1084	-1.00
MATCH	192.9	159.0921	159.0917	-2.73	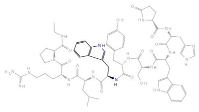	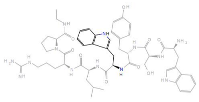	159.0920	159.0917	-1.95
						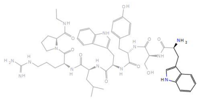	159.0920	159.0917	-1.95
MATCH	27.8	170.0604	170.0600	-2.24	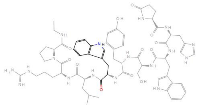	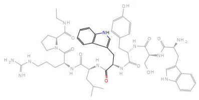	170.0603	170.0600	-1.30
MATCH	27.8	170.0604	170.0600	-2.24	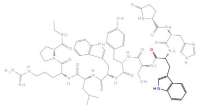	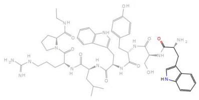	170.0603	170.0600	-1.30
MATCH	9.0	187.0868	187.0866	-1.34	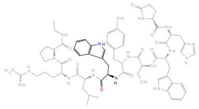	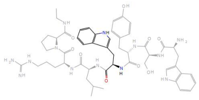	187.0873	187.0866	-3.55
MATCH	9.0	187.0868	187.0866	-1.34	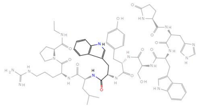	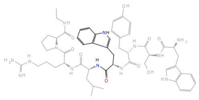	187.0873	187.0866	-3.55
MATCH	9.0	187.0868	187.0866	-1.34	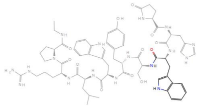	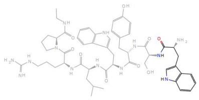	187.0873	187.0866	-3.55
						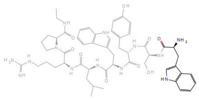	187.0873	187.0866	-3.55

Metabolite: M3 -248 RT=2.54

Type	score	sub. m/z observed	sub. m/z calculated	sub ppm			met. m/z observed	met. m/z calculated	met. ppm
MATCH	3.9	237.1351	237.1346	-2.24	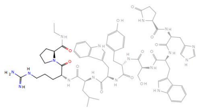	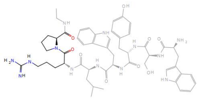	237.1352	237.1346	-2.53
MATCH	20.8	253.1663	253.1659	-1.65	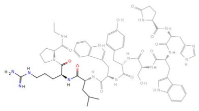	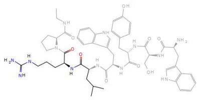	253.1659	253.1659	0.04
MATCH	4.1	255.1490	255.1492	0.80	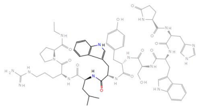	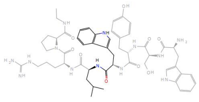	255.1504	255.1492	-4.72
MATCH	7.7	270.1928	270.1925	-1.17	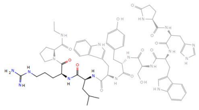	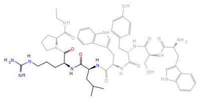	270.1926	270.1925	-0.57
MATCH	8.4	272.1760	272.1757	-0.81	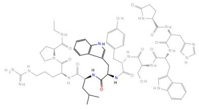	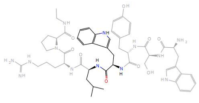	272.1770	272.1757	-4.52
MATCH	6.9	274.1194	274.1186	-2.78	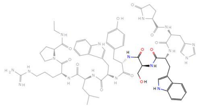	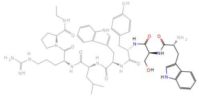	274.1184	274.1186	0.88
						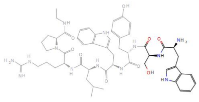	274.1184	274.1186	0.88
MATCH	7.8	282.1925	282.1925	-0.27	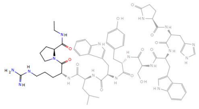	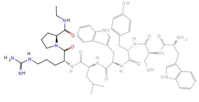	282.1924	282.1925	0.19
MATCH	41.8	299.2199	299.2190	-2.91	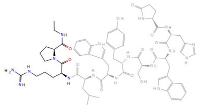	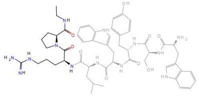	299.2194	299.2190	-1.19

Metabolite: M3 -248 RT=2.54

Type	score	sub. m/z observed	sub. m/z calculated	sub ppm			met. m/z observed	met. m/z calculated	met. ppm
MATCH	6.7	322.1557	322.1550	-2.31	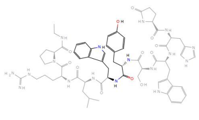	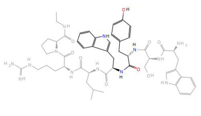	322.1548	322.1550	0.61
MATCH	5.5	350.1511	350.1499	-3.25	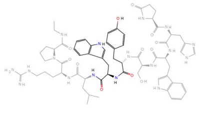	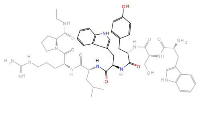	350.1505	350.1499	-1.73
MATCH	5.5	350.1511	350.1499	-3.25	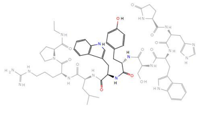	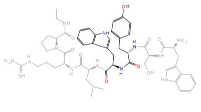	350.1505	350.1499	-1.73
MATCH	102.9	407.1849	407.1826	-5.51	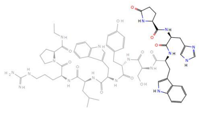	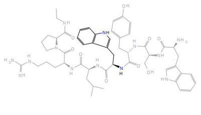	159.0920	159.0917	-1.95
						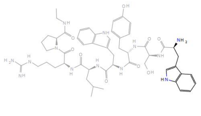	159.0920	159.0917	-1.95
MATCH	33.0	412.3042	412.3031	-2.70	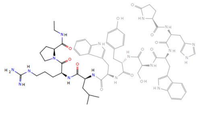	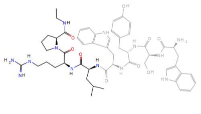	412.3040	412.3031	-2.35
MATCH	21.6	435.1786	435.1775	-2.41	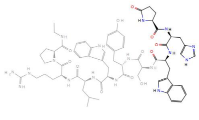	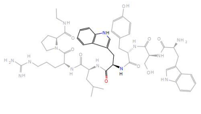	187.0873	187.0866	-3.55
						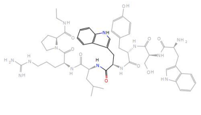	187.0873	187.0866	-3.55
						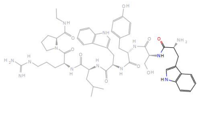	187.0873	187.0866	-3.55

Metabolite: M3 -248 RT=2.54

Type	score	sub. m/z observed	sub. m/z calculated	sub ppm		met. m/z observed	met. m/z calculated	met. ppm
					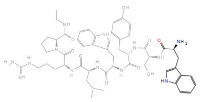	187.0873	187.0866	-3.55
MATCH	6.4	456.2740	456.2718	-4.81	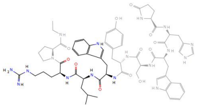 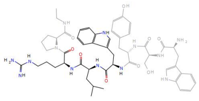	456.2715	456.2718	0.49
MATCH	4.8	494.2130	494.2146	3.33	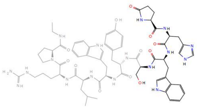 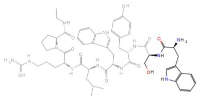	246.1240	246.1237	-1.23
MATCH	14.0	504.2007	504.1990	-3.40	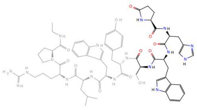 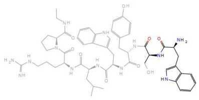	256.1092	256.1081	-4.46
MATCH	15.3	522.2109	522.2096	-2.58	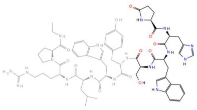 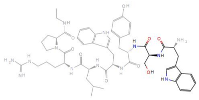	274.1184	274.1186	0.88
					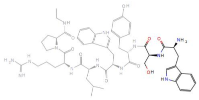	274.1184	274.1186	0.88
MATCH	33.4	598.3840	598.3824	-2.76	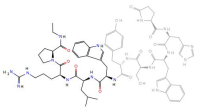 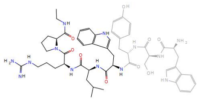	598.3842	598.3824	-2.99
MATCH	13.2	667.2625	667.2623	-0.33	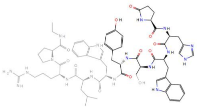 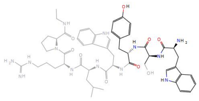	419.1709	419.1714	1.13
MATCH	5.4	685.2719	685.2729	1.49	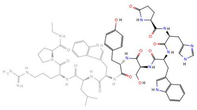 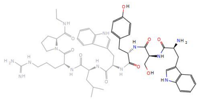	437.1815	437.1819	1.01

Metabolite: M3 -248 RT=2.54

Type	score	sub. m/z observed	sub. m/z calculated	sub ppm		met. m/z observed	met. m/z calculated	met. ppm
MATCH	21.6	761.4484	761.4457	-3.48	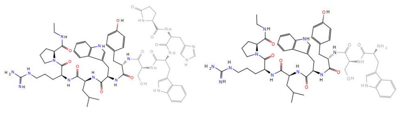	761.4479	761.4457	-2.92
MATCH	33.1	848.4802	848.4777	-2.96	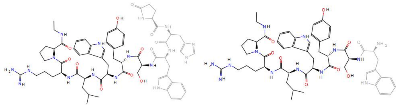	848.4816	848.4777	-4.62
MISMATCH	-7.9	428.2220	428.2208	-2.62	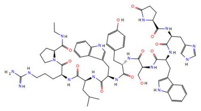	428.2220	428.2220	0.00
MISMATCH	-103.9	428.2220	428.2208	-2.62	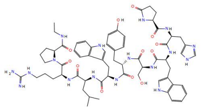	641.8292	641.8292	0.00
MISMATCH	-104.0	641.8296	641.8276	-3.10	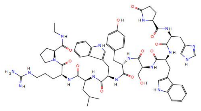	428.2220	428.2220	0.00
MISMATCH	-200.0	641.8296	641.8276	-3.10	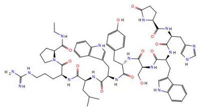	641.8292	641.8292	0.00
MISMATCH	-5.6	1282.6517	1282.6480	-2.87	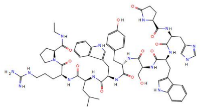	428.2220	428.2220	0.00
MISMATCH	-101.6	1282.6517	1282.6480	-2.87	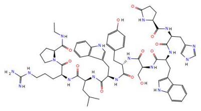	641.8292	641.8292	0.00
MISMATCH	-3.0	169.0766	169.0846	47.41	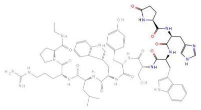	169.0761	169.0761	0.00

MS (+) FT

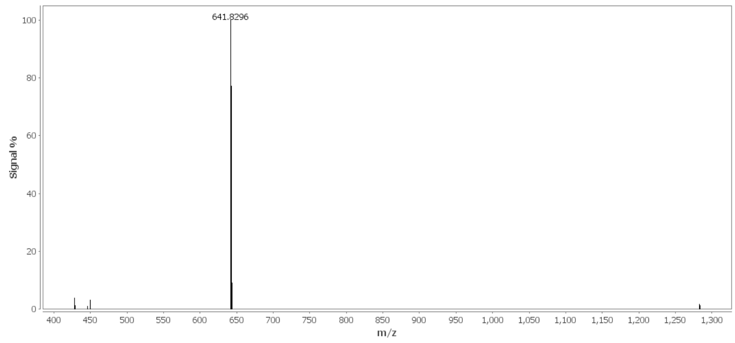

MS (+) FT

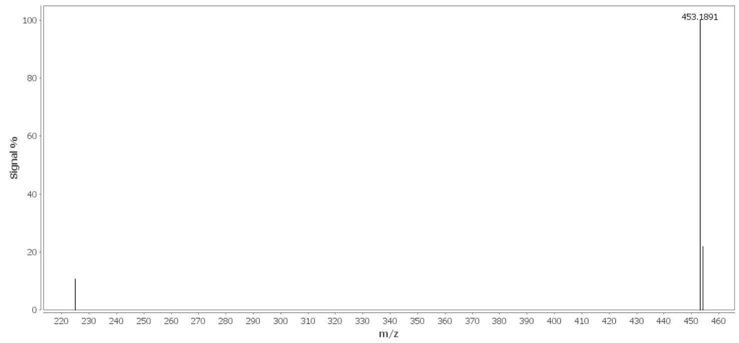

MS2 (+) FT activ = HCD:ce =

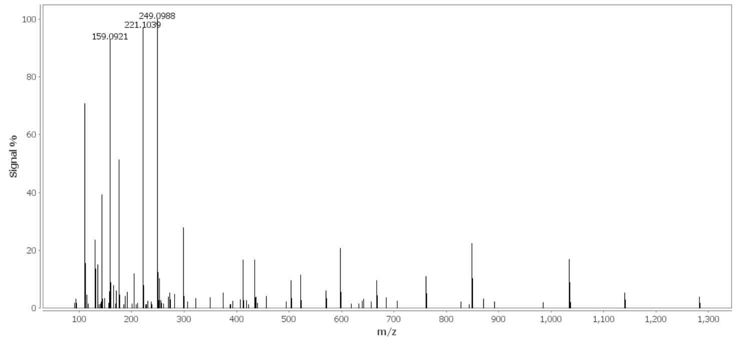

MS2 (+) FT activ = HCD:ce =

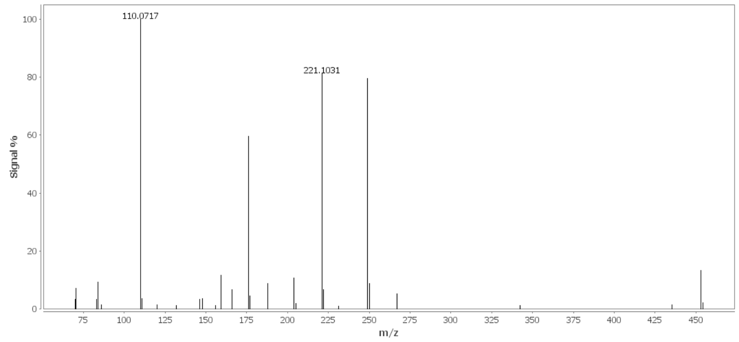

Metabolite: M1 -829 RT=0.51

Type	score	sub. m/z observed	sub. m/z calculated	sub ppm			met. m/z observed	met. m/z calculated	met. ppm
MATCH	103.9	428.2220	428.2208	-2.62	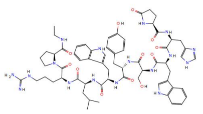	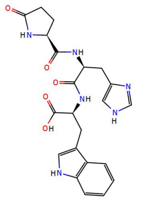	453.1891	453.1881	-2.23
						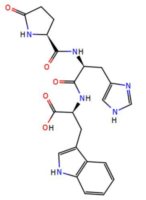	453.1891	453.1881	-2.23
						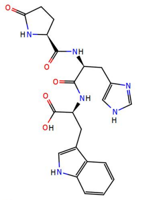	453.1891	453.1881	-2.23
MATCH	200.0	641.8296	641.8276	-3.10	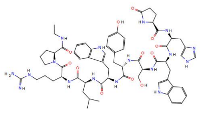	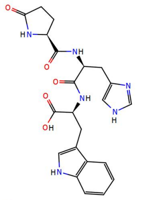	453.1891	453.1881	-2.23
						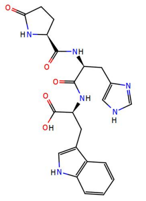	453.1891	453.1881	-2.23

Metabolite: M1 -829 RT=0.51

Type	score	sub. m/z observed	sub. m/z calculated	sub ppm			met. m/z observed	met. m/z calculated	met. ppm
						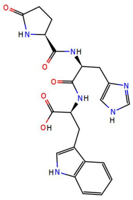	453.1891	453.1881	-2.23
MATCH	101.6	1282.6517	1282.6480	-2.87	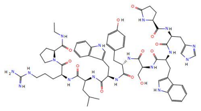	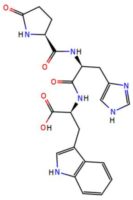	453.1891	453.1881	-2.23
						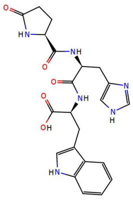	453.1891	453.1881	-2.23
						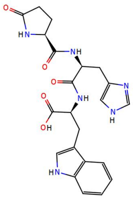	453.1891	453.1881	-2.23
MATCH	170.7	110.0720	110.0713	-6.29	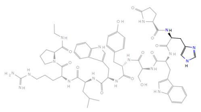	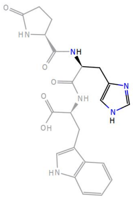	110.0717	110.0713	-3.55
MATCH	104.6	159.0921	159.0917	-2.73	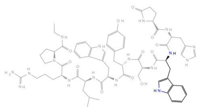	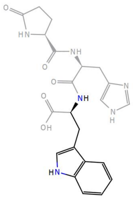	159.0915	159.0917	0.94
MATCH	14.3	166.0615	166.0611	-2.26	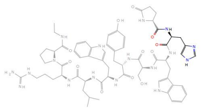	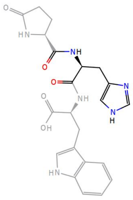	166.0610	166.0611	0.66
MATCH	178.0	221.1039	221.1033	-2.69	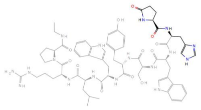	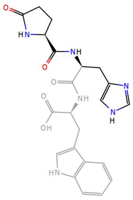	221.1031	221.1033	0.71
MATCH	179.4	249.0988	249.0982	-2.32	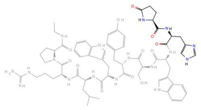	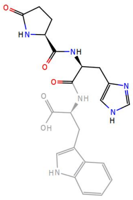	249.0981	249.0982	0.64

Metabolite: M1 -829 RT=0.51

Type	score	sub. m/z observed	sub. m/z calculated	sub ppm			met. m/z observed	met. m/z calculated	met. ppm
MATCH	16.4	641.8288	641.8276	-1.74	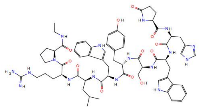	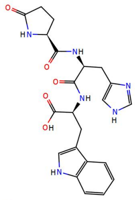	453.1852	453.1881	6.43
						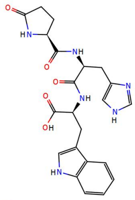	453.1852	453.1881	6.43
MATCH	16.9	1282.6527	1282.6480	-3.71	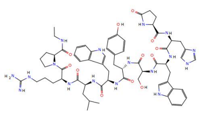	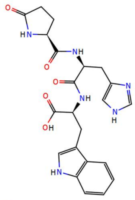	453.1852	453.1881	6.43
						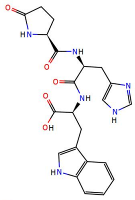	453.1852	453.1881	6.43
MET_MATCH						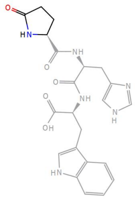	84.0451	84.0444	-8.25
MET_MATCH						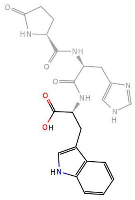	188.0705	188.0706	0.81
MET_MATCH						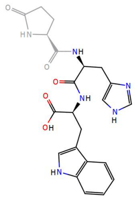	342.1569	342.1561	-2.50
