# Supplementary material for: Software-aided approach to investigate peptide structure and metabolic susceptibility of amide bonds in peptide drugs based on high resolution mass spectrometry
Source: PLoS One. 2017 Nov 1;12(11):e0186461. doi: 10.1371/journal.pone.0186461 (PMC5665424; doi:10.1371/journal.pone.0186461)
Supplement: S1 File — (ZIP) [file pone.0186461.s007.zip › SFiles/S13_File.pdf]

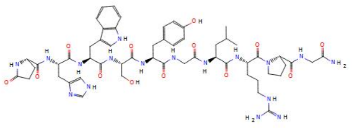

Gonadorelin

Property name	Property value
Time	0min, 5min, 15min, 45min, 120min
Instrument	ThermoQAPlus
Matrix	chymotrypsin
Acquisition Mode	ddMS2

Chromatograms

Time=0min

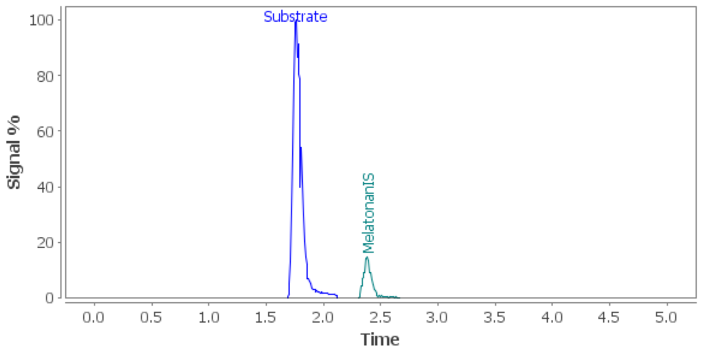

Time=5min

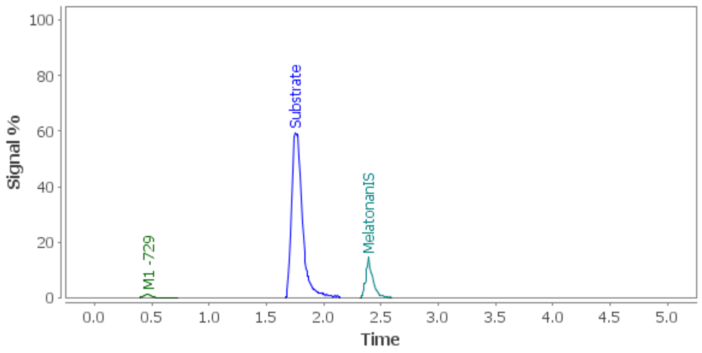

Time=15min

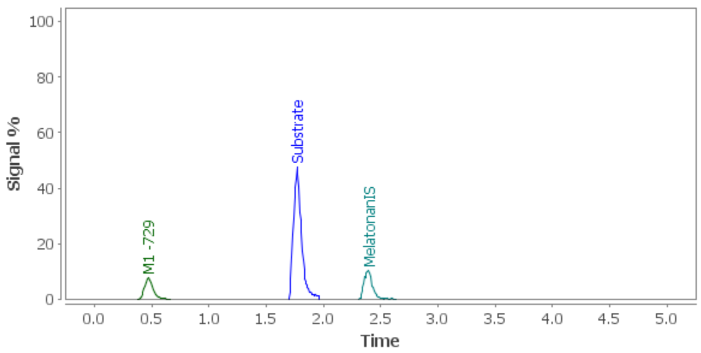

Time=45min

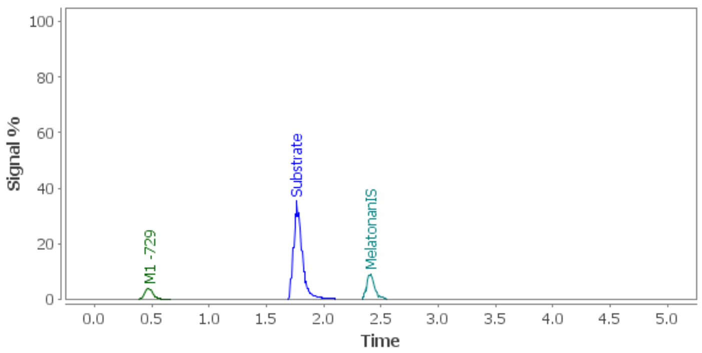

Time=120min

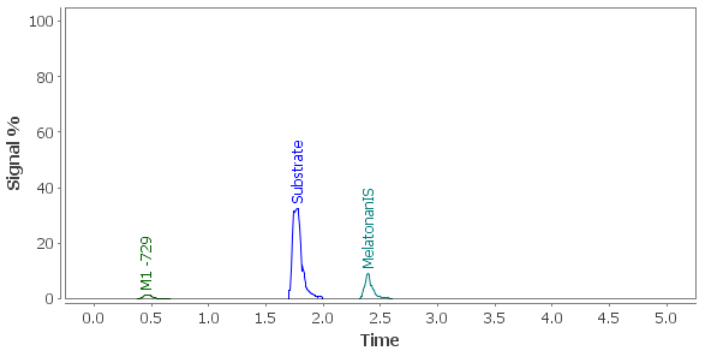

Custom Charts

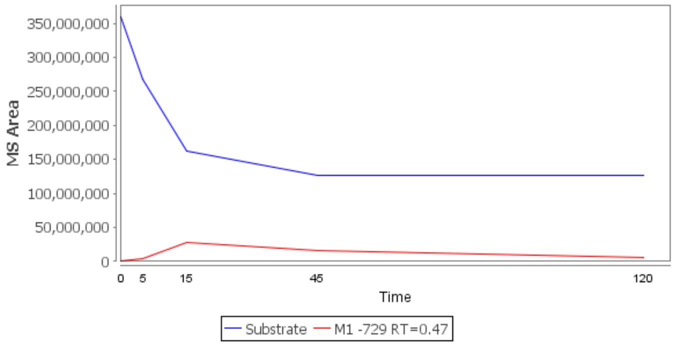

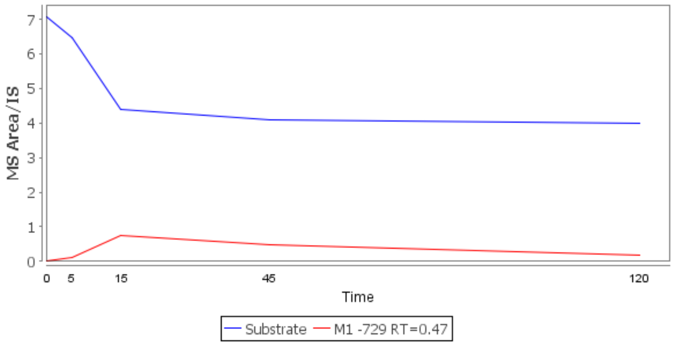

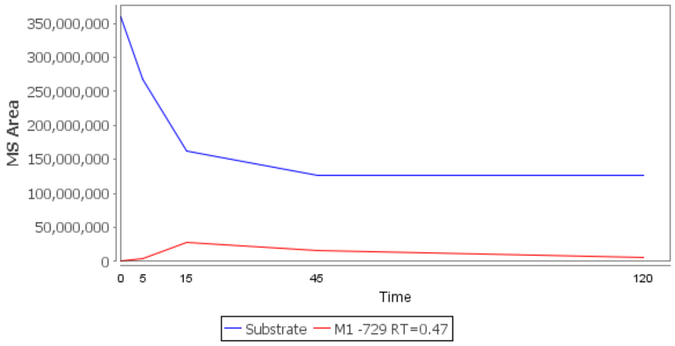

Fragmentation

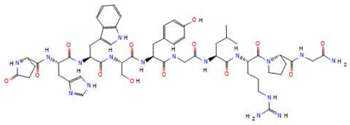

Gonadorelin

MS (+) FT

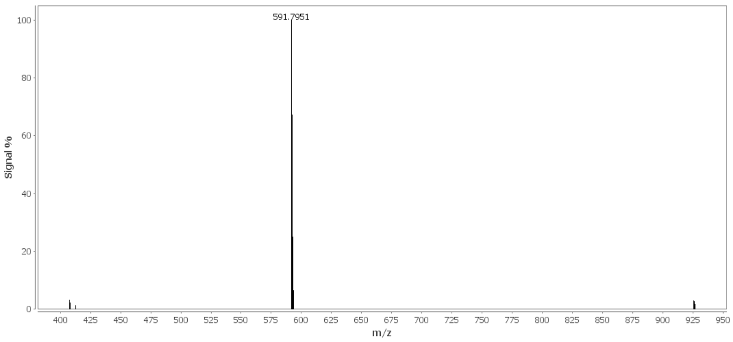

MS (+) FT

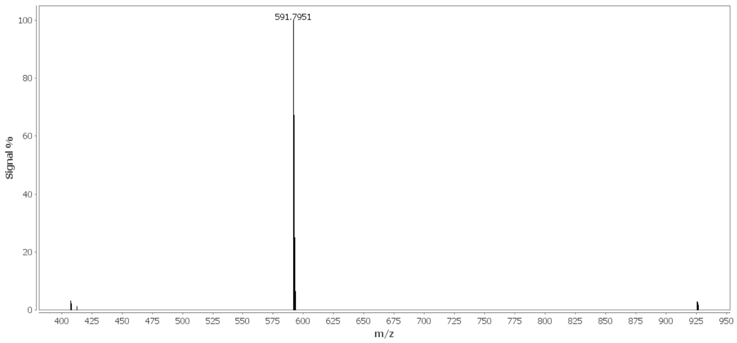

MS2 (+) FT activ = HCD:ce =

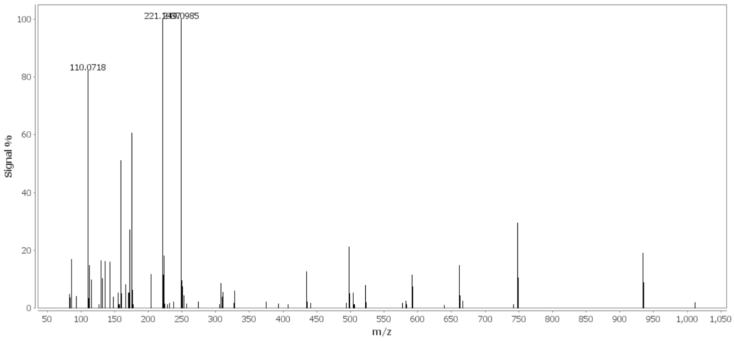

MS2 (+) FT activ = HCD:ce =

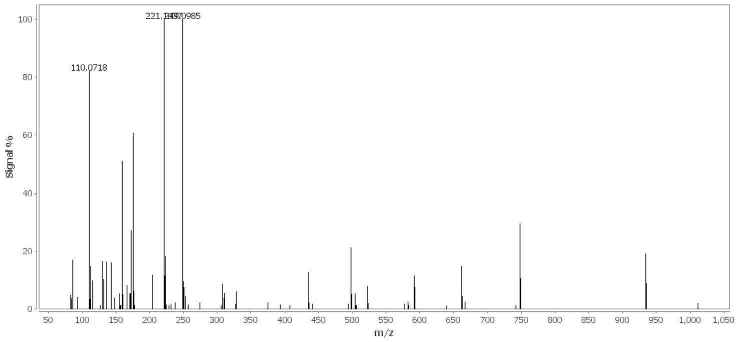

Metabolite: Substrate

Type	score	sub. m/z observed	sub. m/z calculated	sub ppm		met. m/z observed	met. m/z calculated	met. ppm
MATCH	200.0	591.7951	591.7938	-2.27	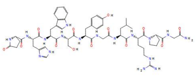 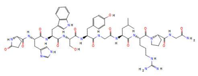	591.7951	591.7938	-2.27
MATCH	21.8	591.7945	591.7938	-1.19	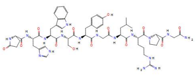 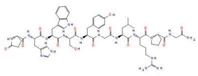	591.7945	591.7938	-1.19
MATCH	169.8	249.0985	249.0982	-1.08	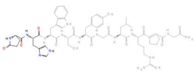 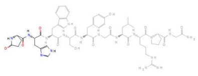	249.0985	249.0982	-1.08

Metabolite: Substrate

Type	score	sub. m/z observed	sub. m/z calculated	sub ppm		met. m/z observed	met. m/z calculated	met. ppm
MATCH	183.4	221.1037	221.1033	-1.77	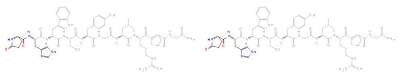	221.1037	221.1033	-1.77
MATCH	16.1	166.0614	166.0611	-1.67	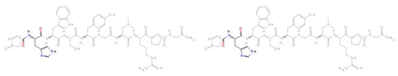	166.0614	166.0611	-1.67
MATCH	62.4	159.0918	159.0917	-0.77	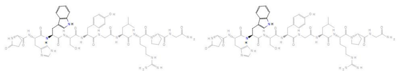	159.0918	159.0917	-0.77
MATCH	182.2	110.0718	110.0713	-4.53	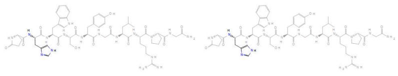	110.0718	110.0713	-4.53
MATCH	6.6	93.0453	93.0447	-6.66	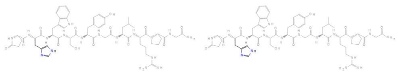	93.0453	93.0447	-6.66

MS (+) FT

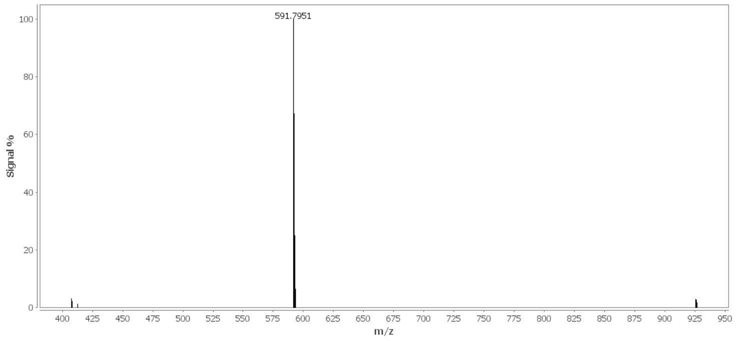

MS (+) FT

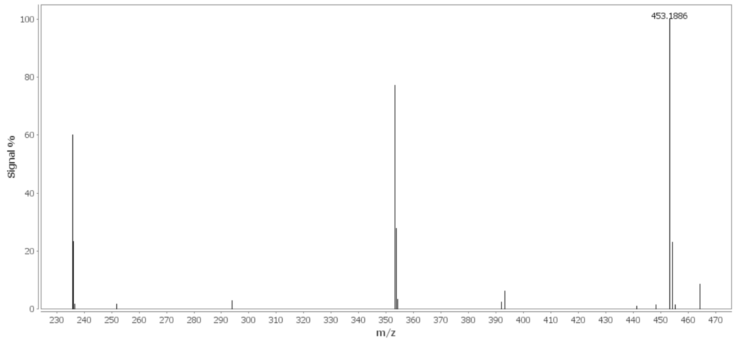

MS2 (+) FT activ = HCD:ce =

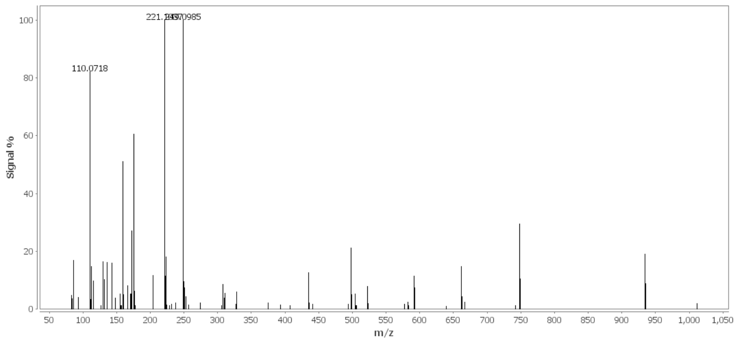

MS2 (+) FT activ = HCD:ce =

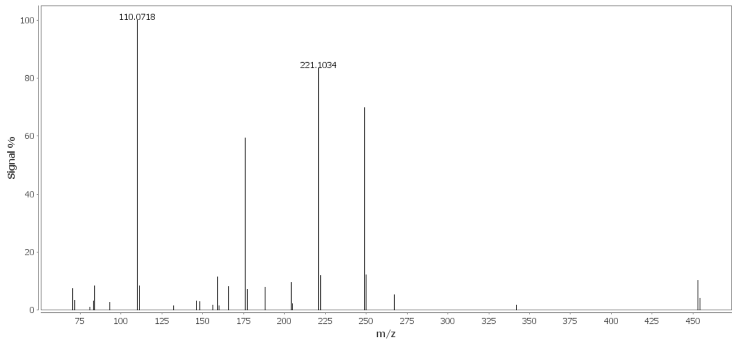

Metabolite: M1 -729 RT=0.47

Type	score	sub. m/z observed	sub. m/z calculated	sub ppm			met. m/z observed	met. m/z calculated	met. ppm
MATCH	200.0	591.7951	591.7938	-2.27	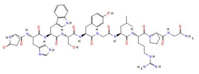	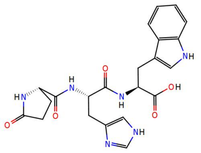	453.1886	453.1881	-1.19
MATCH	6.6	93.0453	93.0447	-6.66	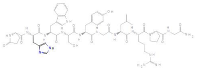	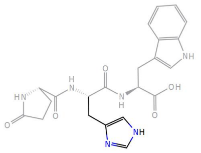	93.0454	93.0447	-7.00
MATCH	182.2	110.0718	110.0713	-4.53	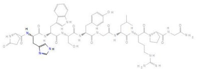	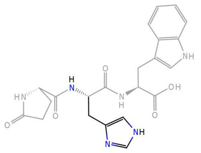	110.0718	110.0713	-4.37
MATCH	62.4	159.0918	159.0917	-0.77	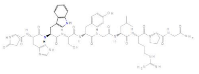	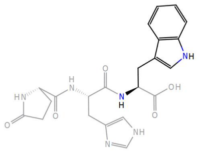	159.0918	159.0917	-1.07
MATCH	16.1	166.0614	166.0611	-1.67	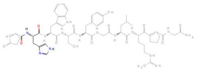	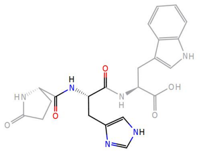	166.0612	166.0611	-0.33
MATCH	183.4	221.1037	221.1033	-1.77	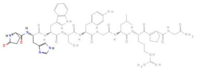	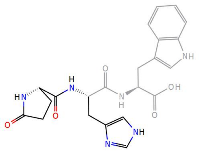	221.1034	221.1033	-0.38
MATCH	169.8	249.0985	249.0982	-1.08	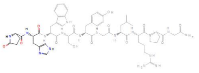	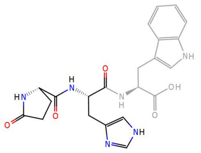	249.0983	249.0982	-0.35
MATCH	21.8	591.7945	591.7938	-1.19	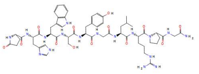	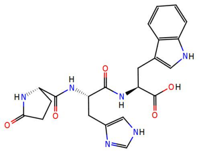	453.1873	453.1881	1.79
MET_MATCH						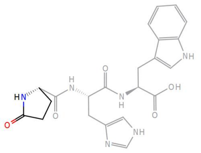	84.0452	84.0444	-9.52

Metabolite: M1 -729 RT=0.47

Type	score	sub. m/z observed	sub. m/z calculated	sub ppm		met. m/z observed	met. m/z calculated	met. ppm
MET_MATCH					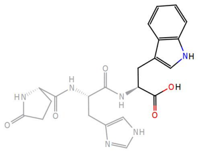	188.0708	188.0706	-1.07
MET_MATCH					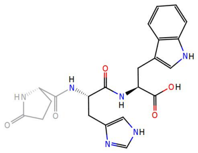	342.1562	342.1561	-0.36
