# Supplementary material for: Software-aided approach to investigate peptide structure and metabolic susceptibility of amide bonds in peptide drugs based on high resolution mass spectrometry
Source: PLoS One. 2017 Nov 1;12(11):e0186461. doi: 10.1371/journal.pone.0186461 (PMC5665424; doi:10.1371/journal.pone.0186461)
Supplement: S1 File — (ZIP) [file pone.0186461.s007.zip › SFiles/S14_File.pdf]

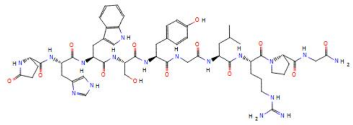

Gonadorelin

Property name	Property value
Time	0min, 5min, 15min, 45min, 120min
Instrument	ThermoQAPlus
Matrix	trypsin
Acquisition Mode	ddMS2

Chromatograms

Time=0min

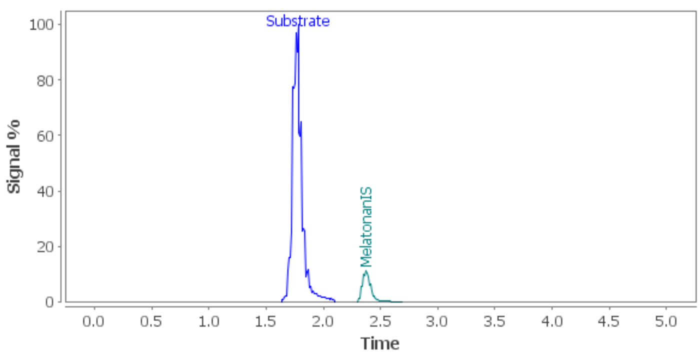

Time=5min

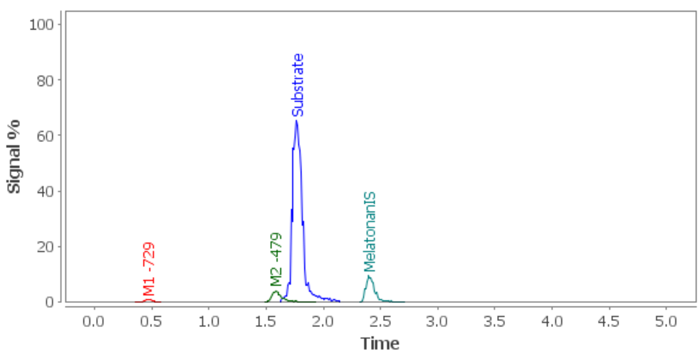

Time=15min

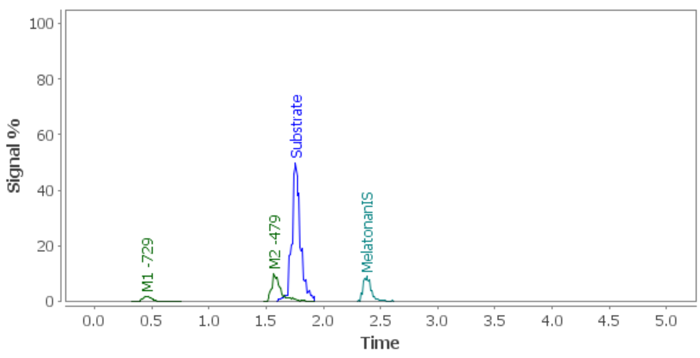

Time=45min

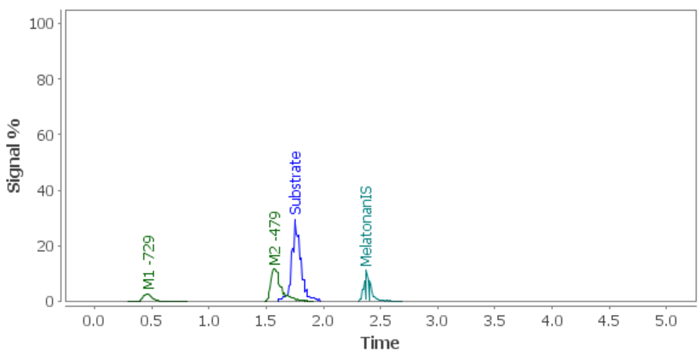

Time=120min

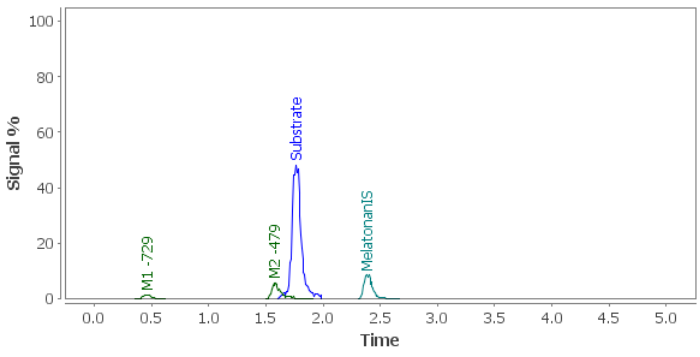

Custom Charts

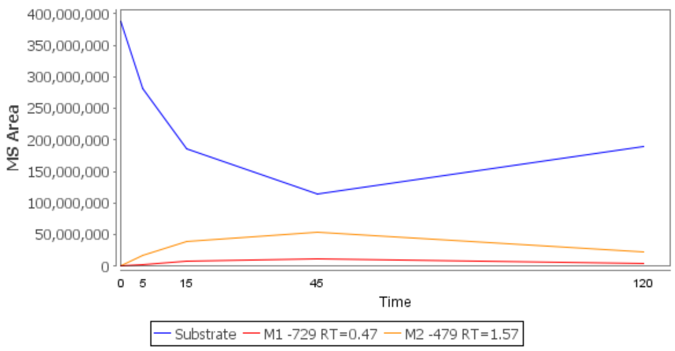

Fragmentation

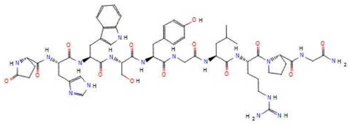

Gonadorelin

MS (+) FT

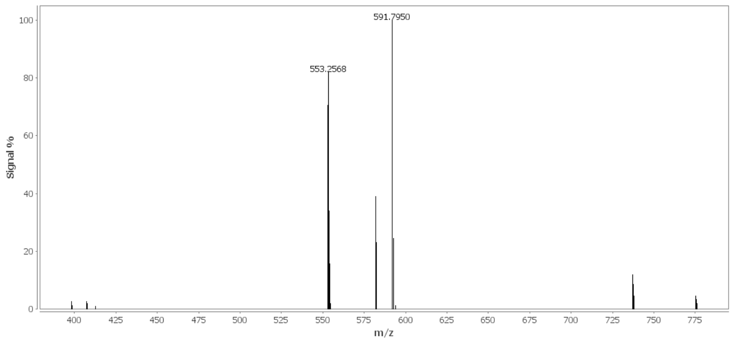

MS (+) FT

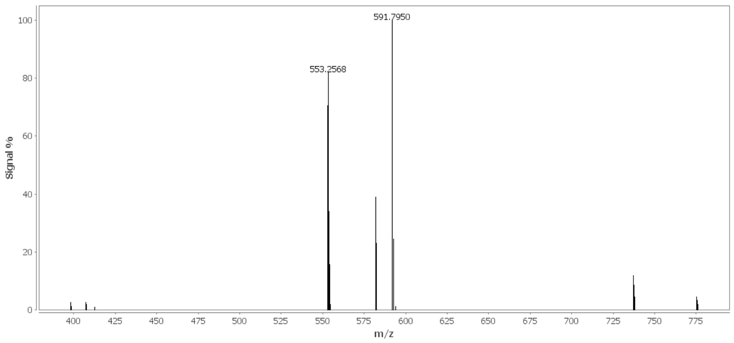

MS2 (+) FT activ = HCD:ce =

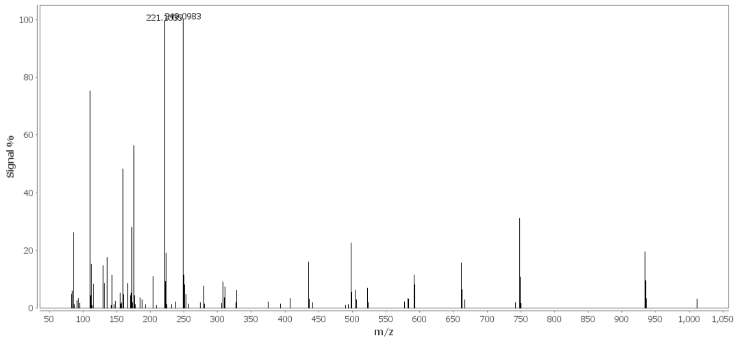

MS2 (+) FT activ = HCD:ce =

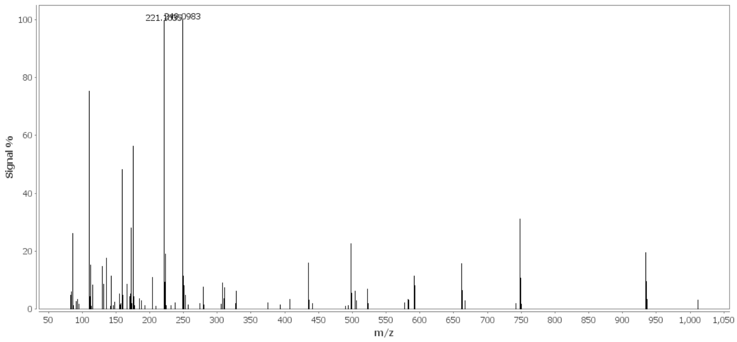

Metabolite: Substrate

Type	score	sub. m/z observed	sub. m/z calculated	sub ppm		met. m/z observed	met. m/z calculated	met. ppm
MATCH	21.9	934.4909	934.4894	-1.60	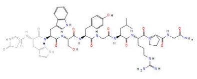 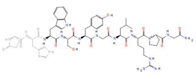	934.4909	934.4894	-1.60
MATCH	32.5	748.4095	748.4100	0.77	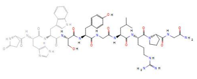 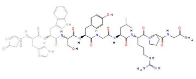	748.4095	748.4100	0.77
MATCH	17.9	661.3782	661.3780	-0.29	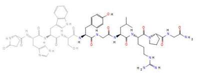 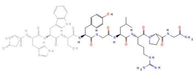	661.3782	661.3780	-0.29

Metabolite: Substrate

Type	score	sub. m/z observed	sub. m/z calculated	sub ppm		met. m/z observed	met. m/z calculated	met. ppm
MATCH	200.0	591.7950	591.7938	-1.98	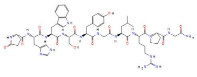 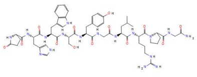	591.7950	591.7938	-1.98
MATCH	31.2	591.7943	591.7938	-0.82	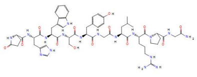 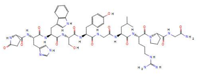	591.7943	591.7938	-0.82
MATCH	13.7	522.2091	522.2096	0.91	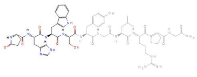 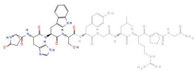	522.2091	522.2096	0.91
MATCH	7.8	504.2000	504.1990	-1.94	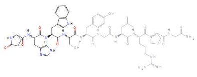 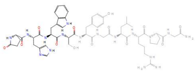	504.2000	504.1990	-1.94
MATCH	3.0	494.2134	494.2146	2.47	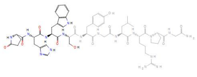 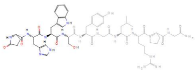	494.2134	494.2146	2.47
MATCH	37.3	435.1777	435.1775	-0.40	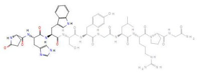 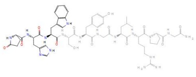	435.1777	435.1775	-0.40
MATCH	7.3	407.1830	407.1826	-0.95	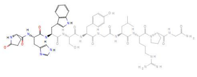 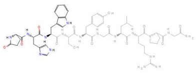	407.1830	407.1826	-0.95
MATCH	9.3	274.1180	274.1186	2.09	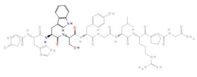 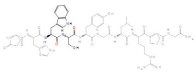	274.1180	274.1186	2.09
MATCH	9.3	274.1180	274.1186	2.09	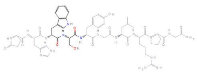 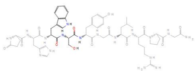	274.1180	274.1186	2.09

Metabolite: Substrate

Type	score	sub. m/z observed	sub. m/z calculated	sub ppm			met. m/z observed	met. m/z calculated	met. ppm
MATCH	4.6	257.0908	257.0921	4.76	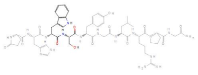	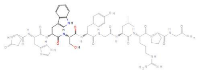	257.0908	257.0921	4.76
MATCH	200.0	249.0983	249.0982	-0.29	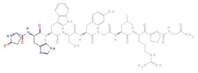	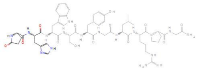	249.0983	249.0982	-0.29
MATCH	187.8	221.1035	221.1033	-1.10	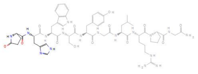	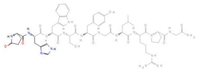	221.1035	221.1033	-1.10
MATCH	9.3	170.0602	170.0600	-1.11	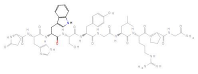	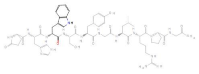	170.0602	170.0600	-1.11
MATCH	15.6	166.0611	166.0611	-0.25	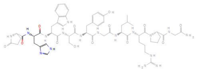	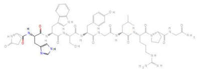	166.0611	166.0611	-0.25
MATCH	123.9	159.0917	159.0917	-0.18	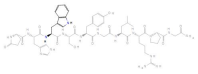	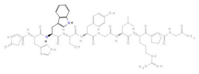	159.0917	159.0917	-0.18
MATCH	20.6	136.0758	136.0757	-0.96	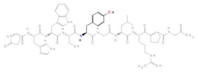	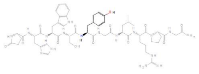	136.0758	136.0757	-0.96
MATCH	175.2	110.0717	110.0713	-3.84	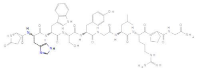	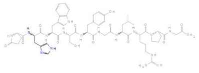	110.0717	110.0713	-3.84
MATCH	4.6	93.0455	93.0447	-8.08	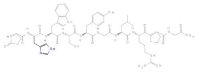	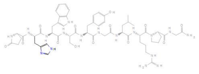	93.0455	93.0447	-8.08

Metabolite: Substrate

Type	score	sub. m/z observed	sub. m/z calculated	sub ppm		met. m/z observed	met. m/z calculated	met. ppm
MISMATCH	-29.2	86.0971	86.0964	-8.04	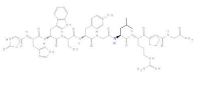 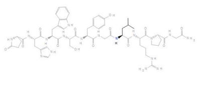	86.0971	86.0964	-8.04
MATCH	13.6	84.0451	84.0444	-8.02	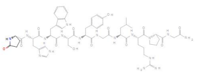 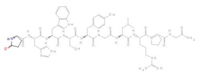	84.0451	84.0444	-8.02

MS (+) FT

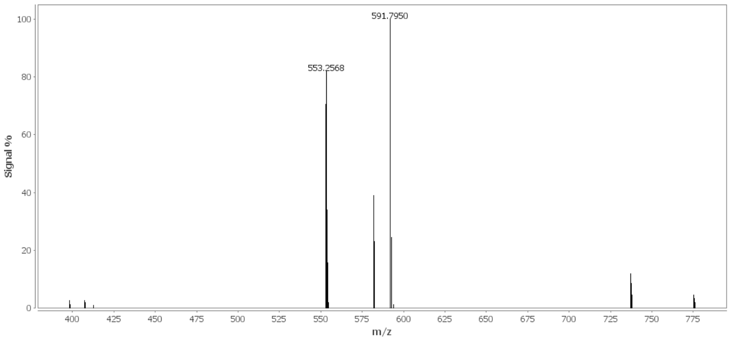

MS (+) FT

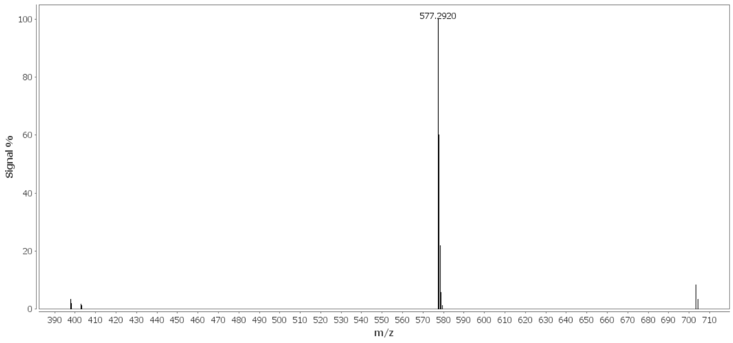

MS2 (+) FT activ = HCD:ce =

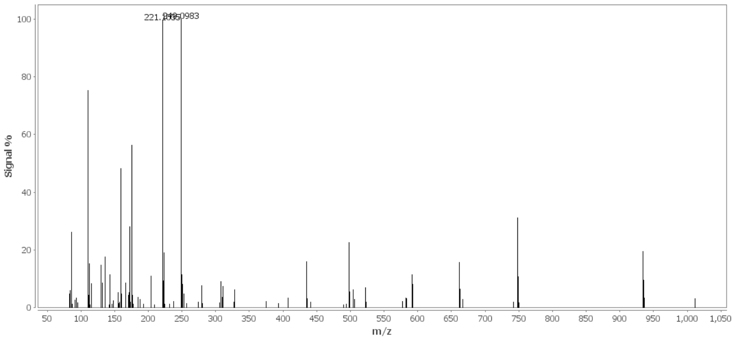

MS2 (+) FT activ = HCD:ce =

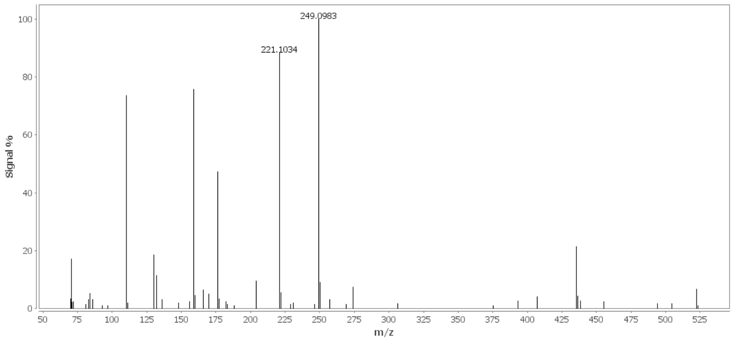

Metabolite: M2 -479 RT=1.57

Type	score	sub. m/z observed	sub. m/z calculated	sub ppm		met. m/z observed	met. m/z calculated	met. ppm
MATCH	108.3	591.7950	591.7938	-1.98	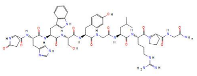 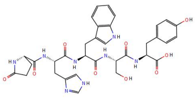	703.2849	703.2835	-2.12
MATCH	11.2	84.0451	84.0444	-8.02	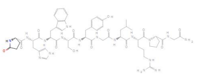 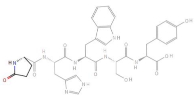	84.0450	84.0444	-7.56

Metabolite: M2 -479 RT=1.57

Type	score	sub. m/z observed	sub. m/z calculated	sub ppm		met. m/z observed	met. m/z calculated	met. ppm
MATCH	4.3	93.0455	93.0447	-8.08	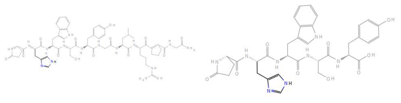	93.0453	93.0447	-6.35
MATCH	148.7	110.0717	110.0713	-3.84	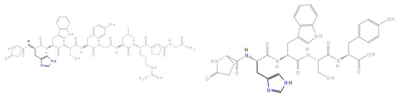	110.0717	110.0713	-4.11
MATCH	20.6	136.0758	136.0757	-0.96	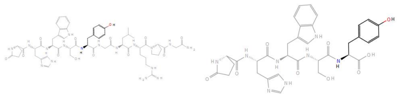	136.0758	136.0757	-0.93
MATCH	123.9	159.0917	159.0917	-0.18	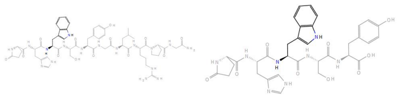	159.0918	159.0917	-0.77
MATCH	14.9	166.0611	166.0611	-0.25	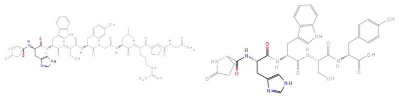	166.0610	166.0611	0.77
MATCH	9.3	170.0602	170.0600	-1.11	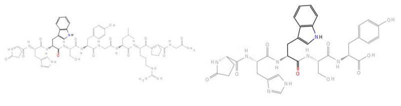	170.0600	170.0600	0.35
MATCH	187.8	221.1035	221.1033	-1.10	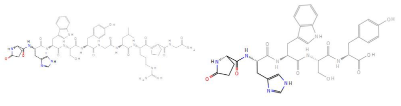	221.1034	221.1033	-0.36
MATCH	200.0	249.0983	249.0982	-0.29	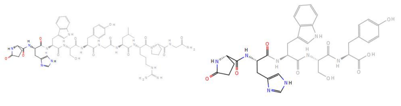	249.0983	249.0982	-0.43
MATCH	4.6	257.0908	257.0921	4.76	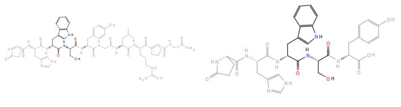	257.0921	257.0921	-0.30

Metabolite: M2 -479 RT=1.57

Type	score	sub. m/z observed	sub. m/z calculated	sub ppm		met. m/z observed	met. m/z calculated	met. ppm
MATCH	9.3	274.1180	274.1186	2.09	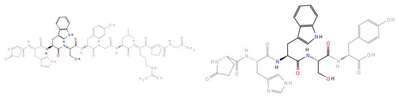	274.1181	274.1186	1.80
MATCH	9.3	274.1180	274.1186	2.09	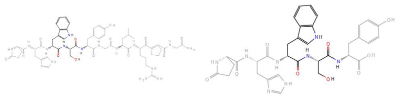	274.1181	274.1186	1.80
MATCH	7.3	407.1830	407.1826	-0.95	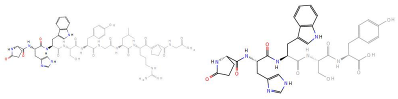	407.1846	407.1826	-4.92
MATCH	37.3	435.1777	435.1775	-0.40	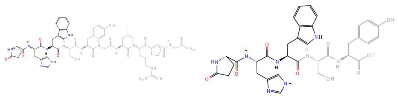	435.1777	435.1775	-0.43
MATCH	3.0	494.2134	494.2146	2.47	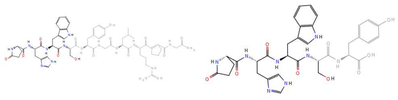	494.2145	494.2146	0.22
MATCH	7.8	504.2000	504.1990	-1.94	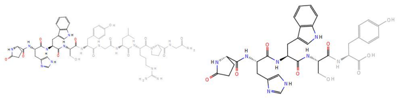	504.2003	504.1990	-2.52
MATCH	13.7	522.2091	522.2096	0.91	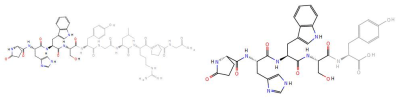	522.2110	522.2096	-2.86
MATCH	17.9	661.3782	661.3780	-0.29	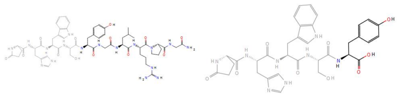	182.0817	182.0812	-2.78
MATCH	32.5	748.4095	748.4100	0.77	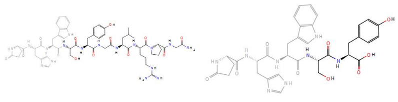	269.1134	269.1132	-0.90

Metabolite: M2 -479 RT=1.57

Type	score	sub. m/z observed	sub. m/z calculated	sub ppm		met. m/z observed	met. m/z calculated	met. ppm
MATCH	21.9	934.4909	934.4894	-1.60	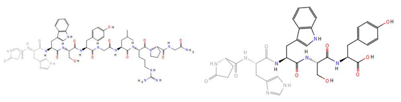	455.1924	455.1925	0.21
MISMATCH	-29.2	86.0971	86.0964	-8.04	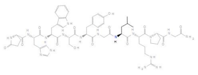	86.0972	86.0972	0.00
MET_MATCH					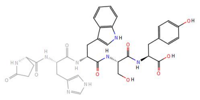	438.1654	438.1660	1.32

MS (+) FT

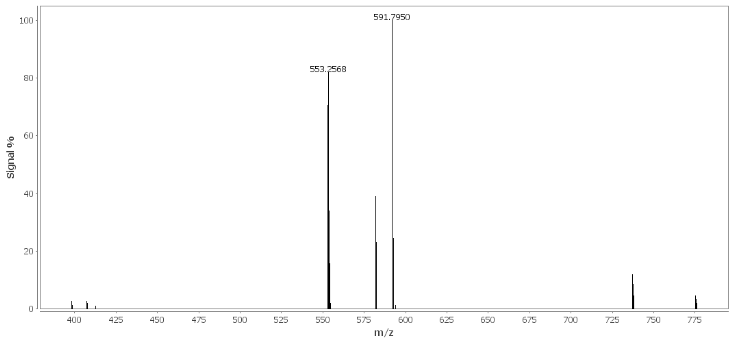

MS (+) FT

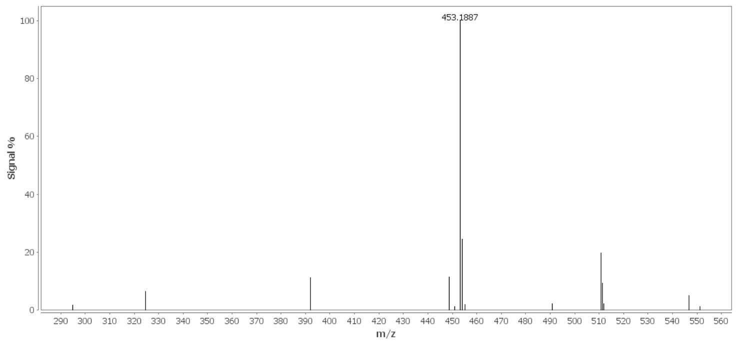

MS2 (+) FT activ = HCD:ce =

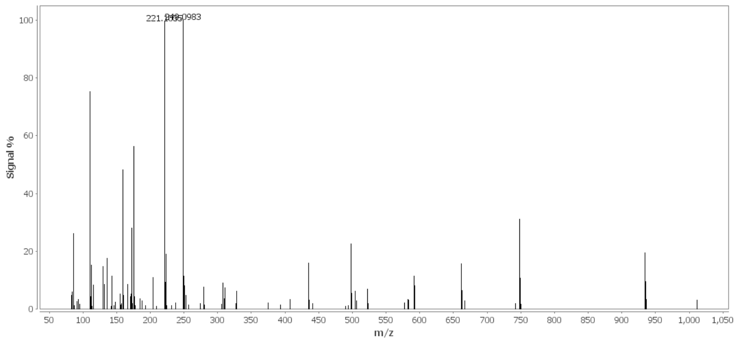

MS2 (+) FT activ = HCD:ce =

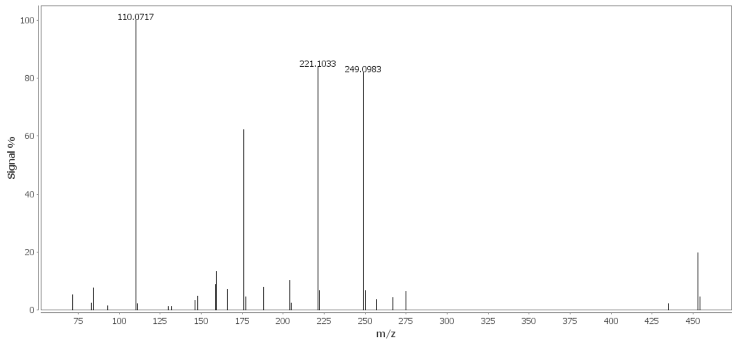

Metabolite: M1 -729 RT=0.47

Type	score	sub. m/z observed	sub. m/z calculated	sub ppm		met. m/z observed	met. m/z calculated	met. ppm
MATCH	200.0	591.7950	591.7938	-1.98	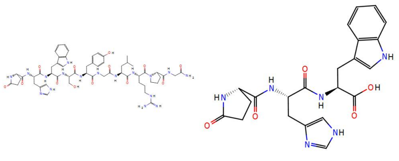	453.1887	453.1881	-1.42

Metabolite: M1 -729 RT=0.47

Type	score	sub. m/z observed	sub. m/z calculated	sub ppm		met. m/z observed	met. m/z calculated	met. ppm
MATCH	13.6	84.0451	84.0444	-8.02	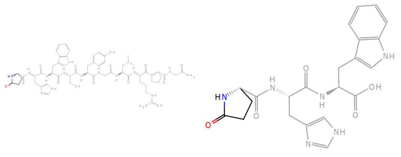	84.0450	84.0444	-7.41
MATCH	4.6	93.0455	93.0447	-8.08	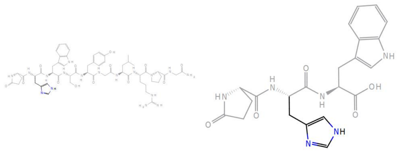	93.0453	93.0447	-6.21
MATCH	175.2	110.0717	110.0713	-3.84	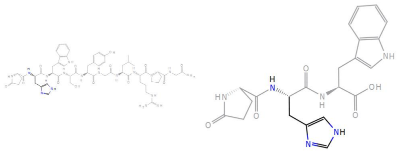	110.0717	110.0713	-3.89
MATCH	61.4	159.0917	159.0917	-0.18	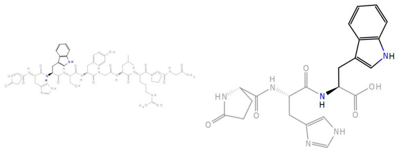	159.0916	159.0917	0.42
MATCH	15.6	166.0611	166.0611	-0.25	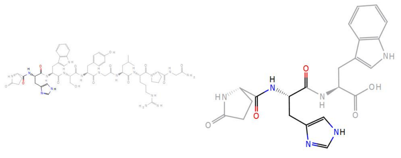	166.0610	166.0611	0.88
MATCH	183.3	221.1035	221.1033	-1.10	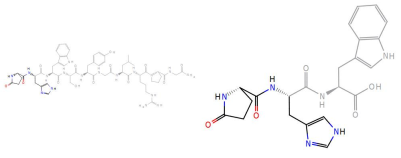	221.1033	221.1033	0.19
MATCH	181.9	249.0983	249.0982	-0.29	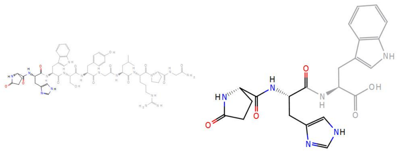	249.0983	249.0982	-0.25
MATCH	31.2	591.7943	591.7938	-0.82	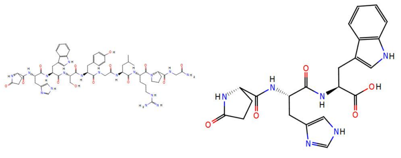	453.1883	453.1881	-0.53
MET_MATCH					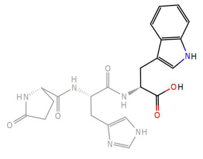	188.0706	188.0706	-0.09

Metabolite: M1 -729 RT=0.47

Type	score	sub. m/z observed	sub. m/z calculated	sub ppm		met. m/z observed	met. m/z calculated	met. ppm
MET_MATCH					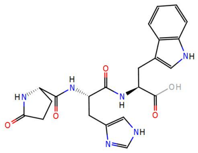	435.1767	435.1775	2.01
