# Supplementary material for: Software-aided approach to investigate peptide structure and metabolic susceptibility of amide bonds in peptide drugs based on high resolution mass spectrometry
Source: PLoS One. 2017 Nov 1;12(11):e0186461. doi: 10.1371/journal.pone.0186461 (PMC5665424; doi:10.1371/journal.pone.0186461)
Supplement: S1 File — (ZIP) [file pone.0186461.s007.zip › SFiles/S15_File.pdf]

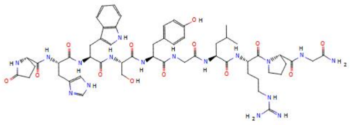

Gonadorelin

Property name	Property value
Time	0min, 5min, 15min, 45min, 120min
Instrument	ThermoQAPlus
Matrix	elastase
Acquisition Mode	ddMS2

Chromatograms

Time=0min

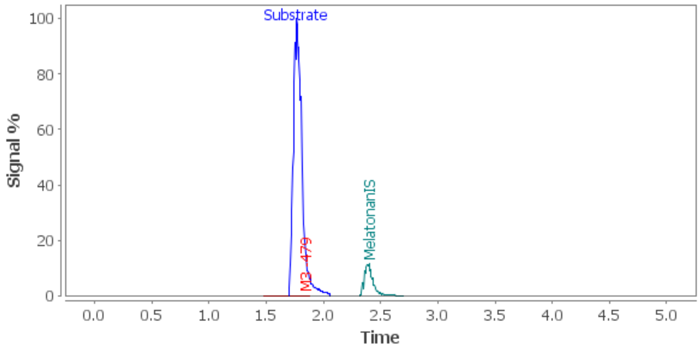

Time=5min

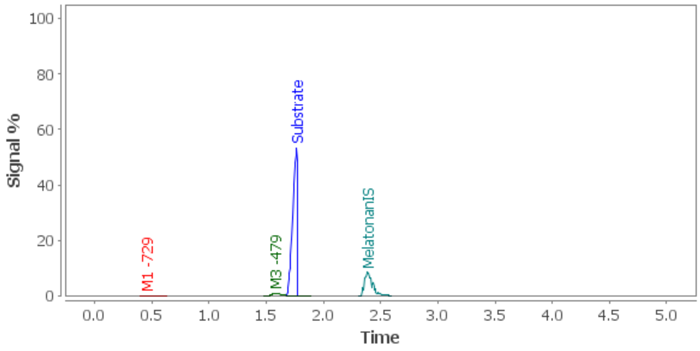

Time=15min

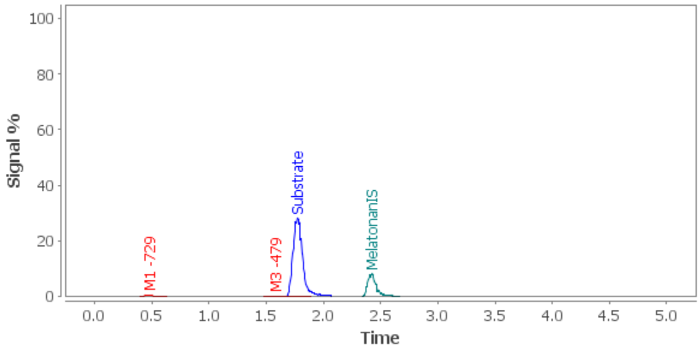

Time=45min

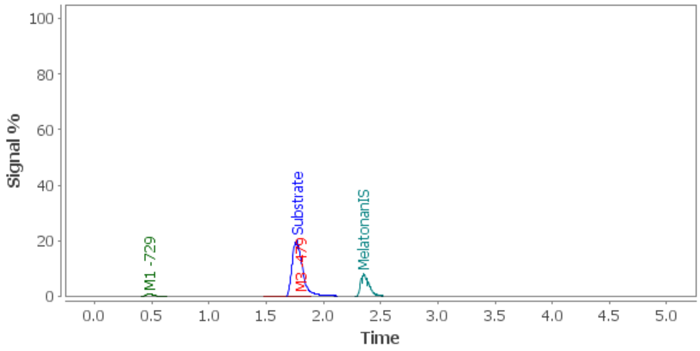

Time=120min

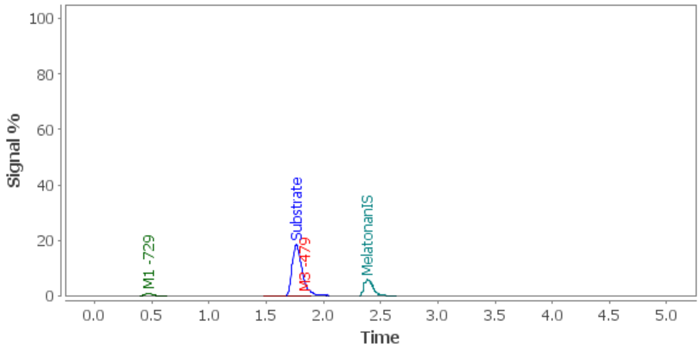

Custom Charts

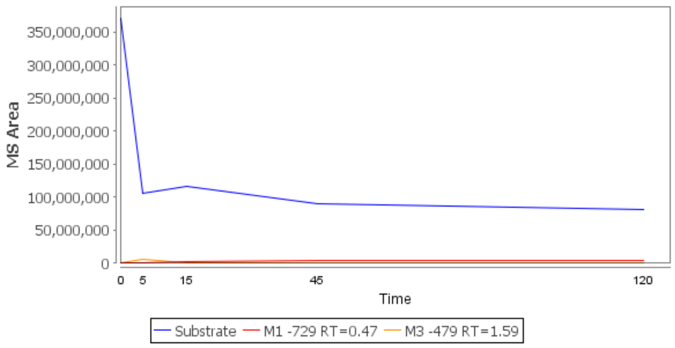

Fragmentation

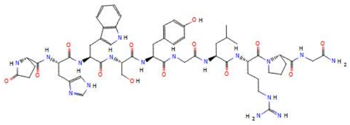

Gonadorelin

MS (+) FT

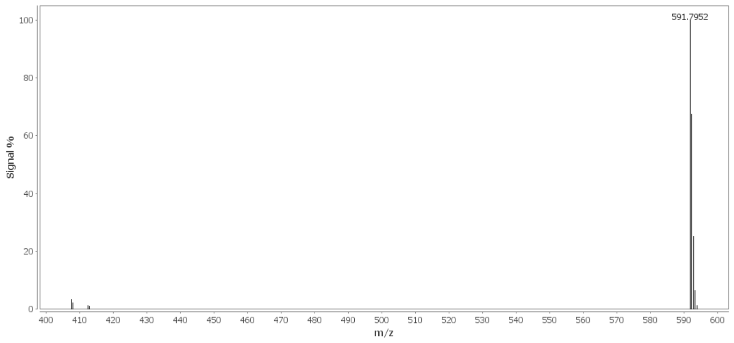

MS (+) FT

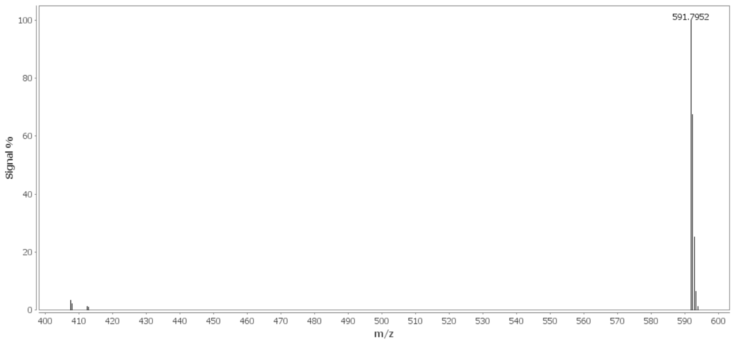

MS2 (+) FT activ = HCD:ce =

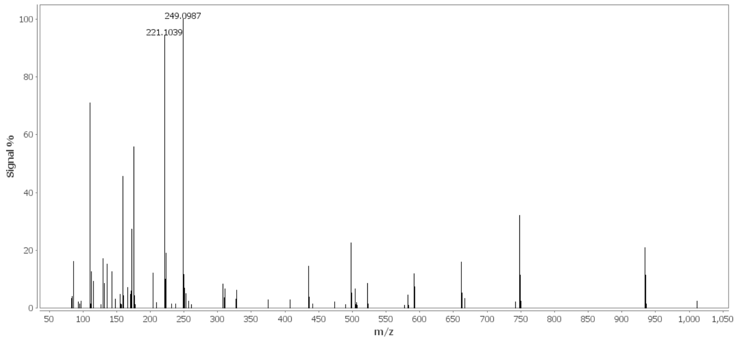

MS2 (+) FT activ = HCD:ce =

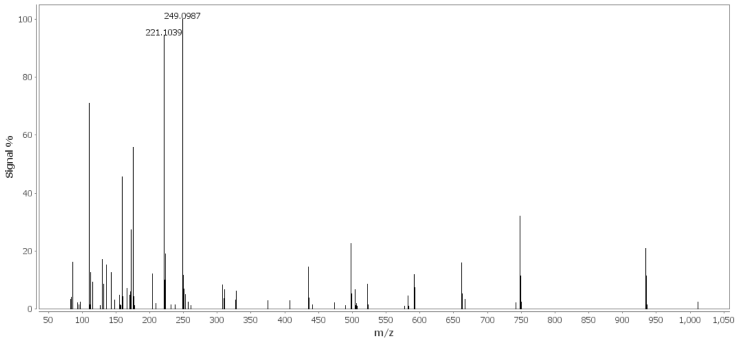

Metabolite: Substrate

Type	score	sub. m/z observed	sub. m/z calculated	sub ppm		met. m/z observed	met. m/z calculated	met. ppm
MATCH	27.2	591.7957	591.7938	-3.19	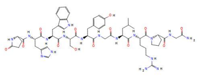 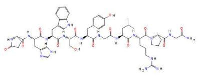	591.7957	591.7938	-3.19
MATCH	200.0	591.7952	591.7938	-2.41	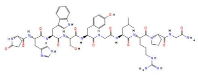 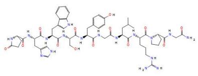	591.7952	591.7938	-2.41
MATCH	10.9	522.2101	522.2096	-1.06	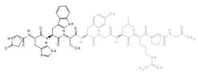 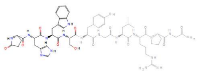	522.2101	522.2096	-1.06

Metabolite: Substrate

Type	score	sub. m/z observed	sub. m/z calculated	sub ppm		met. m/z observed	met. m/z calculated	met. ppm
MATCH	31.8	435.1785	435.1775	-2.17	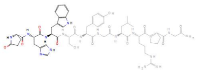 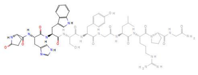	435.1785	435.1775	-2.17
MATCH	200.0	249.0987	249.0982	-1.81	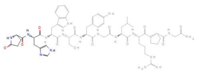 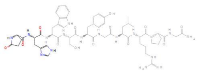	249.0987	249.0982	-1.81
MATCH	180.5	221.1039	221.1033	-2.62	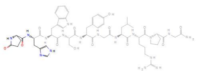 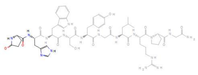	221.1039	221.1033	-2.62
MATCH	6.8	170.0604	170.0600	-2.17	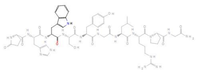 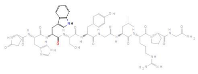	170.0604	170.0600	-2.17
MATCH	12.4	166.0615	166.0611	-2.61	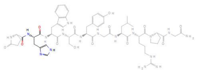 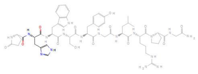	166.0615	166.0611	-2.61
MATCH	113.5	159.0920	159.0917	-1.89	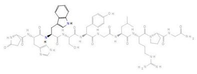 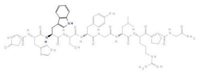	159.0920	159.0917	-1.89
MATCH	16.9	136.0760	136.0757	-2.50	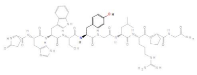 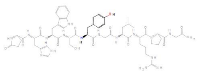	136.0760	136.0757	-2.50
MATCH	142.1	110.0719	110.0713	-5.36	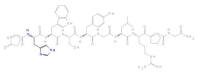 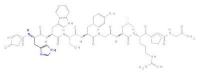	110.0719	110.0713	-5.36
MISMATCH	-19.1	86.0972	86.0964	-9.45	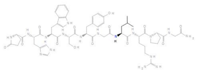 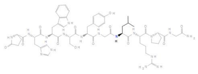	86.0972	86.0964	-9.45

Metabolite: Substrate

Type	score	sub. m/z observed	sub. m/z calculated	sub ppm	met. m/z observed	met. m/z calculated	met. ppm
MATCH	7.7	84.0453	84.0444	-11.3	84.0453	84.0444	-11.3

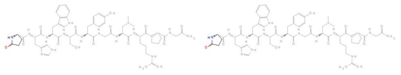

MS (+) FT

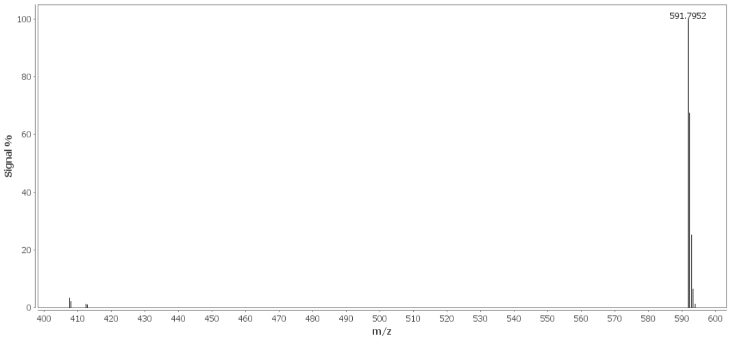

MS (+) FT

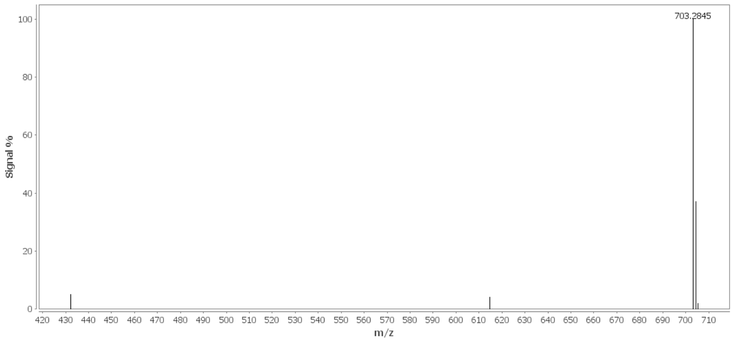

MS2 (+) FT activ = HCD:ce =

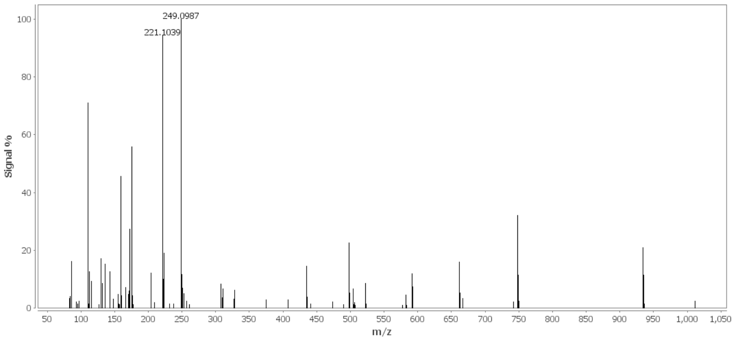

MS2 (+) FT activ = HCD:ce =

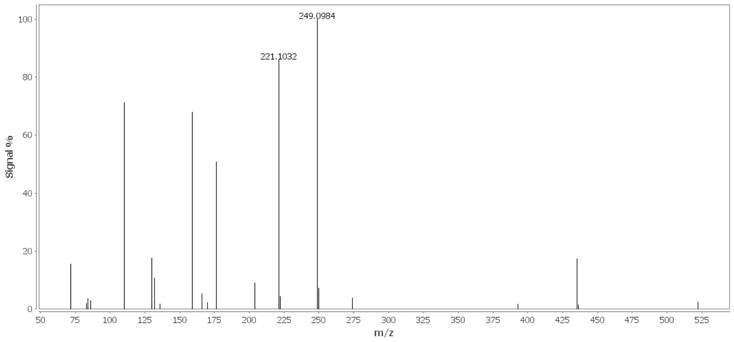

Metabolite: M3 -479 RT=1.59

Type	score	sub. m/z observed	sub. m/z calculated	sub ppm	met. m/z observed	met. m/z calculated	met. ppm
MATCH	200.0	591.7952	591.7938	-2.41	703.2845	703.2835	-1.52

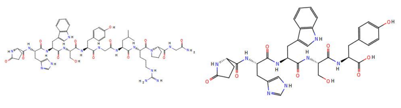

Type	score	sub. m/z observed	sub. m/z calculated	sub ppm	met. m/z observed	met. m/z calculated	met. ppm
MATCH	7.7	84.0453	84.0444	-11.3	84.0452	84.0444	-9.69

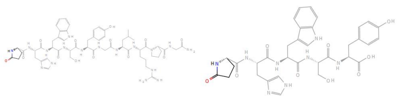

Type	score	sub. m/z observed	sub. m/z calculated	sub ppm	met. m/z observed	met. m/z calculated	met. ppm
MATCH	142.1	110.0719	110.0713	-5.36	110.0717	110.0713	-4.31

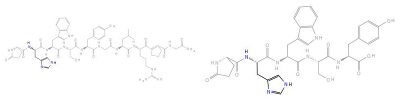

Metabolite: M3 -479 RT=1.59

Type	score	sub. m/z observed	sub. m/z calculated	sub ppm		met. m/z observed	met. m/z calculated	met. ppm
MATCH	16.9	136.0760	136.0757	-2.50	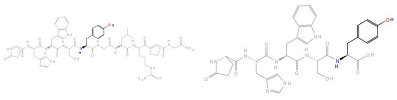	136.0759	136.0757	-1.75
MATCH	113.5	159.0920	159.0917	-1.89	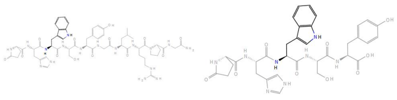	159.0917	159.0917	-0.22
MATCH	12.4	166.0615	166.0611	-2.61	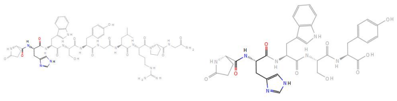	166.0610	166.0611	0.38
MATCH	6.8	170.0604	170.0600	-2.17	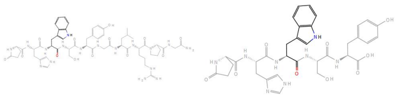	170.0602	170.0600	-0.74
MATCH	180.5	221.1039	221.1033	-2.62	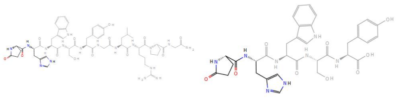	221.1032	221.1033	0.27
MATCH	200.0	249.0987	249.0982	-1.81	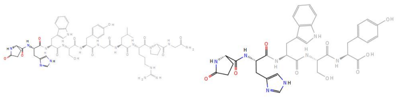	249.0984	249.0982	-0.57
MATCH	31.8	435.1785	435.1775	-2.17	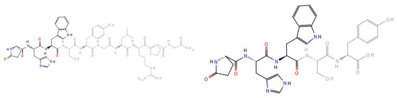	435.1763	435.1775	2.76
MATCH	10.9	522.2101	522.2096	-1.06	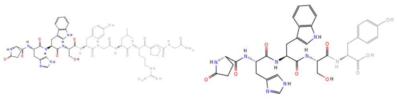	522.2064	522.2096	6.01
MISMATCH	-19.1	86.0972	86.0964	-9.45	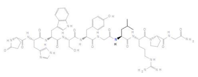	86.0972	86.0972	0.00

MS (+) FT

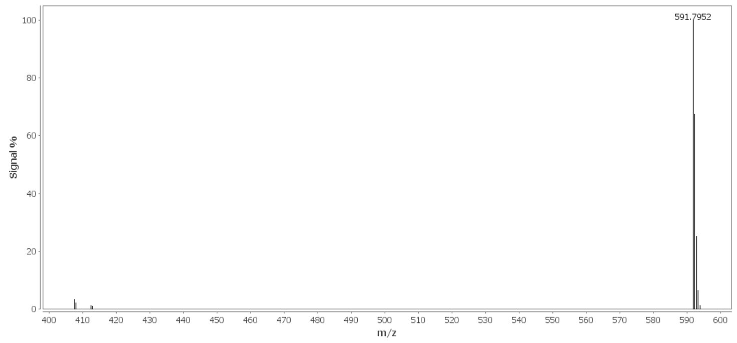

MS (+) FT

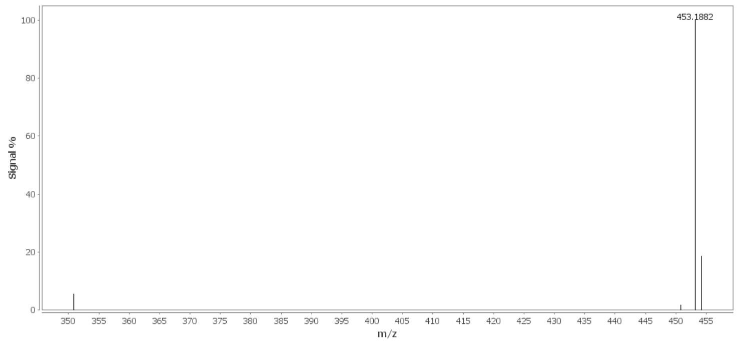

MS2 (+) FT activ = HCD:ce =

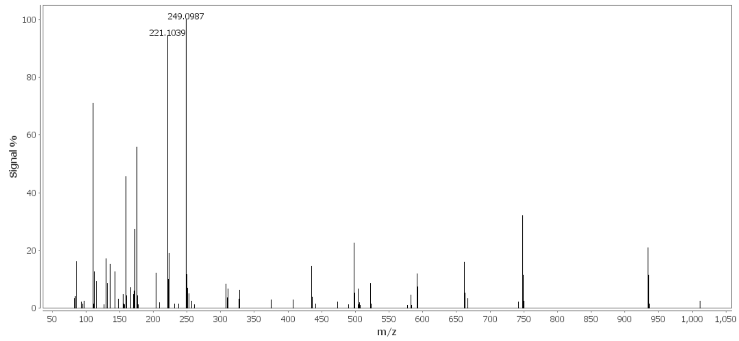

MS2 (+) FT activ = HCD:ce =

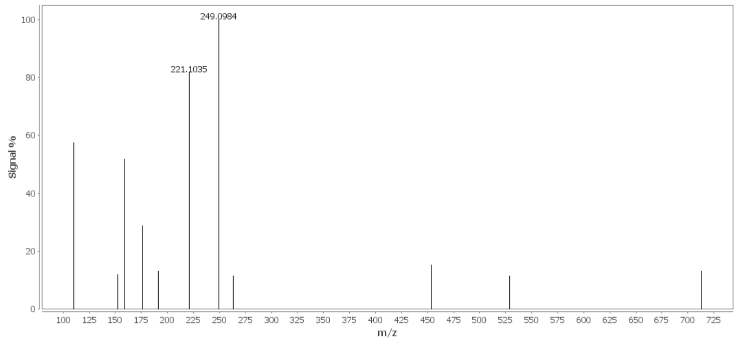

Metabolite: M1 -729 RT=0.47

Type	score	sub. m/z observed	sub. m/z calculated	sub ppm			met. m/z observed	met. m/z calculated	met. ppm
MATCH	200.0	591.7952	591.7938	-2.41	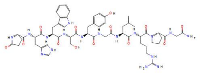	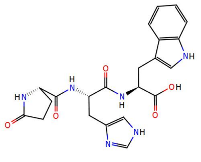	453.1882	453.1881	-0.16
MATCH	128.3	110.0719	110.0713	-5.36	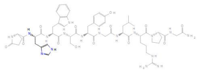	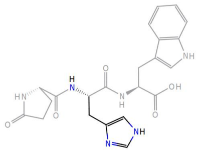	110.0715	110.0713	-1.87
MATCH	176.2	221.1039	221.1033	-2.62	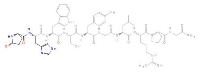	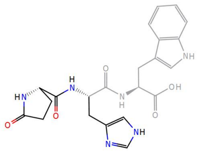	221.1035	221.1033	-0.90
MATCH	200.0	249.0987	249.0982	-1.81	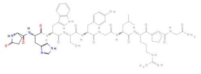	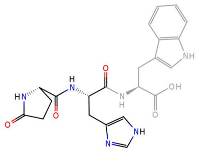	249.0984	249.0982	-0.81
MATCH	27.2	591.7957	591.7938	-3.19	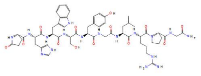	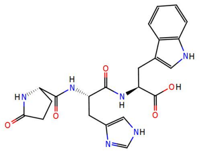	453.1855	453.1881	5.69
