# Supplementary material for: Software-aided approach to investigate peptide structure and metabolic susceptibility of amide bonds in peptide drugs based on high resolution mass spectrometry
Source: PLoS One. 2017 Nov 1;12(11):e0186461. doi: 10.1371/journal.pone.0186461 (PMC5665424; doi:10.1371/journal.pone.0186461)
Supplement: S1 File — (ZIP) [file pone.0186461.s007.zip › SFiles/S16_File.pdf]

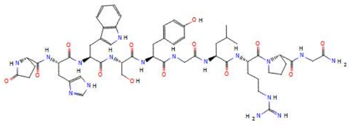

Gonadorelin

Property name	Property value
Time	0min, 5min, 15min, 45min, 120min
Instrument	ThermoQAPlus
Acquisition Mode	ddMS2
Matrix	pepsin

Chromatograms

Time=0min

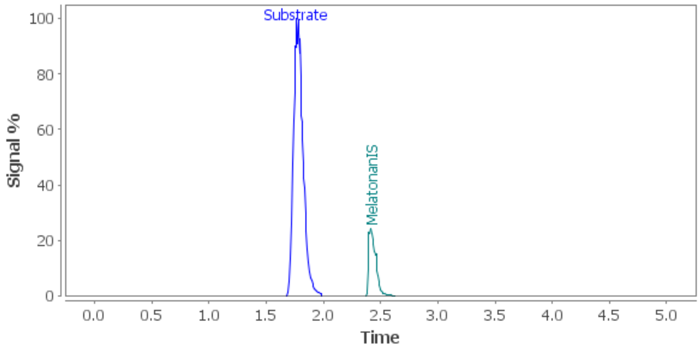

Time=5min

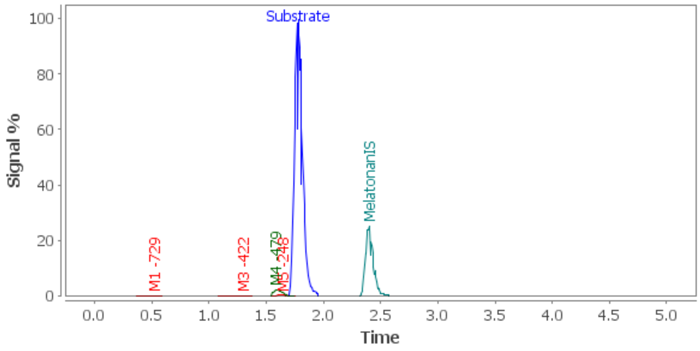

Time=15min

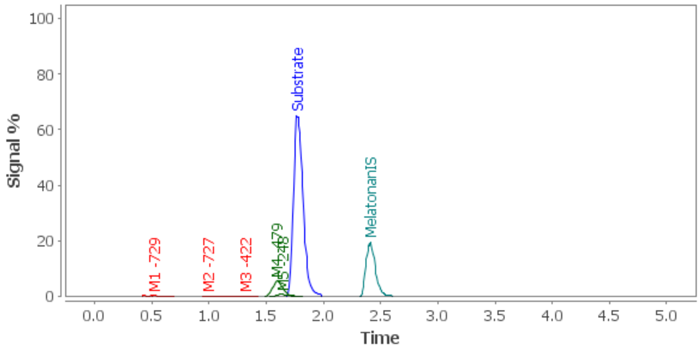

Time=45min

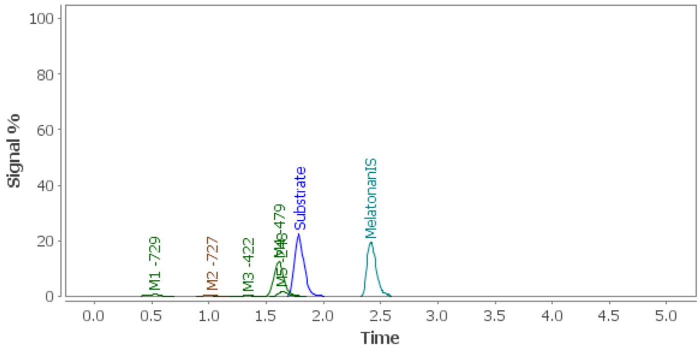

Time=120min

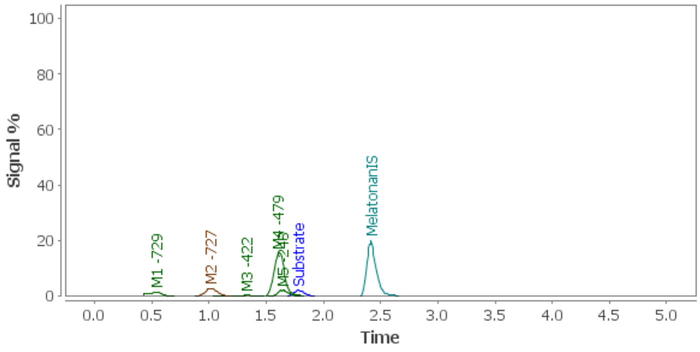

Custom Charts

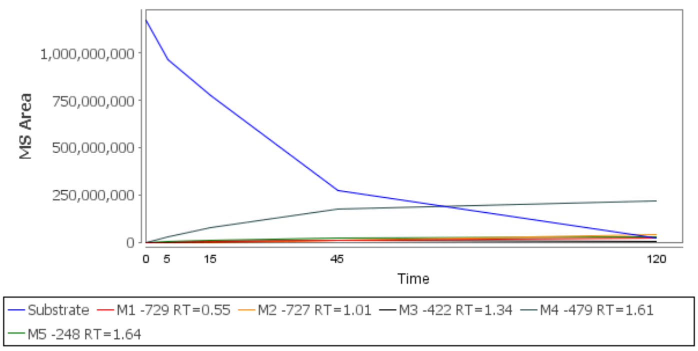

Fragmentation

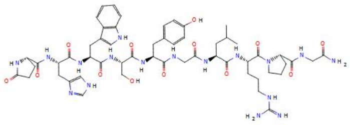

Gonadorelin

MS (+) FT

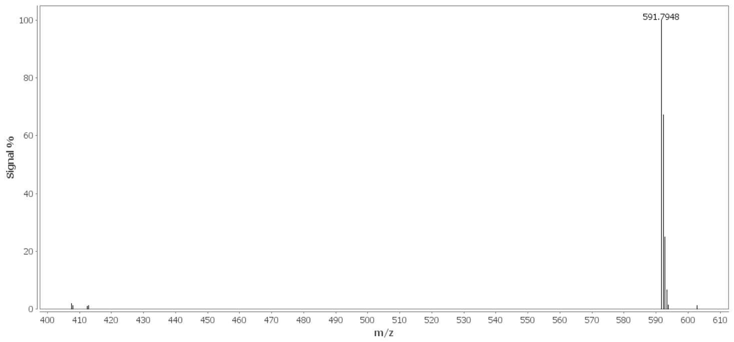

MS (+) FT

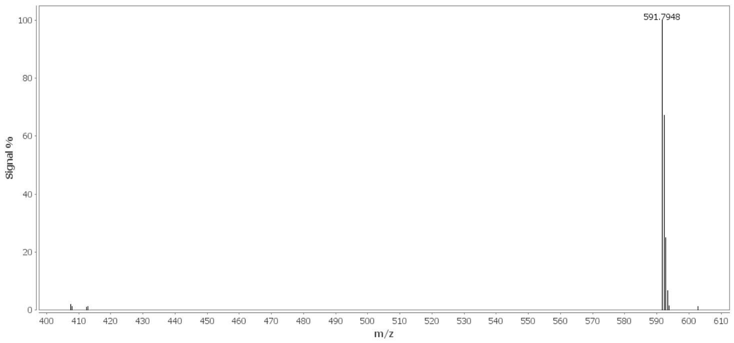

MS2 (+) FT activ = HCD:ce =

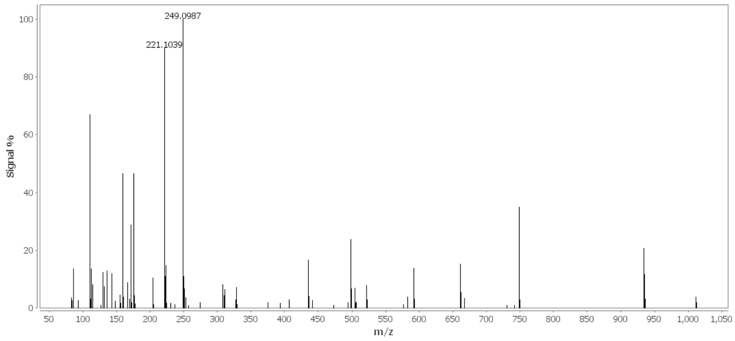

MS2 (+) FT activ = HCD:ce =

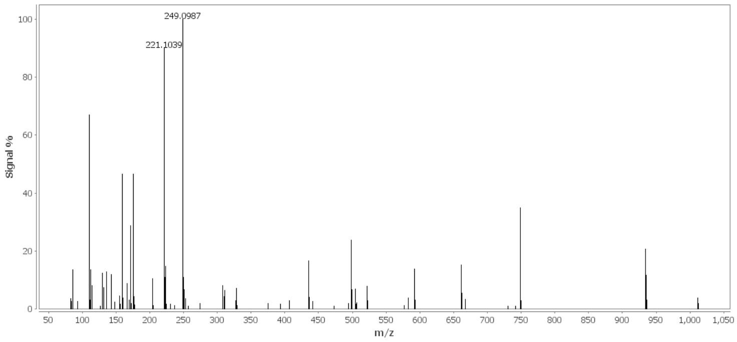

Metabolite: Substrate

Type	score	sub. m/z observed	sub. m/z calculated	sub ppm		met. m/z observed	met. m/z calculated	met. ppm
MATCH	23.7	934.4909	934.4894	-1.67	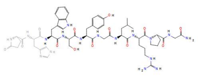 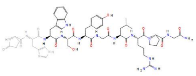	934.4909	934.4894	-1.67
MATCH	52.4	748.4114	748.4100	-1.86	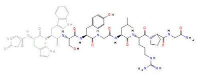 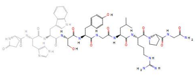	748.4114	748.4100	-1.86
MATCH	39.9	661.3794	661.3780	-2.01	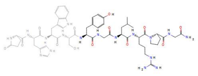 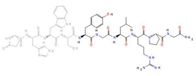	661.3794	661.3780	-2.01

Metabolite: Substrate

Type	score	sub. m/z observed	sub. m/z calculated	sub ppm		met. m/z observed	met. m/z calculated	met. ppm
MATCH	27.9	591.7958	591.7938	-3.38	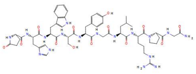 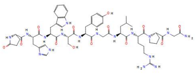	591.7958	591.7938	-3.38
MATCH	200.0	591.7948	591.7938	-1.77	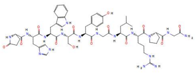 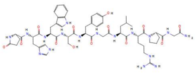	591.7948	591.7938	-1.77
MATCH	16.8	522.2112	522.2096	-3.08	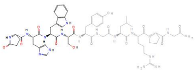 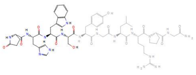	522.2112	522.2096	-3.08
MATCH	13.3	504.2001	504.1990	-2.20	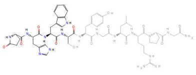 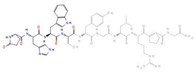	504.2001	504.1990	-2.20
MATCH	49.8	498.3158	498.3147	-2.22	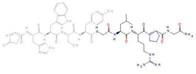 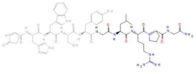	498.3158	498.3147	-2.22
MATCH	8.4	494.2120	494.2146	5.39	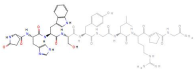 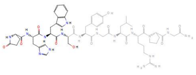	494.2120	494.2146	5.39
MATCH	6.1	441.2943	441.2932	-2.34	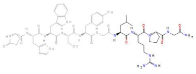 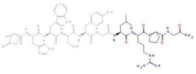	441.2943	441.2932	-2.34
MATCH	41.5	435.1786	435.1775	-2.36	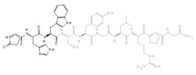 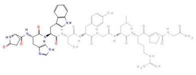	435.1786	435.1775	-2.36
MATCH	102.8	407.1831	407.1826	-1.24	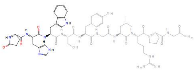 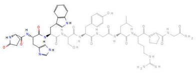	407.1831	407.1826	-1.24

Metabolite: Substrate

Type	score	sub. m/z observed	sub. m/z calculated	sub ppm		met. m/z observed	met. m/z calculated	met. ppm
MATCH	13.4	328.2098	328.2092	-1.87	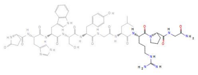 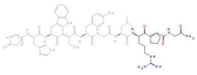	328.2098	328.2092	-1.87
MATCH	6.8	327.2143	327.2139	-1.06	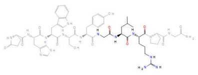 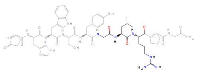	327.2143	327.2139	-1.06
MATCH	12.6	311.1840	311.1826	-4.37	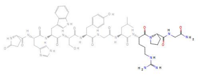 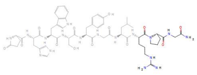	311.1840	311.1826	-4.37
MATCH	5.9	310.1882	310.1874	-2.73	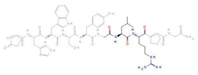 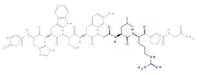	310.1882	310.1874	-2.73
MATCH	13.2	308.1250	308.1241	-3.03	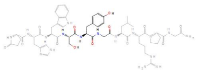 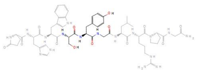	308.1250	308.1241	-3.03
MATCH	13.2	308.1250	308.1241	-3.03	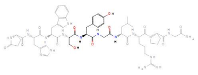 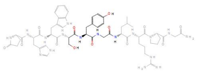	308.1250	308.1241	-3.03
MATCH	9.2	274.1189	274.1186	-0.86	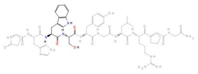 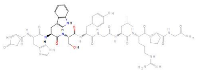	274.1189	274.1186	-0.86
MATCH	10.2	274.1189	274.1186	-0.86	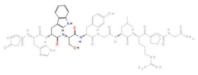 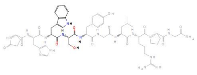	274.1189	274.1186	-0.86
MATCH	17.5	257.0927	257.0921	-2.28	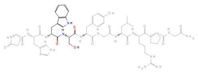 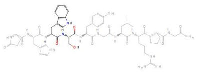	257.0927	257.0921	-2.28

Metabolite: Substrate

Type	score	sub. m/z observed	sub. m/z calculated	sub ppm		met. m/z observed	met. m/z calculated	met. ppm
MATCH	8.4	253.1660	253.1659	-0.32	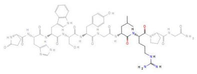 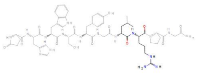	253.1660	253.1659	-0.32
MATCH	200.0	249.0987	249.0982	-1.79	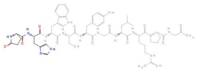 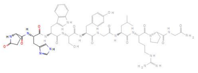	249.0987	249.0982	-1.79
MATCH	186.3	221.1039	221.1033	-2.84	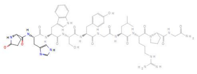 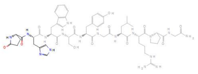	221.1039	221.1033	-2.84
MATCH	54.3	172.1082	172.1081	-0.79	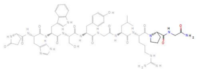 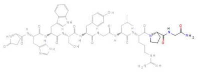	172.1082	172.1081	-0.79
MATCH	11.1	171.1130	171.1128	-0.92	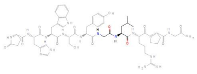 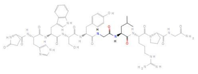	171.1130	171.1128	-0.92
MATCH	11.1	171.1130	171.1128	-0.92	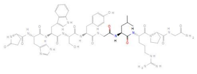 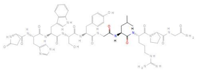	171.1130	171.1128	-0.92
MATCH	45.8	170.0605	170.0600	-2.46	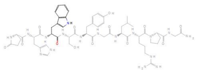 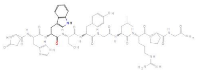	170.0605	170.0600	-2.46
MATCH	17.6	166.0615	166.0611	-2.28	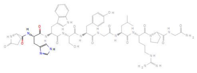 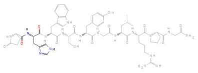	166.0615	166.0611	-2.28
MATCH	116.6	159.0920	159.0917	-1.91	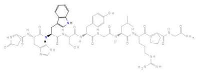 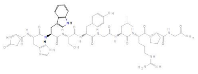	159.0920	159.0917	-1.91

Metabolite: Substrate

Type	score	sub. m/z observed	sub. m/z calculated	sub ppm			met. m/z observed	met. m/z calculated	met. ppm
MATCH	7.9	155.0816	155.0815	-0.52	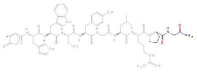	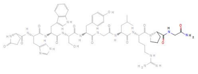	155.0816	155.0815	-0.52
MATCH	7.9	155.0816	155.0815	-0.52	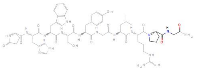	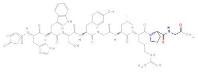	155.0816	155.0815	-0.52
MISMATCH	-4.6	148.0873	148.0793	-54.2	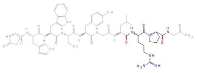	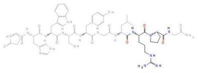	148.0873	148.0793	-54.2
MATCH	25.0	143.1182	143.1179	-2.43	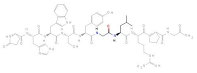	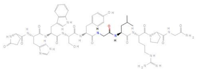	143.1182	143.1179	-2.43
MATCH	42.0	136.0761	136.0757	-3.16	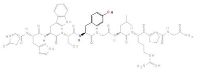	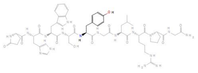	136.0761	136.0757	-3.16
MATCH	84.4	130.0654	130.0575	-61.1	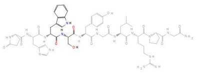	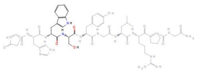	130.0654	130.0575	-61.1
MATCH	19.1	115.0871	115.0866	-4.34	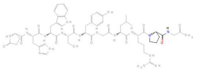	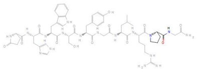	115.0871	115.0866	-4.34
MATCH	29.4	112.0876	112.0869	-5.62	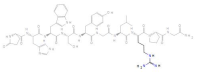	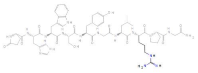	112.0876	112.0869	-5.62
MATCH	166.8	110.0719	110.0713	-5.46	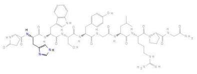	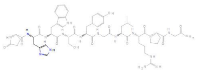	110.0719	110.0713	-5.46

Metabolite: Substrate

Type	score	sub. m/z observed	sub. m/z calculated	sub ppm		met. m/z observed	met. m/z calculated	met. ppm
MISMATCH	-3.9	93.0454	93.0553	105.7		93.0454	93.0553	105.7

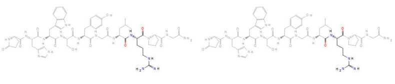

Type	score	sub. m/z observed	sub. m/z calculated	sub ppm		met. m/z observed	met. m/z calculated	met. ppm
MATCH	3.9	93.0454	93.0447	-7.76		93.0454	93.0447	-7.76

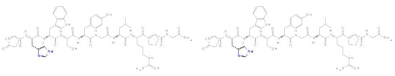

Type	score	sub. m/z observed	sub. m/z calculated	sub ppm		met. m/z observed	met. m/z calculated	met. ppm
MISMATCH	62.4	86.0972	86.0964	-9.19		86.0972	86.0964	-9.19

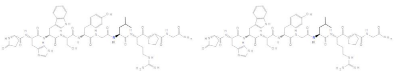

MS (+) FT

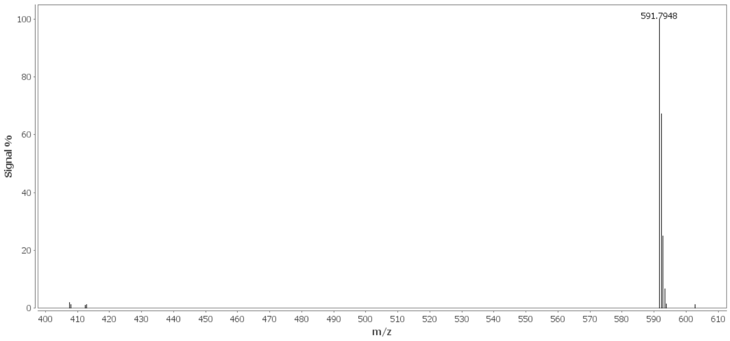

MS (+) FT

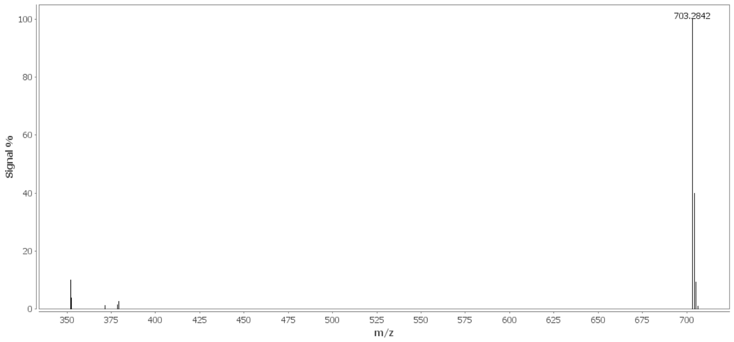

MS2 (+) FT activ = HCD:ce =

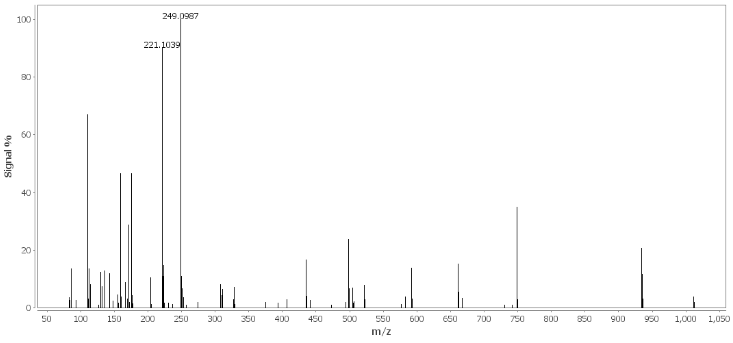

MS2 (+) FT activ = HCD:ce =

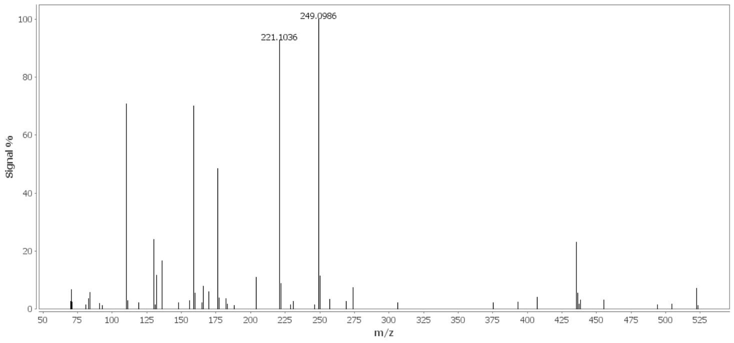

Metabolite: M4 -479 RT=1.61

Type	score	sub. m/z observed	sub. m/z calculated	sub ppm		met. m/z observed	met. m/z calculated	met. ppm
MATCH	109.9	591.7948	591.7938	-1.77		352.1459	352.1454	-1.57

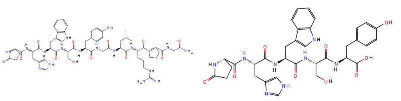

Metabolite: M4 -479 RT=1.61

Type	score	sub. m/z observed	sub. m/z calculated	sub ppm			met. m/z observed	met. m/z calculated	met. ppm
MATCH	200.0	591.7948	591.7938	-1.77	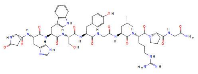	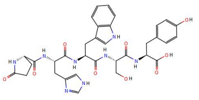	703.2842	703.2835	-1.13
MATCH	3.9	93.0454	93.0447	-7.76	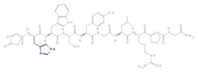	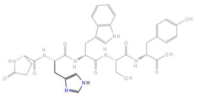	93.0453	93.0447	-6.60
MATCH	137.4	110.0719	110.0713	-5.46	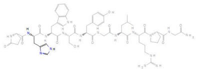	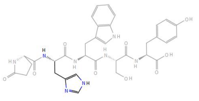	110.0719	110.0713	-5.29
MATCH	36.4	130.0654	130.0575	-61.1	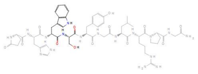	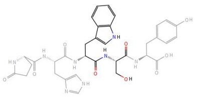	130.0655	130.0575	-61.1
MATCH	29.7	136.0761	136.0757	-3.16	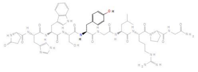	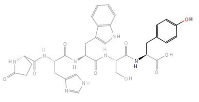	136.0761	136.0757	-2.76
MATCH	116.6	159.0920	159.0917	-1.91	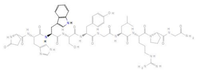	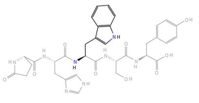	159.0919	159.0917	-1.72
MATCH	16.8	166.0615	166.0611	-2.28	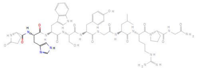	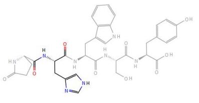	166.0614	166.0611	-1.70
MATCH	9.1	170.0605	170.0600	-2.46	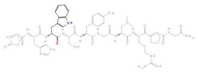	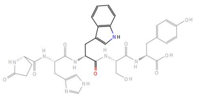	170.0603	170.0600	-1.72
MATCH	182.6	221.1039	221.1033	-2.84	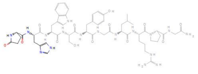	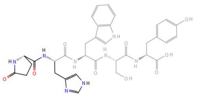	221.1036	221.1033	-1.28

Metabolite: M4 -479 RT=1.61

Type	score	sub. m/z observed	sub. m/z calculated	sub ppm		met. m/z observed	met. m/z calculated	met. ppm
MATCH	200.0	249.0987	249.0982	-1.79	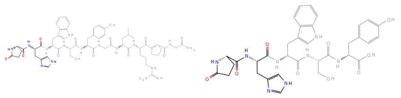	249.0986	249.0982	-1.49
MATCH	4.5	257.0927	257.0921	-2.28	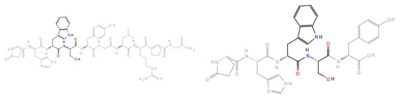	257.0919	257.0921	0.64
MATCH	9.2	274.1189	274.1186	-0.86	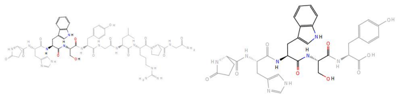	274.1188	274.1186	-0.74
MATCH	9.2	274.1189	274.1186	-0.86	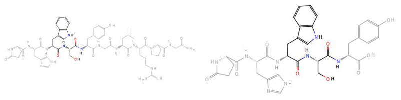	274.1188	274.1186	-0.74
MATCH	6.9	407.1831	407.1826	-1.24	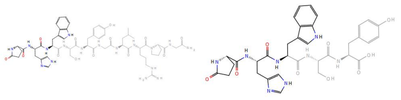	407.1838	407.1826	-2.84
MATCH	39.7	435.1786	435.1775	-2.36	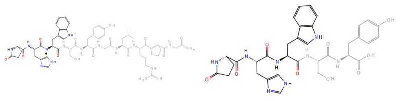	435.1784	435.1775	-1.90
MATCH	3.3	494.2120	494.2146	5.39	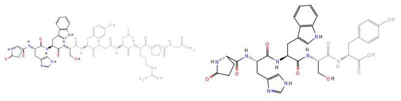	494.2164	494.2146	-3.60
MATCH	8.5	504.2001	504.1990	-2.20	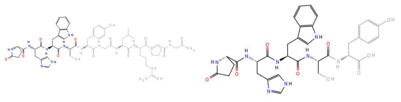	504.2009	504.1990	-3.80
MATCH	15.0	522.2112	522.2096	-3.08	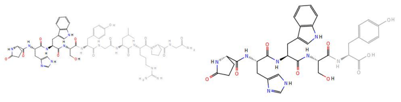	522.2110	522.2096	-2.81

Metabolite: M4 -479 RT=1.61

Type	score	sub. m/z observed	sub. m/z calculated	sub ppm		met. m/z observed	met. m/z calculated	met. ppm
MATCH	18.8	661.3794	661.3780	-2.01	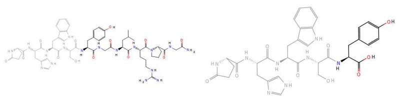	182.0818	182.0812	-3.30
MATCH	37.4	748.4114	748.4100	-1.86	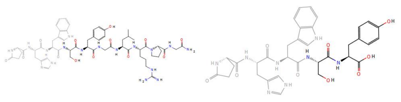	269.1131	269.1132	0.26
MATCH	23.7	934.4909	934.4894	-1.67	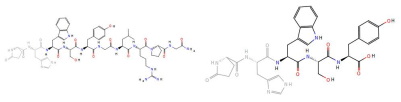	455.1933	455.1925	-1.84
MISMATCH	-3.9	93.0454	93.0553	105.7	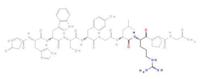	93.0453	93.0453	0.00
MISMATCH	-4.6	148.0873	148.0793	-54.2	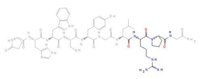	148.0871	148.0871	0.00
MET_MATCH					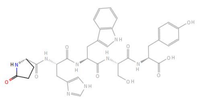	84.0452	84.0444	-9.71
MET_MATCH					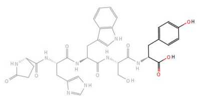	165.0547	165.0546	-0.56
MET_MATCH					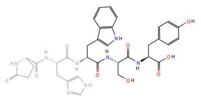	438.1658	438.1660	0.27

MS (+) FT

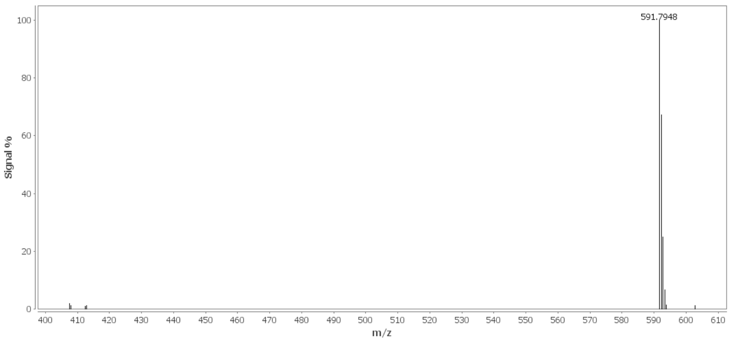

MS (+) FT

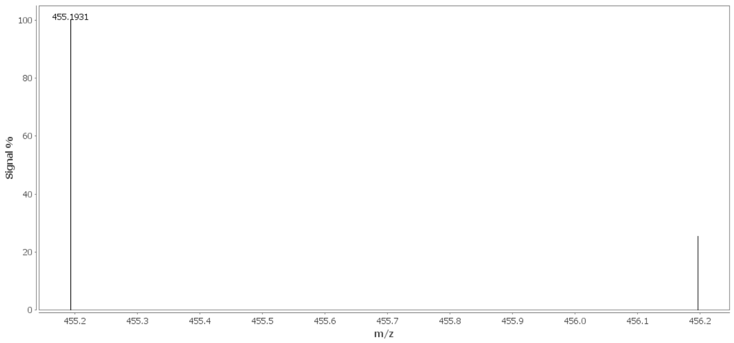

MS2 (+) FT activ = HCD:ce =

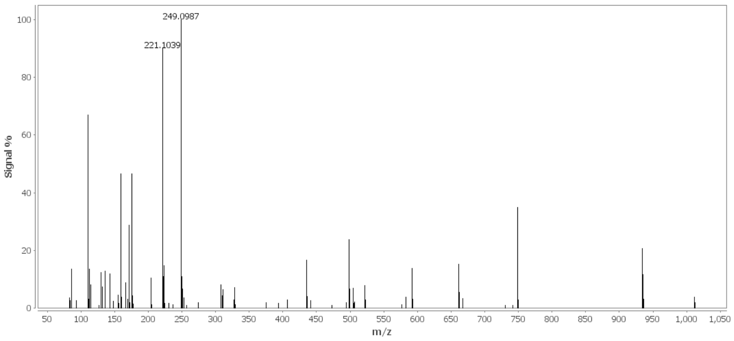

MS2 (+) FT activ = HCD:ce =

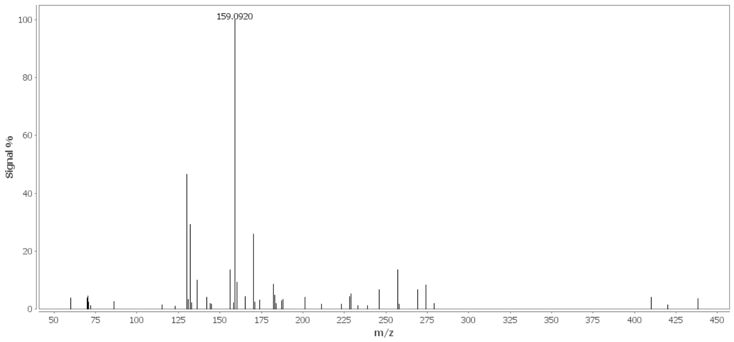

Metabolite: M2 -727 RT=1.01

Type	score	sub. m/z observed	sub. m/z calculated	sub ppm		met. m/z observed	met. m/z calculated	met. ppm
MATCH	200.0	591.7948	591.7938	-1.77	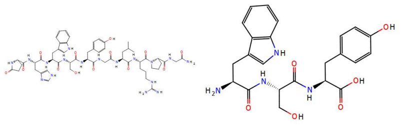	455.1931	455.1925	-1.36
MATCH	23.0	136.0761	136.0757	-3.16	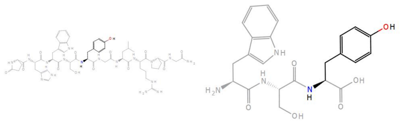	136.0760	136.0757	-2.36
MATCH	29.1	170.0605	170.0600	-2.46	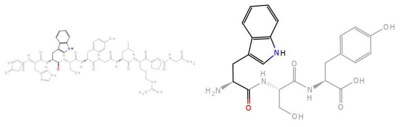	170.0603	170.0600	-1.54
MATCH	14.6	257.0927	257.0921	-2.28	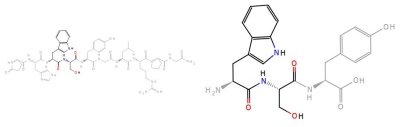	257.0923	257.0921	-0.79
MATCH	10.2	274.1189	274.1186	-0.86	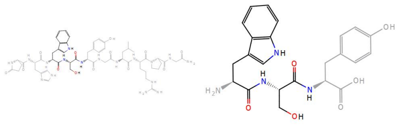	274.1190	274.1186	-1.51

Metabolite: M2 -727 RT=1.01

Type	score	sub. m/z observed	sub. m/z calculated	sub ppm		met. m/z observed	met. m/z calculated	met. ppm
					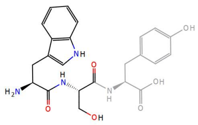	274.1190	274.1186	-1.51
MATCH	102.8	407.1831	407.1826	-1.24	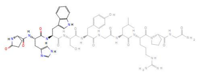 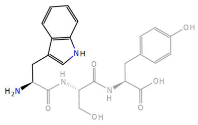	159.0920	159.0917	-1.77
MATCH	19.5	435.1786	435.1775	-2.36	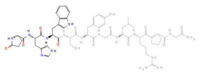 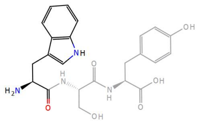	187.0868	187.0866	-1.22
MATCH	8.4	494.2120	494.2146	5.39	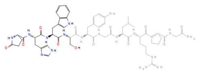 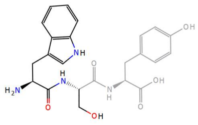	246.1242	246.1237	-1.90
MATCH	16.2	522.2112	522.2096	-3.08	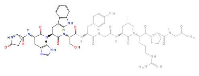 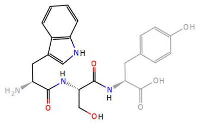	274.1190	274.1186	-1.51
					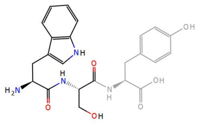	274.1190	274.1186	-1.51
MATCH	23.9	661.3794	661.3780	-2.01	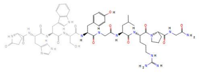 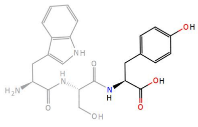	182.0816	182.0812	-2.25
MATCH	41.5	748.4114	748.4100	-1.86	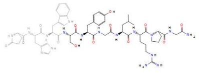 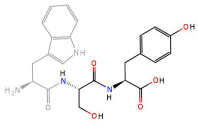	269.1136	269.1132	-1.32
MISMATCH	-16.2	86.0972	86.0964	-9.19	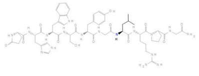	86.0972	86.0972	0.00

Metabolite: M2 -727 RT=1.01

Type	score	sub. m/z observed	sub. m/z calculated	sub ppm		met. m/z observed	met. m/z calculated	met. ppm
MET_MATCH					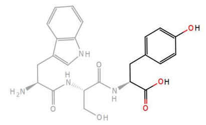	165.0551	165.0546	-2.71
MET_MATCH					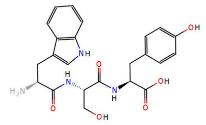	438.1657	438.1660	0.59

MS (+) FT

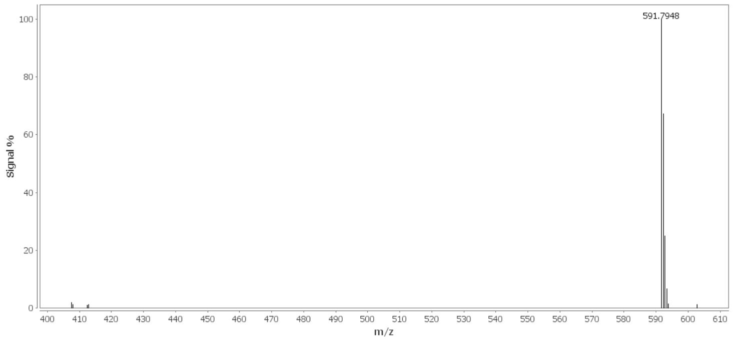

MS (+) FT

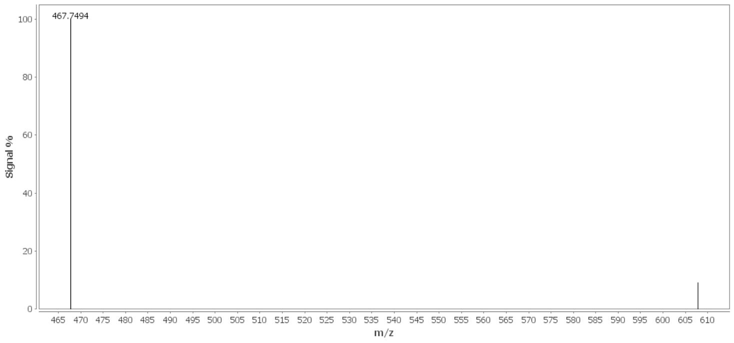

MS2 (+) FT activ = HCD:ce =

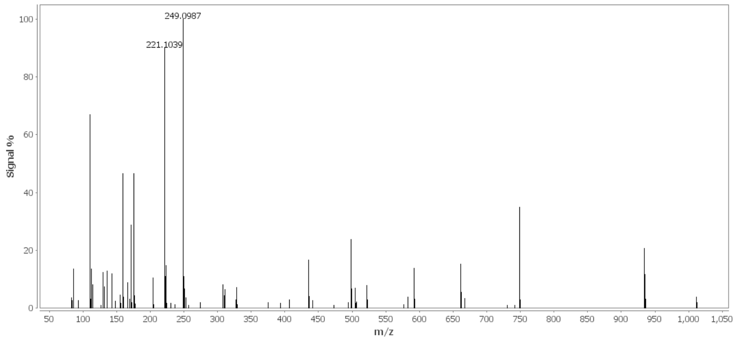

MS2 (+) FT activ = HCD:ce =

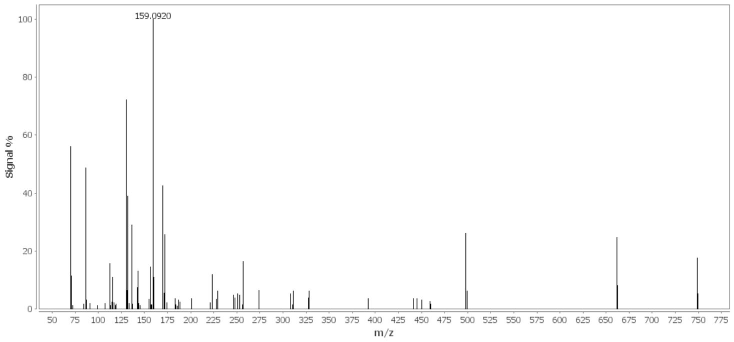

Metabolite: M5 -248 RT=1.64

Type	score	sub. m/z observed	sub. m/z calculated	sub ppm		met. m/z observed	met. m/z calculated	met. ppm
MATCH	200.0	591.7948	591.7938	-1.77	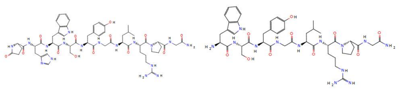	467.7494	467.7483	-2.28
MATCH	62.4	86.0972	86.0964	-9.19	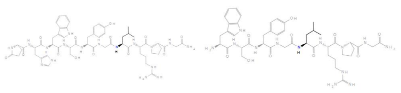	86.0972	86.0964	-9.44

Metabolite: M5 -248 RT=1.64

Type	score	sub. m/z observed	sub. m/z calculated	sub ppm		met. m/z observed	met. m/z calculated	met. ppm
MATCH	29.4	112.0876	112.0869	-5.62	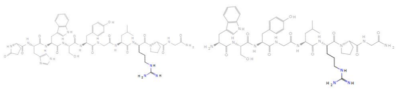	112.0875	112.0869	-4.71
MATCH	19.1	115.0871	115.0866	-4.34	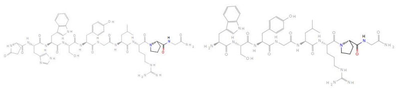	115.0871	115.0866	-4.59
MATCH	84.4	130.0654	130.0575	-61.1	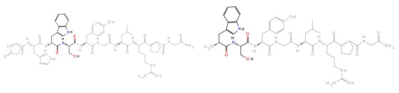	130.0655	130.0575	-61.3
MATCH	42.0	136.0761	136.0757	-3.16	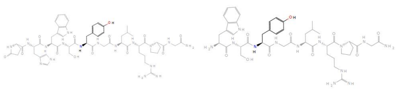	136.0761	136.0757	-3.20
MATCH	25.0	143.1182	143.1179	-2.43	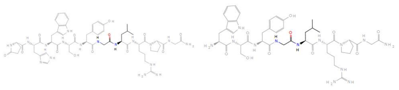	143.1182	143.1179	-2.34
MATCH	7.9	155.0816	155.0815	-0.52	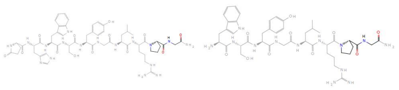	155.0817	155.0815	-1.42
MATCH	7.9	155.0816	155.0815	-0.52	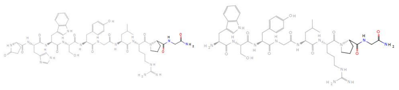	155.0817	155.0815	-1.42
MATCH	45.8	170.0605	170.0600	-2.46	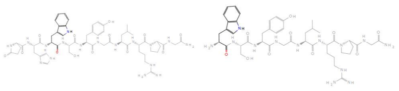	170.0603	170.0600	-1.58
MATCH	11.1	171.1130	171.1128	-0.92	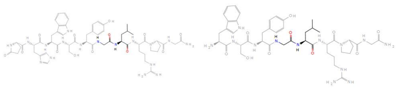	171.1134	171.1128	-3.35

Metabolite: M5 -248 RT=1.64

Type	score	sub. m/z observed	sub. m/z calculated	sub ppm		met. m/z observed	met. m/z calculated	met. ppm
MATCH	11.1	171.1130	171.1128	-0.92	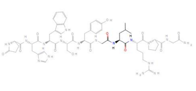 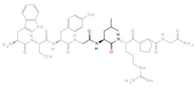	171.1134	171.1128	-3.35
MATCH	54.3	172.1082	172.1081	-0.79	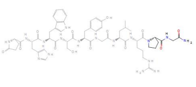 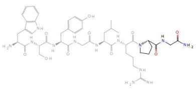	172.1082	172.1081	-1.06
MATCH	8.4	253.1660	253.1659	-0.32	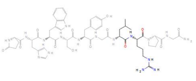 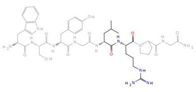	253.1660	253.1659	-0.39
MATCH	17.5	257.0927	257.0921	-2.28	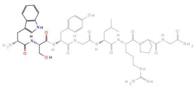	257.0924	257.0921	-1.26
MATCH	8.2	274.1189	274.1186	-0.86	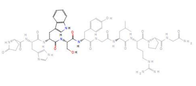 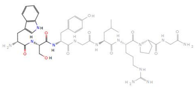	274.1191	274.1186	-1.76
					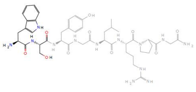	274.1191	274.1186	-1.76
MATCH	13.2	308.1250	308.1241	-3.03	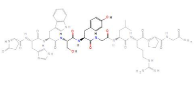 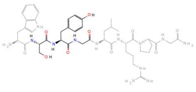	308.1243	308.1241	-0.61
MATCH	13.2	308.1250	308.1241	-3.03	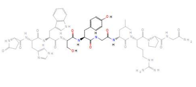 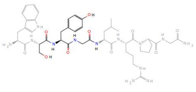	308.1243	308.1241	-0.61
MATCH	5.9	310.1882	310.1874	-2.73	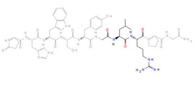 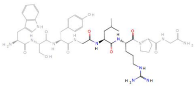	310.1882	310.1874	-2.77

Metabolite: M5 -248 RT=1.64

Type	score	sub. m/z observed	sub. m/z calculated	sub ppm		met. m/z observed	met. m/z calculated	met. ppm
MATCH	12.6	311.1840	311.1826	-4.37	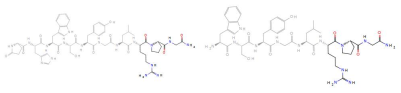	311.1836	311.1826	-3.11
MATCH	6.8	327.2143	327.2139	-1.06	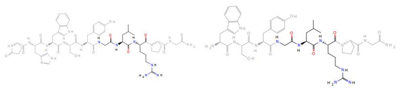	327.2147	327.2139	-2.52
MATCH	13.4	328.2098	328.2092	-1.87	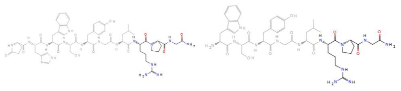	328.2098	328.2092	-1.80
MATCH	102.8	407.1831	407.1826	-1.24	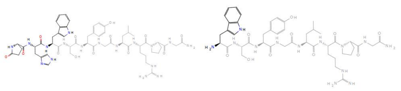	159.0920	159.0917	-1.84
MATCH	19.8	435.1786	435.1775	-2.36	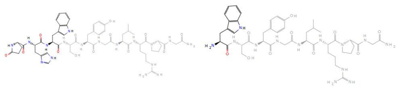	187.0865	187.0866	0.24
MATCH	6.1	441.2943	441.2932	-2.34	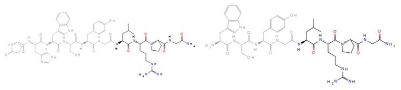	441.2950	441.2932	-3.92
MATCH	6.7	494.2120	494.2146	5.39	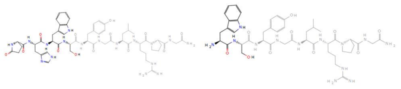	246.1244	246.1237	-2.68
MATCH	49.8	498.3158	498.3147	-2.22	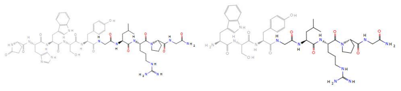	498.3163	498.3147	-3.22
MATCH	8.2	504.2001	504.1990	-2.20	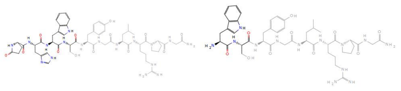	256.1090	256.1081	-3.77

Metabolite: M5 -248 RT=1.64

Type	score	sub. m/z observed	sub. m/z calculated	sub ppm			met. m/z observed	met. m/z calculated	met. ppm
MATCH	14.2	522.2112	522.2096	-3.08	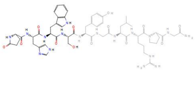	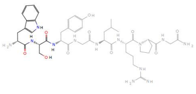	274.1191	274.1186	-1.76
						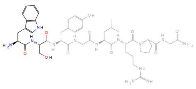	274.1191	274.1186	-1.76
MATCH	39.9	661.3794	661.3780	-2.01	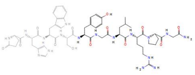	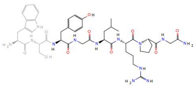	661.3796	661.3780	-2.46
MATCH	52.4	748.4114	748.4100	-1.86	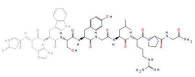	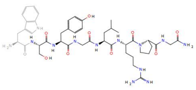	748.4127	748.4100	-3.55
MET_MATCH						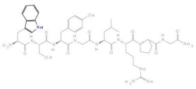	118.0654	118.0651	-1.99
MET_MATCH						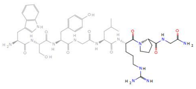	157.1085	157.1028	-36.1
MET_MATCH						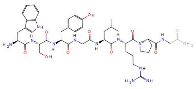	445.2376	445.2376	-0.06
MET_MATCH						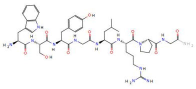	459.2344	459.2350	1.46
MET_MATCH						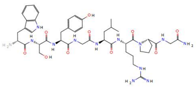	459.2344	459.2350	1.46

MS (+) FT

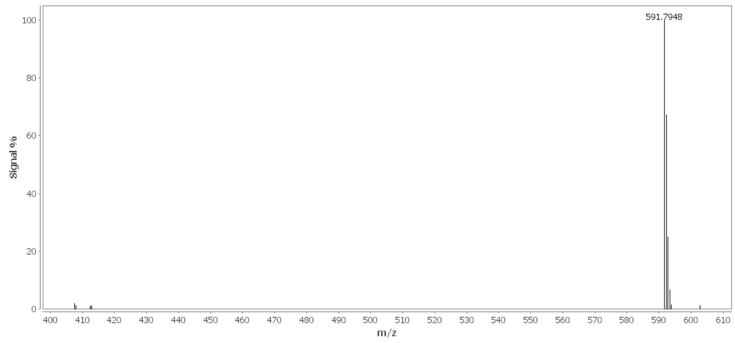

MS (+) FT

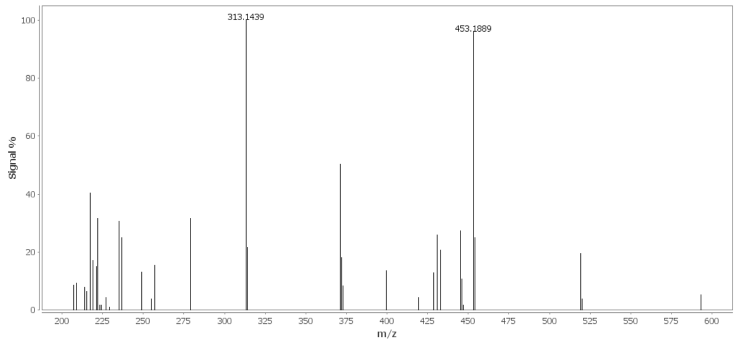

MS2 (+) FT activ = HCD:ce =

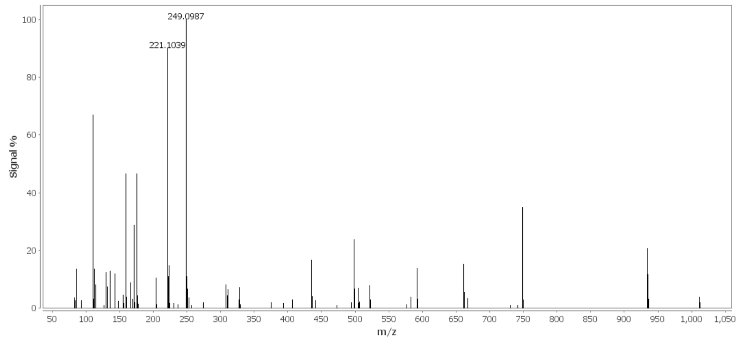

MS2 (+) FT activ = HCD:ce =

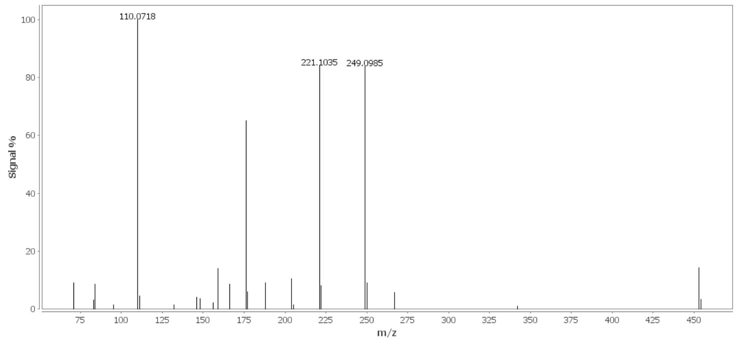

Metabolite: M1 -729 RT=0.55

Type	score	sub. m/z observed	sub. m/z calculated	sub ppm		met. m/z observed	met. m/z calculated	met. ppm
MATCH	196.0	591.7948	591.7938	-1.77	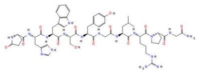 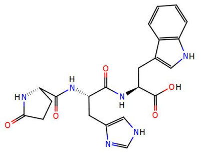	453.1889	453.1881	-1.69
MATCH	166.8	110.0719	110.0713	-5.46	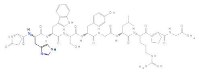 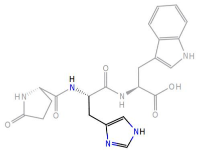	110.0718	110.0713	-4.91
MATCH	60.6	159.0920	159.0917	-1.91	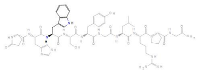 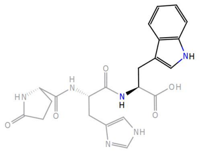	159.0919	159.0917	-1.44
MATCH	17.6	166.0615	166.0611	-2.28	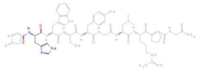 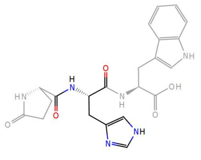	166.0612	166.0611	-0.51
MATCH	174.0	221.1039	221.1033	-2.84	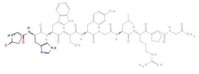 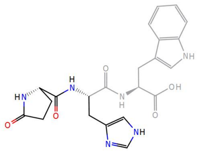	221.1035	221.1033	-1.04

Metabolite: M1 -729 RT=0.55

Type	score	sub. m/z observed	sub. m/z calculated	sub ppm		met. m/z observed	met. m/z calculated	met. ppm
MATCH	183.8	249.0987	249.0982	-1.79	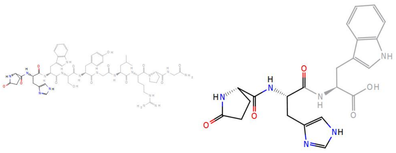	249.0984	249.0982	-0.94
MATCH	27.9	591.7958	591.7938	-3.38	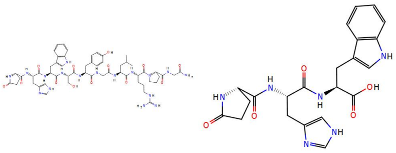	453.1894	453.1881	-2.93
MET_MATCH					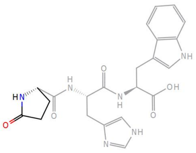	84.0451	84.0444	-8.69
MET_MATCH					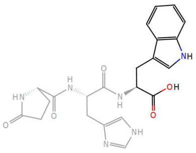	188.0709	188.0706	-1.45
MET_MATCH					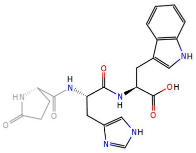	342.1582	342.1561	-6.34

MS (+) FT

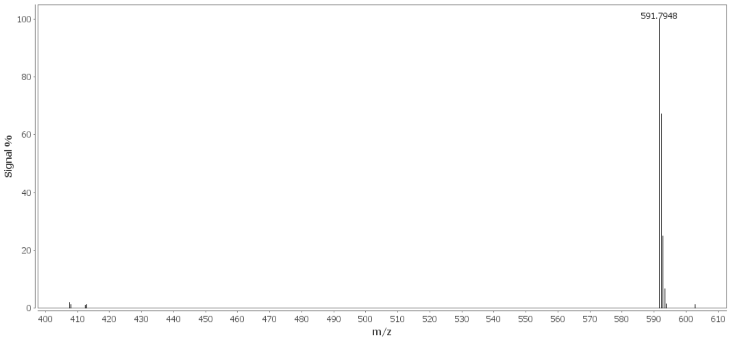

MS (+) FT

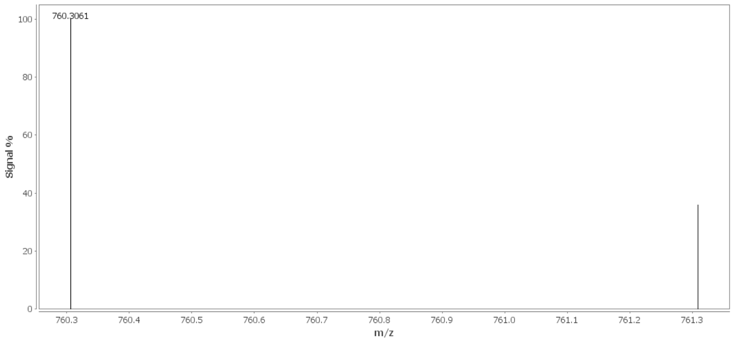

MS2 (+) FT activ = HCD:ce =

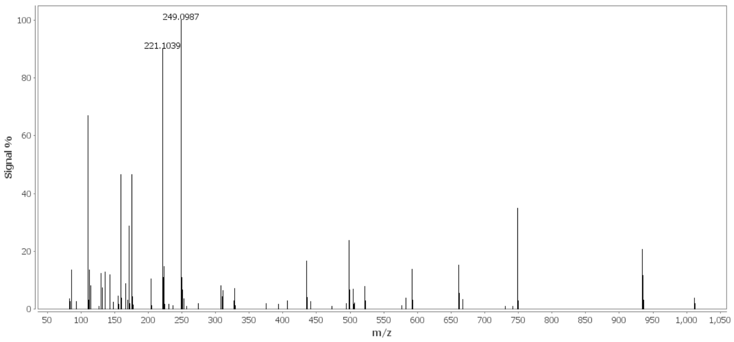

MS2 (+) FT activ = HCD:ce =

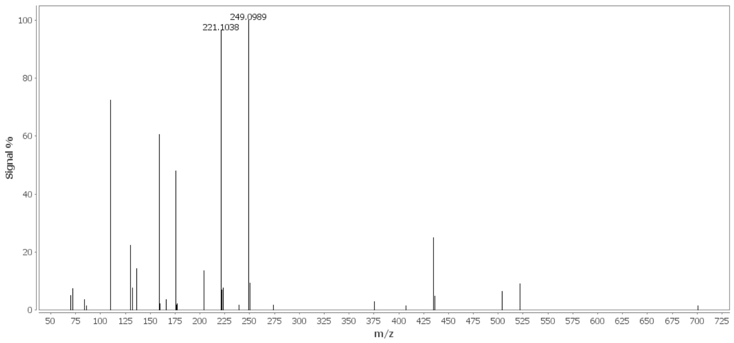

Metabolite: M3 -422 RT=1.34

Type	score	sub. m/z observed	sub. m/z calculated	sub ppm		met. m/z observed	met. m/z calculated	met. ppm
MATCH	200.0	591.7948	591.7938	-1.77	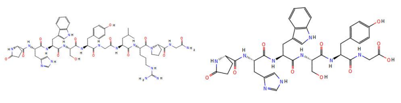	760.3061	760.3049	-1.60
MATCH	139.1	110.0719	110.0713	-5.46	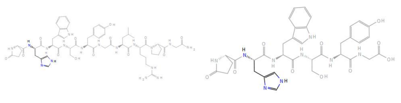	110.0719	110.0713	-5.79
MATCH	27.3	136.0761	136.0757	-3.16	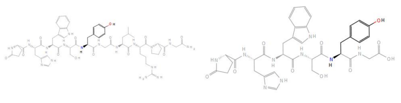	136.0762	136.0757	-3.86
MATCH	107.0	159.0920	159.0917	-1.91	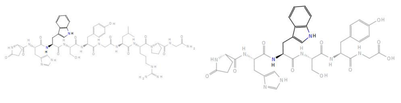	159.0920	159.0917	-2.24
MATCH	12.5	166.0615	166.0611	-2.28	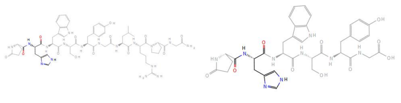	166.0615	166.0611	-2.13
MATCH	186.3	221.1039	221.1033	-2.84	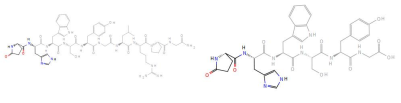	221.1038	221.1033	-2.25
MATCH	200.0	249.0987	249.0982	-1.79	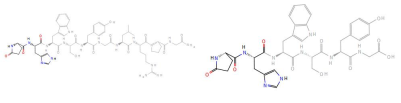	249.0989	249.0982	-2.69
MATCH	3.6	274.1189	274.1186	-0.86	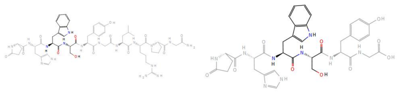	274.1198	274.1186	-4.36
MATCH	3.6	274.1189	274.1186	-0.86	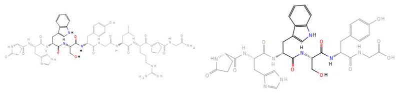	274.1198	274.1186	-4.36

Metabolite: M3 -422 RT=1.34

Type	score	sub. m/z observed	sub. m/z calculated	sub ppm		met. m/z observed	met. m/z calculated	met. ppm
MATCH	4.3	407.1831	407.1826	-1.24	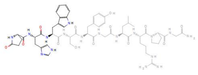 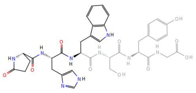	407.1823	407.1826	0.82
MATCH	41.5	435.1786	435.1775	-2.36	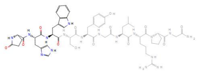 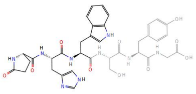	435.1773	435.1775	0.43
MATCH	13.3	504.2001	504.1990	-2.20	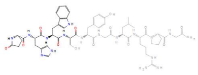 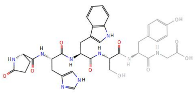	504.1974	504.1990	3.21
MATCH	16.8	522.2112	522.2096	-3.08	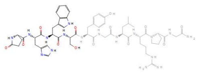 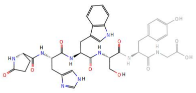	522.2114	522.2096	-3.52
MATCH	16.9	661.3794	661.3780	-2.01	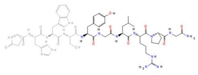 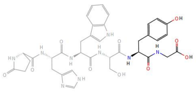	239.1038	239.1026	-4.84
MET_MATCH					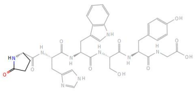	84.0452	84.0444	-9.35
