# Supplementary material for: Software-aided approach to investigate peptide structure and metabolic susceptibility of amide bonds in peptide drugs based on high resolution mass spectrometry
Source: PLoS One. 2017 Nov 1;12(11):e0186461. doi: 10.1371/journal.pone.0186461 (PMC5665424; doi:10.1371/journal.pone.0186461)
Supplement: S1 File — (ZIP) [file pone.0186461.s007.zip › SFiles/S17_File.pdf]

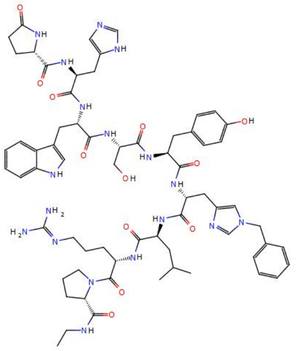

Histrelin

Property name	Property value
Time	0min, 5min, 15min, 45min, 120min
Instrument	ThermoQAPlus
Matrix	chymotrypsin
Acquisition Mode	ddMS2

Chromatograms

Time=0min

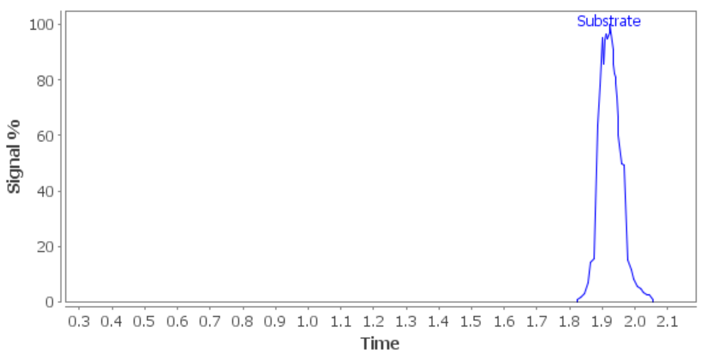

Time=5min

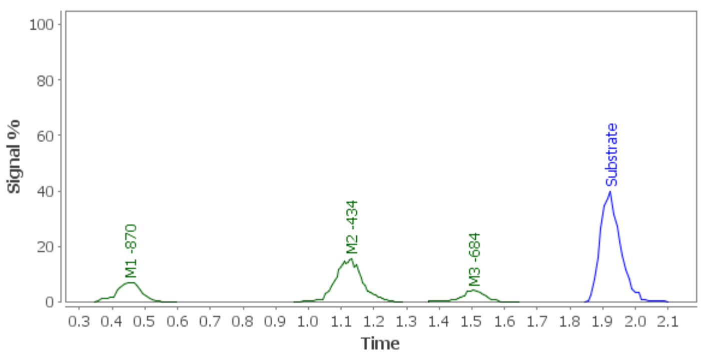

Time=15min

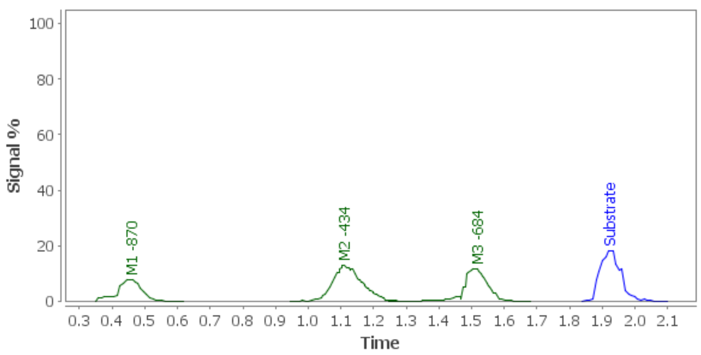

Time=45min

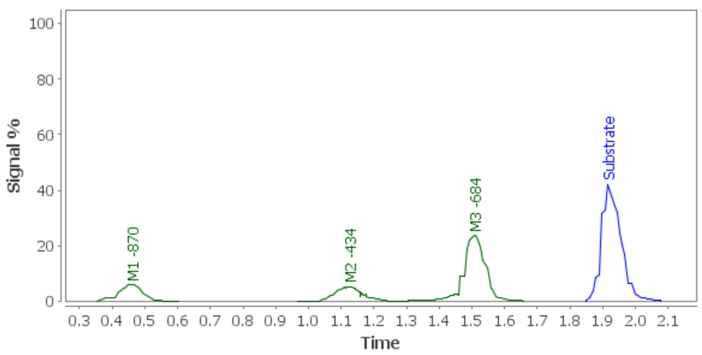

Time=120min

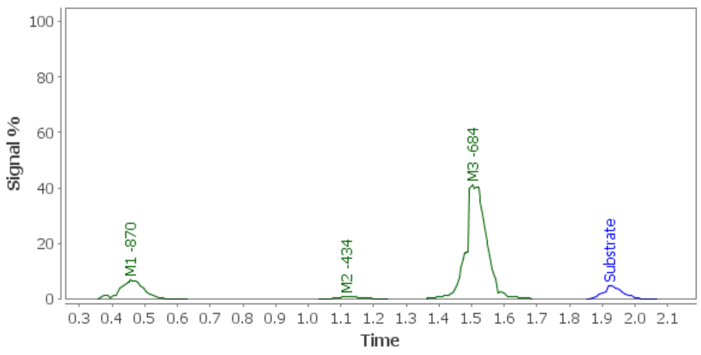

Custom Charts

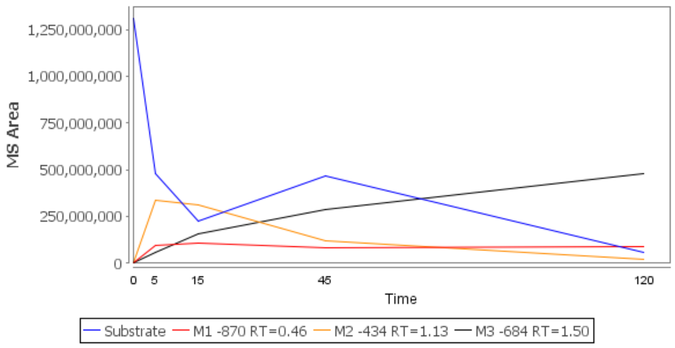

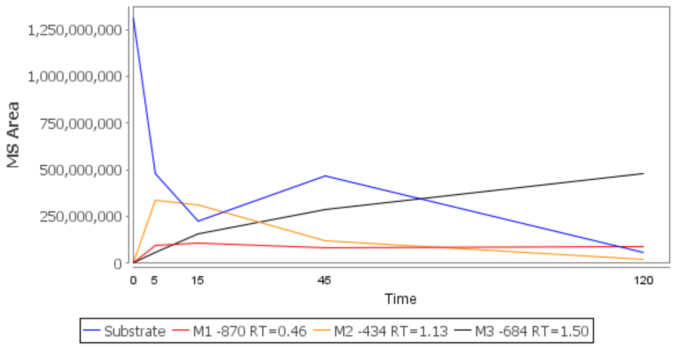

Fragmentation

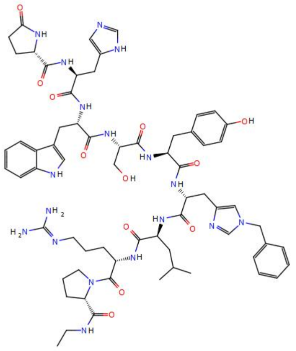

Histrelin

MS (+) FT

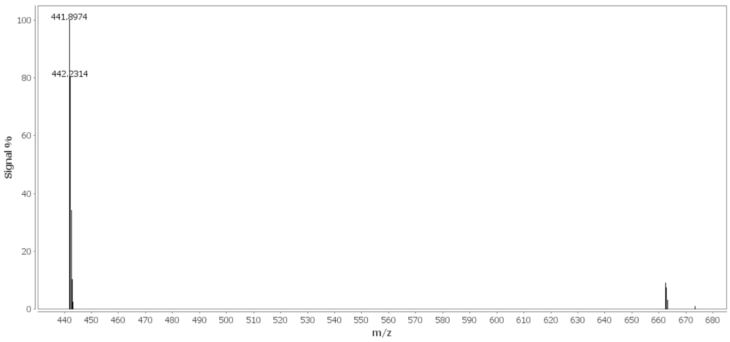

MS (+) FT

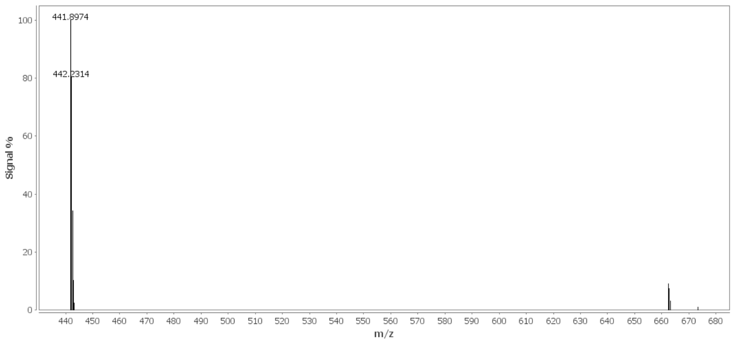

MS2 (+) FT activ = HCD:ce =

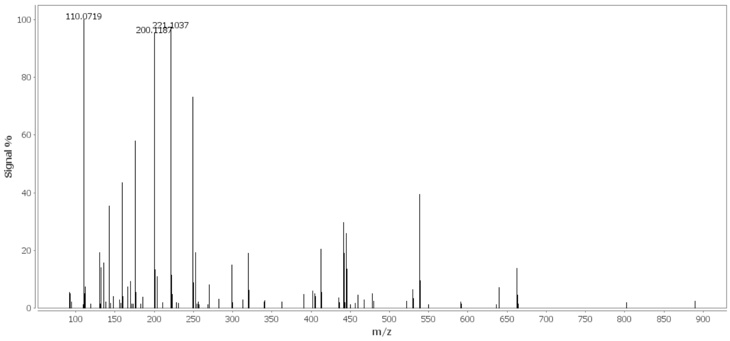

MS2 (+) FT activ = HCD:ce =

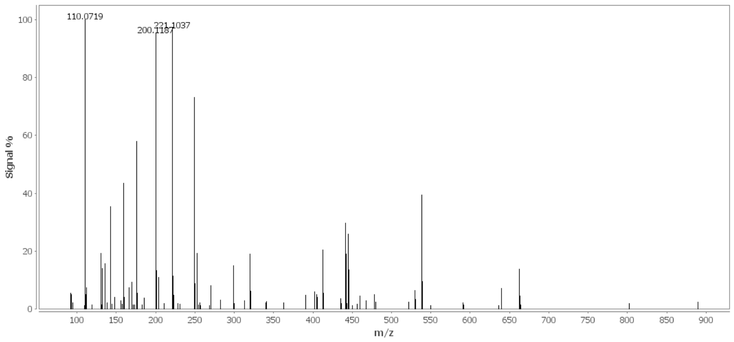

Metabolite: Substrate

Type	score	sub. m/z observed	sub. m/z calculated	sub ppm			met. m/z observed	met. m/z calculated	met. ppm
MATCH	70.6	662.3421	662.3409	-1.84	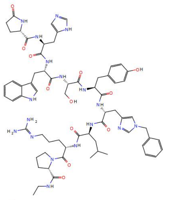	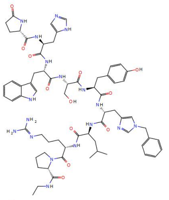	662.3421	662.3409	-1.84
MATCH	109.2	662.3420	662.3409	-1.69	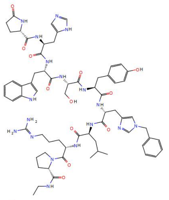	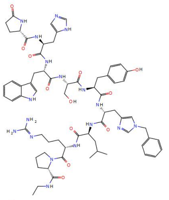	662.3420	662.3409	-1.69
MATCH	4.6	591.2868	591.2856	-1.95	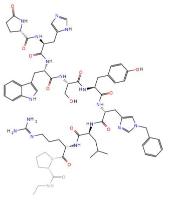	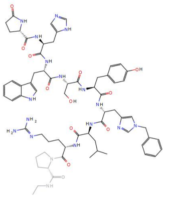	591.2868	591.2856	-1.95

Metabolite: Substrate

Type	score	sub. m/z observed	sub. m/z calculated	sub ppm			met. m/z observed	met. m/z calculated	met. ppm
MISMATCH	4.4	549.3619	549.3620	0.20	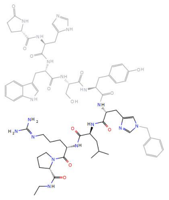	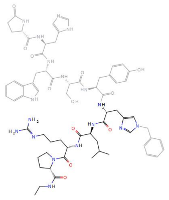	549.3619	549.3620	0.20
MISMATCH	-41.4	538.2963	538.2954	-1.55	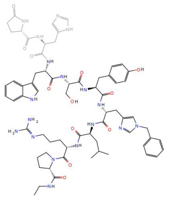	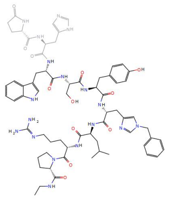	538.2963	538.2954	-1.55
MATCH	4.5	480.2728	480.2718	-2.07	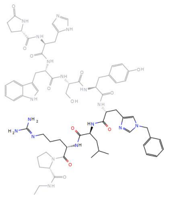	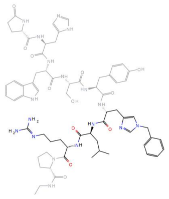	480.2728	480.2718	-2.07
MATCH	7.1	478.2083	478.2085	0.42	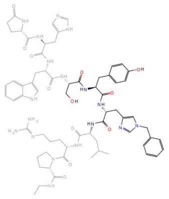	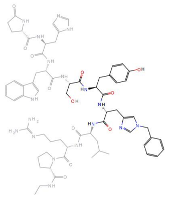	478.2083	478.2085	0.42
MATCH	200.0	441.8974	441.8964	-2.25	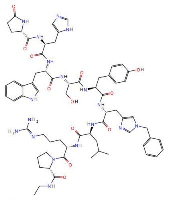	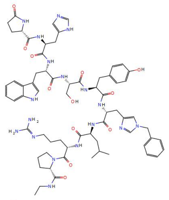	441.8974	441.8964	-2.25
MATCH	86.6	441.8970	441.8964	-1.46	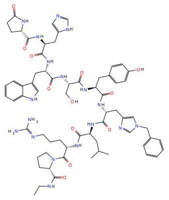	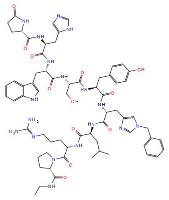	441.8970	441.8964	-1.46
MATCH	34.3	412.3035	412.3031	-1.16	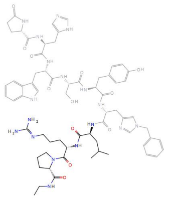	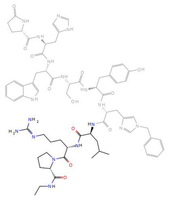	412.3035	412.3031	-1.16
MISMATCH	-6.5	404.8860	404.8857	-0.69	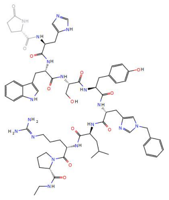	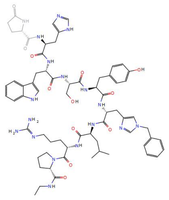	404.8860	404.8857	-0.69
MATCH	28.0	401.7401	401.7398	-0.95	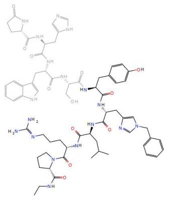	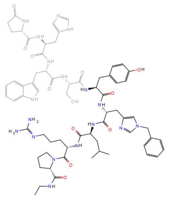	401.7401	401.7398	-0.95

Metabolite: Substrate

Type	score	sub. m/z observed	sub. m/z calculated	sub ppm			met. m/z observed	met. m/z calculated	met. ppm
MATCH	11.7	391.1760	391.1765	1.17	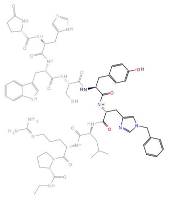	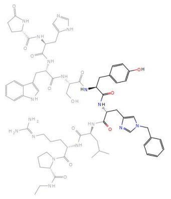	391.1760	391.1765	1.17
MATCH	11.7	391.1760	391.1765	1.17	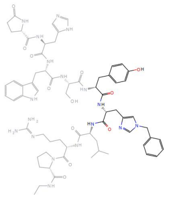	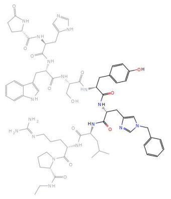	391.1760	391.1765	1.17
MATCH	3.7	363.1814	363.1816	0.47	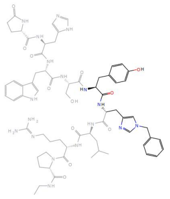	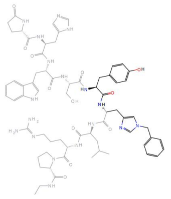	363.1814	363.1816	0.47
MATCH	40.6	320.2088	320.2081	-2.17	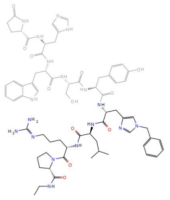	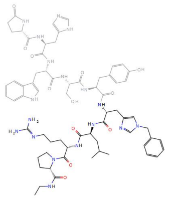	320.2088	320.2081	-2.17
MATCH	4.8	313.2028	313.2023	-1.66	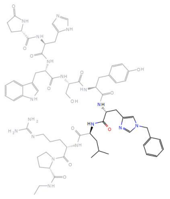	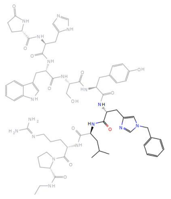	313.2028	313.2023	-1.66
MATCH	20.0	299.2201	299.2190	-3.79	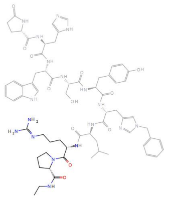	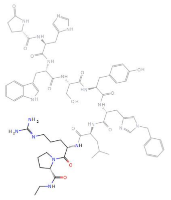	299.2201	299.2190	-3.79
MATCH	4.6	282.1918	282.1925	2.26	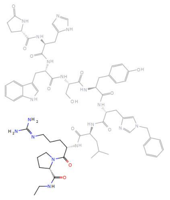	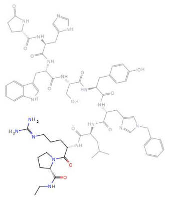	282.1918	282.1925	2.26
MATCH	12.5	270.1932	270.1925	-2.80	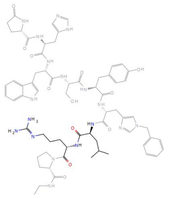	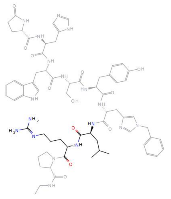	270.1932	270.1925	-2.80
MATCH	3.0	268.1830	268.1768	-23.0	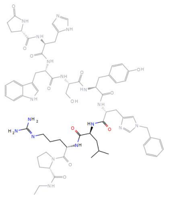	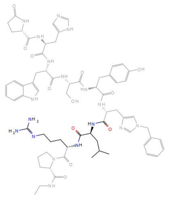	268.1830	268.1768	-23.0

Metabolite: Substrate

Type	score	sub. m/z observed	sub. m/z calculated	sub ppm			met. m/z observed	met. m/z calculated	met. ppm
MATCH	4.7	256.1089	256.1081	-3.13	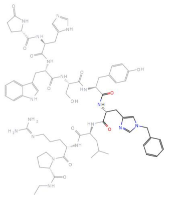	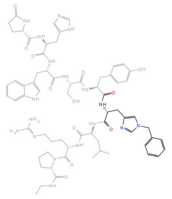	256.1089	256.1081	-3.13
MATCH	31.1	253.1662	253.1659	-1.08	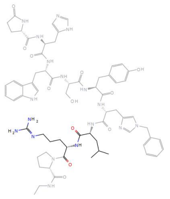	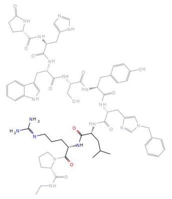	253.1662	253.1659	-1.08
MATCH	148.0	249.0986	249.0982	-1.68	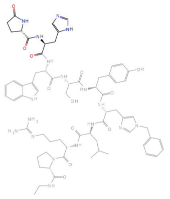	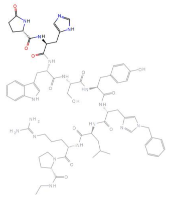	249.0986	249.0982	-1.68
MATCH	3.4	228.1135	228.1131	-1.47	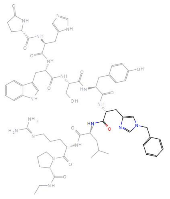	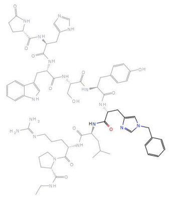	228.1135	228.1131	-1.47
MATCH	3.4	228.1135	228.1131	-1.47	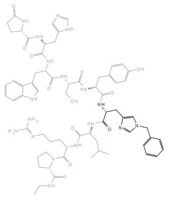	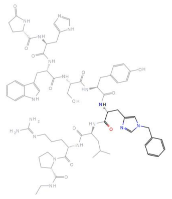	228.1135	228.1131	-1.47
MATCH	178.5	221.1037	221.1033	-1.68	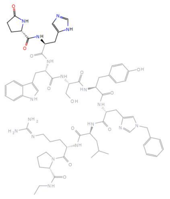	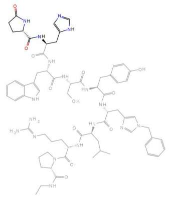	221.1037	221.1033	-1.68
MATCH	3.9	211.1446	211.1357	-42.1	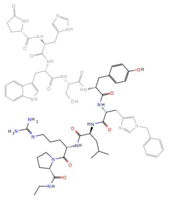	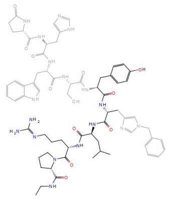	211.1446	211.1357	-42.1
MATCH	125.5	200.1187	200.1182	-2.33	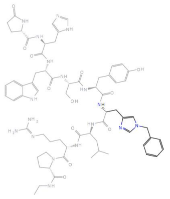	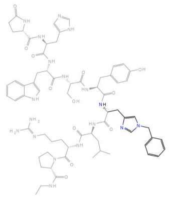	200.1187	200.1182	-2.33
MATCH	5.8	185.1050	185.1033	-9.01	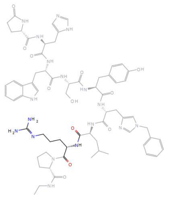	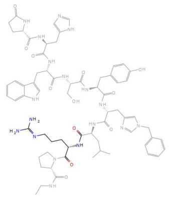	185.1050	185.1033	-9.01

Metabolite: Substrate

Type	score	sub. m/z observed	sub. m/z calculated	sub ppm			met. m/z observed	met. m/z calculated	met. ppm
MATCH	5.8	185.1050	185.1073	12.73	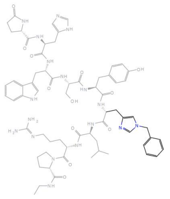	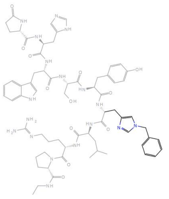	185.1050	185.1073	12.73
MATCH	5.8	185.1050	185.0997	-28.4	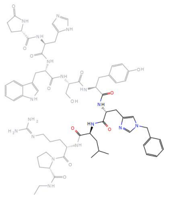	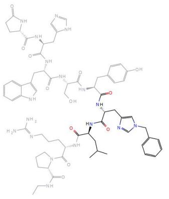	185.1050	185.0997	-28.4
MISMATCH	-2.8	171.0919	171.0897	-12.9	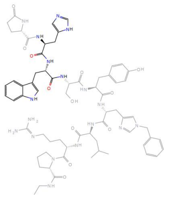	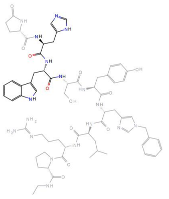	171.0919	171.0897	-12.9
MATCH	14.4	166.0613	166.0611	-0.92	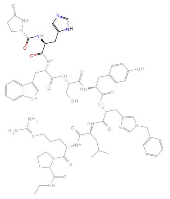	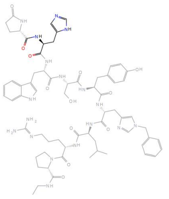	166.0613	166.0611	-0.92
MATCH	3.7	160.0760	160.0743	-10.1	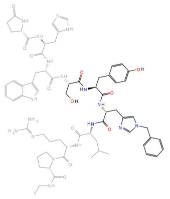	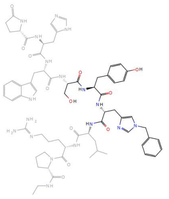	160.0760	160.0743	-10.1
MATCH	55.1	159.0920	159.0917	-1.98	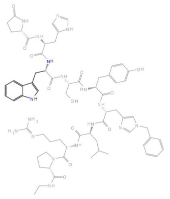	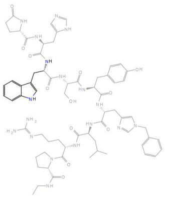	159.0920	159.0917	-1.98
MATCH	3.8	157.1086	157.1084	-1.67	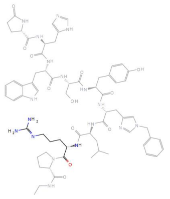	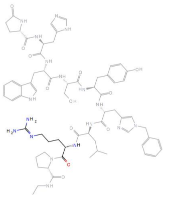	157.1086	157.1084	-1.67
MATCH	58.2	143.1182	143.1179	-2.41	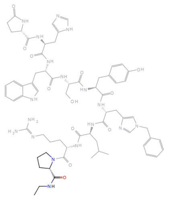	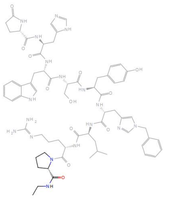	143.1182	143.1179	-2.41
MISMATCH	-3.5	138.0663	138.0662	-0.77	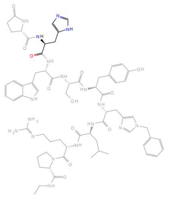	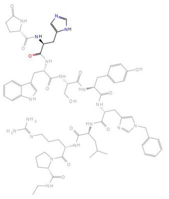	138.0663	138.0662	-0.77

Metabolite: Substrate

Type	score	sub. m/z observed	sub. m/z calculated	sub ppm			met. m/z observed	met. m/z calculated	met. ppm
MISMATCH	-3.5	138.0663	138.0662	-0.77	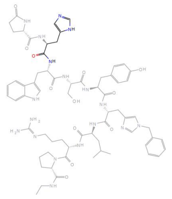	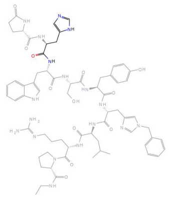	138.0663	138.0662	-0.77
MATCH	31.2	136.0760	136.0757	-2.48	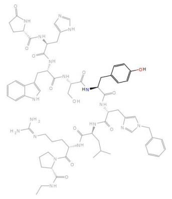	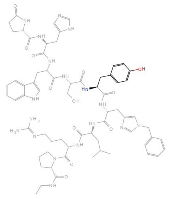	136.0760	136.0757	-2.48
MATCH	3.5	119.0498	119.0491	-5.58	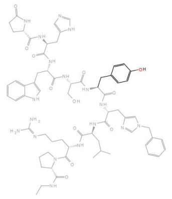	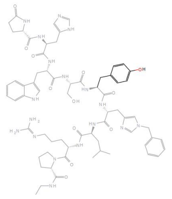	119.0498	119.0491	-5.58
MISMATCH	200.0	110.0719	110.0713	-5.41	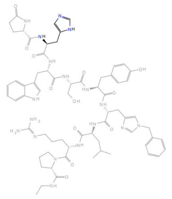	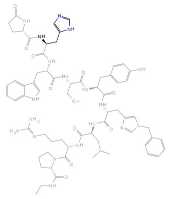	110.0719	110.0713	-5.41
MISMATCH	3.4	95.0610	95.0604	-6.07	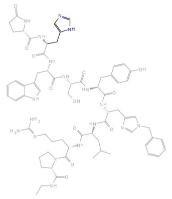	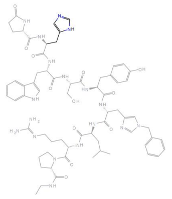	95.0610	95.0604	-6.07

MS (+) FT

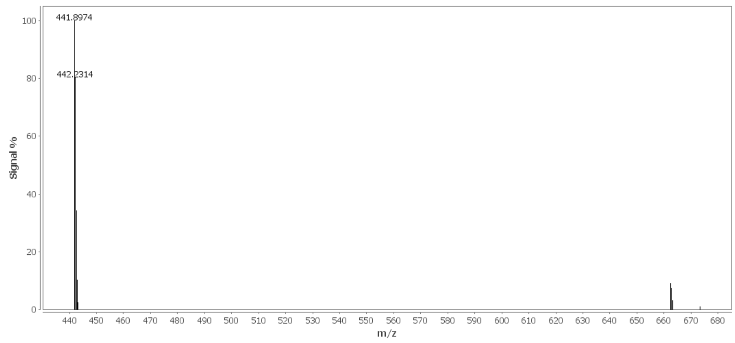

MS (+) FT

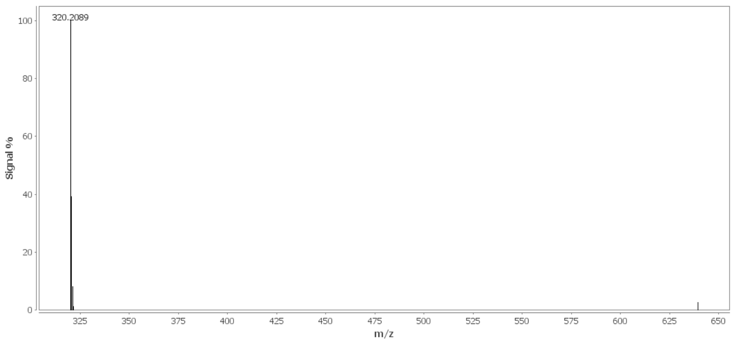

MS2 (+) FT activ = HCD:ce =

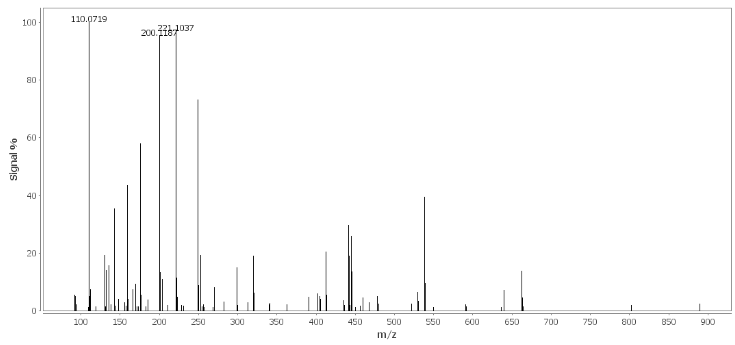

MS2 (+) FT activ = HCD:ce =

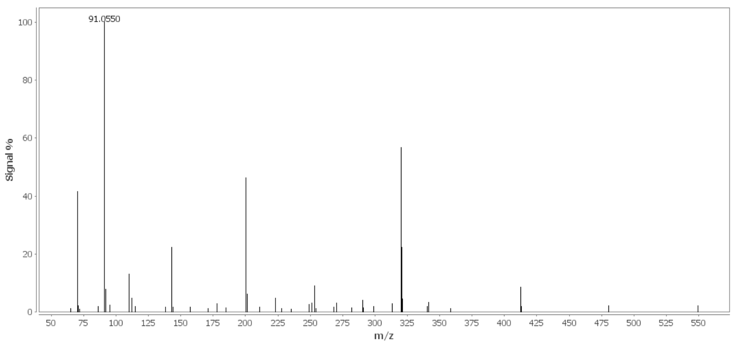

Metabolite: M3 -684 RT=1.50

Type	score	sub. m/z observed	sub. m/z calculated	sub ppm		met. m/z observed	met. m/z calculated	met. ppm
MATCH	200.0	441.8974	441.8964	-2.25	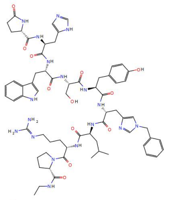 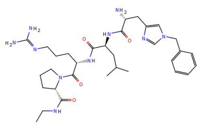	320.2089	320.2081	-2.48
MATCH	200.0	441.8974	441.8964	-2.25	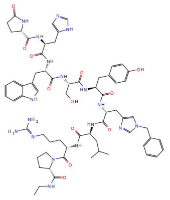 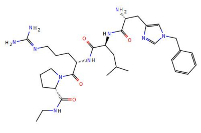	320.2089	320.2081	-2.48
MATCH	102.6	441.8974	441.8964	-2.25	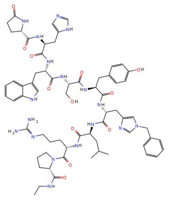 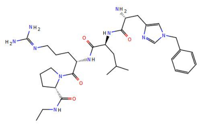	639.4094	639.4089	-0.66
MATCH	102.6	441.8974	441.8964	-2.25	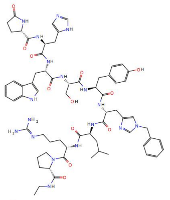 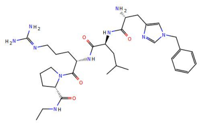	639.4094	639.4089	-0.66
MATCH	109.2	662.3420	662.3409	-1.69	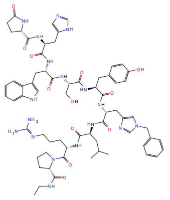 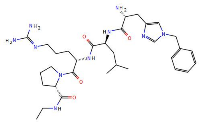	320.2089	320.2081	-2.48
MATCH	109.2	662.3420	662.3409	-1.69	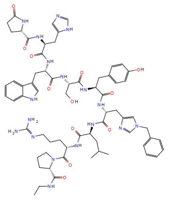 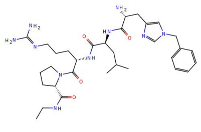	320.2089	320.2081	-2.48
MATCH	11.7	662.3420	662.3409	-1.69	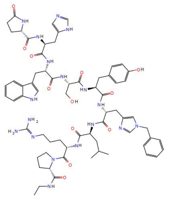 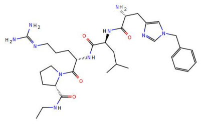	639.4094	639.4089	-0.66
MATCH	11.7	662.3420	662.3409	-1.69	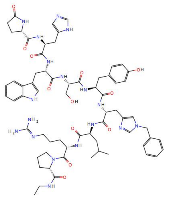 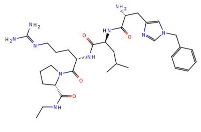	639.4094	639.4089	-0.66
MATCH	57.7	143.1182	143.1179	-2.41	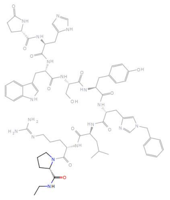 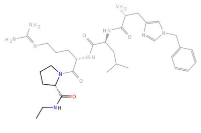	143.1182	143.1179	-2.03

Metabolite: M3 -684 RT=1.50

Type	score	sub. m/z observed	sub. m/z calculated	sub ppm			met. m/z observed	met. m/z calculated	met. ppm
MATCH	3.4	157.1086	157.1084	-1.67	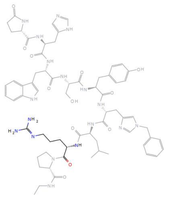	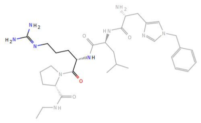	157.1087	157.1084	-2.04
MATCH	5.1	185.1050	185.1033	-9.01	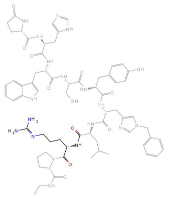	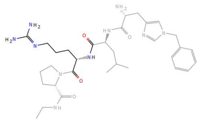	185.1048	185.1033	-8.24
MATCH	5.1	185.1050	185.1073	12.73	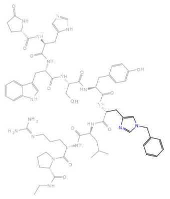	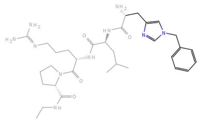	185.1048	185.1073	13.49
MATCH	3.2	228.1135	228.1131	-1.47	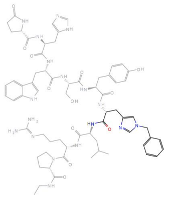	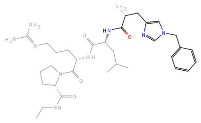	228.1140	228.1131	-3.69
MATCH	28.3	253.1662	253.1659	-1.08	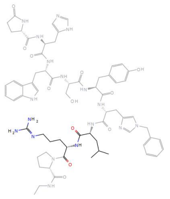	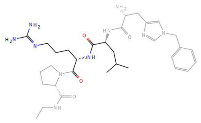	253.1663	253.1659	-1.47
MATCH	3.0	268.1830	268.1768	-23.0	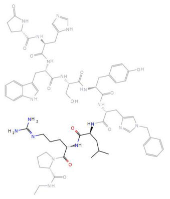	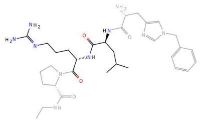	268.1816	268.1768	-17.9
MATCH	3.0	268.1830	268.1768	-23.0	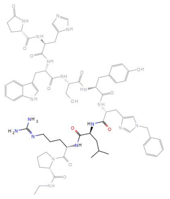	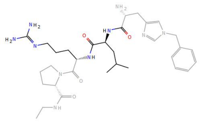	268.1816	268.1768	-17.9
MATCH	11.1	270.1932	270.1925	-2.80	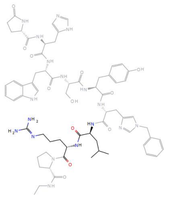	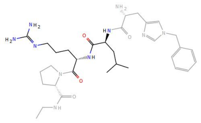	270.1926	270.1925	-0.56
MATCH	4.6	282.1918	282.1925	2.26	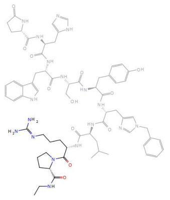	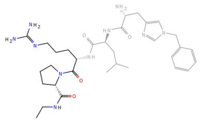	282.1928	282.1925	-1.26

Metabolite: M3 -684 RT=1.50

Type	score	sub. m/z observed	sub. m/z calculated	sub ppm			met. m/z observed	met. m/z calculated	met. ppm
MATCH	16.8	299.2201	299.2190	-3.79	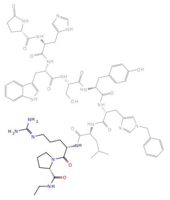	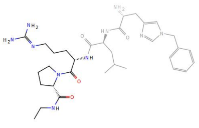	299.2213	299.2190	-7.54
MATCH	28.9	412.3035	412.3031	-1.16	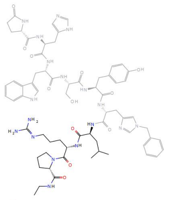	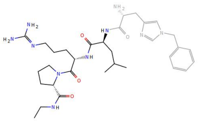	412.3037	412.3031	-1.49
MATCH	86.6	441.8970	441.8964	-1.46	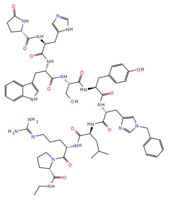	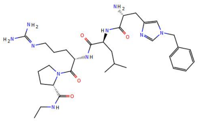	320.2085	320.2081	-1.33
						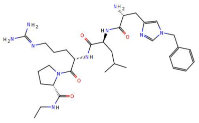	320.2085	320.2081	-1.33
MATCH	4.5	480.2728	480.2718	-2.07	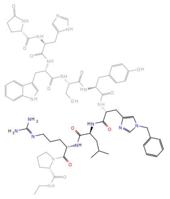	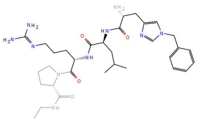	480.2740	480.2718	-4.63
MATCH	4.6	591.2868	591.2856	-1.95	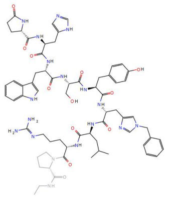	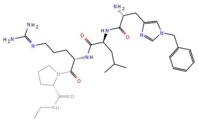	249.1530	249.1528	-0.63
MATCH	70.6	662.3421	662.3409	-1.84	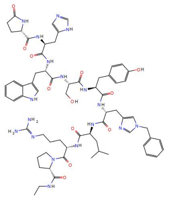	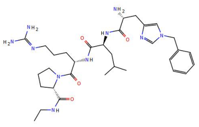	320.2085	320.2081	-1.33
						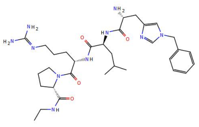	320.2085	320.2081	-1.33
MISMATCH	-4.5	95.0610	95.0604	-6.07	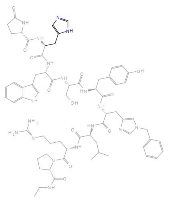		95.0611	95.0611	0.00

Metabolite: M3 -684 RT=1.50

Type	score	sub. m/z observed	sub. m/z calculated	sub ppm		met. m/z observed	met. m/z calculated	met. ppm
MISMATCH	-113.0	110.0719	110.0713	-5.41	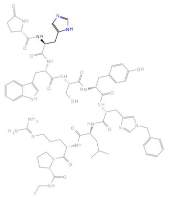	110.0719	110.0719	0.00
MISMATCH	-3.8	138.0663	138.0662	-0.77	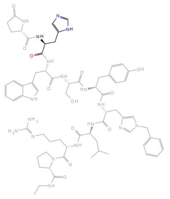	138.0667	138.0667	0.00
MISMATCH	-2.8	171.0919	171.0897	-12.9	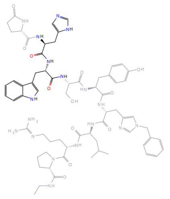	171.0918	171.0918	0.00
MET_MATCH					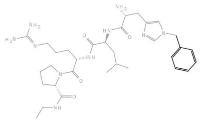	91.0550	91.0542	-8.20
MET_MATCH					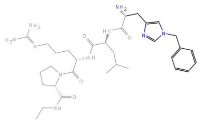	200.1185	200.1182	-1.58
MET_MATCH					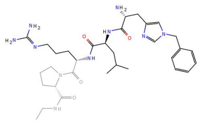	235.1557	235.1553	-1.57
MET_MATCH					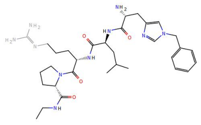	290.6841	290.6839	-0.58
MET_MATCH					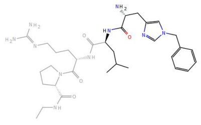	313.2024	313.2023	-0.51
MET_MATCH					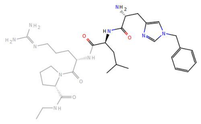	341.1965	341.1972	2.07

Metabolite: M3 -684 RT=1.50

Type	score	sub. m/z observed	sub. m/z calculated	sub ppm		met. m/z observed	met. m/z calculated	met. ppm
MET_MATCH					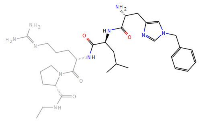	358.2232	358.2238	1.55
MET_MATCH					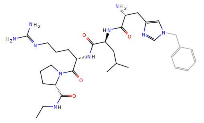	549.3620	549.3620	-0.07

MS (+) FT

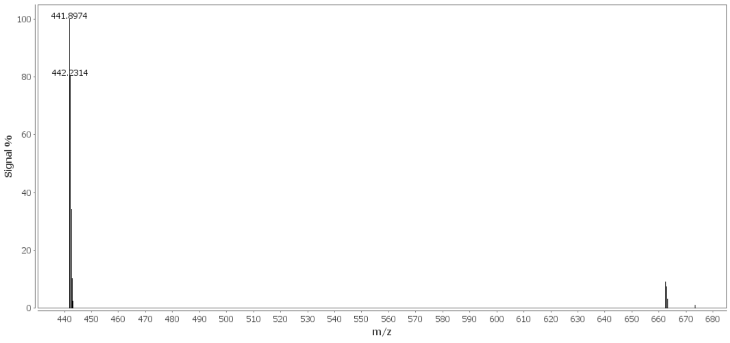

MS (+) FT

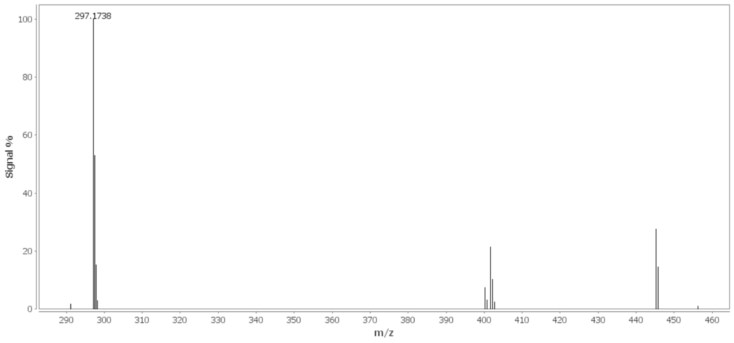

MS2 (+) FT activ = HCD:ce =

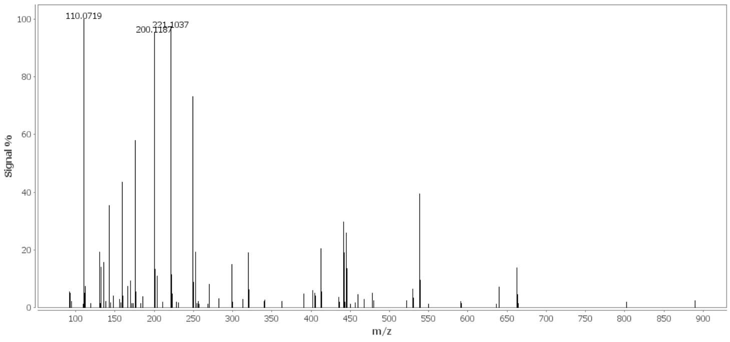

MS2 (+) FT activ = HCD:ce =

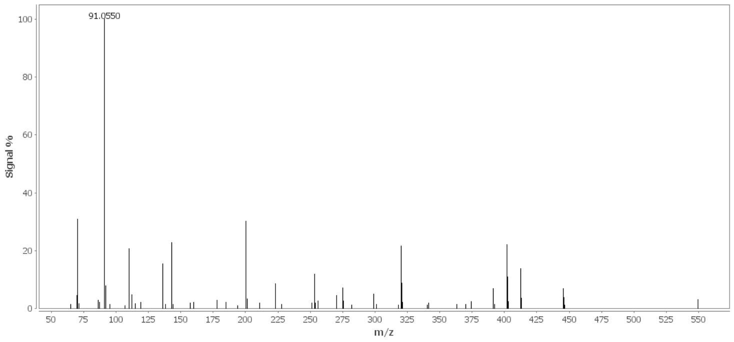

Metabolite: M2 -434 RT=1.13

Type	score	sub. m/z observed	sub. m/z calculated	sub ppm			met. m/z observed	met. m/z calculated	met. ppm
MATCH	200.0	441.8974	441.8964	-2.25	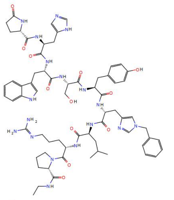	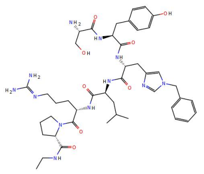	297.1738	297.1729	-2.88
MATCH	200.0	441.8974	441.8964	-2.25	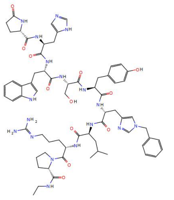	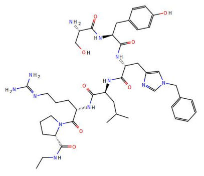	297.1738	297.1729	-2.88

Metabolite: M2 -434 RT=1.13

Type	score	sub. m/z observed	sub. m/z calculated	sub ppm		met. m/z observed	met. m/z calculated	met. ppm
MATCH	127.5	441.8974	441.8964	-2.25	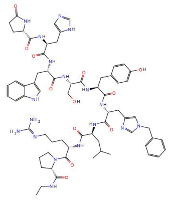 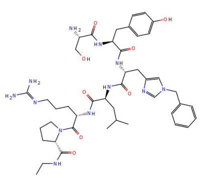	445.2568	445.2558	-2.30
MATCH	127.5	441.8974	441.8964	-2.25	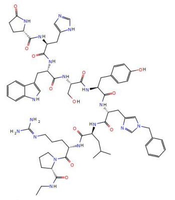 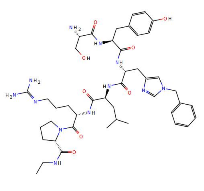	445.2568	445.2558	-2.30
MATCH	109.2	662.3420	662.3409	-1.69	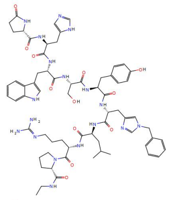 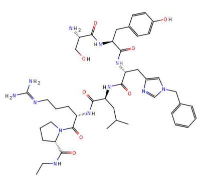	297.1738	297.1729	-2.88
MATCH	109.2	662.3420	662.3409	-1.69	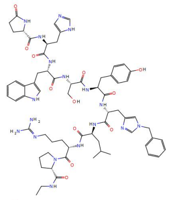 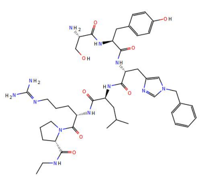	297.1738	297.1729	-2.88
MATCH	36.6	662.3420	662.3409	-1.69	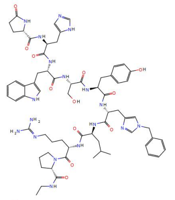 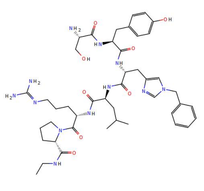	445.2568	445.2558	-2.30
MATCH	36.6	662.3420	662.3409	-1.69	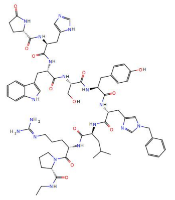 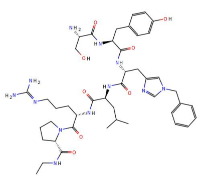	445.2568	445.2558	-2.30
MATCH	3.5	119.0498	119.0491	-5.58	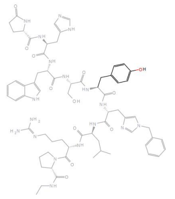 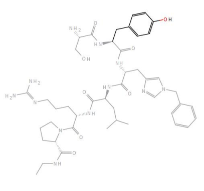	119.0496	119.0491	-3.65
MATCH	31.2	136.0760	136.0757	-2.48	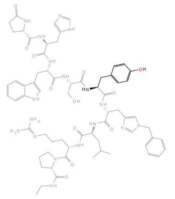 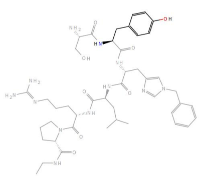	136.0760	136.0757	-2.18
MATCH	58.2	143.1182	143.1179	-2.41	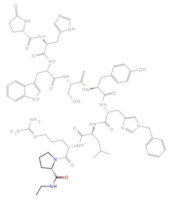 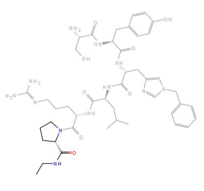	143.1182	143.1179	-2.12

Metabolite: M2 -434 RT=1.13

Type	score	sub. m/z observed	sub. m/z calculated	sub ppm			met. m/z observed	met. m/z calculated	met. ppm
MATCH	3.8	157.1086	157.1084	-1.67	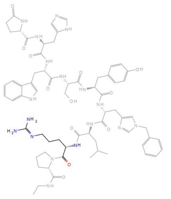	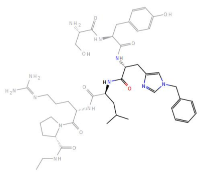	157.1088	157.1048	-25.3
						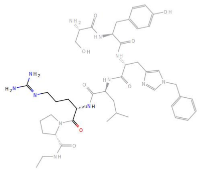	157.1088	157.1084	-2.36
MATCH	3.7	160.0760	160.0743	-10.1	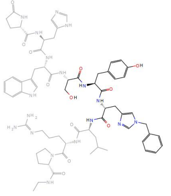	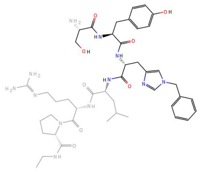	160.0761	160.0743	-10.6
						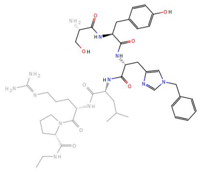	160.0761	160.0743	-10.6
MATCH	5.8	185.1050	185.0997	-28.4	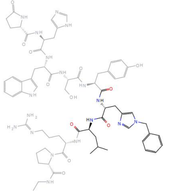	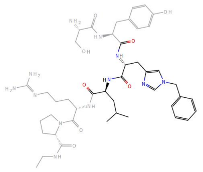	185.1047	185.0997	-27.2
MATCH	5.8	185.1050	185.1033	-9.01	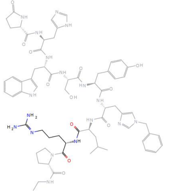	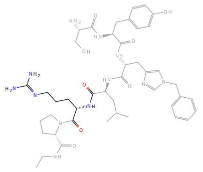	185.1047	185.1033	-7.76
MATCH	5.8	185.1050	185.1073	12.73	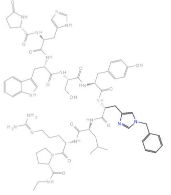	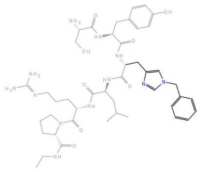	185.1047	185.1073	13.97
MATCH	125.5	200.1187	200.1182	-2.33	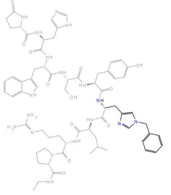	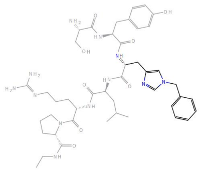	200.1185	200.1182	-1.59
MATCH	3.9	211.1446	211.1357	-42.1	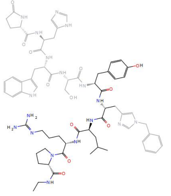	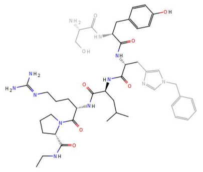	211.1441	211.1357	-39.4

Metabolite: M2 -434 RT=1.13

Type	score	sub. m/z observed	sub. m/z calculated	sub ppm			met. m/z observed	met. m/z calculated	met. ppm
MATCH	3.4	228.1135	228.1131	-1.47	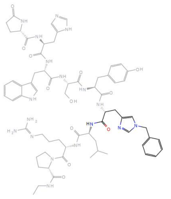	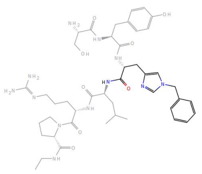	228.1133	228.1131	-0.69
MATCH	3.4	228.1135	228.1131	-1.47	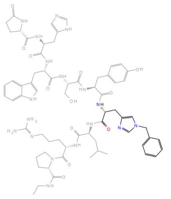	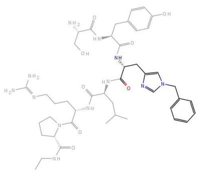	228.1133	228.1131	-0.69
MATCH	31.1	253.1662	253.1659	-1.08	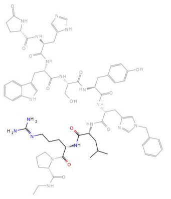	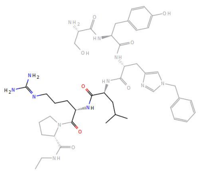	253.1660	253.1659	-0.49
MATCH	4.7	256.1089	256.1081	-3.13	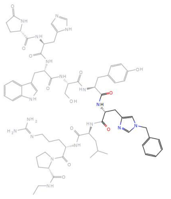	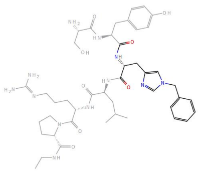	256.1083	256.1081	-0.88
MATCH	12.5	270.1932	270.1925	-2.80	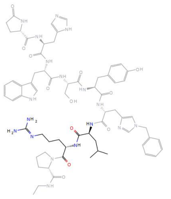	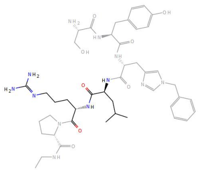	270.1928	270.1925	-1.44
MATCH	4.5	282.1918	282.1925	2.26	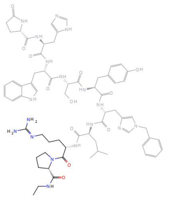	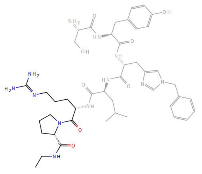	282.1930	282.1925	-1.86
MATCH	20.0	299.2201	299.2190	-3.79	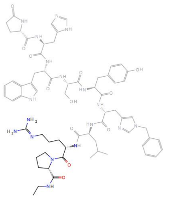	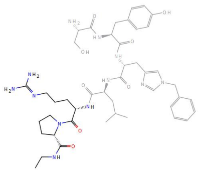	299.2194	299.2190	-1.20
MATCH	4.8	313.2028	313.2023	-1.66	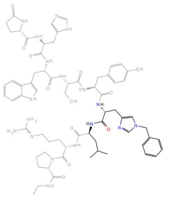	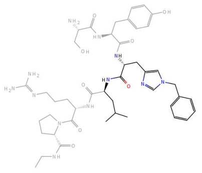	157.1088	157.1048	-25.3
						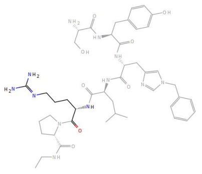	157.1088	157.1084	-2.36

Metabolite: M2 -434 RT=1.13

Type	score	sub. m/z observed	sub. m/z calculated	sub ppm			met. m/z observed	met. m/z calculated	met. ppm
MATCH	40.6	320.2088	320.2081	-2.17	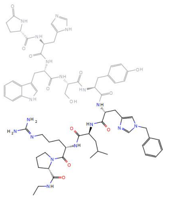	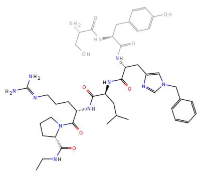	320.2085	320.2081	-1.20
MATCH	3.7	363.1814	363.1816	0.47	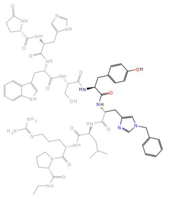	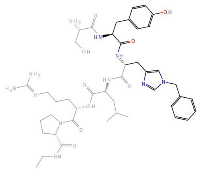	363.1813	363.1816	0.74
MATCH	11.7	391.1760	391.1765	1.17	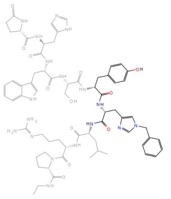	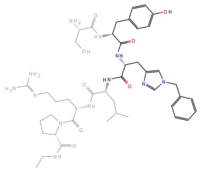	391.1773	391.1765	-2.02
MATCH	11.7	391.1760	391.1765	1.17	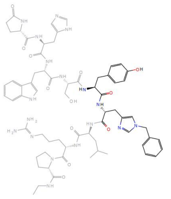	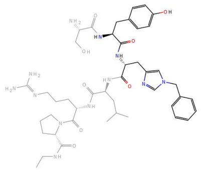	391.1773	391.1765	-2.02
MATCH	28.0	401.7401	401.7398	-0.95	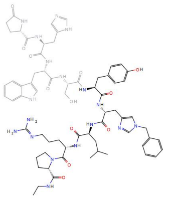	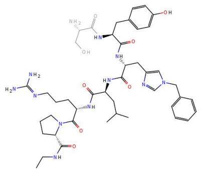	401.7402	401.7398	-1.02
MATCH	34.3	412.3035	412.3031	-1.16	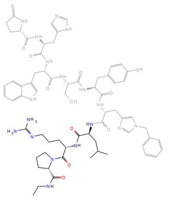	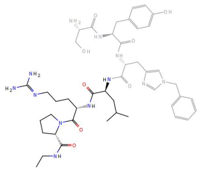	412.3035	412.3031	-1.11
MATCH	36.8	441.8970	441.8964	-1.46	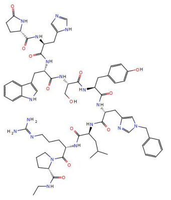	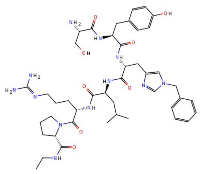	445.2563	445.2558	-1.23
						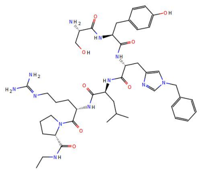	445.2563	445.2558	-1.23
MATCH	7.1	478.2083	478.2085	0.42	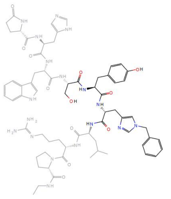	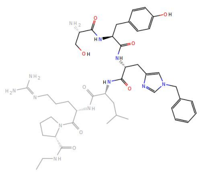	160.0761	160.0743	-10.6

Metabolite: M2 -434 RT=1.13

Type	score	sub. m/z observed	sub. m/z calculated	sub ppm			met. m/z observed	met. m/z calculated	met. ppm
						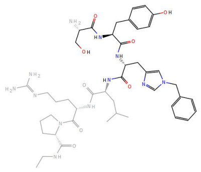	160.0761	160.0743	-10.6
MATCH	4.4	549.3619	549.3620	0.20	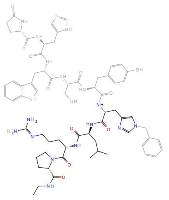	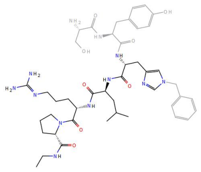	549.3623	549.3620	-0.52
MATCH	20.8	662.3421	662.3409	-1.84	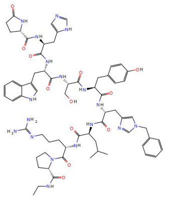	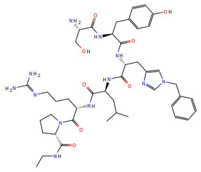	445.2563	445.2558	-1.23
						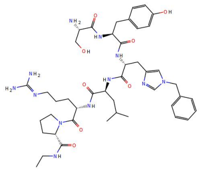	445.2563	445.2558	-1.23
MISMATCH	-3.4	95.0610	95.0604	-6.07	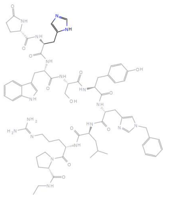		95.0611	95.0611	0.00
MISMATCH	-120.6	110.0719	110.0713	-5.41	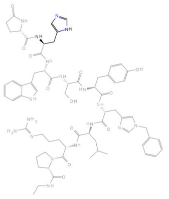		110.0718	110.0718	0.00
MISMATCH	-3.5	138.0663	138.0662	-0.77	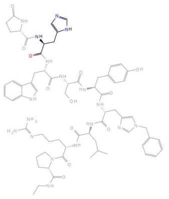		138.0666	138.0666	0.00
MISMATCH	-8.4	549.3619	549.3620	0.20	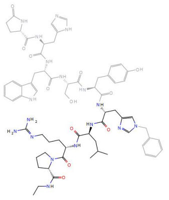		275.1846	275.1846	0.00
MET_MATCH						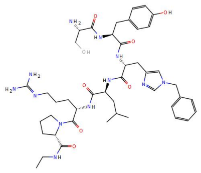	291.1700	291.1694	-1.89

Metabolite: M2 -434 RT=1.13

Type	score	sub. m/z observed	sub. m/z calculated	sub ppm		met. m/z observed	met. m/z calculated	met. ppm
MET_MATCH					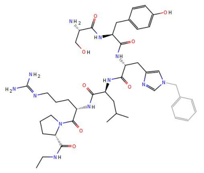	400.2335	400.2323	-2.91
MET_MATCH					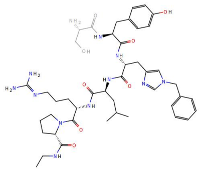	400.7347	400.7319	-6.96
MET_MATCH					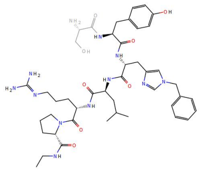	401.7407	401.7398	-2.33
MET_MATCH					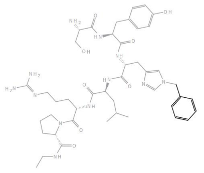	91.0550	91.0542	-7.94
MET_MATCH					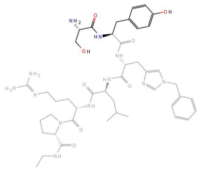	223.1081	223.1077	-1.55

MS (+) FT

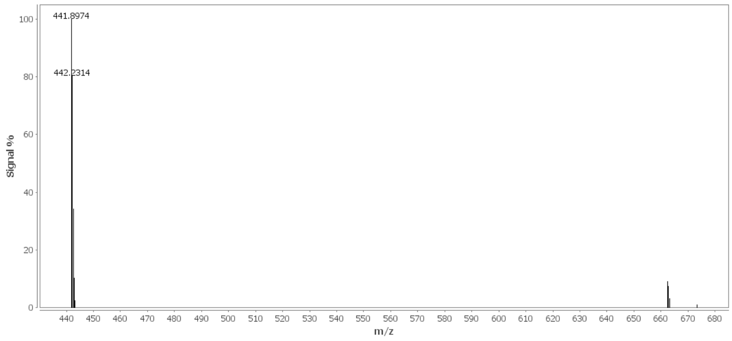

MS (+) FT

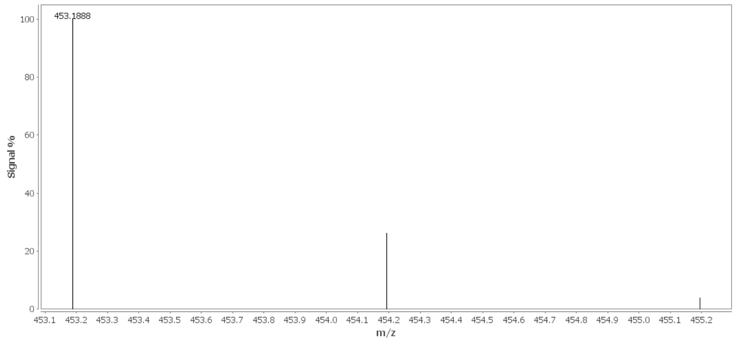

MS2 (+) FT activ = HCD:ce =

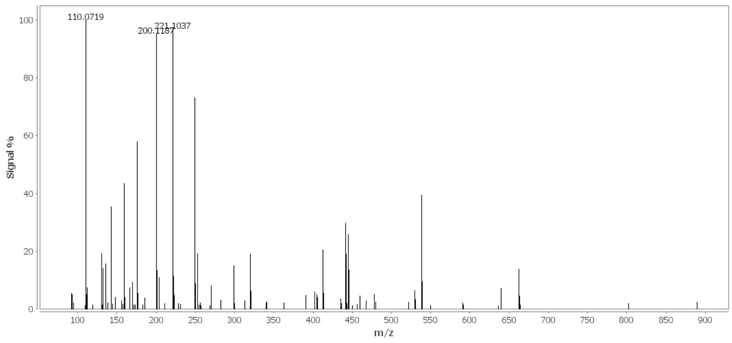

MS2 (+) FT activ = HCD:ce =

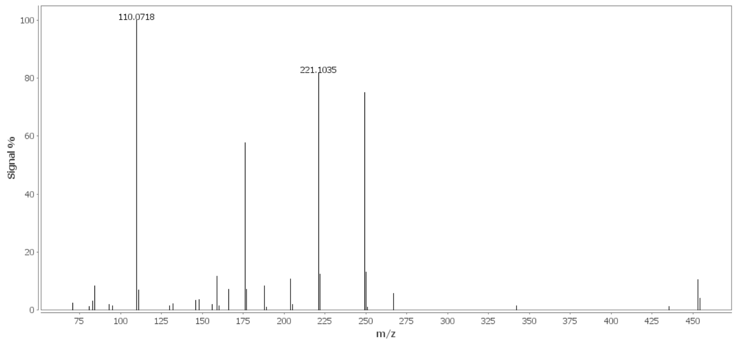

Metabolite: M1 -870 RT=0.46

Type	score	sub. m/z observed	sub. m/z calculated	sub ppm			met. m/z observed	met. m/z calculated	met. ppm
MATCH	200.0	441.8974	441.8964	-2.25	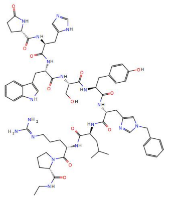	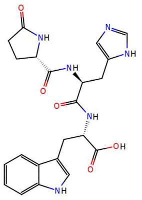	453.1888	453.1881	-1.62
						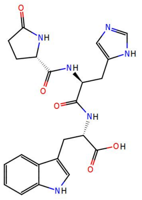	453.1888	453.1881	-1.62
MATCH	109.2	662.3420	662.3409	-1.69	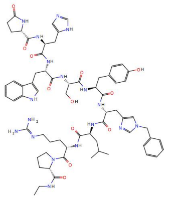	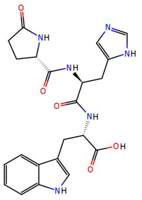	453.1888	453.1881	-1.62
						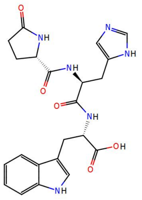	453.1888	453.1881	-1.62
MATCH	3.4	95.0610	95.0604	-6.07	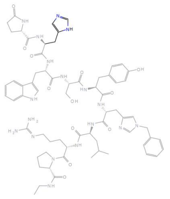	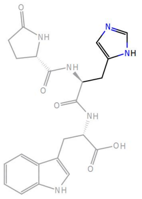	95.0611	95.0604	-7.47
MATCH	200.0	110.0719	110.0713	-5.41	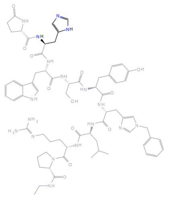	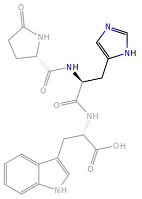	110.0718	110.0713	-4.62
MATCH	55.1	159.0920	159.0917	-1.98	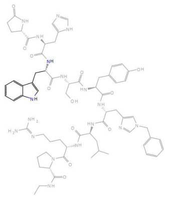	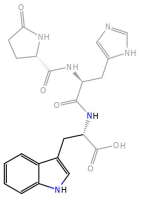	159.0918	159.0917	-1.06
MATCH	14.4	166.0613	166.0611	-0.92	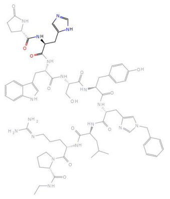	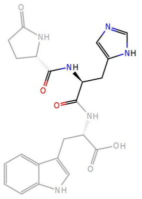	166.0613	166.0611	-0.90
MATCH	178.5	221.1037	221.1033	-1.68	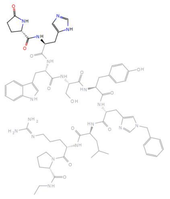	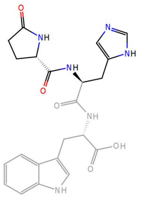	221.1035	221.1033	-0.72

Metabolite: M1 -870 RT=0.46

Type	score	sub. m/z observed	sub. m/z calculated	sub ppm			met. m/z observed	met. m/z calculated	met. ppm
MATCH	148.0	249.0986	249.0982	-1.68	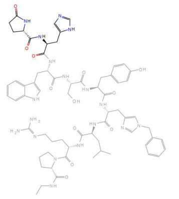	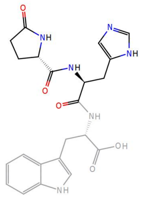	249.0984	249.0982	-0.81
MATCH	40.2	441.8970	441.8964	-1.46	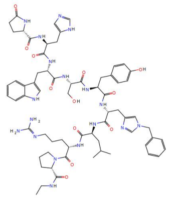	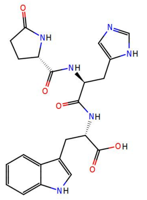	453.1881	453.1881	-0.10
						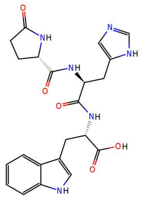	453.1881	453.1881	-0.10
MATCH	24.2	662.3421	662.3409	-1.84	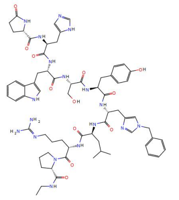	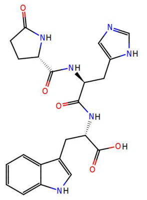	453.1881	453.1881	-0.10
						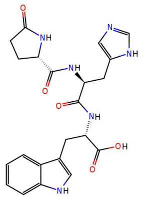	453.1881	453.1881	-0.10
MISMATCH	-6.5	404.8860	404.8857	-0.69	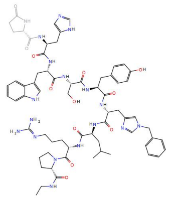		342.1559	342.1559	0.00
MISMATCH	-41.4	538.2963	538.2954	-1.55	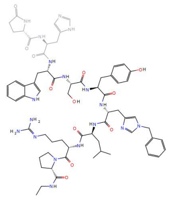		205.0970	205.0970	0.00
MET_MATCH						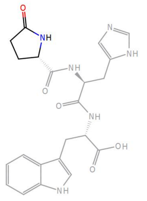	84.0452	84.0444	-9.40
MET_MATCH						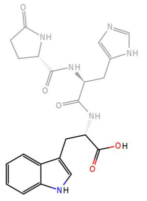	188.0707	188.0706	-0.72

Metabolite: M1 -870 RT=0.46

Type	score	sub. m/z observed	sub. m/z calculated	sub ppm		met. m/z observed	met. m/z calculated	met. ppm
MET_MATCH					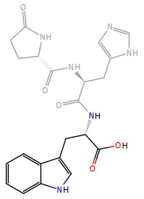	205.0970	205.0972	0.76
MET_MATCH					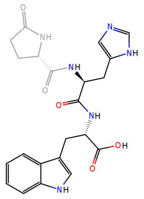	342.1559	342.1561	0.38
MET_MATCH					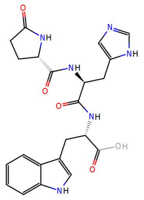	435.1764	435.1775	2.70
