# Supplementary material for: Software-aided approach to investigate peptide structure and metabolic susceptibility of amide bonds in peptide drugs based on high resolution mass spectrometry
Source: PLoS One. 2017 Nov 1;12(11):e0186461. doi: 10.1371/journal.pone.0186461 (PMC5665424; doi:10.1371/journal.pone.0186461)
Supplement: S1 File — (ZIP) [file pone.0186461.s007.zip › SFiles/S18_File.pdf]

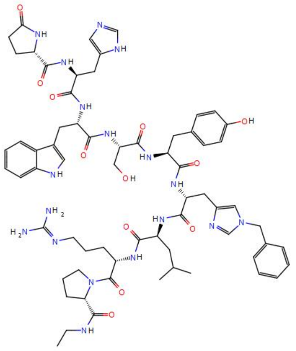

Histrelin

Property name	Property value
Time	0min, 5min, 15min, 45min, 120min
Instrument	ThermoQAPlus
Matrix	trypsin
Acquisition Mode	ddMS2

Chromatograms

Time=0min

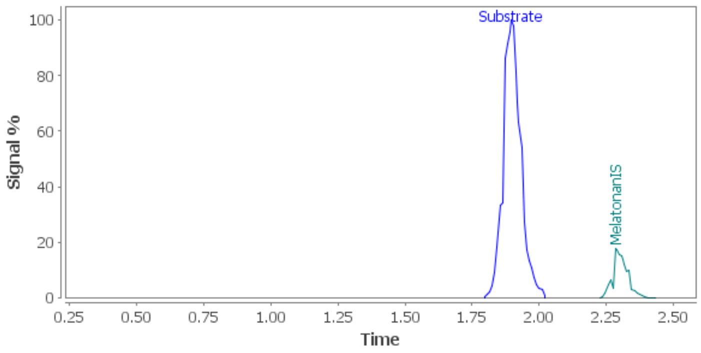

Time=5min

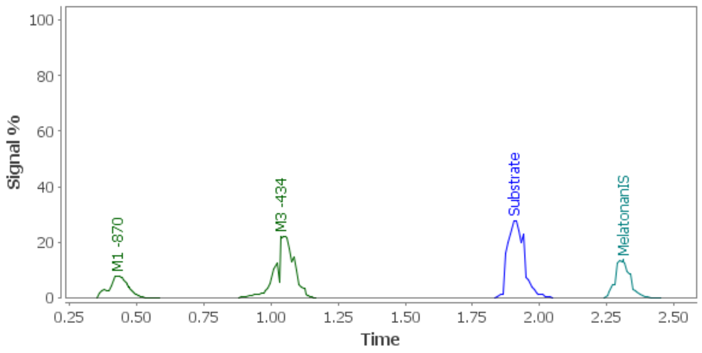

Time=15min

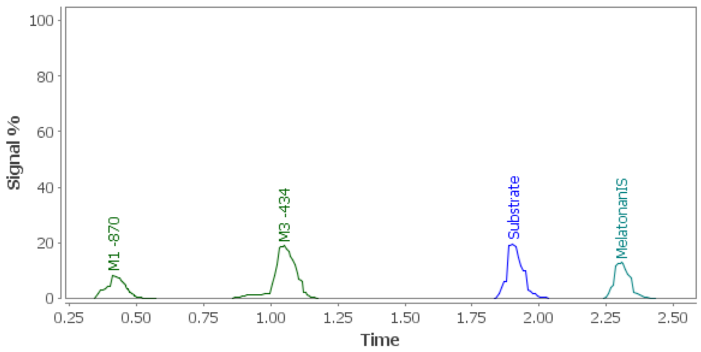

Time=45min

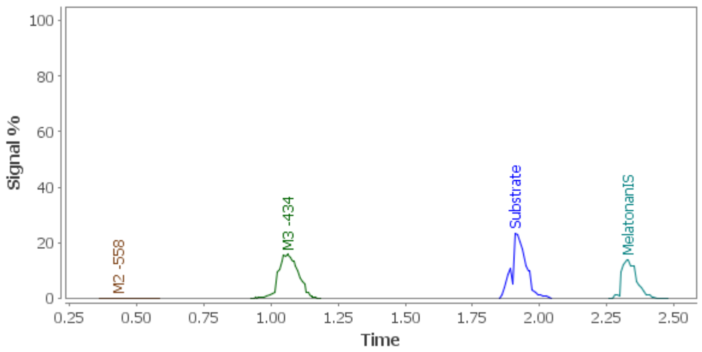

Time=120min

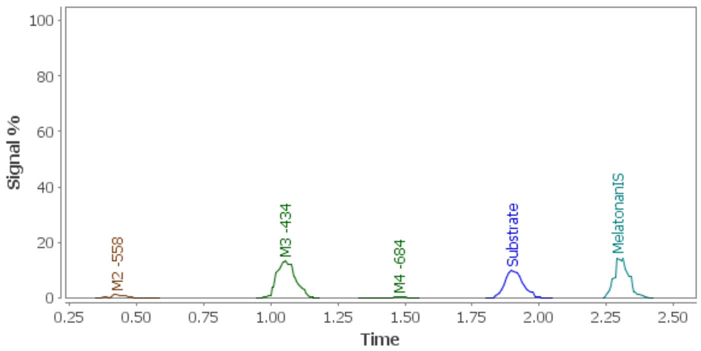

Custom Charts

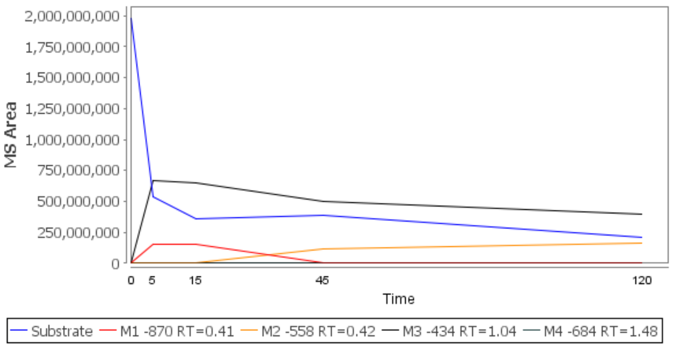

Fragmentation

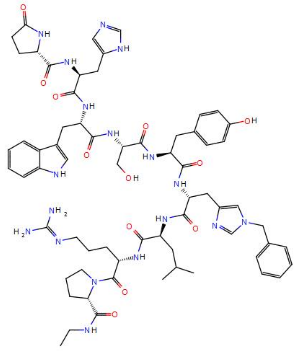

Histrelin

MS (+) FT

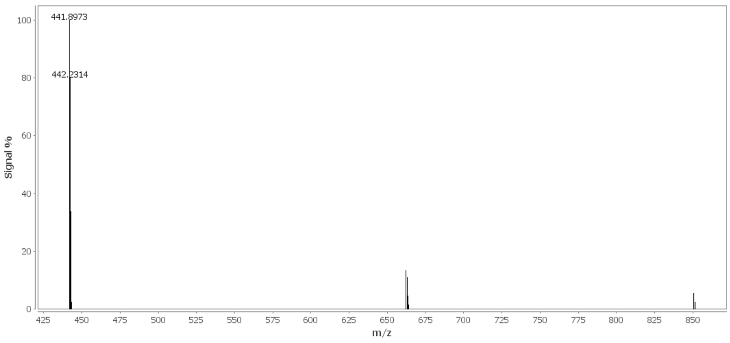

MS (+) FT

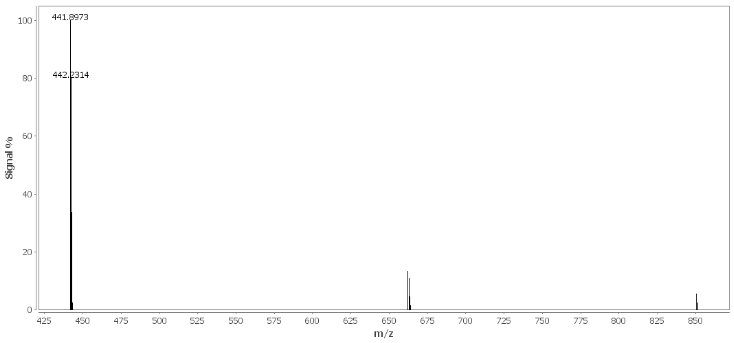

MS2 (+) FT activ = HCD:ce =

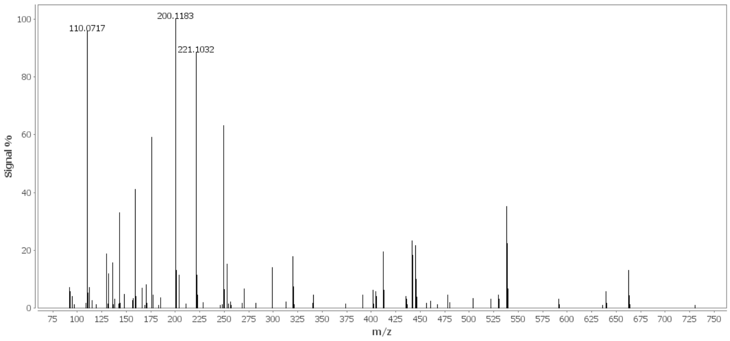

MS2 (+) FT activ = HCD:ce =

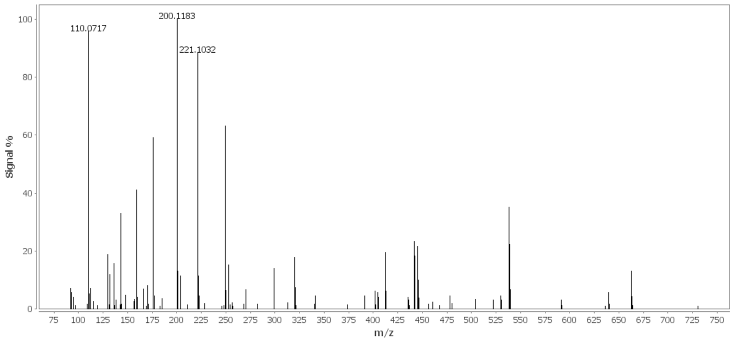

Metabolite: Substrate

Type	score	sub. m/z observed	sub. m/z calculated	sub ppm			met. m/z observed	met. m/z calculated	met. ppm
MATCH	113.3	662.3421	662.3409	-1.73	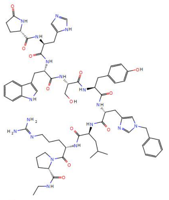	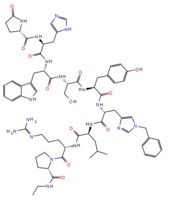	662.3421	662.3409	-1.73
MATCH	70.0	662.3406	662.3409	0.53	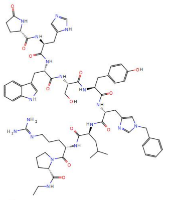	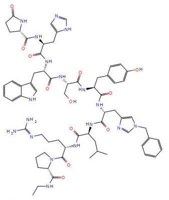	662.3406	662.3409	0.53
MATCH	6.1	591.2862	591.2856	-1.06	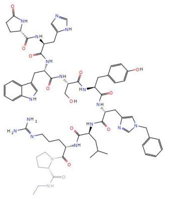	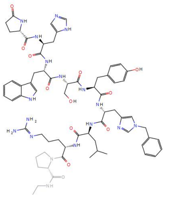	591.2862	591.2856	-1.06

Metabolite: Substrate

Type	score	sub. m/z observed	sub. m/z calculated	sub ppm			met. m/z observed	met. m/z calculated	met. ppm
MISMATCH	-37.1	538.2949	538.2954	1.02	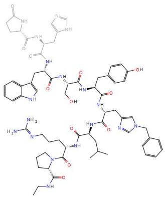	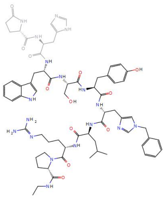	538.2949	538.2954	1.02
MISMATCH	-13.4	529.7866	529.7822	-8.42	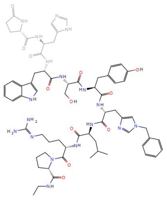	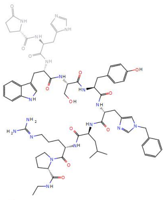	529.7866	529.7822	-8.42
MATCH	7.2	504.2000	504.1990	-2.01	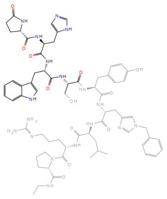	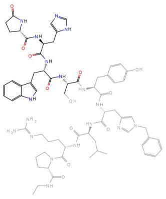	504.2000	504.1990	-2.01
MATCH	3.8	480.2720	480.2718	-0.49	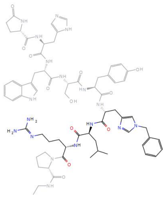	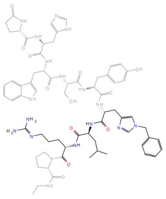	480.2720	480.2718	-0.49
MATCH	6.6	478.2069	478.2085	3.31	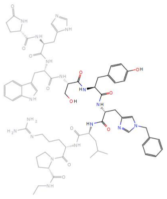	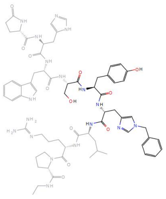	478.2069	478.2085	3.31
MATCH	200.0	441.8973	441.8964	-2.14	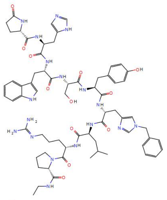	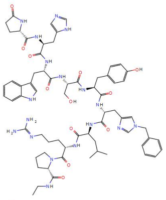	441.8973	441.8964	-2.14
MATCH	80.2	441.8964	441.8964	0.01	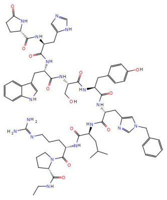	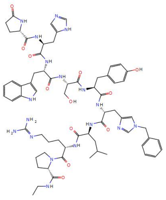	441.8964	441.8964	0.01
MATCH	33.2	412.3028	412.3031	0.70	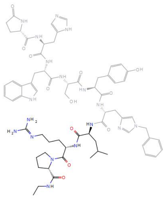	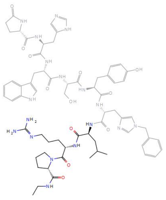	412.3028	412.3031	0.70
MISMATCH	-7.2	404.8834	404.8857	5.66	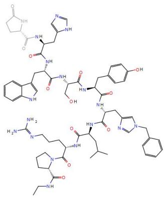	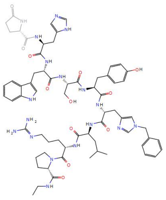	404.8834	404.8857	5.66

Metabolite: Substrate

Type	score	sub. m/z observed	sub. m/z calculated	sub ppm			met. m/z observed	met. m/z calculated	met. ppm
MATCH	27.4	401.7378	401.7398	4.80	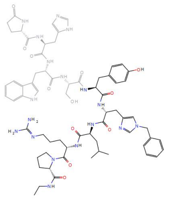	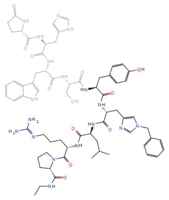	401.7378	401.7398	4.80
MATCH	10.7	391.1755	391.1765	2.44	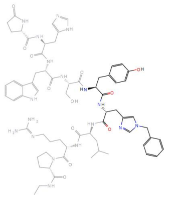	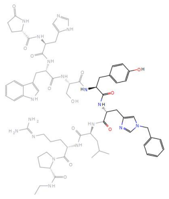	391.1755	391.1765	2.44
MATCH	10.7	391.1755	391.1765	2.44	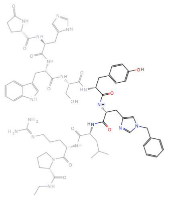	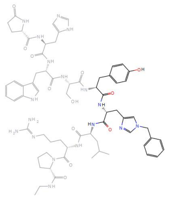	391.1755	391.1765	2.44
MATCH	3.9	374.1509	374.1499	-2.73	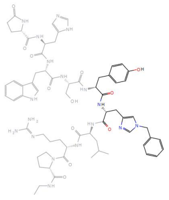	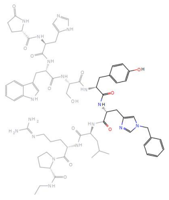	374.1509	374.1499	-2.73
MATCH	6.3	341.1976	341.1972	-1.03	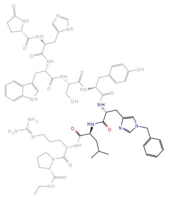	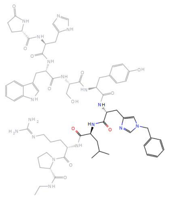	341.1976	341.1972	-1.03
MATCH	7.4	341.1976	341.1972	-1.03	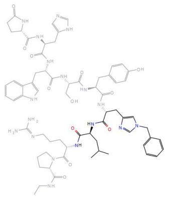	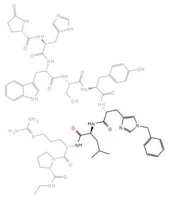	341.1976	341.1972	-1.03
MATCH	41.0	320.2079	320.2081	0.68	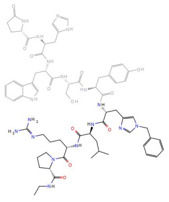	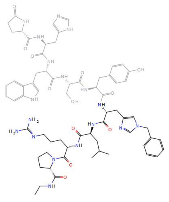	320.2079	320.2081	0.68
MATCH	4.5	313.2025	313.2023	-0.54	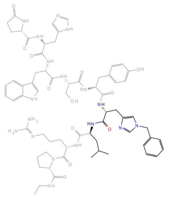	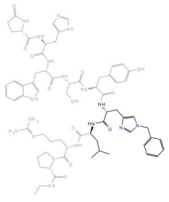	313.2025	313.2023	-0.54
MATCH	28.3	299.2187	299.2190	1.05	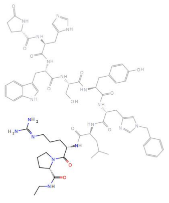	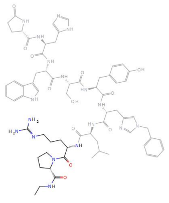	299.2187	299.2190	1.05

Metabolite: Substrate

Type	score	sub. m/z observed	sub. m/z calculated	sub ppm			met. m/z observed	met. m/z calculated	met. ppm
MATCH	3.8	282.1927	282.1925	-0.74	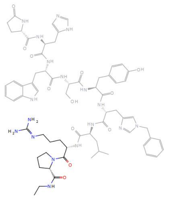	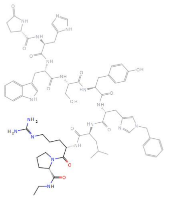	282.1927	282.1925	-0.74
MATCH	11.0	270.1931	270.1925	-2.24	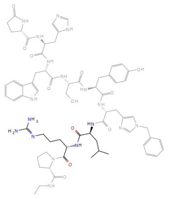	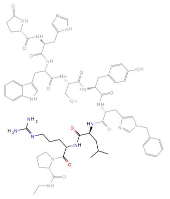	270.1931	270.1925	-2.24
MATCH	4.7	256.1082	256.1081	-0.44	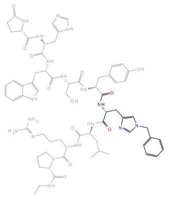	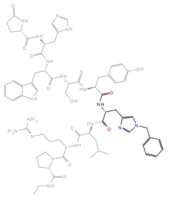	256.1082	256.1081	-0.44
MATCH	26.9	253.1658	253.1659	0.50	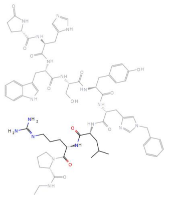	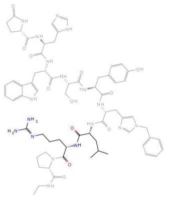	253.1658	253.1659	0.50
MATCH	139.0	249.0980	249.0982	0.94	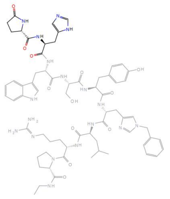	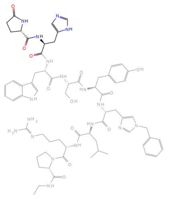	249.0980	249.0982	0.94
MATCH	3.2	228.1132	228.1131	-0.45	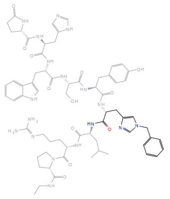	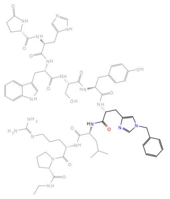	228.1132	228.1131	-0.45
MATCH	3.2	228.1132	228.1131	-0.45	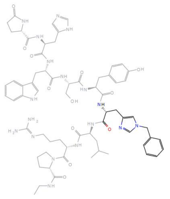	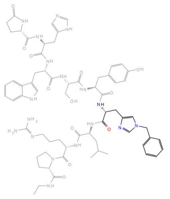	228.1132	228.1131	-0.45
MATCH	167.4	221.1032	221.1033	0.48	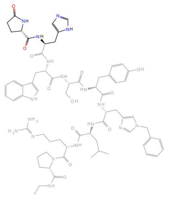	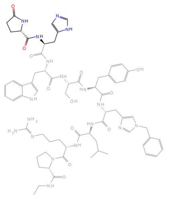	221.1032	221.1033	0.48
MATCH	3.0	211.1441	211.1357	-39.6	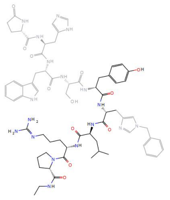	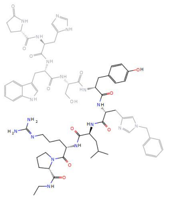	211.1441	211.1357	-39.6

Metabolite: Substrate

Type	score	sub. m/z observed	sub. m/z calculated	sub ppm			met. m/z observed	met. m/z calculated	met. ppm
MATCH	130.4	200.1183	200.1182	-0.24	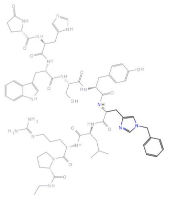	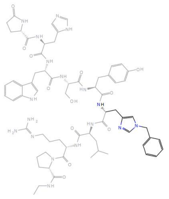	200.1183	200.1182	-0.24
MATCH	5.4	185.1051	185.1033	-9.82	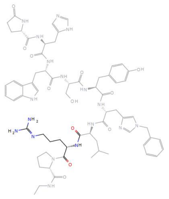	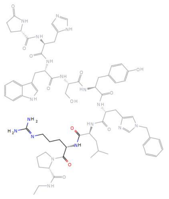	185.1051	185.1033	-9.82
MATCH	5.4	185.1051	185.1073	11.91	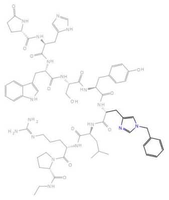	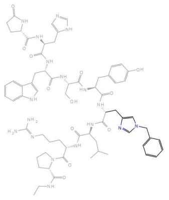	185.1051	185.1073	11.91
MATCH	5.4	185.1051	185.0997	-29.3	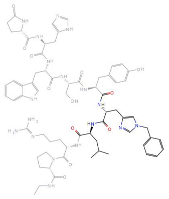	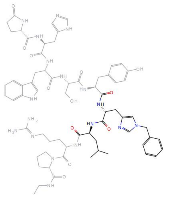	185.1051	185.0997	-29.3
MATCH	14.9	166.0612	166.0611	-0.35	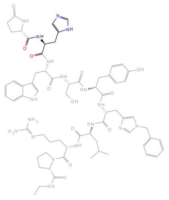	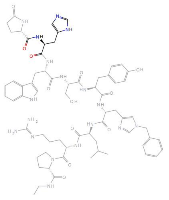	166.0612	166.0611	-0.35
MATCH	4.0	160.0757	160.0743	-8.27	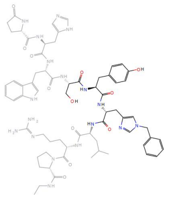	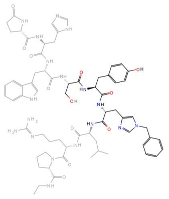	160.0757	160.0743	-8.27
MATCH	52.9	159.0916	159.0917	0.17	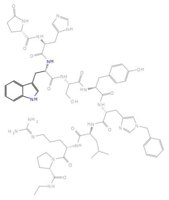	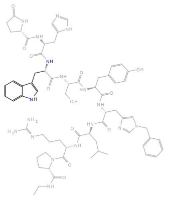	159.0916	159.0917	0.17
MATCH	5.7	157.1082	157.1084	1.38	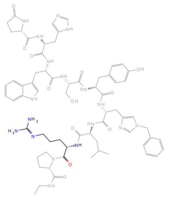	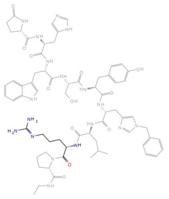	157.1082	157.1084	1.38
MATCH	56.2	143.1179	143.1179	-0.10	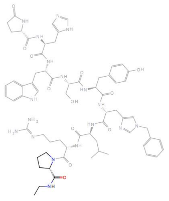	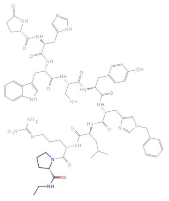	143.1179	143.1179	-0.10

Metabolite: Substrate

Type	score	sub. m/z observed	sub. m/z calculated	sub ppm			met. m/z observed	met. m/z calculated	met. ppm
MATCH	32.8	136.0757	136.0757	-0.42	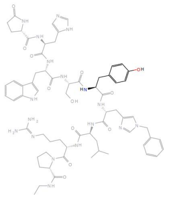	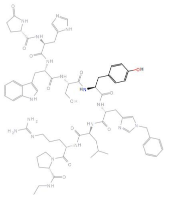	136.0757	136.0757	-0.42
MATCH	3.0	119.0494	119.0491	-2.09	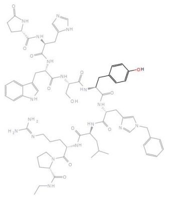	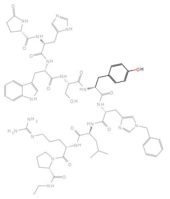	119.0494	119.0491	-2.09
MATCH	4.6	115.0867	115.0866	-1.18	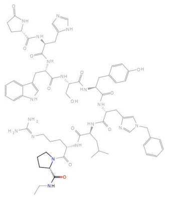	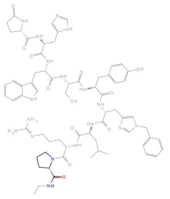	115.0867	115.0866	-1.18
MISMATCH	195.9	110.0717	110.0713	-3.43	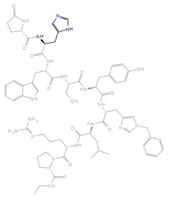	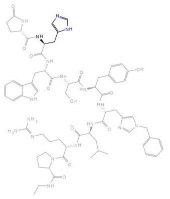	110.0717	110.0713	-3.43
MISMATCH	5.5	95.0608	95.0604	-4.19	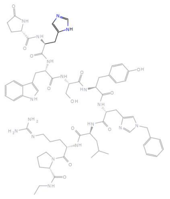	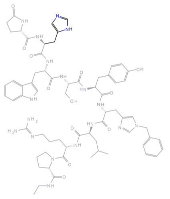	95.0608	95.0604	-4.19

MS (+) FT

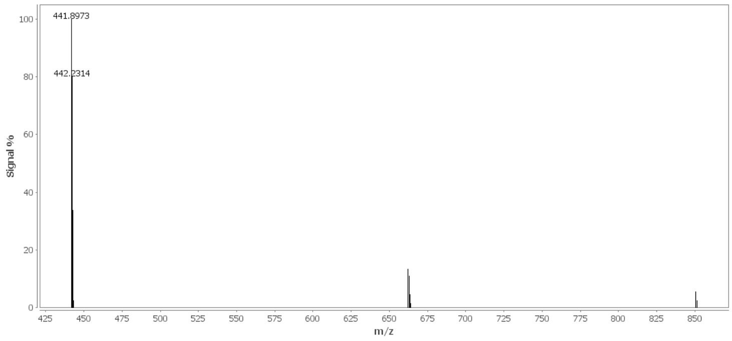

MS (+) FT

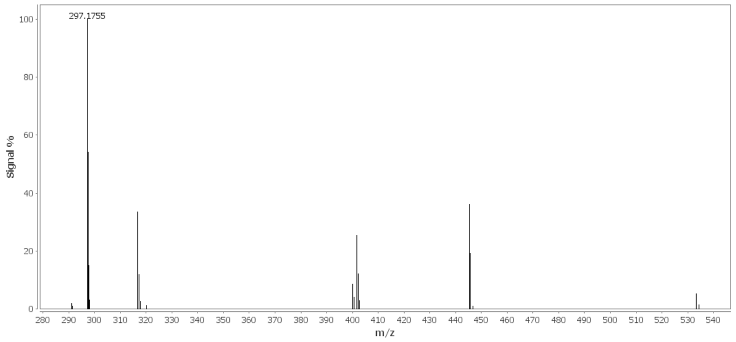

MS2 (+) FT activ = HCD:ce =

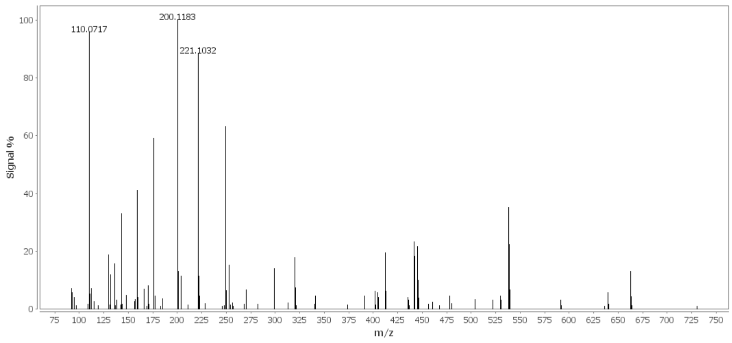

MS2 (+) FT activ = HCD:ce =

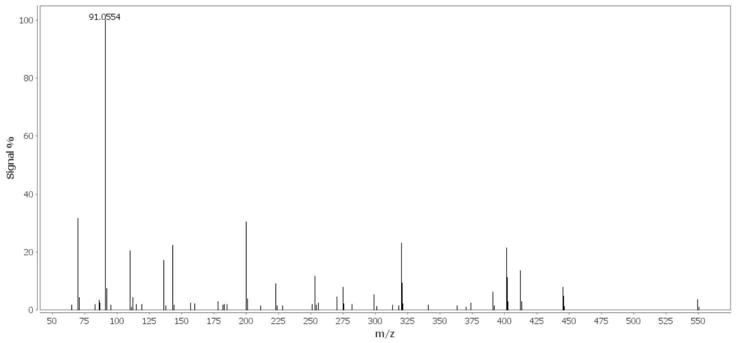

Metabolite: M3 -434 RT=1.04

Type	score	sub. m/z observed	sub. m/z calculated	sub ppm			met. m/z observed	met. m/z calculated	met. ppm
MATCH	200.0	441.8973	441.8964	-2.14	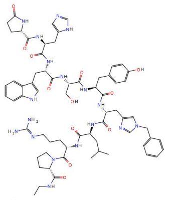	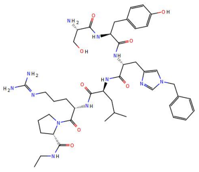	297.1755	297.1729	-8.54
MATCH	200.0	441.8973	441.8964	-2.14	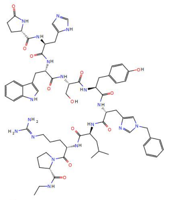	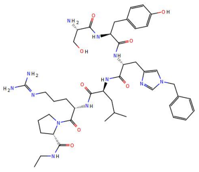	297.1755	297.1729	-8.54
MATCH	136.2	441.8973	441.8964	-2.14	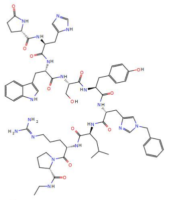	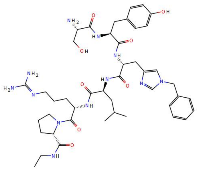	445.2596	445.2558	-8.47
MATCH	136.2	441.8973	441.8964	-2.14	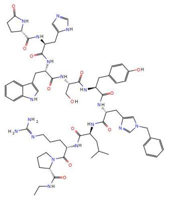	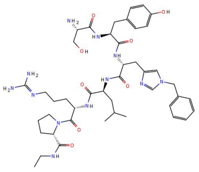	445.2596	445.2558	-8.47
MATCH	113.3	662.3421	662.3409	-1.73	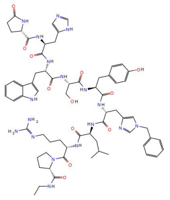	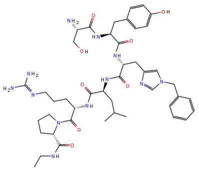	297.1755	297.1729	-8.54
MATCH	113.3	662.3421	662.3409	-1.73	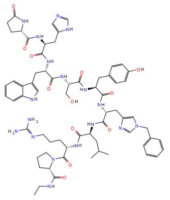	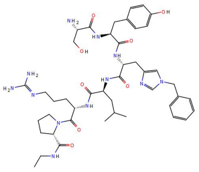	297.1755	297.1729	-8.54
MATCH	49.5	662.3421	662.3409	-1.73	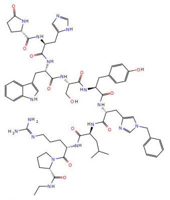	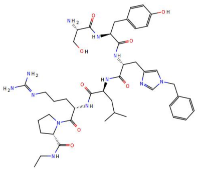	445.2596	445.2558	-8.47
MATCH	49.5	662.3421	662.3409	-1.73	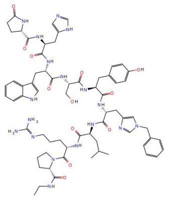	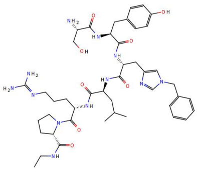	445.2596	445.2558	-8.47
MATCH	4.6	115.0867	115.0866	-1.18	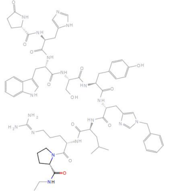	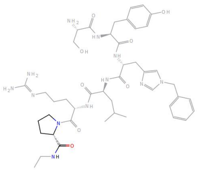	115.0877	115.0866	-9.63

Metabolite: M3 -434 RT=1.04

Type	score	sub. m/z observed	sub. m/z calculated	sub ppm			met. m/z observed	met. m/z calculated	met. ppm
MATCH	3.0	119.0494	119.0491	-2.09	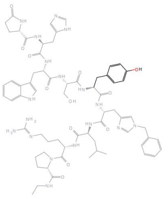	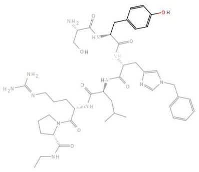	119.0504	119.0491	-10.4
MATCH	32.8	136.0757	136.0757	-0.42	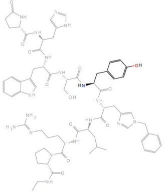	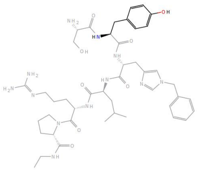	136.0768	136.0757	-7.89
MATCH	55.5	143.1179	143.1179	-0.10	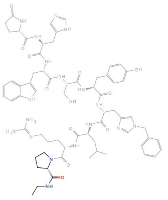	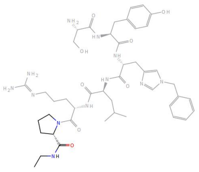	143.1190	143.1179	-7.59
MATCH	5.7	157.1082	157.1084	1.38	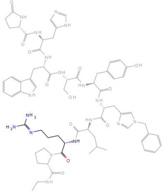	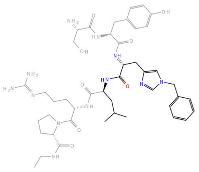	157.1093	157.1048	-28.5
						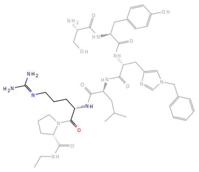	157.1093	157.1084	-5.60
MATCH	4.0	160.0757	160.0743	-8.27	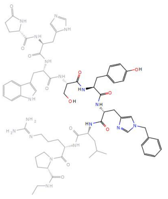	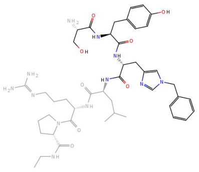	160.0770	160.0743	-16.3
						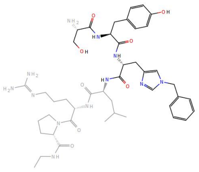	160.0770	160.0743	-16.3
MATCH	5.4	185.1051	185.0997	-29.3	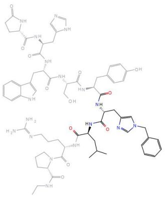	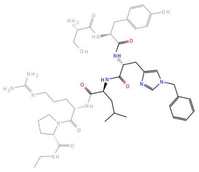	185.1060	185.0997	-34.2
MATCH	5.4	185.1051	185.1033	-9.82	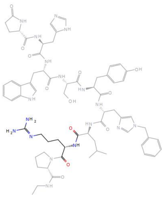	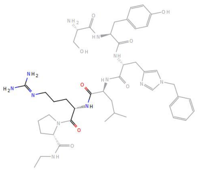	185.1060	185.1033	-14.7

Metabolite: M3 -434 RT=1.04

Type	score	sub. m/z observed	sub. m/z calculated	sub ppm		met. m/z observed	met. m/z calculated	met. ppm
MATCH	5.4	185.1051	185.1073	11.91	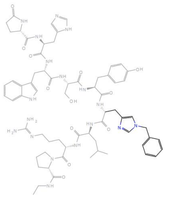 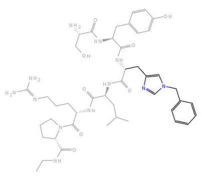	185.1060	185.1073	6.97
MATCH	130.4	200.1183	200.1182	-0.24	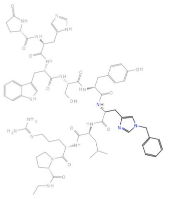 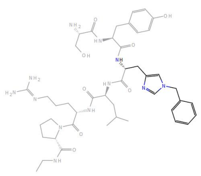	200.1197	200.1182	-7.14
MATCH	3.0	211.1441	211.1357	-39.6	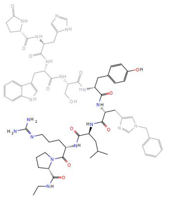 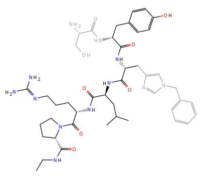	211.1450	211.1357	-43.8
MATCH	3.2	228.1132	228.1131	-0.45	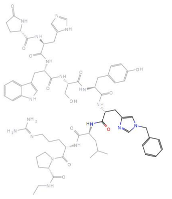 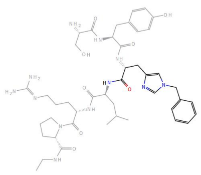	228.1152	228.1131	-9.18
MATCH	3.2	228.1132	228.1131	-0.45	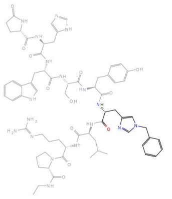 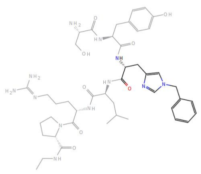	228.1152	228.1131	-9.18
MATCH	26.9	253.1658	253.1659	0.50	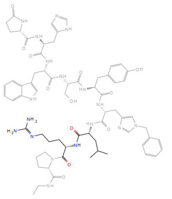 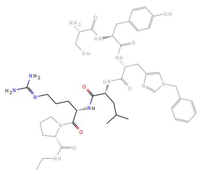	253.1675	253.1659	-6.50
MATCH	4.7	256.1082	256.1081	-0.44	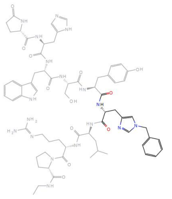 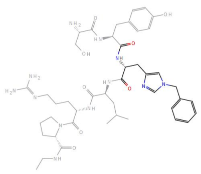	256.1095	256.1081	-5.51
MATCH	11.0	270.1931	270.1925	-2.24	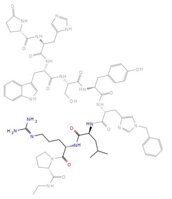 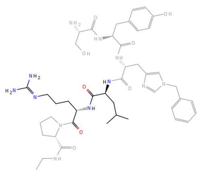	270.1939	270.1925	-5.34
MATCH	3.6	282.1927	282.1925	-0.74	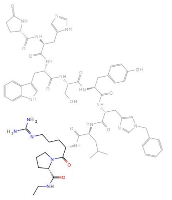 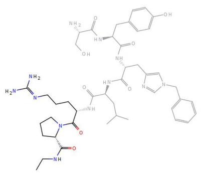	282.1939	282.1925	-5.17

Metabolite: M3 -434 RT=1.04

Type	score	sub. m/z observed	sub. m/z calculated	sub ppm			met. m/z observed	met. m/z calculated	met. ppm
MATCH	19.3	299.2187	299.2190	1.05	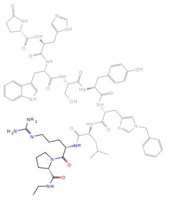	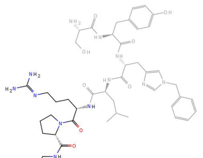	299.2213	299.2190	-7.70
MATCH	4.5	313.2025	313.2023	-0.54	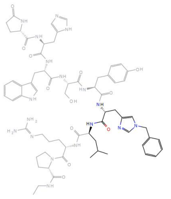	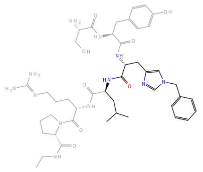	157.1093	157.1048	-28.5
MATCH	4.5	313.2025	313.2023	-0.54	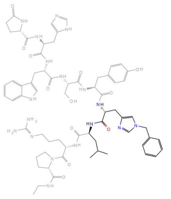	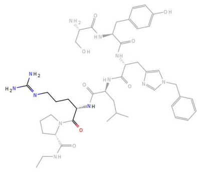	157.1093	157.1084	-5.60
MATCH	3.8	313.2025	313.2023	-0.54	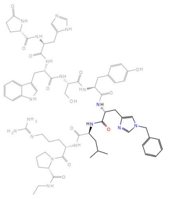	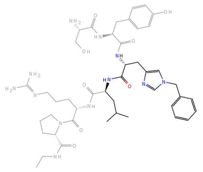	313.2049	313.2023	-8.19
MATCH	41.0	320.2079	320.2081	0.68	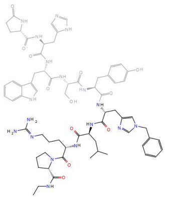	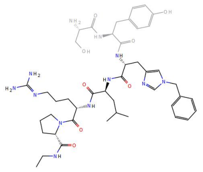	320.2102	320.2081	-6.58
MATCH	6.3	341.1976	341.1972	-1.03	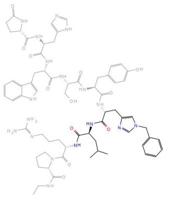	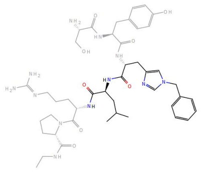	341.1988	341.1972	-4.61
MATCH	6.3	341.1976	341.1972	-1.03	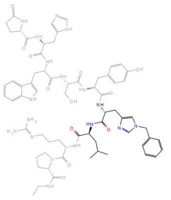	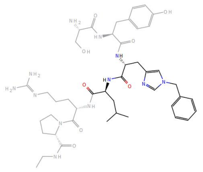	341.1988	341.1972	-4.61
MATCH	3.9	374.1509	374.1499	-2.73	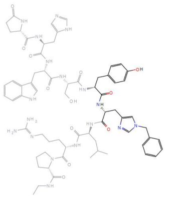	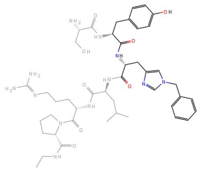	374.1525	374.1499	-7.00
MATCH	10.7	391.1755	391.1765	2.44	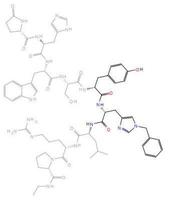	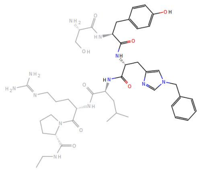	391.1793	391.1765	-7.35

Metabolite: M3 -434 RT=1.04

Type	score	sub. m/z observed	sub. m/z calculated	sub ppm			met. m/z observed	met. m/z calculated	met. ppm
MATCH	10.7	391.1755	391.1765	2.44	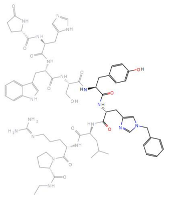	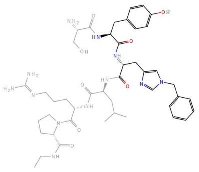	391.1793	391.1765	-7.35
MATCH	27.4	401.7378	401.7398	4.80	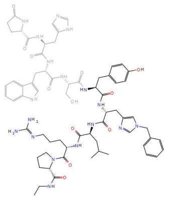	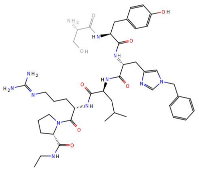	401.7422	401.7398	-5.99
MATCH	33.2	412.3028	412.3031	0.70	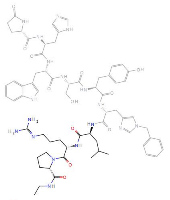	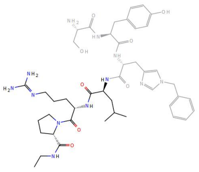	412.3057	412.3031	-6.41
MATCH	31.1	441.8964	441.8964	0.01	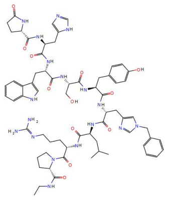	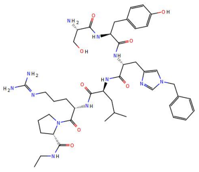	445.2591	445.2558	-7.39
						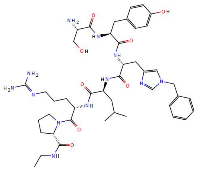	445.2591	445.2558	-7.39
MATCH	6.6	478.2069	478.2085	3.31	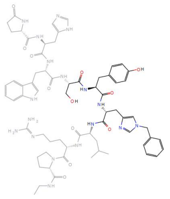	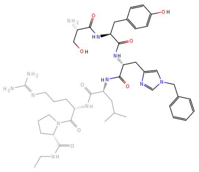	160.0770	160.0743	-16.3
						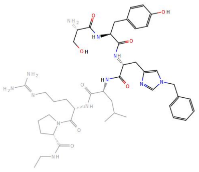	160.0770	160.0743	-16.3
MATCH	7.2	504.2000	504.1990	-2.01	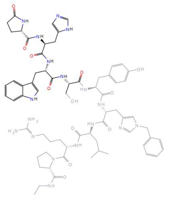	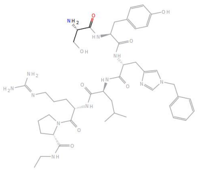	70.0300	70.0287	-17.5
MATCH	20.9	662.3406	662.3409	0.53	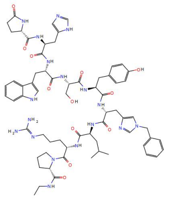	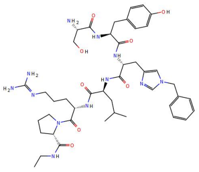	445.2591	445.2558	-7.39

Metabolite: M3 -434 RT=1.04

Type	score	sub. m/z observed	sub. m/z calculated	sub ppm			met. m/z observed	met. m/z calculated	met. ppm
						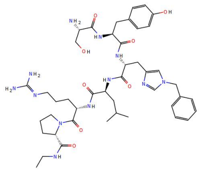	445.2591	445.2558	-7.39
MISMATCH	-5.9	95.0608	95.0604	-4.19	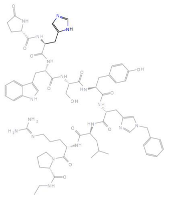		95.0615	95.0615	0.00
MISMATCH	-116.3	110.0717	110.0713	-3.43	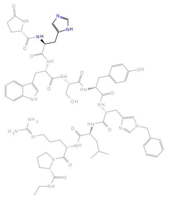		110.0724	110.0724	0.00
MET_MATCH						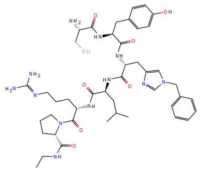	291.1716	291.1694	-7.58
MET_MATCH						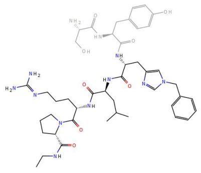	320.2109	320.2081	-8.60
MET_MATCH						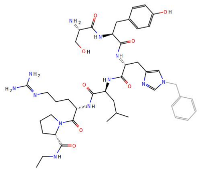	400.2357	400.2323	-8.54
MET_MATCH						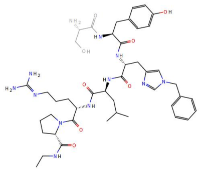	400.7371	400.7319	-12.9
MET_MATCH						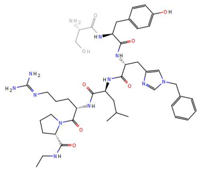	401.7431	401.7398	-8.24
MET_MATCH						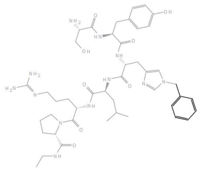	91.0554	91.0542	-13.1

Metabolite: M3 -434 RT=1.04

Type	score	sub. m/z observed	sub. m/z calculated	sub ppm		met. m/z observed	met. m/z calculated	met. ppm
MET_MATCH					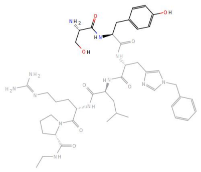	223.1092	223.1077	-6.64

MS (+) FT

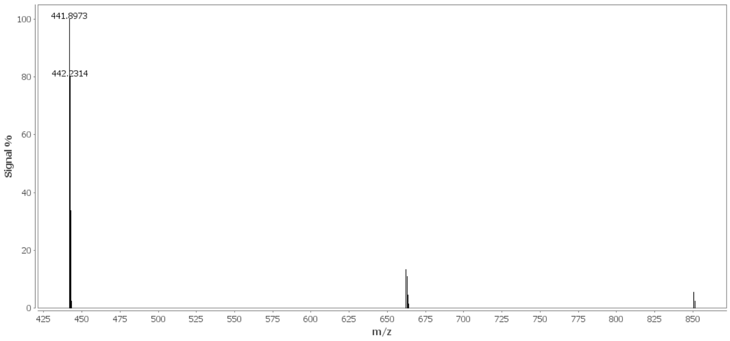

MS (+) FT

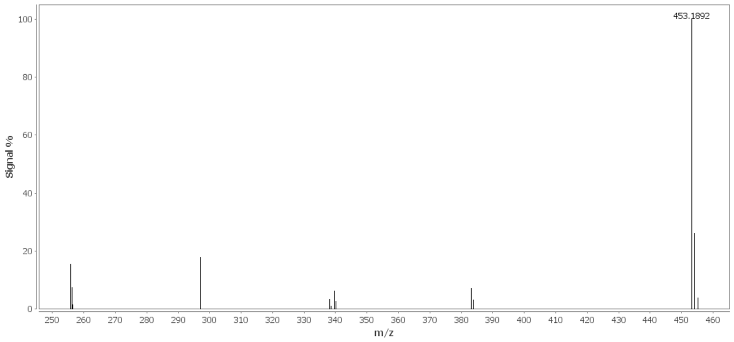

MS2 (+) FT activ = HCD:ce =

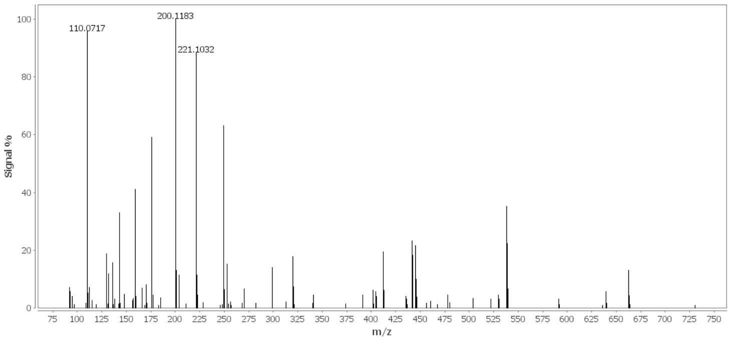

MS2 (+) FT activ = HCD:ce =

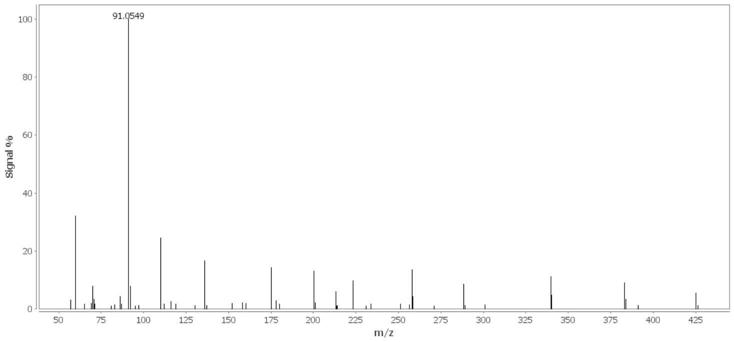

Metabolite: M2 -558 RT=0.42

Type	score	sub. m/z observed	sub. m/z calculated	sub ppm			met. m/z observed	met. m/z calculated	met. ppm
MATCH	115.5	441.8973	441.8964	-2.14	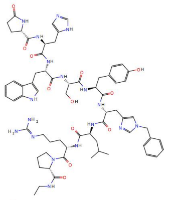	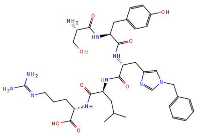	255.8068	255.8063	-2.25
MATCH	115.5	441.8973	441.8964	-2.14	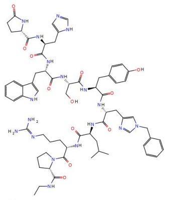	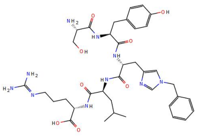	255.8068	255.8063	-2.25
MATCH	107.1	441.8973	441.8964	-2.14	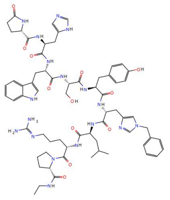	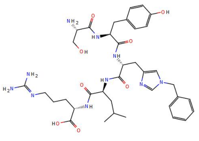	383.2068	383.2058	-2.61

Metabolite: M2 -558 RT=0.42

Type	score	sub. m/z observed	sub. m/z calculated	sub ppm			met. m/z observed	met. m/z calculated	met. ppm
MATCH	107.1	441.8973	441.8964	-2.14	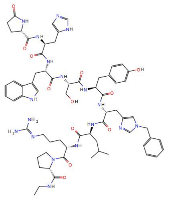	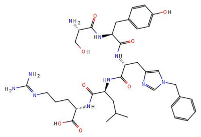	383.2068	383.2058	-2.61
MATCH	28.9	662.3421	662.3409	-1.73	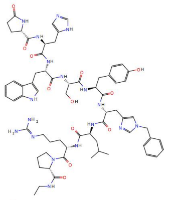	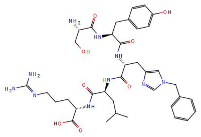	255.8068	255.8063	-2.25
MATCH	28.9	662.3421	662.3409	-1.73	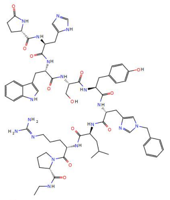	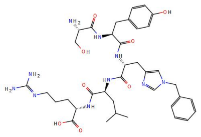	255.8068	255.8063	-2.25
MATCH	20.4	662.3421	662.3409	-1.73	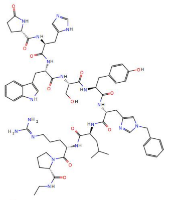	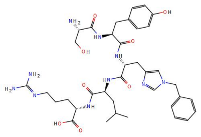	383.2068	383.2058	-2.61
MATCH	20.4	662.3421	662.3409	-1.73	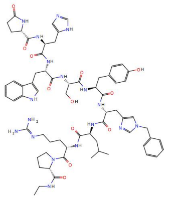	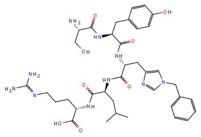	383.2068	383.2058	-2.61
MATCH	2.8	119.0494	119.0491	-2.09	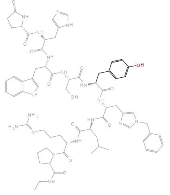	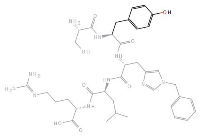	119.0498	119.0491	-5.28
MATCH	32.4	136.0757	136.0757	-0.42	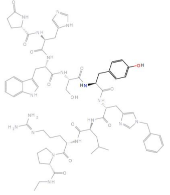	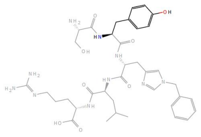	136.0759	136.0757	-1.54
MATCH	3.8	160.0757	160.0743	-8.27	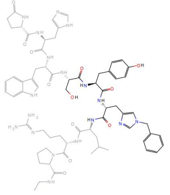	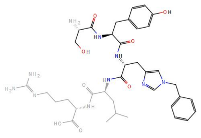	160.0762	160.0743	-11.3
						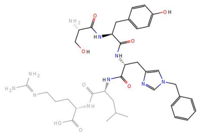	160.0762	160.0743	-11.3

Metabolite: M2 -558 RT=0.42

Type	score	sub. m/z observed	sub. m/z calculated	sub ppm		met. m/z observed	met. m/z calculated	met. ppm
MATCH	113.0	200.1183	200.1182	-0.24	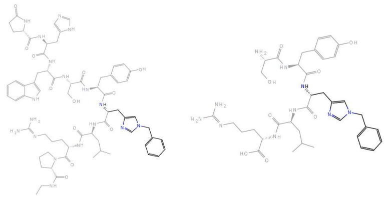	200.1183	200.1182	-0.47
MATCH	3.8	282.1927	282.1925	-0.74	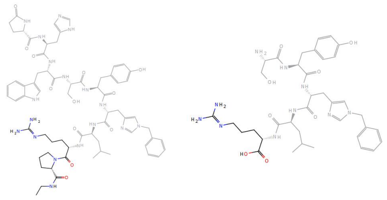	158.0926	158.0924	-1.00
MATCH	28.3	299.2187	299.2190	1.05	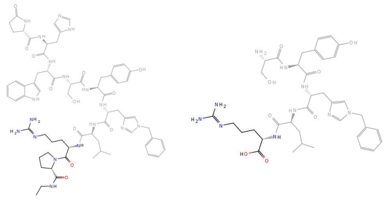	175.1191	175.1190	-0.90
MATCH	31.5	320.2079	320.2081	0.68	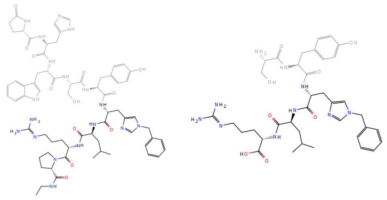	258.1583	258.1581	-0.73
MATCH	5.8	391.1755	391.1765	2.44	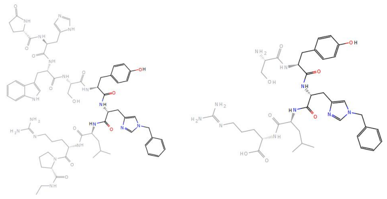	391.1754	391.1765	2.68
MATCH	5.8	391.1755	391.1765	2.44	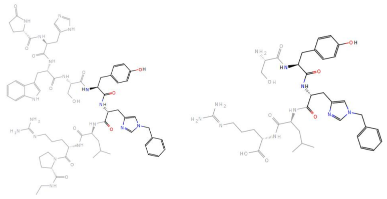	391.1754	391.1765	2.68
MATCH	17.2	401.7378	401.7398	4.80	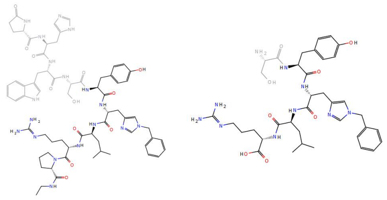	339.6897	339.6897	0.09
MATCH	28.1	412.3028	412.3031	0.70	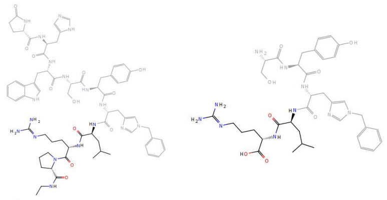	288.2030	288.2030	0.07
MATCH	32.3	441.8964	441.8964	0.01	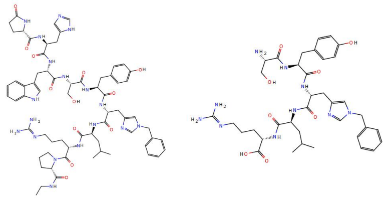	383.2059	383.2058	-0.41

Metabolite: M2 -558 RT=0.42

Type	score	sub. m/z observed	sub. m/z calculated	sub ppm			met. m/z observed	met. m/z calculated	met. ppm
						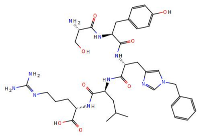	383.2059	383.2058	-0.41
MATCH	6.5	478.2069	478.2085	3.31	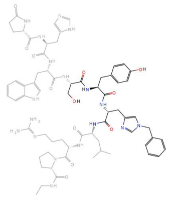	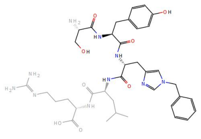	160.0762	160.0743	-11.3
						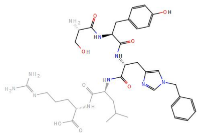	160.0762	160.0743	-11.3
MATCH	5.9	504.2000	504.1990	-2.01	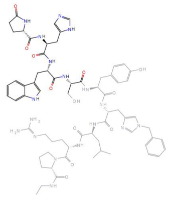	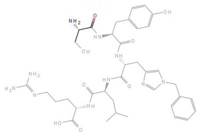	70.0296	70.0287	-12.2
MATCH	22.1	662.3406	662.3409	0.53	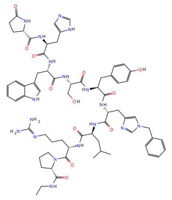	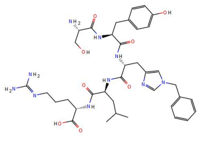	383.2059	383.2058	-0.41
						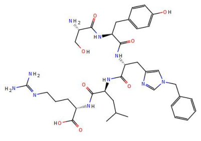	383.2059	383.2058	-0.41
MISMATCH	-120.3	110.0717	110.0713	-3.43	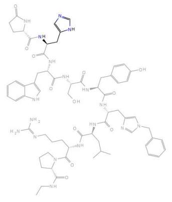		110.0718	110.0718	0.00
MET_MATCH						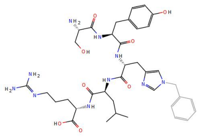	338.1831	338.1823	-2.51
MET_MATCH						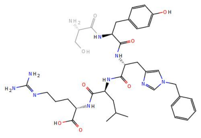	339.6906	339.6897	-2.42

Metabolite: M2 -558 RT=0.42

Type	score	sub. m/z observed	sub. m/z calculated	sub ppm		met. m/z observed	met. m/z calculated	met. ppm
MET_MATCH					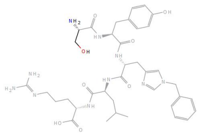	60.0454	60.0444	-16.0
MET_MATCH					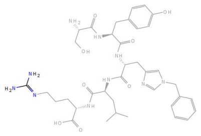	60.0566	60.0556	-15.6
MET_MATCH					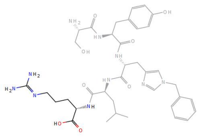	87.0560	87.0553	-8.26
MET_MATCH					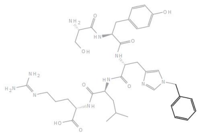	91.0549	91.0542	-7.61
MET_MATCH					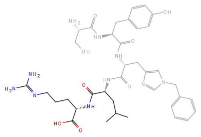	91.0549	91.0637	96.12
MET_MATCH					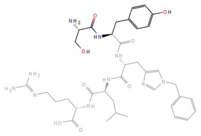	223.1080	223.1077	-1.13
MET_MATCH					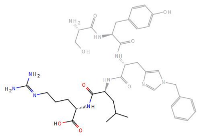	271.1772	271.1765	-2.86

MS (+) FT

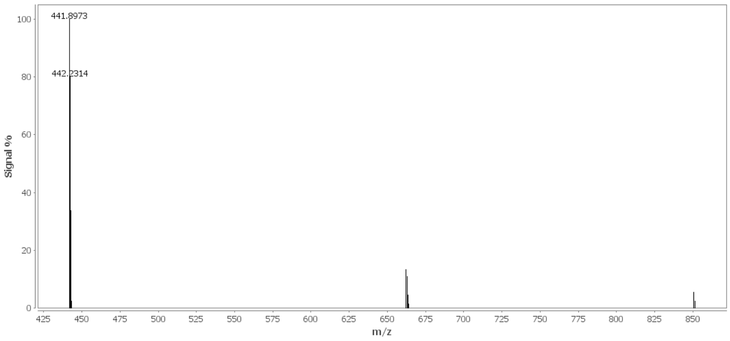

MS (+) FT

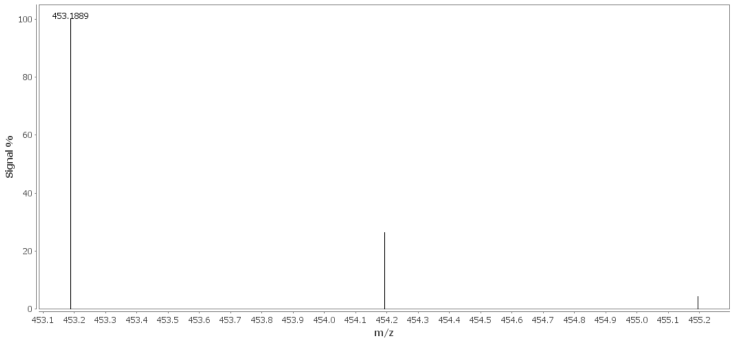

MS2 (+) FT activ = HCD:ce =

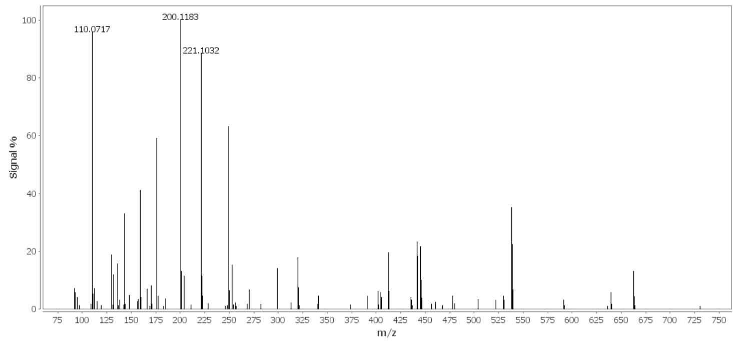

MS2 (+) FT activ = HCD:ce =

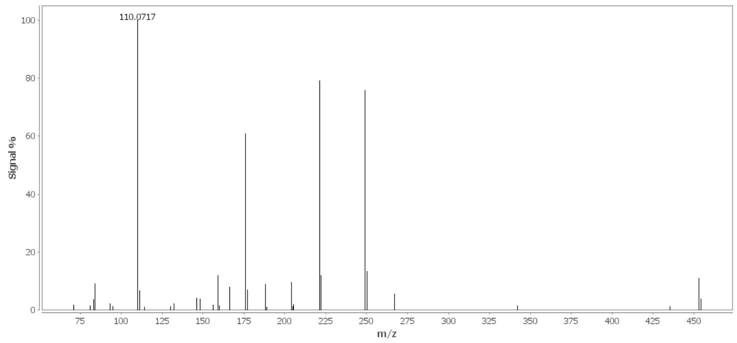

Metabolite: M1 -870 RT=0.41

Type	score	sub. m/z observed	sub. m/z calculated	sub ppm			met. m/z observed	met. m/z calculated	met. ppm
MATCH	200.0	441.8973	441.8964	-2.14	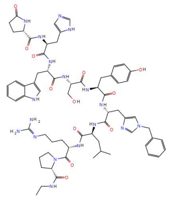	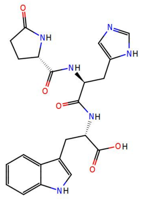	453.1889	453.1881	-1.69
						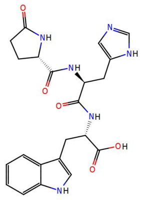	453.1889	453.1881	-1.69
MATCH	113.3	662.3421	662.3409	-1.73	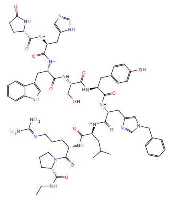	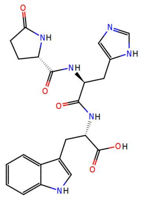	453.1889	453.1881	-1.69
						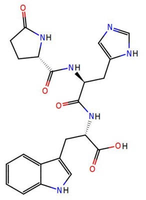	453.1889	453.1881	-1.69
MATCH	5.5	95.0608	95.0604	-4.19	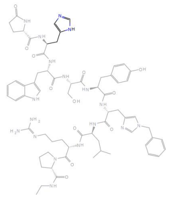	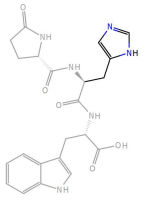	95.0610	95.0604	-6.08
MATCH	195.9	110.0717	110.0713	-3.43	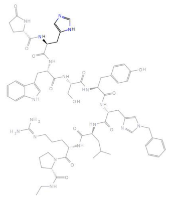	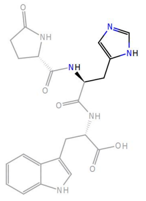	110.0717	110.0713	-3.78
MATCH	52.9	159.0916	159.0917	0.17	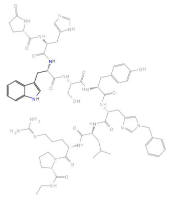	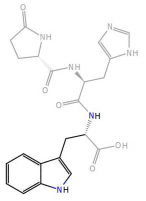	159.0917	159.0917	-0.30

Metabolite: M1 -870 RT=0.41

Type	score	sub. m/z observed	sub. m/z calculated	sub ppm			met. m/z observed	met. m/z calculated	met. ppm
MATCH	14.9	166.0612	166.0611	-0.35	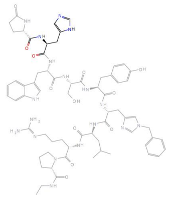	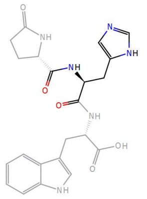	166.0611	166.0611	-0.28
MATCH	167.4	221.1032	221.1033	0.48	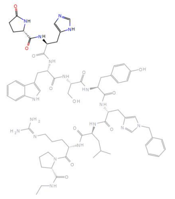	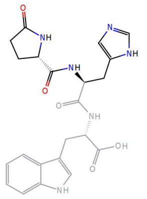	221.1033	221.1033	0.18
MATCH	139.0	249.0980	249.0982	0.94	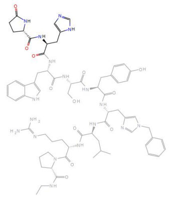	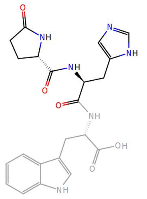	249.0982	249.0982	0.12
MATCH	34.1	441.8964	441.8964	0.01	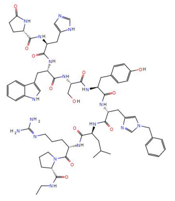	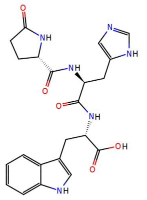	453.1877	453.1881	0.77
						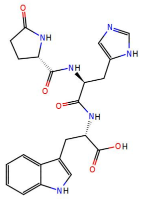	453.1877	453.1881	0.77
MATCH	23.9	662.3406	662.3409	0.53	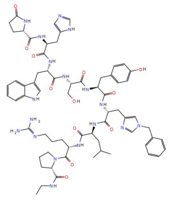	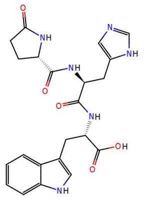	453.1877	453.1881	0.77
						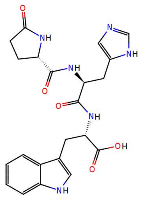	453.1877	453.1881	0.77
MISMATCH	-7.2	404.8834	404.8857	5.66	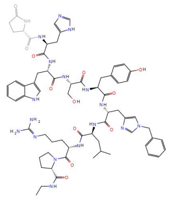		342.1559	342.1559	0.00
MISMATCH	-13.4	529.7866	529.7822	-8.42	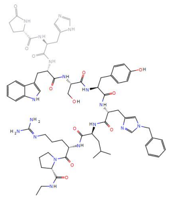		188.0706	188.0706	0.00

Metabolite: M1 -870 RT=0.41

Type	score	sub. m/z observed	sub. m/z calculated	sub ppm		met. m/z observed	met. m/z calculated	met. ppm
MISMATCH	-37.1	538.2949	538.2954	1.02	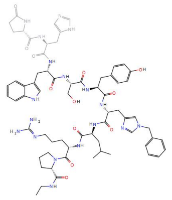	205.0975	205.0975	0.00
MET_MATCH					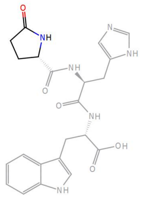	84.0451	84.0444	-8.70
MET_MATCH					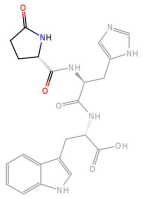	114.0553	114.0550	-3.43
MET_MATCH					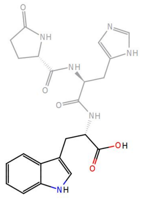	188.0706	188.0706	0.22
MET_MATCH					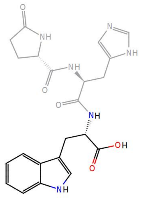	205.0975	205.0972	-1.90
MET_MATCH					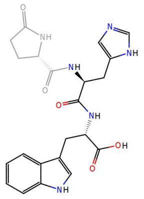	342.1559	342.1561	0.47
MET_MATCH					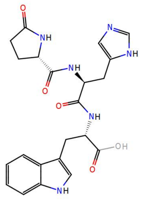	435.1776	435.1775	-0.10

MS (+) FT

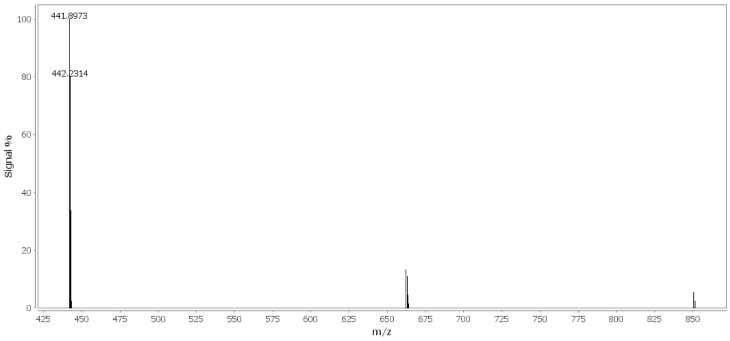

MS (+) FT

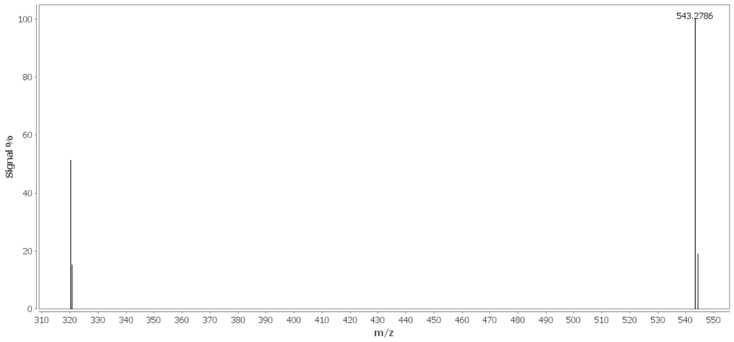

MS2 (+) FT activ = HCD:ce =

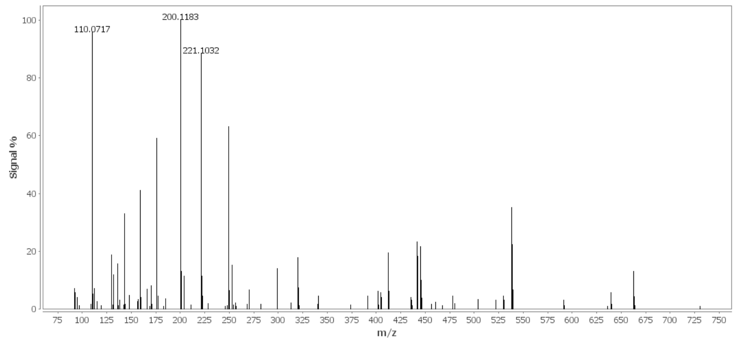

MS2 (+) FT activ = HCD:ce =

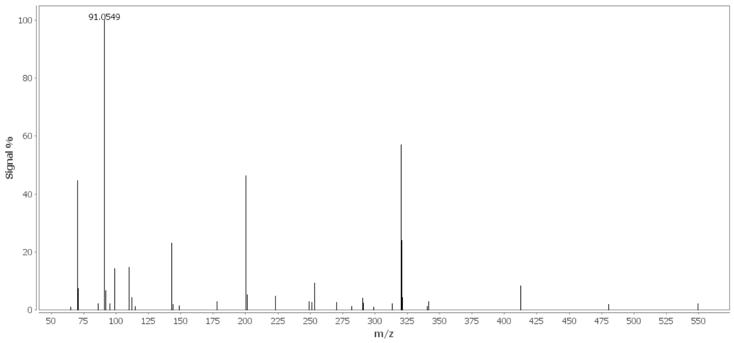

Metabolite: M4 -684 RT=1.48

Type	score	sub. m/z observed	sub. m/z calculated	sub ppm		met. m/z observed	met. m/z calculated	met. ppm
MATCH	151.2	441.8973	441.8964	-2.14	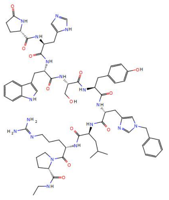 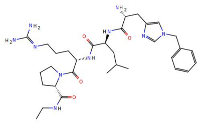	320.2088	320.2081	-2.24
					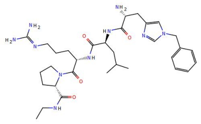	320.2088	320.2081	-2.24
MATCH	64.5	662.3421	662.3409	-1.73	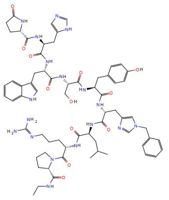 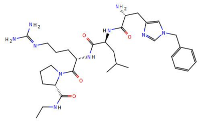	320.2088	320.2081	-2.24
					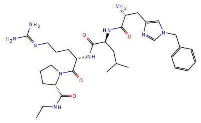	320.2088	320.2081	-2.24
MATCH	3.8	115.0867	115.0866	-1.18	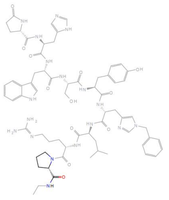 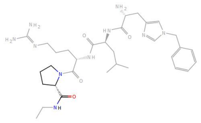	115.0871	115.0866	-4.81
MATCH	56.2	143.1179	143.1179	-0.10	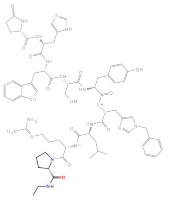 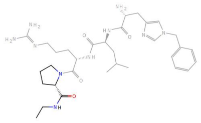	143.1181	143.1179	-1.57
MATCH	24.6	253.1658	253.1659	0.50	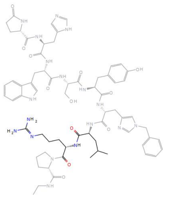 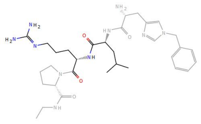	253.1656	253.1659	1.17

Metabolite: M4 -684 RT=1.48

Type	score	sub. m/z observed	sub. m/z calculated	sub ppm			met. m/z observed	met. m/z calculated	met. ppm
MATCH	9.3	270.1931	270.1925	-2.24	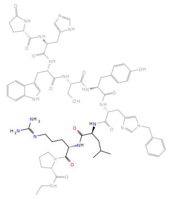	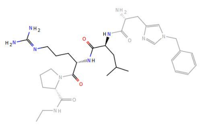	270.1924	270.1925	0.14
MATCH	2.9	282.1927	282.1925	-0.74	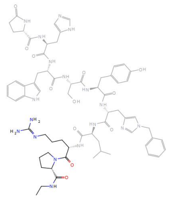	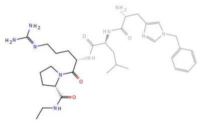	282.1924	282.1925	0.24
MATCH	15.2	299.2187	299.2190	1.05	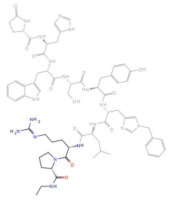	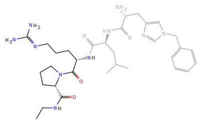	299.2200	299.2190	-3.24
MATCH	7.4	341.1976	341.1972	-1.03	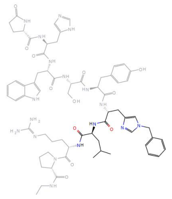	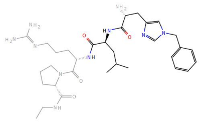	341.1986	341.1972	-4.03
MATCH	27.9	412.3028	412.3031	0.70	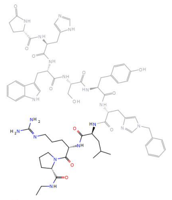	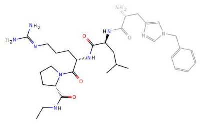	412.3041	412.3031	-2.56
MATCH	80.2	441.8964	441.8964	0.01	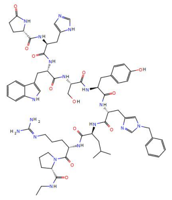	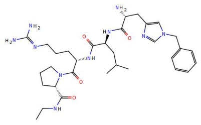	320.2085	320.2081	-1.21
						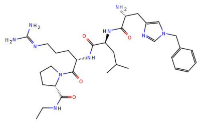	320.2085	320.2081	-1.21
MATCH	3.8	480.2720	480.2718	-0.49	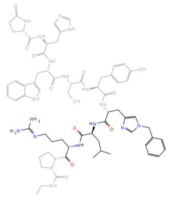	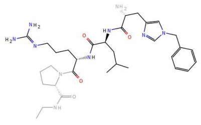	480.2712	480.2718	1.09
MATCH	6.1	591.2862	591.2856	-1.06	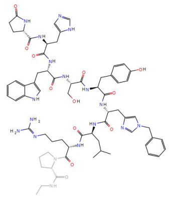	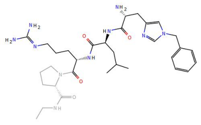	249.1541	249.1528	-5.11

Metabolite: M4 -684 RT=1.48

Type	score	sub. m/z observed	sub. m/z calculated	sub ppm			met. m/z observed	met. m/z calculated	met. ppm
MATCH	70.0	662.3406	662.3409	0.53	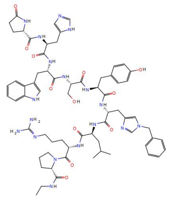	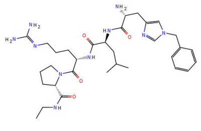	320.2085	320.2081	-1.21
						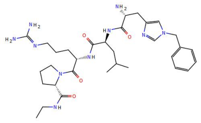	320.2085	320.2081	-1.21
MISMATCH	-6.3	95.0608	95.0604	-4.19	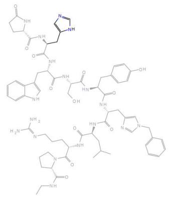		95.0609	95.0609	0.00
MISMATCH	-110.6	110.0717	110.0713	-3.43	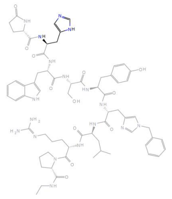		110.0719	110.0719	0.00
MET_MATCH						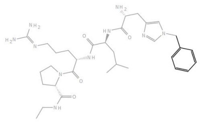	91.0549	91.0542	-7.56
MET_MATCH						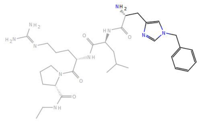	200.1183	200.1182	-0.56
MET_MATCH						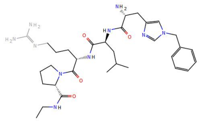	290.6851	290.6839	-3.99
MET_MATCH						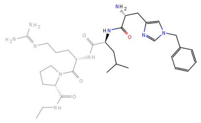	313.2020	313.2023	0.93
MET_MATCH						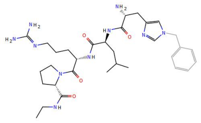	549.3607	549.3620	2.29
