# Supplementary material for: Software-aided approach to investigate peptide structure and metabolic susceptibility of amide bonds in peptide drugs based on high resolution mass spectrometry
Source: PLoS One. 2017 Nov 1;12(11):e0186461. doi: 10.1371/journal.pone.0186461 (PMC5665424; doi:10.1371/journal.pone.0186461)
Supplement: S1 File — (ZIP) [file pone.0186461.s007.zip › SFiles/S19_File.pdf]

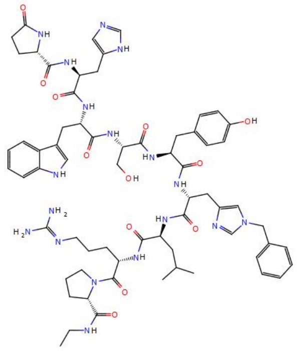

Histrelin

Property name	Property value
Time	0min, 5min, 15min, 45min, 120min
Instrument	ThermoQAPlus
Matrix	elastase
Acquisition Mode	ddMS2

Chromatograms

Time=0min

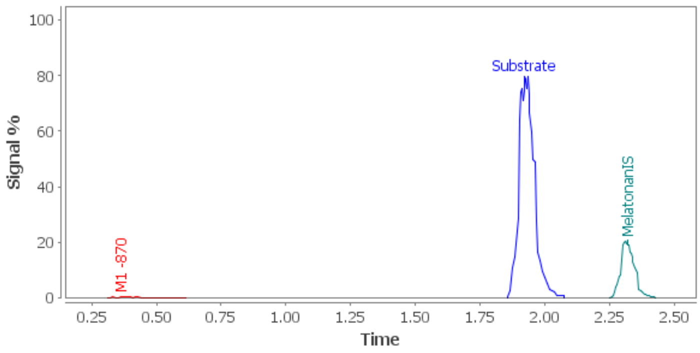

Time=5min

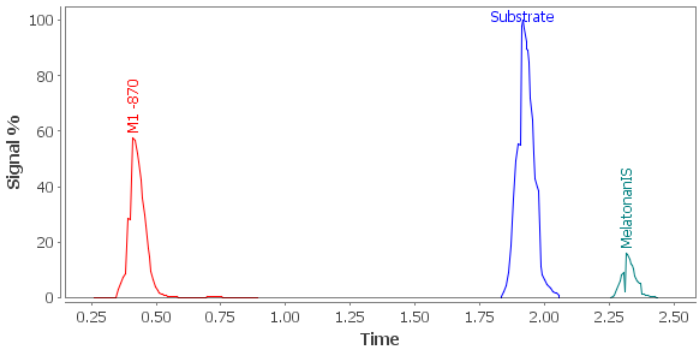

Time=15min

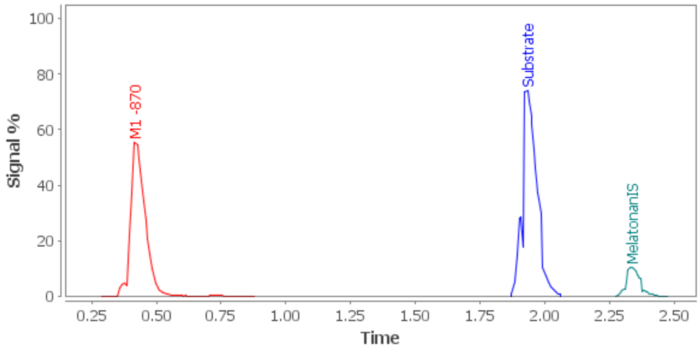

Time=45min

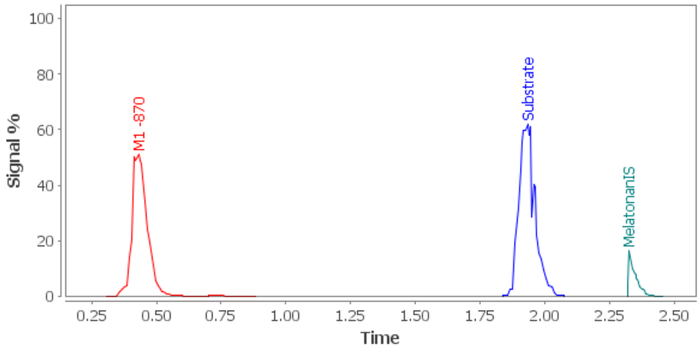

Time=120min

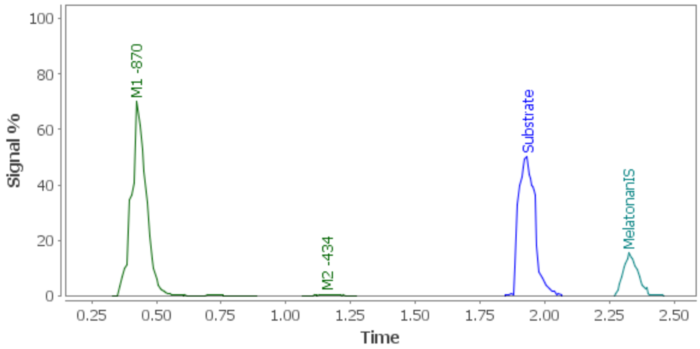

Custom Charts

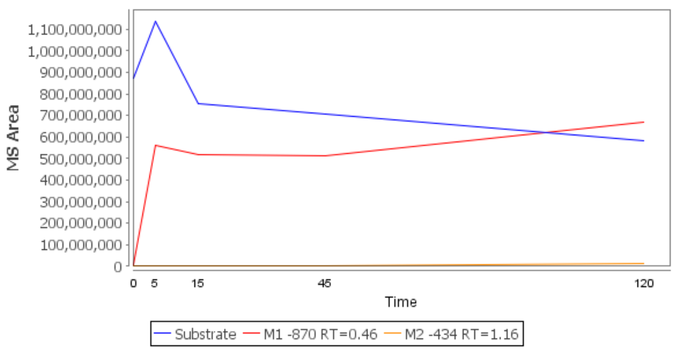

Fragmentation

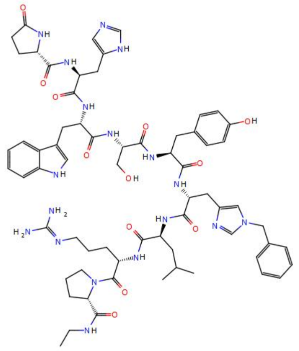

Histrelin

MS (+) FT

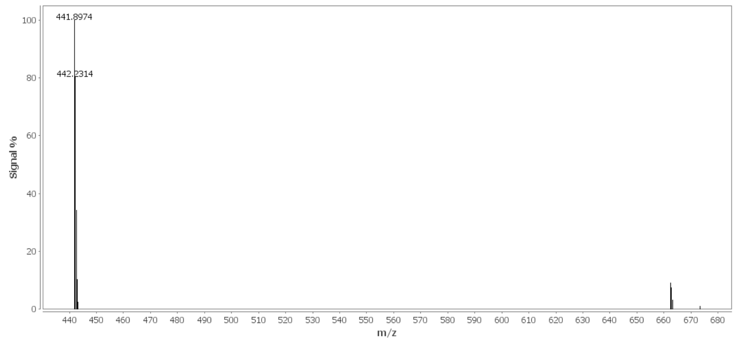

MS (+) FT

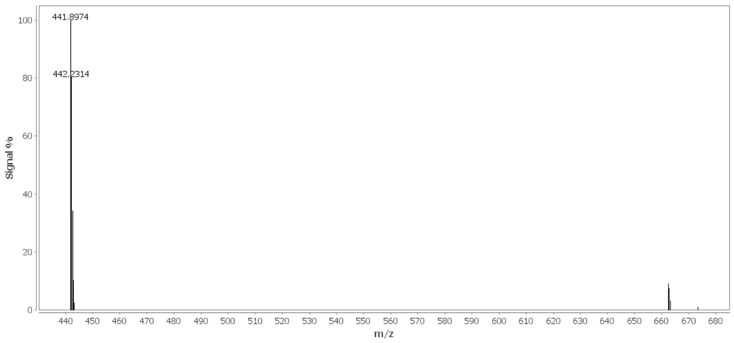

MS2 (+) FT activ = HCD:ce =

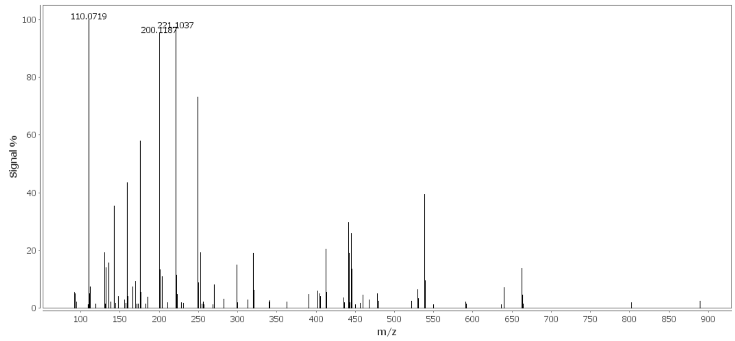

MS2 (+) FT activ = HCD:ce =

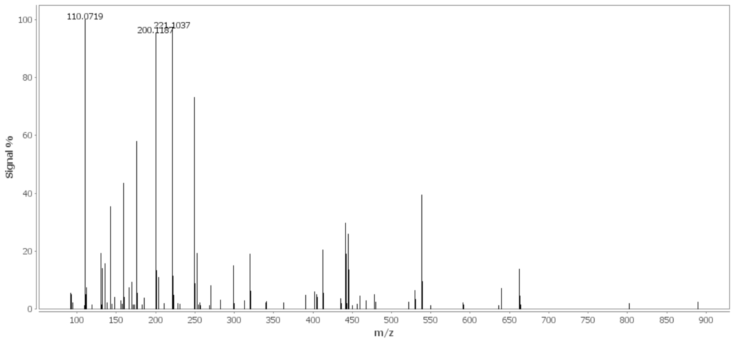

Metabolite: Substrate

Type	score	sub. m/z observed	sub. m/z calculated	sub ppm			met. m/z observed	met. m/z calculated	met. ppm
MATCH	15.9	662.3424	662.3409	-2.22	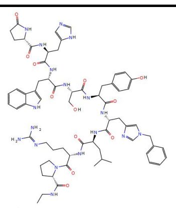	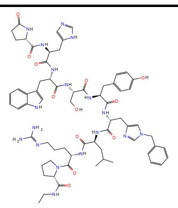	662.3424	662.3409	-2.22
MATCH	108.2	662.3420	662.3409	-1.68	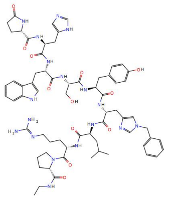	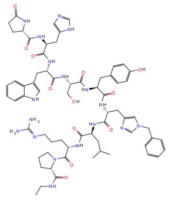	662.3420	662.3409	-1.68
MISMATCH	4.3	549.3606	549.3620	2.60	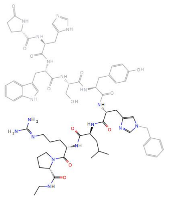	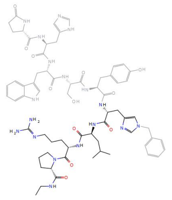	549.3606	549.3620	2.60

Metabolite: Substrate

Type	score	sub. m/z observed	sub. m/z calculated	sub ppm			met. m/z observed	met. m/z calculated	met. ppm
MISMATCH	-13.9	529.7839	529.7822	-3.25	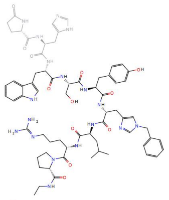	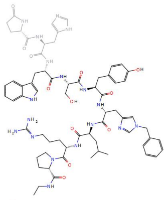	529.7839	529.7822	-3.25
MATCH	6.5	504.1988	504.1990	0.44	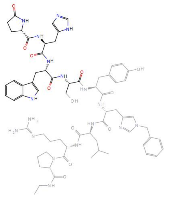	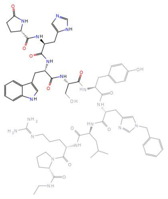	504.1988	504.1990	0.44
MATCH	39.8	441.8973	441.8964	-2.09	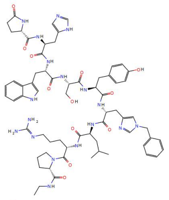	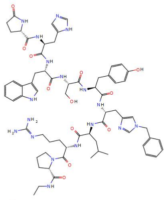	441.8973	441.8964	-2.09
MATCH	200.0	441.8973	441.8964	-2.04	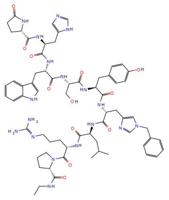	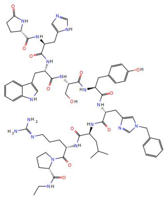	441.8973	441.8964	-2.04
MATCH	34.7	412.3038	412.3031	-1.75	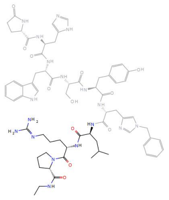	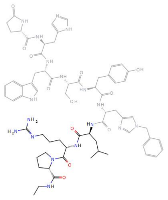	412.3038	412.3031	-1.75
MATCH	30.7	401.7412	401.7398	-3.59	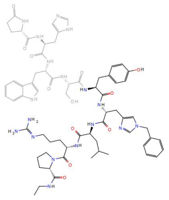	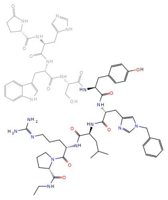	401.7412	401.7398	-3.59
MATCH	10.5	391.1766	391.1765	-0.41	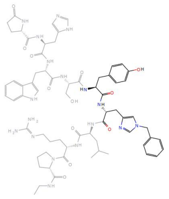	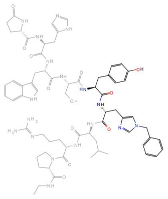	391.1766	391.1765	-0.41
MATCH	10.5	391.1766	391.1765	-0.41	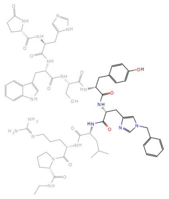	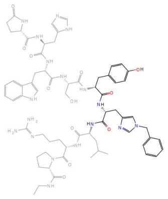	391.1766	391.1765	-0.41
MATCH	3.6	374.1505	374.1499	-1.61	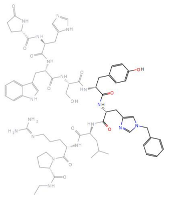	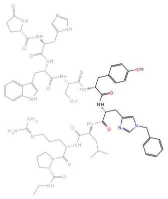	374.1505	374.1499	-1.61

Metabolite: Substrate

Type	score	sub. m/z observed	sub. m/z calculated	sub ppm			met. m/z observed	met. m/z calculated	met. ppm
MATCH	46.4	320.2088	320.2081	-2.24	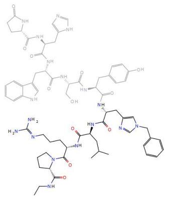	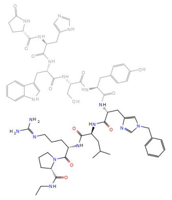	320.2088	320.2081	-2.24
MATCH	4.8	313.2023	313.2023	-0.11	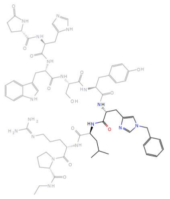	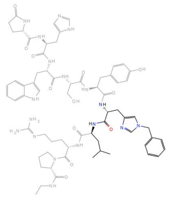	313.2023	313.2023	-0.11
MATCH	16.5	299.2196	299.2190	-2.14	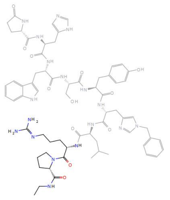	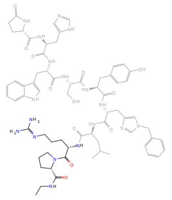	299.2196	299.2190	-2.14
MATCH	10.1	270.1929	270.1925	-1.57	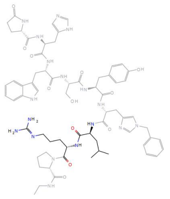	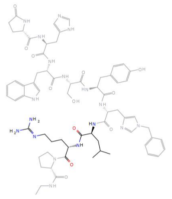	270.1929	270.1925	-1.57
MATCH	4.7	256.1082	256.1081	-0.75	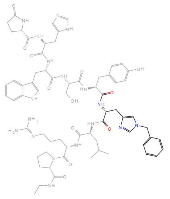	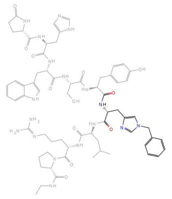	256.1082	256.1081	-0.75
MATCH	29.9	253.1663	253.1659	-1.68	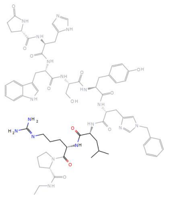	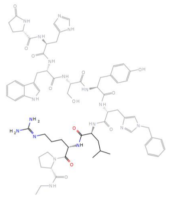	253.1663	253.1659	-1.68
MATCH	150.5	249.0987	249.0982	-1.92	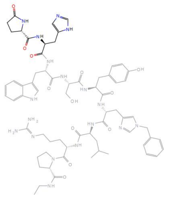	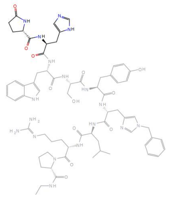	249.0987	249.0982	-1.92
MATCH	181.4	221.1038	221.1033	-2.11	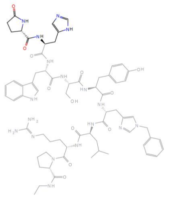	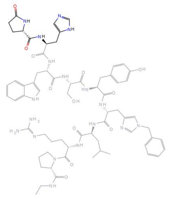	221.1038	221.1033	-2.11
MATCH	119.8	200.1187	200.1182	-2.45	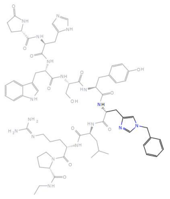	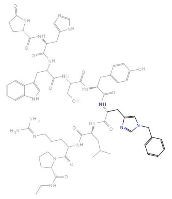	200.1187	200.1182	-2.45

Metabolite: Substrate

Type	score	sub. m/z observed	sub. m/z calculated	sub ppm			met. m/z observed	met. m/z calculated	met. ppm
MATCH	4.0	185.1052	185.1033	-10.1	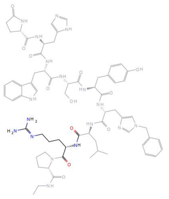	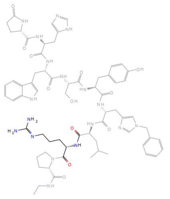	185.1052	185.1033	-10.1
MATCH	4.0	185.1052	185.1073	11.54	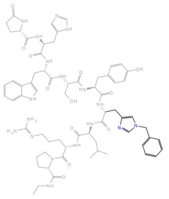	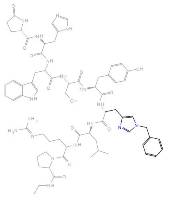	185.1052	185.1073	11.54
MATCH	4.0	185.1052	185.0997	-29.6	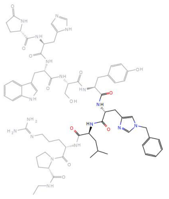	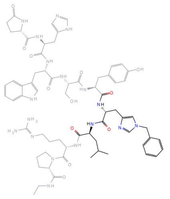	185.1052	185.0997	-29.6
MATCH	13.6	166.0616	166.0611	-3.04	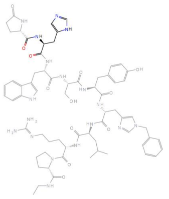	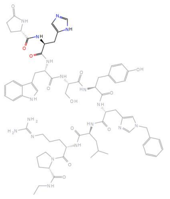	166.0616	166.0611	-3.04
MATCH	57.6	159.0921	159.0917	-2.39	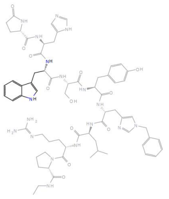	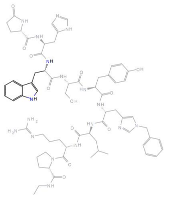	159.0921	159.0917	-2.39
MATCH	4.5	157.1086	157.1084	-1.28	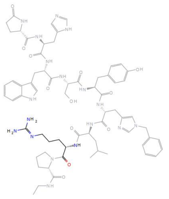	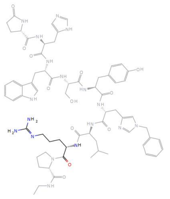	157.1086	157.1084	-1.28
MISMATCH	57.7	143.1183	143.1179	-2.74	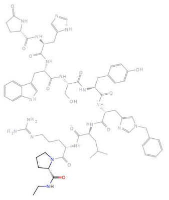	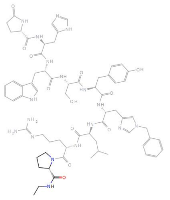	143.1183	143.1179	-2.74
MATCH	32.4	136.0762	136.0757	-3.41	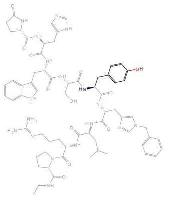	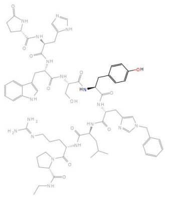	136.0762	136.0757	-3.41
MISMATCH	200.0	110.0719	110.0713	-5.76	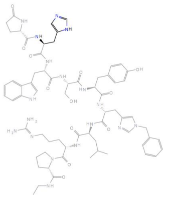	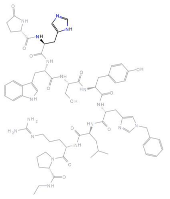	110.0719	110.0713	-5.76

MS (+) FT

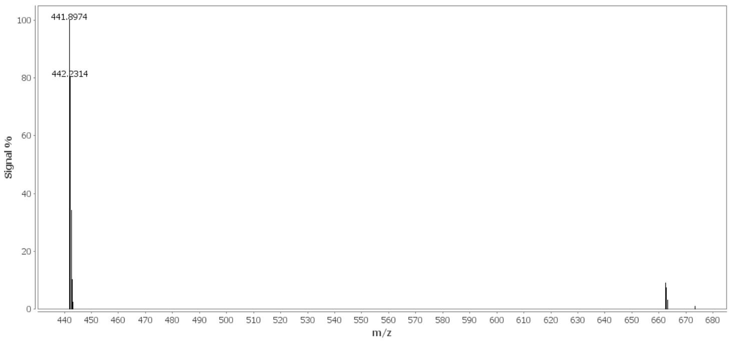

MS (+) FT

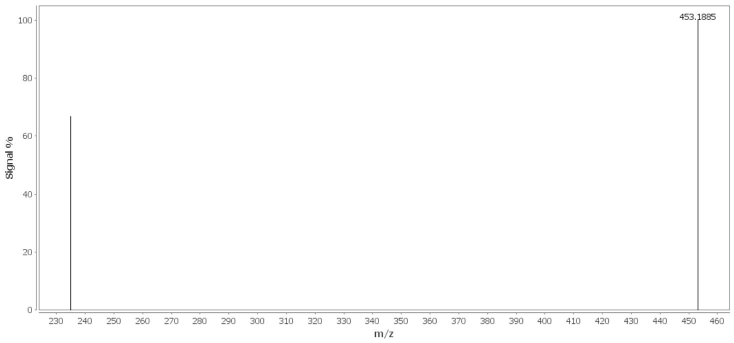

MS2 (+) FT activ = HCD:ce =

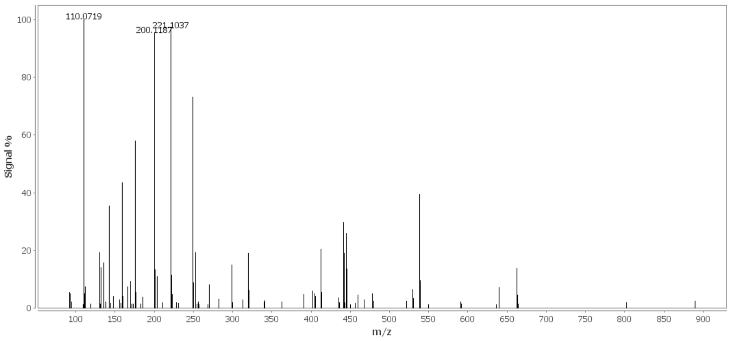

MS2 (+) FT activ = HCD:ce =

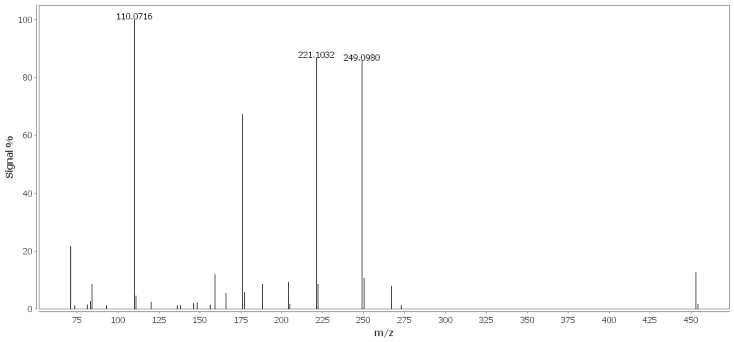

Metabolite: M1 -870 RT=0.46

Type	score	sub. m/z observed	sub. m/z calculated	sub ppm			met. m/z observed	met. m/z calculated	met. ppm
MATCH	200.0	441.8973	441.8964	-2.04	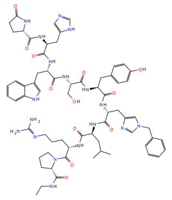	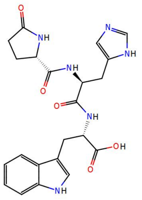	453.1885	453.1881	-0.87
						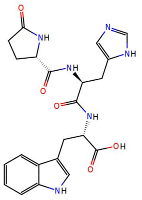	453.1885	453.1881	-0.87
MATCH	108.2	662.3420	662.3409	-1.68	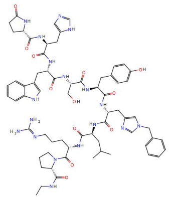	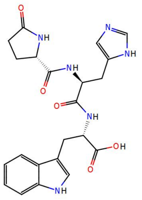	453.1885	453.1881	-0.87
						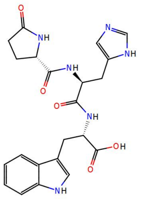	453.1885	453.1881	-0.87
MATCH	200.0	110.0719	110.0713	-5.76	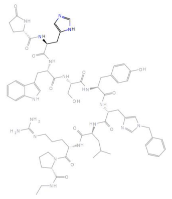	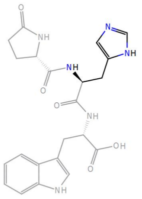	110.0716	110.0713	-3.33

Metabolite: M1 -870 RT=0.46

Type	score	sub. m/z observed	sub. m/z calculated	sub ppm			met. m/z observed	met. m/z calculated	met. ppm
MATCH	57.6	159.0921	159.0917	-2.39	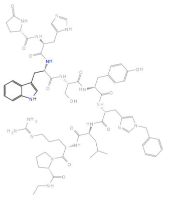	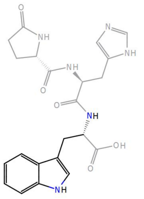	159.0920	159.0917	-2.21
MATCH	13.6	166.0616	166.0611	-3.04	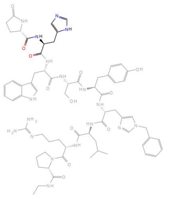	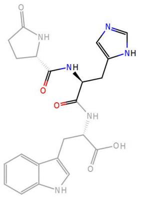	166.0614	166.0611	-1.52
MATCH	181.4	221.1038	221.1033	-2.11	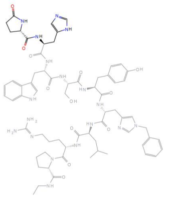	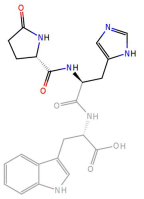	221.1032	221.1033	0.42
MATCH	150.5	249.0987	249.0982	-1.92	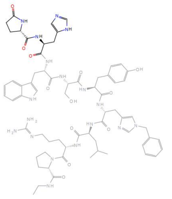	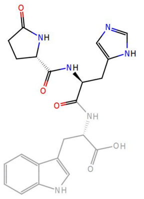	249.0980	249.0982	0.90
MATCH	39.8	441.8973	441.8964	-2.09	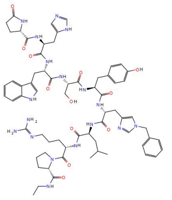	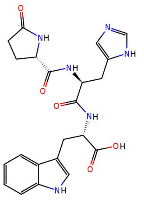	453.1873	453.1881	1.78
						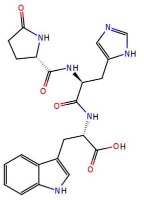	453.1873	453.1881	1.78
MATCH	15.9	662.3424	662.3409	-2.22	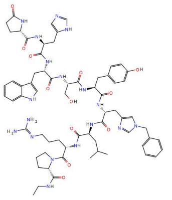	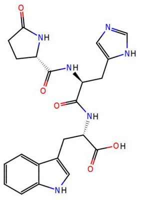	453.1873	453.1881	1.78
						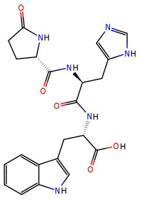	453.1873	453.1881	1.78
MISMATCH	-18.2	136.0762	136.0757	-3.41	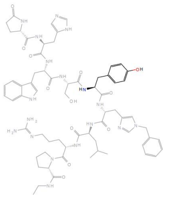		136.0763	136.0763	0.00

Metabolite: M1 -870 RT=0.46

Type	score	sub. m/z observed	sub. m/z calculated	sub ppm		met. m/z observed	met. m/z calculated	met. ppm
MISMATCH	-13.9	529.7839	529.7822	-3.25	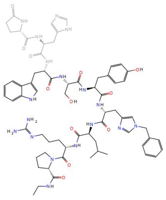	188.0704	188.0704	0.00
MET_MATCH					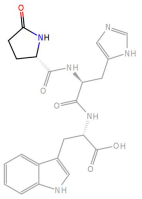	84.0449	84.0444	-6.33
MET_MATCH					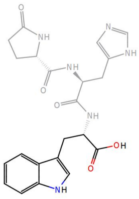	188.0704	188.0706	1.31

MS (+) FT

MS (+) FT

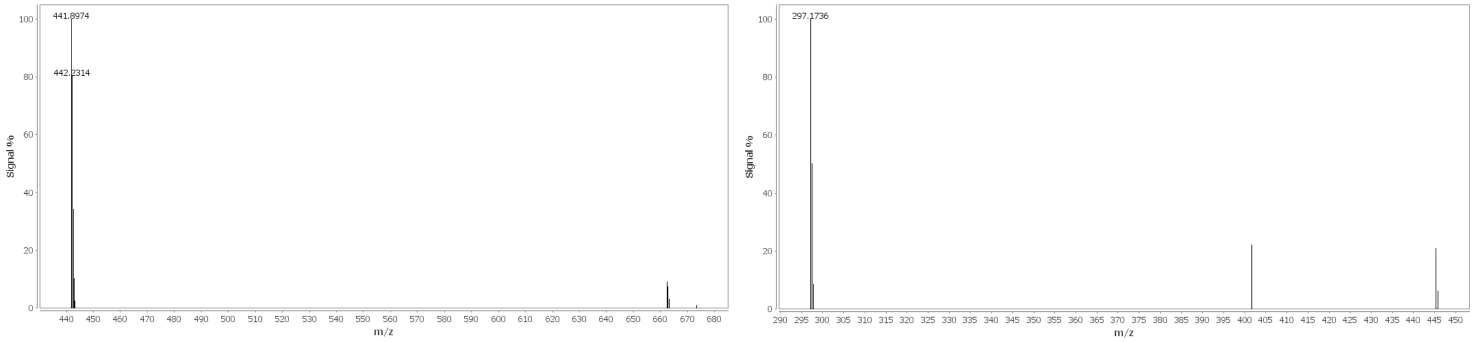

MS2 (+) FT activ = HCD:ce =

MS2 (+) FT activ = HCD:ce =

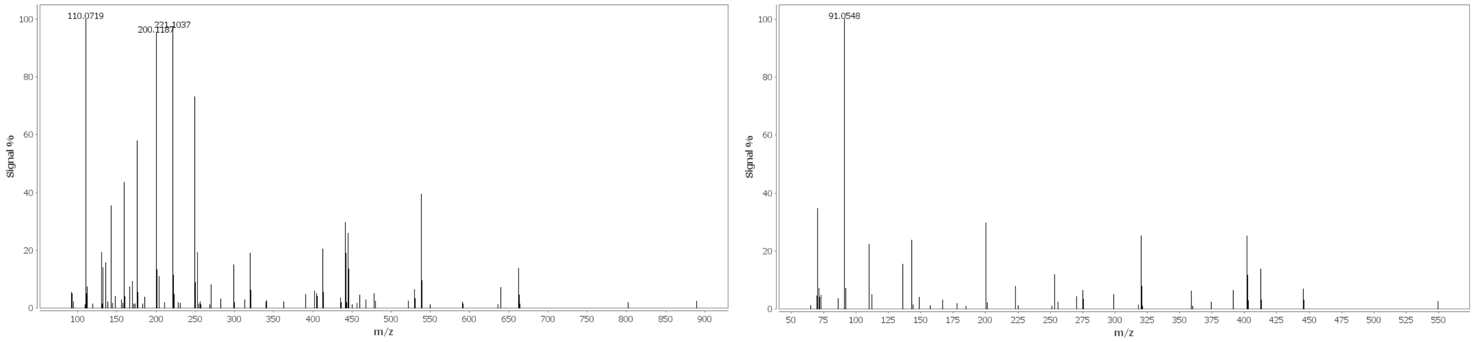

Metabolite: M2 -434 RT=1.16

Type	score	sub. m/z observed	sub. m/z calculated	sub ppm		met. m/z observed	met. m/z calculated	met. ppm
MATCH	200.0	441.8973	441.8964	-2.04	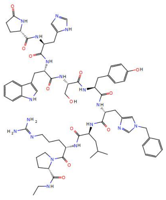 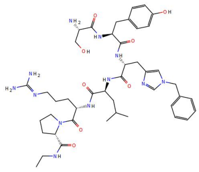	297.1736	297.1729	-2.17

Metabolite: M2 -434 RT=1.16

Type	score	sub. m/z observed	sub. m/z calculated	sub ppm		met. m/z observed	met. m/z calculated	met. ppm
MATCH	200.0	441.8973	441.8964	-2.04	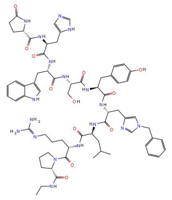 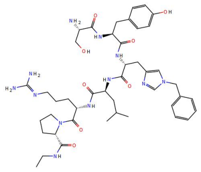	297.1736	297.1729	-2.17
MATCH	120.8	441.8973	441.8964	-2.04	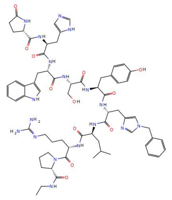 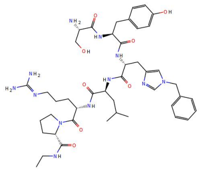	445.2563	445.2558	-1.21
MATCH	120.8	441.8973	441.8964	-2.04	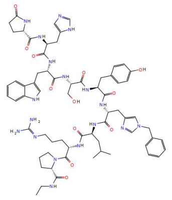 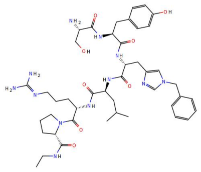	445.2563	445.2558	-1.21
MATCH	108.2	662.3420	662.3409	-1.68	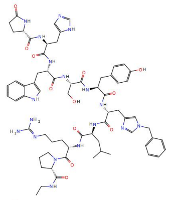 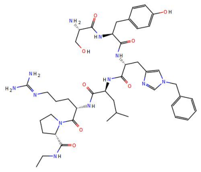	297.1736	297.1729	-2.17
MATCH	108.2	662.3420	662.3409	-1.68	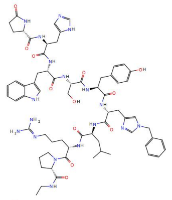 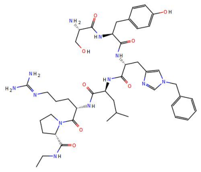	297.1736	297.1729	-2.17
MATCH	29.0	662.3420	662.3409	-1.68	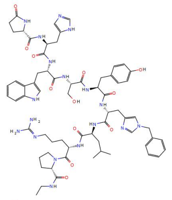 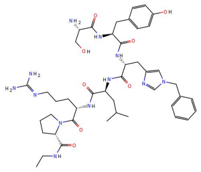	445.2563	445.2558	-1.21
MATCH	29.0	662.3420	662.3409	-1.68	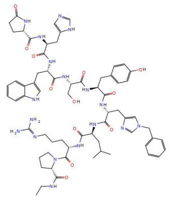 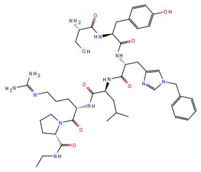	445.2563	445.2558	-1.21
MATCH	32.4	136.0762	136.0757	-3.41	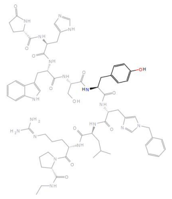 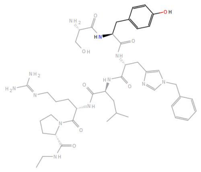	136.0758	136.0757	-0.92
MATCH	57.7	143.1183	143.1179	-2.74	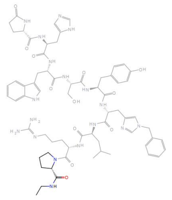 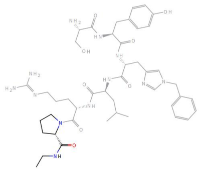	143.1180	143.1179	-0.63

Metabolite: M2 -434 RT=1.16

Type	score	sub. m/z observed	sub. m/z calculated	sub ppm			met. m/z observed	met. m/z calculated	met. ppm
MATCH	4.5	157.1086	157.1084	-1.28	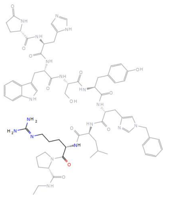	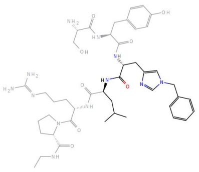	157.1086	157.1048	-24.3
						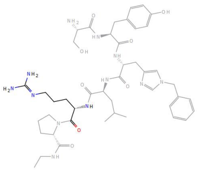	157.1086	157.1084	-1.36
MATCH	4.0	185.1052	185.0997	-29.6	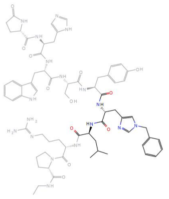	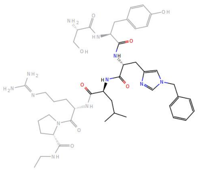	185.1043	185.0997	-25.0
MATCH	4.0	185.1052	185.1033	-10.1	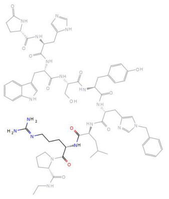	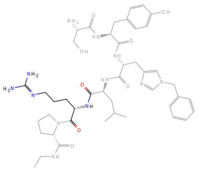	185.1043	185.1033	-5.55
MATCH	4.0	185.1052	185.1073	11.54	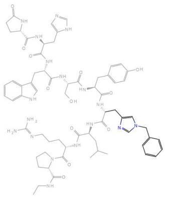	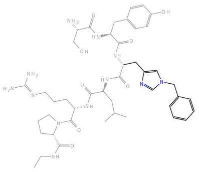	185.1043	185.1073	16.19
MATCH	119.8	200.1187	200.1182	-2.45	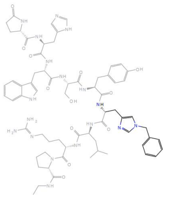	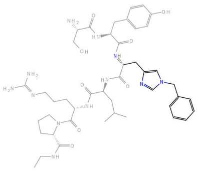	200.1183	200.1182	-0.15
MATCH	29.9	253.1663	253.1659	-1.68	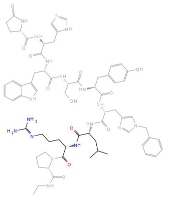	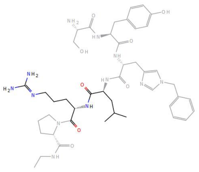	253.1658	253.1659	0.56
MATCH	4.7	256.1082	256.1081	-0.75	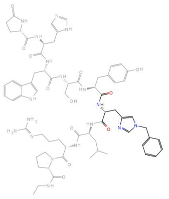	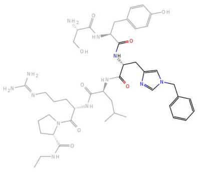	256.1077	256.1081	1.26
MATCH	10.1	270.1929	270.1925	-1.57	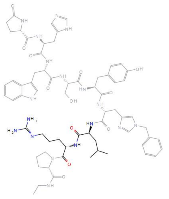	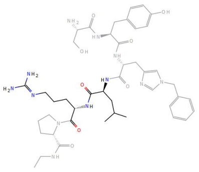	270.1924	270.1925	0.05

Metabolite: M2 -434 RT=1.16

Type	score	sub. m/z observed	sub. m/z calculated	sub ppm			met. m/z observed	met. m/z calculated	met. ppm
MATCH	16.5	299.2196	299.2190	-2.14	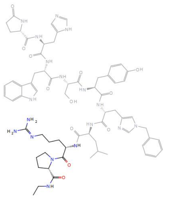	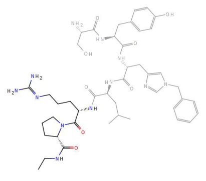	299.2193	299.2190	-0.90
MATCH	4.8	313.2023	313.2023	-0.11	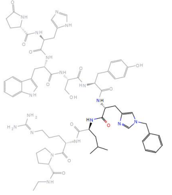	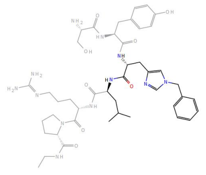	157.1086	157.1048	-24.3
						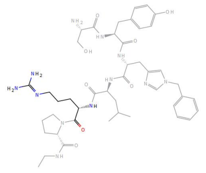	157.1086	157.1084	-1.36
MATCH	46.4	320.2088	320.2081	-2.24	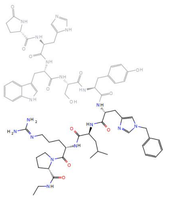	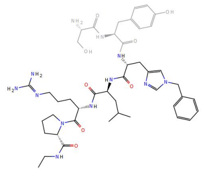	320.2081	320.2081	0.05
MATCH	3.6	374.1505	374.1499	-1.61	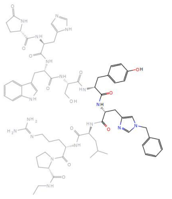	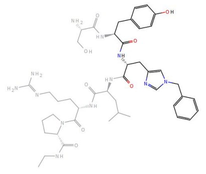	374.1496	374.1499	0.89
MATCH	10.5	391.1766	391.1765	-0.41	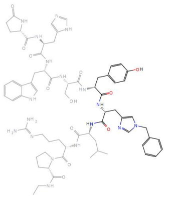	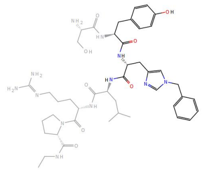	391.1769	391.1765	-1.00
MATCH	10.5	391.1766	391.1765	-0.41	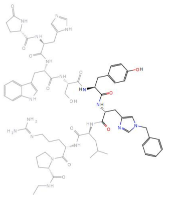	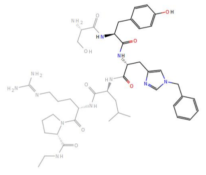	391.1769	391.1765	-1.00
MATCH	30.7	401.7412	401.7398	-3.59	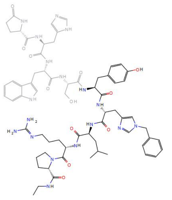	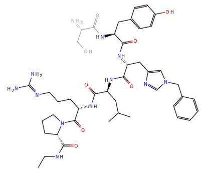	401.7396	401.7398	0.37
MATCH	34.7	412.3038	412.3031	-1.75	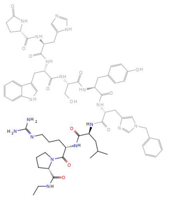	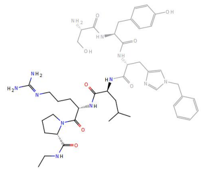	412.3026	412.3031	1.17

Metabolite: M2 -434 RT=1.16

Type	score	sub. m/z observed	sub. m/z calculated	sub ppm			met. m/z observed	met. m/z calculated	met. ppm
MATCH	34.2	441.8973	441.8964	-2.09	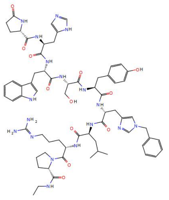	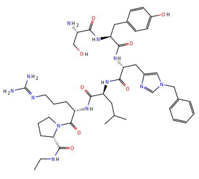	445.2561	445.2558	-0.82
						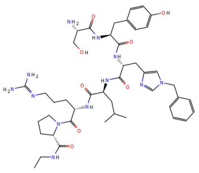	445.2561	445.2558	-0.82
MATCH	6.5	504.1988	504.1990	0.44	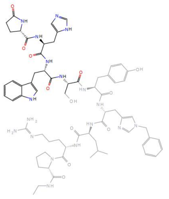	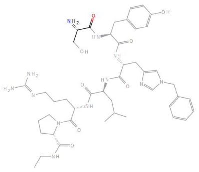	70.0295	70.0287	-11.4
MATCH	4.3	549.3606	549.3620	2.60	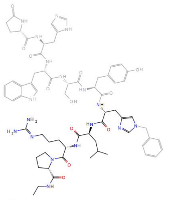	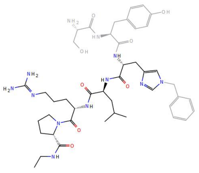	549.3609	549.3620	1.94
MATCH	10.3	662.3424	662.3409	-2.22	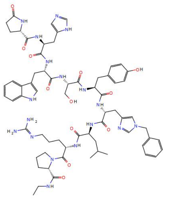	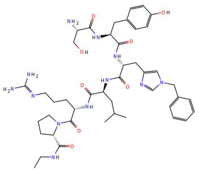	445.2561	445.2558	-0.82
						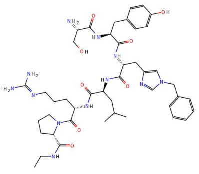	445.2561	445.2558	-0.82
MISMATCH	-122.3	110.0719	110.0713	-5.76	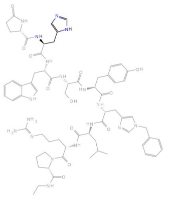		110.0717	110.0717	0.00
MISMATCH	-38.1	143.1183	143.1179	-2.74	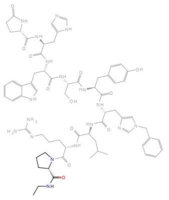		72.0690	72.0690	0.00
MISMATCH	-8.1	549.3606	549.3620	2.60	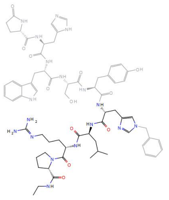		275.1846	275.1846	0.00

Metabolite: M2 -434 RT=1.16

Type	score	sub. m/z observed	sub. m/z calculated	sub ppm		met. m/z observed	met. m/z calculated	met. ppm
MET_MATCH					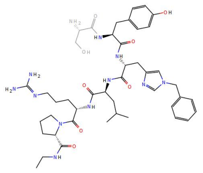	401.7406	401.7398	-2.03
MET_MATCH					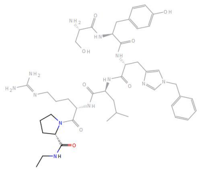	72.0690	72.0626	-89.4
MET_MATCH					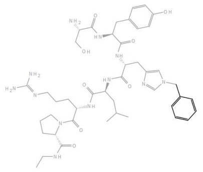	91.0548	91.0542	-5.84
MET_MATCH					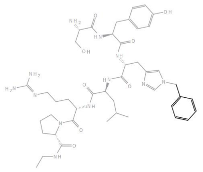	91.0584	91.0542	-45.4
MET_MATCH					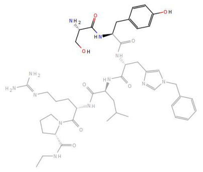	223.1075	223.1077	1.15
