# Supplementary material for: Software-aided approach to investigate peptide structure and metabolic susceptibility of amide bonds in peptide drugs based on high resolution mass spectrometry
Source: PLoS One. 2017 Nov 1;12(11):e0186461. doi: 10.1371/journal.pone.0186461 (PMC5665424; doi:10.1371/journal.pone.0186461)
Supplement: S1 File — (ZIP) [file pone.0186461.s007.zip › SFiles/S1_File.pdf]

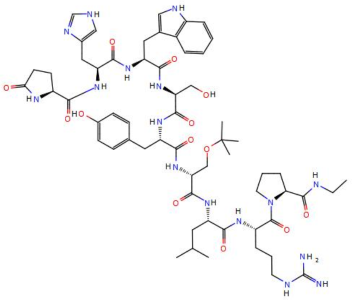

Buserelin

Property name	Property value
Time	0min, 5min, 15min, 45min, 120min
Instrument	ThermoQAPlus
Matrix	chymotrypsin
Acquisition Mode	ddMS2

Chromatograms

Time=0min

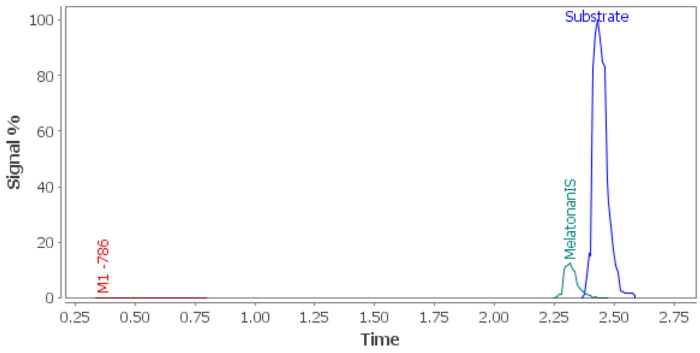

Time=5min

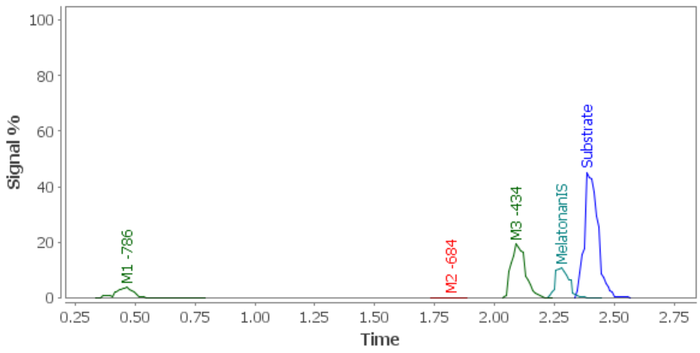

Time=15min

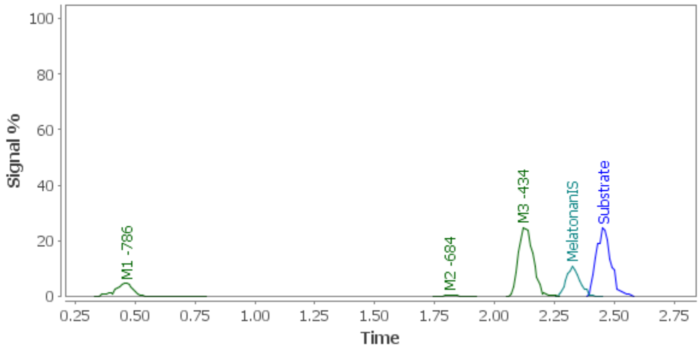

Time=45min

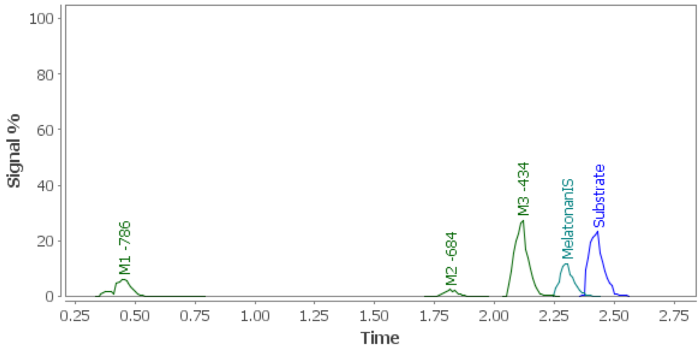

Time=120min

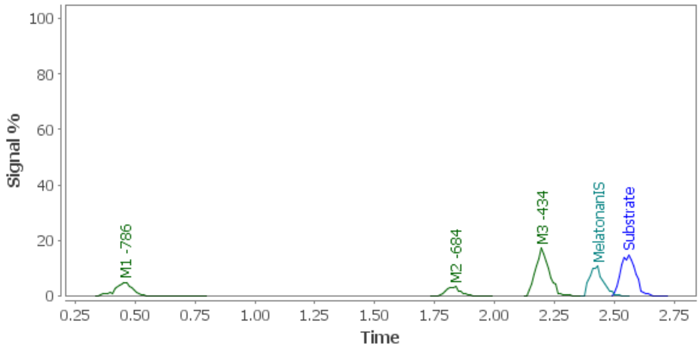

Custom Charts

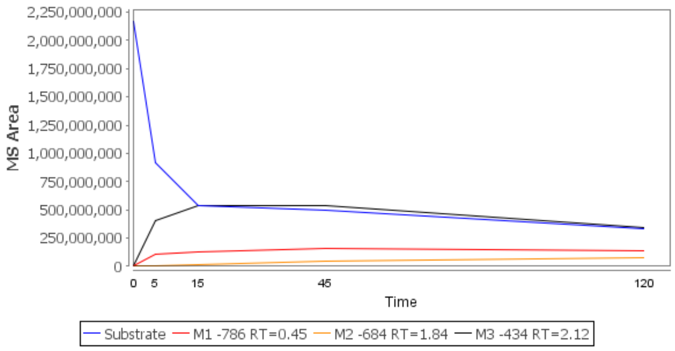

Fragmentation

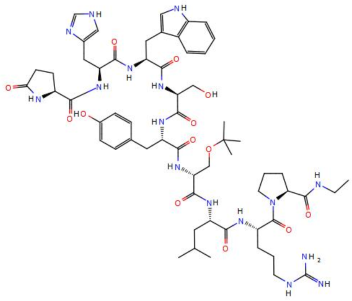

Buserelin

MS (+) FT

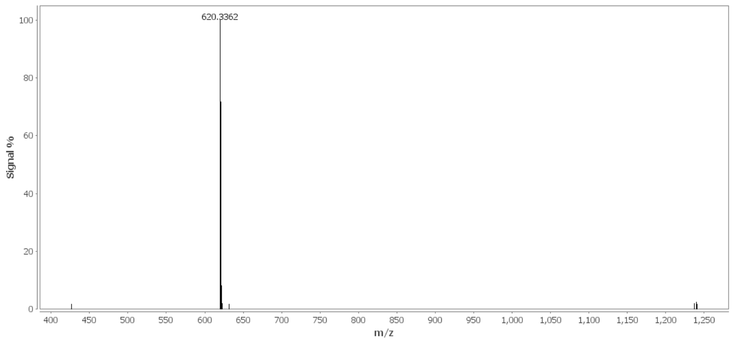

MS (+) FT

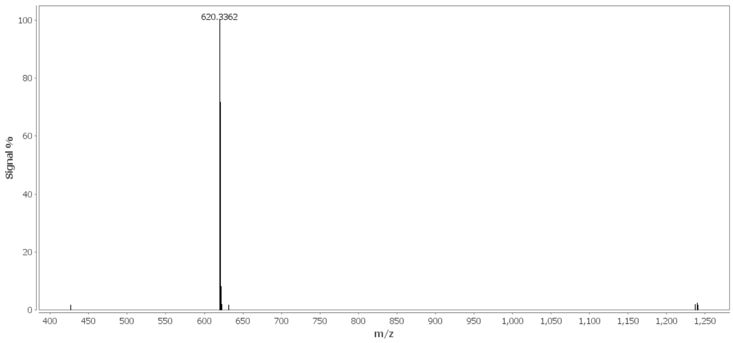

MS2 (+) FT activ = HCD:ce =

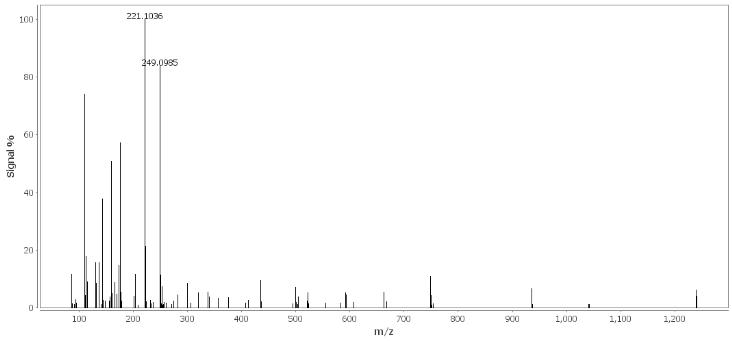

MS2 (+) FT activ = HCD:ce =

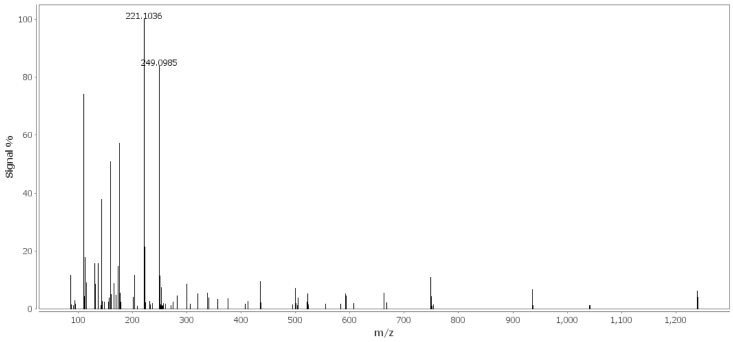

Metabolite: Substrate

Type	score	sub. m/z observed	sub. m/z calculated	sub ppm			met. m/z observed	met. m/z calculated	met. ppm
MATCH	102.4	1239.6668	1239.6633	-2.86	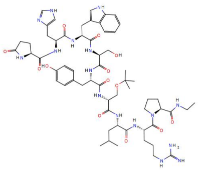	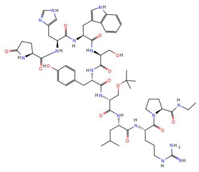	1239.6668	1239.6633	-2.86
MATCH	17.1	1239.6657	1239.6633	-1.95	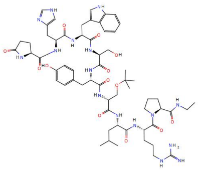	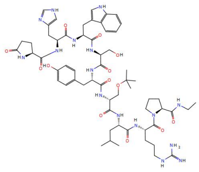	1239.6657	1239.6633	-1.95
MATCH	5.8	754.2883	754.2944	7.98	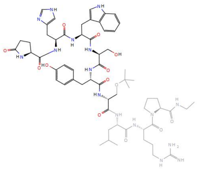	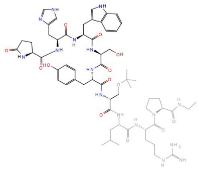	754.2883	754.2944	7.98

Metabolite: Substrate

Type	score	sub. m/z observed	sub. m/z calculated	sub ppm			met. m/z observed	met. m/z calculated	met. ppm
MATCH	200.0	620.3362	620.3353	-1.47	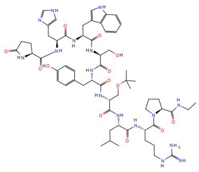	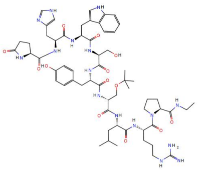	620.3362	620.3353	-1.47
MISMATCH	10.3	592.3055	592.3040	-2.57	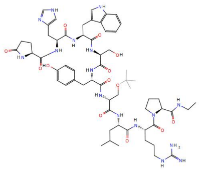	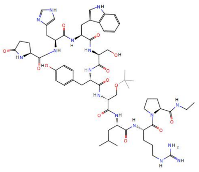	592.3055	592.3040	-2.57
MATCH	6.0	583.2991	583.2987	-0.68	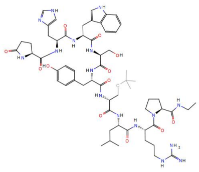	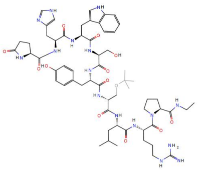	583.2991	583.2987	-0.68
MATCH	3.1	555.3963	555.3977	2.50	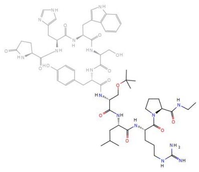	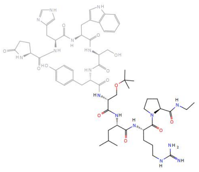	555.3963	555.3977	2.50
MATCH	9.1	521.2445	521.2487	8.02	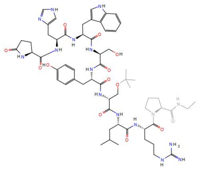	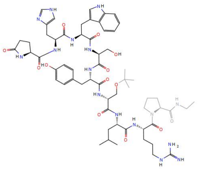	521.2445	521.2487	8.02
MATCH	6.9	504.2000	504.1990	-1.97	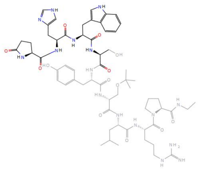	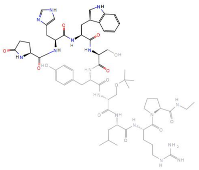	504.2000	504.1990	-1.97
MATCH	46.8	499.3351	499.3351	-0.03	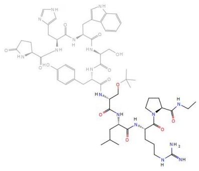	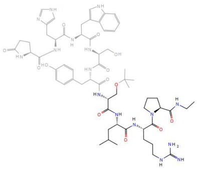	499.3351	499.3351	-0.03
MATCH	20.9	494.2126	494.2146	4.15	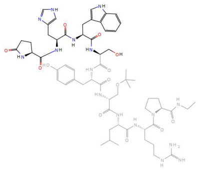	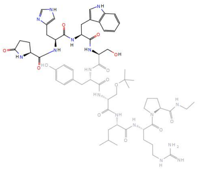	494.2126	494.2146	4.15
MATCH	14.9	412.3019	412.3031	2.73	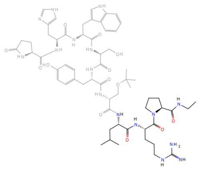	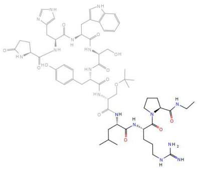	412.3019	412.3031	2.73

Metabolite: Substrate

Type	score	sub. m/z observed	sub. m/z calculated	sub ppm			met. m/z observed	met. m/z calculated	met. ppm
MATCH	52.8	299.2194	299.2190	-1.29	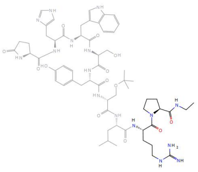	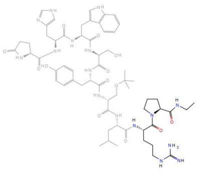	299.2194	299.2190	-1.29
MATCH	9.0	282.1927	282.1925	-0.86	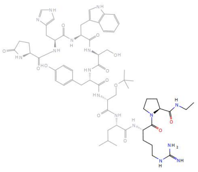	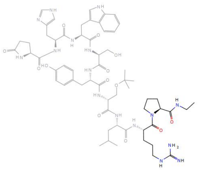	282.1927	282.1925	-0.86
MATCH	3.6	270.1922	270.1925	1.05	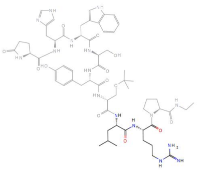	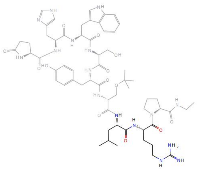	270.1922	270.1925	1.05
MATCH	19.6	261.1132	261.1164	12.32	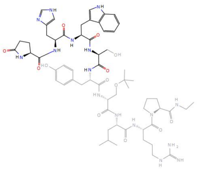	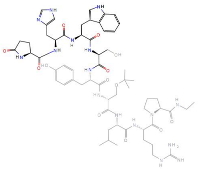	261.1132	261.1164	12.32
MATCH	19.6	253.1663	253.1659	-1.57	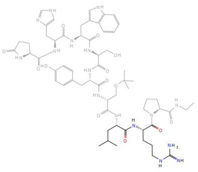	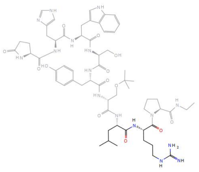	253.1663	253.1659	-1.57
MATCH	161.4	249.0985	249.0982	-1.14	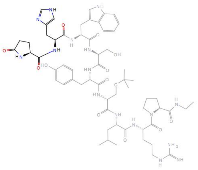	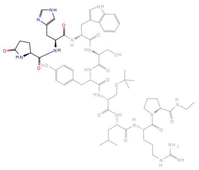	249.0985	249.0982	-1.14
MATCH	3.8	237.1351	237.1346	-2.17	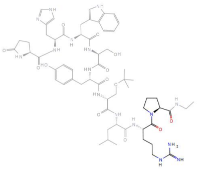	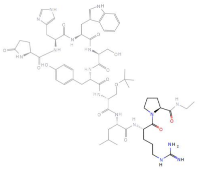	237.1351	237.1346	-2.17
MATCH	180.3	221.1036	221.1033	-1.54	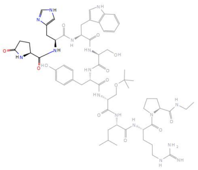	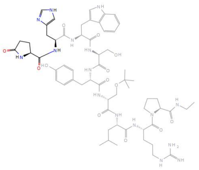	221.1036	221.1033	-1.54
MATCH	15.8	166.0614	166.0611	-1.84	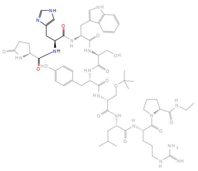	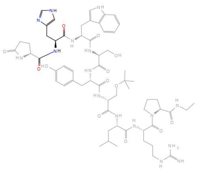	166.0614	166.0611	-1.84

Metabolite: Substrate

Type	score	sub. m/z observed	sub. m/z calculated	sub ppm			met. m/z observed	met. m/z calculated	met. ppm
MATCH	62.3	159.0920	159.0917	-1.73	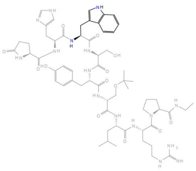	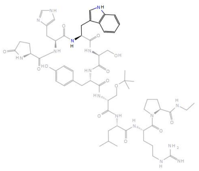	159.0920	159.0917	-1.73
MATCH	8.8	157.1087	157.1084	-2.26	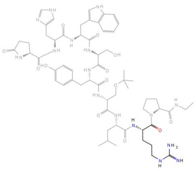	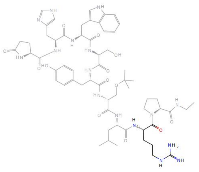	157.1087	157.1084	-2.26
MATCH	75.8	143.1182	143.1179	-2.15	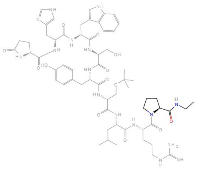	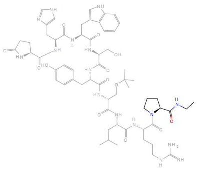	143.1182	143.1179	-2.15
MATCH	79.1	136.0760	136.0757	-2.50	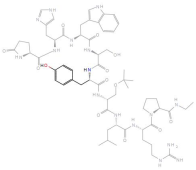	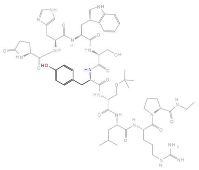	136.0760	136.0757	-2.50
MATCH	22.1	115.0871	115.0866	-4.13	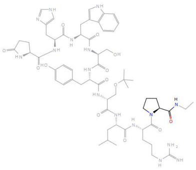	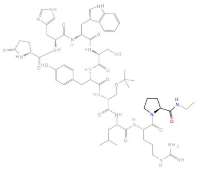	115.0871	115.0866	-4.13
MATCH	35.8	112.0875	112.0869	-4.95	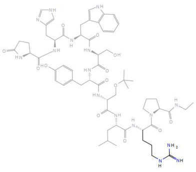	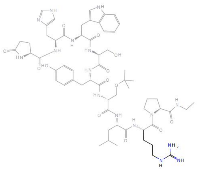	112.0875	112.0869	-4.95
MATCH	174.0	110.0719	110.0713	-5.25	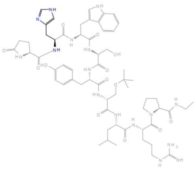	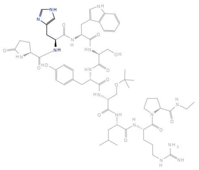	110.0719	110.0713	-5.25
MATCH	4.7	95.0611	95.0604	-7.32	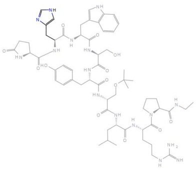	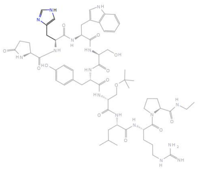	95.0611	95.0604	-7.32
MATCH	4.6	91.0550	91.0522	-30.0	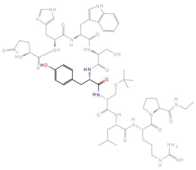	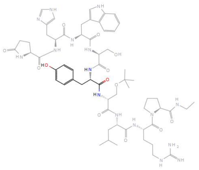	91.0550	91.0522	-30.0

Metabolite: Substrate

Type	score	sub. m/z observed	sub. m/z calculated	sub ppm			met. m/z observed	met. m/z calculated	met. ppm
MATCH	53.9	86.0973	86.0964	-9.80	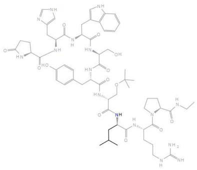	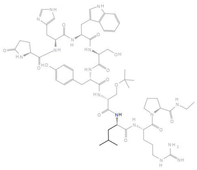	86.0973	86.0964	-9.80

MS (+) FT

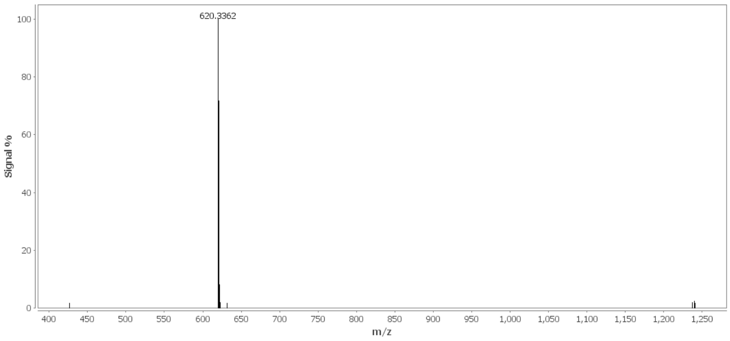

MS (+) FT

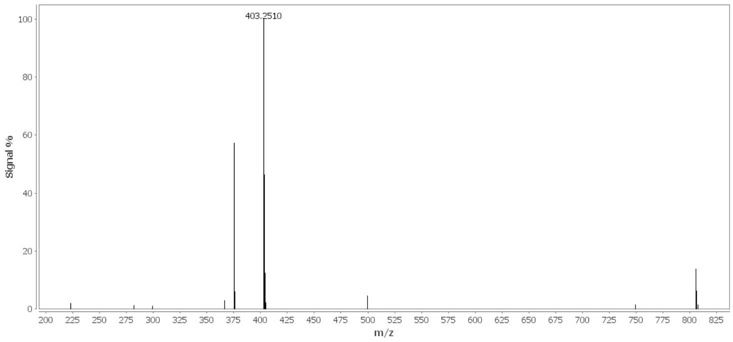

MS2 (+) FT activ = HCD:ce =

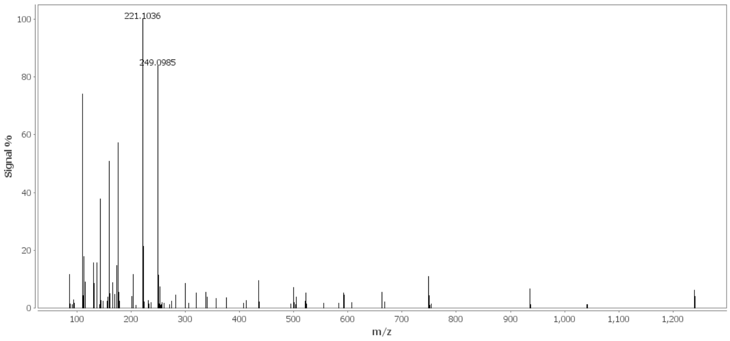

MS2 (+) FT activ = HCD:ce =

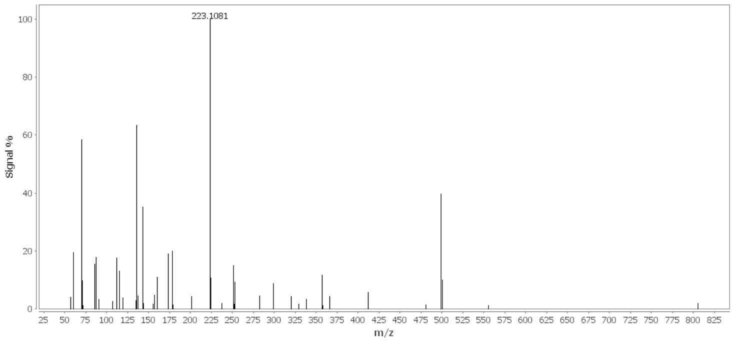

Metabolite: M3 -434 RT=2.12

Type	score	sub. m/z observed	sub. m/z calculated	sub ppm			met. m/z observed	met. m/z calculated	met. ppm
MATCH	200.0	620.3362	620.3353	-1.47	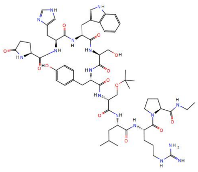	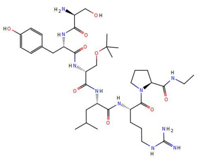	403.2510	403.2502	-2.05
MATCH	200.0	620.3362	620.3353	-1.47	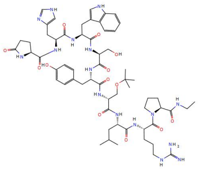	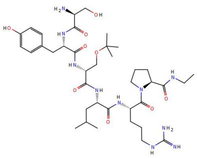	403.2510	403.2502	-2.05
MATCH	113.7	620.3362	620.3353	-1.47	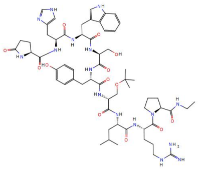	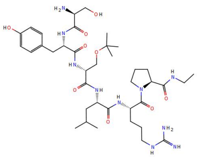	805.4944	805.4930	-1.63

Metabolite: M3 -434 RT=2.12

Type	score	sub. m/z observed	sub. m/z calculated	sub ppm			met. m/z observed	met. m/z calculated	met. ppm
MATCH	113.7	620.3362	620.3353	-1.47	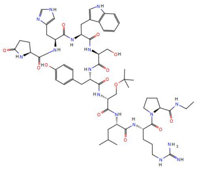	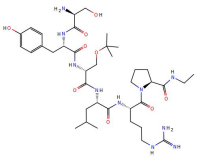	805.4944	805.4930	-1.63
MATCH	102.4	1239.6668	1239.6633	-2.86	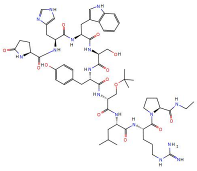	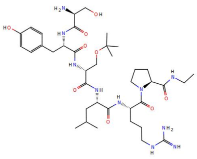	403.2510	403.2502	-2.05
MATCH	102.4	1239.6668	1239.6633	-2.86	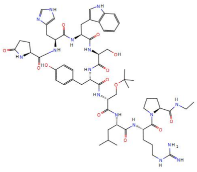	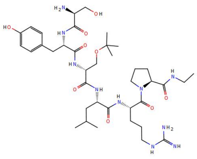	403.2510	403.2502	-2.05
MATCH	16.1	1239.6668	1239.6633	-2.86	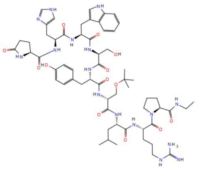	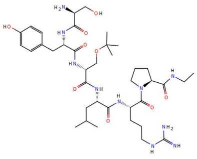	805.4944	805.4930	-1.63
MATCH	16.1	1239.6668	1239.6633	-2.86	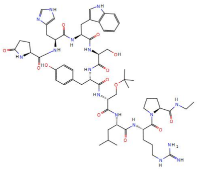	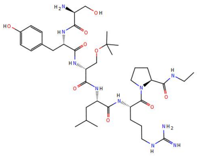	805.4944	805.4930	-1.63
MATCH	27.1	86.0973	86.0964	-9.80	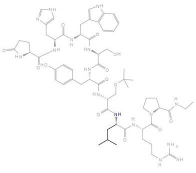	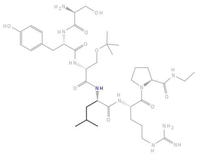	86.0972	86.0964	-9.51
MATCH	4.6	91.0550	91.0522	-30.0	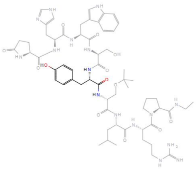	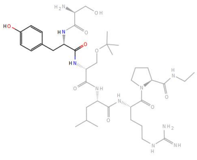	91.0550	91.0522	-30.7
MATCH	35.5	112.0875	112.0869	-4.95	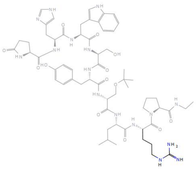	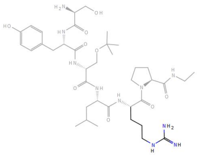	112.0875	112.0869	-4.98
MATCH	22.1	115.0871	115.0866	-4.13	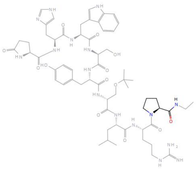	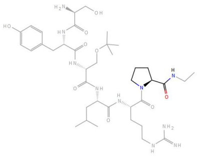	115.0872	115.0866	-4.89

Metabolite: M3 -434 RT=2.12

Type	score	sub. m/z observed	sub. m/z calculated	sub ppm			met. m/z observed	met. m/z calculated	met. ppm
MATCH	79.1	136.0760	136.0757	-2.50	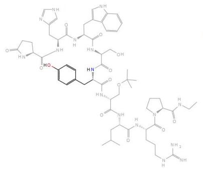	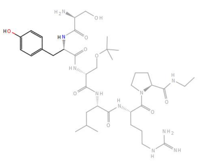	136.0760	136.0757	-2.59
MATCH	72.8	143.1182	143.1179	-2.15	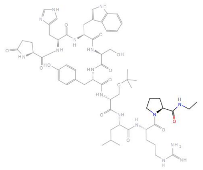	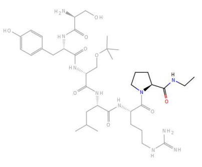	143.1182	143.1179	-2.38
MATCH	8.8	157.1087	157.1084	-2.26	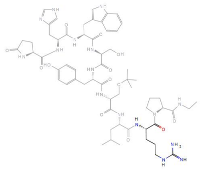	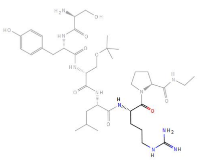	157.1088	157.1084	-2.61
MATCH	3.8	237.1351	237.1346	-2.17	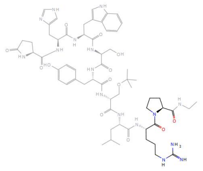	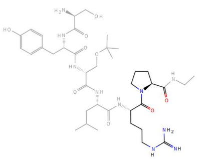	237.1348	237.1346	-0.96
MATCH	16.6	253.1663	253.1659	-1.57	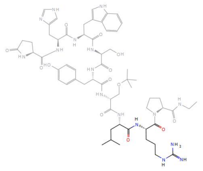	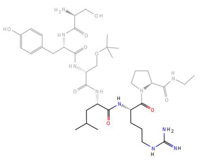	253.1665	253.1659	-2.48
MATCH	19.6	261.1132	261.1164	12.32	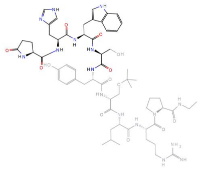	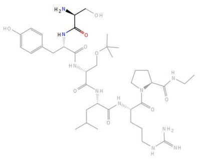	87.0561	87.0553	-8.96
MATCH	9.0	282.1927	282.1925	-0.86	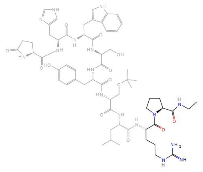	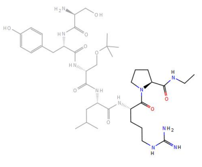	282.1927	282.1925	-0.73
MATCH	17.6	299.2194	299.2190	-1.29	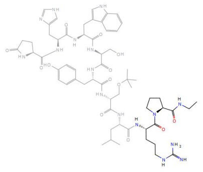	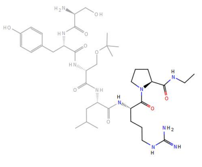	299.2195	299.2190	-1.53
MATCH	8.4	412.3019	412.3031	2.73	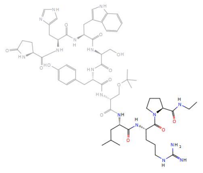	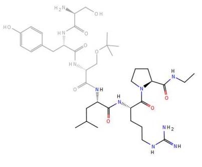	412.3043	412.3031	-3.05

Metabolite: M3 -434 RT=2.12

Type	score	sub. m/z observed	sub. m/z calculated	sub ppm			met. m/z observed	met. m/z calculated	met. ppm
MATCH	20.9	494.2126	494.2146	4.15	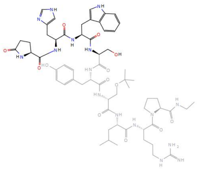	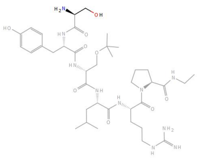	60.0454	60.0444	-16.2
MATCH	46.8	499.3351	499.3351	-0.03	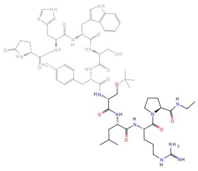	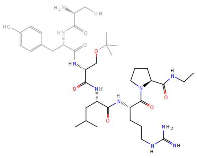	499.3358	499.3351	-1.42
MATCH	6.9	504.2000	504.1990	-1.97	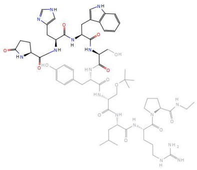	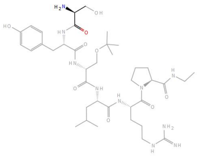	70.0298	70.0287	-14.4
MATCH	3.1	555.3963	555.3977	2.50	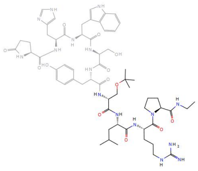	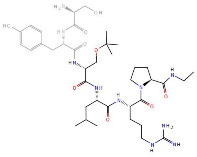	555.3978	555.3977	-0.16
MATCH	6.0	583.2991	583.2987	-0.68	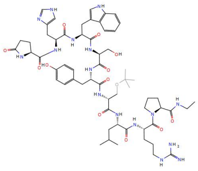	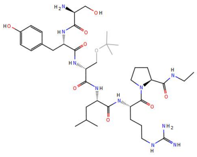	366.2147	366.2136	-3.03
MATCH	5.8	754.2883	754.2944	7.98	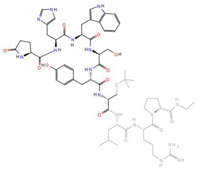	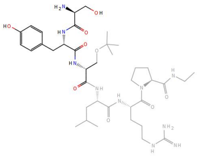	320.1244	320.1241	-1.00
MATCH	8.2	1239.6657	1239.6633	-1.95	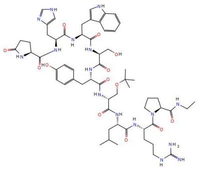	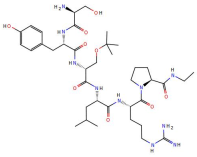	805.4946	805.4930	-1.90
MET_MATCH						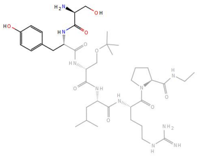	223.1081	223.1077	-1.82
MET_MATCH						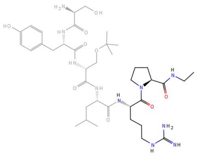	299.2195	299.2190	-1.75

Metabolite: M3 -434 RT=2.12

Type	score	sub. m/z observed	sub. m/z calculated	sub ppm		met. m/z observed	met. m/z calculated	met. ppm
MET_MATCH					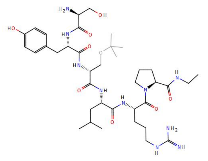	366.2143	366.2136	-1.99
MET_MATCH					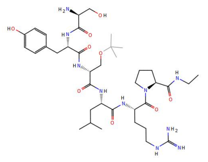	375.2195	375.2189	-1.70
MET_MATCH					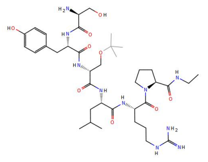	749.4326	749.4304	-2.81
MET_MATCH					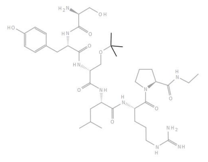	57.0709	57.0699	-18.1
MET_MATCH					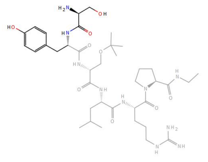	223.1081	223.1077	-1.56
MET_MATCH					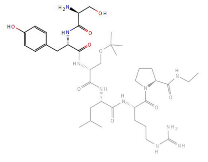	251.1033	251.1026	-2.68

MS (+) FT

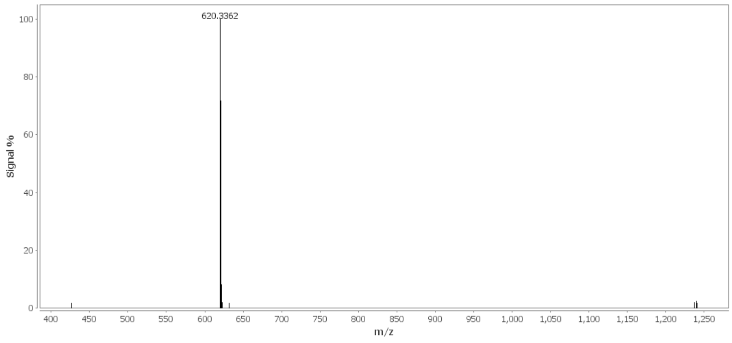

MS (+) FT

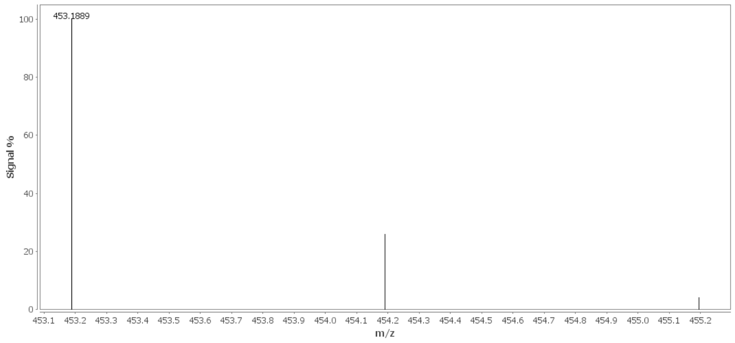

MS2 (+) FT activ = HCD:ce =

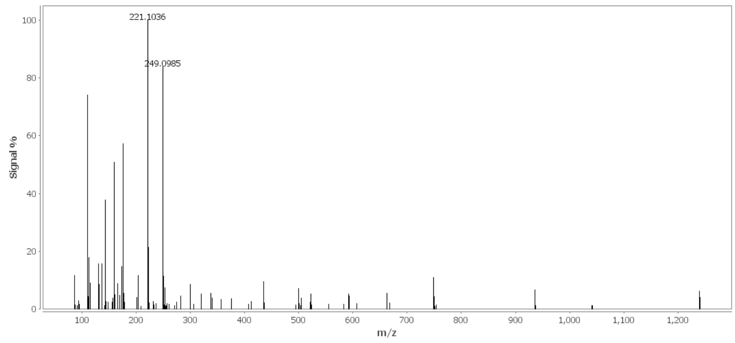

MS2 (+) FT activ = HCD:ce =

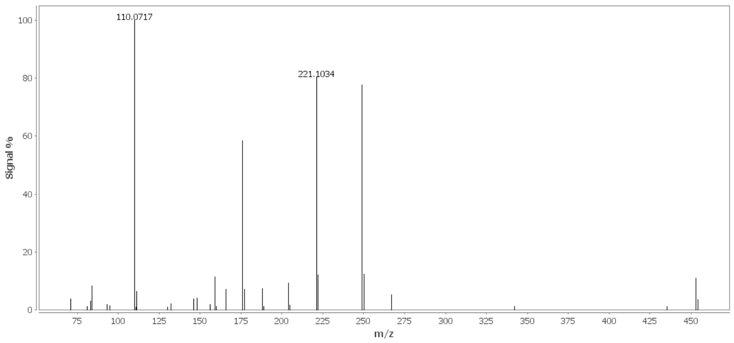

Metabolite: M1 -786 RT=0.45

Type	score	sub. m/z observed	sub. m/z calculated	sub ppm		met. m/z observed	met. m/z calculated	met. ppm
MATCH	200.0	620.3362	620.3353	-1.47	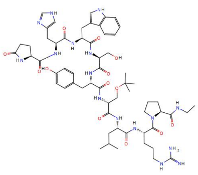 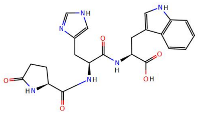	453.1889	453.1881	-1.70
					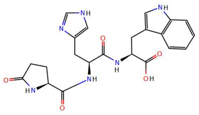	453.1889	453.1881	-1.70
MATCH	102.4	1239.6668	1239.6633	-2.86	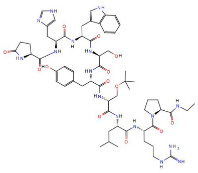 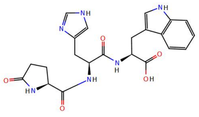	453.1889	453.1881	-1.70
					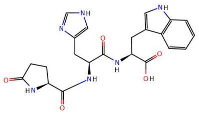	453.1889	453.1881	-1.70
MATCH	4.7	93.0455	93.0447	-8.65	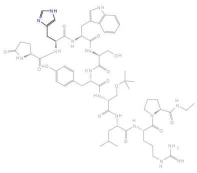 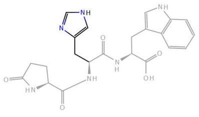	93.0454	93.0447	-7.58
MATCH	3.2	95.0611	95.0604	-7.32	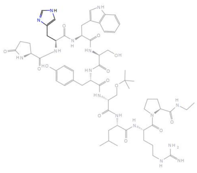 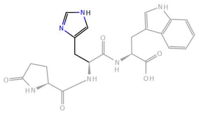	95.0610	95.0604	-6.13
MATCH	174.0	110.0719	110.0713	-5.25	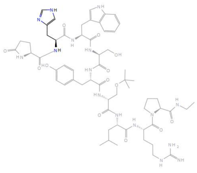 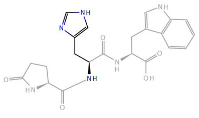	110.0717	110.0713	-4.29

Metabolite: M1 -786 RT=0.45

Type	score	sub. m/z observed	sub. m/z calculated	sub ppm		met. m/z observed	met. m/z calculated	met. ppm
MATCH	62.3	159.0920	159.0917	-1.73	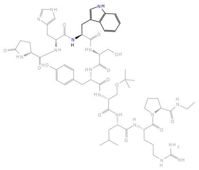 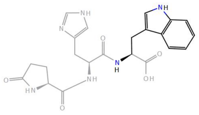	159.0918	159.0917	-0.93
MATCH	15.8	166.0614	166.0611	-1.84	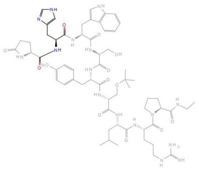 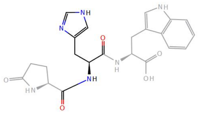	166.0612	166.0611	-0.58
MATCH	180.3	221.1036	221.1033	-1.54	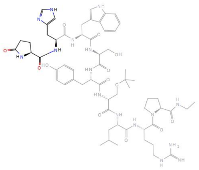 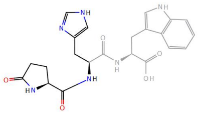	221.1034	221.1033	-0.52
MATCH	161.4	249.0985	249.0982	-1.14	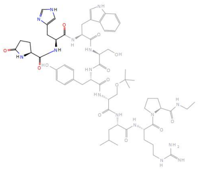 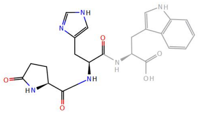	249.0983	249.0982	-0.42
MATCH	17.1	1239.6657	1239.6633	-1.95	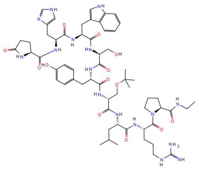 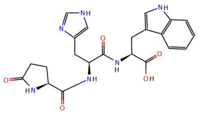	453.1883	453.1881	-0.53
MET_MATCH					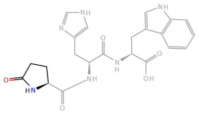	84.0451	84.0444	-8.91
MET_MATCH					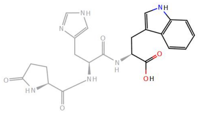	188.0706	188.0706	-0.07
MET_MATCH					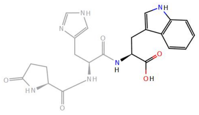	205.0975	205.0972	-1.47
MET_MATCH					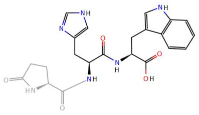	342.1564	342.1561	-0.97

Metabolite: M1 -786 RT=0.45

Type	score	sub. m/z observed	sub. m/z calculated	sub ppm		met. m/z observed	met. m/z calculated	met. ppm
MET_MATCH					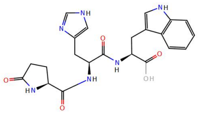	435.1785	435.1775	-2.30

MS (+) FT

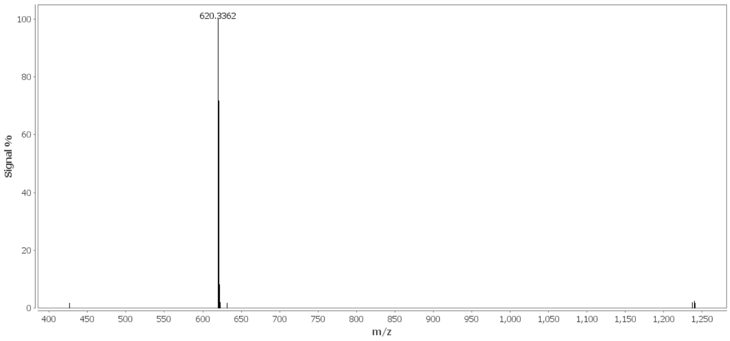

MS (+) FT

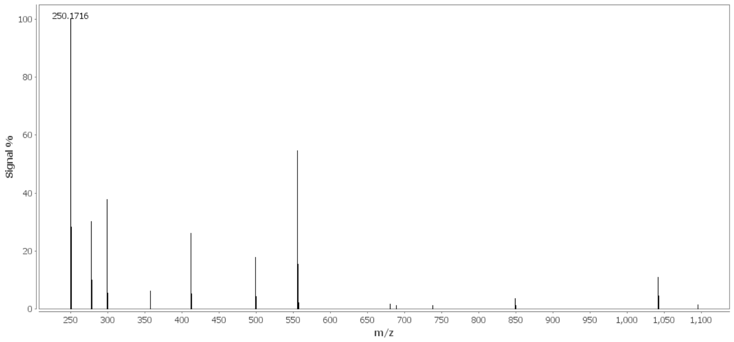

MS2 (+) FT activ = HCD:ce =

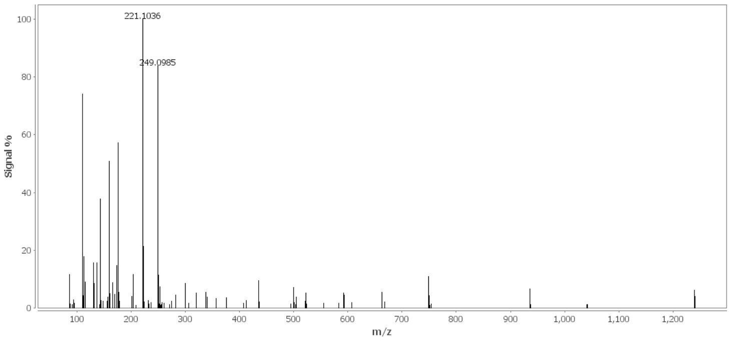

MS2 (+) FT activ = HCD:ce =

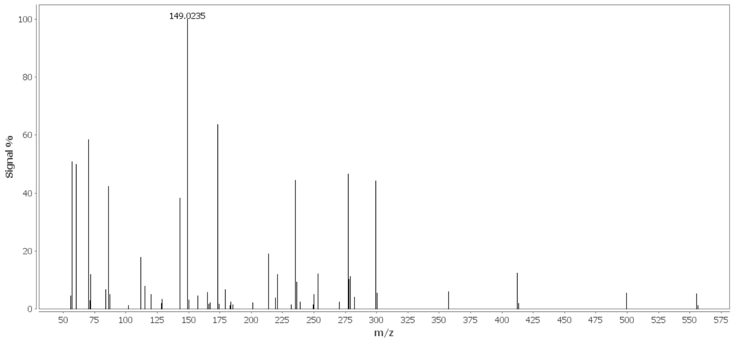

Metabolite: M2 -684 RT=1.84

Type	score	sub. m/z observed	sub. m/z calculated	sub ppm			met. m/z observed	met. m/z calculated	met. ppm
MATCH	130.1	620.3362	620.3353	-1.47	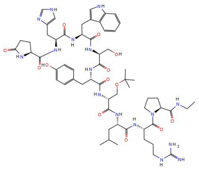	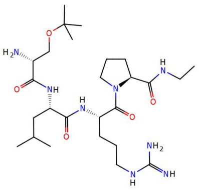	278.2032	278.2025	-2.56
MATCH	130.1	620.3362	620.3353	-1.47	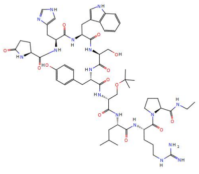	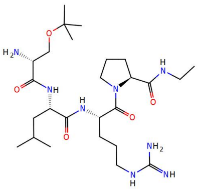	278.2032	278.2025	-2.56
MATCH	154.6	620.3362	620.3353	-1.47	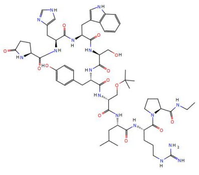	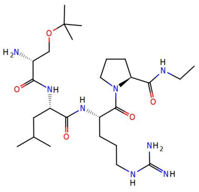	555.3988	555.3977	-2.01

Metabolite: M2 -684 RT=1.84

Type	score	sub. m/z observed	sub. m/z calculated	sub ppm		met. m/z observed	met. m/z calculated	met. ppm
MATCH	154.6	620.3362	620.3353	-1.47	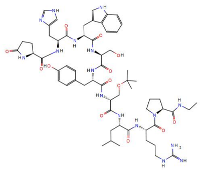 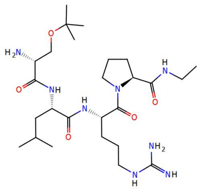	555.3988	555.3977	-2.01
MATCH	32.5	1239.6668	1239.6633	-2.86	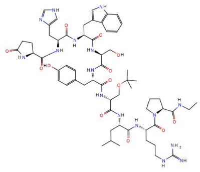 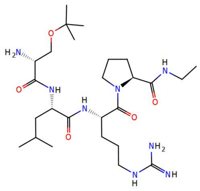	278.2032	278.2025	-2.56
MATCH	32.5	1239.6668	1239.6633	-2.86	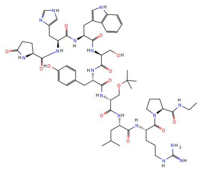 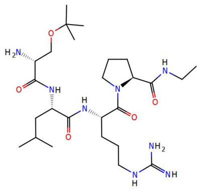	278.2032	278.2025	-2.56
MATCH	57.0	1239.6668	1239.6633	-2.86	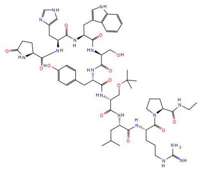 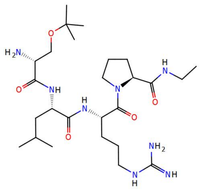	555.3988	555.3977	-2.01
MATCH	57.0	1239.6668	1239.6633	-2.86	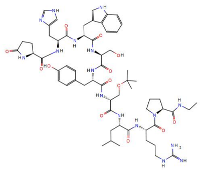 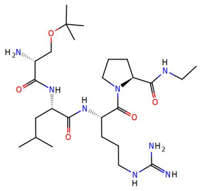	555.3988	555.3977	-2.01
MATCH	53.9	86.0973	86.0964	-9.80	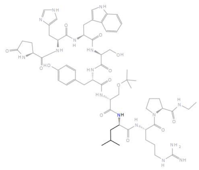 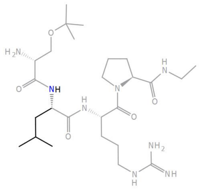	86.0972	86.0964	-8.89
MATCH	35.8	112.0875	112.0869	-4.95	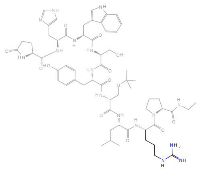 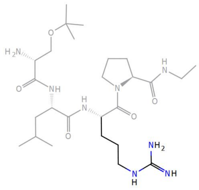	112.0873	112.0869	-3.70
MATCH	16.8	115.0871	115.0866	-4.13	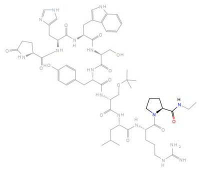 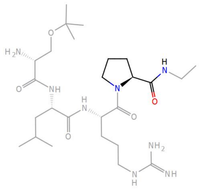	115.0871	115.0866	-4.53
MATCH	75.8	143.1182	143.1179	-2.15	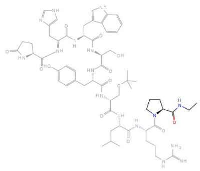 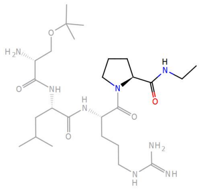	143.1181	143.1179	-1.70

Metabolite: M2 -684 RT=1.84

Type	score	sub. m/z observed	sub. m/z calculated	sub ppm		met. m/z observed	met. m/z calculated	met. ppm
MATCH	8.4	157.1087	157.1084	-2.26	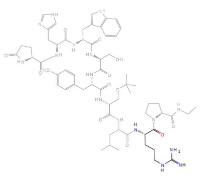 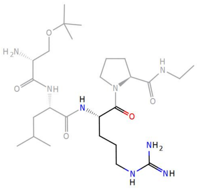	157.1082	157.1084	0.99
MATCH	19.6	253.1663	253.1659	-1.57	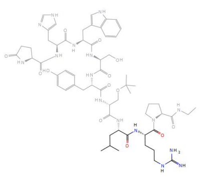 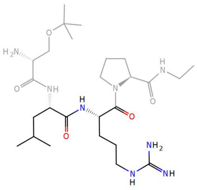	253.1659	253.1659	-0.09
MATCH	3.6	270.1922	270.1925	1.05	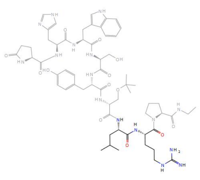 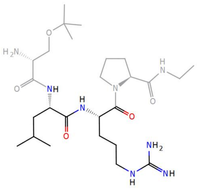	270.1929	270.1925	-1.55
MATCH	8.6	282.1927	282.1925	-0.86	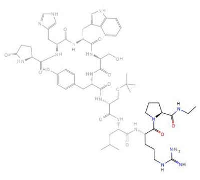 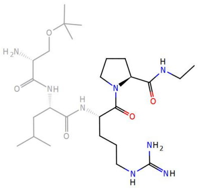	282.1921	282.1925	1.22
MATCH	52.8	299.2194	299.2190	-1.29	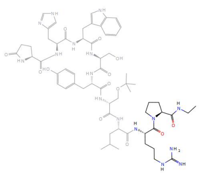 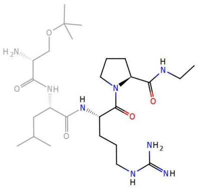	299.2193	299.2190	-1.11
MATCH	14.9	412.3019	412.3031	2.73	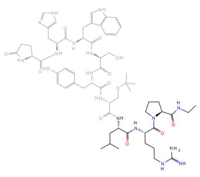 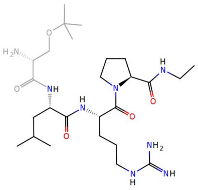	412.3027	412.3031	0.91
MATCH	9.1	521.2445	521.2487	8.02	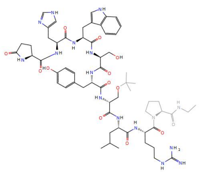 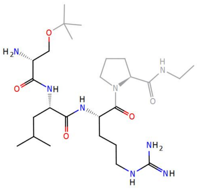	179.1070	179.1159	49.44
MATCH	8.3	521.2445	521.2487	8.02	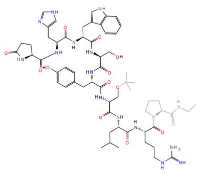 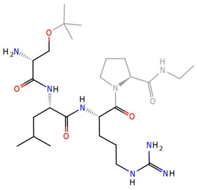	357.2248	357.2245	-0.76
MATCH	10.3	592.3055	592.3040	-2.57	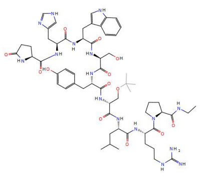 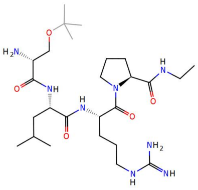	250.1705	250.1712	2.61

Metabolite: M2 -684 RT=1.84

Type	score	sub. m/z observed	sub. m/z calculated	sub ppm		met. m/z observed	met. m/z calculated	met. ppm
MATCH	11.5	1239.6657	1239.6633	-1.95	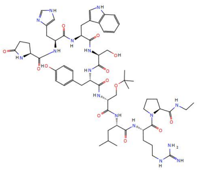 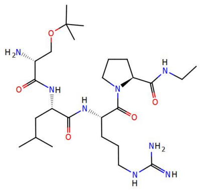	555.3973	555.3977	0.75
MISMATCH	-10.8	592.3055	592.3040	-2.57	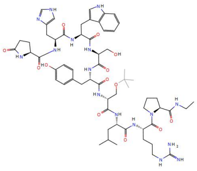	499.3355	499.3355	0.00
MET_MATCH					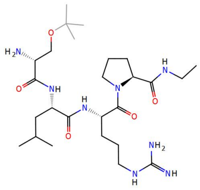	250.1716	250.1712	-1.74
MET_MATCH					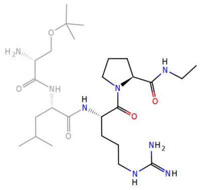	299.2197	299.2190	-2.19
MET_MATCH					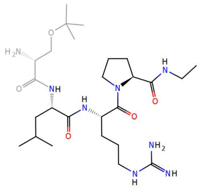	412.3038	412.3031	-1.85
MET_MATCH					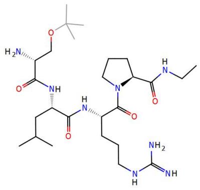	499.3362	499.3351	-2.17
MET_MATCH					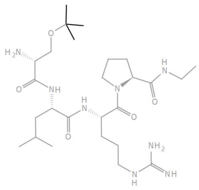	57.0708	57.0699	-17.0
MET_MATCH					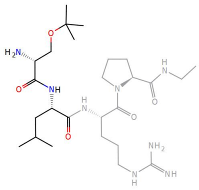	129.1025	129.0966	-45.5
MET_MATCH					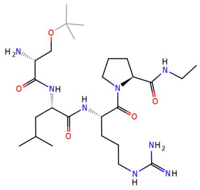	499.3355	499.3351	-0.90
