# Supplementary material for: Software-aided approach to investigate peptide structure and metabolic susceptibility of amide bonds in peptide drugs based on high resolution mass spectrometry
Source: PLoS One. 2017 Nov 1;12(11):e0186461. doi: 10.1371/journal.pone.0186461 (PMC5665424; doi:10.1371/journal.pone.0186461)
Supplement: S1 File — (ZIP) [file pone.0186461.s007.zip › SFiles/S20_File.pdf]

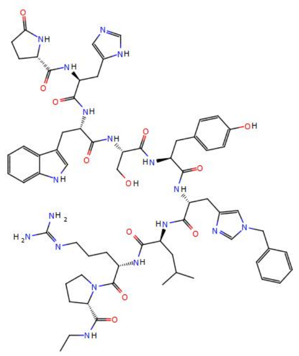

Histrelin

Property name	Property value
Time	0min, 5min, 15min, 45min, 120min
Instrument	ThermoQAPlus
Acquisition Mode	ddMS2
Matrix	pepsin

Chromatograms

Time=0min

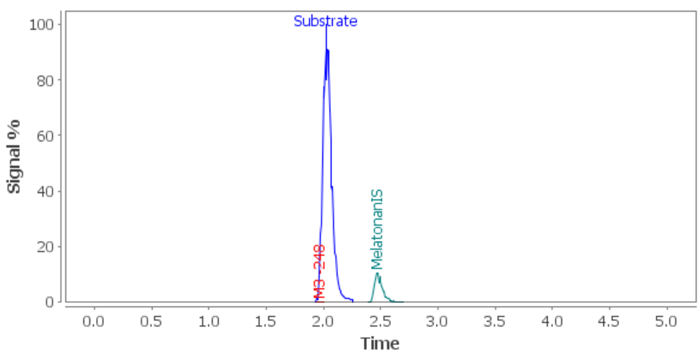

Time=5min

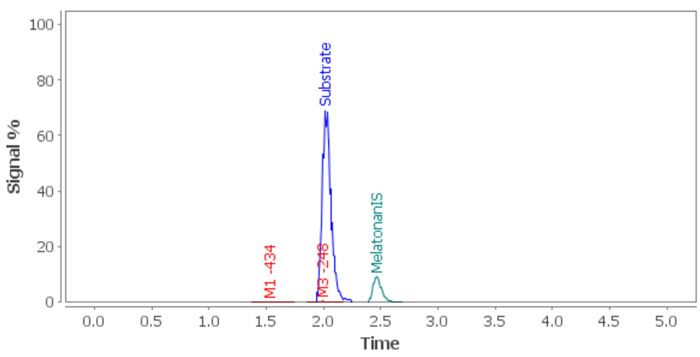

Time=15min

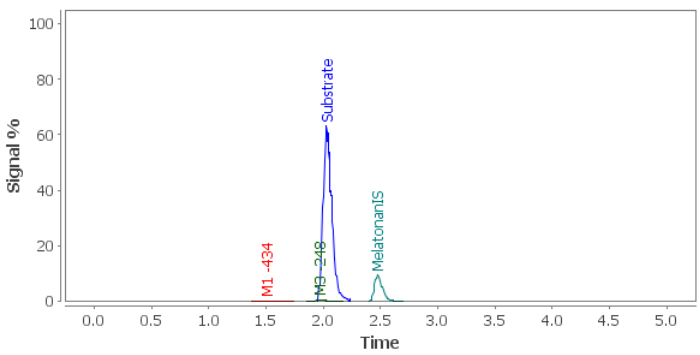

Time=45min

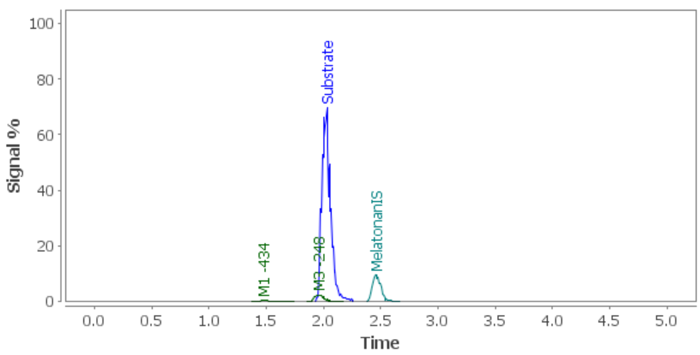

Time=120min

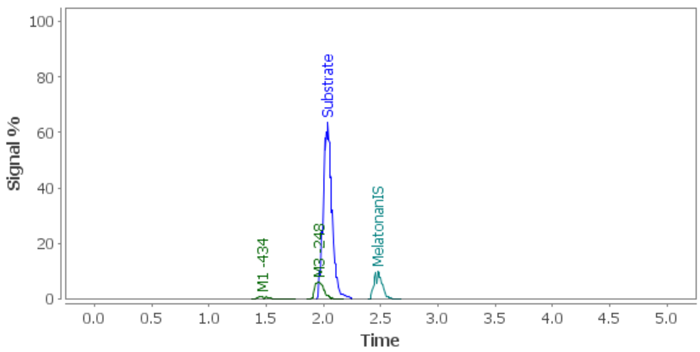

Custom Charts

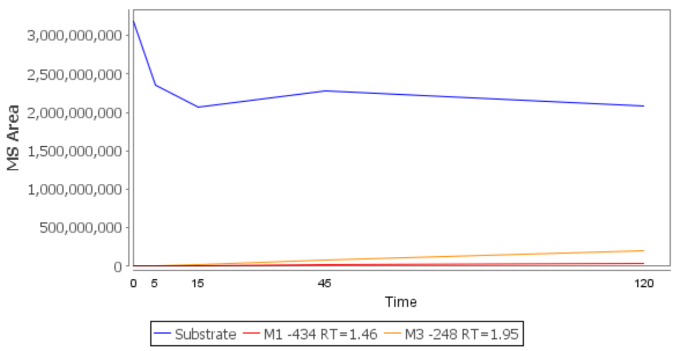

Fragmentation

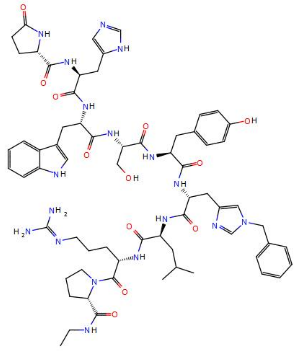

Histrelin

MS (+) FT

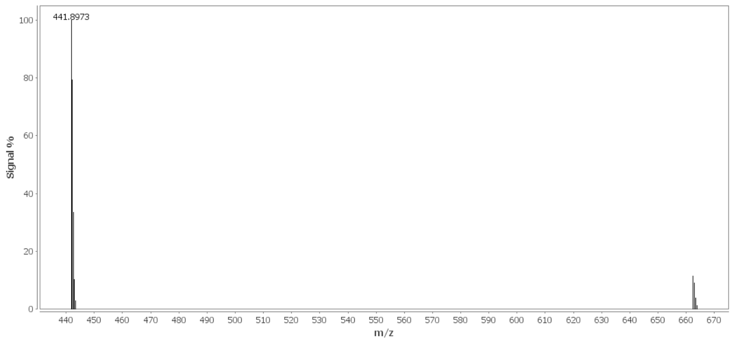

MS (+) FT

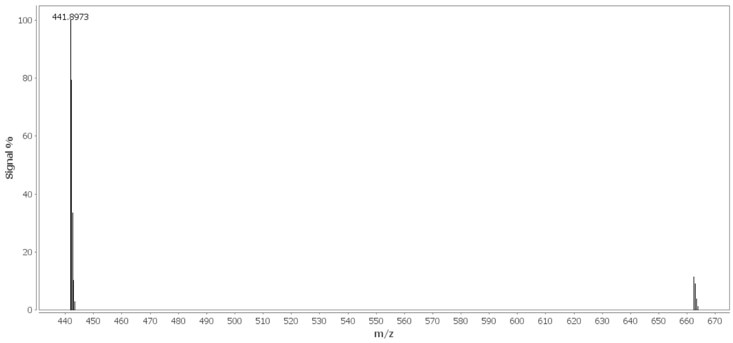

MS2 (+) FT activ = HCD:ce =

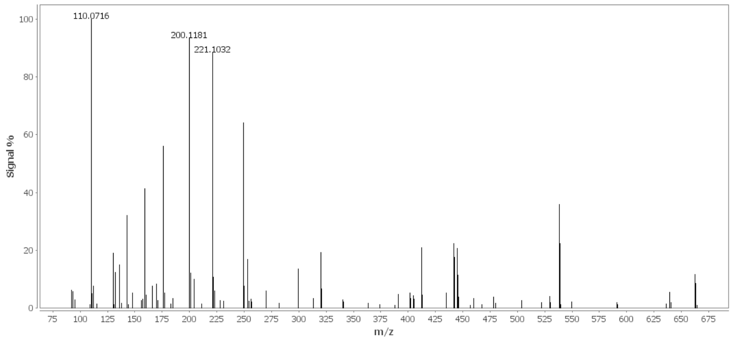

MS2 (+) FT activ = HCD:ce =

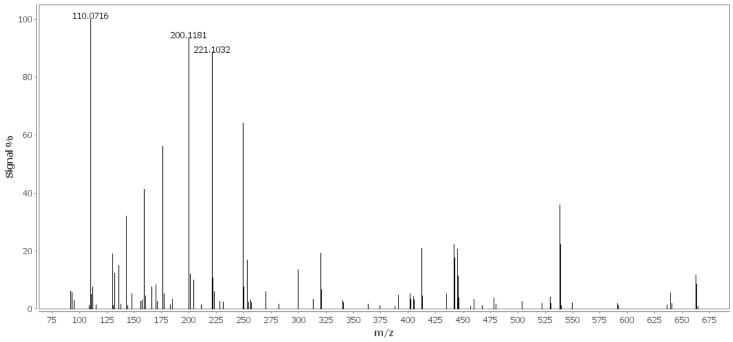

Metabolite: Substrate

Type	score	sub. m/z observed	sub. m/z calculated	sub ppm			met. m/z observed	met. m/z calculated	met. ppm
MATCH	111.4	662.3417	662.3409	-1.13	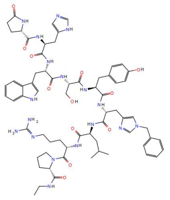	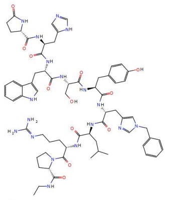	662.3417	662.3409	-1.13
MATCH	18.8	662.3409	662.3409	0.08	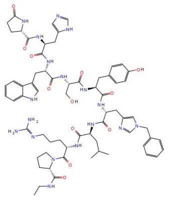	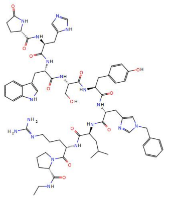	662.3409	662.3409	0.08
MATCH	9.2	639.4060	639.4089	4.53	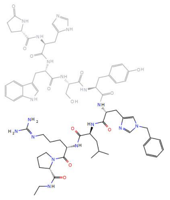	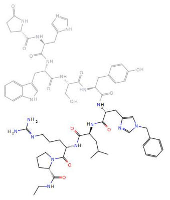	639.4060	639.4089	4.53

Metabolite: Substrate

Type	score	sub. m/z observed	sub. m/z calculated	sub ppm			met. m/z observed	met. m/z calculated	met. ppm
MATCH	6.1	549.3630	549.3620	-1.78	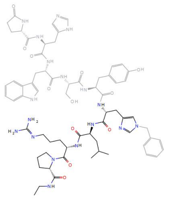	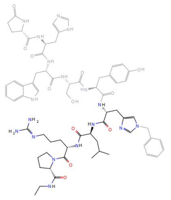	549.3630	549.3620	-1.78
MISMATCH	5.5	529.7818	529.7822	0.76	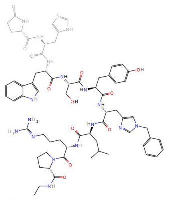	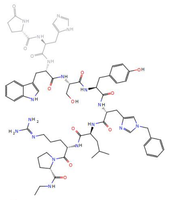	529.7818	529.7822	0.76
MATCH	6.9	504.1997	504.1990	-1.32	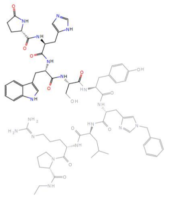	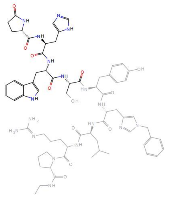	504.1997	504.1990	-1.32
MATCH	8.8	478.2079	478.2085	1.18	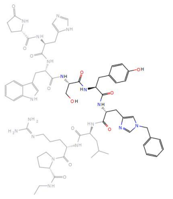	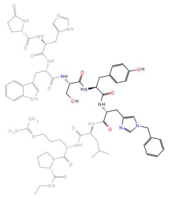	478.2079	478.2085	1.18
MATCH	8.8	478.2079	478.2085	1.18	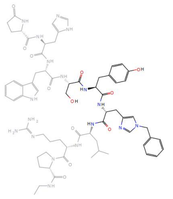	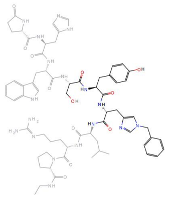	478.2079	478.2085	1.18
MATCH	55.9	445.2560	445.2558	-0.42	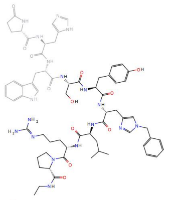	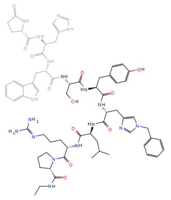	445.2560	445.2558	-0.42
MATCH	200.0	441.8973	441.8964	-2.12	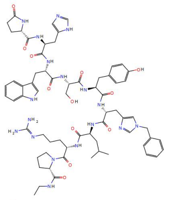	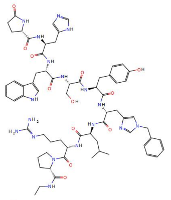	441.8973	441.8964	-2.12
MATCH	29.5	441.8964	441.8964	-0.15	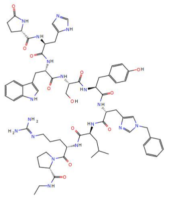	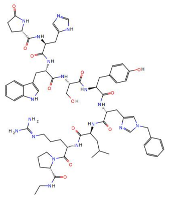	441.8964	441.8964	-0.15
MATCH	36.1	412.3025	412.3031	1.41	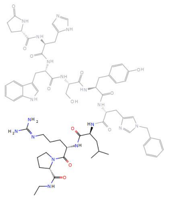	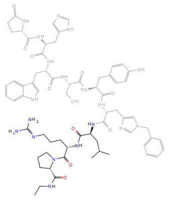	412.3025	412.3031	1.41

Metabolite: Substrate

Type	score	sub. m/z observed	sub. m/z calculated	sub ppm			met. m/z observed	met. m/z calculated	met. ppm
MATCH	27.2	401.7394	401.7398	0.88	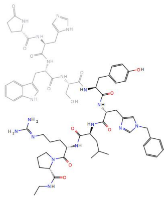	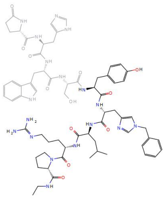	401.7394	401.7398	0.88
MATCH	11.4	391.1760	391.1765	1.17	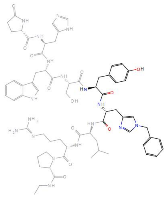	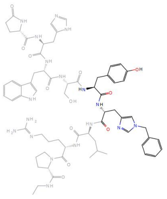	391.1760	391.1765	1.17
MATCH	11.4	391.1760	391.1765	1.17	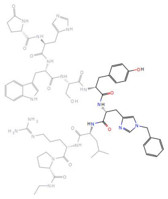	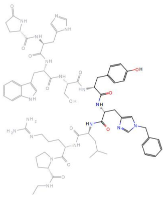	391.1760	391.1765	1.17
MATCH	3.4	374.1498	374.1499	0.23	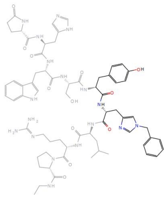	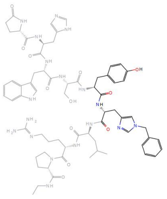	374.1498	374.1499	0.23
MATCH	2.6	363.1819	363.1816	-0.90	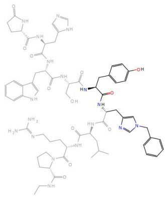	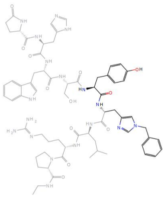	363.1819	363.1816	-0.90
MATCH	44.1	320.2079	320.2081	0.75	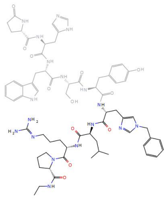	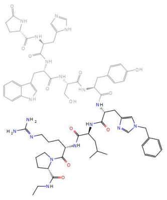	320.2079	320.2081	0.75
MATCH	5.4	313.2015	313.2023	2.38	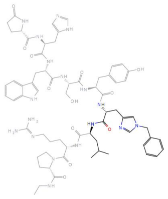	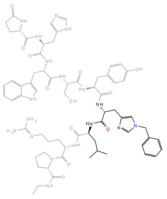	313.2015	313.2023	2.38
MATCH	22.1	299.2186	299.2190	1.38	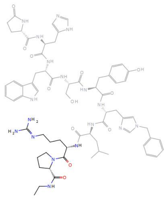	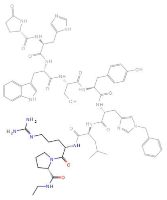	299.2186	299.2190	1.38
MATCH	3.1	282.1920	282.1925	1.57	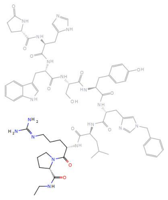	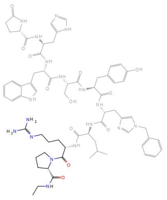	282.1920	282.1925	1.57

Metabolite: Substrate

Type	score	sub. m/z observed	sub. m/z calculated	sub ppm			met. m/z observed	met. m/z calculated	met. ppm
MATCH	10.8	270.1925	270.1925	-0.18	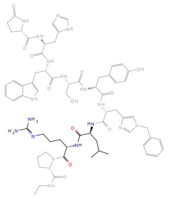	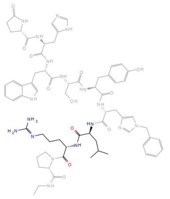	270.1925	270.1925	-0.18
MATCH	5.2	256.1075	256.1081	2.13	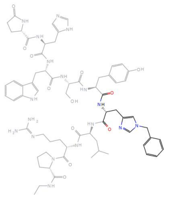	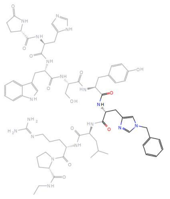	256.1075	256.1081	2.13
MATCH	30.0	253.1657	253.1659	0.79	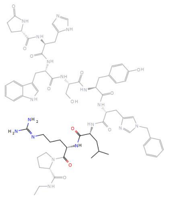	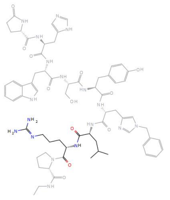	253.1657	253.1659	0.79
MATCH	3.7	211.1437	211.1357	-37.7	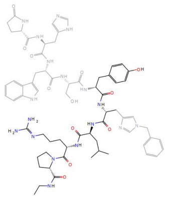	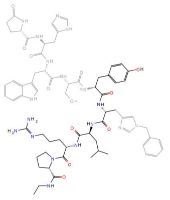	211.1437	211.1357	-37.7
MATCH	157.2	200.1181	200.1182	0.58	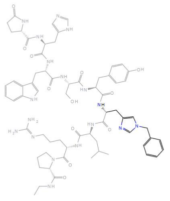	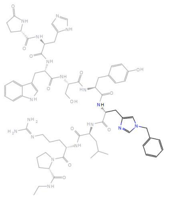	200.1181	200.1182	0.58
MATCH	5.9	185.1052	185.1033	-10.0	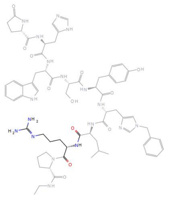	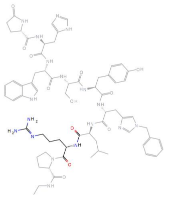	185.1052	185.1033	-10.0
MATCH	5.9	185.1052	185.1073	11.68	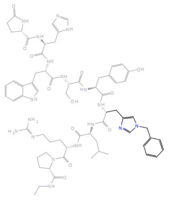	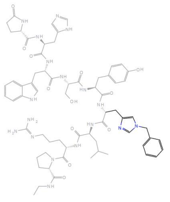	185.1052	185.1073	11.68
MATCH	5.9	185.1052	185.0997	-29.5	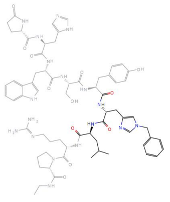	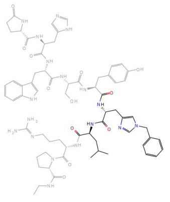	185.1052	185.0997	-29.5
MATCH	107.2	170.0601	170.0600	-0.06	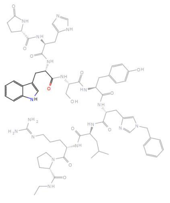	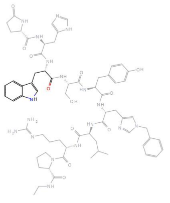	170.0601	170.0600	-0.06

Metabolite: Substrate

Type	score	sub. m/z observed	sub. m/z calculated	sub ppm			met. m/z observed	met. m/z calculated	met. ppm
MATCH	93.1	159.0916	159.0917	0.49	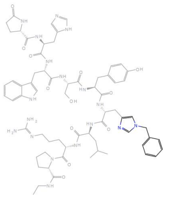	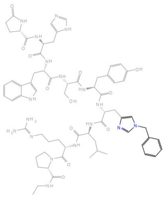	159.0916	159.0917	0.49
MATCH	5.1	157.1078	157.1084	3.59	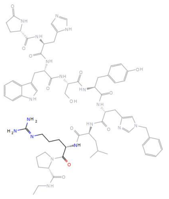	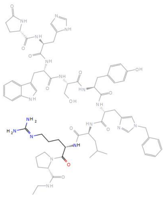	157.1078	157.1084	3.59
MATCH	62.4	143.1178	143.1179	0.46	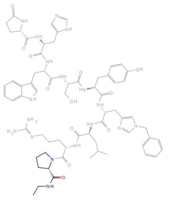	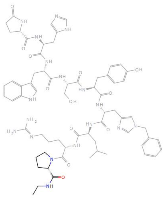	143.1178	143.1179	0.46
MATCH	33.0	136.0756	136.0757	0.47	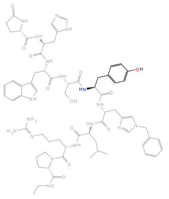	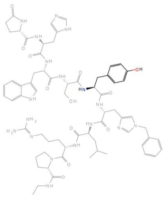	136.0756	136.0757	0.47
MATCH	41.1	130.0651	130.0575	-58.6	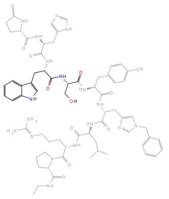	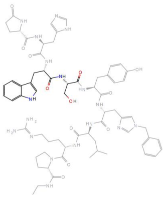	130.0651	130.0575	-58.6
MATCH	3.7	115.0870	115.0866	-3.66	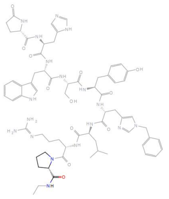	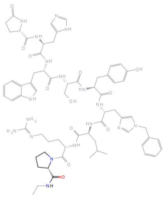	115.0870	115.0866	-3.66
MISMATCH	-118.1	110.0716	110.0713	-2.65	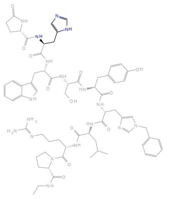	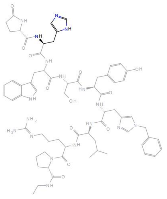	110.0716	110.0713	-2.65
MISMATCH	-5.2	95.0607	95.0604	-3.06	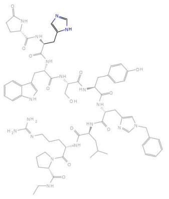	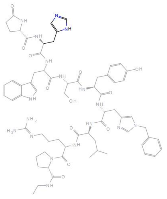	95.0607	95.0604	-3.06

MS (+) FT

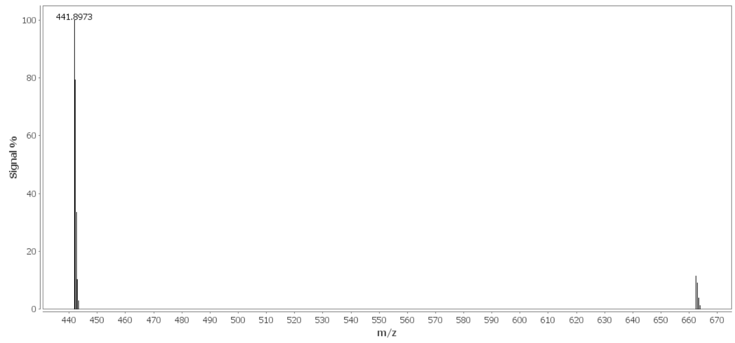

MS (+) FT

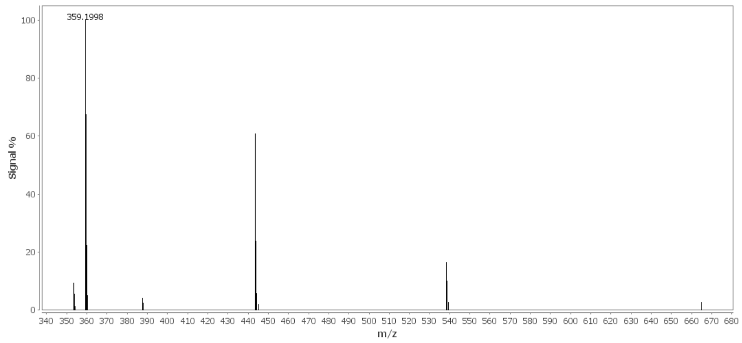

MS2 (+) FT activ = HCD:ce =

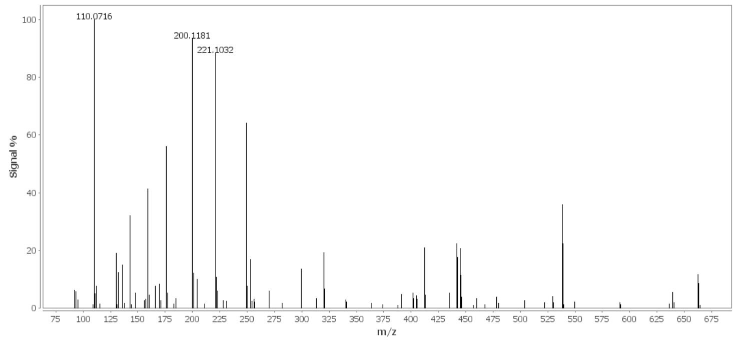

MS2 (+) FT activ = HCD:ce =

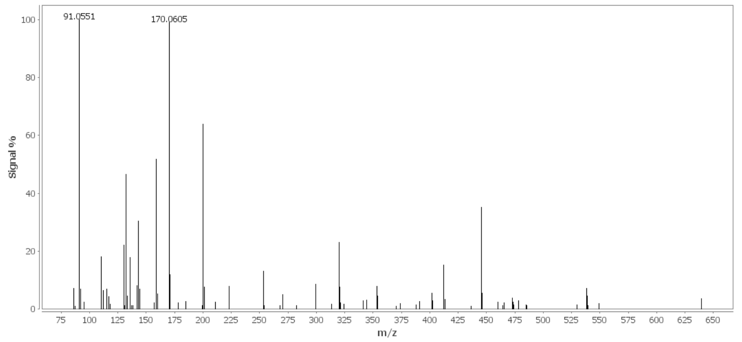

Metabolite: M3 -248 RT=1.95

Type	score	sub. m/z observed	sub. m/z calculated	sub ppm			met. m/z observed	met. m/z calculated	met. ppm
MATCH	200.0	441.8973	441.8964	-2.12	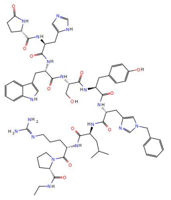	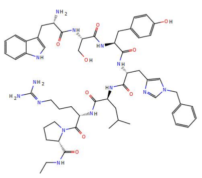	359.1998	359.1994	-1.23
MATCH	200.0	441.8973	441.8964	-2.12	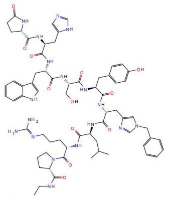	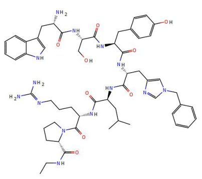	359.1998	359.1994	-1.23
MATCH	116.3	441.8973	441.8964	-2.12	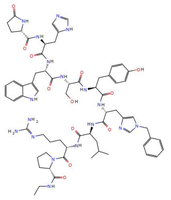	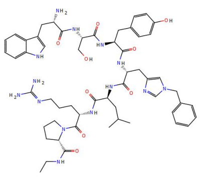	538.2964	538.2954	-1.73
MATCH	116.3	441.8973	441.8964	-2.12	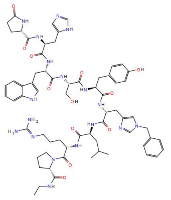	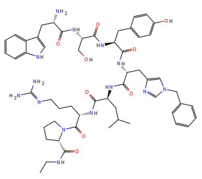	538.2964	538.2954	-1.73
MATCH	111.4	662.3417	662.3409	-1.13	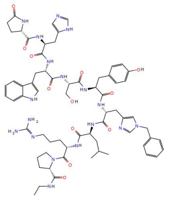	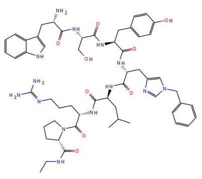	359.1998	359.1994	-1.23

Metabolite: M3 -248 RT=1.95

Type	score	sub. m/z observed	sub. m/z calculated	sub ppm			met. m/z observed	met. m/z calculated	met. ppm
MATCH	111.4	662.3417	662.3409	-1.13	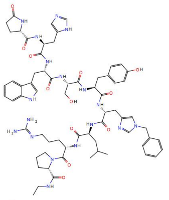	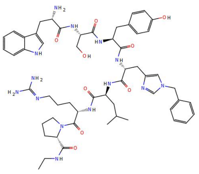	359.1998	359.1994	-1.23
MATCH	27.8	662.3417	662.3409	-1.13	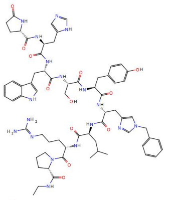	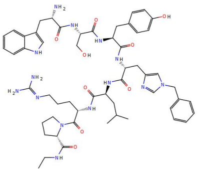	538.2964	538.2954	-1.73
MATCH	27.8	662.3417	662.3409	-1.13	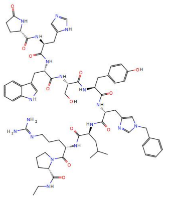	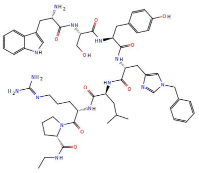	538.2964	538.2954	-1.73
MATCH	3.7	115.0870	115.0866	-3.66	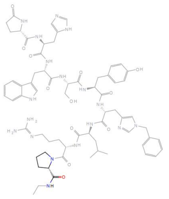	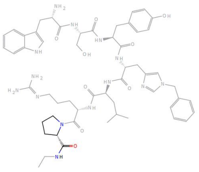	115.0872	115.0866	-5.52
MATCH	41.1	130.0651	130.0575	-58.6	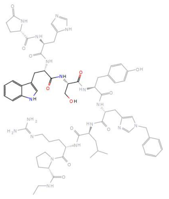	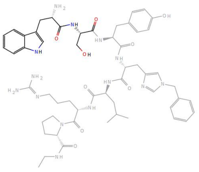	130.0656	130.0575	-62.3
MATCH	32.8	136.0756	136.0757	0.47	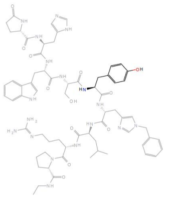	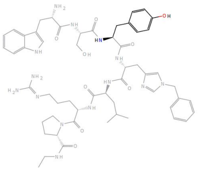	136.0763	136.0757	-4.16
MATCH	62.4	143.1178	143.1179	0.46	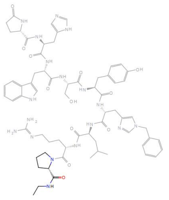	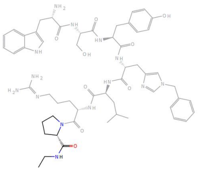	143.1184	143.1179	-3.68
MATCH	5.1	157.1078	157.1084	3.59	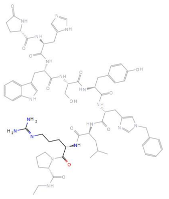	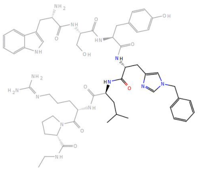	157.1087	157.1048	-24.9
						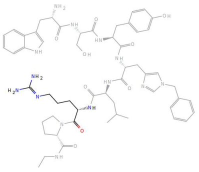	157.1087	157.1084	-2.03

Metabolite: M3 -248 RT=1.95

Type	score	sub. m/z observed	sub. m/z calculated	sub ppm		met. m/z observed	met. m/z calculated	met. ppm
MATCH	93.1	159.0916	159.0917	0.49	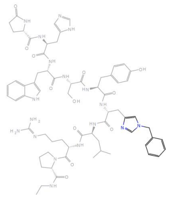 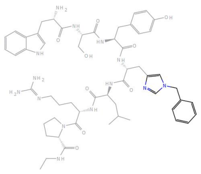	159.0922	159.0917	-2.99
MATCH	107.2	170.0601	170.0600	-0.06	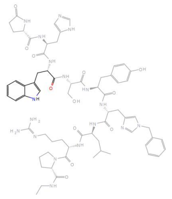 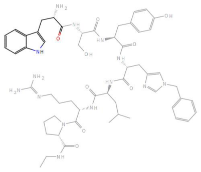	170.0605	170.0600	-2.86
MATCH	5.9	185.1052	185.0997	-29.5	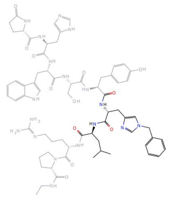 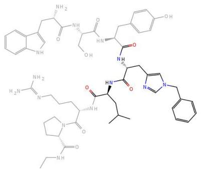	185.1056	185.0997	-31.7
MATCH	5.9	185.1052	185.1033	-10.0	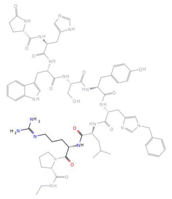 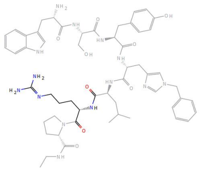	185.1056	185.1033	-12.2
MATCH	5.9	185.1052	185.1073	11.68	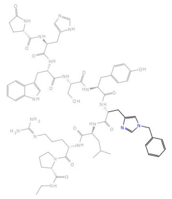 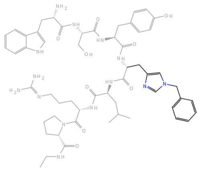	185.1056	185.1073	9.50
MATCH	157.2	200.1181	200.1182	0.58	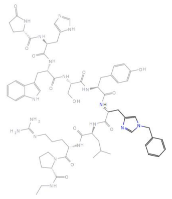 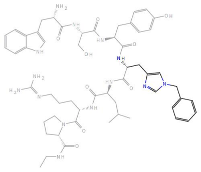	200.1188	200.1182	-2.92
MATCH	3.7	211.1437	211.1357	-37.7	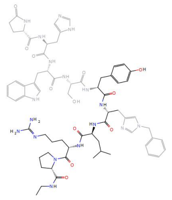 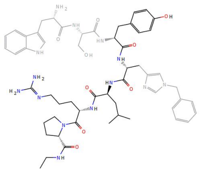	211.1446	211.1357	-42.0
MATCH	30.0	253.1657	253.1659	0.79	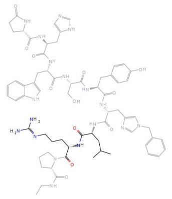 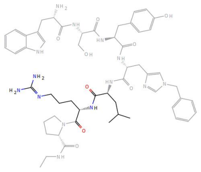	253.1665	253.1659	-2.18
MATCH	10.8	270.1925	270.1925	-0.18	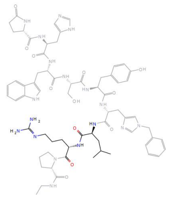 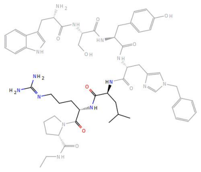	270.1934	270.1925	-3.44

Metabolite: M3 -248 RT=1.95

Type	score	sub. m/z observed	sub. m/z calculated	sub ppm			met. m/z observed	met. m/z calculated	met. ppm
MATCH	3.1	282.1920	282.1925	1.57	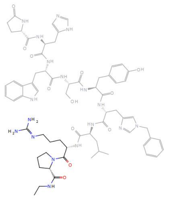	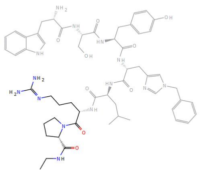	282.1925	282.1925	-0.24
MATCH	22.1	299.2186	299.2190	1.38	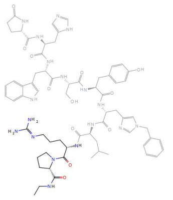	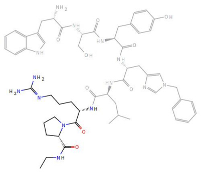	299.2200	299.2190	-3.44
MATCH	5.4	313.2015	313.2023	2.38	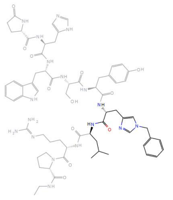	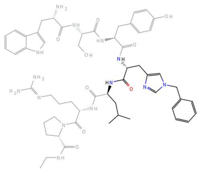	157.1087	157.1048	-24.9
MATCH	5.4	313.2015	313.2023	2.38	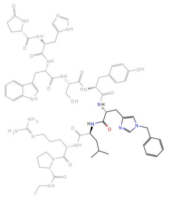	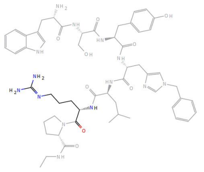	157.1087	157.1084	-2.03
MATCH	5.0	313.2015	313.2023	2.38	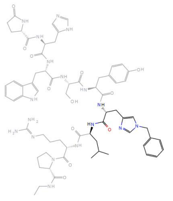	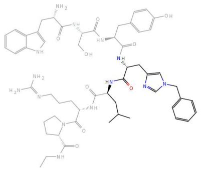	313.2024	313.2023	-0.26
MATCH	42.5	320.2079	320.2081	0.75	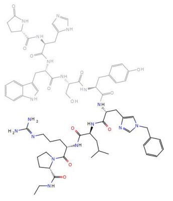	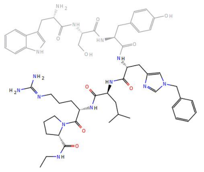	320.2088	320.2081	-2.17
MATCH	7.4	391.1760	391.1765	1.17	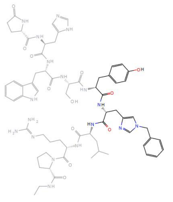	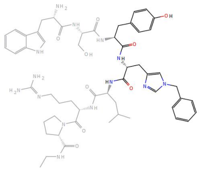	391.1764	391.1765	0.26
MATCH	7.4	391.1760	391.1765	1.17	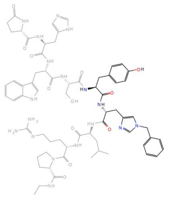	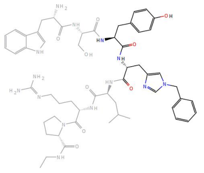	391.1764	391.1765	0.26
MATCH	10.6	401.7394	401.7398	0.88	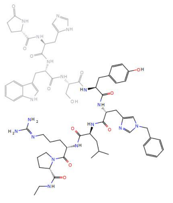	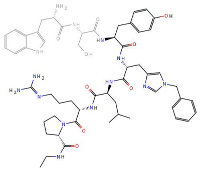	401.7423	401.7398	-6.37

Metabolite: M3 -248 RT=1.95

Type	score	sub. m/z observed	sub. m/z calculated	sub ppm			met. m/z observed	met. m/z calculated	met. ppm
MATCH	36.1	412.3025	412.3031	1.41	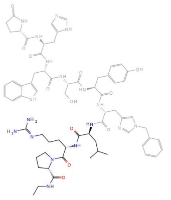	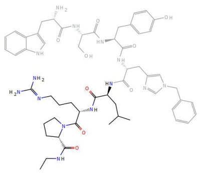	412.3037	412.3031	-1.44
MATCH	29.5	441.8964	441.8964	-0.15	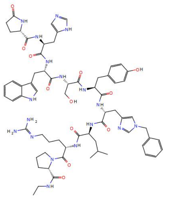	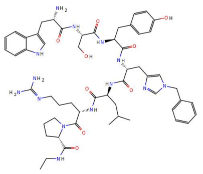	538.2971	538.2954	-3.10
						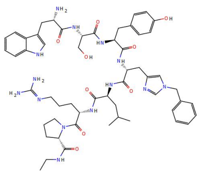	538.2971	538.2954	-3.10
MATCH	55.9	445.2560	445.2558	-0.42	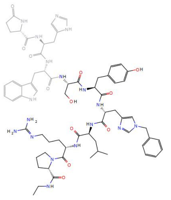	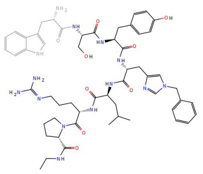	445.2573	445.2558	-3.52
MATCH	8.8	478.2079	478.2085	1.18	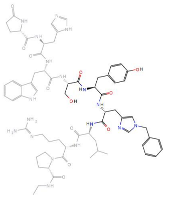	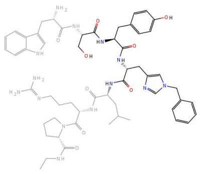	160.0761	160.0743	-10.7
MATCH	8.8	478.2079	478.2085	1.18	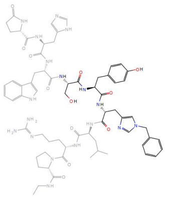	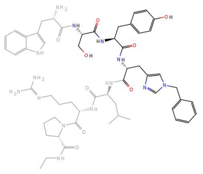	160.0761	160.0743	-10.7
MATCH	6.6	478.2079	478.2085	1.18	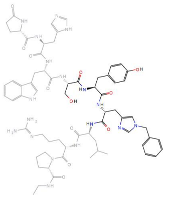	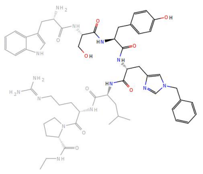	478.2081	478.2085	0.75
MATCH	6.6	478.2079	478.2085	1.18	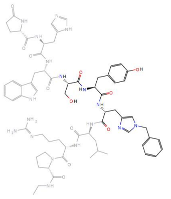	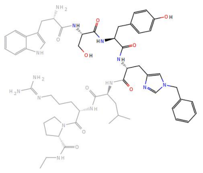	478.2081	478.2085	0.75
MATCH	5.5	529.7818	529.7822	0.76	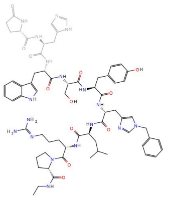	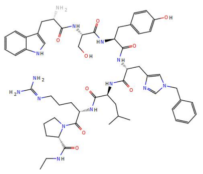	529.7849	529.7822	-5.20

Metabolite: M3 -248 RT=1.95

Type	score	sub. m/z observed	sub. m/z calculated	sub ppm			met. m/z observed	met. m/z calculated	met. ppm
MATCH	4.0	549.3630	549.3620	-1.78	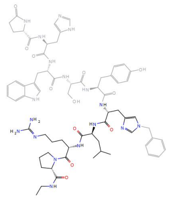	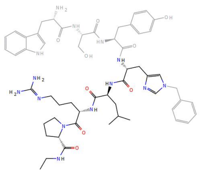	549.3615	549.3620	0.96
MATCH	9.2	639.4060	639.4089	4.53	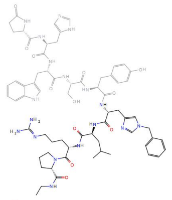	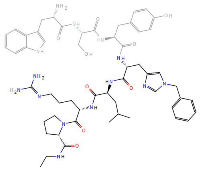	639.4077	639.4089	1.88
MATCH	18.8	662.3409	662.3409	0.08	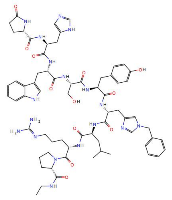	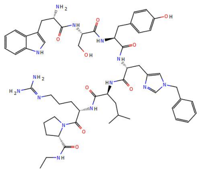	538.2971	538.2954	-3.10
						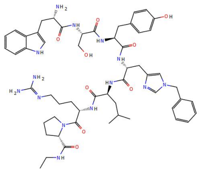	538.2971	538.2954	-3.10
MISMATCH	-5.2	95.0607	95.0604	-3.06	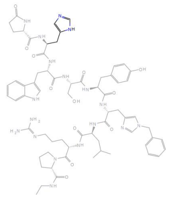		95.0614	95.0614	0.00
MISMATCH	-118.1	110.0716	110.0713	-2.65	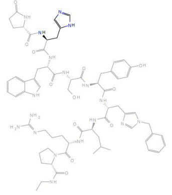		110.0719	110.0719	0.00
MISMATCH	-12.0	529.7818	529.7822	0.76	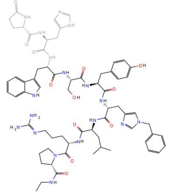		353.5250	353.5250	0.00
MET_MATCH						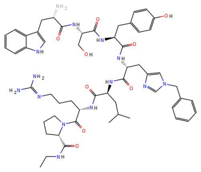	353.5247	353.5239	-2.35
MET_MATCH						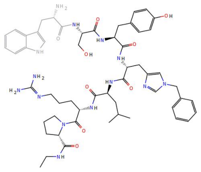	445.2561	445.2558	-0.81

Metabolite: M3 -248 RT=1.95

Type	score	sub. m/z observed	sub. m/z calculated	sub ppm		met. m/z observed	met. m/z calculated	met. ppm
MET_MATCH					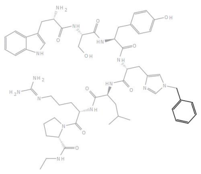	91.0551	91.0542	-9.63
MET_MATCH					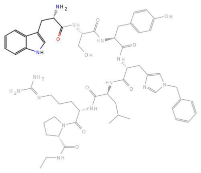	95.0614	95.0548	-69.6
MET_MATCH					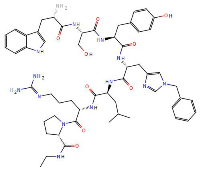	353.5250	353.5239	-3.26

MS (+) FT

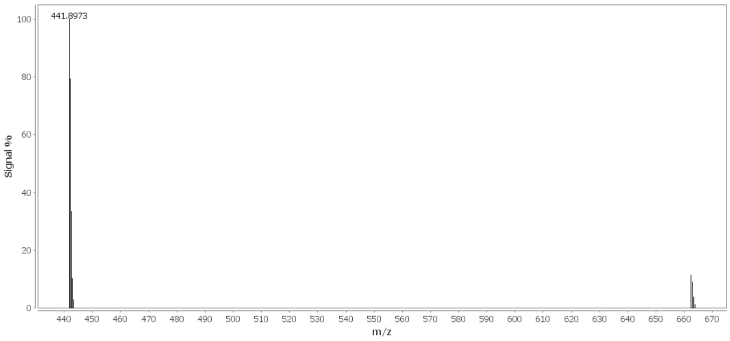

MS (+) FT

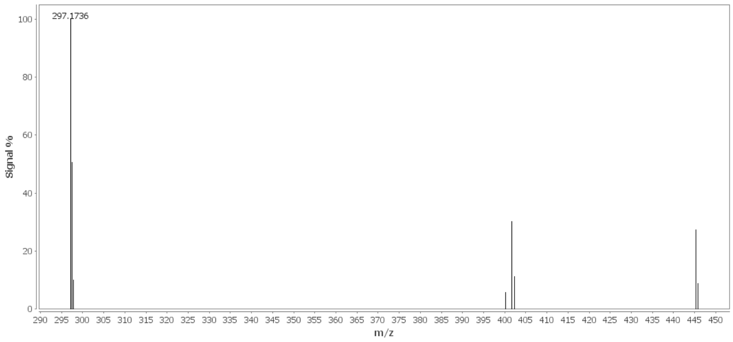

MS2 (+) FT activ = HCD:ce =

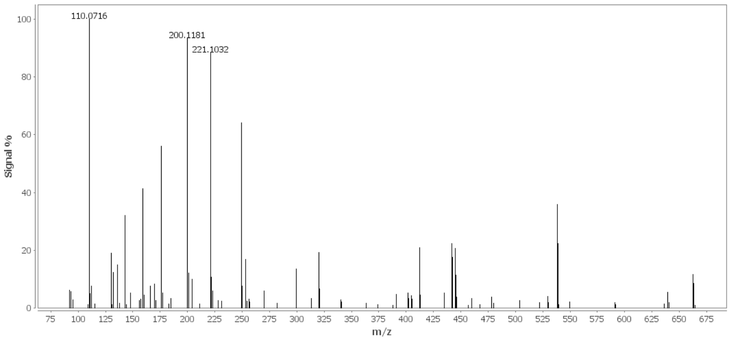

MS2 (+) FT activ = HCD:ce =

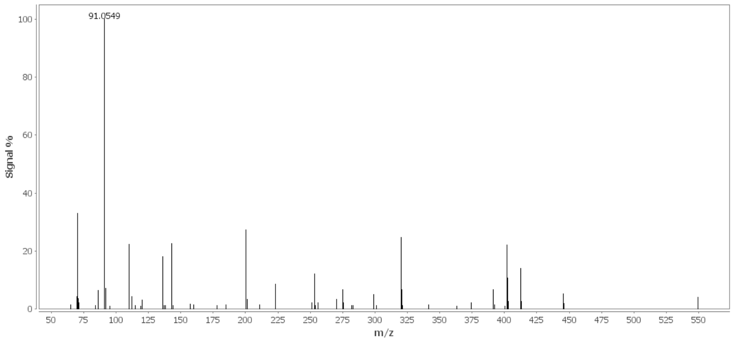

Metabolite: M1 -434 RT=1.46

Type	score	sub. m/z observed	sub. m/z calculated	sub ppm			met. m/z observed	met. m/z calculated	met. ppm
MATCH	200.0	441.8973	441.8964	-2.12	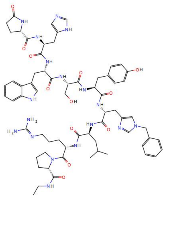	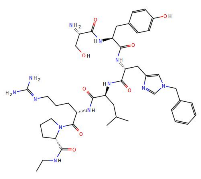	297.1736	297.1729	-2.27

Metabolite: M1 -434 RT=1.46

Type	score	sub. m/z observed	sub. m/z calculated	sub ppm			met. m/z observed	met. m/z calculated	met. ppm
MATCH	200.0	441.8973	441.8964	-2.12	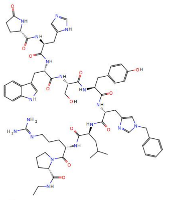	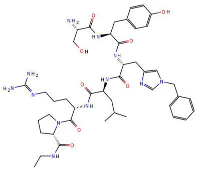	297.1736	297.1729	-2.27
MATCH	127.3	441.8973	441.8964	-2.12	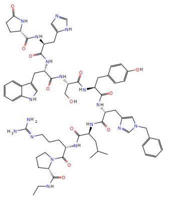	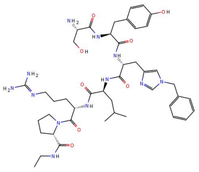	445.2568	445.2558	-2.24
MATCH	127.3	441.8973	441.8964	-2.12	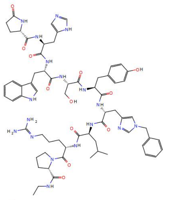	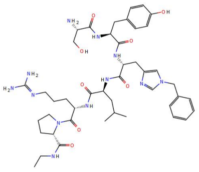	445.2568	445.2558	-2.24
MATCH	111.4	662.3417	662.3409	-1.13	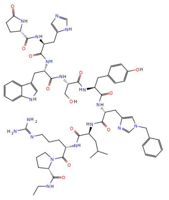	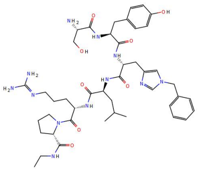	297.1736	297.1729	-2.27
MATCH	111.4	662.3417	662.3409	-1.13	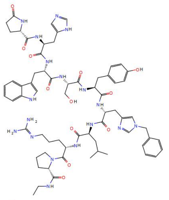	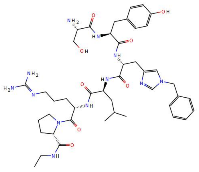	297.1736	297.1729	-2.27
MATCH	38.7	662.3417	662.3409	-1.13	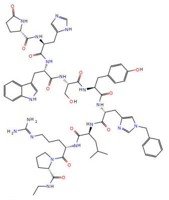	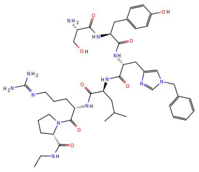	445.2568	445.2558	-2.24
MATCH	38.7	662.3417	662.3409	-1.13	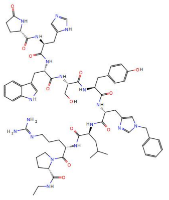	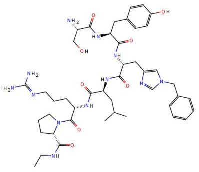	445.2568	445.2558	-2.24
MATCH	2.5	115.0870	115.0866	-3.66	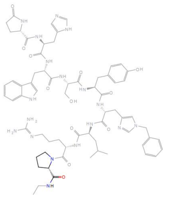	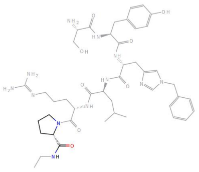	115.0869	115.0866	-2.97
MATCH	33.0	136.0756	136.0757	0.47	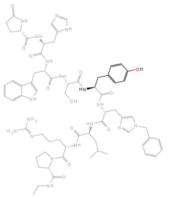	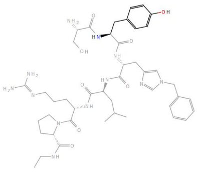	136.0760	136.0757	-2.15

Metabolite: M1 -434 RT=1.46

Type	score	sub. m/z observed	sub. m/z calculated	sub ppm			met. m/z observed	met. m/z calculated	met. ppm
MATCH	54.5	143.1178	143.1179	0.46	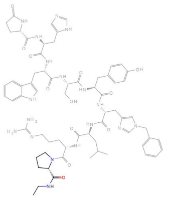	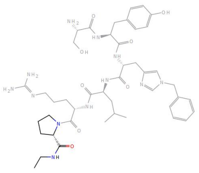	143.1181	143.1179	-1.80
MATCH	4.6	157.1078	157.1084	3.59	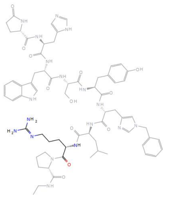	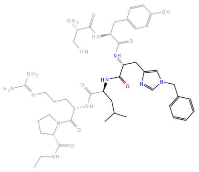	157.1083	157.1048	-22.6
						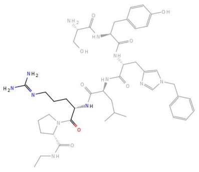	157.1083	157.1084	0.35
MATCH	4.8	185.1052	185.0997	-29.5	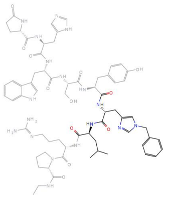	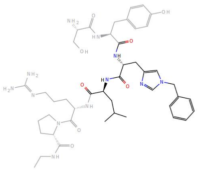	185.1041	185.0997	-23.8
MATCH	4.8	185.1052	185.1033	-10.0	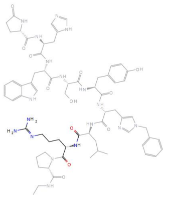	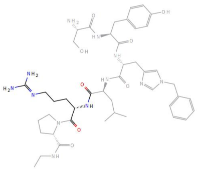	185.1041	185.1033	-4.34
MATCH	4.8	185.1052	185.1073	11.68	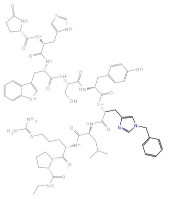	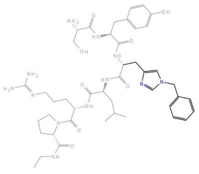	185.1041	185.1073	17.39
MATCH	120.6	200.1181	200.1182	0.58	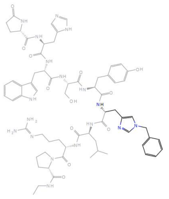	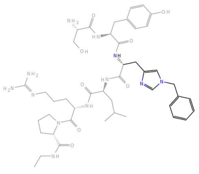	200.1185	200.1182	-1.57
MATCH	2.8	211.1437	211.1357	-37.7	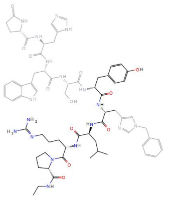	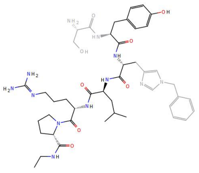	211.1444	211.1357	-41.1
MATCH	29.0	253.1657	253.1659	0.79	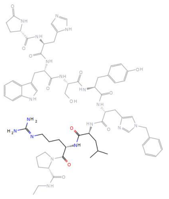	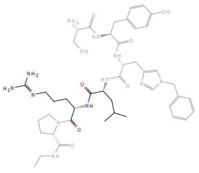	253.1661	253.1659	-0.64

Metabolite: M1 -434 RT=1.46

Type	score	sub. m/z observed	sub. m/z calculated	sub ppm		met. m/z observed	met. m/z calculated	met. ppm
MATCH	5.2	256.1075	256.1081	2.13	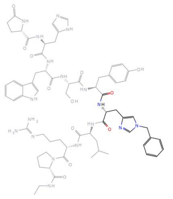 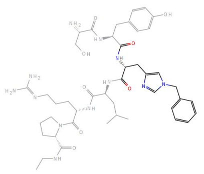	256.1085	256.1081	-1.61
MATCH	9.3	270.1925	270.1925	-0.18	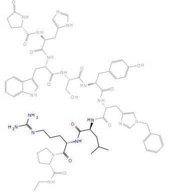 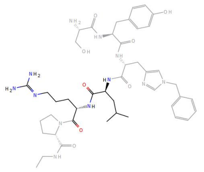	270.1925	270.1925	-0.30
MATCH	3.0	282.1920	282.1925	1.57	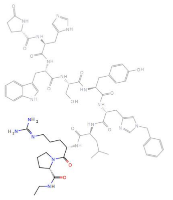 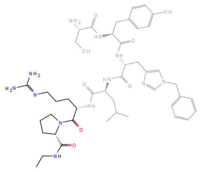	282.1926	282.1925	-0.45
MATCH	18.5	299.2186	299.2190	1.38	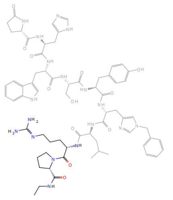 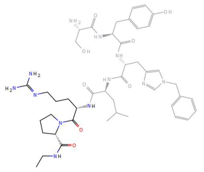	299.2193	299.2190	-0.87
MATCH	4.9	313.2015	313.2023	2.38	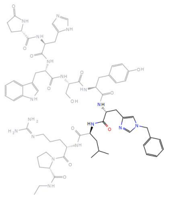 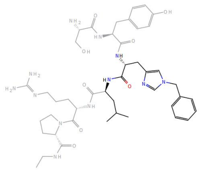	157.1083	157.1048	-22.6
					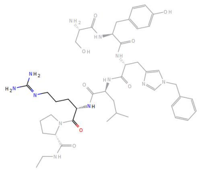	157.1083	157.1084	0.35
MATCH	44.1	320.2079	320.2081	0.75	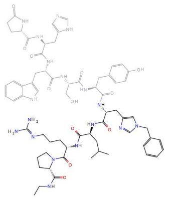 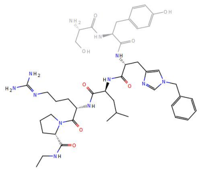	320.2086	320.2081	-1.47
MATCH	2.6	363.1819	363.1816	-0.90	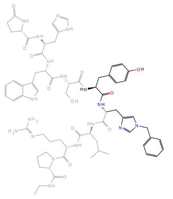 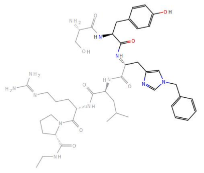	363.1821	363.1816	-1.59
MATCH	3.4	374.1498	374.1499	0.23	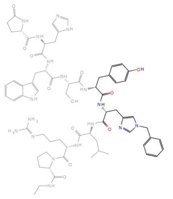 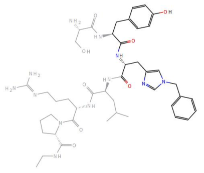	374.1506	374.1499	-1.71

Metabolite: M1 -434 RT=1.46

Type	score	sub. m/z observed	sub. m/z calculated	sub ppm			met. m/z observed	met. m/z calculated	met. ppm
MATCH	11.4	391.1760	391.1765	1.17	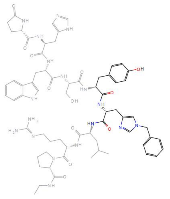	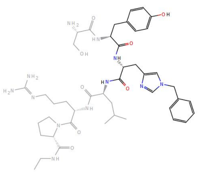	391.1771	391.1765	-1.68
MATCH	11.4	391.1760	391.1765	1.17	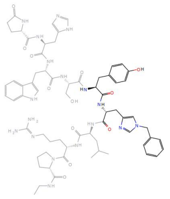	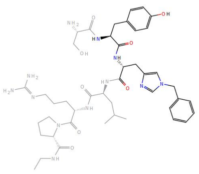	391.1771	391.1765	-1.68
MATCH	27.2	401.7394	401.7398	0.88	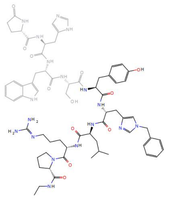	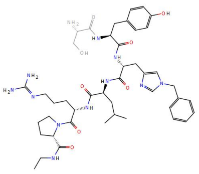	401.7405	401.7398	-1.73
MATCH	34.9	412.3025	412.3031	1.41	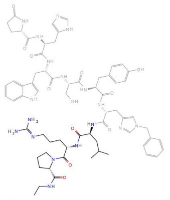	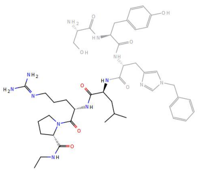	412.3032	412.3031	-0.37
MATCH	27.5	441.8964	441.8964	-0.15	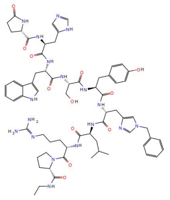	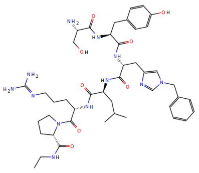	445.2573	445.2558	-3.31
						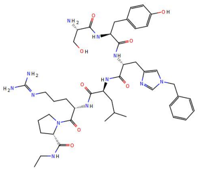	445.2573	445.2558	-3.31
MATCH	5.1	478.2079	478.2085	1.18	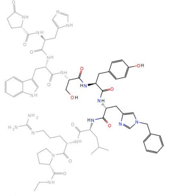	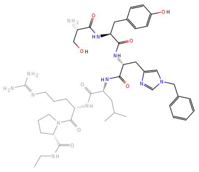	160.0760	160.0743	-10.5
MATCH	6.9	504.1997	504.1990	-1.32	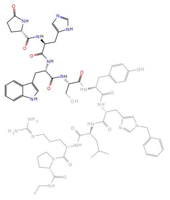	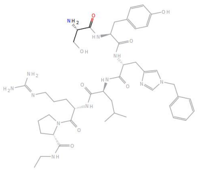	70.0297	70.0287	-13.2
MATCH	6.1	549.3630	549.3620	-1.78	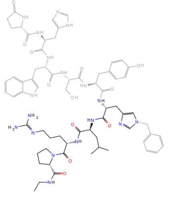	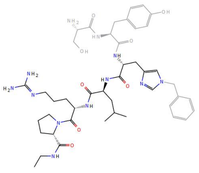	549.3623	549.3620	-0.59

Metabolite: M1 -434 RT=1.46

Type	score	sub. m/z observed	sub. m/z calculated	sub ppm			met. m/z observed	met. m/z calculated	met. ppm
MATCH	16.9	662.3409	662.3409	0.08	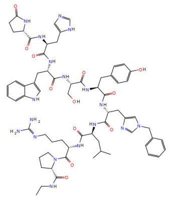	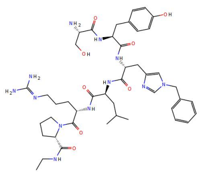	445.2573	445.2558	-3.31
						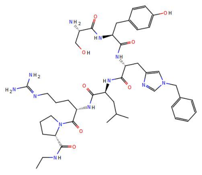	445.2573	445.2558	-3.31
MISMATCH	-122.3	110.0716	110.0713	-2.65	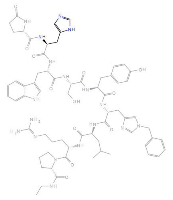		110.0718	110.0718	0.00
MISMATCH	-8.8	549.3630	549.3620	-1.78	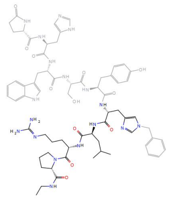		275.1849	275.1849	0.00
MET_MATCH						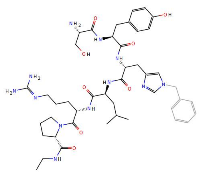	400.2338	400.2323	-3.74
MET_MATCH						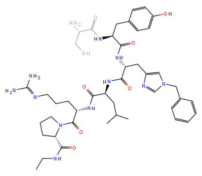	401.7404	401.7398	-1.54
MET_MATCH						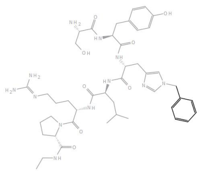	91.0549	91.0542	-7.45
MET_MATCH						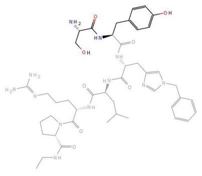	223.1076	223.1077	0.38
MET_MATCH						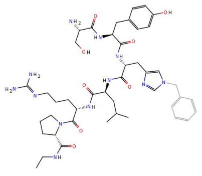	400.2328	400.2323	-1.16
