# Supplementary material for: Software-aided approach to investigate peptide structure and metabolic susceptibility of amide bonds in peptide drugs based on high resolution mass spectrometry
Source: PLoS One. 2017 Nov 1;12(11):e0186461. doi: 10.1371/journal.pone.0186461 (PMC5665424; doi:10.1371/journal.pone.0186461)
Supplement: S1 File — (ZIP) [file pone.0186461.s007.zip › SFiles/S21_File.pdf]

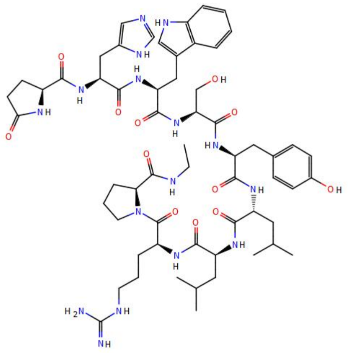

Leuprolide

Property name	Property value
Time	0min, 5min, 15min, 45min, 120min
Instrument	ThermoQAPlus
Matrix	chymotrypsin
Acquisition Mode	ddMS2

Chromatograms

Time=0min

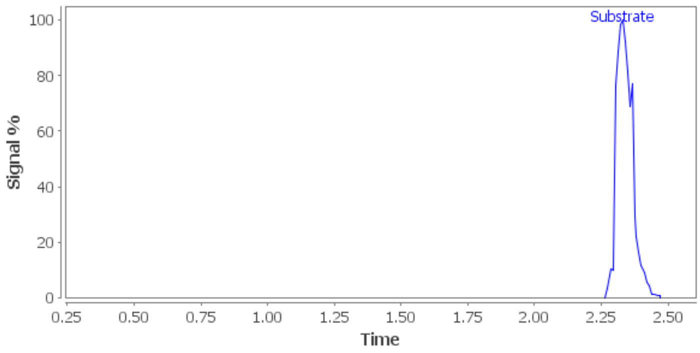

Time=5min

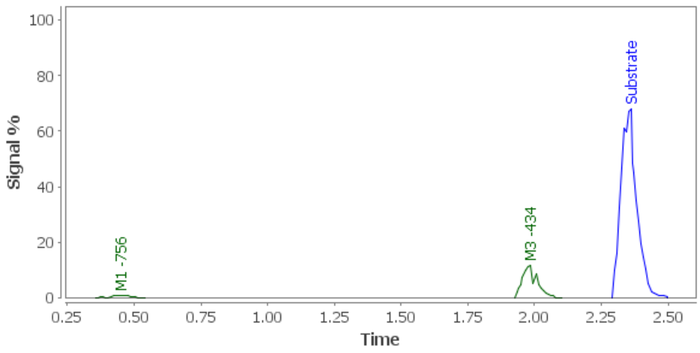

Time=15min

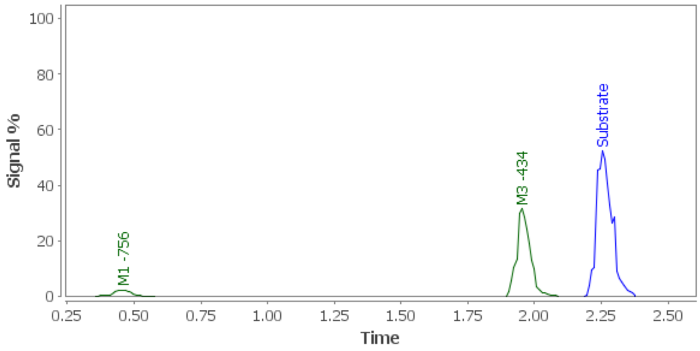

Time=45min

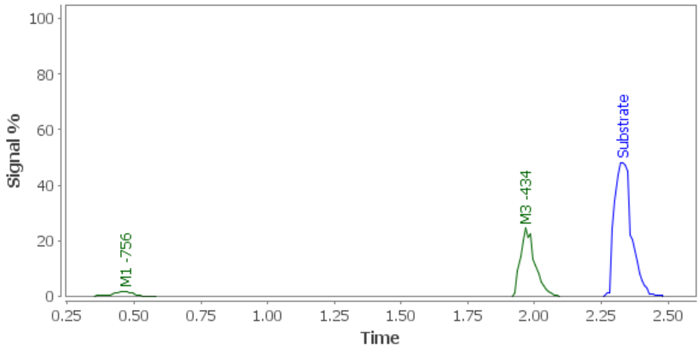

Time=120min

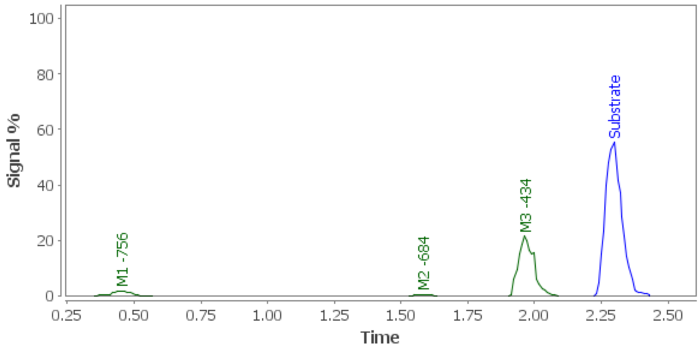

Custom Charts

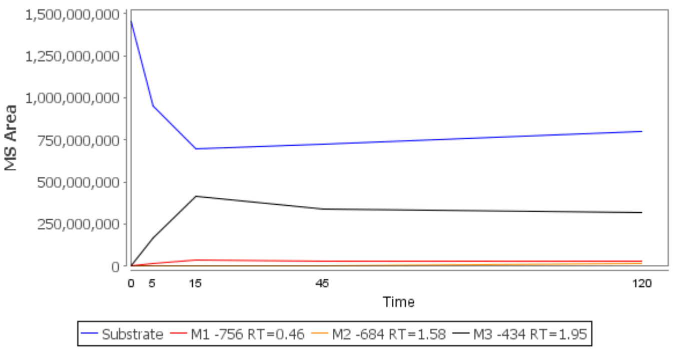

Fragmentation

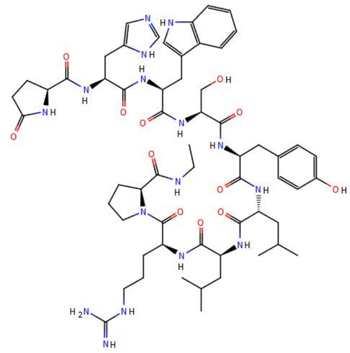

Leuprolide

MS (+) FT

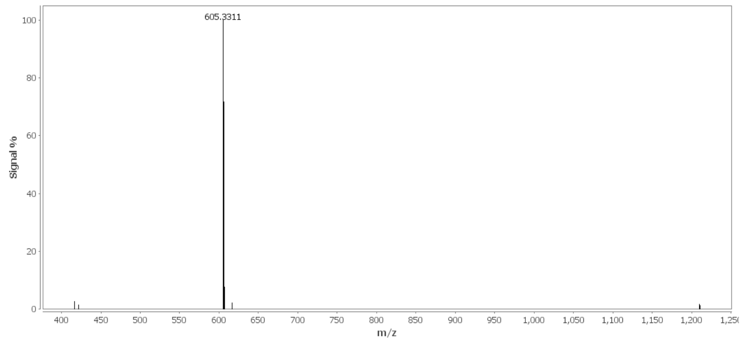

MS (+) FT

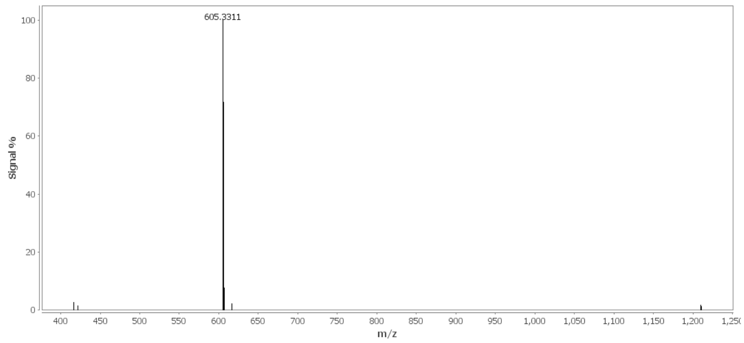

MS2 (+) FT activ = HCD:ce =

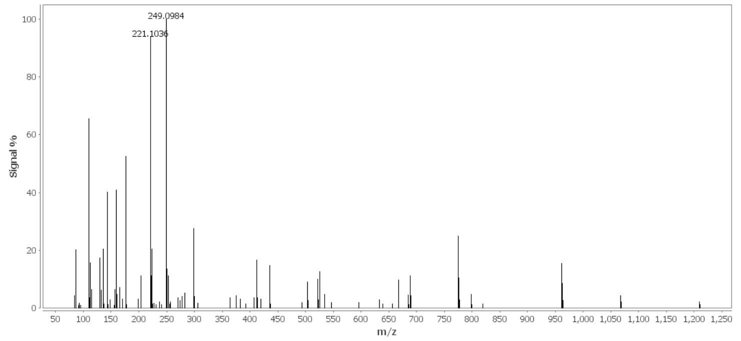

MS2 (+) FT activ = HCD:ce =

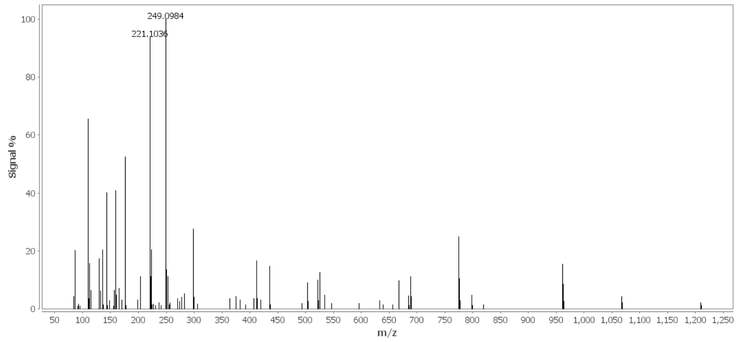

Metabolite: Substrate

Type	score	sub. m/z observed	sub. m/z calculated	sub ppm		met. m/z observed	met. m/z calculated	met. ppm
MATCH	16.0	1209.6547	1209.6527	-1.61	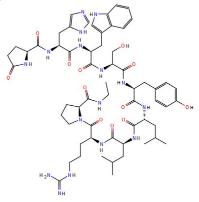 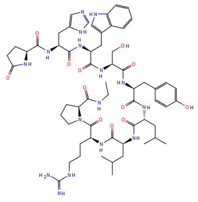	1209.6547	1209.6527	-1.61
MATCH	102.3	1209.6540	1209.6527	-1.08	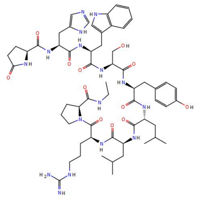 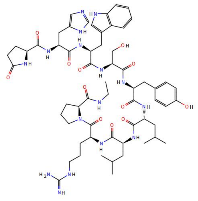	1209.6540	1209.6527	-1.08
MATCH	7.1	1067.5341	1067.5421	7.53	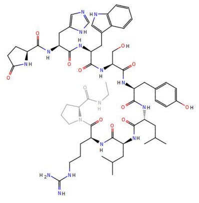 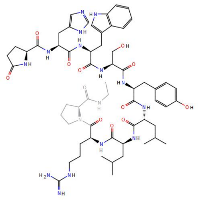	1067.5341	1067.5421	7.53

Metabolite: Substrate

Type	score	sub. m/z observed	sub. m/z calculated	sub ppm			met. m/z observed	met. m/z calculated	met. ppm
MATCH	18.0	961.5625	961.5618	-0.69	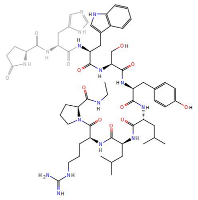	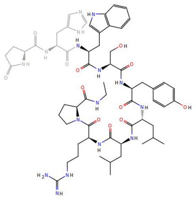	961.5625	961.5618	-0.69
MATCH	6.1	798.3510	798.3570	7.48	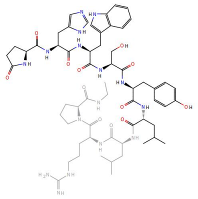	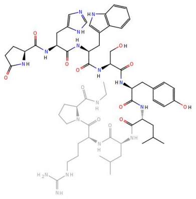	798.3510	798.3570	7.48
MATCH	25.5	685.2697	685.2729	4.58	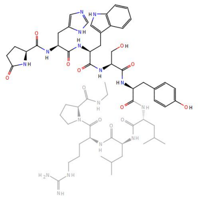	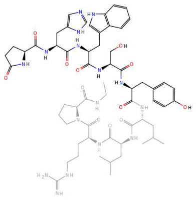	685.2697	685.2729	4.58
MATCH	101.5	657.2782	657.2780	-0.32	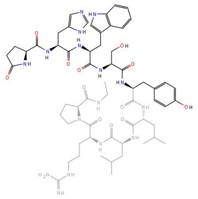	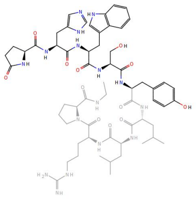	657.2782	657.2780	-0.32
MATCH	200.0	605.3310	605.3300	-1.71	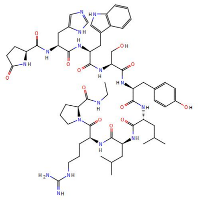	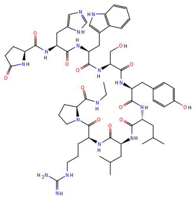	605.3310	605.3300	-1.71
MATCH	12.6	605.3281	605.3300	3.08	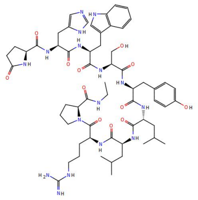	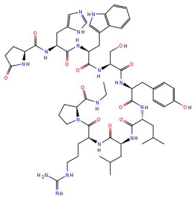	605.3281	605.3300	3.08
MATCH	32.8	525.3880	525.3871	-1.64	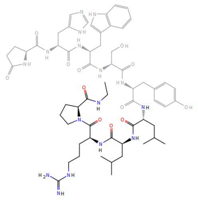	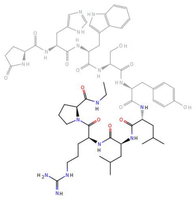	525.3880	525.3871	-1.64
MATCH	11.9	504.1981	504.1990	1.80	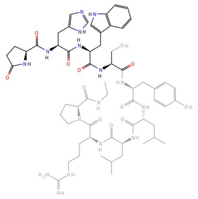	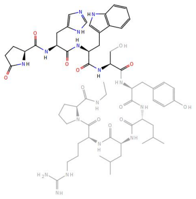	504.1981	504.1990	1.80
MATCH	16.3	494.2143	494.2146	0.72	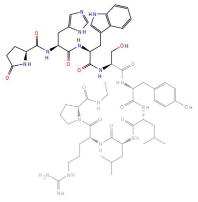	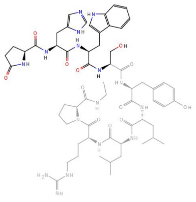	494.2143	494.2146	0.72

Metabolite: Substrate

Type	score	sub. m/z observed	sub. m/z calculated	sub ppm			met. m/z observed	met. m/z calculated	met. ppm
MATCH	37.7	412.3030	412.3031	0.08	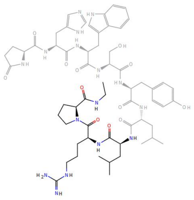	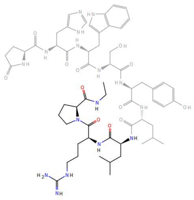	412.3030	412.3031	0.08
MATCH	11.7	383.2763	383.2765	0.52	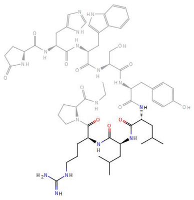	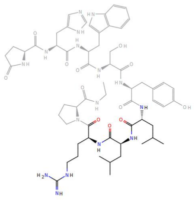	383.2763	383.2765	0.52
MATCH	4.0	366.2497	366.2500	0.82	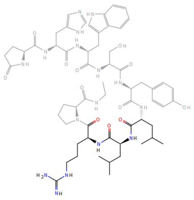	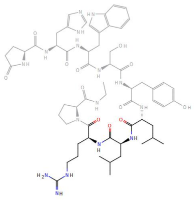	366.2497	366.2500	0.82
MATCH	7.0	364.1853	364.1867	3.96	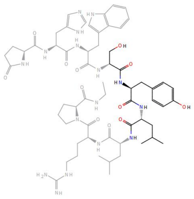	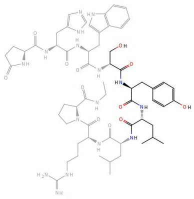	364.1853	364.1867	3.96
MATCH	39.1	299.2198	299.2190	-2.58	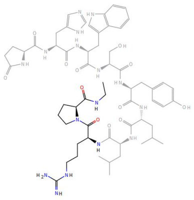	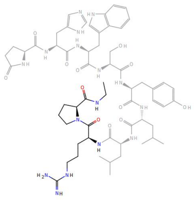	299.2198	299.2190	-2.58
MATCH	8.8	282.1920	282.1925	1.72	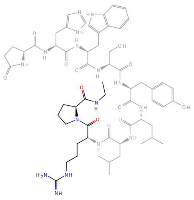	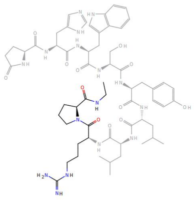	282.1920	282.1925	1.72
MATCH	10.7	270.1927	270.1925	-0.89	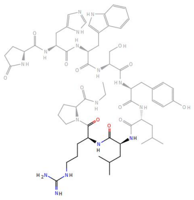	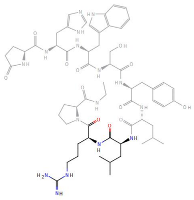	270.1927	270.1925	-0.89
MATCH	19.7	261.1127	261.1164	14.10	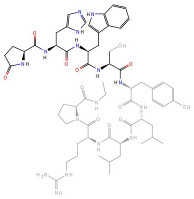	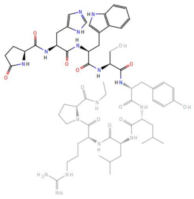	261.1127	261.1164	14.10
MATCH	34.8	253.1661	253.1659	-0.69	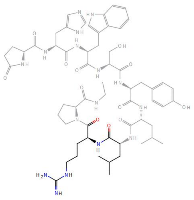	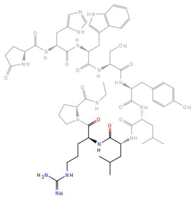	253.1661	253.1659	-0.69

Metabolite: Substrate

Type	score	sub. m/z observed	sub. m/z calculated	sub ppm			met. m/z observed	met. m/z calculated	met. ppm
MATCH	10.3	249.1580	249.1598	7.06	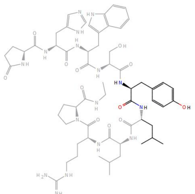	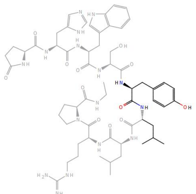	249.1580	249.1598	7.06
MATCH	173.1	249.0985	249.0982	-1.00	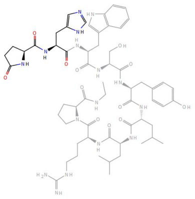	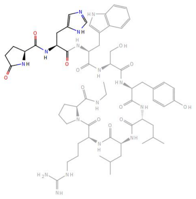	249.0985	249.0982	-1.00
MATCH	5.8	237.1342	237.1346	1.61	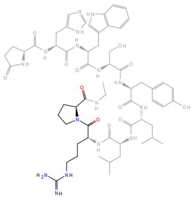	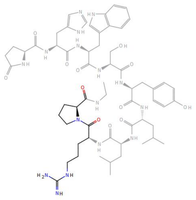	237.1342	237.1346	1.61
MATCH	3.1	227.1747	227.1754	3.25	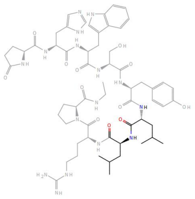	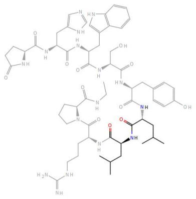	227.1747	227.1754	3.25
MATCH	3.1	227.1747	227.1754	3.25	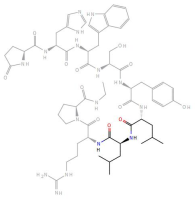	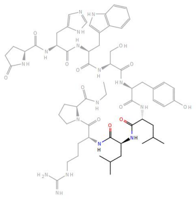	227.1747	227.1754	3.25
MATCH	177.0	221.1036	221.1033	-1.24	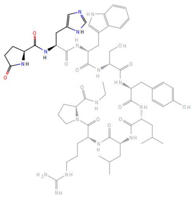	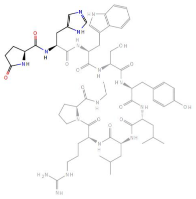	221.1036	221.1033	-1.24
MATCH	2.2	209.1401	209.1397	-2.15	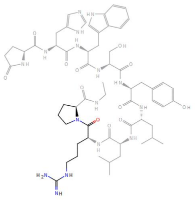	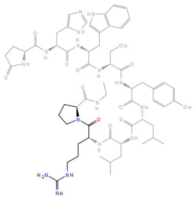	209.1401	209.1397	-2.15
MATCH	9.9	199.1808	199.1805	-1.40	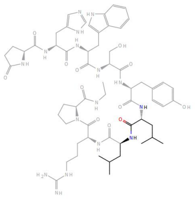	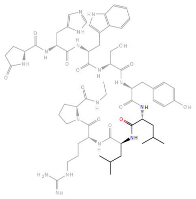	199.1808	199.1805	-1.40
MATCH	14.2	166.0612	166.0611	-0.66	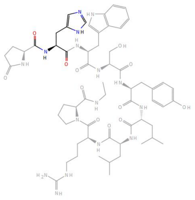	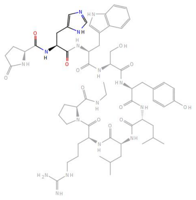	166.0612	166.0611	-0.66

Metabolite: Substrate

Type	score	sub. m/z observed	sub. m/z calculated	sub ppm			met. m/z observed	met. m/z calculated	met. ppm
MATCH	58.6	159.0919	159.0917	-1.42	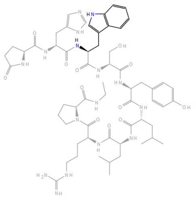	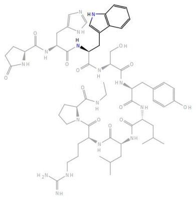	159.0919	159.0917	-1.42
MATCH	11.7	157.1087	157.1084	-1.79	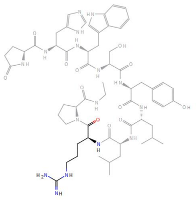	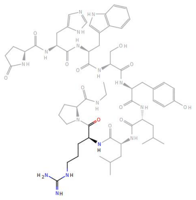	157.1087	157.1084	-1.79
MATCH	76.9	143.1182	143.1179	-2.40	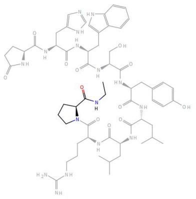	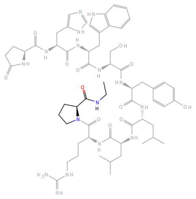	143.1182	143.1179	-2.40
MATCH	88.1	136.0760	136.0757	-2.23	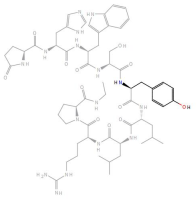	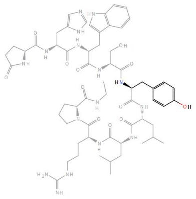	136.0760	136.0757	-2.23
MATCH	15.1	115.0871	115.0866	-4.03	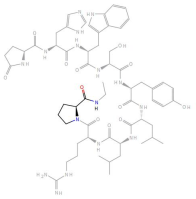	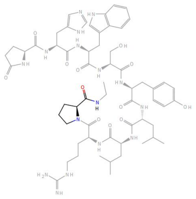	115.0871	115.0866	-4.03
MATCH	34.8	112.0875	112.0869	-4.76	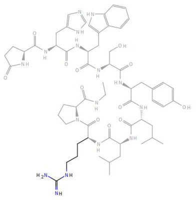	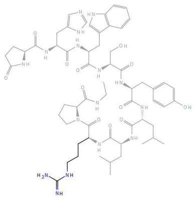	112.0875	112.0869	-4.76
MATCH	172.3	110.0718	110.0713	-5.11	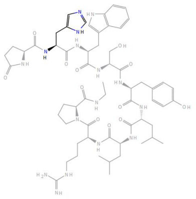	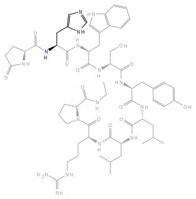	110.0718	110.0713	-5.11
MATCH	4.9	93.0453	93.0447	-6.26	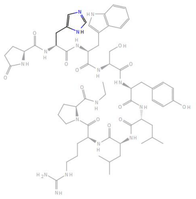	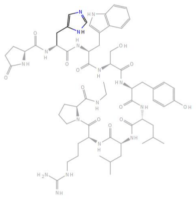	93.0453	93.0447	-6.26
MATCH	4.4	91.0549	91.0522	-28.9	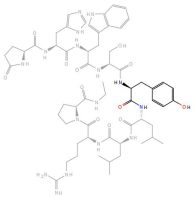	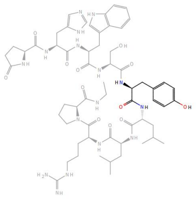	91.0549	91.0522	-28.9

Metabolite: Substrate

Type	score	sub. m/z observed	sub. m/z calculated	sub ppm			met. m/z observed	met. m/z calculated	met. ppm
MATCH	120.8	86.0972	86.0964	-9.20	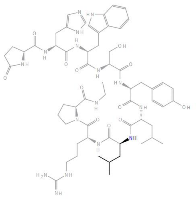	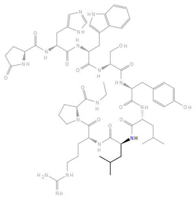	86.0972	86.0964	-9.20
MATCH	82.9	86.0972	86.0964	-9.20	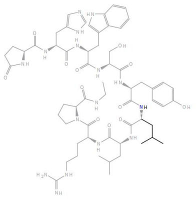	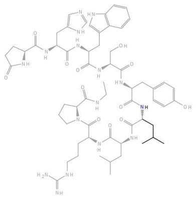	86.0972	86.0964	-9.20
MATCH	12.2	84.0455	84.0444	-12.8	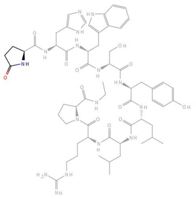	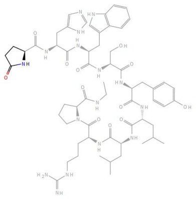	84.0455	84.0444	-12.8

MS (+) FT

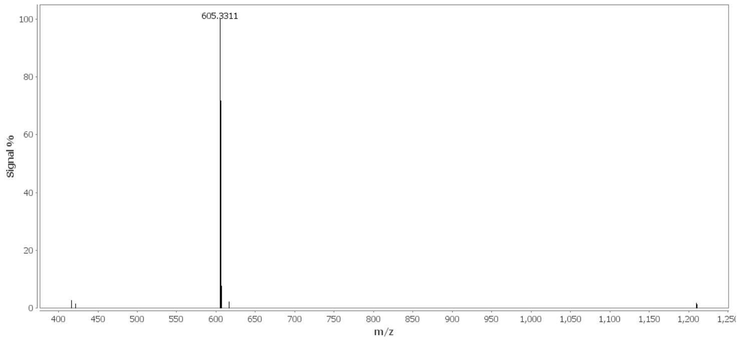

MS (+) FT

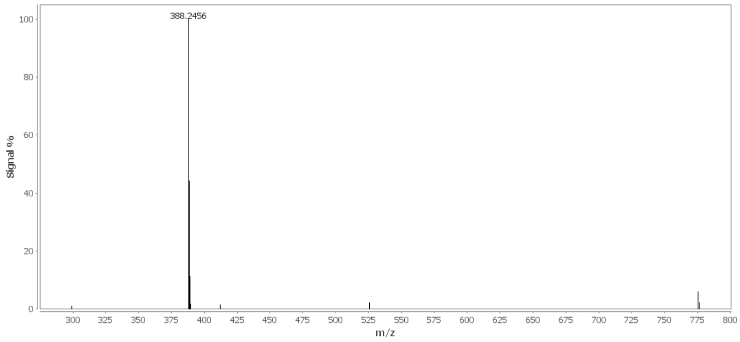

MS2 (+) FT activ = HCD:ce =

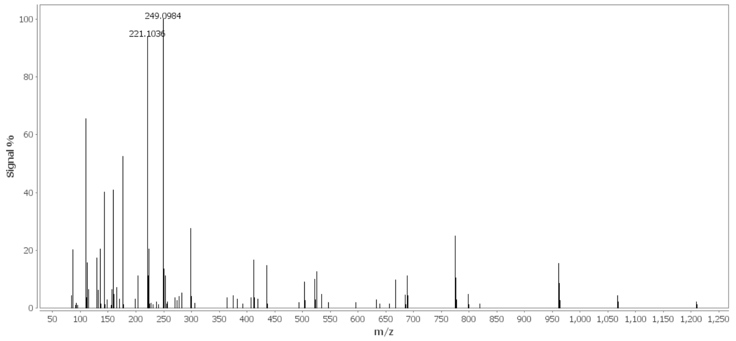

MS2 (+) FT activ = HCD:ce =

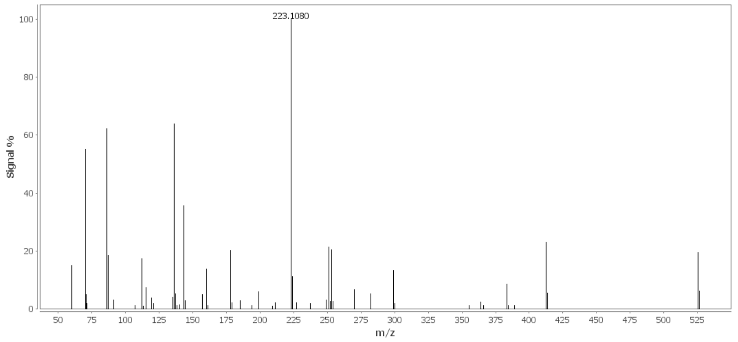

Metabolite: M3 -434 RT=1.95

Type	score	sub. m/z observed	sub. m/z calculated	sub ppm			met. m/z observed	met. m/z calculated	met. ppm
MATCH	200.0	605.3310	605.3300	-1.71	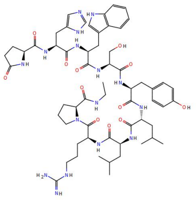	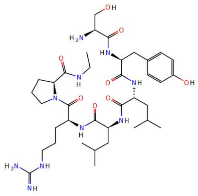	388.2456	388.2449	-1.79

Metabolite: M3 -434 RT=1.95

Type	score	sub. m/z observed	sub. m/z calculated	sub ppm			met. m/z observed	met. m/z calculated	met. ppm
MATCH	200.0	605.3310	605.3300	-1.71	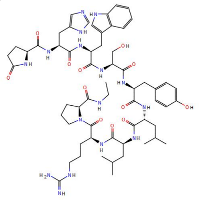	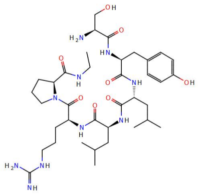	388.2456	388.2449	-1.79
MATCH	105.9	605.3310	605.3300	-1.71	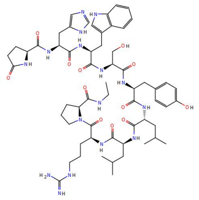	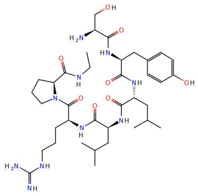	775.4840	775.4825	-2.02
MATCH	105.9	605.3310	605.3300	-1.71	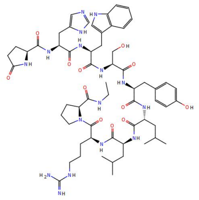	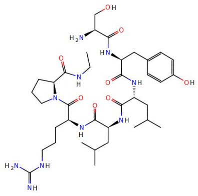	775.4840	775.4825	-2.02
MATCH	102.3	1209.6540	1209.6527	-1.08	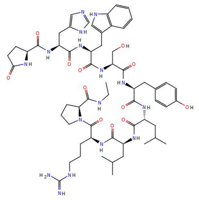	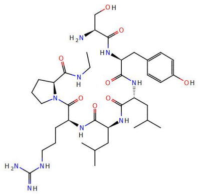	388.2456	388.2449	-1.79
MATCH	102.3	1209.6540	1209.6527	-1.08	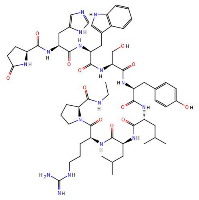	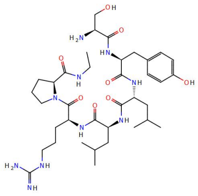	388.2456	388.2449	-1.79
MATCH	8.2	1209.6540	1209.6527	-1.08	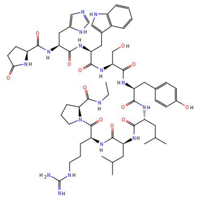	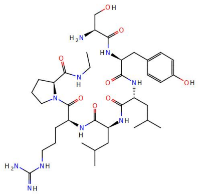	775.4840	775.4825	-2.02
MATCH	8.2	1209.6540	1209.6527	-1.08	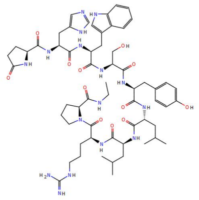	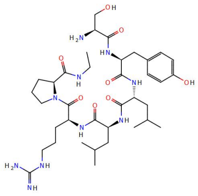	775.4840	775.4825	-2.02
MATCH	82.9	86.0972	86.0964	-9.20	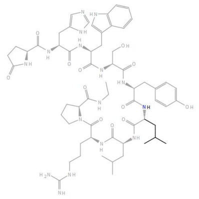	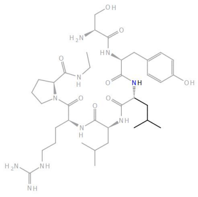	86.0972	86.0964	-9.38
MATCH	82.9	86.0972	86.0964	-9.20	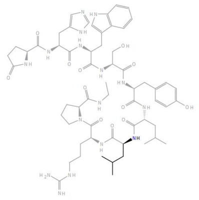	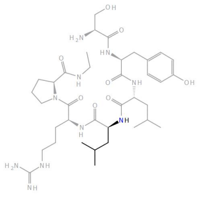	86.0972	86.0964	-9.38

Metabolite: M3 -434 RT=1.95

Type	score	sub. m/z observed	sub. m/z calculated	sub ppm			met. m/z observed	met. m/z calculated	met. ppm
MATCH	4.4	91.0549	91.0522	-28.9	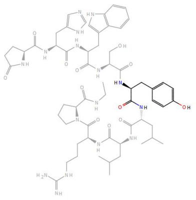	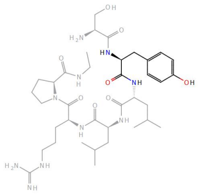	91.0550	91.0522	-30.6
MATCH	34.8	112.0875	112.0869	-4.76	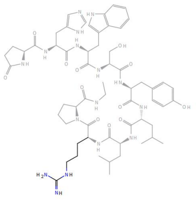	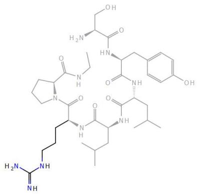	112.0874	112.0869	-4.67
MATCH	15.1	115.0871	115.0866	-4.03	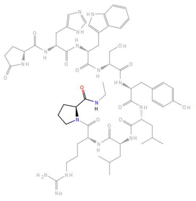	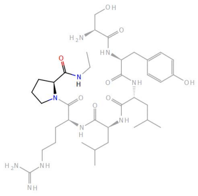	115.0871	115.0866	-4.33
MATCH	88.1	136.0760	136.0757	-2.23	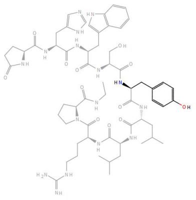	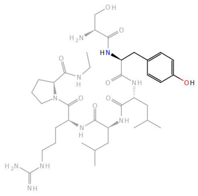	136.0760	136.0757	-2.44
MATCH	76.9	143.1182	143.1179	-2.40	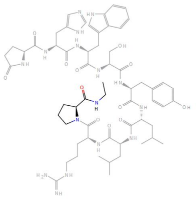	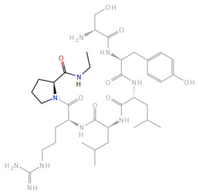	143.1182	143.1179	-2.05
MATCH	11.7	157.1087	157.1084	-1.79	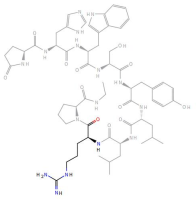	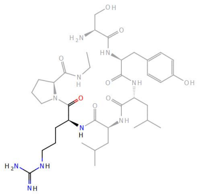	157.1086	157.1084	-1.61
MATCH	9.9	199.1808	199.1805	-1.40	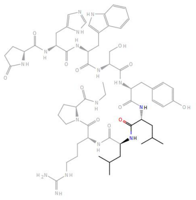	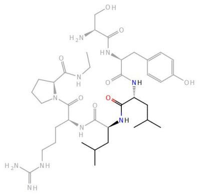	199.1810	199.1805	-2.74
MATCH	2.2	209.1401	209.1397	-2.15	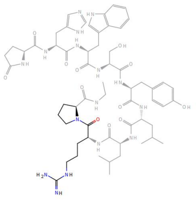	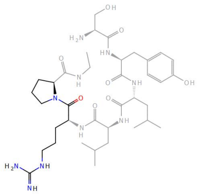	209.1401	209.1397	-2.04
MATCH	3.1	227.1747	227.1754	3.25	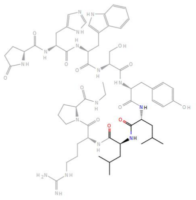	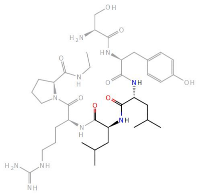	227.1758	227.1754	-1.82

Metabolite: M3 -434 RT=1.95

Type	score	sub. m/z observed	sub. m/z calculated	sub ppm			met. m/z observed	met. m/z calculated	met. ppm
MATCH	3.1	227.1747	227.1754	3.25	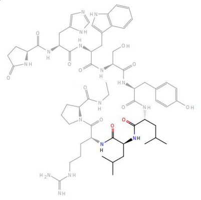	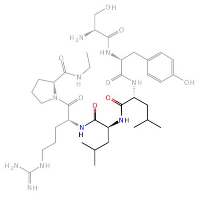	227.1758	227.1754	-1.82
MATCH	5.8	237.1342	237.1346	1.61	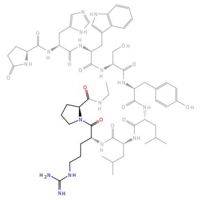	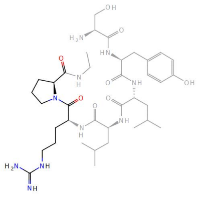	237.1346	237.1346	-0.06
MATCH	10.3	249.1580	249.1598	7.06	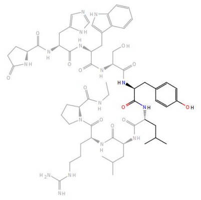	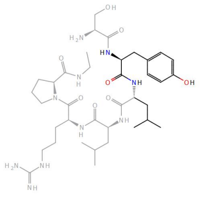	249.1599	249.1598	-0.70
MATCH	32.8	253.1661	253.1659	-0.69	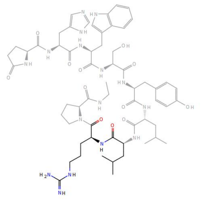	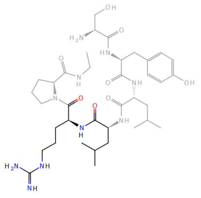	253.1662	253.1659	-1.14
MATCH	19.7	261.1127	261.1164	14.10	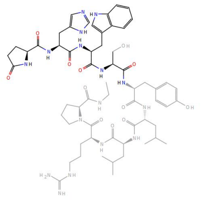	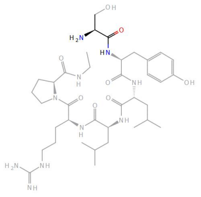	87.0561	87.0553	-9.02
MATCH	10.2	270.1927	270.1925	-0.89	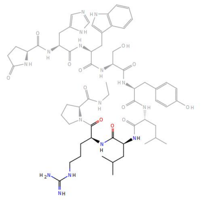	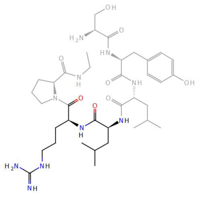	270.1926	270.1925	-0.49
MATCH	8.8	282.1920	282.1925	1.72	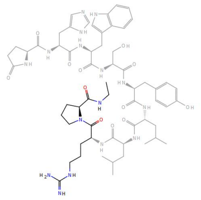	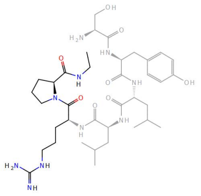	282.1927	282.1925	-0.80
MATCH	39.0	299.2198	299.2190	-2.58	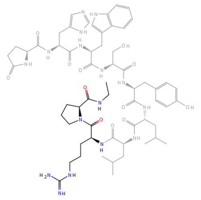	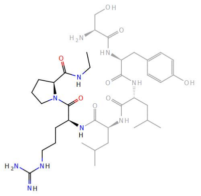	299.2195	299.2190	-1.66
MATCH	7.0	364.1853	364.1867	3.96	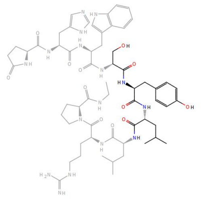	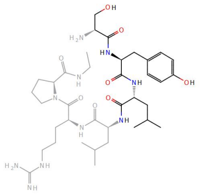	364.1855	364.1867	3.35

Metabolite: M3 -434 RT=1.95

Type	score	sub. m/z observed	sub. m/z calculated	sub ppm			met. m/z observed	met. m/z calculated	met. ppm
						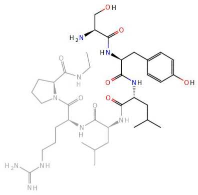	364.1855	364.1867	3.35
MATCH	4.0	366.2497	366.2500	0.82	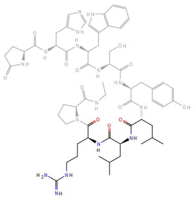	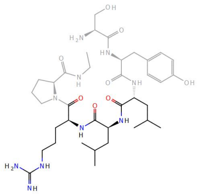	366.2495	366.2500	1.16
MATCH	11.7	383.2763	383.2765	0.52	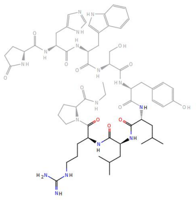	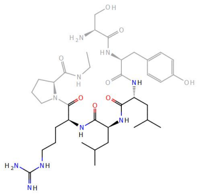	383.2770	383.2765	-1.36
MATCH	37.0	412.3030	412.3031	0.08	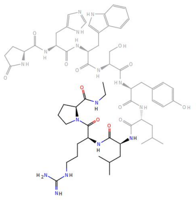	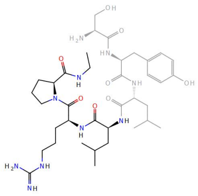	412.3034	412.3031	-0.76
MATCH	16.3	494.2143	494.2146	0.72	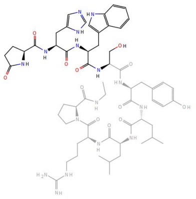	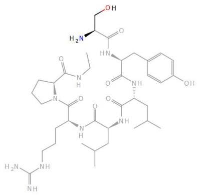	60.0454	60.0444	-16.7
MATCH	11.9	504.1981	504.1990	1.80	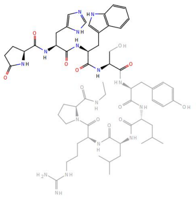	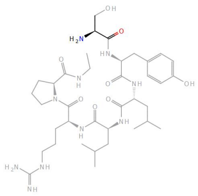	70.0297	70.0287	-13.5
MATCH	32.8	525.3880	525.3871	-1.64	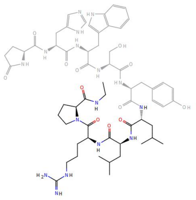	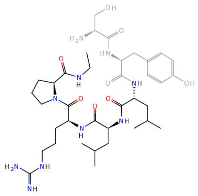	525.3879	525.3871	-1.45
MATCH	101.5	657.2782	657.2780	-0.32	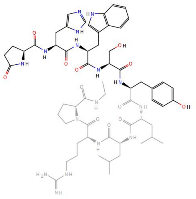	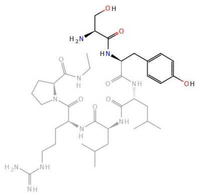	223.1080	223.1077	-1.21
MATCH	25.5	685.2697	685.2729	4.58	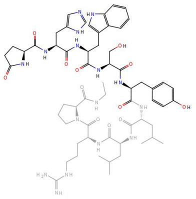	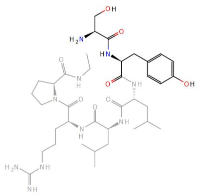	251.1029	251.1026	-1.10

Metabolite: M3 -434 RT=1.95

Type	score	sub. m/z observed	sub. m/z calculated	sub ppm			met. m/z observed	met. m/z calculated	met. ppm
MATCH	6.1	798.3510	798.3570	7.48	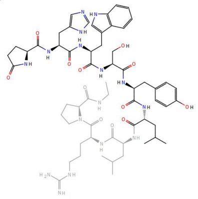	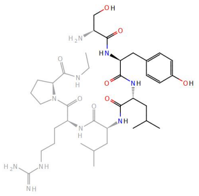	364.1855	364.1867	3.35
						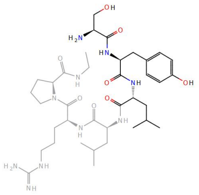	364.1855	364.1867	3.35
MET_MATCH						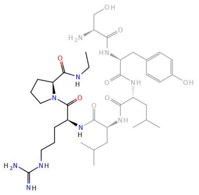	299.2196	299.2190	-1.87
MET_MATCH						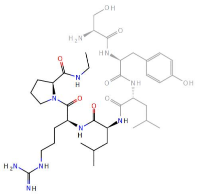	412.3041	412.3031	-2.42
MET_MATCH						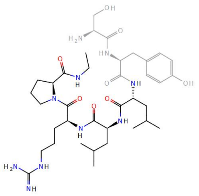	525.3876	525.3871	-0.94

MS (+) FT

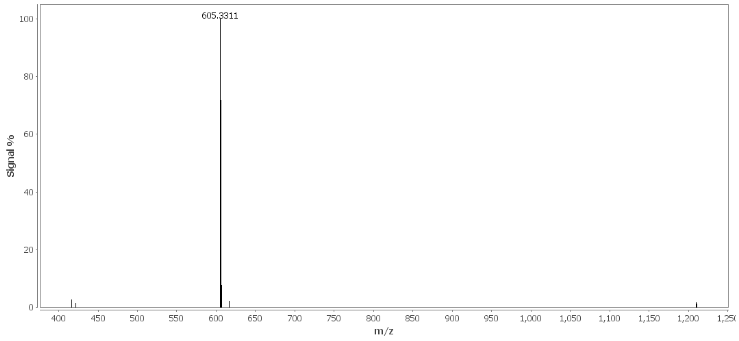

MS (+) FT

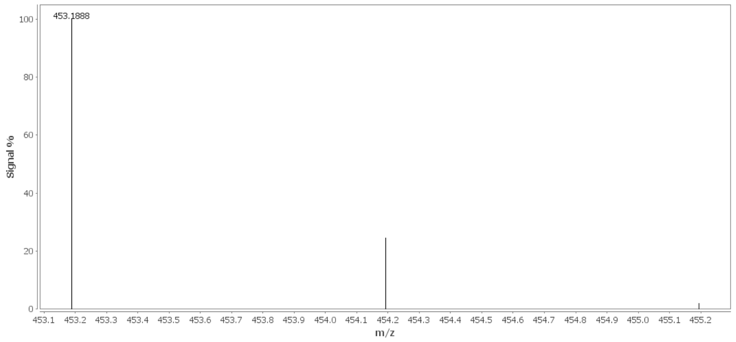

MS2 (+) FT activ = HCD:ce =

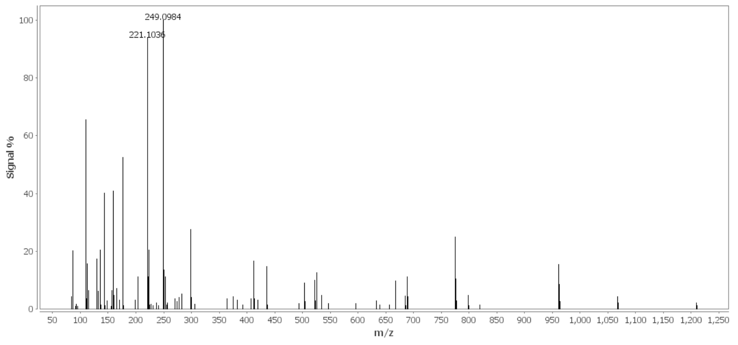

MS2 (+) FT activ = HCD:ce =

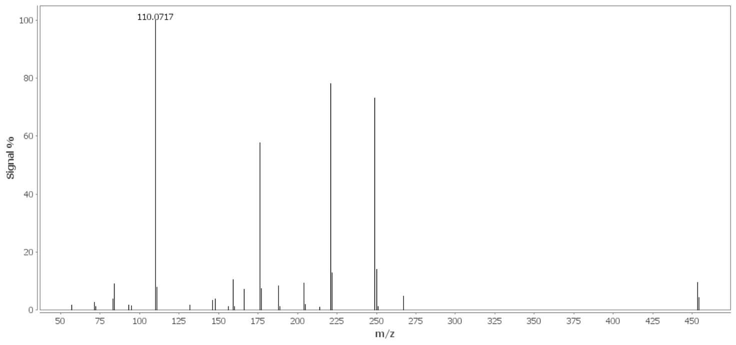

Metabolite: M1 -756 RT=0.46

Type	score	sub. m/z observed	sub. m/z calculated	sub ppm			met. m/z observed	met. m/z calculated	met. ppm
MATCH	200.0	605.3310	605.3300	-1.71	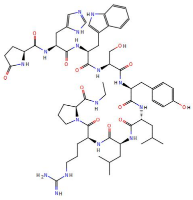	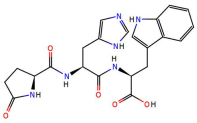	453.1888	453.1881	-1.51
						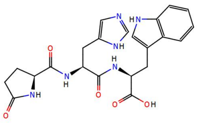	453.1888	453.1881	-1.51
MATCH	102.3	1209.6540	1209.6527	-1.08	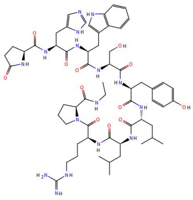	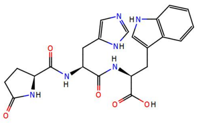	453.1888	453.1881	-1.51
						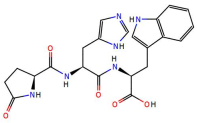	453.1888	453.1881	-1.51
MATCH	12.2	84.0455	84.0444	-12.8	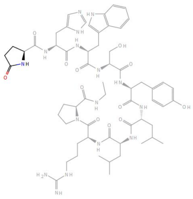	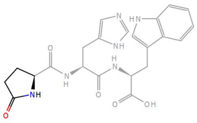	84.0452	84.0444	-9.38
MATCH	4.9	93.0453	93.0447	-6.26	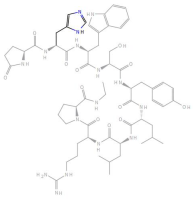	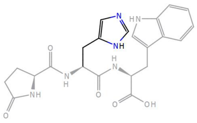	93.0454	93.0447	-7.70
MATCH	2.6	95.0612	95.0604	-8.95	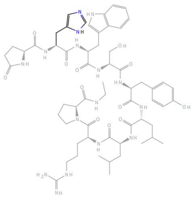	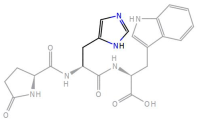	95.0611	95.0604	-7.87
MATCH	172.3	110.0718	110.0713	-5.11	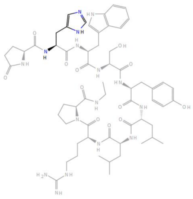	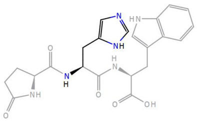	110.0717	110.0713	-4.22
MATCH	58.6	159.0919	159.0917	-1.42	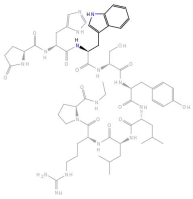	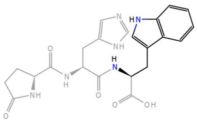	159.0918	159.0917	-0.51

Metabolite: M1 -756 RT=0.46

Type	score	sub. m/z observed	sub. m/z calculated	sub ppm			met. m/z observed	met. m/z calculated	met. ppm
MATCH	14.2	166.0612	166.0611	-0.66	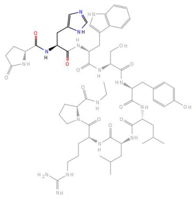	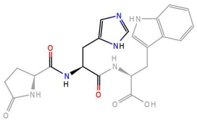	166.0613	166.0611	-1.21
MATCH	177.0	221.1036	221.1033	-1.24	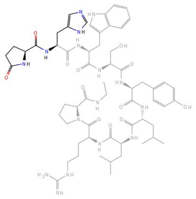	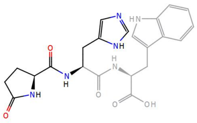	221.1034	221.1033	-0.26
MATCH	173.1	249.0985	249.0982	-1.00	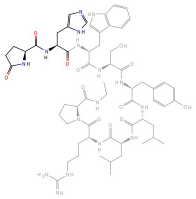	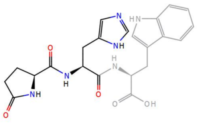	249.0983	249.0982	-0.27
MATCH	12.6	605.3281	605.3300	3.08	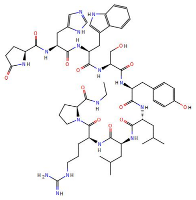	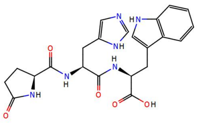	453.1884	453.1881	-0.76
						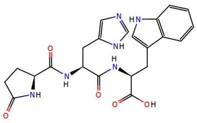	453.1884	453.1881	-0.76
MATCH	18.0	961.5625	961.5618	-0.69	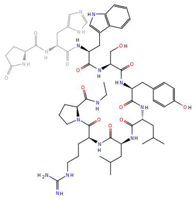	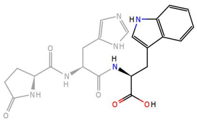	205.0977	205.0972	-2.50
MATCH	16.0	1209.6547	1209.6527	-1.61	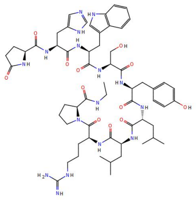	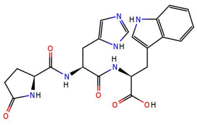	453.1884	453.1881	-0.76
						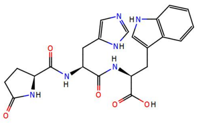	453.1884	453.1881	-0.76
MET_MATCH						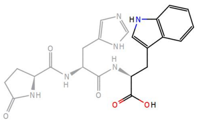	188.0705	188.0706	0.32

MS (+) FT

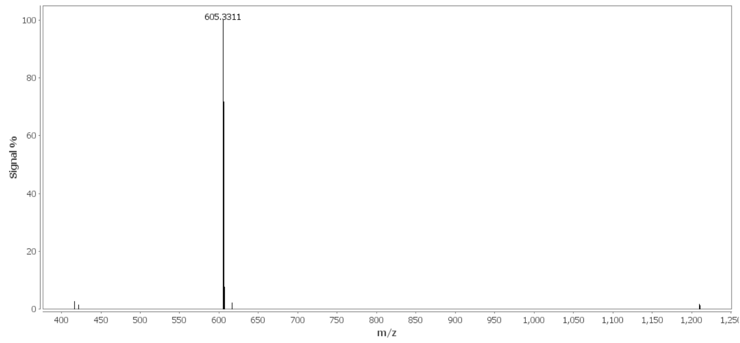

MS (+) FT

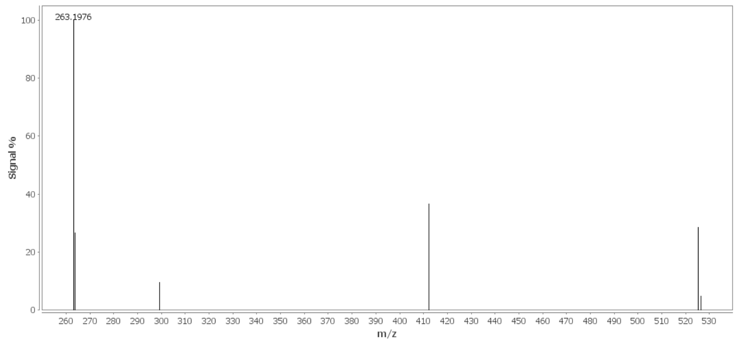

MS2 (+) FT activ = HCD:ce =

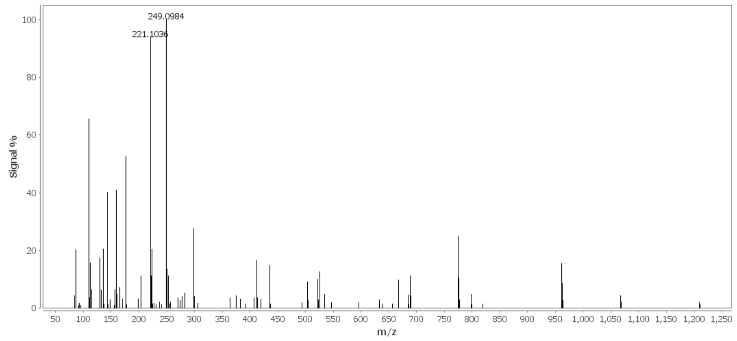

MS2 (+) FT activ = HCD:ce =

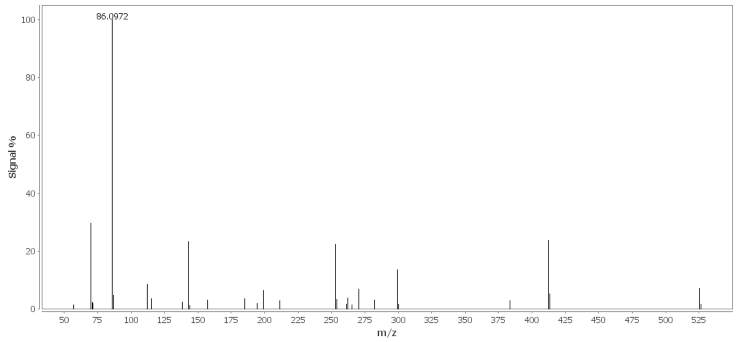

Metabolite: M2 -684 RT=1.58

Type	score	sub. m/z observed	sub. m/z calculated	sub ppm		met. m/z observed	met. m/z calculated	met. ppm
MATCH	200.0	605.3310	605.3300	-1.71	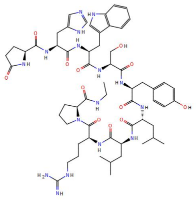 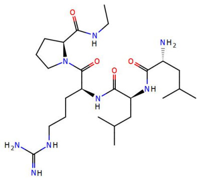	263.1976	263.1972	-1.67
MATCH	200.0	605.3310	605.3300	-1.71	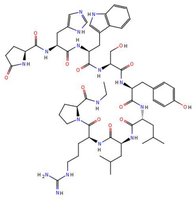 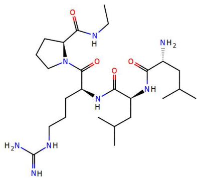	263.1976	263.1972	-1.67
MATCH	128.4	605.3310	605.3300	-1.71	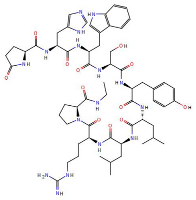 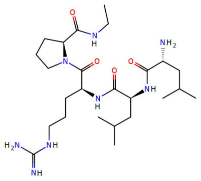	525.3881	525.3871	-1.78
MATCH	128.4	605.3310	605.3300	-1.71	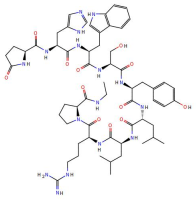 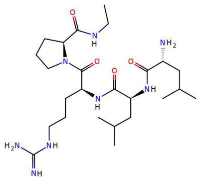	525.3881	525.3871	-1.78
MATCH	102.3	1209.6540	1209.6527	-1.08	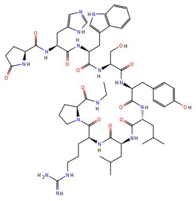 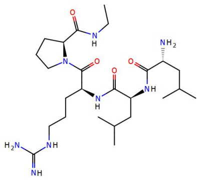	263.1976	263.1972	-1.67

Metabolite: M2 -684 RT=1.58

Type	score	sub. m/z observed	sub. m/z calculated	sub ppm			met. m/z observed	met. m/z calculated	met. ppm
MATCH	102.3	1209.6540	1209.6527	-1.08	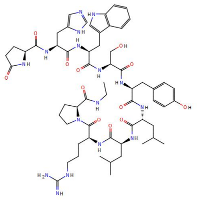	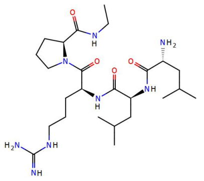	263.1976	263.1972	-1.67
MATCH	30.7	1209.6540	1209.6527	-1.08	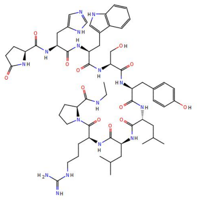	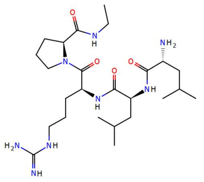	525.3881	525.3871	-1.78
MATCH	30.7	1209.6540	1209.6527	-1.08	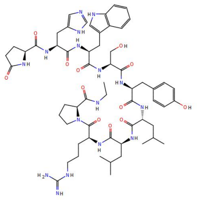	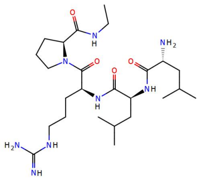	525.3881	525.3871	-1.78
MATCH	120.8	86.0972	86.0964	-9.20	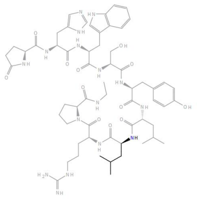	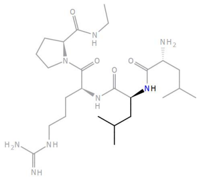	86.0972	86.0964	-8.93
MATCH	26.2	112.0875	112.0869	-4.76	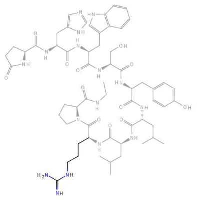	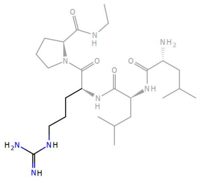	112.0875	112.0869	-4.77
MATCH	11.2	115.0871	115.0866	-4.03	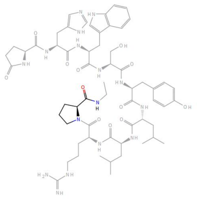	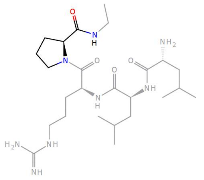	115.0870	115.0866	-3.33
MATCH	64.4	143.1182	143.1179	-2.40	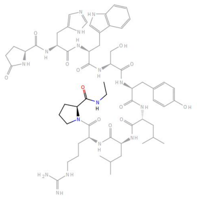	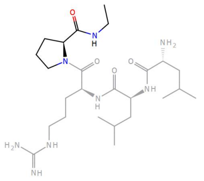	143.1182	143.1179	-1.83
MATCH	9.7	157.1087	157.1084	-1.79	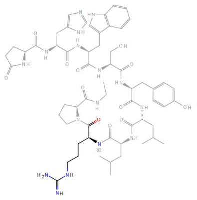	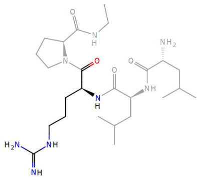	157.1089	157.1084	-3.23
MATCH	34.8	253.1661	253.1659	-0.69	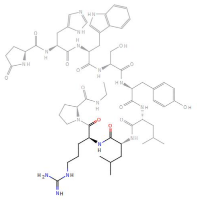	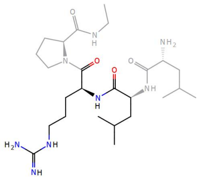	253.1660	253.1659	-0.32

Metabolite: M2 -684 RT=1.58

Type	score	sub. m/z observed	sub. m/z calculated	sub ppm			met. m/z observed	met. m/z calculated	met. ppm
MATCH	10.7	270.1927	270.1925	-0.89	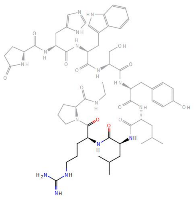	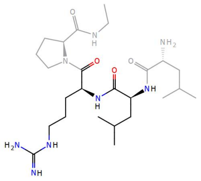	270.1927	270.1925	-0.99
MATCH	6.6	282.1920	282.1925	1.72	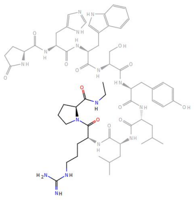	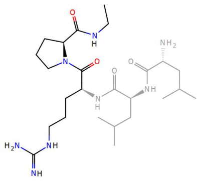	282.1924	282.1925	0.11
MATCH	39.1	299.2198	299.2190	-2.58	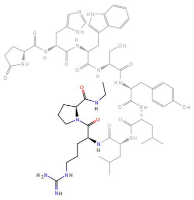	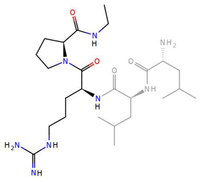	299.2194	299.2190	-1.20
MATCH	37.7	412.3030	412.3031	0.08	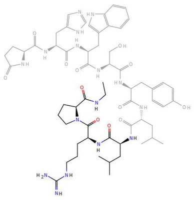	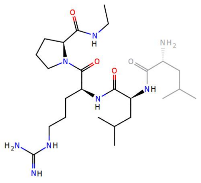	412.3035	412.3031	-1.05
MATCH	10.4	605.3281	605.3300	3.08	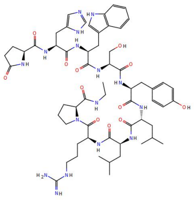	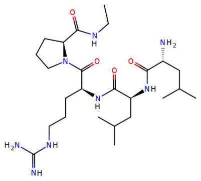	525.3876	525.3871	-0.90
						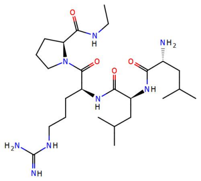	525.3876	525.3871	-0.90
MATCH	7.1	1067.5341	1067.5421	7.53	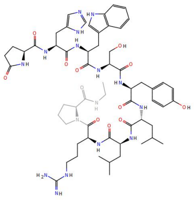	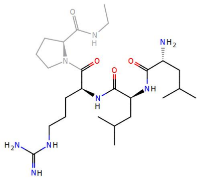	383.2770	383.2765	-1.24
MATCH	13.8	1209.6547	1209.6527	-1.61	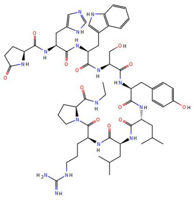	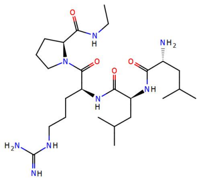	525.3876	525.3871	-0.90
						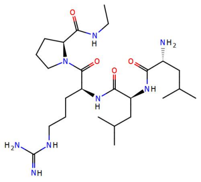	525.3876	525.3871	-0.90

Metabolite: M2 -684 RT=1.58

Type	score	sub. m/z observed	sub. m/z calculated	sub ppm		met. m/z observed	met. m/z calculated	met. ppm
MET_MATCH					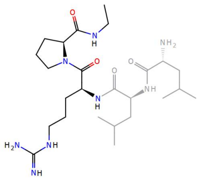	299.2198	299.2190	-2.72
MET_MATCH					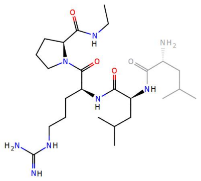	412.3037	412.3031	-1.46
MET_MATCH					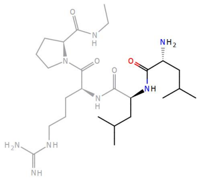	199.1808	199.1805	-1.48
