# Supplementary material for: Software-aided approach to investigate peptide structure and metabolic susceptibility of amide bonds in peptide drugs based on high resolution mass spectrometry
Source: PLoS One. 2017 Nov 1;12(11):e0186461. doi: 10.1371/journal.pone.0186461 (PMC5665424; doi:10.1371/journal.pone.0186461)
Supplement: S1 File — (ZIP) [file pone.0186461.s007.zip › SFiles/S22_File.pdf]

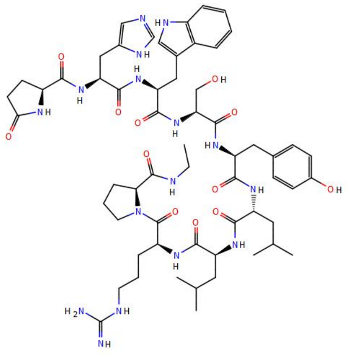

Leuprolide

Property name	Property value
Time	0min, 5min, 15min, 45min, 120min
Instrument	ThermoQAPlus
Matrix	trypsin
Acquisition Mode	ddMS2

Chromatograms

Time=0min

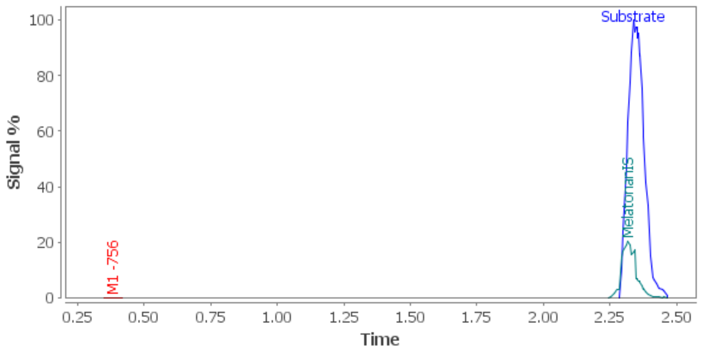

Time=5min

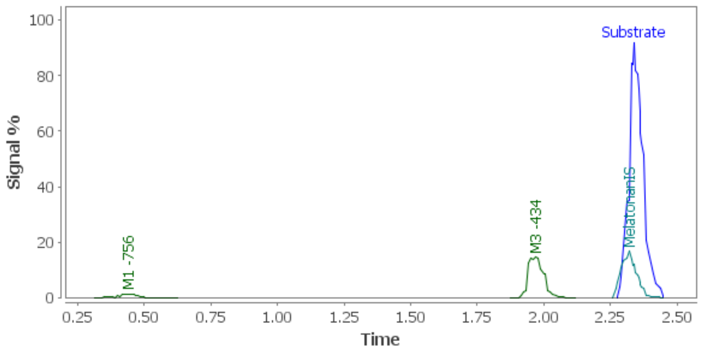

Time=15min

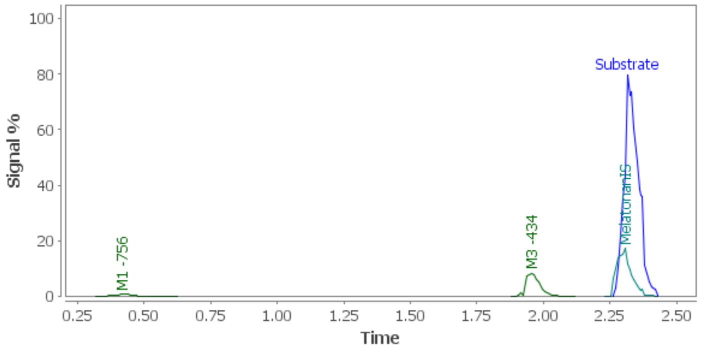

Time=45min

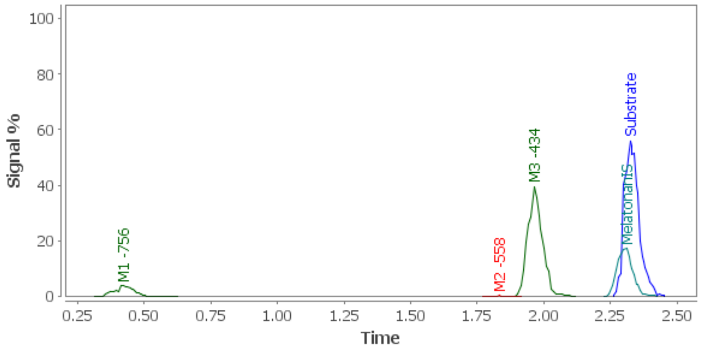

Time=120min

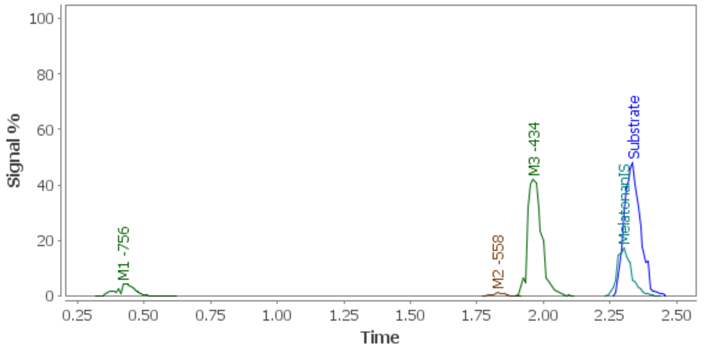

Custom Charts

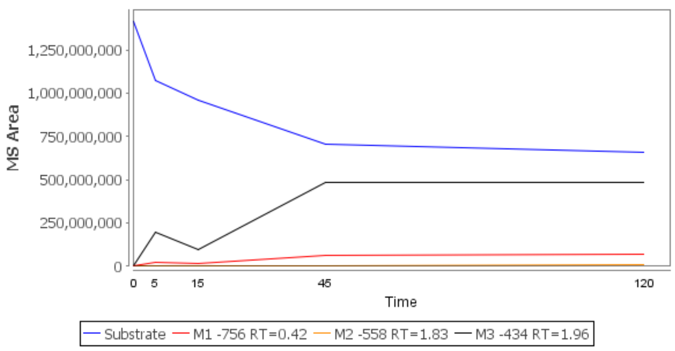

Fragmentation

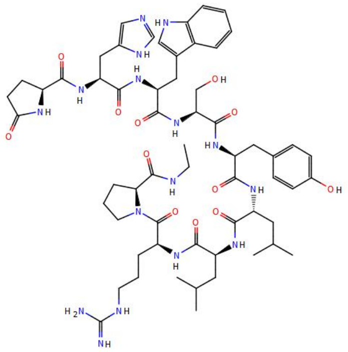

Leuprolide

MS (+) FT

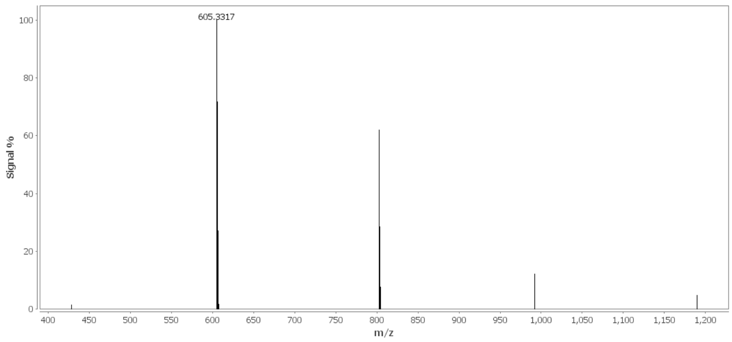

MS (+) FT

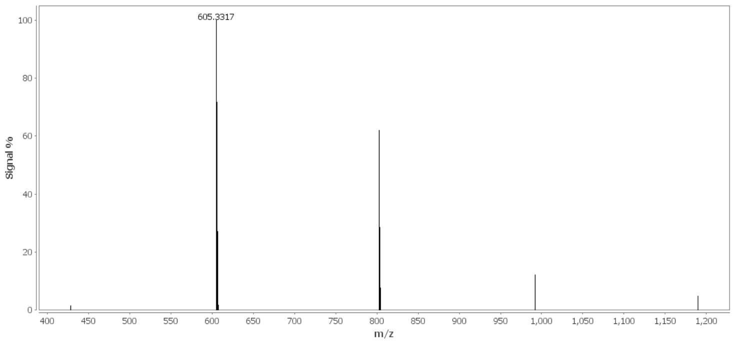

MS2 (+) FT activ = HCD:ce =

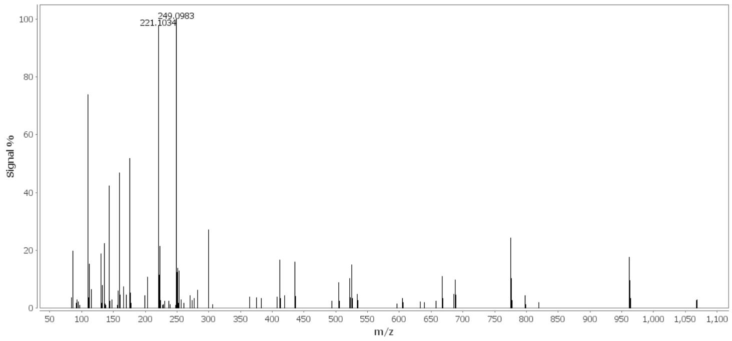

MS2 (+) FT activ = HCD:ce =

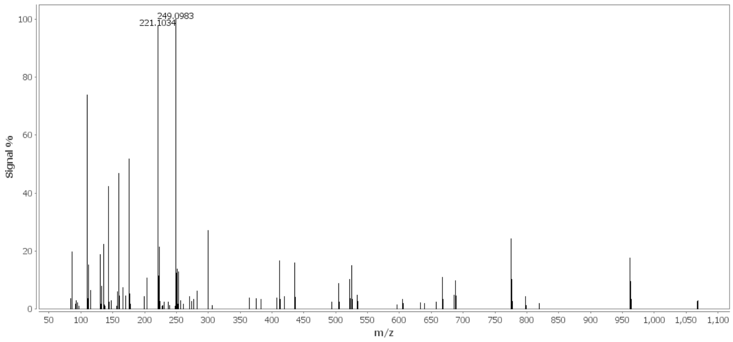

Metabolite: Substrate

Type	score	sub. m/z observed	sub. m/z calculated	sub ppm			met. m/z observed	met. m/z calculated	met. ppm
MATCH	19.5	961.5611	961.5618	0.69	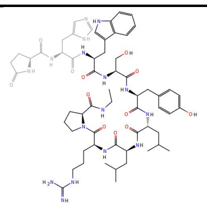	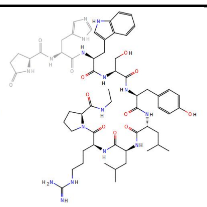	961.5611	961.5618	0.69
MATCH	7.3	798.3551	798.3570	2.37	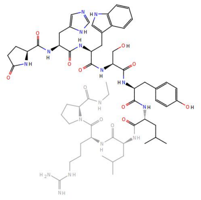	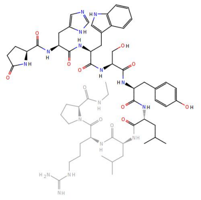	798.3551	798.3570	2.37
MATCH	11.8	688.4504	688.4505	0.04	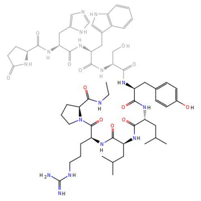	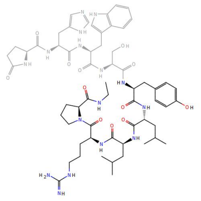	688.4504	688.4505	0.04

Metabolite: Substrate

Type	score	sub. m/z observed	sub. m/z calculated	sub ppm			met. m/z observed	met. m/z calculated	met. ppm
MATCH	23.4	685.2729	685.2729	-0.02	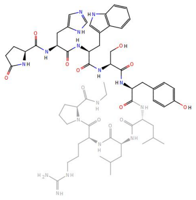	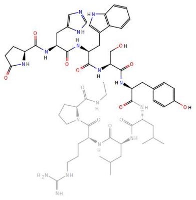	685.2729	685.2729	-0.02
MATCH	102.5	657.2747	657.2780	4.97	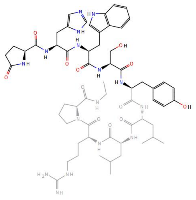	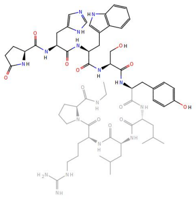	657.2747	657.2780	4.97
MATCH	200.0	605.3317	605.3300	-2.87	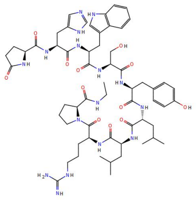	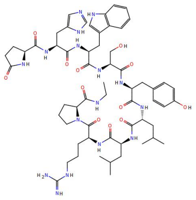	605.3317	605.3300	-2.87
MATCH	14.7	605.3271	605.3300	4.78	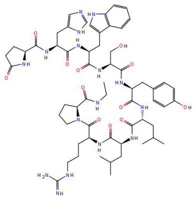	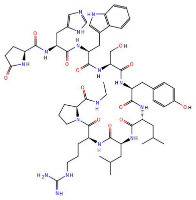	605.3271	605.3300	4.78
MATCH	66.0	525.3865	525.3871	1.18	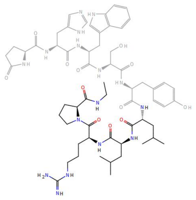	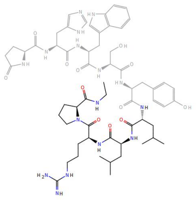	525.3865	525.3871	1.18
MATCH	12.0	504.1999	504.1990	-1.84	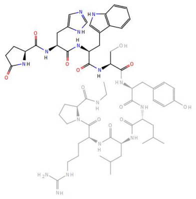	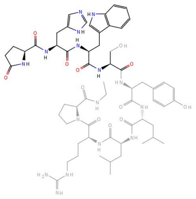	504.1999	504.1990	-1.84
MATCH	39.8	494.2144	494.2146	0.49	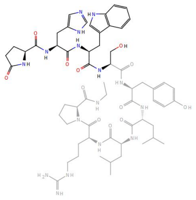	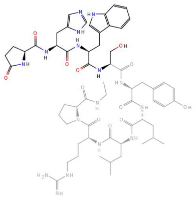	494.2144	494.2146	0.49
MATCH	39.3	412.3038	412.3031	-1.71	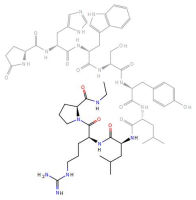	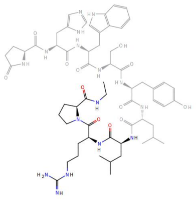	412.3038	412.3031	-1.71
MATCH	11.8	383.2752	383.2765	3.51	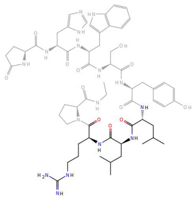	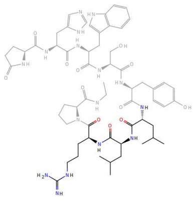	383.2752	383.2765	3.51

Metabolite: Substrate

Type	score	sub. m/z observed	sub. m/z calculated	sub ppm			met. m/z observed	met. m/z calculated	met. ppm
MATCH	6.9	364.1871	364.1867	-1.08	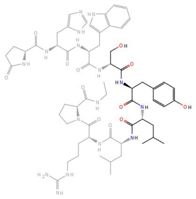	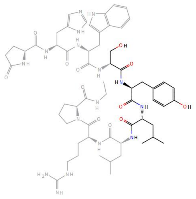	364.1871	364.1867	-1.08
MATCH	78.4	299.2192	299.2190	-0.77	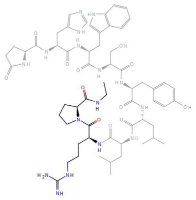	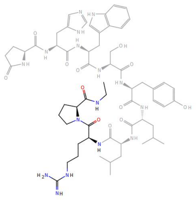	299.2192	299.2190	-0.77
MATCH	18.3	282.1927	282.1925	-0.80	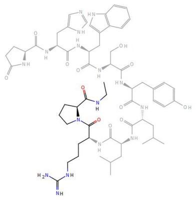	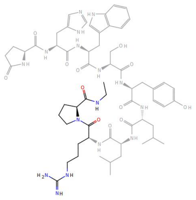	282.1927	282.1925	-0.80
MATCH	10.4	270.1936	270.1925	-4.08	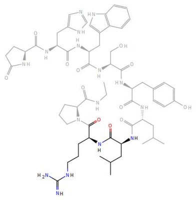	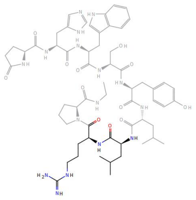	270.1936	270.1925	-4.08
MATCH	22.4	261.1135	261.1164	11.15	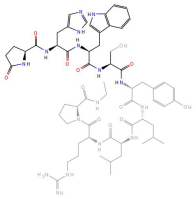	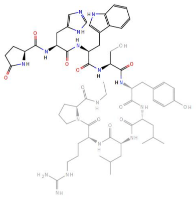	261.1135	261.1164	11.15
MATCH	34.1	253.1658	253.1659	0.39	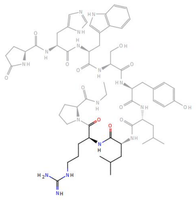	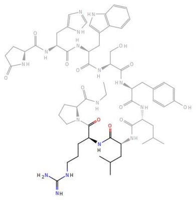	253.1658	253.1659	0.39
MATCH	10.0	249.1565	249.1598	12.95	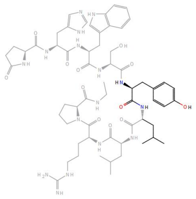	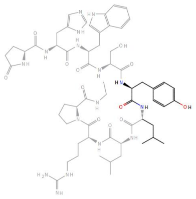	249.1565	249.1598	12.95
MATCH	175.7	249.0983	249.0982	-0.16	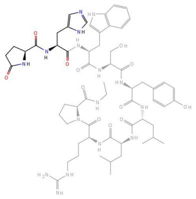	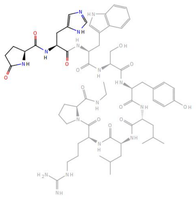	249.0983	249.0982	-0.16
MATCH	4.3	237.1345	237.1346	0.57	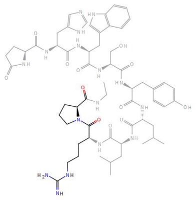	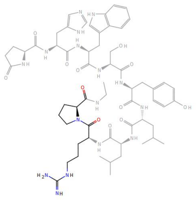	237.1345	237.1346	0.57

Metabolite: Substrate

Type	score	sub. m/z observed	sub. m/z calculated	sub ppm			met. m/z observed	met. m/z calculated	met. ppm
MATCH	3.7	227.1755	227.1754	-0.47	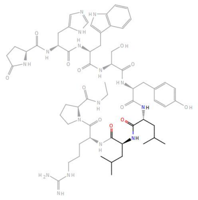	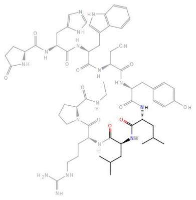	227.1755	227.1754	-0.47
MATCH	3.7	227.1755	227.1754	-0.47	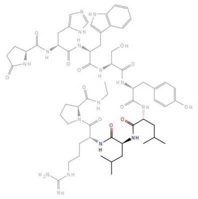	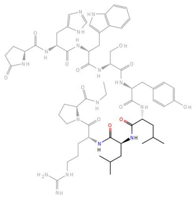	227.1755	227.1754	-0.47
MATCH	176.7	221.1034	221.1033	-0.36	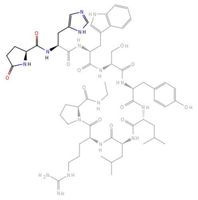	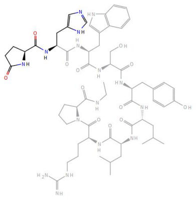	221.1034	221.1033	-0.36
MATCH	15.7	199.1811	199.1805	-3.31	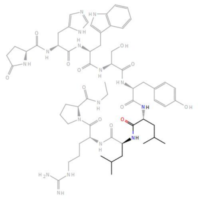	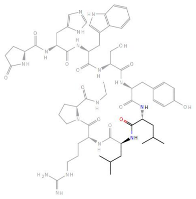	199.1811	199.1805	-3.31
MATCH	15.0	166.0612	166.0611	-0.60	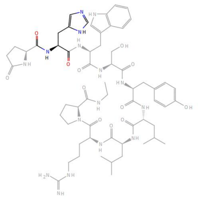	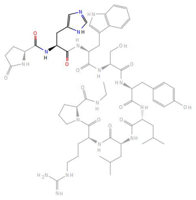	166.0612	166.0611	-0.60
MATCH	58.8	159.0918	159.0917	-0.67	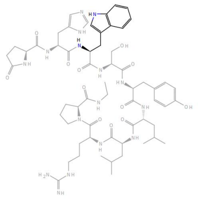	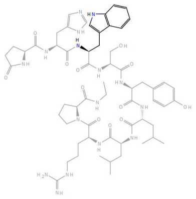	159.0918	159.0917	-0.67
MATCH	11.5	157.1084	157.1084	-0.28	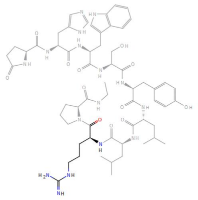	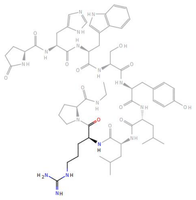	157.1084	157.1084	-0.28
MATCH	76.6	143.1181	143.1179	-1.28	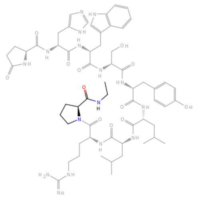	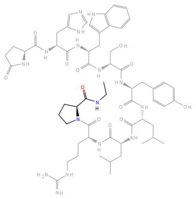	143.1181	143.1179	-1.28
MATCH	103.8	136.0759	136.0757	-1.26	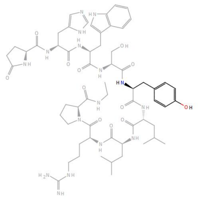	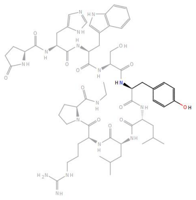	136.0759	136.0757	-1.26

Metabolite: Substrate

Type	score	sub. m/z observed	sub. m/z calculated	sub ppm			met. m/z observed	met. m/z calculated	met. ppm
MATCH	13.0	115.0870	115.0866	-3.58	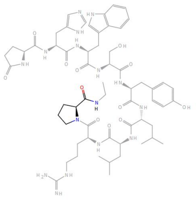	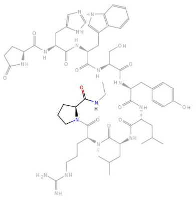	115.0870	115.0866	-3.58
MATCH	31.5	112.0874	112.0869	-4.08	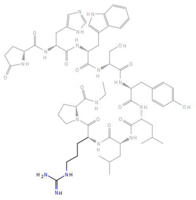	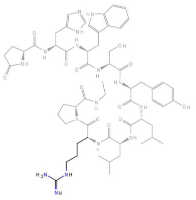	112.0874	112.0869	-4.08
MATCH	173.9	110.0717	110.0713	-4.11	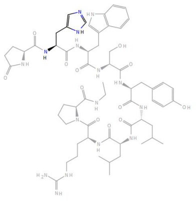	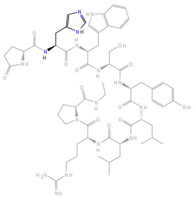	110.0717	110.0713	-4.11
MATCH	5.1	95.0610	95.0604	-7.05	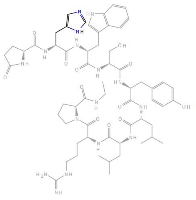	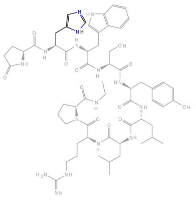	95.0610	95.0604	-7.05
MATCH	7.3	91.0551	91.0522	-31.1	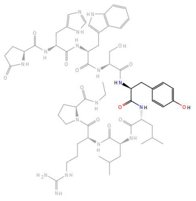	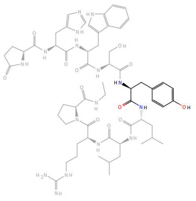	91.0551	91.0522	-31.1
MATCH	100.3	86.0971	86.0964	-8.39	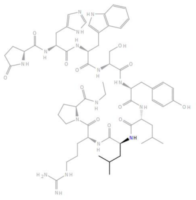	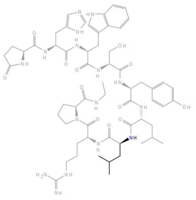	86.0971	86.0964	-8.39
MATCH	100.3	86.0971	86.0964	-8.39	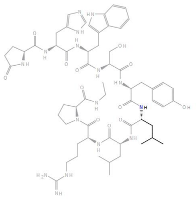	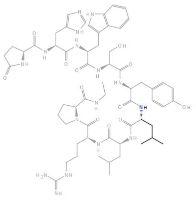	86.0971	86.0964	-8.39
MATCH	12.2	84.0452	84.0444	-9.13	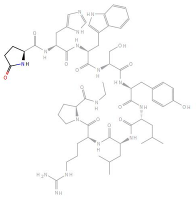	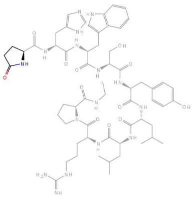	84.0452	84.0444	-9.13

MS (+) FT

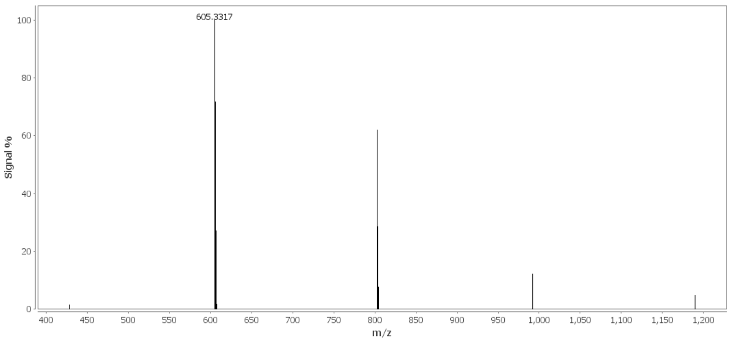

MS (+) FT

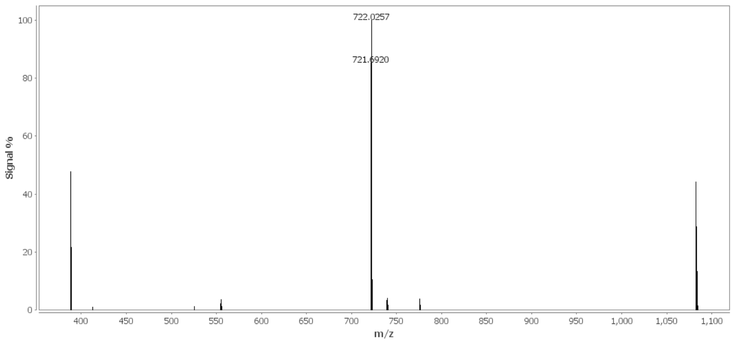

MS2 (+) FT activ = HCD:ce =

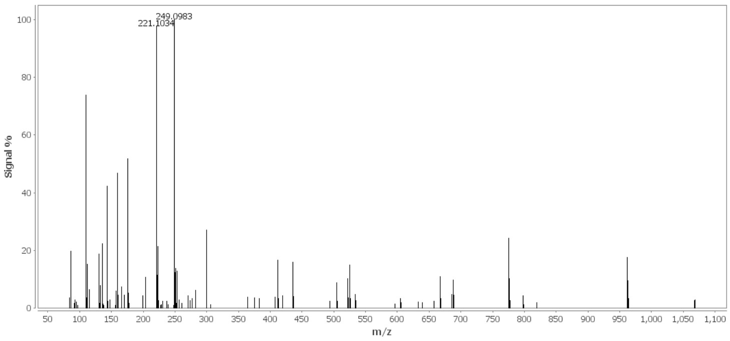

MS2 (+) FT activ = HCD:ce =

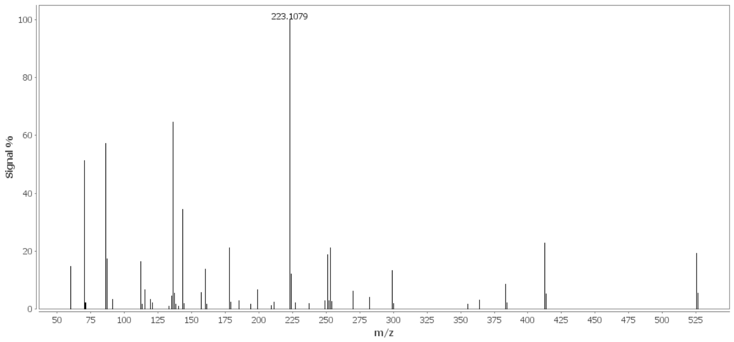

Metabolite: M3 -434 RT=1.96

Type	score	sub. m/z observed	sub. m/z calculated	sub ppm			met. m/z observed	met. m/z calculated	met. ppm
MATCH	147.7	605.3317	605.3300	-2.87	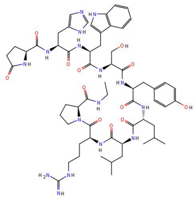	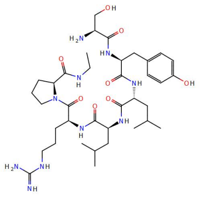	388.2455	388.2449	-1.60
MATCH	103.7	605.3317	605.3300	-2.87	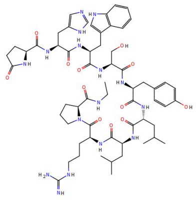	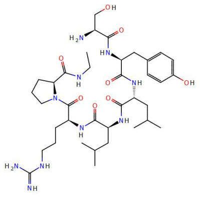	775.4841	775.4825	-2.02
MATCH	76.7	86.0971	86.0964	-8.39	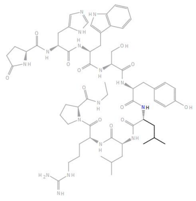	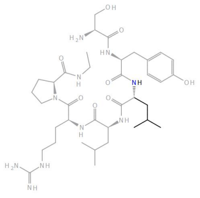	86.0972	86.0964	-8.90
MATCH	76.7	86.0971	86.0964	-8.39	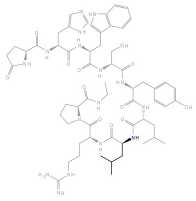	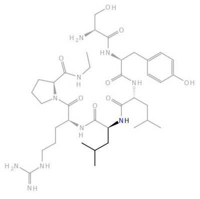	86.0972	86.0964	-8.90
MATCH	5.0	91.0551	91.0522	-31.1	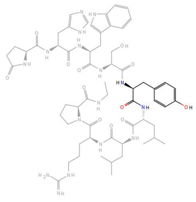	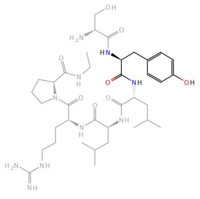	91.0550	91.0522	-30.5

Metabolite: M3 -434 RT=1.96

Type	score	sub. m/z observed	sub. m/z calculated	sub ppm		met. m/z observed	met. m/z calculated	met. ppm
MATCH	31.5	112.0874	112.0869	-4.08	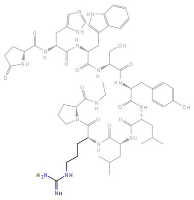 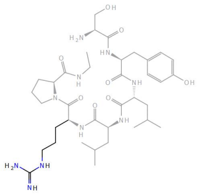	112.0874	112.0869	-4.49
MATCH	13.0	115.0870	115.0866	-3.58	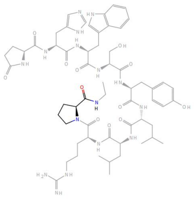 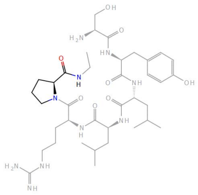	115.0871	115.0866	-4.14
MATCH	86.7	136.0759	136.0757	-1.26	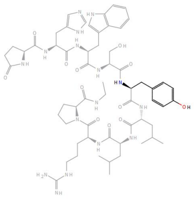 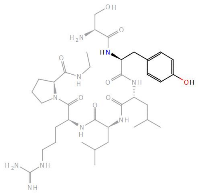	136.0760	136.0757	-1.95
MATCH	76.6	143.1181	143.1179	-1.28	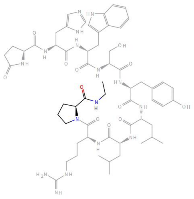 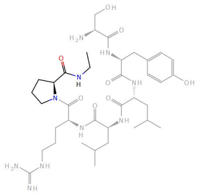	143.1181	143.1179	-1.69
MATCH	11.5	157.1084	157.1084	-0.28	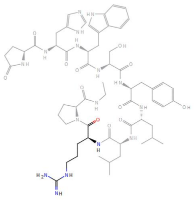 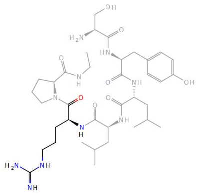	157.1085	157.1084	-0.92
MATCH	11.1	199.1811	199.1805	-3.31	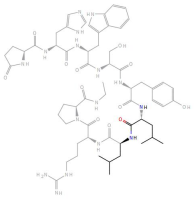 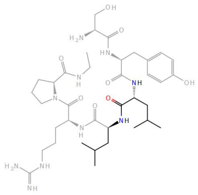	199.1808	199.1805	-1.72
MATCH	3.2	227.1755	227.1754	-0.47	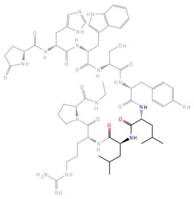 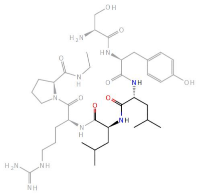	227.1756	227.1754	-0.73
MATCH	3.2	227.1755	227.1754	-0.47	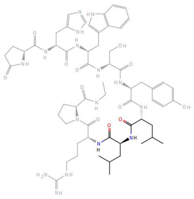 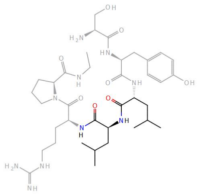	227.1756	227.1754	-0.73
MATCH	4.3	237.1345	237.1346	0.57	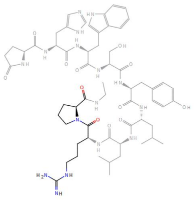 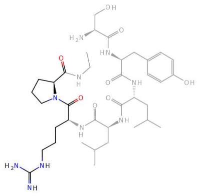	237.1352	237.1346	-2.63

Metabolite: M3 -434 RT=1.96

Type	score	sub. m/z observed	sub. m/z calculated	sub ppm			met. m/z observed	met. m/z calculated	met. ppm
MATCH	8.2	249.1565	249.1598	12.95	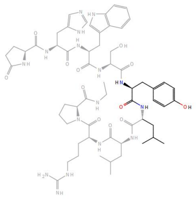	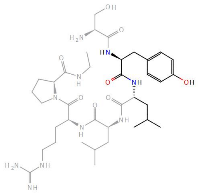	249.1608	249.1598	-4.31
MATCH	34.1	253.1658	253.1659	0.39	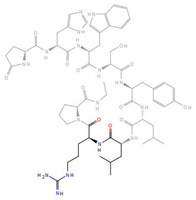	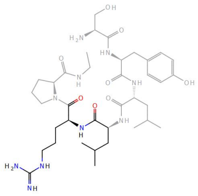	253.1660	253.1659	-0.43
MATCH	19.1	261.1135	261.1164	11.15	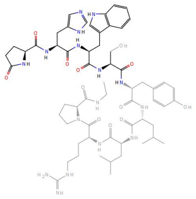	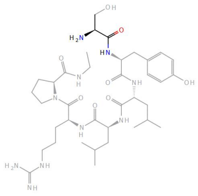	87.0561	87.0553	-8.82
MATCH	10.4	270.1936	270.1925	-4.08	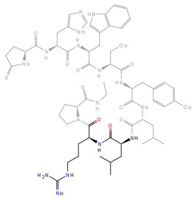	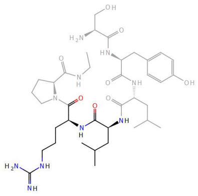	270.1926	270.1925	-0.62
MATCH	10.4	282.1927	282.1925	-0.80	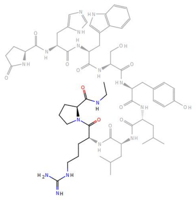	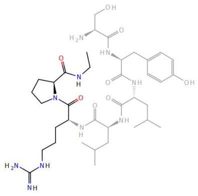	282.1916	282.1925	2.99
MATCH	40.5	299.2192	299.2190	-0.77	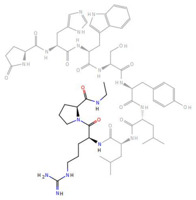	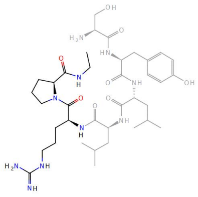	299.2192	299.2190	-0.73
MATCH	6.9	364.1871	364.1867	-1.08	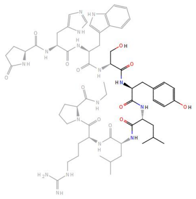	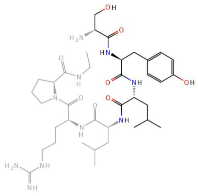	364.1865	364.1867	0.54
						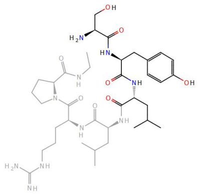	364.1865	364.1867	0.54
MATCH	11.8	383.2752	383.2765	3.51	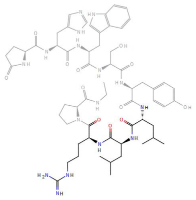	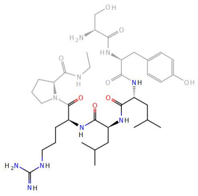	383.2767	383.2765	-0.38

Metabolite: M3 -434 RT=1.96

Type	score	sub. m/z observed	sub. m/z calculated	sub ppm			met. m/z observed	met. m/z calculated	met. ppm
MATCH	39.3	412.3038	412.3031	-1.71	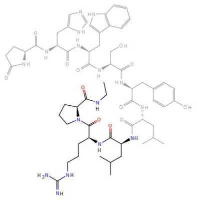	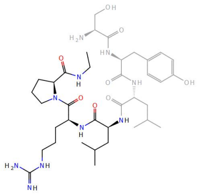	412.3033	412.3031	-0.65
MATCH	17.2	494.2144	494.2146	0.49	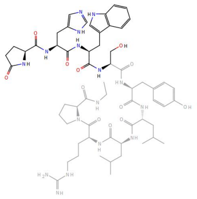	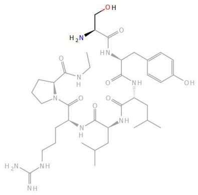	60.0453	60.0444	-15.9
MATCH	10.7	504.1999	504.1990	-1.84	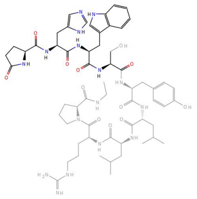	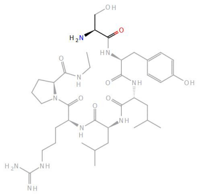	70.0297	70.0287	-13.1
MATCH	34.2	525.3865	525.3871	1.18	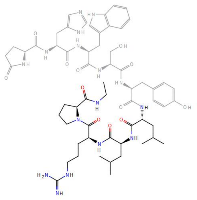	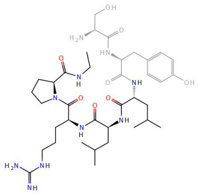	525.3877	525.3871	-1.05
MATCH	102.5	657.2747	657.2780	4.97	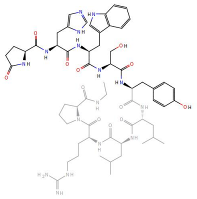	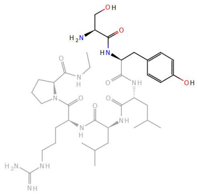	223.1079	223.1077	-0.75
MATCH	23.4	685.2729	685.2729	-0.02	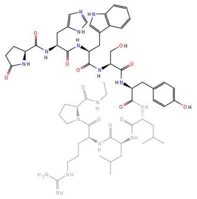	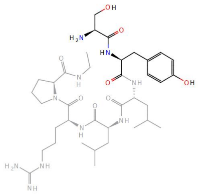	251.1028	251.1026	-0.67
MATCH	7.3	798.3551	798.3570	2.37	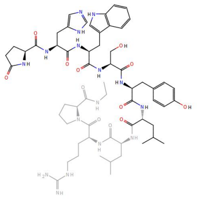	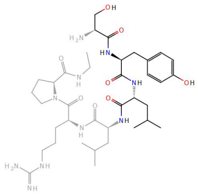	364.1865	364.1867	0.54
						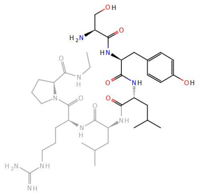	364.1865	364.1867	0.54
MET_MATCH						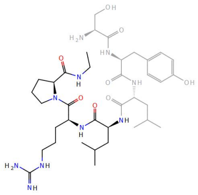	412.3040	412.3031	-2.33

Metabolite: M3 -434 RT=1.96

Type	score	sub. m/z observed	sub. m/z calculated	sub ppm		met. m/z observed	met. m/z calculated	met. ppm
MET_MATCH					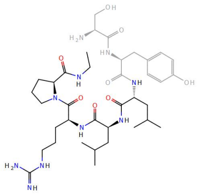	525.3878	525.3871	-1.31

MS (+) FT

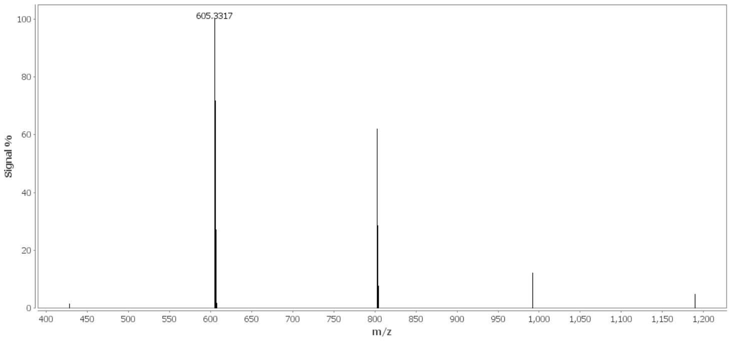

MS (+) FT

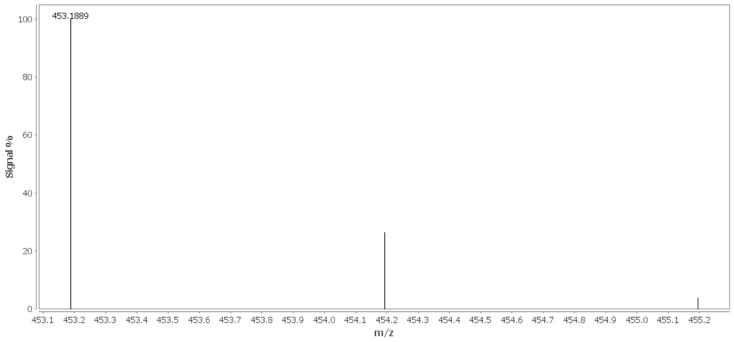

MS2 (+) FT activ = HCD:ce =

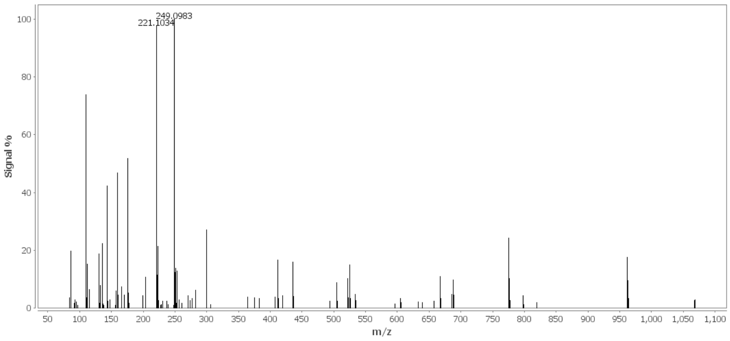

MS2 (+) FT activ = HCD:ce =

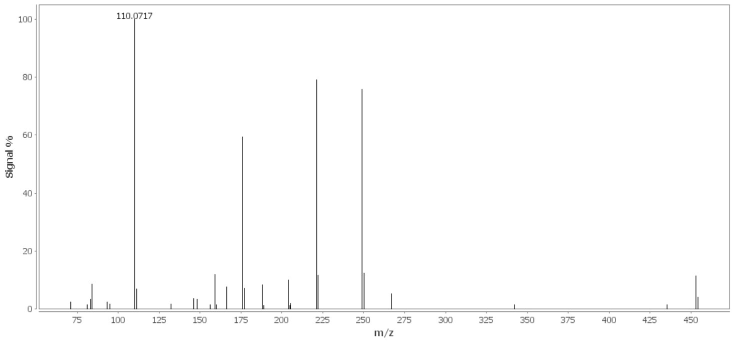

Metabolite: M1 -756 RT=0.42

Type	score	sub. m/z observed	sub. m/z calculated	sub ppm			met. m/z observed	met. m/z calculated	met. ppm
MATCH	200.0	605.3317	605.3300	-2.87	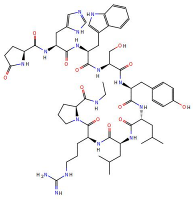	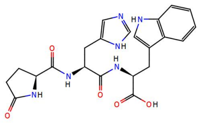	453.1889	453.1881	-1.68
MATCH	12.2	84.0452	84.0444	-9.13	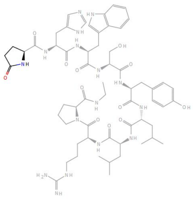	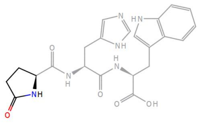	84.0451	84.0444	-8.48
MATCH	5.1	93.0455	93.0447	-8.02	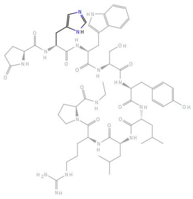	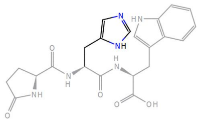	93.0451	93.0447	-4.17

Metabolite: M1 -756 RT=0.42

Type	score	sub. m/z observed	sub. m/z calculated	sub ppm		met. m/z observed	met. m/z calculated	met. ppm
MATCH	3.7	95.0610	95.0604	-7.05	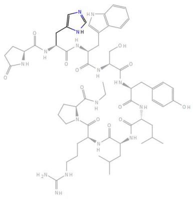 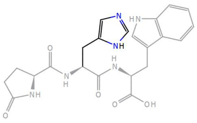	95.0608	95.0604	-4.82
MATCH	173.9	110.0717	110.0713	-4.11	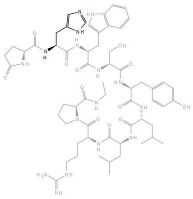 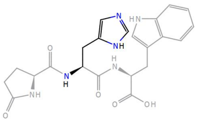	110.0717	110.0713	-3.56
MATCH	58.8	159.0918	159.0917	-0.67	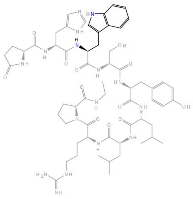 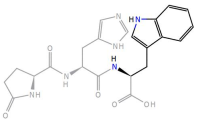	159.0917	159.0917	0.03
MATCH	15.0	166.0612	166.0611	-0.60	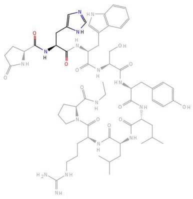 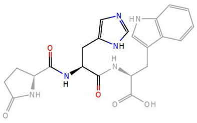	166.0611	166.0611	0.15
MATCH	176.7	221.1034	221.1033	-0.36	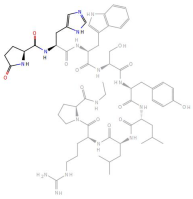 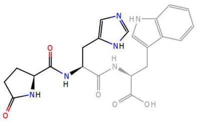	221.1032	221.1033	0.39
MATCH	175.7	249.0983	249.0982	-0.16	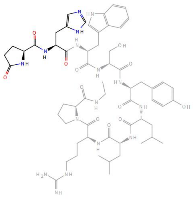 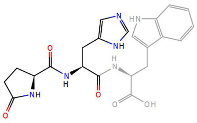	249.0981	249.0982	0.36
MATCH	14.7	605.3271	605.3300	4.78	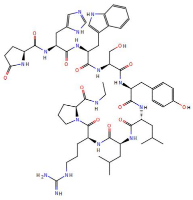 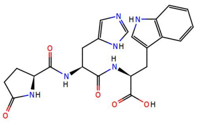	453.1875	453.1881	1.36
MATCH	19.5	961.5611	961.5618	0.69	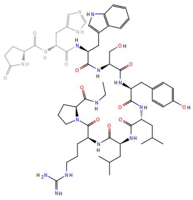 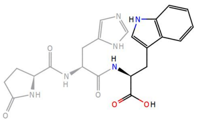	205.0974	205.0972	-1.17
MET_MATCH					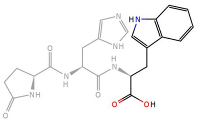	188.0704	188.0706	0.91

Metabolite: M1 -756 RT=0.42

Type	score	sub. m/z observed	sub. m/z calculated	sub ppm		met. m/z observed	met. m/z calculated	met. ppm
MET_MATCH					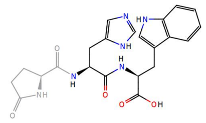	342.1568	342.1561	-2.17
MET_MATCH					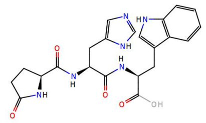	435.1789	435.1775	-3.21

MS (+) FT

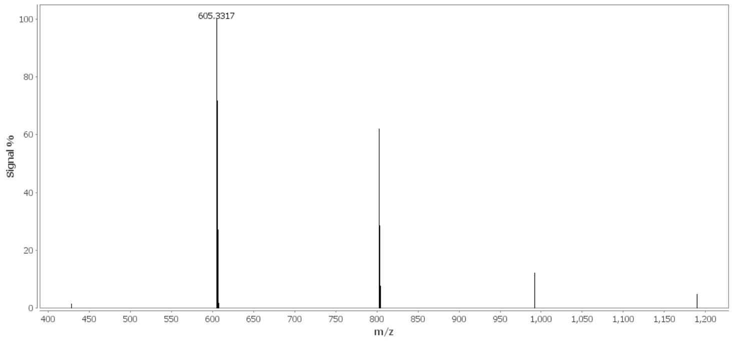

MS (+) FT

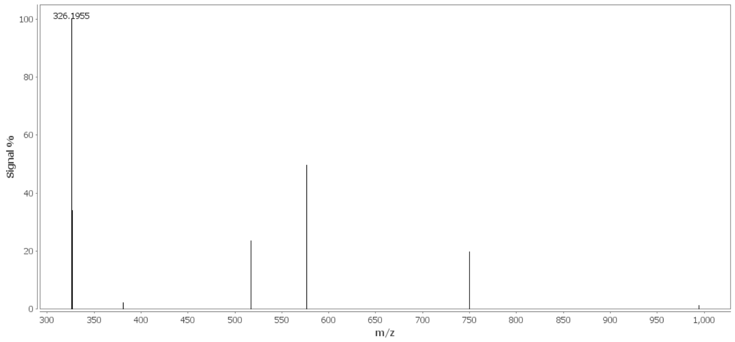

MS2 (+) FT activ = HCD:ce =

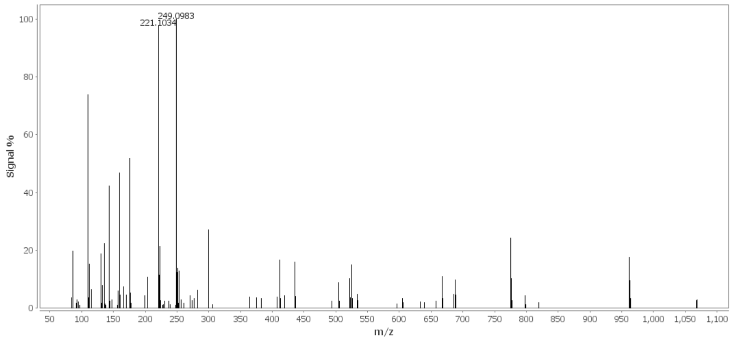

MS2 (+) FT activ = HCD:ce =

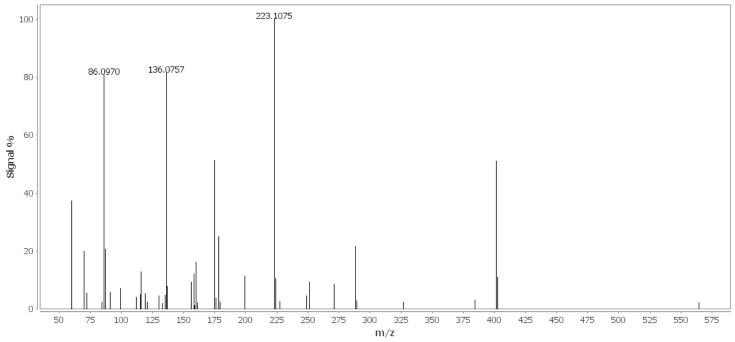

Metabolite: M2 -558 RT=1.83

Type	score	sub. m/z observed	sub. m/z calculated	sub ppm			met. m/z observed	met. m/z calculated	met. ppm
MATCH	200.0	605.3317	605.3300	-2.87	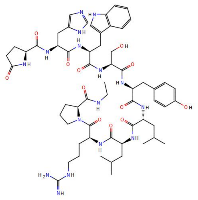	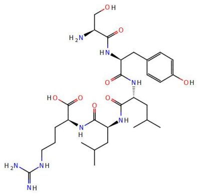	326.1955	326.1949	-2.01
MATCH	100.3	86.0971	86.0964	-8.39	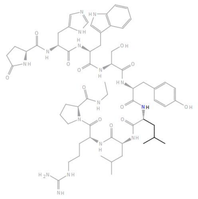	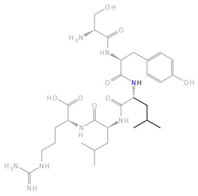	86.0970	86.0964	-6.84

Metabolite: M2 -558 RT=1.83

Type	score	sub. m/z observed	sub. m/z calculated	sub ppm			met. m/z observed	met. m/z calculated	met. ppm
MATCH	100.3	86.0971	86.0964	-8.39	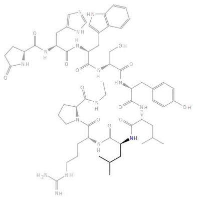	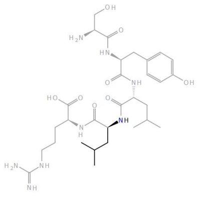	86.0970	86.0964	-6.84
MATCH	7.3	91.0551	91.0522	-31.1	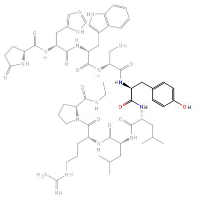	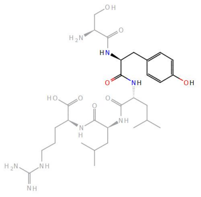	91.0547	91.0522	-27.7
MATCH	19.3	112.0874	112.0869	-4.08	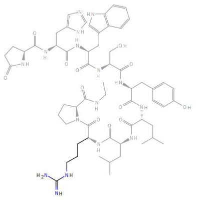	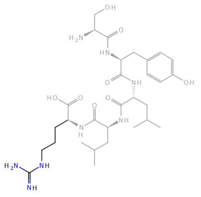	112.0872	112.0869	-2.91
MATCH	103.8	136.0759	136.0757	-1.26	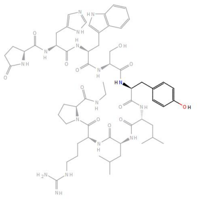	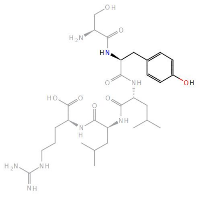	136.0757	136.0757	0.16
MATCH	15.7	199.1811	199.1805	-3.31	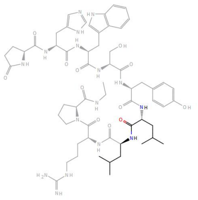	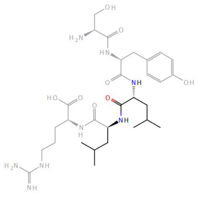	199.1806	199.1805	-0.42
MATCH	3.7	227.1755	227.1754	-0.47	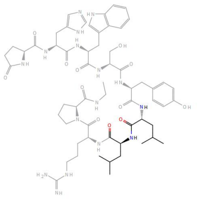	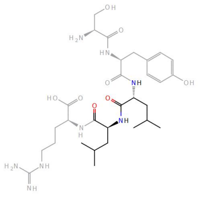	227.1763	227.1754	-4.08
MATCH	3.7	227.1755	227.1754	-0.47	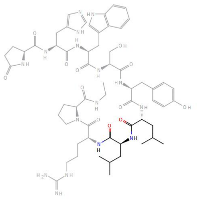	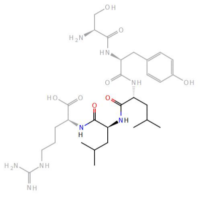	227.1763	227.1754	-4.08
MATCH	10.0	249.1565	249.1598	12.95	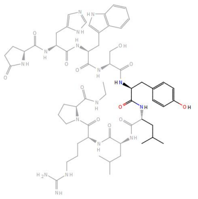	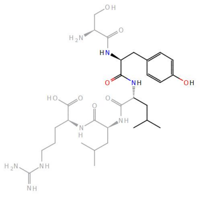	249.1604	249.1598	-2.66
MATCH	22.4	261.1135	261.1164	11.15	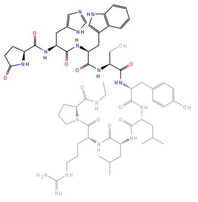	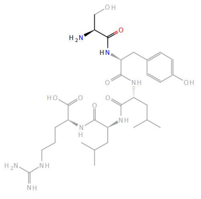	87.0559	87.0553	-6.58

Metabolite: M2 -558 RT=1.83

Type	score	sub. m/z observed	sub. m/z calculated	sub ppm		met. m/z observed	met. m/z calculated	met. ppm
MATCH	18.3	282.1927	282.1925	-0.80	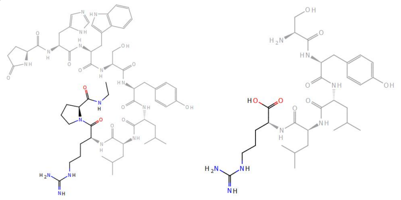	158.0924	158.0924	-0.22
MATCH	78.4	299.2192	299.2190	-0.77	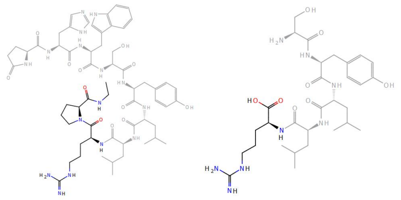	175.1188	175.1190	0.67
MATCH	38.1	412.3038	412.3031	-1.71	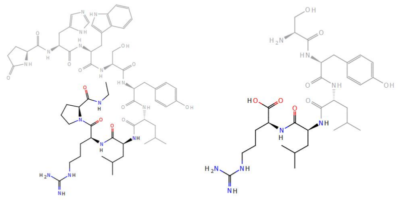	288.2027	288.2030	1.00
MATCH	39.8	494.2144	494.2146	0.49	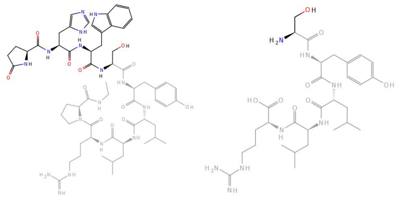	60.0453	60.0444	-14.3
MATCH	12.0	504.1999	504.1990	-1.84	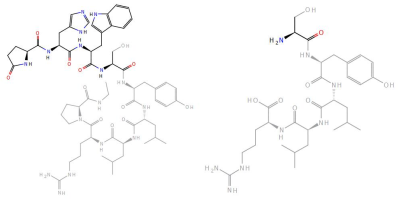	70.0295	70.0287	-10.4
MATCH	66.0	525.3865	525.3871	1.18	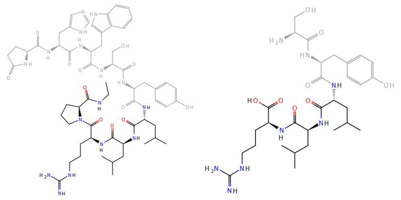	401.2867	401.2871	1.07
MATCH	102.5	657.2747	657.2780	4.97	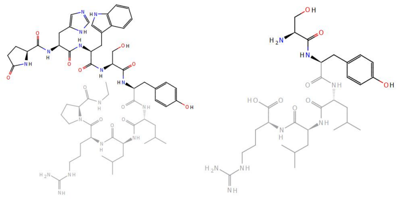	223.1075	223.1077	1.13
MATCH	14.1	685.2729	685.2729	-0.02	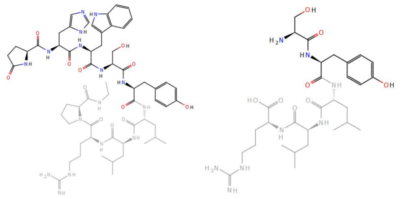	251.1022	251.1026	1.77
MATCH	11.8	688.4504	688.4505	0.04	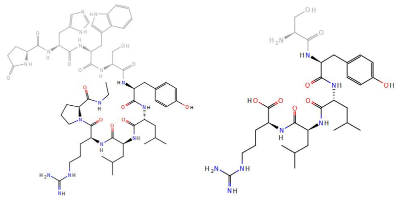	564.3467	564.3504	6.61

Metabolite: M2 -558 RT=1.83

Type	score	sub. m/z observed	sub. m/z calculated	sub ppm		met. m/z observed	met. m/z calculated	met. ppm
MET_MATCH					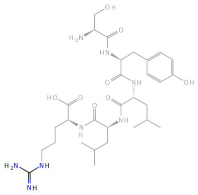	60.0565	60.0556	-14.3
MET_MATCH					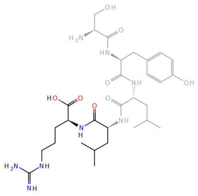	271.1758	271.1765	2.64
MET_MATCH					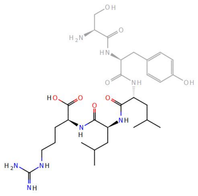	384.2602	384.2605	0.80
