# Supplementary material for: Software-aided approach to investigate peptide structure and metabolic susceptibility of amide bonds in peptide drugs based on high resolution mass spectrometry
Source: PLoS One. 2017 Nov 1;12(11):e0186461. doi: 10.1371/journal.pone.0186461 (PMC5665424; doi:10.1371/journal.pone.0186461)
Supplement: S1 File — (ZIP) [file pone.0186461.s007.zip › SFiles/S23_File.pdf]

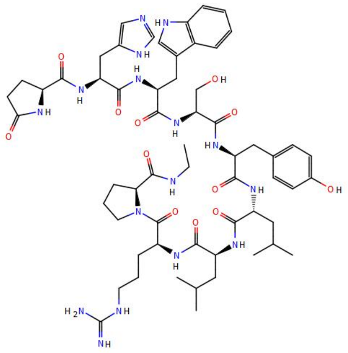

Leuprolide

Property name	Property value
Time	0min, 5min, 15min, 45min, 120min
Instrument	ThermoQAPlus
Matrix	elastase
Acquisition Mode	ddMS2

Chromatograms

Time=0min

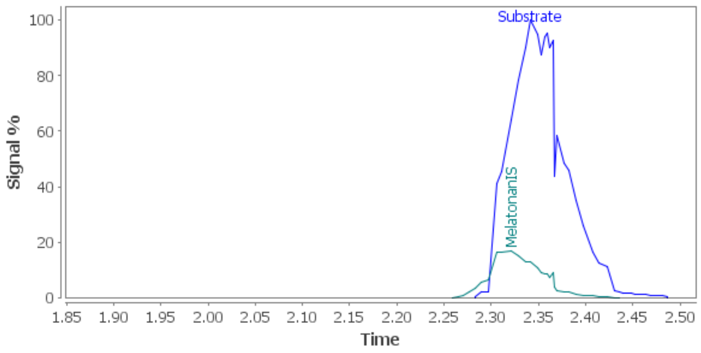

Time=5min

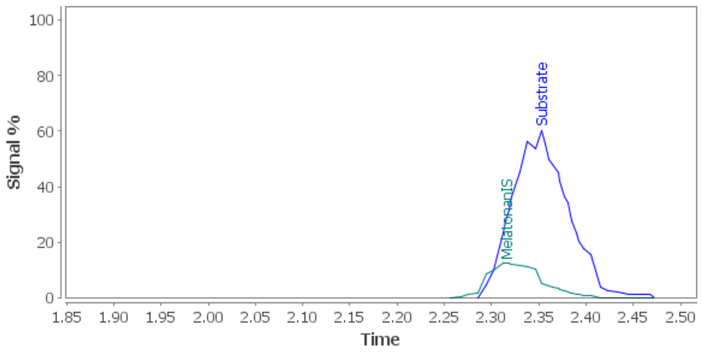

Time=15min

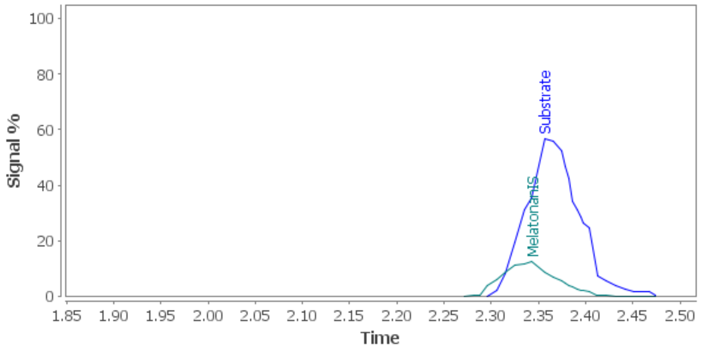

Time=45min

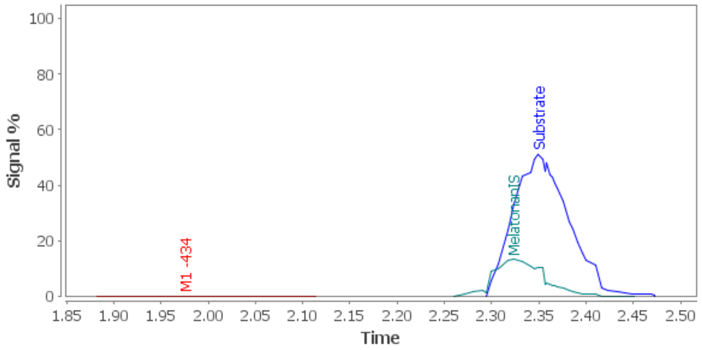

Time=120min

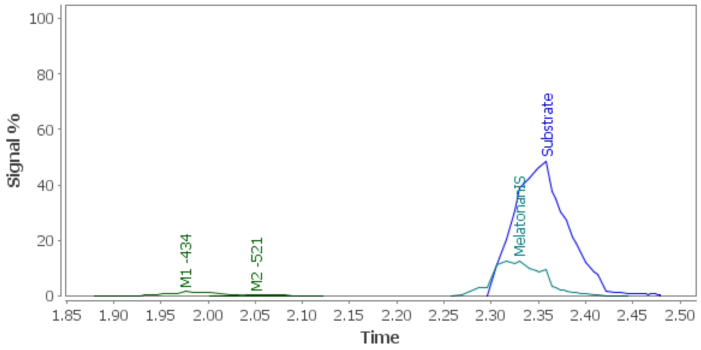

Custom Charts

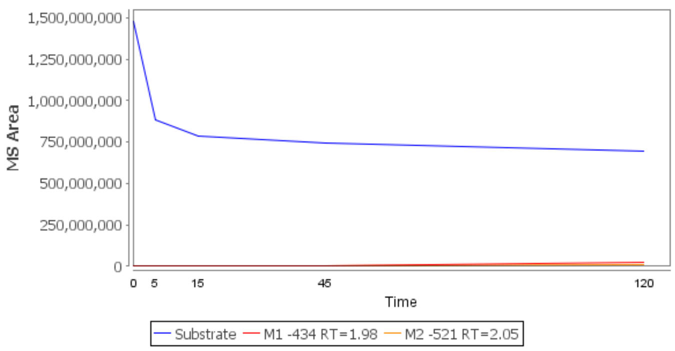

Fragmentation

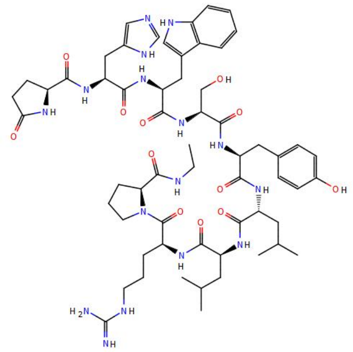

Leuprolide

MS (+) FT

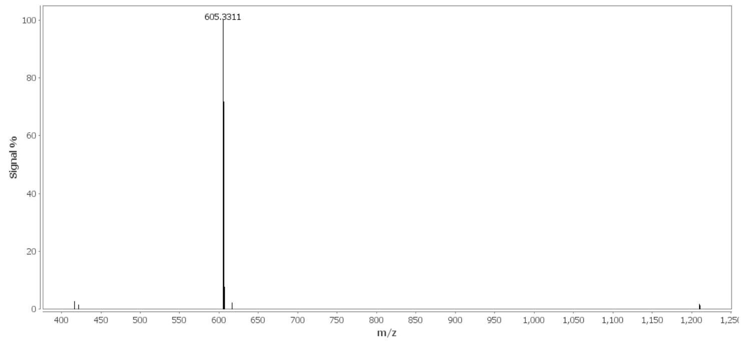

MS (+) FT

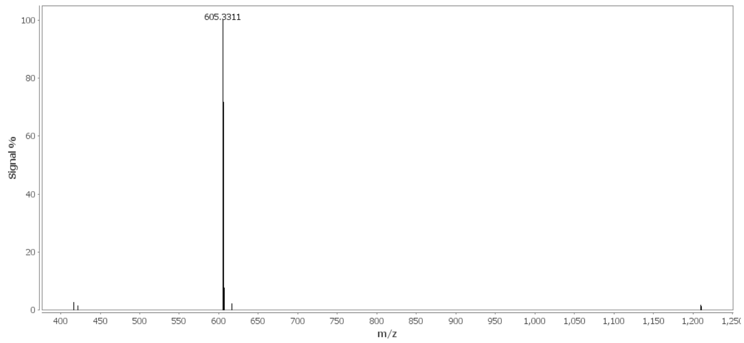

MS2 (+) FT activ = HCD:ce =

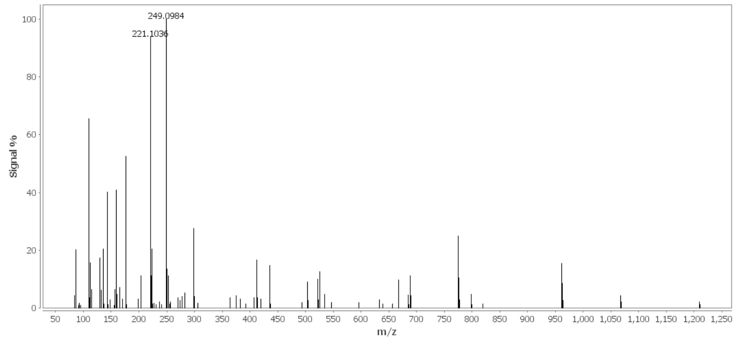

MS2 (+) FT activ = HCD:ce =

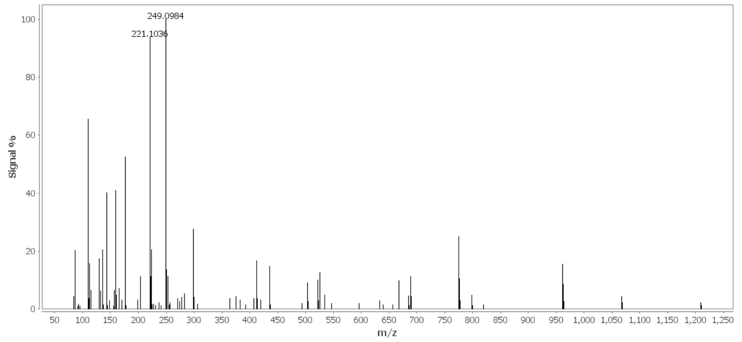

Metabolite: Substrate

Type	score	sub. m/z observed	sub. m/z calculated	sub ppm			met. m/z observed	met. m/z calculated	met. ppm
MATCH	101.8	1209.6547	1209.6527	-1.59	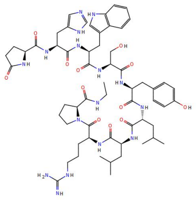	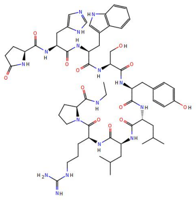	1209.6547	1209.6527	-1.59
MATCH	7.2	798.3562	798.3570	0.95	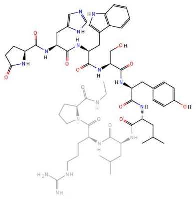	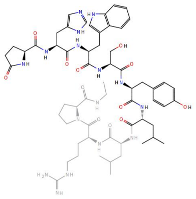	798.3562	798.3570	0.95
MATCH	23.6	685.2738	685.2729	-1.29	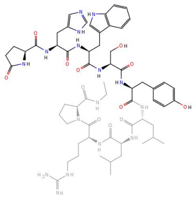	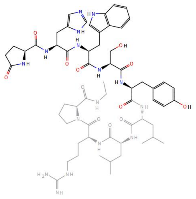	685.2738	685.2729	-1.29

Metabolite: Substrate

Type	score	sub. m/z observed	sub. m/z calculated	sub ppm			met. m/z observed	met. m/z calculated	met. ppm
MATCH	101.4	657.2772	657.2780	1.10	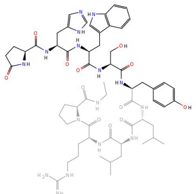	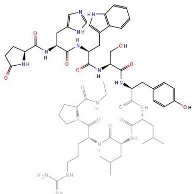	657.2772	657.2780	1.10
MATCH	200.0	605.3311	605.3300	-1.79	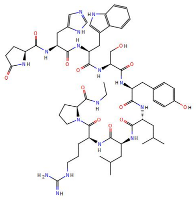	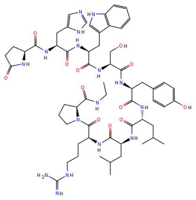	605.3311	605.3300	-1.79
MATCH	30.5	525.3876	525.3871	-0.80	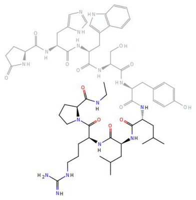	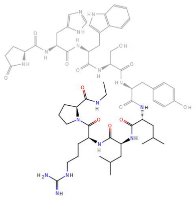	525.3876	525.3871	-0.80
MATCH	10.8	504.1992	504.1990	-0.47	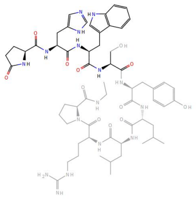	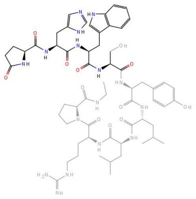	504.1992	504.1990	-0.47
MATCH	17.6	494.2143	494.2146	0.76	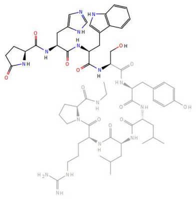	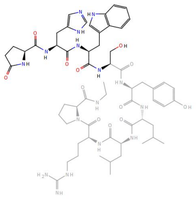	494.2143	494.2146	0.76
MATCH	40.0	412.3040	412.3031	-2.34	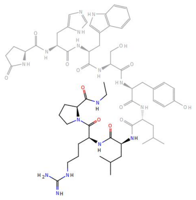	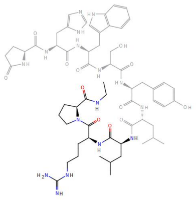	412.3040	412.3031	-2.34
MATCH	12.0	383.2770	383.2765	-1.21	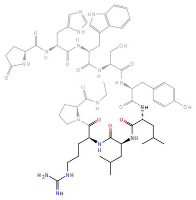	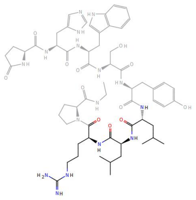	383.2770	383.2765	-1.21
MATCH	5.9	364.1871	364.1867	-1.18	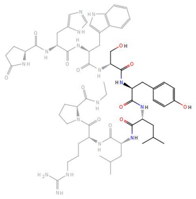	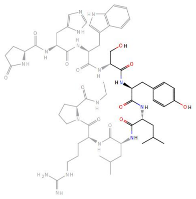	364.1871	364.1867	-1.18
MATCH	40.6	299.2194	299.2190	-1.47	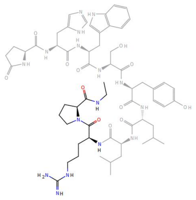	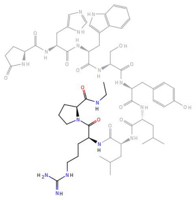	299.2194	299.2190	-1.47

Metabolite: Substrate

Type	score	sub. m/z observed	sub. m/z calculated	sub ppm			met. m/z observed	met. m/z calculated	met. ppm
MATCH	10.3	282.1930	282.1925	-1.77	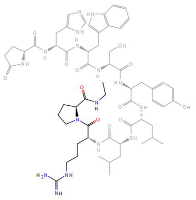	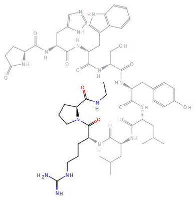	282.1930	282.1925	-1.77
MATCH	10.1	270.1925	270.1925	-0.02	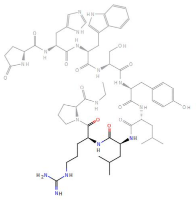	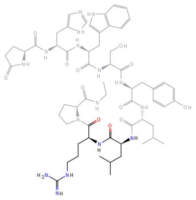	270.1925	270.1925	-0.02
MATCH	32.1	253.1658	253.1659	0.37	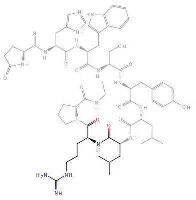	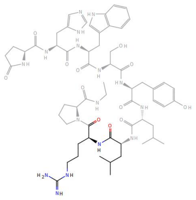	253.1658	253.1659	0.37
MATCH	7.4	249.1563	249.1598	13.83	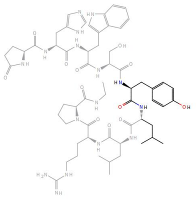	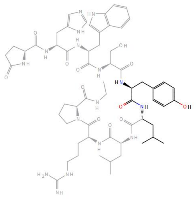	249.1563	249.1598	13.83
MATCH	3.7	237.1351	237.1346	-2.26	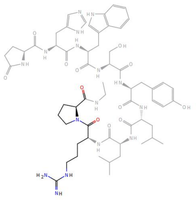	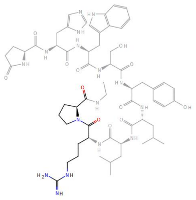	237.1351	237.1346	-2.26
MATCH	3.8	227.1758	227.1754	-1.54	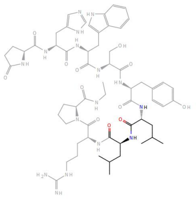	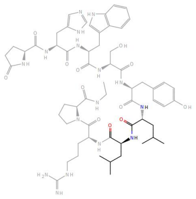	227.1758	227.1754	-1.54
MATCH	3.8	227.1758	227.1754	-1.54	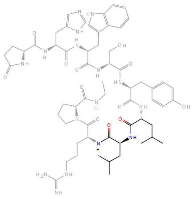	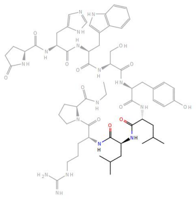	227.1758	227.1754	-1.54
MATCH	10.1	199.1802	199.1805	1.48	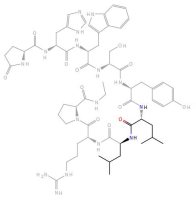	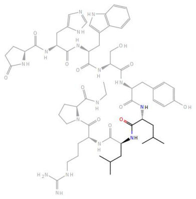	199.1802	199.1805	1.48
MATCH	12.8	157.1086	157.1084	-1.22	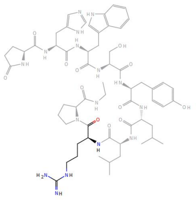	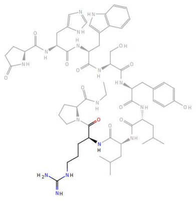	157.1086	157.1084	-1.22

Metabolite: Substrate

Type	score	sub. m/z observed	sub. m/z calculated	sub ppm			met. m/z observed	met. m/z calculated	met. ppm
MATCH	75.1	143.1181	143.1179	-1.70	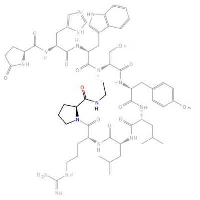	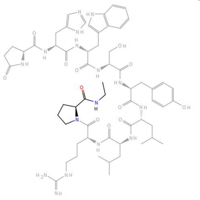	143.1181	143.1179	-1.70
MATCH	84.4	136.0760	136.0757	-2.37	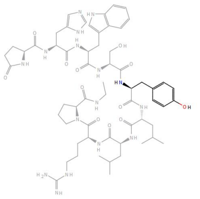	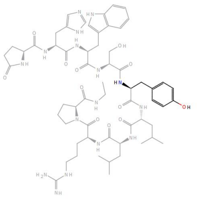	136.0760	136.0757	-2.37
MATCH	13.9	115.0870	115.0866	-3.97	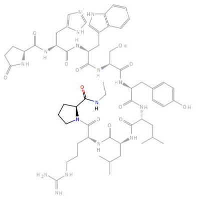	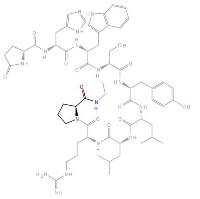	115.0870	115.0866	-3.97
MATCH	30.1	112.0874	112.0869	-4.67	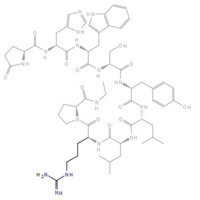	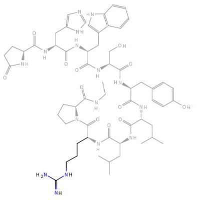	112.0874	112.0869	-4.67
MISMATCH	-90.1	110.0718	110.0713	-4.79	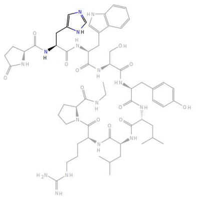	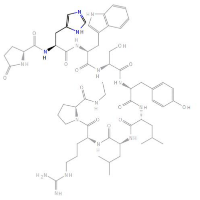	110.0718	110.0713	-4.79
MATCH	3.1	93.0456	93.0553	104.5	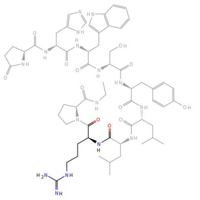	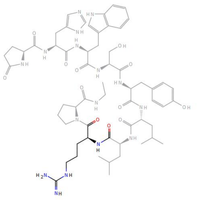	93.0456	93.0553	104.5
MISMATCH	-3.1	93.0456	93.0447	-8.98	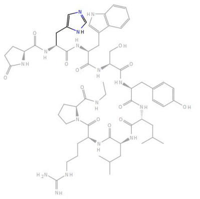	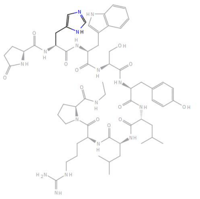	93.0456	93.0447	-8.98
MATCH	5.2	91.0549	91.0522	-29.0	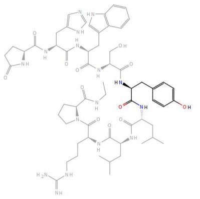	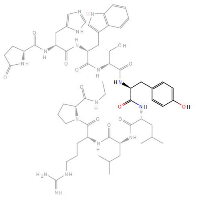	91.0549	91.0522	-29.0
MATCH	120.2	86.0972	86.0964	-9.02	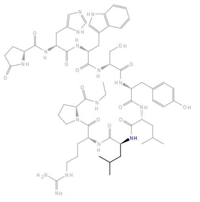	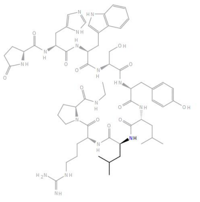	86.0972	86.0964	-9.02

Metabolite: Substrate

Type	score	sub. m/z observed	sub. m/z calculated	sub ppm			met. m/z observed	met. m/z calculated	met. ppm
MATCH	120.2	86.0972	86.0964	-9.02	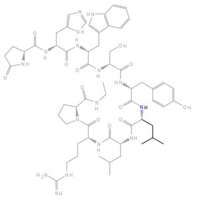	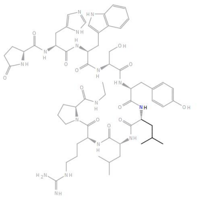	86.0972	86.0964	-9.02

MS (+) FT

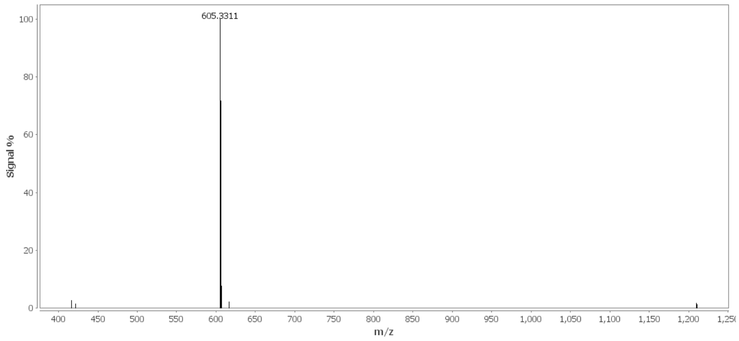

MS (+) FT

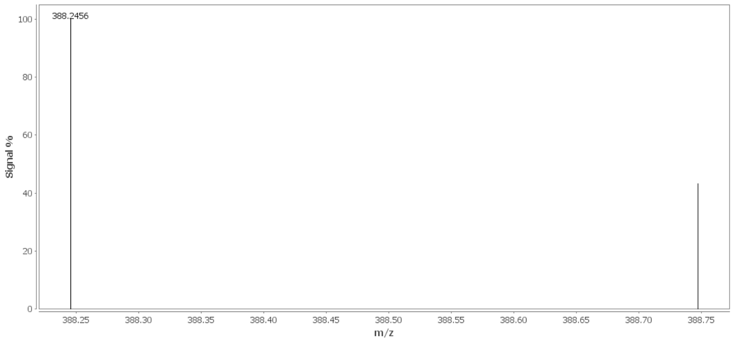

MS2 (+) FT activ = HCD:ce =

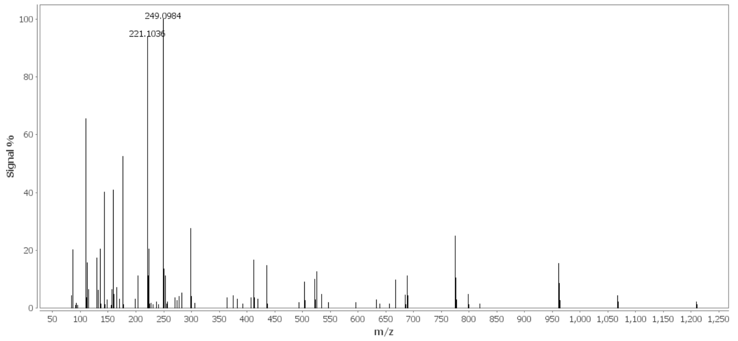

MS2 (+) FT activ = HCD:ce =

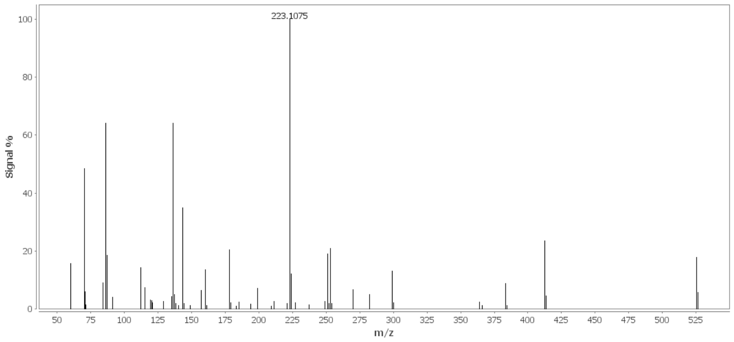

Metabolite: M1 -434 RT=1.98

Type	score	sub. m/z observed	sub. m/z calculated	sub ppm			met. m/z observed	met. m/z calculated	met. ppm
MATCH	200.0	605.3311	605.3300	-1.79	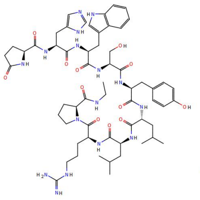	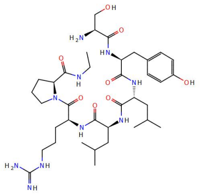	388.2456	388.2449	-1.77
						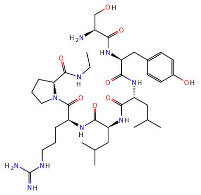	388.2456	388.2449	-1.77
MATCH	101.8	1209.6547	1209.6527	-1.59	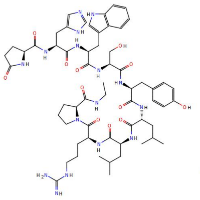	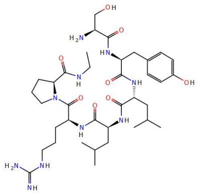	388.2456	388.2449	-1.77

Metabolite: M1 -434 RT=1.98

Type	score	sub. m/z observed	sub. m/z calculated	sub ppm			met. m/z observed	met. m/z calculated	met. ppm
						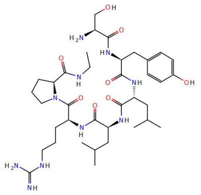	388.2456	388.2449	-1.77
MATCH	84.2	86.0972	86.0964	-9.02	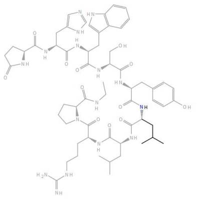	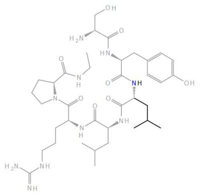	86.0971	86.0964	-7.58
MATCH	84.2	86.0972	86.0964	-9.02	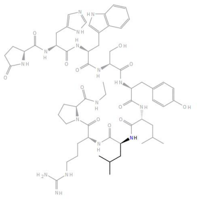	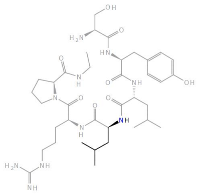	86.0971	86.0964	-7.58
MATCH	5.2	91.0549	91.0522	-29.0	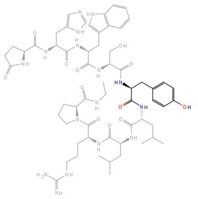	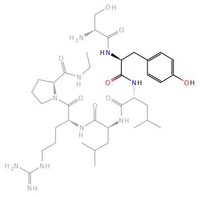	91.0548	91.0522	-28.5
MATCH	30.1	112.0874	112.0869	-4.67	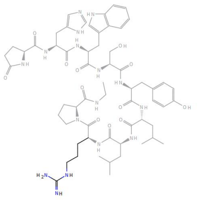	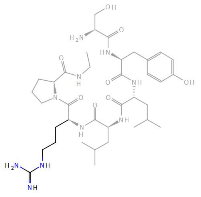	112.0873	112.0869	-3.10
MATCH	13.9	115.0870	115.0866	-3.97	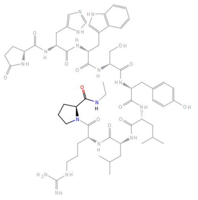	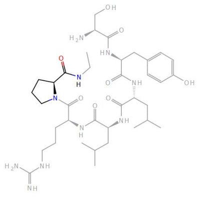	115.0869	115.0866	-2.69
MATCH	84.4	136.0760	136.0757	-2.37	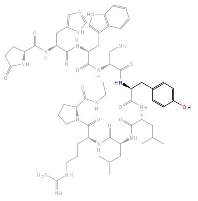	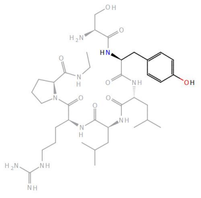	136.0758	136.0757	-0.62
MATCH	75.1	143.1181	143.1179	-1.70	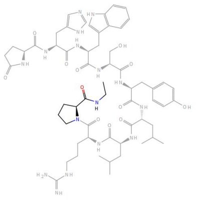	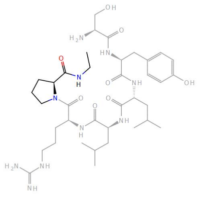	143.1179	143.1179	-0.38
MATCH	12.8	157.1086	157.1084	-1.22	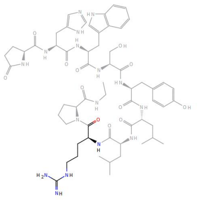	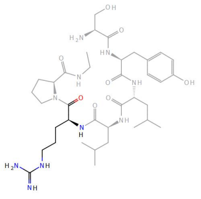	157.1083	157.1084	0.66

Metabolite: M1 -434 RT=1.98

Type	score	sub. m/z observed	sub. m/z calculated	sub ppm			met. m/z observed	met. m/z calculated	met. ppm
MATCH	10.1	199.1802	199.1805	1.48	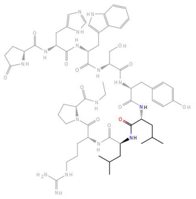	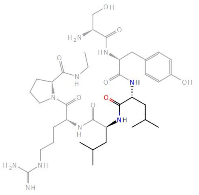	199.1805	199.1805	-0.13
MATCH	3.8	227.1758	227.1754	-1.54	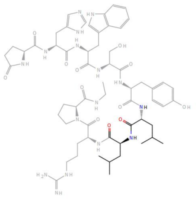	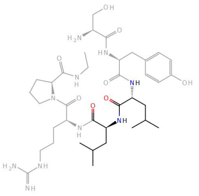	227.1748	227.1754	2.76
MATCH	3.8	227.1758	227.1754	-1.54	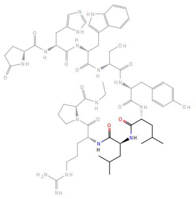	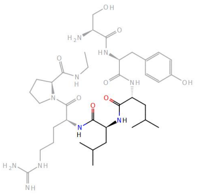	227.1748	227.1754	2.76
MATCH	3.7	237.1351	237.1346	-2.26	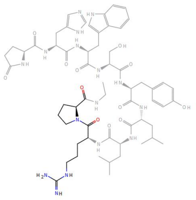	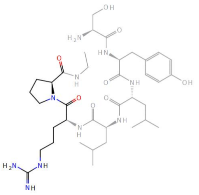	237.1346	237.1346	0.02
MATCH	7.4	249.1563	249.1598	13.83	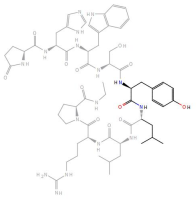	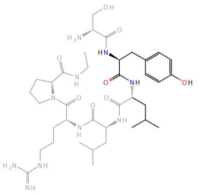	249.1595	249.1598	1.05
MATCH	32.1	253.1658	253.1659	0.37	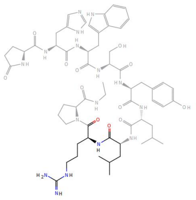	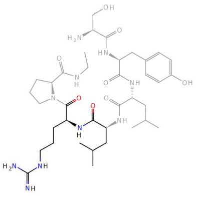	253.1659	253.1659	-0.03
MATCH	10.1	270.1925	270.1925	-0.02	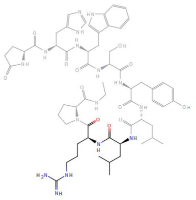	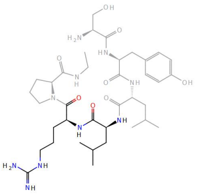	270.1923	270.1925	0.40
MATCH	10.3	282.1930	282.1925	-1.77	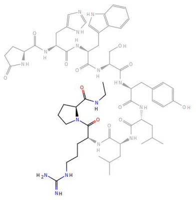	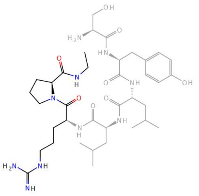	282.1921	282.1925	1.20
MATCH	40.6	299.2194	299.2190	-1.47	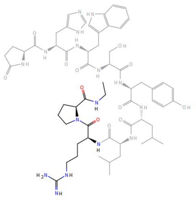	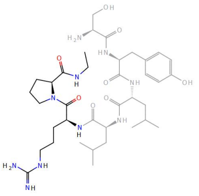	299.2191	299.2190	-0.49

Metabolite: M1 -434 RT=1.98

Type	score	sub. m/z observed	sub. m/z calculated	sub ppm			met. m/z observed	met. m/z calculated	met. ppm
MATCH	5.9	364.1871	364.1867	-1.18	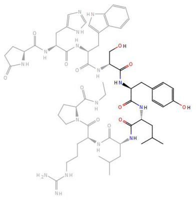	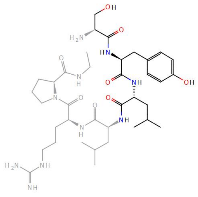	364.1870	364.1867	-0.83
						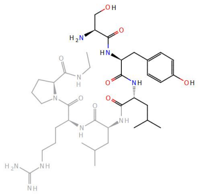	364.1870	364.1867	-0.83
MATCH	12.0	383.2770	383.2765	-1.21	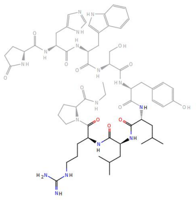	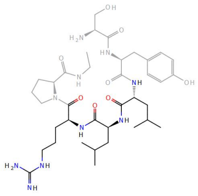	383.2759	383.2765	1.59
MATCH	40.0	412.3040	412.3031	-2.34	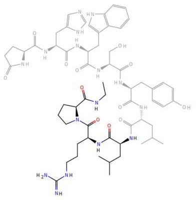	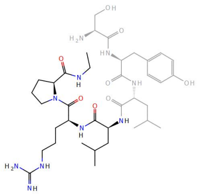	412.3027	412.3031	0.78
MATCH	17.6	494.2143	494.2146	0.76	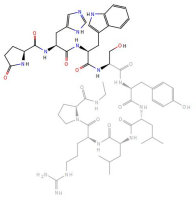	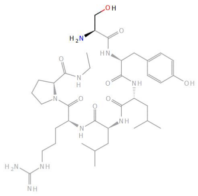	60.0453	60.0444	-14.3
MATCH	10.8	504.1992	504.1990	-0.47	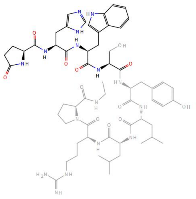	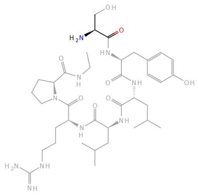	70.0294	70.0287	-10.1
MATCH	30.5	525.3876	525.3871	-0.80	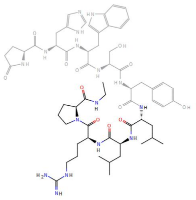	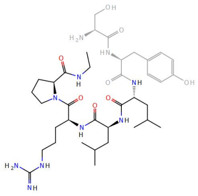	525.3870	525.3871	0.27
MATCH	101.4	657.2772	657.2780	1.10	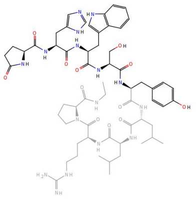	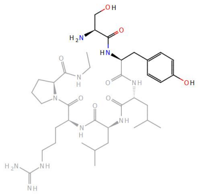	223.1075	223.1077	0.79
MATCH	23.6	685.2738	685.2729	-1.29	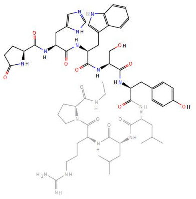	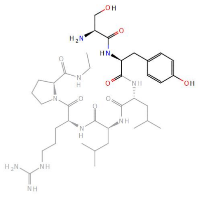	251.1025	251.1026	0.52

Metabolite: M1 -434 RT=1.98

Type	score	sub. m/z observed	sub. m/z calculated	sub ppm			met. m/z observed	met. m/z calculated	met. ppm
MATCH	7.2	798.3562	798.3570	0.95	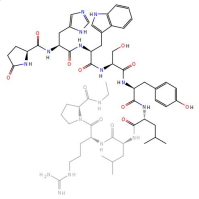	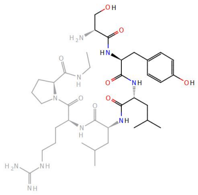	364.1870	364.1867	-0.83
						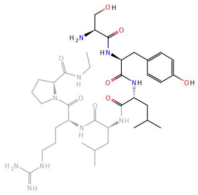	364.1870	364.1867	-0.83

MS (+) FT

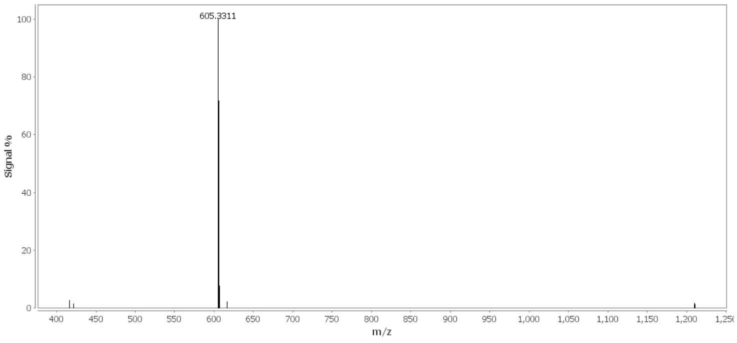

MS (+) FT

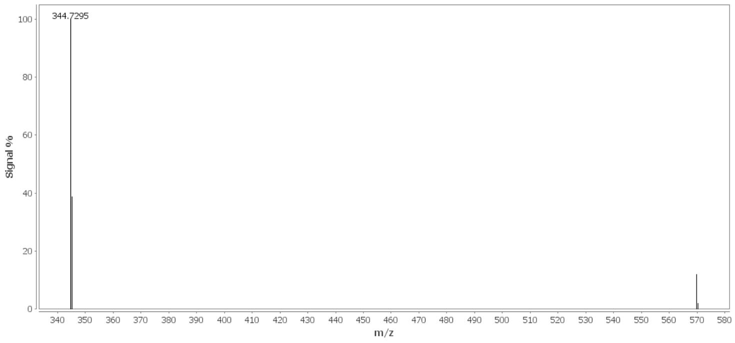

MS2 (+) FT activ = HCD:ce =

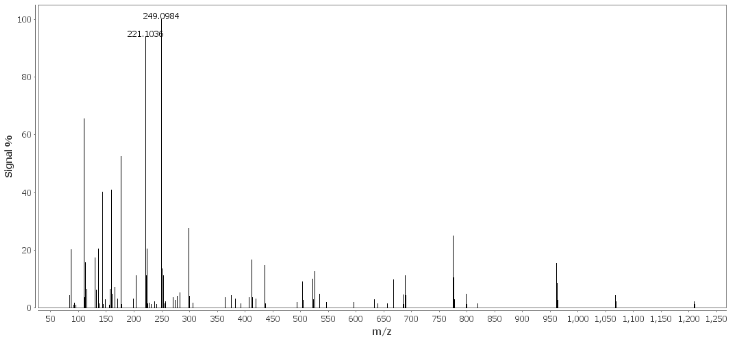

MS2 (+) FT activ = HCD:ce =

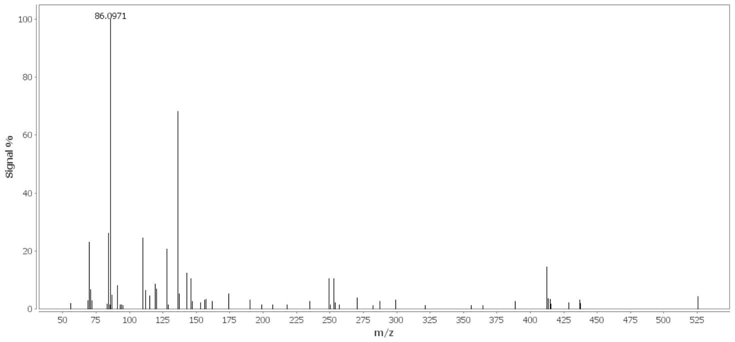

Metabolite: M2 -521 RT=2.05

Type	score	sub. m/z observed	sub. m/z calculated	sub ppm			met. m/z observed	met. m/z calculated	met. ppm
MATCH	200.0	605.3311	605.3300	-1.79	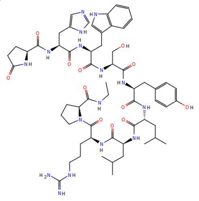	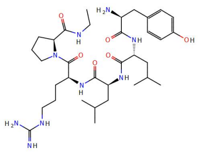	344.7295	344.7289	-1.86
						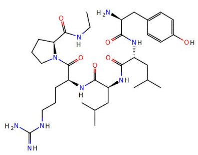	344.7295	344.7289	-1.86

Metabolite: M2 -521 RT=2.05

Type	score	sub. m/z observed	sub. m/z calculated	sub ppm		met. m/z observed	met. m/z calculated	met. ppm
MATCH	101.8	1209.6547	1209.6527	-1.59	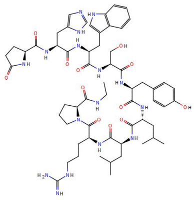 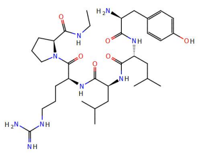	344.7295	344.7289	-1.86
					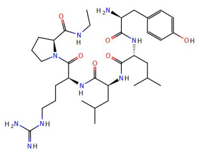	344.7295	344.7289	-1.86
MATCH	120.2	86.0972	86.0964	-9.02	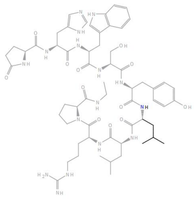 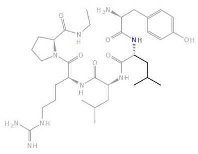	86.0971	86.0964	-7.86
MATCH	120.2	86.0972	86.0964	-9.02	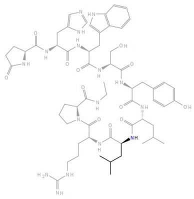 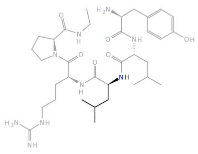	86.0971	86.0964	-7.86
MATCH	3.1	93.0456	93.0553	104.5	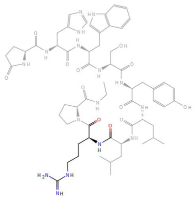 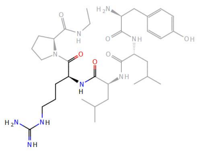	93.0453	93.0553	107.1
MATCH	22.2	112.0874	112.0869	-4.67	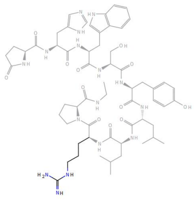 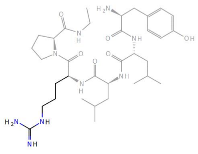	112.0873	112.0869	-3.55
MATCH	11.0	115.0870	115.0866	-3.97	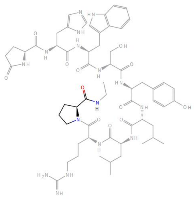 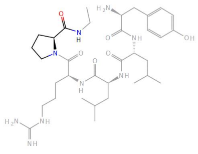	115.0869	115.0866	-2.71
MATCH	52.5	143.1181	143.1179	-1.70	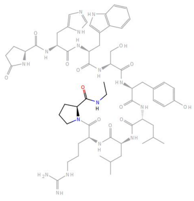 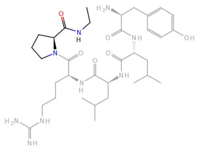	143.1180	143.1179	-1.10
MATCH	4.4	199.1802	199.1805	1.48	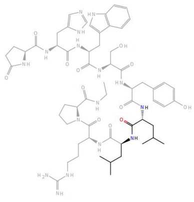 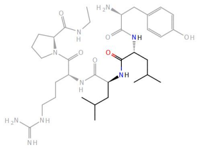	199.1804	199.1805	0.20

Metabolite: M2 -521 RT=2.05

Type	score	sub. m/z observed	sub. m/z calculated	sub ppm		met. m/z observed	met. m/z calculated	met. ppm
MATCH	21.5	253.1658	253.1659	0.37	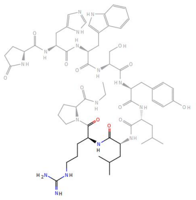 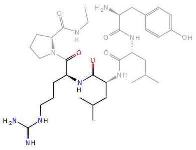	253.1656	253.1659	1.13
MATCH	7.3	270.1925	270.1925	-0.02	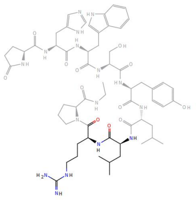 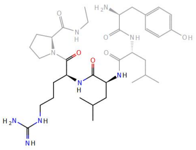	270.1925	270.1925	-0.02
MATCH	6.5	282.1930	282.1925	-1.77	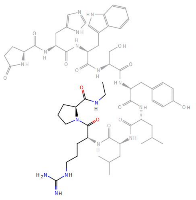 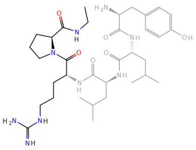	282.1923	282.1925	0.57
MATCH	30.5	299.2194	299.2190	-1.47	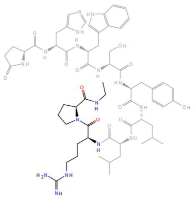 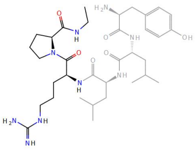	299.2190	299.2190	0.07
MATCH	31.1	412.3040	412.3031	-2.34	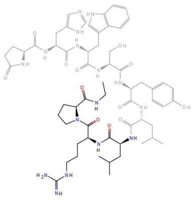 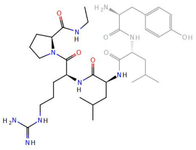	412.3034	412.3031	-0.75
MATCH	16.9	525.3876	525.3871	-0.80	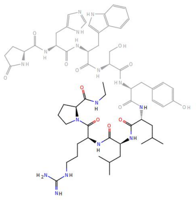 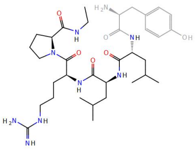	525.3881	525.3871	-1.91
MATCH	69.5	657.2772	657.2780	1.10	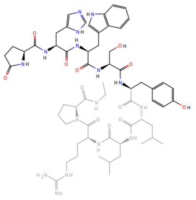 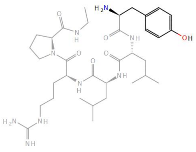	136.0758	136.0757	-0.73
MISMATCH	-3.1	93.0456	93.0447	-8.98	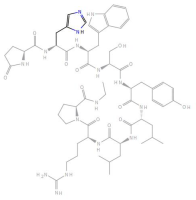	93.0453	93.0453	0.00
MISMATCH	-90.1	110.0718	110.0713	-4.79	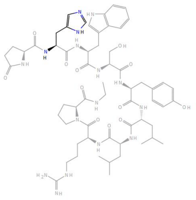	110.0717	110.0717	0.00

Metabolite: M2 -521 RT=2.05

Type	score	sub. m/z observed	sub. m/z calculated	sub ppm		met. m/z observed	met. m/z calculated	met. ppm
MET_MATCH					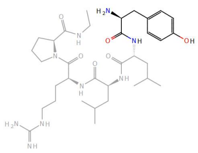	91.0549	91.0522	-29.4
MET_MATCH					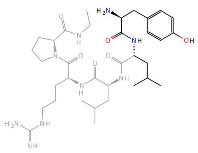	249.1595	249.1598	1.13
