# Supplementary material for: Software-aided approach to investigate peptide structure and metabolic susceptibility of amide bonds in peptide drugs based on high resolution mass spectrometry
Source: PLoS One. 2017 Nov 1;12(11):e0186461. doi: 10.1371/journal.pone.0186461 (PMC5665424; doi:10.1371/journal.pone.0186461)
Supplement: S1 File — (ZIP) [file pone.0186461.s007.zip › SFiles/S24_File.pdf]

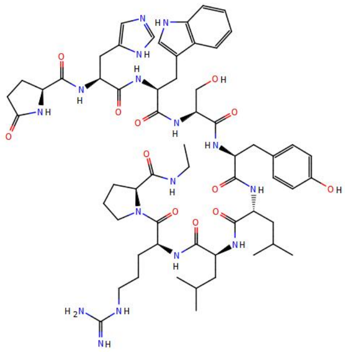

# Leuprolide

| Property name | Property value |
|---|---|
| Time | 0min, 5min, 15min, 45min, 120min |
| Instrument | ThermoQAPlus |
| Acquisition Mode | ddMS2 |
| Matrix | pepsin |

## Chromatograms

### Time=0min

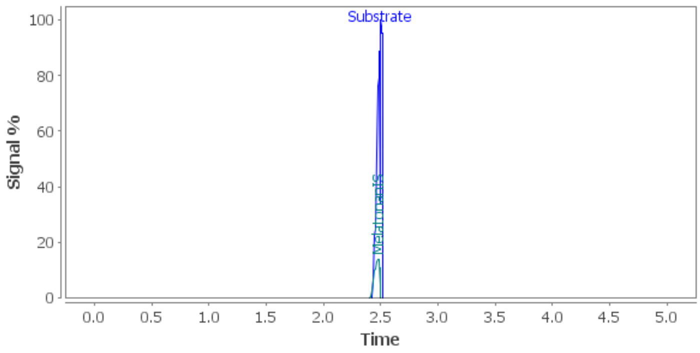


### Time=5min

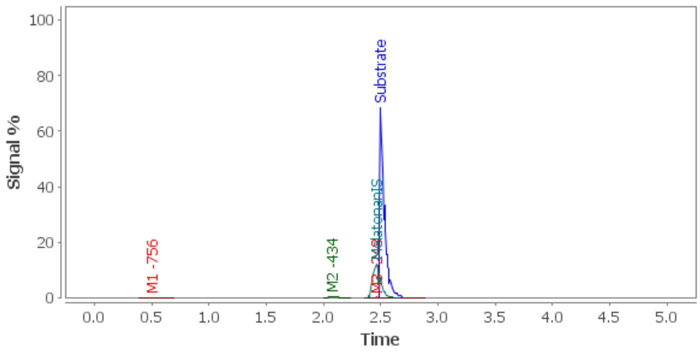


### Time=15min

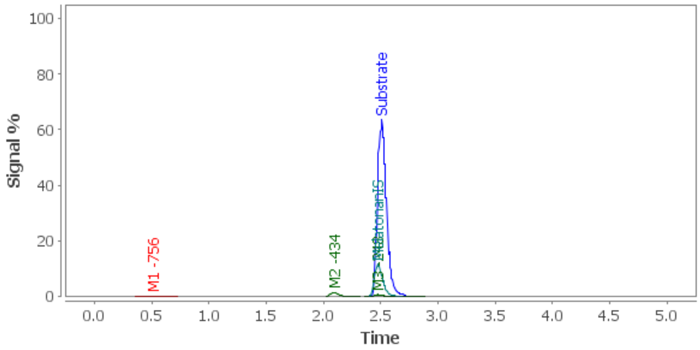


### Time=45min

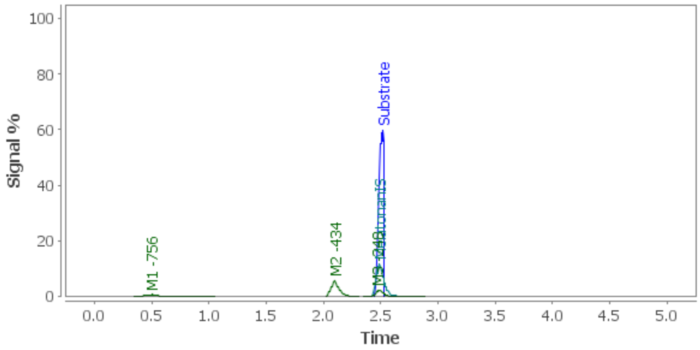


### Time=120min

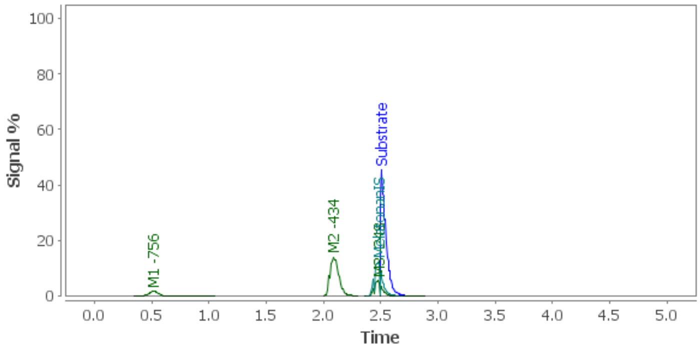

# Custom Charts

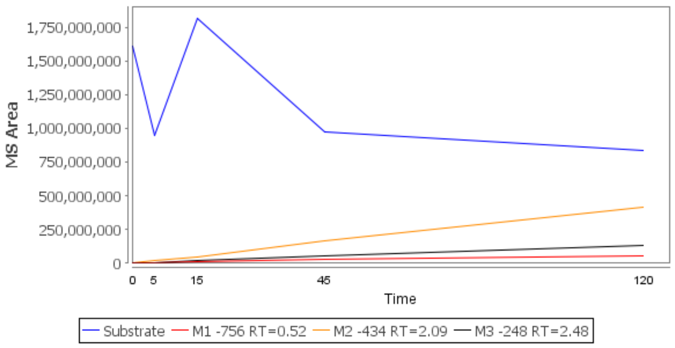

# Fragmentation

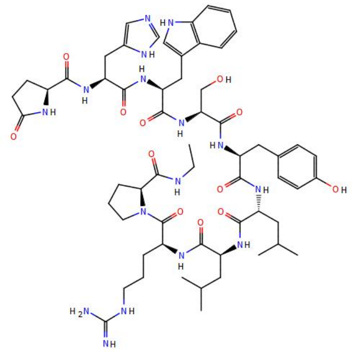

Leuprolide

MS (+) FT

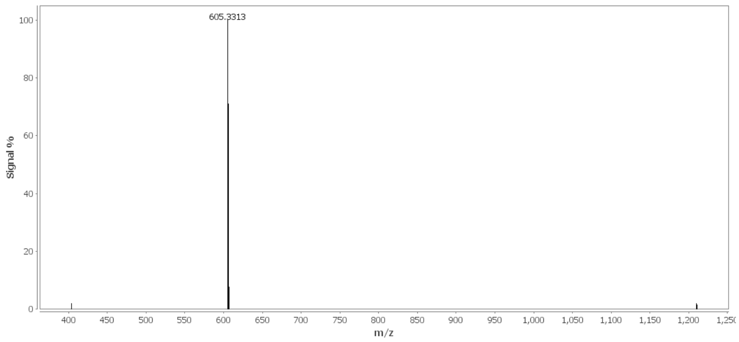


MS (+) FT

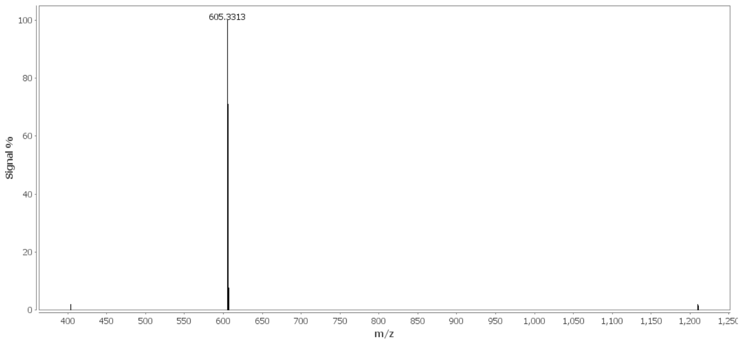


MS2 (+) FT activ = HCD:ce =

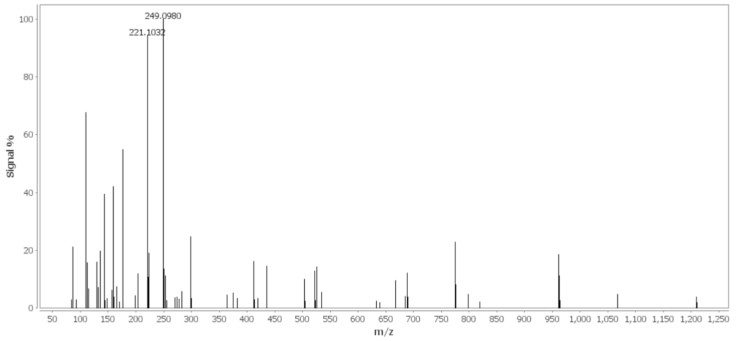


MS2 (+) FT activ = HCD:ce =

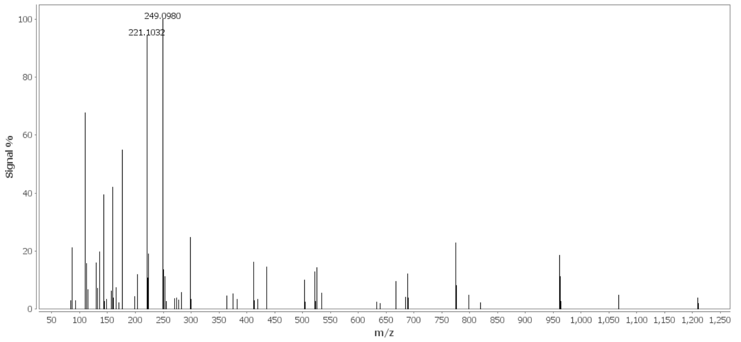


Metabolite: Substrate

| Type | score | sub. m/z observed | sub. m/z calculated | sub ppm | | | met. m/z observed | met. m/z calculated | met. ppm |
|---|---|---|---|---|---|---|---|---|---|
| MATCH | 102.0 | 1209.6540 | 1209.6527 | -1.07 | 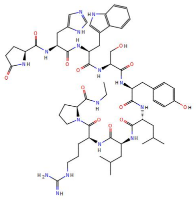 | 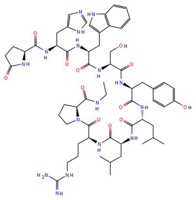 | 1209.6540 | 1209.6527 | -1.07 |
| MATCH | 13.9 | 1209.6530 | 1209.6527 | -0.24 | 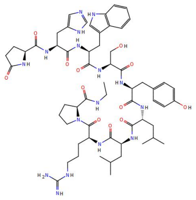 | 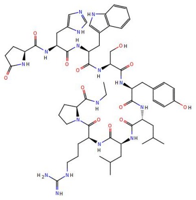 | 1209.6530 | 1209.6527 | -0.24 |
| MATCH | 6.7 | 798.3591 | 798.3570 | -2.72 | 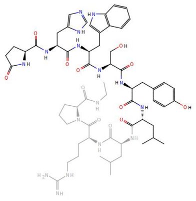 | 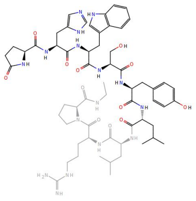 | 798.3591 | 798.3570 | -2.72 |

Metabolite: Substrate

| Type | score | sub. m/z observed | sub. m/z calculated | sub ppm | | | met. m/z observed | met. m/z calculated | met. ppm |
|---|---|---|---|---|---|---|---|---|---|
| MATCH | 37.0 | 775.4812 | 775.4825 | 1.69 | 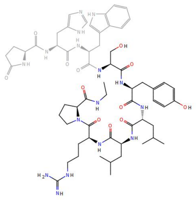 | 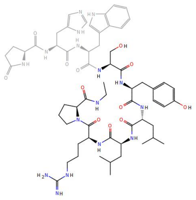 | 775.4812 | 775.4825 | 1.69 |
| MATCH | 30.9 | 688.4495 | 688.4505 | 1.44 | 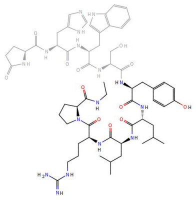 | 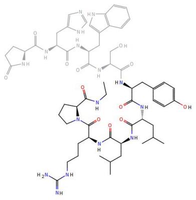 | 688.4495 | 688.4505 | 1.44 |
| MATCH | 20.3 | 685.2681 | 685.2729 | 7.02 | 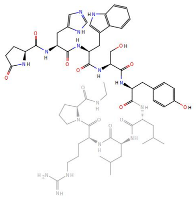 | 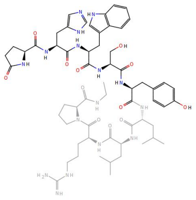 | 685.2681 | 685.2729 | 7.02 |
| MATCH | 15.6 | 667.2608 | 667.2623 | 2.28 | 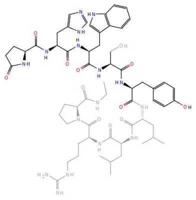 | 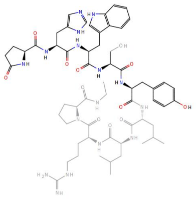 | 667.2608 | 667.2623 | 2.28 |
| MATCH | 3.6 | 633.3670 | 633.3719 | 7.70 | 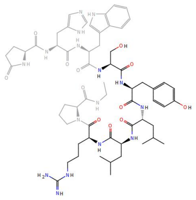 | 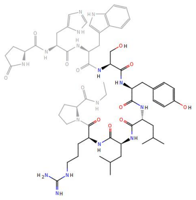 | 633.3670 | 633.3719 | 7.70 |
| MATCH | 200.0 | 605.3313 | 605.3300 | -2.14 | 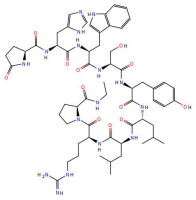 | 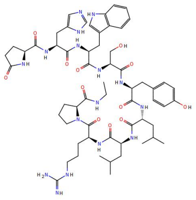 | 605.3313 | 605.3300 | -2.14 |
| MATCH | 30.6 | 525.3871 | 525.3871 | 0.07 | 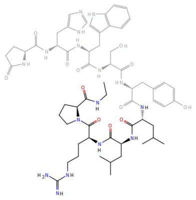 | 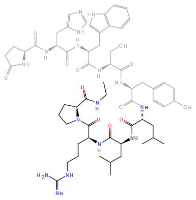 | 525.3871 | 525.3871 | 0.07 |
| MATCH | 18.5 | 522.2090 | 522.2096 | 1.05 | 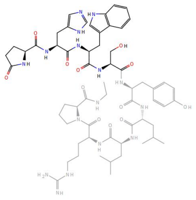 | 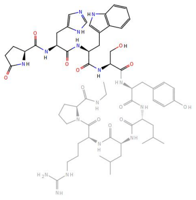 | 522.2090 | 522.2096 | 1.05 |
| MATCH | 15.7 | 504.1985 | 504.1990 | 0.92 | 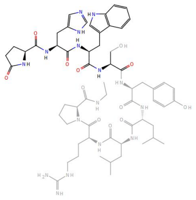 | 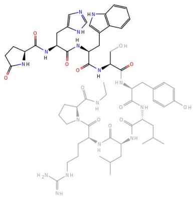 | 504.1985 | 504.1990 | 0.92 |

Metabolite: Substrate

| Type | score | sub. m/z observed | sub. m/z calculated | sub ppm | | | met. m/z observed | met. m/z calculated | met. ppm |
|---|---|---|---|---|---|---|---|---|---|
| MATCH | 17.4 | 435.1772 | 435.1775 | 0.77 | 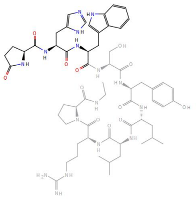 | 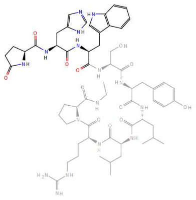 | 435.1772 | 435.1775 | 0.77 |
| MATCH | 39.7 | 412.3026 | 412.3031 | 1.04 | 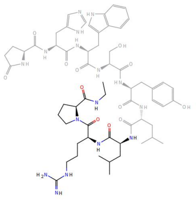 | 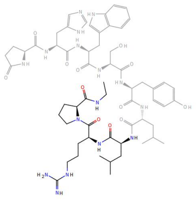 | 412.3026 | 412.3031 | 1.04 |
| MATCH | 102.0 | 403.8897 | 403.8891 | -1.45 | 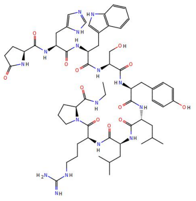 | 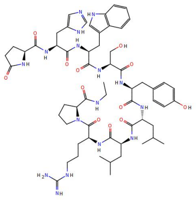 | 403.8897 | 403.8891 | -1.45 |
| MATCH | 10.7 | 383.2754 | 383.2765 | 2.83 | 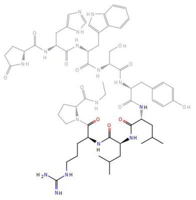 | 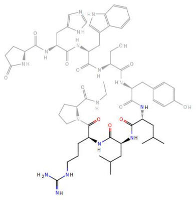 | 383.2754 | 383.2765 | 2.83 |
| MATCH | 6.4 | 364.1880 | 364.1867 | -3.62 | 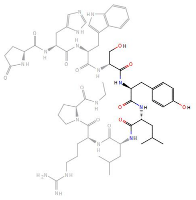 | 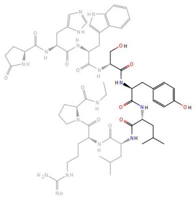 | 364.1880 | 364.1867 | -3.62 |
| MATCH | 6.0 | 364.1880 | 364.1867 | -3.62 | 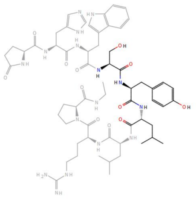 | 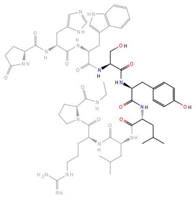 | 364.1880 | 364.1867 | -3.62 |
| MATCH | 45.4 | 299.2189 | 299.2190 | 0.45 | 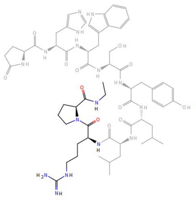 | 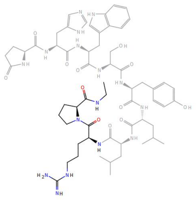 | 299.2189 | 299.2190 | 0.45 |
| MATCH | 11.0 | 282.1935 | 282.1925 | -3.54 | 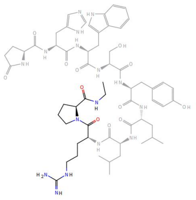 | 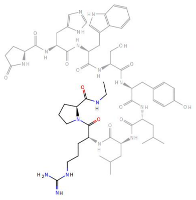 | 282.1935 | 282.1925 | -3.54 |
| MATCH | 7.0 | 277.1543 | 277.1547 | 1.25 | 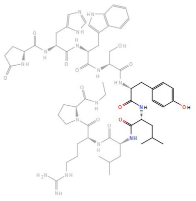 | 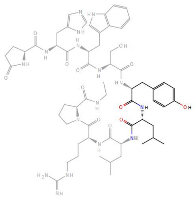 | 277.1543 | 277.1547 | 1.25 |

Metabolite: Substrate

| Type | score | sub. m/z observed | sub. m/z calculated | sub ppm | | | met. m/z observed | met. m/z calculated | met. ppm |
|---|---|---|---|---|---|---|---|---|---|
| MATCH | 7.0 | 277.1543 | 277.1547 | 1.25 | 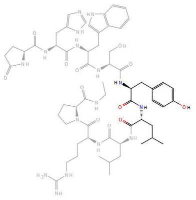 | 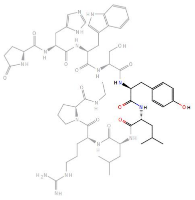 | 277.1543 | 277.1547 | 1.25 |
| MATCH | 9.5 | 274.1184 | 274.1186 | 0.84 | 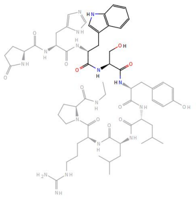 | 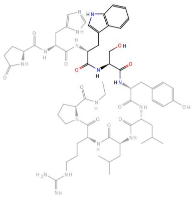 | 274.1184 | 274.1186 | 0.84 |
| MATCH | 10.7 | 270.1921 | 270.1925 | 1.30 | 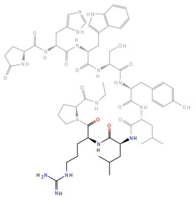 | 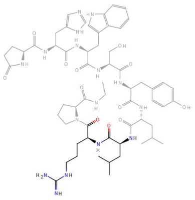 | 270.1921 | 270.1925 | 1.30 |
| MATCH | 35.7 | 253.1657 | 253.1659 | 0.90 | 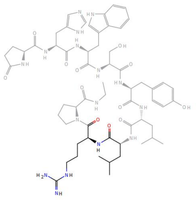 | 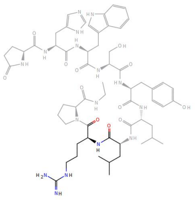 | 253.1657 | 253.1659 | 0.90 |
| MATCH | 14.1 | 249.1594 | 249.1598 | 1.48 | 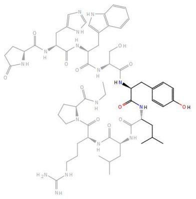 | 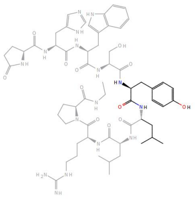 | 249.1594 | 249.1598 | 1.48 |
| MATCH | 174.2 | 249.0980 | 249.0982 | 0.71 | 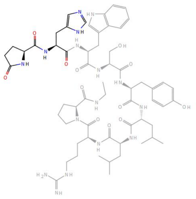 | 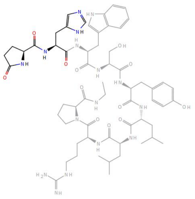 | 249.0980 | 249.0982 | 0.71 |
| MATCH | 175.7 | 221.1032 | 221.1033 | 0.47 | 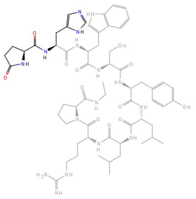 | 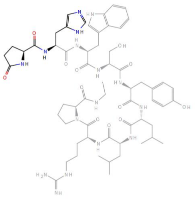 | 221.1032 | 221.1033 | 0.47 |
| MATCH | 11.0 | 199.1803 | 199.1805 | 1.17 | 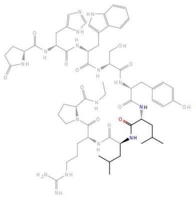 | 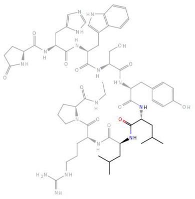 | 199.1803 | 199.1805 | 1.17 |
| MATCH | 43.1 | 170.0601 | 170.0600 | -0.63 | 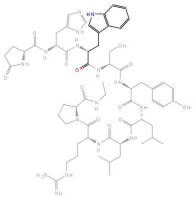 | 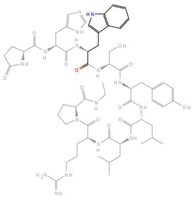 | 170.0601 | 170.0600 | -0.63 |

Metabolite: Substrate

| Type | score | sub. m/z observed | sub. m/z calculated | sub ppm | | | met. m/z observed | met. m/z calculated | met. ppm |
|---|---|---|---|---|---|---|---|---|---|
| MATCH | 15.6 | 166.0609 | 166.0611 | 0.96 | 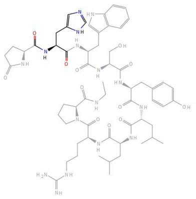 | 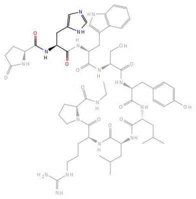 | 166.0609 | 166.0611 | 0.96 |
| MATCH | 54.1 | 159.0917 | 159.0917 | -0.08 | 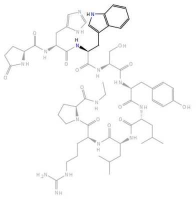 | 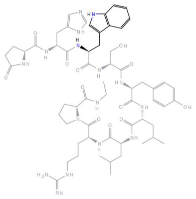 | 159.0917 | 159.0917 | -0.08 |
| MATCH | 12.8 | 157.1082 | 157.1084 | 1.03 | 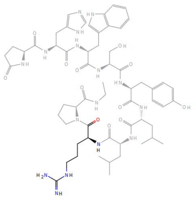 | 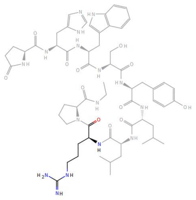 | 157.1082 | 157.1084 | 1.03 |
| MATCH | 79.9 | 143.1179 | 143.1179 | -0.39 | 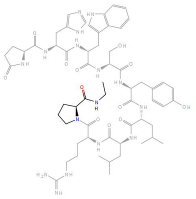 | 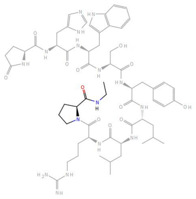 | 143.1179 | 143.1179 | -0.39 |
| MATCH | 99.3 | 136.0758 | 136.0757 | -0.68 | 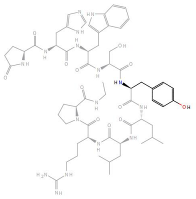 | 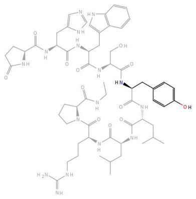 | 136.0758 | 136.0757 | -0.68 |
| MATCH | 83.8 | 130.0651 | 130.0575 | -58.4 | 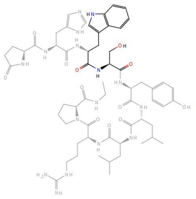 | 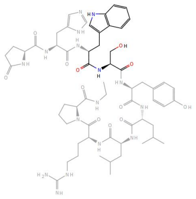 | 130.0651 | 130.0575 | -58.4 |
| MATCH | 15.4 | 115.0868 | 115.0866 | -2.20 | 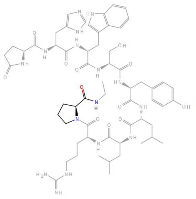 | 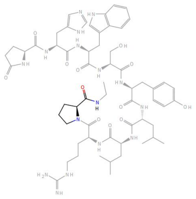 | 115.0868 | 115.0866 | -2.20 |
| MATCH | 35.9 | 112.0872 | 112.0869 | -2.51 | 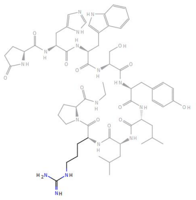 | 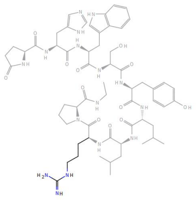 | 112.0872 | 112.0869 | -2.51 |
| MATCH | 167.7 | 110.0716 | 110.0713 | -3.25 | 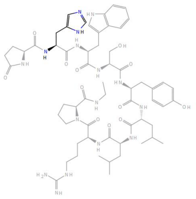 | 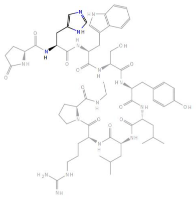 | 110.0716 | 110.0713 | -3.25 |

Metabolite: Substrate

| Type | score | sub. m/z observed | sub. m/z calculated | sub ppm | | | met. m/z observed | met. m/z calculated | met. ppm |
|---|---|---|---|---|---|---|---|---|---|
| MATCH | 4.6 | 93.0453 | 93.0447 | -6.45 | 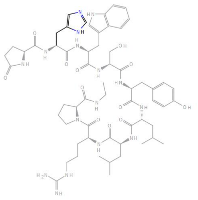 | 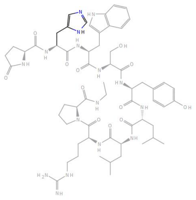 | 93.0453 | 93.0447 | -6.45 |
| MATCH | 86.2 | 86.0971 | 86.0964 | -7.32 | 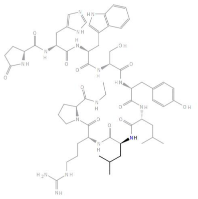 | 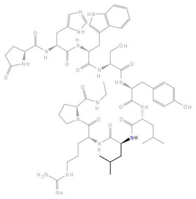 | 86.0971 | 86.0964 | -7.32 |
| MATCH | 86.2 | 86.0971 | 86.0964 | -7.32 | 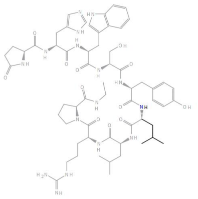 | 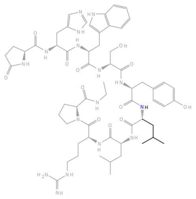 | 86.0971 | 86.0964 | -7.32 |
| MATCH | 12.2 | 84.0453 | 84.0444 | -11.3 | 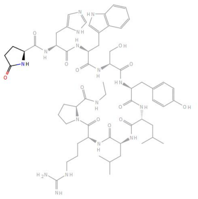 | 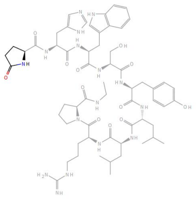 | 84.0453 | 84.0444 | -11.3 |

MS (+) FT

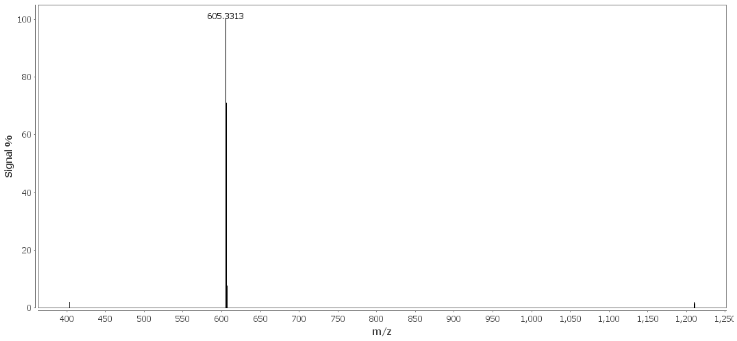


MS (+) FT

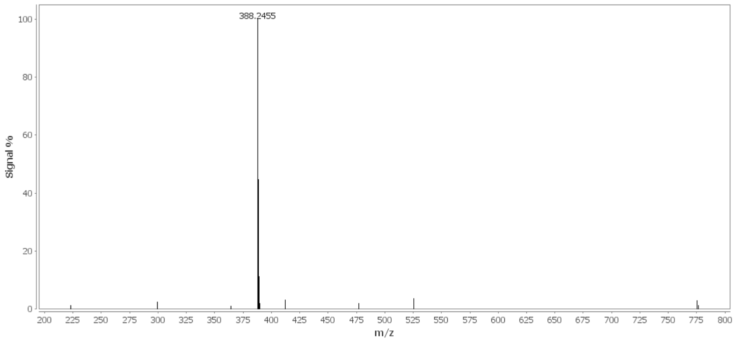


MS2 (+) FT activ = HCD:ce =

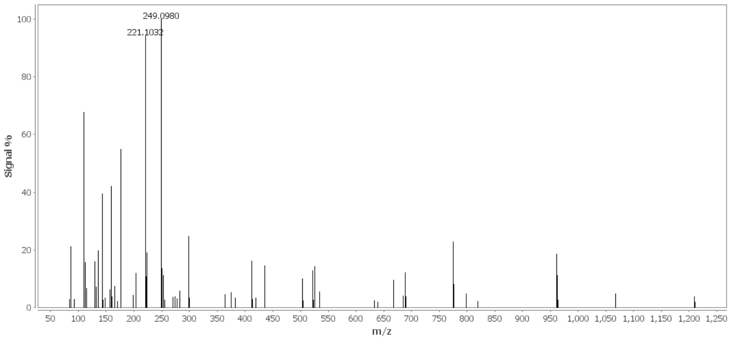


MS2 (+) FT activ = HCD:ce =

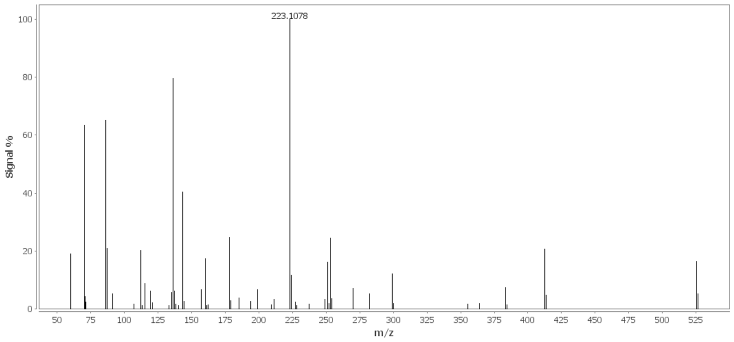


Metabolite: M2 -434 RT=2.09

| Type | score | sub. m/z observed | sub. m/z calculated | sub ppm | met. m/z observed | met. m/z calculated | met. ppm |
|---|---|---|---|---|---|---|---|

Metabolite: M2 -434 RT=2.09

| Type | score | sub. m/z observed | sub. m/z calculated | sub ppm | | | met. m/z observed | met. m/z calculated | met. ppm |
|---|---|---|---|---|---|---|---|---|---|
| MATCH | 102.0 | 403.8897 | 403.8891 | -1.45 | 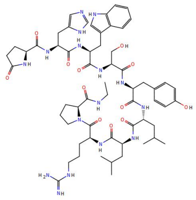 | 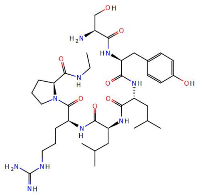 | 388.2455 | 388.2449 | -1.53 |
| MATCH | 102.0 | 403.8897 | 403.8891 | -1.45 | 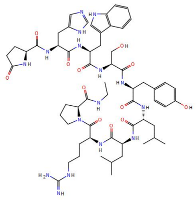 | 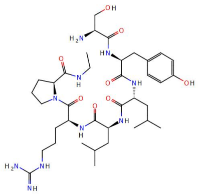 | 388.2455 | 388.2449 | -1.53 |
| | | | | | | 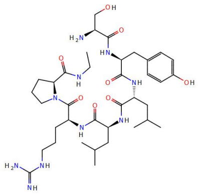 | 388.2455 | 388.2449 | -1.53 |
| MATCH | 5.0 | 403.8897 | 403.8891 | -1.45 | 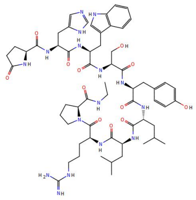 | 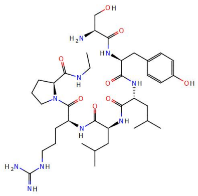 | 775.4829 | 775.4825 | -0.56 |
| MATCH | 5.0 | 403.8897 | 403.8891 | -1.45 | 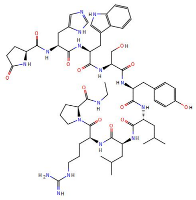 | 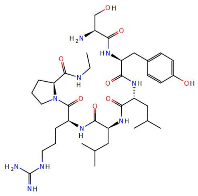 | 775.4829 | 775.4825 | -0.56 |
| | | | | | | 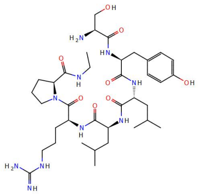 | 775.4829 | 775.4825 | -0.56 |
| MATCH | 200.0 | 605.3313 | 605.3300 | -2.14 | 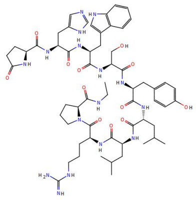 | 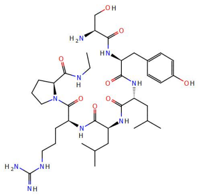 | 388.2455 | 388.2449 | -1.53 |
| MATCH | 200.0 | 605.3313 | 605.3300 | -2.14 | 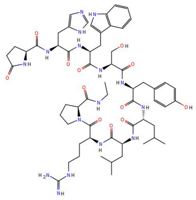 | 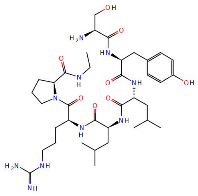 | 388.2455 | 388.2449 | -1.53 |
| | | | | | | 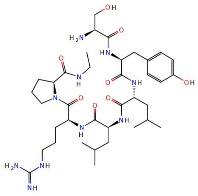 | 388.2455 | 388.2449 | -1.53 |

Metabolite: M2 -434 RT=2.09

| Type | score | sub. m/z observed | sub. m/z calculated | sub ppm | | | met. m/z observed | met. m/z calculated | met. ppm |
|---|---|---|---|---|---|---|---|---|---|
| MATCH | 103.0 | 605.3313 | 605.3300 | -2.14 | 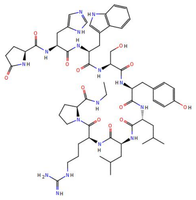 | 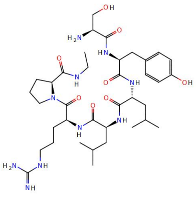 | 775.4829 | 775.4825 | -0.56 |
| MATCH | 103.0 | 605.3313 | 605.3300 | -2.14 | 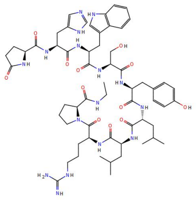 | 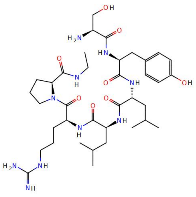 | 775.4829 | 775.4825 | -0.56 |
| | | | | | | 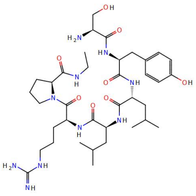 | 775.4829 | 775.4825 | -0.56 |
| MATCH | 102.0 | 1209.6540 | 1209.6527 | -1.07 | 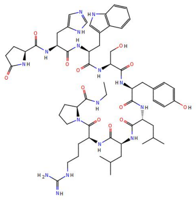 | 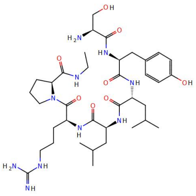 | 388.2455 | 388.2449 | -1.53 |
| MATCH | 102.0 | 1209.6540 | 1209.6527 | -1.07 | 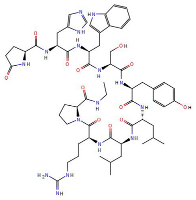 | 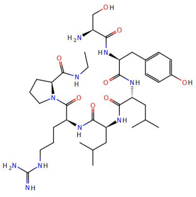 | 388.2455 | 388.2449 | -1.53 |
| | | | | | | 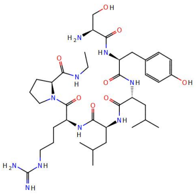 | 388.2455 | 388.2449 | -1.53 |
| MATCH | 5.0 | 1209.6540 | 1209.6527 | -1.07 | 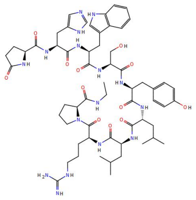 | 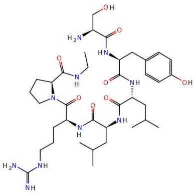 | 775.4829 | 775.4825 | -0.56 |
| MATCH | 5.0 | 1209.6540 | 1209.6527 | -1.07 | 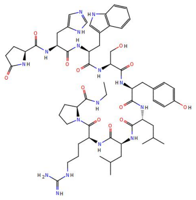 | 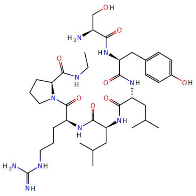 | 775.4829 | 775.4825 | -0.56 |
| | | | | | | 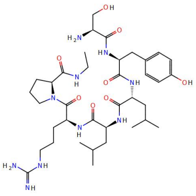 | 775.4829 | 775.4825 | -0.56 |

Metabolite: M2 -434 RT=2.09

| Type | score | sub. m/z observed | sub. m/z calculated | sub ppm | | | met. m/z observed | met. m/z calculated | met. ppm |
|---|---|---|---|---|---|---|---|---|---|
| MATCH | 86.2 | 86.0971 | 86.0964 | -7.32 | 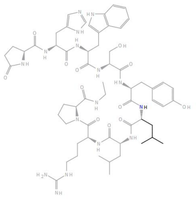 | 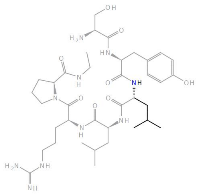 | 86.0972 | 86.0964 | -8.58 |
| MATCH | 86.2 | 86.0971 | 86.0964 | -7.32 | 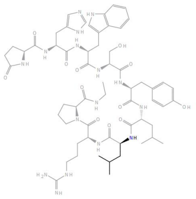 | 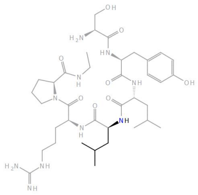 | 86.0972 | 86.0964 | -8.58 |
| MATCH | 35.9 | 112.0872 | 112.0869 | -2.51 | 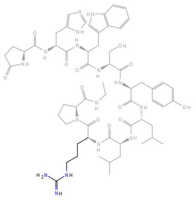 | 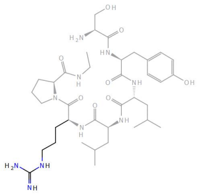 | 112.0874 | 112.0869 | -4.08 |
| MATCH | 15.4 | 115.0868 | 115.0866 | -2.20 | 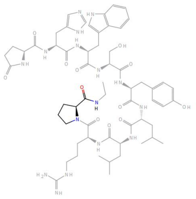 | 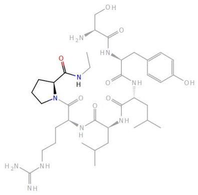 | 115.0869 | 115.0866 | -2.98 |
| MATCH | 99.3 | 136.0758 | 136.0757 | -0.68 | 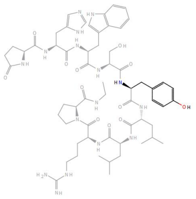 | 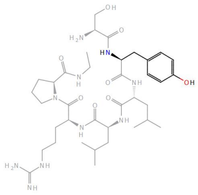 | 136.0759 | 136.0757 | -1.51 |
| MATCH | 79.9 | 143.1179 | 143.1179 | -0.39 | 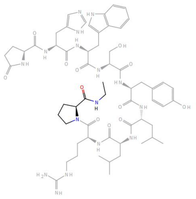 | 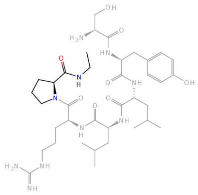 | 143.1181 | 143.1179 | -1.20 |
| MATCH | 12.8 | 157.1082 | 157.1084 | 1.03 | 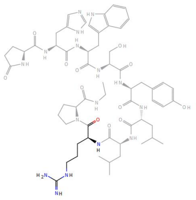 | 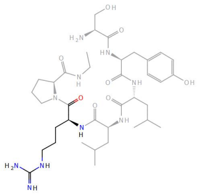 | 157.1086 | 157.1084 | -1.27 |
| MATCH | 11.0 | 199.1803 | 199.1805 | 1.17 | 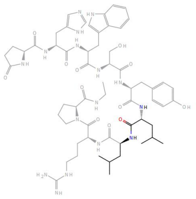 | 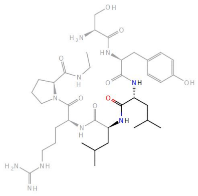 | 199.1806 | 199.1805 | -0.65 |
| MATCH | 8.2 | 249.1594 | 249.1598 | 1.48 | 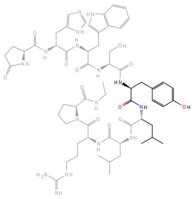 | 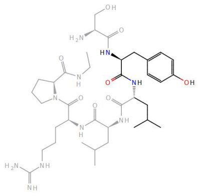 | 249.1598 | 249.1598 | -0.28 |

Metabolite: M2 -434 RT=2.09

| Type | score | sub. m/z observed | sub. m/z calculated | sub ppm | | | met. m/z observed | met. m/z calculated | met. ppm |
|---|---|---|---|---|---|---|---|---|---|
| MATCH | 35.7 | 253.1657 | 253.1659 | 0.90 | 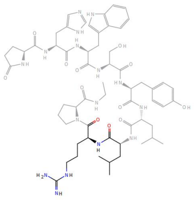 | 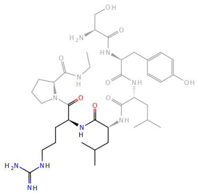 | 253.1660 | 253.1659 | -0.35 |
| MATCH | 10.7 | 270.1921 | 270.1925 | 1.30 | 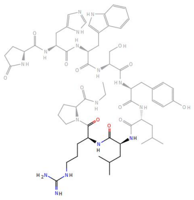 | 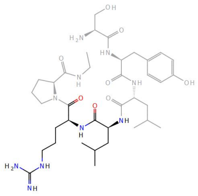 | 270.1927 | 270.1925 | -0.78 |
| MATCH | 11.0 | 282.1935 | 282.1925 | -3.54 | 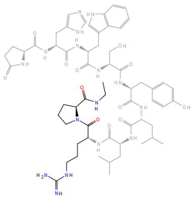 | 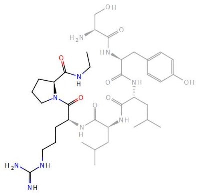 | 282.1923 | 282.1925 | 0.42 |
| MATCH | 36.9 | 299.2189 | 299.2190 | 0.45 | 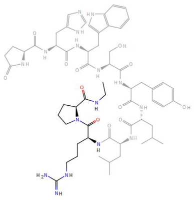 | 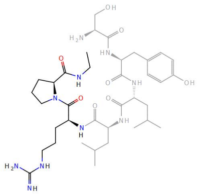 | 299.2192 | 299.2190 | -0.81 |
| MATCH | 6.4 | 364.1880 | 364.1867 | -3.62 | 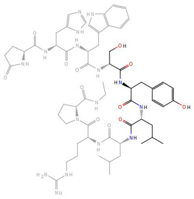 | 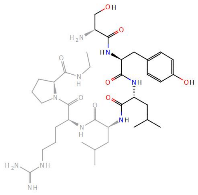 | 364.1869 | 364.1867 | -0.49 |
| | | | | | | 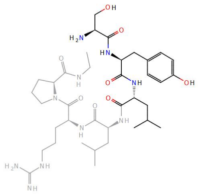 | 364.1869 | 364.1867 | -0.49 |
| MATCH | 10.7 | 383.2754 | 383.2765 | 2.83 | 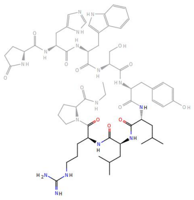 | 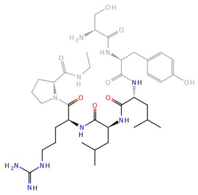 | 383.2762 | 383.2765 | 0.85 |
| MATCH | 36.8 | 412.3026 | 412.3031 | 1.04 | 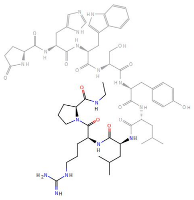 | 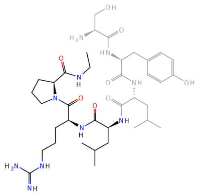 | 412.3033 | 412.3031 | -0.59 |
| MATCH | 12.6 | 504.1985 | 504.1990 | 0.92 | 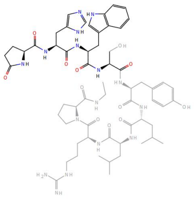 | 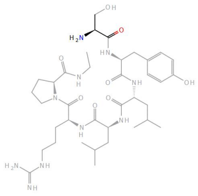 | 70.0296 | 70.0287 | -12.6 |

Metabolite: M2 -434 RT=2.09

| Type | score | sub. m/z observed | sub. m/z calculated | sub ppm | | | met. m/z observed | met. m/z calculated | met. ppm |
|---|---|---|---|---|---|---|---|---|---|
| MATCH | 30.6 | 525.3871 | 525.3871 | 0.07 | 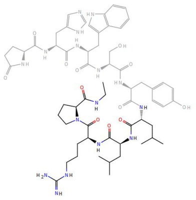 | 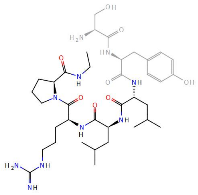 | 525.3879 | 525.3871 | -1.44 |
| MATCH | 20.3 | 685.2681 | 685.2729 | 7.02 | 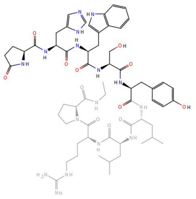 | 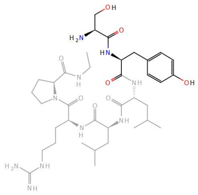 | 251.1027 | 251.1026 | -0.42 |
| MATCH | 6.7 | 798.3591 | 798.3570 | -2.72 | 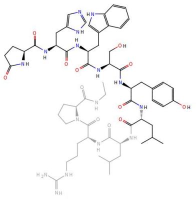 | 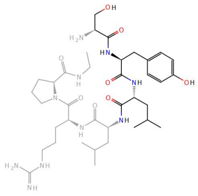 | 364.1869 | 364.1867 | -0.49 |
| | | | | | | 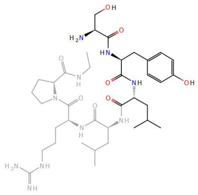 | 364.1869 | 364.1867 | -0.49 |
| MET_MATCH | | | | | | 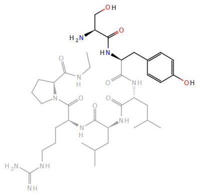 | 223.1080 | 223.1077 | -1.16 |
| MET_MATCH | | | | | | 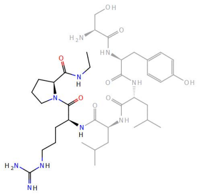 | 299.2197 | 299.2190 | -2.20 |
| MET_MATCH | | | | | | 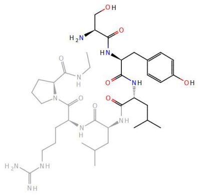 | 364.1873 | 364.1867 | -1.54 |
| MET_MATCH | | | | | | 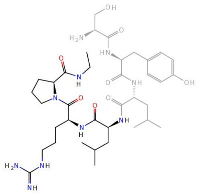 | 412.3038 | 412.3031 | -1.76 |
| MET_MATCH | | | | | | 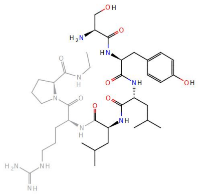 | 477.2717 | 477.2708 | -1.98 |

Metabolite: M2 -434 RT=2.09

| Type | score | sub. m/z observed | sub. m/z calculated | sub ppm | | met. m/z observed | met. m/z calculated | met. ppm |
|---|---|---|---|---|---|---|---|---|
| MET_MATCH | | | | | 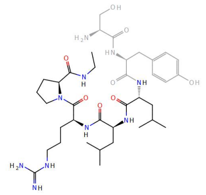 | 525.3879 | 525.3871 | -1.50 |
| MET_MATCH | | | | | 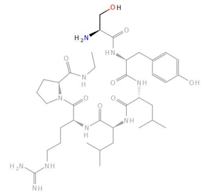 | 60.0453 | 60.0444 | -15.8 |
| MET_MATCH | | | | | 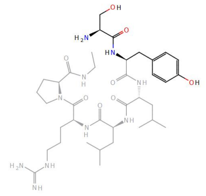 | 223.1078 | 223.1077 | -0.28 |

MS (+) FT

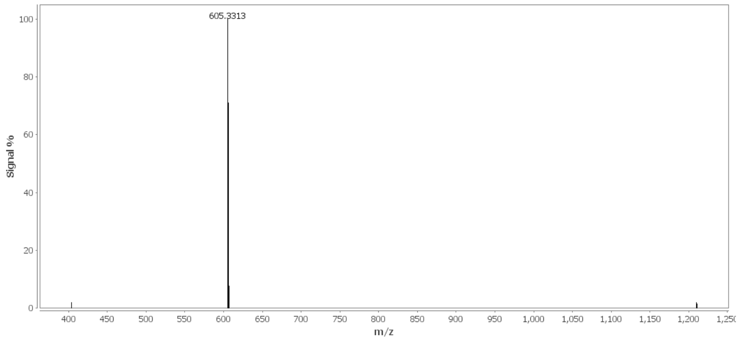


MS (+) FT

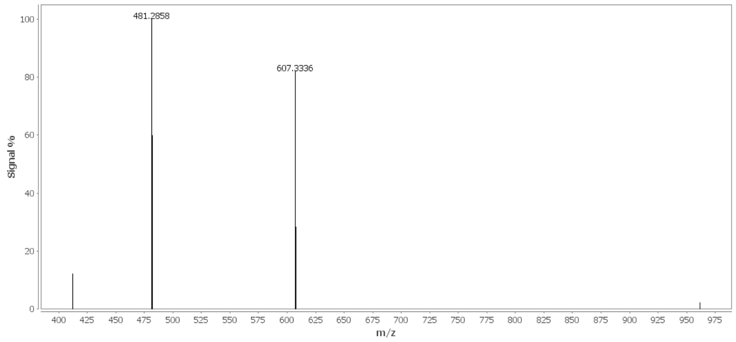


MS2 (+) FT activ = HCD:ce =

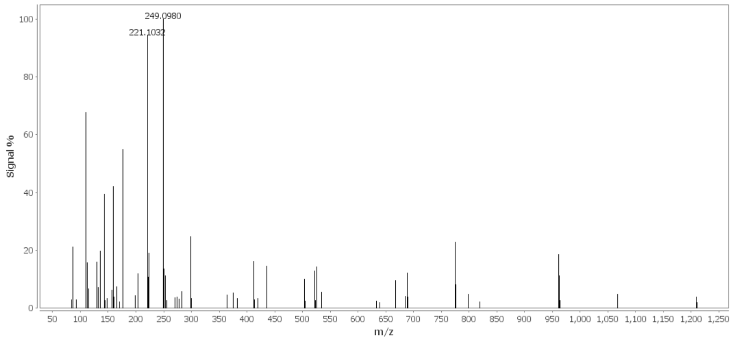


MS2 (+) FT activ = HCD:ce =

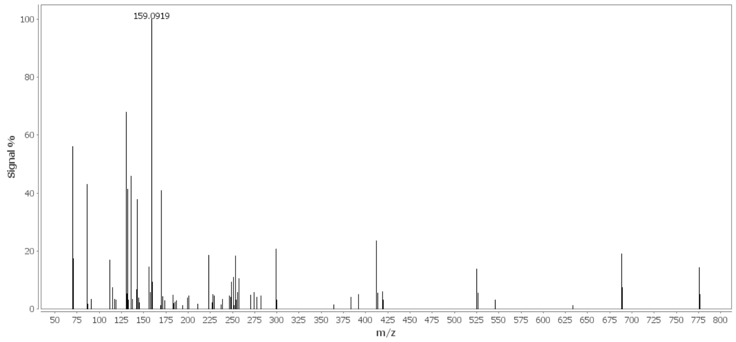


Metabolite: M3 -248 RT=2.48

| Type | score | sub. m/z observed | sub. m/z calculated | sub ppm | | | met. m/z observed | met. m/z calculated | met. ppm |
|---|---|---|---|---|---|---|---|---|---|
| MATCH | 102.0 | 403.8897 | 403.8891 | -1.45 | 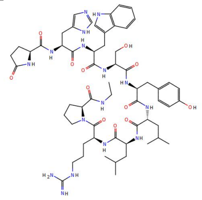 | 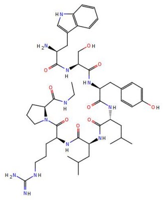 | 481.2858 | 481.2845 | -2.59 |

Metabolite: M3 -248 RT=2.48

| Type | score | sub. m/z observed | sub. m/z calculated | sub ppm | | | met. m/z observed | met. m/z calculated | met. ppm |
|---|---|---|---|---|---|---|---|---|---|
| MATCH | 102.0 | 403.8897 | 403.8891 | -1.45 | 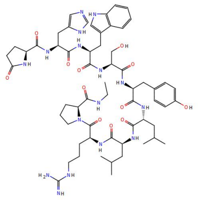 | 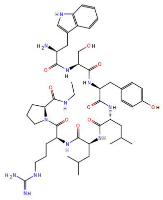 | 481.2858 | 481.2845 | -2.59 |
| | | | | | | 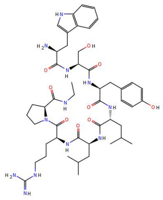 | 481.2858 | 481.2845 | -2.59 |
| MATCH | 4.3 | 403.8897 | 403.8891 | -1.45 | 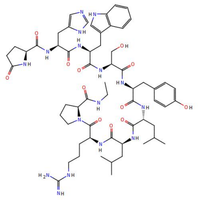 | 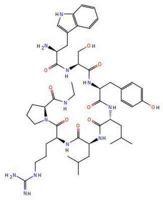 | 961.5596 | 961.5618 | 2.28 |
| MATCH | 4.3 | 403.8897 | 403.8891 | -1.45 | 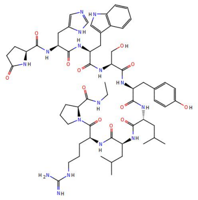 | 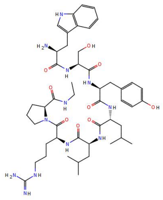 | 961.5596 | 961.5618 | 2.28 |
| | | | | | | 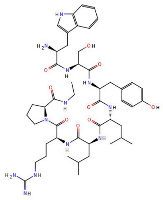 | 961.5596 | 961.5618 | 2.28 |
| MATCH | 200.0 | 605.3313 | 605.3300 | -2.14 | 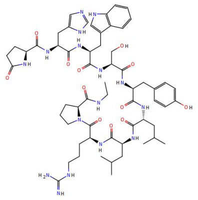 | 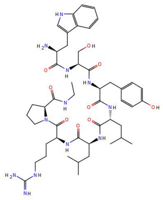 | 481.2858 | 481.2845 | -2.59 |
| MATCH | 200.0 | 605.3313 | 605.3300 | -2.14 | 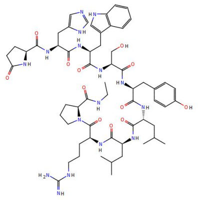 | 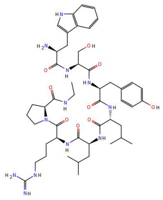 | 481.2858 | 481.2845 | -2.59 |
| | | | | | | 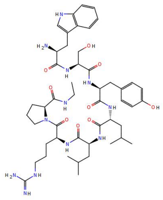 | 481.2858 | 481.2845 | -2.59 |
| MATCH | 102.3 | 605.3313 | 605.3300 | -2.14 | 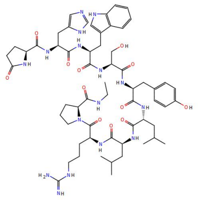 | 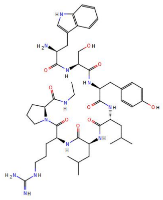 | 961.5596 | 961.5618 | 2.28 |

Metabolite: M3 -248 RT=2.48

| Type | score | sub. m/z observed | sub. m/z calculated | sub ppm | | | met. m/z observed | met. m/z calculated | met. ppm |
|---|---|---|---|---|---|---|---|---|---|
| MATCH | 102.3 | 605.3313 | 605.3300 | -2.14 | 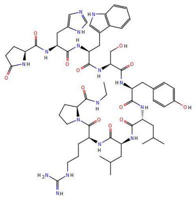 | 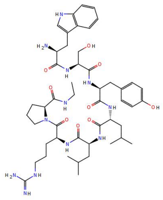 | 961.5596 | 961.5618 | 2.28 |
| | | | | | | 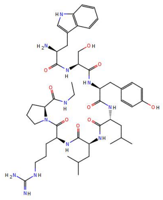 | 961.5596 | 961.5618 | 2.28 |
| MATCH | 102.0 | 1209.6540 | 1209.6527 | -1.07 | 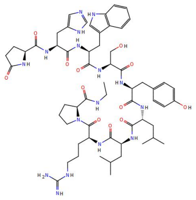 | 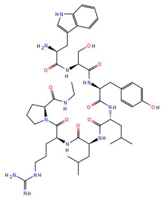 | 481.2858 | 481.2845 | -2.59 |
| MATCH | 102.0 | 1209.6540 | 1209.6527 | -1.07 | 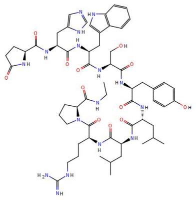 | 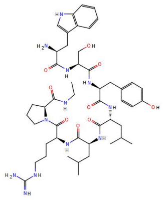 | 481.2858 | 481.2845 | -2.59 |
| | | | | | | 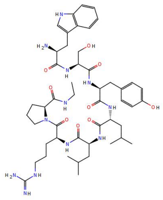 | 481.2858 | 481.2845 | -2.59 |
| MATCH | 4.3 | 1209.6540 | 1209.6527 | -1.07 | 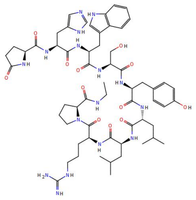 | 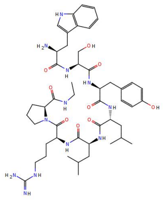 | 961.5596 | 961.5618 | 2.28 |
| MATCH | 4.3 | 1209.6540 | 1209.6527 | -1.07 | 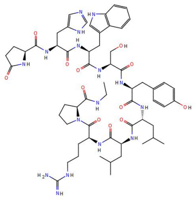 | 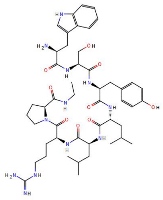 | 961.5596 | 961.5618 | 2.28 |
| | | | | | | 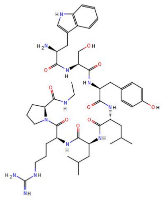 | 961.5596 | 961.5618 | 2.28 |
| MATCH | 64.2 | 86.0971 | 86.0964 | -7.32 | 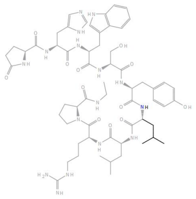 | 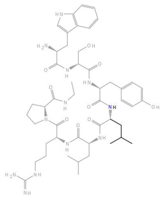 | 86.0972 | 86.0964 | -9.09 |

Metabolite: M3 -248 RT=2.48

| Type | score | sub. m/z observed | sub. m/z calculated | sub ppm | | | met. m/z observed | met. m/z calculated | met. ppm |
|---|---|---|---|---|---|---|---|---|---|
| MATCH | 64.2 | 86.0971 | 86.0964 | -7.32 | 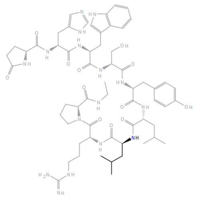 | 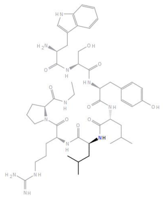 | 86.0972 | 86.0964 | -9.09 |
| MATCH | 32.4 | 112.0872 | 112.0869 | -2.51 | 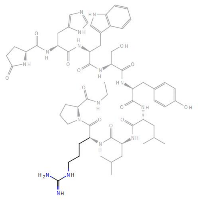 | 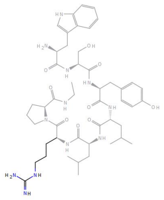 | 112.0875 | 112.0869 | -4.86 |
| MATCH | 14.0 | 115.0868 | 115.0866 | -2.20 | 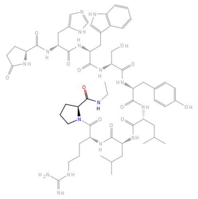 | 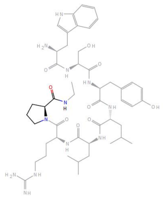 | 115.0871 | 115.0866 | -4.57 |
| MATCH | 83.8 | 130.0651 | 130.0575 | -58.4 | 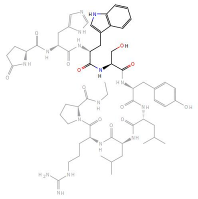 | 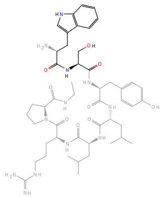 | 130.0654 | 130.0575 | -60.6 |
| MATCH | 65.5 | 136.0758 | 136.0757 | -0.68 | 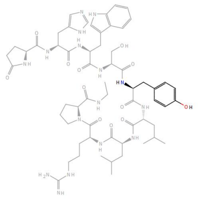 | 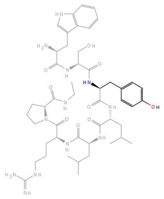 | 136.0759 | 136.0757 | -1.72 |
| MATCH | 77.2 | 143.1179 | 143.1179 | -0.39 | 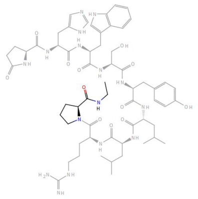 | 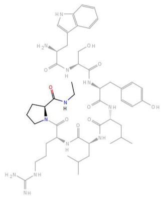 | 143.1182 | 143.1179 | -1.96 |
| MATCH | 11.8 | 157.1082 | 157.1084 | 1.03 | 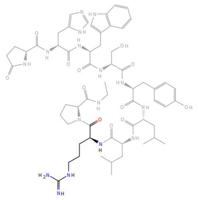 | 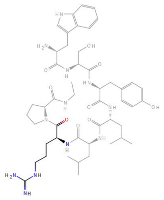 | 157.1086 | 157.1084 | -1.08 |
| MATCH | 43.1 | 170.0601 | 170.0600 | -0.63 | 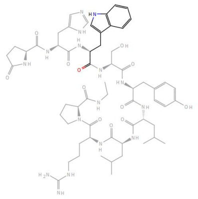 | 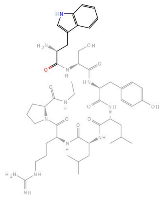 | 170.0602 | 170.0600 | -1.18 |
| MATCH | 8.1 | 199.1803 | 199.1805 | 1.17 | 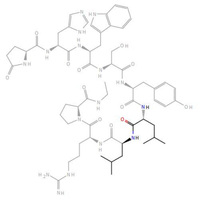 | 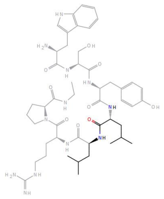 | 199.1808 | 199.1805 | -1.63 |

Metabolite: M3 -248 RT=2.48

| Type | score | sub. m/z observed | sub. m/z calculated | sub ppm | | | met. m/z observed | met. m/z calculated | met. ppm |
|---|---|---|---|---|---|---|---|---|---|
| MATCH | 14.1 | 249.1594 | 249.1598 | 1.48 | 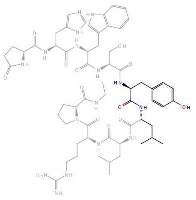 | 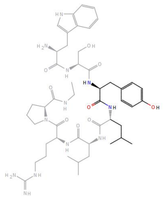 | 249.1600 | 249.1598 | -1.01 |
| MATCH | 29.5 | 253.1657 | 253.1659 | 0.90 | 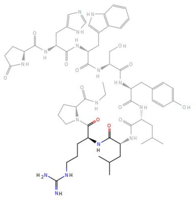 | 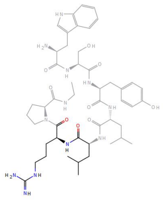 | 253.1661 | 253.1659 | -0.84 |
| MATCH | 8.2 | 270.1921 | 270.1925 | 1.30 | 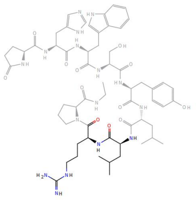 | 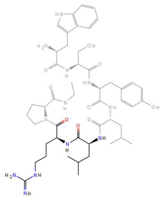 | 270.1937 | 270.1925 | -4.47 |
| MATCH | 9.5 | 274.1184 | 274.1186 | 0.84 | 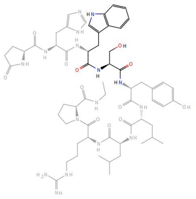 | 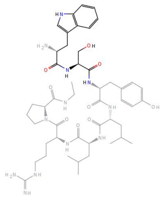 | 274.1186 | 274.1186 | 0.07 |
| | | | | | | 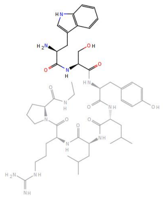 | 274.1186 | 274.1186 | 0.07 |
| MATCH | 7.0 | 277.1543 | 277.1547 | 1.25 | 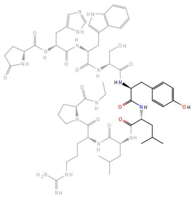 | 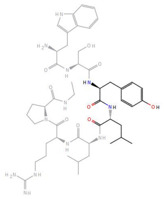 | 277.1555 | 277.1547 | -3.09 |
| MATCH | 7.0 | 277.1543 | 277.1547 | 1.25 | 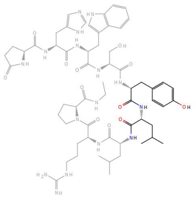 | 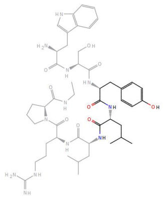 | 277.1555 | 277.1547 | -3.09 |
| MATCH | 10.2 | 282.1935 | 282.1925 | -3.54 | 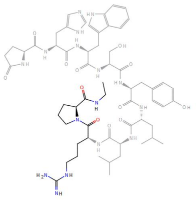 | 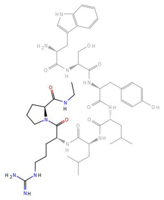 | 282.1928 | 282.1925 | -1.07 |
| MATCH | 45.4 | 299.2189 | 299.2190 | 0.45 | 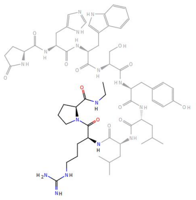 | 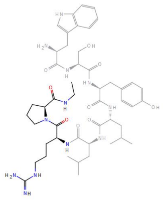 | 299.2195 | 299.2190 | -1.80 |

Metabolite: M3 -248 RT=2.48

| Type | score | sub. m/z observed | sub. m/z calculated | sub ppm | | | met. m/z observed | met. m/z calculated | met. ppm |
|---|---|---|---|---|---|---|---|---|---|
| MATCH | 6.0 | 364.1880 | 364.1867 | -3.62 | 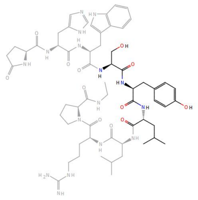 | 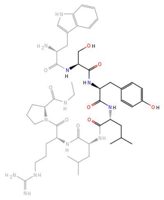 | 364.1850 | 364.1867 | 4.59 |
| MATCH | 6.0 | 364.1880 | 364.1867 | -3.62 | 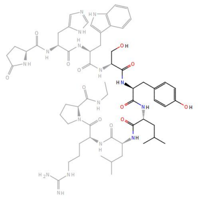 | 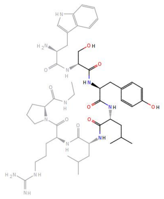 | 364.1850 | 364.1867 | 4.59 |
| MATCH | 7.5 | 383.2754 | 383.2765 | 2.83 | 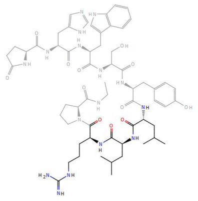 | 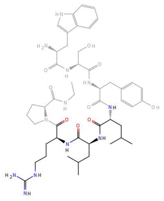 | 383.2752 | 383.2765 | 3.42 |
| MATCH | 39.7 | 412.3026 | 412.3031 | 1.04 | 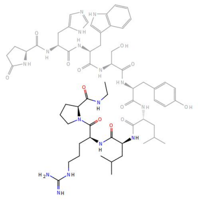 | 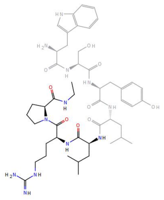 | 412.3033 | 412.3031 | -0.50 |
| MATCH | 17.4 | 435.1772 | 435.1775 | 0.77 | 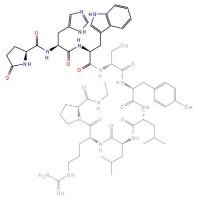 | 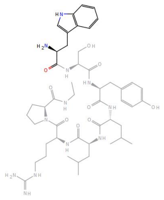 | 187.0866 | 187.0866 | -0.12 |
| MATCH | 15.7 | 504.1985 | 504.1990 | 0.92 | 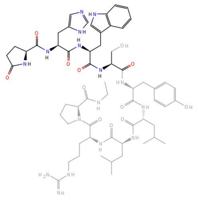 | 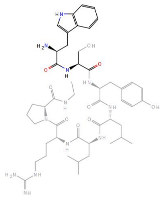 | 256.1085 | 256.1081 | -1.93 |
| MATCH | 18.5 | 522.2090 | 522.2096 | 1.05 | 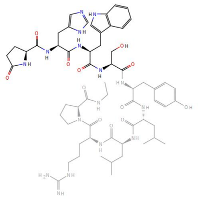 | 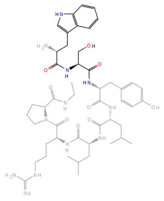 | 274.1186 | 274.1186 | 0.07 |
| | | | | | | 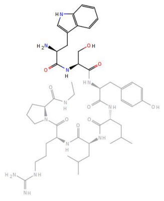 | 274.1186 | 274.1186 | 0.07 |
| MATCH | 27.9 | 525.3871 | 525.3871 | 0.07 | 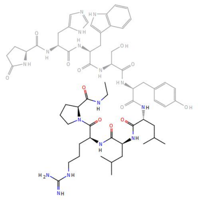 | 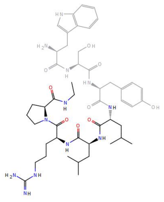 | 525.3869 | 525.3871 | 0.39 |

Metabolite: M3 -248 RT=2.48

| Type | score | sub. m/z observed | sub. m/z calculated | sub ppm | | | met. m/z observed | met. m/z calculated | met. ppm |
|---|---|---|---|---|---|---|---|---|---|
| MATCH | 3.6 | 633.3670 | 633.3719 | 7.70 | 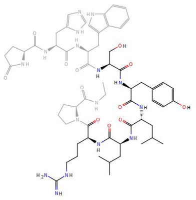 | 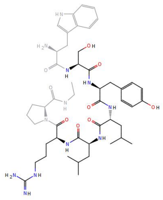 | 633.3698 | 633.3719 | 3.28 |
| MATCH | 15.6 | 667.2608 | 667.2623 | 2.28 | 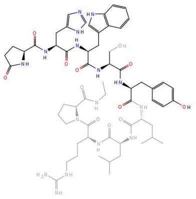 | 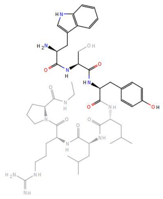 | 419.1710 | 419.1714 | 0.96 |
| MATCH | 30.9 | 688.4495 | 688.4505 | 1.44 | 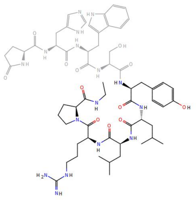 | 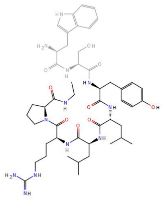 | 688.4522 | 688.4505 | -2.51 |
| MATCH | 37.0 | 775.4812 | 775.4825 | 1.69 | 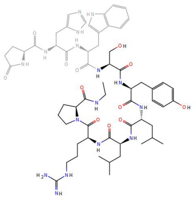 | 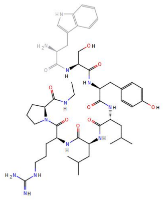 | 775.4822 | 775.4825 | 0.32 |
| MET_MATCH | | | | | | 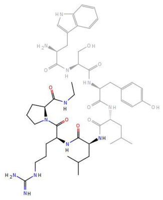 | 412.3028 | 412.3031 | 0.61 |
| MET_MATCH | | | | | | 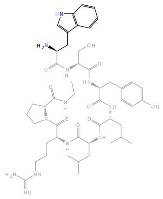 | 159.0919 | 159.0917 | -1.26 |
| MET_MATCH | | | | | | 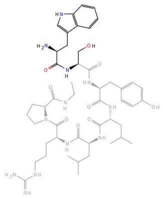 | 246.1238 | 246.1237 | -0.24 |

MS (+) FT

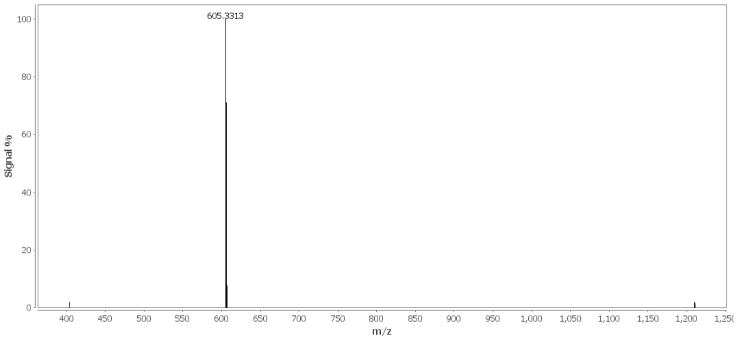


MS (+) FT

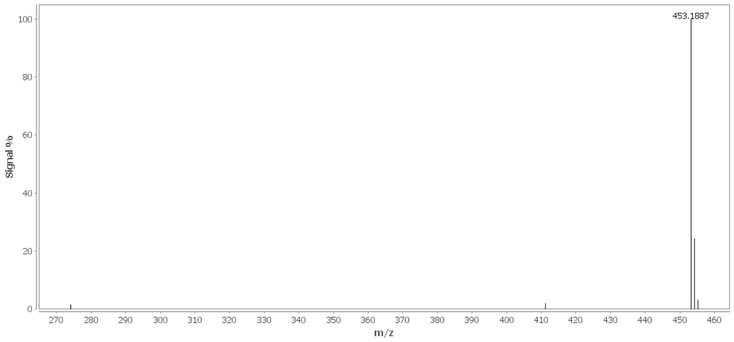

MS2 (+) FT activ = HCD:ce =

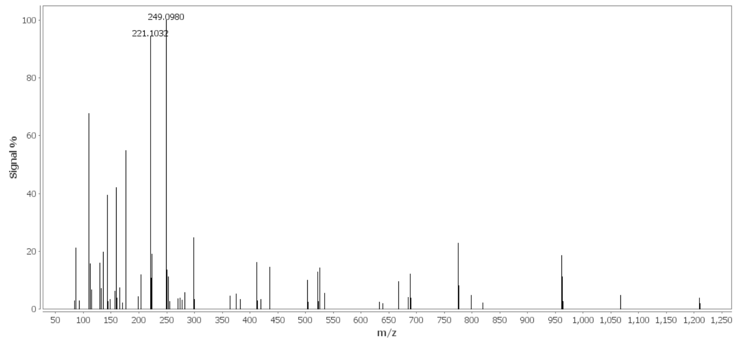


MS2 (+) FT activ = HCD:ce =

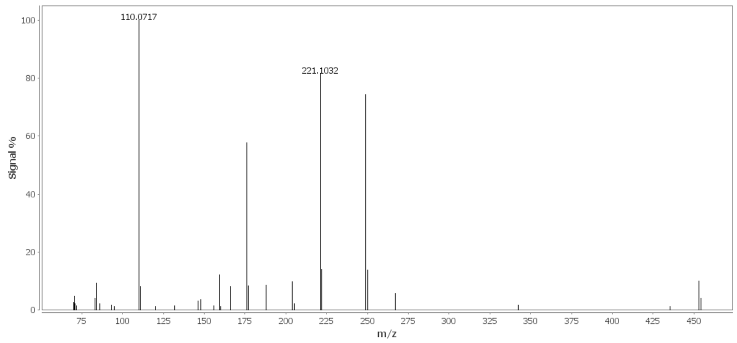


Metabolite: M1 -756 RT=0.52

| Type | score | sub. m/z observed | sub. m/z calculated | sub ppm | | | met. m/z observed | met. m/z calculated | met. ppm |
|---|---|---|---|---|---|---|---|---|---|
| MATCH | 102.0 | 403.8897 | 403.8891 | -1.45 | 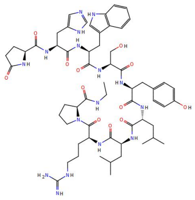 | 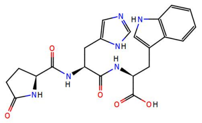 | 453.1887 | 453.1881 | -1.24 |
| | | | | | | 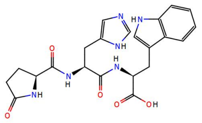 | 453.1887 | 453.1881 | -1.24 |
| | | | | | | 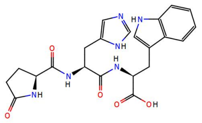 | 453.1887 | 453.1881 | -1.24 |
| MATCH | 200.0 | 605.3313 | 605.3300 | -2.14 | 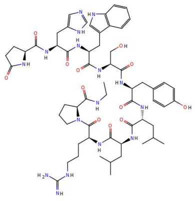 | 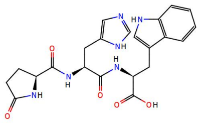 | 453.1887 | 453.1881 | -1.24 |
| | | | | | | 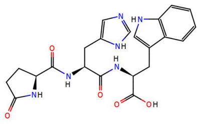 | 453.1887 | 453.1881 | -1.24 |
| | | | | | | 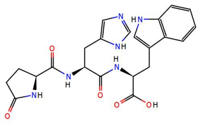 | 453.1887 | 453.1881 | -1.24 |
| MATCH | 102.0 | 1209.6540 | 1209.6527 | -1.07 | 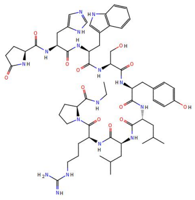 | 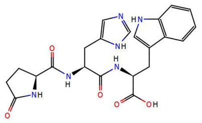 | 453.1887 | 453.1881 | -1.24 |

Metabolite: M1 -756 RT=0.52

| Type | score | sub. m/z observed | sub. m/z calculated | sub ppm | | | met. m/z observed | met. m/z calculated | met. ppm |
|---|---|---|---|---|---|---|---|---|---|
| | | | | | | 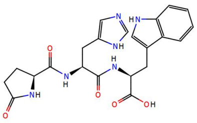 | 453.1887 | 453.1881 | -1.24 |
| | | | | | | 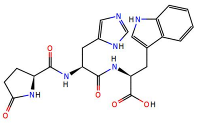 | 453.1887 | 453.1881 | -1.24 |
| MATCH | 12.2 | 84.0453 | 84.0444 | -11.3 | 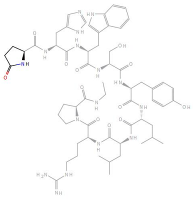 | 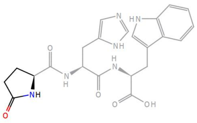 | 84.0451 | 84.0444 | -8.47 |
| MATCH | 4.6 | 93.0453 | 93.0447 | -6.45 | 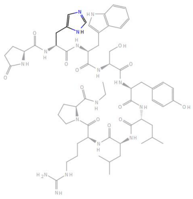 | 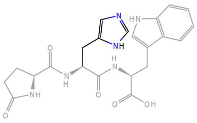 | 93.0452 | 93.0447 | -5.28 |
| MATCH | 167.7 | 110.0716 | 110.0713 | -3.25 | 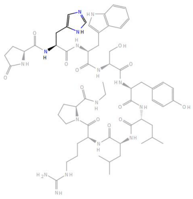 | 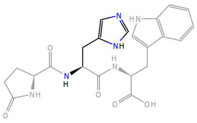 | 110.0717 | 110.0713 | -3.50 |
| MATCH | 54.1 | 159.0917 | 159.0917 | -0.08 | 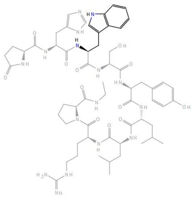 | 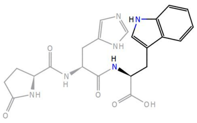 | 159.0916 | 159.0917 | 0.21 |
| MATCH | 15.6 | 166.0609 | 166.0611 | 0.96 | 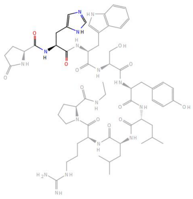 | 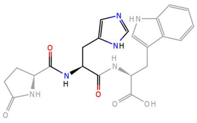 | 166.0611 | 166.0611 | 0.01 |
| MATCH | 175.7 | 221.1032 | 221.1033 | 0.47 | 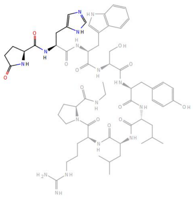 | 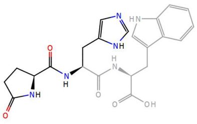 | 221.1032 | 221.1033 | 0.38 |
| MATCH | 174.2 | 249.0980 | 249.0982 | 0.71 | 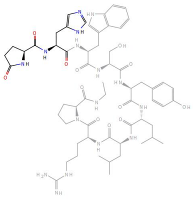 | 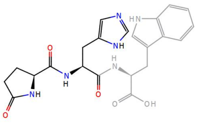 | 249.0981 | 249.0982 | 0.45 |

Metabolite: M1 -756 RT=0.52

| Type | score | sub. m/z observed | sub. m/z calculated | sub ppm | | met. m/z observed | met. m/z calculated | met. ppm |
|---|---|---|---|---|---|---|---|---|
| MATCH | 13.9 | 1209.6530 | 1209.6527 | -0.24 | 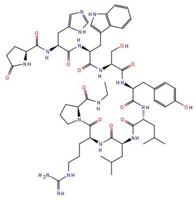 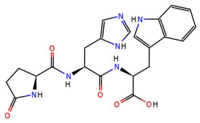 | 453.1886 | 453.1881 | -1.05 |
| MISMATCH | -23.4 | 86.0971 | 86.0964 | -7.32 | 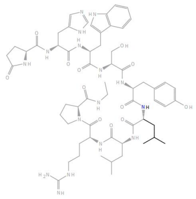 | 86.0970 | 86.0970 | 0.00 |
| MET_MATCH | | | | | 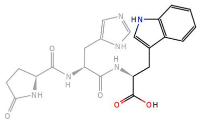 | 188.0705 | 188.0706 | 0.80 |
| MET_MATCH | | | | | 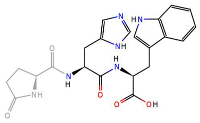 | 342.1554 | 342.1561 | 1.92 |
| MET_MATCH | | | | | 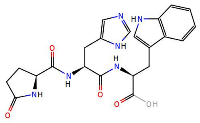 | 435.1763 | 435.1775 | 2.77 |
