# Supplementary material for: Software-aided approach to investigate peptide structure and metabolic susceptibility of amide bonds in peptide drugs based on high resolution mass spectrometry
Source: PLoS One. 2017 Nov 1;12(11):e0186461. doi: 10.1371/journal.pone.0186461 (PMC5665424; doi:10.1371/journal.pone.0186461)
Supplement: S1 File — (ZIP) [file pone.0186461.s007.zip › SFiles/S25_File.pdf]

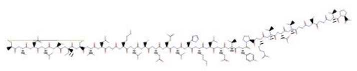

Calcitonin

Property name	Property value
Time	0min, 5min, 15min, 45min, 120min
Instrument	ThermoQAPlus
Matrix	chymotrypsin
Acquisition Mode	ddMS2

Chromatograms

Time=0min

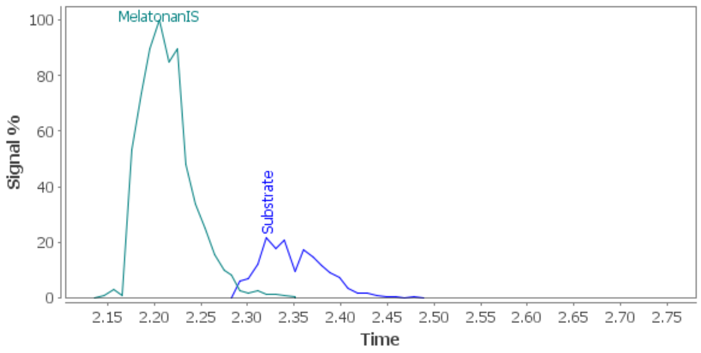

Time=5min

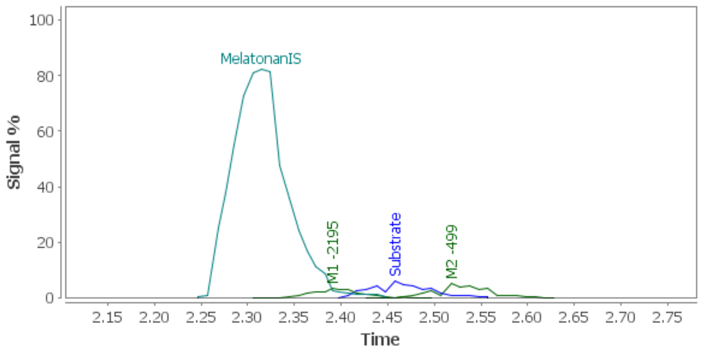

Time=15min

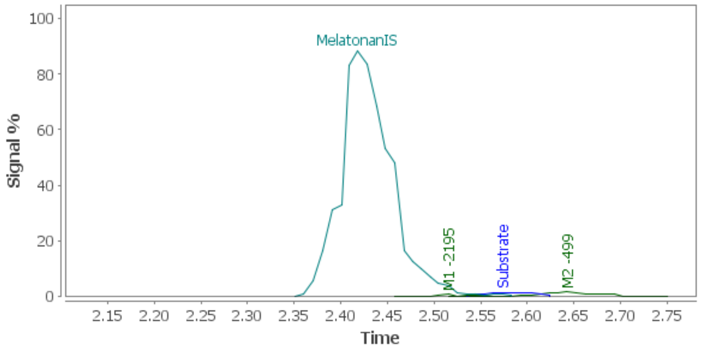

Time=45min

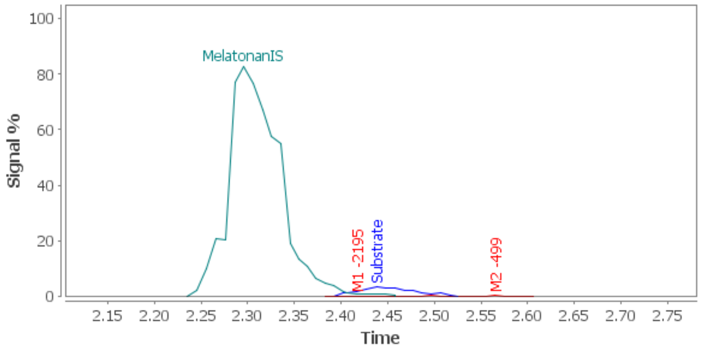

Time=120min

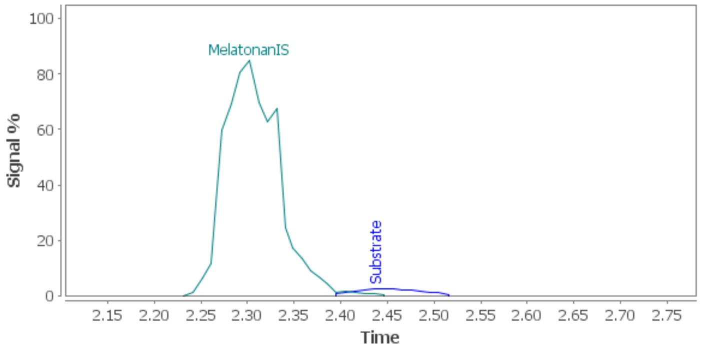

Custom Charts

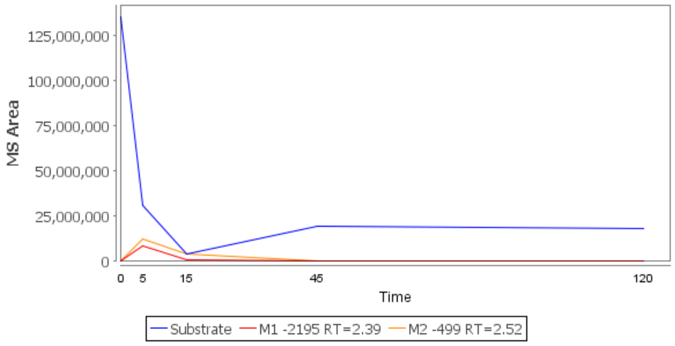

Fragmentation

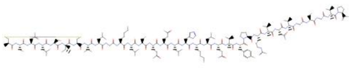

Calcitonin

MS (+) FT

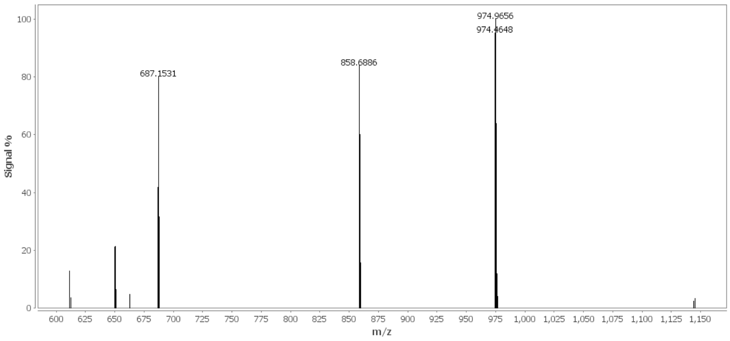

MS (+) FT

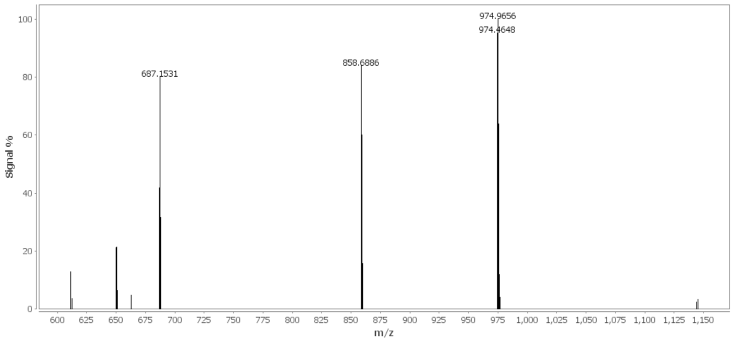

MS2 (+) FT activ = HCD:ce =

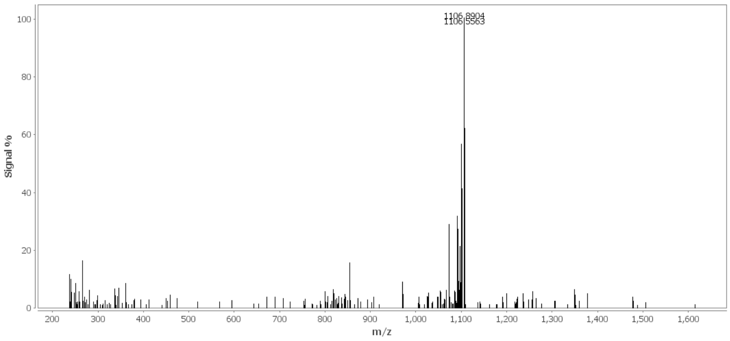

MS2 (+) FT activ = HCD:ce =

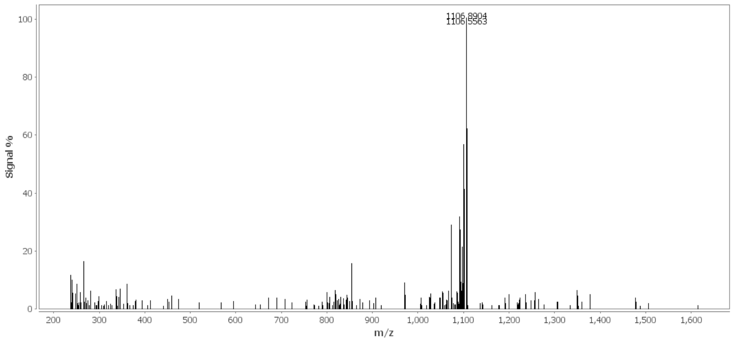

Metabolite: Substrate

Type	score	sub. m/z observed	sub. m/z calculated	sub ppm		met. m/z observed	met. m/z calculated	met. ppm
MISMATCH	-103.2	1223.1190	1223.1169	-1.74	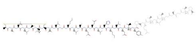 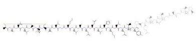	1223.1190	1223.1169	-1.74
MATCH	102.5	1144.2528	1144.2450	-6.79	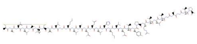 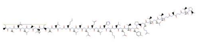	1144.2528	1144.2450	-6.79
MATCH	145.5	858.4366	858.4356	-1.16	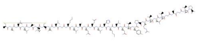 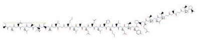	858.4366	858.4356	-1.16

Metabolite: Substrate

Type	score	sub. m/z observed	sub. m/z calculated	sub ppm		met. m/z observed	met. m/z calculated	met. ppm
MATCH	25.4	842.9307	842.9355	5.75	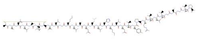 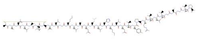	842.9307	842.9355	5.75
MATCH	25.4	842.9307	842.9236	-8.38	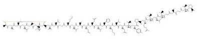 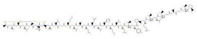	842.9307	842.9236	-8.38
MATCH	25.4	842.9307	842.9355	5.75	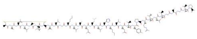 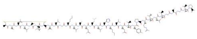	842.9307	842.9355	5.75
MATCH	25.4	842.9307	842.9355	5.75	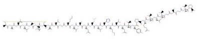 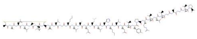	842.9307	842.9355	5.75
MATCH	25.4	842.9307	842.9236	-8.38	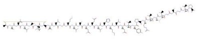 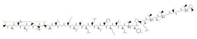	842.9307	842.9236	-8.38
MATCH	25.4	842.9307	842.9236	-8.38	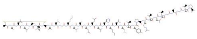 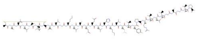	842.9307	842.9236	-8.38
MATCH	25.4	842.9307	842.9236	-8.38	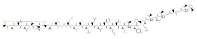 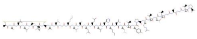	842.9307	842.9236	-8.38
MATCH	25.4	842.9307	842.9236	-8.38	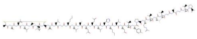 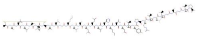	842.9307	842.9236	-8.38
MATCH	25.4	842.9307	842.9236	-8.38	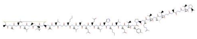 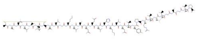	842.9307	842.9236	-8.38

Metabolite: Substrate

Type	score	sub. m/z observed	sub. m/z calculated	sub ppm			met. m/z observed	met. m/z calculated	met. ppm
MATCH	25.4	842.9307	842.9355	5.75	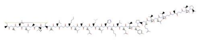	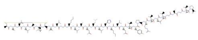	842.9307	842.9355	5.75
MATCH	25.4	842.9307	842.9236	-8.38	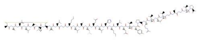	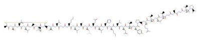	842.9307	842.9236	-8.38
MATCH	25.4	842.9307	842.9236	-8.38	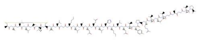	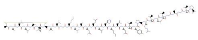	842.9307	842.9236	-8.38
MATCH	25.4	842.9307	842.9236	-8.38	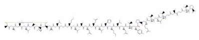	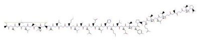	842.9307	842.9236	-8.38
MATCH	25.4	842.9307	842.9236	-8.38	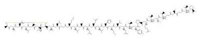	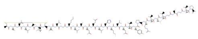	842.9307	842.9236	-8.38
MATCH	25.4	842.9307	842.9236	-8.38	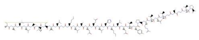	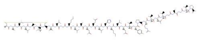	842.9307	842.9236	-8.38
MATCH	25.4	842.9307	842.9236	-8.38	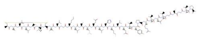	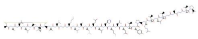	842.9307	842.9236	-8.38
MATCH	25.4	842.9307	842.9236	-8.38	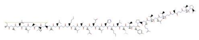	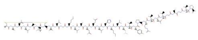	842.9307	842.9236	-8.38
MATCH	25.4	842.9307	842.9236	-8.38	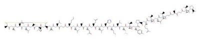	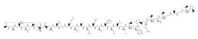	842.9307	842.9236	-8.38

Metabolite: Substrate

Type	score	sub. m/z observed	sub. m/z calculated	sub ppm			met. m/z observed	met. m/z calculated	met. ppm
MATCH	25.4	842.9307	842.9236	-8.38	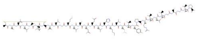	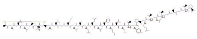	842.9307	842.9236	-8.38
MATCH	25.4	842.9307	842.9236	-8.38	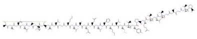	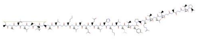	842.9307	842.9236	-8.38
MATCH	25.4	842.9307	842.9236	-8.38	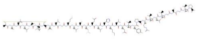	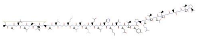	842.9307	842.9236	-8.38
MATCH	25.4	842.9307	842.9355	5.75	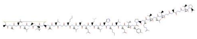	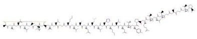	842.9307	842.9355	5.75
MATCH	25.4	842.9307	842.9236	-8.38	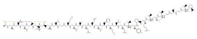	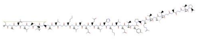	842.9307	842.9236	-8.38
MATCH	25.4	842.9307	842.9236	-8.38	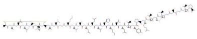	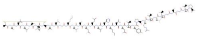	842.9307	842.9236	-8.38
MATCH	25.4	842.9307	842.9236	-8.38	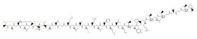	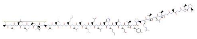	842.9307	842.9236	-8.38
MATCH	25.4	842.9307	842.9355	5.75	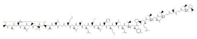	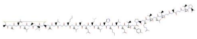	842.9307	842.9355	5.75
MATCH	25.4	842.9307	842.9236	-8.38	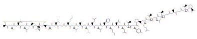	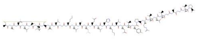	842.9307	842.9236	-8.38

Metabolite: Substrate

Type	score	sub. m/z observed	sub. m/z calculated	sub ppm			met. m/z observed	met. m/z calculated	met. ppm
MATCH	25.4	842.9307	842.9236	-8.38	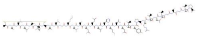	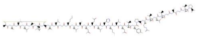	842.9307	842.9236	-8.38
MATCH	25.4	842.9307	842.9236	-8.38	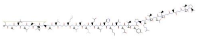	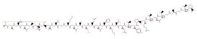	842.9307	842.9236	-8.38
MATCH	25.4	842.9307	842.9236	-8.38	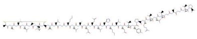	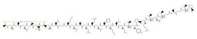	842.9307	842.9236	-8.38
MATCH	100.0	686.9569	686.9499	-10.0	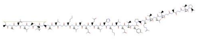	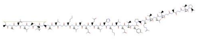	686.9569	686.9499	-10.0
MATCH	44.5	345.1405	345.1445	11.57	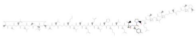	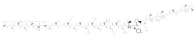	345.1405	345.1445	11.57
MISMATCH	-28.0	337.0651	337.0635	-4.92	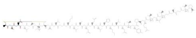	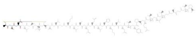	337.0651	337.0635	-4.92
MISMATCH	-22.4	325.1886	325.1870	-4.74	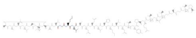	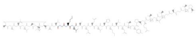	325.1886	325.1870	-4.74
MISMATCH	-22.4	325.1886	325.1870	-4.74	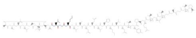	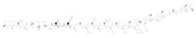	325.1886	325.1870	-4.74
MISMATCH	-22.4	325.1886	325.1870	-4.74	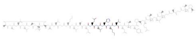	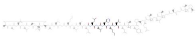	325.1886	325.1870	-4.74

Metabolite: Substrate

Type	score	sub. m/z observed	sub. m/z calculated	sub ppm			met. m/z observed	met. m/z calculated	met. ppm
MISMATCH	-30.8	299.2092	299.2078	-4.73	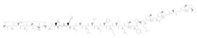	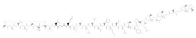	299.2092	299.2078	-4.73
MISMATCH	-30.8	299.2092	299.2078	-4.73	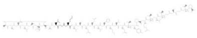	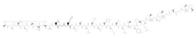	299.2092	299.2078	-4.73
MISMATCH	-30.8	299.2092	299.2078	-4.73	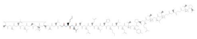	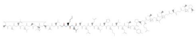	299.2092	299.2078	-4.73
MISMATCH	-30.8	299.2092	299.2078	-4.73	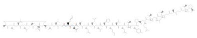	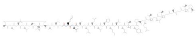	299.2092	299.2078	-4.73
MISMATCH	-70.1	266.1619	266.1661	15.90	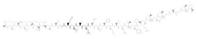	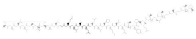	266.1619	266.1661	15.90
MISMATCH	-70.1	266.1619	266.1612	-2.69	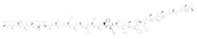	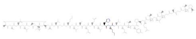	266.1619	266.1612	-2.69
MISMATCH	-70.1	266.1619	266.1612	-2.69	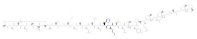	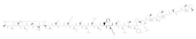	266.1619	266.1612	-2.69
MISMATCH	-28.4	258.1076	258.1084	3.44	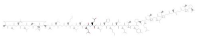	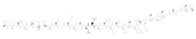	258.1076	258.1084	3.44
MISMATCH	-28.4	258.1076	258.1084	3.44	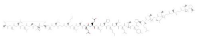	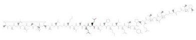	258.1076	258.1084	3.44

Metabolite: Substrate

Type	score	sub. m/z observed	sub. m/z calculated	sub ppm		met. m/z observed	met. m/z calculated	met. ppm
MISMATCH	-35.1	251.1510	251.1503	-2.99	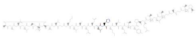 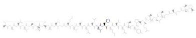	251.1510	251.1503	-2.99
MISMATCH	-35.1	251.1510	251.1503	-2.99	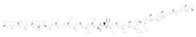 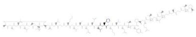	251.1510	251.1503	-2.99
MISMATCH	-25.1	248.1515	248.1499	-6.35	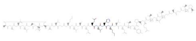 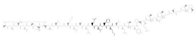	248.1515	248.1499	-6.35
MISMATCH	-46.2	242.1505	242.1499	-2.48	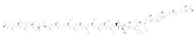 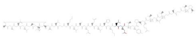	242.1505	242.1499	-2.48
MISMATCH	-46.2	242.1505	242.1499	-2.48	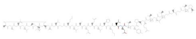 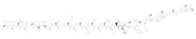	242.1505	242.1499	-2.48
MISMATCH	-24.1	238.1188	238.1186	-0.68	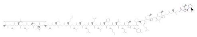 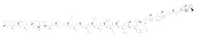	238.1188	238.1186	-0.68
MISMATCH	-59.7	237.1366	237.1463	40.63	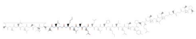 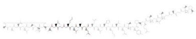	237.1366	237.1463	40.63

MS (+) FT

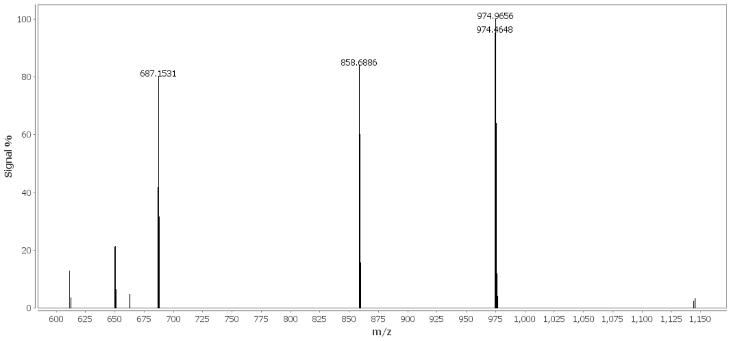

MS (+) FT

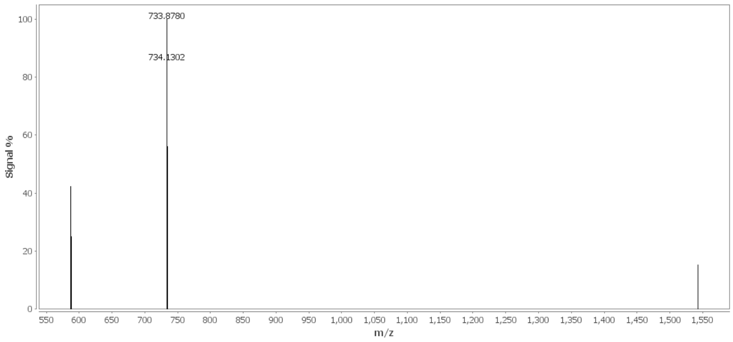

MS2 (+) FT activ = HCD:ce =

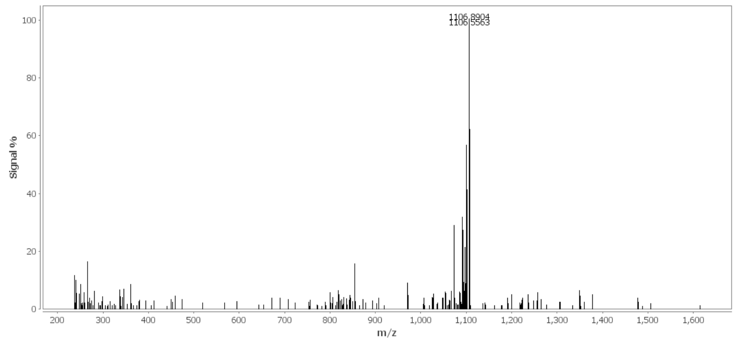

MS2 (+) FT activ = HCD:ce =

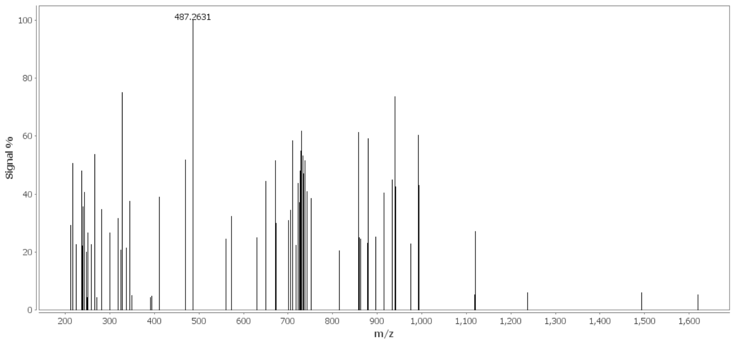

Metabolite: M2 -499 RT=2.52

Type	score	sub. m/z observed	sub. m/z calculated	sub ppm		met. m/z observed	met. m/z calculated	met. ppm
MATCH	72.6	686.9569	686.9499	-10.0	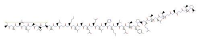 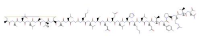	587.1038	587.1021	-2.87
MATCH	72.6	686.9569	686.9499	-10.0	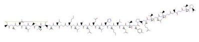 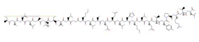	587.1038	587.1021	-2.87
					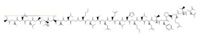	587.1038	587.1021	-2.87
MATCH	100.0	686.9569	686.9499	-10.0	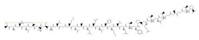 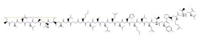	733.6277	733.6258	-2.48
MATCH	100.0	686.9569	686.9499	-10.0	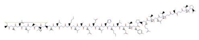 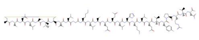	733.6277	733.6258	-2.48
					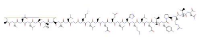	733.6277	733.6258	-2.48
MATCH	76.3	858.4366	858.4356	-1.16	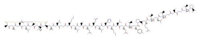 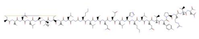	587.1038	587.1021	-2.87

Metabolite: M2 -499 RT=2.52

Type	score	sub. m/z observed	sub. m/z calculated	sub ppm		met. m/z observed	met. m/z calculated	met. ppm
MATCH	76.3	858.4366	858.4356	-1.16	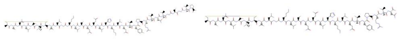	587.1038	587.1021	-2.87
					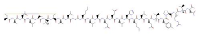	587.1038	587.1021	-2.87
MATCH	103.7	858.4366	858.4356	-1.16	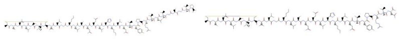	733.6277	733.6258	-2.48
MATCH	103.7	858.4366	858.4356	-1.16	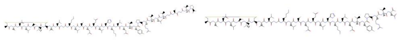	733.6277	733.6258	-2.48
					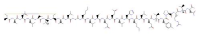	733.6277	733.6258	-2.48
MATCH	33.3	1144.2528	1144.2450	-6.79	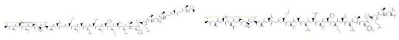	587.1038	587.1021	-2.87
MATCH	33.3	1144.2528	1144.2450	-6.79	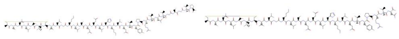	587.1038	587.1021	-2.87
					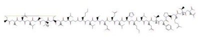	587.1038	587.1021	-2.87
MATCH	60.6	1144.2528	1144.2450	-6.79	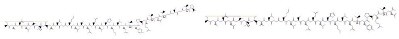	733.6277	733.6258	-2.48

Metabolite: M2 -499 RT=2.52

Type	score	sub. m/z observed	sub. m/z calculated	sub ppm		met. m/z observed	met. m/z calculated	met. ppm
MATCH	60.6	1144.2528	1144.2450	-6.79	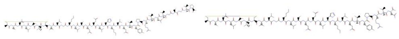	733.6277	733.6258	-2.48
					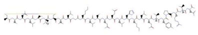	733.6277	733.6258	-2.48
MATCH	44.5	345.1405	345.1445	11.57	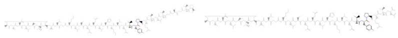	345.1406	345.1445	11.31
MATCH	25.4	842.9307	842.9236	-8.38	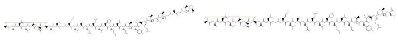	718.1176	718.1138	-5.18
MATCH	25.4	842.9307	842.9236	-8.38	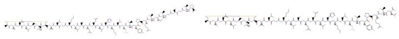	718.1176	718.1138	-5.18
MATCH	25.4	842.9307	842.9236	-8.38	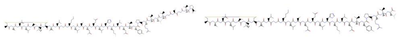	718.1176	718.1138	-5.18
MATCH	25.4	842.9307	842.9236	-8.38	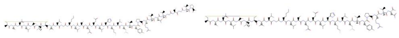	718.1176	718.1138	-5.18
MATCH	25.4	842.9307	842.9236	-8.38	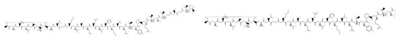	718.1176	718.1138	-5.18
MATCH	25.4	842.9307	842.9236	-8.38	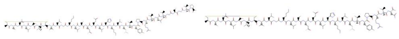	718.1176	718.1138	-5.18

Metabolite: M2 -499 RT=2.52

Type	score	sub. m/z observed	sub. m/z calculated	sub ppm		met. m/z observed	met. m/z calculated	met. ppm
MATCH	25.4	842.9307	842.9236	-8.38	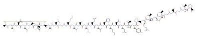 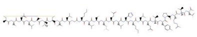	718.1176	718.1138	-5.18
MATCH	25.4	842.9307	842.9236	-8.38	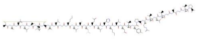 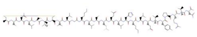	718.1176	718.1138	-5.18
MATCH	25.4	842.9307	842.9236	-8.38	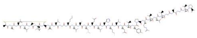 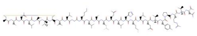	718.1176	718.1138	-5.18
MATCH	25.4	842.9307	842.9236	-8.38	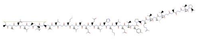 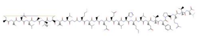	718.1176	718.1138	-5.18
MATCH	25.4	842.9307	842.9236	-8.38	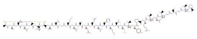 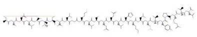	718.1176	718.1138	-5.18
MATCH	25.4	842.9307	842.9236	-8.38	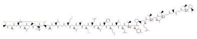 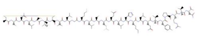	718.1176	718.1138	-5.18
MATCH	25.4	842.9307	842.9236	-8.38	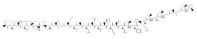 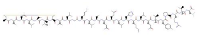	718.1176	718.1138	-5.18
MATCH	25.4	842.9307	842.9236	-8.38	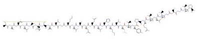 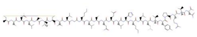	718.1176	718.1138	-5.18
MATCH	25.4	842.9307	842.9236	-8.38	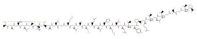 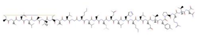	718.1176	718.1138	-5.18

Metabolite: M2 -499 RT=2.52

Type	score	sub. m/z observed	sub. m/z calculated	sub ppm		met. m/z observed	met. m/z calculated	met. ppm
MATCH	25.4	842.9307	842.9236	-8.38	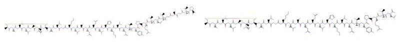	718.1176	718.1138	-5.18
MATCH	25.4	842.9307	842.9236	-8.38	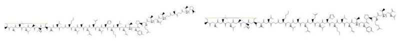	718.1176	718.1138	-5.18
MATCH	25.4	842.9307	842.9236	-8.38	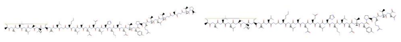	718.1176	718.1138	-5.18
MATCH	25.4	842.9307	842.9236	-8.38	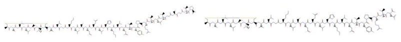	718.1176	718.1138	-5.18
MATCH	25.4	842.9307	842.9236	-8.38	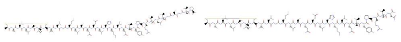	718.1176	718.1138	-5.18
MATCH	25.4	842.9307	842.9236	-8.38	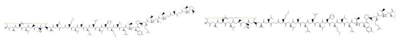	718.1176	718.1138	-5.18
MATCH	25.4	842.9307	842.9236	-8.38	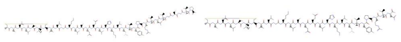	718.1176	718.1138	-5.18
MATCH	25.4	842.9307	842.9236	-8.38	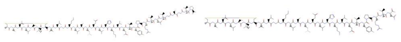	718.1176	718.1138	-5.18
MATCH	25.4	842.9307	842.9236	-8.38	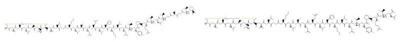	718.1176	718.1138	-5.18

Metabolite: M2 -499 RT=2.52

Type	score	sub. m/z observed	sub. m/z calculated	sub ppm		met. m/z observed	met. m/z calculated	met. ppm
MATCH	25.4	842.9307	842.9355	5.75	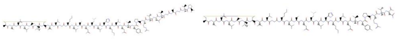	718.1176	718.1257	11.40
MATCH	25.4	842.9307	842.9355	5.75	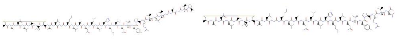	718.1176	718.1257	11.40
MATCH	25.4	842.9307	842.9355	5.75	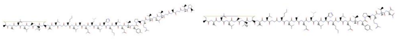	718.1176	718.1257	11.40
MATCH	25.4	842.9307	842.9355	5.75	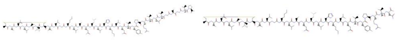	718.1176	718.1257	11.40
MATCH	25.4	842.9307	842.9355	5.75	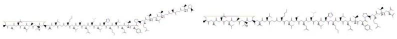	718.1176	718.1257	11.40
MATCH	25.4	842.9307	842.9355	5.75	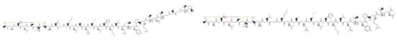	718.1176	718.1257	11.40
MISMATCH	-59.7	237.1366	237.1463	40.63	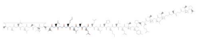	237.1366	237.1366	0.00
MISMATCH	-64.4	237.1366	237.1463	40.63	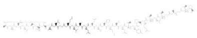	709.3975	709.3975	0.00
MISMATCH	-24.1	238.1188	238.1186	-0.68	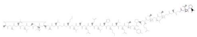	238.1186	238.1186	0.00

Metabolite: M2 -499 RT=2.52

Type	score	sub. m/z observed	sub. m/z calculated	sub ppm		met. m/z observed	met. m/z calculated	met. ppm
MISMATCH	-46.2	242.1505	242.1499	-2.48	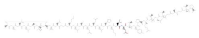	242.1507	242.1507	0.00
MISMATCH	-25.1	248.1515	248.1499	-6.35	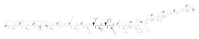	248.1518	248.1518	0.00
MISMATCH	-35.1	251.1510	251.1503	-2.99	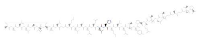	251.1504	251.1504	0.00
MISMATCH	-28.4	258.1076	258.1084	3.44	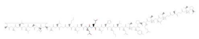	258.1094	258.1094	0.00
MISMATCH	-70.1	266.1619	266.1612	-2.69	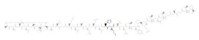	266.1615	266.1615	0.00
MISMATCH	-30.8	299.2092	299.2078	-4.73	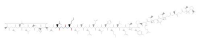	299.2089	299.2089	0.00
MISMATCH	-22.4	325.1886	325.1870	-4.74	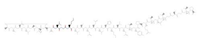	325.1872	325.1872	0.00
MISMATCH	-28.0	337.0651	337.0635	-4.92	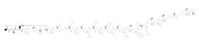	337.0642	337.0642	0.00
MISMATCH	-103.2	1223.1190	1223.1169	-1.74	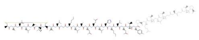	487.2631	487.2631	0.00

Metabolite: M2 -499 RT=2.52

Type	score	sub. m/z observed	sub. m/z calculated	sub ppm		met. m/z observed	met. m/z calculated	met. ppm
MET_MATCH					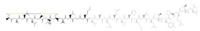	225.1234	225.1147	-38.7
MET_MATCH					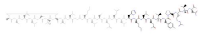	248.9290	248.9345	22.07
MET_MATCH					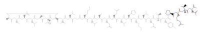	487.2631	487.2623	-1.56
MET_MATCH					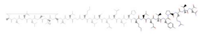	560.8116	560.8091	-4.53
MET_MATCH					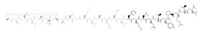	629.3385	629.3386	0.10
MET_MATCH					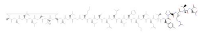	650.3283	650.3256	-4.09
MET_MATCH					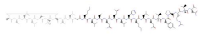	671.7037	671.7007	-4.40
MET_MATCH					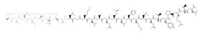	709.3975	709.3954	-2.98
MET_MATCH					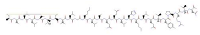	722.3760	722.3705	-7.62

Metabolite: M2 -499 RT=2.52

Type	score	sub. m/z observed	sub. m/z calculated	sub ppm		met. m/z observed	met. m/z calculated	met. ppm
MET_MATCH					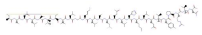	722.3760	722.3705	-7.62
MET_MATCH					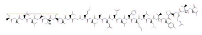	722.3760	722.3705	-7.62
MET_MATCH					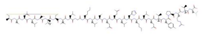	722.3760	722.3705	-7.62
MET_MATCH					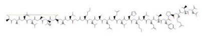	729.1286	729.1232	-7.41
MET_MATCH					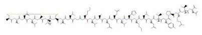	729.1286	729.1232	-7.41
MET_MATCH					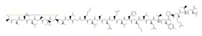	729.1286	729.1232	-7.41
MET_MATCH					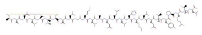	729.1286	729.1232	-7.41
MET_MATCH					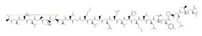	729.1286	729.1232	-7.41
MET_MATCH					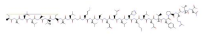	729.1286	729.1232	-7.41

Metabolite: M2 -499 RT=2.52

Type	score	sub. m/z observed	sub. m/z calculated	sub ppm		met. m/z observed	met. m/z calculated	met. ppm
MET_MATCH					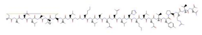	729.3732	729.3692	-5.52
MET_MATCH					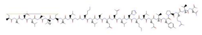	729.3732	729.3692	-5.52
MET_MATCH					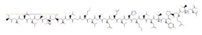	729.3732	729.3692	-5.52
MET_MATCH					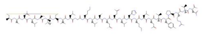	729.3732	729.3692	-5.52
MET_MATCH					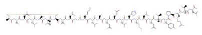	729.3732	729.3692	-5.52
MET_MATCH					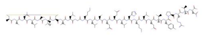	729.3732	729.3692	-5.52
MET_MATCH					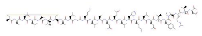	729.3732	729.3692	-5.52
MET_MATCH					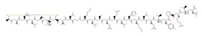	733.6262	733.6258	-0.52
MET_MATCH					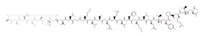	742.4161	742.4182	2.87

Metabolite: M2 -499 RT=2.52

Type	score	sub. m/z observed	sub. m/z calculated	sub ppm		met. m/z observed	met. m/z calculated	met. ppm
MET_MATCH					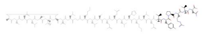	751.3772	751.3733	-5.15
MET_MATCH					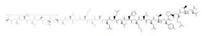	814.4321	814.4312	-1.17
MET_MATCH					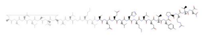	857.9498	857.9472	-3.00
MET_MATCH					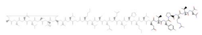	862.4084	862.4054	-3.58
MET_MATCH					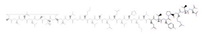	879.4329	879.4319	-1.09
MET_MATCH					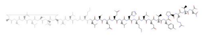	914.4905	914.4892	-1.44
MET_MATCH					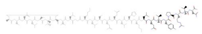	975.4888	975.4894	0.60
MET_MATCH					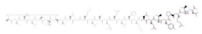	992.5104	992.5160	5.64
MET_MATCH					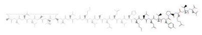	1120.6127	1120.6109	-1.55

MS (+) FT

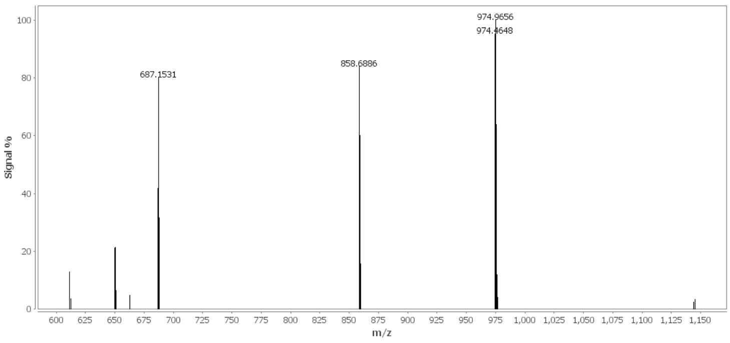

MS (+) FT

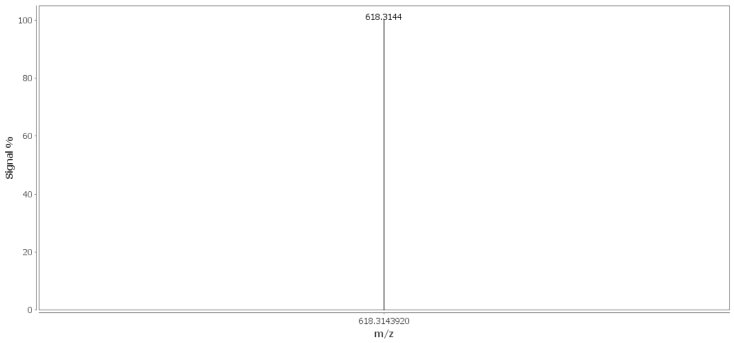

Metabolite: M1 -2195 RT=2.39

Type	score	sub. m/z observed	sub. m/z calculated	sub ppm		met. m/z observed	met. m/z calculated	met. ppm
MATCH	145.5	858.4366	858.4356	-1.16	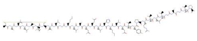 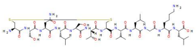	618.3144	618.3098	-7.48
					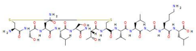	618.3144	618.3098	-7.48
MATCH	102.5	1144.2528	1144.2450	-6.79	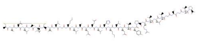 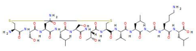	618.3144	618.3098	-7.48
					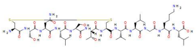	618.3144	618.3098	-7.48
