# Supplementary material for: Software-aided approach to investigate peptide structure and metabolic susceptibility of amide bonds in peptide drugs based on high resolution mass spectrometry
Source: PLoS One. 2017 Nov 1;12(11):e0186461. doi: 10.1371/journal.pone.0186461 (PMC5665424; doi:10.1371/journal.pone.0186461)

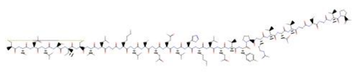

# Calcitonin

| Property name | Property value |
|---|---|
| Time | 0min, 5min, 15min, 45min, 120min |
| Instrument | ThermoQAPlus |
| Matrix | trypsin |
| Acquisition Mode | ddMS2 |

## Chromatograms

Time=0min

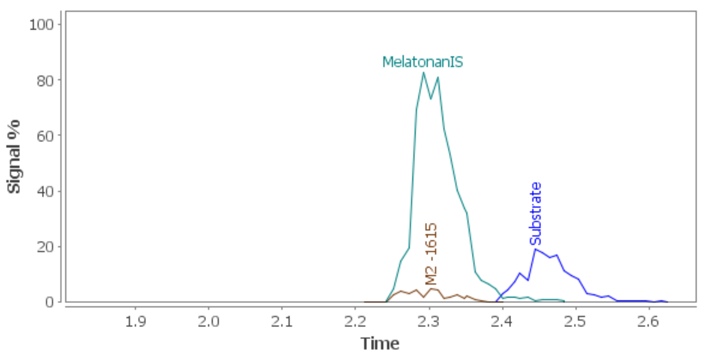


Time=5min

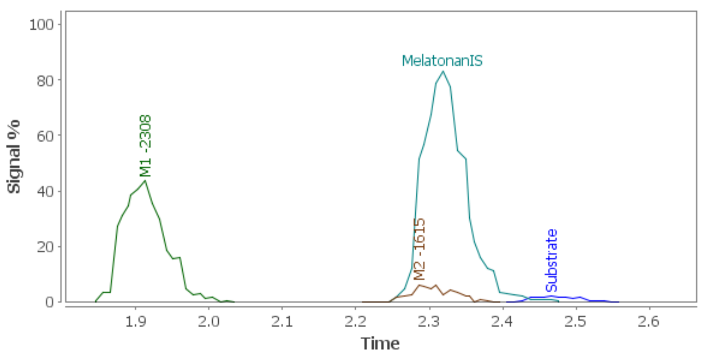


Time=15min

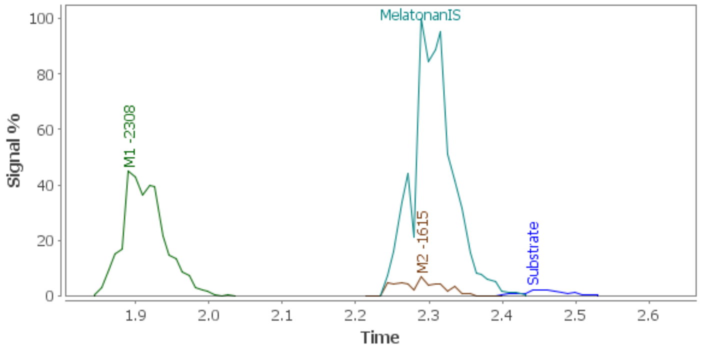


Time=45min

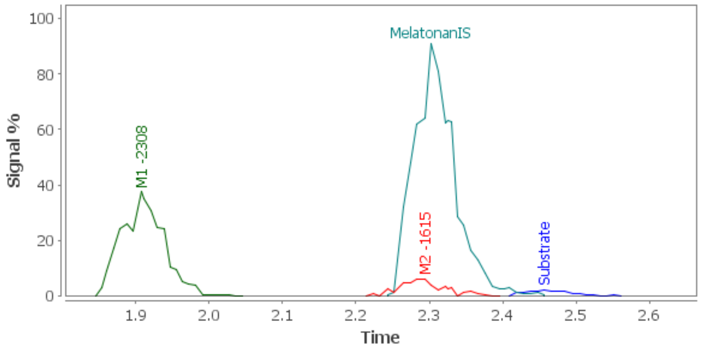


Time=120min

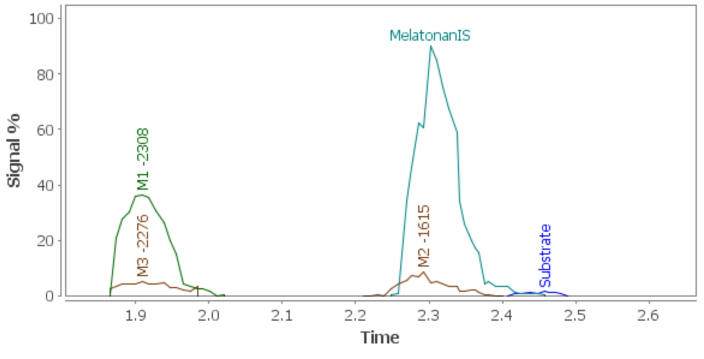

# Custom Charts

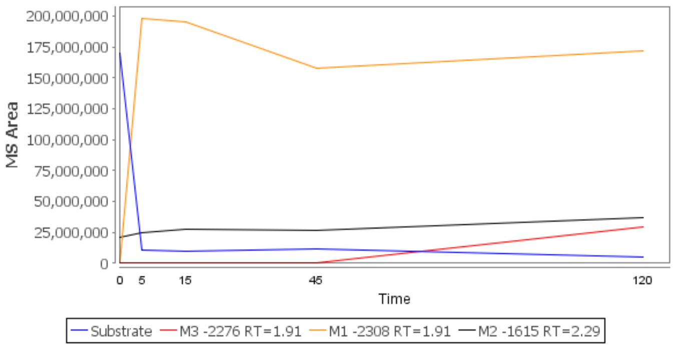

# Fragmentation

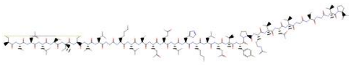

Calcitonin

MS (+) FT

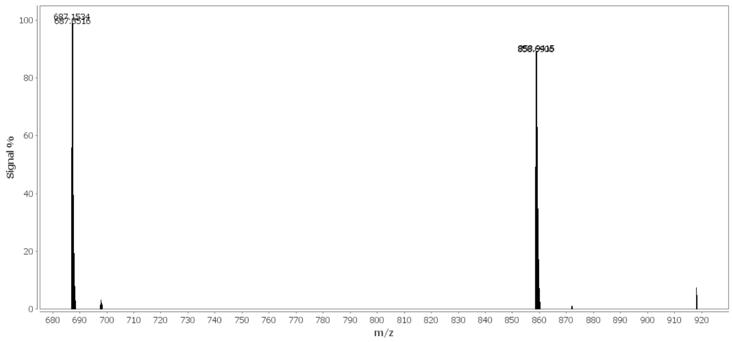


MS (+) FT

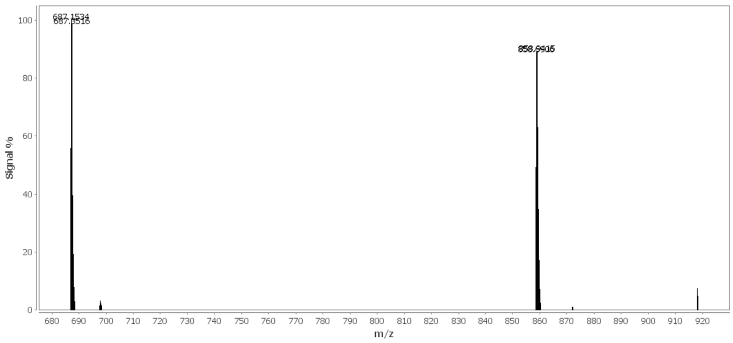


MS2 (+) FT activ = HCD:ce =

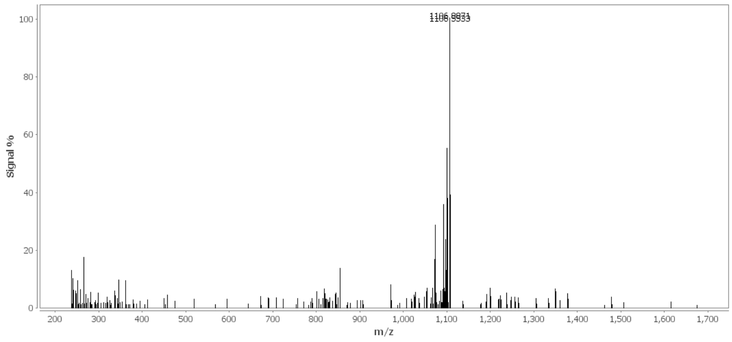


MS2 (+) FT activ = HCD:ce =

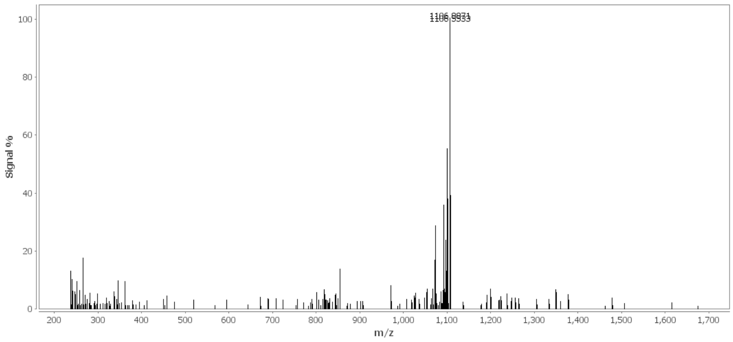


Metabolite: Substrate

| Type | score | sub. m/z observed | sub. m/z calculated | sub ppm | | met. m/z observed | met. m/z calculated | met. ppm |
|---|---|---|---|---|---|---|---|---|
| MATCH | 149.2 | 858.4385 | 858.4356 | -3.35 | | 858.4385 | 858.4356 | -3.35 |
| | | | | | 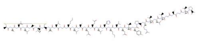 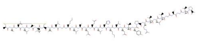 | | | |
| MATCH | 11.4 | 337.0629 | 337.0635 | 1.83 | | 337.0629 | 337.0635 | 1.83 |
| | | | | | 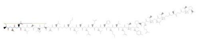 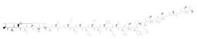 | | | |
| MISMATCH | -6.8 | 270.1448 | 270.1448 | 0.14 | | 270.1448 | 270.1448 | 0.14 |
| | | | | | 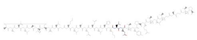 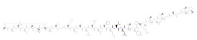 | | | |

Metabolite: Substrate

| Type | score | sub. m/z observed | sub. m/z calculated | sub ppm | | met. m/z observed | met. m/z calculated | met. ppm |
|---|---|---|---|---|---|---|---|---|
| MISMATCH | -6.8 | 270.1448 | 270.1448 | 0.14 | | 270.1448 | 270.1448 | 0.14 |
| | | | | | 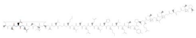 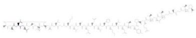 | | | |
| MISMATCH | -6.8 | 270.1448 | 270.1448 | 0.14 | | 270.1448 | 270.1448 | 0.14 |
| | | | | | 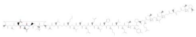 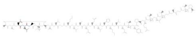 | | | |
| MISMATCH | -8.7 | 258.1076 | 258.1084 | 3.10 | | 258.1076 | 258.1084 | 3.10 |
| | | | | | 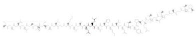 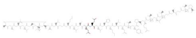 | | | |
| MISMATCH | -8.7 | 258.1076 | 258.1084 | 3.10 | | 258.1076 | 258.1084 | 3.10 |
| | | | | | 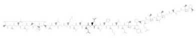 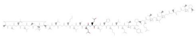 | | | |

MS (+) FT

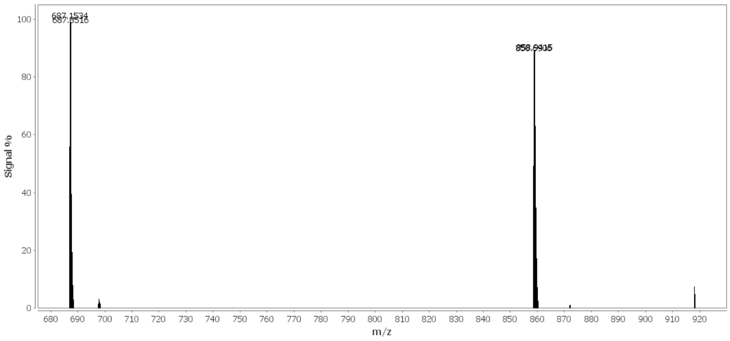


MS (+) FT

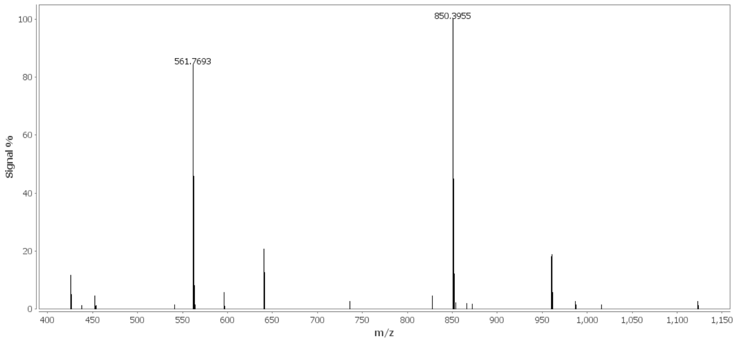


MS2 (+) FT activ = HCD:ce =

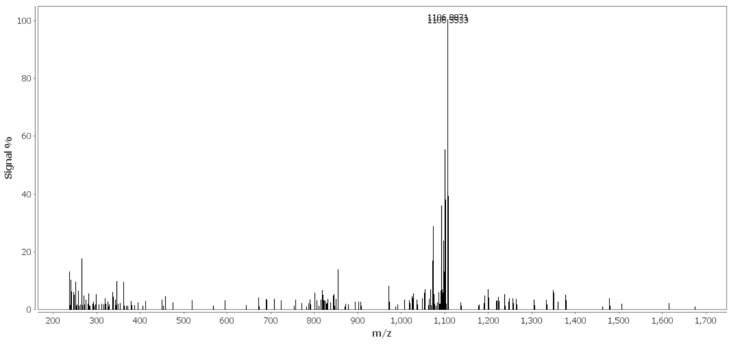


MS2 (+) FT activ = HCD:ce =

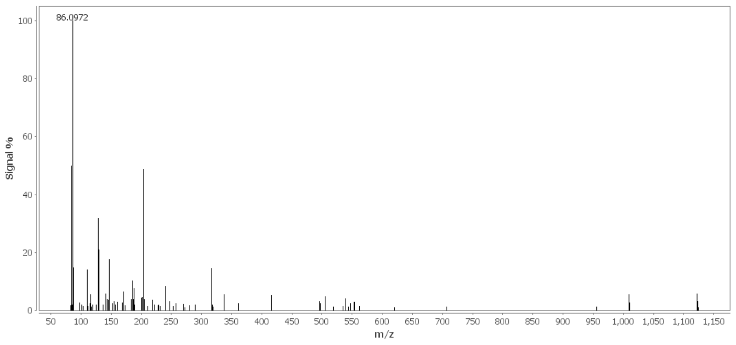


Metabolite: M1 -2308 RT=1.91

| Type | score | sub. m/z observed | sub. m/z calculated | sub ppm | | met. m/z observed | met. m/z calculated | met. ppm |
|---|---|---|---|---|---|---|---|---|

Metabolite: M1 -2308 RT=1.91

| Type | score | sub. m/z observed | sub. m/z calculated | sub ppm | | met. m/z observed | met. m/z calculated | met. ppm |
|---|---|---|---|---|---|---|---|---|
| MATCH | 133.4 | 858.4385 | 858.4356 | -3.35 | 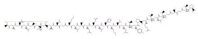 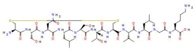 | 561.7693 | 561.7677 | -2.82 |
| MATCH | 51.8 | 858.4385 | 858.4356 | -3.35 | 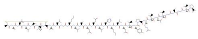 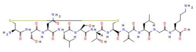 | 1122.5334 | 1122.5282 | -4.63 |
| MATCH | 11.4 | 337.0629 | 337.0635 | 1.83 | 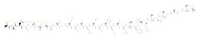 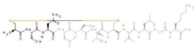 | 337.0634 | 337.0635 | 0.13 |
| MISMATCH | -8.7 | 258.1076 | 258.1084 | 3.10 | 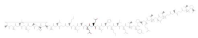 | 258.1094 | 258.1094 | 0.00 |
| MISMATCH | -6.8 | 270.1448 | 270.1448 | 0.14 | 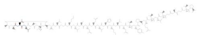 | 270.1459 | 270.1459 | 0.00 |
| MET_MATCH | | | | | 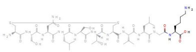 | 87.0561 | 87.0497 | -74.2 |
| MET_MATCH | | | | | 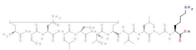 | 130.0866 | 130.0863 | -2.83 |
| MET_MATCH | | | | | 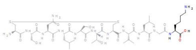 | 147.1130 | 147.1128 | -1.60 |
| MET_MATCH | | | | | 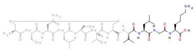 | 200.1396 | 200.1337 | -29.1 |

Metabolite: M1 -2308 RT=1.91

| Type | score | sub. m/z observed | sub. m/z calculated | sub ppm | | met. m/z observed | met. m/z calculated | met. ppm |
|---|---|---|---|---|---|---|---|---|
| MET_MATCH | | | | | 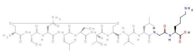 | 204.1348 | 204.1343 | -2.42 |
| MET_MATCH | | | | | 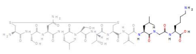 | 317.2185 | 317.2183 | -0.56 |
| MET_MATCH | | | | | 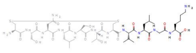 | 416.2874 | 416.2867 | -1.58 |
| MET_MATCH | | | | | 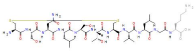 | 496.2191 | 496.2204 | 2.74 |
| MET_MATCH | | | | | 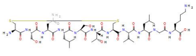 | 539.2561 | 539.2570 | 1.63 |
| MET_MATCH | | | | | 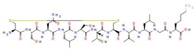 | 553.2584 | 553.2545 | -7.09 |
| MET_MATCH | | | | | 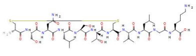 | 553.2584 | 553.2545 | -7.09 |
| MET_MATCH | | | | | 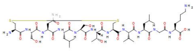 | 553.2584 | 553.2545 | -7.09 |
| MET_MATCH | | | | | 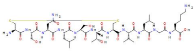 | 561.7690 | 561.7677 | -2.22 |

Metabolite: M1 -2308 RT=1.91

| Type | score | sub. m/z observed | sub. m/z calculated | sub ppm | met. m/z observed | met. m/z calculated | met. ppm |
|---|---|---|---|---|---|---|---|
| MET_MATCH | | | | | 1122.5306 | 1122.5282 | -2.18 |

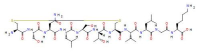

MS (+) FT

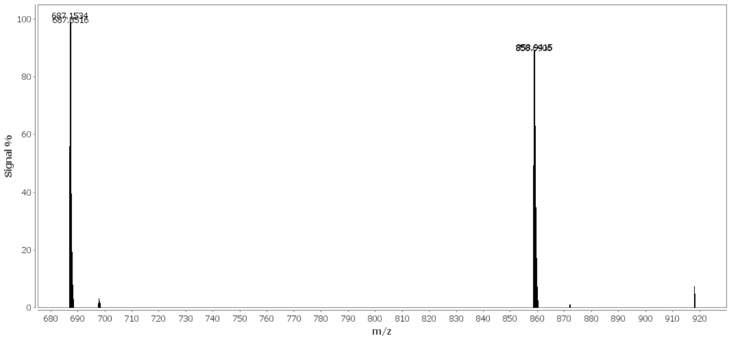


MS (+) FT

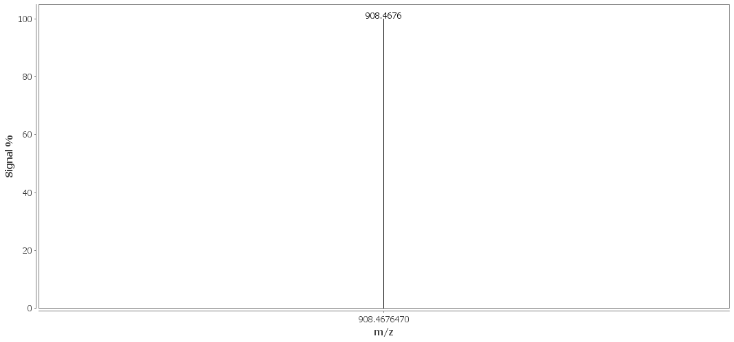


Metabolite: M2 -1615 RT=2.29

| Type | score | sub. m/z observed | sub. m/z calculated | sub ppm | met. m/z observed | met. m/z calculated | met. ppm |
|---|---|---|---|---|---|---|---|
| MATCH | 149.2 | 858.4385 | 858.4356 | -3.35 | 908.4676 | 908.4710 | 3.73 |

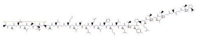

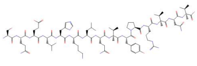

Supplement: S1 File — (ZIP) [file pone.0186461.s007.zip › SFiles/S26_File.pdf]
