# Supplementary material for: Software-aided approach to investigate peptide structure and metabolic susceptibility of amide bonds in peptide drugs based on high resolution mass spectrometry
Source: PLoS One. 2017 Nov 1;12(11):e0186461. doi: 10.1371/journal.pone.0186461 (PMC5665424; doi:10.1371/journal.pone.0186461)

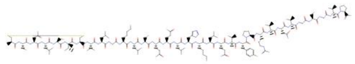

Calcitonin

Property name	Property value
Time	0min, 5min, 15min, 45min, 120min
Instrument	ThermoQAPlus
Matrix	elastase
Acquisition Mode	ddMS2

Chromatograms

Time=0min

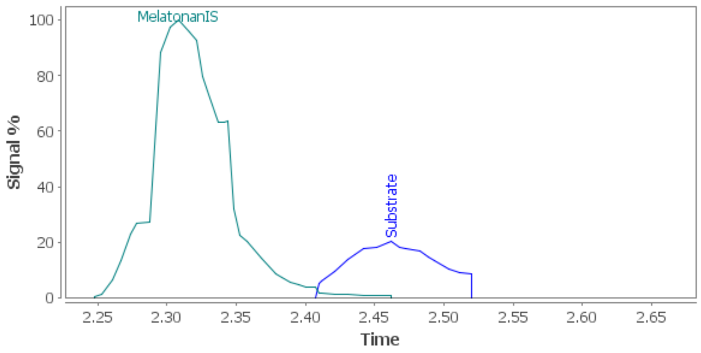

Time=5min

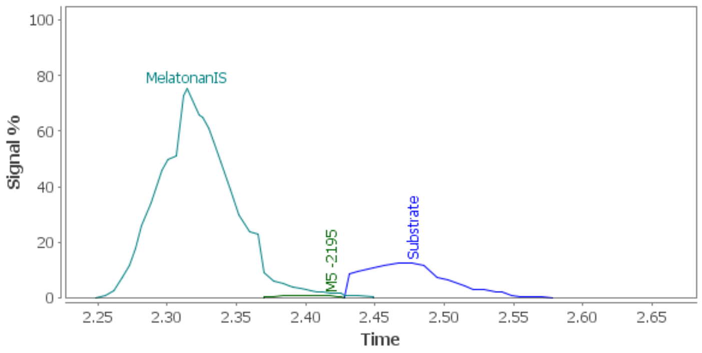

Time=15min

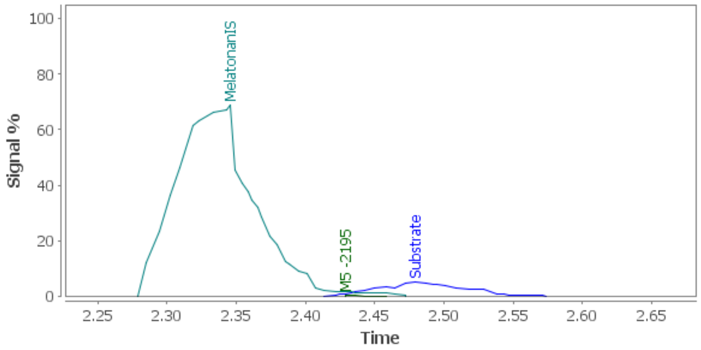

Time=45min

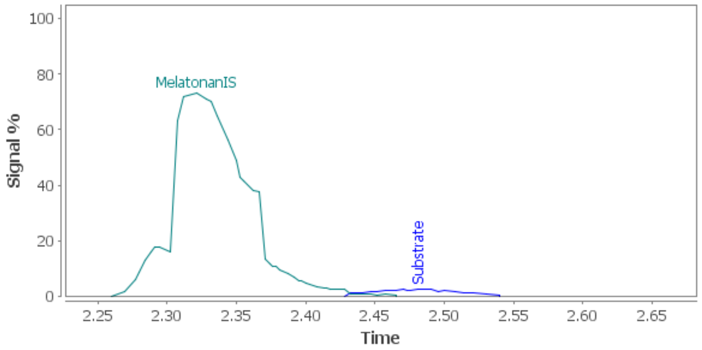

Time=120min

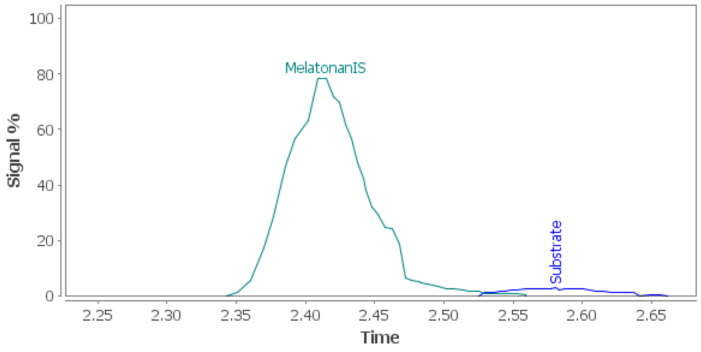

Custom Charts

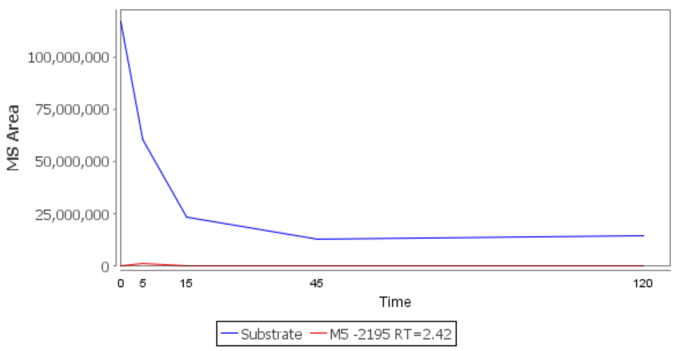

Fragmentation

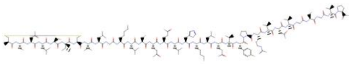

Calcitonin

MS (+) FT

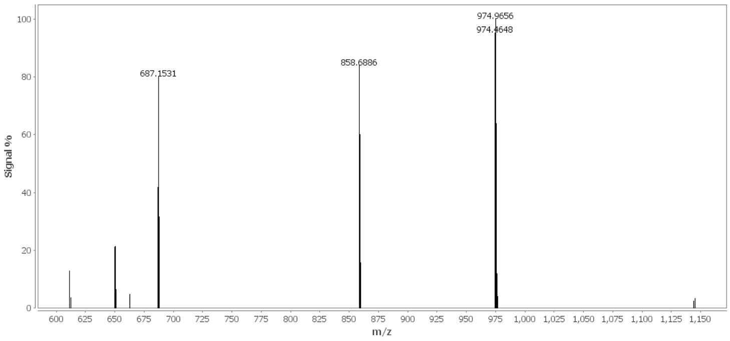

MS (+) FT

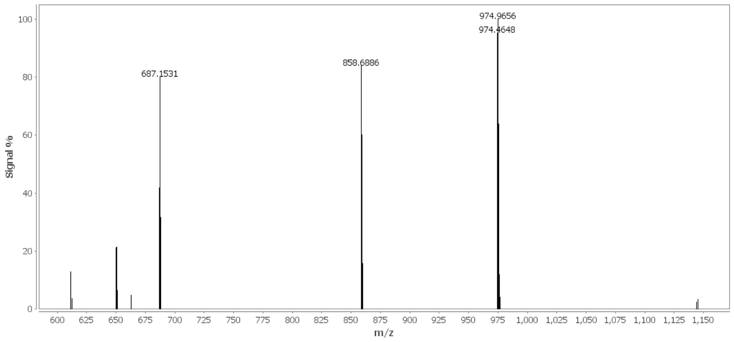

MS2 (+) FT activ = HCD:ce =

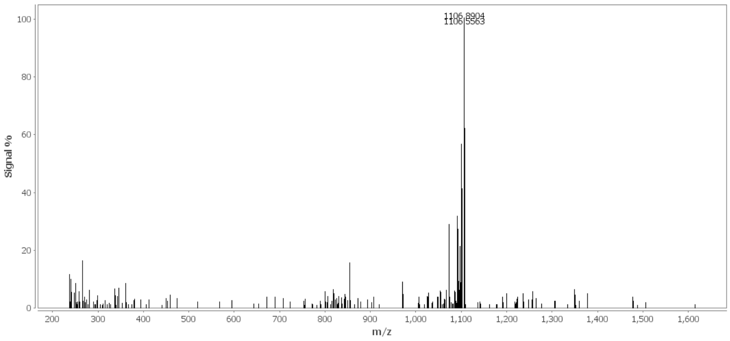

MS2 (+) FT activ = HCD:ce =

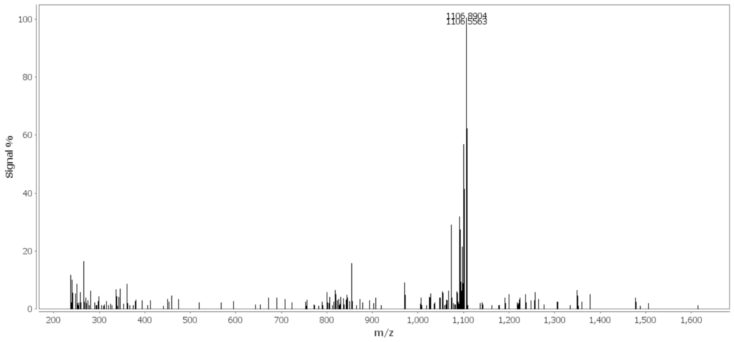

Metabolite: Substrate

Type	score	sub. m/z observed	sub. m/z calculated	sub ppm		met. m/z observed	met. m/z calculated	met. ppm
MISMATCH	-8.5	270.1454	270.1448	-2.21	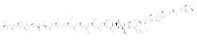 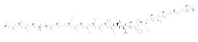	270.1454	270.1448	-2.21
MISMATCH	-8.5	270.1454	270.1448	-2.21	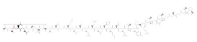 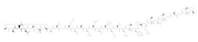	270.1454	270.1448	-2.21
MISMATCH	-8.5	270.1454	270.1448	-2.21	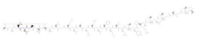 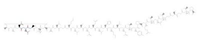	270.1454	270.1448	-2.21

MS (+) FT

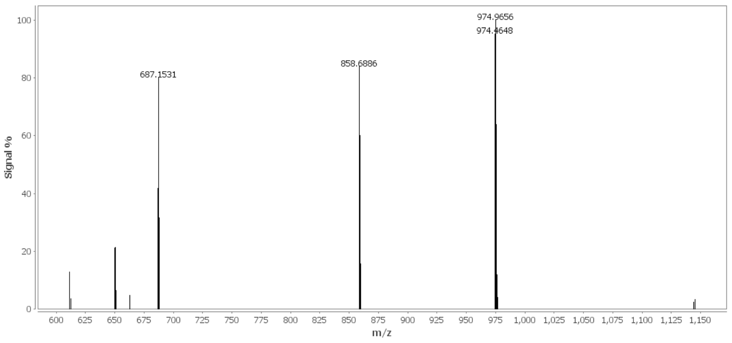

MS (+) FT

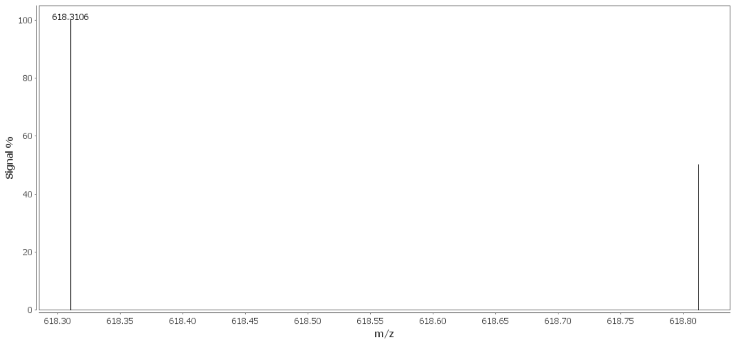

MS2 (+) FT HCD:40.0

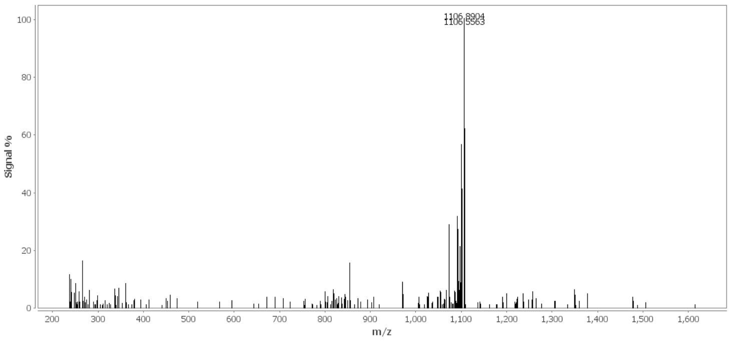

MS2 (+) FT activ = HCD:ce =

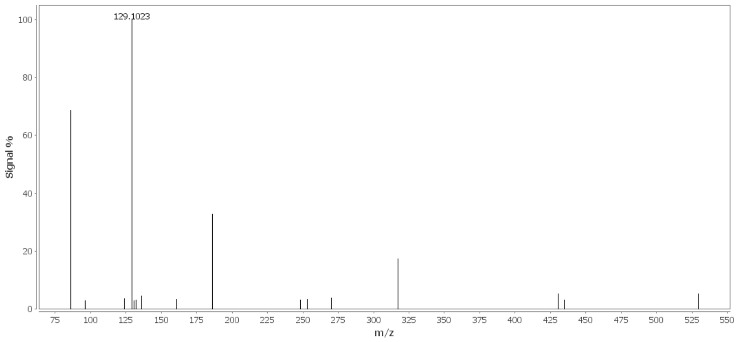

Metabolite: M5 -2195 RT=2.42

Type	score	sub. m/z observed	sub. m/z calculated	sub ppm		met. m/z observed	met. m/z calculated	met. ppm
MISMATCH	-8.5	270.1454	270.1448	-2.21	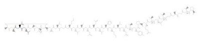	270.1470	270.1470	0.00
MET_MATCH					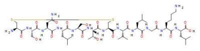	618.3106	618.3098	-1.37
MET_MATCH					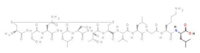	132.1015	132.1019	3.40
MET_MATCH					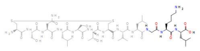	317.2161	317.2183	7.05
MET_MATCH					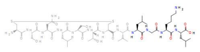	430.3016	430.3024	1.91

Metabolite: M5 -2195 RT=2.42

Type	score	sub. m/z observed	sub. m/z calculated	sub ppm		met. m/z observed	met. m/z calculated	met. ppm
MET_MATCH						529.3702	529.3708	1.08

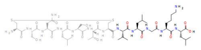

Supplement: S1 File — (ZIP) [file pone.0186461.s007.zip › SFiles/S27_File.pdf]
