# Supplementary material for: Software-aided approach to investigate peptide structure and metabolic susceptibility of amide bonds in peptide drugs based on high resolution mass spectrometry
Source: PLoS One. 2017 Nov 1;12(11):e0186461. doi: 10.1371/journal.pone.0186461 (PMC5665424; doi:10.1371/journal.pone.0186461)
Supplement: S1 File — (ZIP) [file pone.0186461.s007.zip › SFiles/S28_File.pdf]

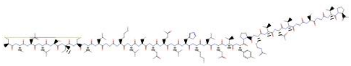

Calcitonin

Property name	Property value
Time	0min, 5min, 15min, 45min, 120min
Instrument	ThermoQAPlus
Acquisition Mode	ddMS2
Matrix	pepsin

Chromatograms

Time=0min

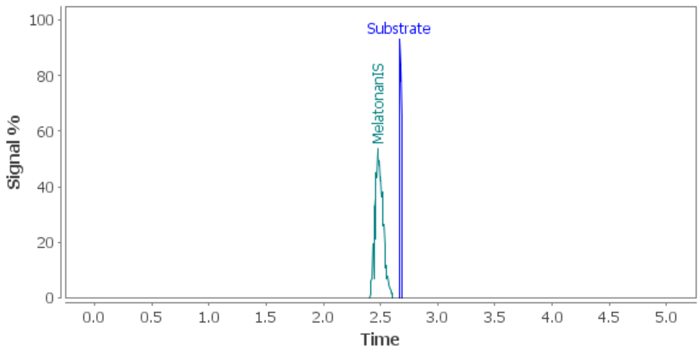

Time=5min

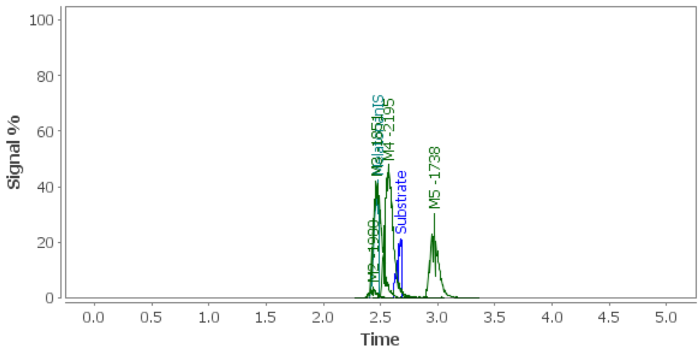

Time=15min

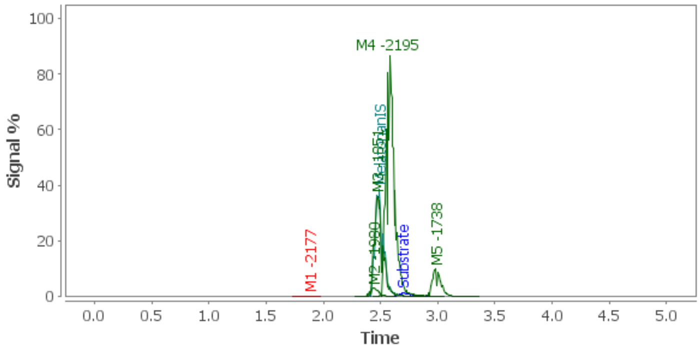

Time=45min

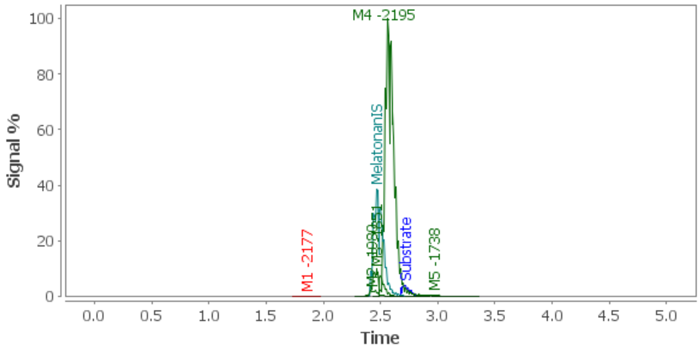

Time=120min

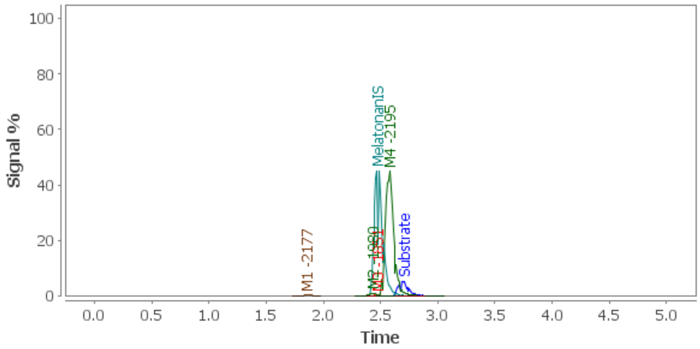

Custom Charts

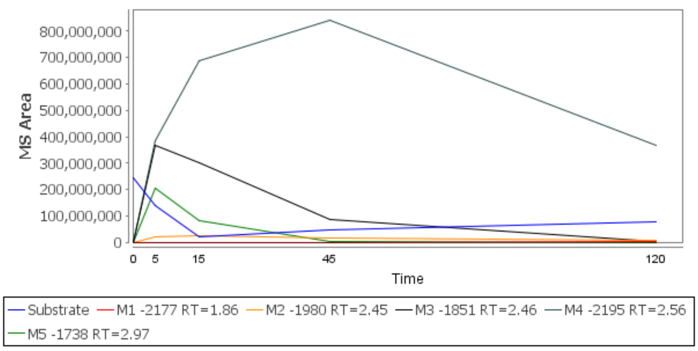

Fragmentation

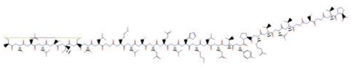

Calcitonin

MS (+) FT

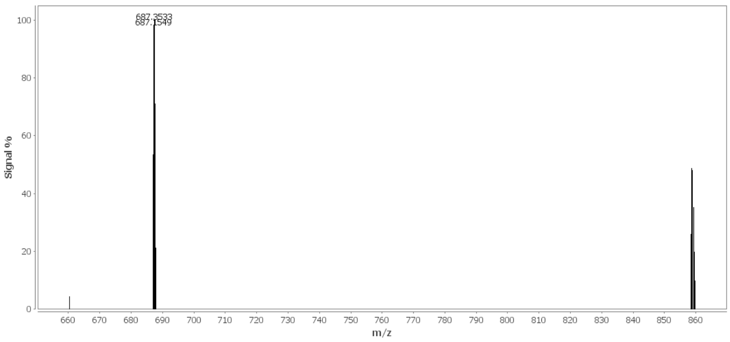

MS (+) FT

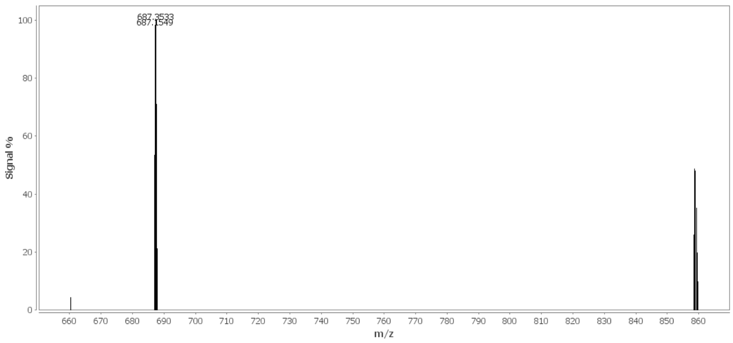

MS2 (+) FT activ = HCD:ce =

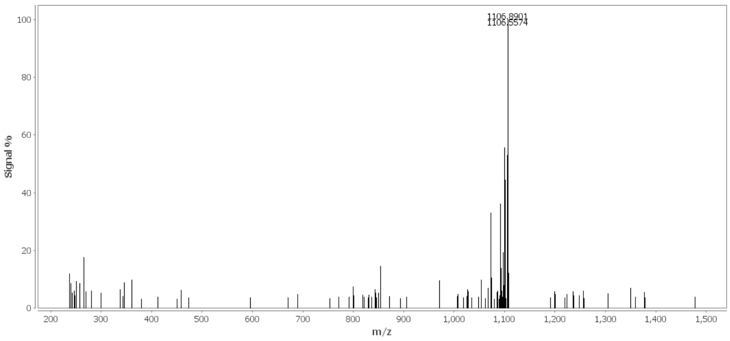

MS2 (+) FT activ = HCD:ce =

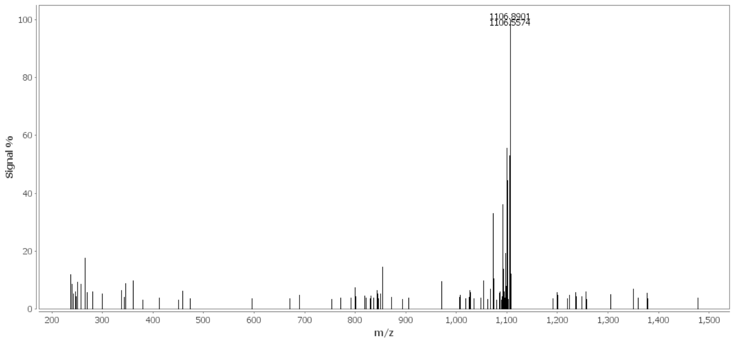

Metabolite: Substrate

Type	score	sub. m/z observed	sub. m/z calculated	sub ppm		met. m/z observed	met. m/z calculated	met. ppm
MATCH	125.8	858.4422	858.4356	-7.64	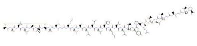 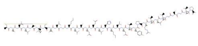	858.4422	858.4356	-7.64
MISMATCH	-6.9	849.9319	849.9223	-11.2	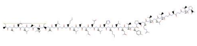 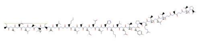	849.9319	849.9223	-11.2
MISMATCH	-6.9	849.9319	849.9223	-11.2	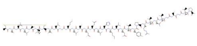 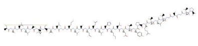	849.9319	849.9223	-11.2

Metabolite: Substrate

Type	score	sub. m/z observed	sub. m/z calculated	sub ppm		met. m/z observed	met. m/z calculated	met. ppm
MISMATCH	-6.9	849.9319	849.9223	-11.2	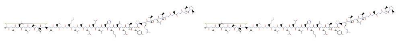	849.9319	849.9223	-11.2
MISMATCH	-6.9	849.9319	849.9223	-11.2	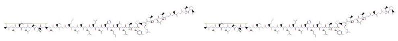	849.9319	849.9223	-11.2
MISMATCH	-6.9	849.9319	849.9223	-11.2	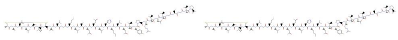	849.9319	849.9223	-11.2
MISMATCH	-6.9	849.9319	849.9223	-11.2	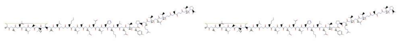	849.9319	849.9223	-11.2
MISMATCH	-6.0	818.6673	818.6695	2.70	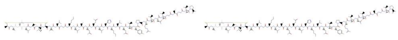	818.6673	818.6695	2.70
MISMATCH	8.5	689.2382	689.2382	-0.09	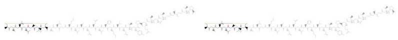	689.2382	689.2382	-0.09
MISMATCH	8.5	689.2382	689.2382	-0.09	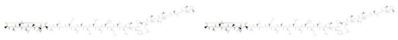	689.2382	689.2382	-0.09
MISMATCH	8.5	689.2382	689.2382	-0.09	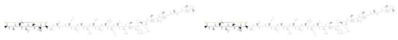	689.2382	689.2382	-0.09
MATCH	11.3	458.2304	458.2245	-12.7	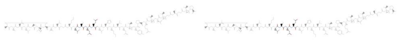	458.2304	458.2245	-12.7

Metabolite: Substrate

Type	score	sub. m/z observed	sub. m/z calculated	sub ppm			met. m/z observed	met. m/z calculated	met. ppm
MATCH	11.3	458.2304	458.2245	-12.7	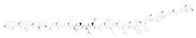	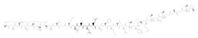	458.2304	458.2245	-12.7
MISMATCH	5.8	450.1498	450.1476	-4.95	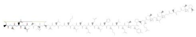	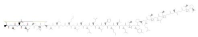	450.1498	450.1476	-4.95
MATCH	16.6	412.2932	412.2918	-3.42	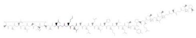	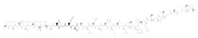	412.2932	412.2918	-3.42
MATCH	16.6	412.2932	412.2918	-3.42	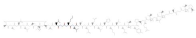	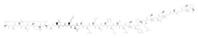	412.2932	412.2918	-3.42
MISMATCH	-24.7	345.1418	345.1405	-3.88	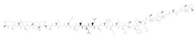	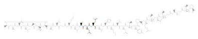	345.1418	345.1405	-3.88
MISMATCH	-24.7	345.1418	345.1405	-3.88	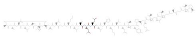	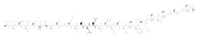	345.1418	345.1405	-3.88
MISMATCH	-9.9	345.1418	345.1445	7.77	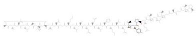	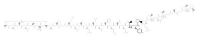	345.1418	345.1445	7.77
MISMATCH	12.9	337.0671	337.0635	-10.7	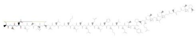	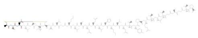	337.0671	337.0635	-10.7
MATCH	23.7	299.2096	299.2078	-6.01	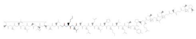	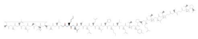	299.2096	299.2078	-6.01

Metabolite: Substrate

Type	score	sub. m/z observed	sub. m/z calculated	sub ppm		met. m/z observed	met. m/z calculated	met. ppm
MISMATCH	23.7	299.2096	299.2078	-6.01	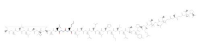 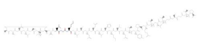	299.2096	299.2078	-6.01
MISMATCH	21.7	299.2096	299.2078	-6.01	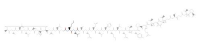 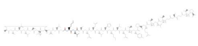	299.2096	299.2078	-6.01
MISMATCH	23.7	299.2096	299.2078	-6.01	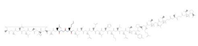 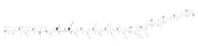	299.2096	299.2078	-6.01
MISMATCH	-8.2	270.1459	270.1448	-3.97	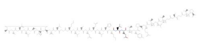 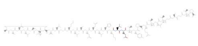	270.1459	270.1448	-3.97
MISMATCH	-8.2	270.1459	270.1486	9.89	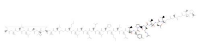 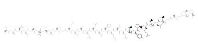	270.1459	270.1486	9.89
MISMATCH	10.9	270.1459	270.1448	-3.97	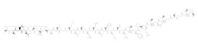 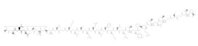	270.1459	270.1448	-3.97
MISMATCH	-8.2	270.1459	270.1448	-3.97	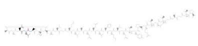 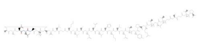	270.1459	270.1448	-3.97
MISMATCH	-10.3	258.1086	258.1084	-0.64	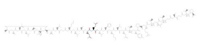 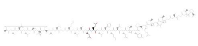	258.1086	258.1084	-0.64
MISMATCH	-10.3	258.1086	258.1084	-0.64	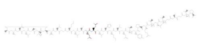 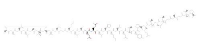	258.1086	258.1084	-0.64

MS (+) FT

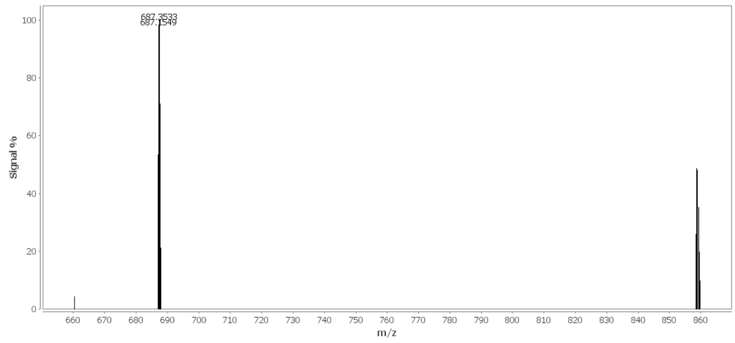

MS (+) FT

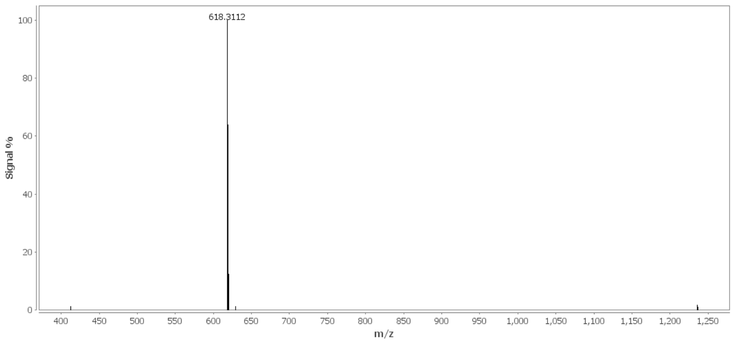

MS2 (+) FT activ = HCD:ce =

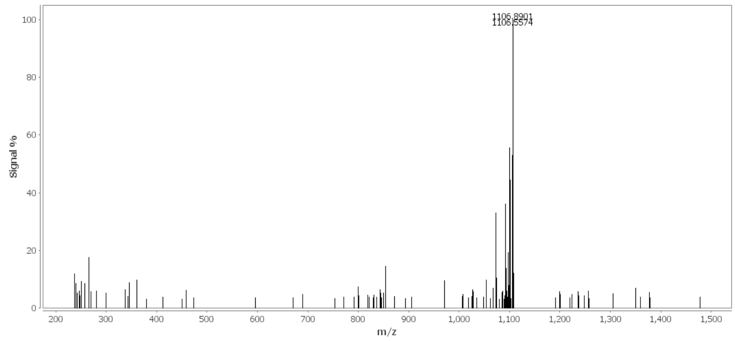

MS2 (+) FT activ = HCD:ce =

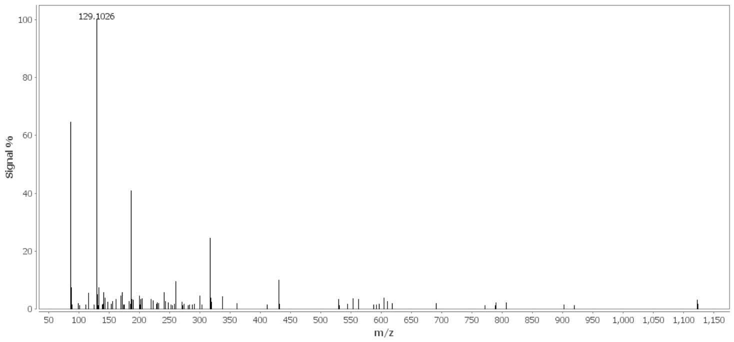

Metabolite: M4 -2195 RT=2.56

Type	score	sub. m/z observed	sub. m/z calculated	sub ppm		met. m/z observed	met. m/z calculated	met. ppm
MATCH	27.1	858.4422	858.4356	-7.64	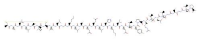 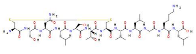	412.5431	412.5423	-1.89
MATCH	9.8	299.2096	299.2078	-6.01	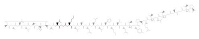 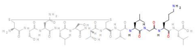	299.2087	299.2078	-3.12
MATCH	9.8	299.2096	299.2078	-6.01	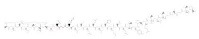 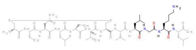	299.2087	299.2078	-3.12
MISMATCH	-10.3	258.1086	258.1084	-0.64	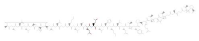	258.1088	258.1088	0.00
MISMATCH	-8.2	270.1459	270.1448	-3.97	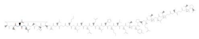	270.1451	270.1451	0.00

Metabolite: M4 -2195 RT=2.56

Type	score	sub. m/z observed	sub. m/z calculated	sub ppm		met. m/z observed	met. m/z calculated	met. ppm
MISMATCH	-10.6	337.0671	337.0635	-10.7	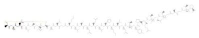	337.0636	337.0636	0.00
MET_MATCH					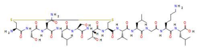	618.3112	618.3098	-2.39
MET_MATCH					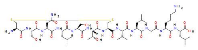	1235.6146	1235.6123	-1.93
MET_MATCH					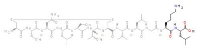	243.1706	243.1703	-1.22
MET_MATCH					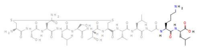	260.1972	260.1969	-1.32
MET_MATCH					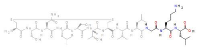	317.2188	317.2183	-1.40
MET_MATCH					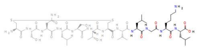	430.3036	430.3024	-2.74
MET_MATCH					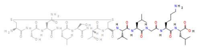	529.3693	529.3708	2.87
MET_MATCH					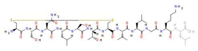	552.7657	552.7625	-5.85

Metabolite: M4 -2195 RT=2.56

Type	score	sub. m/z observed	sub. m/z calculated	sub ppm		met. m/z observed	met. m/z calculated	met. ppm
MET_MATCH					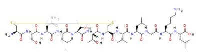	595.7994	595.7990	-0.57
MET_MATCH					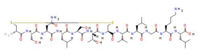	609.8038	609.7965	-11.9
MET_MATCH					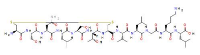	609.8038	609.7965	-11.9
MET_MATCH					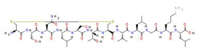	609.8038	609.7965	-11.9
MET_MATCH					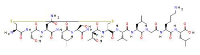	618.3112	618.3098	-2.26
MET_MATCH					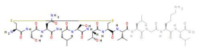	806.3139	806.3171	4.07
MET_MATCH					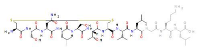	919.4039	919.4012	-2.91

MS (+) FT

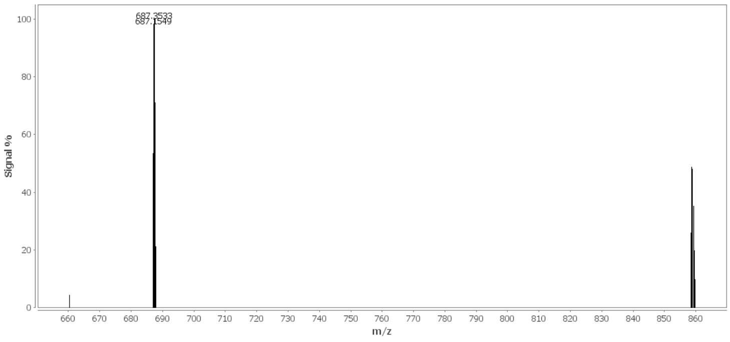

MS (+) FT

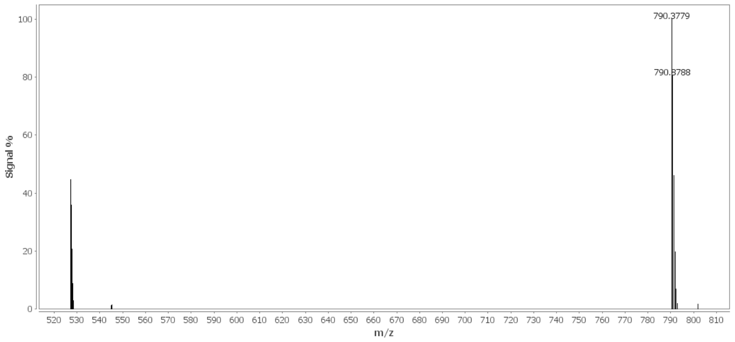

MS2 (+) FT activ = HCD:ce =

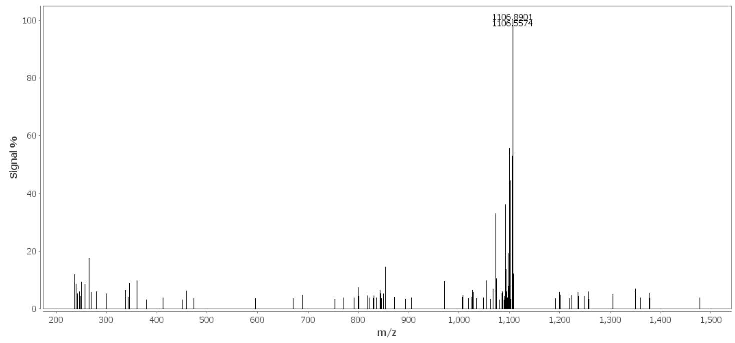

MS2 (+) FT activ = HCD:ce =

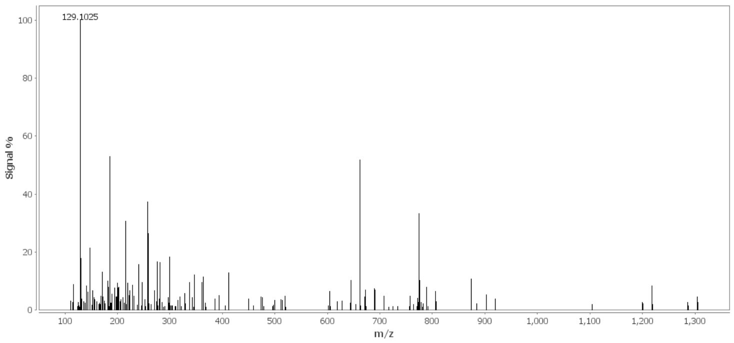

Metabolite: M3 -1851 RT=2.46

Type	score	sub. m/z observed	sub. m/z calculated	sub ppm		met. m/z observed	met. m/z calculated	met. ppm
MATCH	70.5	858.4422	858.4356	-7.64	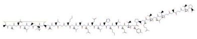 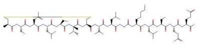	527.2547	527.2533	-2.61
MATCH	23.7	299.2096	299.2078	-6.01	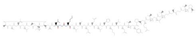 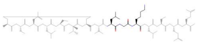	299.2080	299.2078	-0.73
MATCH	23.7	299.2096	299.2078	-6.01	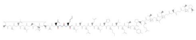 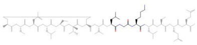	299.2080	299.2078	-0.73
MATCH	23.7	299.2096	299.2078	-6.01	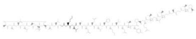 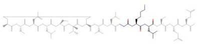	299.2080	299.2078	-0.73
MATCH	16.6	412.2932	412.2918	-3.42	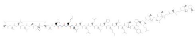 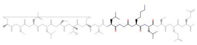	412.2929	412.2918	-2.68
MATCH	16.6	412.2932	412.2918	-3.42	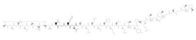 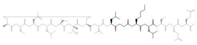	412.2929	412.2918	-2.68
MISMATCH	-12.6	270.1459	270.1448	-3.97	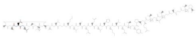	270.1449	270.1449	0.00

Metabolite: M3 -1851 RT=2.46

Type	score	sub. m/z observed	sub. m/z calculated	sub ppm		met. m/z observed	met. m/z calculated	met. ppm
MISMATCH	-15.9	337.0671	337.0635	-10.7	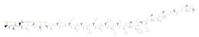	337.0632	337.0632	0.00
MISMATCH	-9.9	345.1418	345.1445	7.77	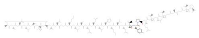	345.1418	345.1418	0.00
MISMATCH	-6.8	450.1498	450.1476	-4.95	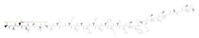	450.1479	450.1479	0.00
MISMATCH	-12.2	689.2382	689.2382	-0.09	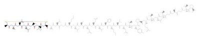	689.2377	689.2377	0.00
MET_MATCH					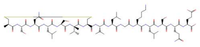	790.3779	790.3764	-1.94
MET_MATCH					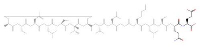	130.0501	130.0499	-1.41
MET_MATCH					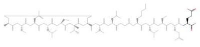	148.0606	148.0604	-1.05
MET_MATCH					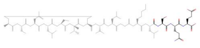	195.0766	195.0688	-40.0
MET_MATCH					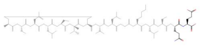	259.0915	259.0925	3.55

Metabolite: M3 -1851 RT=2.46

Type	score	sub. m/z observed	sub. m/z calculated	sub ppm		met. m/z observed	met. m/z calculated	met. ppm
MET_MATCH					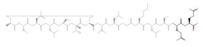	276.1189	276.1190	0.52
MET_MATCH					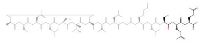	346.1250	346.1245	-1.49
MET_MATCH					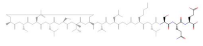	363.1516	363.1510	-1.49
MET_MATCH					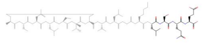	459.2108	459.2086	-4.88
MET_MATCH					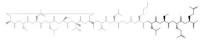	476.2376	476.2351	-5.29
MET_MATCH					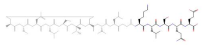	604.3313	604.3301	-2.05
MET_MATCH					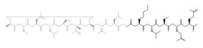	644.3250	644.3250	0.04
MET_MATCH					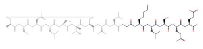	661.3522	661.3515	-1.04
MET_MATCH					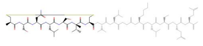	707.2414	707.2487	10.33

Metabolite: M3 -1851 RT=2.46

Type	score	sub. m/z observed	sub. m/z calculated	sub ppm		met. m/z observed	met. m/z calculated	met. ppm
MET_MATCH					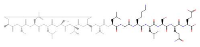	757.4115	757.4090	-3.25
MET_MATCH					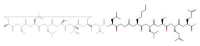	774.4370	774.4356	-1.85
MET_MATCH					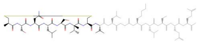	778.3253	778.3222	-3.93
MET_MATCH					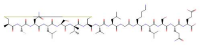	781.3742	781.3711	-4.05
MET_MATCH					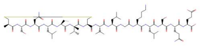	781.3742	781.3711	-4.05
MET_MATCH					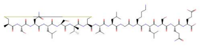	781.3742	781.3711	-4.05
MET_MATCH					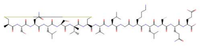	781.3742	781.3711	-4.05
MET_MATCH					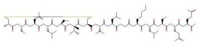	781.8719	781.8631	-11.2
MET_MATCH					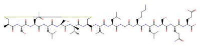	781.8719	781.8631	-11.2

Metabolite: M3 -1851 RT=2.46

Type	score	sub. m/z observed	sub. m/z calculated	sub ppm		met. m/z observed	met. m/z calculated	met. ppm
MET_MATCH					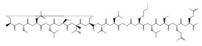	781.8719	781.8631	-11.2
MET_MATCH					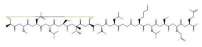	781.8719	781.8631	-11.2
MET_MATCH					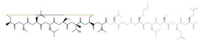	806.3143	806.3171	3.53
MET_MATCH					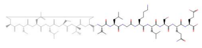	873.5078	873.5040	-4.29
MET_MATCH					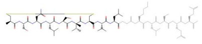	919.3991	919.4012	2.25
MET_MATCH					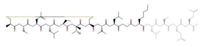	1104.5154	1104.5176	2.00
MET_MATCH					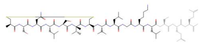	1217.6035	1217.6017	-1.49
MET_MATCH					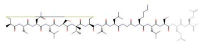	1304.6332	1304.6337	0.37

MS (+) FT

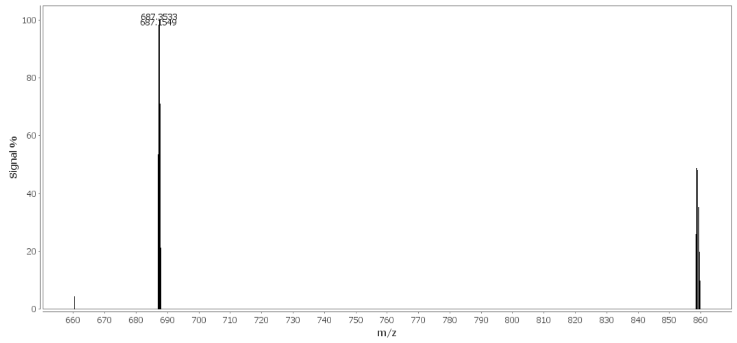

MS (+) FT

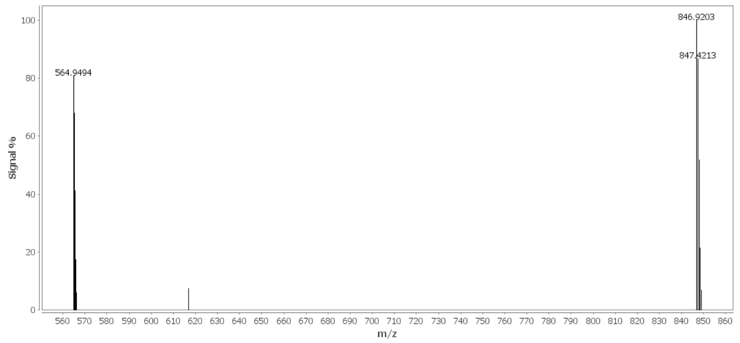

MS2 (+) FT activ = HCD:ce =

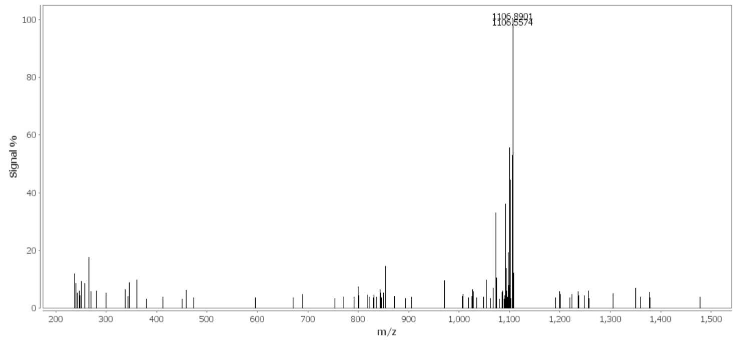

MS2 (+) FT activ = HCD:ce =

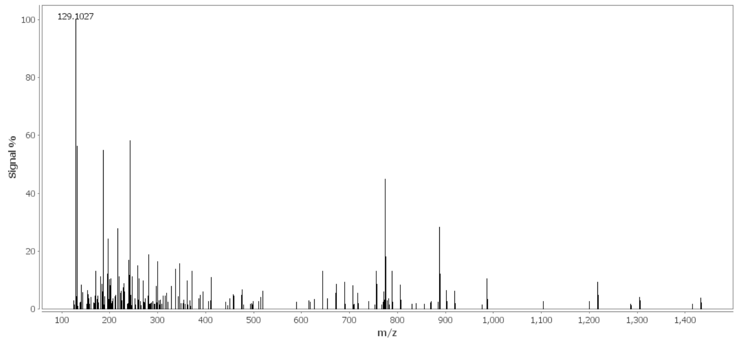

Metabolite: M5 -1738 RT=2.97

Type	score	sub. m/z observed	sub. m/z calculated	sub ppm		met. m/z observed	met. m/z calculated	met. ppm
MATCH	106.6	858.4422	858.4356	-7.64	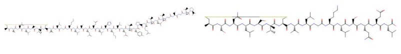	564.9494	564.9480	-2.51
MATCH	21.7	299.2096	299.2078	-6.01	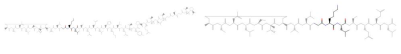	299.2089	299.2078	-3.84
MATCH	21.7	299.2096	299.2078	-6.01	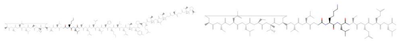	299.2089	299.2078	-3.84
MATCH	14.6	412.2932	412.2918	-3.42	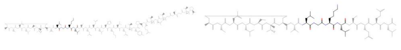	412.2935	412.2918	-4.12
MATCH	14.6	412.2932	412.2918	-3.42	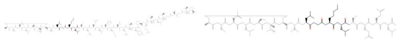	412.2935	412.2918	-4.12

Metabolite: M5 -1738 RT=2.97

Type	score	sub. m/z observed	sub. m/z calculated	sub ppm		met. m/z observed	met. m/z calculated	met. ppm
MATCH	11.3	458.2304	458.2245	-12.7	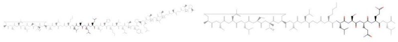	458.2266	458.2245	-4.51
MATCH	11.3	458.2304	458.2245	-12.7	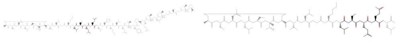	458.2266	458.2245	-4.51
MISMATCH	-23.5	258.1086	258.1084	-0.64	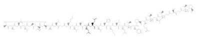	258.1092	258.1092	0.00
MISMATCH	-15.5	270.1459	270.1448	-3.97	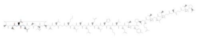	270.1455	270.1455	0.00
MISMATCH	-20.2	337.0671	337.0635	-10.7	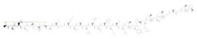	337.0644	337.0644	0.00
MISMATCH	-24.7	345.1418	345.1405	-3.88	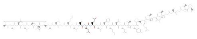	345.1416	345.1416	0.00
MISMATCH	-6.7	450.1498	450.1476	-4.95	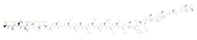	450.1487	450.1487	0.00
MISMATCH	-14.0	689.2382	689.2382	-0.09	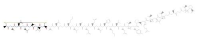	689.2409	689.2409	0.00
MISMATCH	-6.0	818.6673	818.6695	2.70	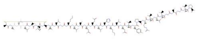	767.3796	767.3796	0.00

Metabolite: M5 -1738 RT=2.97

Type	score	sub. m/z observed	sub. m/z calculated	sub ppm		met. m/z observed	met. m/z calculated	met. ppm
MISMATCH	-6.9	849.9319	849.9223	-11.2	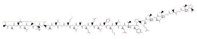	829.9095	829.9095	0.00
MET_MATCH					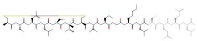	616.8172	616.8099	-11.7
MET_MATCH					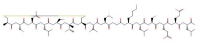	846.9203	846.9184	-2.30
MET_MATCH					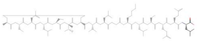	132.1023	132.1019	-2.83
MET_MATCH					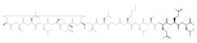	195.1134	195.1052	-41.9
MET_MATCH					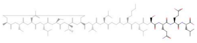	230.1142	230.1079	-27.1
MET_MATCH					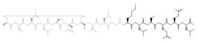	239.1400	239.1377	-9.64
MET_MATCH					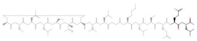	261.1456	261.1445	-4.19
MET_MATCH					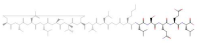	294.1458	294.1554	32.48

Metabolite: M5 -1738 RT=2.97

Type	score	sub. m/z observed	sub. m/z calculated	sub ppm		met. m/z observed	met. m/z calculated	met. ppm
MET_MATCH					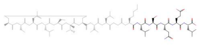	308.1617	308.1529	-28.5
MET_MATCH					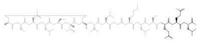	389.2041	389.2031	-2.59
MET_MATCH					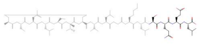	476.2364	476.2351	-2.62
MET_MATCH					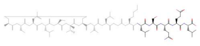	589.3228	589.3192	-6.08
MET_MATCH					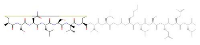	707.2499	707.2487	-1.61
MET_MATCH					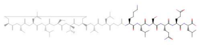	717.4223	717.4141	-11.3
MET_MATCH					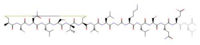	767.3796	767.3736	-7.76
MET_MATCH					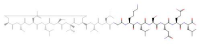	774.4380	774.4356	-3.09
MET_MATCH					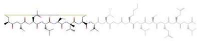	778.3273	778.3222	-6.56

Metabolite: M5 -1738 RT=2.97

Type	score	sub. m/z observed	sub. m/z calculated	sub ppm		met. m/z observed	met. m/z calculated	met. ppm
MET_MATCH					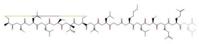	781.3754	781.3711	-5.52
MET_MATCH					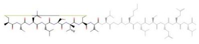	806.3166	806.3171	0.68
MET_MATCH					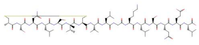	838.4070	838.4051	-2.27
MET_MATCH					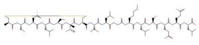	838.4070	838.4051	-2.27
MET_MATCH					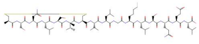	838.4070	838.4051	-2.27
MET_MATCH					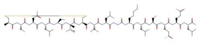	838.4070	838.4051	-2.27
MET_MATCH					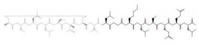	887.5233	887.5197	-4.15
MET_MATCH					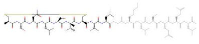	919.4050	919.4012	-4.08
MET_MATCH					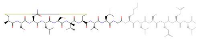	976.4304	976.4227	-7.88

Metabolite: M5 -1738 RT=2.97

Type	score	sub. m/z observed	sub. m/z calculated	sub ppm		met. m/z observed	met. m/z calculated	met. ppm
MET_MATCH						986.5933	986.5881	-5.31
					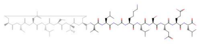			
MET_MATCH						1104.5187	1104.5176	-0.94
					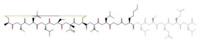			
MET_MATCH						1217.6049	1217.6017	-2.67
					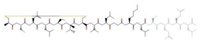			

MS (+) FT

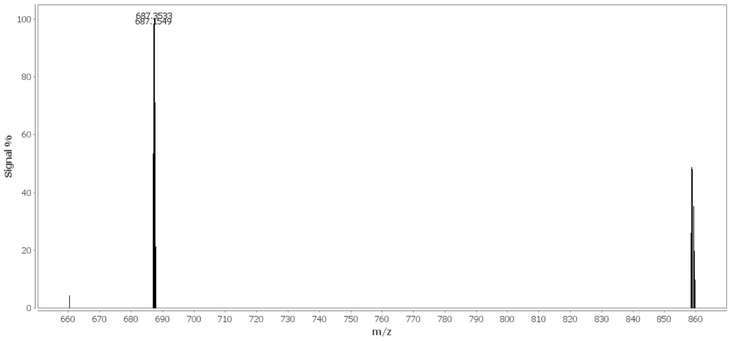

MS (+) FT

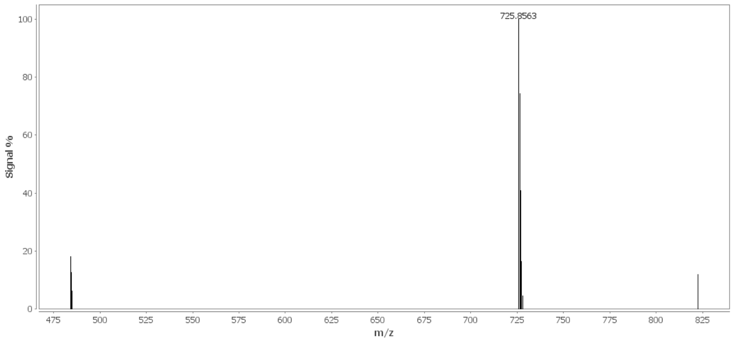

MS2 (+) FT activ = HCD:ce =

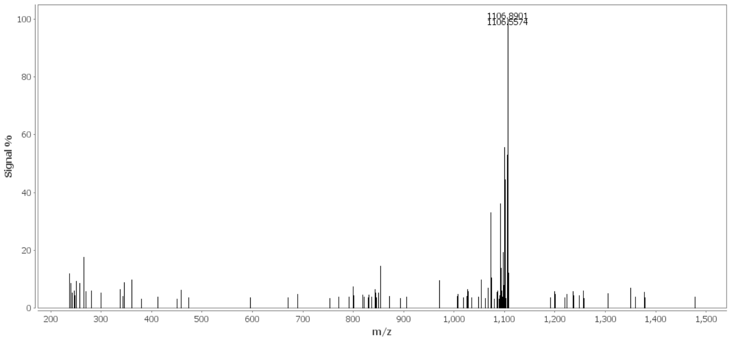

MS2 (+) FT activ = HCD:ce =

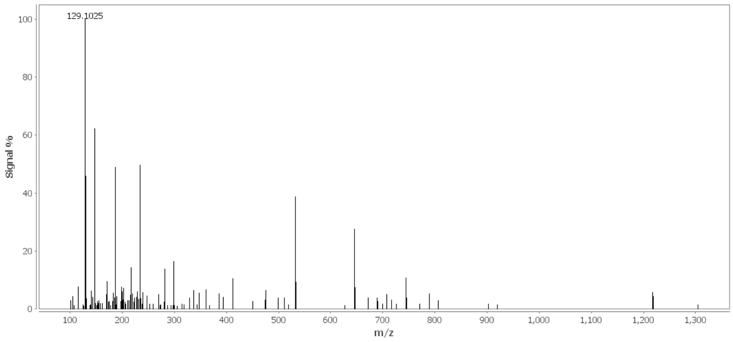

Metabolite: M2 -1980 RT=2.45

Type	score	sub. m/z observed	sub. m/z calculated	sub ppm		met. m/z observed	met. m/z calculated	met. ppm
MATCH	43.9	858.4422	858.4356	-7.64		484.2398	484.2391	-1.37
					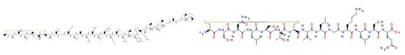			

Metabolite: M2 -1980 RT=2.45

Type	score	sub. m/z observed	sub. m/z calculated	sub ppm		met. m/z observed	met. m/z calculated	met. ppm
MATCH	10.9	270.1459	270.1448	-3.97	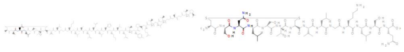	270.1445	270.1448	1.11
MATCH	12.9	337.0671	337.0635	-10.7	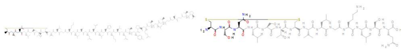	337.0637	337.0635	-0.66
MATCH	5.8	450.1498	450.1476	-4.95	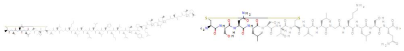	450.1473	450.1476	0.51
MATCH	8.5	689.2382	689.2382	-0.09	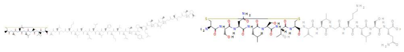	689.2338	689.2382	6.37
MATCH	8.5	689.2382	689.2382	-0.09	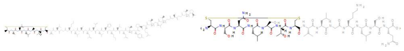	689.2338	689.2382	6.37
MATCH	8.5	689.2382	689.2382	-0.09	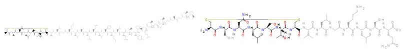	689.2338	689.2382	6.37
MISMATCH	-10.9	270.1459	270.1486	9.89	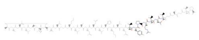	270.1445	270.1445	0.00
MISMATCH	-21.7	299.2096	299.2078	-6.01	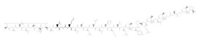	299.2084	299.2084	0.00
MISMATCH	-14.2	412.2932	412.2918	-3.42	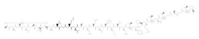	412.2928	412.2928	0.00

Metabolite: M2 -1980 RT=2.45

Type	score	sub. m/z observed	sub. m/z calculated	sub ppm		met. m/z observed	met. m/z calculated	met. ppm
MET_MATCH					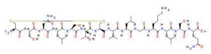	725.8563	725.8551	-1.71
MET_MATCH					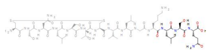	125.0715	125.0621	-74.9
MET_MATCH					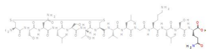	130.0501	130.0499	-2.11
MET_MATCH					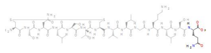	147.0766	147.0764	-1.30
MET_MATCH					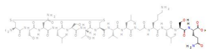	217.0823	217.0819	-1.81
MET_MATCH					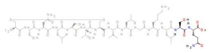	234.1086	234.1084	-0.84
MET_MATCH					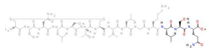	347.1926	347.1925	-0.31
MET_MATCH					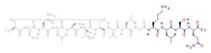	475.2889	475.2875	-2.95
MET_MATCH					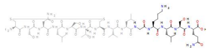	532.3096	532.3089	-1.32

Metabolite: M2 -1980 RT=2.45

Type	score	sub. m/z observed	sub. m/z calculated	sub ppm		met. m/z observed	met. m/z calculated	met. ppm
MET_MATCH					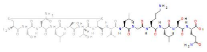	645.3934	645.3930	-0.66
MET_MATCH					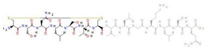	707.2471	707.2487	2.23
MET_MATCH					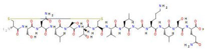	717.3436	717.3418	-2.58
MET_MATCH					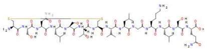	717.3436	717.3418	-2.58
MET_MATCH					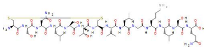	717.3436	717.3418	-2.58
MET_MATCH					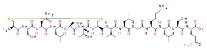	717.3436	717.3418	-2.58
MET_MATCH					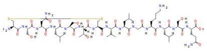	725.8541	725.8551	1.37
MET_MATCH					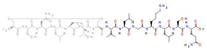	744.4611	744.4614	0.40
MET_MATCH					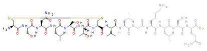	806.3132	806.3171	4.89

Metabolite: M2 -1980 RT=2.45

Type	score	sub. m/z observed	sub. m/z calculated	sub ppm	met. m/z observed	met. m/z calculated	met. ppm
MET_MATCH					919.4033	919.4012	-2.31

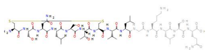

MS (+) FT

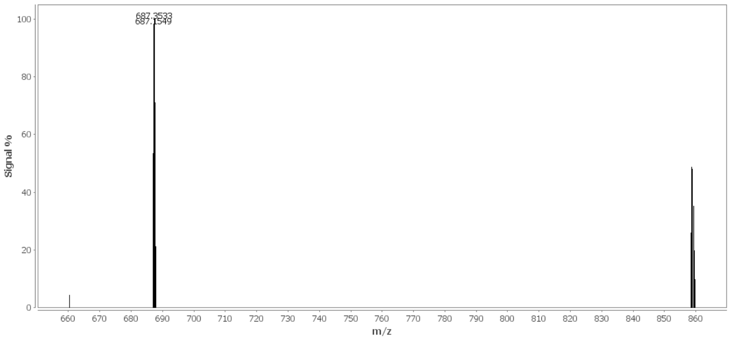

MS (+) FT

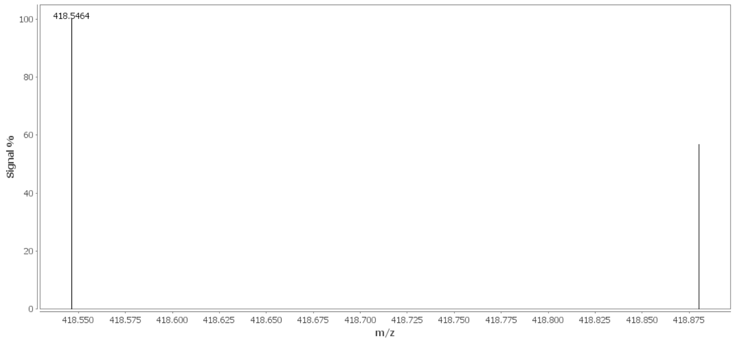

MS2 (+) FT activ = HCD:ce =

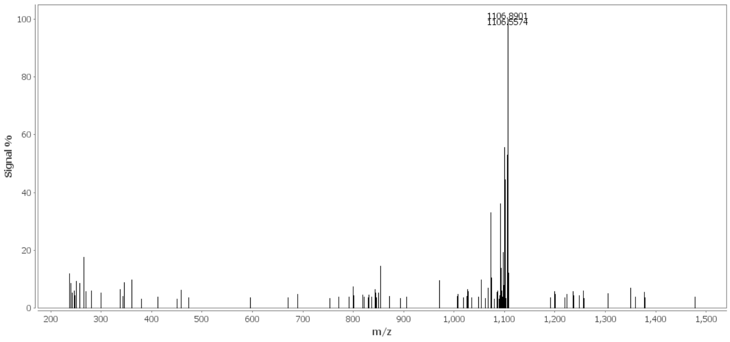

MS2 (+) FT activ = HCD:ce =

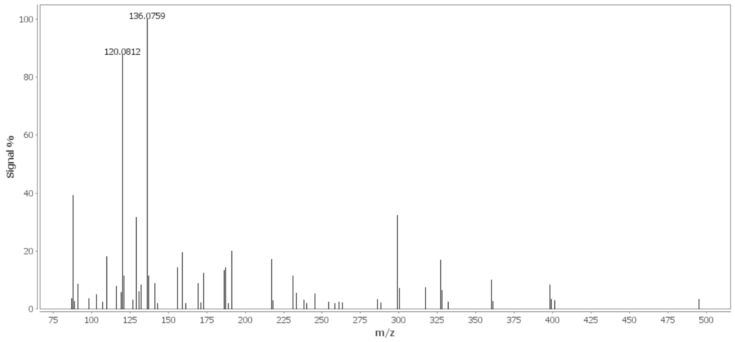

Metabolite: M1 -2177 RT=1.86

Type	score	sub. m/z observed	sub. m/z calculated	sub ppm	met. m/z observed	met. m/z calculated	met. ppm
MATCH	125.8	858.4422	858.4356	-7.64	418.5464	418.5458	-1.45
MISMATCH	-24.8	299.2096	299.2078	-6.01	159.1129	159.1129	0.00
MISMATCH	-12.6	299.2096	299.2078	-6.01	317.2181	317.2181	0.00

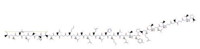

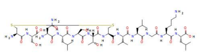

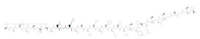

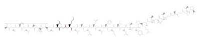

Metabolite: M1 -2177 RT=1.86

Type	score	sub. m/z observed	sub. m/z calculated	sub ppm		met. m/z observed	met. m/z calculated	met. ppm
MET_MATCH					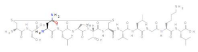	87.0559	87.0553	-7.34
MET_MATCH					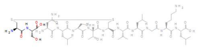	88.0400	88.0393	-8.25
MET_MATCH					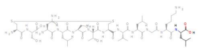	132.1022	132.1019	-1.89
MET_MATCH					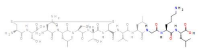	159.1129	159.1128	-0.79
MET_MATCH					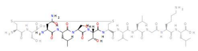	217.1338	217.1239	-45.5
MET_MATCH					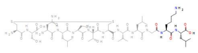	286.1759	286.1761	0.91
MET_MATCH					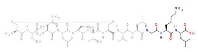	317.2181	317.2183	0.87
MET_MATCH					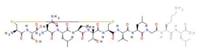	332.1592	332.1493	-29.8
