# Supplementary material for: Software-aided approach to investigate peptide structure and metabolic susceptibility of amide bonds in peptide drugs based on high resolution mass spectrometry
Source: PLoS One. 2017 Nov 1;12(11):e0186461. doi: 10.1371/journal.pone.0186461 (PMC5665424; doi:10.1371/journal.pone.0186461)
Supplement: S1 File — (ZIP) [file pone.0186461.s007.zip › SFiles/S29_File.pdf]

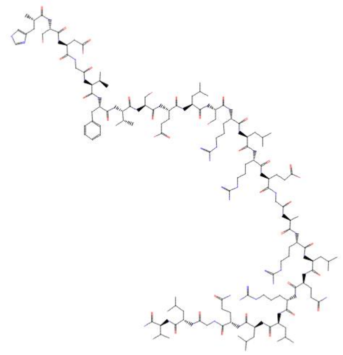

Secretin

Property name	Property value
Time	0min, 5min, 15min, 45min, 120min
Instrument	ThermoQAPlus
Matrix	chymotrypsin
Acquisition Mode	ddMS2

Chromatograms

Time=0min

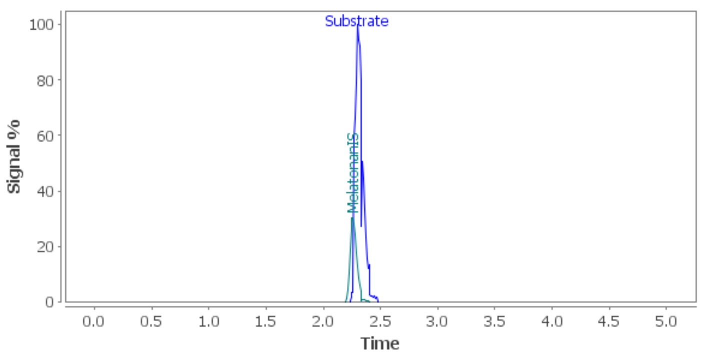

Time=5min

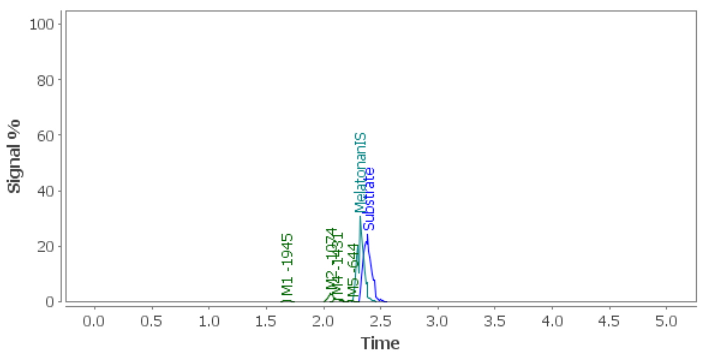

Time=15min

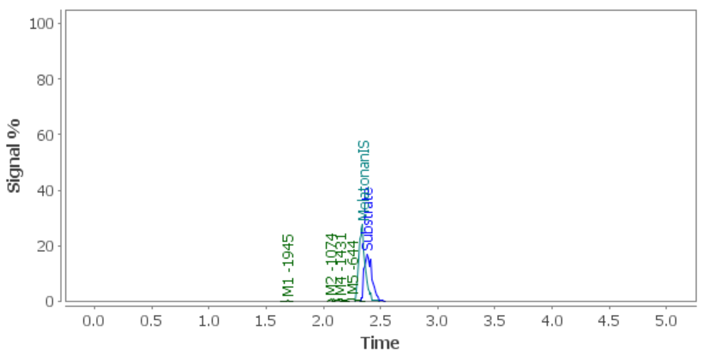

Time=45min

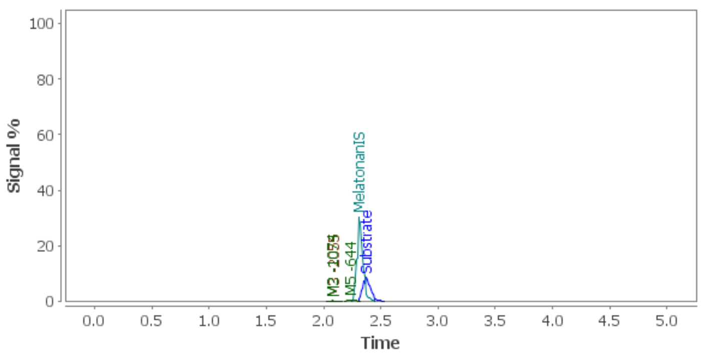

Time=120min

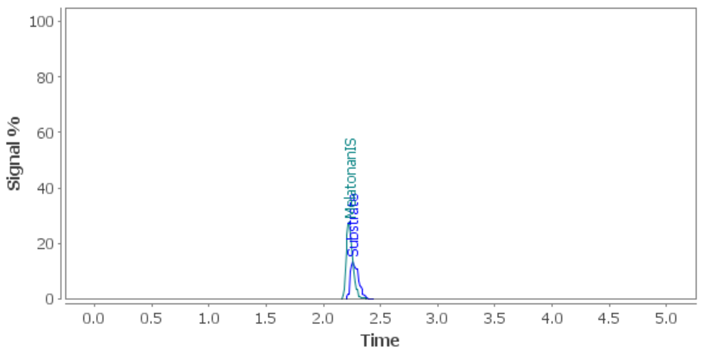

Custom Charts

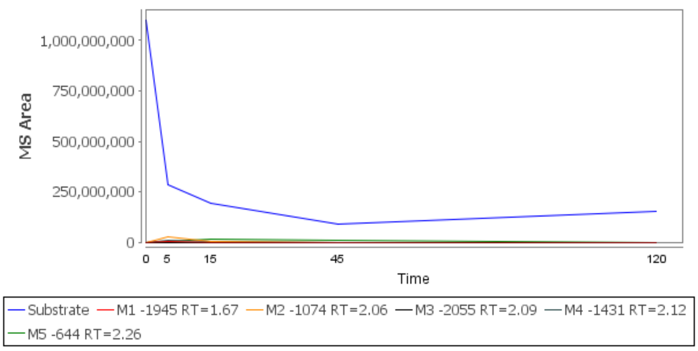

Fragmentation

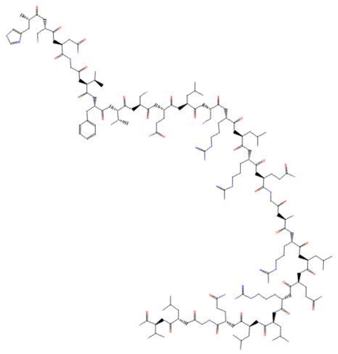

Secretin

MS (+) FT

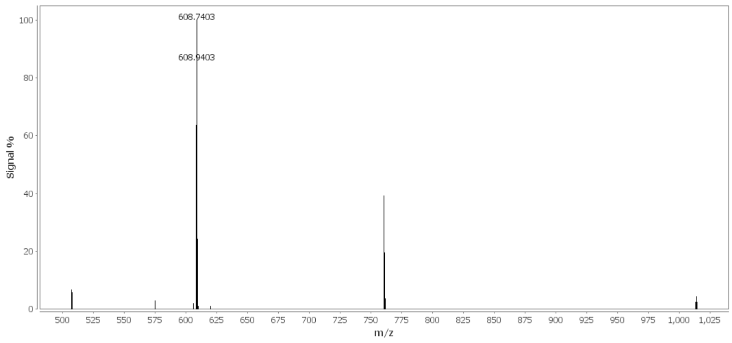

MS (+) FT

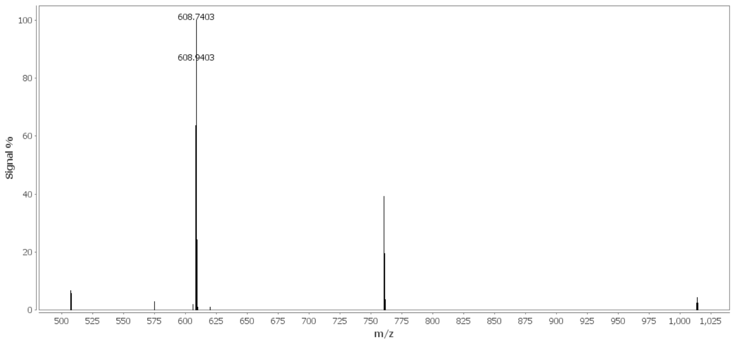

MS2 (+) FT activ = HCD:ce =

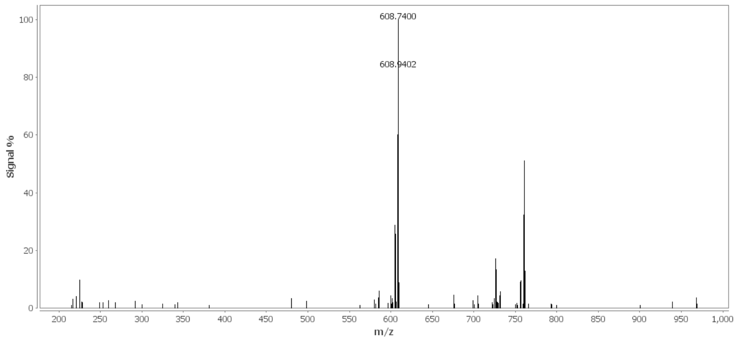

MS2 (+) FT activ = HCD:ce =

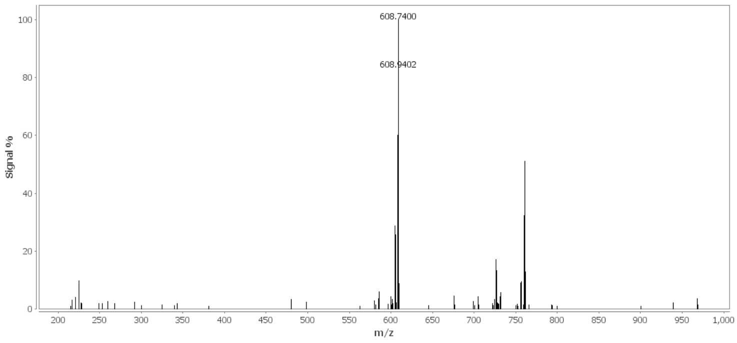

Metabolite: Substrate

Type	score	sub. m/z observed	sub. m/z calculated	sub ppm			met. m/z observed	met. m/z calculated	met. ppm
MATCH	102.4	1013.5654	1013.5584	-6.94	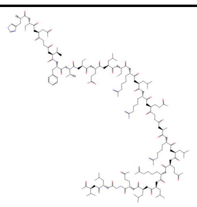	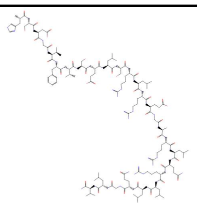	1013.5654	1013.5584	-6.94
MATCH	124.6	760.4255	760.4206	-6.45	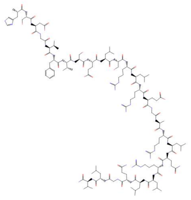	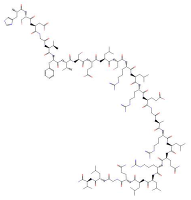	760.4255	760.4206	-6.45
MATCH	132.4	760.4252	760.4206	-6.06	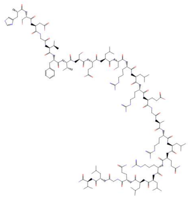	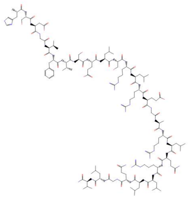	760.4252	760.4206	-6.06

Metabolite: Substrate

Type	score	sub. m/z observed	sub. m/z calculated	sub ppm			met. m/z observed	met. m/z calculated	met. ppm
MATCH	31.0	756.1704	756.1640	-8.44	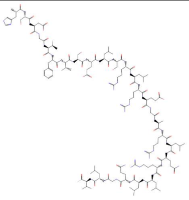	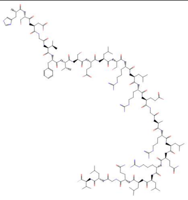	756.1704	756.1640	-8.44
MATCH	31.0	756.1704	756.1640	-8.44	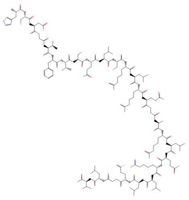	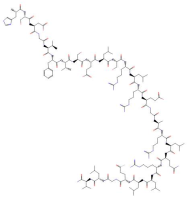	756.1704	756.1640	-8.44
MATCH	31.0	756.1704	756.1640	-8.44	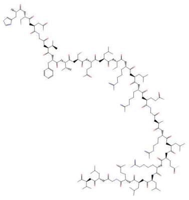	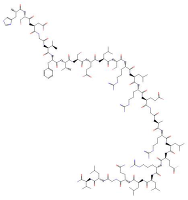	756.1704	756.1640	-8.44
MATCH	8.0	755.9225	755.9180	-6.04	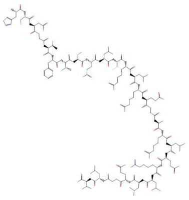	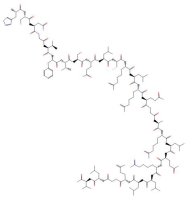	755.9225	755.9180	-6.04
MATCH	8.0	755.9225	755.9180	-6.04	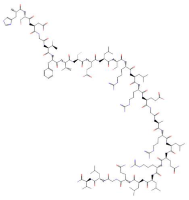	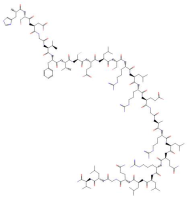	755.9225	755.9180	-6.04
MATCH	8.0	755.9225	755.9180	-6.04	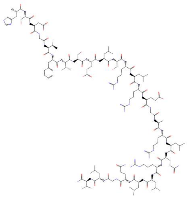	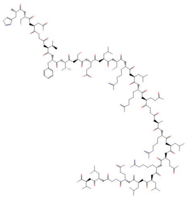	755.9225	755.9180	-6.04
MATCH	41.1	731.3995	731.3969	-3.60	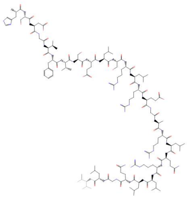	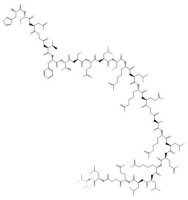	731.3995	731.3969	-3.60
MATCH	34.4	724.3975	724.3981	0.90	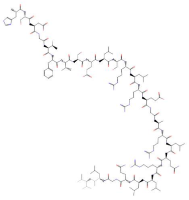	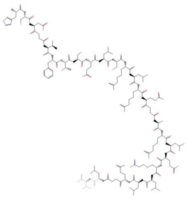	724.3975	724.3981	0.90
MISMATCH	-3.7	698.8734	698.8692	-5.92	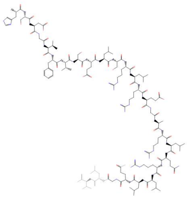	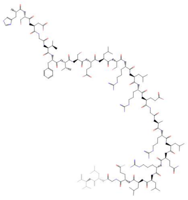	698.8734	698.8692	-5.92

Metabolite: Substrate

Type	score	sub. m/z observed	sub. m/z calculated	sub ppm			met. m/z observed	met. m/z calculated	met. ppm
MISMATCH	-3.7	698.8734	698.8692	-5.92	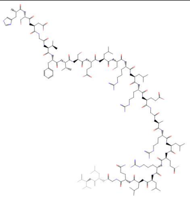	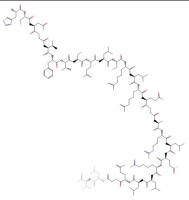	698.8734	698.8692	-5.92
MATCH	163.6	608.5403	608.5379	-3.81	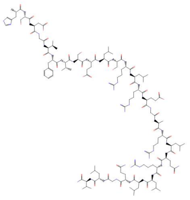	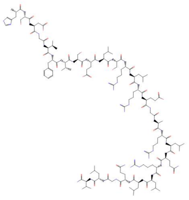	608.5403	608.5379	-3.81
MATCH	160.1	608.5397	608.5379	-2.85	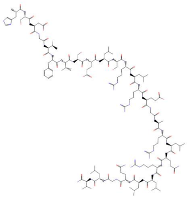	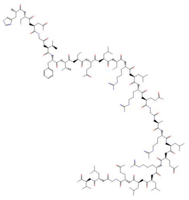	608.5397	608.5379	-2.85
MISMATCH	-16.9	604.9382	604.9358	-3.88	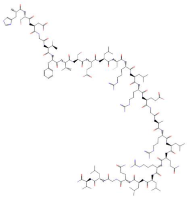	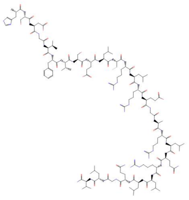	604.9382	604.9358	-3.88
MISMATCH	-11.1	599.5338	599.5337	-0.25	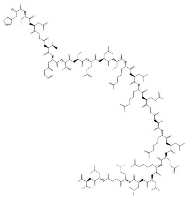	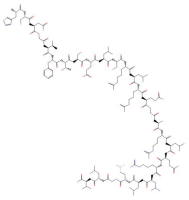	599.5338	599.5337	-0.25
MISMATCH	-11.1	599.5338	599.5337	-0.25	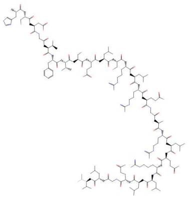	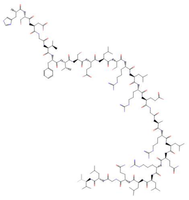	599.5338	599.5337	-0.25
MISMATCH	-11.1	599.5338	599.5337	-0.25	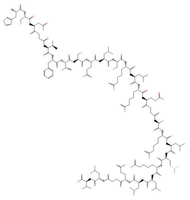	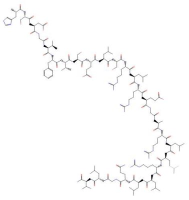	599.5338	599.5337	-0.25
MISMATCH	-8.6	585.3214	585.3190	-4.17	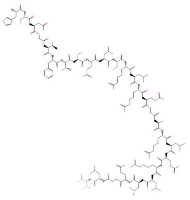	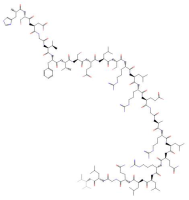	585.3214	585.3190	-4.17
MISMATCH	-3.9	581.9200	581.9136	-10.9	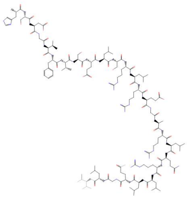	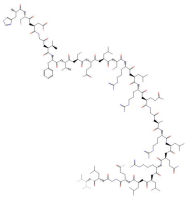	581.9200	581.9136	-10.9

Metabolite: Substrate

Type	score	sub. m/z observed	sub. m/z calculated	sub ppm			met. m/z observed	met. m/z calculated	met. ppm
MISMATCH	-3.9	581.9200	581.9136	-10.9	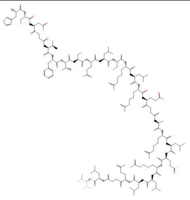	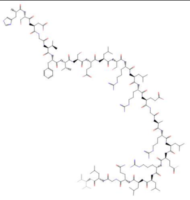	581.9200	581.9136	-10.9
MISMATCH	-32.1	579.7231	579.7200	-5.46	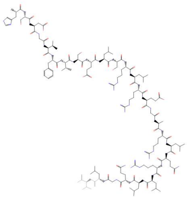	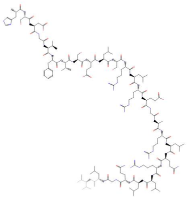	579.7231	579.7200	-5.46
MATCH	6.7	498.1958	498.1943	-3.04	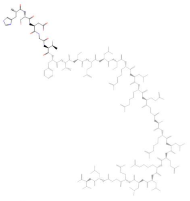	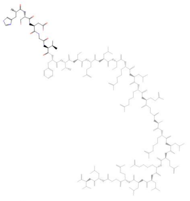	498.1958	498.1943	-3.04
MATCH	15.0	480.1849	480.1837	-2.34	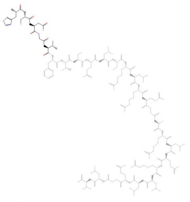	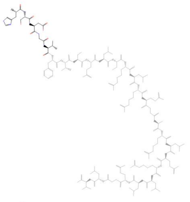	480.1849	480.1837	-2.34
MATCH	15.0	480.1849	480.1837	-2.34	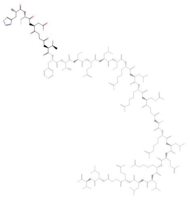	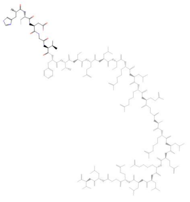	480.1849	480.1837	-2.34
MATCH	4.2	381.2255	381.2245	-2.66	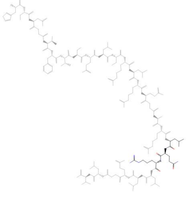	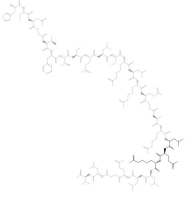	381.2255	381.2245	-2.66
MATCH	4.2	381.2255	381.2245	-2.66	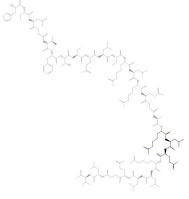	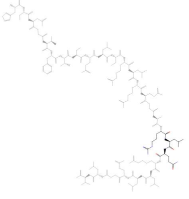	381.2255	381.2245	-2.66
MATCH	4.2	381.2255	381.2245	-2.66	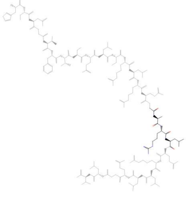	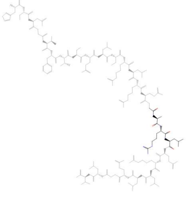	381.2255	381.2245	-2.66
MATCH	4.2	381.2255	381.2245	-2.66	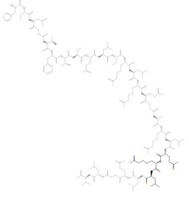	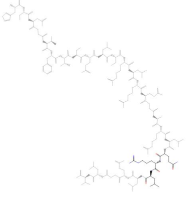	381.2255	381.2245	-2.66

Metabolite: Substrate

Type	score	sub. m/z observed	sub. m/z calculated	sub ppm			met. m/z observed	met. m/z calculated	met. ppm
MATCH	8.1	268.1410	268.1404	-2.03	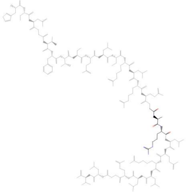	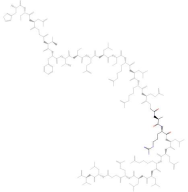	268.1410	268.1404	-2.03
MATCH	8.1	268.1410	268.1404	-2.03	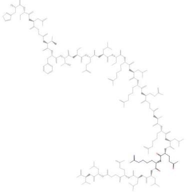	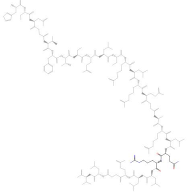	268.1410	268.1404	-2.03
MATCH	8.1	253.1657	253.1659	0.77	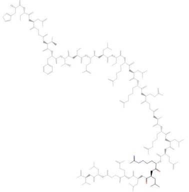	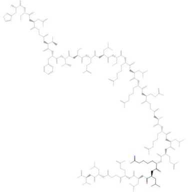	253.1657	253.1659	0.77
MATCH	8.1	253.1657	253.1659	0.77	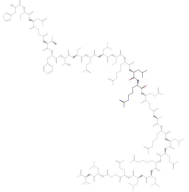	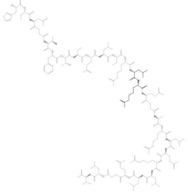	253.1657	253.1659	0.77
MATCH	8.1	253.1657	253.1659	0.77	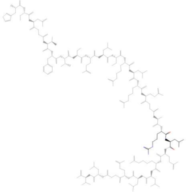	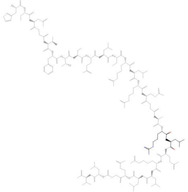	253.1657	253.1659	0.77
MATCH	8.1	253.1657	253.1659	0.77	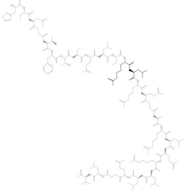	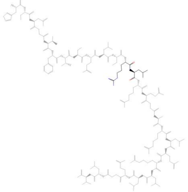	253.1657	253.1659	0.77
MATCH	9.7	225.1711	225.1710	-0.28	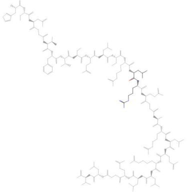	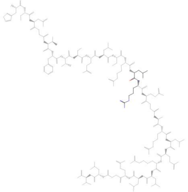	225.1711	225.1710	-0.28
MATCH	9.7	225.1711	225.1710	-0.28	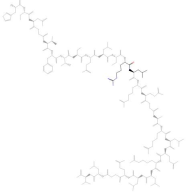	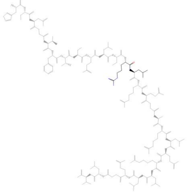	225.1711	225.1710	-0.28
MATCH	9.7	225.1711	225.1710	-0.28	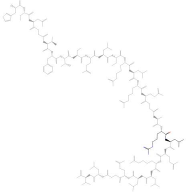	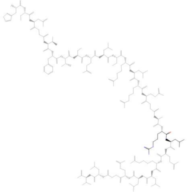	225.1711	225.1710	-0.28

Metabolite: Substrate

Type	score	sub. m/z observed	sub. m/z calculated	sub ppm			met. m/z observed	met. m/z calculated	met. ppm
MATCH	9.7	225.1711	225.1710	-0.28	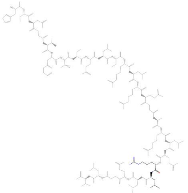	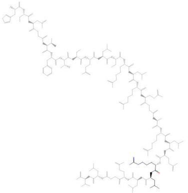	225.1711	225.1710	-0.28
MATCH	21.8	225.0990	225.0982	-3.57	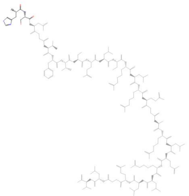	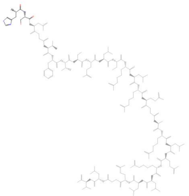	225.0990	225.0982	-3.57
MATCH	7.8	217.0827	217.0819	-3.69	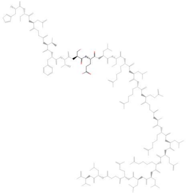	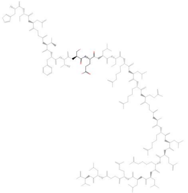	217.0827	217.0819	-3.69
MATCH	7.8	217.0827	217.0819	-3.69	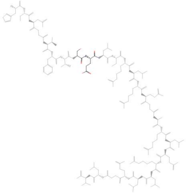	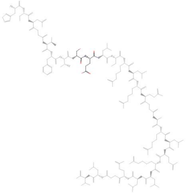	217.0827	217.0819	-3.69

MS (+) FT

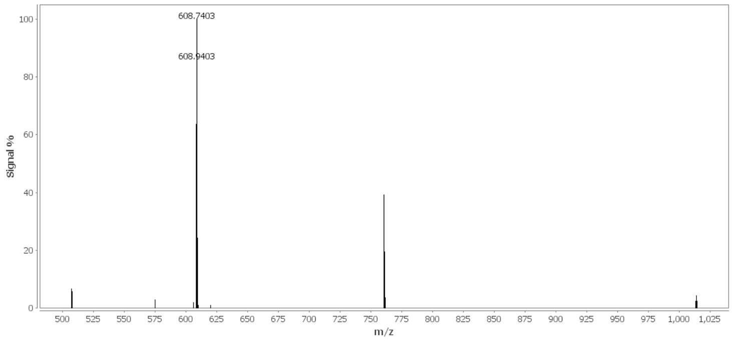

MS (+) FT

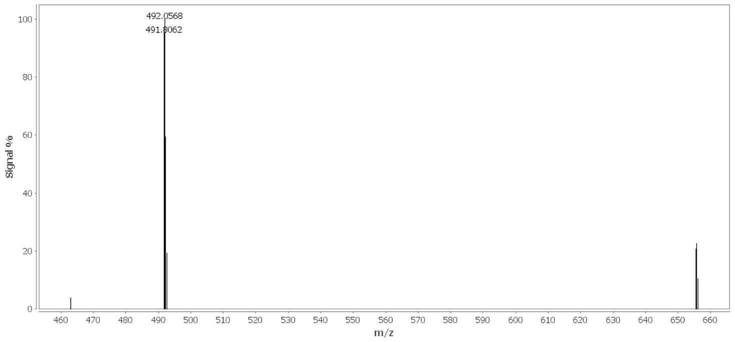

MS2 (+) FT activ = HCD:ce =

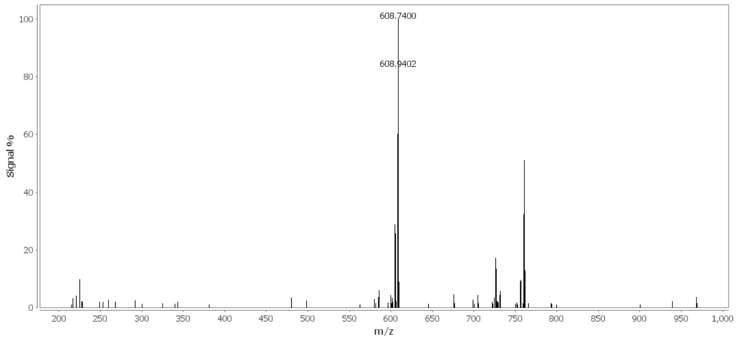

MS2 (+) FT activ = HCD:ce =

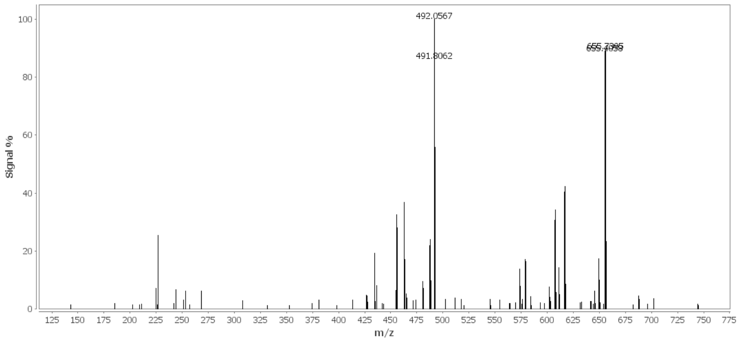

Metabolite: M2 -1074 RT=2.06

Type	score	sub. m/z observed	sub. m/z calculated	sub ppm	met. m/z observed	met. m/z calculated	met. ppm

Metabolite: M2 -1074 RT=2.06

Type	score	sub. m/z observed	sub. m/z calculated	sub ppm			met. m/z observed	met. m/z calculated	met. ppm
MATCH	158.9	608.5403	608.5379	-3.81	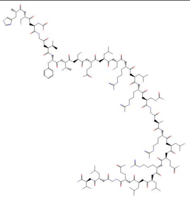	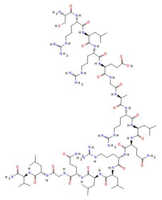	491.8062	491.8052	-2.12
MATCH	158.9	608.5403	608.5379	-3.81	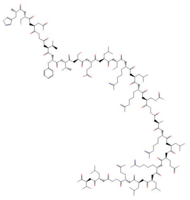	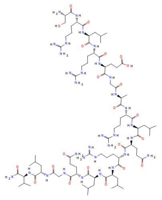	491.8062	491.8052	-2.12
						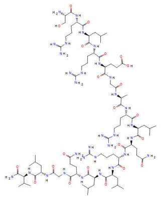	491.8062	491.8052	-2.12
MATCH	84.2	608.5403	608.5379	-3.81	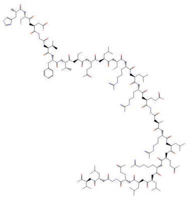	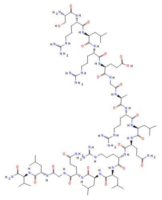	655.4064	655.4045	-2.94
MATCH	84.2	608.5403	608.5379	-3.81	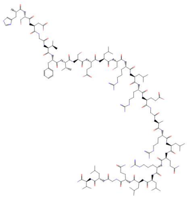	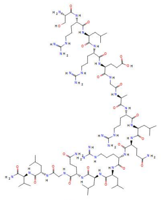	655.4064	655.4045	-2.94
						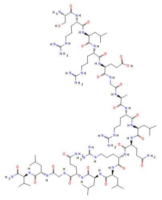	655.4064	655.4045	-2.94
MATCH	120.0	760.4255	760.4206	-6.45	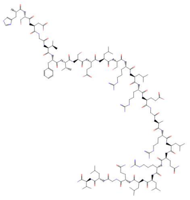	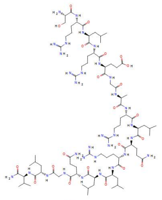	491.8062	491.8052	-2.12
MATCH	120.0	760.4255	760.4206	-6.45	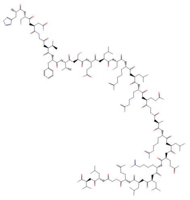	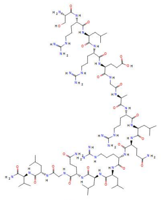	491.8062	491.8052	-2.12
						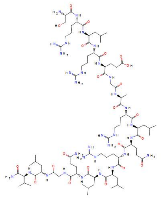	491.8062	491.8052	-2.12

Metabolite: M2 -1074 RT=2.06

Type	score	sub. m/z observed	sub. m/z calculated	sub ppm			met. m/z observed	met. m/z calculated	met. ppm
MATCH	45.2	760.4255	760.4206	-6.45	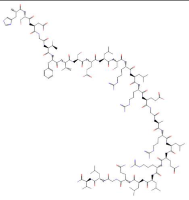	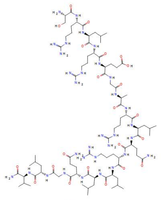	655.4064	655.4045	-2.94
MATCH	45.2	760.4255	760.4206	-6.45	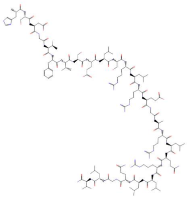	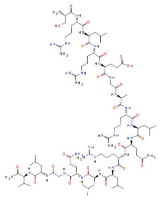	655.4064	655.4045	-2.94
						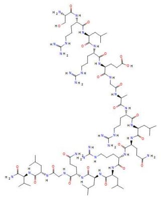	655.4064	655.4045	-2.94
MATCH	97.8	1013.5654	1013.5584	-6.94	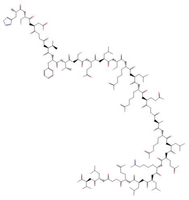	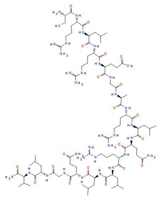	491.8062	491.8052	-2.12
MATCH	97.8	1013.5654	1013.5584	-6.94	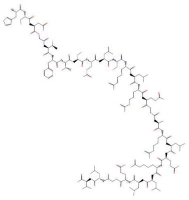	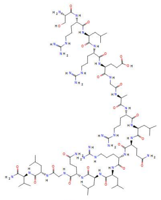	491.8062	491.8052	-2.12
						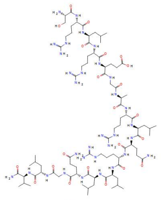	491.8062	491.8052	-2.12
MATCH	23.0	1013.5654	1013.5584	-6.94	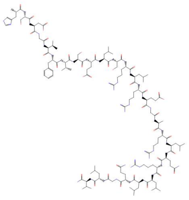	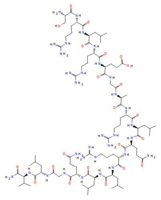	655.4064	655.4045	-2.94
MATCH	23.0	1013.5654	1013.5584	-6.94	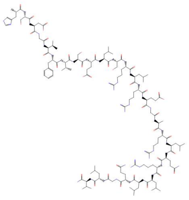	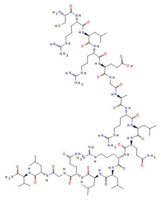	655.4064	655.4045	-2.94
						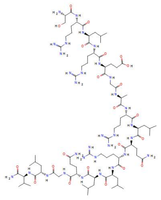	655.4064	655.4045	-2.94

Metabolite: M2 -1074 RT=2.06

Type	score	sub. m/z observed	sub. m/z calculated	sub ppm			met. m/z observed	met. m/z calculated	met. ppm
MATCH	9.7	225.1711	225.1710	-0.28	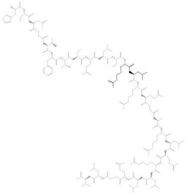	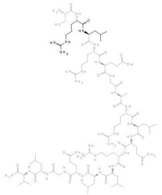	225.1710	225.1710	-0.10
MATCH	9.7	225.1711	225.1710	-0.28	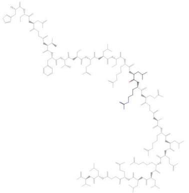	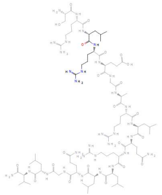	225.1710	225.1710	-0.10
MATCH	9.7	225.1711	225.1710	-0.28	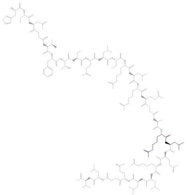	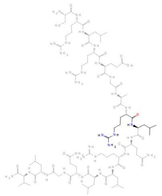	225.1710	225.1710	-0.10
MATCH	9.7	225.1711	225.1710	-0.28	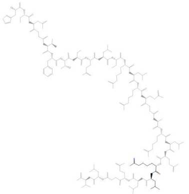	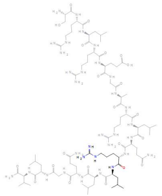	225.1710	225.1710	-0.10
MATCH	8.1	253.1657	253.1659	0.77	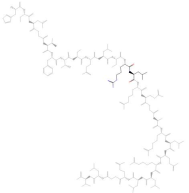	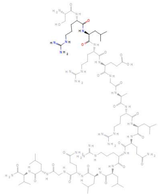	253.1656	253.1659	1.32
MATCH	8.1	253.1657	253.1659	0.77	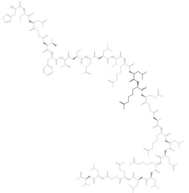	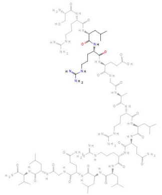	253.1656	253.1659	1.32
MATCH	8.1	253.1657	253.1659	0.77	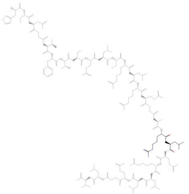	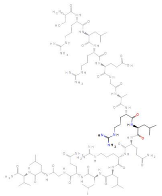	253.1656	253.1659	1.32
MATCH	8.1	253.1657	253.1659	0.77	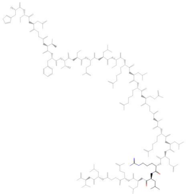	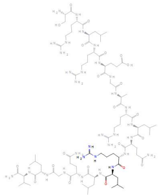	253.1656	253.1659	1.32
MATCH	8.1	268.1410	268.1404	-2.03	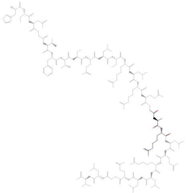	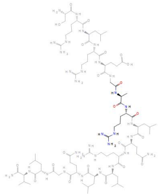	268.1403	268.1404	0.29

Metabolite: M2 -1074 RT=2.06

Type	score	sub. m/z observed	sub. m/z calculated	sub ppm			met. m/z observed	met. m/z calculated	met. ppm
MATCH	8.1	268.1410	268.1404	-2.03	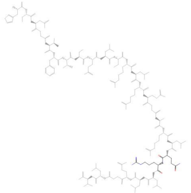	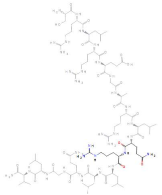	268.1403	268.1404	0.29
MATCH	4.2	381.2255	381.2245	-2.66	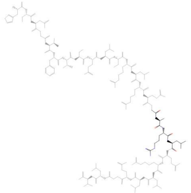	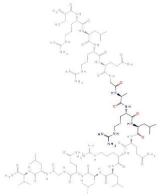	381.2330	381.2245	-22.4
MATCH	4.2	381.2255	381.2245	-2.66	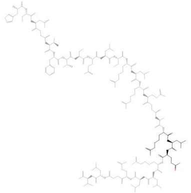	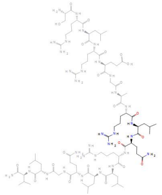	381.2330	381.2245	-22.4
MATCH	4.2	381.2255	381.2245	-2.66	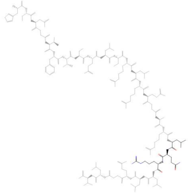	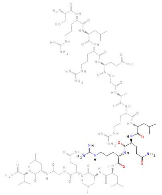	381.2330	381.2245	-22.4
MATCH	4.2	381.2255	381.2245	-2.66	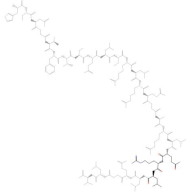	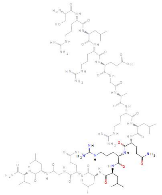	381.2330	381.2245	-22.4
MATCH	146.4	608.5397	608.5379	-2.85	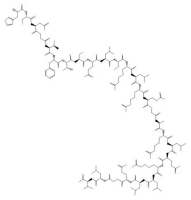	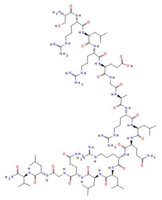	491.8062	491.8052	-2.04
MATCH	146.4	608.5397	608.5379	-2.85	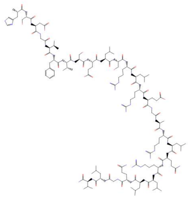	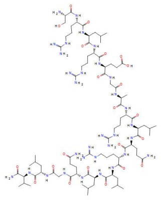	491.8062	491.8052	-2.04
MATCH	149.0	608.5397	608.5379	-2.85	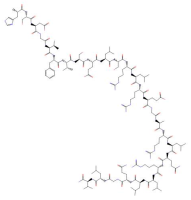	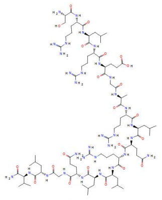	655.4058	655.4045	-1.98
MATCH	149.0	608.5397	608.5379	-2.85	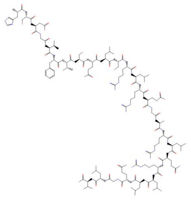	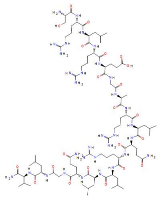	655.4058	655.4045	-1.98

Metabolite: M2 -1074 RT=2.06

Type	score	sub. m/z observed	sub. m/z calculated	sub ppm			met. m/z observed	met. m/z calculated	met. ppm
MATCH	34.4	724.3975	724.3981	0.90	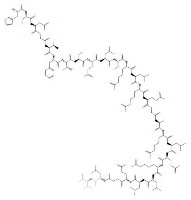	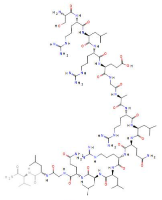	455.7842	455.7827	-3.27
MATCH	41.1	731.3995	731.3969	-3.60	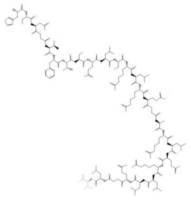	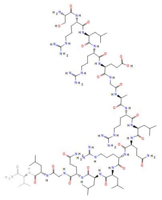	462.7826	462.7814	-2.47
MATCH	31.0	756.1704	756.1640	-8.44	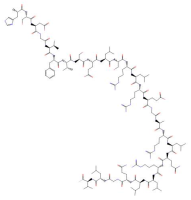	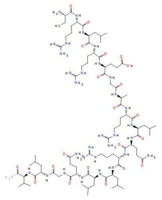	487.5499	487.5485	-2.85
MATCH	31.0	756.1704	756.1640	-8.44	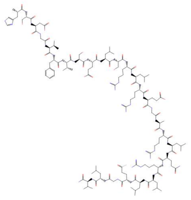	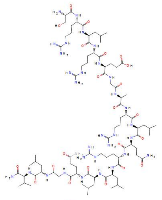	487.5499	487.5485	-2.85
MATCH	31.0	756.1704	756.1640	-8.44	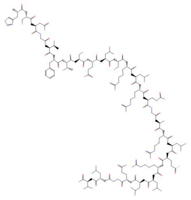	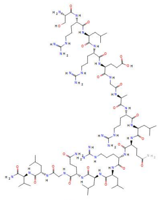	487.5499	487.5485	-2.85
MATCH	118.8	760.4252	760.4206	-6.06	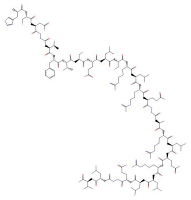	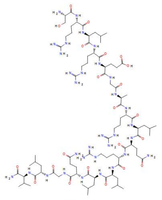	491.8062	491.8052	-2.04
MATCH	118.8	760.4252	760.4206	-6.06	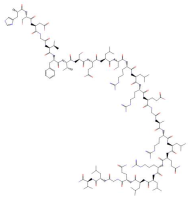	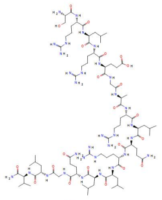	491.8062	491.8052	-2.04
MATCH	121.4	760.4252	760.4206	-6.06	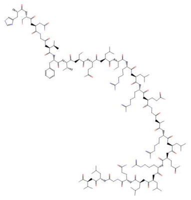	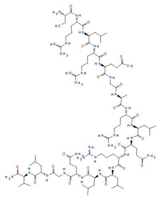	655.4058	655.4045	-1.98
MATCH	121.4	760.4252	760.4206	-6.06	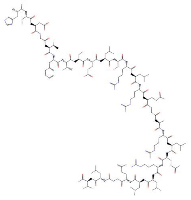	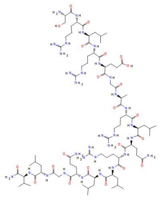	655.4058	655.4045	-1.98

Metabolite: M2 -1074 RT=2.06

Type	score	sub. m/z observed	sub. m/z calculated	sub ppm		met. m/z observed	met. m/z calculated	met. ppm
MISMATCH	-32.1	579.7231	579.7200	-5.46	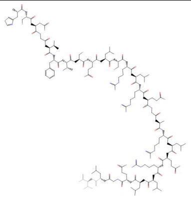	607.3768	607.3768	0.00
MISMATCH	-11.0	581.9200	581.9136	-10.9	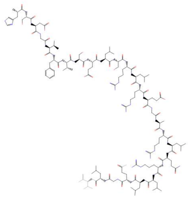	611.0334	611.0334	0.00
MISMATCH	-43.9	585.3214	585.3190	-4.17	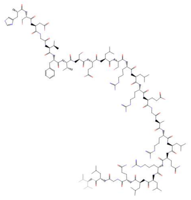	616.7077	616.7077	0.00
MISMATCH	-11.1	599.5338	599.5337	-0.25	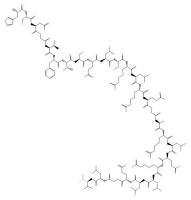	480.5501	480.5501	0.00
MISMATCH	-16.9	604.9382	604.9358	-3.88	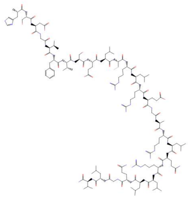	649.4034	649.4034	0.00
MISMATCH	-15.4	698.8734	698.8692	-5.92	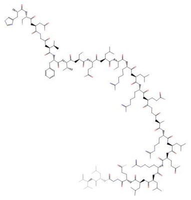	573.3389	573.3389	0.00
MISMATCH	-32.5	724.3975	724.3981	0.90	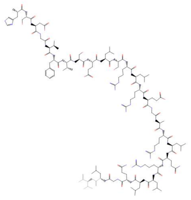	607.3768	607.3768	0.00
MISMATCH	-44.5	731.3995	731.3969	-3.60	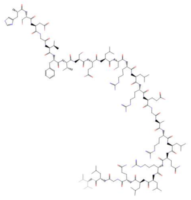	616.7077	616.7077	0.00
MISMATCH	-8.3	755.9225	755.9180	-6.04	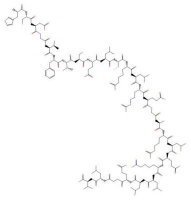	649.4034	649.4034	0.00

Metabolite: M2 -1074 RT=2.06

Type	score	sub. m/z observed	sub. m/z calculated	sub ppm		met. m/z observed	met. m/z calculated	met. ppm
MISMATCH	-26.5	756.1704	756.1640	-8.44	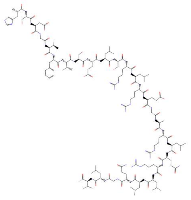	649.7327	649.7327	0.00
MET_MATCH					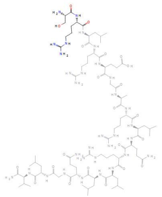	244.1402	244.1404	1.01
MET_MATCH					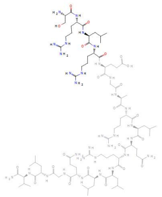	257.1663	257.1664	0.55
MET_MATCH					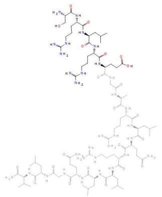	307.6911	307.6903	-2.68
MET_MATCH					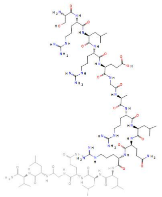	331.6994	331.6984	-3.23
MET_MATCH					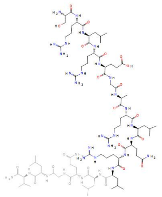	352.9683	352.9707	6.67
MET_MATCH					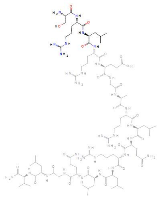	374.2530	374.2510	-5.38
MET_MATCH					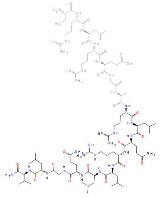	398.2525	398.2594	17.40
MET_MATCH					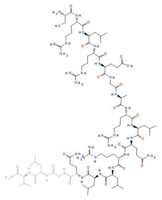	413.2565	413.2563	-0.44

Metabolite: M2 -1074 RT=2.06

Type	score	sub. m/z observed	sub. m/z calculated	sub ppm		met. m/z observed	met. m/z calculated	met. ppm
MET_MATCH					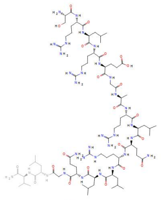	434.5115	434.5104	-2.54
MET_MATCH					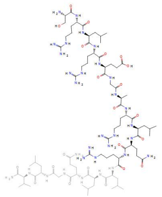	441.9279	441.9287	1.88
MET_MATCH					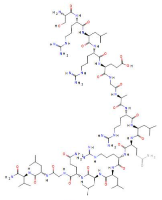	480.5501	480.5498	-0.58
MET_MATCH					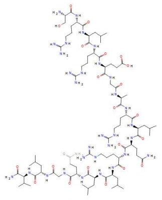	480.5501	480.5498	-0.58
MET_MATCH					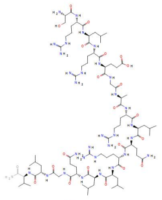	480.5501	480.5498	-0.58
MET_MATCH					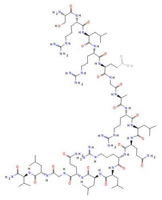	480.8036	480.8077	8.45
MET_MATCH					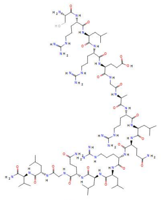	487.8002	487.8064	12.78
MET_MATCH					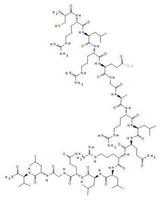	487.8002	487.8064	12.78
MET_MATCH					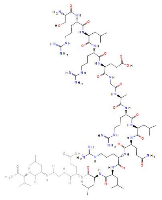	517.3200	517.3181	-3.66

Metabolite: M2 -1074 RT=2.06

Type	score	sub. m/z observed	sub. m/z calculated	sub ppm		met. m/z observed	met. m/z calculated	met. ppm
MET_MATCH					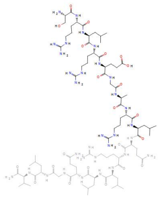	520.3105	520.3096	-1.65
MET_MATCH					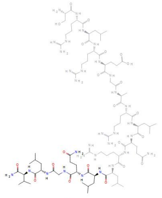	554.3307	554.3297	-1.86
MET_MATCH					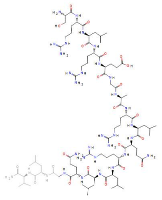	569.6840	569.6798	-7.39
MET_MATCH					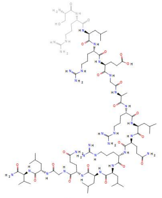	574.3503	574.3601	16.95
MET_MATCH					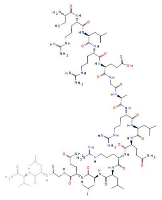	579.0116	579.0114	-0.33
MET_MATCH					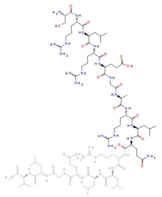	584.3431	584.3389	-7.12
MET_MATCH					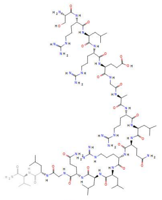	607.3768	607.3745	-3.79
MET_MATCH					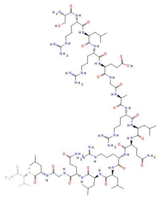	616.7077	616.7061	-2.49
MET_MATCH					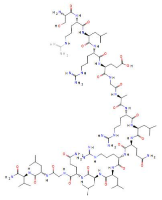	641.3985	641.3972	-2.01

Metabolite: M2 -1074 RT=2.06

Type	score	sub. m/z observed	sub. m/z calculated	sub ppm		met. m/z observed	met. m/z calculated	met. ppm
MET_MATCH					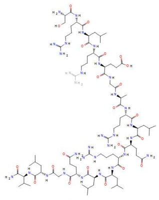	641.3985	641.3972	-2.01
MET_MATCH					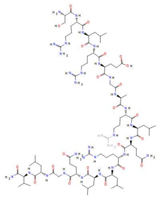	641.3985	641.3972	-2.01
MET_MATCH					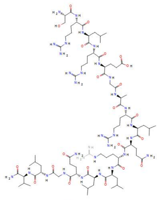	641.3985	641.3972	-2.01
MET_MATCH					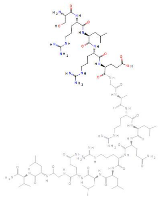	644.3931	644.3838	-14.3
MET_MATCH					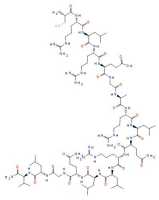	649.4034	649.4009	-3.87
MET_MATCH					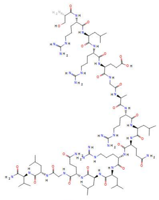	649.7327	649.7289	-5.75
MET_MATCH					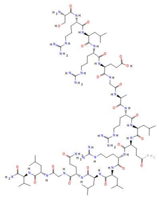	649.7327	649.7289	-5.75
MET_MATCH					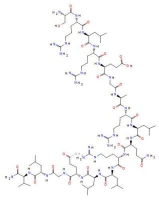	649.7327	649.7289	-5.75
MET_MATCH					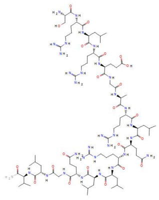	649.7327	649.7289	-5.75

Metabolite: M2 -1074 RT=2.06

Type	score	sub. m/z observed	sub. m/z calculated	sub ppm			met. m/z observed	met. m/z calculated	met. ppm
MET_MATCH						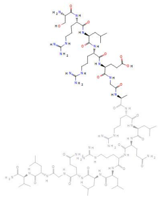	744.4512	744.4475	-4.97

MS (+) FT

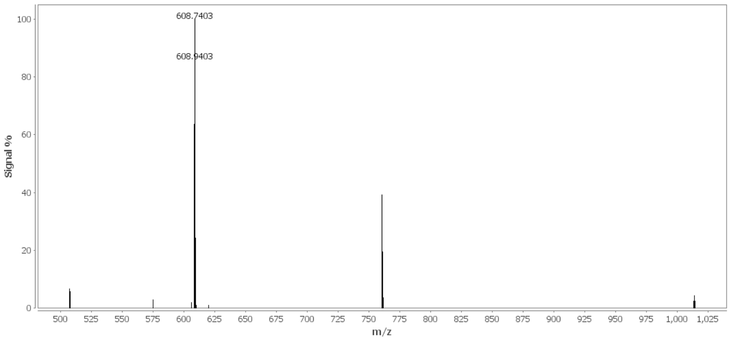

MS (+) FT

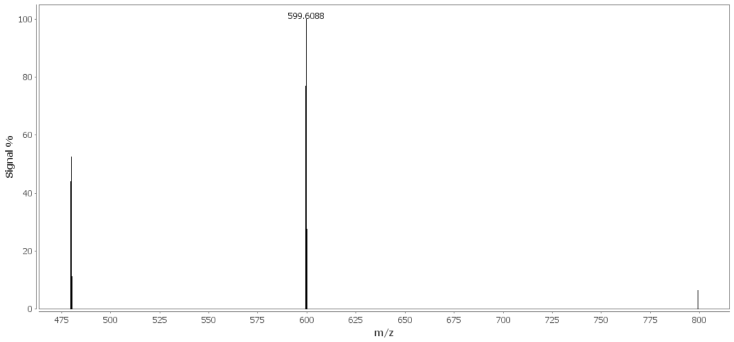

MS2 (+) FT activ = HCD:ce =

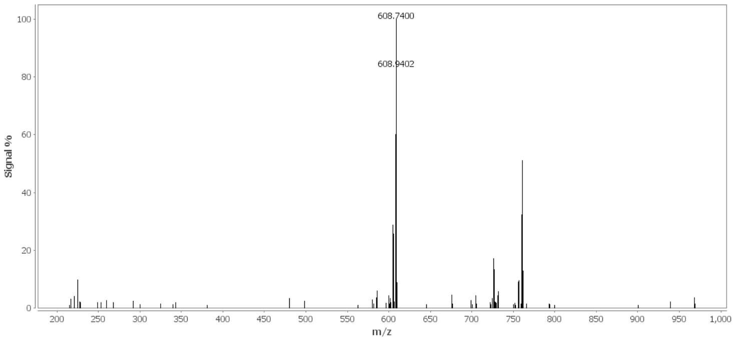

MS2 (+) FT activ = HCD:ce =

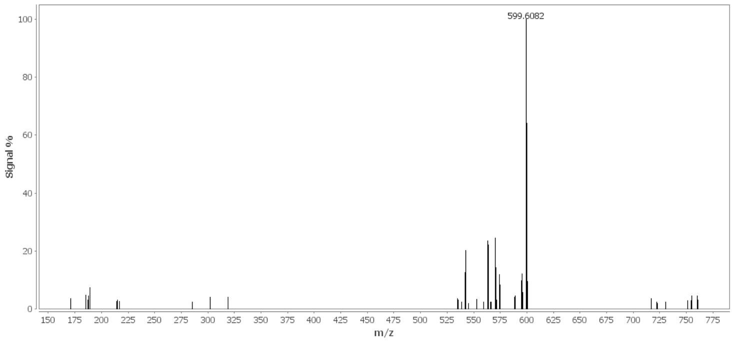

Metabolite: M5 -644 RT=2.26

Type	score	sub. m/z observed	sub. m/z calculated	sub ppm			met. m/z observed	met. m/z calculated	met. ppm
MATCH	107.6	608.5403	608.5379	-3.81	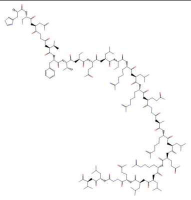	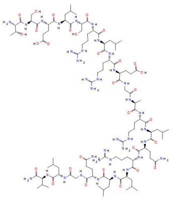	479.6884	479.6869	-3.17
MATCH	107.6	608.5403	608.5379	-3.81	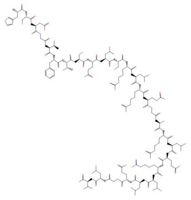	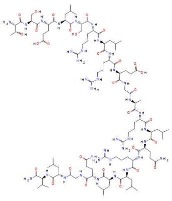	479.6884	479.6869	-3.17
						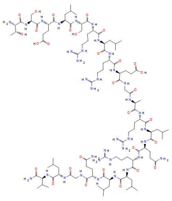	479.6884	479.6869	-3.17

Metabolite: M5 -644 RT=2.26

Type	score	sub. m/z observed	sub. m/z calculated	sub ppm			met. m/z observed	met. m/z calculated	met. ppm
MATCH	140.4	608.5403	608.5379	-3.81	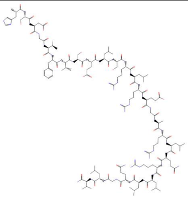	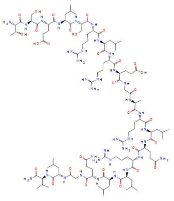	599.3583	599.3568	-2.63
MATCH	140.4	608.5403	608.5379	-3.81	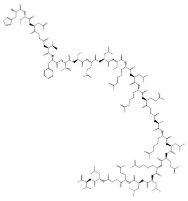	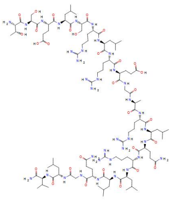	599.3583	599.3568	-2.63
						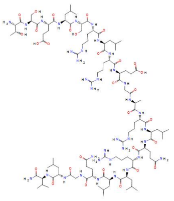	599.3583	599.3568	-2.63
MATCH	68.6	760.4255	760.4206	-6.45	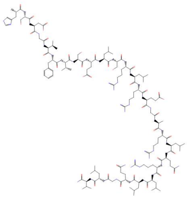	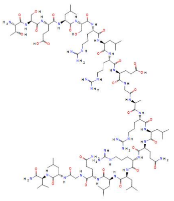	479.6884	479.6869	-3.17
MATCH	68.6	760.4255	760.4206	-6.45	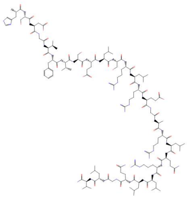	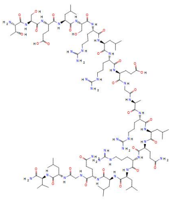	479.6884	479.6869	-3.17
						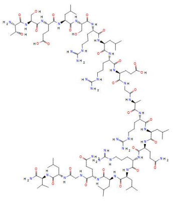	479.6884	479.6869	-3.17
MATCH	101.5	760.4255	760.4206	-6.45	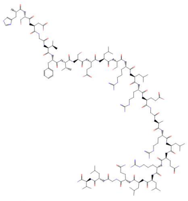	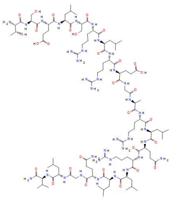	599.3583	599.3568	-2.63
MATCH	101.5	760.4255	760.4206	-6.45	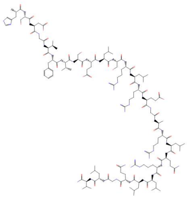	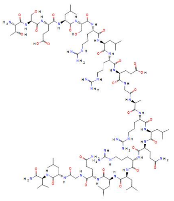	599.3583	599.3568	-2.63
						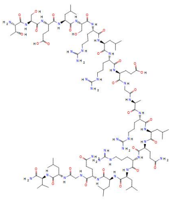	599.3583	599.3568	-2.63

Metabolite: M5 -644 RT=2.26

Type	score	sub. m/z observed	sub. m/z calculated	sub ppm			met. m/z observed	met. m/z calculated	met. ppm
MATCH	46.4	1013.5654	1013.5584	-6.94	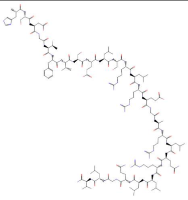	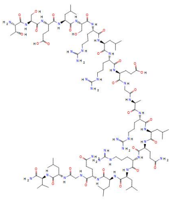	479.6884	479.6869	-3.17
MATCH	46.4	1013.5654	1013.5584	-6.94	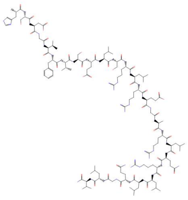	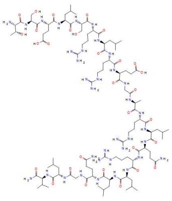	479.6884	479.6869	-3.17
						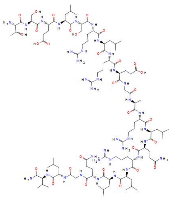	479.6884	479.6869	-3.17
MATCH	79.3	1013.5654	1013.5584	-6.94	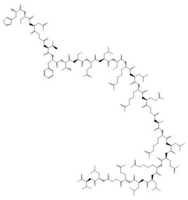	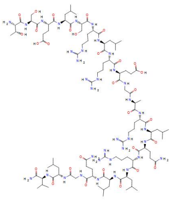	599.3583	599.3568	-2.63
MATCH	79.3	1013.5654	1013.5584	-6.94	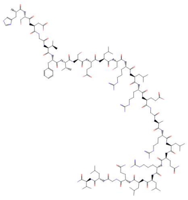	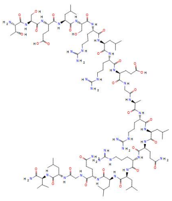	599.3583	599.3568	-2.63
						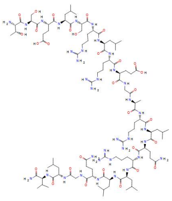	599.3583	599.3568	-2.63
MATCH	5.8	217.0827	217.0819	-3.69	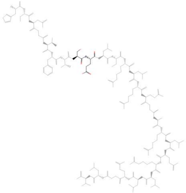	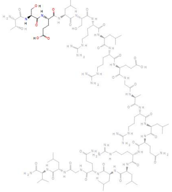	217.0811	217.0819	3.46
MATCH	5.8	217.0827	217.0819	-3.69	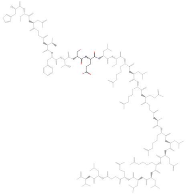	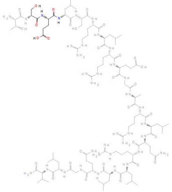	217.0811	217.0819	3.46
MATCH	129.1	608.5397	608.5379	-2.85	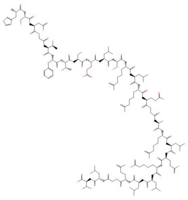	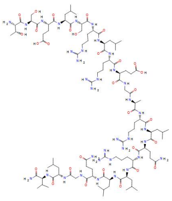	599.3574	599.3568	-1.01

Metabolite: M5 -644 RT=2.26

Type	score	sub. m/z observed	sub. m/z calculated	sub ppm			met. m/z observed	met. m/z calculated	met. ppm
						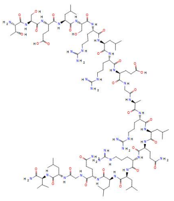	599.3574	599.3568	-1.01
MATCH	25.5	724.3975	724.3981	0.90	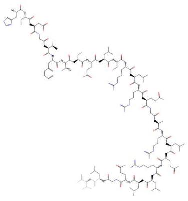	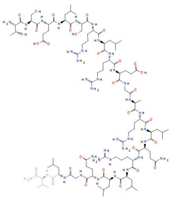	563.3349	563.3343	-1.17
MATCH	21.7	731.3995	731.3969	-3.60	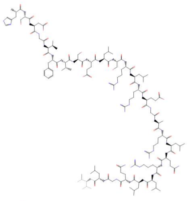	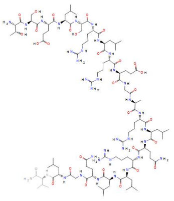	570.3338	570.3330	-1.39
MATCH	8.0	755.9225	755.9180	-6.04	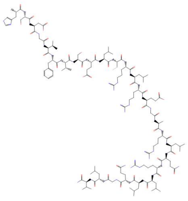	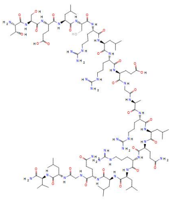	594.8550	594.8541	-1.46
MATCH	8.0	755.9225	755.9180	-6.04	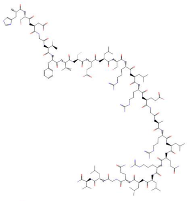	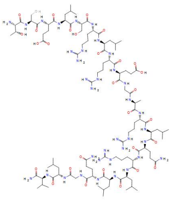	594.8550	594.8541	-1.46
MATCH	8.0	755.9225	755.9180	-6.04	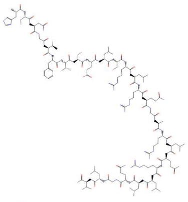	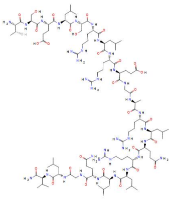	594.8550	594.8541	-1.46
MATCH	18.8	756.1704	756.1640	-8.44	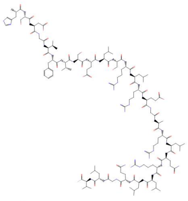	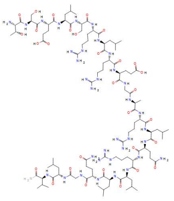	595.1026	595.1001	-4.17
MATCH	18.8	756.1704	756.1640	-8.44	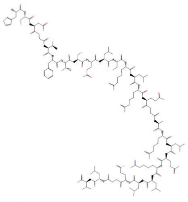	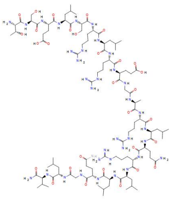	595.1026	595.1001	-4.17
MATCH	18.8	756.1704	756.1640	-8.44	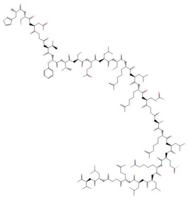	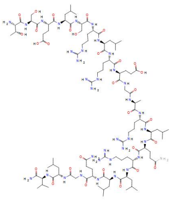	595.1026	595.1001	-4.17

Metabolite: M5 -644 RT=2.26

Type	score	sub. m/z observed	sub. m/z calculated	sub ppm			met. m/z observed	met. m/z calculated	met. ppm
MATCH	101.5	760.4252	760.4206	-6.06	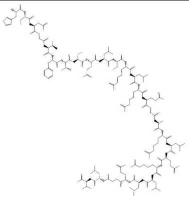	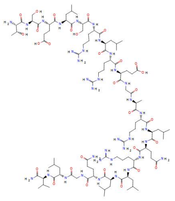	599.3574	599.3568	-1.01
						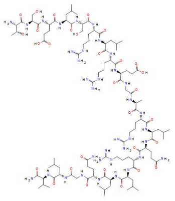	599.3574	599.3568	-1.01
MISMATCH	-3.9	581.9200	581.9136	-10.9	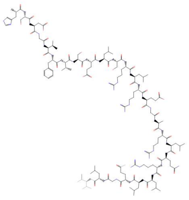		566.0848	566.0848	0.00
MISMATCH	-3.7	698.8734	698.8692	-5.92	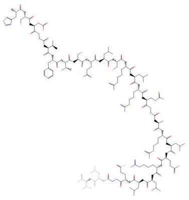		716.7349	716.7349	0.00
MET_MATCH						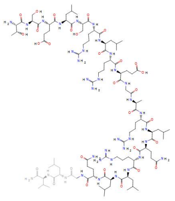	535.0685	535.0633	-9.74
MET_MATCH						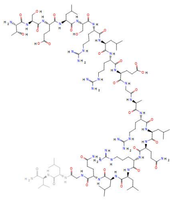	542.0632	542.0620	-2.17
MET_MATCH						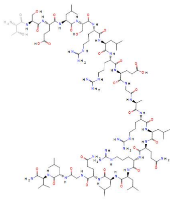	574.0967	574.0948	-3.32
MET_MATCH						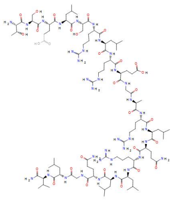	588.3521	588.3593	12.31
MET_MATCH						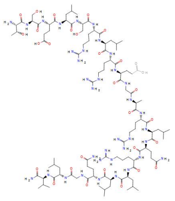	588.3521	588.3593	12.31

Metabolite: M5 -644 RT=2.26

Type	score	sub. m/z observed	sub. m/z calculated	sub ppm		met. m/z observed	met. m/z calculated	met. ppm
MET_MATCH					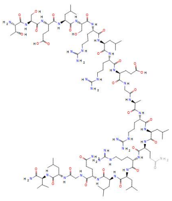	588.6025	588.6053	4.69
MET_MATCH					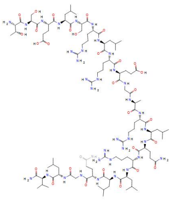	588.6025	588.6053	4.69
MET_MATCH					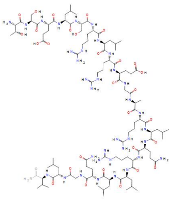	588.6025	588.6053	4.69
MET_MATCH					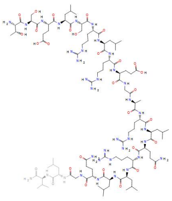	722.4171	722.4136	-4.91
MET_MATCH					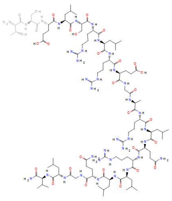	730.4371	730.4378	0.95

MS (+) FT

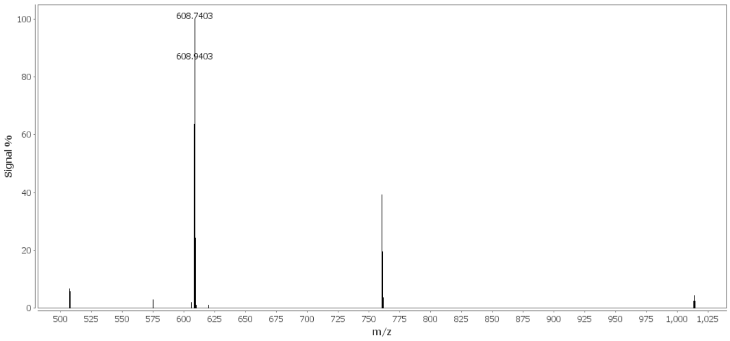

MS (+) FT

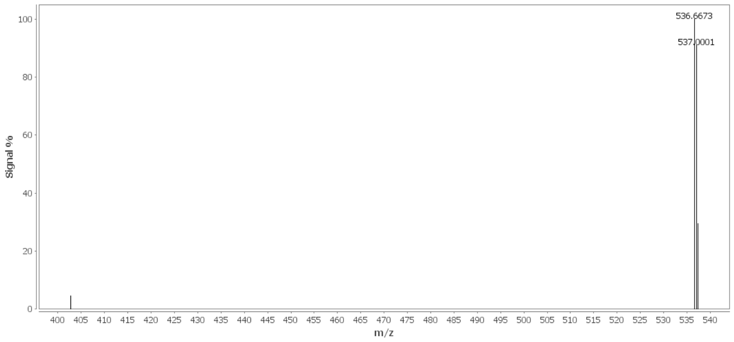

MS2 (+) FT activ = HCD:ce =

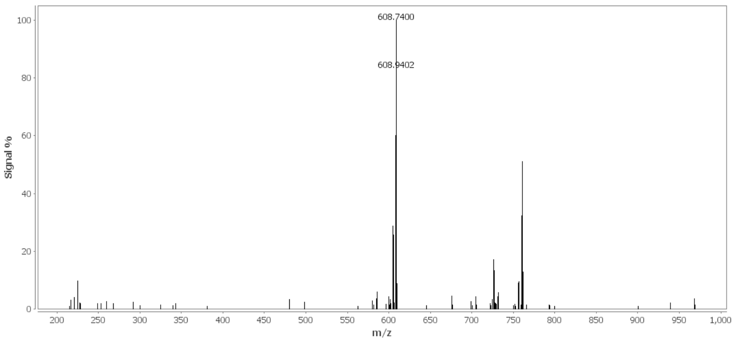

MS2 (+) FT activ = HCD:ce =

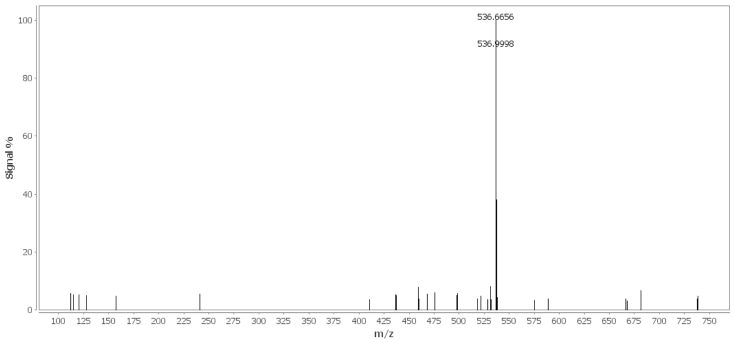

Metabolite: M4 -1431 RT=2.12

Type	score	sub. m/z observed	sub. m/z calculated	sub ppm			met. m/z observed	met. m/z calculated	met. ppm
MATCH	68.0	608.5403	608.5379	-3.81	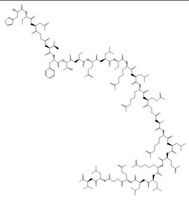	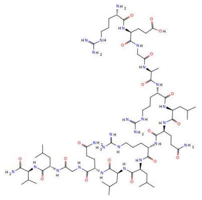	402.7512	402.7509	-0.76
MATCH	68.0	608.5403	608.5379	-3.81	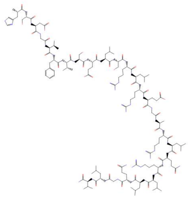	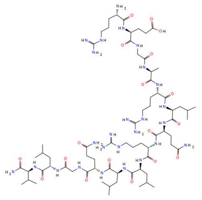	402.7512	402.7509	-0.76
						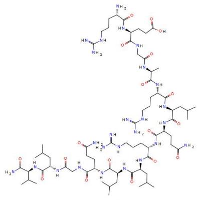	402.7512	402.7509	-0.76
MATCH	163.6	608.5403	608.5379	-3.81	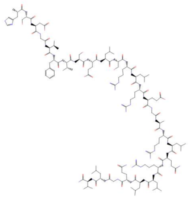	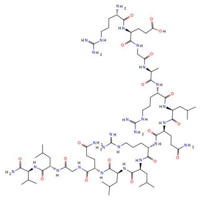	536.6673	536.6654	-3.50
MATCH	163.6	608.5403	608.5379	-3.81	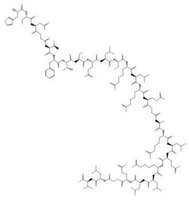	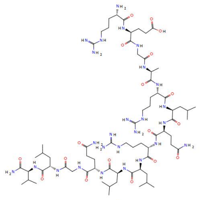	536.6673	536.6654	-3.50
						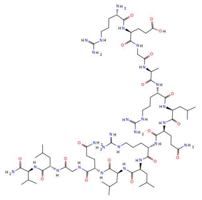	536.6673	536.6654	-3.50
MATCH	29.1	760.4255	760.4206	-6.45	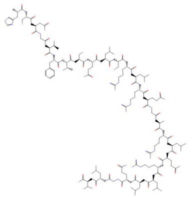	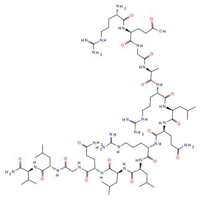	402.7512	402.7509	-0.76
MATCH	29.1	760.4255	760.4206	-6.45	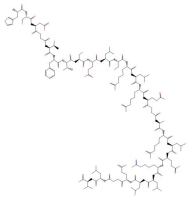	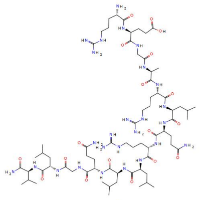	402.7512	402.7509	-0.76
						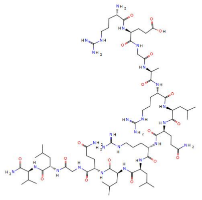	402.7512	402.7509	-0.76

Metabolite: M4 -1431 RT=2.12

Type	score	sub. m/z observed	sub. m/z calculated	sub ppm			met. m/z observed	met. m/z calculated	met. ppm
MATCH	124.6	760.4255	760.4206	-6.45	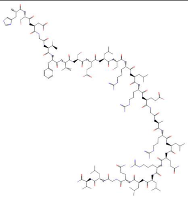	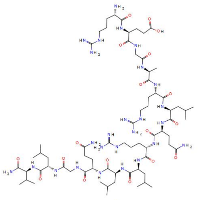	536.6673	536.6654	-3.50
MATCH	124.6	760.4255	760.4206	-6.45	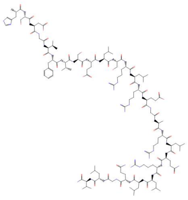	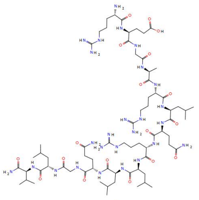	536.6673	536.6654	-3.50
						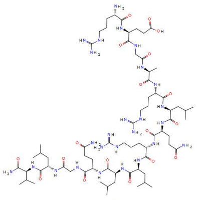	536.6673	536.6654	-3.50
MATCH	6.9	1013.5654	1013.5584	-6.94	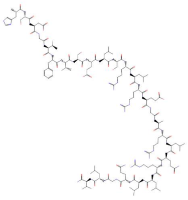	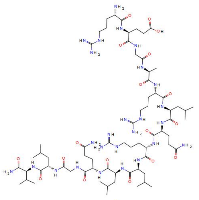	402.7512	402.7509	-0.76
MATCH	6.9	1013.5654	1013.5584	-6.94	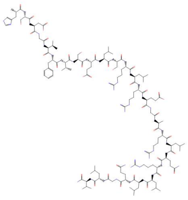	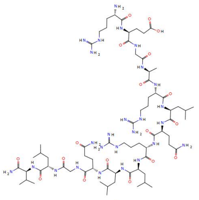	402.7512	402.7509	-0.76
						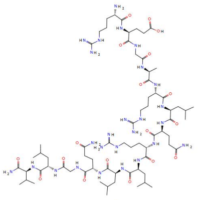	402.7512	402.7509	-0.76
MATCH	102.4	1013.5654	1013.5584	-6.94	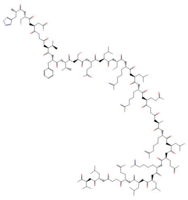	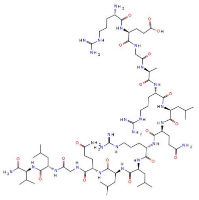	536.6673	536.6654	-3.50
MATCH	102.4	1013.5654	1013.5584	-6.94	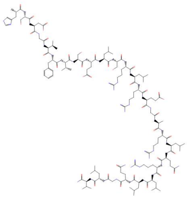	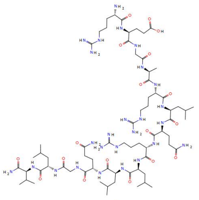	536.6673	536.6654	-3.50
						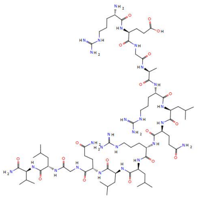	536.6673	536.6654	-3.50

Metabolite: M4 -1431 RT=2.12

Type	score	sub. m/z observed	sub. m/z calculated	sub ppm			met. m/z observed	met. m/z calculated	met. ppm
MATCH	160.1	608.5397	608.5379	-2.85	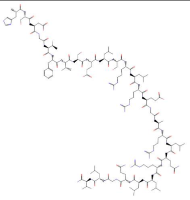	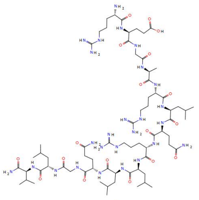	536.6656	536.6654	-0.48
						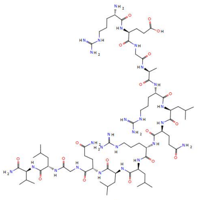	536.6656	536.6654	-0.48
MATCH	132.4	760.4252	760.4206	-6.06	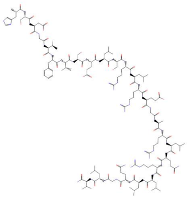	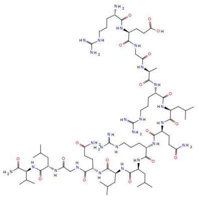	536.6656	536.6654	-0.48
						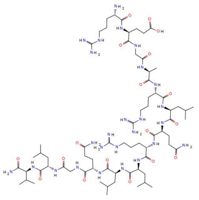	536.6656	536.6654	-0.48
MISMATCH	-8.6	585.3214	585.3190	-4.17	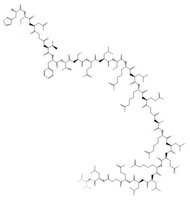		497.9658	497.9658	0.00
MISMATCH	-8.4	698.8734	698.8692	-5.92	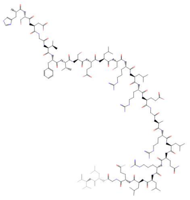		681.4012	681.4012	0.00
MISMATCH	-9.2	731.3995	731.3969	-3.60	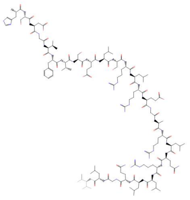		497.9658	497.9658	0.00
MISMATCH	-17.1	756.1704	756.1640	-8.44	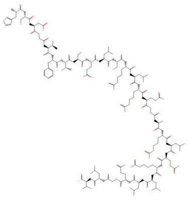		530.9926	530.9926	0.00
MET_MATCH						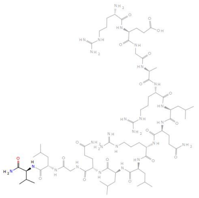	115.0861	115.0866	3.88

Metabolite: M4 -1431 RT=2.12

Type	score	sub. m/z observed	sub. m/z calculated	sub ppm		met. m/z observed	met. m/z calculated	met. ppm
MET_MATCH					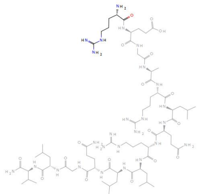	157.1077	157.1084	4.49
MET_MATCH					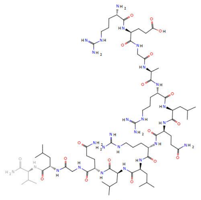	497.9658	497.9671	2.57
MET_MATCH					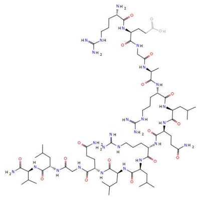	521.9922	522.0021	19.02
MET_MATCH					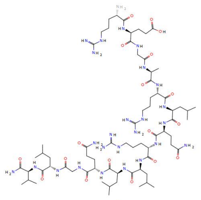	530.9926	530.9899	-5.05
MET_MATCH					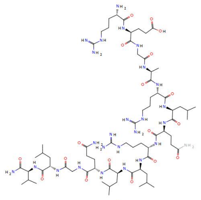	530.9926	530.9899	-5.05
MET_MATCH					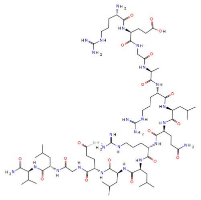	530.9926	530.9899	-5.05
MET_MATCH					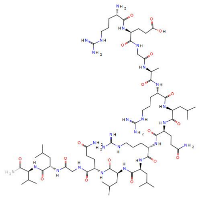	530.9926	530.9899	-5.05

MS (+) FT

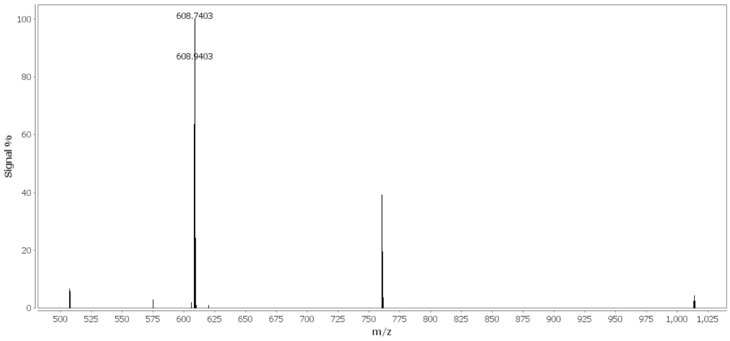

MS (+) FT

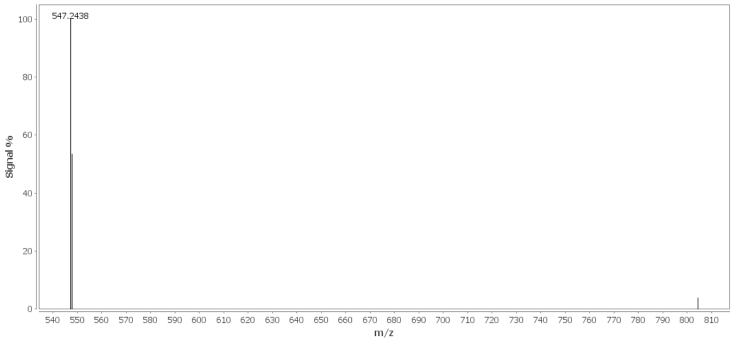

MS2 (+) FT activ = HCD:ce =

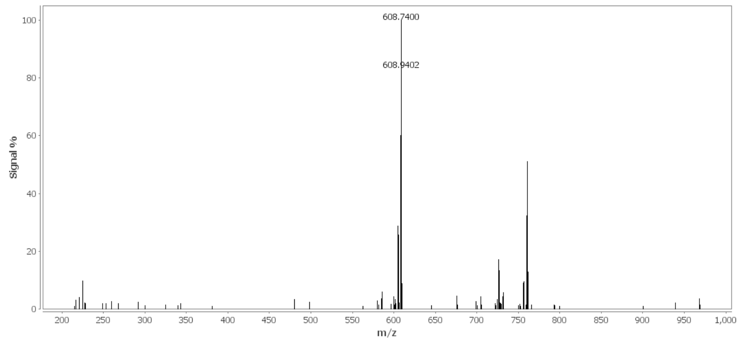

MS2 (+) FT activ = HCD:ce =

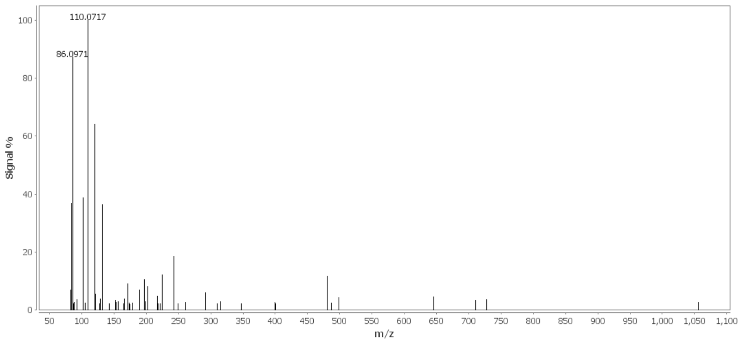

Metabolite: M1 -1945 RT=1.67

Type	score	sub. m/z observed	sub. m/z calculated	sub ppm			met. m/z observed	met. m/z calculated	met. ppm
MATCH	163.6	608.5403	608.5379	-3.81	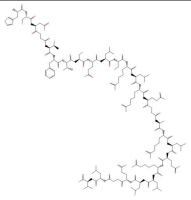	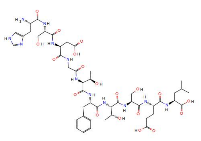	547.2438	547.2435	-0.57
						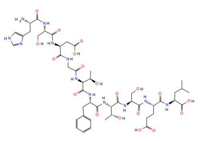	547.2438	547.2435	-0.57
MATCH	124.6	760.4255	760.4206	-6.45	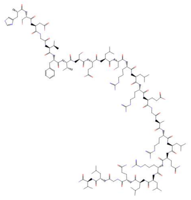	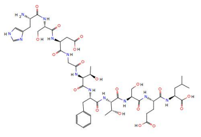	547.2438	547.2435	-0.57
						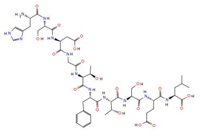	547.2438	547.2435	-0.57
MATCH	7.8	217.0827	217.0819	-3.69	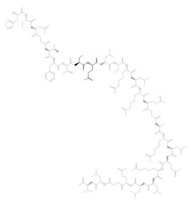	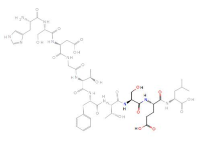	217.0820	217.0819	-0.40
MATCH	7.8	217.0827	217.0819	-3.69	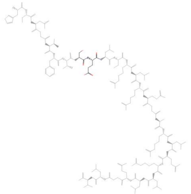	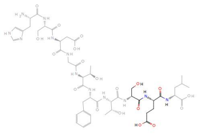	217.0820	217.0819	-0.40
MATCH	21.8	225.0990	225.0982	-3.57	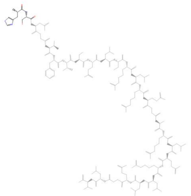	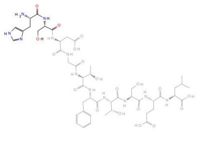	225.0981	225.0982	0.40

Metabolite: M1 -1945 RT=1.67

Type	score	sub. m/z observed	sub. m/z calculated	sub ppm		met. m/z observed	met. m/z calculated	met. ppm
MATCH	15.0	480.1849	480.1837	-2.34	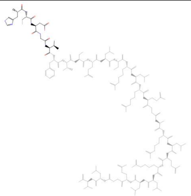 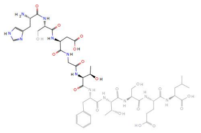	480.1833	480.1837	0.82
MATCH	15.0	480.1849	480.1837	-2.34	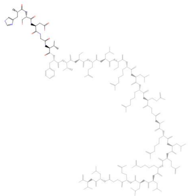 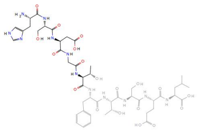	480.1833	480.1837	0.82
MATCH	6.7	498.1958	498.1943	-3.04	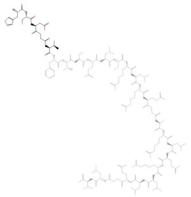 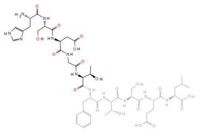	498.1913	498.1943	6.06
MET_MATCH					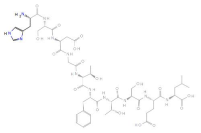	110.0717	110.0713	-3.91
MET_MATCH					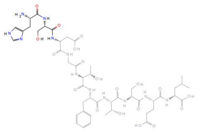	121.5691	121.5660	-25.5
MET_MATCH					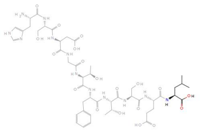	132.1020	132.1019	-0.70
MET_MATCH					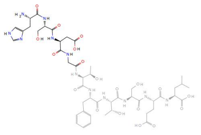	199.0710	199.0770	30.12
MET_MATCH					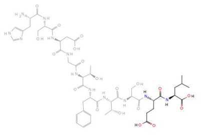	261.1451	261.1445	-2.47
MET_MATCH					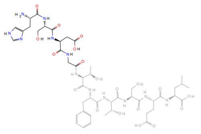	399.1704	399.1623	-20.3

Metabolite: M1 -1945 RT=1.67

Type	score	sub. m/z observed	sub. m/z calculated	sub ppm		met. m/z observed	met. m/z calculated	met. ppm
MET_MATCH					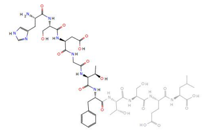	645.2560	645.2627	10.44

MS (+) FT

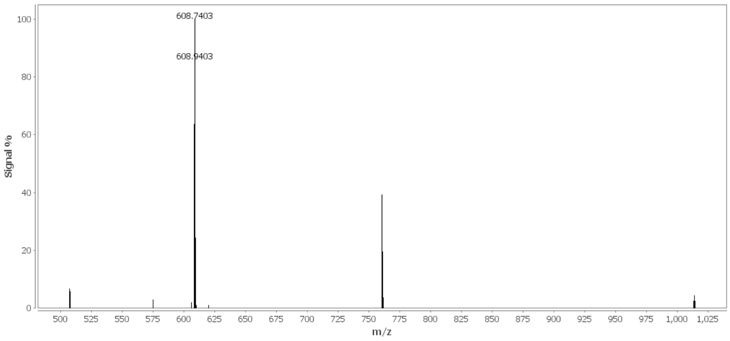

MS (+) FT

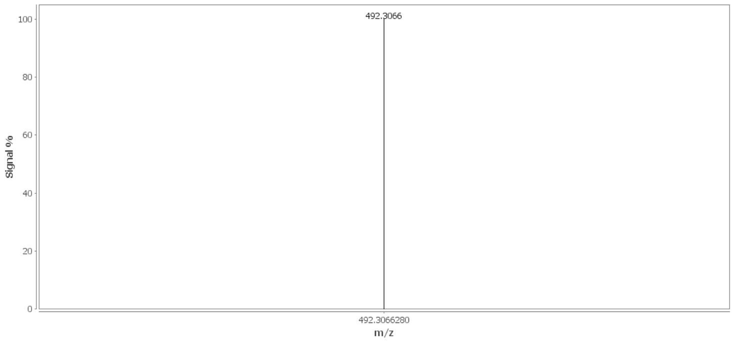

Metabolite: M3 -2055 RT=2.09

Type	score	sub. m/z observed	sub. m/z calculated	sub ppm		met. m/z observed	met. m/z calculated	met. ppm
MATCH	163.6	608.5403	608.5379	-3.81	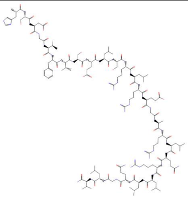 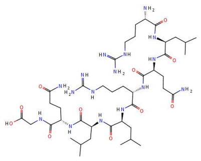	492.3066	492.3091	4.97
