# Supplementary material for: Software-aided approach to investigate peptide structure and metabolic susceptibility of amide bonds in peptide drugs based on high resolution mass spectrometry
Source: PLoS One. 2017 Nov 1;12(11):e0186461. doi: 10.1371/journal.pone.0186461 (PMC5665424; doi:10.1371/journal.pone.0186461)
Supplement: S1 File — (ZIP) [file pone.0186461.s007.zip › SFiles/S2_File.pdf]

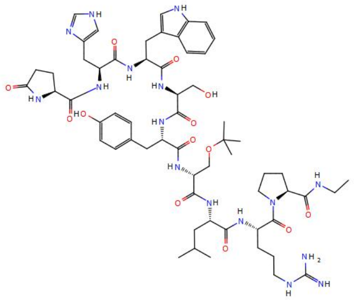

Buserelin

Property name	Property value
Time	0min, 5min, 15min, 45min, 120min
Instrument	ThermoQAPlus
Matrix	trypsin
Acquisition Mode	ddMS2

Chromatograms

Time=0min

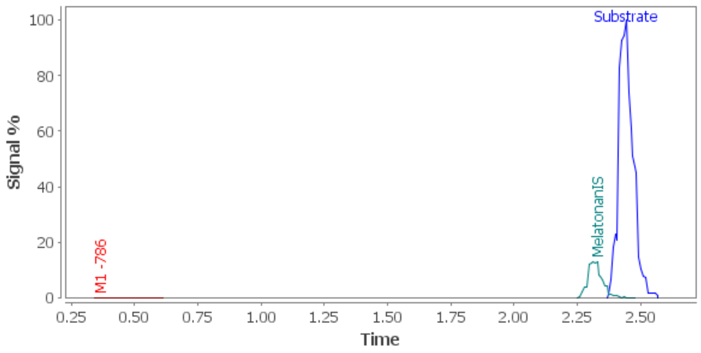

Time=5min

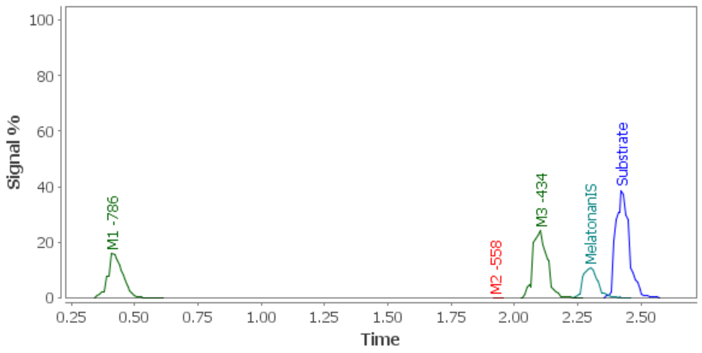

Time=15min

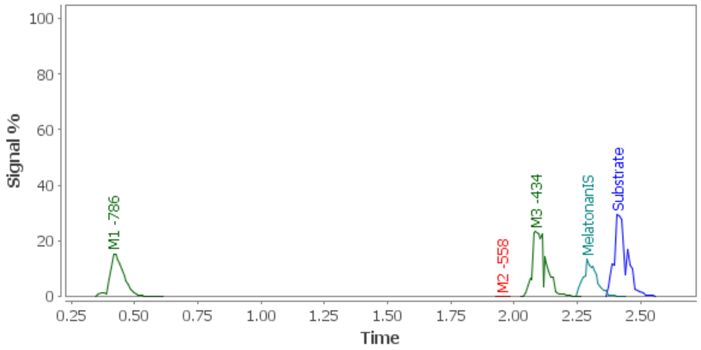

Time=45min

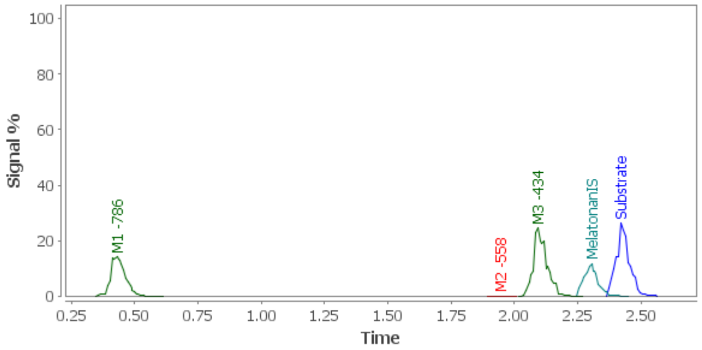

Time=120min

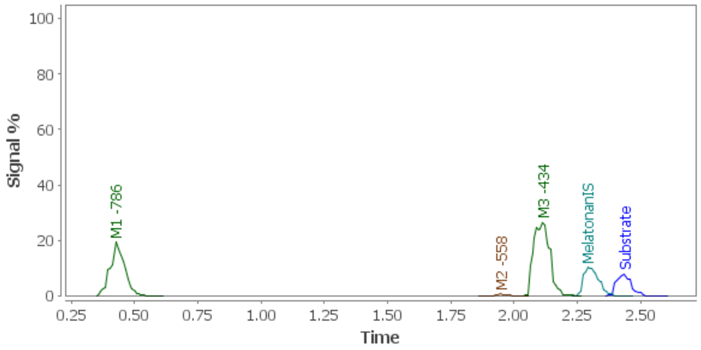

Custom Charts

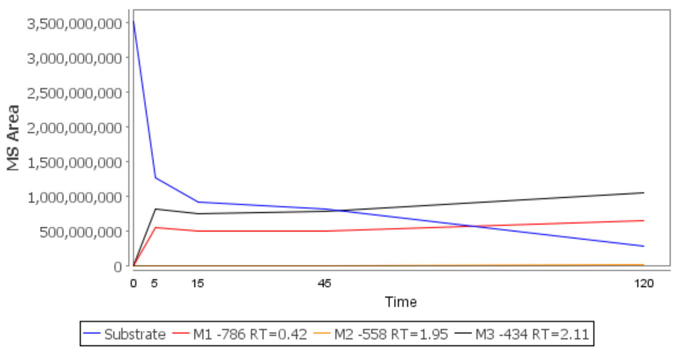

Fragmentation

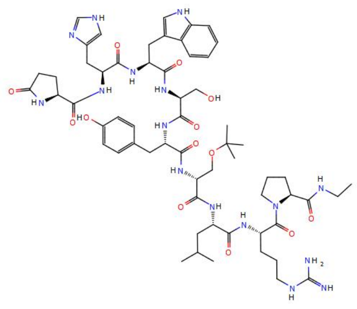

Buserelin

MS (+) FT

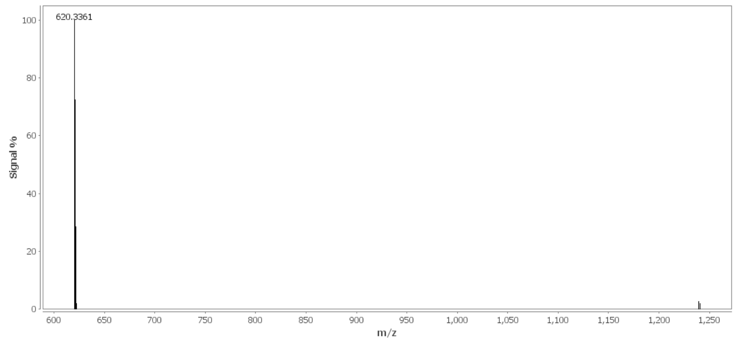

MS (+) FT

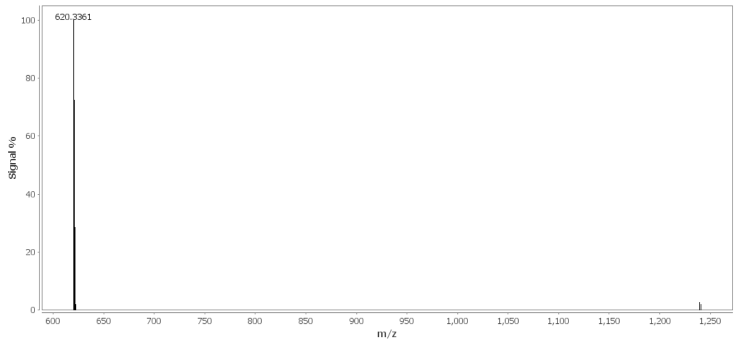

MS2 (+) FT activ = HCD:ce =

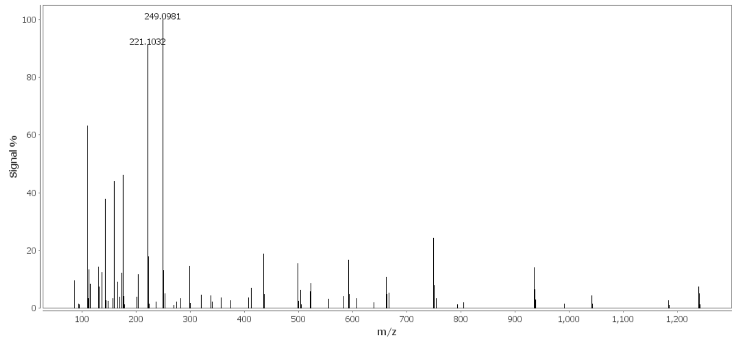

MS2 (+) FT activ = HCD:ce =

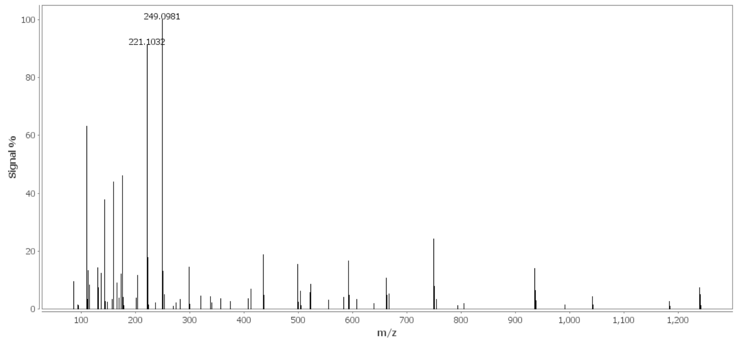

Metabolite: Substrate

Type	score	sub. m/z observed	sub. m/z calculated	sub ppm			met. m/z observed	met. m/z calculated	met. ppm
MATCH	102.7	1239.6651	1239.6633	-1.44	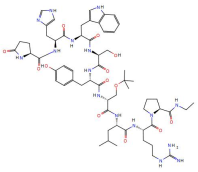	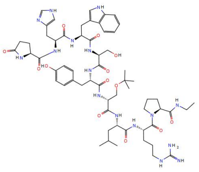	1239.6651	1239.6633	-1.44
MATCH	19.1	1239.6643	1239.6633	-0.84	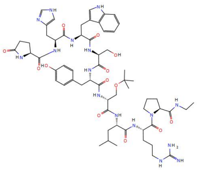	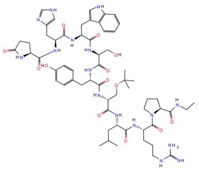	1239.6643	1239.6633	-0.84
MATCH	9.0	754.2867	754.2944	10.21	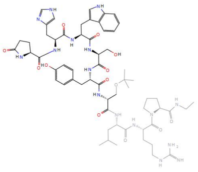	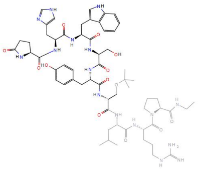	754.2867	754.2944	10.21

Metabolite: Substrate

Type	score	sub. m/z observed	sub. m/z calculated	sub ppm			met. m/z observed	met. m/z calculated	met. ppm
MATCH	12.1	662.3967	662.3984	2.56	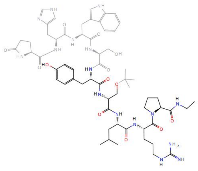	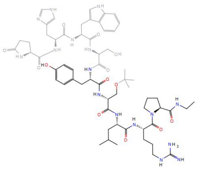	662.3967	662.3984	2.56
MATCH	200.0	620.3361	620.3353	-1.35	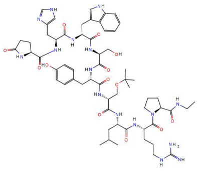	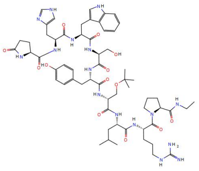	620.3361	620.3353	-1.35
MISMATCH	-22.1	592.3033	592.3040	1.19	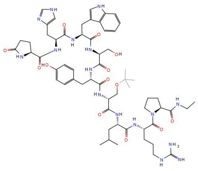	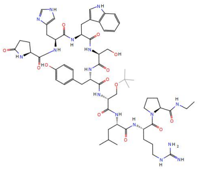	592.3033	592.3040	1.19
MATCH	6.9	583.2963	583.2987	4.21	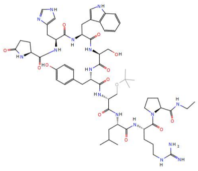	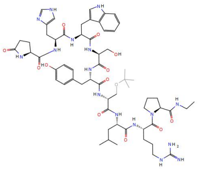	583.2963	583.2987	4.21
MATCH	4.4	555.3963	555.3977	2.49	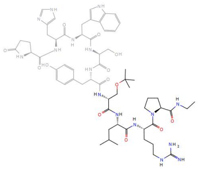	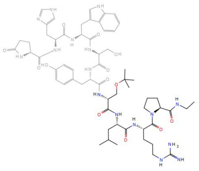	555.3963	555.3977	2.49
MATCH	9.9	504.2002	504.1990	-2.49	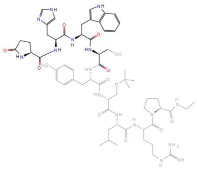	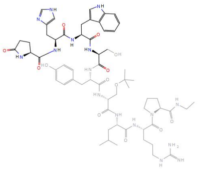	504.2002	504.1990	-2.49
MATCH	56.0	499.3347	499.3351	0.78	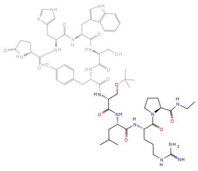	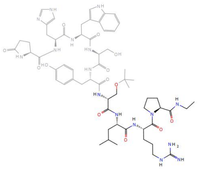	499.3347	499.3351	0.78
MATCH	12.9	412.3022	412.3031	2.19	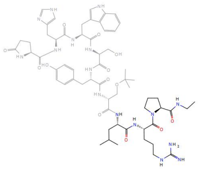	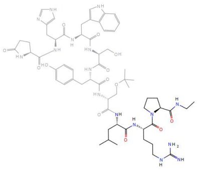	412.3022	412.3031	2.19
MATCH	58.1	299.2190	299.2190	0.09	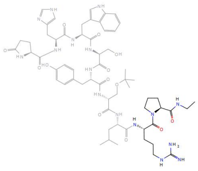	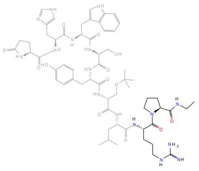	299.2190	299.2190	0.09

Metabolite: Substrate

Type	score	sub. m/z observed	sub. m/z calculated	sub ppm		met. m/z observed	met. m/z calculated	met. ppm
MATCH	20.2	282.1912	282.1925	4.53	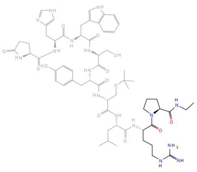 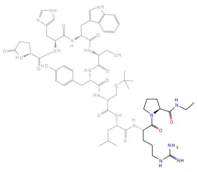	282.1912	282.1925	4.53
MATCH	2.1	270.1927	270.1925	-1.09	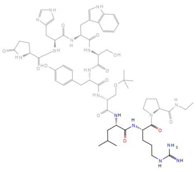 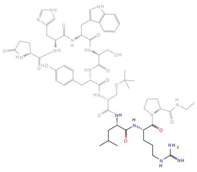	270.1927	270.1925	-1.09
MISMATCH	13.4	253.1659	253.1659	0.12	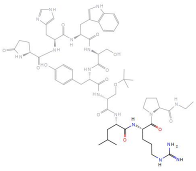 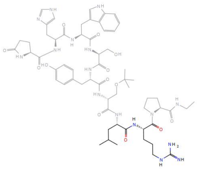	253.1659	253.1659	0.12
MATCH	179.3	249.0981	249.0982	0.37	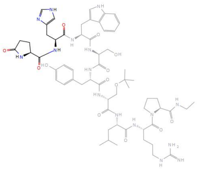 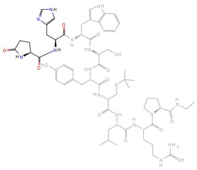	249.0981	249.0982	0.37
MATCH	3.6	237.1349	237.1346	-1.42	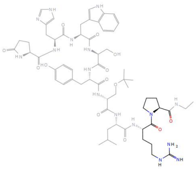 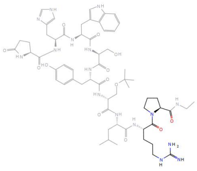	237.1349	237.1346	-1.42
MATCH	171.6	221.1032	221.1033	0.30	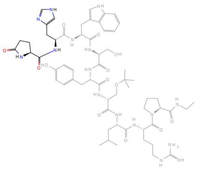 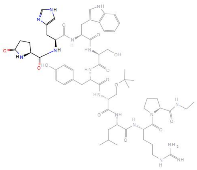	221.1032	221.1033	0.30
MATCH	15.9	166.0611	166.0611	-0.01	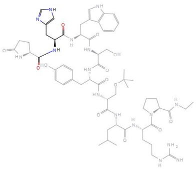 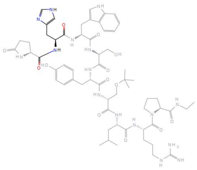	166.0611	166.0611	-0.01
MATCH	55.8	159.0916	159.0917	0.16	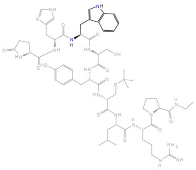 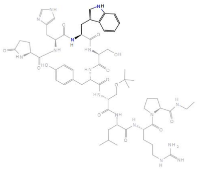	159.0916	159.0917	0.16
MATCH	7.9	157.1086	157.1084	-1.57	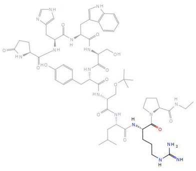 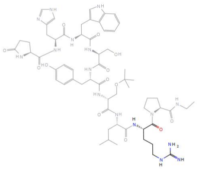	157.1086	157.1084	-1.57

Metabolite: Substrate

Type	score	sub. m/z observed	sub. m/z calculated	sub ppm			met. m/z observed	met. m/z calculated	met. ppm
MATCH	74.7	143.1180	143.1179	-0.75	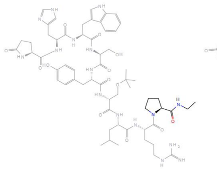	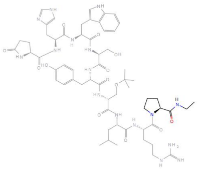	143.1180	143.1179	-0.75
MATCH	104.4	136.0757	136.0757	-0.32	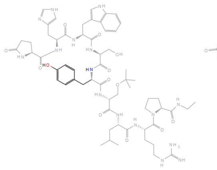	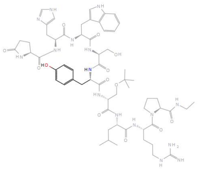	136.0757	136.0757	-0.32
MATCH	19.7	115.0870	115.0866	-3.66	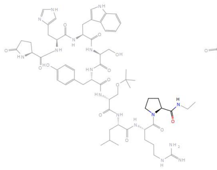	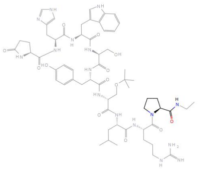	115.0870	115.0866	-3.66
MATCH	32.1	112.0873	112.0869	-3.00	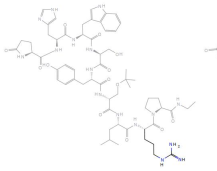	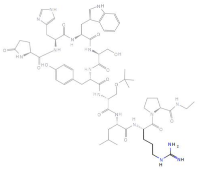	112.0873	112.0869	-3.00
MISMATCH	163.1	110.0716	110.0713	-3.25	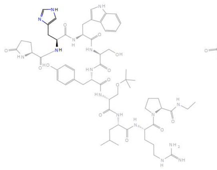	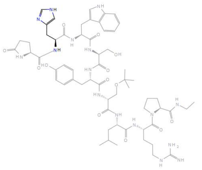	110.0716	110.0713	-3.25
MATCH	3.4	93.0452	93.0447	-5.28	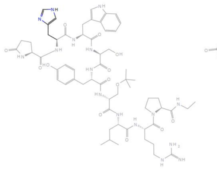	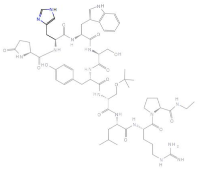	93.0452	93.0447	-5.28
MATCH	57.4	86.0971	86.0964	-7.90	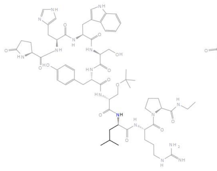	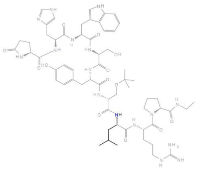	86.0971	86.0964	-7.90

MS (+) FT

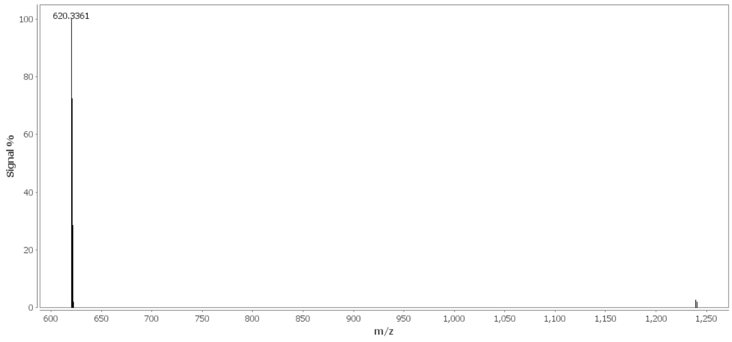

MS (+) FT

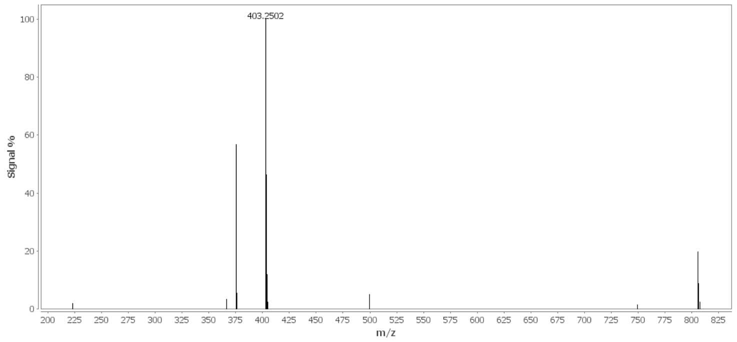

MS2 (+) FT activ = HCD:ce =

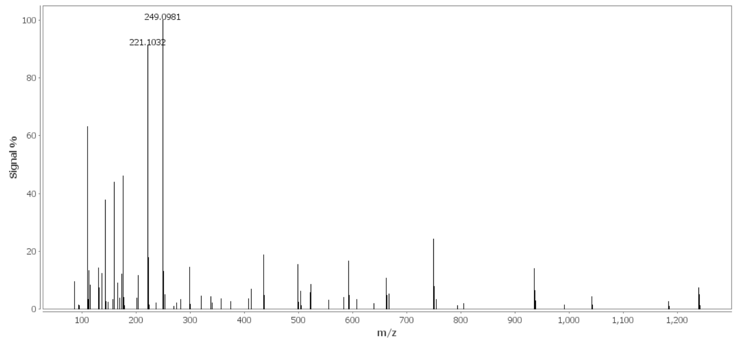

MS2 (+) FT activ = HCD:ce =

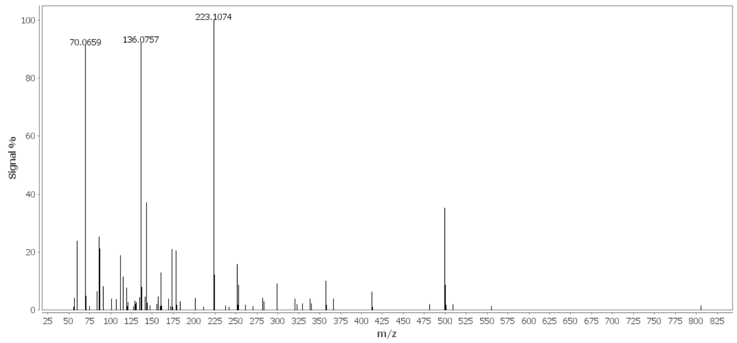

Metabolite: M3 -434 RT=2.11

Type	score	sub. m/z observed	sub. m/z calculated	sub ppm			met. m/z observed	met. m/z calculated	met. ppm
MATCH	200.0	620.3361	620.3353	-1.35	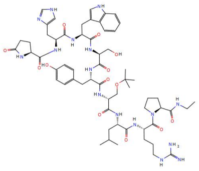	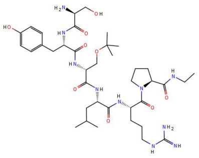	403.2502	403.2502	-0.05
MATCH	200.0	620.3361	620.3353	-1.35	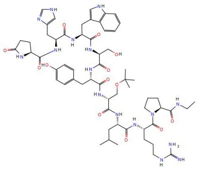	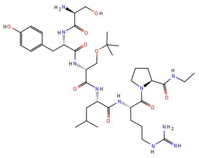	403.2502	403.2502	-0.05
MATCH	119.6	620.3361	620.3353	-1.35	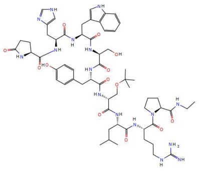	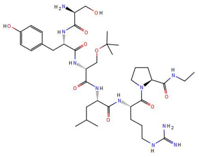	805.4938	805.4930	-0.91
MATCH	119.6	620.3361	620.3353	-1.35	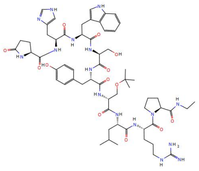	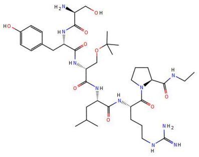	805.4938	805.4930	-0.91
MATCH	102.7	1239.6651	1239.6633	-1.44	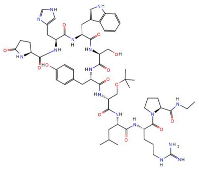	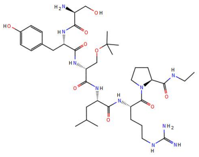	403.2502	403.2502	-0.05
MATCH	102.7	1239.6651	1239.6633	-1.44	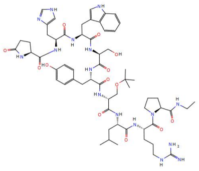	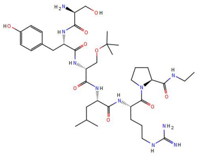	403.2502	403.2502	-0.05
MATCH	22.3	1239.6651	1239.6633	-1.44	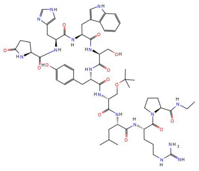	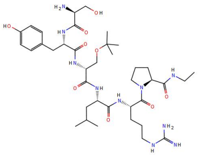	805.4938	805.4930	-0.91

Metabolite: M3 -434 RT=2.11

Type	score	sub. m/z observed	sub. m/z calculated	sub ppm		met. m/z observed	met. m/z calculated	met. ppm
MATCH	22.3	1239.6651	1239.6633	-1.44	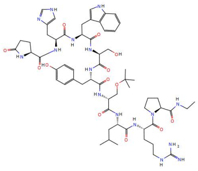 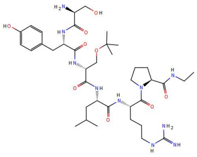	805.4938	805.4930	-0.91
MATCH	34.6	86.0971	86.0964	-7.90	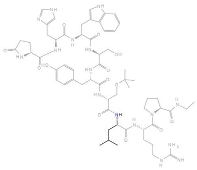 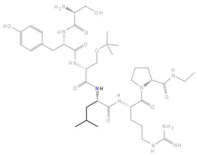	86.0970	86.0964	-7.02
MATCH	32.1	112.0873	112.0869	-3.00	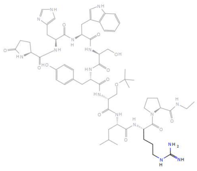 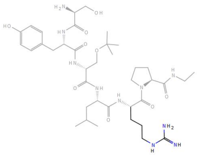	112.0872	112.0869	-2.56
MATCH	19.7	115.0870	115.0866	-3.66	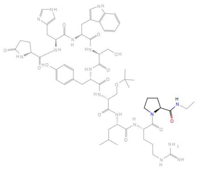 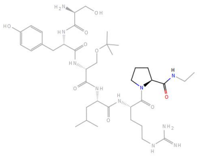	115.0868	115.0866	-1.91
MATCH	104.4	136.0757	136.0757	-0.32	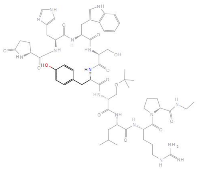 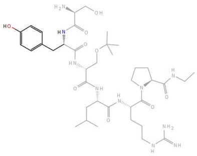	136.0757	136.0757	0.06
MATCH	74.7	143.1180	143.1179	-0.75	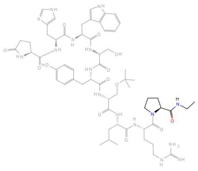 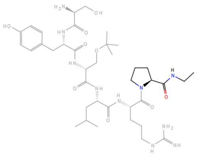	143.1178	143.1179	0.34
MATCH	7.9	157.1086	157.1084	-1.57	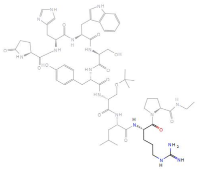 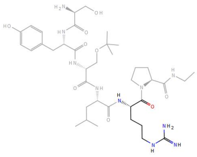	157.1081	157.1084	2.02
MATCH	3.6	237.1349	237.1346	-1.42	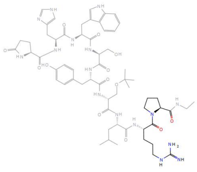 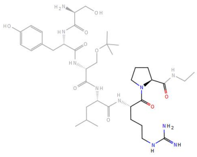	237.1341	237.1346	2.07
MATCH	13.4	253.1659	253.1659	0.12	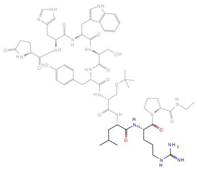 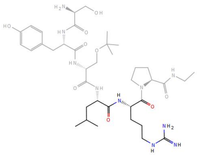	253.1653	253.1659	2.27

Metabolite: M3 -434 RT=2.11

Type	score	sub. m/z observed	sub. m/z calculated	sub ppm			met. m/z observed	met. m/z calculated	met. ppm
MATCH	2.1	270.1927	270.1925	-1.09	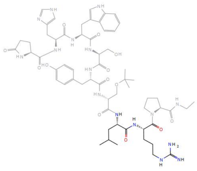	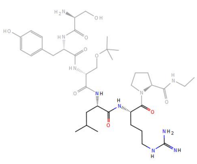	270.1919	270.1925	2.12
MATCH	7.3	282.1912	282.1925	4.53	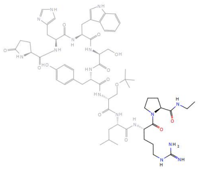	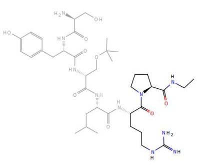	282.1900	282.1925	8.82
MATCH	23.5	299.2190	299.2190	0.09	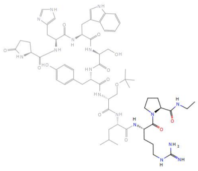	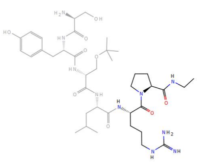	299.2186	299.2190	1.46
MATCH	12.9	412.3022	412.3031	2.19	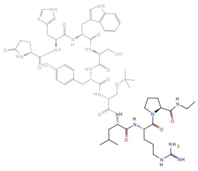	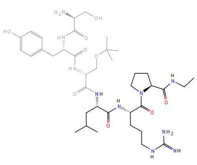	412.3025	412.3031	1.30
MATCH	50.5	499.3347	499.3351	0.78	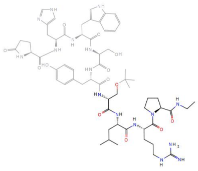	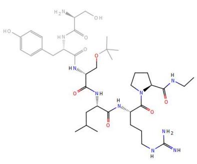	499.3344	499.3351	1.41
MATCH	9.9	504.2002	504.1990	-2.49	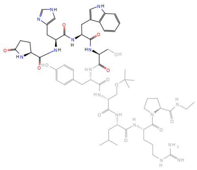	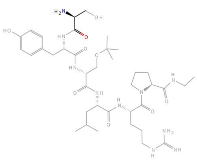	70.0296	70.0287	-11.6
MATCH	4.4	555.3963	555.3977	2.49	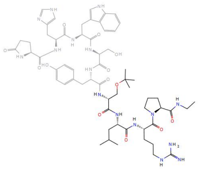	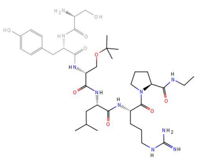	555.3961	555.3977	2.92
MATCH	6.9	583.2963	583.2987	4.21	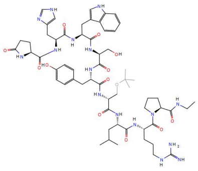	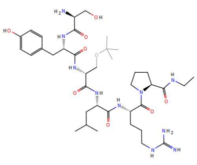	366.2128	366.2136	2.18
MATCH	7.2	754.2867	754.2944	10.21	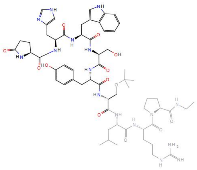	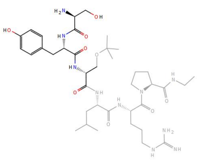	320.1234	320.1241	2.21

Metabolite: M3 -434 RT=2.11

Type	score	sub. m/z observed	sub. m/z calculated	sub ppm		met. m/z observed	met. m/z calculated	met. ppm
MATCH	8.9	1239.6643	1239.6633	-0.84	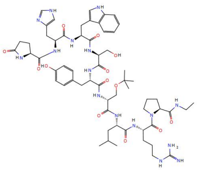 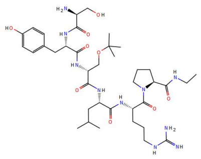	805.4894	805.4930	4.55
MISMATCH	-6.1	253.1659	253.1659	0.12	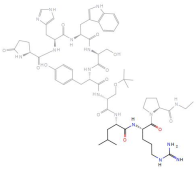	127.0867	127.0867	0.00
MET_MATCH					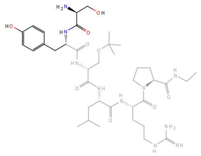	223.1073	223.1077	1.75
MET_MATCH					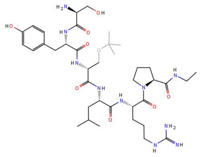	366.2135	366.2136	0.32
MET_MATCH					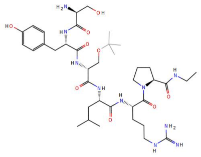	375.2188	375.2189	0.22
MET_MATCH					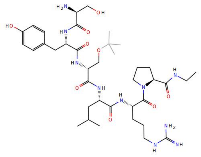	749.4294	749.4304	1.39
MET_MATCH					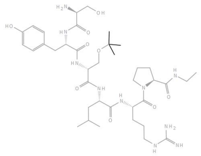	57.0707	57.0699	-15.0
MET_MATCH					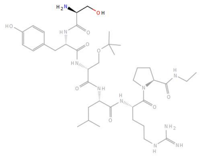	60.0453	60.0444	-14.4
MET_MATCH					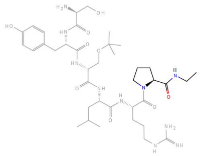	141.1022	141.1022	0.08

Metabolite: M3 -434 RT=2.11

Type	score	sub. m/z observed	sub. m/z calculated	sub ppm		met. m/z observed	met. m/z calculated	met. ppm
MET_MATCH					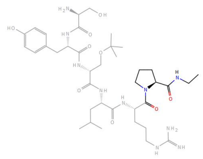	169.0970	169.0972	0.92
MET_MATCH					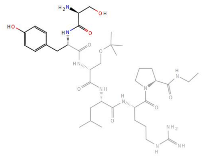	223.1074	223.1077	1.27
MET_MATCH					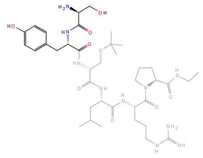	251.1022	251.1026	1.84

MS (+) FT

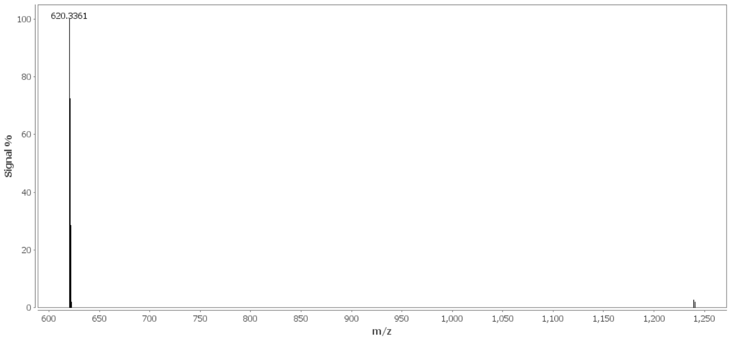

MS (+) FT

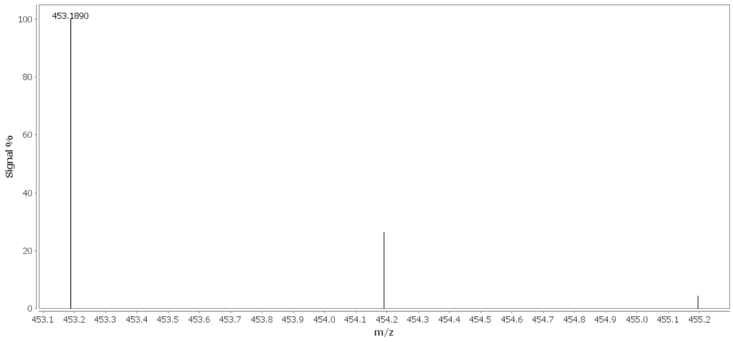

MS2 (+) FT activ = HCD:ce =

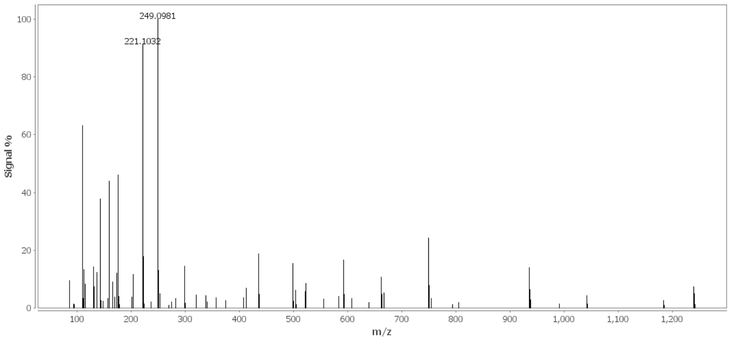

MS2 (+) FT activ = HCD:ce =

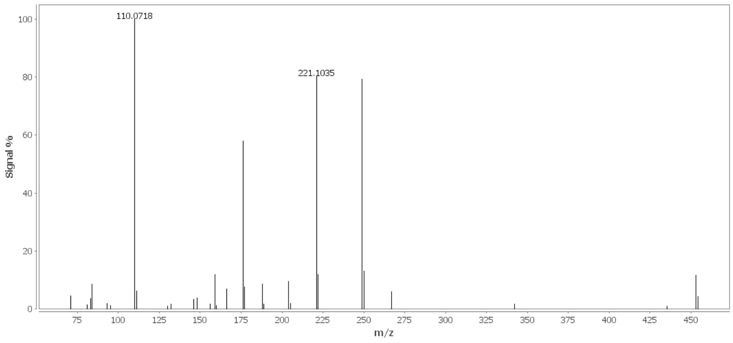

Metabolite: M1 -786 RT=0.42

Type	score	sub. m/z observed	sub. m/z calculated	sub ppm		met. m/z observed	met. m/z calculated	met. ppm
MATCH	200.0	620.3361	620.3353	-1.35	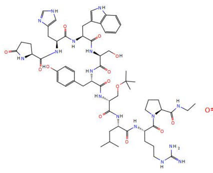 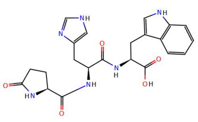	453.1890	453.1881	-1.90

Metabolite: M1 -786 RT=0.42

Type	score	sub. m/z observed	sub. m/z calculated	sub ppm			met. m/z observed	met. m/z calculated	met. ppm
						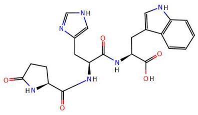	453.1890	453.1881	-1.90
MATCH	102.7	1239.6651	1239.6633	-1.44	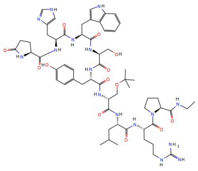	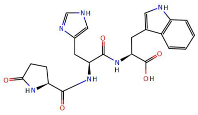	453.1890	453.1881	-1.90
						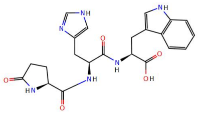	453.1890	453.1881	-1.90
MATCH	3.4	93.0452	93.0447	-5.28	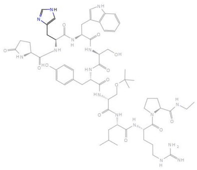	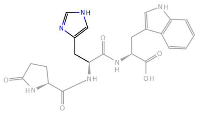	93.0454	93.0447	-7.71
MATCH	2.5	95.0610	95.0604	-6.89	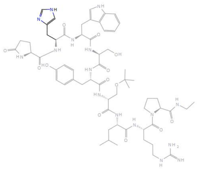	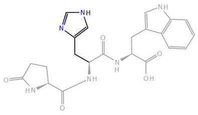	95.0610	95.0604	-6.86
MATCH	163.1	110.0716	110.0713	-3.25	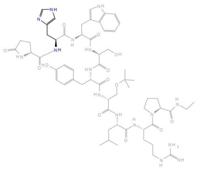	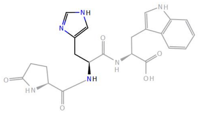	110.0718	110.0713	-4.71
MATCH	55.8	159.0916	159.0917	0.16	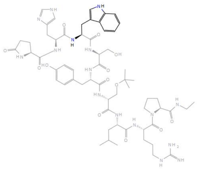	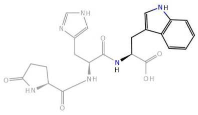	159.0918	159.0917	-0.98
MATCH	15.9	166.0611	166.0611	-0.01	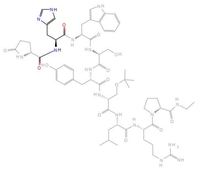	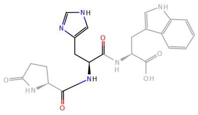	166.0613	166.0611	-1.08
MATCH	171.6	221.1032	221.1033	0.30	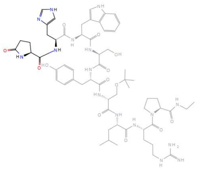	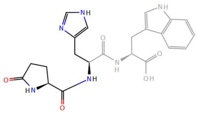	221.1035	221.1033	-0.83

Metabolite: M1 -786 RT=0.42

Type	score	sub. m/z observed	sub. m/z calculated	sub ppm		met. m/z observed	met. m/z calculated	met. ppm
MATCH	179.3	249.0981	249.0982	0.37	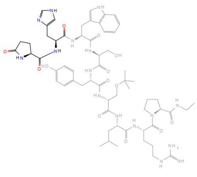 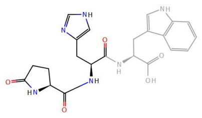	249.0984	249.0982	-0.86
MATCH	19.1	1239.6643	1239.6633	-0.84	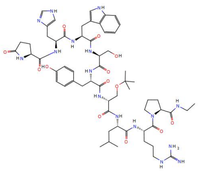 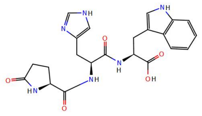	453.1886	453.1881	-1.09
MET_MATCH					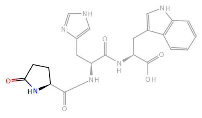	84.0452	84.0444	-9.08
MET_MATCH					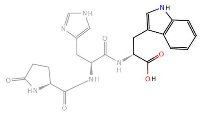	188.0707	188.0706	-0.56
MET_MATCH					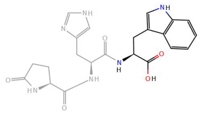	205.0974	205.0972	-1.21
MET_MATCH					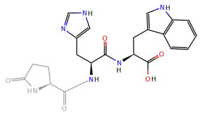	342.1564	342.1561	-1.05
MET_MATCH					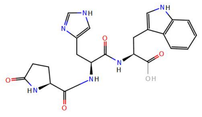	435.1772	435.1775	0.72

MS (+) FT

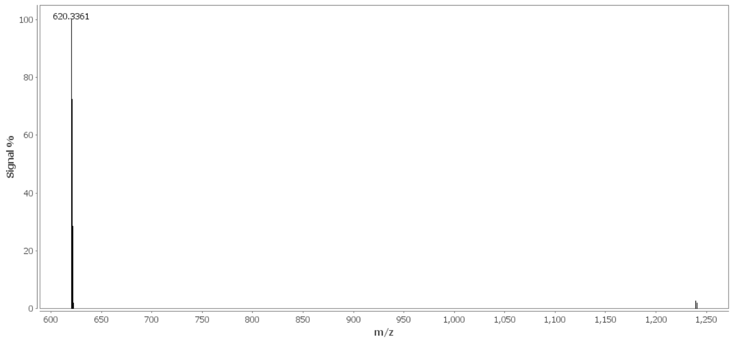

MS (+) FT

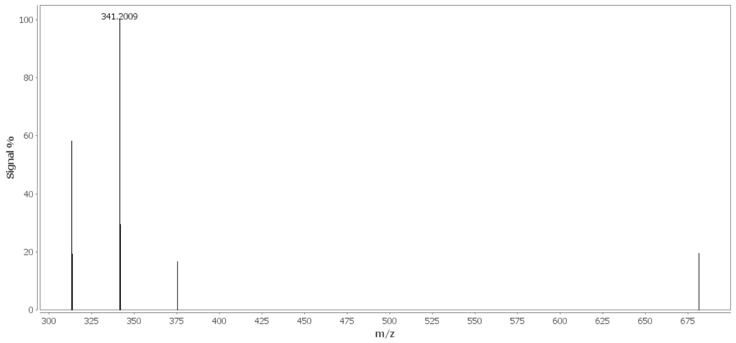

MS2 (+) FT activ = HCD:ce =

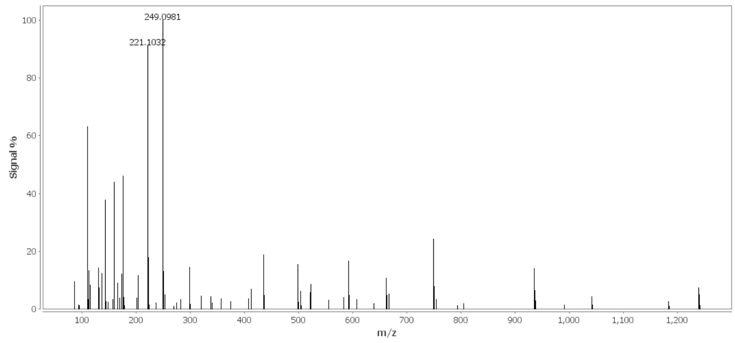

MS2 (+) FT activ = HCD:ce =

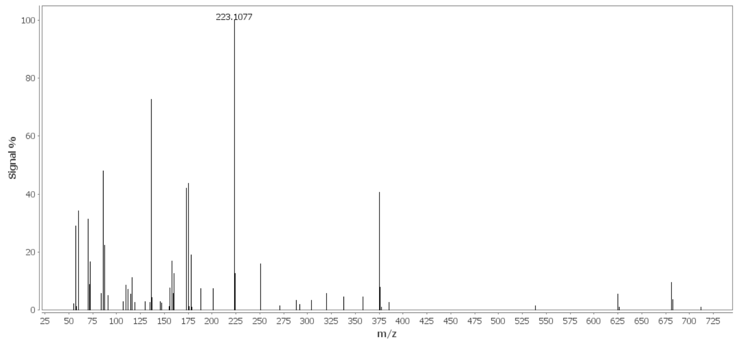

Metabolite: M2 -558 RT=1.95

Type	score	sub. m/z observed	sub. m/z calculated	sub ppm			met. m/z observed	met. m/z calculated	met. ppm
MATCH	200.0	620.3361	620.3353	-1.35	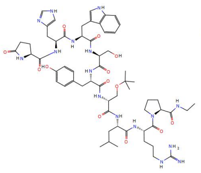	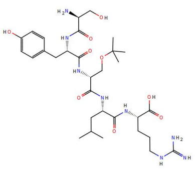	341.2009	341.2001	-2.23
MATCH	200.0	620.3361	620.3353	-1.35	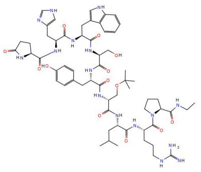	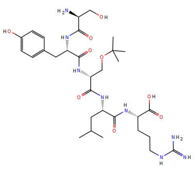	341.2009	341.2001	-2.23
MATCH	119.5	620.3361	620.3353	-1.35	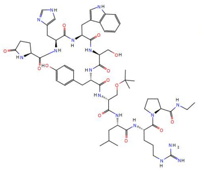	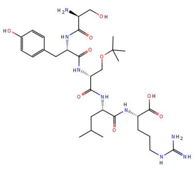	681.3956	681.3930	-3.80
MATCH	119.5	620.3361	620.3353	-1.35	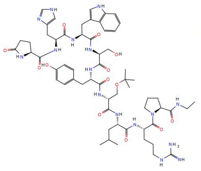	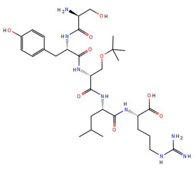	681.3956	681.3930	-3.80
MATCH	102.7	1239.6651	1239.6633	-1.44	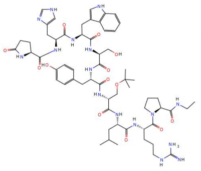	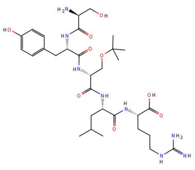	341.2009	341.2001	-2.23
MATCH	102.7	1239.6651	1239.6633	-1.44	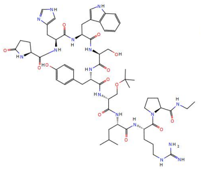	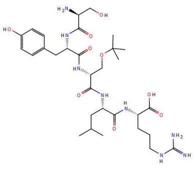	341.2009	341.2001	-2.23
MATCH	22.2	1239.6651	1239.6633	-1.44	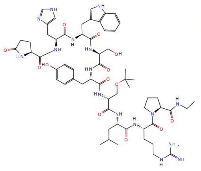	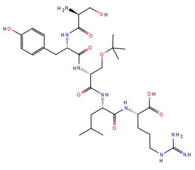	681.3956	681.3930	-3.80

Metabolite: M2 -558 RT=1.95

Type	score	sub. m/z observed	sub. m/z calculated	sub ppm		met. m/z observed	met. m/z calculated	met. ppm
MATCH	22.2	1239.6651	1239.6633	-1.44	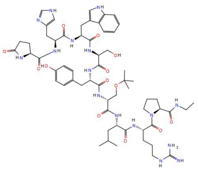 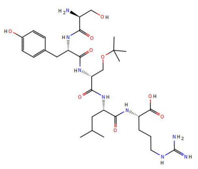	681.3956	681.3930	-3.80
MATCH	57.4	86.0971	86.0964	-7.90	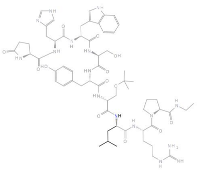 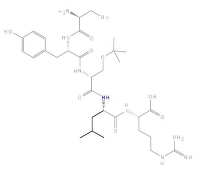	86.0970	86.0964	-6.71
MATCH	20.4	112.0873	112.0869	-3.00	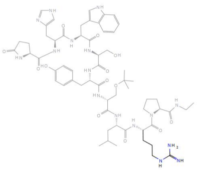 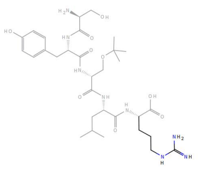	112.0871	112.0869	-1.43
MATCH	84.9	136.0757	136.0757	-0.32	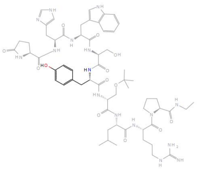 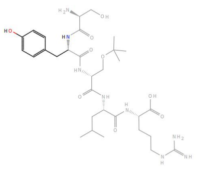	136.0758	136.0757	-1.18
MATCH	20.2	282.1912	282.1925	4.53	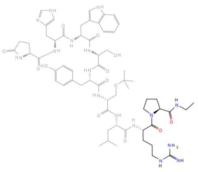 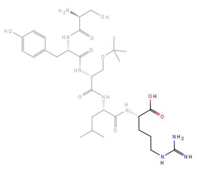	158.0924	158.0924	-0.20
MATCH	58.1	299.2190	299.2190	0.09	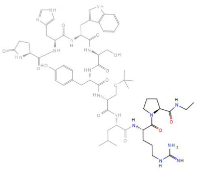 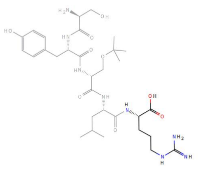	175.1190	175.1190	-0.11
MATCH	10.2	412.3022	412.3031	2.19	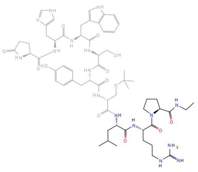 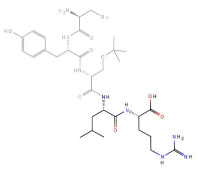	288.2029	288.2030	0.26
MATCH	56.0	499.3347	499.3351	0.78	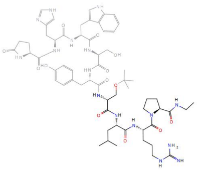 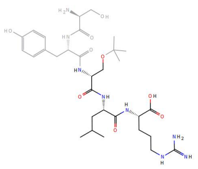	375.2358	375.2350	-2.12
MATCH	6.4	583.2963	583.2987	4.21	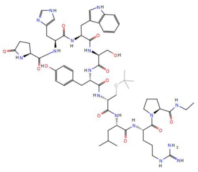 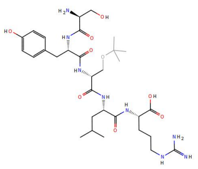	304.1640	304.1636	-1.36

Metabolite: M2 -558 RT=1.95

Type	score	sub. m/z observed	sub. m/z calculated	sub ppm			met. m/z observed	met. m/z calculated	met. ppm
MATCH	12.1	662.3967	662.3984	2.56	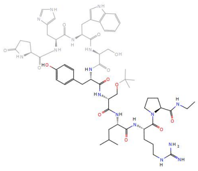	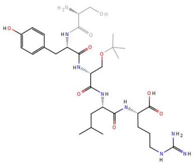	538.2988	538.2984	-0.73
MATCH	9.0	754.2867	754.2944	10.21	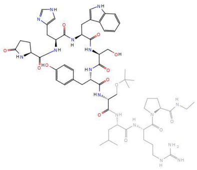	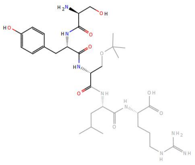	320.1233	320.1241	2.34
MATCH	17.1	1239.6643	1239.6633	-0.84	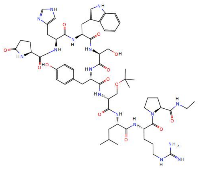	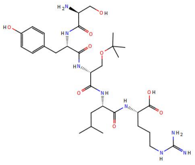	681.3896	681.3930	5.01
MISMATCH	-71.7	110.0716	110.0713	-3.25	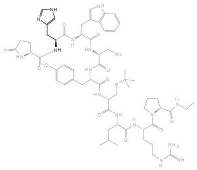		110.0713	110.0713	0.00
MISMATCH	-22.1	592.3033	592.3040	1.19	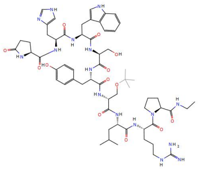		625.3311	625.3311	0.00
MET_MATCH						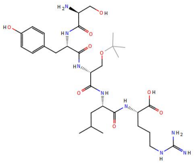	313.1695	313.1688	-2.25
MET_MATCH						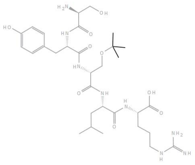	57.0710	57.0699	-18.8
MET_MATCH						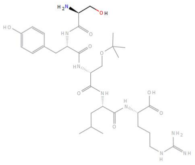	60.0454	60.0444	-16.6
MET_MATCH						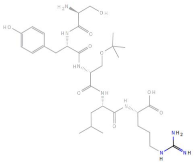	60.0564	60.0556	-13.0

Metabolite: M2 -558 RT=1.95

Type	score	sub. m/z observed	sub. m/z calculated	sub ppm		met. m/z observed	met. m/z calculated	met. ppm
MET_MATCH					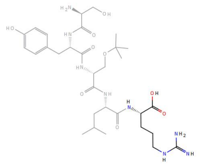	87.0560	87.0553	-8.33
MET_MATCH					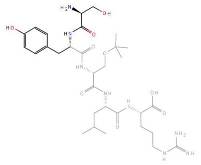	223.1077	223.1077	-0.13
MET_MATCH					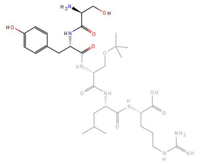	251.1019	251.1026	3.04
MET_MATCH					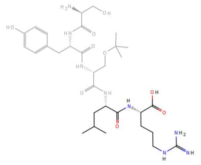	271.1771	271.1765	-2.20
MET_MATCH					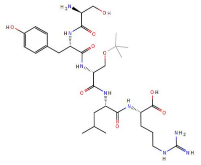	625.3311	625.3304	-1.14
