# Supplementary material for: Software-aided approach to investigate peptide structure and metabolic susceptibility of amide bonds in peptide drugs based on high resolution mass spectrometry
Source: PLoS One. 2017 Nov 1;12(11):e0186461. doi: 10.1371/journal.pone.0186461 (PMC5665424; doi:10.1371/journal.pone.0186461)
Supplement: S1 File — (ZIP) [file pone.0186461.s007.zip › SFiles/S30_File.pdf]

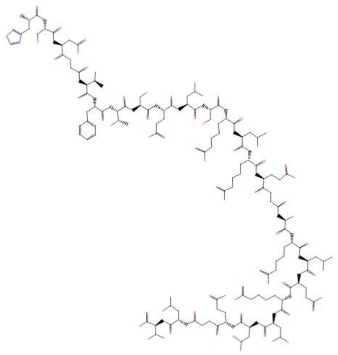

Secretin

Property name	Property value
Time	0min, 5min, 15min, 45min, 120min
Instrument	ThermoQAPlus
Matrix	trypsin
Acquisition Mode	ddMS2

Chromatograms

Time=0min

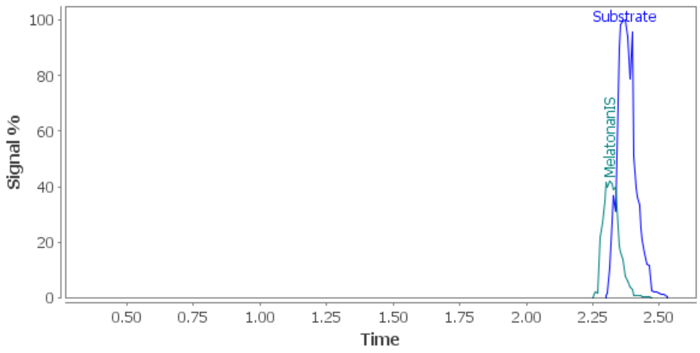

Time=5min

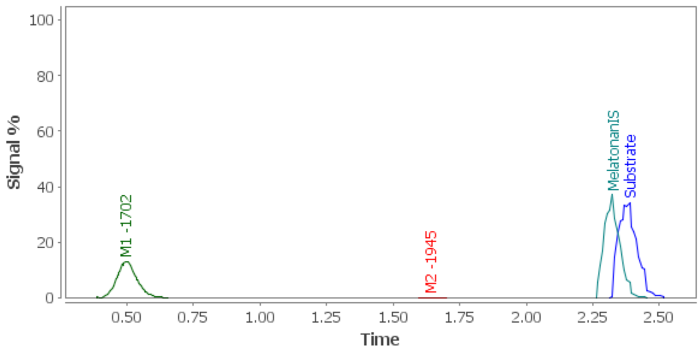

Time=15min

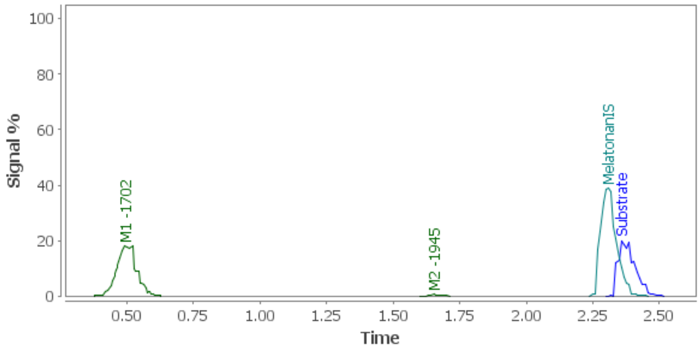

Time=45min

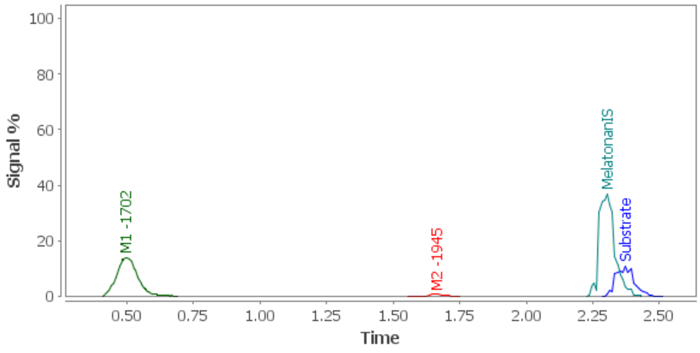

Time=120min

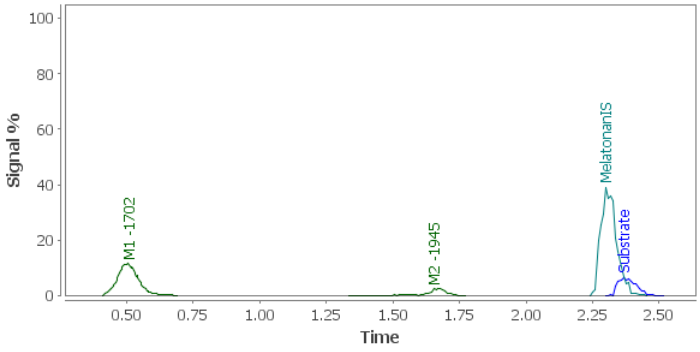

Custom Charts

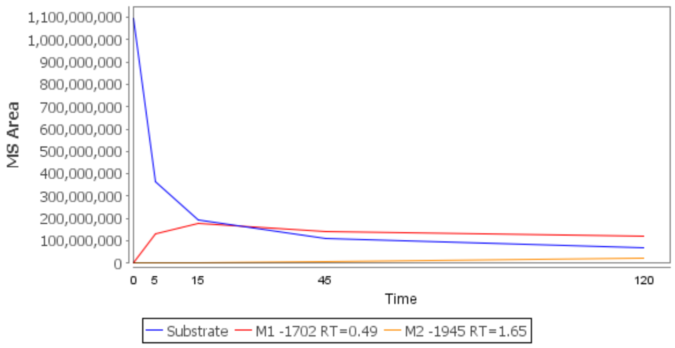

Fragmentation

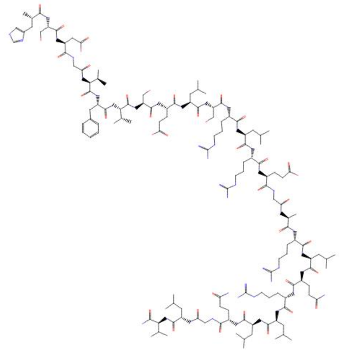

Secretin

MS (+) FT

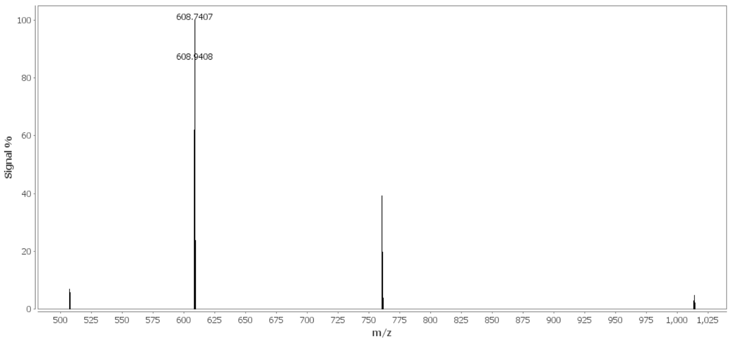

MS (+) FT

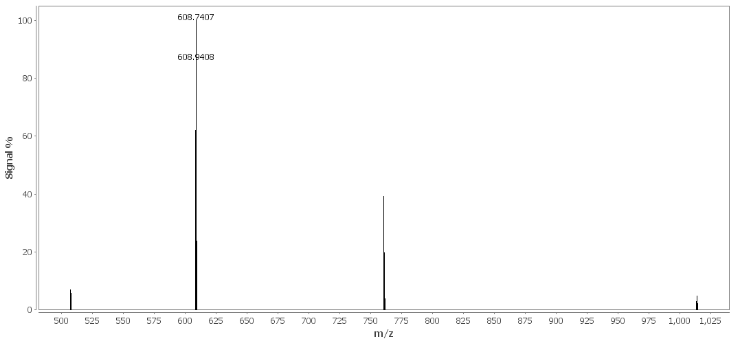

MS2 (+) FT activ = HCD:ce =

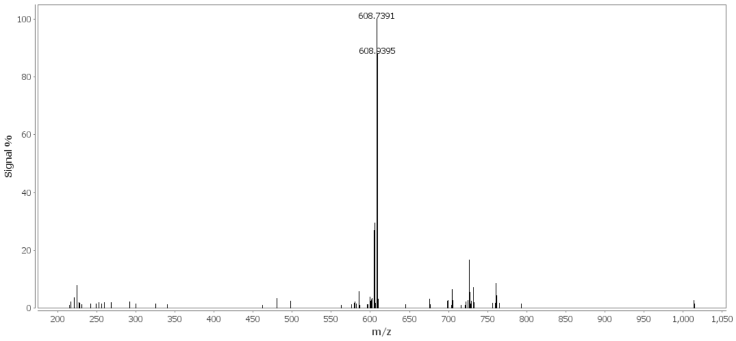

MS2 (+) FT activ = HCD:ce =

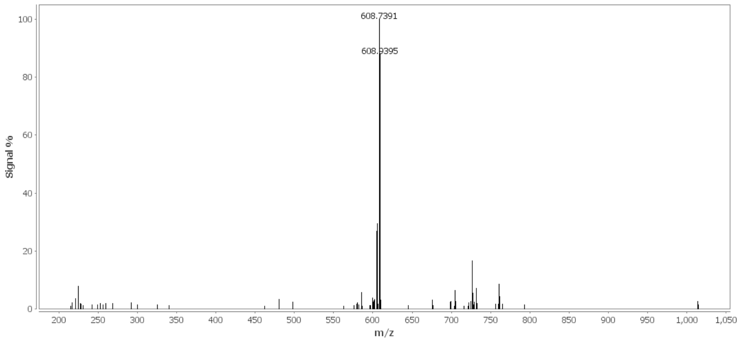

Metabolite: Substrate

Type	score	sub. m/z observed	sub. m/z calculated	sub ppm			met. m/z observed	met. m/z calculated	met. ppm
MATCH	103.0	1013.5659	1013.5584	-7.41	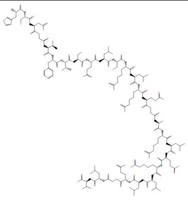	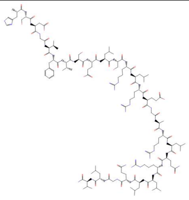	1013.5659	1013.5584	-7.41
MISMATCH	-20.2	765.1248	765.1240	-0.98	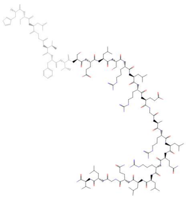	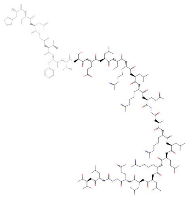	765.1248	765.1240	-0.98
MATCH	124.7	760.4259	760.4206	-6.98	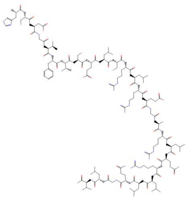	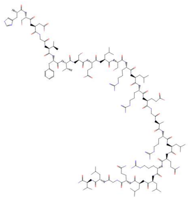	760.4259	760.4206	-6.98

Metabolite: Substrate

Type	score	sub. m/z observed	sub. m/z calculated	sub ppm			met. m/z observed	met. m/z calculated	met. ppm
MATCH	15.3	760.4247	760.4206	-5.42	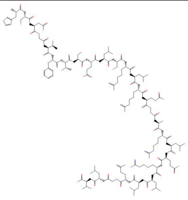	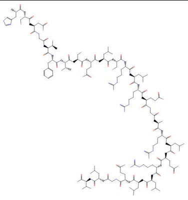	760.4247	760.4206	-5.42
MISMATCH	-11.4	726.1565	726.1559	-0.81	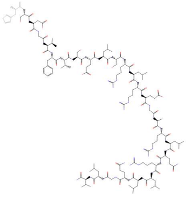	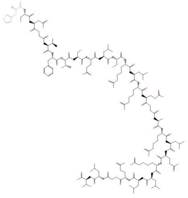	726.1565	726.1559	-0.81
MISMATCH	-6.2	704.3987	704.3979	-1.13	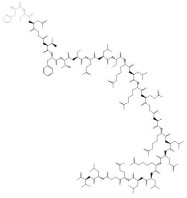	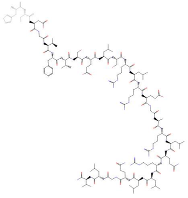	704.3987	704.3979	-1.13
MISMATCH	-4.3	675.6415	675.6411	-0.50	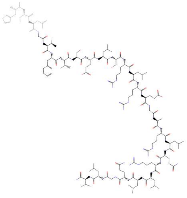	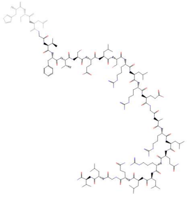	675.6415	675.6411	-0.50
MATCH	7.8	645.2637	645.2627	-1.57	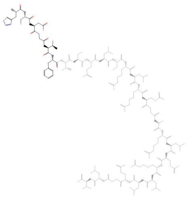	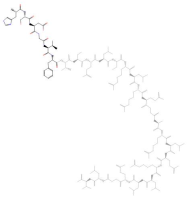	645.2637	645.2627	-1.57
MATCH	162.0	608.5406	608.5379	-4.37	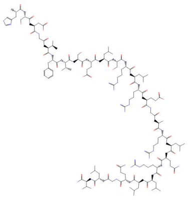	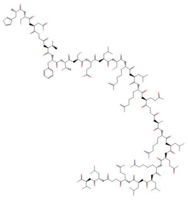	608.5406	608.5379	-4.37
MATCH	71.1	608.5390	608.5379	-1.69	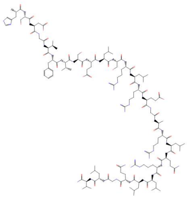	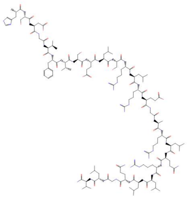	608.5390	608.5379	-1.69
MISMATCH	-14.3	604.9367	604.9358	-1.41	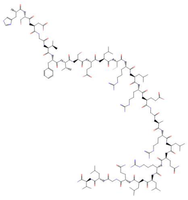	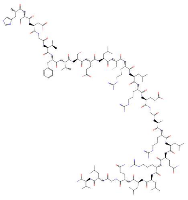	604.9367	604.9358	-1.41
MISMATCH	-14.3	604.9367	604.9358	-1.41	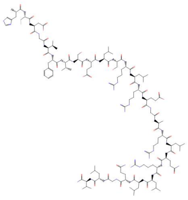	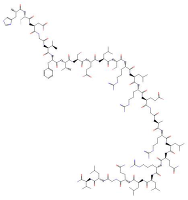	604.9367	604.9358	-1.41

Metabolite: Substrate

Type	score	sub. m/z observed	sub. m/z calculated	sub ppm			met. m/z observed	met. m/z calculated	met. ppm
MISMATCH	-14.3	604.9367	604.9358	-1.41	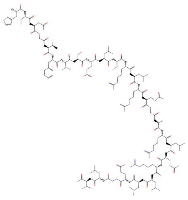	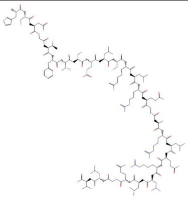	604.9367	604.9358	-1.41
MISMATCH	-14.3	604.9367	604.9358	-1.41	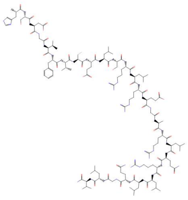	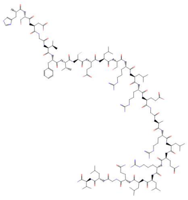	604.9367	604.9358	-1.41
MISMATCH	-14.3	604.9367	604.9358	-1.41	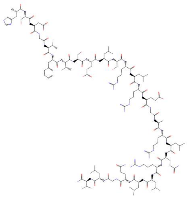	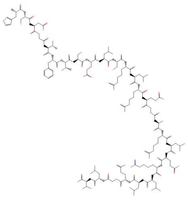	604.9367	604.9358	-1.41
MATCH	11.7	498.1939	498.1943	0.84	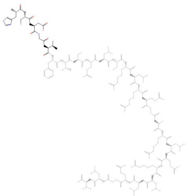	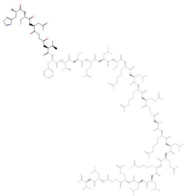	498.1939	498.1943	0.84
MATCH	14.9	480.1837	480.1837	-0.01	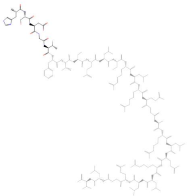	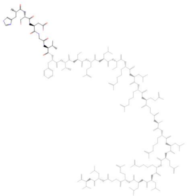	480.1837	480.1837	-0.01
MATCH	14.9	480.1837	480.1837	-0.01	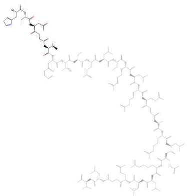	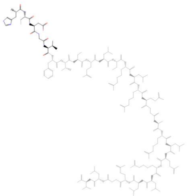	480.1837	480.1837	-0.01
MATCH	3.9	340.1247	340.1252	1.33	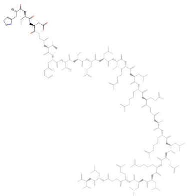	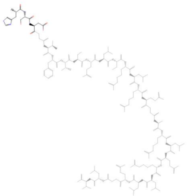	340.1247	340.1252	1.33
MISMATCH	-6.3	268.1409	268.1397	-4.21	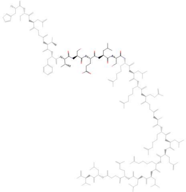	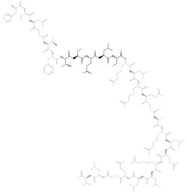	268.1409	268.1397	-4.21
MATCH	2.4	249.1230	249.1234	1.57	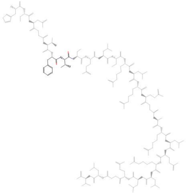	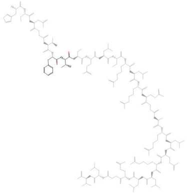	249.1230	249.1234	1.57

Metabolite: Substrate

Type	score	sub. m/z observed	sub. m/z calculated	sub ppm			met. m/z observed	met. m/z calculated	met. ppm
MATCH	2.4	249.1230	249.1234	1.57	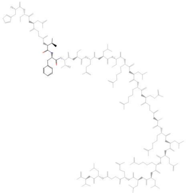	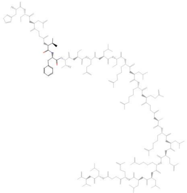	249.1230	249.1234	1.57
MATCH	2.4	249.1230	249.1234	1.57	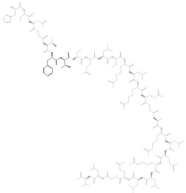	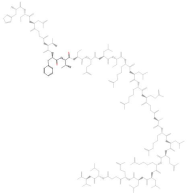	249.1230	249.1234	1.57
MATCH	2.4	249.1230	249.1234	1.57	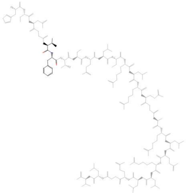	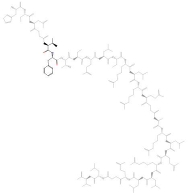	249.1230	249.1234	1.57
MATCH	21.8	225.0986	225.0982	-1.51	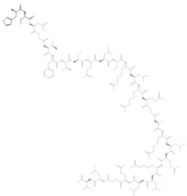	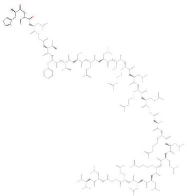	225.0986	225.0982	-1.51
MISMATCH	-5.0	221.1288	221.1264	-10.7	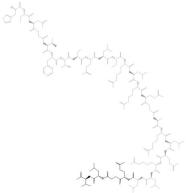	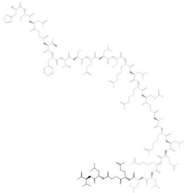	221.1288	221.1264	-10.7
MATCH	11.9	217.0822	217.0819	-1.54	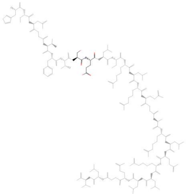	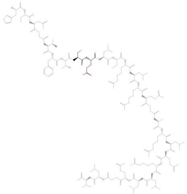	217.0822	217.0819	-1.54
MATCH	11.9	217.0822	217.0819	-1.54	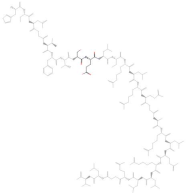	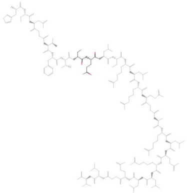	217.0822	217.0819	-1.54
MISMATCH	-3.8	215.1388	215.1446	27.01	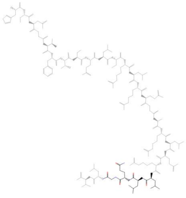	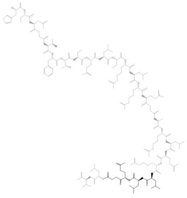	215.1388	215.1446	27.01
MISMATCH	-3.8	215.1388	215.1446	27.01	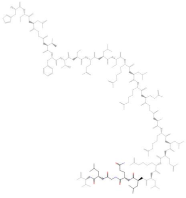	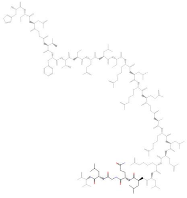	215.1388	215.1446	27.01

MS (+) FT

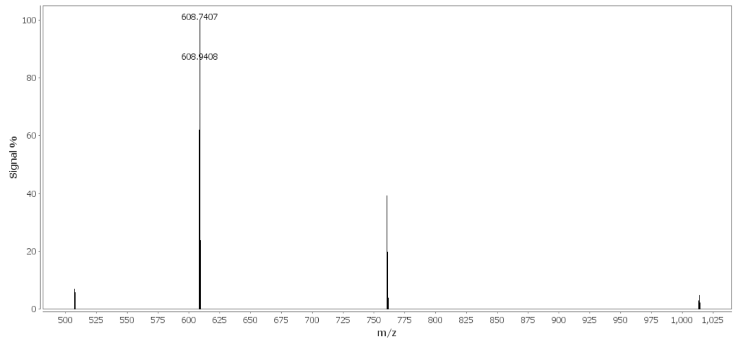

MS (+) FT

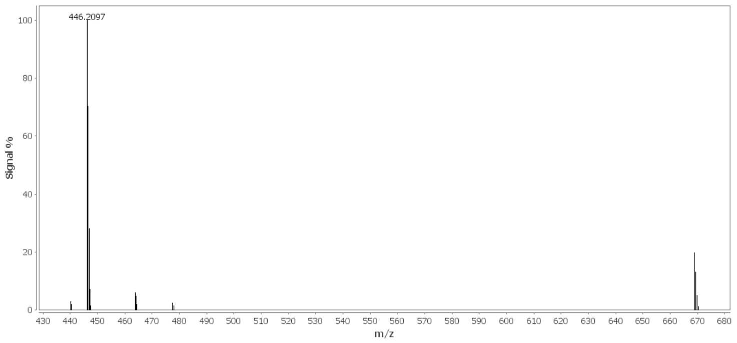

MS2 (+) FT activ = HCD:ce =

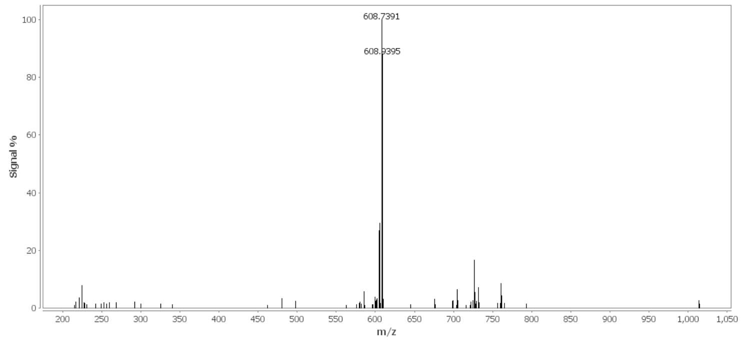

MS2 (+) FT activ = HCD:ce =

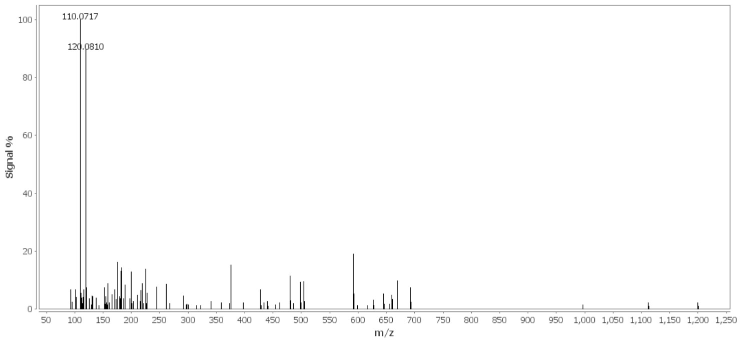

Metabolite: M1 -1702 RT=0.49

Type	score	sub. m/z observed	sub. m/z calculated	sub ppm		met. m/z observed	met. m/z calculated	met. ppm
MATCH	162.0	608.5406	608.5379	-4.37	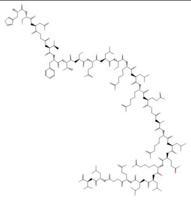 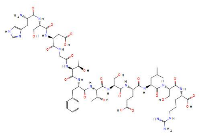	446.2097	446.2091	-1.34
MATCH	162.0	608.5406	608.5379	-4.37	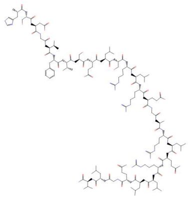 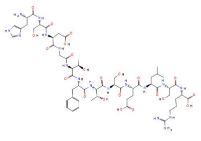	446.2097	446.2091	-1.34
					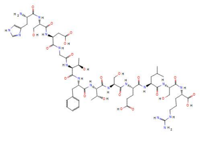	446.2097	446.2091	-1.34
MATCH	81.7	608.5406	608.5379	-4.37	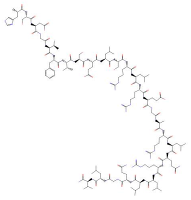 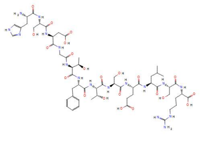	668.8111	668.8100	-1.59
MATCH	81.7	608.5406	608.5379	-4.37	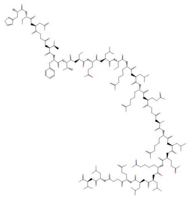 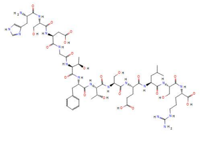	668.8111	668.8100	-1.59

Metabolite: M1 -1702 RT=0.49

Type	score	sub. m/z observed	sub. m/z calculated	sub ppm			met. m/z observed	met. m/z calculated	met. ppm
MATCH	124.7	760.4259	760.4206	-6.98	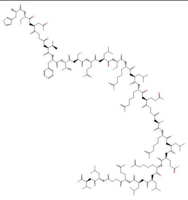	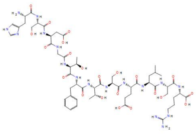	446.2097	446.2091	-1.34
MATCH	124.7	760.4259	760.4206	-6.98	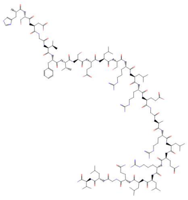	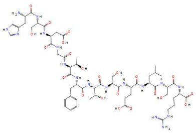	446.2097	446.2091	-1.34
						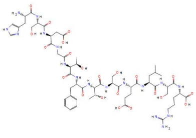	446.2097	446.2091	-1.34
MATCH	44.5	760.4259	760.4206	-6.98	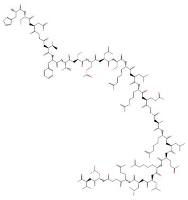	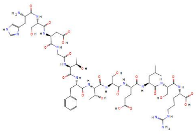	668.8111	668.8100	-1.59
MATCH	44.5	760.4259	760.4206	-6.98	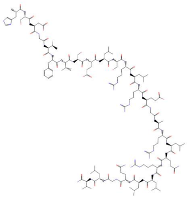	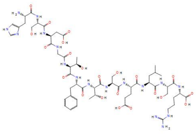	668.8111	668.8100	-1.59
MATCH	103.0	1013.5659	1013.5584	-7.41	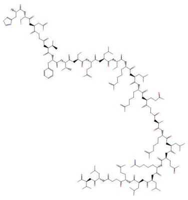	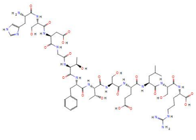	446.2097	446.2091	-1.34
						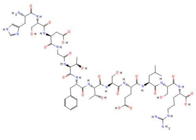	446.2097	446.2091	-1.34
						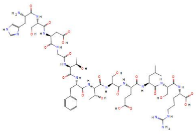	446.2097	446.2091	-1.34
MATCH	8.5	217.0822	217.0819	-1.54	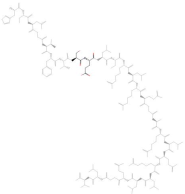	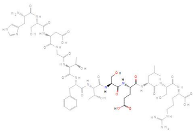	217.0817	217.0819	0.88

Metabolite: M1 -1702 RT=0.49

Type	score	sub. m/z observed	sub. m/z calculated	sub ppm			met. m/z observed	met. m/z calculated	met. ppm
MATCH	8.5	217.0822	217.0819	-1.54	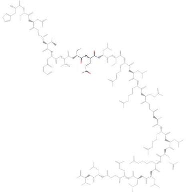	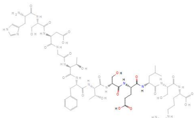	217.0817	217.0819	0.88
MATCH	21.8	225.0986	225.0982	-1.51	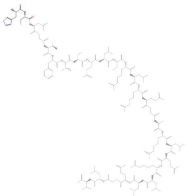	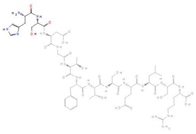	225.0982	225.0982	-0.14
MATCH	3.9	340.1247	340.1252	1.33	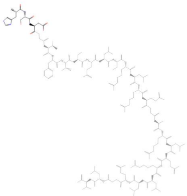	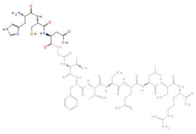	340.1249	340.1252	0.79
MATCH	14.9	480.1837	480.1837	-0.01	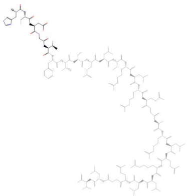	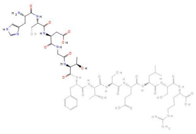	480.1838	480.1837	-0.18
MATCH	14.9	480.1837	480.1837	-0.01	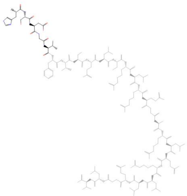	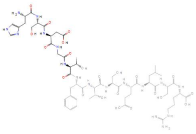	480.1838	480.1837	-0.18
MATCH	11.7	498.1939	498.1943	0.84	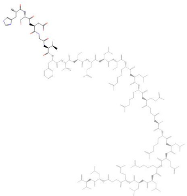	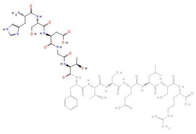	498.1930	498.1943	2.64
MATCH	71.1	608.5390	608.5379	-1.69	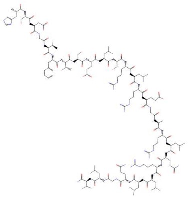	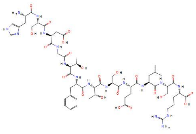	668.8098	668.8100	0.30
						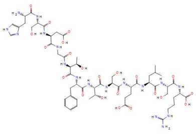	668.8098	668.8100	0.30
MATCH	6.6	645.2637	645.2627	-1.57	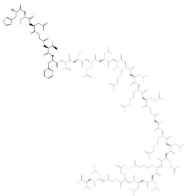	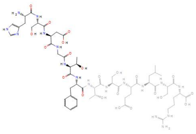	645.2627	645.2627	-0.03

Metabolite: M1 -1702 RT=0.49

Type	score	sub. m/z observed	sub. m/z calculated	sub ppm		met. m/z observed	met. m/z calculated	met. ppm
MATCH	15.3	760.4247	760.4206	-5.42	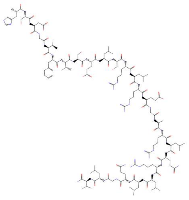 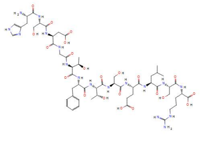	668.8098	668.8100	0.30
					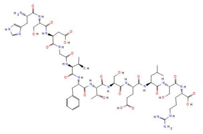	668.8098	668.8100	0.30
MISMATCH	-3.8	215.1388	215.1446	27.01	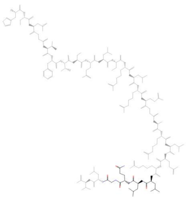	215.1387	215.1387	0.00
MISMATCH	-6.3	268.1409	268.1397	-4.21	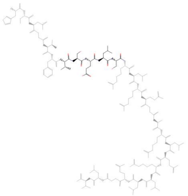	179.0927	179.0927	0.00
MISMATCH	-14.3	604.9367	604.9358	-1.41	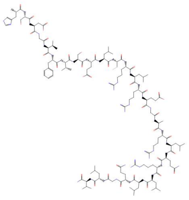	440.2052	440.2052	0.00
MISMATCH	-16.4	604.9367	604.9358	-1.41	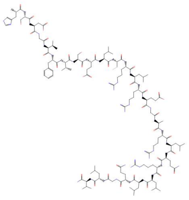	659.8045	659.8045	0.00
MISMATCH	-4.3	675.6415	675.6411	-0.50	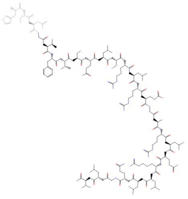	997.4917	997.4917	0.00
MISMATCH	-6.2	704.3987	704.3979	-1.13	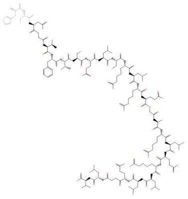	1112.5192	1112.5192	0.00
MISMATCH	-11.4	726.1565	726.1559	-0.81	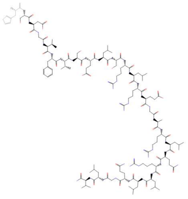	1199.5512	1199.5512	0.00

Metabolite: M1 -1702 RT=0.49

Type	score	sub. m/z observed	sub. m/z calculated	sub ppm		met. m/z observed	met. m/z calculated	met. ppm
MISMATCH	-20.2	765.1248	765.1240	-0.98	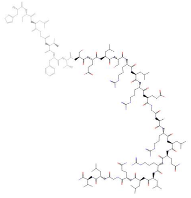	591.3091	591.3091	0.00
MET_MATCH					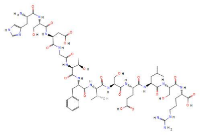	440.2061	440.2056	-1.19
MET_MATCH					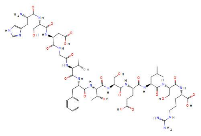	440.2061	440.2056	-1.19
MET_MATCH					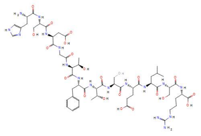	440.2061	440.2056	-1.19
MET_MATCH					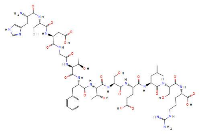	440.2061	440.2056	-1.19
MET_MATCH					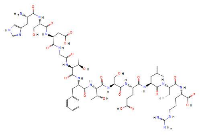	440.2061	440.2056	-1.19
MET_MATCH					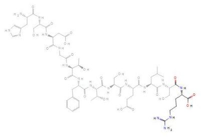	102.0555	102.0606	49.70
MET_MATCH					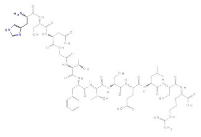	110.0717	110.0713	-3.48
MET_MATCH					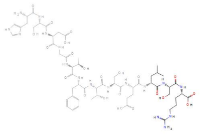	120.0810	120.0743	-55.3

Metabolite: M1 -1702 RT=0.49

Type	score	sub. m/z observed	sub. m/z calculated	sub ppm		met. m/z observed	met. m/z calculated	met. ppm
MET_MATCH					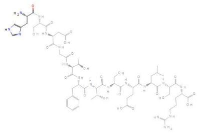	138.0662	138.0662	-0.09
MET_MATCH					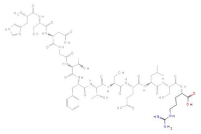	158.0922	158.0924	1.14
MET_MATCH					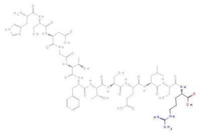	175.1189	175.1190	0.05
MET_MATCH					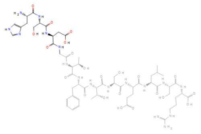	186.0914	186.0873	-21.7
MET_MATCH					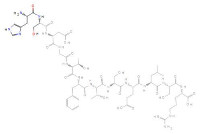	197.1031	197.1033	1.19
MET_MATCH					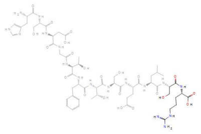	245.1240	245.1244	1.73
MET_MATCH					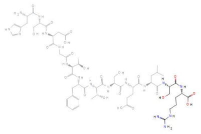	262.1507	262.1510	0.91
MET_MATCH					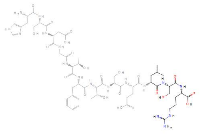	358.2082	358.2085	0.79
MET_MATCH					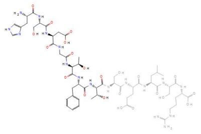	373.6566	373.6588	5.86

Metabolite: M1 -1702 RT=0.49

Type	score	sub. m/z observed	sub. m/z calculated	sub ppm		met. m/z observed	met. m/z calculated	met. ppm
MET_MATCH					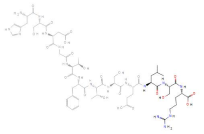	375.2347	375.2350	0.96
MET_MATCH					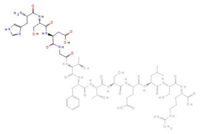	397.1460	397.1466	1.59
MET_MATCH					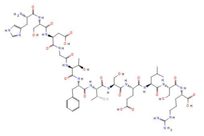	440.2052	440.2056	0.95
MET_MATCH					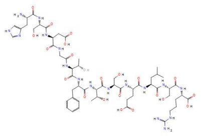	440.2052	440.2056	0.95
MET_MATCH					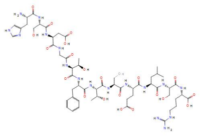	440.2052	440.2056	0.95
MET_MATCH					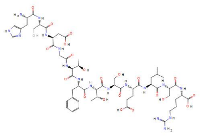	440.2052	440.2056	0.95
MET_MATCH					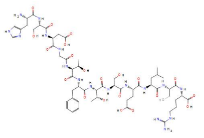	440.2052	440.2056	0.95
MET_MATCH					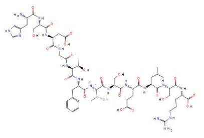	440.8711	440.8775	14.37
MET_MATCH					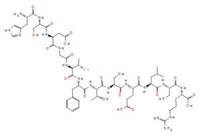	440.8711	440.8775	14.37

Metabolite: M1 -1702 RT=0.49

Type	score	sub. m/z observed	sub. m/z calculated	sub ppm		met. m/z observed	met. m/z calculated	met. ppm
MET_MATCH					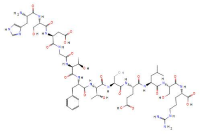	440.8711	440.8775	14.37
MET_MATCH					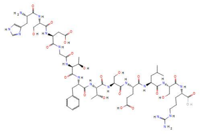	440.8711	440.8775	14.37
MET_MATCH					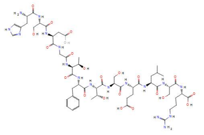	440.8711	440.8775	14.37
MET_MATCH					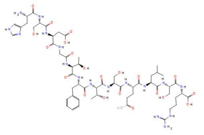	440.8711	440.8775	14.37
MET_MATCH					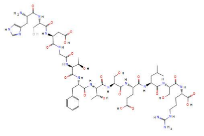	440.8711	440.8775	14.37
MET_MATCH					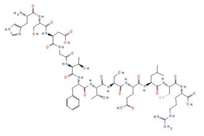	440.8711	440.8775	14.37
MET_MATCH					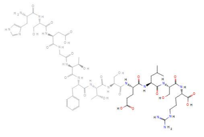	504.2776	504.2776	0.03
MET_MATCH					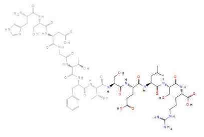	591.3091	591.3097	1.02
MET_MATCH					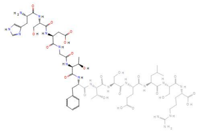	617.2676	617.2678	0.39

Metabolite: M1 -1702 RT=0.49

Type	score	sub. m/z observed	sub. m/z calculated	sub ppm		met. m/z observed	met. m/z calculated	met. ppm
MET_MATCH					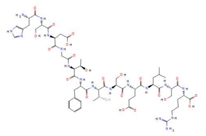	659.8045	659.8047	0.34
MET_MATCH					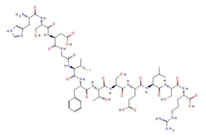	659.8045	659.8047	0.34
MET_MATCH					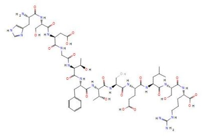	659.8045	659.8047	0.34
MET_MATCH					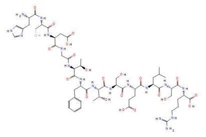	659.8045	659.8047	0.34
MET_MATCH					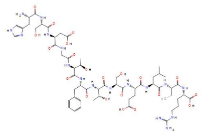	659.8045	659.8047	0.34
MET_MATCH					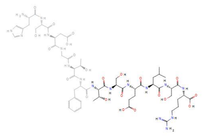	692.3575	692.3573	-0.18
MET_MATCH					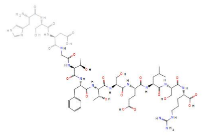	997.4917	997.4949	3.16
MET_MATCH					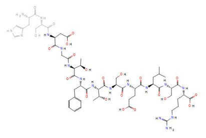	1112.5192	1112.5218	2.39
MET_MATCH					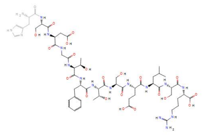	1199.5512	1199.5539	2.23

MS (+) FT

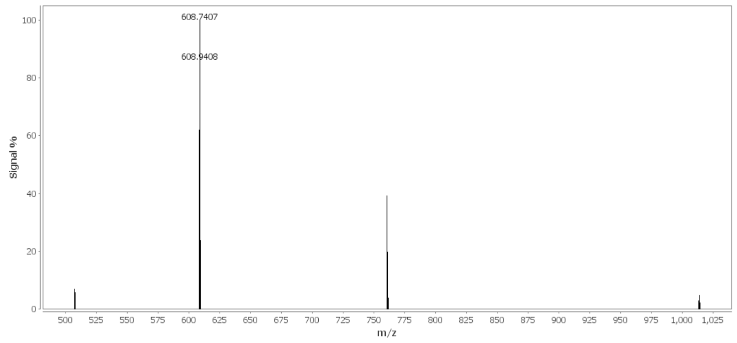

MS (+) FT

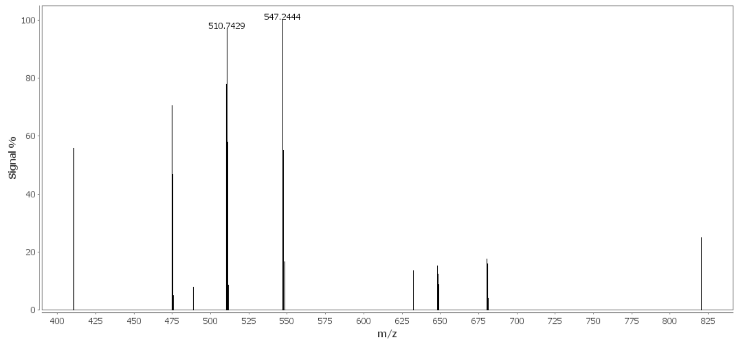

MS2 (+) FT activ = HCD:ce =

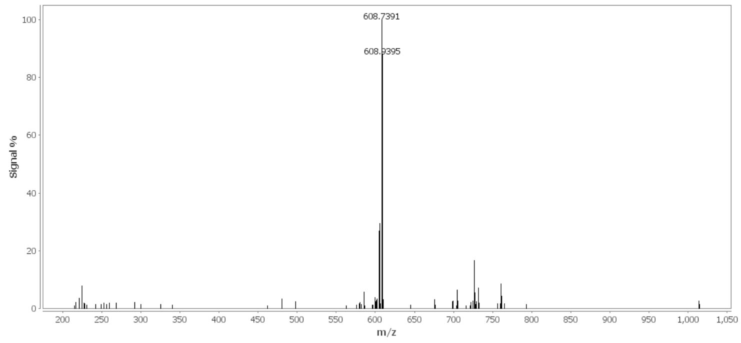

MS2 (+) FT activ = HCD:ce =

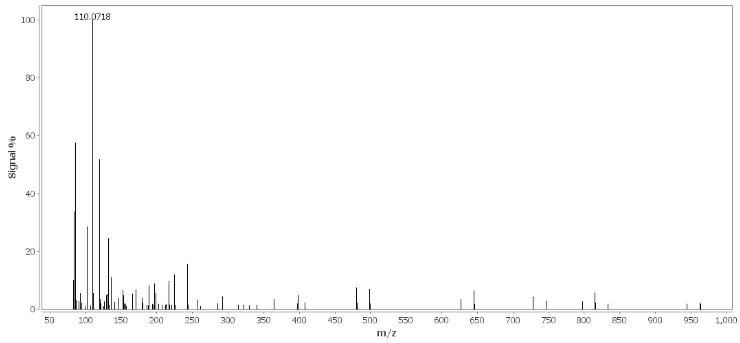

Metabolite: M2 -1945 RT=1.65

Type	score	sub. m/z observed	sub. m/z calculated	sub ppm		met. m/z observed	met. m/z calculated	met. ppm
MATCH	162.0	608.5406	608.5379	-4.37	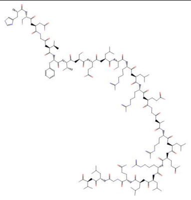 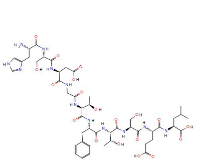	547.2444	547.2435	-1.70
					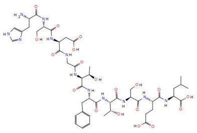	547.2444	547.2435	-1.70
MATCH	124.7	760.4259	760.4206	-6.98	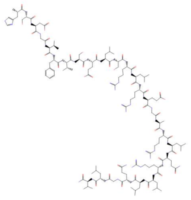 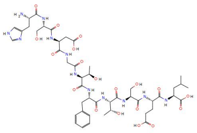	547.2444	547.2435	-1.70
					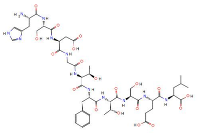	547.2444	547.2435	-1.70
MATCH	11.9	217.0822	217.0819	-1.54	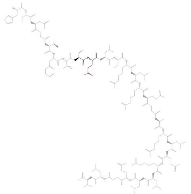 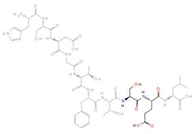	217.0820	217.0819	-0.38

Metabolite: M2 -1945 RT=1.65

Type	score	sub. m/z observed	sub. m/z calculated	sub ppm		met. m/z observed	met. m/z calculated	met. ppm
MATCH	11.9	217.0822	217.0819	-1.54	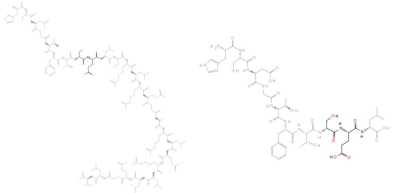	217.0820	217.0819	-0.38
MATCH	19.9	225.0986	225.0982	-1.51	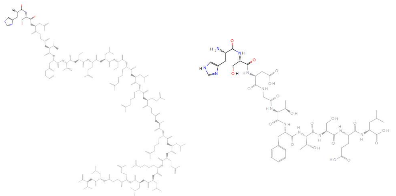	225.0985	225.0982	-1.12
MATCH	2.4	249.1230	249.1234	1.57	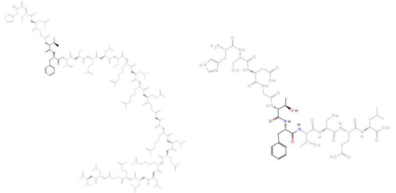	125.0715	125.0653	-49.2
MATCH	2.4	249.1230	249.1234	1.57	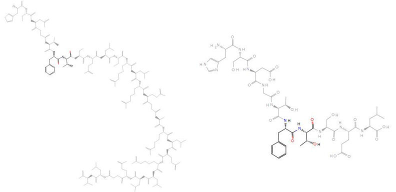	125.0715	125.0653	-49.2
MATCH	2.4	249.1230	249.1234	1.57	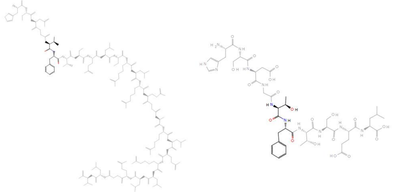	125.0715	125.0653	-49.2
MATCH	2.4	249.1230	249.1234	1.57	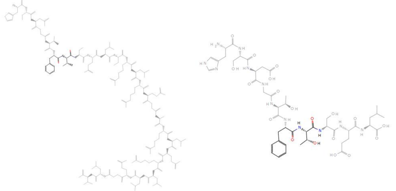	125.0715	125.0653	-49.2
MATCH	2.8	340.1247	340.1252	1.33	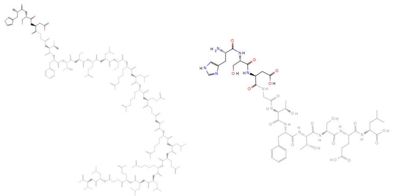	340.1241	340.1252	3.25
MATCH	10.7	480.1837	480.1837	-0.01	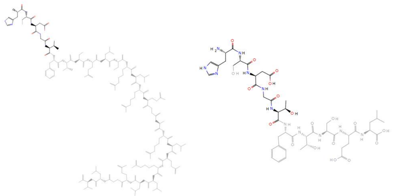	480.1831	480.1837	1.37
MATCH	10.7	480.1837	480.1837	-0.01	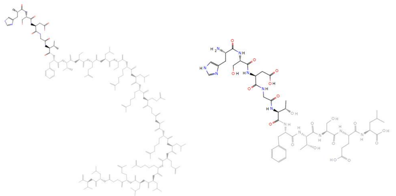	480.1831	480.1837	1.37

Metabolite: M2 -1945 RT=1.65

Type	score	sub. m/z observed	sub. m/z calculated	sub ppm		met. m/z observed	met. m/z calculated	met. ppm
MATCH	9.3	498.1939	498.1943	0.84	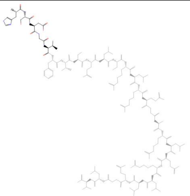 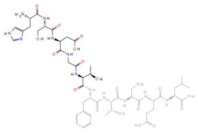	498.1947	498.1943	-0.74
MATCH	7.8	645.2637	645.2627	-1.57	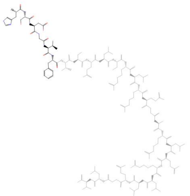 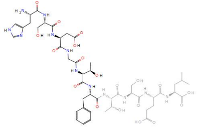	645.2613	645.2627	2.19
MISMATCH	-5.0	221.1288	221.1264	-10.7	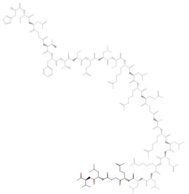	221.1285	221.1285	0.00
MET_MATCH					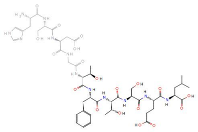	680.3186	680.3137	-7.19
MET_MATCH					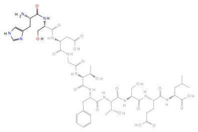	99.0557	99.0553	-4.35
MET_MATCH					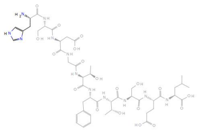	110.0718	110.0713	-4.63
MET_MATCH					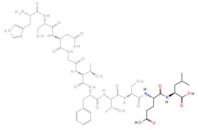	130.0654	130.0681	20.80
MET_MATCH					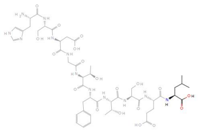	130.0866	130.0863	-2.81
MET_MATCH					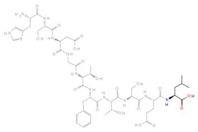	132.1022	132.1019	-2.03

Metabolite: M2 -1945 RT=1.65

Type	score	sub. m/z observed	sub. m/z calculated	sub ppm		met. m/z observed	met. m/z calculated	met. ppm
MET_MATCH					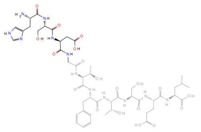	186.0909	186.0873	-19.3
MET_MATCH					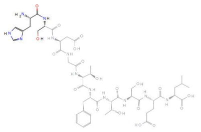	197.1036	197.1033	-1.47
MET_MATCH					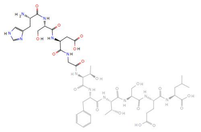	199.0716	199.0770	26.87
MET_MATCH					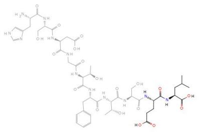	261.1498	261.1445	-20.4
MET_MATCH					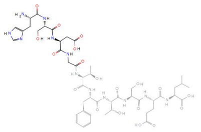	397.1463	397.1466	0.81
MET_MATCH					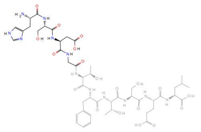	399.1659	399.1623	-8.97
MET_MATCH					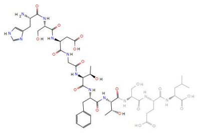	746.3046	746.3104	7.75
