# Supplementary material for: Software-aided approach to investigate peptide structure and metabolic susceptibility of amide bonds in peptide drugs based on high resolution mass spectrometry
Source: PLoS One. 2017 Nov 1;12(11):e0186461. doi: 10.1371/journal.pone.0186461 (PMC5665424; doi:10.1371/journal.pone.0186461)
Supplement: S1 File — (ZIP) [file pone.0186461.s007.zip › SFiles/S31_File.pdf]

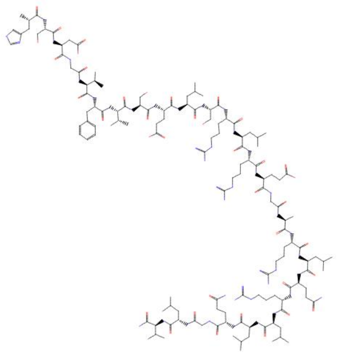

Secretin

Property name	Property value
Time	0min, 5min, 15min, 45min, 120min
Instrument	ThermoQAPlus
Matrix	elastase
Acquisition Mode	ddMS2

Chromatograms

Time=0min

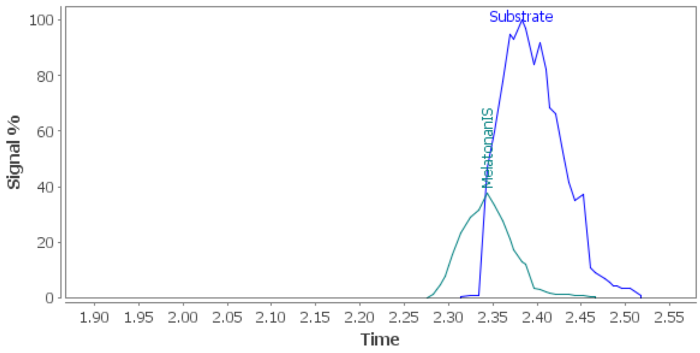

Time=5min

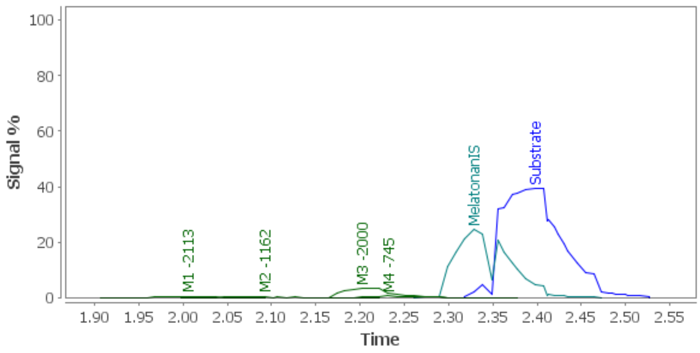

Time=15min

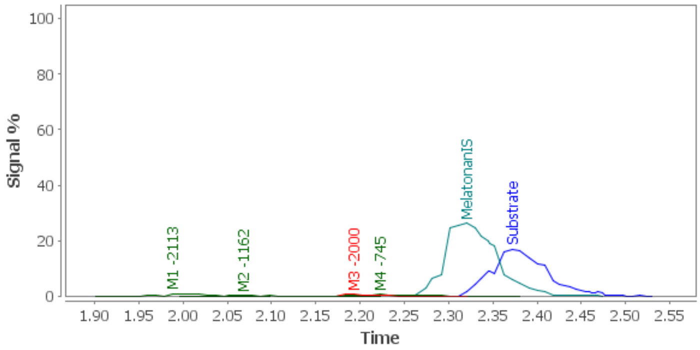

Time=45min

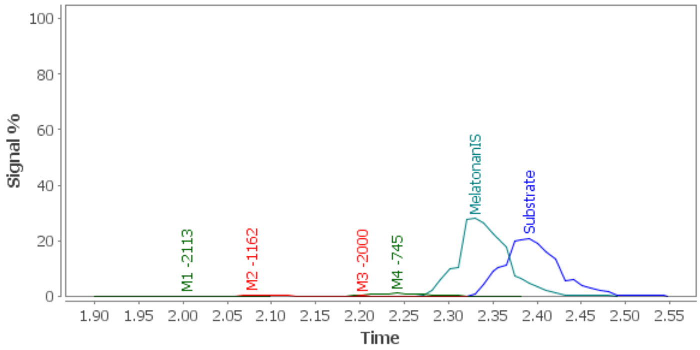

Time=120min

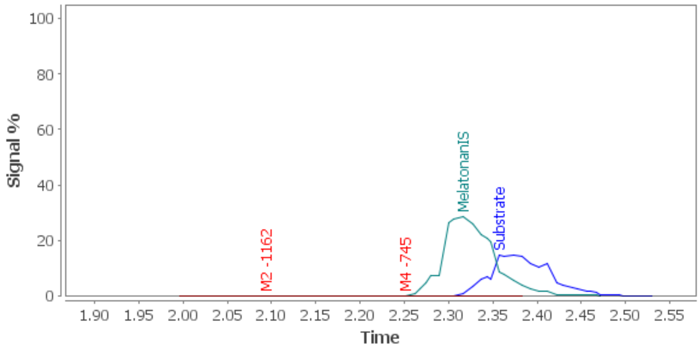

Custom Charts

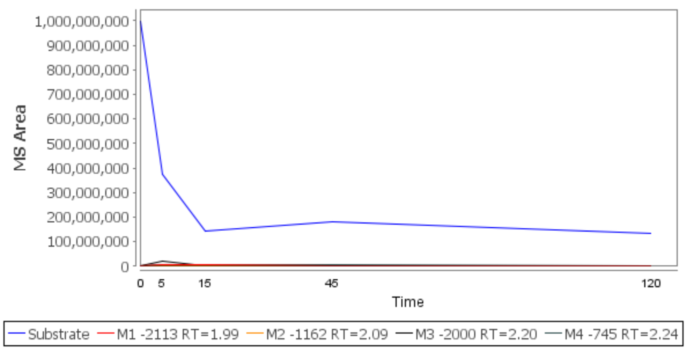

Fragmentation

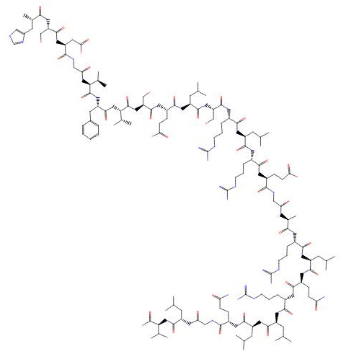

Secretin

MS (+) FT

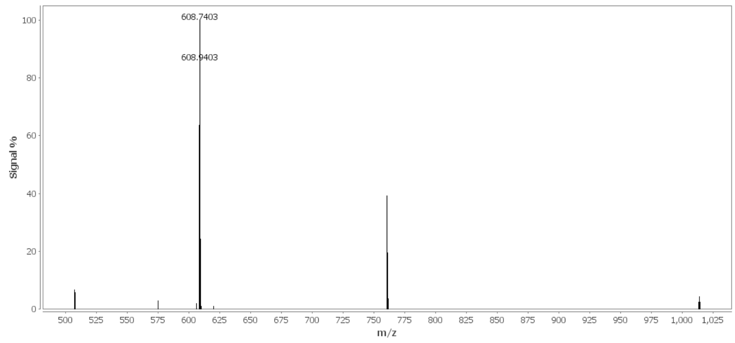

MS (+) FT

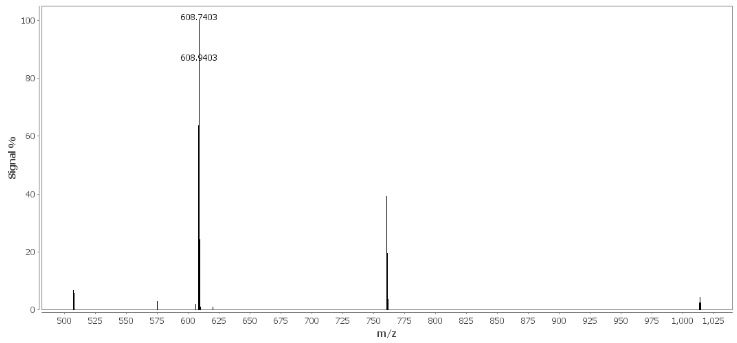

MS2 (+) FT activ = HCD:ce =

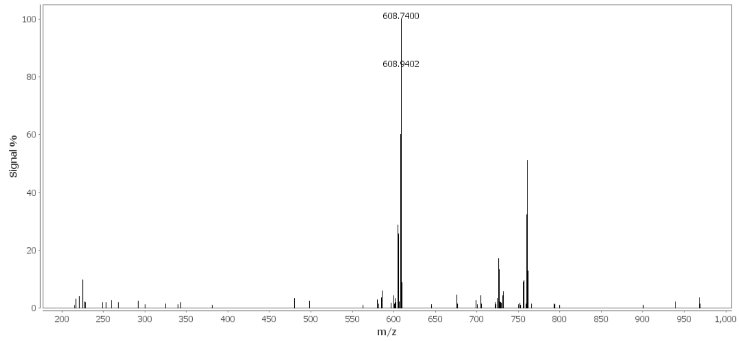

MS2 (+) FT activ = HCD:ce =

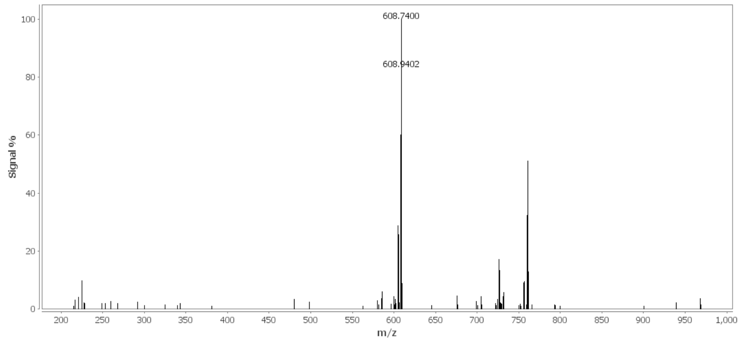

Metabolite: Substrate

Type	score	sub. m/z observed	sub. m/z calculated	sub ppm			met. m/z observed	met. m/z calculated	met. ppm
MATCH	102.3	1013.5658	1013.5584	-7.29	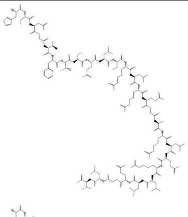	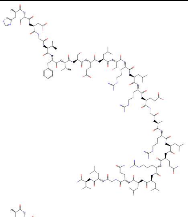	1013.5658	1013.5584	-7.29
MATCH	112.2	760.4258	760.4206	-6.83	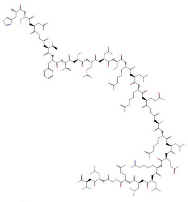	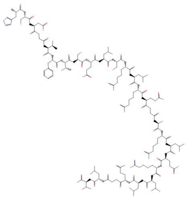	760.4258	760.4206	-6.83
MATCH	122.3	760.4250	760.4206	-5.83	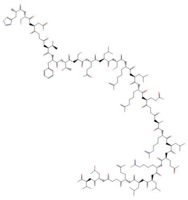	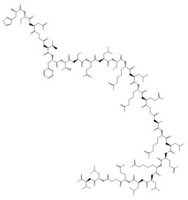	760.4250	760.4206	-5.83

Metabolite: Substrate

Type	score	sub. m/z observed	sub. m/z calculated	sub ppm			met. m/z observed	met. m/z calculated	met. ppm
MATCH	36.5	756.1710	756.1640	-9.27	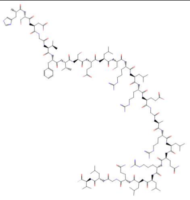	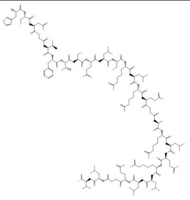	756.1710	756.1640	-9.27
MATCH	36.5	756.1710	756.1640	-9.27	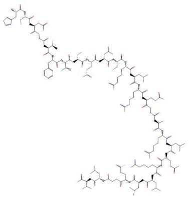	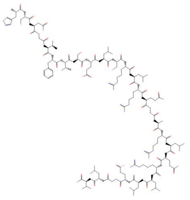	756.1710	756.1640	-9.27
MATCH	36.5	756.1710	756.1640	-9.27	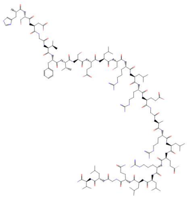	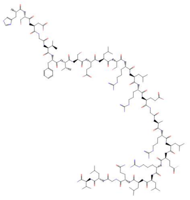	756.1710	756.1640	-9.27
MISMATCH	-11.8	755.9237	755.9180	-7.61	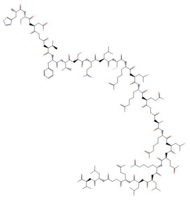	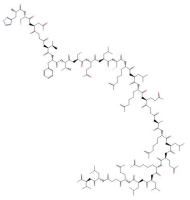	755.9237	755.9180	-7.61
MISMATCH	-11.8	755.9237	755.9180	-7.61	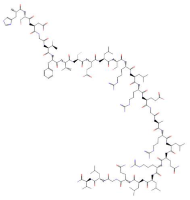	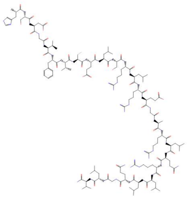	755.9237	755.9180	-7.61
MISMATCH	39.3	731.3960	731.3969	1.17	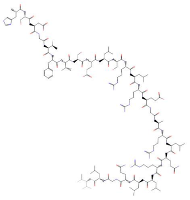	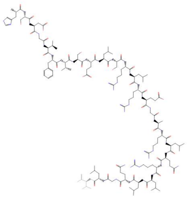	731.3960	731.3969	1.17
MATCH	79.4	724.4044	724.3981	-8.60	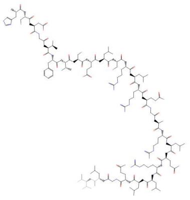	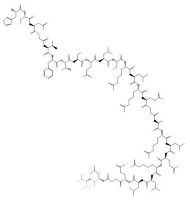	724.4044	724.3981	-8.60
MISMATCH	-21.6	698.8711	698.8692	-2.72	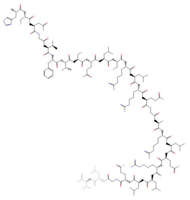	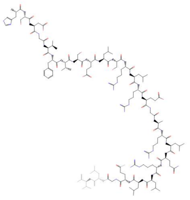	698.8711	698.8692	-2.72
MISMATCH	-21.6	698.8711	698.8692	-2.72	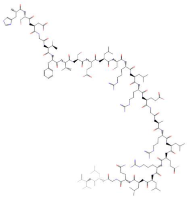	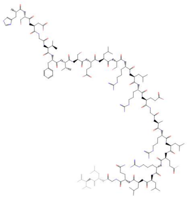	698.8711	698.8692	-2.72

Metabolite: Substrate

Type	score	sub. m/z observed	sub. m/z calculated	sub ppm			met. m/z observed	met. m/z calculated	met. ppm
MATCH	163.0	608.5402	608.5379	-3.65	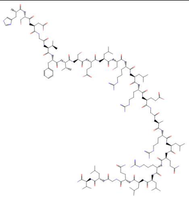	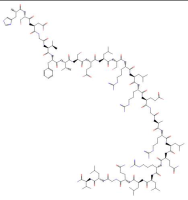	608.5402	608.5379	-3.65
MATCH	143.2	608.5397	608.5379	-2.87	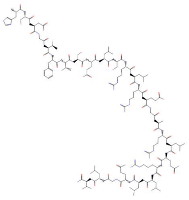	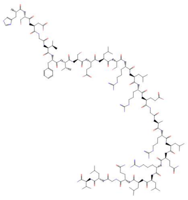	608.5397	608.5379	-2.87
MISMATCH	-18.2	604.9378	604.9358	-3.28	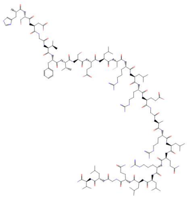	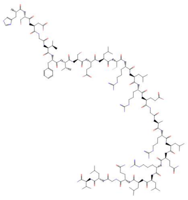	604.9378	604.9358	-3.28
MISMATCH	-18.2	604.9378	604.9358	-3.28	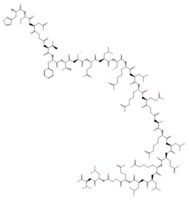	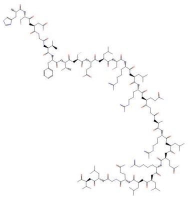	604.9378	604.9358	-3.28
MISMATCH	-5.2	585.3190	585.3190	-0.02	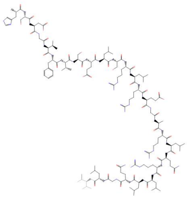	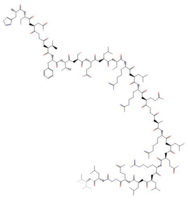	585.3190	585.3190	-0.02
MISMATCH	-75.5	579.7240	579.7200	-6.96	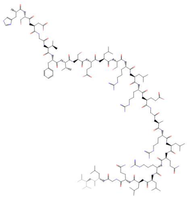	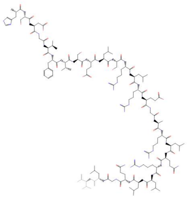	579.7240	579.7200	-6.96
MATCH	7.6	381.2240	381.2245	1.17	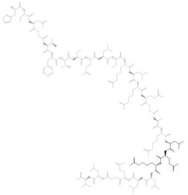	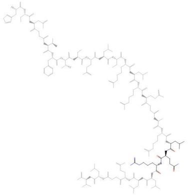	381.2240	381.2245	1.17
MATCH	7.6	381.2240	381.2245	1.17	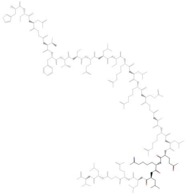	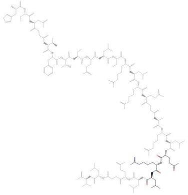	381.2240	381.2245	1.17
MISMATCH	-9.0	268.1409	268.1397	-4.18	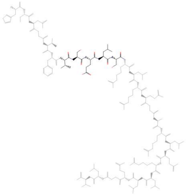	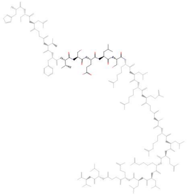	268.1409	268.1397	-4.18

Metabolite: Substrate

Type	score	sub. m/z observed	sub. m/z calculated	sub ppm			met. m/z observed	met. m/z calculated	met. ppm
MATCH	14.9	268.1409	268.1404	-1.68	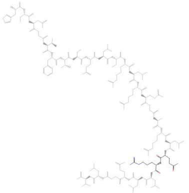	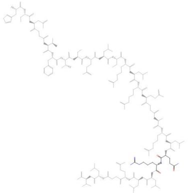	268.1409	268.1404	-1.68
MATCH	26.5	242.1495	242.1499	1.53	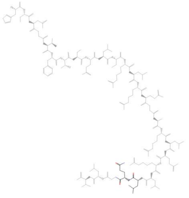	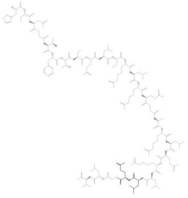	242.1495	242.1499	1.53
MATCH	26.5	242.1495	242.1499	1.53	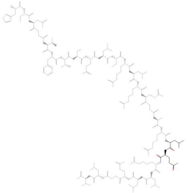	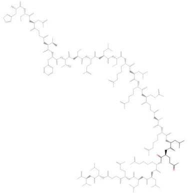	242.1495	242.1499	1.53
MATCH	2.6	242.1495	242.1544	20.09	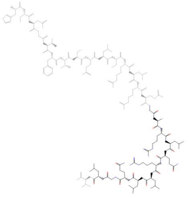	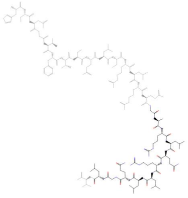	242.1495	242.1544	20.09
MATCH	26.5	242.1495	242.1499	1.53	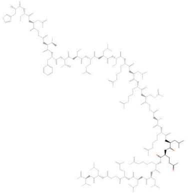	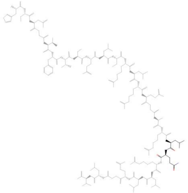	242.1495	242.1499	1.53
MATCH	2.6	242.1495	242.1544	20.09	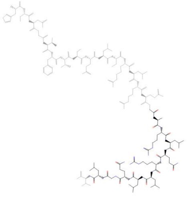	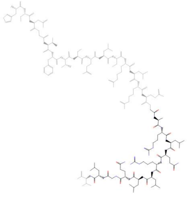	242.1495	242.1544	20.09
MATCH	26.5	242.1495	242.1555	24.73	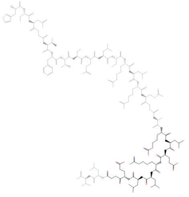	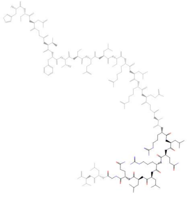	242.1495	242.1555	24.73
MATCH	26.5	242.1495	242.1499	1.53	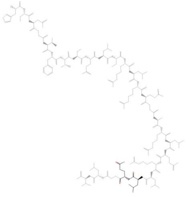	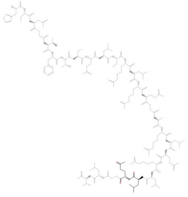	242.1495	242.1499	1.53
MATCH	26.5	242.1495	242.1555	24.73	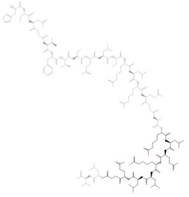	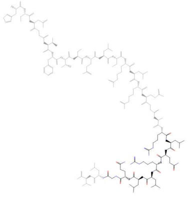	242.1495	242.1555	24.73

Metabolite: Substrate

Type	score	sub. m/z observed	sub. m/z calculated	sub ppm			met. m/z observed	met. m/z calculated	met. ppm
MATCH	2.6	242.1495	242.1594	40.86	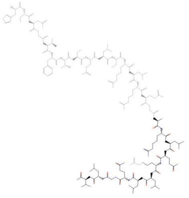	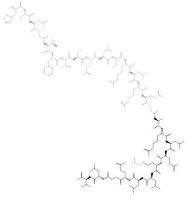	242.1495	242.1594	40.86
MATCH	2.6	242.1495	242.1594	40.86	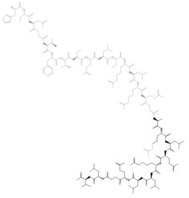	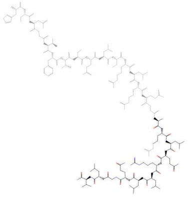	242.1495	242.1594	40.86
MATCH	37.6	225.1716	225.1710	-2.56	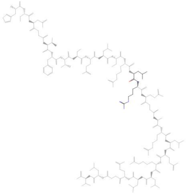	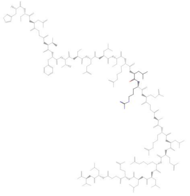	225.1716	225.1710	-2.56
MATCH	37.6	225.1716	225.1710	-2.56	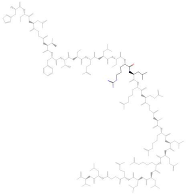	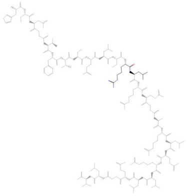	225.1716	225.1710	-2.56
MATCH	37.6	225.1716	225.1710	-2.56	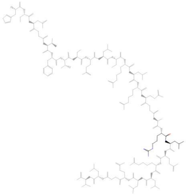	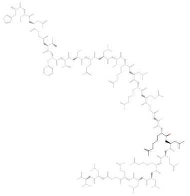	225.1716	225.1710	-2.56
MATCH	37.6	225.1716	225.1710	-2.56	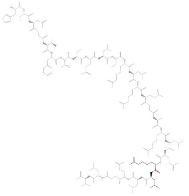	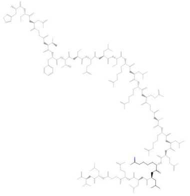	225.1716	225.1710	-2.56
MATCH	16.2	217.0824	217.0819	-2.43	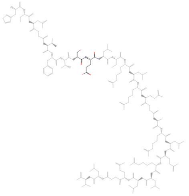	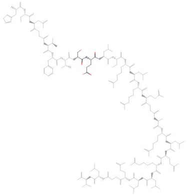	217.0824	217.0819	-2.43

MS (+) FT

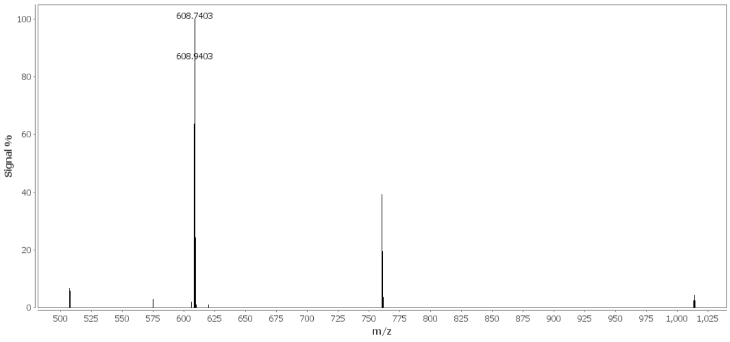

MS (+) FT

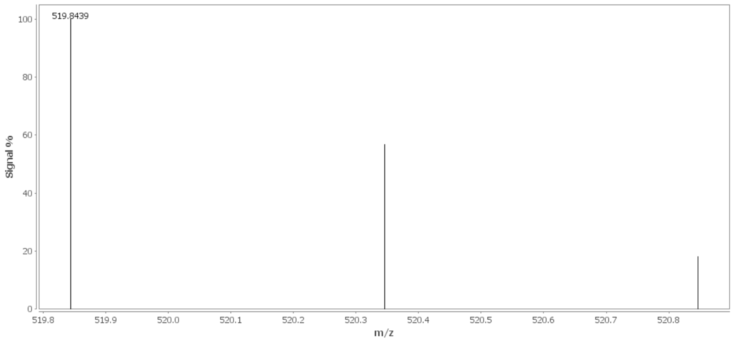

MS2 (+) FT activ = HCD:ce =

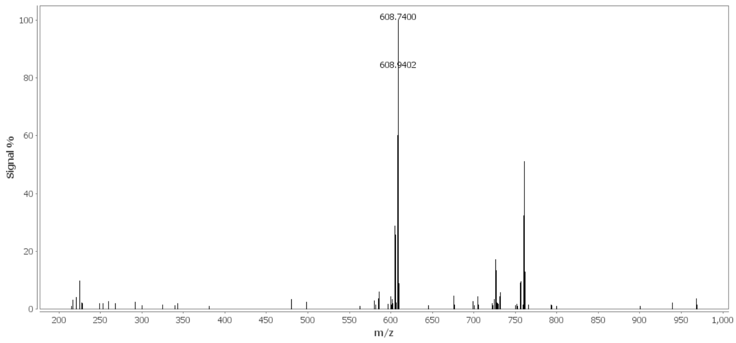

MS2 (+) FT activ = HCD:ce =

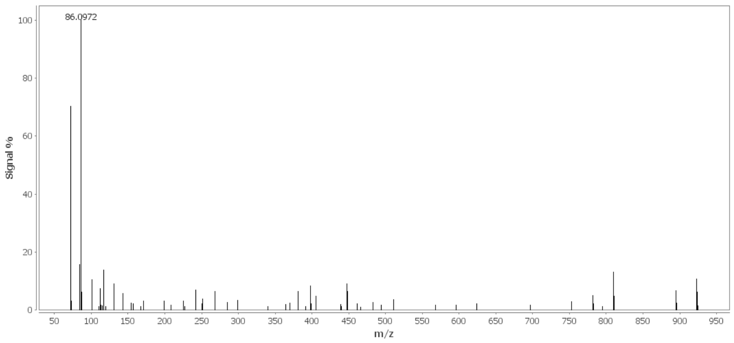

Metabolite: M3 -2000 RT=2.20

Type	score	sub. m/z observed	sub. m/z calculated	sub ppm			met. m/z observed	met. m/z calculated	met. ppm
MATCH	163.0	608.5402	608.5379	-3.65	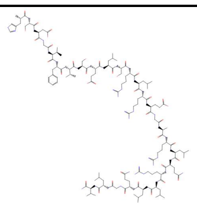	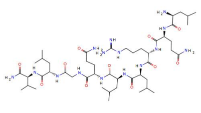	519.8439	519.8428	-2.23
						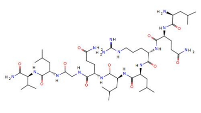	519.8439	519.8428	-2.23
						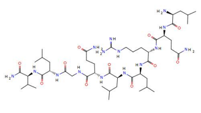	519.8439	519.8428	-2.23
MATCH	122.3	760.4250	760.4206	-5.83	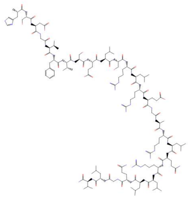	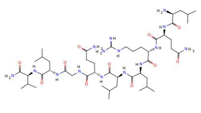	519.8439	519.8428	-2.23
						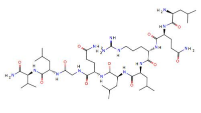	519.8439	519.8428	-2.23
						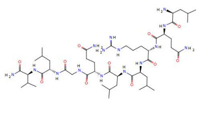	519.8439	519.8428	-2.23
MATCH	102.3	1013.5658	1013.5584	-7.29	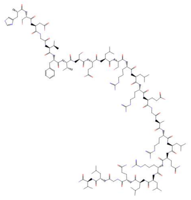	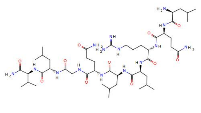	519.8439	519.8428	-2.23

Metabolite: M3 -2000 RT=2.20

Type	score	sub. m/z observed	sub. m/z calculated	sub ppm		met. m/z observed	met. m/z calculated	met. ppm
					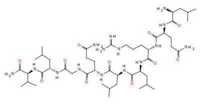	519.8439	519.8428	-2.23
					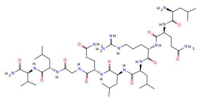	519.8439	519.8428	-2.23
MATCH	6.0	225.1716	225.1710	-2.56	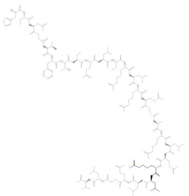 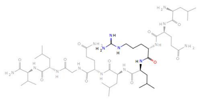	225.1711	225.1710	-0.55
MATCH	8.2	242.1495	242.1499	1.53	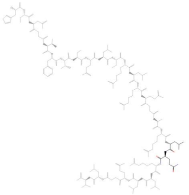 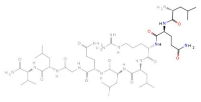	242.1502	242.1499	-1.26
MATCH	8.2	242.1495	242.1499	1.53	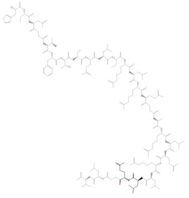 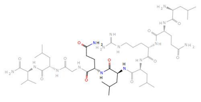	242.1502	242.1499	-1.26
MATCH	8.2	242.1495	242.1499	1.53	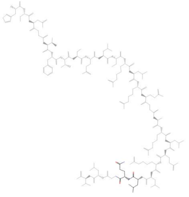 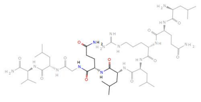	242.1502	242.1499	-1.26
MATCH	9.0	268.1409	268.1404	-1.68	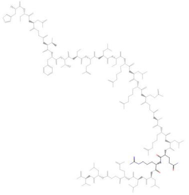 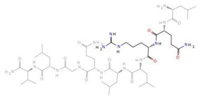	268.1413	268.1404	-3.16
MATCH	7.6	381.2240	381.2245	1.17	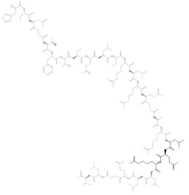 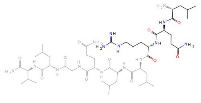	381.2246	381.2245	-0.32
MATCH	7.6	381.2240	381.2245	1.17	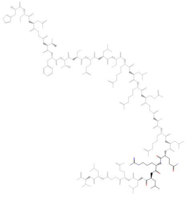 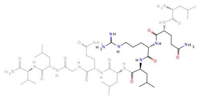	381.2246	381.2245	-0.32

Metabolite: M3 -2000 RT=2.20

Type	score	sub. m/z observed	sub. m/z calculated	sub ppm		met. m/z observed	met. m/z calculated	met. ppm
MISMATCH	-9.0	268.1409	268.1397	-4.18	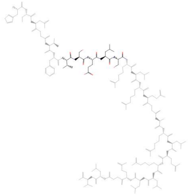	268.1413	268.1413	0.00
MISMATCH	-5.2	585.3190	585.3190	-0.02	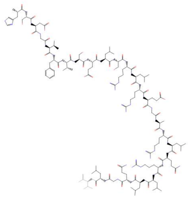	461.7931	461.7931	0.00
MISMATCH	-13.9	585.3190	585.3190	-0.02	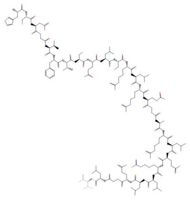	922.5835	922.5835	0.00
MISMATCH	-6.4	731.3960	731.3969	1.17	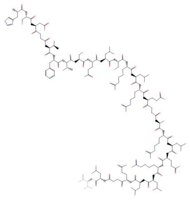	461.7931	461.7931	0.00
MISMATCH	-15.0	731.3960	731.3969	1.17	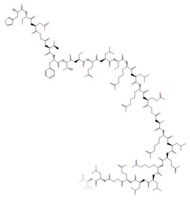	922.5835	922.5835	0.00
MISMATCH	-14.1	756.1710	756.1640	-9.27	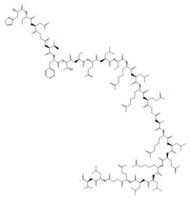	511.3353	511.3353	0.00
MET_MATCH					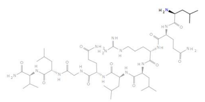	86.0972	86.0964	-8.42
MET_MATCH					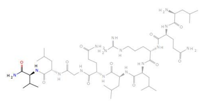	115.0870	115.0866	-3.79
MET_MATCH					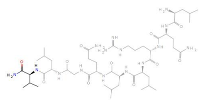	117.1027	117.1022	-3.71

Metabolite: M3 -2000 RT=2.20

Type	score	sub. m/z observed	sub. m/z calculated	sub ppm		met. m/z observed	met. m/z calculated	met. ppm
MET_MATCH					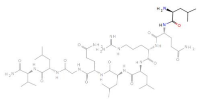	131.1181	131.1179	-1.27
MET_MATCH					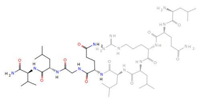	208.1445	208.1368	-36.7
MET_MATCH					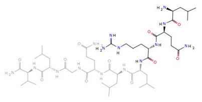	208.1445	208.1424	-9.72
MET_MATCH					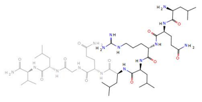	298.7158	298.7158	-0.23
MET_MATCH					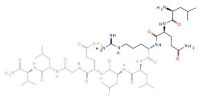	370.2560	370.2561	0.29
MET_MATCH					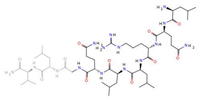	391.2557	391.2558	0.24
MET_MATCH					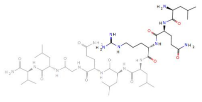	398.2503	398.2510	1.75
MET_MATCH					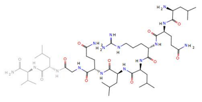	405.2538	405.2532	-1.46
MET_MATCH					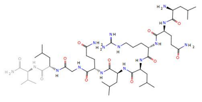	447.7974	447.7978	0.94

Metabolite: M3 -2000 RT=2.20

Type	score	sub. m/z observed	sub. m/z calculated	sub ppm		met. m/z observed	met. m/z calculated	met. ppm
MET_MATCH					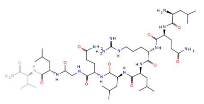	461.7931	461.7953	4.80
MET_MATCH					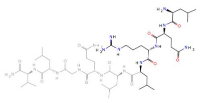	483.3394	483.3402	1.63
MET_MATCH					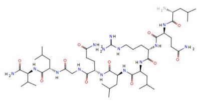	511.3353	511.3295	-11.4
MET_MATCH					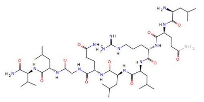	511.3353	511.3295	-11.4
MET_MATCH					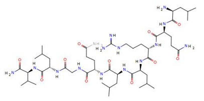	511.3353	511.3295	-11.4
MET_MATCH					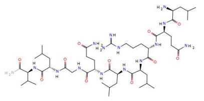	511.3353	511.3295	-11.4
MET_MATCH					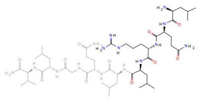	511.3353	511.3351	-0.48
MET_MATCH					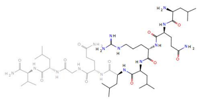	596.4219	596.4242	3.85
MET_MATCH					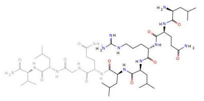	624.4196	624.4192	-0.63

Metabolite: M3 -2000 RT=2.20

Type	score	sub. m/z observed	sub. m/z calculated	sub ppm		met. m/z observed	met. m/z calculated	met. ppm
MET_MATCH					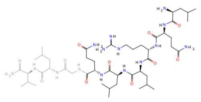	752.4760	752.4777	2.32
MET_MATCH					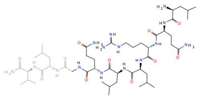	781.5027	781.5043	2.03
MET_MATCH					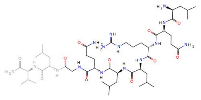	809.4987	809.4992	0.67
MET_MATCH					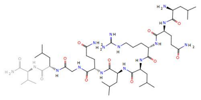	894.5895	894.5883	-1.27
MET_MATCH					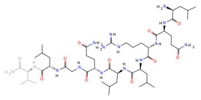	922.5835	922.5833	-0.29

MS (+) FT

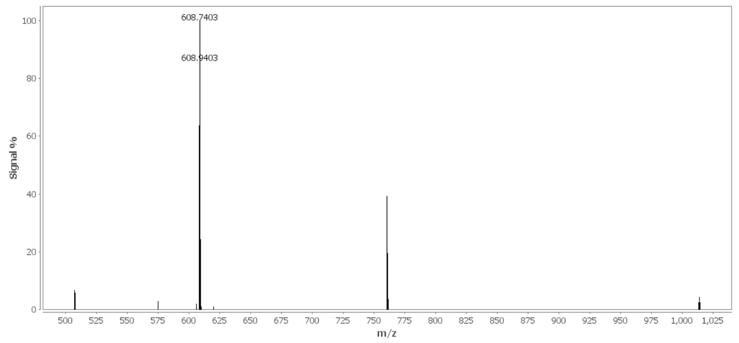

MS (+) FT

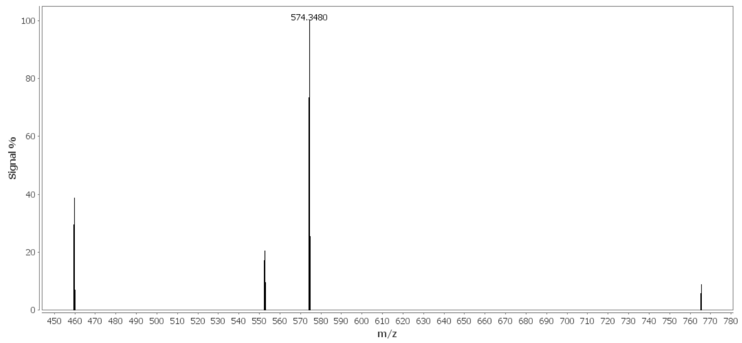

MS2 (+) FT activ = HCD:ce =

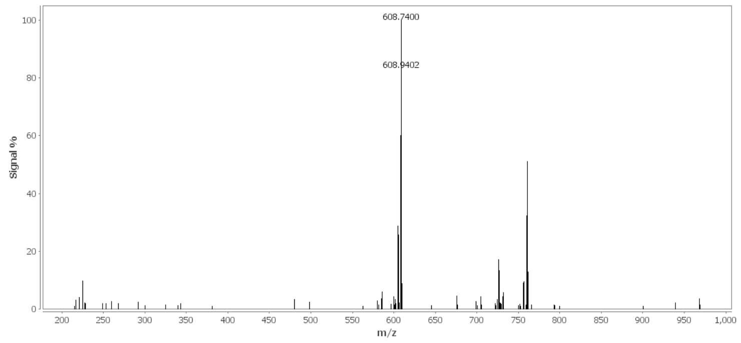

MS2 (+) FT activ = HCD:ce =

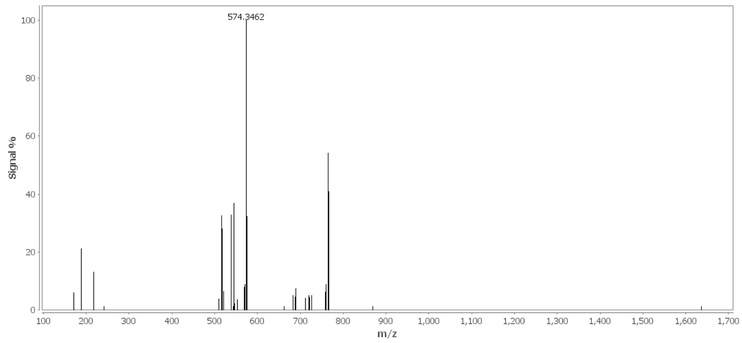

Metabolite: M4 -745 RT=2.24

Type	score	sub. m/z observed	sub. m/z calculated	sub ppm			met. m/z observed	met. m/z calculated	met. ppm
MATCH	92.3	608.5402	608.5379	-3.65	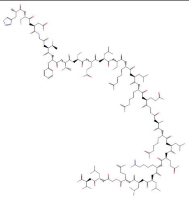	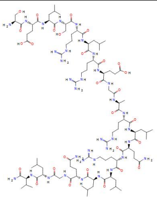	459.4783	459.4773	-2.10
MATCH	92.3	608.5402	608.5379	-3.65	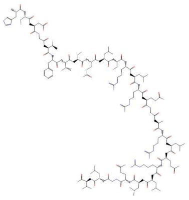	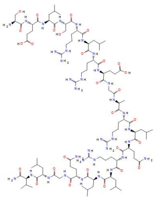	459.4783	459.4773	-2.10
MATCH	92.3	608.5402	608.5379	-3.65	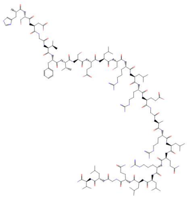	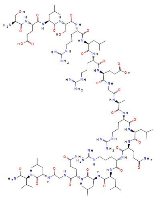	459.4783	459.4773	-2.10
MATCH	136.2	608.5402	608.5379	-3.65	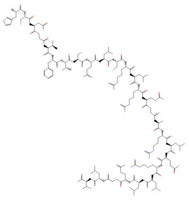	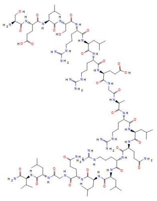	574.0963	574.0948	-2.54
MATCH	136.2	608.5402	608.5379	-3.65	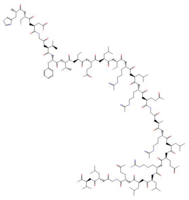	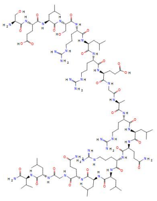	574.0963	574.0948	-2.54
MATCH	136.2	608.5402	608.5379	-3.65	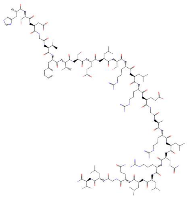	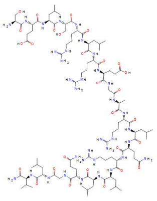	574.0963	574.0948	-2.54
MATCH	68.8	608.5402	608.5379	-3.65	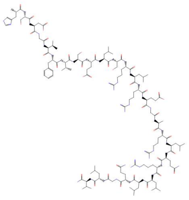	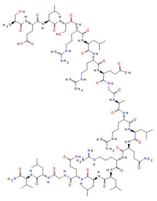	765.1258	765.1240	-2.30
MATCH	68.8	608.5402	608.5379	-3.65	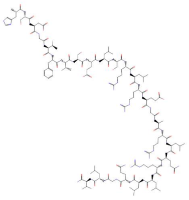	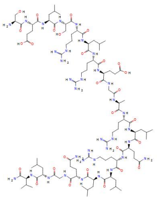	765.1258	765.1240	-2.30
MATCH	68.8	608.5402	608.5379	-3.65	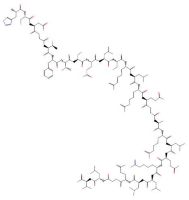	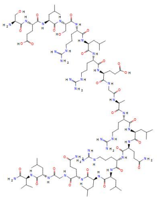	765.1258	765.1240	-2.30

Metabolite: M4 -745 RT=2.24

Type	score	sub. m/z observed	sub. m/z calculated	sub ppm			met. m/z observed	met. m/z calculated	met. ppm
MATCH	51.7	760.4250	760.4206	-5.83	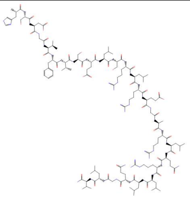	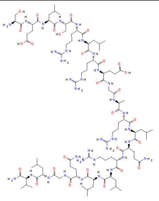	459.4783	459.4773	-2.10
MATCH	51.7	760.4250	760.4206	-5.83	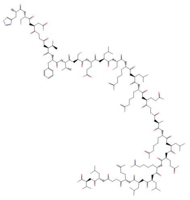	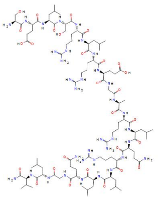	459.4783	459.4773	-2.10
MATCH	51.7	760.4250	760.4206	-5.83	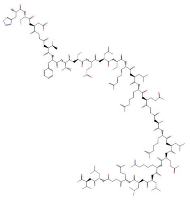	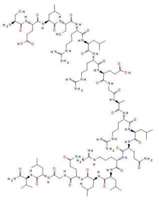	459.4783	459.4773	-2.10
MATCH	95.6	760.4250	760.4206	-5.83	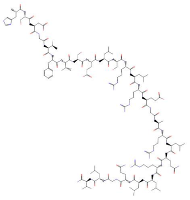	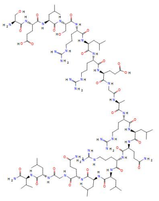	574.0963	574.0948	-2.54
MATCH	95.6	760.4250	760.4206	-5.83	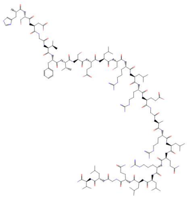	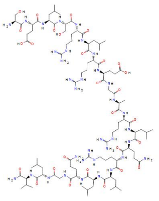	574.0963	574.0948	-2.54
MATCH	95.6	760.4250	760.4206	-5.83	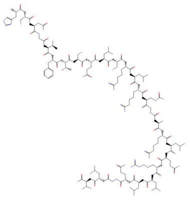	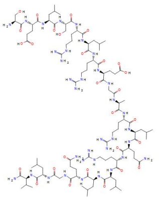	574.0963	574.0948	-2.54
MATCH	28.1	760.4250	760.4206	-5.83	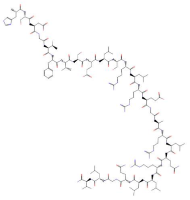	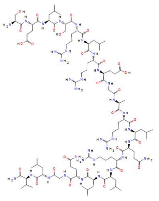	765.1258	765.1240	-2.30
MATCH	28.1	760.4250	760.4206	-5.83	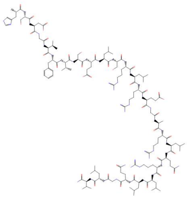	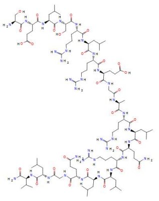	765.1258	765.1240	-2.30
MATCH	28.1	760.4250	760.4206	-5.83	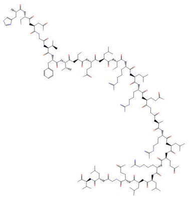	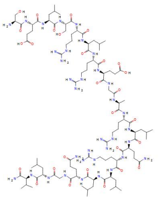	765.1258	765.1240	-2.30

Metabolite: M4 -745 RT=2.24

Type	score	sub. m/z observed	sub. m/z calculated	sub ppm			met. m/z observed	met. m/z calculated	met. ppm
MATCH	31.7	1013.5658	1013.5584	-7.29	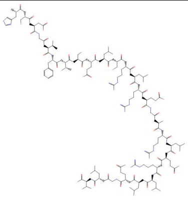	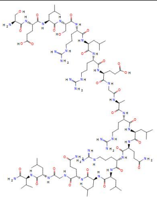	459.4783	459.4773	-2.10
MATCH	31.7	1013.5658	1013.5584	-7.29	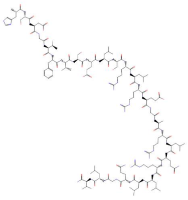	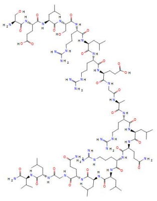	459.4783	459.4773	-2.10
MATCH	31.7	1013.5658	1013.5584	-7.29	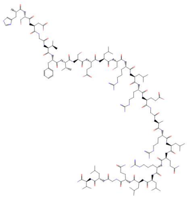	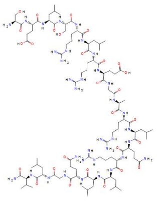	459.4783	459.4773	-2.10
MATCH	75.6	1013.5658	1013.5584	-7.29	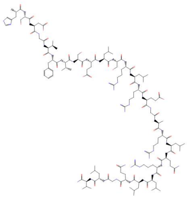	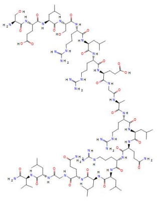	574.0963	574.0948	-2.54
MATCH	75.6	1013.5658	1013.5584	-7.29	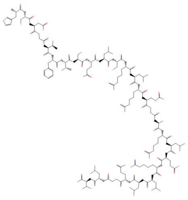	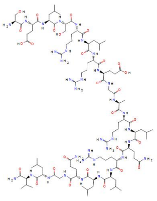	574.0963	574.0948	-2.54
MATCH	75.6	1013.5658	1013.5584	-7.29	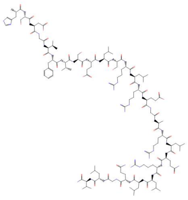	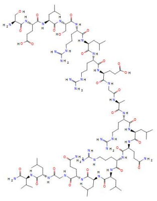	574.0963	574.0948	-2.54
MATCH	8.1	1013.5658	1013.5584	-7.29	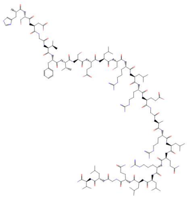	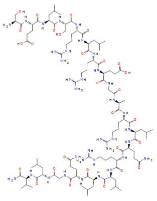	765.1258	765.1240	-2.30
MATCH	8.1	1013.5658	1013.5584	-7.29	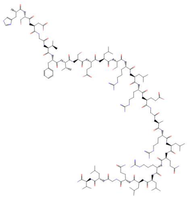	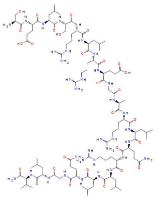	765.1258	765.1240	-2.30
MATCH	8.1	1013.5658	1013.5584	-7.29	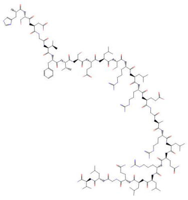	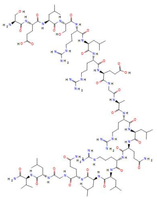	765.1258	765.1240	-2.30

Metabolite: M4 -745 RT=2.24

Type	score	sub. m/z observed	sub. m/z calculated	sub ppm			met. m/z observed	met. m/z calculated	met. ppm
MATCH	16.2	217.0824	217.0819	-2.43	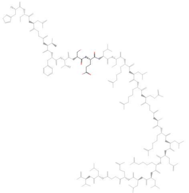	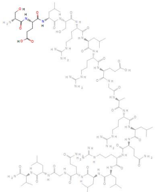	217.0815	217.0819	1.97
MATCH	2.6	242.1495	242.1499	1.53	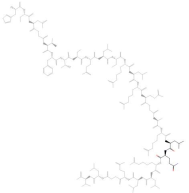	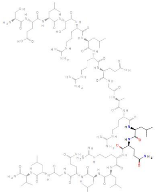	242.1501	242.1499	-0.69
MATCH	2.6	242.1495	242.1499	1.53	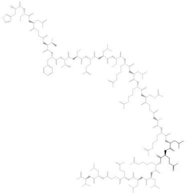	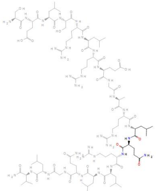	242.1501	242.1499	-0.69
MATCH	2.6	242.1495	242.1499	1.53	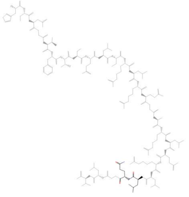	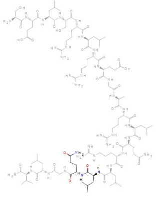	242.1501	242.1499	-0.69
MATCH	2.6	242.1495	242.1499	1.53	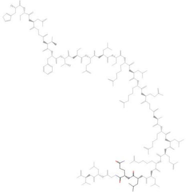	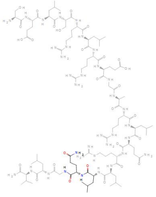	242.1501	242.1499	-0.69
MATCH	2.6	242.1495	242.1544	20.09	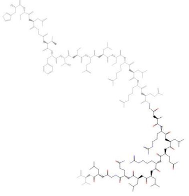	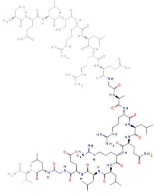	242.1501	242.1544	17.86
MATCH	2.6	242.1495	242.1544	20.09	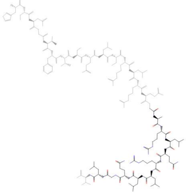	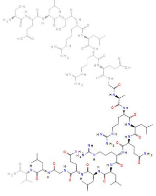	242.1501	242.1544	17.86
MATCH	2.6	242.1495	242.1555	24.73	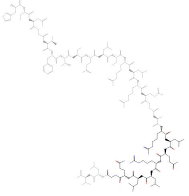	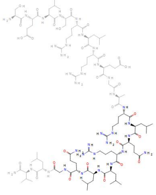	242.1501	242.1555	22.51
MATCH	2.6	242.1495	242.1555	24.73	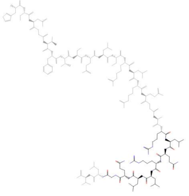	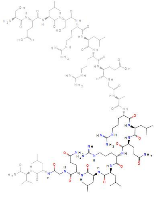	242.1501	242.1555	22.51

Metabolite: M4 -745 RT=2.24

Type	score	sub. m/z observed	sub. m/z calculated	sub ppm			met. m/z observed	met. m/z calculated	met. ppm
MATCH	2.6	242.1495	242.1594	40.86	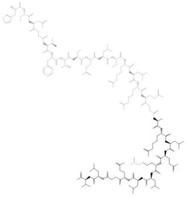	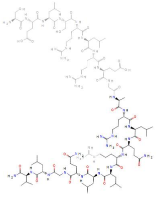	242.1501	242.1594	38.64
MATCH	2.6	242.1495	242.1594	40.86	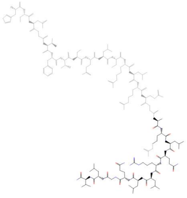	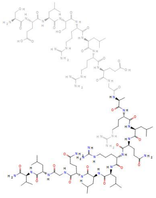	242.1501	242.1594	38.64
MATCH	117.3	608.5397	608.5379	-2.87	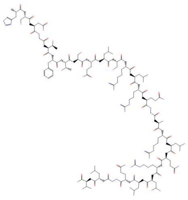	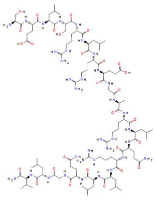	574.0946	574.0948	0.40
MATCH	117.3	608.5397	608.5379	-2.87	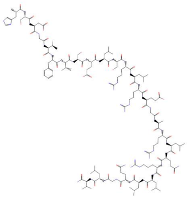	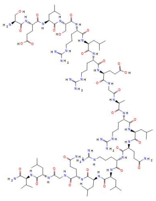	574.0946	574.0948	0.40
MATCH	116.8	608.5397	608.5379	-2.87	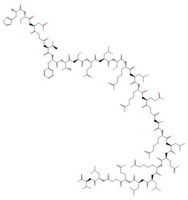	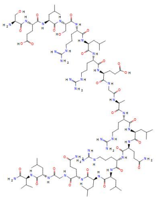	765.1252	765.1240	-1.52
MATCH	116.8	608.5397	608.5379	-2.87	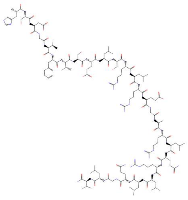	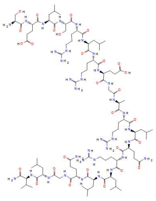	765.1252	765.1240	-1.52
MATCH	33.6	724.4044	724.3981	-8.60	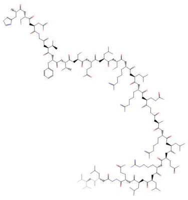	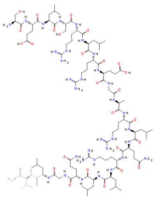	538.0737	538.0724	-2.55
MATCH	27.7	731.3960	731.3969	1.17	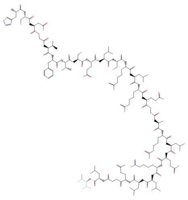	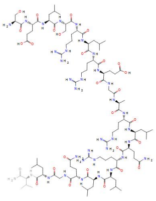	545.0722	545.0711	-2.01
MATCH	18.4	756.1710	756.1640	-9.27	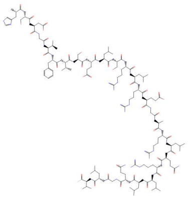	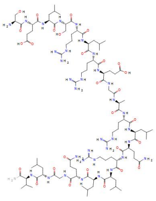	569.8411	569.8382	-5.14

Metabolite: M4 -745 RT=2.24

Type	score	sub. m/z observed	sub. m/z calculated	sub ppm			met. m/z observed	met. m/z calculated	met. ppm
MATCH	18.4	756.1710	756.1640	-9.27	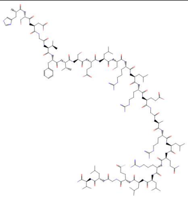	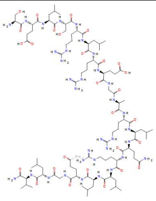	569.8411	569.8382	-5.14
MATCH	18.4	756.1710	756.1640	-9.27	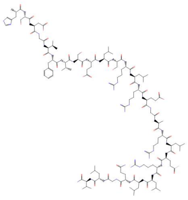	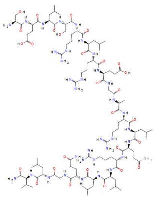	569.8411	569.8382	-5.14
MATCH	86.4	760.4258	760.4206	-6.83	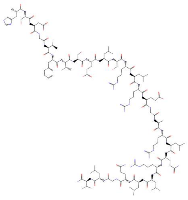	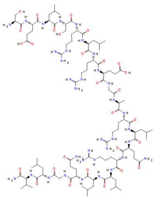	574.0946	574.0948	0.40
MATCH	86.4	760.4258	760.4206	-6.83	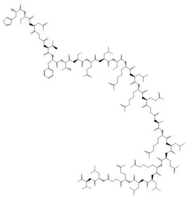	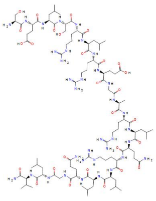	574.0946	574.0948	0.40
MATCH	85.9	760.4258	760.4206	-6.83	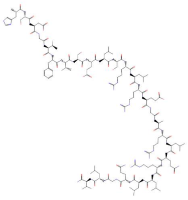	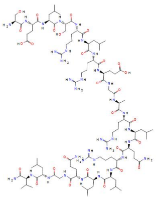	765.1252	765.1240	-1.52
MATCH	85.9	760.4258	760.4206	-6.83	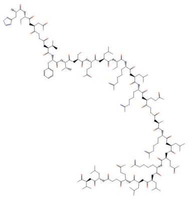	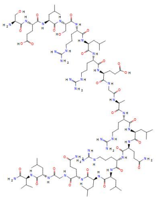	765.1252	765.1240	-1.52
MISMATCH	-8.2	585.3190	585.3190	-0.02	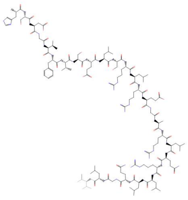		726.4256	726.4256	0.00
MISMATCH	-18.2	604.9378	604.9358	-3.28	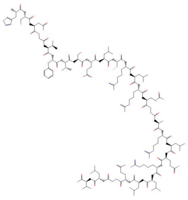		759.1222	759.1222	0.00
MISMATCH	-9.3	731.3960	731.3969	1.17	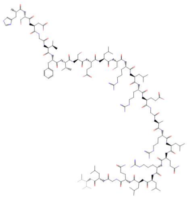		726.4256	726.4256	0.00

Metabolite: M4 -745 RT=2.24

Type	score	sub. m/z observed	sub. m/z calculated	sub ppm		met. m/z observed	met. m/z calculated	met. ppm
MISMATCH	-11.8	755.9237	755.9180	-7.61	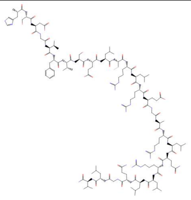	759.1222	759.1222	0.00
MET_MATCH					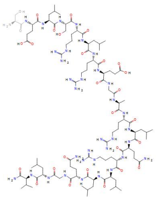	552.3384	552.3368	-2.93
MET_MATCH					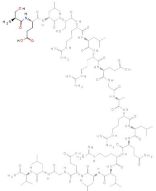	189.0870	189.0870	-0.07
MET_MATCH					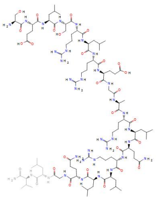	516.7996	516.8001	0.90
MET_MATCH					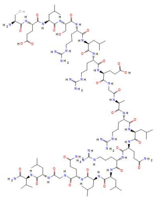	570.0878	570.0961	14.52
MET_MATCH					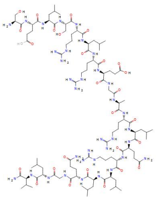	570.0878	570.0961	14.52
MET_MATCH					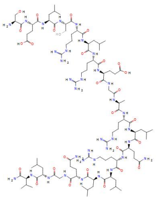	570.0878	570.0961	14.52
MET_MATCH					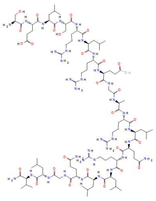	570.0878	570.0961	14.52
MET_MATCH					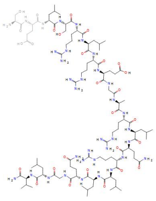	687.4213	687.4236	3.44

Metabolite: M4 -745 RT=2.24

Type	score	sub. m/z observed	sub. m/z calculated	sub ppm		met. m/z observed	met. m/z calculated	met. ppm
MET_MATCH					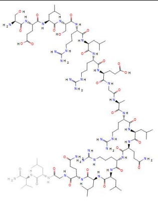	688.7357	688.7310	-6.74
MET_MATCH					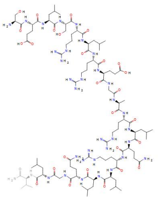	726.4256	726.4257	0.14
MET_MATCH					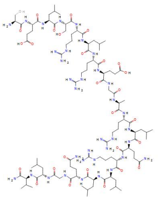	759.1222	759.1205	-2.23
MET_MATCH					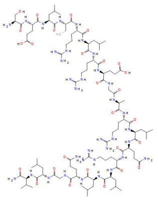	759.1222	759.1205	-2.23
MET_MATCH					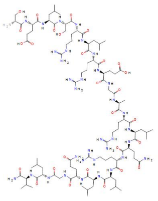	759.4517	759.4485	-4.24
MET_MATCH					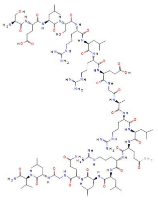	759.4517	759.4485	-4.24
MET_MATCH					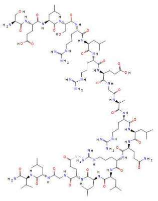	759.4517	759.4485	-4.24
MET_MATCH					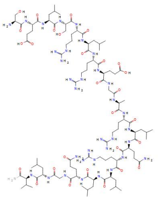	759.4517	759.4485	-4.24

MS (+) FT

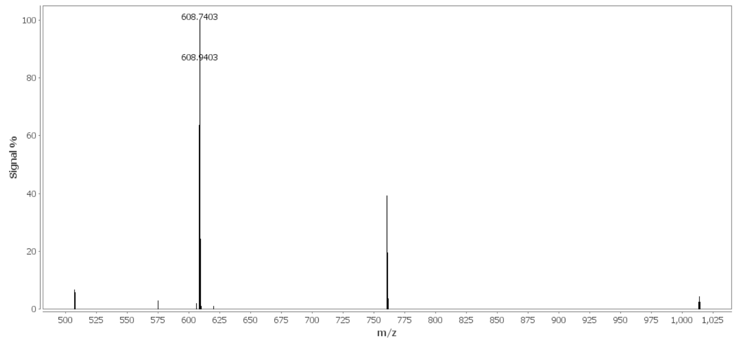

MS (+) FT

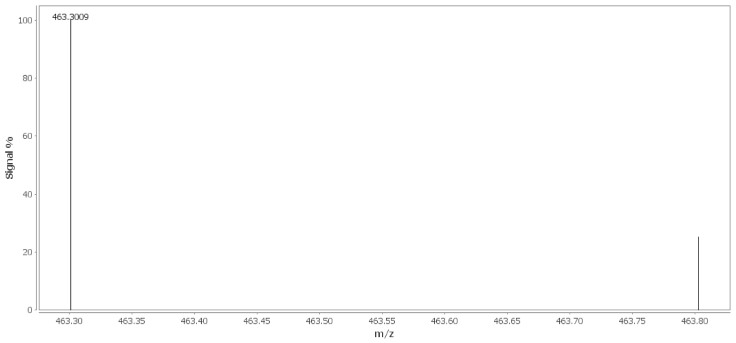

MS2 (+) FT activ = HCD:ce =

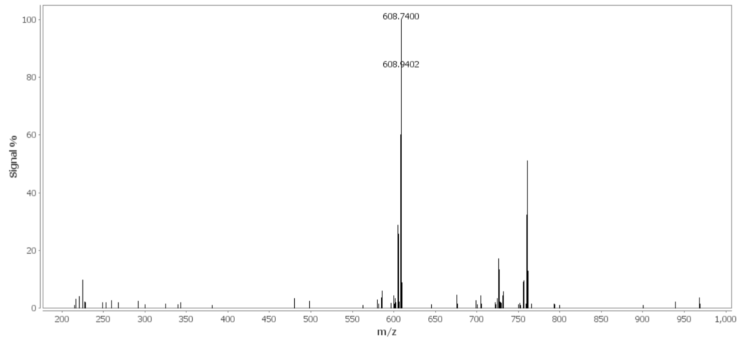

MS2 (+) FT activ = HCD:ce =

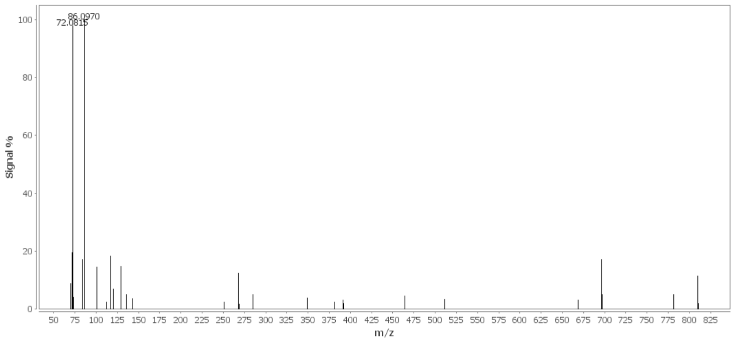

Metabolite: M1 -2113 RT=1.99

Type	score	sub. m/z observed	sub. m/z calculated	sub ppm		met. m/z observed	met. m/z calculated	met. ppm
MATCH	163.0	608.5402	608.5379	-3.65	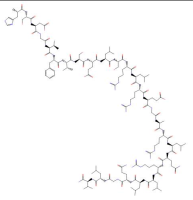 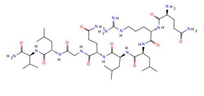	463.3009	463.3007	-0.39
					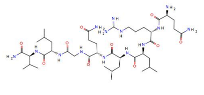	463.3009	463.3007	-0.39
MATCH	122.3	760.4250	760.4206	-5.83	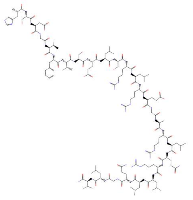 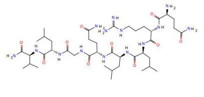	463.3009	463.3007	-0.39
					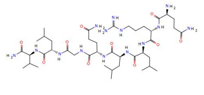	463.3009	463.3007	-0.39
MATCH	14.9	268.1409	268.1404	-1.68	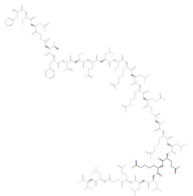 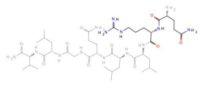	268.1394	268.1404	3.88

Metabolite: M1 -2113 RT=1.99

Type	score	sub. m/z observed	sub. m/z calculated	sub ppm		met. m/z observed	met. m/z calculated	met. ppm
MATCH	3.5	381.2240	381.2245	1.17	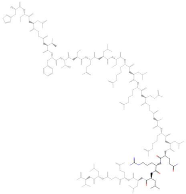 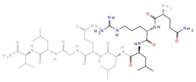	381.2242	381.2245	0.86
MISMATCH	-14.9	268.1409	268.1397	-4.18	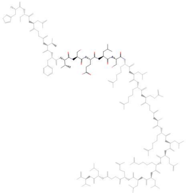	268.1394	268.1394	0.00
MISMATCH	-14.4	585.3190	585.3190	-0.02	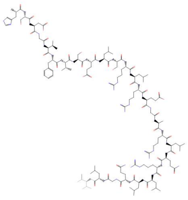	809.4940	809.4940	0.00
MISMATCH	-15.6	731.3960	731.3969	1.17	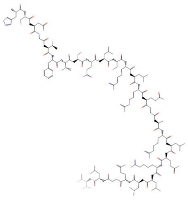	809.4940	809.4940	0.00
MET_MATCH					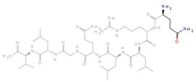	101.0713	101.0709	-3.76
MET_MATCH					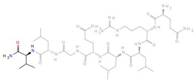	117.1024	117.1022	-1.30
MET_MATCH					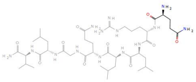	129.0656	129.0659	2.22
MET_MATCH					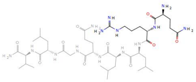	285.1658	285.1670	4.08
MET_MATCH					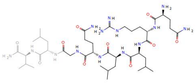	348.7095	348.7112	4.86

Metabolite: M1 -2113 RT=1.99

Type	score	sub. m/z observed	sub. m/z calculated	sub ppm		met. m/z observed	met. m/z calculated	met. ppm
MET_MATCH					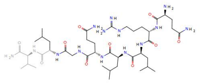	391.2546	391.2558	3.05
MET_MATCH					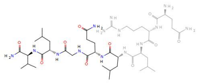	511.3337	511.3239	-19.2
MET_MATCH					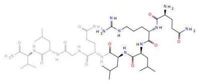	511.3337	511.3351	2.71
MET_MATCH					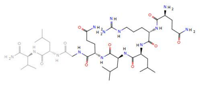	668.4185	668.4202	2.63
MET_MATCH					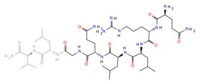	696.4121	696.4151	4.33
MET_MATCH					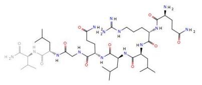	781.4989	781.5043	6.87
MET_MATCH					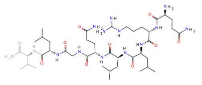	809.4940	809.4992	6.41

MS (+) FT

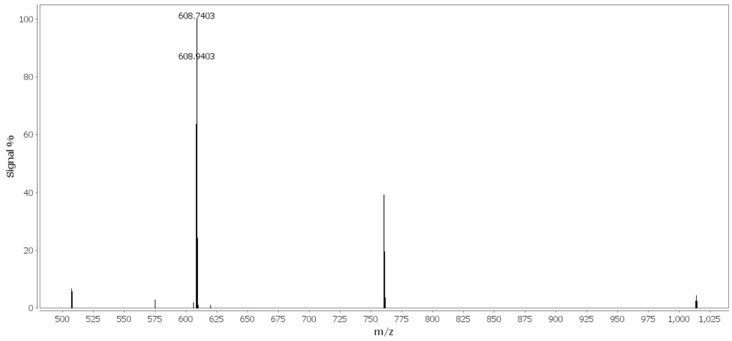

MS (+) FT

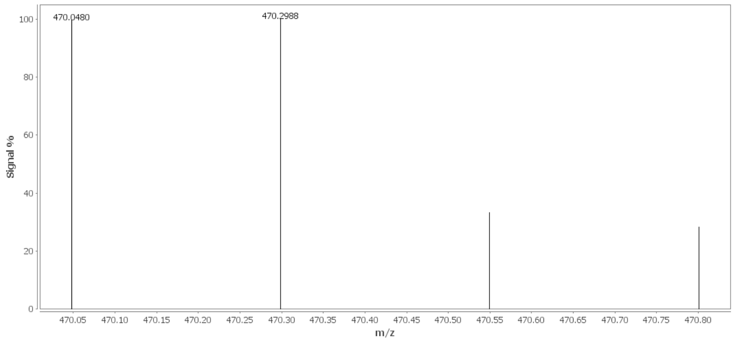

MS2 (+) FT activ = HCD:ce =

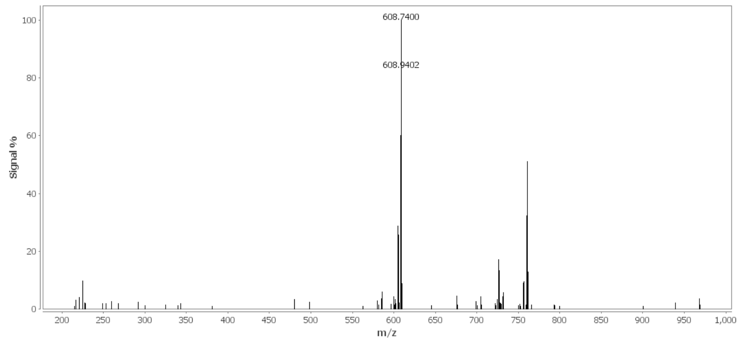

MS2 (+) FT activ = HCD:ce =

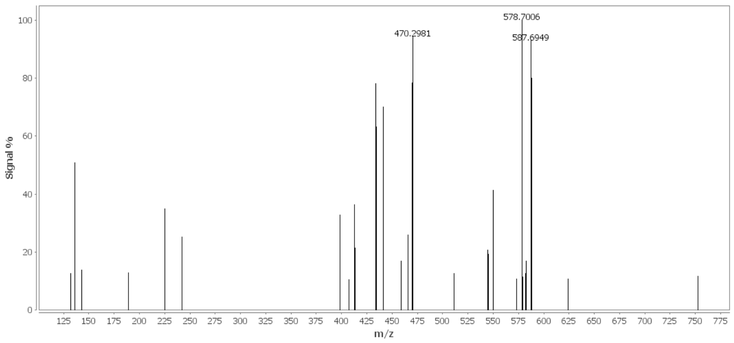

Metabolite: M2 -1162 RT=2.09

Type	score	sub. m/z observed	sub. m/z calculated	sub ppm			met. m/z observed	met. m/z calculated	met. ppm
MATCH	162.6	608.5402	608.5379	-3.65	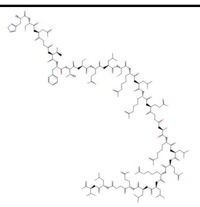	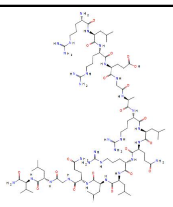	470.0480	470.0472	-1.77
						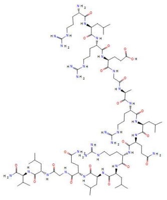	470.0480	470.0472	-1.77
						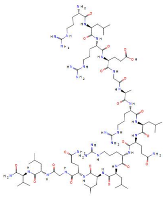	470.0480	470.0472	-1.77
MATCH	122.0	760.4250	760.4206	-5.83	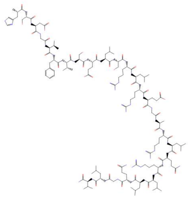	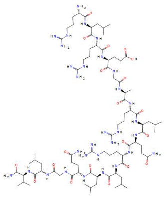	470.0480	470.0472	-1.77
						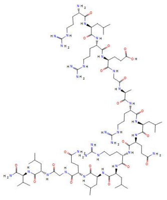	470.0480	470.0472	-1.77
						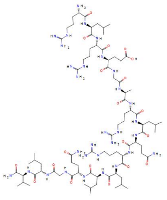	470.0480	470.0472	-1.77
MATCH	101.9	1013.5658	1013.5584	-7.29	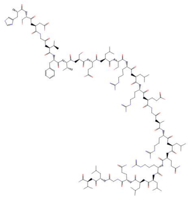	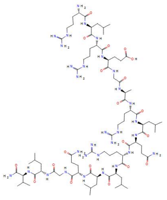	470.0480	470.0472	-1.77

Metabolite: M2 -1162 RT=2.09

Type	score	sub. m/z observed	sub. m/z calculated	sub ppm			met. m/z observed	met. m/z calculated	met. ppm
						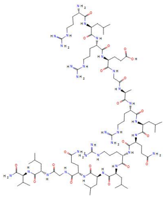	470.0480	470.0472	-1.77
						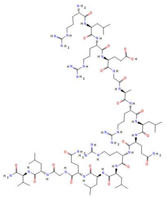	470.0480	470.0472	-1.77
MATCH	37.6	225.1716	225.1710	-2.56	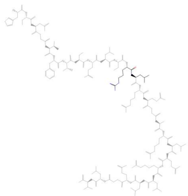	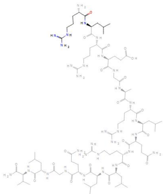	225.1707	225.1710	1.39
MATCH	37.6	225.1716	225.1710	-2.56	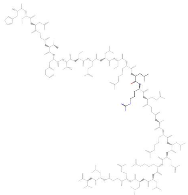	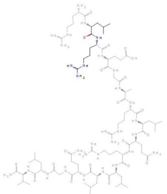	225.1707	225.1710	1.39
MATCH	37.6	225.1716	225.1710	-2.56	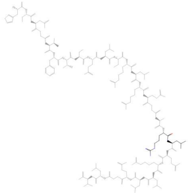	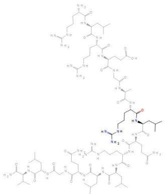	225.1707	225.1710	1.39
MATCH	37.6	225.1716	225.1710	-2.56	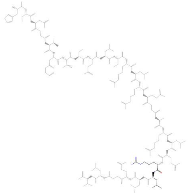	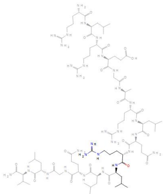	225.1707	225.1710	1.39
MATCH	26.5	242.1495	242.1499	1.53	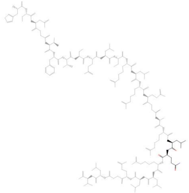	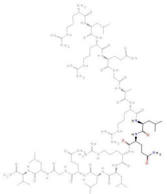	242.1494	242.1499	2.01
MATCH	26.5	242.1495	242.1499	1.53	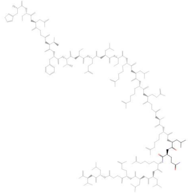	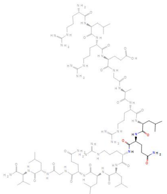	242.1494	242.1499	2.01
MATCH	26.5	242.1495	242.1499	1.53	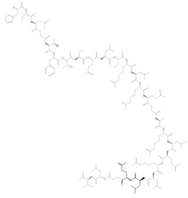	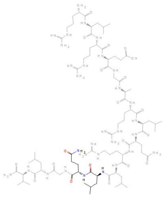	242.1494	242.1499	2.01

Metabolite: M2 -1162 RT=2.09

Type	score	sub. m/z observed	sub. m/z calculated	sub ppm			met. m/z observed	met. m/z calculated	met. ppm
MATCH	26.5	242.1495	242.1499	1.53	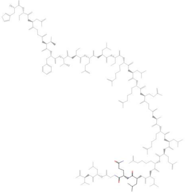	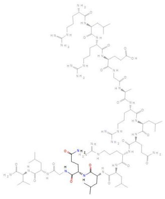	242.1494	242.1499	2.01
MATCH	26.5	242.1495	242.1555	24.73	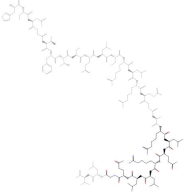	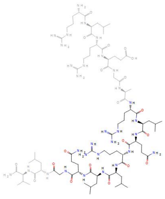	242.1494	242.1555	25.20
MATCH	26.5	242.1495	242.1555	24.73	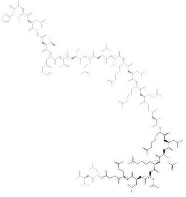	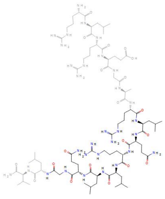	242.1494	242.1555	25.20
MATCH	143.2	608.5397	608.5379	-2.87	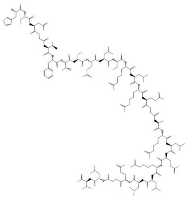	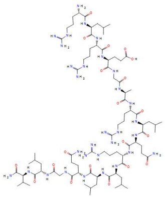	470.0481	470.0472	-1.97
						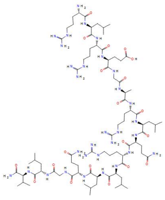	470.0481	470.0472	-1.97
MATCH	79.4	724.4044	724.3981	-8.60	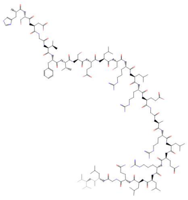	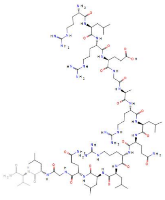	434.0246	434.0247	0.20
MATCH	39.3	731.3960	731.3969	1.17	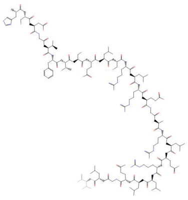	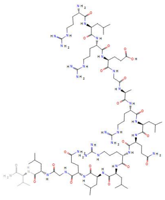	441.0275	441.0234	-9.19
MATCH	36.5	756.1710	756.1640	-9.27	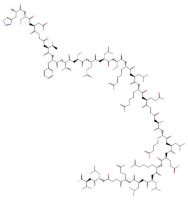	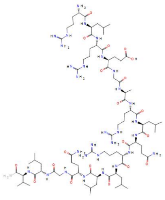	465.7935	465.7905	-6.44
MATCH	36.5	756.1710	756.1640	-9.27	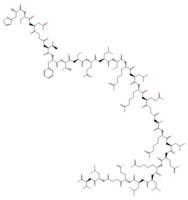	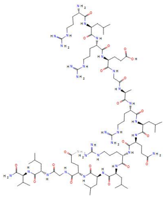	465.7935	465.7905	-6.44

Metabolite: M2 -1162 RT=2.09

Type	score	sub. m/z observed	sub. m/z calculated	sub ppm			met. m/z observed	met. m/z calculated	met. ppm
MATCH	36.5	756.1710	756.1640	-9.27	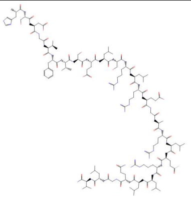	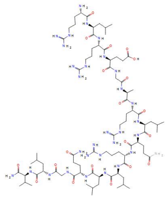	465.7935	465.7905	-6.44
MATCH	112.2	760.4258	760.4206	-6.83	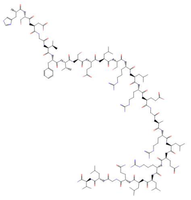	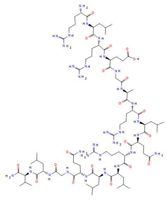	470.0481	470.0472	-1.97
						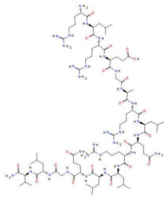	470.0481	470.0472	-1.97
MISMATCH	-75.5	579.7240	579.7200	-6.96	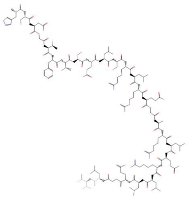		578.3674	578.3674	0.00
MISMATCH	-96.1	585.3190	585.3190	-0.02	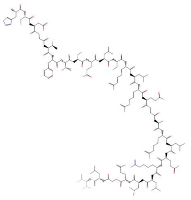		587.6949	587.6949	0.00
MISMATCH	-21.6	698.8711	698.8692	-2.72	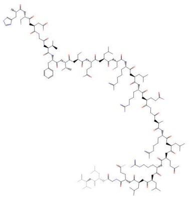		544.3269	544.3269	0.00
MISMATCH	-75.3	724.4044	724.3981	-8.60	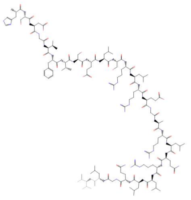		578.3674	578.3674	0.00
MISMATCH	-97.2	731.3960	731.3969	1.17	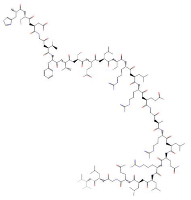		587.6949	587.6949	0.00
MET_MATCH						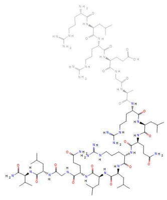	398.2516	398.2594	19.73

Metabolite: M2 -1162 RT=2.09

Type	score	sub. m/z observed	sub. m/z calculated	sub ppm		met. m/z observed	met. m/z calculated	met. ppm
MET_MATCH					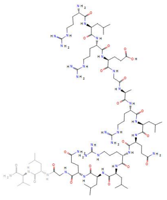	412.7518	412.7524	1.51
MET_MATCH					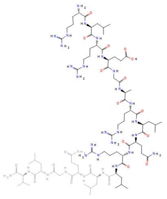	441.2751	441.2811	13.63
MET_MATCH					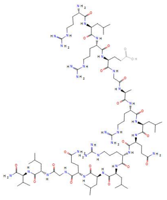	459.0433	459.0497	13.90
MET_MATCH					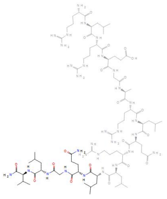	511.3330	511.3239	-17.8
MET_MATCH					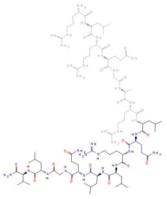	511.3330	511.3295	-6.91
MET_MATCH					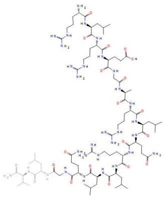	549.9994	550.0008	2.51
MET_MATCH					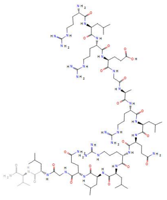	578.3674	578.3638	-6.14
MET_MATCH					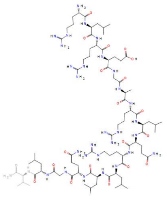	587.6949	587.6955	0.91
MET_MATCH					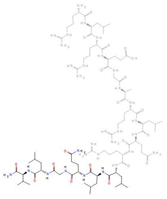	624.4179	624.4079	-15.9
