# Supplementary material for: Software-aided approach to investigate peptide structure and metabolic susceptibility of amide bonds in peptide drugs based on high resolution mass spectrometry
Source: PLoS One. 2017 Nov 1;12(11):e0186461. doi: 10.1371/journal.pone.0186461 (PMC5665424; doi:10.1371/journal.pone.0186461)
Supplement: S1 File — (ZIP) [file pone.0186461.s007.zip › SFiles/S32_File.pdf]

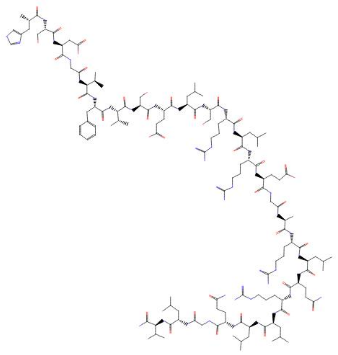

Secretin

Property name	Property value
Time	0min, 5min, 15min, 45min, 120min
Instrument	ThermoQAPlus
Acquisition Mode	ddMS2
Matrix	pepsin

Chromatograms

Time=0min

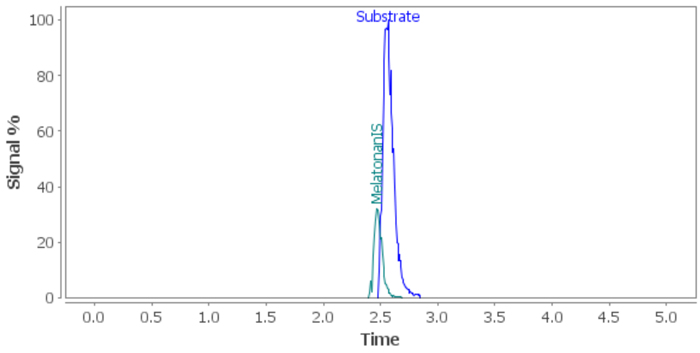

Time=5min

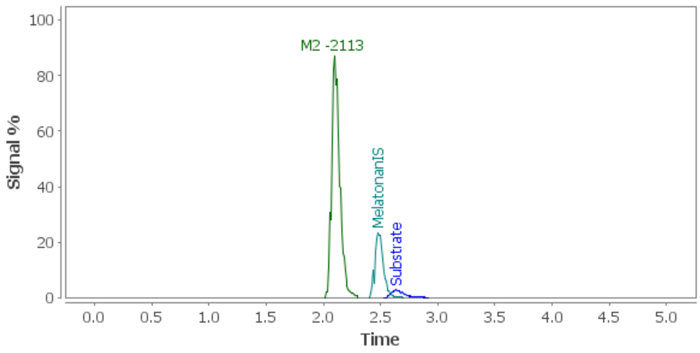

Time=15min

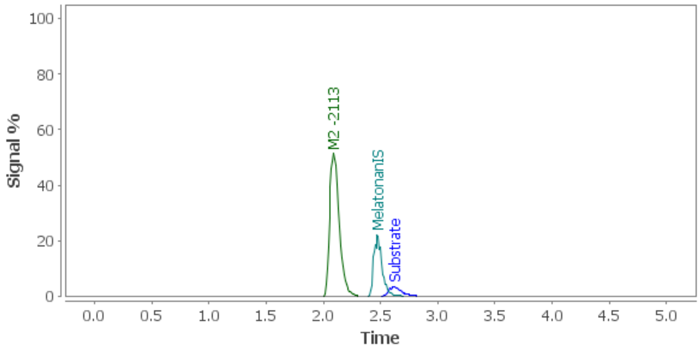

Time=45min

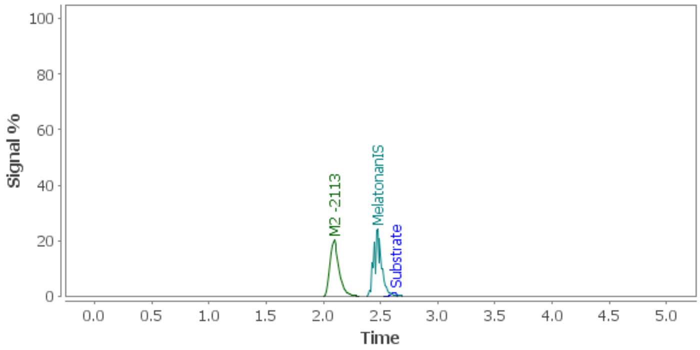

Time=120min

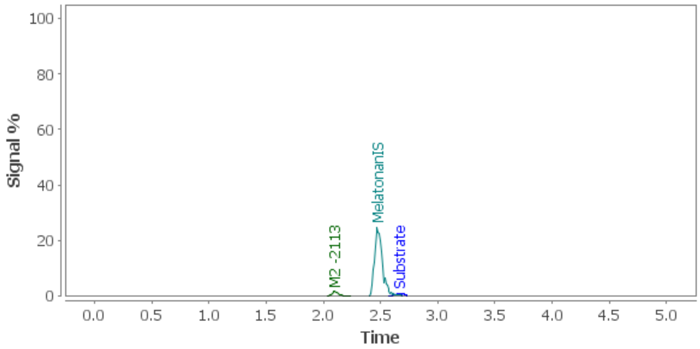

Custom Charts

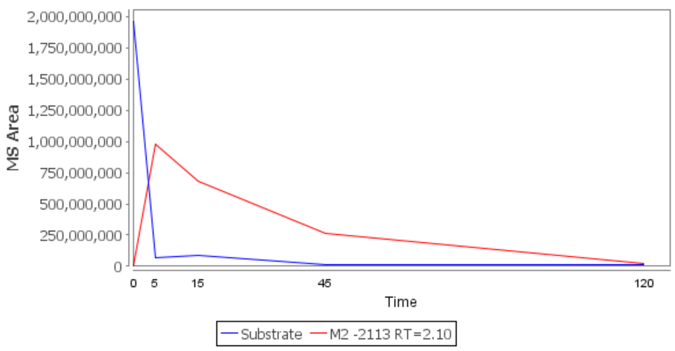

Fragmentation

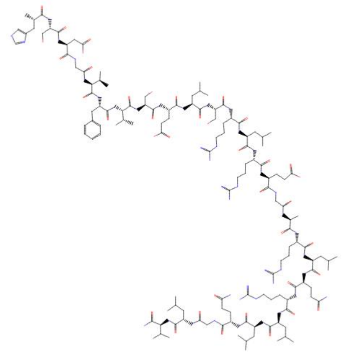

Secretin

MS (+) FT

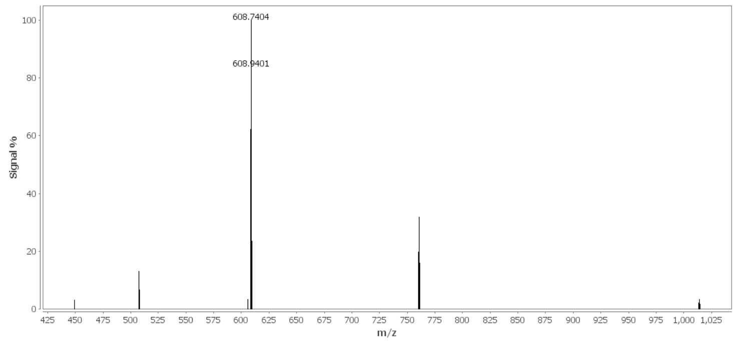

MS (+) FT

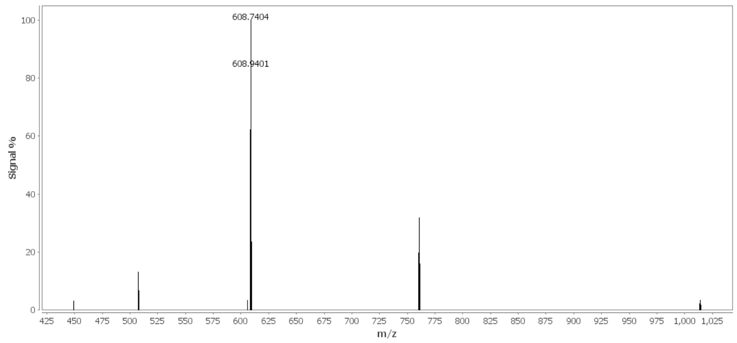

MS2 (+) FT activ = HCD:ce =

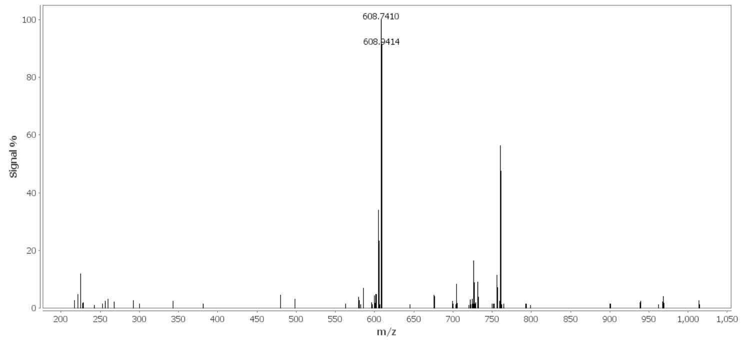

MS2 (+) FT activ = HCD:ce =

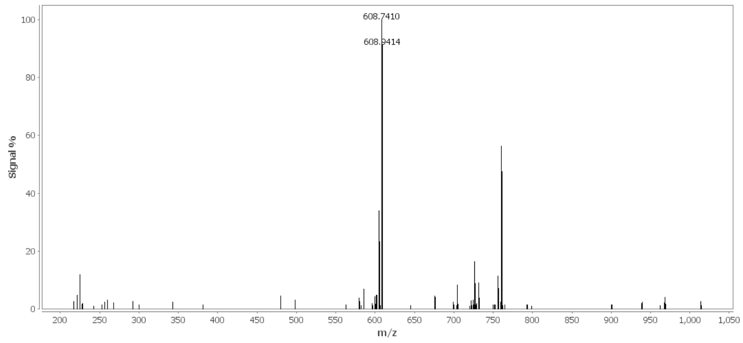

Metabolite: Substrate

Type	score	sub. m/z observed	sub. m/z calculated	sub ppm			met. m/z observed	met. m/z calculated	met. ppm
MATCH	102.1	1013.5651	1013.5584	-6.58	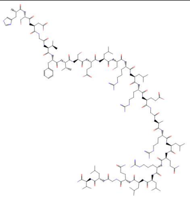	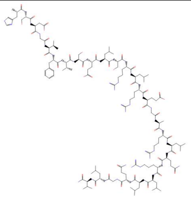	1013.5651	1013.5584	-6.58
MATCH	119.8	760.4253	760.4206	-6.11	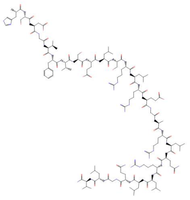	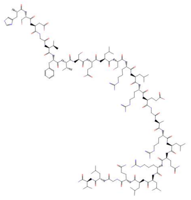	760.4253	760.4206	-6.11
MISMATCH	-7.1	731.3995	731.3969	-3.57	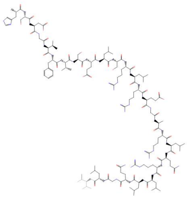	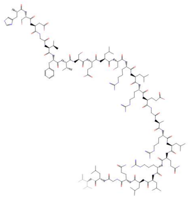	731.3995	731.3969	-3.57

Metabolite: Substrate

Type	score	sub. m/z observed	sub. m/z calculated	sub ppm			met. m/z observed	met. m/z calculated	met. ppm
MATCH	162.2	608.5406	608.5379	-4.33	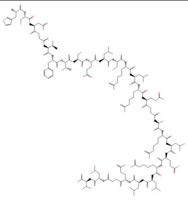	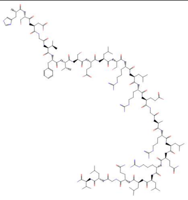	608.5406	608.5379	-4.33
MISMATCH	-3.0	597.3370	597.3467	16.34	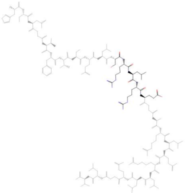	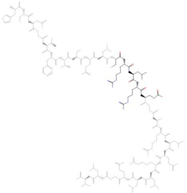	597.3370	597.3467	16.34
MISMATCH	-6.5	585.3220	585.3190	-5.16	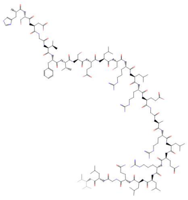	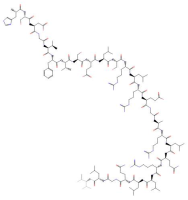	585.3220	585.3190	-5.16
MISMATCH	-7.5	562.7056	562.7021	-6.20	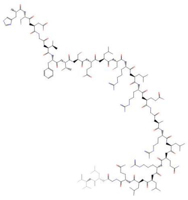	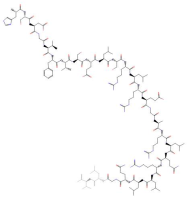	562.7056	562.7021	-6.20
MATCH	5.9	381.2251	381.2245	-1.50	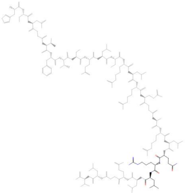	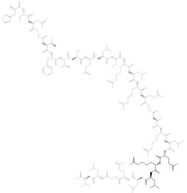	381.2251	381.2245	-1.50
MATCH	20.2	268.1406	268.1404	-0.80	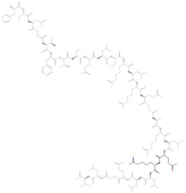	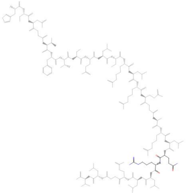	268.1406	268.1404	-0.80
MISMATCH	-20.2	268.1406	268.1397	-3.29	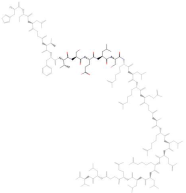	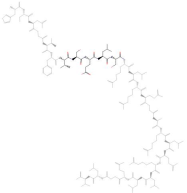	268.1406	268.1397	-3.29
MATCH	2.1	242.1506	242.1499	-2.94	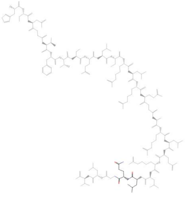	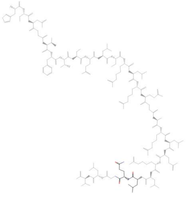	242.1506	242.1499	-2.94
MATCH	2.1	242.1506	242.1499	-2.94	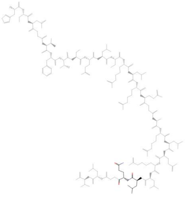	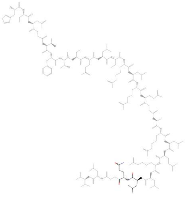	242.1506	242.1499	-2.94

Metabolite: Substrate

Type	score	sub. m/z observed	sub. m/z calculated	sub ppm			met. m/z observed	met. m/z calculated	met. ppm
MATCH	4.3	225.1714	225.1710	-1.68	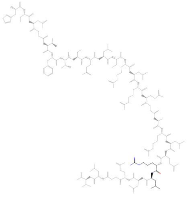	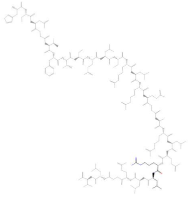	225.1714	225.1710	-1.68

MS (+) FT

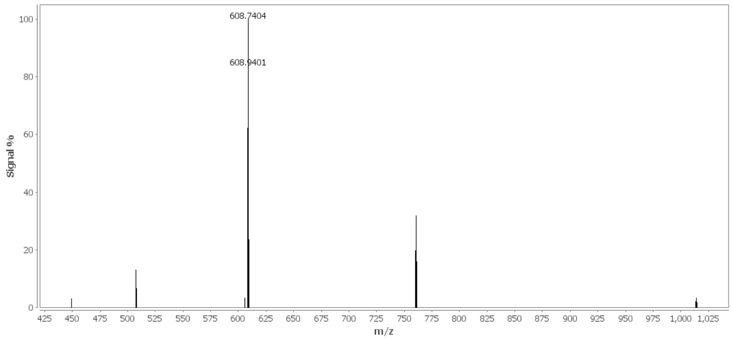

MS (+) FT

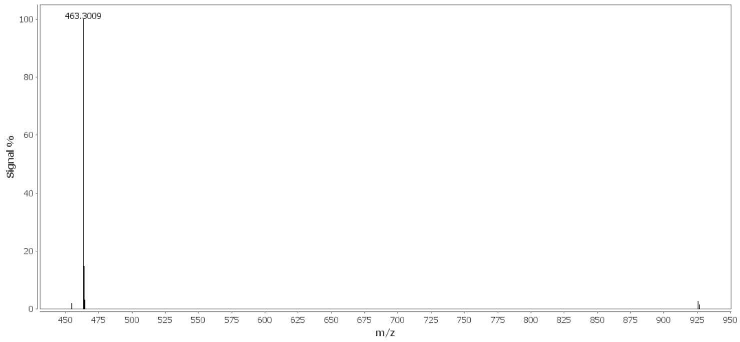

MS2 (+) FT activ = HCD:ce =

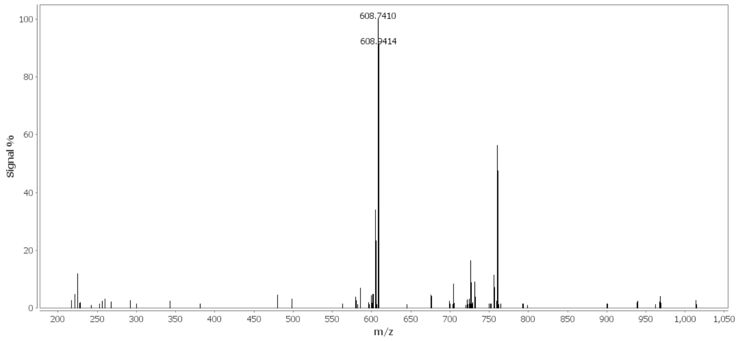

MS2 (+) FT activ = HCD:ce =

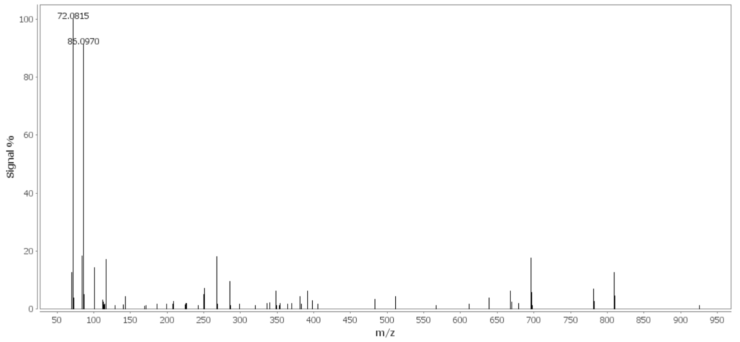

Metabolite: M2 -2113 RT=2.10

Type	score	sub. m/z observed	sub. m/z calculated	sub ppm			met. m/z observed	met. m/z calculated	met. ppm
MATCH	162.2	608.5406	608.5379	-4.33	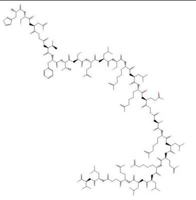	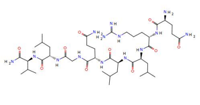	463.3009	463.3007	-0.43
						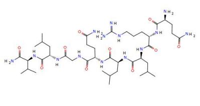	463.3009	463.3007	-0.43
						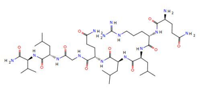	463.3009	463.3007	-0.43

Metabolite: M2 -2113 RT=2.10

Type	score	sub. m/z observed	sub. m/z calculated	sub ppm			met. m/z observed	met. m/z calculated	met. ppm
MATCH	119.8	760.4253	760.4206	-6.11	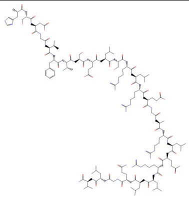	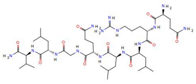	463.3009	463.3007	-0.43
						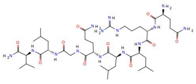	463.3009	463.3007	-0.43
						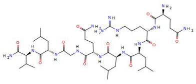	463.3009	463.3007	-0.43
MATCH	102.1	1013.5651	1013.5584	-6.58	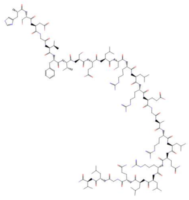	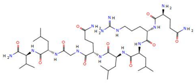	463.3009	463.3007	-0.43
						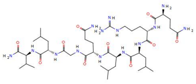	463.3009	463.3007	-0.43
						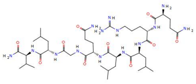	463.3009	463.3007	-0.43
MATCH	4.3	225.1714	225.1710	-1.68	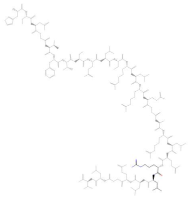	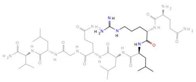	225.1695	225.1710	6.76
MATCH	2.1	242.1506	242.1499	-2.94	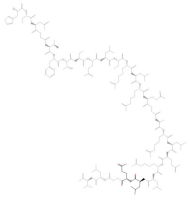	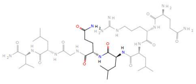	242.1493	242.1499	2.51
MATCH	2.1	242.1506	242.1499	-2.94	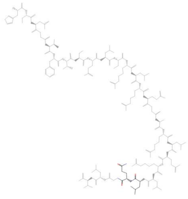	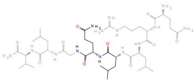	242.1493	242.1499	2.51

Metabolite: M2 -2113 RT=2.10

Type	score	sub. m/z observed	sub. m/z calculated	sub ppm		met. m/z observed	met. m/z calculated	met. ppm
MATCH	20.2	268.1406	268.1404	-0.80	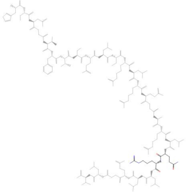 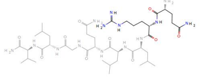	268.1398	268.1404	2.48
MATCH	5.9	381.2251	381.2245	-1.50	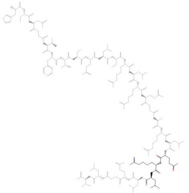 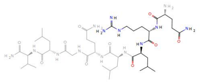	381.2231	381.2245	3.64
MISMATCH	-20.2	268.1406	268.1397	-3.29	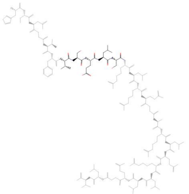	268.1398	268.1398	0.00
MISMATCH	-7.5	562.7056	562.7021	-6.20	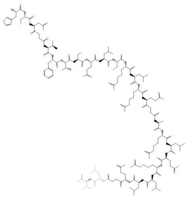	348.7101	348.7101	0.00
MISMATCH	-6.5	585.3220	585.3190	-5.16	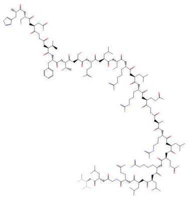	405.2512	405.2512	0.00
MISMATCH	-3.0	597.3370	597.3467	16.34	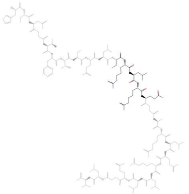	299.1706	299.1706	0.00
MISMATCH	-7.1	731.3995	731.3969	-3.57	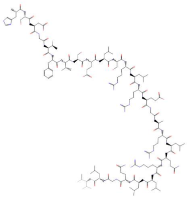	405.2512	405.2512	0.00
MET_MATCH					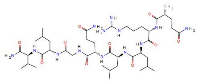	454.7884	454.7874	-2.15
MET_MATCH					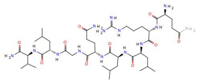	454.7884	454.7874	-2.15

Metabolite: M2 -2113 RT=2.10

Type	score	sub. m/z observed	sub. m/z calculated	sub ppm		met. m/z observed	met. m/z calculated	met. ppm
MET_MATCH					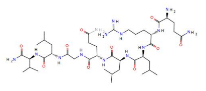	454.7884	454.7874	-2.15
MET_MATCH					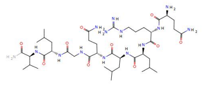	454.7884	454.7874	-2.15
MET_MATCH					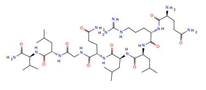	925.5935	925.5942	0.72
MET_MATCH					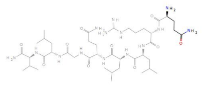	101.0713	101.0709	-3.20
MET_MATCH					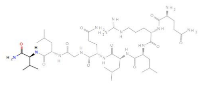	115.0868	115.0866	-1.53
MET_MATCH					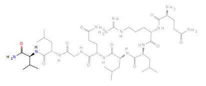	117.1023	117.1022	-0.75
MET_MATCH					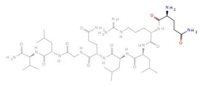	129.0657	129.0659	1.05
MET_MATCH					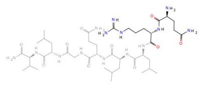	285.1662	285.1670	2.82
MET_MATCH					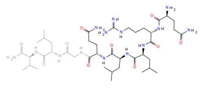	320.2010	320.2005	-1.80

Metabolite: M2 -2113 RT=2.10

Type	score	sub. m/z observed	sub. m/z calculated	sub ppm		met. m/z observed	met. m/z calculated	met. ppm
MET_MATCH					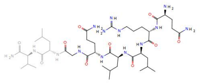	348.7101	348.7112	3.17
MET_MATCH					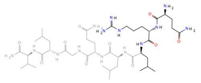	370.2545	370.2561	4.43
MET_MATCH					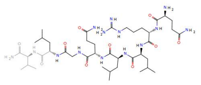	391.2548	391.2558	2.63
MET_MATCH					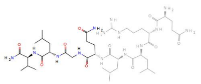	398.2491	398.2398	-23.4
MET_MATCH					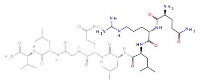	398.2491	398.2510	4.75
MET_MATCH					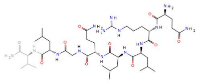	405.2512	405.2532	5.15
MET_MATCH					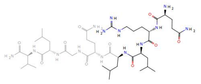	483.3384	483.3402	3.76
MET_MATCH					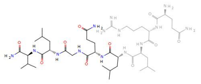	511.3319	511.3239	-15.7
MET_MATCH					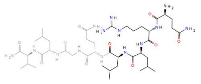	511.3319	511.3351	6.26

Metabolite: M2 -2113 RT=2.10

Type	score	sub. m/z observed	sub. m/z calculated	sub ppm		met. m/z observed	met. m/z calculated	met. ppm
MET_MATCH					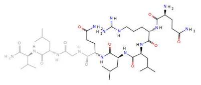	611.3926	611.3988	10.04
MET_MATCH					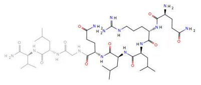	639.3867	639.3937	10.96
MET_MATCH					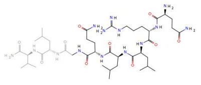	668.4194	668.4202	1.17
MET_MATCH					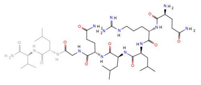	696.4135	696.4151	2.33
MET_MATCH					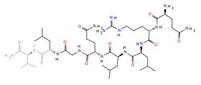	781.5004	781.5043	4.97
MET_MATCH					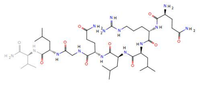	809.4970	809.4992	2.74
MET_MATCH					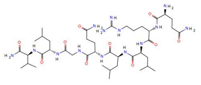	925.5940	925.5942	0.17
