# Supplementary material for: Software-aided approach to investigate peptide structure and metabolic susceptibility of amide bonds in peptide drugs based on high resolution mass spectrometry
Source: PLoS One. 2017 Nov 1;12(11):e0186461. doi: 10.1371/journal.pone.0186461 (PMC5665424; doi:10.1371/journal.pone.0186461)
Supplement: S1 File — (ZIP) [file pone.0186461.s007.zip › SFiles/S33_File.pdf]

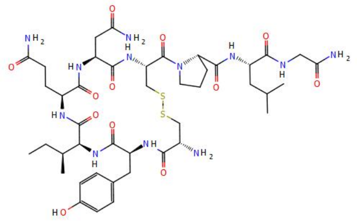

Oxytocin

Property name	Property value
Time	0min, 5min, 15min, 45min, 120min
Instrument	ThermoQAPlus
Matrix	chymotrypsin
Acquisition Mode	ddMS2

Chromatograms

Time=0min

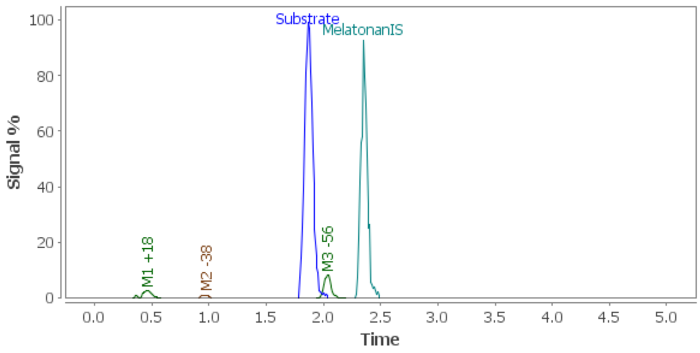

Time=5min

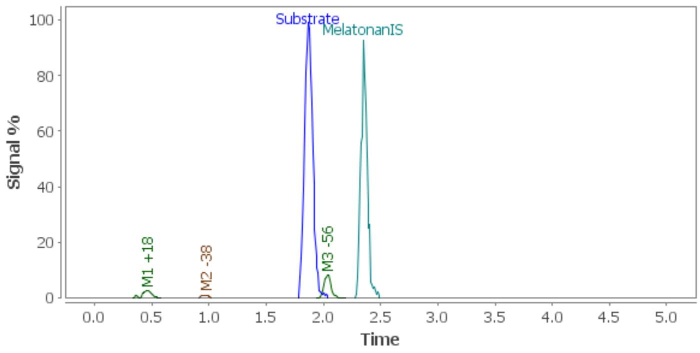

Time=15min

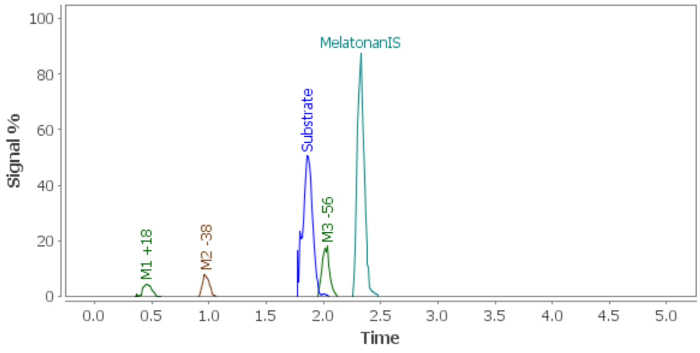

Time=45min

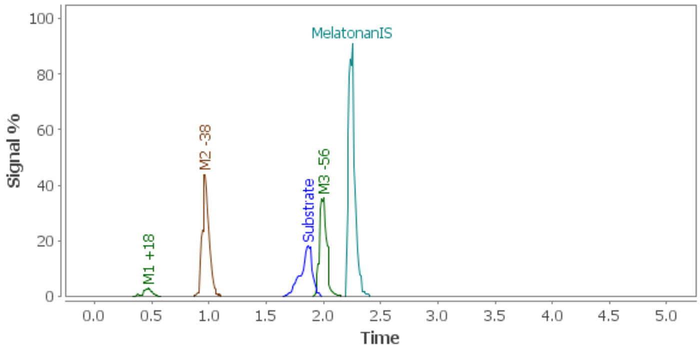

Time=120min

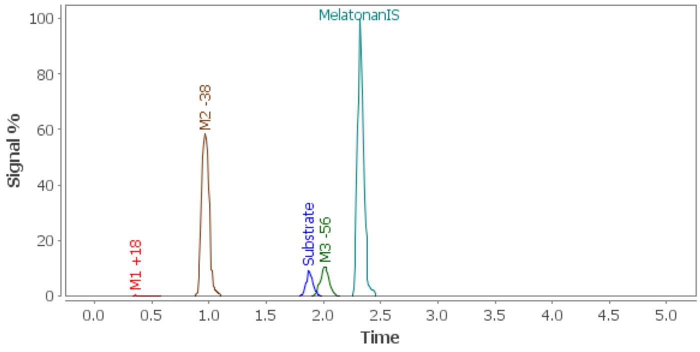

Custom Charts

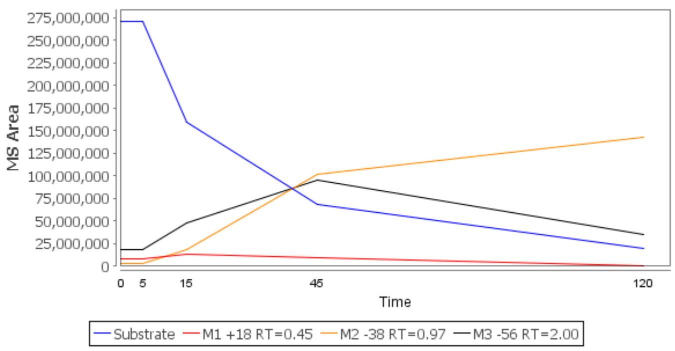

Fragmentation

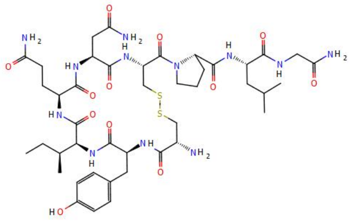

Oxytocin

MS (+) FT

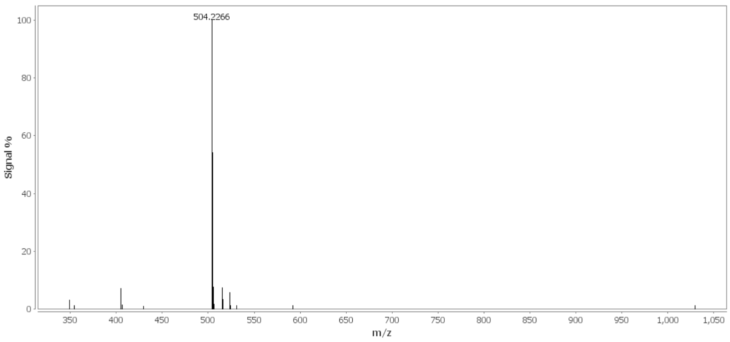

MS (+) FT

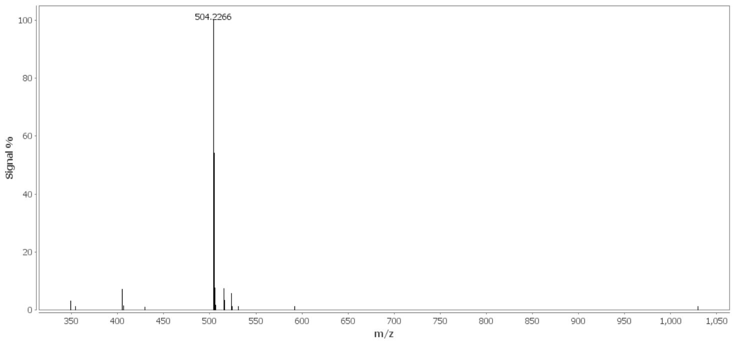

MS2 (+) FT activ = HCD:ce =

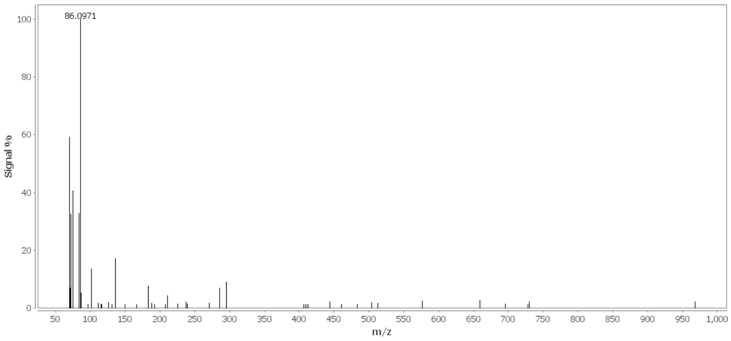

MS2 (+) FT activ = HCD:ce =

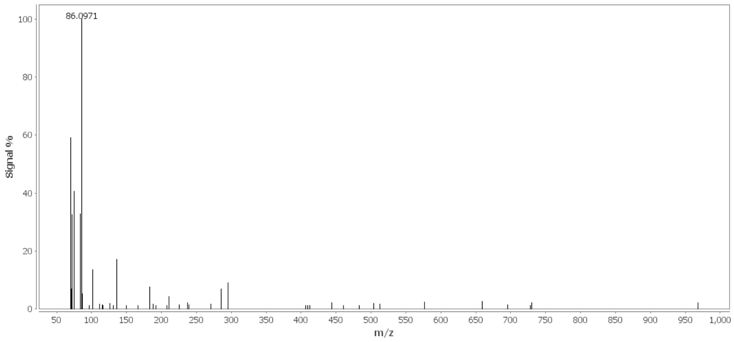

Metabolite: Substrate

Type	score	sub. m/z observed	sub. m/z calculated	sub ppm			met. m/z observed	met. m/z calculated	met. ppm
MATCH	200.0	504.2266	504.2255	-2.09	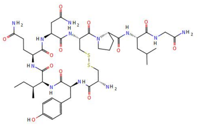	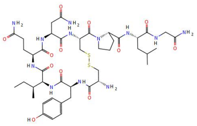	504.2266	504.2255	-2.09
MATCH	37.2	285.1926	285.1921	-1.79	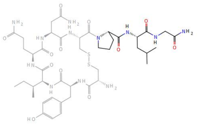	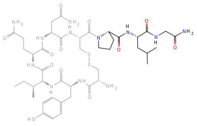	285.1926	285.1921	-1.79
MATCH	3.2	271.0577	271.0569	-2.70	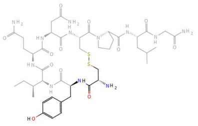	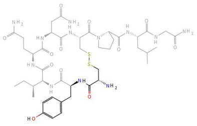	271.0577	271.0569	-2.70

Metabolite: Substrate

Type	score	sub. m/z observed	sub. m/z calculated	sub ppm		met. m/z observed	met. m/z calculated	met. ppm
MATCH	14.0	183.1498	183.1492	-3.22	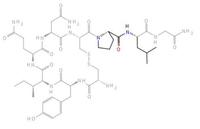 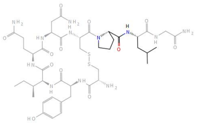	183.1498	183.1492	-3.22
MATCH	49.3	136.0766	136.0757	-6.47	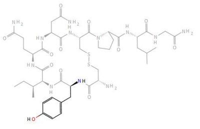 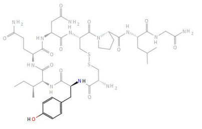	136.0766	136.0757	-6.47
MATCH	35.4	101.0717	101.0709	-7.26	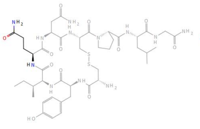 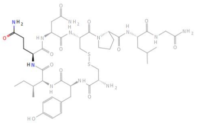	101.0717	101.0709	-7.26
MATCH	200.0	86.0973	86.0964	-9.70	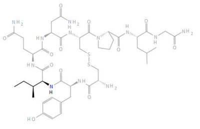 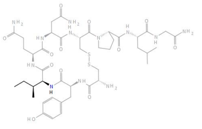	86.0973	86.0964	-9.70
MATCH	200.0	86.0973	86.0964	-9.70	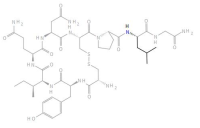 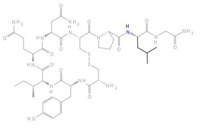	86.0973	86.0964	-9.70
MATCH	69.8	84.0454	84.0444	-11.7	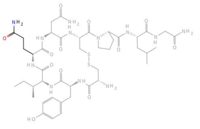 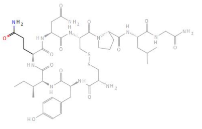	84.0454	84.0444	-11.7
MATCH	144.4	70.0660	70.0651	-12.5	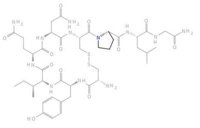 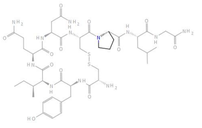	70.0660	70.0651	-12.5

MS (+) FT

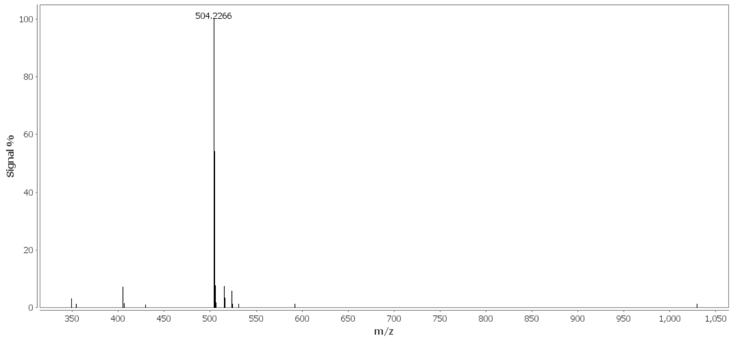

MS (+) FT

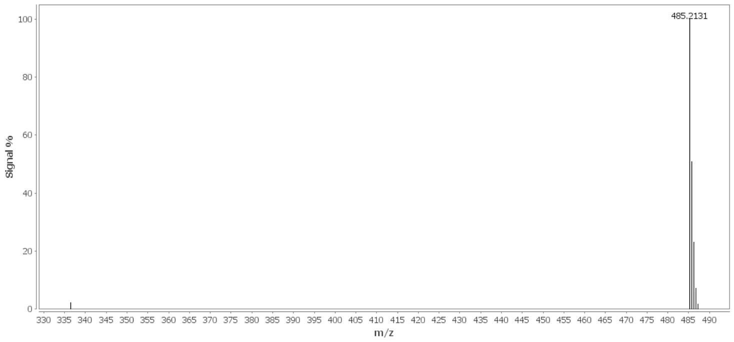

MS2 (+) FT activ = HCD:ce =

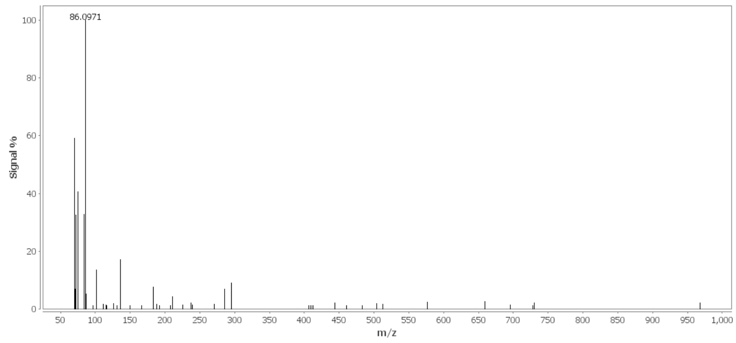

MS2 (+) FT activ = HCD:ce =

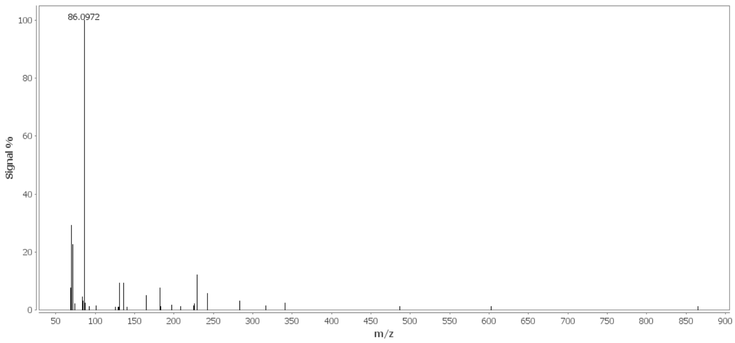

Metabolite: M2 -38 RT=0.97

Type	score	sub. m/z observed	sub. m/z calculated	sub ppm		met. m/z observed	met. m/z calculated	met. ppm
MATCH	200.0	504.2266	504.2255	-2.09	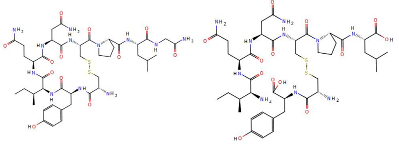	485.2131	485.2121	-2.08
MATCH	97.5	70.0660	70.0651	-12.5	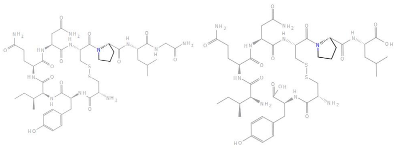	70.0660	70.0651	-12.1
MATCH	37.8	84.0454	84.0444	-11.7	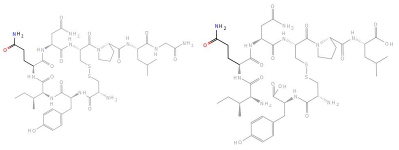	84.0451	84.0444	-8.31
MATCH	200.0	86.0973	86.0964	-9.70	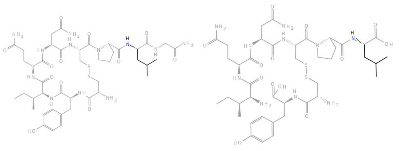	86.0972	86.0964	-8.41
MATCH	19.3	101.0717	101.0709	-7.26	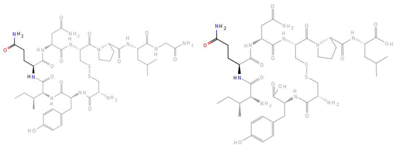	101.0716	101.0709	-6.71
MATCH	27.4	136.0766	136.0757	-6.47	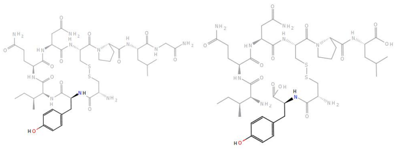	136.0761	136.0757	-2.90
MATCH	8.0	183.1498	183.1492	-3.22	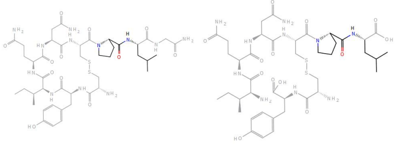	183.1499	183.1492	-4.14

Metabolite: M2 -38 RT=0.97

Type	score	sub. m/z observed	sub. m/z calculated	sub ppm			met. m/z observed	met. m/z calculated	met. ppm
MATCH	15.1	285.1926	285.1921	-1.79	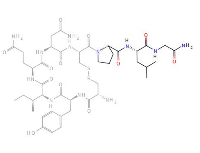	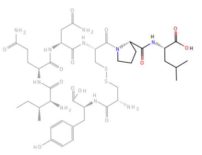	229.1544	229.1547	1.30
MET_MATCH						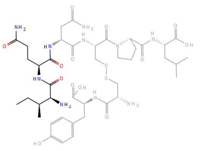	130.0842	130.0919	59.23
MET_MATCH						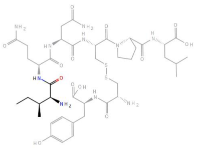	131.1182	131.1179	-2.30
MET_MATCH						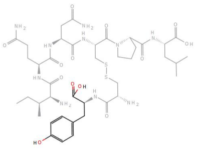	165.0550	165.0546	-2.05
MET_MATCH						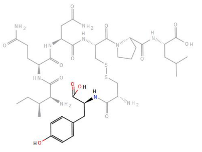	182.0816	182.0812	-2.55
MET_MATCH						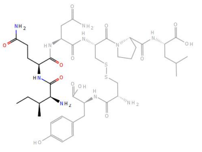	242.1500	242.1499	-0.31
MET_MATCH						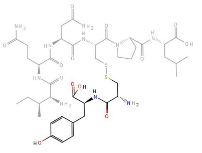	283.0751	283.0747	-1.33
MET_MATCH						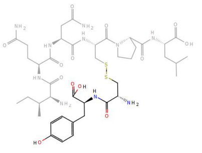	317.0624	317.0624	0.05

MS (+) FT

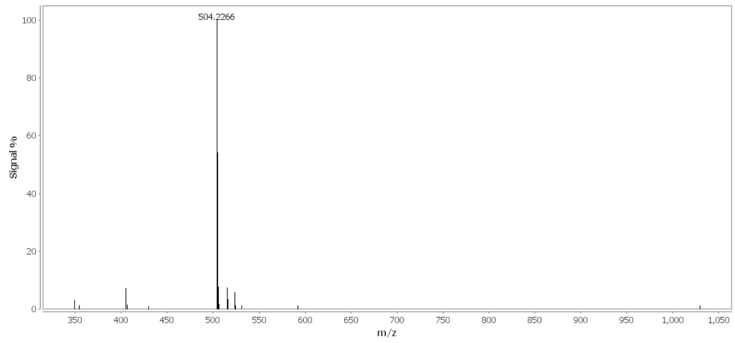

MS (+) FT

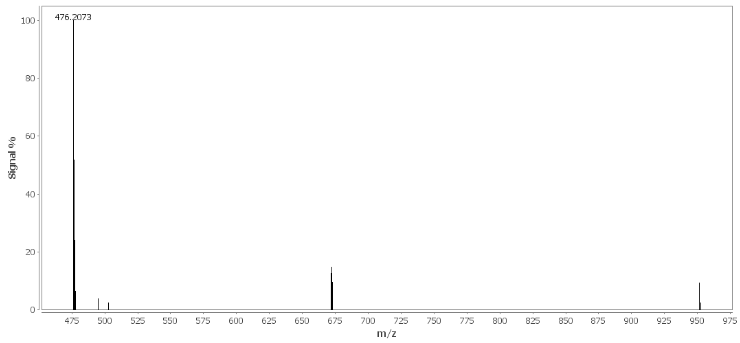

MS2 (+) FT activ = HCD:ce =

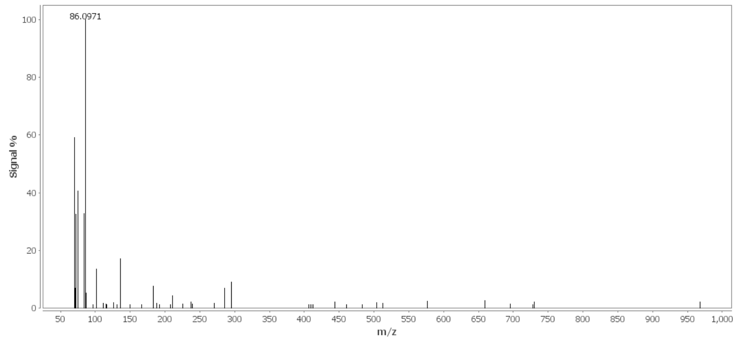

MS2 (+) FT activ = HCD:ce =

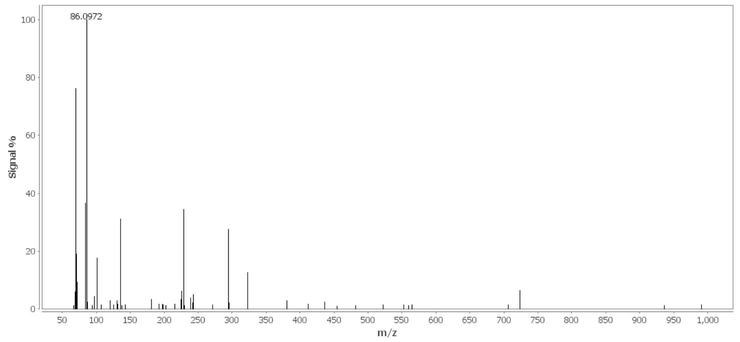

Metabolite: M3 -56 RT=2.00

Type	score	sub. m/z observed	sub. m/z calculated	sub ppm		met. m/z observed	met. m/z calculated	met. ppm
MATCH	200.0	504.2266	504.2255	-2.09	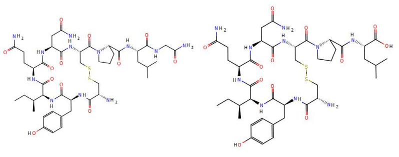	476.2073	476.2068	-1.17
MATCH	109.3	504.2266	504.2255	-2.09	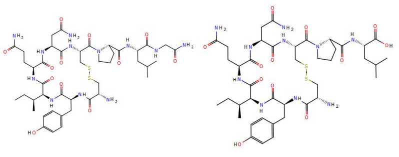	951.4080	951.4063	-1.84
MATCH	144.4	70.0660	70.0651	-12.5	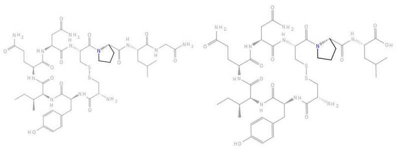	70.0661	70.0651	-13.2
MATCH	69.8	84.0454	84.0444	-11.7	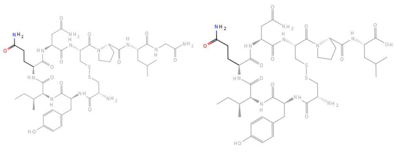	84.0452	84.0444	-9.22
MATCH	200.0	86.0973	86.0964	-9.70	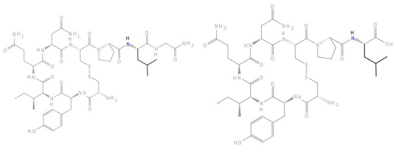	86.0972	86.0964	-8.71

Metabolite: M3 -56 RT=2.00

Type	score	sub. m/z observed	sub. m/z calculated	sub ppm		met. m/z observed	met. m/z calculated	met. ppm
MATCH	200.0	86.0973	86.0964	-9.70	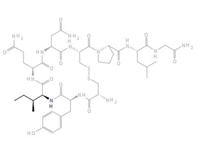 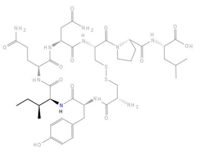	86.0972	86.0964	-8.71
MATCH	35.4	101.0717	101.0709	-7.26	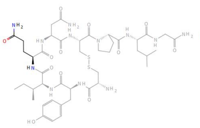 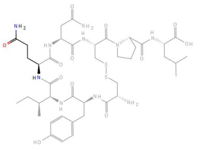	101.0716	101.0709	-6.74
MATCH	49.3	136.0766	136.0757	-6.47	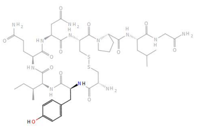 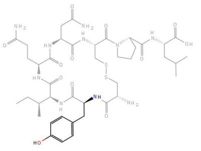	136.0759	136.0757	-1.83
MATCH	3.2	271.0577	271.0569	-2.70	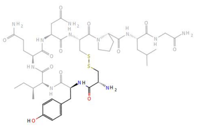 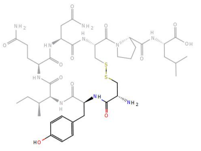	271.0574	271.0569	-1.58
MATCH	37.2	285.1926	285.1921	-1.79	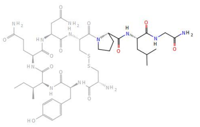 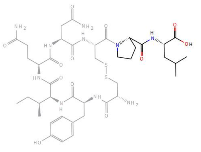	229.1550	229.1547	-1.33
MET_MATCH					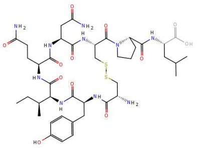	454.2059	454.2119	13.20
MET_MATCH					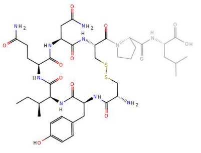	723.2547	723.2589	5.87

MS (+) FT

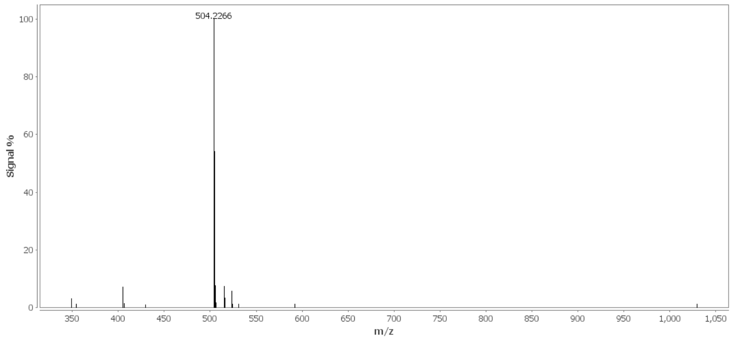

MS (+) FT

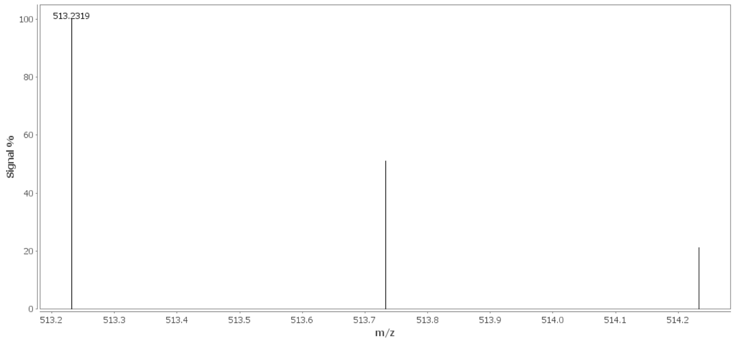

MS2 (+) FT activ = HCD:ce =

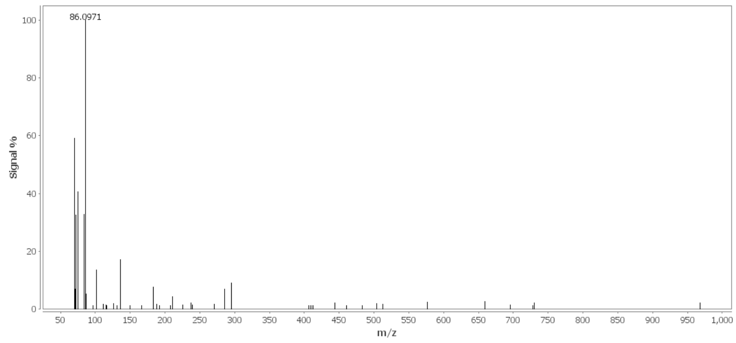

MS2 (+) FT activ = HCD:ce =

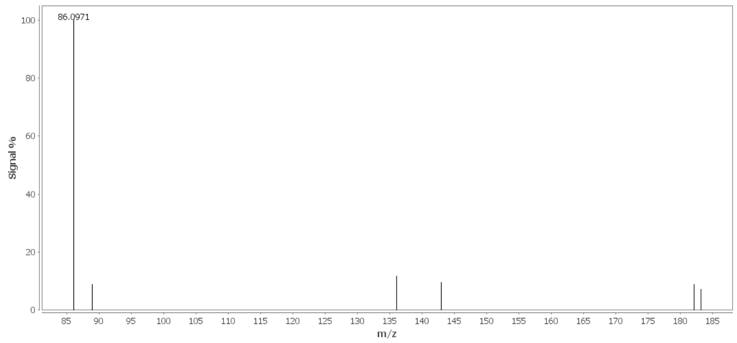

Metabolite: M1 +18 RT=0.45

Type	score	sub. m/z observed	sub. m/z calculated	sub ppm			met. m/z observed	met. m/z calculated	met. ppm
MATCH	200.0	504.2266	504.2255	-2.09	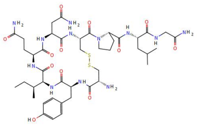	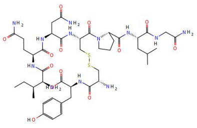	513.2319	513.2308	-2.09
MATCH	200.0	86.0973	86.0964	-9.70	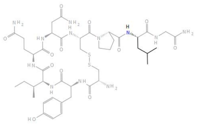	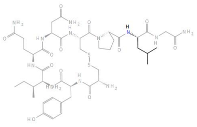	86.0971	86.0964	-8.28
MATCH	29.9	136.0766	136.0757	-6.47	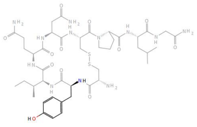	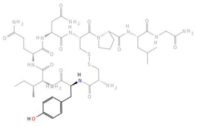	136.0765	136.0757	-5.89
MATCH	14.0	183.1498	183.1492	-3.22	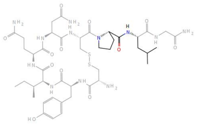	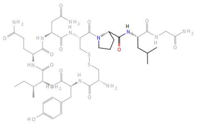	183.1492	183.1492	0.03
MET_MATCH						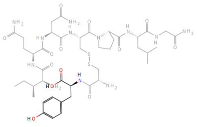	182.0809	182.0812	1.22
