# Supplementary material for: Software-aided approach to investigate peptide structure and metabolic susceptibility of amide bonds in peptide drugs based on high resolution mass spectrometry
Source: PLoS One. 2017 Nov 1;12(11):e0186461. doi: 10.1371/journal.pone.0186461 (PMC5665424; doi:10.1371/journal.pone.0186461)
Supplement: S1 File — (ZIP) [file pone.0186461.s007.zip › SFiles/S35_File.pdf]

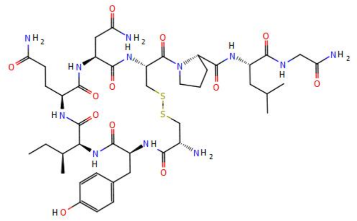

Oxytocin

Property name	Property value
Time	0min, 5min, 15min, 45min, 120min
Instrument	ThermoQAPlus
Matrix	elastase
Acquisition Mode	ddMS2

Chromatograms

Time=0min

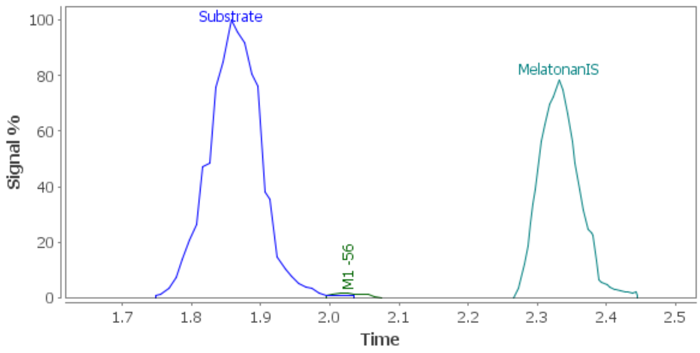

Time=5min

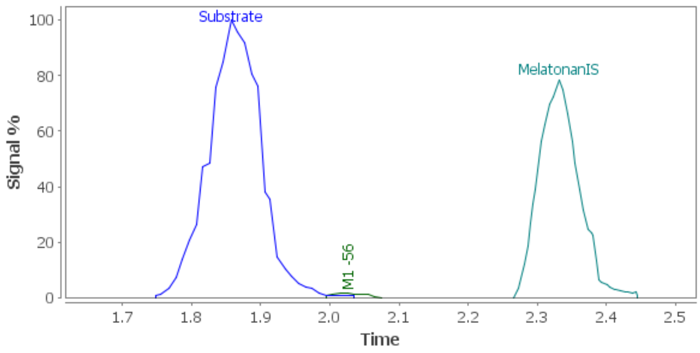

Time=15min

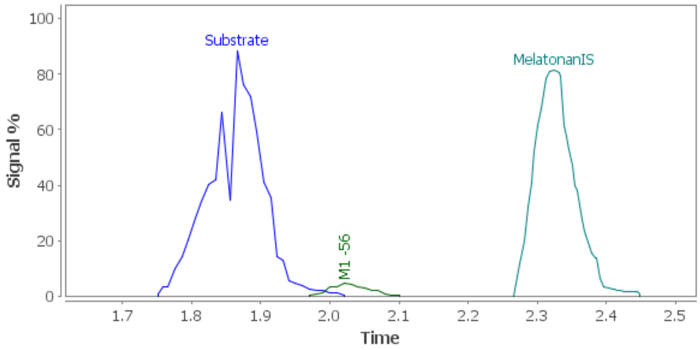

Time=45min

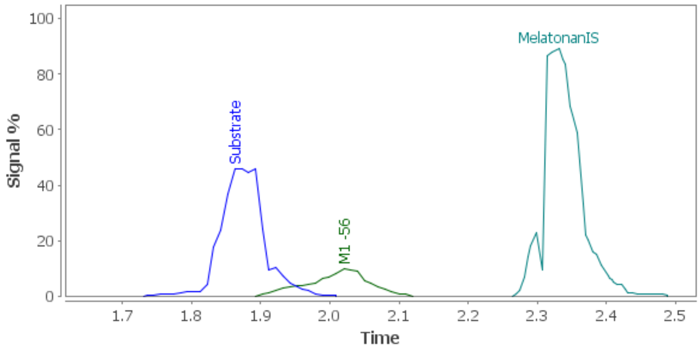

Time=120min

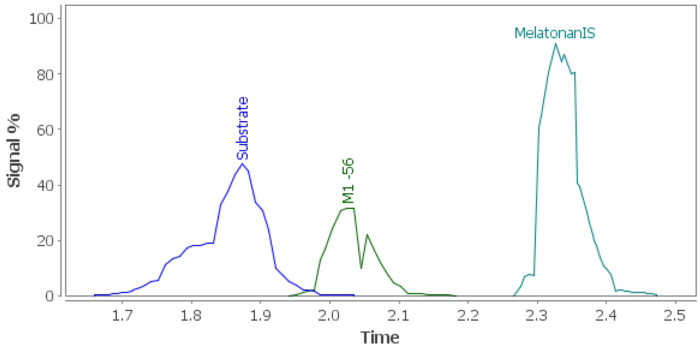

Custom Charts

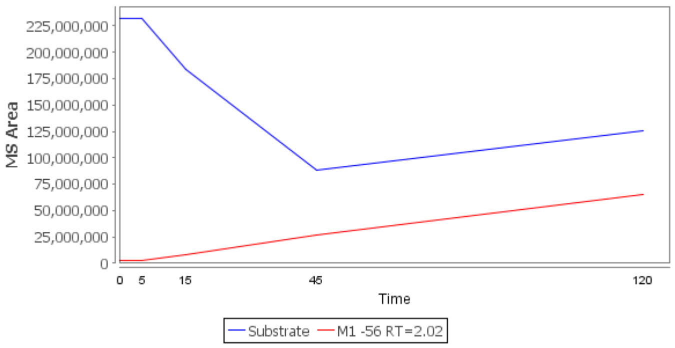

Fragmentation

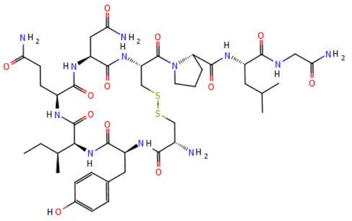

Oxytocin

MS (+) FT

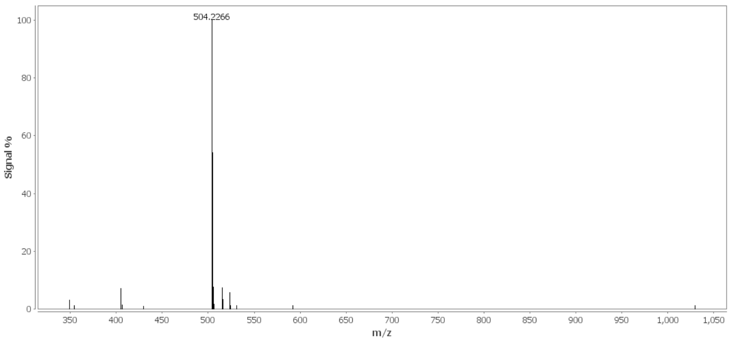

MS (+) FT

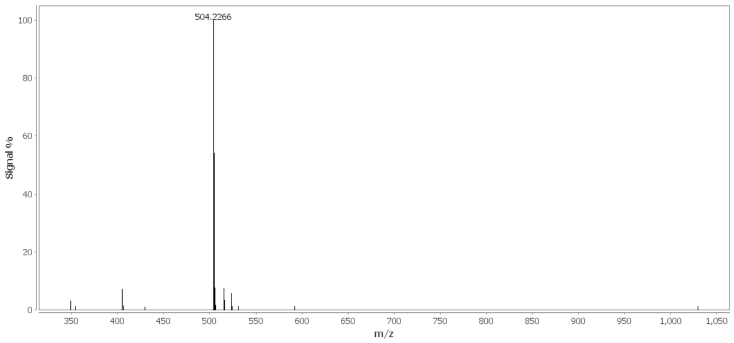

MS2 (+) FT activ = HCD:ce =

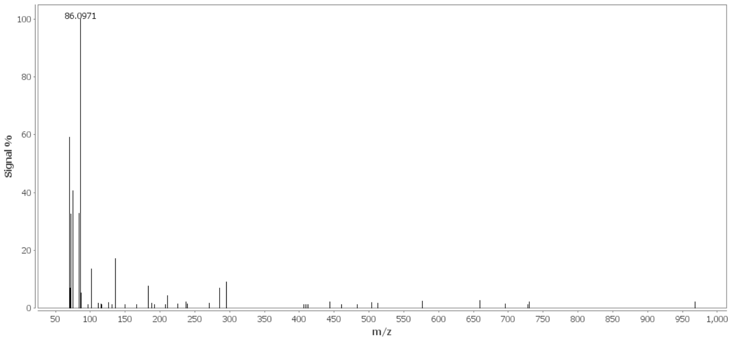

MS2 (+) FT activ = HCD:ce =

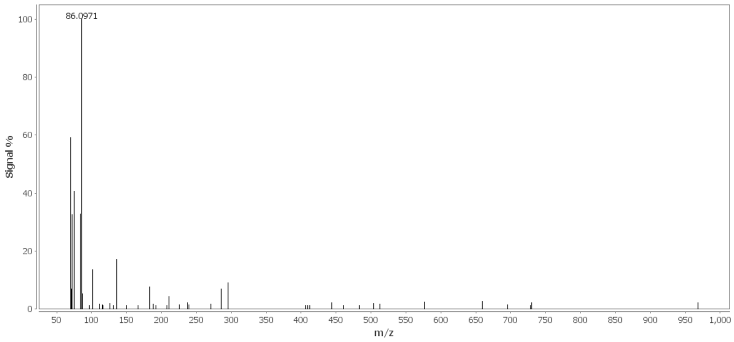

Metabolite: Substrate

Type	score	sub. m/z observed	sub. m/z calculated	sub ppm			met. m/z observed	met. m/z calculated	met. ppm
MATCH	200.0	504.2266	504.2255	-2.12	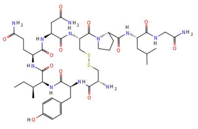	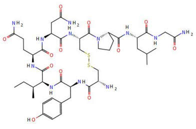	504.2266	504.2255	-2.12
MATCH	36.0	285.1926	285.1921	-1.62	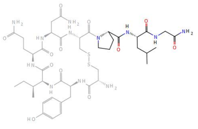	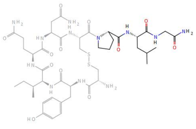	285.1926	285.1921	-1.62
MATCH	43.9	136.0758	136.0757	-0.47	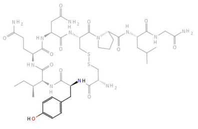	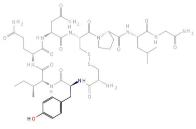	136.0758	136.0757	-0.47

Metabolite: Substrate

Type	score	sub. m/z observed	sub. m/z calculated	sub ppm			met. m/z observed	met. m/z calculated	met. ppm
MATCH	28.3	101.0714	101.0709	-4.41	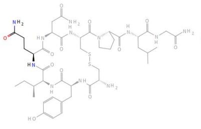	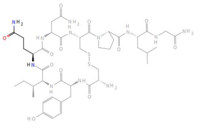	101.0714	101.0709	-4.41
MATCH	200.0	86.0971	86.0964	-7.81	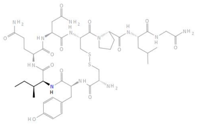	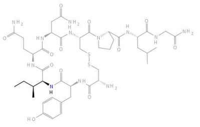	86.0971	86.0964	-7.81
MATCH	200.0	86.0971	86.0964	-7.81	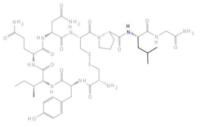	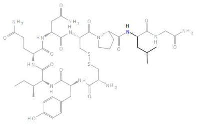	86.0971	86.0964	-7.81
MATCH	70.7	84.0451	84.0444	-8.98	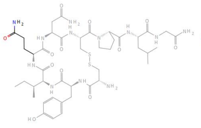	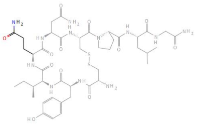	84.0451	84.0444	-8.98
MATCH	134.0	70.0660	70.0651	-12.2	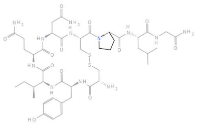	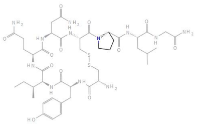	70.0660	70.0651	-12.2

MS (+) FT

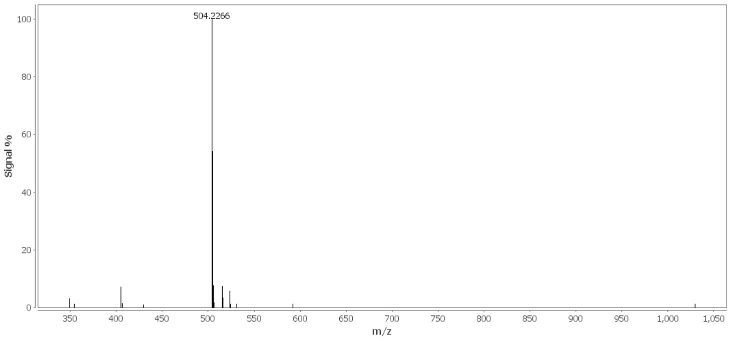

MS (+) FT

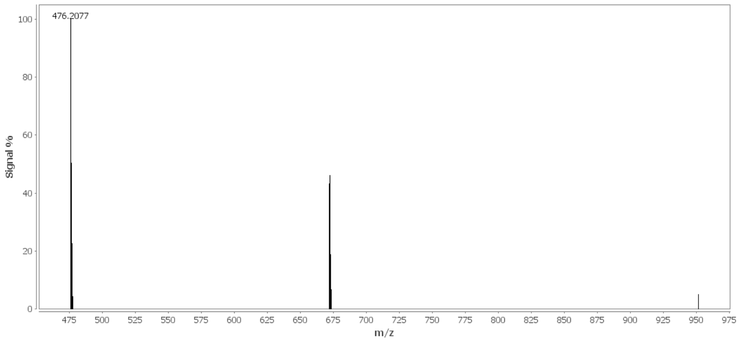

MS2 (+) FT activ = HCD:ce =

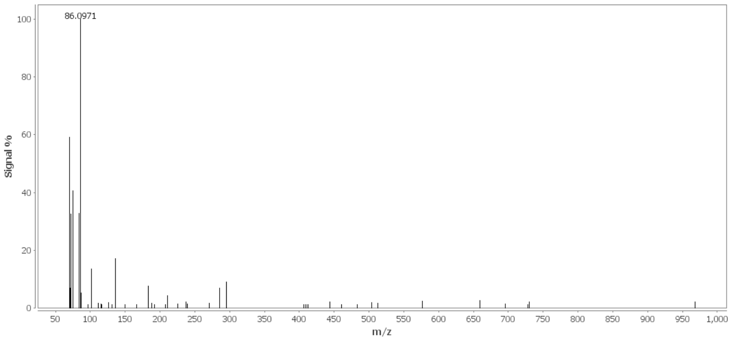

MS2 (+) FT activ = HCD:ce =

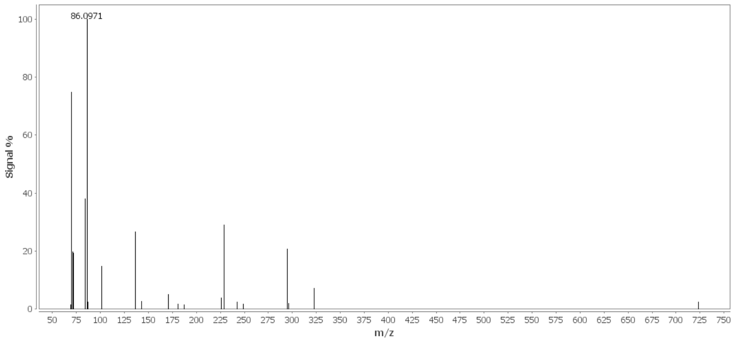

Metabolite: M1 -56 RT=2.02

Type	score	sub. m/z observed	sub. m/z calculated	sub ppm		met. m/z observed	met. m/z calculated	met. ppm
MATCH	200.0	504.2266	504.2255	-2.12	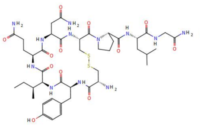 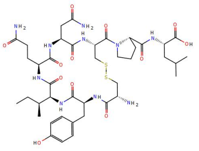	476.2077	476.2068	-1.93
MATCH	105.0	504.2266	504.2255	-2.12	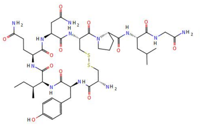 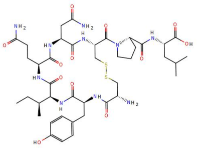	951.4081	951.4063	-1.87
MATCH	134.0	70.0660	70.0651	-12.2	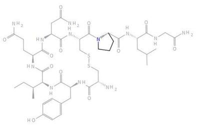 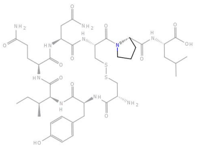	70.0660	70.0651	-11.9
MATCH	70.7	84.0451	84.0444	-8.98	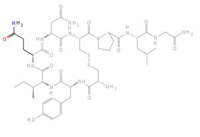 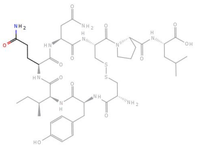	84.0450	84.0444	-7.59
MATCH	200.0	86.0971	86.0964	-7.81	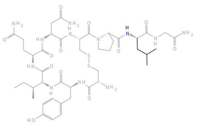 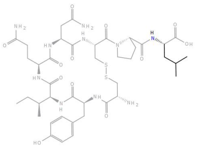	86.0971	86.0964	-7.85
MATCH	200.0	86.0971	86.0964	-7.81	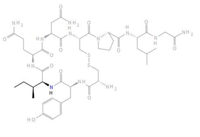 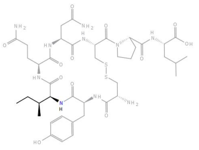	86.0971	86.0964	-7.85
MATCH	28.3	101.0714	101.0709	-4.41	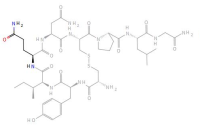 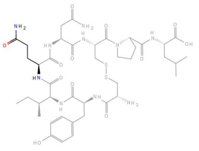	101.0714	101.0709	-4.57
MATCH	43.9	136.0758	136.0757	-0.47	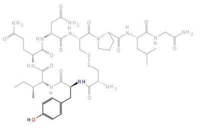 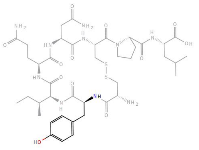	136.0757	136.0757	-0.21
MATCH	36.0	285.1926	285.1921	-1.62	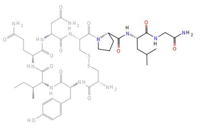 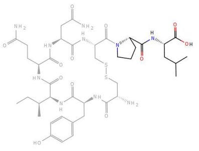	229.1543	229.1547	1.53

Metabolite: M1 -56 RT=2.02

Type	score	sub. m/z observed	sub. m/z calculated	sub ppm		met. m/z observed	met. m/z calculated	met. ppm
MET_MATCH					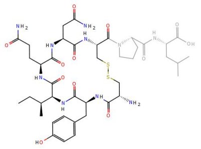	723.2560	723.2589	4.03
