# Supplementary material for: Software-aided approach to investigate peptide structure and metabolic susceptibility of amide bonds in peptide drugs based on high resolution mass spectrometry
Source: PLoS One. 2017 Nov 1;12(11):e0186461. doi: 10.1371/journal.pone.0186461 (PMC5665424; doi:10.1371/journal.pone.0186461)
Supplement: S1 File — (ZIP) [file pone.0186461.s007.zip › SFiles/S37_File.pdf]

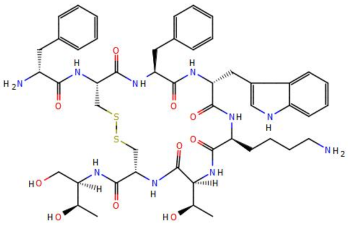

Object 1

Property name	Property value
Time	0min, 5min, 15min, 45min, 120min
Instrument	ThermoQAPlus
Matrix	chymotrypsin
Acquisition Mode	ddMS2

Chromatograms

Time=0min

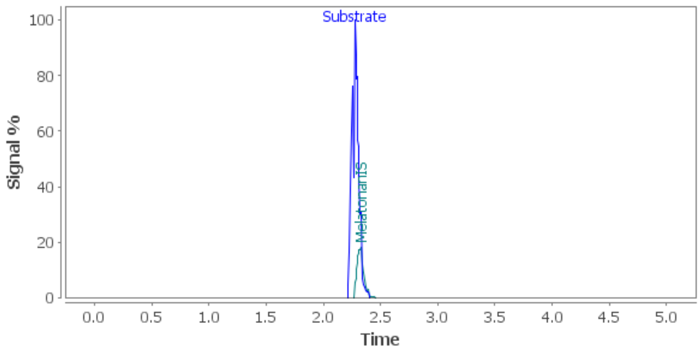

Time=5min

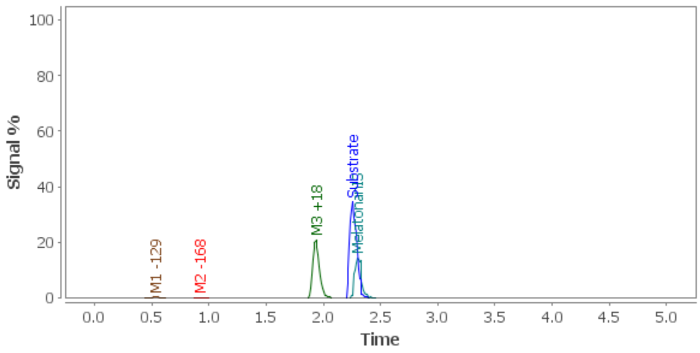

Time=15min

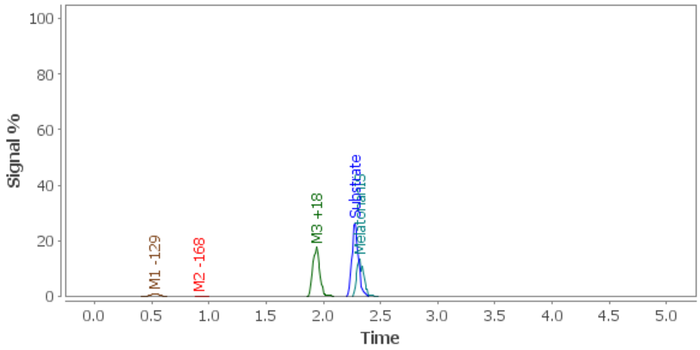

Time=45min

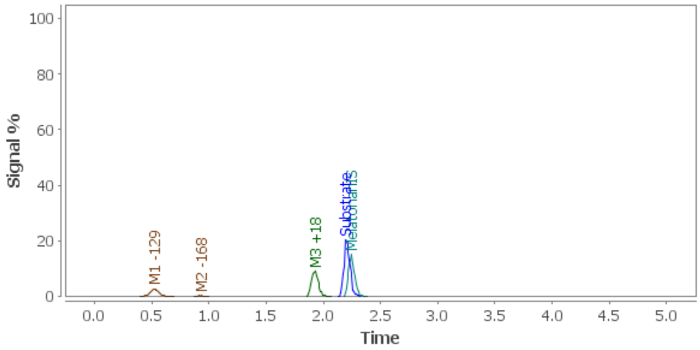

Time=120min

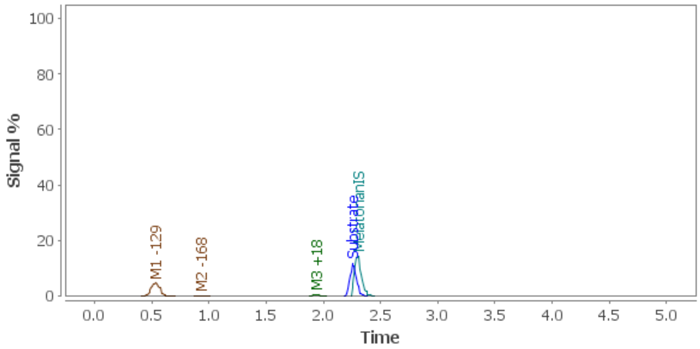

Custom Charts

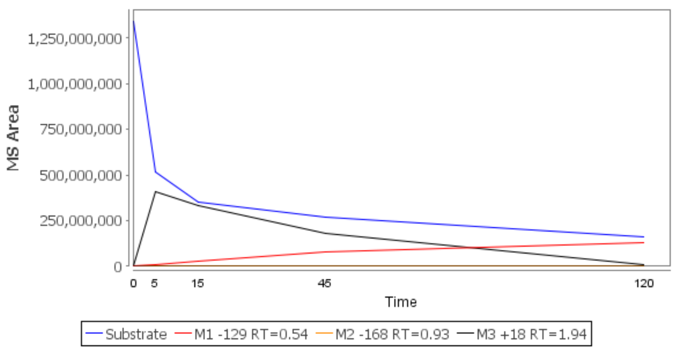

Fragmentation

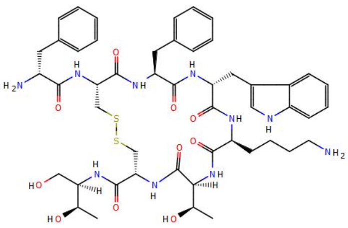

Object 1

MS (+) FT

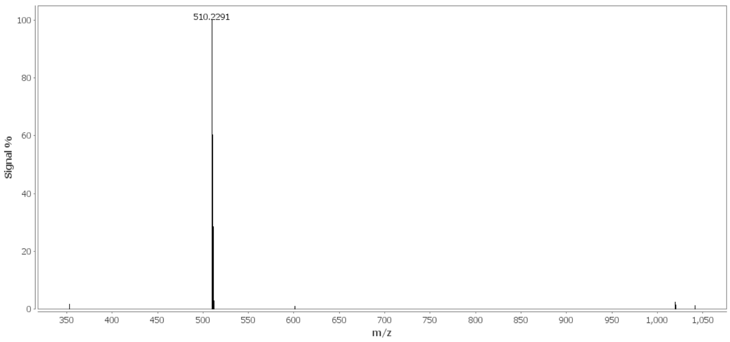

MS (+) FT

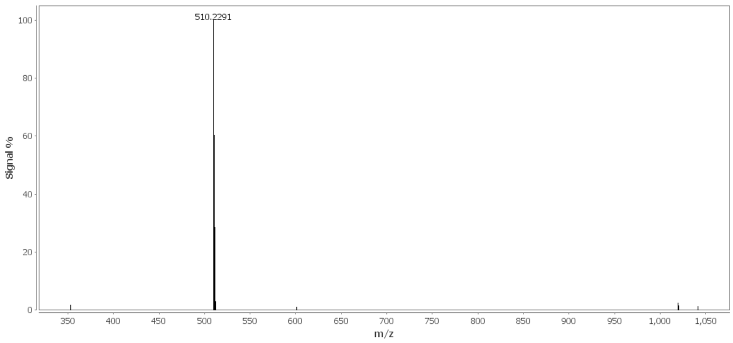

MS2 (+) FT activ = HCD:ce =

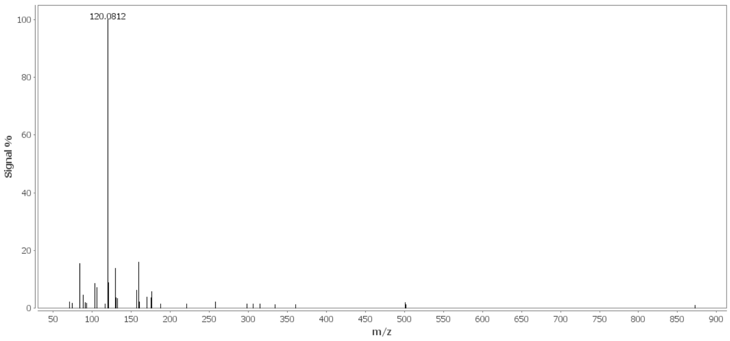

MS2 (+) FT activ = HCD:ce =

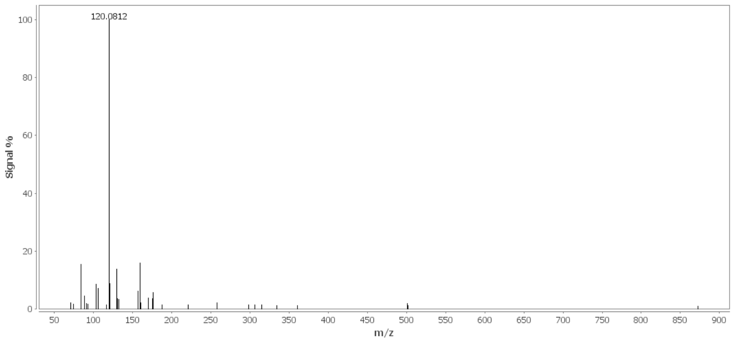

Metabolite: Substrate

Type	score	sub. m/z observed	sub. m/z calculated	sub ppm		met. m/z observed	met. m/z calculated	met. ppm
MISMATCH	102.4	1019.4516	1019.4478	-3.78	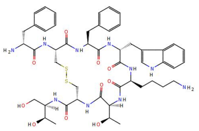 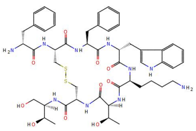	1019.4516	1019.4478	-3.78
MATCH	2.1	872.3735	872.3793	6.74	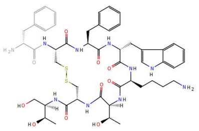 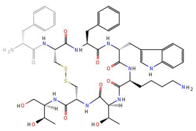	872.3735	872.3793	6.74
MISMATCH	200.0	510.2291	510.2275	-3.06	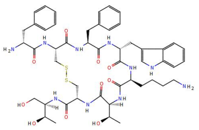 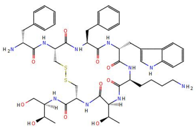	510.2291	510.2275	-3.06

Metabolite: Substrate

Type	score	sub. m/z observed	sub. m/z calculated	sub ppm			met. m/z observed	met. m/z calculated	met. ppm
MISMATCH	-4.0	360.1979	360.2030	14.20	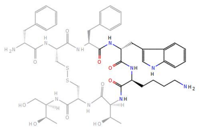	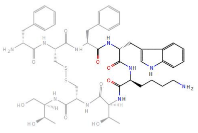	360.1979	360.2030	14.20
MISMATCH	-3.1	334.1549	334.1550	0.25	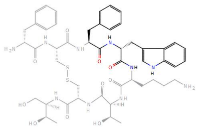	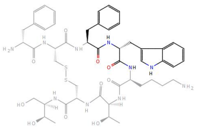	334.1549	334.1550	0.25
MISMATCH	-3.4	298.1555	298.1550	-1.64	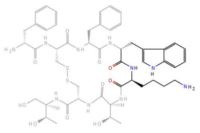	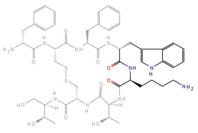	298.1555	298.1550	-1.64
MISMATCH	-5.1	258.1453	258.1448	-1.78	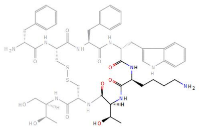	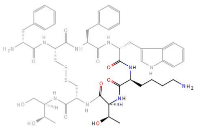	258.1453	258.1448	-1.78
MISMATCH	3.5	221.0747	221.0743	-1.73	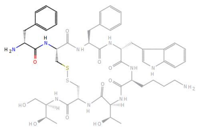	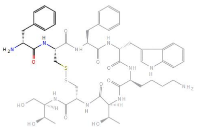	221.0747	221.0743	-1.73
MISMATCH	-3.6	187.0869	187.0866	-1.45	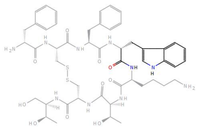	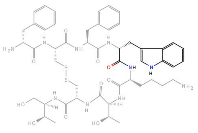	187.0869	187.0866	-1.45
MISMATCH	47.6	159.0918	159.0917	-0.85	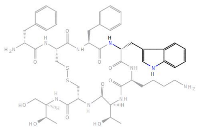	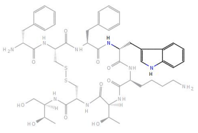	159.0918	159.0917	-0.85
MISMATCH	-13.4	157.0973	157.0972	-1.24	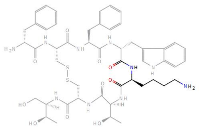	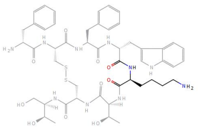	157.0973	157.0972	-1.24
MISMATCH	10.7	130.0653	130.0681	20.94	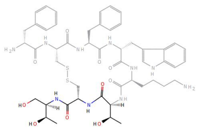	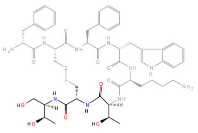	130.0653	130.0681	20.94

Metabolite: Substrate

Type	score	sub. m/z observed	sub. m/z calculated	sub ppm			met. m/z observed	met. m/z calculated	met. ppm
MISMATCH	-27.7	129.1025	129.1022	-1.96	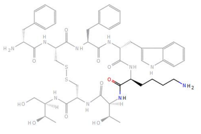	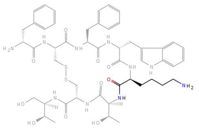	129.1025	129.1022	-1.96
MATCH	200.0	120.0812	120.0808	-3.24	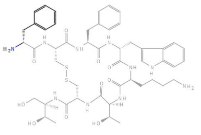	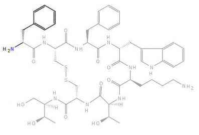	120.0812	120.0808	-3.24
MISMATCH	-2.9	116.0710	116.0706	-3.06	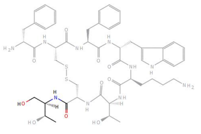	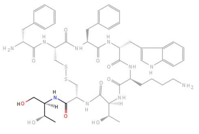	116.0710	116.0706	-3.06
MISMATCH	-2.9	116.0710	116.0706	-3.06	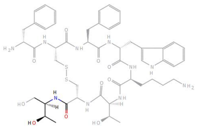	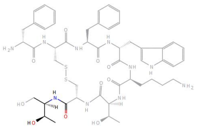	116.0710	116.0706	-3.06
MISMATCH	-11.6	106.0868	106.0863	-5.58	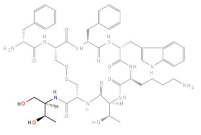	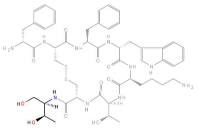	106.0868	106.0863	-5.58
MATCH	14.0	103.0548	103.0542	-5.83	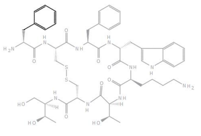	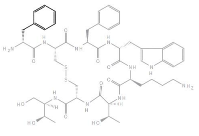	103.0548	103.0542	-5.83
MISMATCH	-12.1	103.0548	103.0542	-5.83	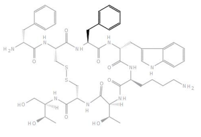	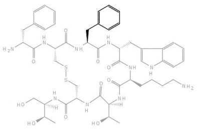	103.0548	103.0542	-5.83
MISMATCH	-3.3	93.0705	93.0679	-28.3	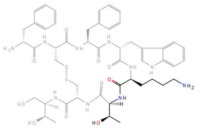	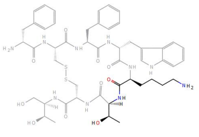	93.0705	93.0679	-28.3
MISMATCH	-7.0	88.0764	88.0757	-8.27	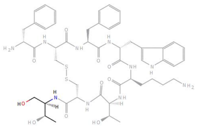	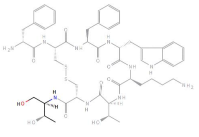	88.0764	88.0757	-8.27

Metabolite: Substrate

Type	score	sub. m/z observed	sub. m/z calculated	sub ppm			met. m/z observed	met. m/z calculated	met. ppm
MISMATCH	-7.0	88.0764	88.0757	-8.27	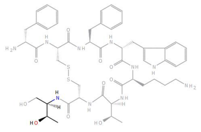	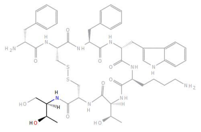	88.0764	88.0757	-8.27
MISMATCH	-53.5	84.0816	84.0808	-9.34	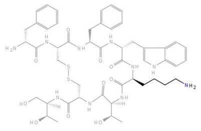	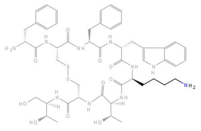	84.0816	84.0808	-9.34

MS (+) FT

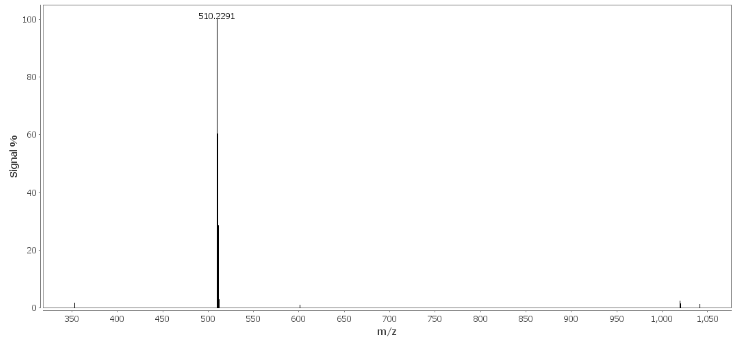

MS (+) FT

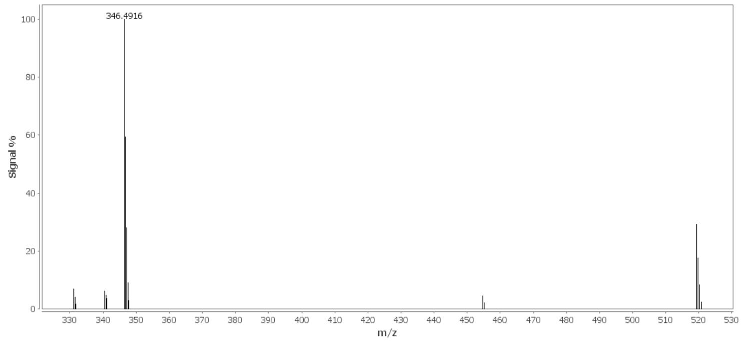

MS2 (+) FT activ = HCD:ce =

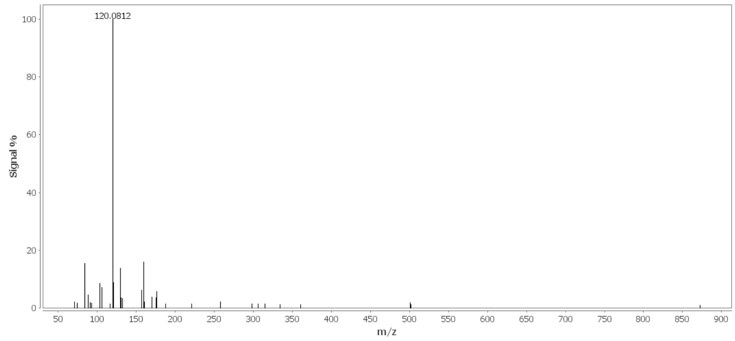

MS2 (+) FT activ = HCD:ce =

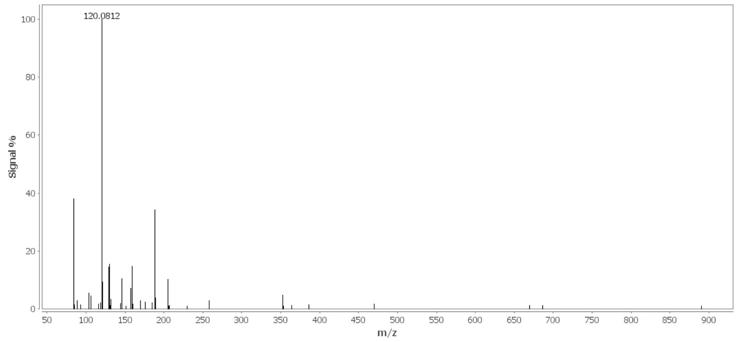

Metabolite: M3 +18 RT=1.94

Type	score	sub. m/z observed	sub. m/z calculated	sub ppm			met. m/z observed	met. m/z calculated	met. ppm
MATCH	200.0	510.2291	510.2275	-3.06	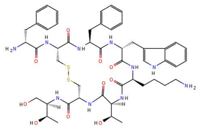	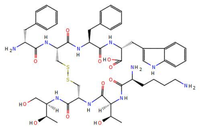	346.4916	346.4910	-1.99
MATCH	200.0	510.2291	510.2275	-3.06	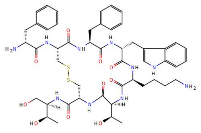	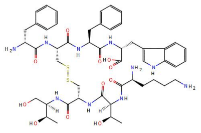	346.4916	346.4910	-1.99

Metabolite: M3 +18 RT=1.94

Type	score	sub. m/z observed	sub. m/z calculated	sub ppm			met. m/z observed	met. m/z calculated	met. ppm
MATCH	129.2	510.2291	510.2275	-3.06	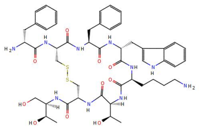	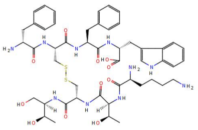	519.2340	519.2328	-2.33
MATCH	129.2	510.2291	510.2275	-3.06	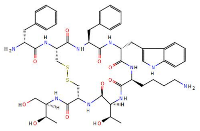	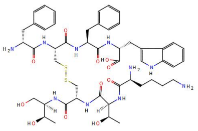	519.2340	519.2328	-2.33
MATCH	102.4	1019.4516	1019.4478	-3.78	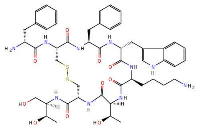	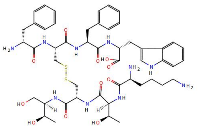	346.4916	346.4910	-1.99
MATCH	102.4	1019.4516	1019.4478	-3.78	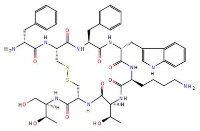	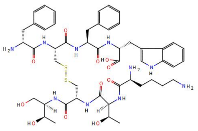	346.4916	346.4910	-1.99
MATCH	31.6	1019.4516	1019.4478	-3.78	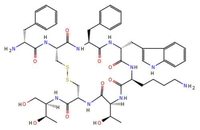	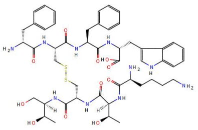	519.2340	519.2328	-2.33
MATCH	31.6	1019.4516	1019.4478	-3.78	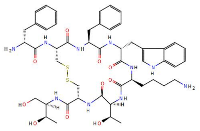	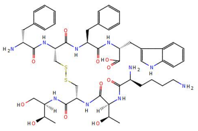	519.2340	519.2328	-2.33
MATCH	14.0	103.0548	103.0542	-5.83	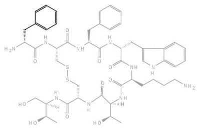	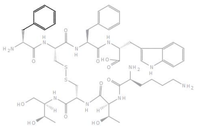	103.0549	103.0542	-6.12
MATCH	200.0	120.0812	120.0808	-3.24	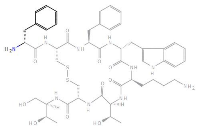	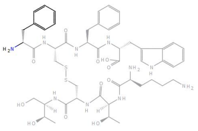	120.0812	120.0808	-3.60
MATCH	2.1	872.3735	872.3793	6.74	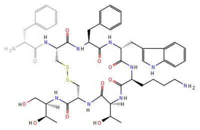	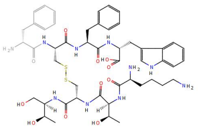	890.3885	890.3899	1.56

Metabolite: M3 +18 RT=1.94

Type	score	sub. m/z observed	sub. m/z calculated	sub ppm		met. m/z observed	met. m/z calculated	met. ppm
MISMATCH	-106.2	510.2291	510.2275	-3.06	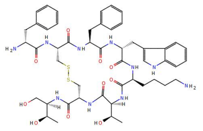	340.4880	340.4880	0.00
MISMATCH	-8.6	1019.4516	1019.4478	-3.78	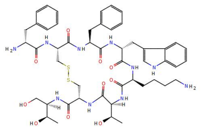	340.4880	340.4880	0.00
MISMATCH	-53.5	84.0816	84.0808	-9.34	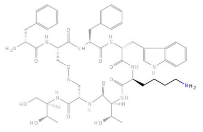	84.0816	84.0816	0.00
MISMATCH	-7.3	88.0764	88.0757	-8.27	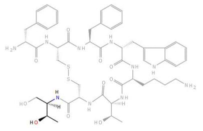	88.0764	88.0764	0.00
MISMATCH	-9.0	88.0764	88.0757	-8.27	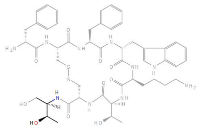	106.0868	106.0868	0.00
MISMATCH	-3.3	93.0705	93.0679	-28.3	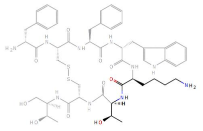	93.0705	93.0705	0.00
MISMATCH	-11.6	106.0868	106.0863	-5.58	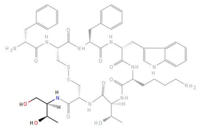	106.0868	106.0868	0.00
MISMATCH	-2.9	116.0710	116.0706	-3.06	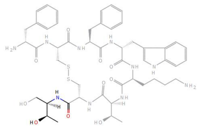	116.0710	116.0710	0.00
MISMATCH	-28.4	129.1025	129.1022	-1.96	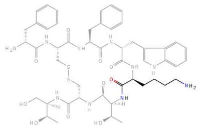	129.1025	129.1025	0.00

Metabolite: M3 +18 RT=1.94

Type	score	sub. m/z observed	sub. m/z calculated	sub ppm		met. m/z observed	met. m/z calculated	met. ppm
MISMATCH	-19.1	130.0653	130.0681	20.94	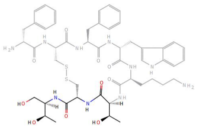	130.0654	130.0654	0.00
MISMATCH	-13.4	157.0973	157.0972	-1.24	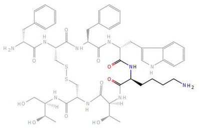	157.0975	157.0975	0.00
MISMATCH	-30.9	159.0918	159.0917	-0.85	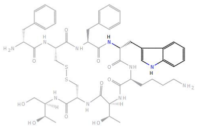	159.0919	159.0919	0.00
MISMATCH	-6.9	187.0869	187.0866	-1.45	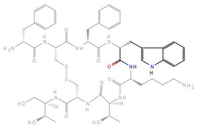	103.0549	103.0549	0.00
MISMATCH	-11.6	187.0869	187.0866	-1.45	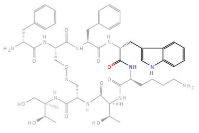	205.0974	205.0974	0.00
MISMATCH	-5.1	258.1453	258.1448	-1.78	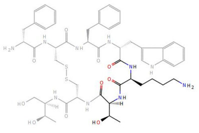	258.1452	258.1452	0.00
MISMATCH	-3.5	334.1549	334.1550	0.25	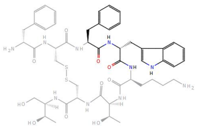	118.0655	118.0655	0.00
MISMATCH	-6.0	334.1549	334.1550	0.25	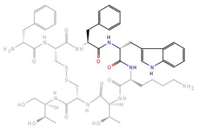	352.1658	352.1658	0.00
MET_MATCH					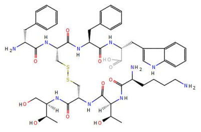	331.1566	331.1558	-2.33

Metabolite: M3 +18 RT=1.94

Type	score	sub. m/z observed	sub. m/z calculated	sub ppm		met. m/z observed	met. m/z calculated	met. ppm
MET_MATCH					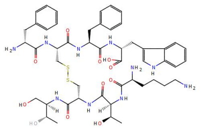	340.4880	340.4874	-1.66
MET_MATCH					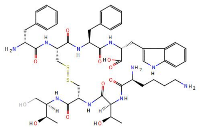	340.4880	340.4874	-1.66
MET_MATCH					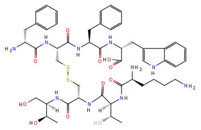	340.4880	340.4874	-1.66
MET_MATCH					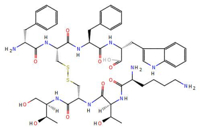	341.1509	341.1593	24.80
MET_MATCH					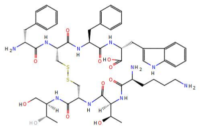	341.1509	341.1593	24.80
MET_MATCH					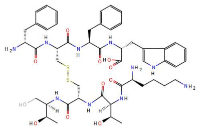	341.1509	341.1593	24.80
MET_MATCH					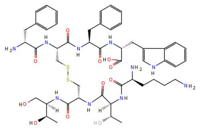	341.1509	341.1593	24.80
MET_MATCH					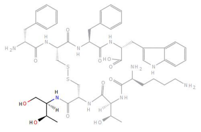	106.0868	106.0863	-4.95
MET_MATCH					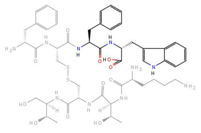	118.0655	118.0600	-45.8

Metabolite: M3 +18 RT=1.94

Type	score	sub. m/z observed	sub. m/z calculated	sub ppm		met. m/z observed	met. m/z calculated	met. ppm
MET_MATCH					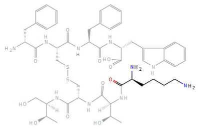	129.1025	129.1022	-2.23
MET_MATCH					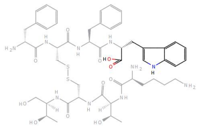	188.0709	188.0706	-1.55
MET_MATCH					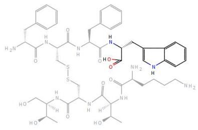	205.0974	205.0972	-1.24
MET_MATCH					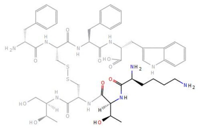	230.1501	230.1499	-0.62
MET_MATCH					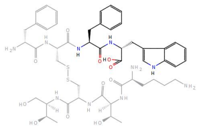	352.1658	352.1656	-0.71
MET_MATCH					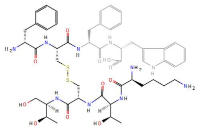	352.1658	352.1669	3.15
MET_MATCH					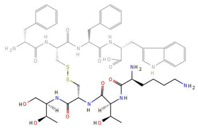	470.2091	470.2102	2.16
MET_MATCH					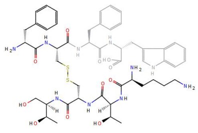	686.2962	686.3000	5.53

MS (+) FT

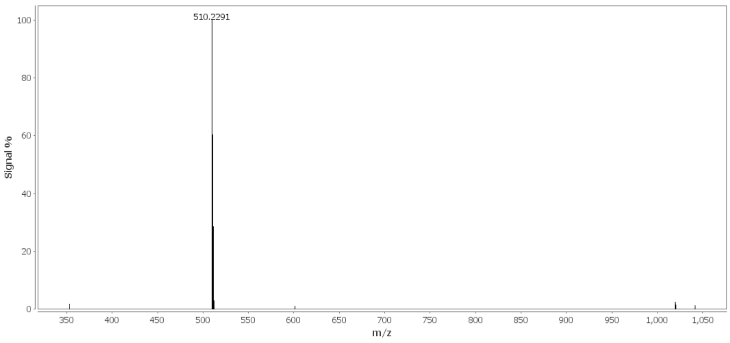

MS (+) FT

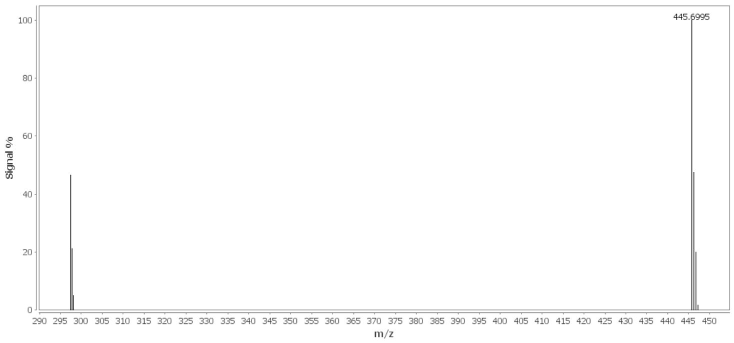

MS2 (+) FT activ = HCD:ce =

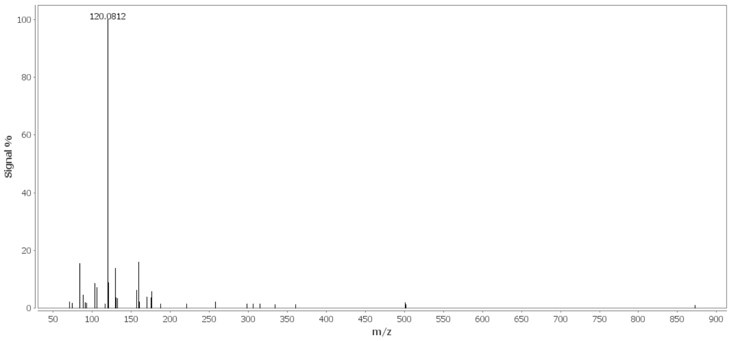

MS2 (+) FT activ = HCD:ce =

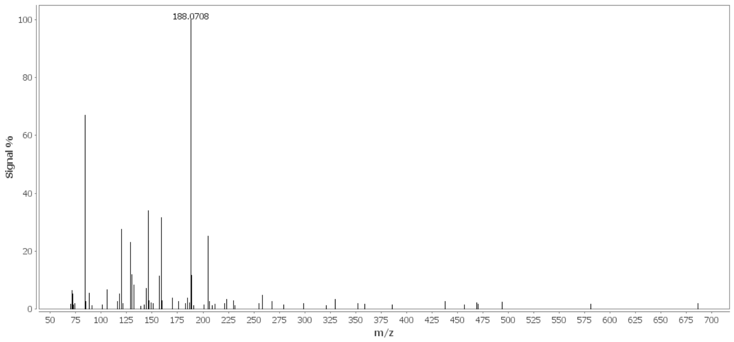

Metabolite: M1 -129 RT=0.54

Type	score	sub. m/z observed	sub. m/z calculated	sub ppm			met. m/z observed	met. m/z calculated	met. ppm
MATCH	146.6	510.2291	510.2275	-3.06	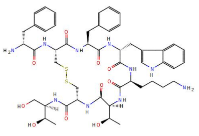	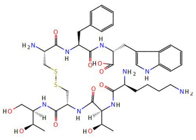	297.4688	297.4682	-2.22
MATCH	146.6	510.2291	510.2275	-3.06	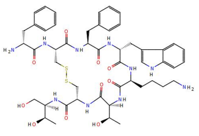	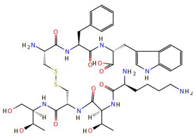	297.4688	297.4682	-2.22
MATCH	200.0	510.2291	510.2275	-3.06	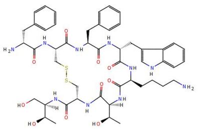	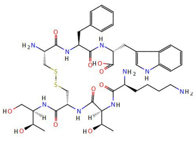	445.6995	445.6986	-1.95
MATCH	200.0	510.2291	510.2275	-3.06	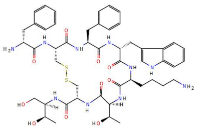	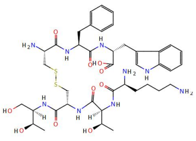	445.6995	445.6986	-1.95
MATCH	49.0	1019.4516	1019.4478	-3.78	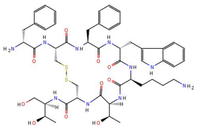	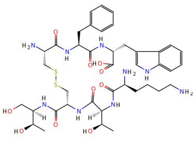	297.4688	297.4682	-2.22

Metabolite: M1 -129 RT=0.54

Type	score	sub. m/z observed	sub. m/z calculated	sub ppm			met. m/z observed	met. m/z calculated	met. ppm
MATCH	49.0	1019.4516	1019.4478	-3.78	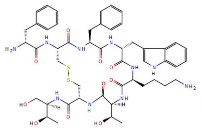	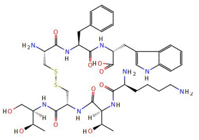	297.4688	297.4682	-2.22
MATCH	102.4	1019.4516	1019.4478	-3.78	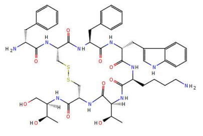	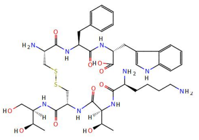	445.6995	445.6986	-1.95
MATCH	102.4	1019.4516	1019.4478	-3.78	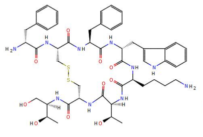	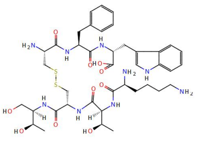	445.6995	445.6986	-1.95
MATCH	47.6	159.0918	159.0917	-0.85	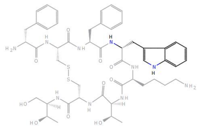	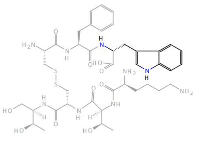	159.0918	159.0917	-0.96
MATCH	3.5	221.0747	221.0743	-1.73	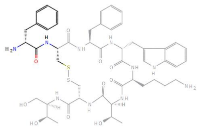	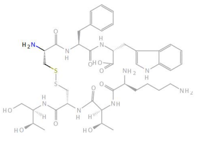	74.0066	74.0059	-9.82
MISMATCH	-82.4	84.0816	84.0808	-9.34	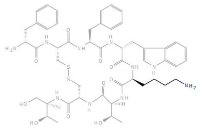		84.0816	84.0816	0.00
MISMATCH	-10.0	88.0764	88.0757	-8.27	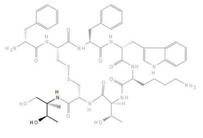		88.0764	88.0764	0.00
MISMATCH	-11.1	88.0764	88.0757	-8.27	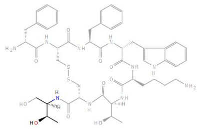		106.0868	106.0868	0.00
MISMATCH	-13.7	106.0868	106.0863	-5.58	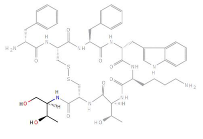		106.0868	106.0868	0.00

Metabolite: M1 -129 RT=0.54

Type	score	sub. m/z observed	sub. m/z calculated	sub ppm		met. m/z observed	met. m/z calculated	met. ppm
MISMATCH	-4.0	116.0710	116.0706	-3.06	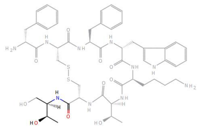	116.0708	116.0708	0.00
MISMATCH	-127.7	120.0812	120.0808	-3.24	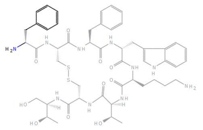	120.0812	120.0812	0.00
MISMATCH	-37.0	129.1025	129.1022	-1.96	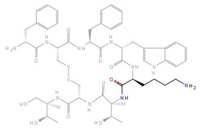	129.1026	129.1026	0.00
MISMATCH	-15.4	130.0653	130.0681	20.94	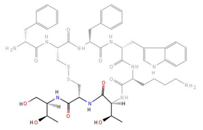	130.0653	130.0653	0.00
MISMATCH	-11.7	132.0809	132.0837	21.34	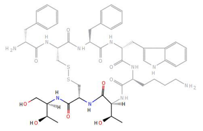	132.0809	132.0809	0.00
MISMATCH	-17.7	157.0973	157.0972	-1.24	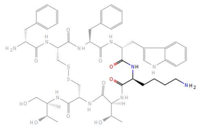	157.0974	157.0974	0.00
MISMATCH	-3.6	187.0869	187.0866	-1.45	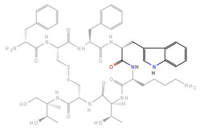	187.0869	187.0869	0.00
MISMATCH	-26.6	187.0869	187.0866	-1.45	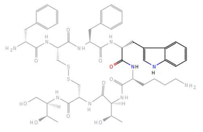	205.0975	205.0975	0.00
MISMATCH	-3.3	221.0747	221.0743	-1.73	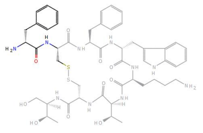	221.0748	221.0748	0.00

Metabolite: M1 -129 RT=0.54

Type	score	sub. m/z observed	sub. m/z calculated	sub ppm		met. m/z observed	met. m/z calculated	met. ppm
MISMATCH	-7.0	258.1453	258.1448	-1.78	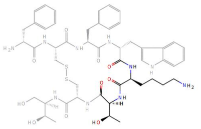	258.1452	258.1452	0.00
MISMATCH	-3.4	298.1555	298.1550	-1.64	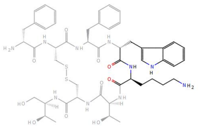	151.0869	151.0869	0.00
MISMATCH	-6.6	334.1549	334.1550	0.25	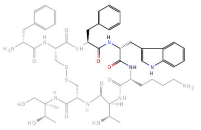	118.0655	118.0655	0.00
MISMATCH	-3.4	334.1549	334.1550	0.25	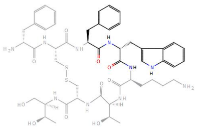	187.0869	187.0869	0.00
MISMATCH	-26.4	334.1549	334.1550	0.25	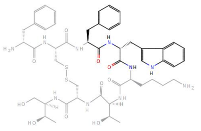	205.0975	205.0975	0.00
MISMATCH	-3.1	334.1549	334.1550	0.25	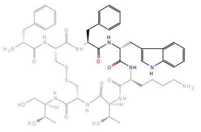	352.1652	352.1652	0.00
MISMATCH	-4.0	360.1979	360.2030	14.20	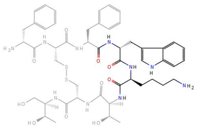	116.0708	116.0708	0.00
MET_MATCH					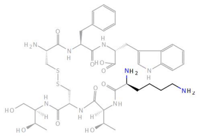	101.1078	101.1073	-4.81
MET_MATCH					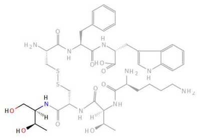	106.0868	106.0863	-5.21

Metabolite: M1 -129 RT=0.54

Type	score	sub. m/z observed	sub. m/z calculated	sub ppm		met. m/z observed	met. m/z calculated	met. ppm
MET_MATCH					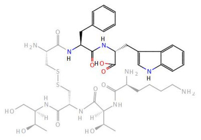	118.0655	118.0600	-46.5
MET_MATCH					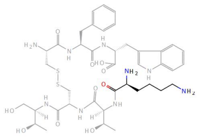	129.1026	129.1022	-2.52
MET_MATCH					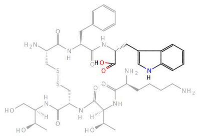	188.0708	188.0706	-1.06
MET_MATCH					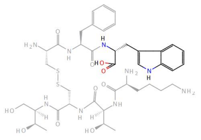	205.0975	205.0972	-1.52
MET_MATCH					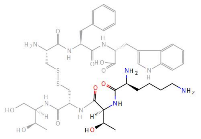	230.1504	230.1499	-2.25
MET_MATCH					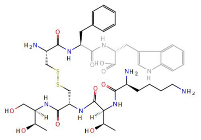	329.6568	329.6562	-1.83
MET_MATCH					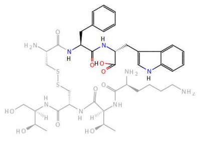	352.1652	352.1656	0.93
MET_MATCH					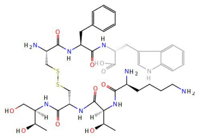	352.1652	352.1669	4.80
MET_MATCH					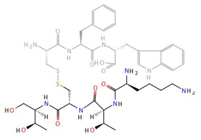	438.2398	438.2381	-4.02

Metabolite: M1 -129 RT=0.54

Type	score	sub. m/z observed	sub. m/z calculated	sub ppm		met. m/z observed	met. m/z calculated	met. ppm
MET_MATCH					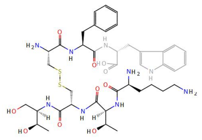	686.2963	686.3000	5.40

MS (+) FT

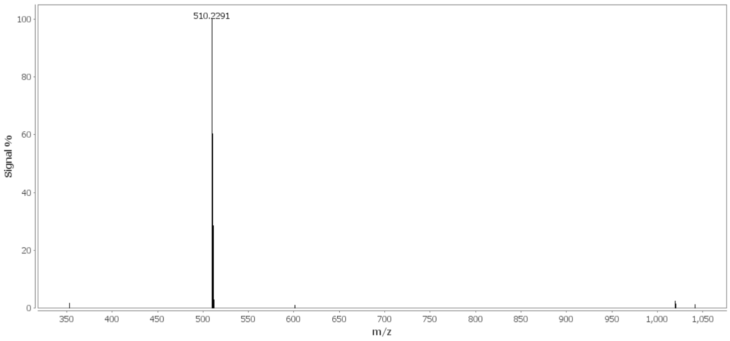

MS (+) FT

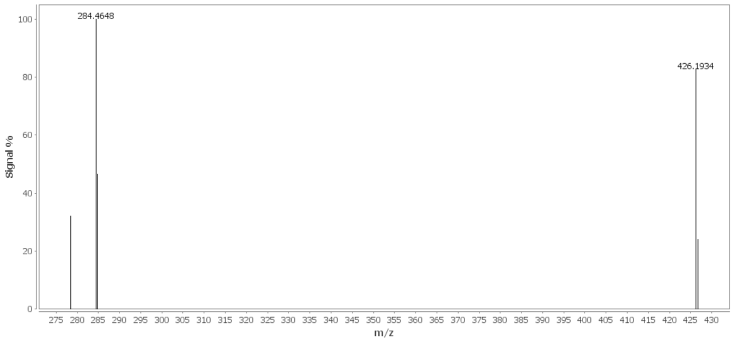

MS2 (+) FT activ = HCD:ce =

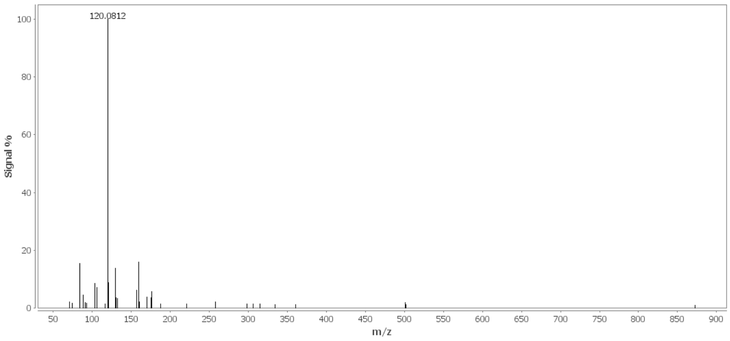

MS2 (+) FT activ = HCD:ce =

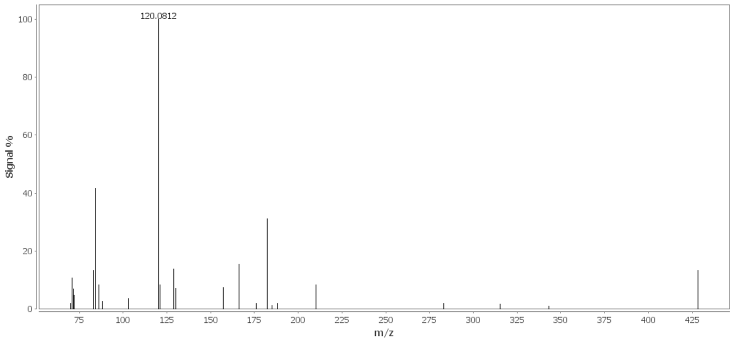

Metabolite: M2 -168 RT=0.93

Type	score	sub. m/z observed	sub. m/z calculated	sub ppm			met. m/z observed	met. m/z calculated	met. ppm
MATCH	200.0	510.2291	510.2275	-3.06	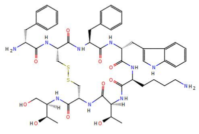	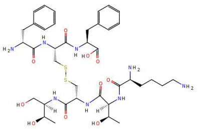	284.4648	284.4645	-1.09
MATCH	200.0	510.2291	510.2275	-3.06	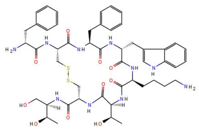	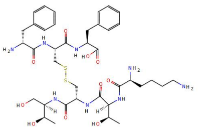	284.4648	284.4645	-1.09
MATCH	182.6	510.2291	510.2275	-3.06	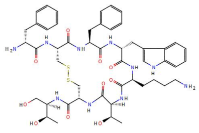	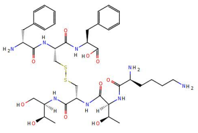	426.1934	426.1931	-0.66

Metabolite: M2 -168 RT=0.93

Type	score	sub. m/z observed	sub. m/z calculated	sub ppm		met. m/z observed	met. m/z calculated	met. ppm
MATCH	182.6	510.2291	510.2275	-3.06	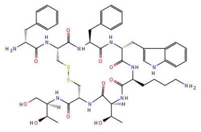 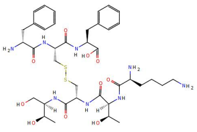	426.1934	426.1931	-0.66
MATCH	102.4	1019.4516	1019.4478	-3.78	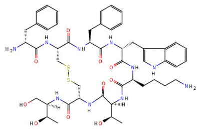 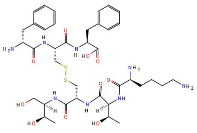	284.4648	284.4645	-1.09
MATCH	102.4	1019.4516	1019.4478	-3.78	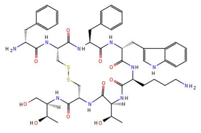 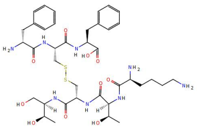	284.4648	284.4645	-1.09
MATCH	85.0	1019.4516	1019.4478	-3.78	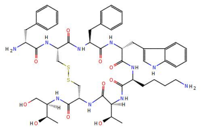 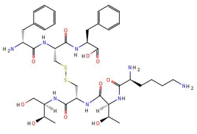	426.1934	426.1931	-0.66
MATCH	85.0	1019.4516	1019.4478	-3.78	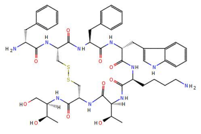 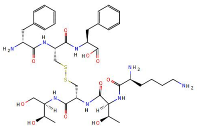	426.1934	426.1931	-0.66
MATCH	10.7	130.0653	130.0681	20.94	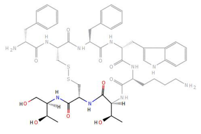 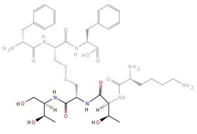	130.0654	130.0681	20.20
MISMATCH	-132.1	510.2291	510.2275	-3.06	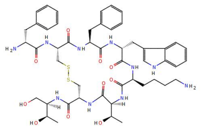	278.4615	278.4615	0.00
MISMATCH	-34.6	1019.4516	1019.4478	-3.78	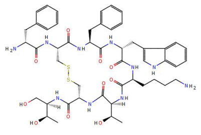	278.4615	278.4615	0.00
MISMATCH	-57.0	84.0816	84.0808	-9.34	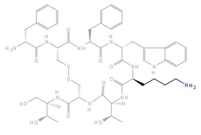	84.0816	84.0816	0.00

Metabolite: M2 -168 RT=0.93

Type	score	sub. m/z observed	sub. m/z calculated	sub ppm		met. m/z observed	met. m/z calculated	met. ppm
MISMATCH	-7.0	88.0764	88.0757	-8.27	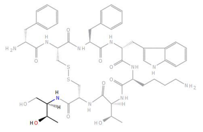	88.0765	88.0765	0.00
MISMATCH	-12.1	103.0548	103.0542	-5.83	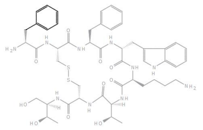	103.0549	103.0549	0.00
MISMATCH	-200.0	120.0812	120.0808	-3.24	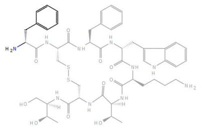	120.0812	120.0812	0.00
MISMATCH	-27.7	129.1025	129.1022	-1.96	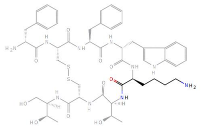	129.1027	129.1027	0.00
MISMATCH	-16.8	334.1549	334.1550	0.25	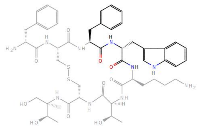	166.0867	166.0867	0.00
MET_MATCH					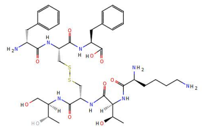	278.4615	278.4610	-1.96
MET_MATCH					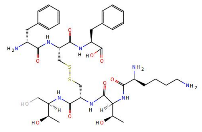	278.4615	278.4610	-1.96
MET_MATCH					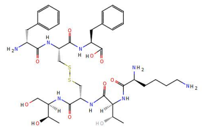	278.4615	278.4610	-1.96
MET_MATCH					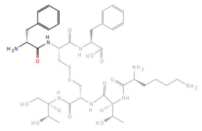	83.0612	83.0548	-78.0

Metabolite: M2 -168 RT=0.93

Type	score	sub. m/z observed	sub. m/z calculated	sub ppm		met. m/z observed	met. m/z calculated	met. ppm
MET_MATCH					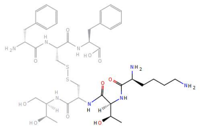	83.0612	83.0637	29.27
MET_MATCH					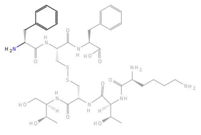	120.0812	120.0808	-3.61
MET_MATCH					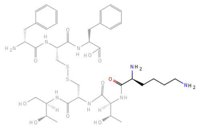	129.1027	129.1022	-3.49
MET_MATCH					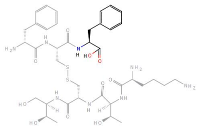	166.0867	166.0863	-2.52
