# Supplementary material for: Software-aided approach to investigate peptide structure and metabolic susceptibility of amide bonds in peptide drugs based on high resolution mass spectrometry
Source: PLoS One. 2017 Nov 1;12(11):e0186461. doi: 10.1371/journal.pone.0186461 (PMC5665424; doi:10.1371/journal.pone.0186461)
Supplement: S1 File — (ZIP) [file pone.0186461.s007.zip › SFiles/S38_File.pdf]

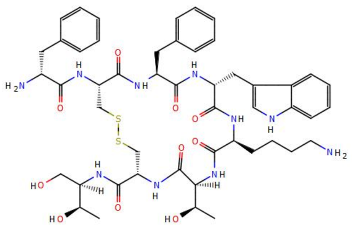

Object 1

Property name	Property value
Time	0min, 5min, 15min, 45min, 120min
Instrument	ThermoQAPlus
Matrix	elastase
Acquisition Mode	ddMS2

Chromatograms

Time=0min

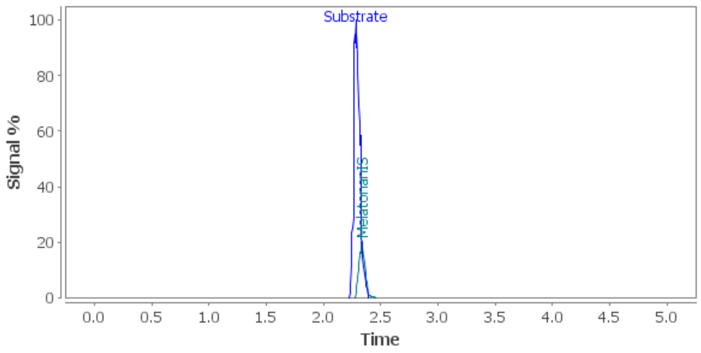

Time=5min

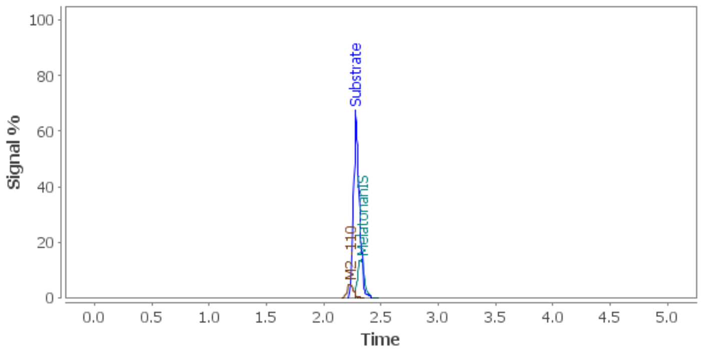

Time=15min

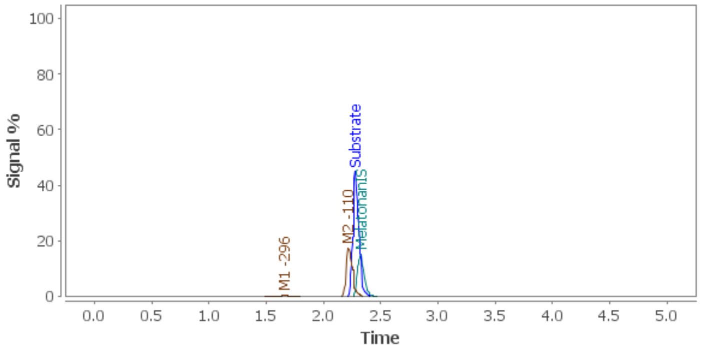

Time=45min

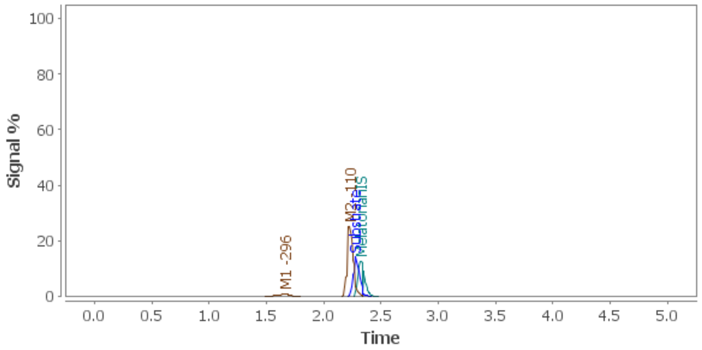

Time=120min

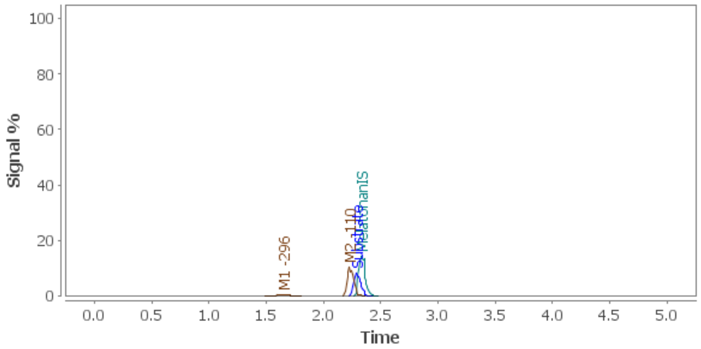

Custom Charts

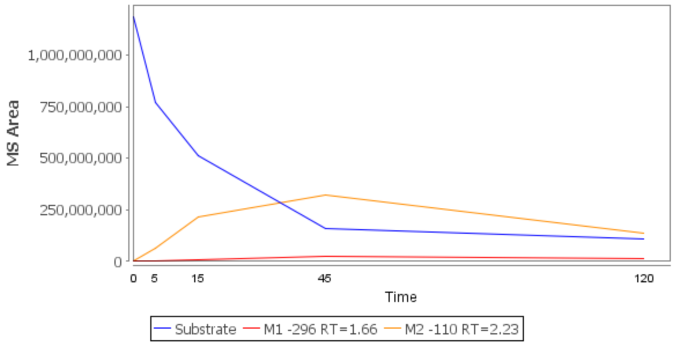

Fragmentation

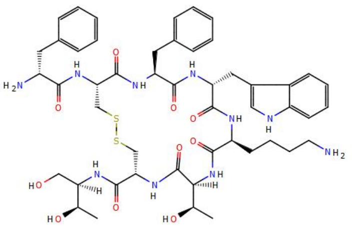

Object 1

MS (+) FT

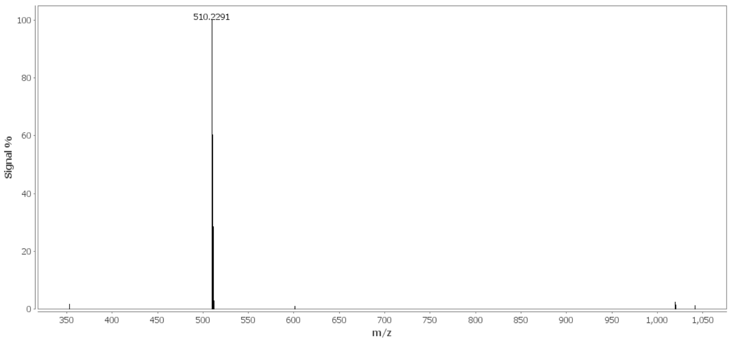

MS (+) FT

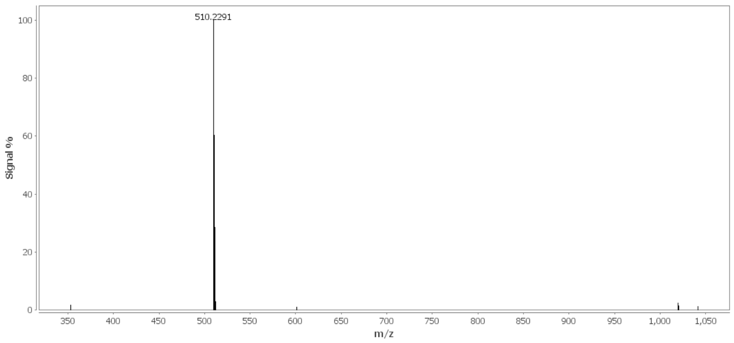

MS2 (+) FT activ = HCD:ce =

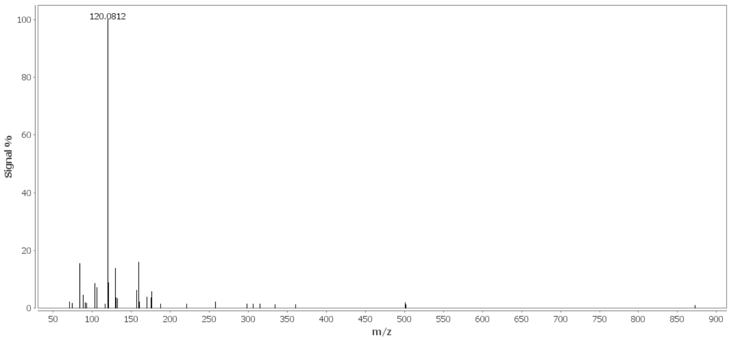

MS2 (+) FT activ = HCD:ce =

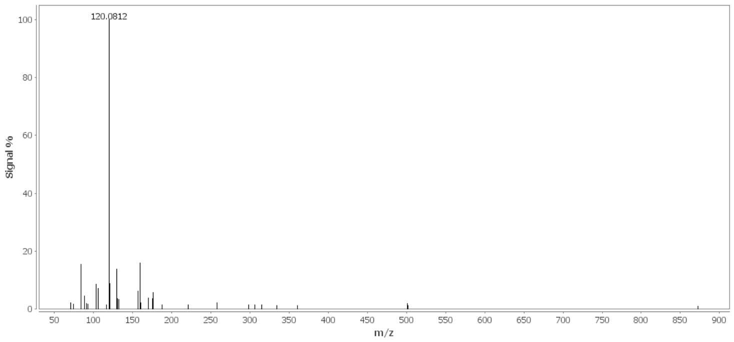

Metabolite: Substrate

Type	score	sub. m/z observed	sub. m/z calculated	sub ppm		met. m/z observed	met. m/z calculated	met. ppm
MATCH	101.7	1019.4500	1019.4478	-2.21	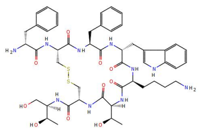 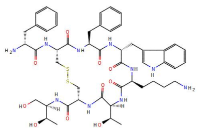	1019.4500	1019.4478	-2.21
MATCH	200.0	510.2289	510.2275	-2.67	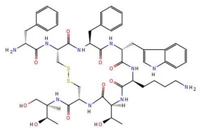 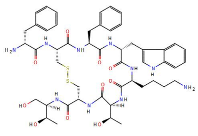	510.2289	510.2275	-2.67
MISMATCH	-5.5	315.1819	315.1816	-1.10	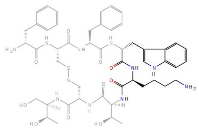 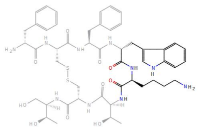	315.1819	315.1816	-1.10

Metabolite: Substrate

Type	score	sub. m/z observed	sub. m/z calculated	sub ppm			met. m/z observed	met. m/z calculated	met. ppm
MISMATCH	-24.7	258.1453	258.1448	-1.75	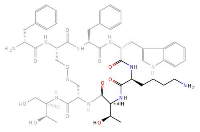	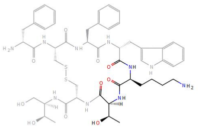	258.1453	258.1448	-1.75
MISMATCH	-5.4	187.0870	187.0866	-1.99	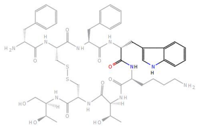	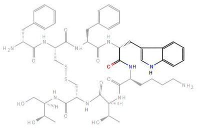	187.0870	187.0866	-1.99
MISMATCH	-29.9	159.0921	159.0917	-2.38	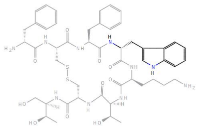	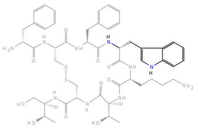	159.0921	159.0917	-2.38
MISMATCH	-5.9	132.0812	132.0837	19.34	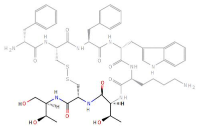	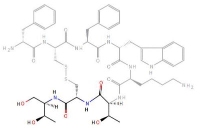	132.0812	132.0837	19.34
MATCH	200.0	120.0813	120.0808	-4.56	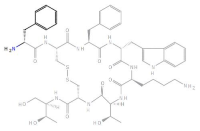	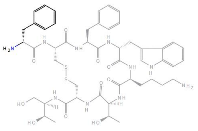	120.0813	120.0808	-4.56
MISMATCH	-5.1	116.0711	116.0706	-4.29	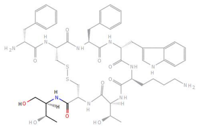	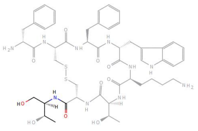	116.0711	116.0706	-4.29
MISMATCH	-5.1	116.0711	116.0706	-4.29	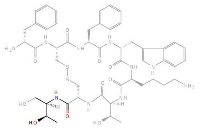	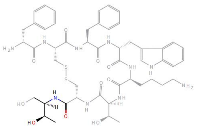	116.0711	116.0706	-4.29
MISMATCH	-32.3	106.0870	106.0863	-6.72	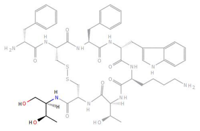	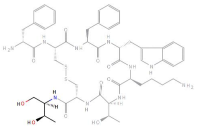	106.0870	106.0863	-6.72
MATCH	11.1	103.0550	103.0542	-7.39	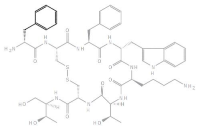	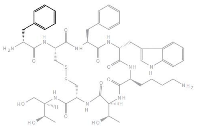	103.0550	103.0542	-7.39

Metabolite: Substrate

Type	score	sub. m/z observed	sub. m/z calculated	sub ppm		met. m/z observed	met. m/z calculated	met. ppm
MISMATCH	-19.7	88.0765	88.0757	-9.37	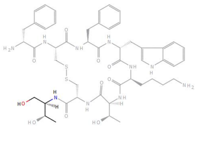 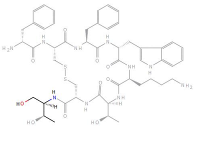	88.0765	88.0757	-9.37
MISMATCH	-19.7	88.0765	88.0757	-9.37	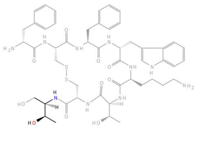 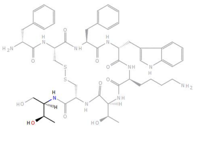	88.0765	88.0757	-9.37
MISMATCH	-15.8	84.0817	84.0808	-10.6	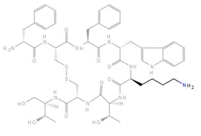 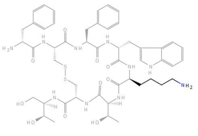	84.0817	84.0808	-10.6

MS (+) FT

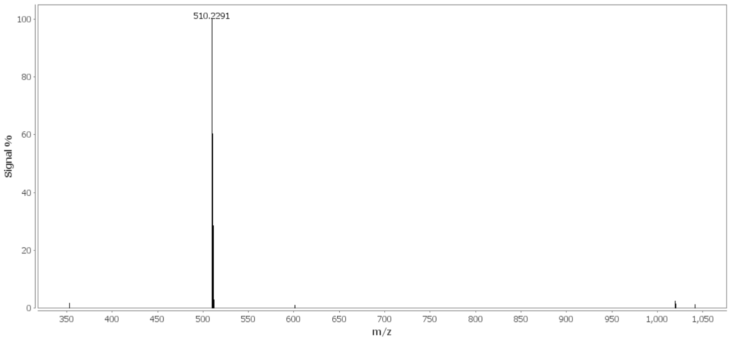

MS (+) FT

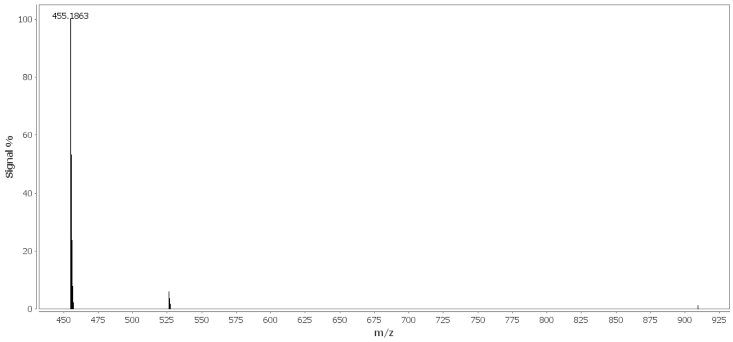

MS2 (+) FT activ = HCD:ce =

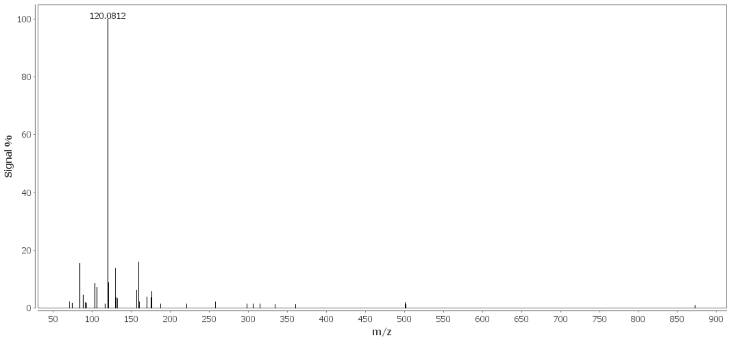

MS2 (+) FT activ = HCD:ce =

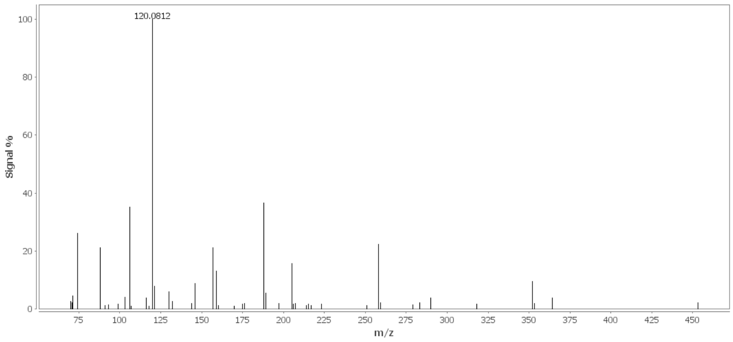

Metabolite: M2 -110 RT=2.23

Type	score	sub. m/z observed	sub. m/z calculated	sub ppm		met. m/z observed	met. m/z calculated	met. ppm
MATCH	200.0	510.2289	510.2275	-2.67	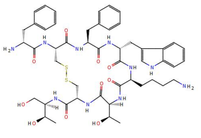 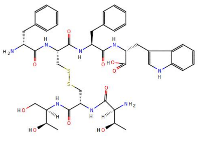	455.1863	455.1853	-2.07

Metabolite: M2 -110 RT=2.23

Type	score	sub. m/z observed	sub. m/z calculated	sub ppm		met. m/z observed	met. m/z calculated	met. ppm
MATCH	200.0	510.2289	510.2275	-2.67	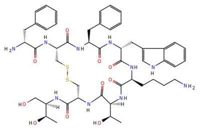 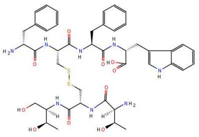	455.1863	455.1853	-2.07
MATCH	101.3	510.2289	510.2275	-2.67	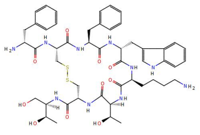 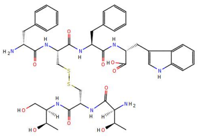	909.3651	909.3634	-1.93
MATCH	101.3	510.2289	510.2275	-2.67	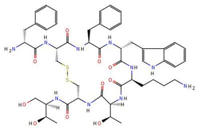 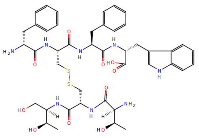	909.3651	909.3634	-1.93
MATCH	101.7	1019.4500	1019.4478	-2.21	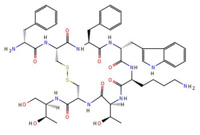 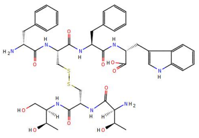	455.1863	455.1853	-2.07
MATCH	101.7	1019.4500	1019.4478	-2.21	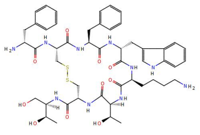 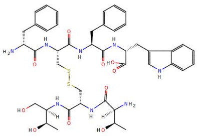	455.1863	455.1853	-2.07
MATCH	3.0	1019.4500	1019.4478	-2.21	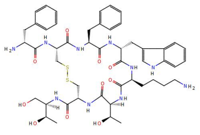 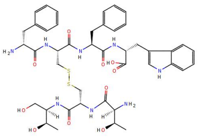	909.3651	909.3634	-1.93
MATCH	3.0	1019.4500	1019.4478	-2.21	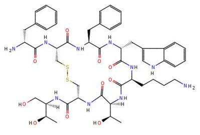 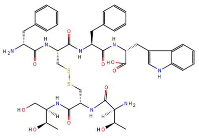	909.3651	909.3634	-1.93
MATCH	11.1	103.0550	103.0542	-7.39	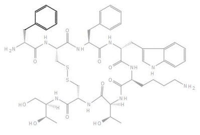 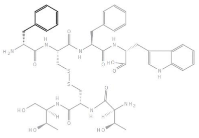	103.0549	103.0542	-6.11
MATCH	200.0	120.0813	120.0808	-4.56	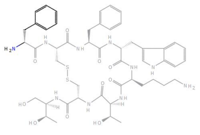 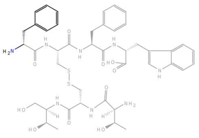	120.0812	120.0808	-3.65

Metabolite: M2 -110 RT=2.23

Type	score	sub. m/z observed	sub. m/z calculated	sub ppm		met. m/z observed	met. m/z calculated	met. ppm
MISMATCH	-25.4	88.0765	88.0757	-9.37	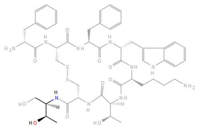	88.0764	88.0764	0.00
MISMATCH	-39.5	88.0765	88.0757	-9.37	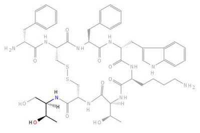	106.0869	106.0869	0.00
MISMATCH	-42.6	106.0870	106.0863	-6.72	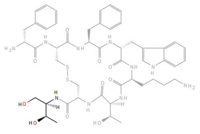	106.0869	106.0869	0.00
MISMATCH	-5.3	116.0711	116.0706	-4.29	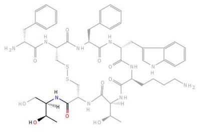	116.0711	116.0711	0.00
MISMATCH	-9.4	130.0655	130.0681	19.87	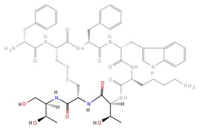	130.0655	130.0655	0.00
MISMATCH	-5.9	132.0812	132.0837	19.34	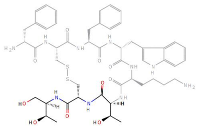	132.0810	132.0810	0.00
MISMATCH	-29.9	159.0921	159.0917	-2.38	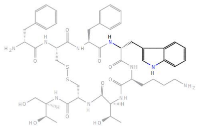	159.0919	159.0919	0.00
MISMATCH	-24.7	258.1453	258.1448	-1.75	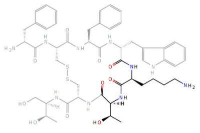	258.1451	258.1451	0.00
MISMATCH	-5.5	315.1819	315.1816	-1.10	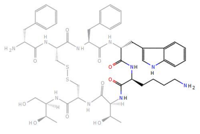	103.0549	103.0549	0.00

Metabolite: M2 -110 RT=2.23

Type	score	sub. m/z observed	sub. m/z calculated	sub ppm		met. m/z observed	met. m/z calculated	met. ppm
MISMATCH	-17.2	315.1819	315.1816	-1.10	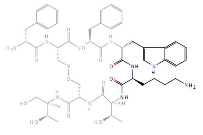	205.0975	205.0975	0.00
MET_MATCH					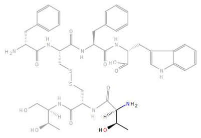	74.0609	74.0600	-11.9
MET_MATCH					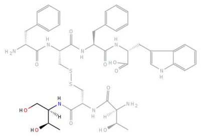	106.0869	106.0863	-5.88
MET_MATCH					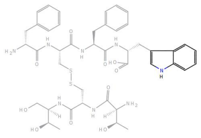	118.0655	118.0651	-3.51
MET_MATCH					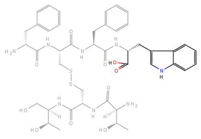	188.0709	188.0706	-1.38
MET_MATCH					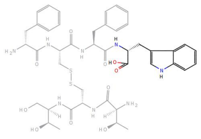	205.0975	205.0972	-1.60
MET_MATCH					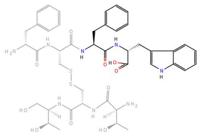	352.1659	352.1656	-1.03

MS (+) FT

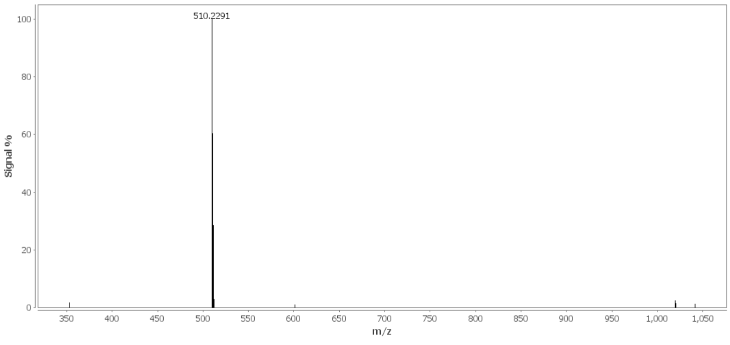

MS (+) FT

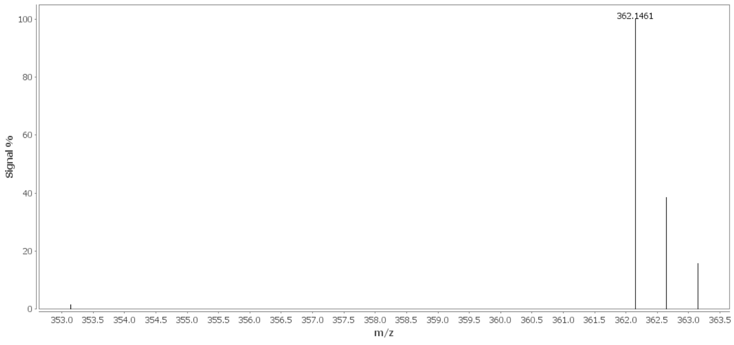

MS2 (+) FT activ = HCD:ce =

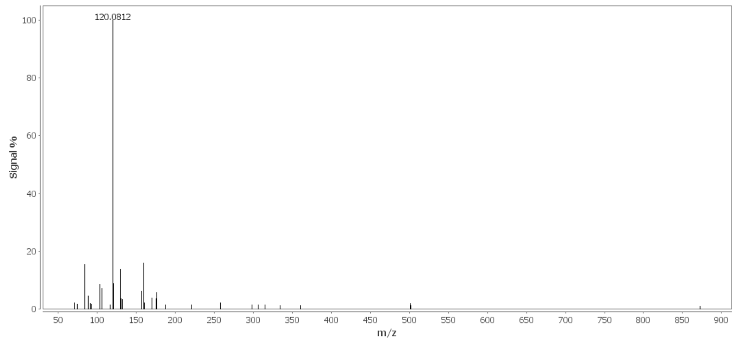

MS2 (+) FT activ = HCD:ce =

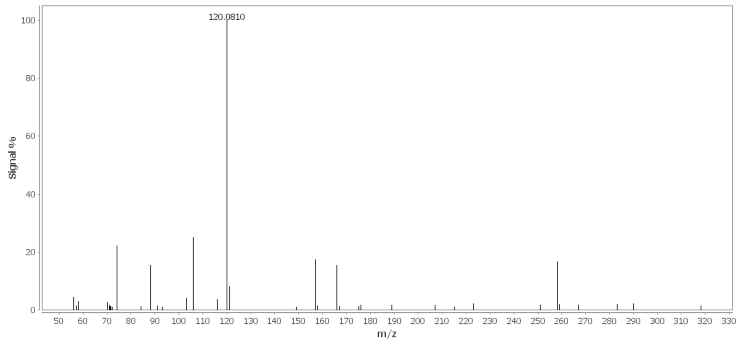

Metabolite: M1 -296 RT=1.66

Type	score	sub. m/z observed	sub. m/z calculated	sub ppm			met. m/z observed	met. m/z calculated	met. ppm
MATCH	200.0	510.2289	510.2275	-2.67	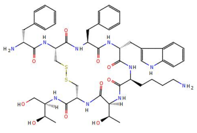	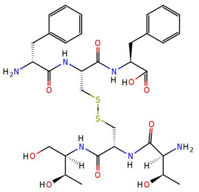	362.1461	362.1457	-1.25
						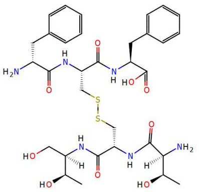	362.1461	362.1457	-1.25
MATCH	101.7	1019.4500	1019.4478	-2.21	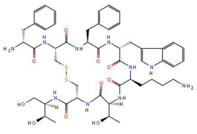	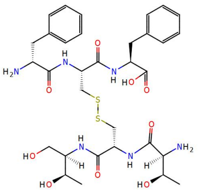	362.1461	362.1457	-1.25
						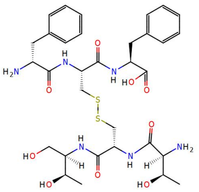	362.1461	362.1457	-1.25
MATCH	11.1	103.0550	103.0542	-7.39	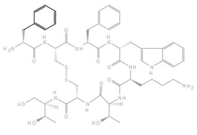	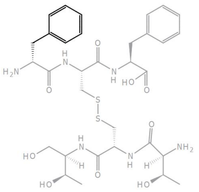	103.0547	103.0542	-4.58
MATCH	200.0	120.0813	120.0808	-4.56	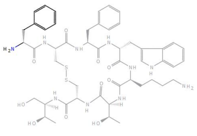	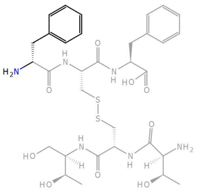	120.0810	120.0808	-1.72
MISMATCH	-101.5	510.2289	510.2275	-2.67	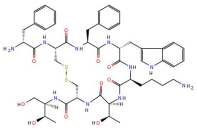		353.1412	353.1412	0.00

Metabolite: M1 -296 RT=1.66

Type	score	sub. m/z observed	sub. m/z calculated	sub ppm		met. m/z observed	met. m/z calculated	met. ppm
MISMATCH	-3.2	1019.4500	1019.4478	-2.21	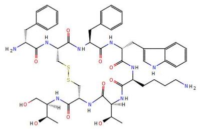	353.1412	353.1412	0.00
MISMATCH	-15.8	84.0817	84.0808	-10.6	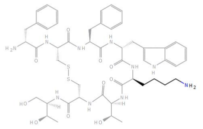	84.0814	84.0814	0.00
MISMATCH	-19.7	88.0765	88.0757	-9.37	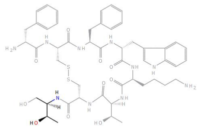	88.0763	88.0763	0.00
MISMATCH	-29.2	88.0765	88.0757	-9.37	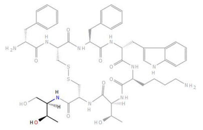	106.0867	106.0867	0.00
MISMATCH	-32.3	106.0870	106.0863	-6.72	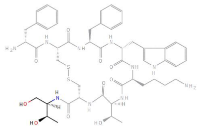	106.0867	106.0867	0.00
MISMATCH	-5.1	116.0711	116.0706	-4.29	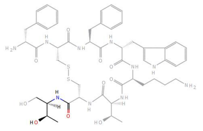	116.0709	116.0709	0.00
MISMATCH	-5.4	187.0870	187.0866	-1.99	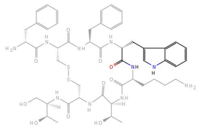	103.0547	103.0547	0.00
MET_MATCH					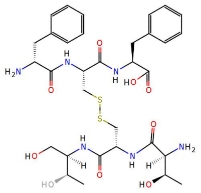	353.1412	353.1404	-2.22
MET_MATCH					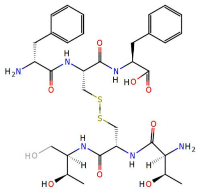	353.1412	353.1404	-2.22

Metabolite: M1 -296 RT=1.66

Type	score	sub. m/z observed	sub. m/z calculated	sub ppm		met. m/z observed	met. m/z calculated	met. ppm
MET_MATCH					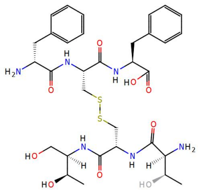	353.1412	353.1404	-2.22
MET_MATCH					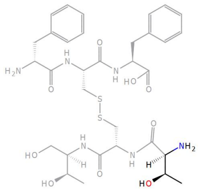	74.0608	74.0600	-10.1
MET_MATCH					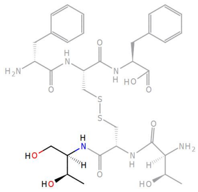	106.0867	106.0863	-4.01
MET_MATCH					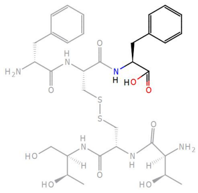	166.0862	166.0863	0.29
