# Supplementary material for: Software-aided approach to investigate peptide structure and metabolic susceptibility of amide bonds in peptide drugs based on high resolution mass spectrometry
Source: PLoS One. 2017 Nov 1;12(11):e0186461. doi: 10.1371/journal.pone.0186461 (PMC5665424; doi:10.1371/journal.pone.0186461)
Supplement: S1 File — (ZIP) [file pone.0186461.s007.zip › SFiles/S39_File.pdf]

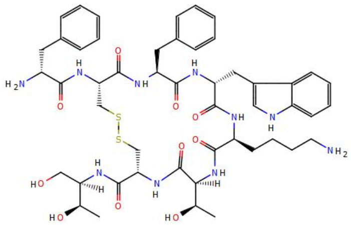

Object 1

Property name	Property value
Time	0min, 5min, 15min, 45min, 120min
Instrument	ThermoQAPlus
Matrix	trypsin
Acquisition Mode	ddMS2

Chromatograms

Time=0min

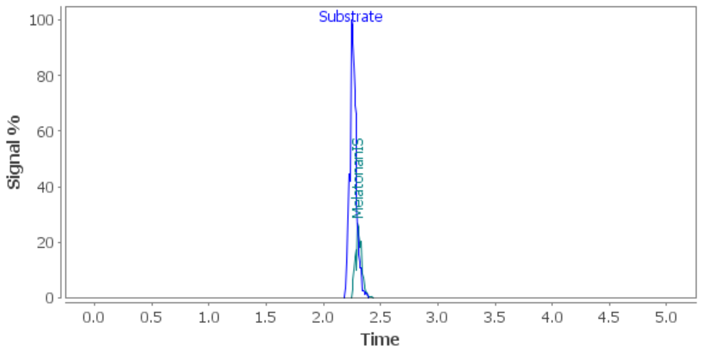

Time=5min

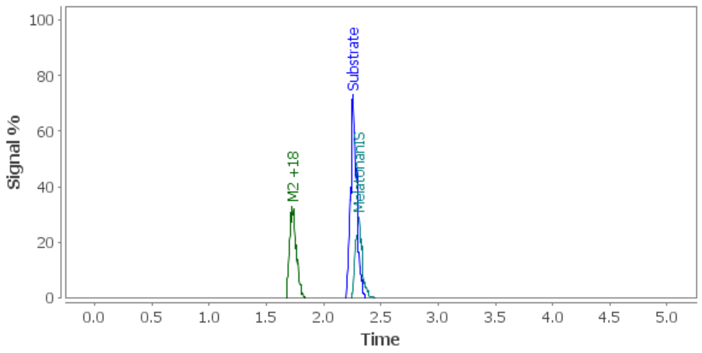

Time=15min

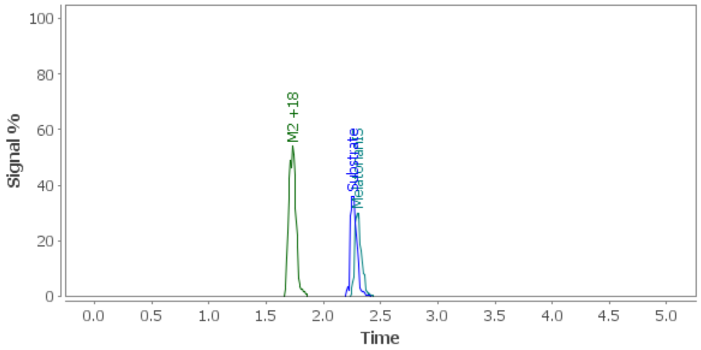

Time=45min

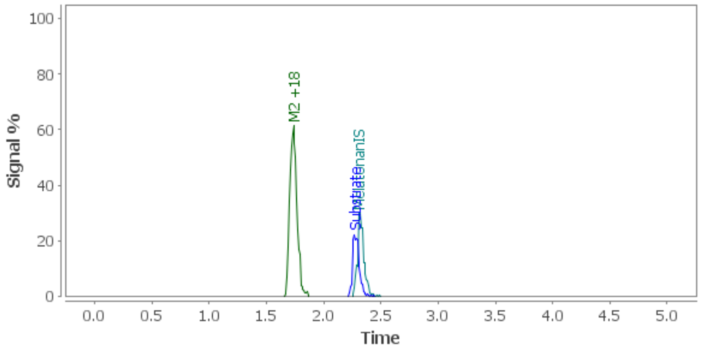

Time=120min

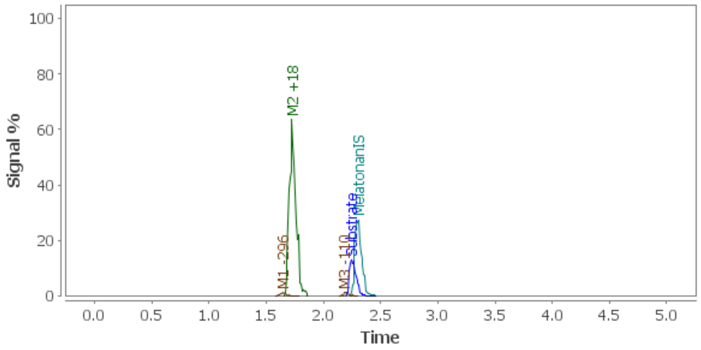

Custom Charts

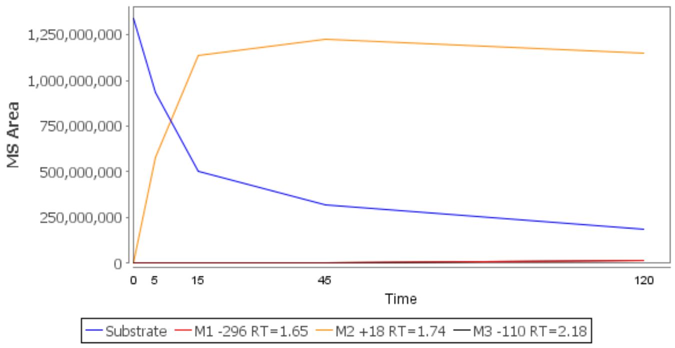

Fragmentation

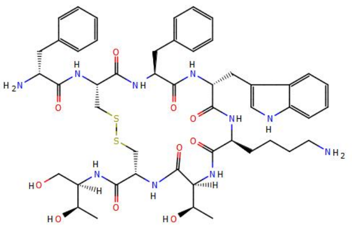

Object 1

MS (+) FT

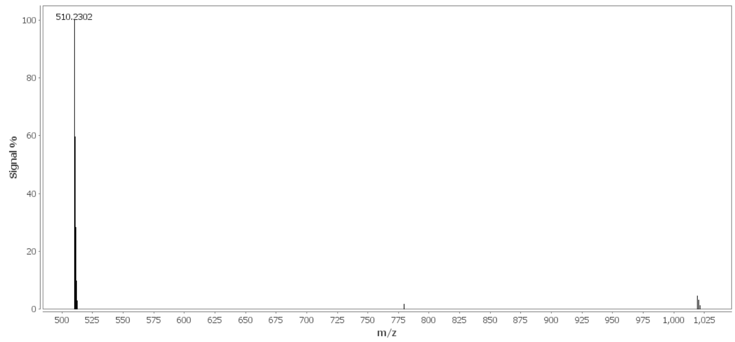

MS (+) FT

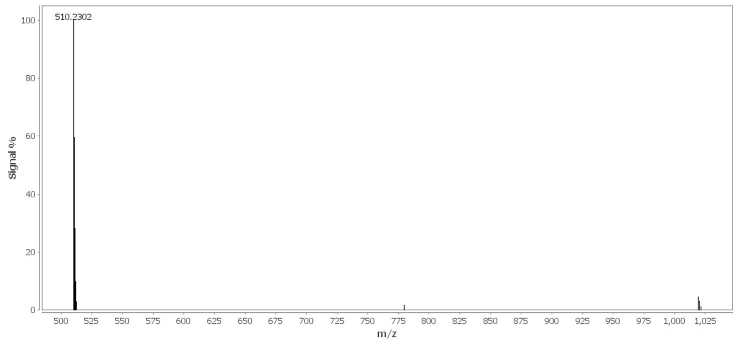

MS2 (+) FT activ = HCD:ce =

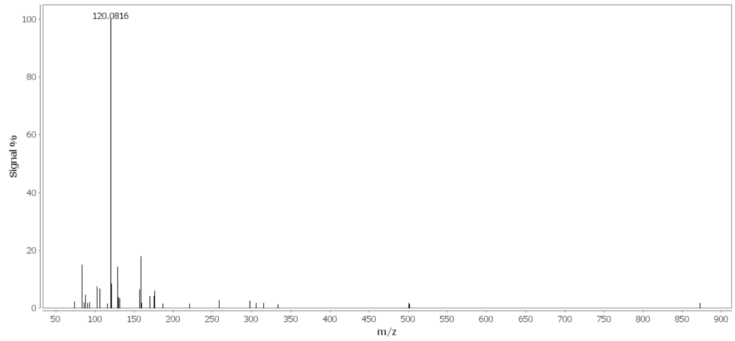

MS2 (+) FT activ = HCD:ce =

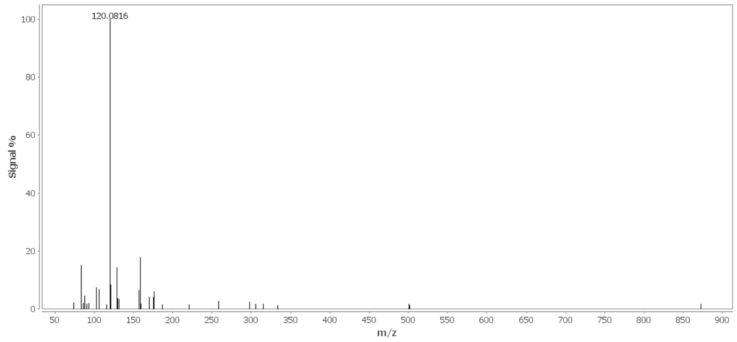

Metabolite: Substrate

Type	score	sub. m/z observed	sub. m/z calculated	sub ppm		met. m/z observed	met. m/z calculated	met. ppm
MATCH	104.5	1019.4524	1019.4478	-4.59	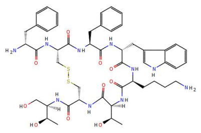 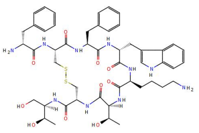	1019.4524	1019.4478	-4.59
MATCH	200.0	510.2302	510.2275	-5.29	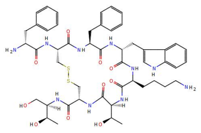 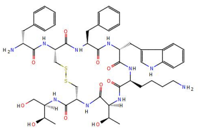	510.2302	510.2275	-5.29
MISMATCH	-2.5	334.1564	334.1550	-4.10	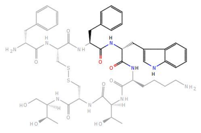 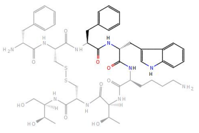	334.1564	334.1550	-4.10

Metabolite: Substrate

Type	score	sub. m/z observed	sub. m/z calculated	sub ppm			met. m/z observed	met. m/z calculated	met. ppm
MISMATCH	-2.5	334.1564	334.1550	-4.10	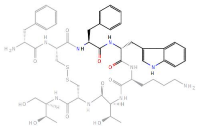	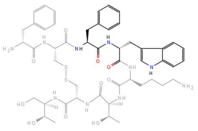	334.1564	334.1550	-4.10
MISMATCH	-6.0	315.1835	315.1816	-6.24	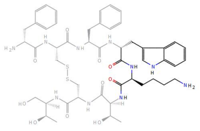	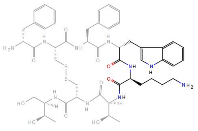	315.1835	315.1816	-6.24
MISMATCH	-4.6	306.1615	306.1601	-4.67	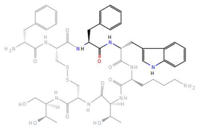	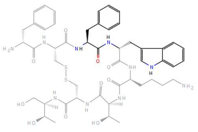	306.1615	306.1601	-4.67
MISMATCH	3.7	298.1562	298.1550	-3.90	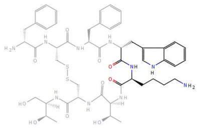	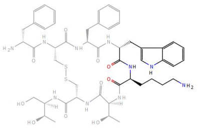	298.1562	298.1550	-3.90
MISMATCH	28.4	258.1459	258.1448	-4.26	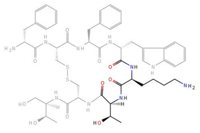	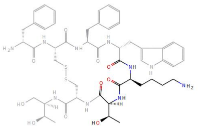	258.1459	258.1448	-4.26
MISMATCH	-2.6	221.0750	221.0743	-3.15	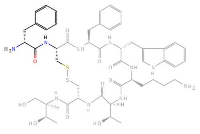	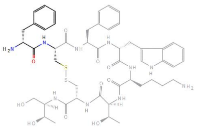	221.0750	221.0743	-3.15
MISMATCH	-2.7	187.0878	187.0866	-6.70	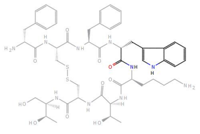	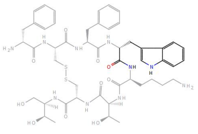	187.0878	187.0866	-6.70
MISMATCH	-10.9	175.0874	175.0866	-4.62	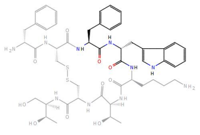	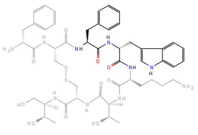	175.0874	175.0866	-4.62
MISMATCH	-5.8	170.0609	170.0600	-5.22	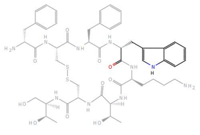	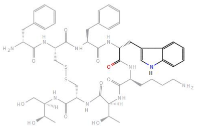	170.0609	170.0600	-5.22

Metabolite: Substrate

Type	score	sub. m/z observed	sub. m/z calculated	sub ppm			met. m/z observed	met. m/z calculated	met. ppm
MISMATCH	-29.5	159.0924	159.0917	-4.75	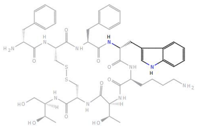	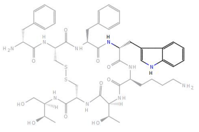	159.0924	159.0917	-4.75
MATCH	39.2	157.0979	157.0972	-4.76	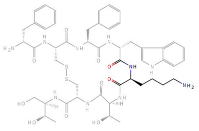	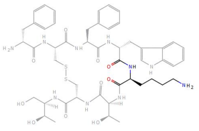	157.0979	157.0972	-4.76
MATCH	9.3	130.0659	130.0681	16.59	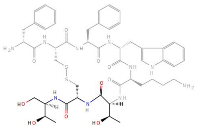	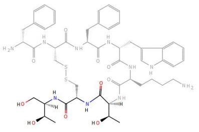	130.0659	130.0681	16.59
MISMATCH	20.6	129.1030	129.1022	-5.87	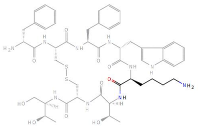	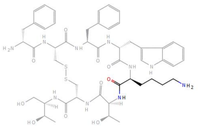	129.1030	129.1022	-5.87
MISMATCH	20.6	129.1030	129.1022	-5.87	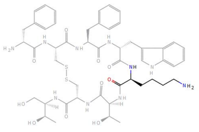	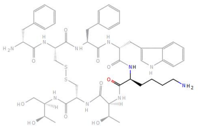	129.1030	129.1022	-5.87
MISMATCH	-200.0	120.0816	120.0808	-6.76	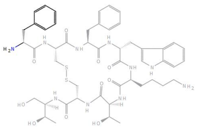	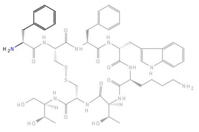	120.0816	120.0808	-6.76
MISMATCH	4.3	116.0713	116.0706	-5.71	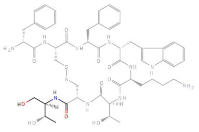	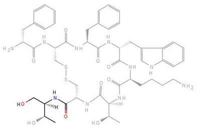	116.0713	116.0706	-5.71
MISMATCH	4.3	116.0713	116.0706	-5.71	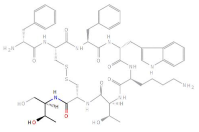	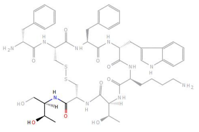	116.0713	116.0706	-5.71
MISMATCH	29.0	106.0872	106.0863	-8.80	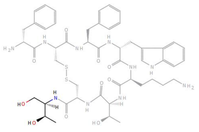	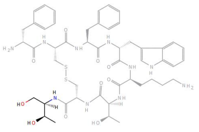	106.0872	106.0863	-8.80

Metabolite: Substrate

Type	score	sub. m/z observed	sub. m/z calculated	sub ppm		met. m/z observed	met. m/z calculated	met. ppm
MISMATCH	-11.9	103.0552	103.0542	-9.58	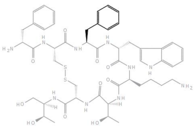 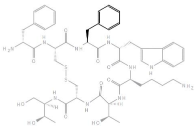	103.0552	103.0542	-9.58
MISMATCH	-11.9	103.0552	103.0542	-9.58	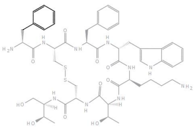 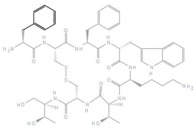	103.0552	103.0542	-9.58
MISMATCH	20.8	88.0767	88.0757	-11.3	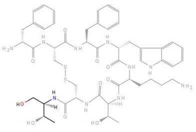 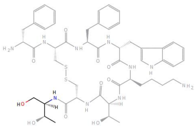	88.0767	88.0757	-11.3
MISMATCH	20.8	88.0767	88.0757	-11.3	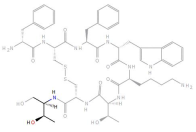 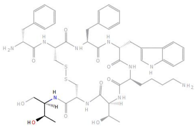	88.0767	88.0757	-11.3
MISMATCH	38.6	86.0975	86.0964	-12.4	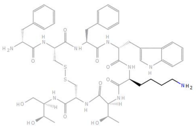 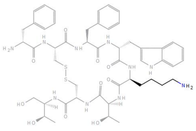	86.0975	86.0964	-12.4
MISMATCH	-37.2	74.0611	74.0600	-14.8	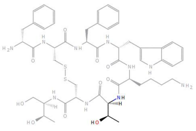 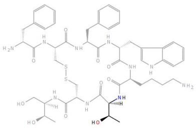	74.0611	74.0600	-14.8

MS (+) FT

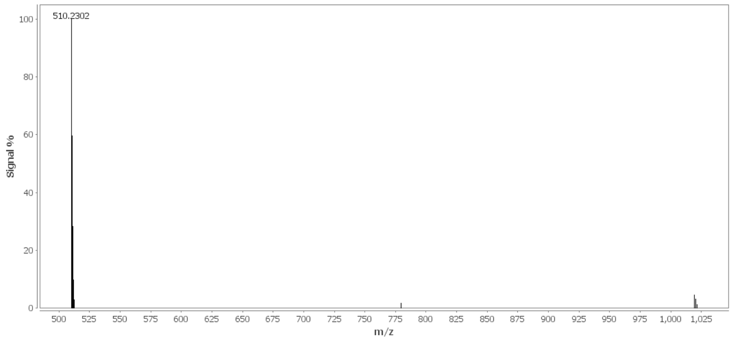

MS (+) FT

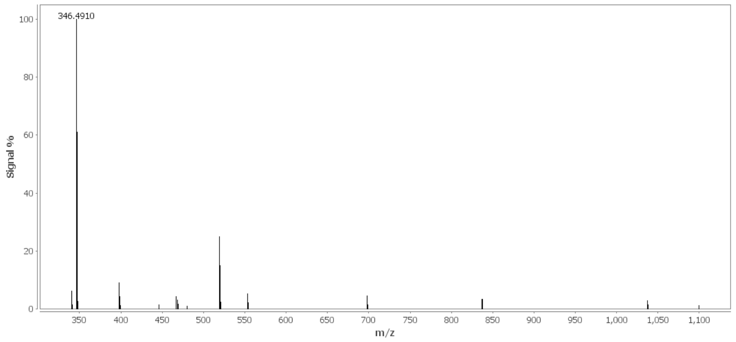

MS2 (+) FT activ = HCD:ce =

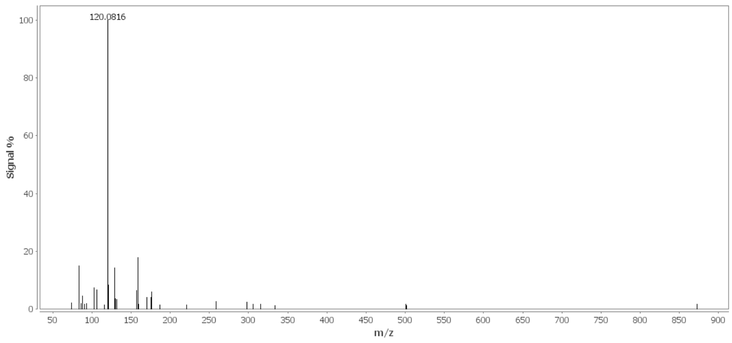

MS2 (+) FT activ = HCD:ce =

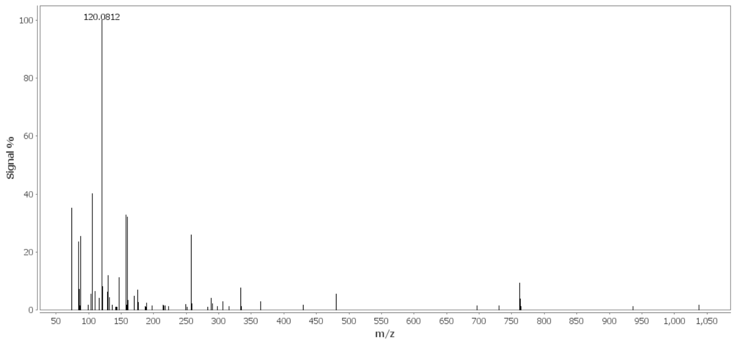

Metabolite: M2 +18 RT=1.74

Type	score	sub. m/z observed	sub. m/z calculated	sub ppm			met. m/z observed	met. m/z calculated	met. ppm
MATCH	200.0	510.2302	510.2275	-5.29	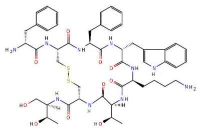	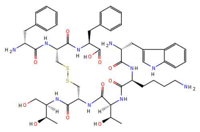	346.4910	346.4910	-0.17
MATCH	200.0	510.2302	510.2275	-5.29	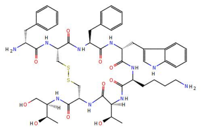	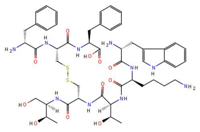	346.4910	346.4910	-0.17
MATCH	124.9	510.2302	510.2275	-5.29	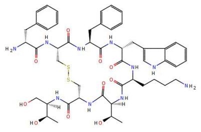	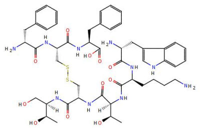	519.2332	519.2328	-0.73
MATCH	124.9	510.2302	510.2275	-5.29	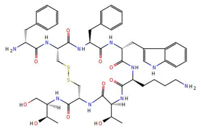	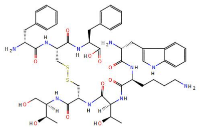	519.2332	519.2328	-0.73
MATCH	102.8	510.2302	510.2275	-5.29	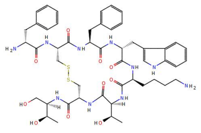	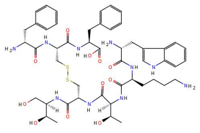	1037.4595	1037.4583	-1.09
MATCH	102.8	510.2302	510.2275	-5.29	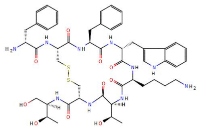	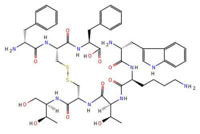	1037.4595	1037.4583	-1.09
MATCH	104.5	1019.4524	1019.4478	-4.59	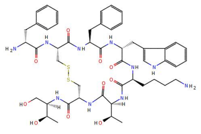	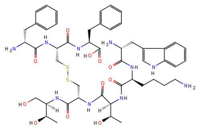	346.4910	346.4910	-0.17

Metabolite: M2 +18 RT=1.74

Type	score	sub. m/z observed	sub. m/z calculated	sub ppm		met. m/z observed	met. m/z calculated	met. ppm
MATCH	104.5	1019.4524	1019.4478	-4.59	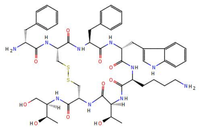 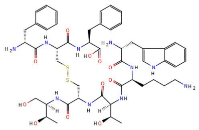	346.4910	346.4910	-0.17
MATCH	29.4	1019.4524	1019.4478	-4.59	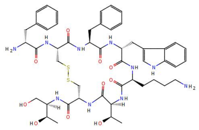 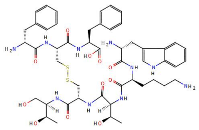	519.2332	519.2328	-0.73
MATCH	29.4	1019.4524	1019.4478	-4.59	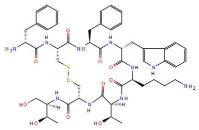 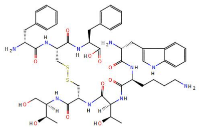	519.2332	519.2328	-0.73
MATCH	7.4	1019.4524	1019.4478	-4.59	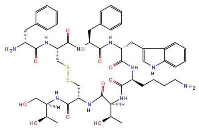 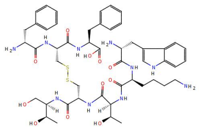	1037.4595	1037.4583	-1.09
MATCH	7.4	1019.4524	1019.4478	-4.59	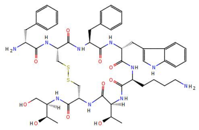 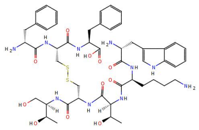	1037.4595	1037.4583	-1.09
MATCH	38.6	84.0819	84.0808	-12.9	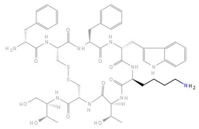 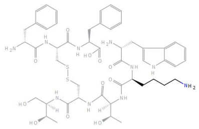	84.0815	84.0808	-9.02
MATCH	9.0	86.0975	86.0964	-12.4	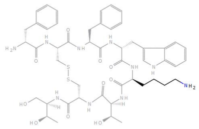 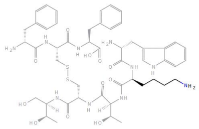	86.0971	86.0964	-7.39
MATCH	20.6	129.1030	129.1022	-5.87	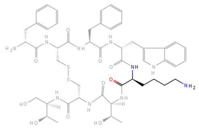 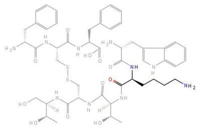	129.1023	129.1022	-0.74
MATCH	20.6	129.1030	129.1022	-5.87	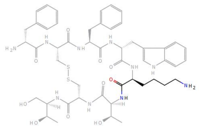 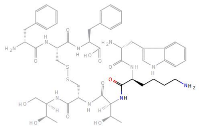	129.1023	129.1022	-0.74

Metabolite: M2 +18 RT=1.74

Type	score	sub. m/z observed	sub. m/z calculated	sub ppm		met. m/z observed	met. m/z calculated	met. ppm
MATCH	39.2	157.0979	157.0972	-4.76	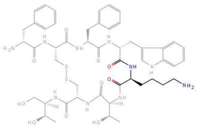 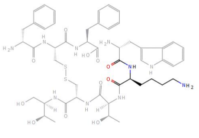	157.0973	157.0972	-0.74
MATCH	28.4	258.1459	258.1448	-4.26	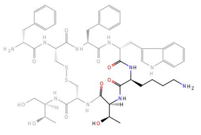 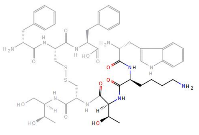	258.1451	258.1448	-0.92
MATCH	3.7	298.1562	298.1550	-3.90	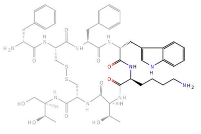 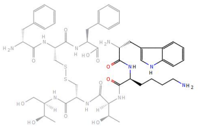	298.1553	298.1550	-1.04
MISMATCH	-106.1	510.2302	510.2275	-5.29	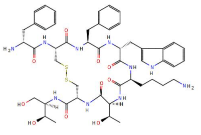	340.4873	340.4873	0.00
MISMATCH	-10.7	1019.4524	1019.4478	-4.59	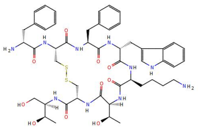	340.4873	340.4873	0.00
MISMATCH	-37.2	74.0611	74.0600	-14.8	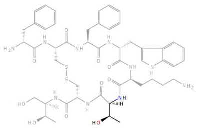	74.0609	74.0609	0.00
MISMATCH	-30.1	88.0767	88.0757	-11.3	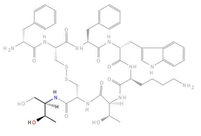	88.0764	88.0764	0.00
MISMATCH	-44.7	88.0767	88.0757	-11.3	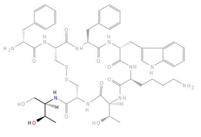	106.0868	106.0868	0.00
MISMATCH	-12.8	103.0552	103.0542	-9.58	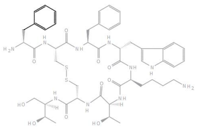	103.0549	103.0549	0.00

Metabolite: M2 +18 RT=1.74

Type	score	sub. m/z observed	sub. m/z calculated	sub ppm		met. m/z observed	met. m/z calculated	met. ppm
MISMATCH	-46.7	106.0872	106.0863	-8.80	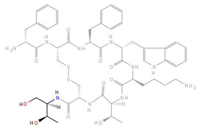	106.0868	106.0868	0.00
MISMATCH	-5.5	116.0713	116.0706	-5.71	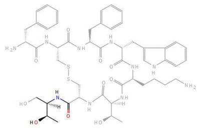	116.0709	116.0709	0.00
MISMATCH	-200.0	120.0816	120.0808	-6.76	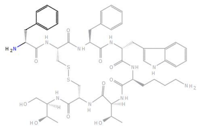	120.0812	120.0812	0.00
MISMATCH	-49.4	129.1030	129.1022	-5.87	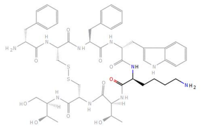	74.0609	74.0609	0.00
MISMATCH	-25.4	129.1030	129.1022	-5.87	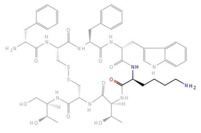	147.1129	147.1129	0.00
MISMATCH	-5.2	130.0659	130.0681	16.59	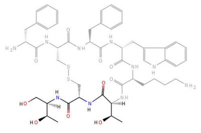	87.0558	87.0558	0.00
MISMATCH	-9.0	130.0659	130.0681	16.59	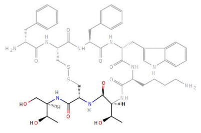	130.0655	130.0655	0.00
MISMATCH	-7.7	132.0815	132.0837	17.03	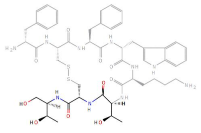	132.0810	132.0810	0.00
MISMATCH	-8.9	170.0609	170.0600	-5.22	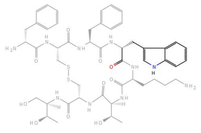	170.0601	170.0601	0.00

Metabolite: M2 +18 RT=1.74

Type	score	sub. m/z observed	sub. m/z calculated	sub ppm		met. m/z observed	met. m/z calculated	met. ppm
MISMATCH	-10.9	175.0874	175.0866	-4.62	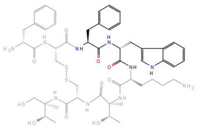	175.0867	175.0867	0.00
MISMATCH	-6.9	187.0878	187.0866	-6.70	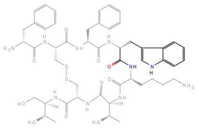	103.0549	103.0549	0.00
MISMATCH	-2.7	187.0878	187.0866	-6.70	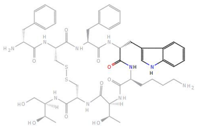	187.0870	187.0870	0.00
MISMATCH	-3.7	298.1562	298.1550	-3.90	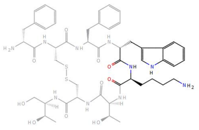	316.1660	316.1660	0.00
MISMATCH	-4.6	306.1615	306.1601	-4.67	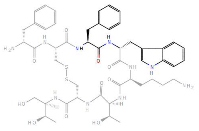	306.1601	306.1601	0.00
MISMATCH	-9.2	315.1835	315.1816	-6.24	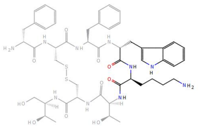	333.1918	333.1918	0.00
MISMATCH	-2.5	334.1564	334.1550	-4.10	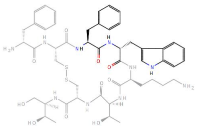	334.1549	334.1549	0.00
MET_MATCH					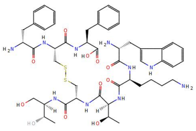	340.4873	340.4874	0.37
MET_MATCH					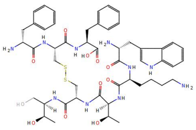	340.4873	340.4874	0.37

Metabolite: M2 +18 RT=1.74

Type	score	sub. m/z observed	sub. m/z calculated	sub ppm		met. m/z observed	met. m/z calculated	met. ppm
MET_MATCH					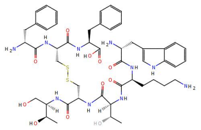	340.4873	340.4874	0.37
MET_MATCH					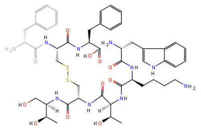	445.6985	445.6986	0.16
MET_MATCH					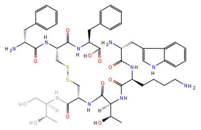	466.6934	466.6933	-0.10
MET_MATCH					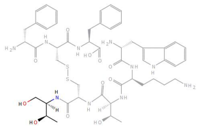	106.0868	106.0863	-4.80
MET_MATCH					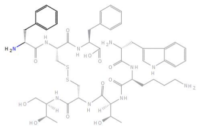	120.0812	120.0808	-3.42
MET_MATCH					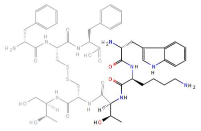	130.0865	130.0830	-27.1
MET_MATCH					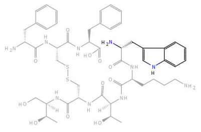	159.0919	159.0917	-1.23
MET_MATCH					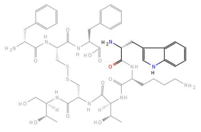	187.0870	187.0866	-2.13
MET_MATCH					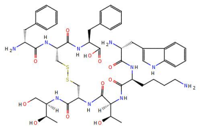	1037.4575	1037.4583	0.76

MS (+) FT

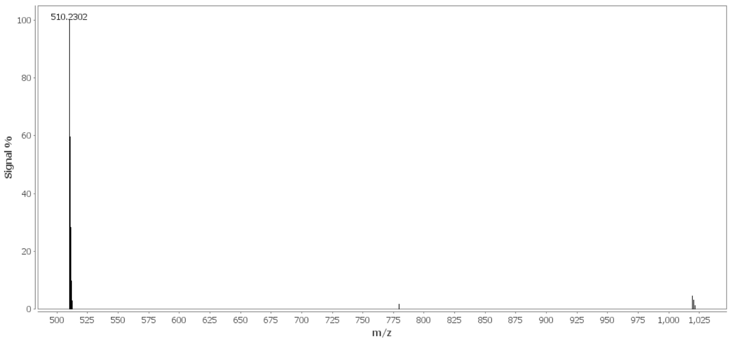

MS (+) FT

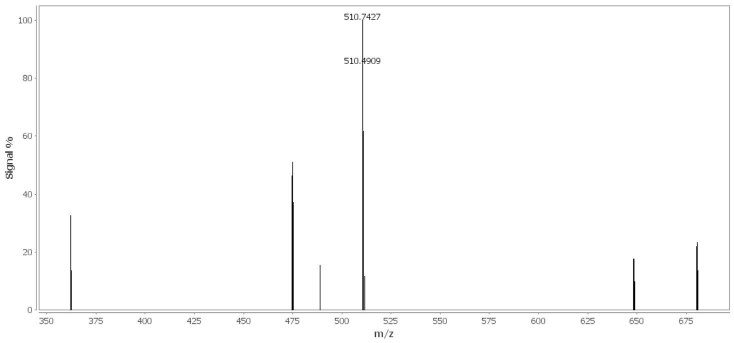

MS2 (+) FT activ = HCD:ce =

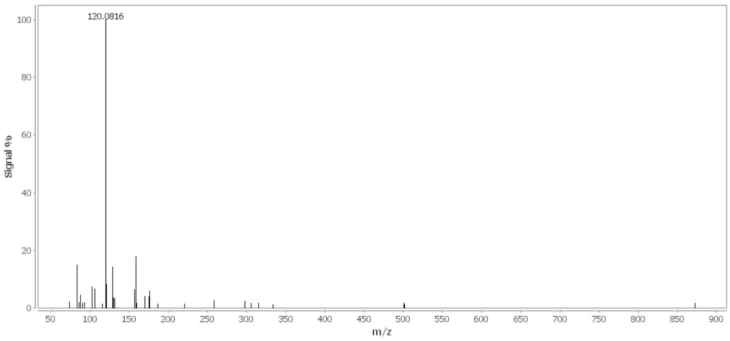

MS2 (+) FT activ = HCD:ce =

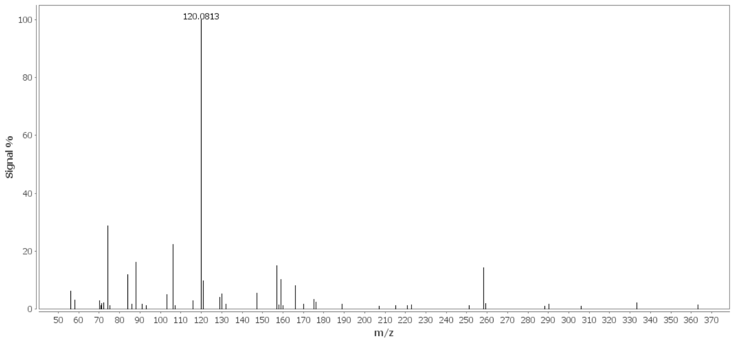

Metabolite: M1 -296 RT=1.65

Type	score	sub. m/z observed	sub. m/z calculated	sub ppm			met. m/z observed	met. m/z calculated	met. ppm
MATCH	132.5	510.2302	510.2275	-5.29	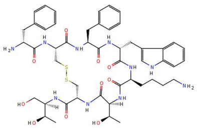	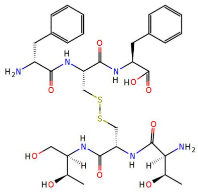	362.1466	362.1457	-2.72
						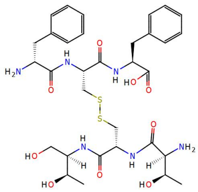	362.1466	362.1457	-2.72
MATCH	37.0	1019.4524	1019.4478	-4.59	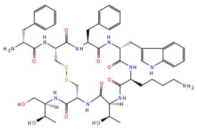	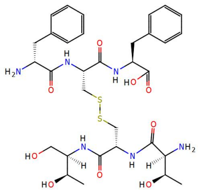	362.1466	362.1457	-2.72
						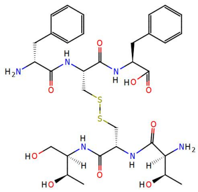	362.1466	362.1457	-2.72
MATCH	20.8	88.0767	88.0757	-11.3	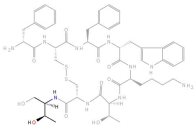	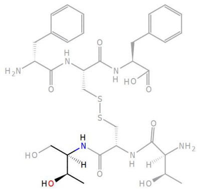	88.0765	88.0757	-9.34

Metabolite: M1 -296 RT=1.65

Type	score	sub. m/z observed	sub. m/z calculated	sub ppm			met. m/z observed	met. m/z calculated	met. ppm
MATCH	20.8	88.0767	88.0757	-11.3	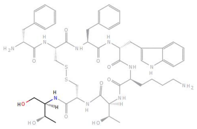	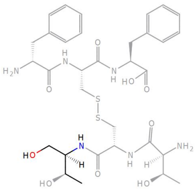	88.0765	88.0757	-9.34
MATCH	29.0	106.0872	106.0863	-8.80	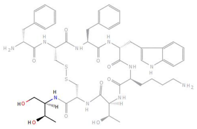	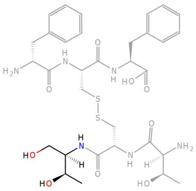	106.0869	106.0863	-6.50
MATCH	4.3	116.0713	116.0706	-5.71	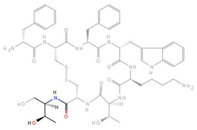	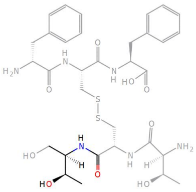	116.0711	116.0706	-4.31
MATCH	4.3	116.0713	116.0706	-5.71	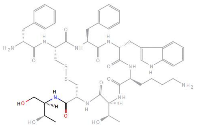	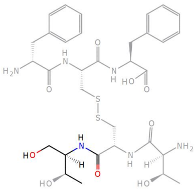	116.0711	116.0706	-4.31
MATCH	6.1	130.0659	130.0681	16.59	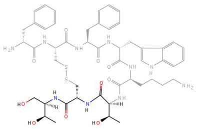	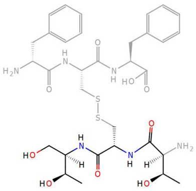	130.0655	130.0681	19.65
MISMATCH	-26.8	84.0819	84.0808	-12.9	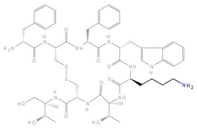		84.0816	84.0816	0.00
MISMATCH	-3.6	86.0975	86.0964	-12.4	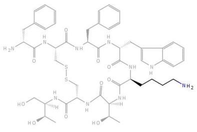		86.0973	86.0973	0.00
MISMATCH	-12.4	103.0552	103.0542	-9.58	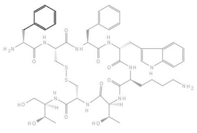		103.0550	103.0550	0.00
MISMATCH	-200.0	120.0816	120.0808	-6.76	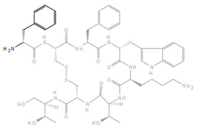		120.0813	120.0813	0.00

Metabolite: M1 -296 RT=1.65

Type	score	sub. m/z observed	sub. m/z calculated	sub ppm		met. m/z observed	met. m/z calculated	met. ppm
MISMATCH	-5.8	170.0609	170.0600	-5.22	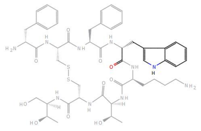	170.0604	170.0604	0.00
MISMATCH	-6.5	187.0878	187.0866	-6.70	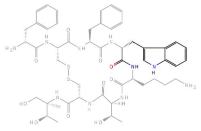	103.0550	103.0550	0.00
MISMATCH	-2.6	221.0750	221.0743	-3.15	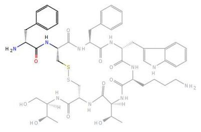	221.0753	221.0753	0.00
MET_MATCH					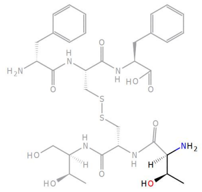	74.0610	74.0600	-12.6
MET_MATCH					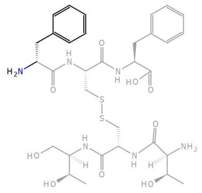	120.0813	120.0808	-4.16
MET_MATCH					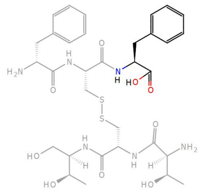	166.0866	166.0863	-2.34

MS (+) FT

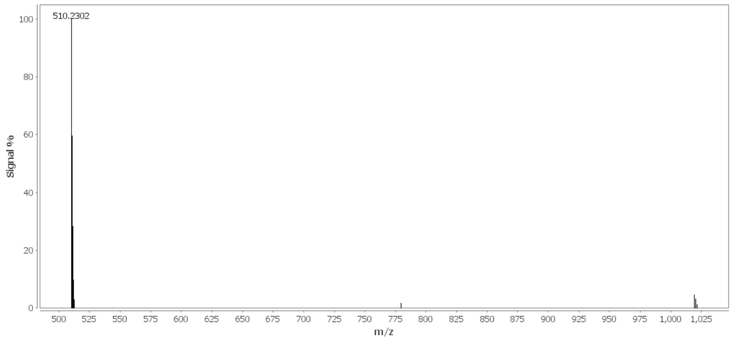

MS (+) FT

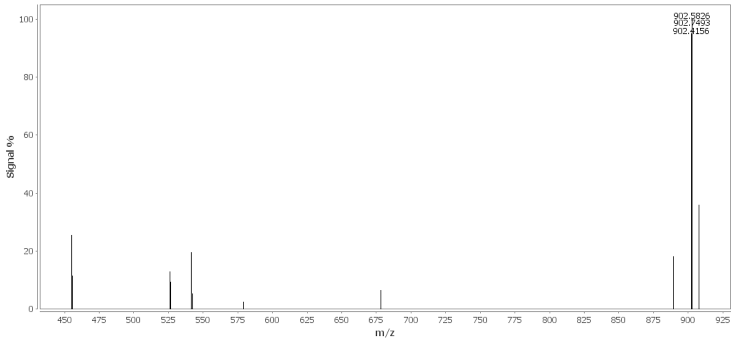

MS2 (+) FT activ = HCD:ce =

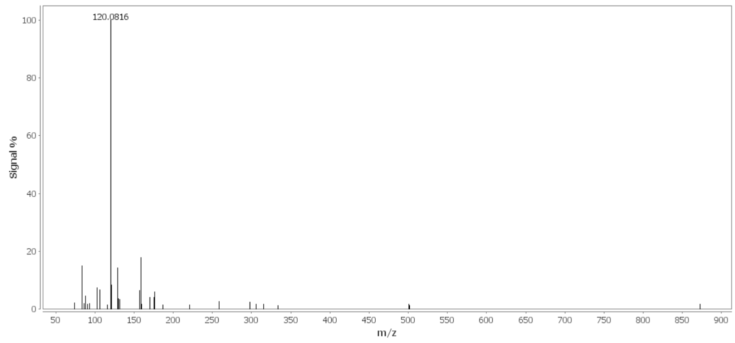

MS2 (+) FT activ = HCD:ce =

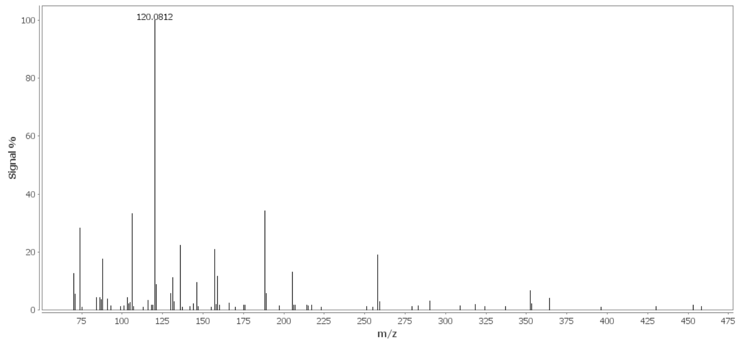

Metabolite: M3 -110 RT=2.18

Type	score	sub. m/z observed	sub. m/z calculated	sub ppm			met. m/z observed	met. m/z calculated	met. ppm
MATCH	125.5	510.2302	510.2275	-5.29	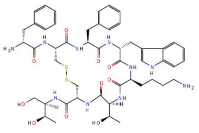	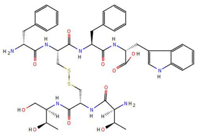	455.1867	455.1853	-3.04
						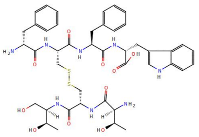	455.1867	455.1853	-3.04
MATCH	30.0	1019.4524	1019.4478	-4.59	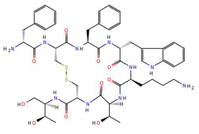	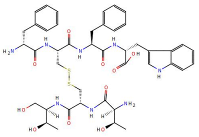	455.1867	455.1853	-3.04
						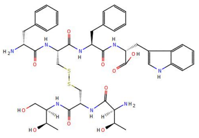	455.1867	455.1853	-3.04
MATCH	9.3	130.0659	130.0681	16.59	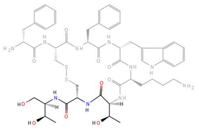	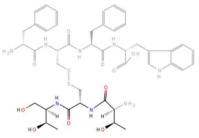	130.0654	130.0681	20.47
MISMATCH	-6.3	86.0975	86.0964	-12.4	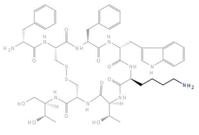		86.0973	86.0973	0.00
MISMATCH	-22.1	88.0767	88.0757	-11.3	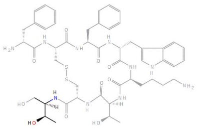		88.0764	88.0764	0.00

Metabolite: M3 -110 RT=2.18

Type	score	sub. m/z observed	sub. m/z calculated	sub ppm		met. m/z observed	met. m/z calculated	met. ppm
MISMATCH	-37.7	88.0767	88.0757	-11.3	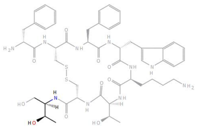	106.0869	106.0869	0.00
MISMATCH	-11.9	103.0552	103.0542	-9.58	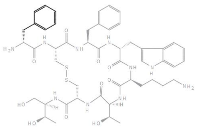	103.0548	103.0548	0.00
MISMATCH	-39.7	106.0872	106.0863	-8.80	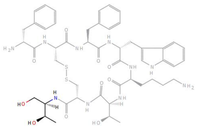	106.0869	106.0869	0.00
MISMATCH	-5.0	116.0713	116.0706	-5.71	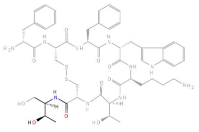	116.0710	116.0710	0.00
MISMATCH	-200.0	120.0816	120.0808	-6.76	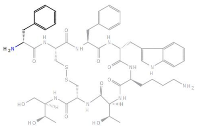	120.0812	120.0812	0.00
MISMATCH	-29.5	159.0924	159.0917	-4.75	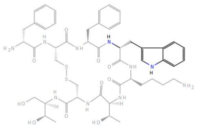	159.0917	159.0917	0.00
MISMATCH	-21.7	258.1459	258.1448	-4.26	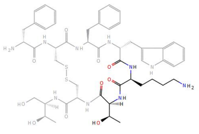	258.1452	258.1452	0.00
MISMATCH	-6.0	315.1835	315.1816	-6.24	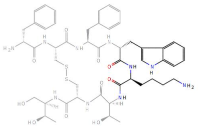	103.0548	103.0548	0.00
MISMATCH	-14.7	315.1835	315.1816	-6.24	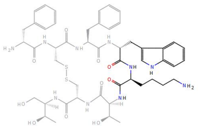	205.0975	205.0975	0.00

Metabolite: M3 -110 RT=2.18

Type	score	sub. m/z observed	sub. m/z calculated	sub ppm		met. m/z observed	met. m/z calculated	met. ppm
MET_MATCH					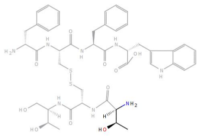	74.0609	74.0600	-11.9
MET_MATCH					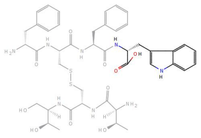	103.0548	103.0522	-24.8
MET_MATCH					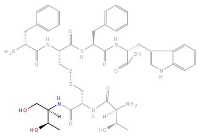	104.0628	104.0706	75.31
MET_MATCH					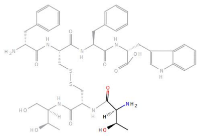	104.0628	104.0706	75.31
MET_MATCH					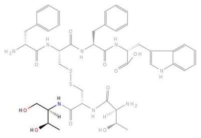	106.0869	106.0863	-6.00
MET_MATCH					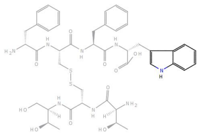	118.0656	118.0651	-4.00
MET_MATCH					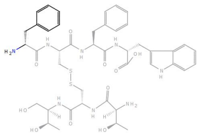	120.0812	120.0808	-3.46
MET_MATCH					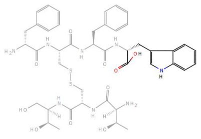	188.0708	188.0706	-1.12
MET_MATCH					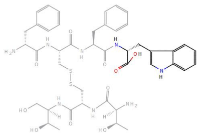	205.0975	205.0972	-1.56

Metabolite: M3 -110 RT=2.18

Type	score	sub. m/z observed	sub. m/z calculated	sub ppm		met. m/z observed	met. m/z calculated	met. ppm
MET_MATCH					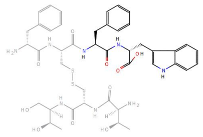	352.1661	352.1656	-1.53
MET_MATCH					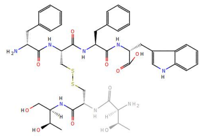	396.1395	396.1482	21.91
