# Supplementary material for: Software-aided approach to investigate peptide structure and metabolic susceptibility of amide bonds in peptide drugs based on high resolution mass spectrometry
Source: PLoS One. 2017 Nov 1;12(11):e0186461. doi: 10.1371/journal.pone.0186461 (PMC5665424; doi:10.1371/journal.pone.0186461)
Supplement: S1 File — (ZIP) [file pone.0186461.s007.zip › SFiles/S3_File.pdf]

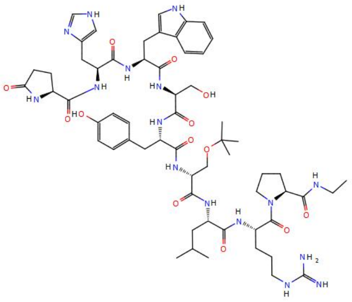

Buserelin

Property name	Property value
Time	0min, 5min, 15min, 45min, 120min
Instrument	ThermoQAPlus
Matrix	elastase
Acquisition Mode	ddMS2

Chromatograms

Time=0min

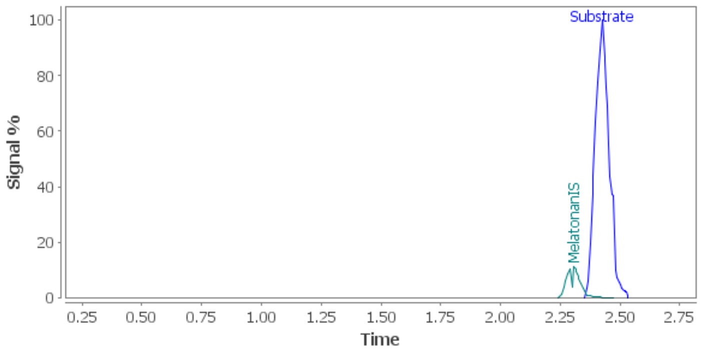

Time=5min

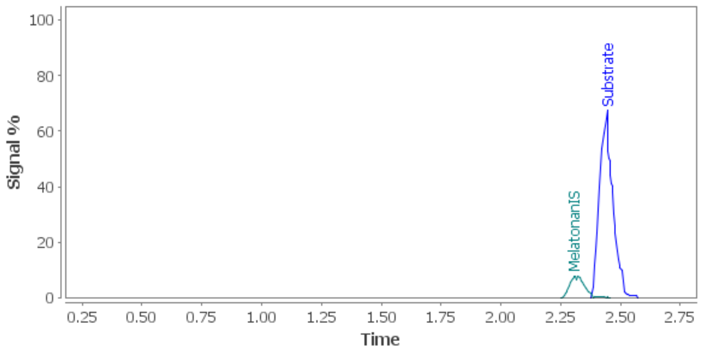

Time=15min

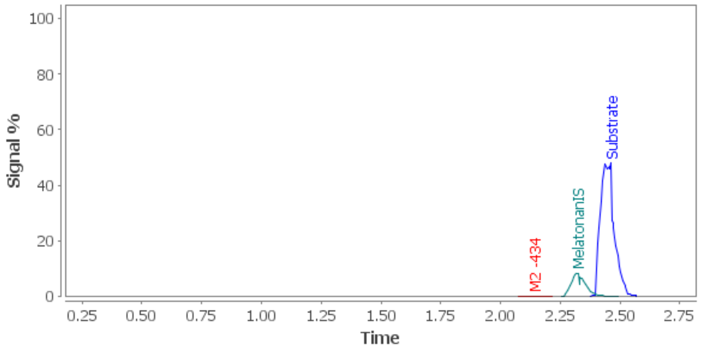

Time=45min

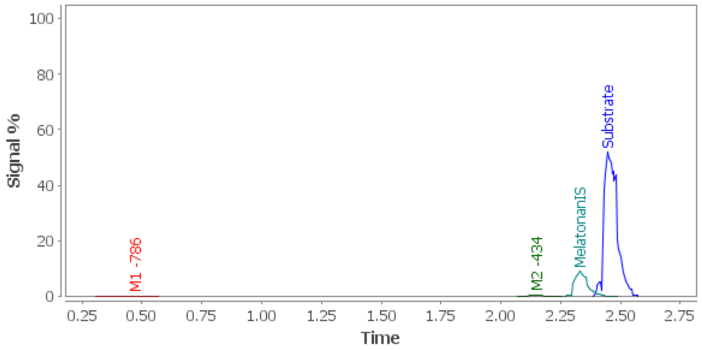

Time=120min

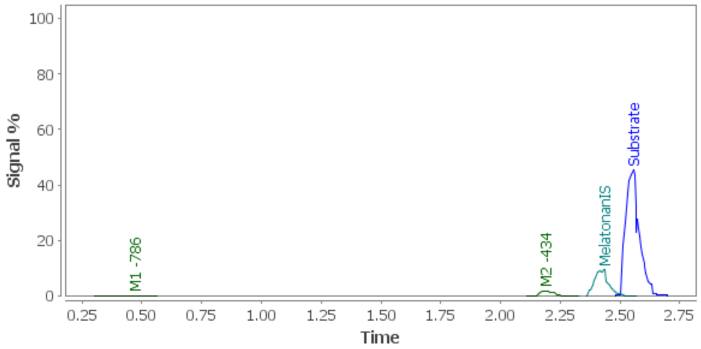

Custom Charts

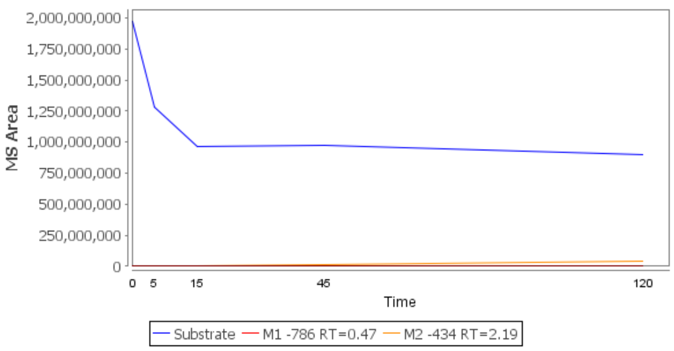

Fragmentation

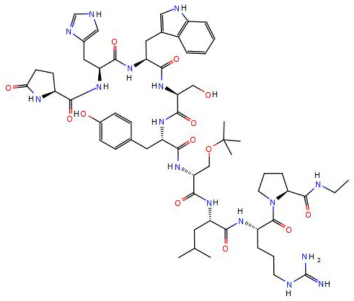

Buserelin

MS (+) FT

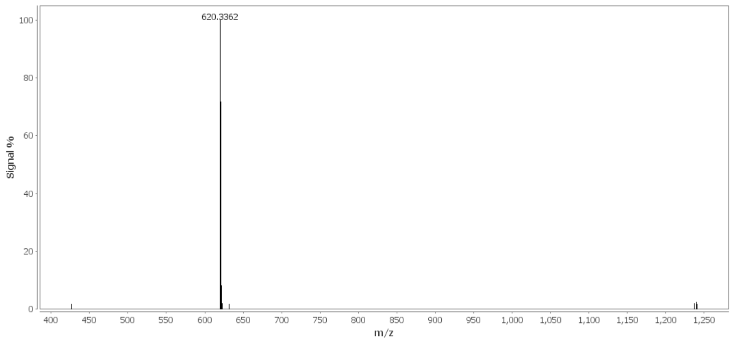

MS (+) FT

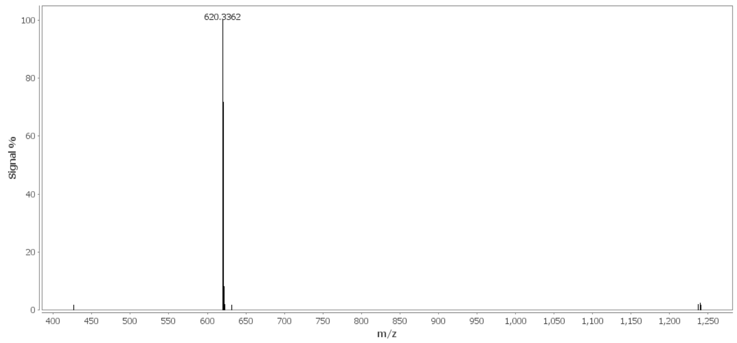

MS2 (+) FT activ = HCD:ce =

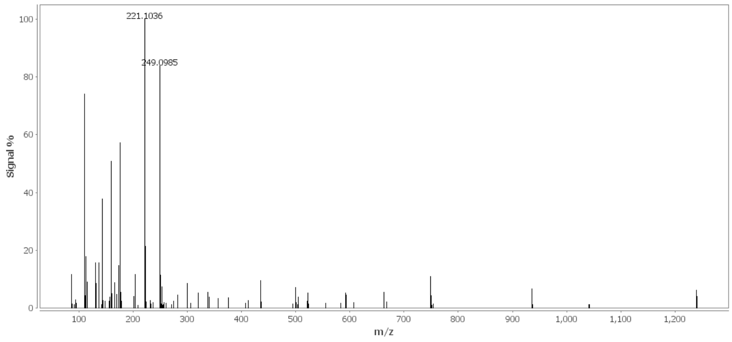

MS2 (+) FT activ = HCD:ce =

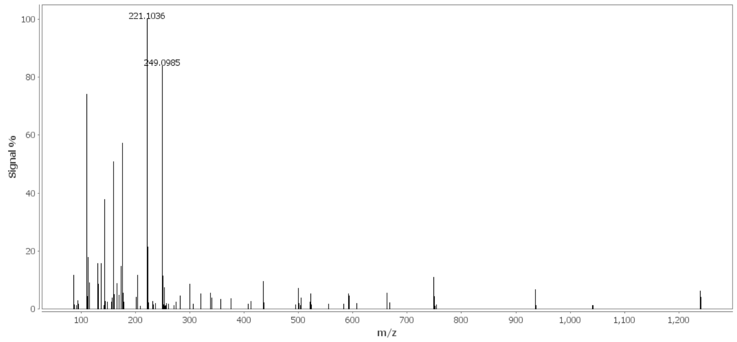

Metabolite: Substrate

Type	score	sub. m/z observed	sub. m/z calculated	sub ppm			met. m/z observed	met. m/z calculated	met. ppm
MATCH	102.0	1239.6660	1239.6633	-2.20	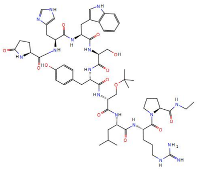	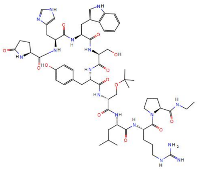	1239.6660	1239.6633	-2.20
MATCH	15.0	1239.6657	1239.6633	-1.95	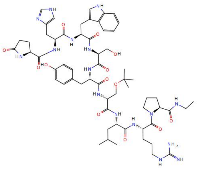	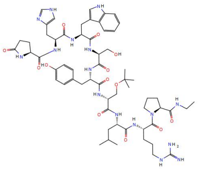	1239.6657	1239.6633	-1.95
MATCH	4.2	754.2892	754.2944	6.83	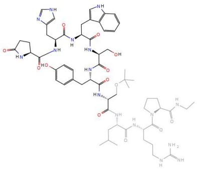	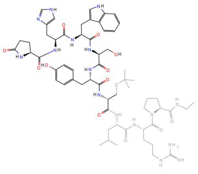	754.2892	754.2944	6.83

Metabolite: Substrate

Type	score	sub. m/z observed	sub. m/z calculated	sub ppm			met. m/z observed	met. m/z calculated	met. ppm
MATCH	200.0	620.3365	620.3353	-1.88	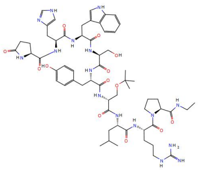	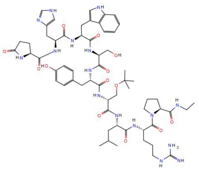	620.3365	620.3353	-1.88
MATCH	7.7	583.2991	583.2987	-0.68	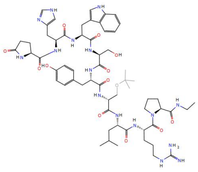	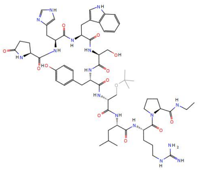	583.2991	583.2987	-0.68
MATCH	3.0	555.3973	555.3977	0.77	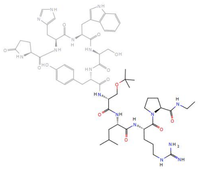	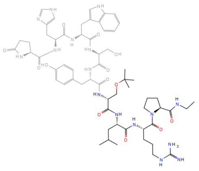	555.3973	555.3977	0.77
MATCH	8.2	504.1996	504.1990	-1.19	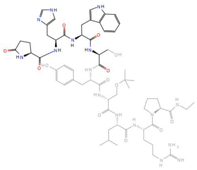	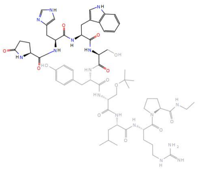	504.1996	504.1990	-1.19
MATCH	58.7	499.3360	499.3351	-1.79	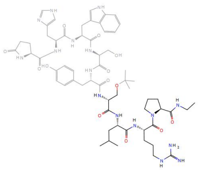	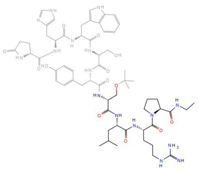	499.3360	499.3351	-1.79
MATCH	14.8	412.3036	412.3031	-1.39	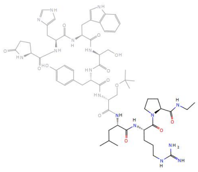	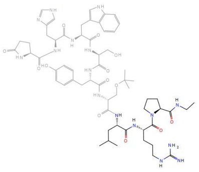	412.3036	412.3031	-1.39
MATCH	25.6	299.2192	299.2190	-0.75	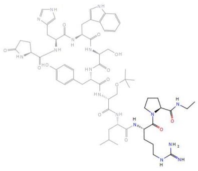	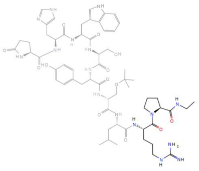	299.2192	299.2190	-0.75
MATCH	7.6	282.1926	282.1925	-0.61	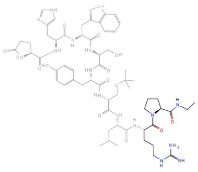	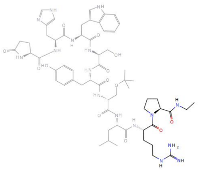	282.1926	282.1925	-0.61
MATCH	11.2	253.1658	253.1659	0.40	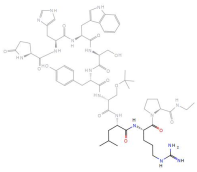	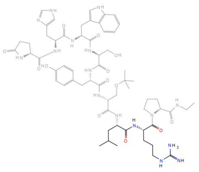	253.1658	253.1659	0.40

Metabolite: Substrate

Type	score	sub. m/z observed	sub. m/z calculated	sub ppm			met. m/z observed	met. m/z calculated	met. ppm
MATCH	179.9	249.0984	249.0982	-0.62	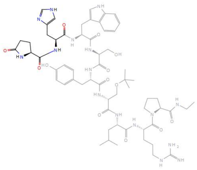	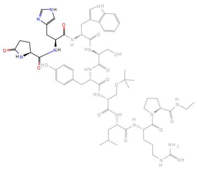	249.0984	249.0982	-0.62
MATCH	176.9	221.1035	221.1033	-0.97	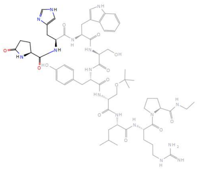	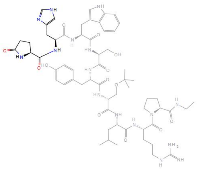	221.1035	221.1033	-0.97
MATCH	14.0	166.0615	166.0611	-2.17	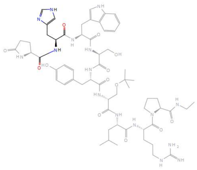	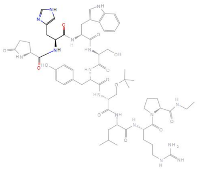	166.0615	166.0611	-2.17
MATCH	56.0	159.0919	159.0917	-1.14	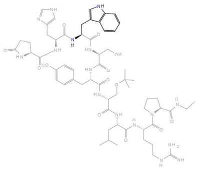	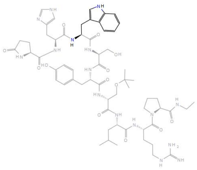	159.0919	159.0917	-1.14
MATCH	6.6	157.1084	157.1084	0.19	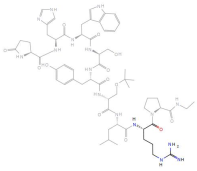	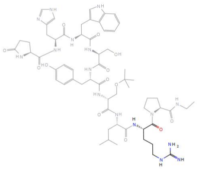	157.1084	157.1084	0.19
MATCH	69.5	143.1181	143.1179	-1.29	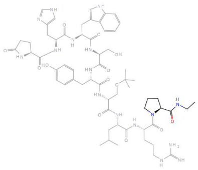	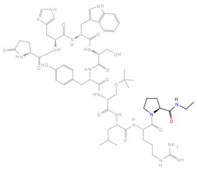	143.1181	143.1179	-1.29
MATCH	56.4	136.0759	136.0757	-1.77	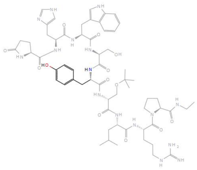	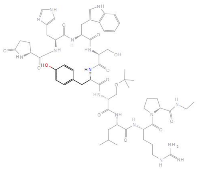	136.0759	136.0757	-1.77
MATCH	15.6	115.0870	115.0866	-3.64	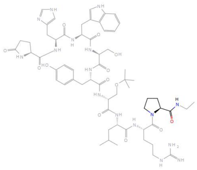	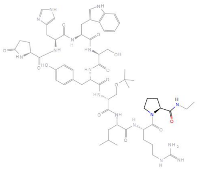	115.0870	115.0866	-3.64
MATCH	25.8	112.0874	112.0869	-4.04	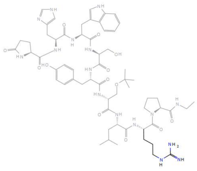	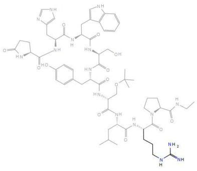	112.0874	112.0869	-4.04

Metabolite: Substrate

Type	score	sub. m/z observed	sub. m/z calculated	sub ppm			met. m/z observed	met. m/z calculated	met. ppm
MATCH	162.7	110.0718	110.0713	-4.62	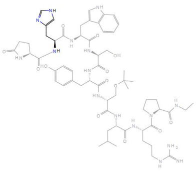	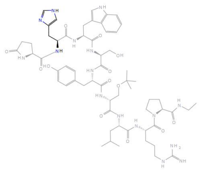	110.0718	110.0713	-4.62
MATCH	17.2	86.0972	86.0964	-9.30	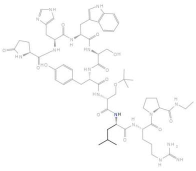	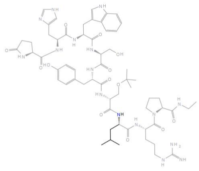	86.0972	86.0964	-9.30

MS (+) FT

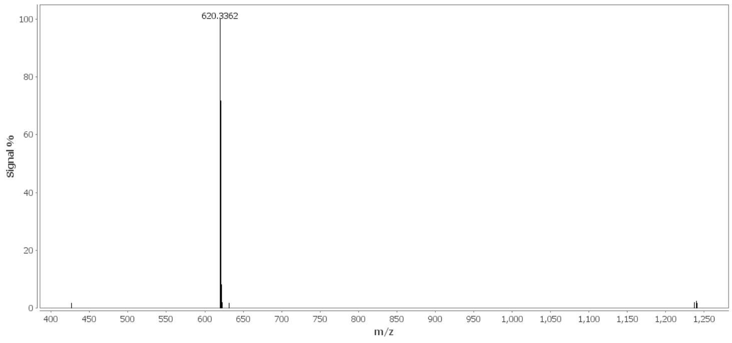

MS (+) FT

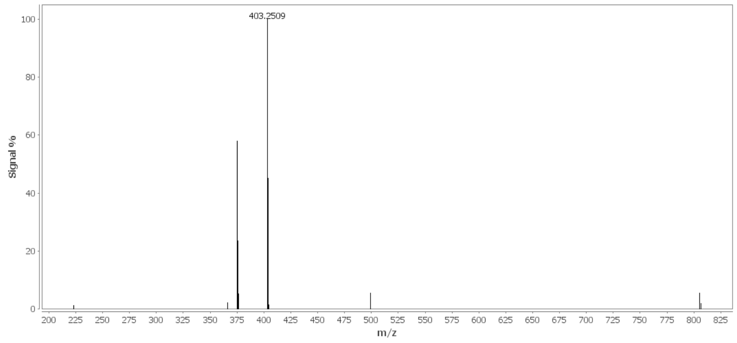

MS2 (+) FT activ = HCD:ce =

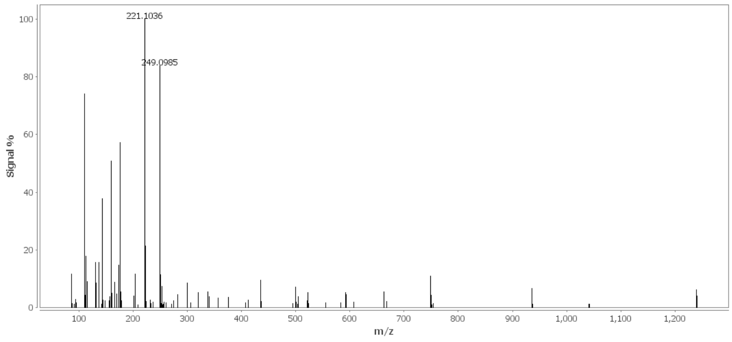

MS2 (+) FT activ = HCD:ce =

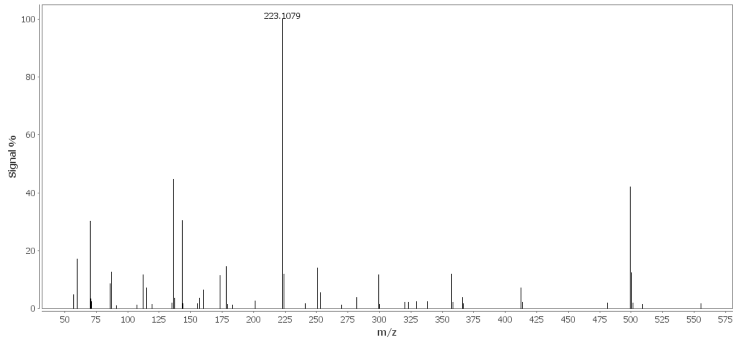

Metabolite: M2 -434 RT=2.19

Type	score	sub. m/z observed	sub. m/z calculated	sub ppm			met. m/z observed	met. m/z calculated	met. ppm
MATCH	200.0	620.3365	620.3353	-1.88	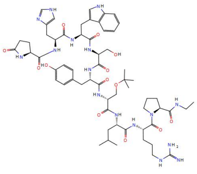	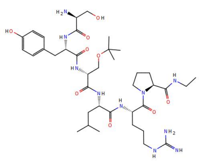	403.2509	403.2502	-1.90
MATCH	200.0	620.3365	620.3353	-1.88	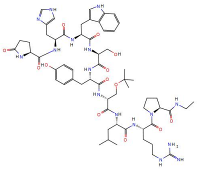	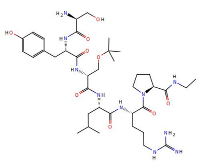	403.2509	403.2502	-1.90

Metabolite: M2 -434 RT=2.19

Type	score	sub. m/z observed	sub. m/z calculated	sub ppm			met. m/z observed	met. m/z calculated	met. ppm
MATCH	105.5	620.3365	620.3353	-1.88	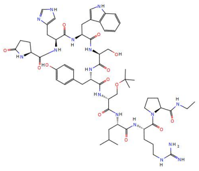	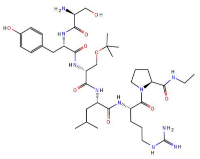	805.4939	805.4930	-1.08
MATCH	105.5	620.3365	620.3353	-1.88	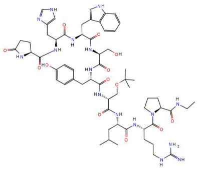	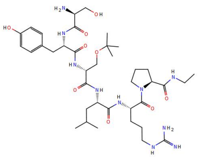	805.4939	805.4930	-1.08
MATCH	102.0	1239.6660	1239.6633	-2.20	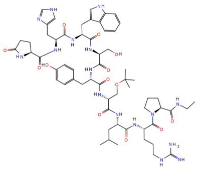	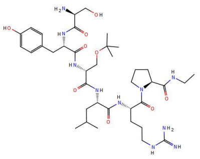	403.2509	403.2502	-1.90
MATCH	102.0	1239.6660	1239.6633	-2.20	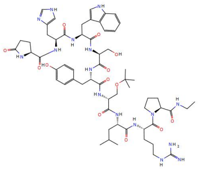	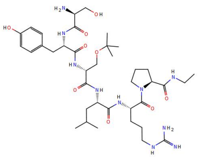	403.2509	403.2502	-1.90
MATCH	7.5	1239.6660	1239.6633	-2.20	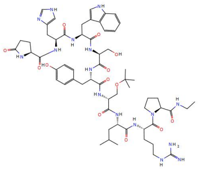	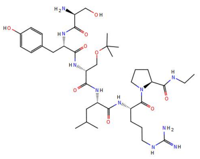	805.4939	805.4930	-1.08
MATCH	7.5	1239.6660	1239.6633	-2.20	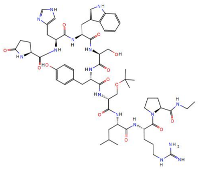	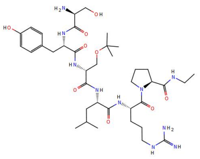	805.4939	805.4930	-1.08
MATCH	17.2	86.0972	86.0964	-9.30	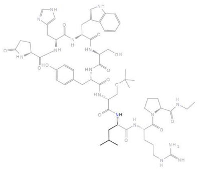	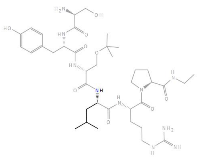	86.0972	86.0964	-8.54
MATCH	25.8	112.0874	112.0869	-4.04	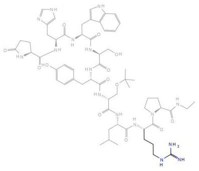	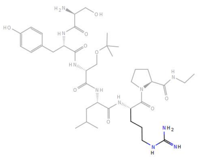	112.0874	112.0869	-4.14
MATCH	15.6	115.0870	115.0866	-3.64	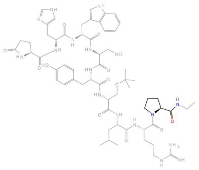	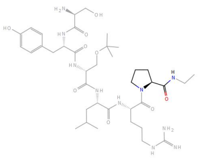	115.0869	115.0866	-2.52

Metabolite: M2 -434 RT=2.19

Type	score	sub. m/z observed	sub. m/z calculated	sub ppm			met. m/z observed	met. m/z calculated	met. ppm
MATCH	56.4	136.0759	136.0757	-1.77	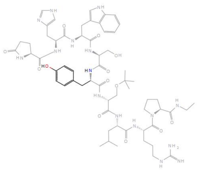	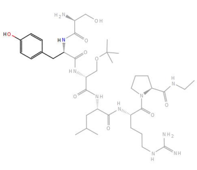	136.0760	136.0757	-1.93
MATCH	69.5	143.1181	143.1179	-1.29	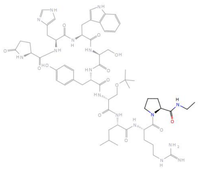	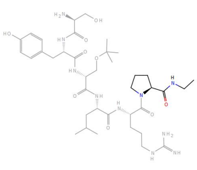	143.1181	143.1179	-1.48
MATCH	6.6	157.1084	157.1084	0.19	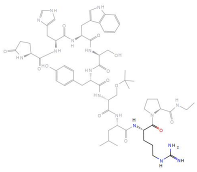	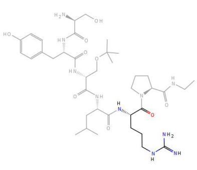	157.1086	157.1084	-1.10
MATCH	11.2	253.1658	253.1659	0.40	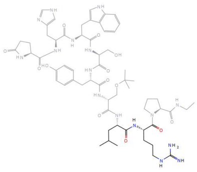	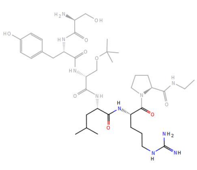	253.1657	253.1659	0.88
MATCH	7.6	282.1926	282.1925	-0.61	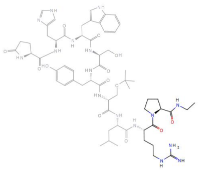	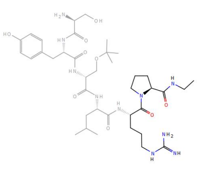	282.1928	282.1925	-1.40
MATCH	25.6	299.2192	299.2190	-0.75	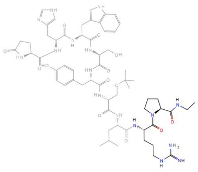	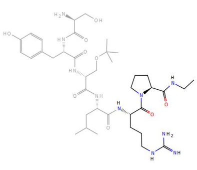	299.2190	299.2190	0.07
MATCH	14.8	412.3036	412.3031	-1.39	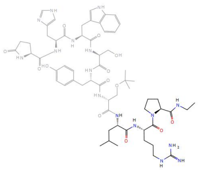	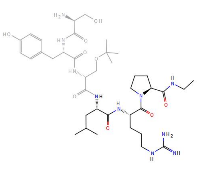	412.3037	412.3031	-1.46
MATCH	58.7	499.3360	499.3351	-1.79	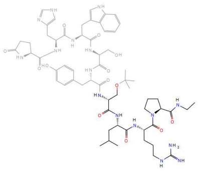	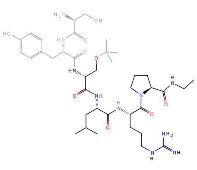	499.3354	499.3351	-0.68
MATCH	8.2	504.1996	504.1990	-1.19	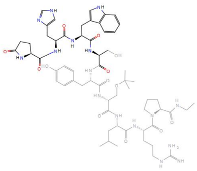	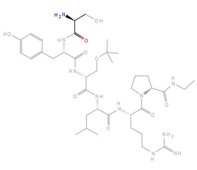	70.0295	70.0287	-11.0

Metabolite: M2 -434 RT=2.19

Type	score	sub. m/z observed	sub. m/z calculated	sub ppm			met. m/z observed	met. m/z calculated	met. ppm
MATCH	3.0	555.3973	555.3977	0.77	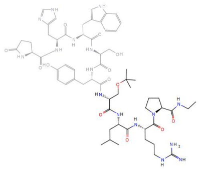	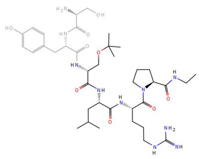	555.3953	555.3977	4.37
MATCH	7.7	583.2991	583.2987	-0.68	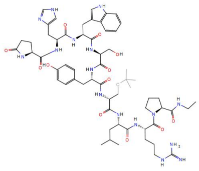	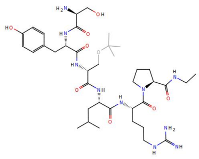	366.2134	366.2136	0.59
MATCH	4.2	754.2892	754.2944	6.83	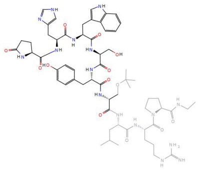	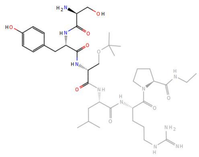	320.1251	320.1241	-3.06
MET_MATCH						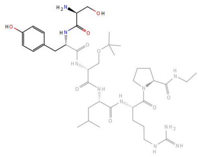	223.1091	223.1077	-6.14
MET_MATCH						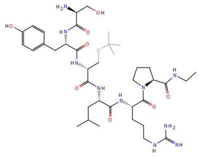	366.2137	366.2136	-0.28
MET_MATCH						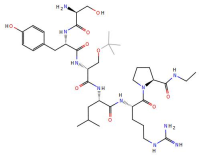	375.2195	375.2189	-1.68
MET_MATCH						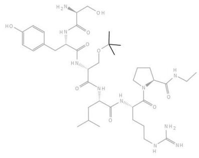	57.0709	57.0699	-17.2
MET_MATCH						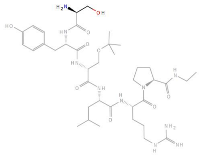	60.0454	60.0444	-16.0
MET_MATCH						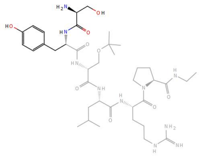	223.1079	223.1077	-0.73

Metabolite: M2 -434 RT=2.19

Type	score	sub. m/z observed	sub. m/z calculated	sub ppm		met. m/z observed	met. m/z calculated	met. ppm
MET_MATCH					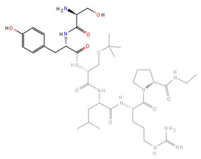	251.1028	251.1026	-0.63

MS (+) FT

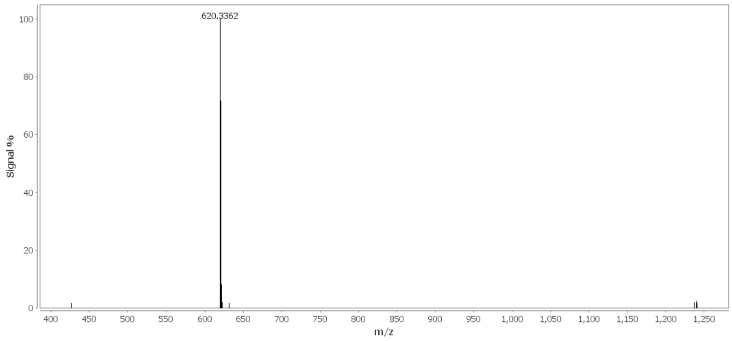

MS (+) FT

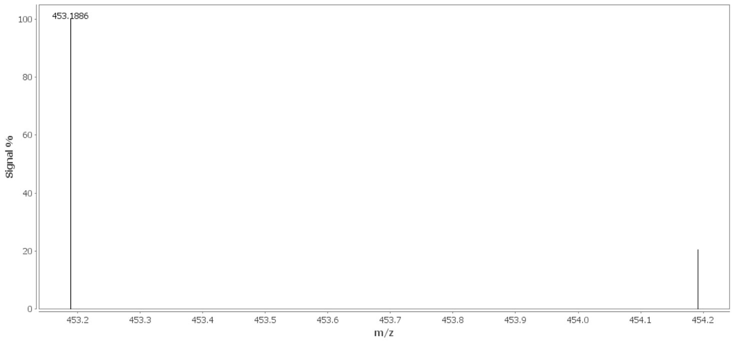

MS2 (+) FT activ = HCD:ce =

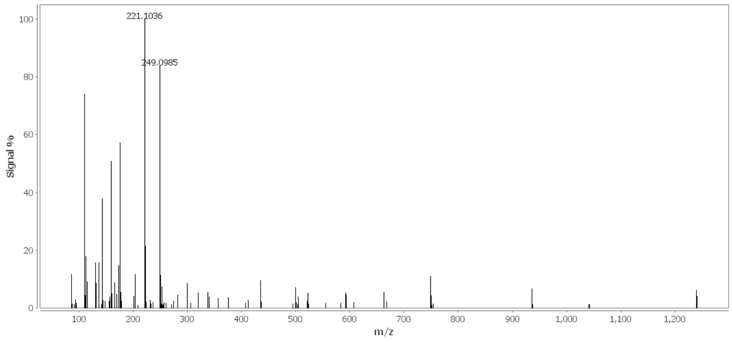

MS2 (+) FT activ = HCD:ce =

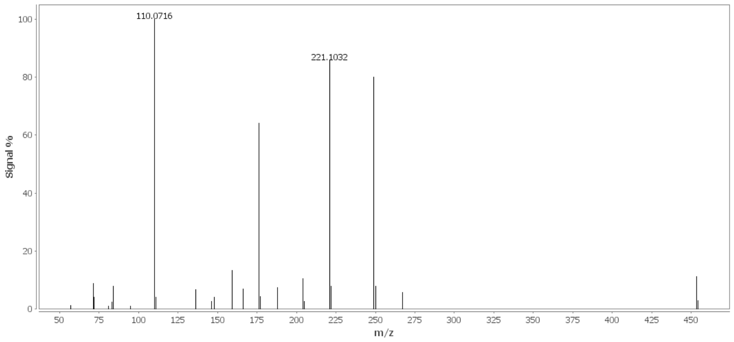

Metabolite: M1 -786 RT=0.47

Type	score	sub. m/z observed	sub. m/z calculated	sub ppm			met. m/z observed	met. m/z calculated	met. ppm
MATCH	200.0	620.3365	620.3353	-1.88	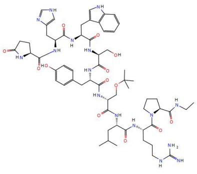	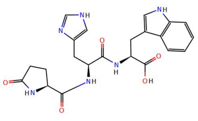	453.1886	453.1881	-1.04
						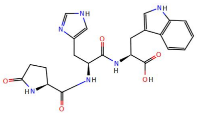	453.1886	453.1881	-1.04
MATCH	102.0	1239.6660	1239.6633	-2.20	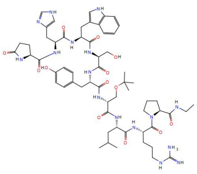	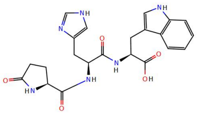	453.1886	453.1881	-1.04

Metabolite: M1 -786 RT=0.47

Type	score	sub. m/z observed	sub. m/z calculated	sub ppm			met. m/z observed	met. m/z calculated	met. ppm
						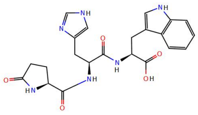	453.1886	453.1881	-1.04
MATCH	162.7	110.0718	110.0713	-4.62	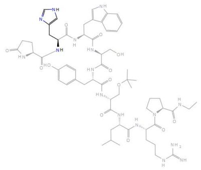	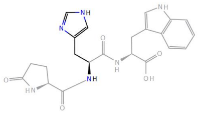	110.0716	110.0713	-3.18
MATCH	56.0	159.0919	159.0917	-1.14	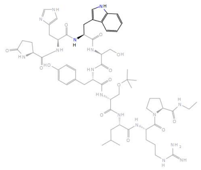	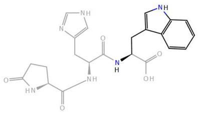	159.0916	159.0917	0.33
MATCH	14.0	166.0615	166.0611	-2.17	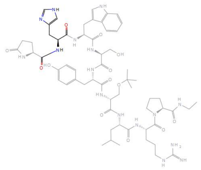	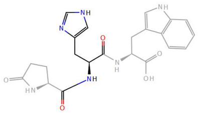	166.0610	166.0611	0.72
MATCH	176.9	221.1035	221.1033	-0.97	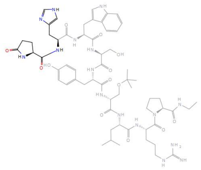	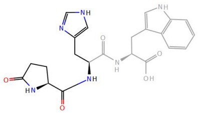	221.1032	221.1033	0.35
MATCH	179.9	249.0984	249.0982	-0.62	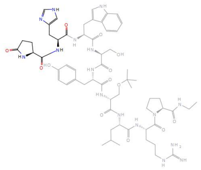	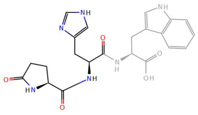	249.0980	249.0982	0.82
MATCH	15.0	1239.6657	1239.6633	-1.95	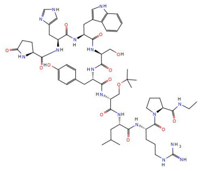	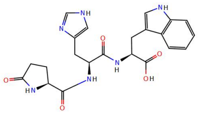	453.1879	453.1881	0.43
MISMATCH	-18.4	136.0759	136.0757	-1.77	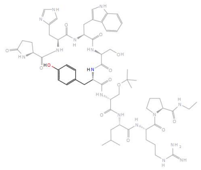		136.0757	136.0757	0.00
MET_MATCH						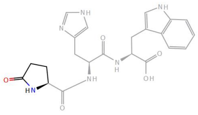	84.0450	84.0444	-7.69

Metabolite: M1 -786 RT=0.47

Type	score	sub. m/z observed	sub. m/z calculated	sub ppm		met. m/z observed	met. m/z calculated	met. ppm
MET_MATCH					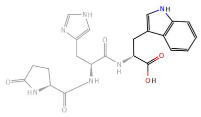	188.0707	188.0706	-0.36
