# Supplementary material for: Software-aided approach to investigate peptide structure and metabolic susceptibility of amide bonds in peptide drugs based on high resolution mass spectrometry
Source: PLoS One. 2017 Nov 1;12(11):e0186461. doi: 10.1371/journal.pone.0186461 (PMC5665424; doi:10.1371/journal.pone.0186461)
Supplement: S1 File — (ZIP) [file pone.0186461.s007.zip › SFiles/S42_File.pdf]

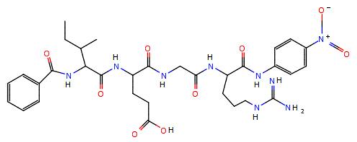

TrySub

Property name	Property value
Time	0min, 5min, 15min, 45min, 120min
Instrument	ThermoQAPlus
Matrix	trypsin
Acquisition Mode	ddMS2

Chromatograms

Time=0min

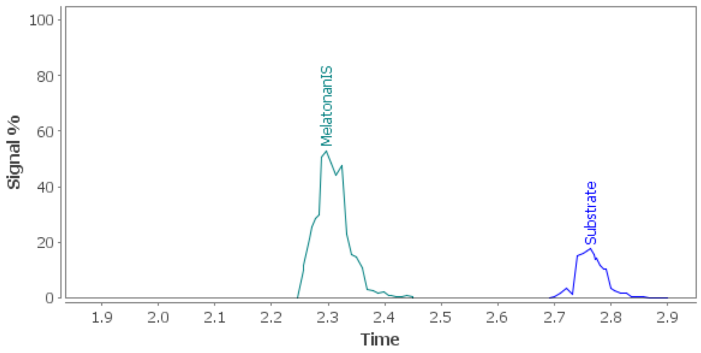

Time=5min

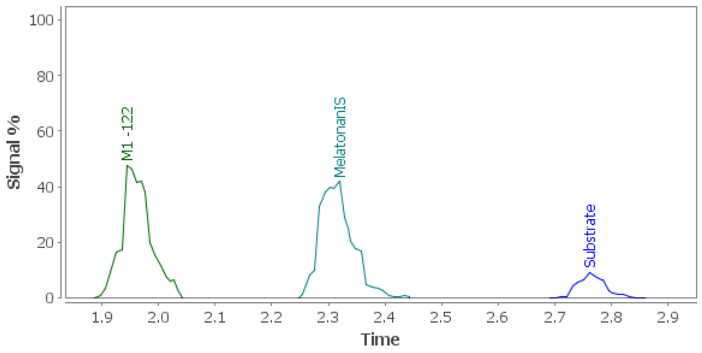

Time=15min

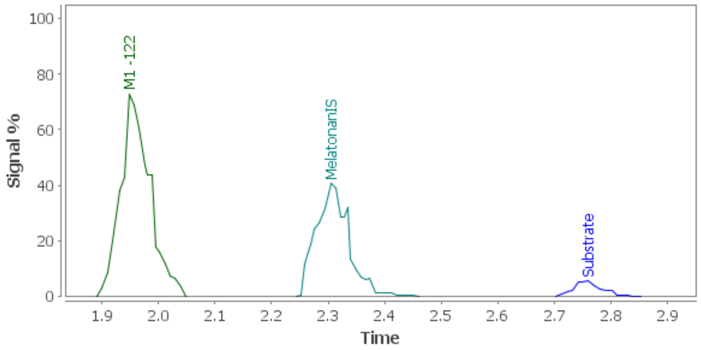

Time=45min

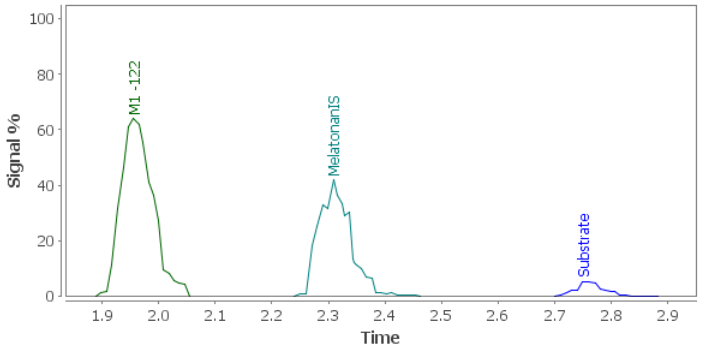

Time=120min

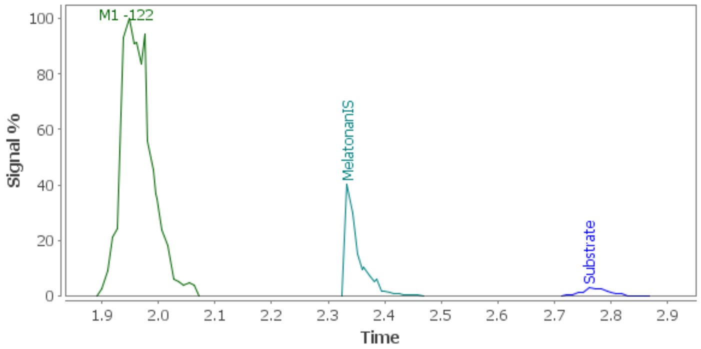

Custom Charts

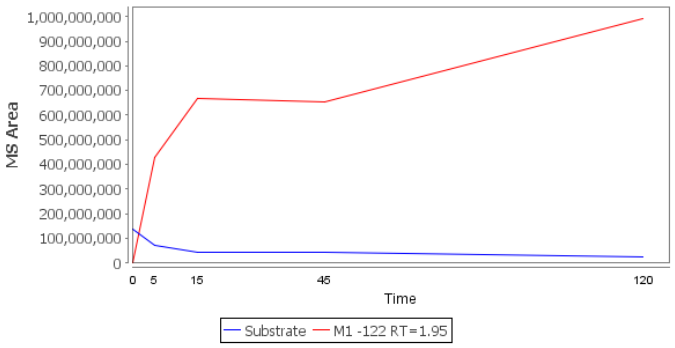

Fragmentation

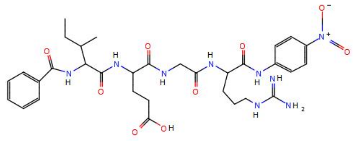

TrySub

MS (+) FT

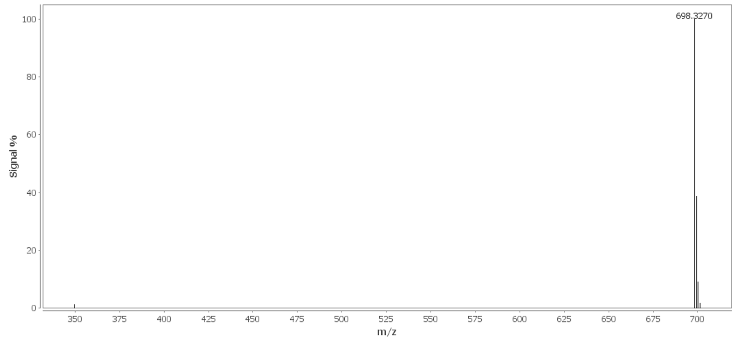

MS (+) FT

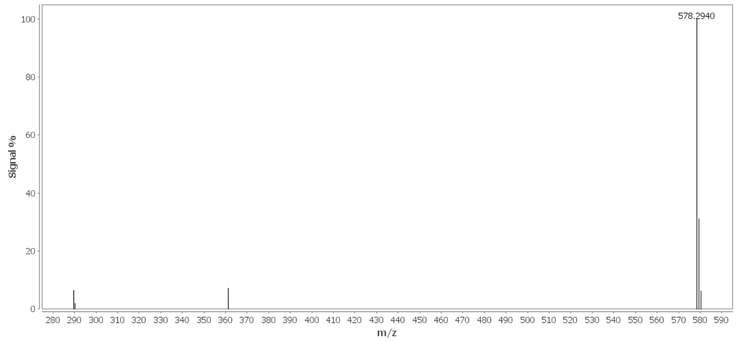

Metabolite: M1 -122 RT=1.95

Type	score	sub. m/z observed	sub. m/z calculated	sub ppm		met. m/z observed	met. m/z calculated	met. ppm
MET_MATCH					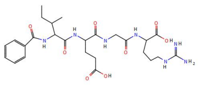	289.6509	289.6503	-2.10
MET_MATCH					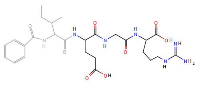	361.1835	361.1830	-1.26
MET_MATCH					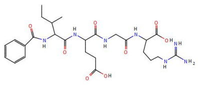	578.2940	578.2933	-1.22
