# Supplementary material for: Software-aided approach to investigate peptide structure and metabolic susceptibility of amide bonds in peptide drugs based on high resolution mass spectrometry
Source: PLoS One. 2017 Nov 1;12(11):e0186461. doi: 10.1371/journal.pone.0186461 (PMC5665424; doi:10.1371/journal.pone.0186461)
Supplement: S1 File — (ZIP) [file pone.0186461.s007.zip › SFiles/S43_File.pdf]

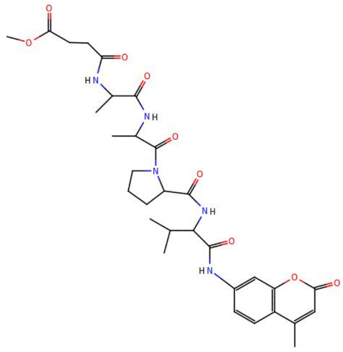

ElasSub

Property name	Property value
Time	0min, 5min, 15min, 45min, 120min
Instrument	ThermoQAPlus
Matrix	elastase
Acquisition Mode	ddMS2

Chromatograms

Time=0min

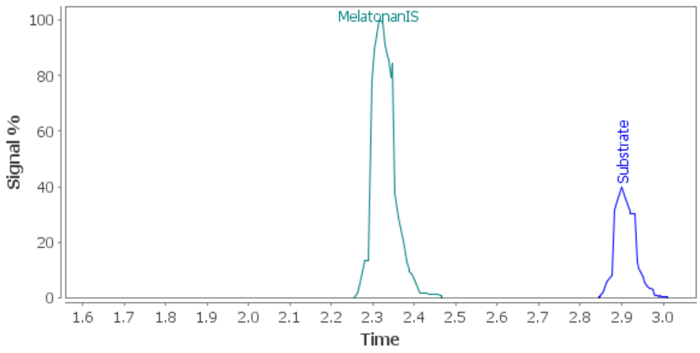

Time=5min

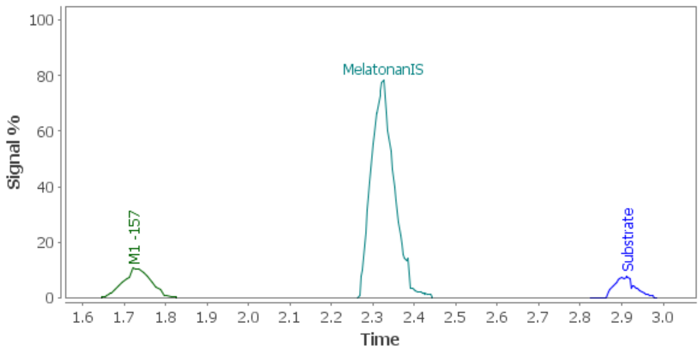

Time=15min

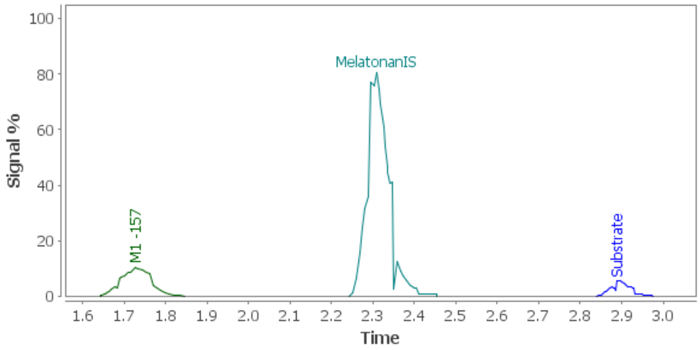

Time=45min

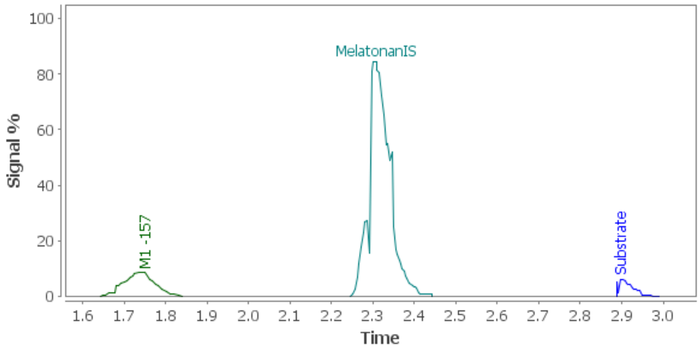

Time=120min

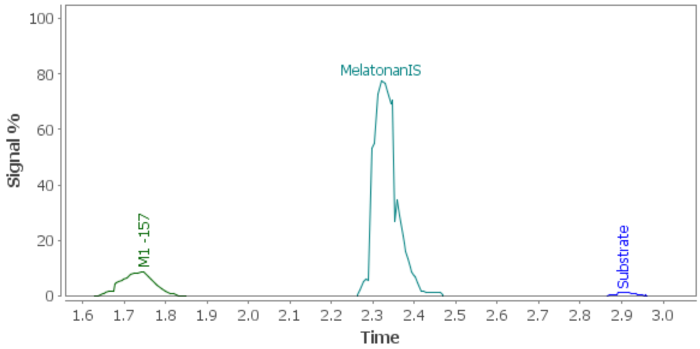

Custom Charts

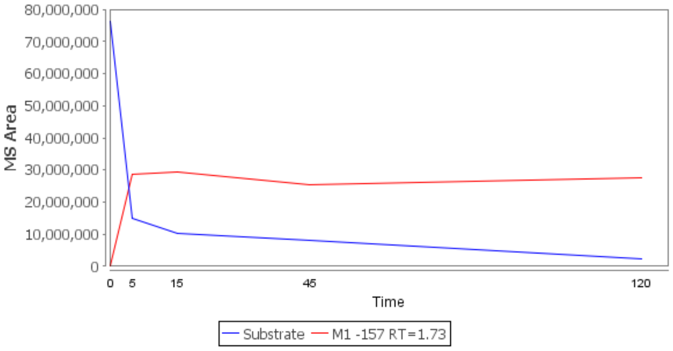

Fragmentation

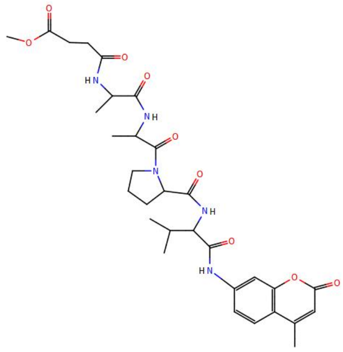

ElasSub

MS (+) FT

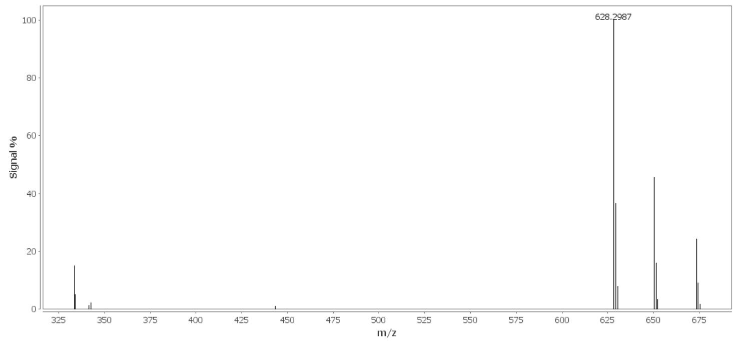

MS (+) FT

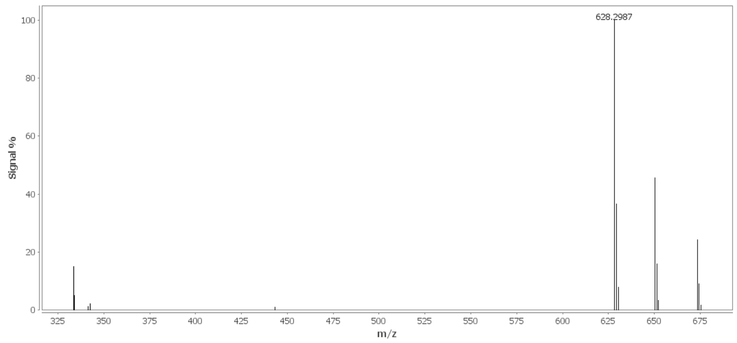

MS2 (+) FT activ = HCD:ce =

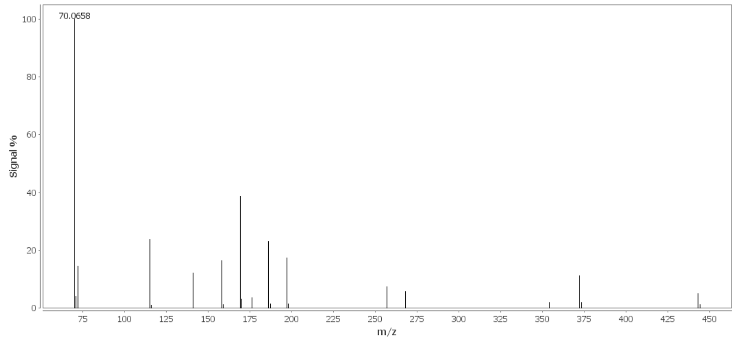

MS2 (+) FT activ = HCD:ce =

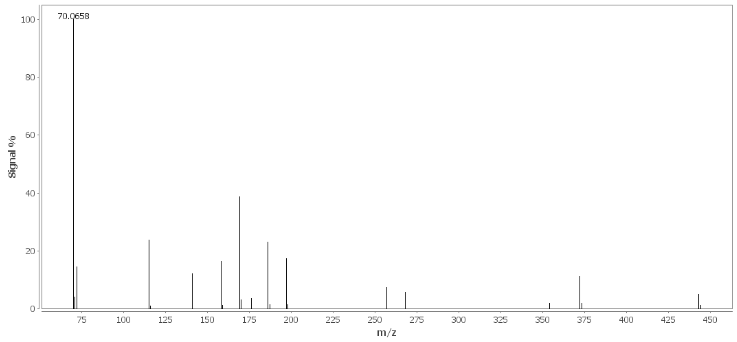

Metabolite: Substrate

Type	score	sub. m/z observed	sub. m/z calculated	sub ppm			met. m/z observed	met. m/z calculated	met. ppm
MATCH	200.0	628.2987	628.2977	-1.52	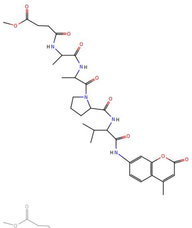	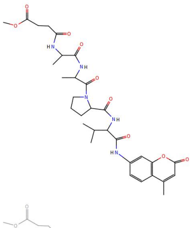	628.2987	628.2977	-1.52
MATCH	8.9	443.2287	443.2289	0.48	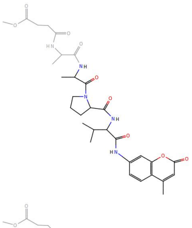	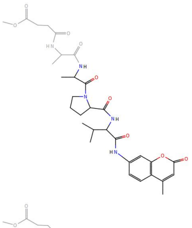	443.2287	443.2289	0.48
MATCH	84.8	372.1910	372.1918	2.17	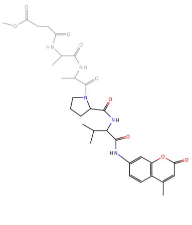	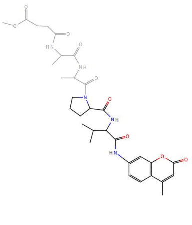	372.1910	372.1918	2.17

Metabolite: Substrate

Type	score	sub. m/z observed	sub. m/z calculated	sub ppm			met. m/z observed	met. m/z calculated	met. ppm
MATCH	10.7	257.1127	257.1132	1.94	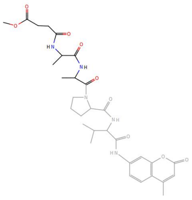	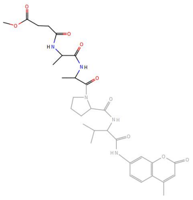	257.1127	257.1132	1.94
MATCH	45.7	186.0759	186.0761	1.23	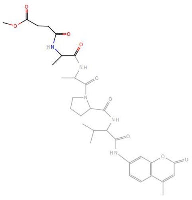	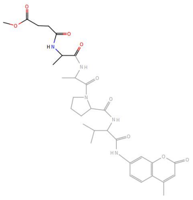	186.0759	186.0761	1.23
MATCH	43.0	169.1333	169.1335	1.40	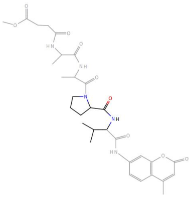	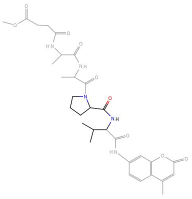	169.1333	169.1335	1.40
MATCH	18.0	169.0969	169.0972	1.30	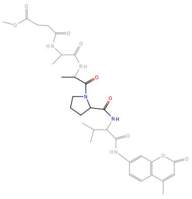	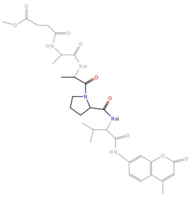	169.0969	169.0972	1.30
MATCH	18.0	169.0969	169.0972	1.30	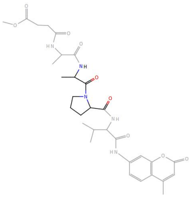	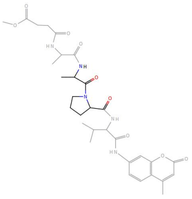	169.0969	169.0972	1.30
MATCH	33.2	158.0810	158.0812	1.08	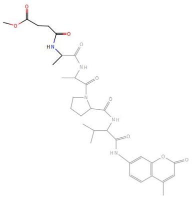	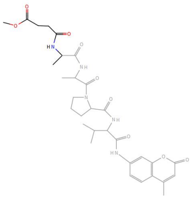	158.0810	158.0812	1.08
MATCH	15.4	141.1022	141.1022	0.60	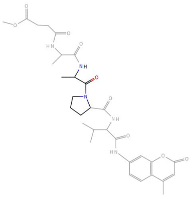	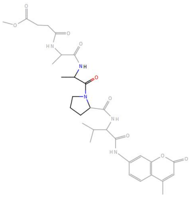	141.1022	141.1022	0.60
MATCH	47.6	115.0391	115.0390	-1.38	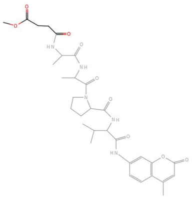	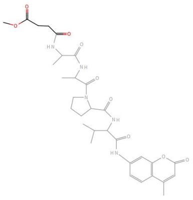	115.0391	115.0390	-1.38
MATCH	200.0	70.0658	70.0651	-10.3	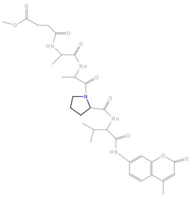	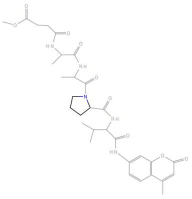	70.0658	70.0651	-10.3

MS (+) FT

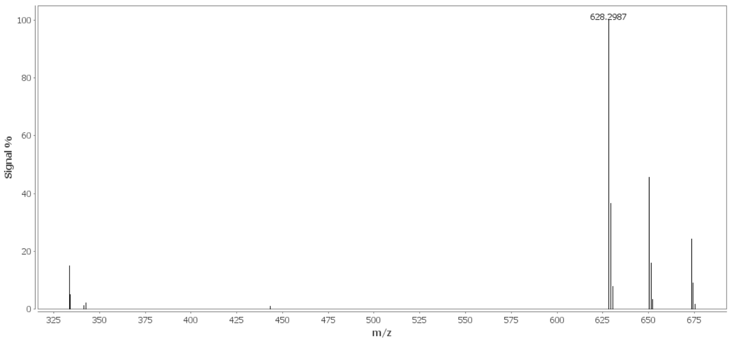

MS (+) FT

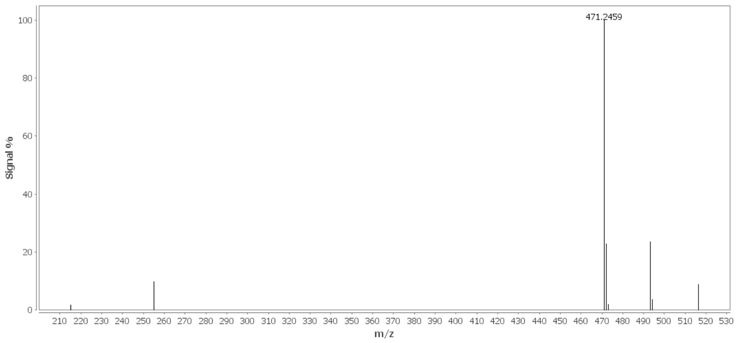

MS2 (+) FT activ = HCD:ce =

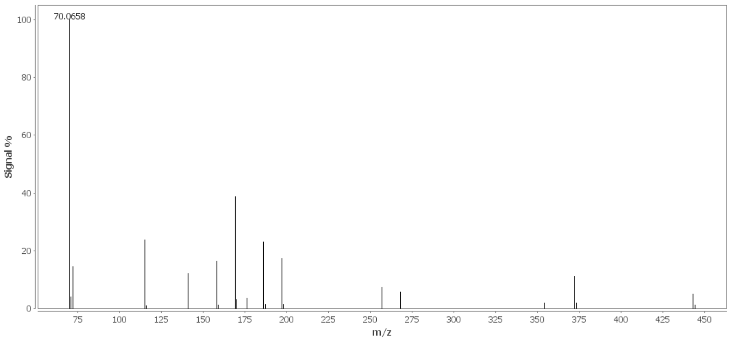

MS2 (+) FT activ = HCD:ce =

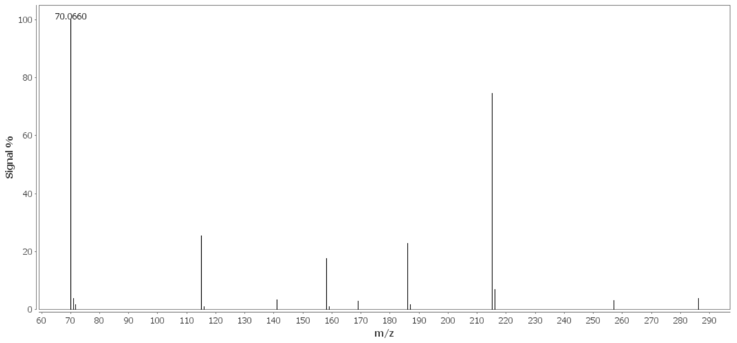

Metabolite: M1 -157 RT=1.73

Type	score	sub. m/z observed	sub. m/z calculated	sub ppm			met. m/z observed	met. m/z calculated	met. ppm
MATCH	200.0	628.2987	628.2977	-1.52	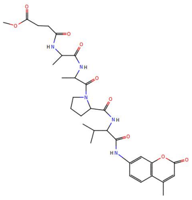	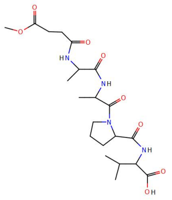	471.2459	471.2449	-1.96
MATCH	200.0	70.0658	70.0651	-10.3	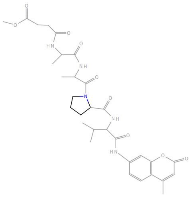	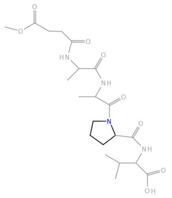	70.0660	70.0651	-12.1
MATCH	47.6	115.0391	115.0390	-1.38	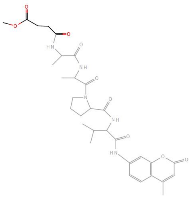	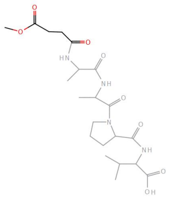	115.0394	115.0390	-3.32
MATCH	15.4	141.1022	141.1022	0.60	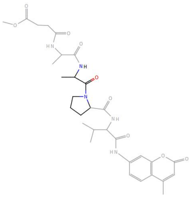	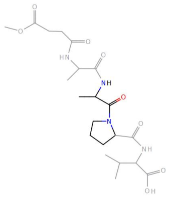	141.1024	141.1022	-1.39
MATCH	33.2	158.0810	158.0812	1.08	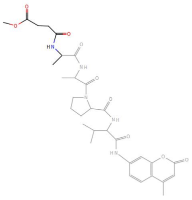	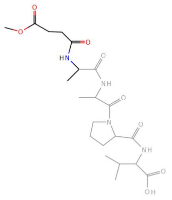	158.0813	158.0812	-0.68

Metabolite: M1 -157 RT=1.73

Type	score	sub. m/z observed	sub. m/z calculated	sub ppm			met. m/z observed	met. m/z calculated	met. ppm
MATCH	18.0	169.0969	169.0972	1.30	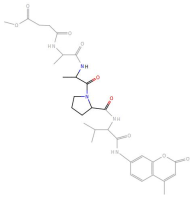	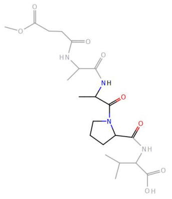	169.0972	169.0972	-0.47
MATCH	18.0	169.0969	169.0972	1.30	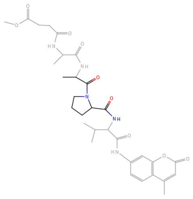	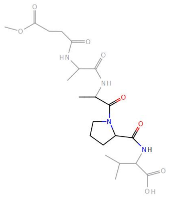	169.0972	169.0972	-0.47
MATCH	43.0	169.1333	169.1335	1.40	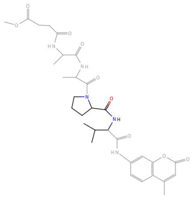	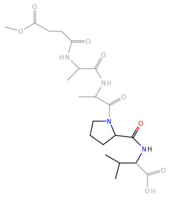	169.1337	169.1335	-0.86
MATCH	45.7	186.0759	186.0761	1.23	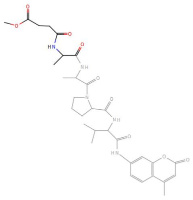	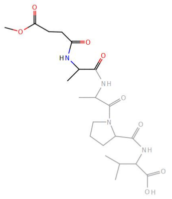	186.0762	186.0761	-0.74
MATCH	10.7	257.1127	257.1132	1.94	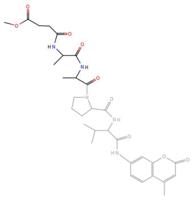	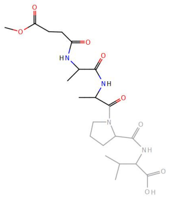	257.1134	257.1132	-0.93
MATCH	84.8	372.1910	372.1918	2.17	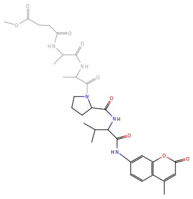	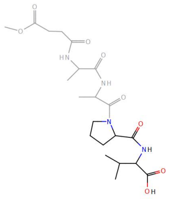	215.1391	215.1390	-0.48
MATCH	8.9	443.2287	443.2289	0.48	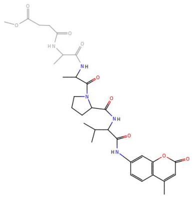	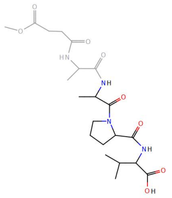	286.1761	286.1761	0.23
MET_MATCH						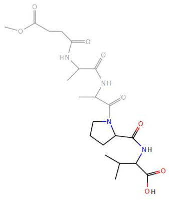	215.1396	215.1390	-2.57
