# Supplementary material for: Software-aided approach to investigate peptide structure and metabolic susceptibility of amide bonds in peptide drugs based on high resolution mass spectrometry
Source: PLoS One. 2017 Nov 1;12(11):e0186461. doi: 10.1371/journal.pone.0186461 (PMC5665424; doi:10.1371/journal.pone.0186461)
Supplement: S1 File — (ZIP) [file pone.0186461.s007.zip › SFiles/S44_File.pdf]

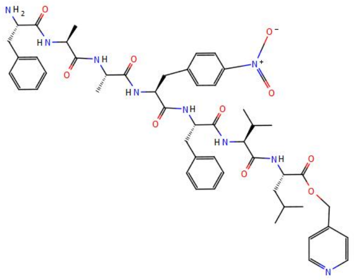

PepsinSub

Property name	Property value
Time	0min, 5min, 15min, 45min, 120min
Instrument	ThermoQAPlus
Acquisition Mode	ddMS2
Matrix	pepsin

Chromatograms

Time=0min

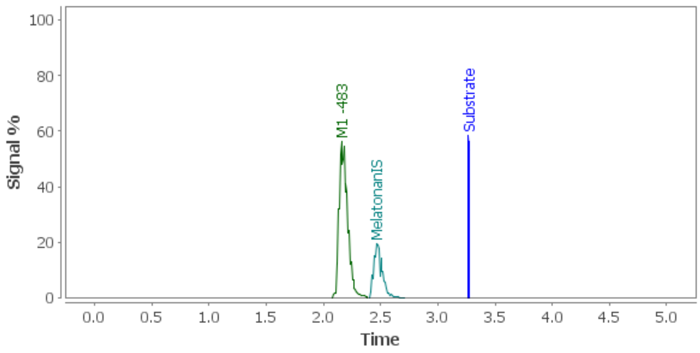

Time=5min

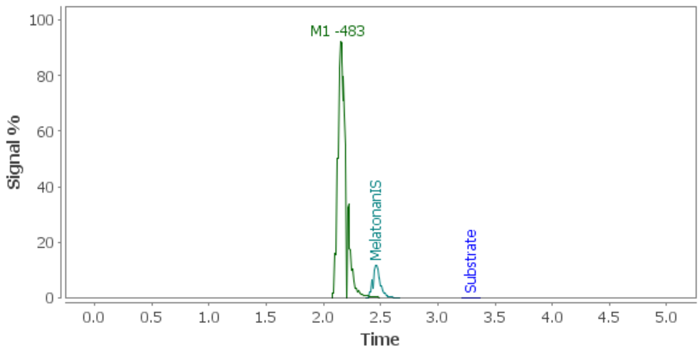

Time=15min

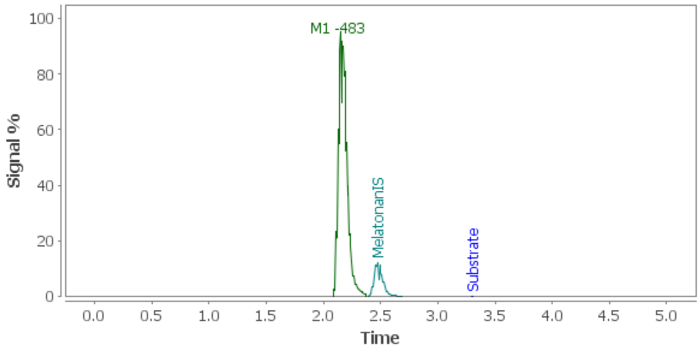

Time=45min

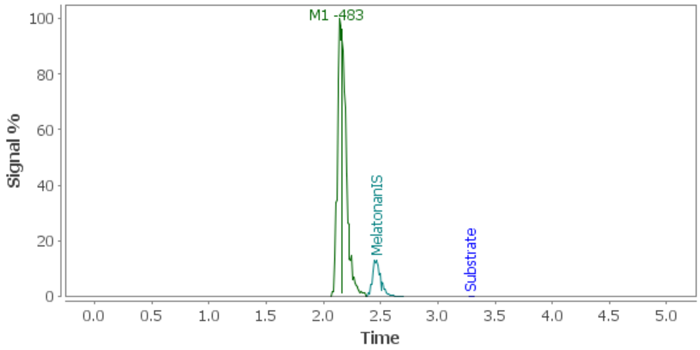

Time=120min

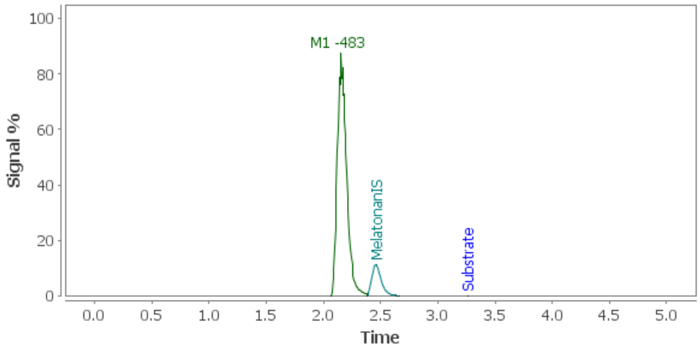

Custom Charts

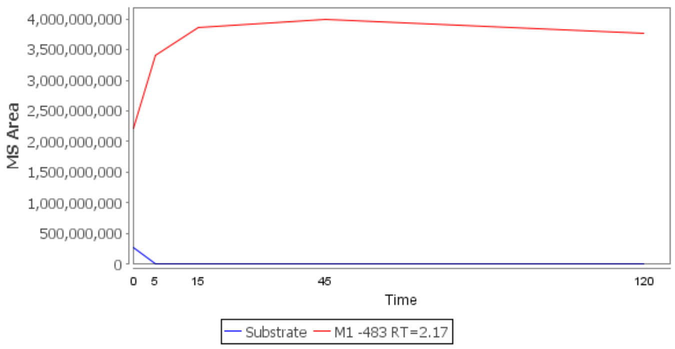

Fragmentation

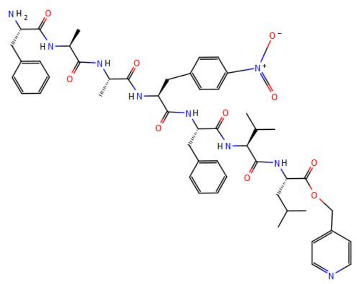

PepsinSub

MS (+) FT

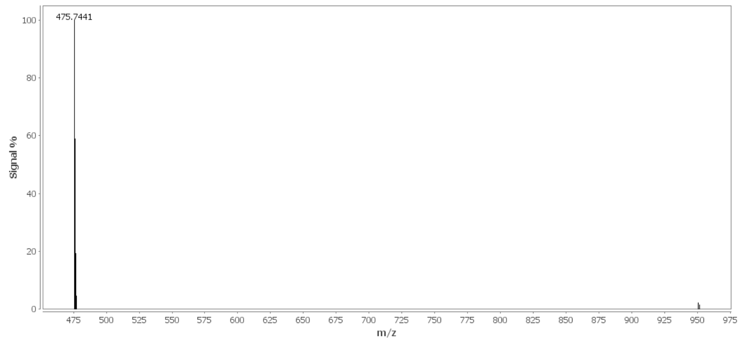

MS (+) FT

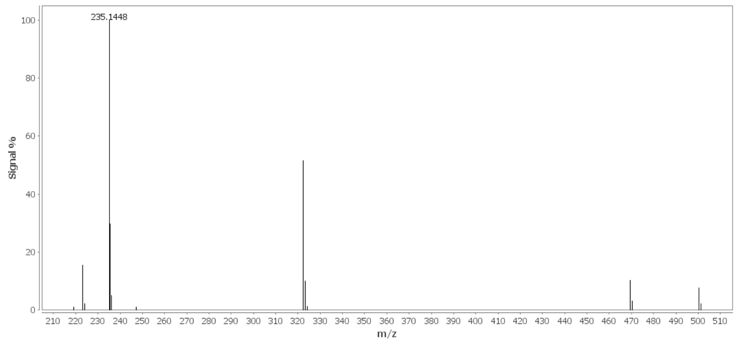

Metabolite: M1 -483 RT=2.17

Type	score	sub. m/z observed	sub. m/z calculated	sub ppm		met. m/z observed	met. m/z calculated	met. ppm
MET_MATCH					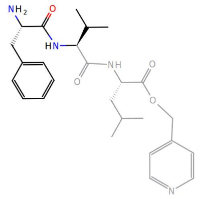	219.1496	219.1492	-1.90
MET_MATCH					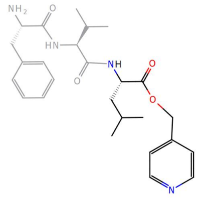	223.1445	223.1441	-1.96
MET_MATCH					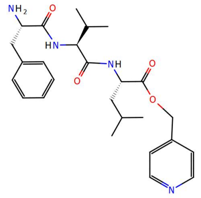	235.1448	235.1441	-2.83
MET_MATCH					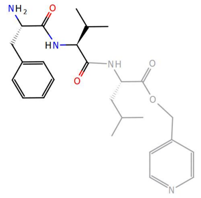	247.1448	247.1441	-2.62
MET_MATCH					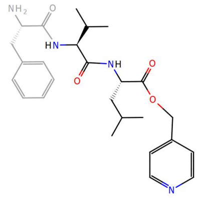	322.2133	322.2125	-2.46

Metabolite: M1 -483 RT=2.17

Type	score	sub. m/z observed	sub. m/z calculated	sub ppm		met. m/z observed	met. m/z calculated	met. ppm
MET_MATCH					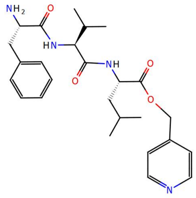	469.2824	469.2809	-3.11
