# Supplementary material for: Software-aided approach to investigate peptide structure and metabolic susceptibility of amide bonds in peptide drugs based on high resolution mass spectrometry
Source: PLoS One. 2017 Nov 1;12(11):e0186461. doi: 10.1371/journal.pone.0186461 (PMC5665424; doi:10.1371/journal.pone.0186461)
Supplement: S1 File — (ZIP) [file pone.0186461.s007.zip › SFiles/S45_File.pdf]

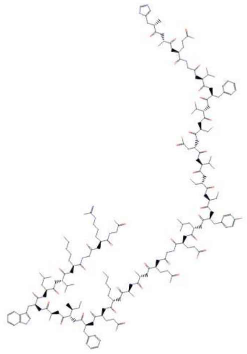

GLP1

Property name	Property value
Time	0min, 5min, 15min, 30min, 60min
Instrument	ddMS2
Matrix	DPP4

Chromatograms

Time=0min

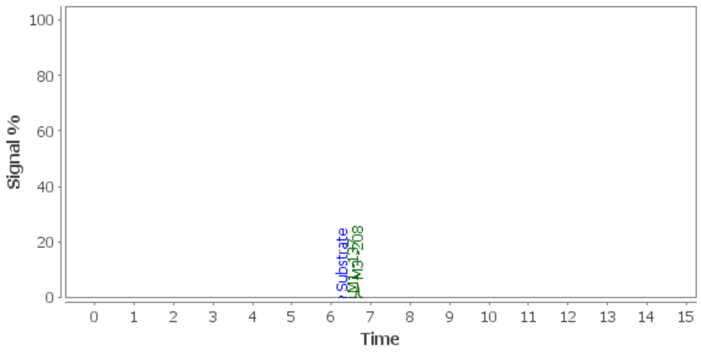

Time=5min

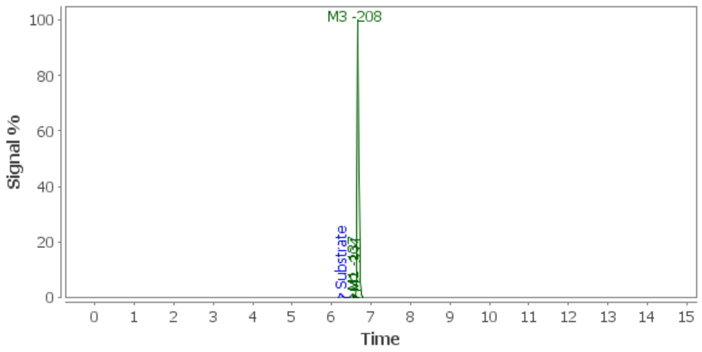

Time=15min

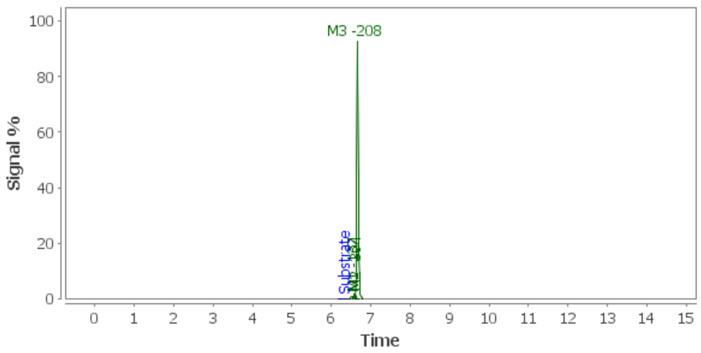

Time=30min

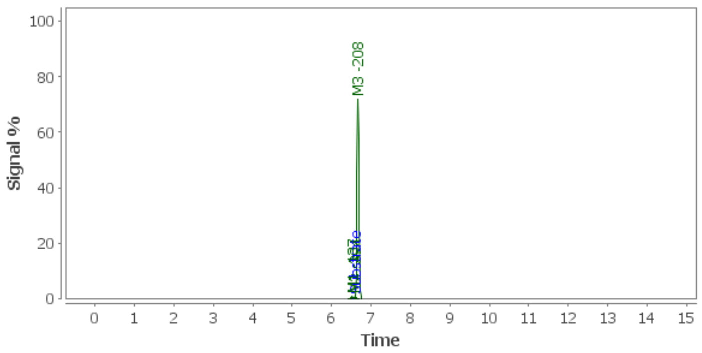

Time=60min

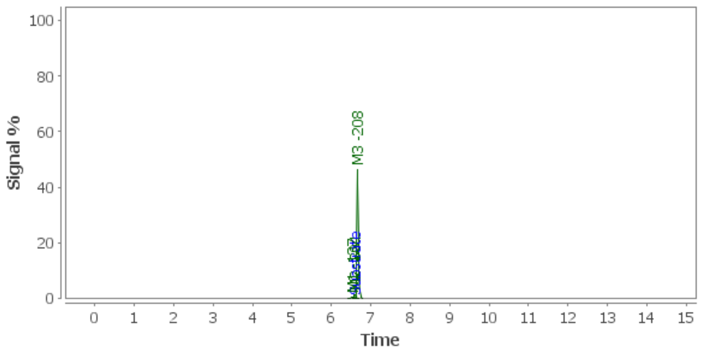

Custom Charts

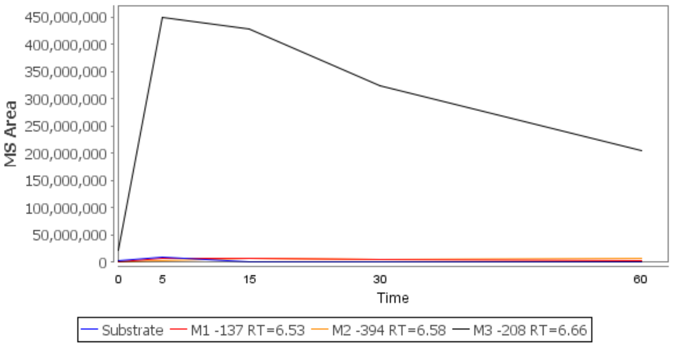

Fragmentation

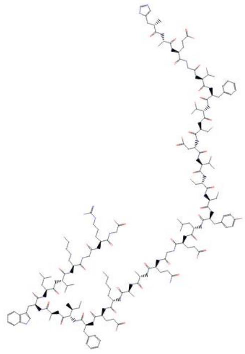

GLP1

MS (+) FT

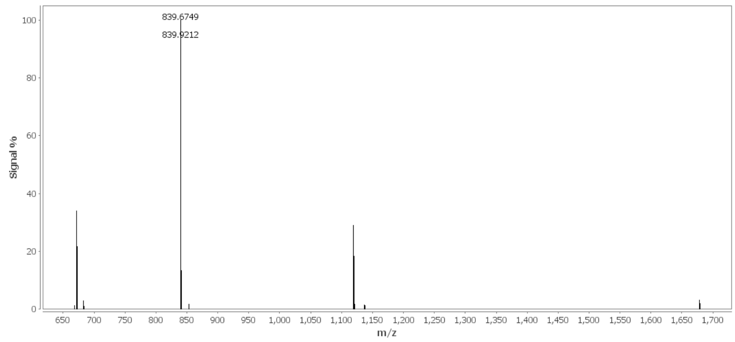

MS (+) FT

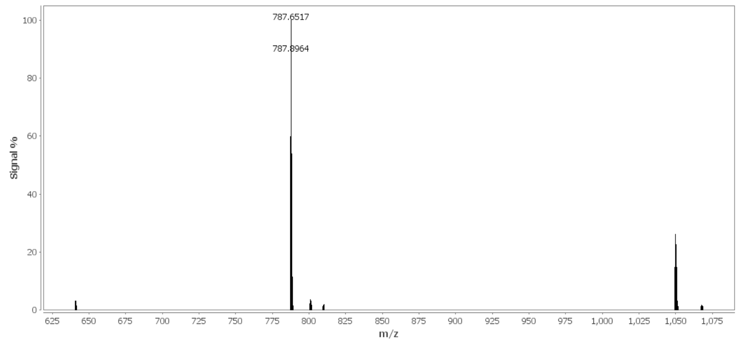

MS2 (+) FT activ = HCD:ce =

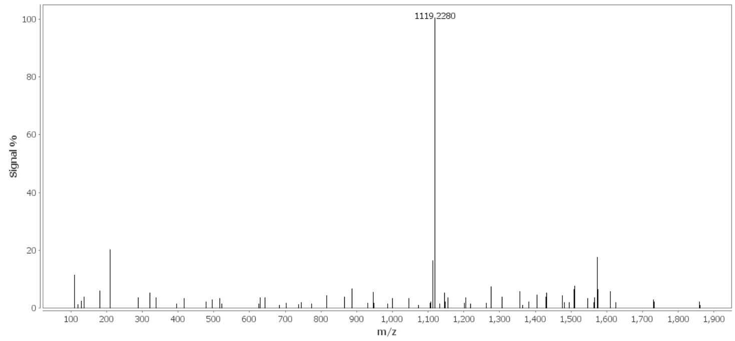

MS2 (+) FT activ = HCD:ce =

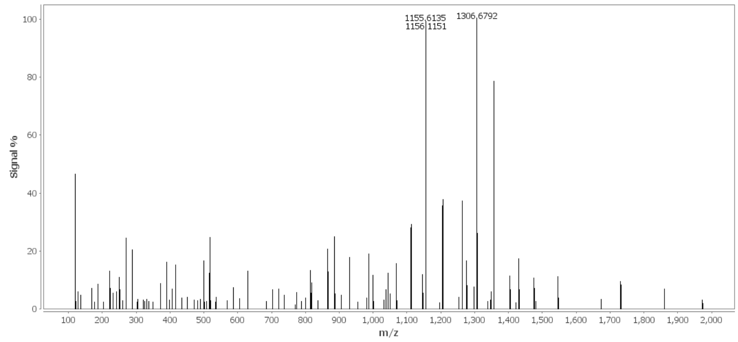

Metabolite: M3 -208 RT=6.66

Type	score	sub. m/z observed	sub. m/z calculated	sub ppm			met. m/z observed	met. m/z calculated	met. ppm
MATCH	78.2	671.7420	671.7409	-1.66	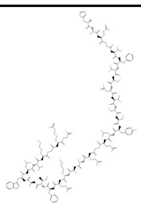	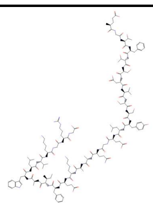	787.4065	787.4003	-7.95
MATCH	78.2	671.7420	671.7409	-1.66	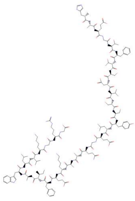	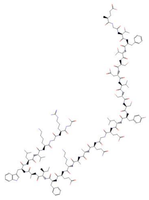	787.4065	787.4003	-7.95
						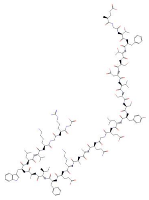	787.4065	787.4003	-7.95

Metabolite: M3 -208 RT=6.66

Type	score	sub. m/z observed	sub. m/z calculated	sub ppm			met. m/z observed	met. m/z calculated	met. ppm
MATCH	33.3	671.7420	671.7409	-1.66	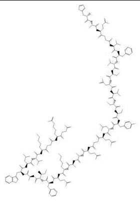	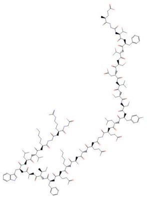	1049.5284	1049.5313	2.79
MATCH	33.3	671.7420	671.7409	-1.66	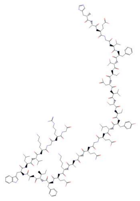	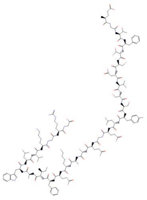	1049.5284	1049.5313	2.79
						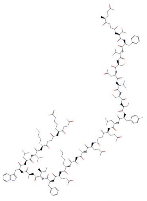	1049.5284	1049.5313	2.79
						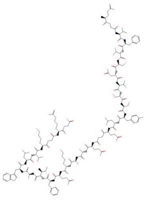	1049.5284	1049.5313	2.79
MATCH	112.9	839.4275	839.4243	-3.78	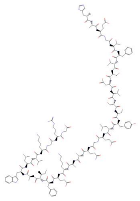	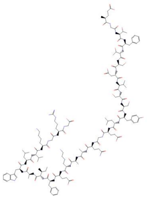	787.4065	787.4003	-7.95
MATCH	112.9	839.4275	839.4243	-3.78	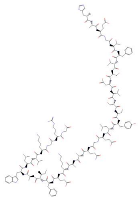	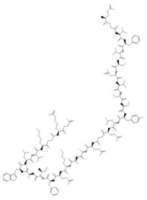	787.4065	787.4003	-7.95
						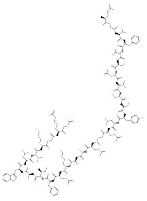	787.4065	787.4003	-7.95
MATCH	67.9	839.4275	839.4243	-3.78	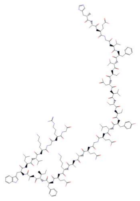	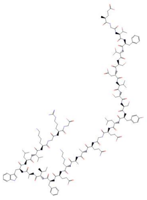	1049.5284	1049.5313	2.79
MATCH	67.9	839.4275	839.4243	-3.78	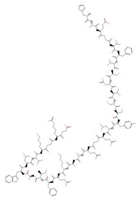	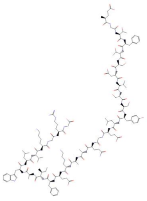	1049.5284	1049.5313	2.79

Metabolite: M3 -208 RT=6.66

Type	score	sub. m/z observed	sub. m/z calculated	sub ppm			met. m/z observed	met. m/z calculated	met. ppm
						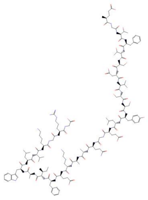	1049.5284	1049.5313	2.79
						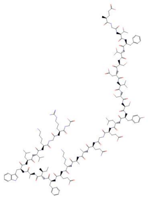	1049.5284	1049.5313	2.79
MATCH	76.3	1118.8934	1118.8966	2.91	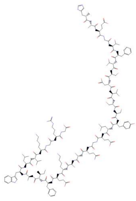	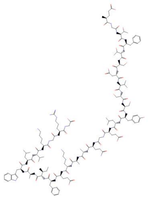	787.4065	787.4003	-7.95
MATCH	76.3	1118.8934	1118.8966	2.91	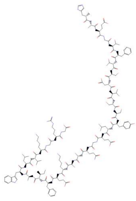	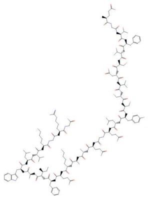	787.4065	787.4003	-7.95
						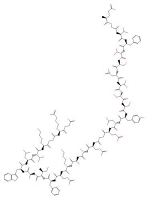	787.4065	787.4003	-7.95
MATCH	31.4	1118.8934	1118.8966	2.91	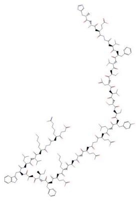	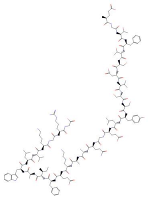	1049.5284	1049.5313	2.79
MATCH	31.4	1118.8934	1118.8966	2.91	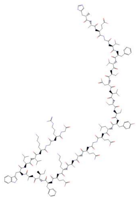	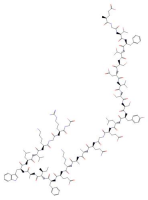	1049.5284	1049.5313	2.79
						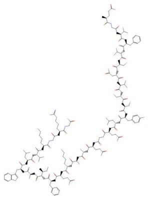	1049.5284	1049.5313	2.79
						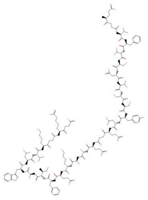	1049.5284	1049.5313	2.79

Metabolite: M3 -208 RT=6.66

Type	score	sub. m/z observed	sub. m/z calculated	sub ppm			met. m/z observed	met. m/z calculated	met. ppm
MATCH	16.1	1677.8363	1677.8413	2.99	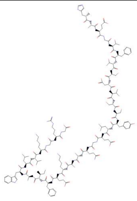	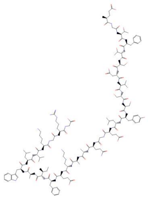	1049.5284	1049.5313	2.79
						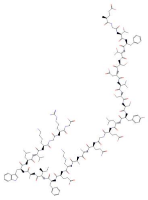	1049.5284	1049.5313	2.79
						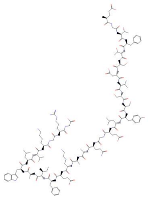	1049.5284	1049.5313	2.79
						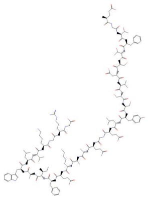	1049.5284	1049.5313	2.79
MATCH	47.7	120.0808	120.0743	-54.1	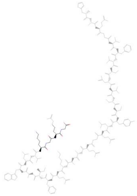	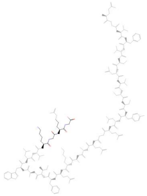	120.0809	120.0743	-54.4
MATCH	8.5	129.1021	129.1022	0.98	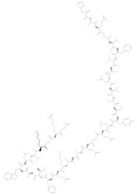	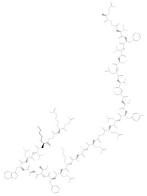	129.1022	129.1022	0.63
MATCH	8.5	129.1021	129.1022	0.98	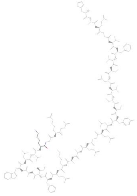	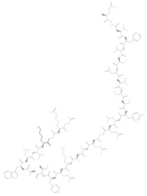	129.1022	129.1022	0.63
MATCH	8.5	129.1021	129.1022	0.98	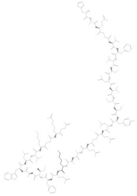	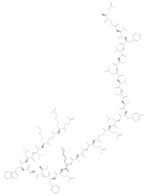	129.1022	129.1022	0.63
MATCH	8.5	129.1021	129.1022	0.98	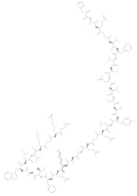	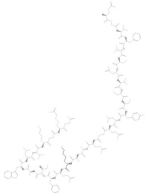	129.1022	129.1022	0.63

Metabolite: M3 -208 RT=6.66

Type	score	sub. m/z observed	sub. m/z calculated	sub ppm			met. m/z observed	met. m/z calculated	met. ppm
MATCH	8.6	136.0755	136.0693	-45.8	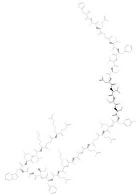	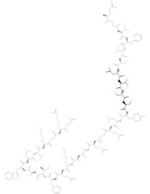	136.0755	136.0693	-45.8
MATCH	8.6	136.0755	136.0693	-45.8	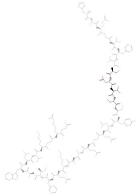	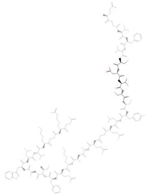	136.0755	136.0693	-45.8
MATCH	8.6	136.0755	136.0706	-35.9	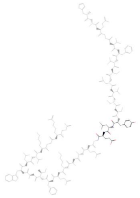	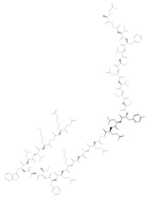	136.0755	136.0706	-36.0
MATCH	8.6	136.0755	136.0706	-35.9	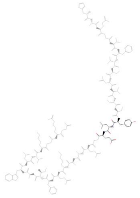	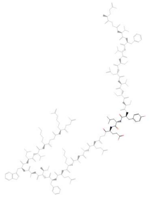	136.0755	136.0706	-36.0
MATCH	8.6	136.0755	136.0737	-13.3	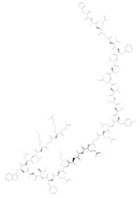	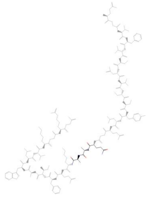	136.0755	136.0737	-13.4
MATCH	8.6	136.0755	136.0737	-13.3	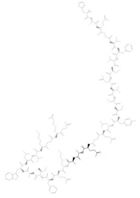	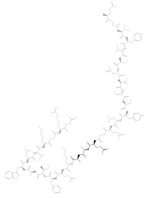	136.0755	136.0737	-13.4
MATCH	13.9	289.1612	289.1619	2.41	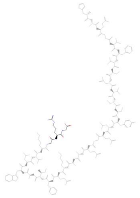	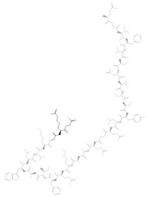	289.1611	289.1619	2.69
MATCH	9.9	395.1662	395.1674	2.91	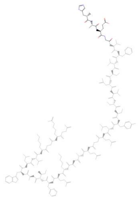	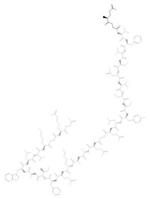	187.0711	187.0713	1.29
MATCH	18.7	417.2558	417.2568	2.43	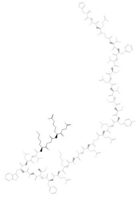	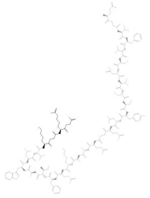	417.2562	417.2568	1.65

Metabolite: M3 -208 RT=6.66

Type	score	sub. m/z observed	sub. m/z calculated	sub ppm			met. m/z observed	met. m/z calculated	met. ppm
MATCH	26.6	478.2034	478.2045	2.25	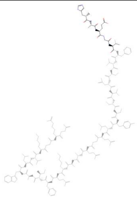	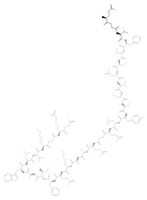	270.1080	270.1084	1.75
MATCH	23.4	496.2138	496.2150	2.53	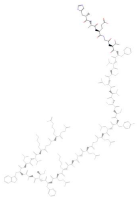	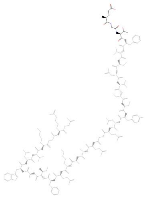	288.1187	288.1190	1.09
MATCH	15.7	516.3241	516.3253	2.30	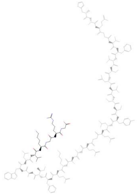	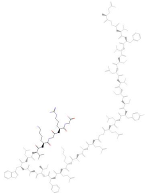	516.3248	516.3253	0.89
MATCH	12.7	625.2723	625.2729	0.91	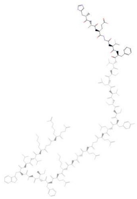	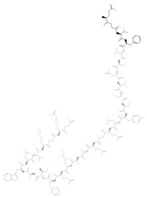	417.1759	417.1769	2.27
MATCH	16.8	629.4084	629.4093	1.50	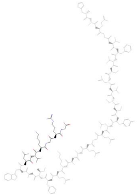	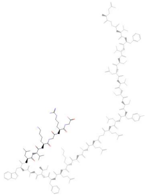	629.4081	629.4093	1.97
MATCH	7.4	643.2815	643.2835	3.02	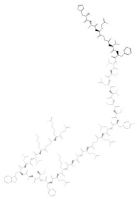	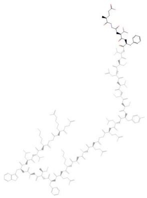	435.1866	435.1874	1.93
MATCH	4.7	702.4102	702.4115	1.83	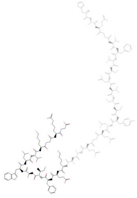	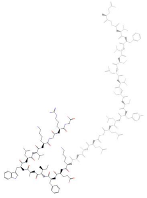	702.4088	702.4115	3.95
MATCH	5.3	737.9282	737.9301	2.52	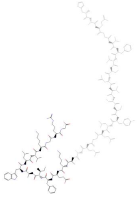	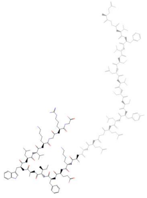	737.9271	737.9301	4.01
MATCH	7.3	773.4469	773.4486	2.29	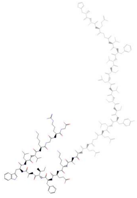	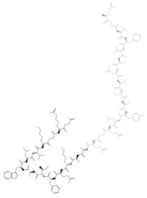	773.4462	773.4486	3.20

Metabolite: M3 -208 RT=6.66

Type	score	sub. m/z observed	sub. m/z calculated	sub ppm			met. m/z observed	met. m/z calculated	met. ppm
MATCH	17.4	815.4866	815.4886	2.53	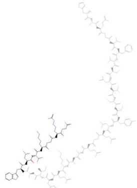	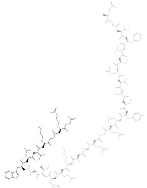	815.4878	815.4886	1.03
MATCH	24.3	865.9855	865.9887	3.67	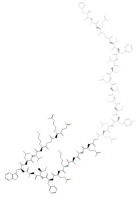	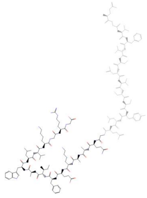	865.9873	865.9887	1.54
MATCH	31.6	886.5234	886.5257	2.63	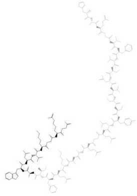	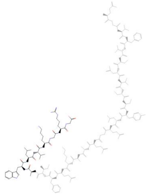	886.5242	886.5257	1.74
MATCH	19.4	930.5064	930.5100	3.85	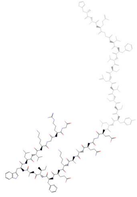	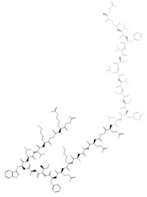	930.5086	930.5100	1.49
MATCH	10.1	946.3875	946.3901	2.79	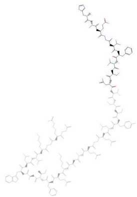	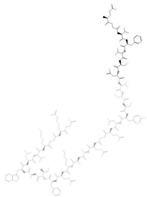	738.2902	738.2941	5.30
MATCH	20.3	987.0513	987.0520	0.67	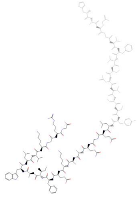	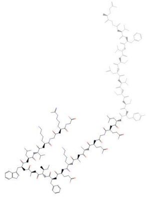	987.0509	987.0520	1.11
MATCH	15.0	999.6076	999.6098	2.24	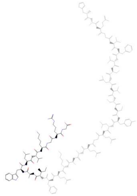	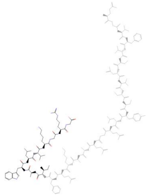	999.6071	999.6098	2.67
MATCH	6.3	1045.4547	1045.4585	3.60	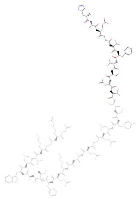	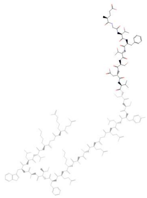	837.3597	837.3625	3.35
MATCH	16.5	1112.8909	1112.8931	1.94	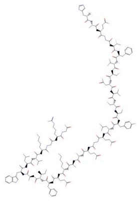	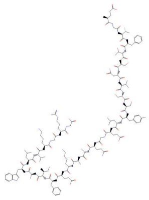	1043.5275	1043.5278	0.29

Metabolite: M3 -208 RT=6.66

Type	score	sub. m/z observed	sub. m/z calculated	sub ppm			met. m/z observed	met. m/z calculated	met. ppm
MATCH	16.5	1112.8909	1112.8931	1.94	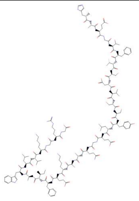	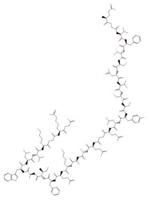	1043.5275	1043.5278	0.29
MATCH	16.5	1112.8909	1112.8931	1.94	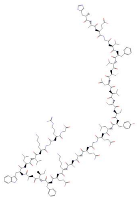	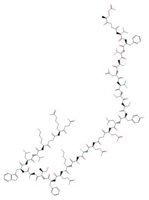	1043.5275	1043.5278	0.29
MATCH	16.5	1112.8909	1112.8931	1.94	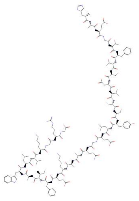	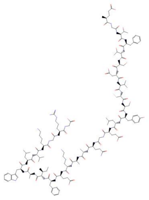	1043.5275	1043.5278	0.29
MATCH	16.5	1112.8909	1112.8931	1.94	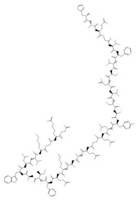	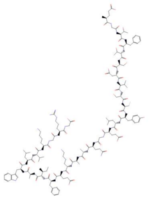	1043.5275	1043.5278	0.29
MATCH	62.2	1118.8951	1118.8966	1.40	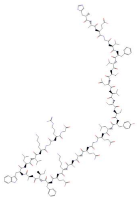	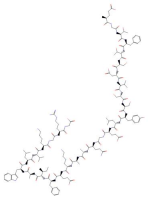	1049.5251	1049.5313	5.85
MATCH	17.4	1146.6769	1146.6782	1.12	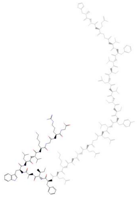	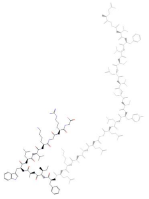	1146.6854	1146.6782	-6.23
MATCH	102.8	1155.6132	1155.6157	2.12	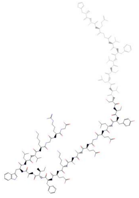	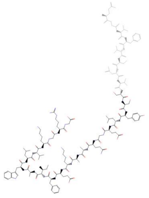	1155.6135	1155.6157	1.89
MATCH	39.1	1205.1474	1205.1499	2.05	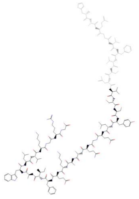	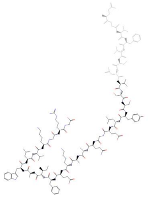	1205.1480	1205.1499	1.53
MATCH	38.7	1262.6607	1262.6634	2.13	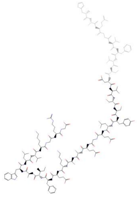	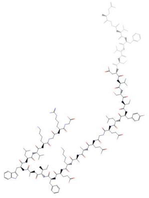	1262.6612	1262.6634	1.74

Metabolite: M3 -208 RT=6.66

Type	score	sub. m/z observed	sub. m/z calculated	sub ppm			met. m/z observed	met. m/z calculated	met. ppm
MATCH	24.0	1275.7182	1275.7208	2.04	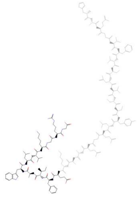	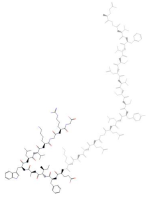	1275.7168	1275.7208	3.15
MATCH	78.0	1306.1754	1306.1794	3.07	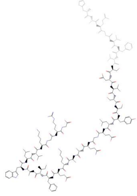	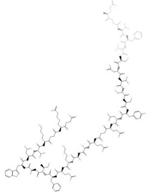	1306.1773	1306.1794	1.55
MATCH	72.9	1356.7014	1356.7032	1.31	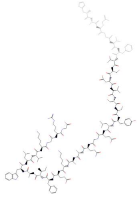	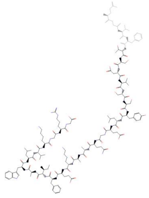	1356.7012	1356.7032	1.49
MATCH	15.9	1403.8138	1403.8158	1.39	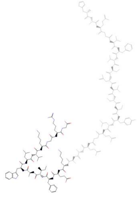	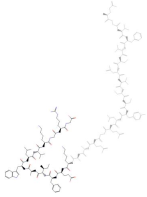	1403.8137	1403.8158	1.49
MATCH	18.9	1430.2360	1430.2374	1.01	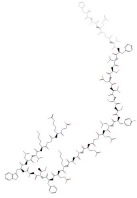	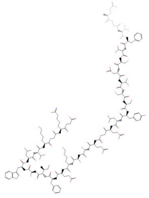	1430.2338	1430.2374	2.57
MATCH	15.0	1474.8479	1474.8529	3.41	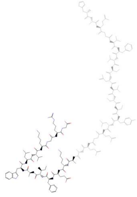	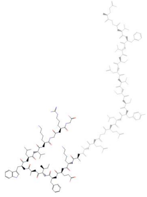	1474.8512	1474.8529	1.15
MATCH	14.6	1545.8874	1545.8900	1.68	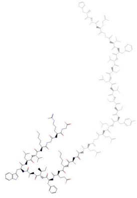	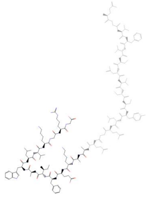	1545.8888	1545.8900	0.79
MATCH	12.5	1730.9696	1730.9700	0.29	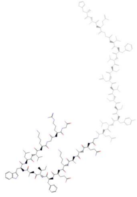	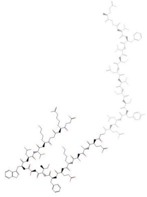	1730.9659	1730.9700	2.37
MATCH	9.0	1860.0044	1860.0126	4.43	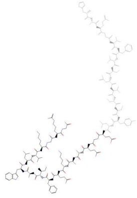	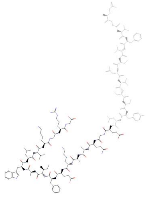	1860.0114	1860.0126	0.69

Metabolite: M3 -208 RT=6.66

Type	score	sub. m/z observed	sub. m/z calculated	sub ppm		met. m/z observed	met. m/z calculated	met. ppm
MISMATCH	-4.6	987.0513	987.0520	0.67	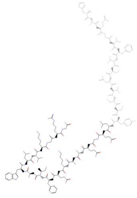	1973.0889	1973.0889	0.00
MISMATCH	-5.0	1155.6132	1155.6157	2.12	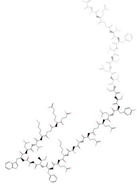	770.7440	770.7440	0.00
MET_MATCH					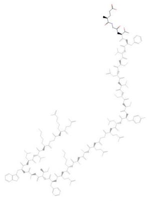	260.1235	260.1241	2.16
MET_MATCH					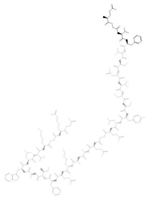	407.1912	407.1925	3.16
MET_MATCH					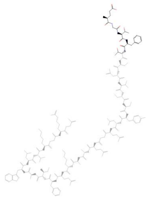	508.2396	508.2402	1.17
MET_MATCH					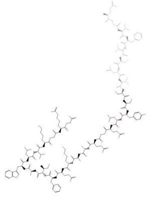	770.7440	770.7462	2.93
MET_MATCH					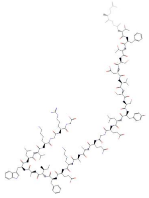	981.8386	981.8344	-4.22
MET_MATCH					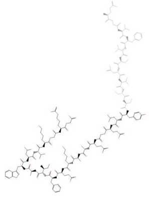	1068.5811	1068.5837	2.43
MET_MATCH					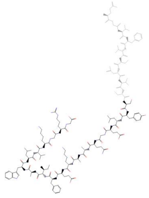	1112.0978	1112.0997	1.66

Metabolite: M3 -208 RT=6.66

Type	score	sub. m/z observed	sub. m/z calculated	sub ppm		met. m/z observed	met. m/z calculated	met. ppm
MET_MATCH					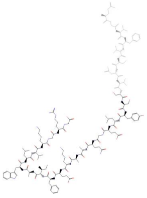	1147.1094	1147.1024	-6.07
MET_MATCH					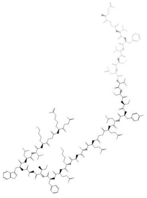	1196.6371	1196.6366	-0.39
MET_MATCH					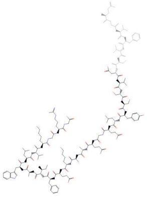	1254.1466	1254.1501	2.78
MET_MATCH					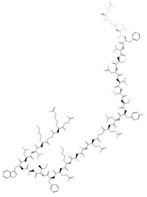	1421.7273	1421.7241	-2.21
MET_MATCH					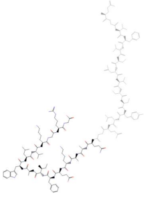	1673.9392	1673.9486	5.60
MET_MATCH					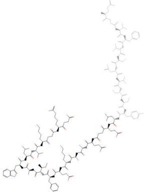	1973.0889	1973.0967	3.97

MS (+) FT

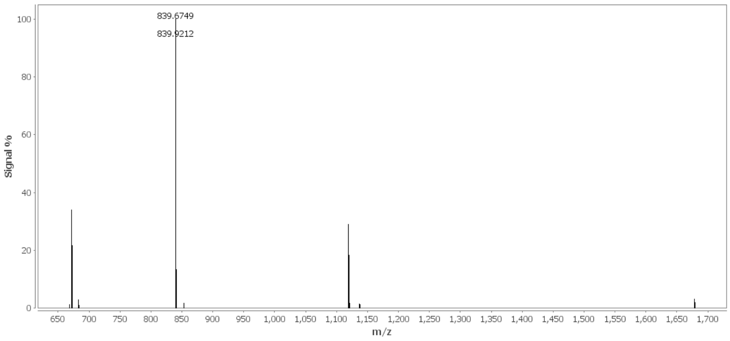

MS (+) FT

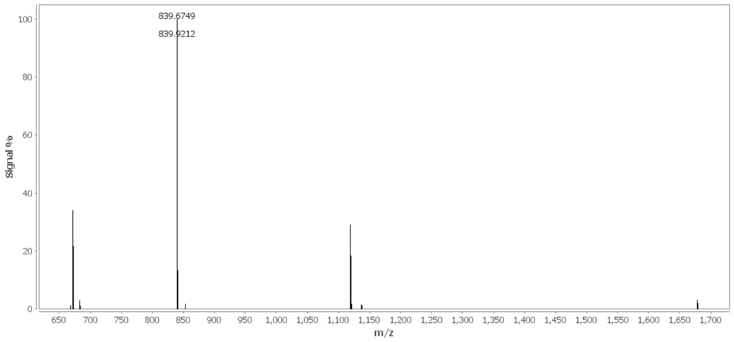

MS2 (+) FT activ = HCD:ce =

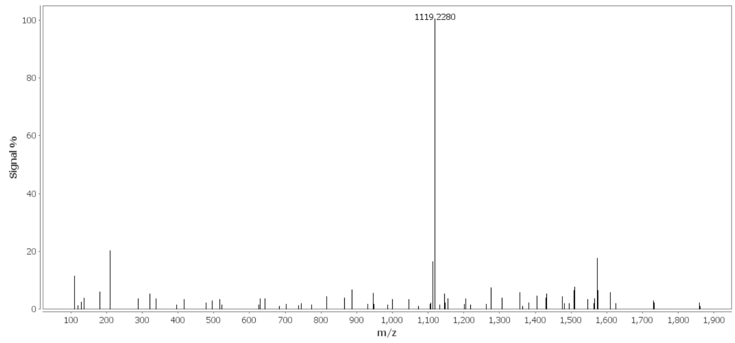

MS2 (+) FT activ = HCD:ce =

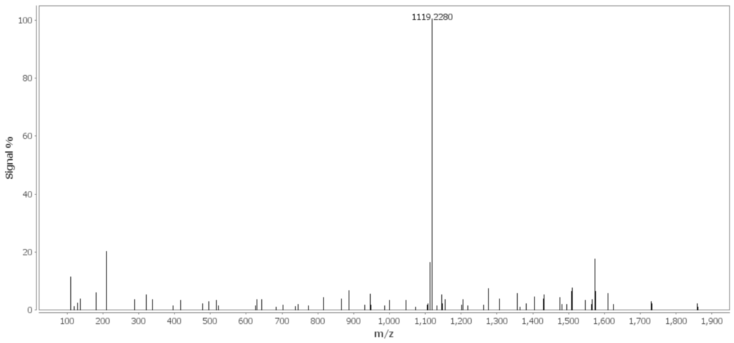

Metabolite: Substrate

Type	score	sub. m/z observed	sub. m/z calculated	sub ppm			met. m/z observed	met. m/z calculated	met. ppm
MATCH	9.0	1860.0044	1860.0126	4.43	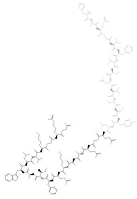	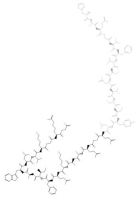	1860.0044	1860.0126	4.43
MATCH	12.5	1730.9696	1730.9700	0.29	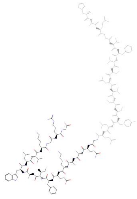	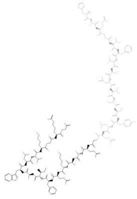	1730.9696	1730.9700	0.29
MATCH	62.1	1677.8363	1677.8413	2.99	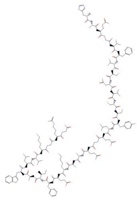	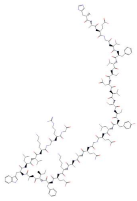	1677.8363	1677.8413	2.99
MATCH	14.6	1545.8874	1545.8900	1.68	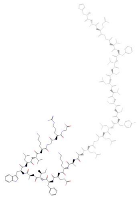	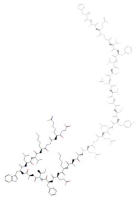	1545.8874	1545.8900	1.68
MATCH	15.0	1474.8479	1474.8529	3.41	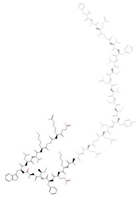	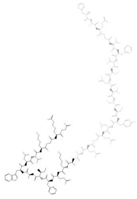	1474.8479	1474.8529	3.41
MATCH	18.9	1430.2360	1430.2374	1.01	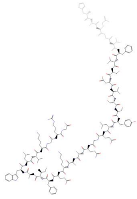	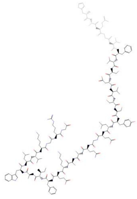	1430.2360	1430.2374	1.01
MATCH	15.9	1403.8138	1403.8158	1.39	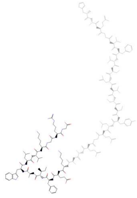	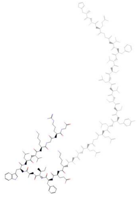	1403.8138	1403.8158	1.39

Metabolite: Substrate

Type	score	sub. m/z observed	sub. m/z calculated	sub ppm			met. m/z observed	met. m/z calculated	met. ppm
MATCH	72.9	1356.7014	1356.7032	1.31	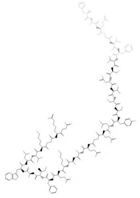	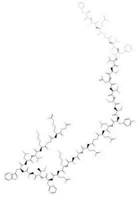	1356.7014	1356.7032	1.31
MATCH	78.0	1306.1754	1306.1794	3.07	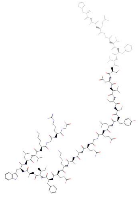	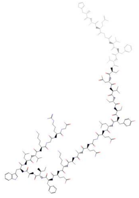	1306.1754	1306.1794	3.07
MATCH	24.0	1275.7182	1275.7208	2.04	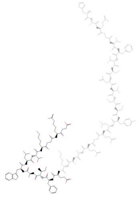	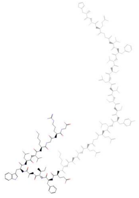	1275.7182	1275.7208	2.04
MATCH	38.7	1262.6607	1262.6634	2.13	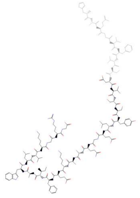	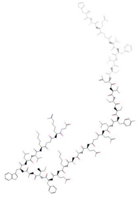	1262.6607	1262.6634	2.13
MATCH	39.1	1205.1474	1205.1499	2.05	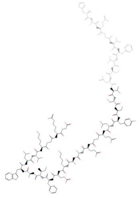	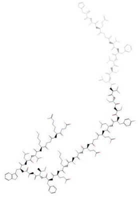	1205.1474	1205.1499	2.05
MISMATCH	102.8	1155.6132	1155.6157	2.12	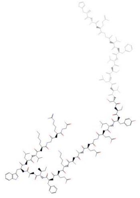	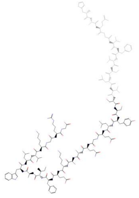	1155.6132	1155.6157	2.12
MATCH	17.4	1146.6769	1146.6782	1.12	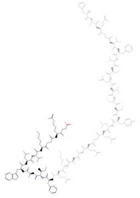	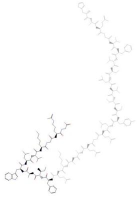	1146.6769	1146.6782	1.12
MATCH	62.2	1118.8951	1118.8966	1.40	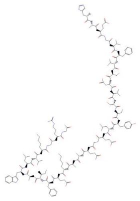	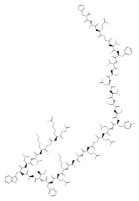	1118.8951	1118.8966	1.40
MATCH	77.5	1118.8934	1118.8966	2.91	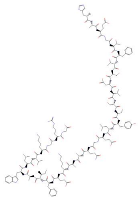	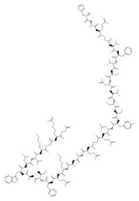	1118.8934	1118.8966	2.91

Metabolite: Substrate

Type	score	sub. m/z observed	sub. m/z calculated	sub ppm			met. m/z observed	met. m/z calculated	met. ppm
MATCH	16.5	1112.8909	1112.8931	1.94	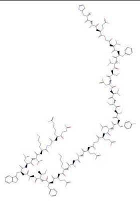	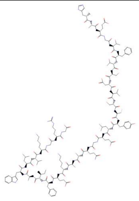	1112.8909	1112.8931	1.94
MATCH	16.5	1112.8909	1112.8931	1.94	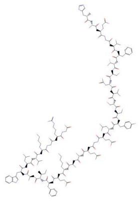	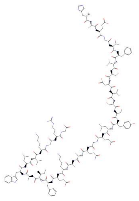	1112.8909	1112.8931	1.94
MATCH	16.5	1112.8909	1112.8931	1.94	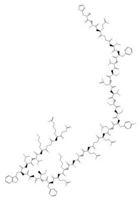	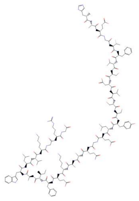	1112.8909	1112.8931	1.94
MATCH	16.5	1112.8909	1112.8931	1.94	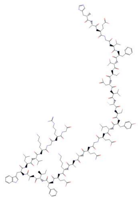	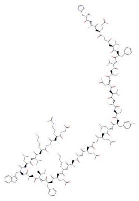	1112.8909	1112.8931	1.94
MATCH	16.5	1112.8909	1112.8931	1.94	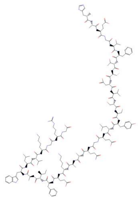	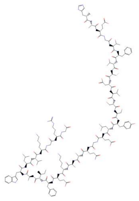	1112.8909	1112.8931	1.94
MATCH	6.3	1045.4547	1045.4585	3.60	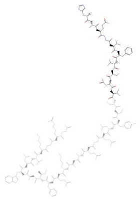	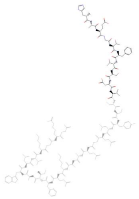	1045.4547	1045.4585	3.60
MATCH	15.0	999.6076	999.6098	2.24	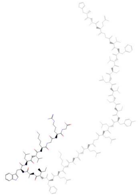	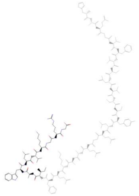	999.6076	999.6098	2.24
MISMATCH	38.3	987.0513	987.0520	0.67	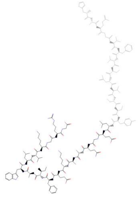	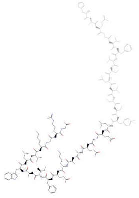	987.0513	987.0520	0.67
MATCH	10.1	946.3875	946.3901	2.79	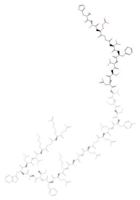	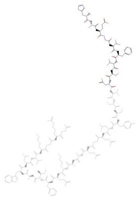	946.3875	946.3901	2.79

Metabolite: Substrate

Type	score	sub. m/z observed	sub. m/z calculated	sub ppm			met. m/z observed	met. m/z calculated	met. ppm
MATCH	54.3	930.5064	930.5100	3.85	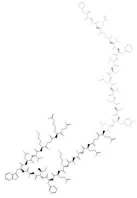	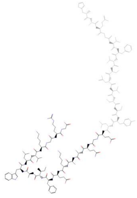	930.5064	930.5100	3.85
MATCH	35.9	886.5234	886.5257	2.63	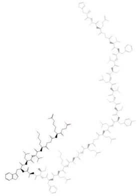	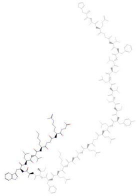	886.5234	886.5257	2.63
MATCH	60.6	865.9855	865.9887	3.67	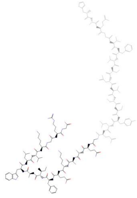	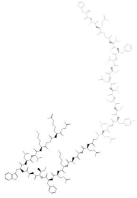	865.9855	865.9887	3.67
MATCH	114.0	839.4275	839.4243	-3.78	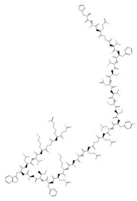	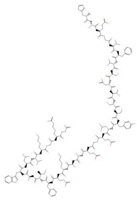	839.4275	839.4243	-3.78
MATCH	34.9	815.4866	815.4886	2.53	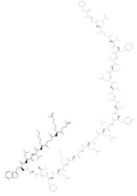	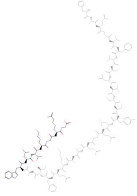	815.4866	815.4886	2.53
MATCH	7.3	773.4469	773.4486	2.29	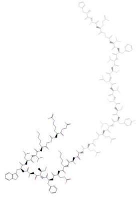	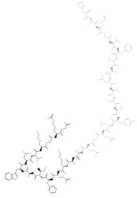	773.4469	773.4486	2.29
MATCH	5.3	737.9282	737.9301	2.52	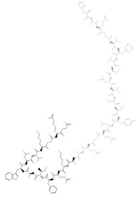	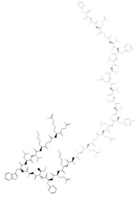	737.9282	737.9301	2.52
MATCH	4.7	702.4102	702.4115	1.83	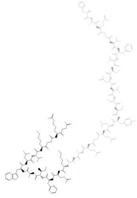	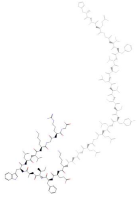	702.4102	702.4115	1.83
MATCH	79.3	671.7420	671.7409	-1.66	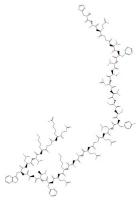	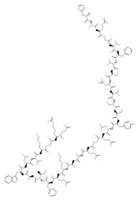	671.7420	671.7409	-1.66

Metabolite: Substrate

Type	score	sub. m/z observed	sub. m/z calculated	sub ppm			met. m/z observed	met. m/z calculated	met. ppm
MATCH	33.2	643.2815	643.2835	3.02	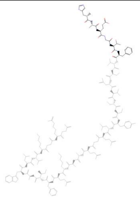	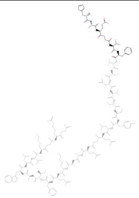	643.2815	643.2835	3.02
MATCH	21.5	629.4084	629.4093	1.50	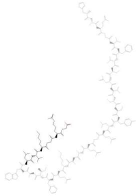	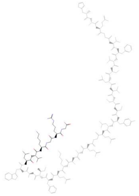	629.4084	629.4093	1.50
MATCH	12.7	625.2723	625.2729	0.91	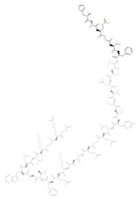	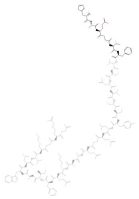	625.2723	625.2729	0.91
MATCH	16.8	516.3241	516.3253	2.30	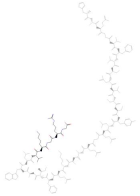	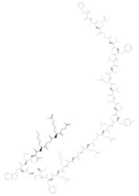	516.3241	516.3253	2.30
MATCH	23.4	496.2138	496.2150	2.53	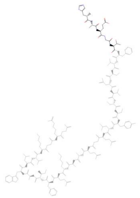	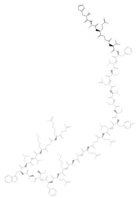	496.2138	496.2150	2.53
MATCH	26.6	478.2034	478.2045	2.25	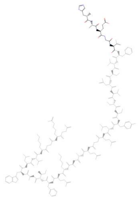	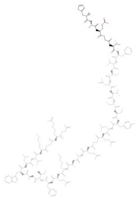	478.2034	478.2045	2.25
MATCH	18.7	417.2558	417.2568	2.43	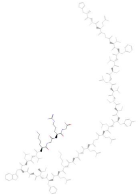	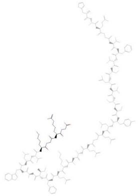	417.2558	417.2568	2.43
MATCH	9.9	395.1662	395.1674	2.91	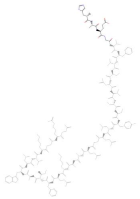	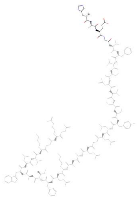	395.1662	395.1674	2.91
MATCH	65.3	338.1447	338.1459	3.46	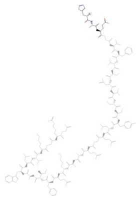	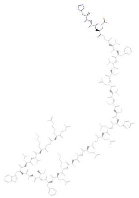	338.1447	338.1459	3.46

Metabolite: Substrate

Type	score	sub. m/z observed	sub. m/z calculated	sub ppm			met. m/z observed	met. m/z calculated	met. ppm
MATCH	13.9	289.1612	289.1619	2.41	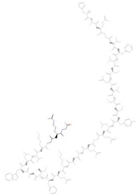	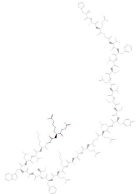	289.1612	289.1619	2.41
MATCH	19.3	136.0755	136.0737	-13.3	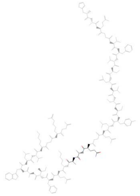	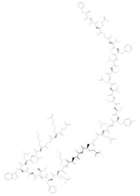	136.0755	136.0737	-13.3
MATCH	19.3	136.0755	136.0693	-45.8	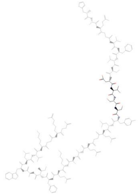	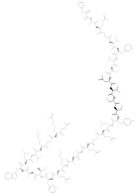	136.0755	136.0693	-45.8
MATCH	19.3	136.0755	136.0706	-35.9	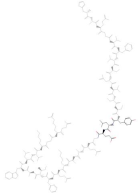	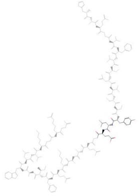	136.0755	136.0706	-35.9
MATCH	19.3	136.0755	136.0693	-45.8	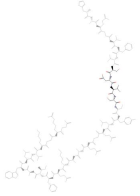	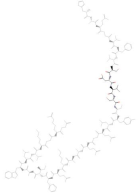	136.0755	136.0693	-45.8
MATCH	19.3	136.0755	136.0737	-13.3	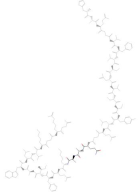	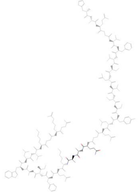	136.0755	136.0737	-13.3
MATCH	19.3	136.0755	136.0706	-35.9	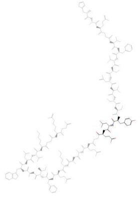	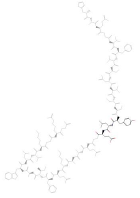	136.0755	136.0706	-35.9
MATCH	8.5	129.1021	129.1022	0.98	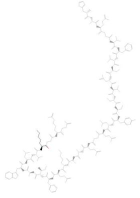	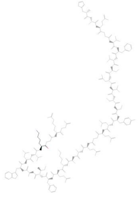	129.1021	129.1022	0.98
MATCH	8.5	129.1021	129.1022	0.98	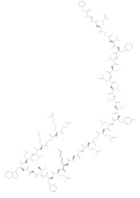	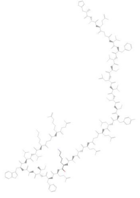	129.1021	129.1022	0.98

Metabolite: Substrate

Type	score	sub. m/z observed	sub. m/z calculated	sub ppm			met. m/z observed	met. m/z calculated	met. ppm
MATCH	8.5	129.1021	129.1022	0.98	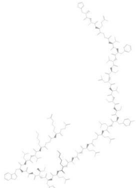	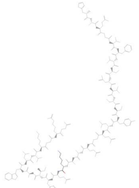	129.1021	129.1022	0.98
MATCH	8.5	129.1021	129.1022	0.98	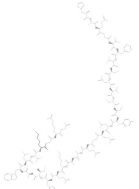	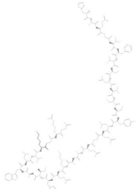	129.1021	129.1022	0.98
MATCH	96.4	120.0808	120.0743	-54.1	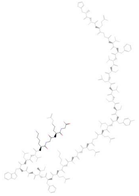	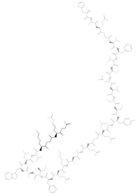	120.0808	120.0743	-54.1

MS (+) FT

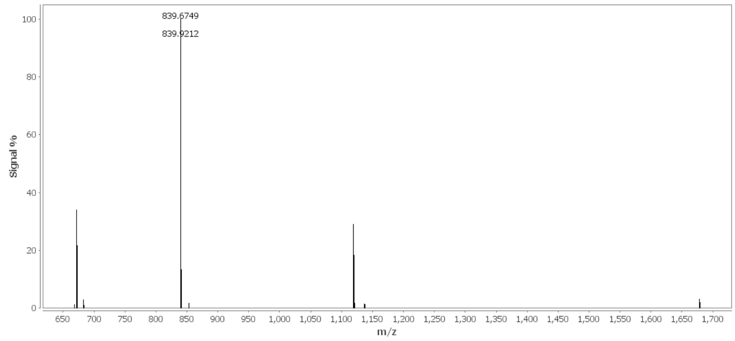

MS (+) FT

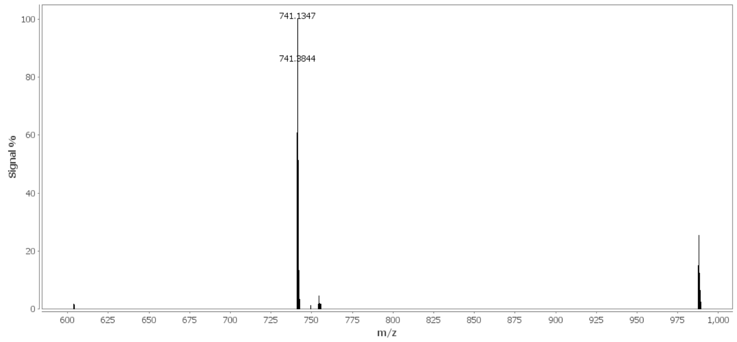

MS2 (+) FT activ = HCD:ce =

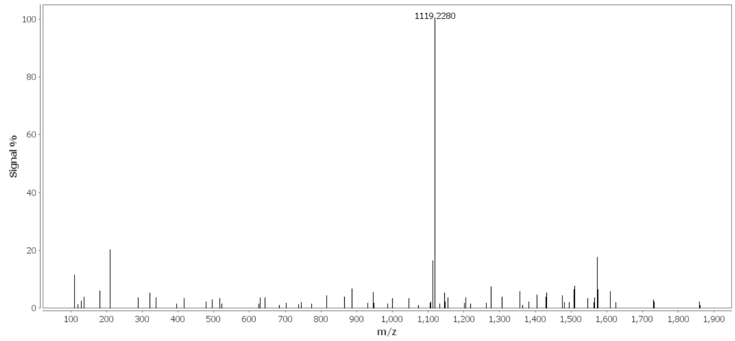

MS2 (+) FT activ = HCD:ce =

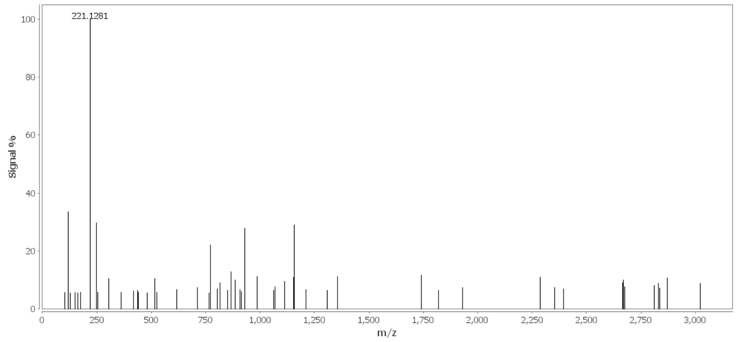

Metabolite: M2 -394 RT=6.58

Type	score	sub. m/z observed	sub. m/z calculated	sub ppm			met. m/z observed	met. m/z calculated	met. ppm
MATCH	79.3	671.7420	671.7409	-1.66	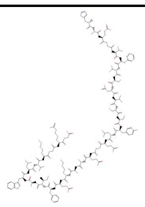	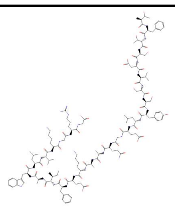	740.8847	740.8843	-0.52

Metabolite: M2 -394 RT=6.58

Type	score	sub. m/z observed	sub. m/z calculated	sub ppm			met. m/z observed	met. m/z calculated	met. ppm
MATCH	79.3	671.7420	671.7409	-1.66	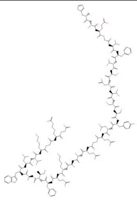	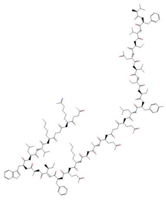	740.8847	740.8843	-0.52
						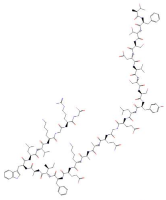	740.8847	740.8843	-0.52
						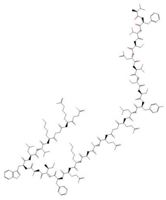	740.8847	740.8843	-0.52
MATCH	33.5	671.7420	671.7409	-1.66	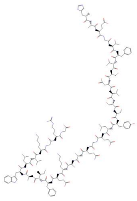	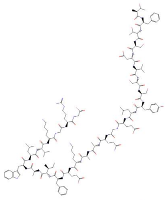	987.5089	987.5099	1.01
MATCH	33.5	671.7420	671.7409	-1.66	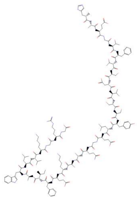	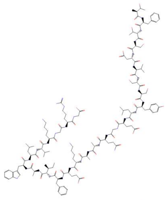	987.5089	987.5099	1.01
						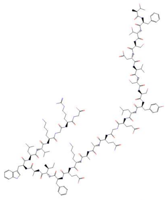	987.5089	987.5099	1.01
						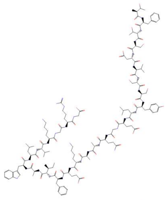	987.5089	987.5099	1.01
MATCH	114.0	839.4275	839.4243	-3.78	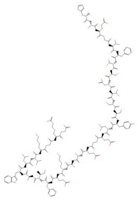	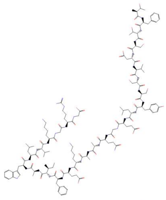	740.8847	740.8843	-0.52
MATCH	114.0	839.4275	839.4243	-3.78	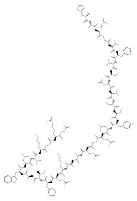	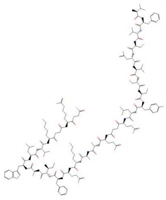	740.8847	740.8843	-0.52

Metabolite: M2 -394 RT=6.58

Type	score	sub. m/z observed	sub. m/z calculated	sub ppm			met. m/z observed	met. m/z calculated	met. ppm
						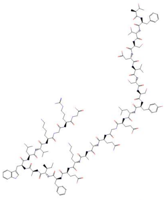	740.8847	740.8843	-0.52
						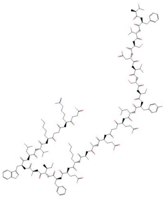	740.8847	740.8843	-0.52
MATCH	68.1	839.4275	839.4243	-3.78	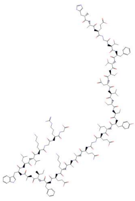	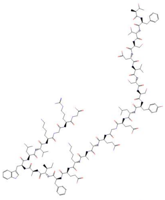	987.5089	987.5099	1.01
MATCH	68.1	839.4275	839.4243	-3.78	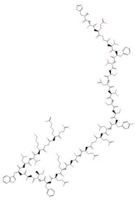	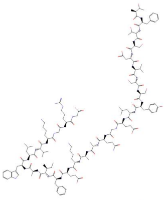	987.5089	987.5099	1.01
						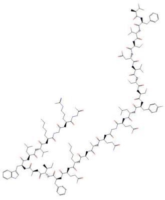	987.5089	987.5099	1.01
						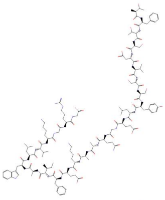	987.5089	987.5099	1.01
MATCH	77.5	1118.8934	1118.8966	2.91	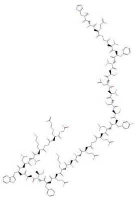	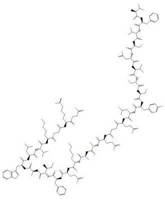	740.8847	740.8843	-0.52
MATCH	77.5	1118.8934	1118.8966	2.91	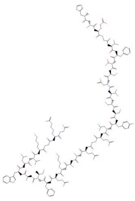	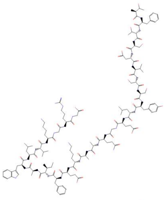	740.8847	740.8843	-0.52
						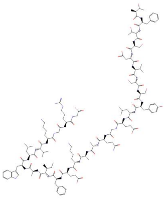	740.8847	740.8843	-0.52

Metabolite: M2 -394 RT=6.58

Type	score	sub. m/z observed	sub. m/z calculated	sub ppm			met. m/z observed	met. m/z calculated	met. ppm
						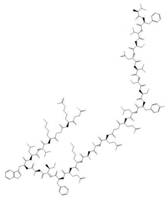	740.8847	740.8843	-0.52
MATCH	31.6	1118.8934	1118.8966	2.91	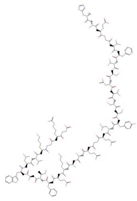	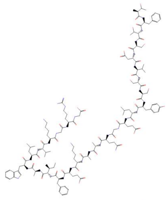	987.5089	987.5099	1.01
MATCH	31.6	1118.8934	1118.8966	2.91	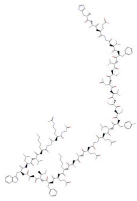	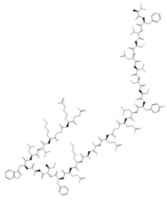	987.5089	987.5099	1.01
						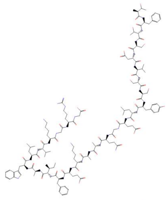	987.5089	987.5099	1.01
						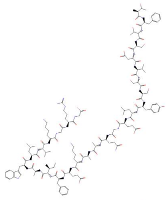	987.5089	987.5099	1.01
MATCH	62.1	1677.8363	1677.8413	2.99	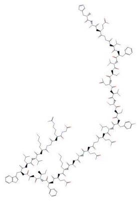	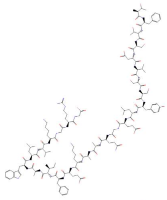	740.8847	740.8843	-0.52
MATCH	62.1	1677.8363	1677.8413	2.99	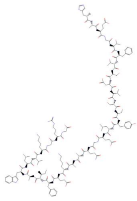	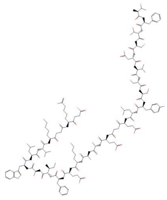	740.8847	740.8843	-0.52
						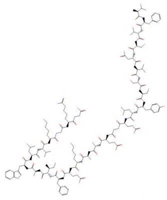	740.8847	740.8843	-0.52
						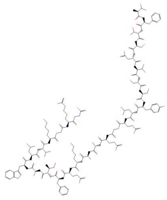	740.8847	740.8843	-0.52

Metabolite: M2 -394 RT=6.58

Type	score	sub. m/z observed	sub. m/z calculated	sub ppm			met. m/z observed	met. m/z calculated	met. ppm
MATCH	16.3	1677.8363	1677.8413	2.99	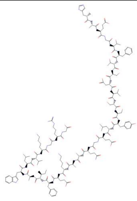	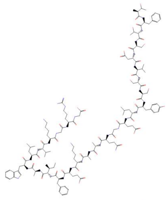	987.5089	987.5099	1.01
MATCH	16.3	1677.8363	1677.8413	2.99	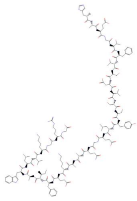	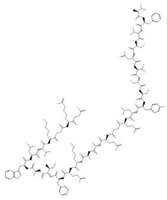	987.5089	987.5099	1.01
						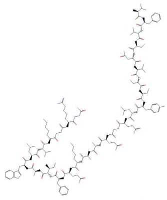	987.5089	987.5099	1.01
						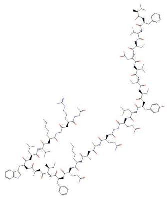	987.5089	987.5099	1.01
MATCH	34.7	120.0808	120.0743	-54.1	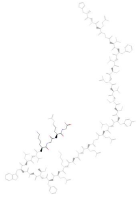	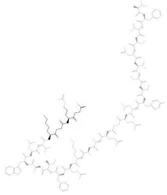	120.0808	120.0743	-53.6
MATCH	9.7	417.2558	417.2568	2.43	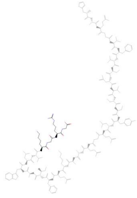	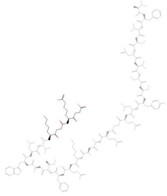	417.2555	417.2568	3.16
MATCH	13.9	516.3241	516.3253	2.30	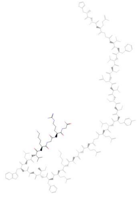	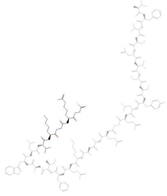	516.3250	516.3253	0.47
MATCH	33.2	643.2815	643.2835	3.02	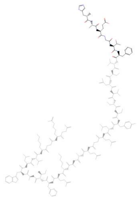	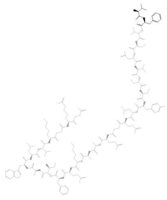	249.1230	249.1234	1.60
MATCH	13.2	815.4866	815.4886	2.53	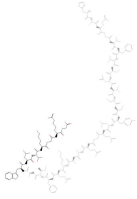	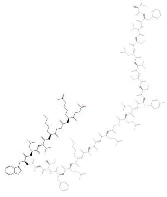	815.4827	815.4886	7.24

Metabolite: M2 -394 RT=6.58

Type	score	sub. m/z observed	sub. m/z calculated	sub ppm			met. m/z observed	met. m/z calculated	met. ppm
MATCH	16.5	865.9855	865.9887	3.67	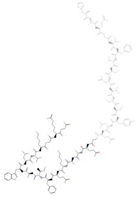	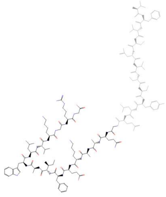	865.9873	865.9887	1.57
MATCH	16.5	886.5234	886.5257	2.63	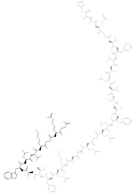	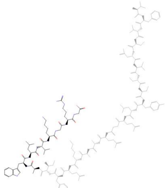	886.5225	886.5257	3.64
MATCH	29.4	930.5064	930.5100	3.85	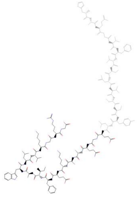	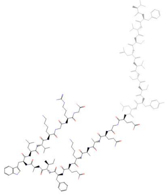	930.5070	930.5100	3.23
MATCH	12.6	987.0513	987.0520	0.67	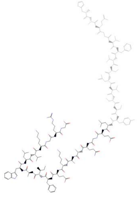	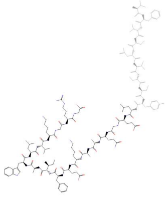	987.0507	987.0520	1.29
MATCH	14.5	1155.6132	1155.6157	2.12	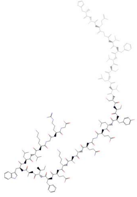	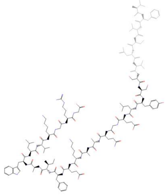	1155.6052	1155.6157	9.05
MISMATCH	-25.5	1155.6132	1155.6157	2.12	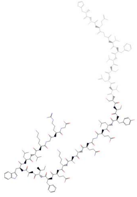		770.7456	770.7456	0.00
MISMATCH	-10.4	1205.1474	1205.1499	2.05	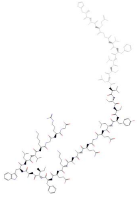		803.7579	803.7579	0.00
MET_MATCH						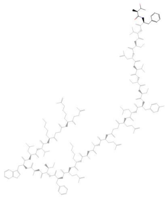	221.1281	221.1285	1.46
MET_MATCH						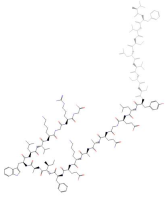	712.7247	712.7249	0.27

Metabolite: M2 -394 RT=6.58

Type	score	sub. m/z observed	sub. m/z calculated	sub ppm			met. m/z observed	met. m/z calculated	met. ppm
MET_MATCH						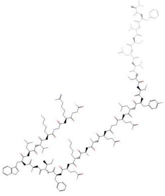	770.7456	770.7462	0.79
MET_MATCH						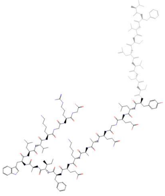	1068.5793	1068.5837	4.03
MET_MATCH						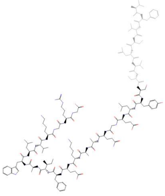	1112.1011	1112.0997	-1.26

MS (+) FT

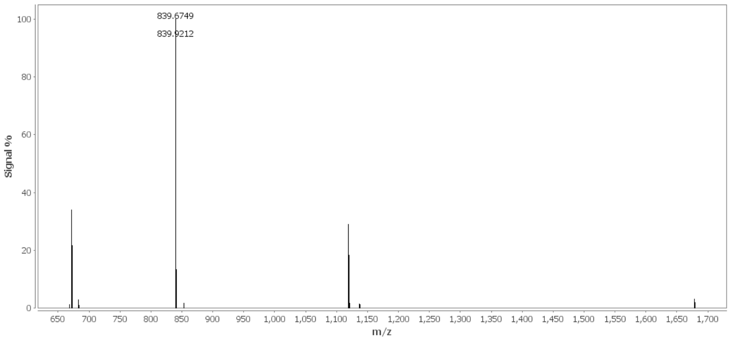

MS (+) FT

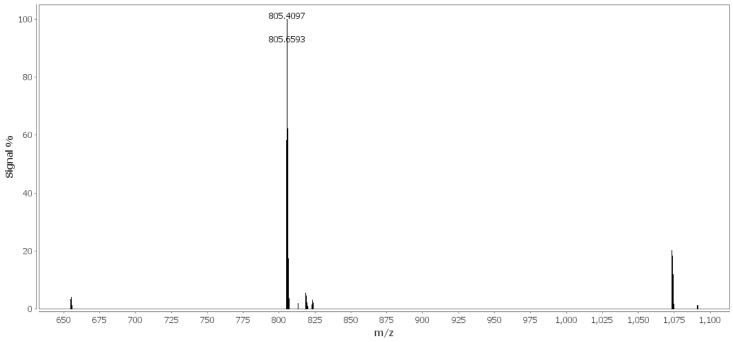

MS2 (+) FT activ = HCD:ce =

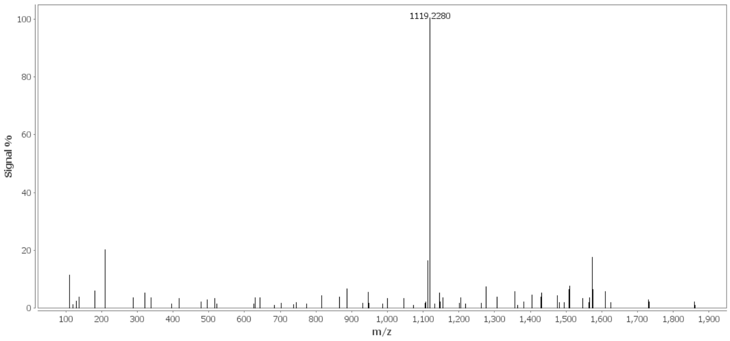

MS2 (+) FT activ = HCD:ce =

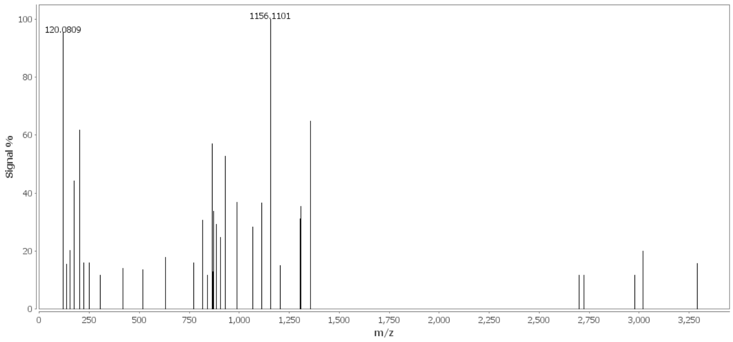

Metabolite: M1 -137 RT=6.53

Type	score	sub. m/z observed	sub. m/z calculated	sub ppm			met. m/z observed	met. m/z calculated	met. ppm
MATCH	76.6	671.7420	671.7409	-1.66	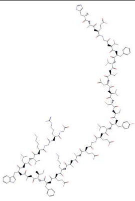	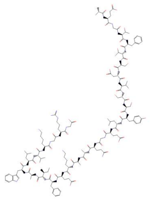	805.1598	805.1596	-0.26

Metabolite: M1 -137 RT=6.53

Type	score	sub. m/z observed	sub. m/z calculated	sub ppm			met. m/z observed	met. m/z calculated	met. ppm
MATCH	76.6	671.7420	671.7409	-1.66	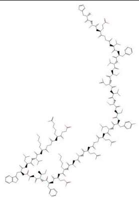	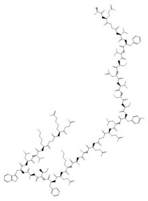	805.1598	805.1596	-0.26
						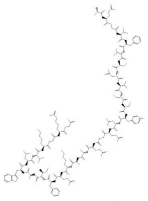	805.1598	805.1596	-0.26
						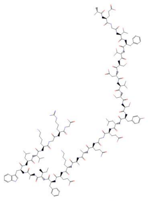	805.1598	805.1596	-0.26
MATCH	29.6	671.7420	671.7409	-1.66	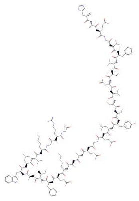	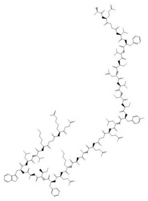	1073.2084	1073.2103	1.83
MATCH	29.6	671.7420	671.7409	-1.66	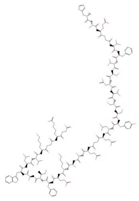	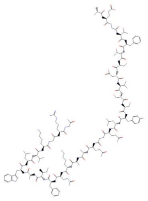	1073.2084	1073.2103	1.83
						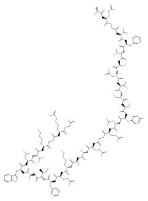	1073.2084	1073.2103	1.83
						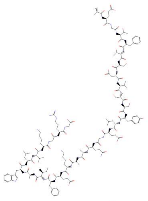	1073.2084	1073.2103	1.83
MATCH	111.2	839.4275	839.4243	-3.78	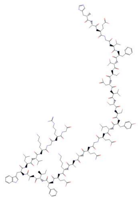	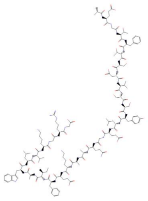	805.1598	805.1596	-0.26
MATCH	111.2	839.4275	839.4243	-3.78	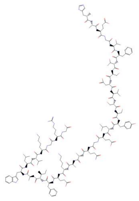	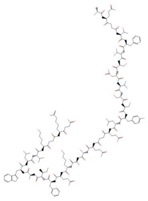	805.1598	805.1596	-0.26

Metabolite: M1 -137 RT=6.53

Type	score	sub. m/z observed	sub. m/z calculated	sub ppm			met. m/z observed	met. m/z calculated	met. ppm
						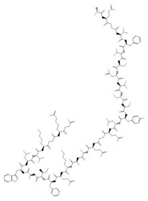	805.1598	805.1596	-0.26
						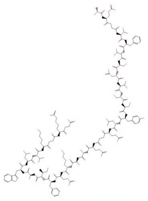	805.1598	805.1596	-0.26
MATCH	64.2	839.4275	839.4243	-3.78	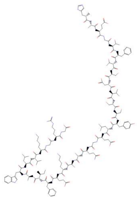	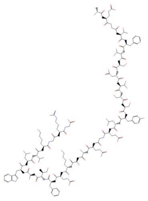	1073.2084	1073.2103	1.83
MATCH	64.2	839.4275	839.4243	-3.78	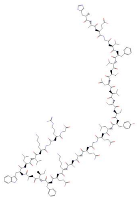	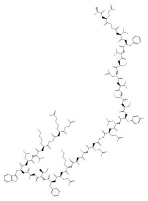	1073.2084	1073.2103	1.83
						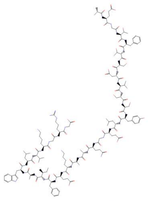	1073.2084	1073.2103	1.83
						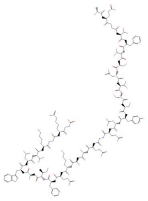	1073.2084	1073.2103	1.83
MATCH	74.7	1118.8934	1118.8966	2.91	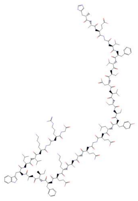	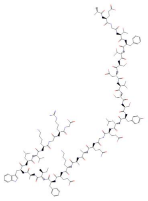	805.1598	805.1596	-0.26
MATCH	74.7	1118.8934	1118.8966	2.91	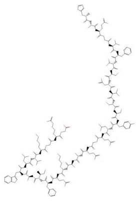	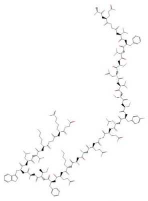	805.1598	805.1596	-0.26
						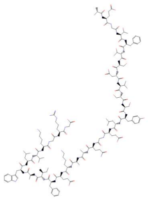	805.1598	805.1596	-0.26

Metabolite: M1 -137 RT=6.53

Type	score	sub. m/z observed	sub. m/z calculated	sub ppm			met. m/z observed	met. m/z calculated	met. ppm
						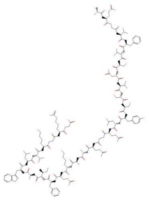	805.1598	805.1596	-0.26
MATCH	27.7	1118.8934	1118.8966	2.91	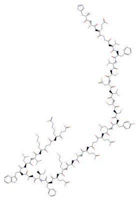	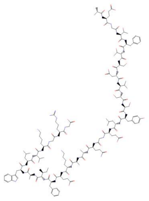	1073.2084	1073.2103	1.83
MATCH	27.7	1118.8934	1118.8966	2.91	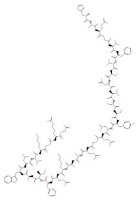	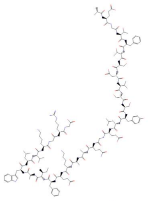	1073.2084	1073.2103	1.83
						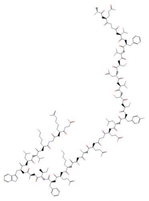	1073.2084	1073.2103	1.83
						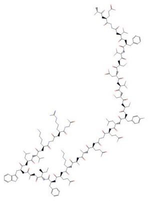	1073.2084	1073.2103	1.83
MATCH	59.4	1677.8363	1677.8413	2.99	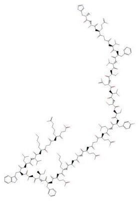	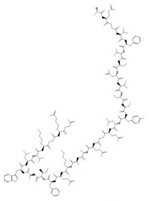	805.1598	805.1596	-0.26
MATCH	59.4	1677.8363	1677.8413	2.99	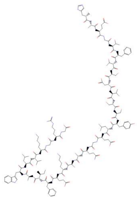	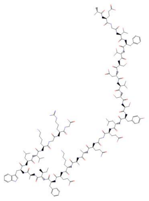	805.1598	805.1596	-0.26
						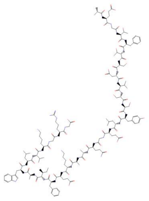	805.1598	805.1596	-0.26
						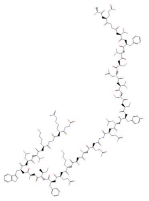	805.1598	805.1596	-0.26

Metabolite: M1 -137 RT=6.53

Type	score	sub. m/z observed	sub. m/z calculated	sub ppm			met. m/z observed	met. m/z calculated	met. ppm
MATCH	12.4	1677.8363	1677.8413	2.99	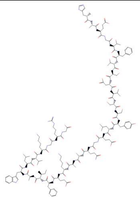	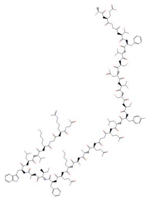	1073.2084	1073.2103	1.83
MATCH	12.4	1677.8363	1677.8413	2.99	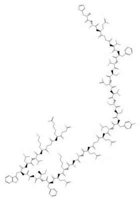	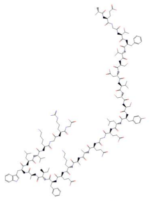	1073.2084	1073.2103	1.83
						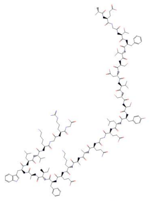	1073.2084	1073.2103	1.83
						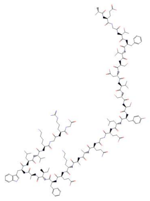	1073.2084	1073.2103	1.83
MATCH	96.4	120.0808	120.0743	-54.1	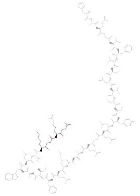	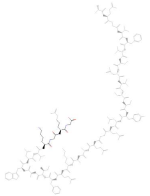	120.0809	120.0743	-54.1
MATCH	19.3	136.0755	136.0693	-45.8	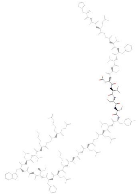	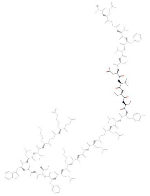	136.0755	136.0693	-45.6
MATCH	19.3	136.0755	136.0693	-45.8	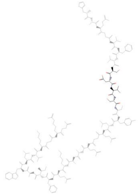	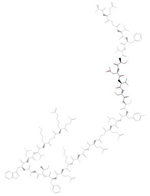	136.0755	136.0693	-45.6
MATCH	19.3	136.0755	136.0706	-35.9	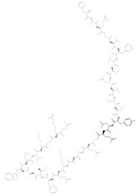	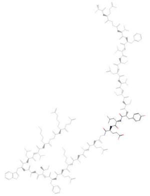	136.0755	136.0706	-35.7
MATCH	19.3	136.0755	136.0706	-35.9	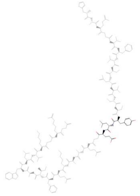	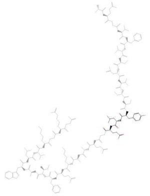	136.0755	136.0706	-35.7

Metabolite: M1 -137 RT=6.53

Type	score	sub. m/z observed	sub. m/z calculated	sub ppm			met. m/z observed	met. m/z calculated	met. ppm
MATCH	19.3	136.0755	136.0737	-13.3	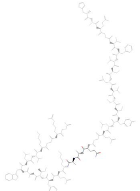	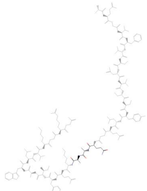	136.0755	136.0737	-13.1
MATCH	19.3	136.0755	136.0737	-13.3	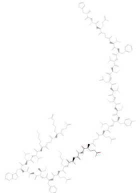	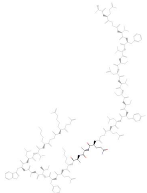	136.0755	136.0737	-13.1
MATCH	65.3	338.1447	338.1459	3.46	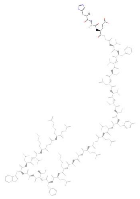	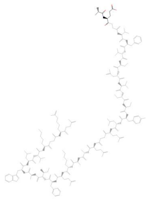	201.0867	201.0870	1.33
MATCH	17.4	417.2558	417.2568	2.43	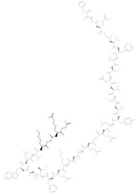	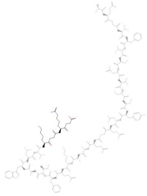	417.2554	417.2568	3.45
MATCH	16.8	516.3241	516.3253	2.30	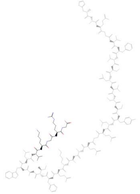	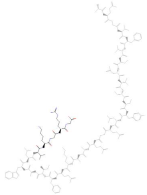	516.3235	516.3253	3.43
MATCH	21.5	629.4084	629.4093	1.50	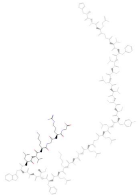	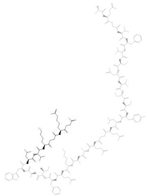	629.4085	629.4093	1.29
MATCH	34.9	815.4866	815.4886	2.53	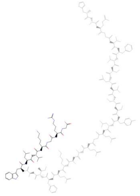	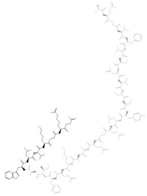	815.4888	815.4886	-0.24
MATCH	60.6	865.9855	865.9887	3.67	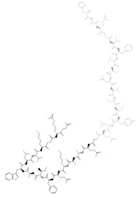	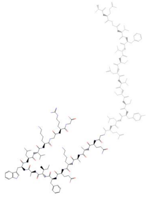	865.9846	865.9887	4.64
MATCH	35.9	886.5234	886.5257	2.63	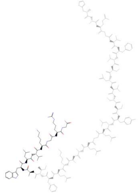	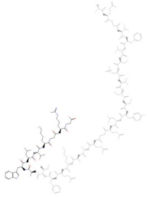	886.5246	886.5257	1.30

Metabolite: M1 -137 RT=6.53

Type	score	sub. m/z observed	sub. m/z calculated	sub ppm			met. m/z observed	met. m/z calculated	met. ppm
MATCH	54.3	930.5064	930.5100	3.85	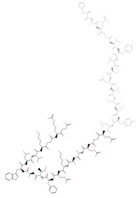	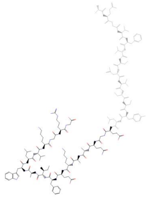	930.5085	930.5100	1.59
MATCH	38.3	987.0513	987.0520	0.67	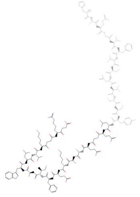	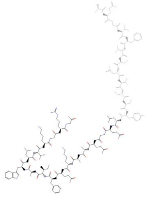	987.0441	987.0520	7.97
MATCH	74.9	1155.6132	1155.6157	2.12	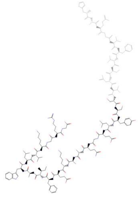	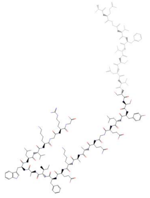	1155.6151	1155.6157	0.47
MATCH	18.5	1205.1474	1205.1499	2.05	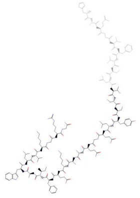	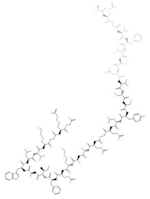	1205.1415	1205.1499	6.98
MATCH	35.0	1306.1754	1306.1794	3.07	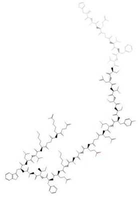	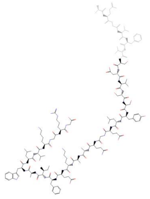	1306.1743	1306.1794	3.91
MATCH	24.0	1356.7014	1356.7032	1.31	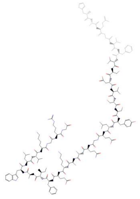	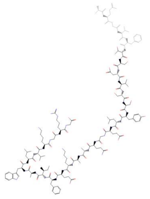	1356.7000	1356.7032	2.41
MISMATCH	-13.0	1262.6607	1262.6634	2.13	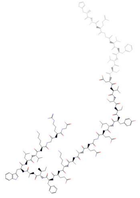		842.1098	842.1098	0.00
MISMATCH	-36.1	1306.1754	1306.1794	3.07	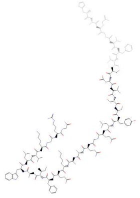		871.1157	871.1157	0.00
MISMATCH	-21.4	1356.7014	1356.7032	1.31	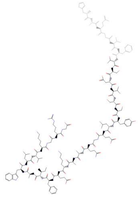		904.8021	904.8021	0.00

Metabolite: M1 -137 RT=6.53

Type	score	sub. m/z observed	sub. m/z calculated	sub ppm		met. m/z observed	met. m/z calculated	met. ppm
MET_MATCH					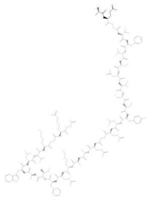	173.0915	173.0921	3.06
MET_MATCH					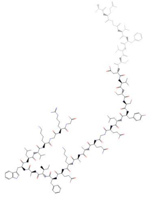	842.1098	842.1113	1.82
MET_MATCH					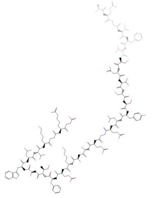	871.1157	871.1220	7.22
MET_MATCH					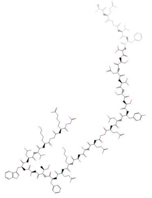	904.8021	904.8046	2.76
MET_MATCH					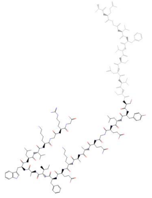	1112.0944	1112.0997	4.74
