# Supplementary material for: Software-aided approach to investigate peptide structure and metabolic susceptibility of amide bonds in peptide drugs based on high resolution mass spectrometry
Source: PLoS One. 2017 Nov 1;12(11):e0186461. doi: 10.1371/journal.pone.0186461 (PMC5665424; doi:10.1371/journal.pone.0186461)
Supplement: S1 File — (ZIP) [file pone.0186461.s007.zip › SFiles/S46_File.pdf]

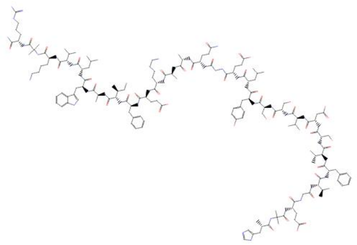

Taspoglutide

Property name	Property value
Time	0min, 2min, 4min, 24min
Instrument	ddMS2
Matrix	DPP4

Chromatograms

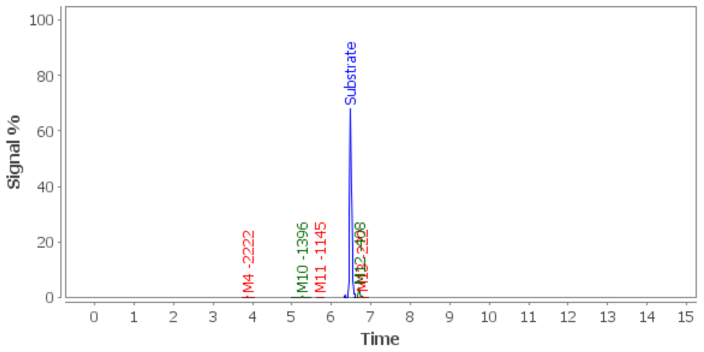

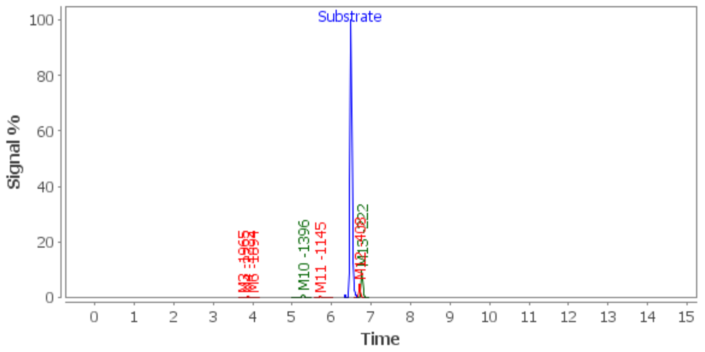

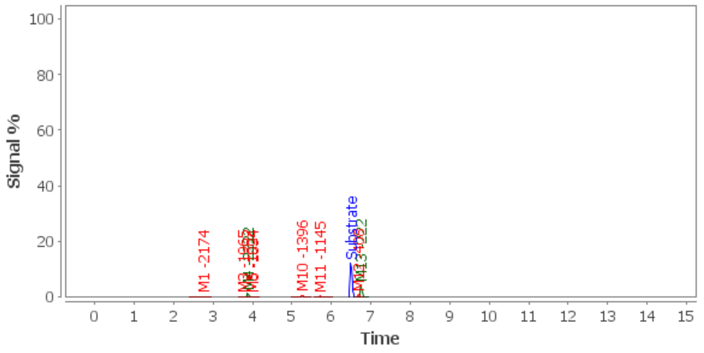

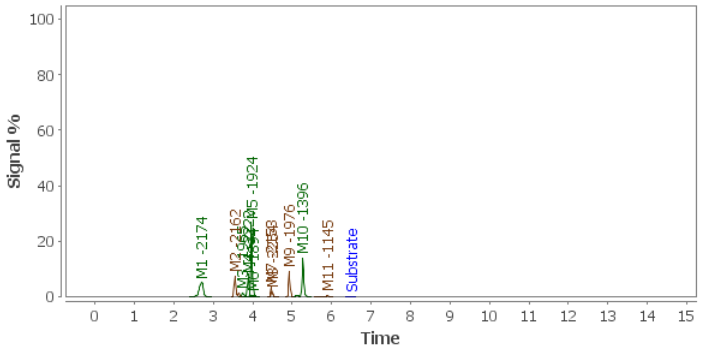

Custom Charts

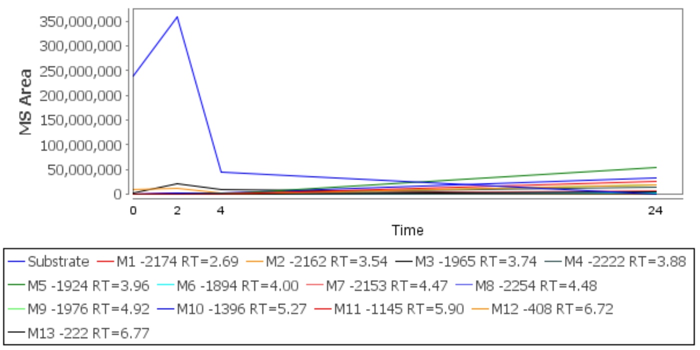

Fragmentation

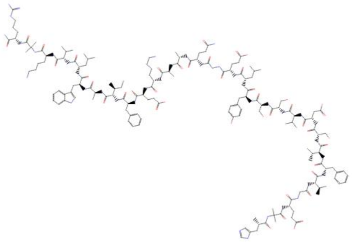

Taspoglutide

MS (+) FT

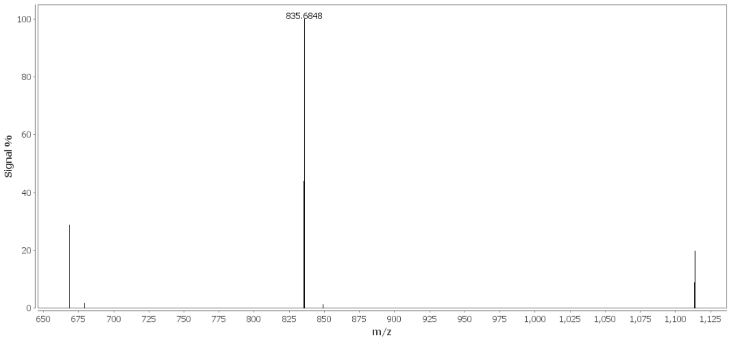

MS (+) FT

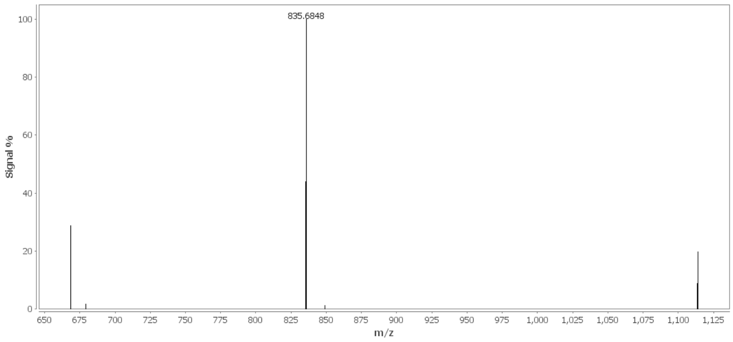

MS2 (+) FT activ = HCD:ce =

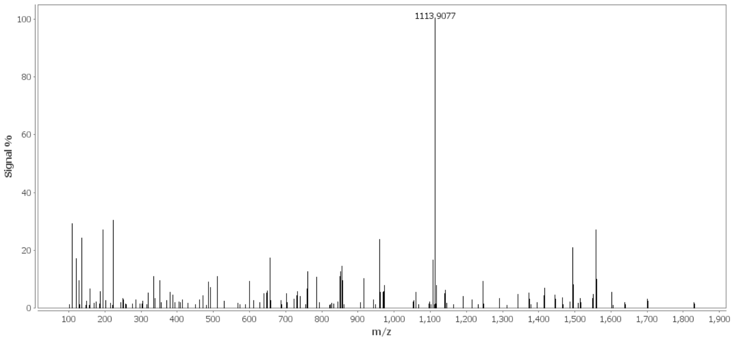

MS2 (+) FT activ = HCD:ce =

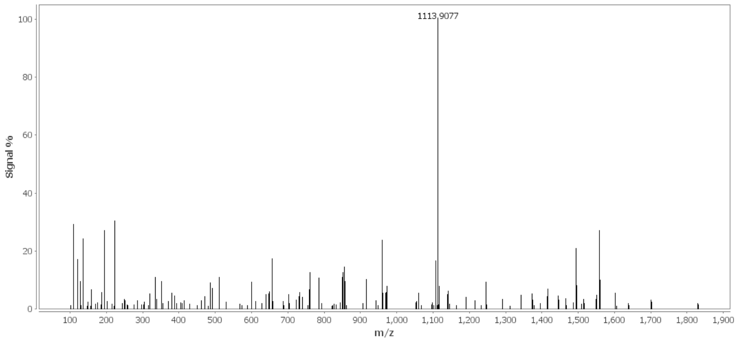

Metabolite: Substrate

Type	score	sub. m/z observed	sub. m/z calculated	sub ppm		met. m/z observed	met. m/z calculated	met. ppm
MATCH	103.0	1700.9942	1700.9959	0.96	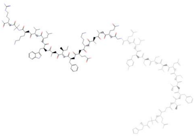 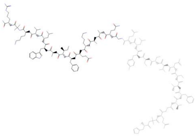	1700.9942	1700.9959	0.96
MATCH	60.5	1638.7199	1638.7282	5.06	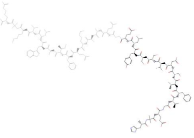 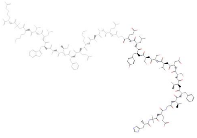	1638.7199	1638.7282	5.06
MATCH	10.6	1601.3333	1601.3326	-0.45	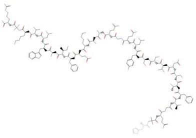 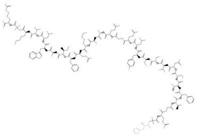	1601.3333	1601.3326	-0.45

Metabolite: Substrate

Type	score	sub. m/z observed	sub. m/z calculated	sub ppm			met. m/z observed	met. m/z calculated	met. ppm
MATCH	18.8	1558.8038	1558.8062	1.56	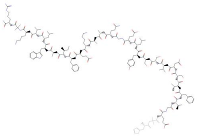	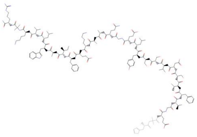	1558.8038	1558.8062	1.56
MATCH	8.3	1549.7979	1549.8009	1.95	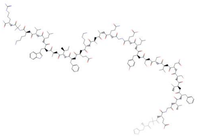	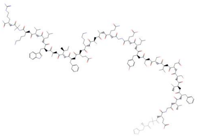	1549.7979	1549.8009	1.95
MATCH	8.3	1549.7979	1549.8009	1.95	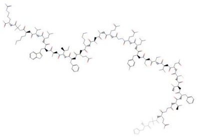	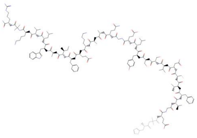	1549.7979	1549.8009	1.95
MATCH	8.3	1549.7979	1549.8009	1.95	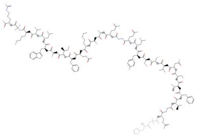	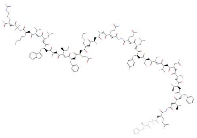	1549.7979	1549.8009	1.95
MATCH	8.3	1549.7979	1549.8009	1.95	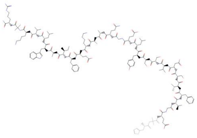	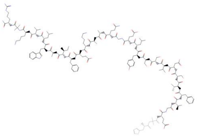	1549.7979	1549.8009	1.95
MISMATCH	10.2	1515.9113	1515.9158	2.97	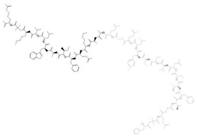	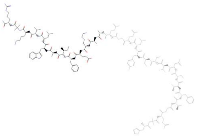	1515.9113	1515.9158	2.97
MISMATCH	-65.8	1509.6797	1509.6856	3.95	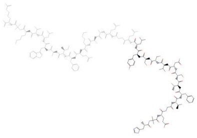	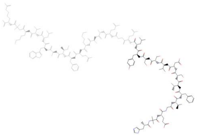	1509.6797	1509.6856	3.95
MISMATCH	12.4	1444.8767	1444.8787	1.42	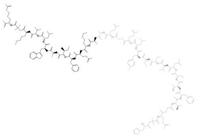	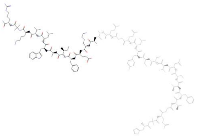	1444.8767	1444.8787	1.42
MISMATCH	-6.5	1415.2481	1415.2503	1.57	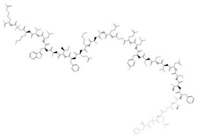	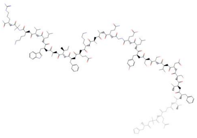	1415.2481	1415.2503	1.57

Metabolite: Substrate

Type	score	sub. m/z observed	sub. m/z calculated	sub ppm			met. m/z observed	met. m/z calculated	met. ppm
MISMATCH	-14.8	1396.5975	1396.6016	2.89	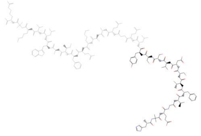	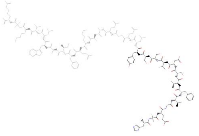	1396.5975	1396.6016	2.89
MATCH	13.0	1378.5812	1378.5910	7.09	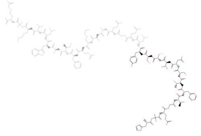	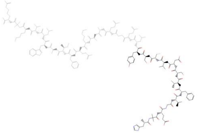	1378.5812	1378.5910	7.09
MATCH	13.0	1378.5812	1378.5910	7.09	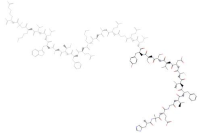	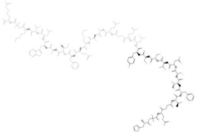	1378.5812	1378.5910	7.09
MATCH	13.0	1378.5812	1378.5910	7.09	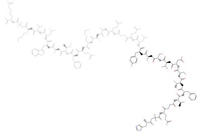	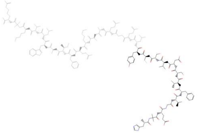	1378.5812	1378.5910	7.09
MATCH	16.8	1373.8392	1373.8416	1.75	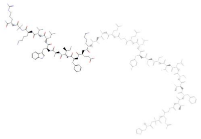	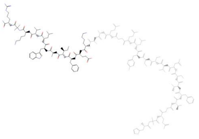	1373.8392	1373.8416	1.75
MISMATCH	22.7	1341.7122	1341.7161	2.93	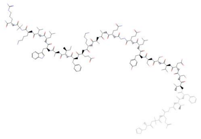	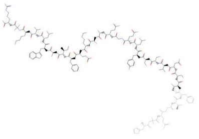	1341.7122	1341.7161	2.93
MISMATCH	24.8	1291.1879	1291.1923	3.43	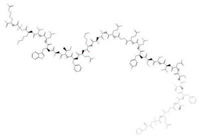	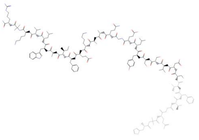	1291.1879	1291.1923	3.43
MISMATCH	-9.2	1247.6634	1247.6686	4.21	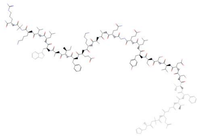	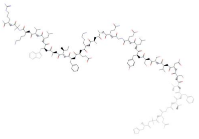	1247.6634	1247.6686	4.21
MISMATCH	25.0	1245.7435	1245.7466	2.52	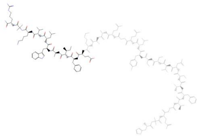	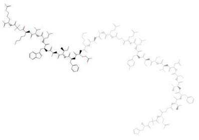	1245.7435	1245.7466	2.52

Metabolite: Substrate

Type	score	sub. m/z observed	sub. m/z calculated	sub ppm			met. m/z observed	met. m/z calculated	met. ppm
MATCH	5.7	1233.5312	1233.5382	5.71	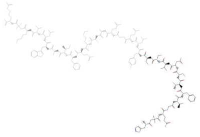	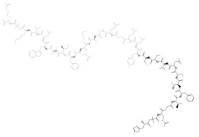	1233.5312	1233.5382	5.71
MATCH	12.2	1215.5245	1215.5277	2.59	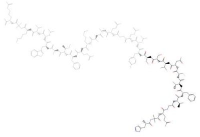	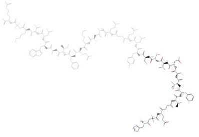	1215.5245	1215.5277	2.59
MATCH	12.2	1215.5245	1215.5277	2.59	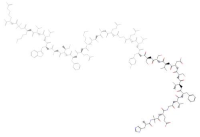	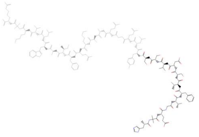	1215.5245	1215.5277	2.59
MATCH	12.2	1215.5245	1215.5277	2.59	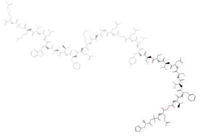	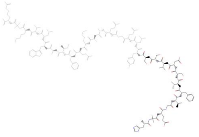	1215.5245	1215.5277	2.59
MATCH	12.2	1215.5245	1215.5277	2.59	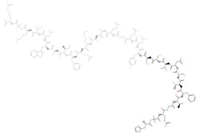	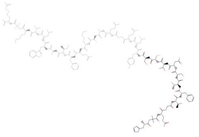	1215.5245	1215.5277	2.59
MATCH	12.2	1215.5245	1215.5277	2.59	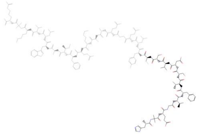	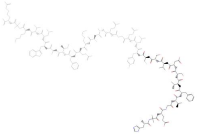	1215.5245	1215.5277	2.59
MISMATCH	22.8	1190.1592	1190.1628	3.04	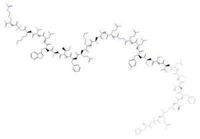	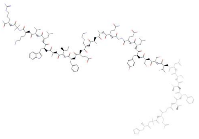	1190.1592	1190.1628	3.04
MATCH	22.6	1146.5040	1146.5062	1.93	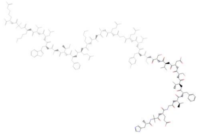	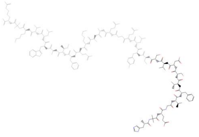	1146.5040	1146.5062	1.93
MISMATCH	95.0	1140.6251	1140.6286	3.06	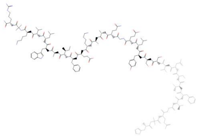	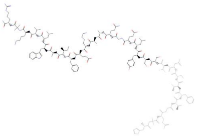	1140.6251	1140.6286	3.06

Metabolite: Substrate

Type	score	sub. m/z observed	sub. m/z calculated	sub ppm		met. m/z observed	met. m/z calculated	met. ppm
MISMATCH	21.5	1116.7004	1116.7040	3.23	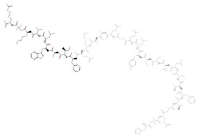 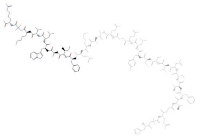	1116.7004	1116.7040	3.23
MATCH	74.4	1113.5749	1113.5771	2.03	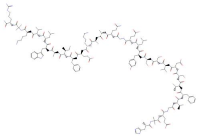 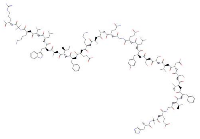	1113.5749	1113.5771	2.03
MATCH	108.8	1113.5734	1113.5771	3.31	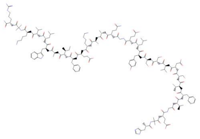 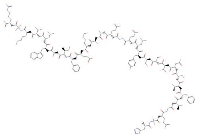	1113.5734	1113.5771	3.31
MISMATCH	-5.0	1112.0134	1112.0137	0.21	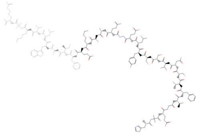 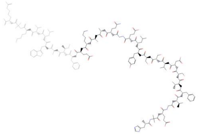	1112.0134	1112.0137	0.21
MISMATCH	-8.5	1101.8988	1101.8981	-0.68	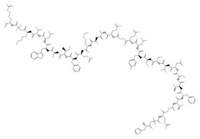 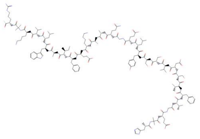	1101.8988	1101.8981	-0.68
MISMATCH	-8.5	1101.8988	1101.8981	-0.68	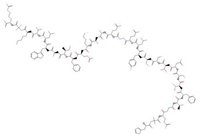 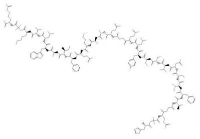	1101.8988	1101.8981	-0.68
MISMATCH	-8.5	1101.8988	1101.8981	-0.68	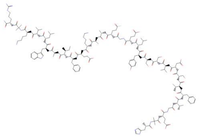 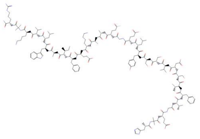	1101.8988	1101.8981	-0.68
MISMATCH	-8.5	1101.8988	1101.8981	-0.68	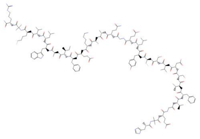 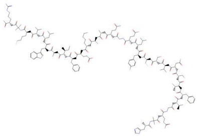	1101.8988	1101.8981	-0.68
MISMATCH	-8.5	1101.8988	1101.8981	-0.68	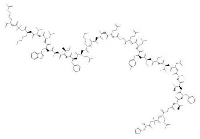 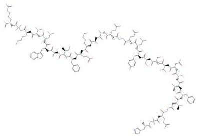	1101.8988	1101.8981	-0.68

Metabolite: Substrate

Type	score	sub. m/z observed	sub. m/z calculated	sub ppm		met. m/z observed	met. m/z calculated	met. ppm
MISMATCH	-8.5	1101.8988	1101.8981	-0.68	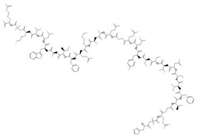 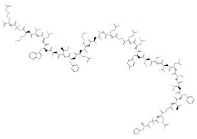	1101.8988	1101.8981	-0.68
MISMATCH	-8.5	1101.8988	1101.8981	-0.68	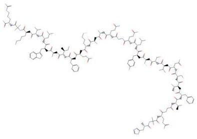 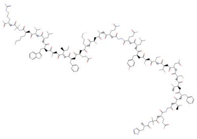	1101.8988	1101.8981	-0.68
MISMATCH	-8.5	1101.8988	1101.8981	-0.68	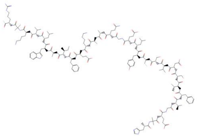 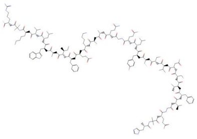	1101.8988	1101.8981	-0.68
MISMATCH	-8.5	1101.8988	1101.8981	-0.68	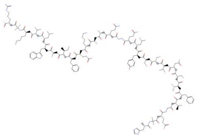 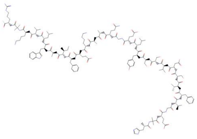	1101.8988	1101.8981	-0.68
MISMATCH	-8.5	1101.8988	1101.8981	-0.68	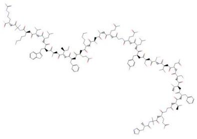 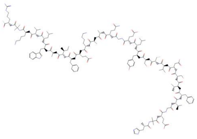	1101.8988	1101.8981	-0.68
MISMATCH	-8.5	1101.8988	1101.8981	-0.68	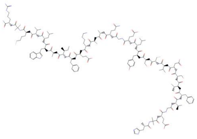 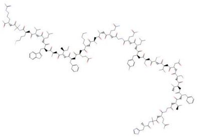	1101.8988	1101.8981	-0.68
MISMATCH	-8.5	1101.8988	1101.8981	-0.68	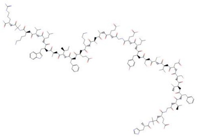 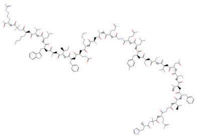	1101.8988	1101.8981	-0.68
MISMATCH	-8.5	1101.8988	1101.8981	-0.68	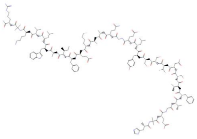 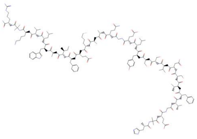	1101.8988	1101.8981	-0.68
MISMATCH	-8.5	1101.8988	1101.8981	-0.68	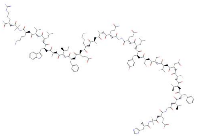 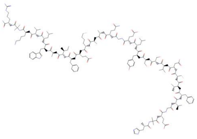	1101.8988	1101.8981	-0.68

Metabolite: Substrate

Type	score	sub. m/z observed	sub. m/z calculated	sub ppm			met. m/z observed	met. m/z calculated	met. ppm
MISMATCH	-8.5	1101.8988	1101.8981	-0.68	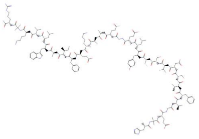	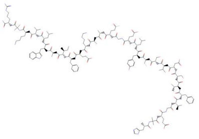	1101.8988	1101.8981	-0.68
MISMATCH	-8.5	1101.8988	1101.8981	-0.68	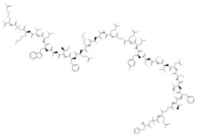	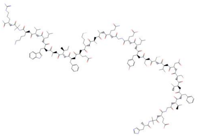	1101.8988	1101.8981	-0.68
MISMATCH	-8.5	1101.8988	1101.8981	-0.68	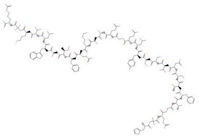	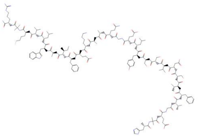	1101.8988	1101.8981	-0.68
MISMATCH	-8.5	1101.8988	1101.8981	-0.68	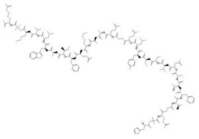	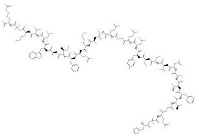	1101.8988	1101.8981	-0.68
MISMATCH	-8.5	1101.8988	1101.8981	-0.68	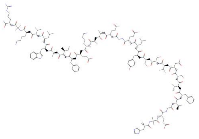	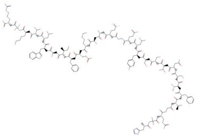	1101.8988	1101.8981	-0.68
MISMATCH	-8.5	1101.8988	1101.8981	-0.68	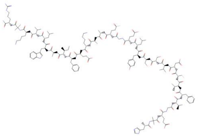	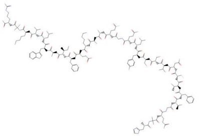	1101.8988	1101.8981	-0.68
MISMATCH	38.2	1097.1096	1097.1126	2.69	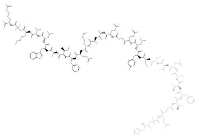	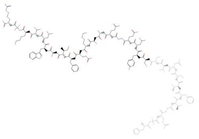	1097.1096	1097.1126	2.69
MATCH	39.6	1059.4718	1059.4742	2.23	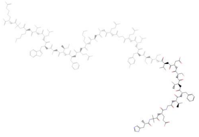	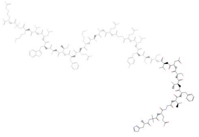	1059.4718	1059.4742	2.23
MISMATCH	20.8	1053.5927	1053.5966	3.63	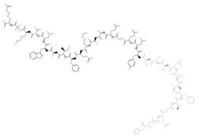	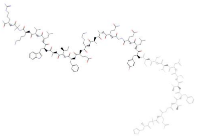	1053.5927	1053.5966	3.63

Metabolite: Substrate

Type	score	sub. m/z observed	sub. m/z calculated	sub ppm			met. m/z observed	met. m/z calculated	met. ppm
MATCH	40.3	972.0625	972.0649	2.44	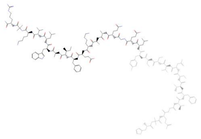	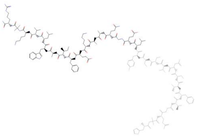	972.0625	972.0649	2.44
MISMATCH	19.7	969.6332	969.6356	2.54	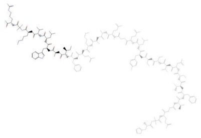	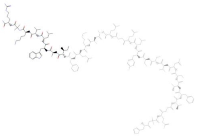	969.6332	969.6356	2.54
MATCH	123.9	960.4032	960.4058	2.64	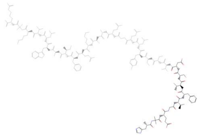	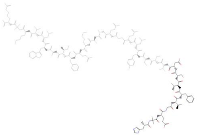	960.4032	960.4058	2.64
MISMATCH	-2.4	947.9247	947.9263	1.74	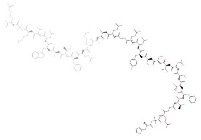	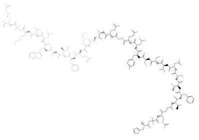	947.9247	947.9263	1.74
MATCH	15.9	942.3926	942.3952	2.80	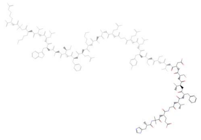	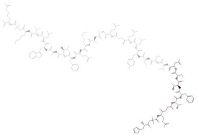	942.3926	942.3952	2.80
MATCH	15.9	942.3926	942.3952	2.80	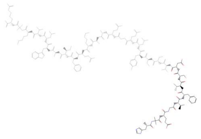	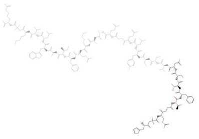	942.3926	942.3952	2.80
MATCH	15.9	942.3926	942.3952	2.80	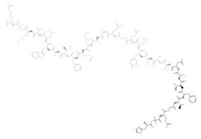	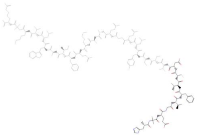	942.3926	942.3952	2.80
MATCH	52.7	915.5203	915.5229	2.76	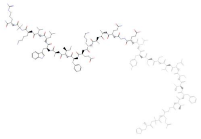	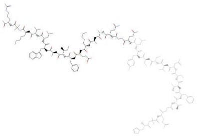	915.5203	915.5229	2.76
MATCH	18.1	906.5161	906.5176	1.60	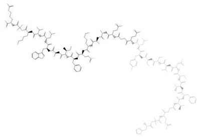	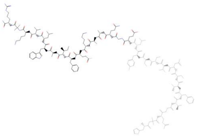	906.5161	906.5176	1.60

Metabolite: Substrate

Type	score	sub. m/z observed	sub. m/z calculated	sub ppm			met. m/z observed	met. m/z calculated	met. ppm
MATCH	18.1	906.5161	906.5176	1.60	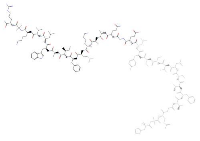	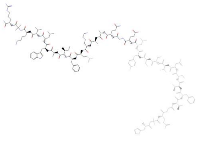	906.5161	906.5176	1.60
MATCH	30.2	906.5161	906.5176	1.60	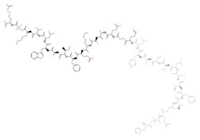	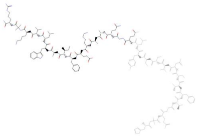	906.5161	906.5176	1.60
MATCH	30.2	906.5161	906.5176	1.60	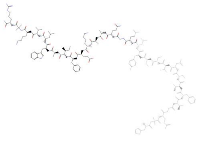	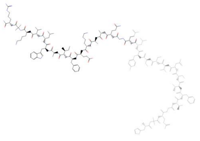	906.5161	906.5176	1.60
MATCH	5.8	861.4701	861.4618	-9.65	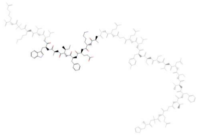	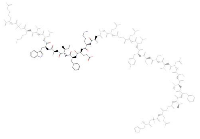	861.4701	861.4618	-9.65
MATCH	5.8	861.4701	861.4667	-3.90	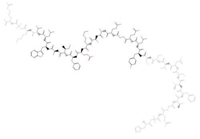	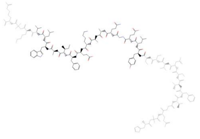	861.4701	861.4667	-3.90
MATCH	5.8	861.4701	861.4667	-3.90	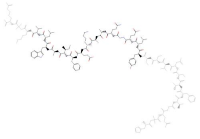	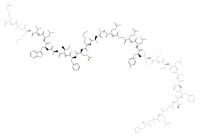	861.4701	861.4667	-3.90
MATCH	5.8	861.4701	861.4667	-3.90	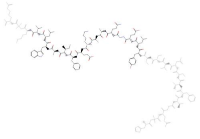	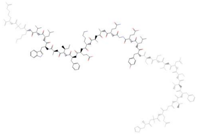	861.4701	861.4667	-3.90
MATCH	78.4	856.5492	856.5516	2.77	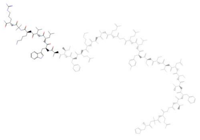	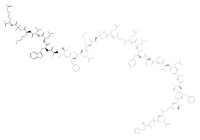	856.5492	856.5516	2.77
MATCH	50.4	850.9987	851.0016	3.36	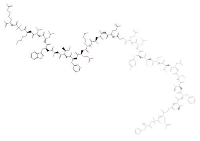	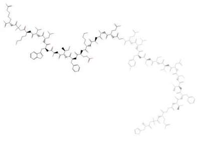	850.9987	851.0016	3.36

Metabolite: Substrate

Type	score	sub. m/z observed	sub. m/z calculated	sub ppm		met. m/z observed	met. m/z calculated	met. ppm
MATCH	12.9	845.3747	845.3788	4.90	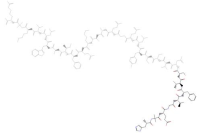 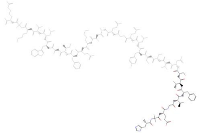	845.3747	845.3788	4.90
MATCH	143.9	835.4371	835.4347	-2.91	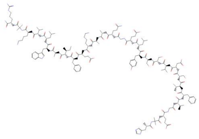 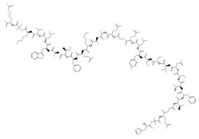	835.4371	835.4347	-2.91
MATCH	7.9	827.3615	827.3682	8.18	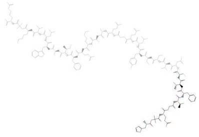 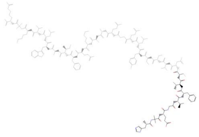	827.3615	827.3682	8.18
MATCH	7.9	827.3615	827.3682	8.18	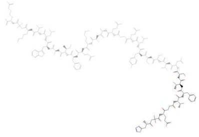 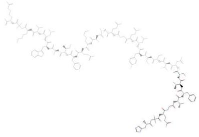	827.3615	827.3682	8.18
MATCH	7.9	827.3615	827.3682	8.18	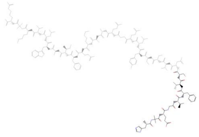 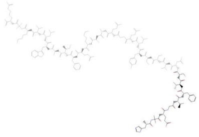	827.3615	827.3682	8.18
MATCH	8.9	822.4887	822.4908	2.58	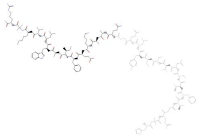 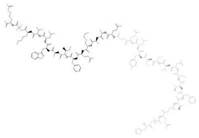	822.4887	822.4908	2.58
MISMATCH	10.7	793.7750	793.7776	3.28	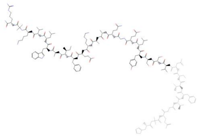 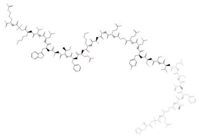	793.7750	793.7776	3.28
MISMATCH	79.6	785.5122	785.5145	2.93	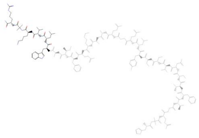 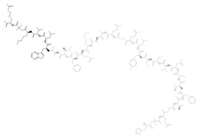	785.5122	785.5145	2.93
MISMATCH	28.0	760.7524	760.7548	3.17	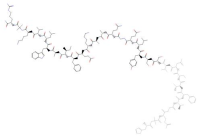 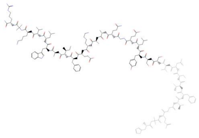	760.7524	760.7548	3.17

Metabolite: Substrate

Type	score	sub. m/z observed	sub. m/z calculated	sub ppm		met. m/z observed	met. m/z calculated	met. ppm
MISMATCH	27.4	758.4573	758.4616	5.67	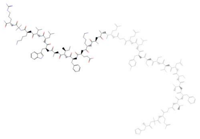 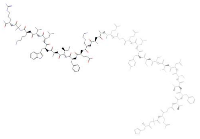	758.4573	758.4616	5.67
MATCH	20.7	758.3455	758.3468	1.71	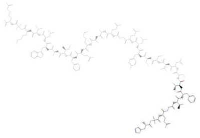 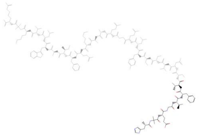	758.3455	758.3468	1.71
MISMATCH	5.6	755.0814	755.0793	-2.78	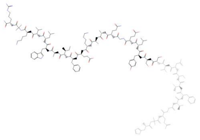 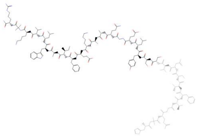	755.0814	755.0793	-2.78
MATCH	28.4	740.3344	740.3362	2.49	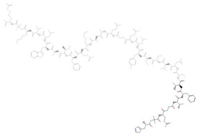 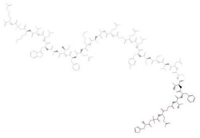	740.3344	740.3362	2.49
MATCH	28.4	740.3344	740.3362	2.49	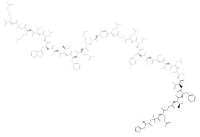 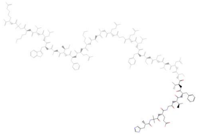	740.3344	740.3362	2.49
MISMATCH	10.0	731.7417	731.7441	3.31	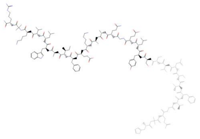 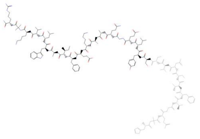	731.7417	731.7441	3.31
MISMATCH	16.8	722.9408	722.9430	2.97	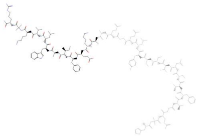 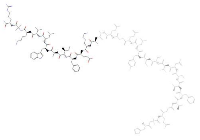	722.9408	722.9430	2.97
MISMATCH	8.3	702.7311	702.7335	3.31	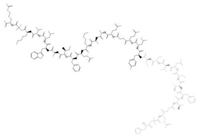 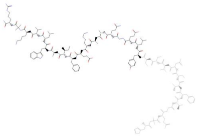	702.7311	702.7335	3.31
MATCH	21.9	687.4217	687.4244	3.99	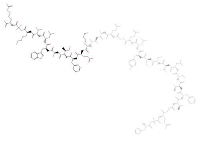 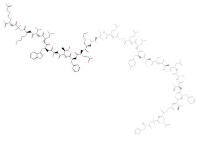	687.4217	687.4244	3.99

Metabolite: Substrate

Type	score	sub. m/z observed	sub. m/z calculated	sub ppm			met. m/z observed	met. m/z calculated	met. ppm
MATCH	112.4	668.5496	668.5492	-0.61	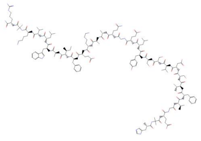	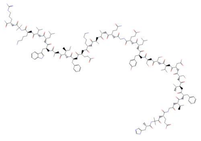	668.5496	668.5492	-0.61
MATCH	52.2	657.2973	657.2991	2.75	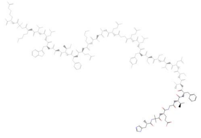	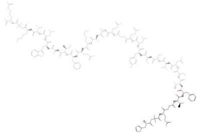	657.2973	657.2991	2.75
MATCH	7.7	648.3771	648.3790	3.00	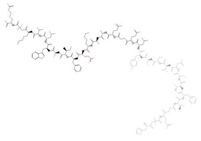	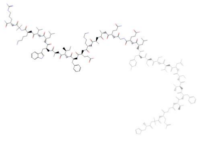	648.3771	648.3790	3.00
MATCH	12.7	639.2876	639.2885	1.49	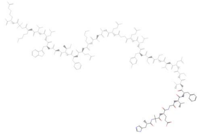	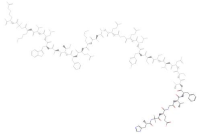	639.2876	639.2885	1.49
MATCH	102.0	629.3021	629.3042	3.35	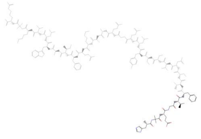	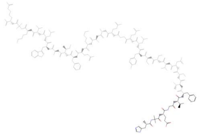	629.3021	629.3042	3.35
MATCH	25.1	610.6825	610.6843	2.98	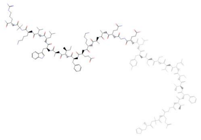	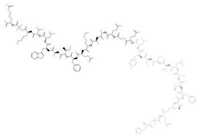	610.6825	610.6843	2.98
MISMATCH	100.2	599.4338	599.4351	2.24	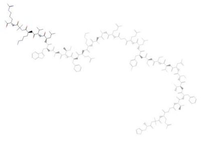	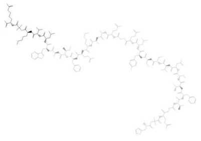	599.4338	599.4351	2.24
MATCH	11.4	589.3329	589.3344	2.62	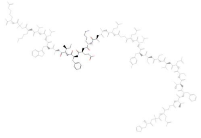	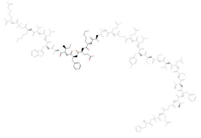	589.3329	589.3344	2.62
MATCH	11.4	589.3329	589.3344	2.62	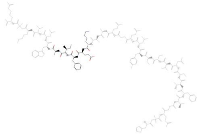	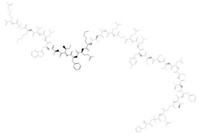	589.3329	589.3344	2.62

Metabolite: Substrate

Type	score	sub. m/z observed	sub. m/z calculated	sub ppm			met. m/z observed	met. m/z calculated	met. ppm
MATCH	11.4	589.3329	589.3344	2.62	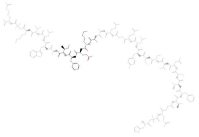	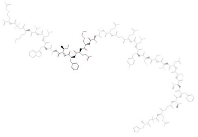	589.3329	589.3344	2.62
MATCH	11.4	589.3329	589.3344	2.62	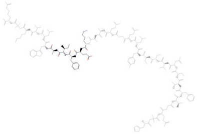	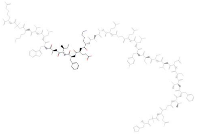	589.3329	589.3344	2.62
MATCH	6.4	573.7547	573.7567	3.55	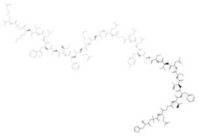	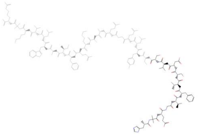	573.7547	573.7567	3.55
MATCH	43.3	567.6678	567.6701	4.15	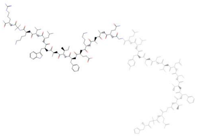	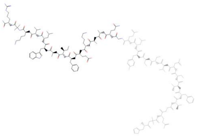	567.6678	567.6701	4.15
MATCH	33.7	510.2295	510.2307	2.28	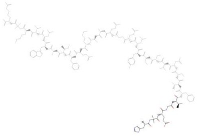	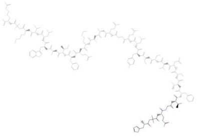	510.2295	510.2307	2.28
MISMATCH	109.1	486.3500	486.3511	2.28	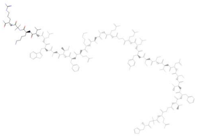	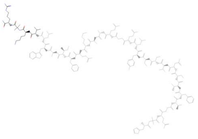	486.3500	486.3511	2.28
MATCH	11.8	471.7005	471.7012	1.61	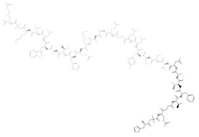	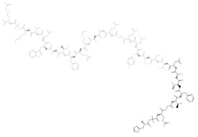	471.7005	471.7012	1.61
MATCH	11.8	471.7005	471.7012	1.61	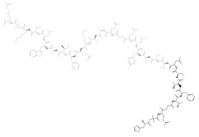	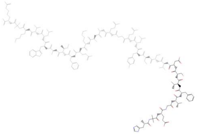	471.7005	471.7012	1.61
MATCH	11.8	471.7005	471.7012	1.61	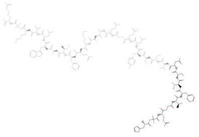	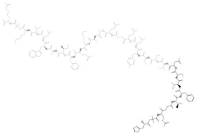	471.7005	471.7012	1.61

Metabolite: Substrate

Type	score	sub. m/z observed	sub. m/z calculated	sub ppm			met. m/z observed	met. m/z calculated	met. ppm
MATCH	9.6	462.6949	462.7009	12.93	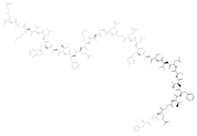	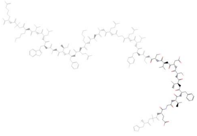	462.6949	462.7009	12.93
MATCH	9.6	462.6949	462.7009	12.93	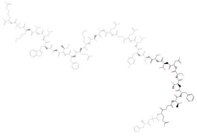	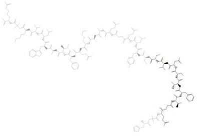	462.6949	462.7009	12.93
MATCH	23.8	428.7782	428.7794	2.80	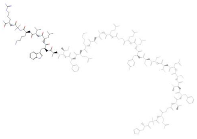	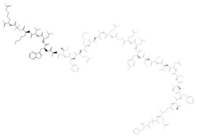	428.7782	428.7794	2.80
MATCH	10.4	414.1869	414.1878	2.02	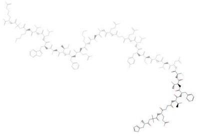	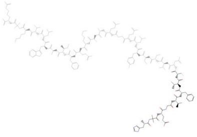	414.1869	414.1878	2.02
MATCH	10.4	414.1869	414.1878	2.02	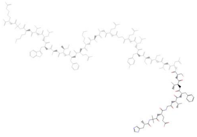	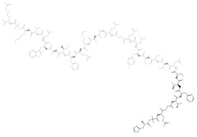	414.1869	414.1878	2.02
MATCH	10.4	414.1869	414.1878	2.02	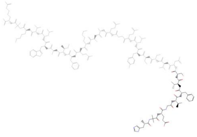	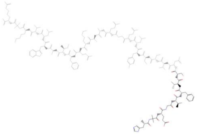	414.1869	414.1878	2.02
MATCH	14.6	409.1821	409.1830	2.21	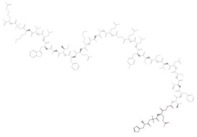	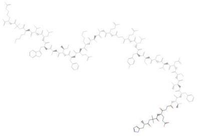	409.1821	409.1830	2.21
MISMATCH	26.2	393.2598	393.2609	2.83	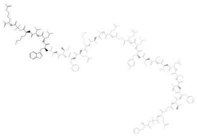	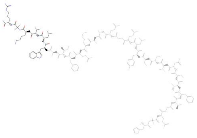	393.2598	393.2609	2.83
MATCH	59.0	387.2815	387.2827	3.02	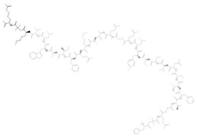	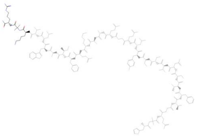	387.2815	387.2827	3.02

Metabolite: Substrate

Type	score	sub. m/z observed	sub. m/z calculated	sub ppm		met. m/z observed	met. m/z calculated	met. ppm
MATCH	21.5	379.6760	379.6770	2.78	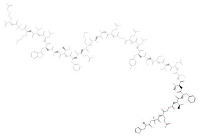 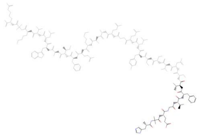	379.6760	379.6770	2.78
MATCH	5.7	370.6705	370.6717	3.48	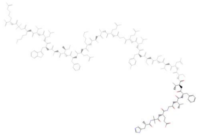 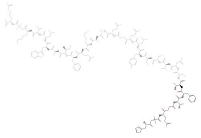	370.6705	370.6717	3.48
MATCH	5.7	370.6705	370.6717	3.48	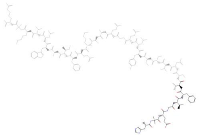 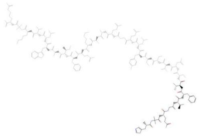	370.6705	370.6717	3.48
MATCH	8.5	356.6731	356.6743	3.31	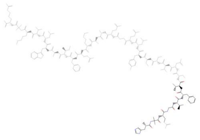 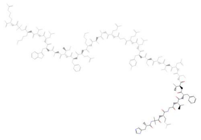	356.6731	356.6743	3.31
MATCH	8.5	356.6731	356.6792	17.18	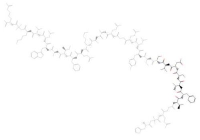 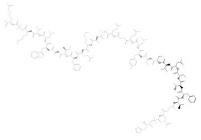	356.6731	356.6792	17.18
MATCH	8.5	356.6731	356.6743	3.31	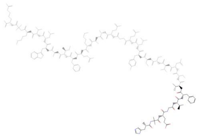 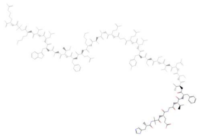	356.6731	356.6743	3.31
MATCH	8.5	356.6731	356.6743	3.31	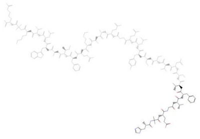 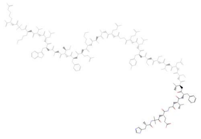	356.6731	356.6743	3.31
MATCH	19.0	352.1605	352.1615	3.00	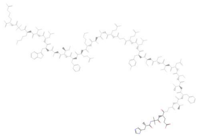 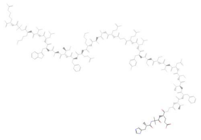	352.1605	352.1615	3.00
MATCH	14.0	338.1337	338.1347	2.83	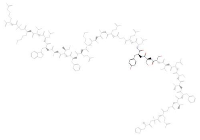 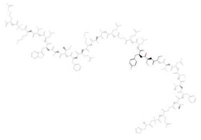	338.1337	338.1347	2.83

Metabolite: Substrate

Type	score	sub. m/z observed	sub. m/z calculated	sub ppm		met. m/z observed	met. m/z calculated	met. ppm
MATCH	14.0	338.1337	338.1347	2.83	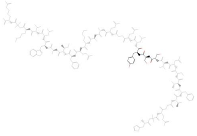 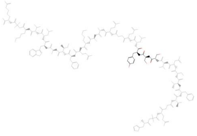	338.1337	338.1347	2.83
MISMATCH	10.7	320.1233	320.1241	2.47	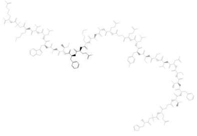 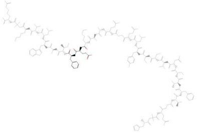	320.1233	320.1241	2.47
MATCH	5.3	315.1288	315.1299	3.46	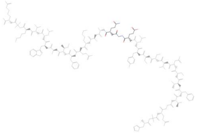 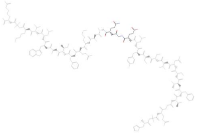	315.1288	315.1299	3.46
MISMATCH	-5.3	315.1288	315.1313	7.71	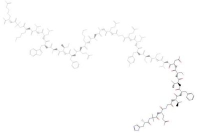 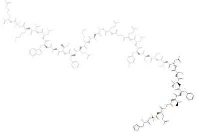	315.1288	315.1313	7.71
MATCH	5.3	315.1288	315.1299	3.46	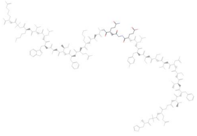 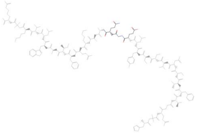	315.1288	315.1299	3.46
MATCH	16.9	304.1131	304.1139	2.82	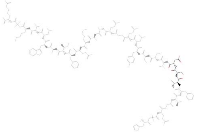 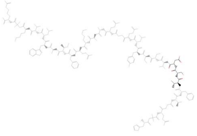	304.1131	304.1139	2.82
MATCH	30.9	304.1131	304.1139	2.82	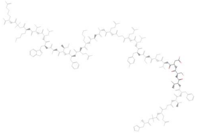 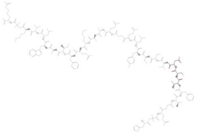	304.1131	304.1139	2.82
MATCH	10.5	302.1338	302.1397	19.74	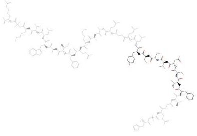 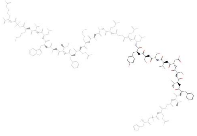	302.1338	302.1397	19.74
MATCH	16.3	302.1338	302.1347	2.91	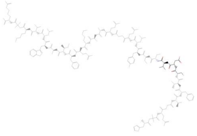 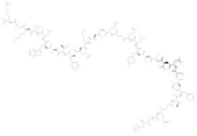	302.1338	302.1347	2.91

Metabolite: Substrate

Type	score	sub. m/z observed	sub. m/z calculated	sub ppm			met. m/z observed	met. m/z calculated	met. ppm
MISMATCH	-10.5	302.1338	302.1413	24.82	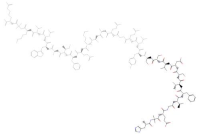	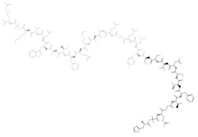	302.1338	302.1413	24.82
MATCH	16.3	302.1338	302.1347	2.91	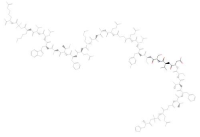	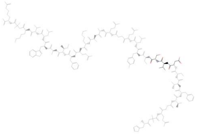	302.1338	302.1347	2.91
MATCH	10.5	302.1338	302.1347	2.91	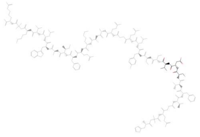	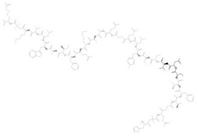	302.1338	302.1347	2.91
MATCH	16.3	302.1338	302.1347	2.91	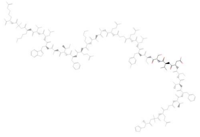	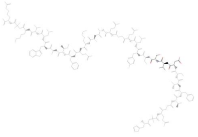	302.1338	302.1347	2.91
MATCH	13.4	297.1187	297.1193	2.22	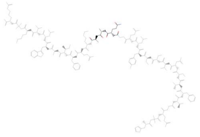	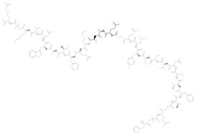	297.1187	297.1193	2.22
MATCH	9.7	286.1027	286.1034	2.41	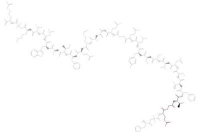	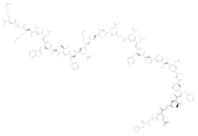	286.1027	286.1034	2.41
MATCH	9.5	286.1027	286.1034	2.41	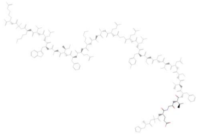	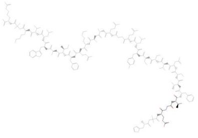	286.1027	286.1034	2.41
MATCH	9.5	286.1027	286.1034	2.41	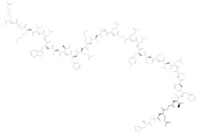	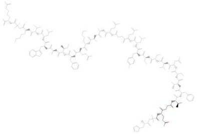	286.1027	286.1034	2.41
MATCH	18.6	259.1870	259.1877	2.62	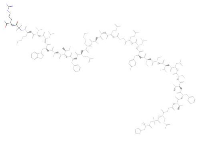	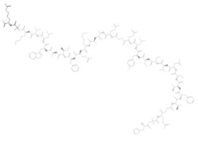	259.1870	259.1877	2.62

Metabolite: Substrate

Type	score	sub. m/z observed	sub. m/z calculated	sub ppm		met. m/z observed	met. m/z calculated	met. ppm
MATCH	16.0	257.1239	257.1244	2.20	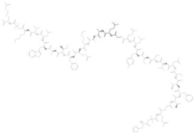 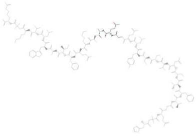	257.1239	257.1244	2.20
MATCH	3.8	257.1239	257.1291	20.25	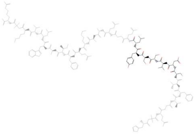 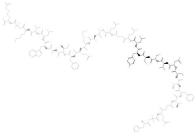	257.1239	257.1291	20.25
MATCH	16.0	257.1239	257.1244	2.20	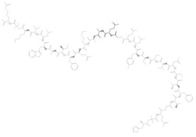 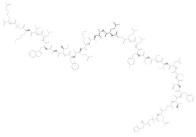	257.1239	257.1244	2.20
MISMATCH	17.3	249.1229	249.1234	2.01	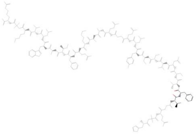 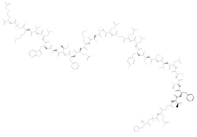	249.1229	249.1234	2.01
MISMATCH	26.5	249.1229	249.1234	2.01	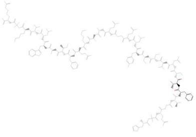 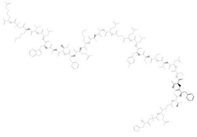	249.1229	249.1234	2.01
MISMATCH	26.5	249.1229	249.1234	2.01	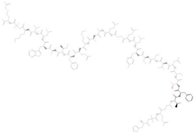 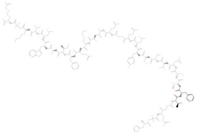	249.1229	249.1234	2.01
MISMATCH	26.5	249.1229	249.1234	2.01	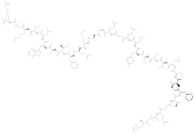 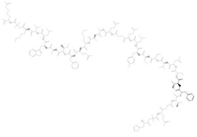	249.1229	249.1234	2.01
MATCH	60.5	243.1333	243.1339	2.72	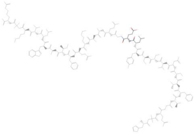 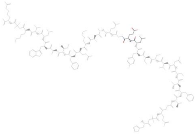	243.1333	243.1339	2.72
MATCH	11.8	243.1333	243.1339	2.72	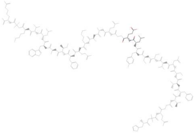 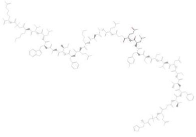	243.1333	243.1339	2.72

Metabolite: Substrate

Type	score	sub. m/z observed	sub. m/z calculated	sub ppm			met. m/z observed	met. m/z calculated	met. ppm
MATCH	48.4	223.1184	223.1190	2.48	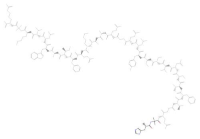	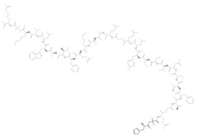	223.1184	223.1190	2.48
MISMATCH	-17.2	223.1071	223.1111	18.23	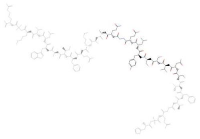	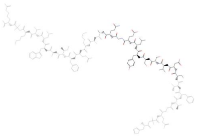	223.1071	223.1111	18.23
MATCH	14.2	223.1071	223.1001	-31.3	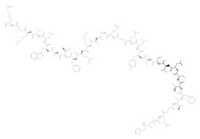	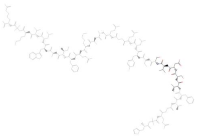	223.1071	223.1001	-31.3
MISMATCH	-17.2	223.1071	223.1039	-14.3	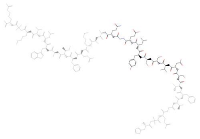	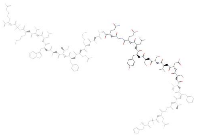	223.1071	223.1039	-14.3
MATCH	14.2	223.1071	223.1077	2.90	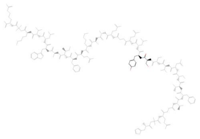	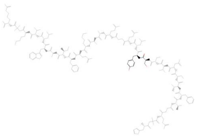	223.1071	223.1077	2.90
MATCH	27.1	221.1278	221.1285	2.74	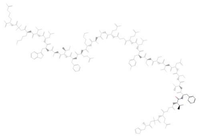	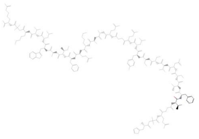	221.1278	221.1285	2.74
MATCH	101.1	221.1278	221.1285	2.74	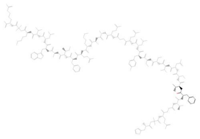	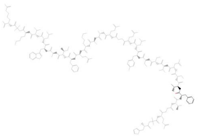	221.1278	221.1285	2.74
MATCH	101.3	215.1383	215.1390	3.14	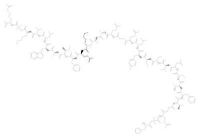	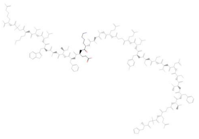	215.1383	215.1390	3.14
MATCH	79.6	215.1383	215.1357	-12.2	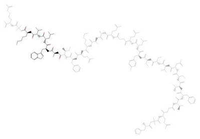	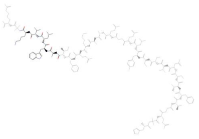	215.1383	215.1357	-12.2

Metabolite: Substrate

Type	score	sub. m/z observed	sub. m/z calculated	sub ppm			met. m/z observed	met. m/z calculated	met. ppm
MATCH	3.8	215.1021	215.1026	2.41	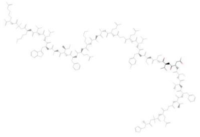	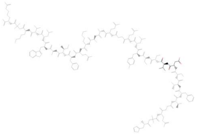	215.1021	215.1026	2.41
MATCH	3.8	215.1021	215.1026	2.41	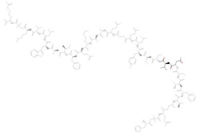	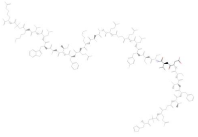	215.1021	215.1026	2.41
MATCH	37.5	203.0657	203.0662	2.70	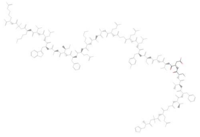	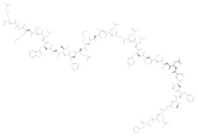	203.0657	203.0662	2.70
MATCH	8.0	203.0657	203.0662	2.70	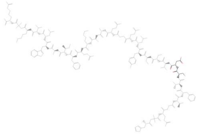	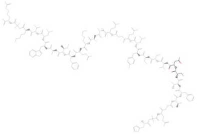	203.0657	203.0662	2.70
MATCH	39.1	195.1237	195.1240	1.96	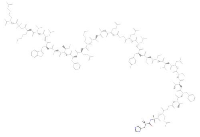	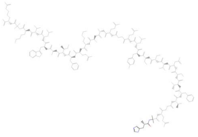	195.1237	195.1240	1.96
MATCH	17.7	187.1073	187.1077	2.11	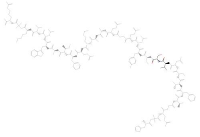	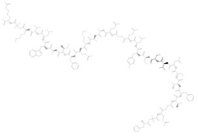	187.1073	187.1077	2.11
MATCH	17.7	187.1073	187.1077	2.11	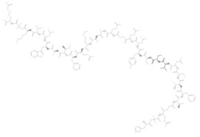	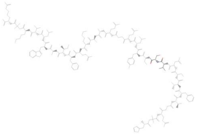	187.1073	187.1077	2.11
MISMATCH	3.4	186.0864	186.0908	23.47	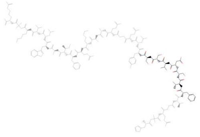	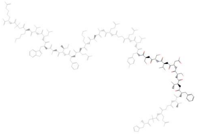	186.0864	186.0908	23.47
MISMATCH	-31.9	186.0864	186.0855	-4.92	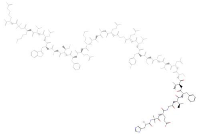	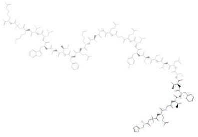	186.0864	186.0855	-4.92

Metabolite: Substrate

Type	score	sub. m/z observed	sub. m/z calculated	sub ppm		met. m/z observed	met. m/z calculated	met. ppm
MATCH	31.9	186.0864	186.0873	4.77	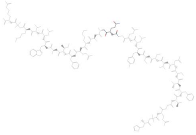 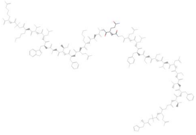	186.0864	186.0873	4.77
MATCH	31.9	186.0864	186.0873	4.77	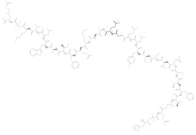 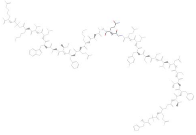	186.0864	186.0873	4.77
MATCH	23.4	175.0710	175.0713	1.66	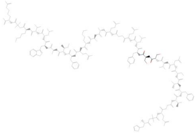 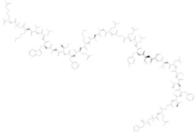	175.0710	175.0713	1.66
MATCH	23.4	175.0710	175.0713	1.66	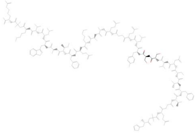 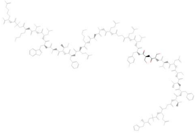	175.0710	175.0713	1.66
MISMATCH	19.6	159.0913	159.0946	20.67	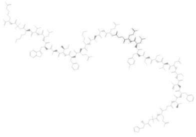 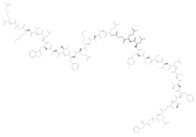	159.0913	159.0946	20.67
MISMATCH	2.9	157.0604	157.0608	2.08	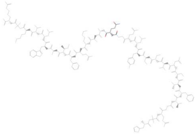 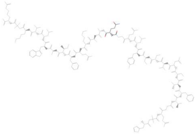	157.0604	157.0608	2.08
MATCH	10.9	147.0760	147.0764	2.67	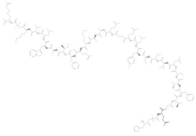 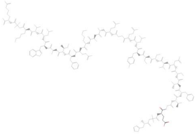	147.0760	147.0764	2.67
MATCH	101.0	147.0760	147.0764	2.67	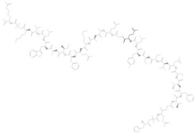 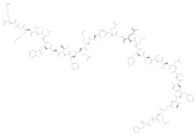	147.0760	147.0764	2.67
MATCH	45.9	136.0755	136.0693	-45.4	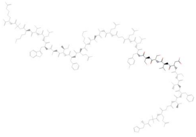 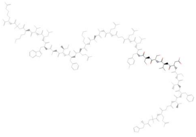	136.0755	136.0693	-45.4

Metabolite: Substrate

Type	score	sub. m/z observed	sub. m/z calculated	sub ppm			met. m/z observed	met. m/z calculated	met. ppm
MATCH	45.9	136.0755	136.0706	-35.6	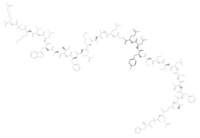	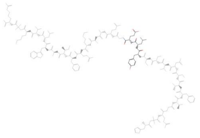	136.0755	136.0706	-35.6
MISMATCH	45.9	136.0755	136.0737	-13.0	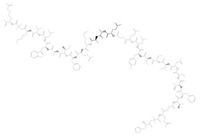	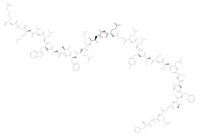	136.0755	136.0737	-13.0
MATCH	45.9	136.0755	136.0706	-35.6	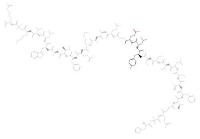	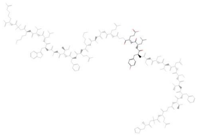	136.0755	136.0706	-35.6
MISMATCH	45.9	136.0755	136.0737	-13.0	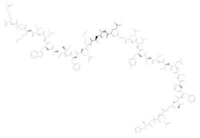	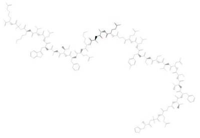	136.0755	136.0737	-13.0
MATCH	45.9	136.0755	136.0693	-45.4	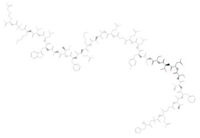	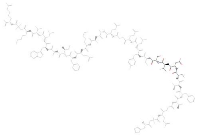	136.0755	136.0693	-45.4
MATCH	10.3	130.0649	130.0671	16.63	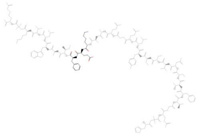	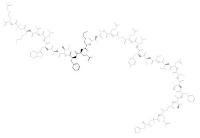	130.0649	130.0671	16.63
MATCH	109.5	129.1021	129.1022	0.79	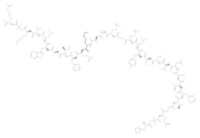	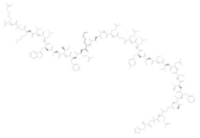	129.1021	129.1022	0.79
MATCH	109.5	129.1021	129.1022	0.79	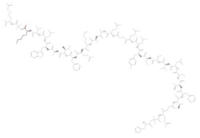	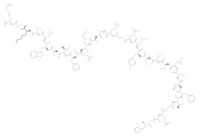	129.1021	129.1022	0.79
MATCH	109.5	129.1021	129.1022	0.79	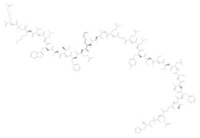	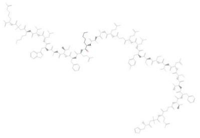	129.1021	129.1022	0.79

Metabolite: Substrate

Type	score	sub. m/z observed	sub. m/z calculated	sub ppm		met. m/z observed	met. m/z calculated	met. ppm
MATCH	109.5	129.1021	129.1022	0.79	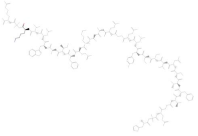 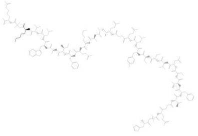	129.1021	129.1022	0.79
MATCH	117.2	120.0808	120.0865	47.43	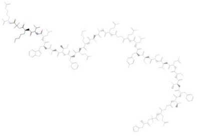 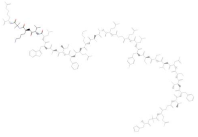	120.0808	120.0865	47.43
MATCH	117.2	120.0808	120.0808	-0.05	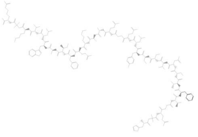 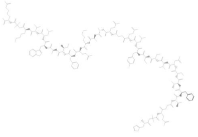	120.0808	120.0808	-0.05
MATCH	117.2	120.0808	120.0808	-0.05	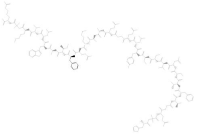 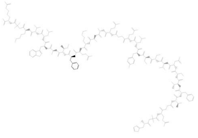	120.0808	120.0808	-0.05
MATCH	117.2	120.0808	120.0731	-63.5	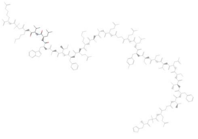 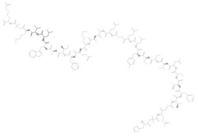	120.0808	120.0731	-63.5
MATCH	43.1	110.0714	110.0713	-1.24	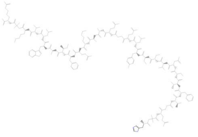 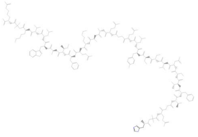	110.0714	110.0713	-1.24
MISMATCH	7.7	102.0552	102.0550	-2.07	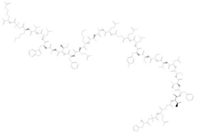 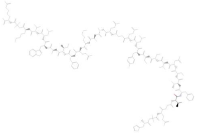	102.0552	102.0550	-2.07
MATCH	15.5	102.0552	102.0606	52.97	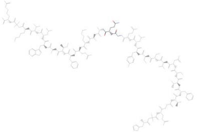 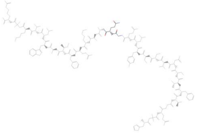	102.0552	102.0606	52.97
MISMATCH	7.7	102.0552	102.0550	-2.07	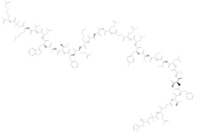 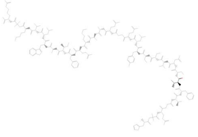	102.0552	102.0550	-2.07

Metabolite: Substrate

Type	score	sub. m/z observed	sub. m/z calculated	sub ppm			met. m/z observed	met. m/z calculated	met. ppm
MATCH	15.5	102.0552	102.0588	35.30	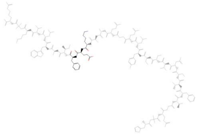	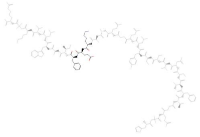	102.0552	102.0588	35.30
MATCH	15.5	102.0552	102.0588	35.30	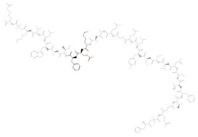	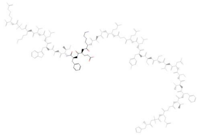	102.0552	102.0588	35.30
MISMATCH	7.7	102.0552	102.0550	-2.07	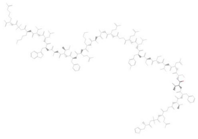	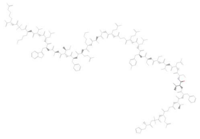	102.0552	102.0550	-2.07
MISMATCH	7.7	102.0552	102.0550	-2.07	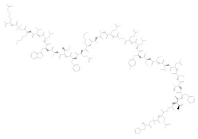	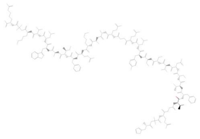	102.0552	102.0550	-2.07

MS (+) FT

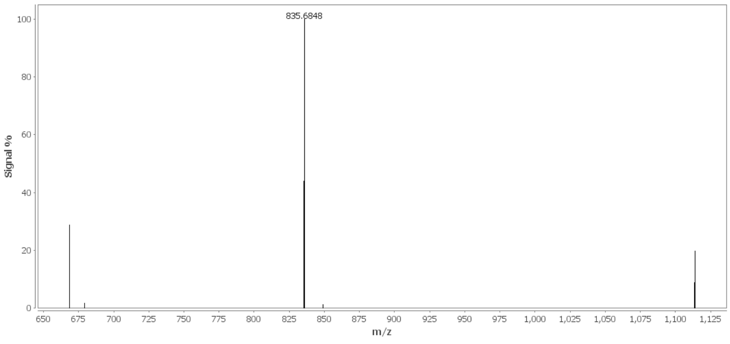

MS (+) FT

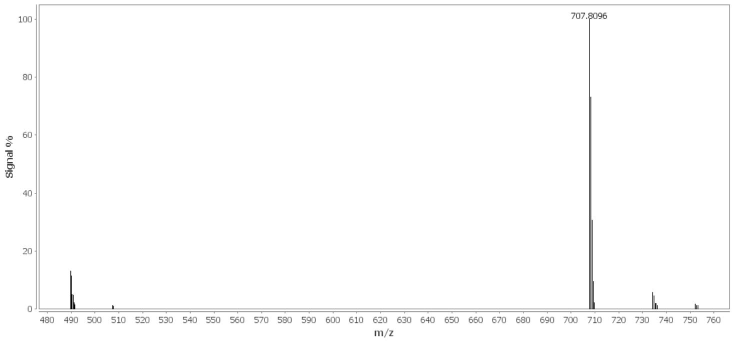

MS2 (+) FT activ = HCD:ce =

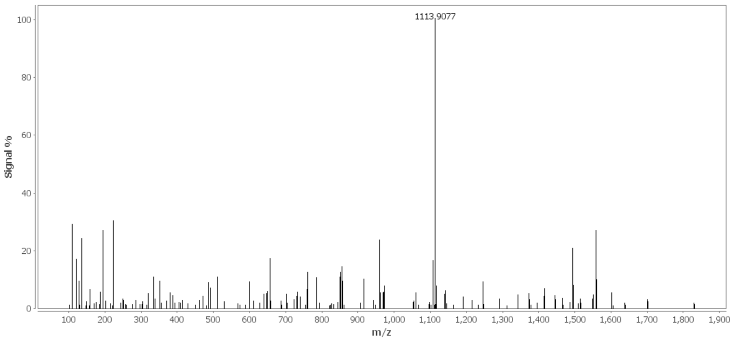

MS2 (+) FT activ = HCD:ce =

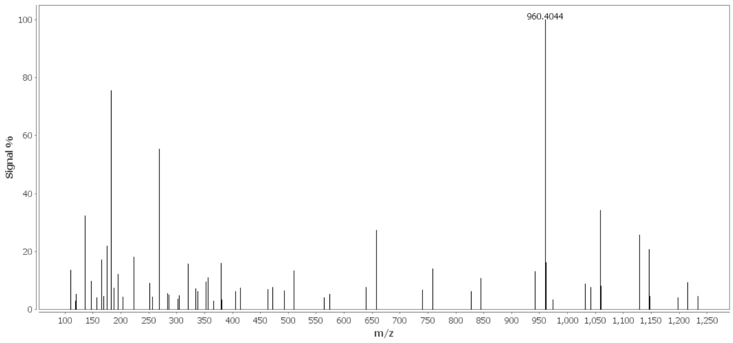

Metabolite: M5 -1924 RT=3.96

Type	score	sub. m/z observed	sub. m/z calculated	sub ppm	met. m/z observed	met. m/z calculated	met. ppm

Metabolite: M5 -1924 RT=3.96

Type	score	sub. m/z observed	sub. m/z calculated	sub ppm			met. m/z observed	met. m/z calculated	met. ppm
MATCH	112.4	668.5496	668.5492	-0.61	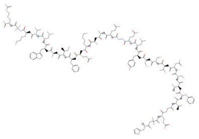	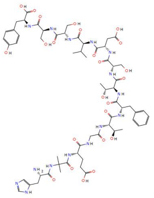	707.8096	707.8097	0.18
						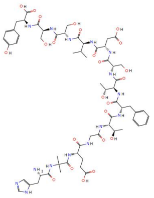	707.8096	707.8097	0.18
						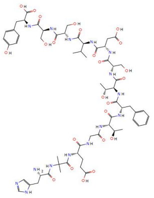	707.8096	707.8097	0.18
MATCH	143.9	835.4371	835.4347	-2.91	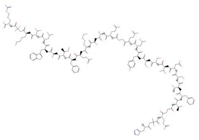	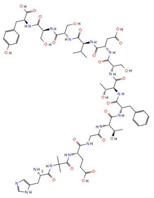	707.8096	707.8097	0.18
						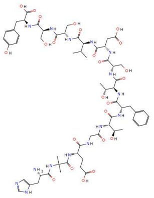	707.8096	707.8097	0.18
						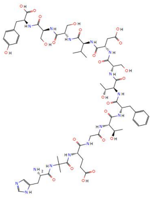	707.8096	707.8097	0.18
MATCH	108.8	1113.5734	1113.5771	3.31	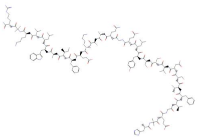	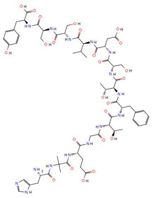	707.8096	707.8097	0.18
						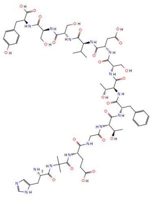	707.8096	707.8097	0.18
						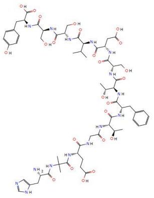	707.8096	707.8097	0.18

Metabolite: M5 -1924 RT=3.96

Type	score	sub. m/z observed	sub. m/z calculated	sub ppm		met. m/z observed	met. m/z calculated	met. ppm
MATCH	42.8	110.0714	110.0713	-1.24	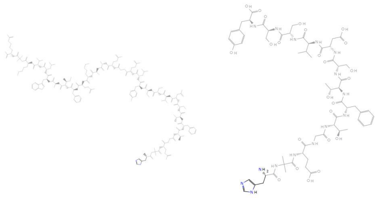	110.0716	110.0713	-2.84
MATCH	22.5	120.0808	120.0808	-0.05	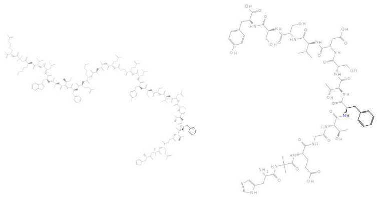	120.0810	120.0808	-1.50
MATCH	10.9	147.0760	147.0764	2.67	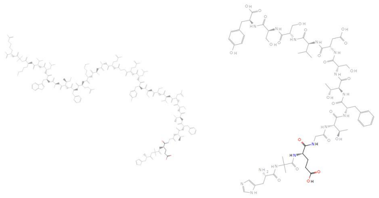	147.0764	147.0764	0.23
MATCH	23.4	175.0710	175.0713	1.66	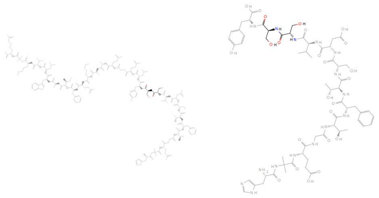	175.0712	175.0713	0.70
MATCH	23.4	175.0710	175.0713	1.66	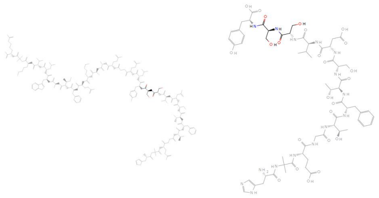	175.0712	175.0713	0.70
MATCH	13.2	187.1073	187.1077	2.11	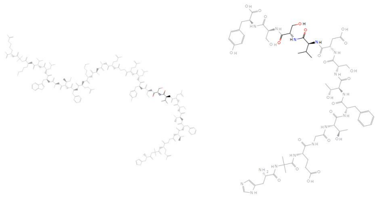	187.1077	187.1077	0.28
MATCH	13.2	187.1073	187.1077	2.11	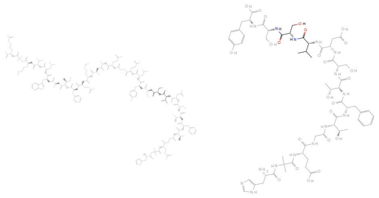	187.1077	187.1077	0.28
MATCH	39.1	195.1237	195.1240	1.96	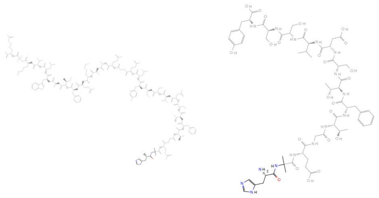	195.1236	195.1240	2.03
MATCH	6.9	203.0657	203.0662	2.70	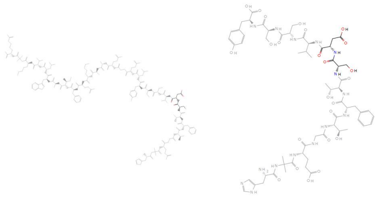	203.0659	203.0662	1.85

Metabolite: M5 -1924 RT=3.96

Type	score	sub. m/z observed	sub. m/z calculated	sub ppm		met. m/z observed	met. m/z calculated	met. ppm
MATCH	6.9	203.0657	203.0662	2.70	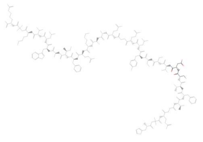 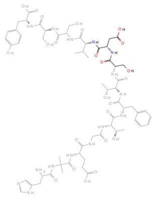	203.0659	203.0662	1.85
MATCH	14.2	223.1071	223.1001	-31.3	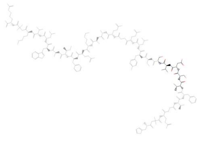 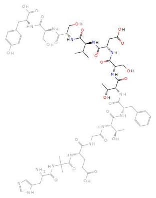	223.1073	223.1001	-32.3
MATCH	14.2	223.1071	223.1077	2.90	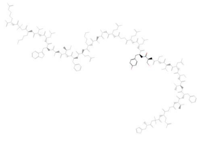 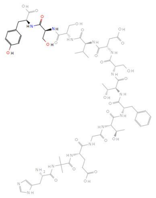	223.1073	223.1077	1.88
MATCH	48.4	223.1184	223.1190	2.48	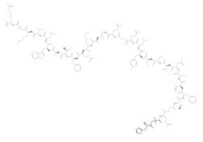 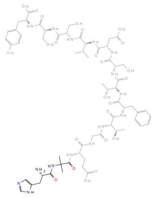	223.1187	223.1190	1.27
MATCH	7.8	286.1027	286.1034	2.41	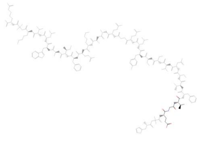 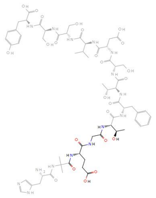	286.1028	286.1034	2.13
MATCH	7.8	286.1027	286.1034	2.41	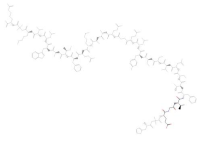 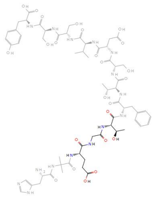	286.1028	286.1034	2.13
MATCH	7.8	286.1027	286.1034	2.41	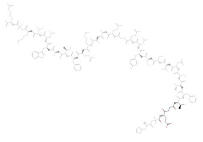 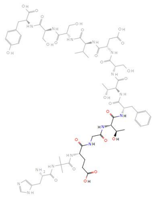	286.1028	286.1034	2.13
MATCH	5.0	302.1338	302.1347	2.91	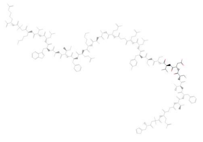 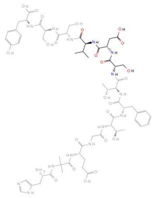	302.1338	302.1347	2.79
MATCH	5.0	302.1338	302.1347	2.91	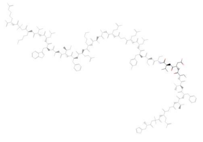 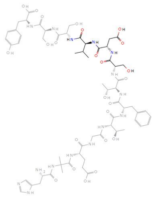	302.1338	302.1347	2.79

Metabolite: M5 -1924 RT=3.96

Type	score	sub. m/z observed	sub. m/z calculated	sub ppm			met. m/z observed	met. m/z calculated	met. ppm
MATCH	5.0	302.1338	302.1347	2.91	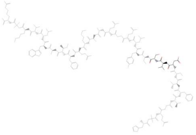	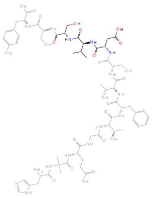	302.1338	302.1347	2.79
MATCH	5.0	302.1338	302.1347	2.91	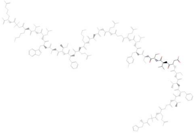	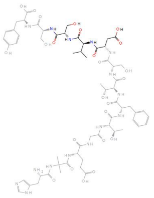	302.1338	302.1347	2.79
MATCH	7.4	304.1131	304.1139	2.82	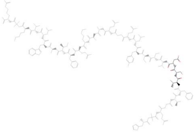	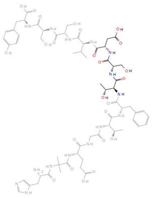	304.1132	304.1139	2.32
MATCH	7.4	304.1131	304.1139	2.82	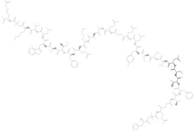	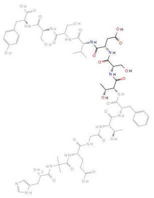	304.1132	304.1139	2.32
MATCH	19.0	352.1605	352.1615	3.00	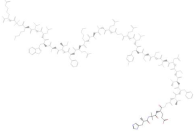	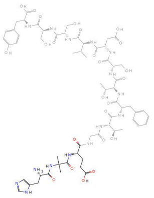	352.1613	352.1615	0.82
MATCH	4.8	356.6731	356.6743	3.31	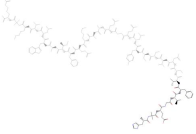	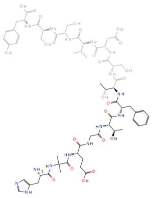	356.6737	356.6743	1.63
MATCH	4.8	356.6731	356.6743	3.31	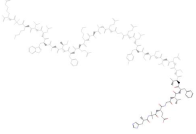	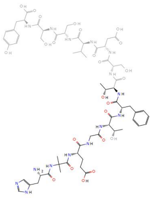	356.6737	356.6743	1.63
MATCH	4.8	356.6731	356.6743	3.31	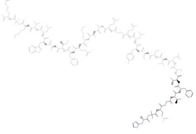	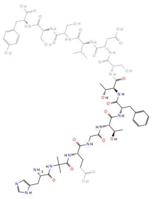	356.6737	356.6743	1.63
MATCH	4.8	356.6731	356.6792	17.18	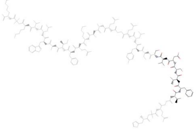	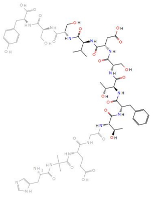	356.6737	356.6792	15.50

Metabolite: M5 -1924 RT=3.96

Type	score	sub. m/z observed	sub. m/z calculated	sub ppm		met. m/z observed	met. m/z calculated	met. ppm
MATCH	21.5	379.6760	379.6770	2.78	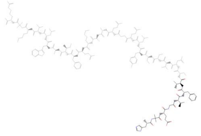 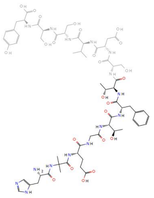	379.6763	379.6770	1.91
MATCH	10.4	414.1869	414.1878	2.02	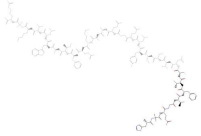 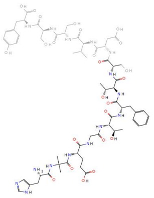	414.1877	414.1878	0.11
MATCH	10.4	414.1869	414.1878	2.02	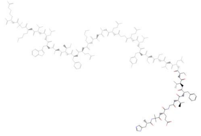 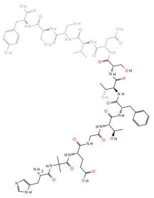	414.1877	414.1878	0.11
MATCH	10.4	414.1869	414.1878	2.02	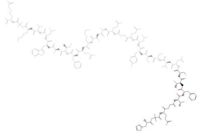 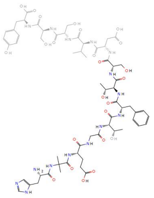	414.1877	414.1878	0.11
MATCH	9.6	462.6949	462.7009	12.93	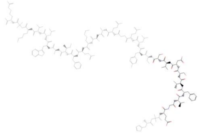 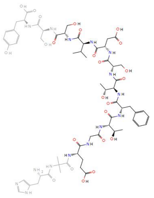	462.6943	462.7009	14.25
MATCH	9.6	462.6949	462.7009	12.93	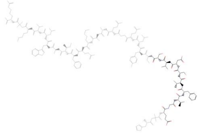 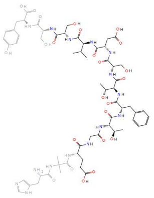	462.6943	462.7009	14.25
MATCH	11.8	471.7005	471.7012	1.61	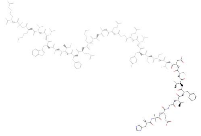 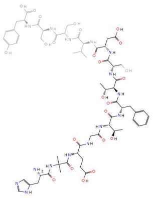	471.7024	471.7012	-2.52
MATCH	11.8	471.7005	471.7012	1.61	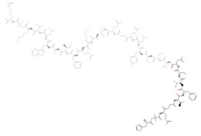 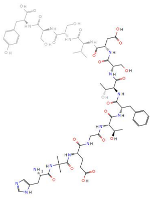	471.7024	471.7012	-2.52
MATCH	11.8	471.7005	471.7012	1.61	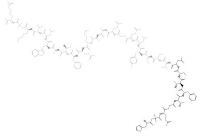 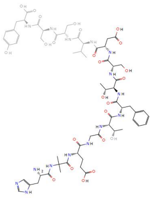	471.7024	471.7012	-2.52

Metabolite: M5 -1924 RT=3.96

Type	score	sub. m/z observed	sub. m/z calculated	sub ppm			met. m/z observed	met. m/z calculated	met. ppm
MATCH	24.3	510.2295	510.2307	2.28	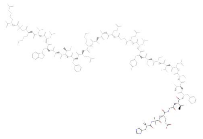	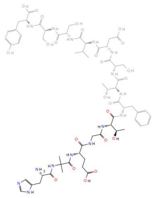	510.2298	510.2307	1.74
MATCH	6.4	573.7547	573.7567	3.55	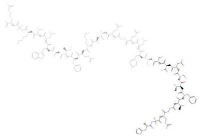	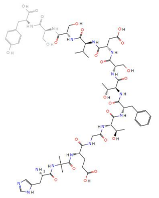	573.7567	573.7567	0.14
MATCH	12.6	639.2876	639.2885	1.49	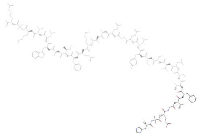	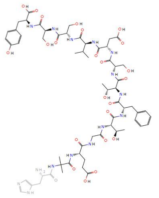	639.2881	639.2802	-12.2
						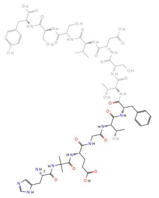	639.2881	639.2885	0.71
MATCH	44.5	657.2973	657.2991	2.75	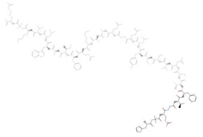	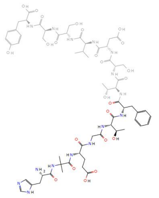	657.2983	657.2991	1.27
MATCH	10.6	740.3344	740.3362	2.49	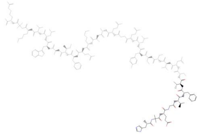	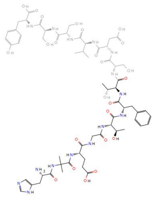	740.3372	740.3362	-1.27
MATCH	10.6	740.3344	740.3362	2.49	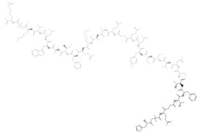	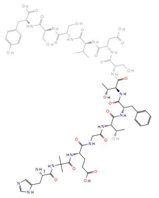	740.3372	740.3362	-1.27
MATCH	20.7	758.3455	758.3468	1.71	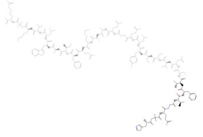	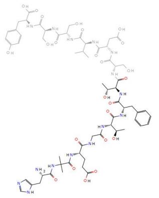	758.3459	758.3468	1.10
MATCH	7.9	827.3615	827.3682	8.18	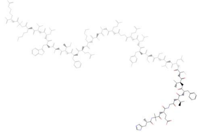	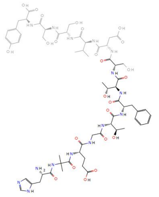	827.3660	827.3682	2.68

Metabolite: M5 -1924 RT=3.96

Type	score	sub. m/z observed	sub. m/z calculated	sub ppm		met. m/z observed	met. m/z calculated	met. ppm
MATCH	7.9	827.3615	827.3682	8.18	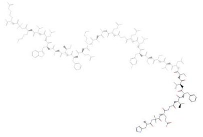 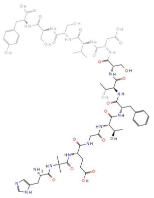	827.3660	827.3682	2.68
MATCH	7.9	827.3615	827.3682	8.18	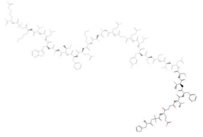 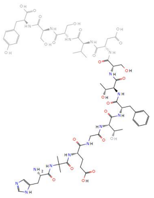	827.3660	827.3682	2.68
MATCH	12.9	845.3747	845.3788	4.90	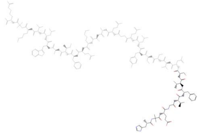 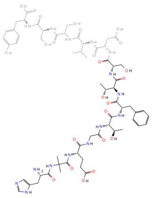	845.3770	845.3788	2.19
MATCH	15.9	942.3926	942.3952	2.80	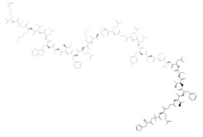 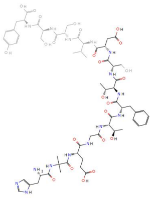	942.3912	942.3952	4.19
MATCH	15.9	942.3926	942.3952	2.80	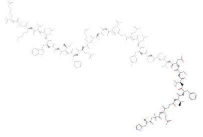 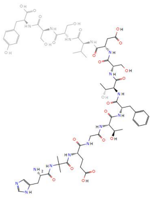	942.3912	942.3952	4.19
MATCH	15.9	942.3926	942.3952	2.80	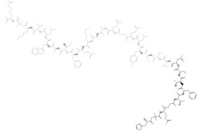 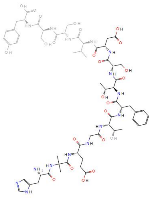	942.3912	942.3952	4.19
MATCH	123.9	960.4032	960.4058	2.64	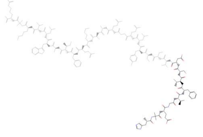 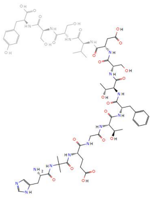	960.4044	960.4058	1.41
MATCH	39.6	1059.4718	1059.4742	2.23	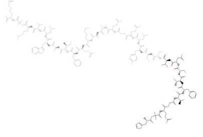 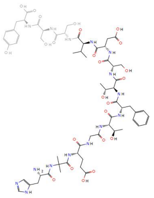	1059.4722	1059.4742	1.83
MATCH	22.6	1146.5040	1146.5062	1.93	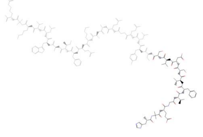 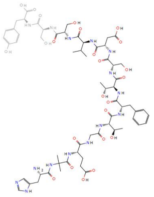	1146.5042	1146.5062	1.78

Metabolite: M5 -1924 RT=3.96

Type	score	sub. m/z observed	sub. m/z calculated	sub ppm			met. m/z observed	met. m/z calculated	met. ppm
MATCH	12.2	1215.5245	1215.5277	2.59	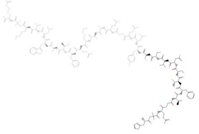	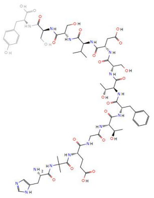	1215.5297	1215.5277	-1.65
MATCH	12.2	1215.5245	1215.5277	2.59	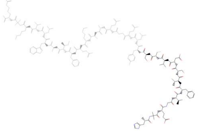	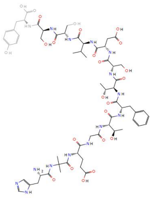	1215.5297	1215.5277	-1.65
MATCH	12.2	1215.5245	1215.5277	2.59	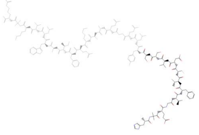	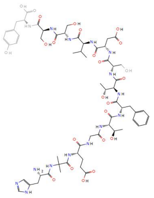	1215.5297	1215.5277	-1.65
MATCH	12.2	1215.5245	1215.5277	2.59	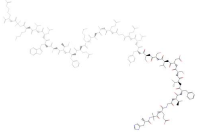	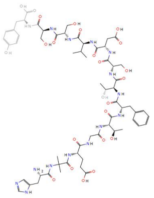	1215.5297	1215.5277	-1.65
MATCH	12.2	1215.5245	1215.5277	2.59	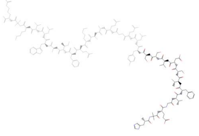	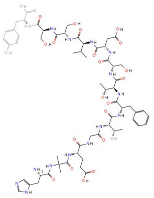	1215.5297	1215.5277	-1.65
MATCH	5.7	1233.5312	1233.5382	5.71	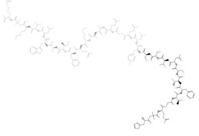	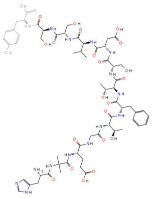	1233.5355	1233.5382	2.19
MATCH	10.6	1601.3333	1601.3326	-0.45	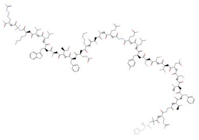	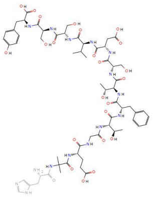	639.2881	639.2802	-12.2
						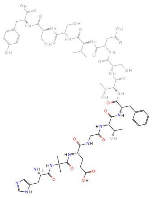	639.2881	639.2885	0.71
MISMATCH	-22.5	120.0808	120.0731	-63.5	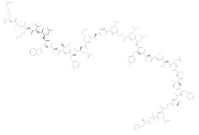		120.0810	120.0810	0.00

Metabolite: M5 -1924 RT=3.96

Type	score	sub. m/z observed	sub. m/z calculated	sub ppm		met. m/z observed	met. m/z calculated	met. ppm
MISMATCH	-56.6	136.0755	136.0737	-13.0	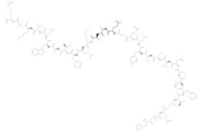	136.0757	136.0757	0.00
MISMATCH	-5.2	157.0604	157.0608	2.08	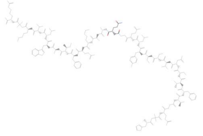	157.0605	157.0605	0.00
MISMATCH	-21.0	320.1233	320.1241	2.47	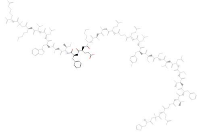	320.1237	320.1237	0.00
MISMATCH	-78.9	702.7311	702.7335	3.31	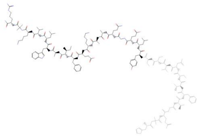	182.0811	182.0811	0.00
MISMATCH	-59.4	731.7417	731.7441	3.31	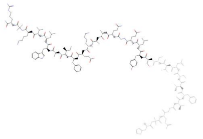	269.1129	269.1129	0.00
MISMATCH	-20.4	760.7524	760.7548	3.17	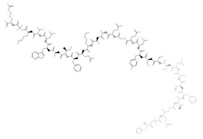	356.1447	356.1447	0.00
MISMATCH	-77.7	1053.5927	1053.5966	3.63	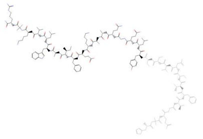	182.0811	182.0811	0.00
MISMATCH	-56.7	1097.1096	1097.1126	2.69	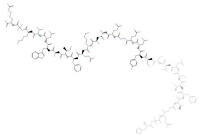	269.1129	269.1129	0.00
MISMATCH	-16.0	1140.6251	1140.6286	3.06	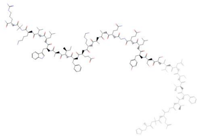	356.1447	356.1447	0.00

Metabolite: M5 -1924 RT=3.96

Type	score	sub. m/z observed	sub. m/z calculated	sub ppm		met. m/z observed	met. m/z calculated	met. ppm
MET_MATCH					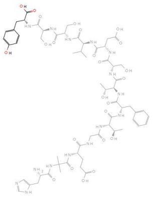	165.0545	165.0546	0.98
MET_MATCH					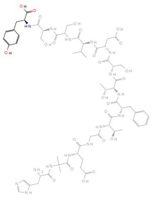	182.0811	182.0812	0.39
MET_MATCH					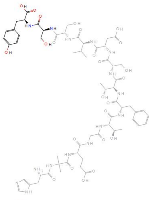	269.1129	269.1132	1.10
MET_MATCH					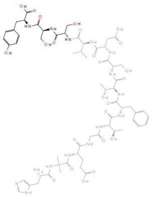	356.1447	356.1452	1.52
MET_MATCH					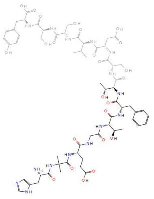	365.6791	365.6796	1.18
MET_MATCH					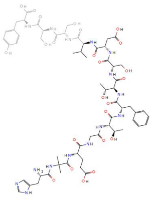	1031.4778	1031.4793	1.42

MS (+) FT

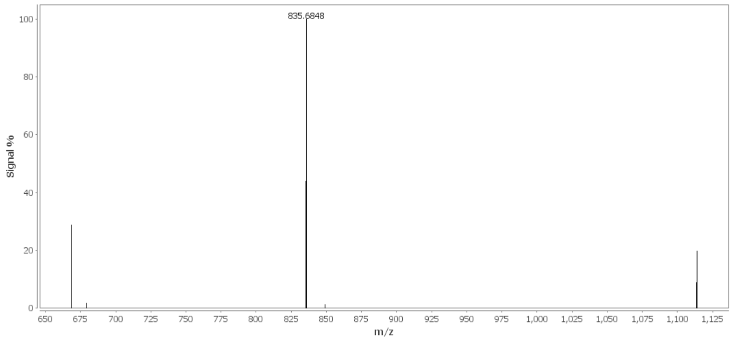

MS (+) FT

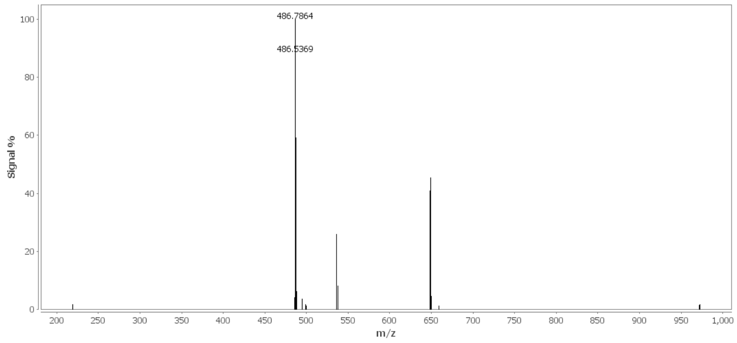

MS2 (+) FT activ = HCD:ce =

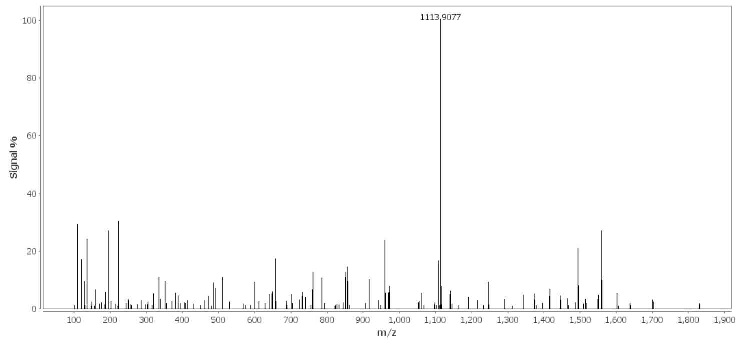

MS2 (+) FT activ = HCD:ce =

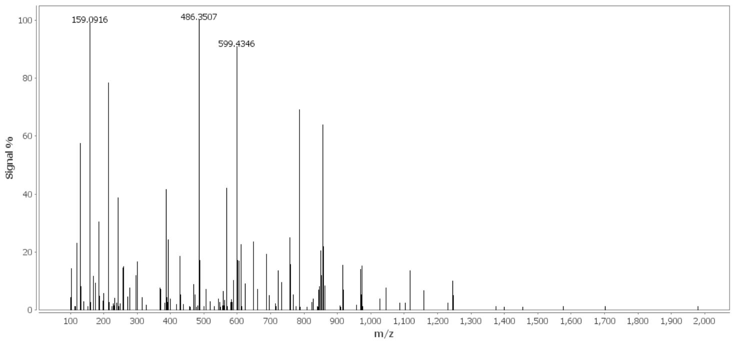

Metabolite: M10 -1396 RT=5.27

Type	score	sub. m/z observed	sub. m/z calculated	sub ppm		met. m/z observed	met. m/z calculated	met. ppm
MATCH	101.0	668.5496	668.5492	-0.61	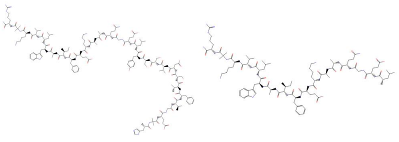	486.5369	486.5361	-1.74
MATCH	101.0	668.5496	668.5492	-0.61	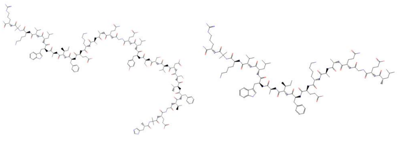	486.5369	486.5361	-1.74
MATCH	101.0	668.5496	668.5492	-0.61	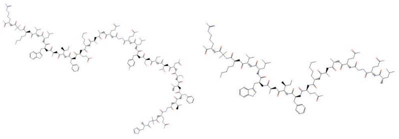	486.5369	486.5361	-1.74
MATCH	53.3	668.5496	668.5492	-0.61	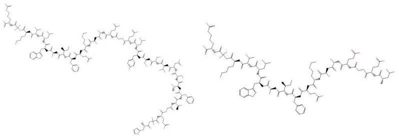	648.3786	648.3790	0.60
MATCH	53.3	668.5496	668.5492	-0.61	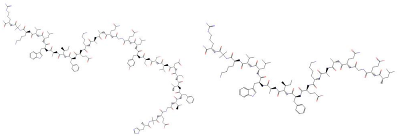	648.3786	648.3790	0.60
MATCH	53.3	668.5496	668.5492	-0.61	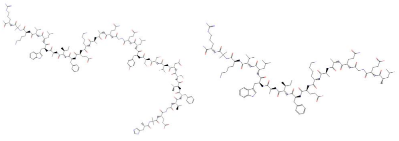	648.3786	648.3790	0.60
MATCH	13.9	668.5496	668.5492	-0.61	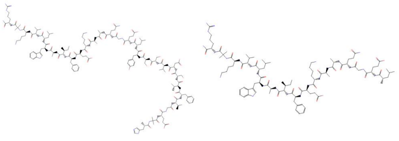	972.0641	972.0649	0.82

Metabolite: M10 -1396 RT=5.27

Type	score	sub. m/z observed	sub. m/z calculated	sub ppm		met. m/z observed	met. m/z calculated	met. ppm
MATCH	13.9	668.5496	668.5492	-0.61	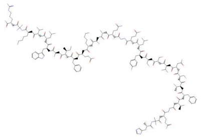 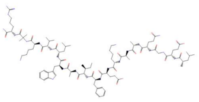	972.0641	972.0649	0.82
MATCH	13.9	668.5496	668.5492	-0.61	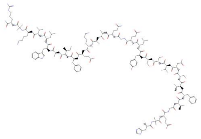 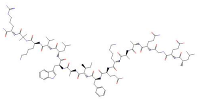	972.0641	972.0649	0.82
MATCH	132.5	835.4371	835.4347	-2.91	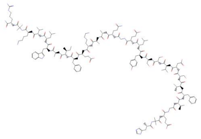 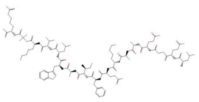	486.5369	486.5361	-1.74
MATCH	132.5	835.4371	835.4347	-2.91	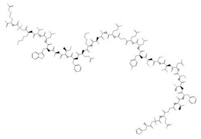 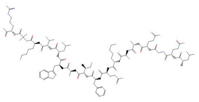	486.5369	486.5361	-1.74
MATCH	132.5	835.4371	835.4347	-2.91	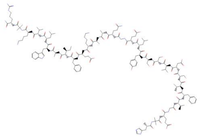 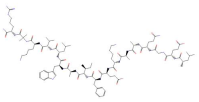	486.5369	486.5361	-1.74
MATCH	84.8	835.4371	835.4347	-2.91	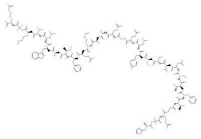 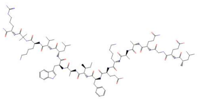	648.3786	648.3790	0.60
MATCH	84.8	835.4371	835.4347	-2.91	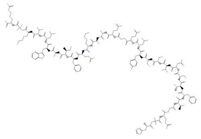 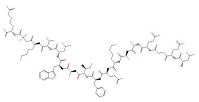	648.3786	648.3790	0.60
MATCH	84.8	835.4371	835.4347	-2.91	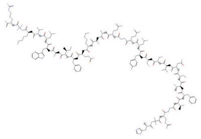 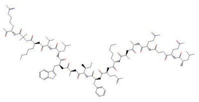	648.3786	648.3790	0.60
MATCH	45.4	835.4371	835.4347	-2.91	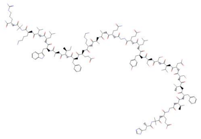 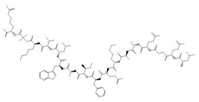	972.0641	972.0649	0.82

Metabolite: M10 -1396 RT=5.27

Type	score	sub. m/z observed	sub. m/z calculated	sub ppm		met. m/z observed	met. m/z calculated	met. ppm
MATCH	45.4	835.4371	835.4347	-2.91	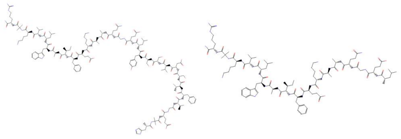	972.0641	972.0649	0.82
MATCH	45.4	835.4371	835.4347	-2.91	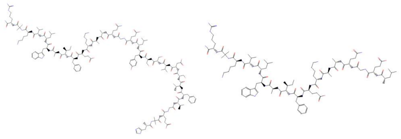	972.0641	972.0649	0.82
MATCH	97.4	1113.5734	1113.5771	3.31	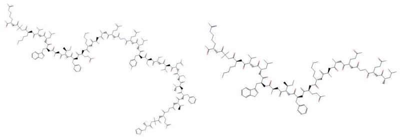	486.5369	486.5361	-1.74
MATCH	97.4	1113.5734	1113.5771	3.31	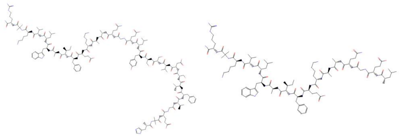	486.5369	486.5361	-1.74
MATCH	97.4	1113.5734	1113.5771	3.31	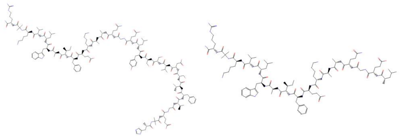	486.5369	486.5361	-1.74
MATCH	49.7	1113.5734	1113.5771	3.31	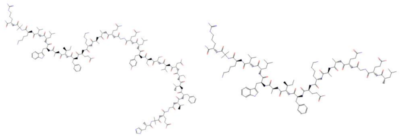	648.3786	648.3790	0.60
MATCH	49.7	1113.5734	1113.5771	3.31	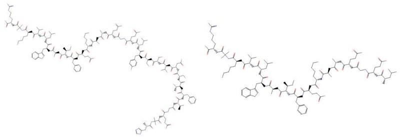	648.3786	648.3790	0.60
MATCH	49.7	1113.5734	1113.5771	3.31	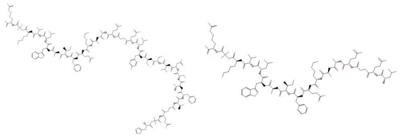	648.3786	648.3790	0.60
MATCH	10.3	1113.5734	1113.5771	3.31	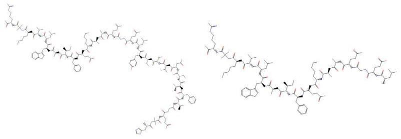	972.0641	972.0649	0.82

Metabolite: M10 -1396 RT=5.27

Type	score	sub. m/z observed	sub. m/z calculated	sub ppm		met. m/z observed	met. m/z calculated	met. ppm
MATCH	10.3	1113.5734	1113.5771	3.31	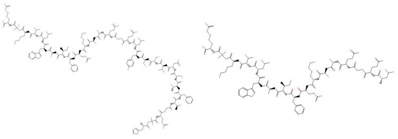	972.0641	972.0649	0.82
MATCH	10.3	1113.5734	1113.5771	3.31	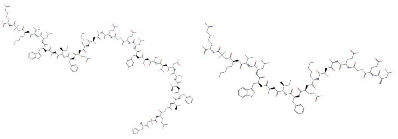	972.0641	972.0649	0.82
MATCH	15.5	102.0552	102.0588	35.30	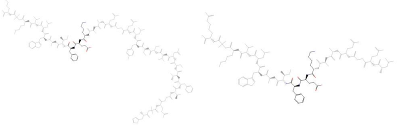	102.0553	102.0588	33.76
MATCH	15.5	102.0552	102.0588	35.30	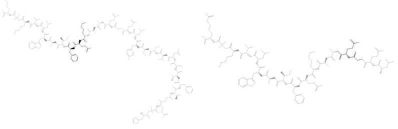	102.0553	102.0606	51.42
MATCH	15.5	102.0552	102.0606	52.97	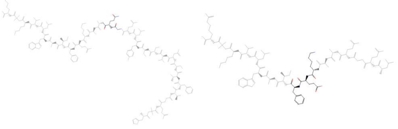	102.0553	102.0588	33.76
MATCH	40.2	120.0808	120.0731	-63.5	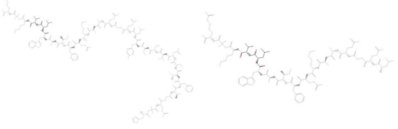	120.0809	120.0731	-64.6
MATCH	40.2	120.0808	120.0808	-0.05	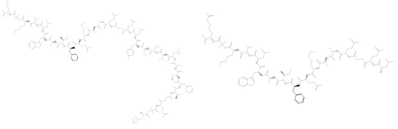	120.0809	120.0808	-1.17
MATCH	40.2	120.0808	120.0865	47.43	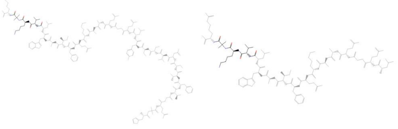	120.0809	120.0865	46.32
MATCH	66.9	129.1021	129.1022	0.79	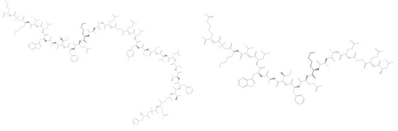	129.1023	129.1022	-0.41

Metabolite: M10 -1396 RT=5.27

Type	score	sub. m/z observed	sub. m/z calculated	sub ppm		met. m/z observed	met. m/z calculated	met. ppm
MATCH	66.9	129.1021	129.1022	0.79	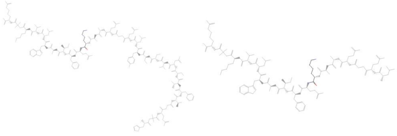	129.1023	129.1022	-0.41
MATCH	66.9	129.1021	129.1022	0.79	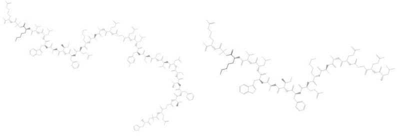	129.1023	129.1022	-0.41
MATCH	66.9	129.1021	129.1022	0.79	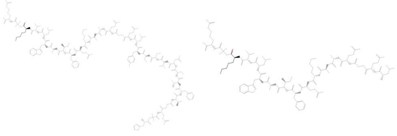	129.1023	129.1022	-0.41
MATCH	31.9	186.0864	186.0873	4.77	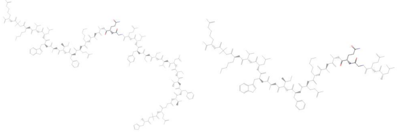	186.0872	186.0873	0.77
MATCH	31.9	186.0864	186.0873	4.77	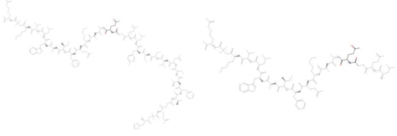	186.0872	186.0873	0.77
MATCH	79.6	215.1383	215.1357	-12.2	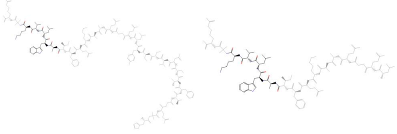	215.1388	215.1357	-14.4
MATCH	79.6	215.1383	215.1390	3.14	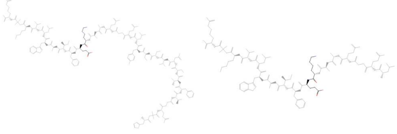	215.1388	215.1390	0.84
MATCH	40.7	243.1333	243.1339	2.72	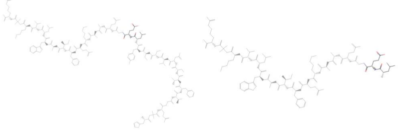	243.1337	243.1339	0.99
					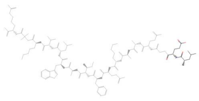	243.1337	243.1339	0.99

Metabolite: M10 -1396 RT=5.27

Type	score	sub. m/z observed	sub. m/z calculated	sub ppm		met. m/z observed	met. m/z calculated	met. ppm
MATCH	7.2	257.1239	257.1244	2.20	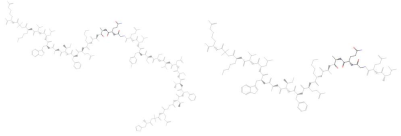	129.0659	129.0659	0.02
MATCH	7.2	257.1239	257.1244	2.20	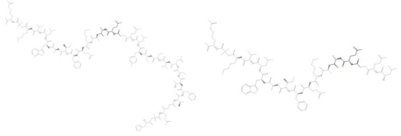	129.0659	129.0659	0.02
MATCH	16.0	257.1239	257.1244	2.20	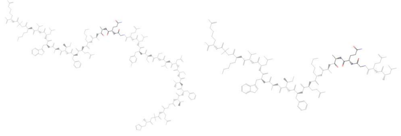	257.1240	257.1244	1.75
MATCH	16.0	257.1239	257.1244	2.20	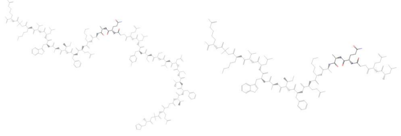	257.1240	257.1244	1.75
MATCH	16.3	259.1870	259.1877	2.62	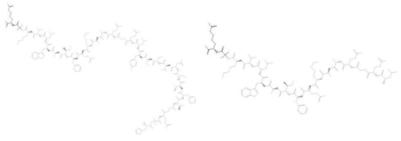	259.1873	259.1877	1.69
MATCH	13.4	297.1187	297.1193	2.22	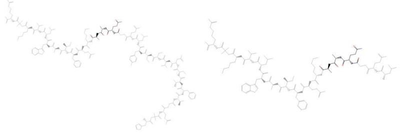	297.1192	297.1193	0.53
MATCH	5.3	315.1288	315.1299	3.46	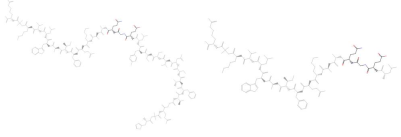	315.1294	315.1299	1.50
MATCH	5.3	315.1288	315.1299	3.46	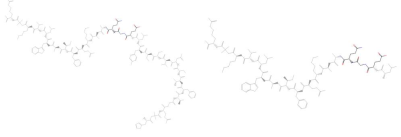	315.1294	315.1299	1.50
MATCH	46.1	387.2815	387.2827	3.02	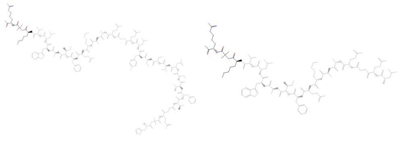	387.2822	387.2827	1.14

Metabolite: M10 -1396 RT=5.27

Type	score	sub. m/z observed	sub. m/z calculated	sub ppm		met. m/z observed	met. m/z calculated	met. ppm
MATCH	26.2	393.2598	393.2609	2.83	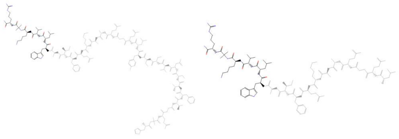	393.2606	393.2609	0.75
MATCH	20.1	428.7782	428.7794	2.80	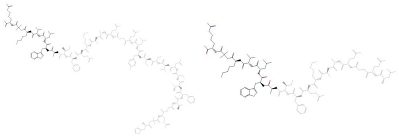	428.7793	428.7794	0.37
MATCH	109.1	486.3500	486.3511	2.28	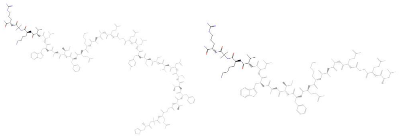	486.3507	486.3511	0.78
MATCH	43.3	567.6678	567.6701	4.15	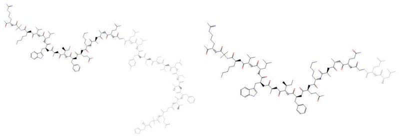	567.6695	567.6701	1.06
MATCH	11.4	589.3329	589.3344	2.62	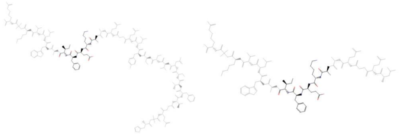	589.3332	589.3344	2.06
MATCH	11.4	589.3329	589.3344	2.62	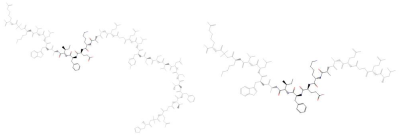	589.3332	589.3344	2.06
MATCH	11.4	589.3329	589.3344	2.62	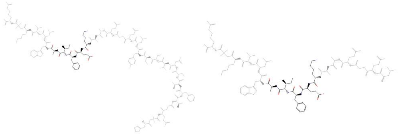	589.3332	589.3344	2.06
MATCH	11.4	589.3329	589.3344	2.62	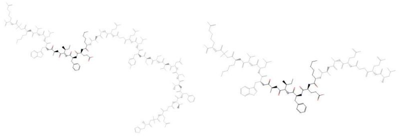	589.3332	589.3344	2.06
MATCH	100.2	599.4338	599.4351	2.24	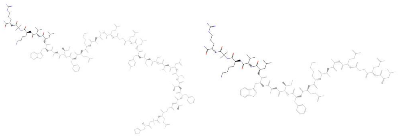	599.4346	599.4351	0.89

Metabolite: M10 -1396 RT=5.27

Type	score	sub. m/z observed	sub. m/z calculated	sub ppm		met. m/z observed	met. m/z calculated	met. ppm
MATCH	25.1	610.6825	610.6843	2.98	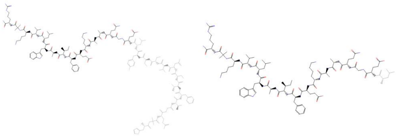	610.6834	610.6843	1.60
MATCH	21.9	687.4217	687.4244	3.99	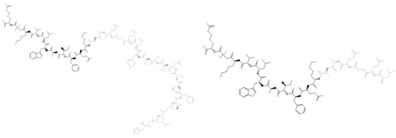	687.4233	687.4244	1.62
MATCH	16.8	722.9408	722.9430	2.97	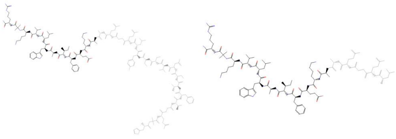	722.9423	722.9430	0.90
MATCH	27.4	758.4573	758.4616	5.67	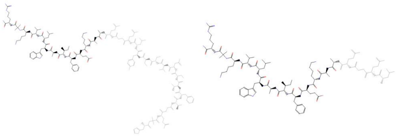	758.4599	758.4616	2.21
MATCH	79.6	785.5122	785.5145	2.93	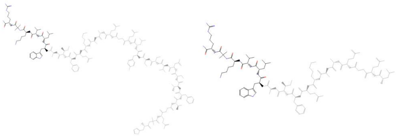	785.5134	785.5145	1.33
MATCH	3.6	822.4887	822.4908	2.58	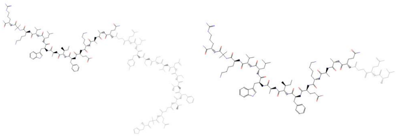	822.4889	822.4908	2.41
MATCH	31.2	850.9987	851.0016	3.36	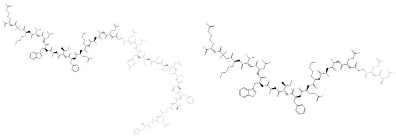	850.9997	851.0016	2.18
MATCH	78.4	856.5492	856.5516	2.77	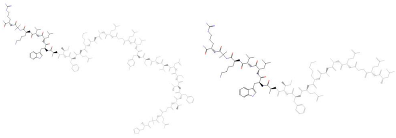	856.5503	856.5516	1.49
MATCH	18.1	906.5161	906.5176	1.60	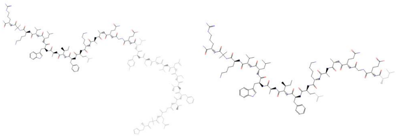	604.6794	604.6808	2.42

Metabolite: M10 -1396 RT=5.27

Type	score	sub. m/z observed	sub. m/z calculated	sub ppm		met. m/z observed	met. m/z calculated	met. ppm
MATCH	18.1	906.5161	906.5176	1.60	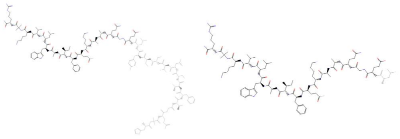	604.6794	604.6808	2.42
MATCH	18.1	906.5161	906.5176	1.60	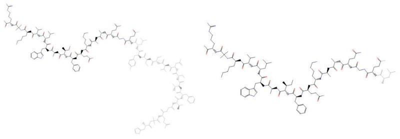	604.6794	604.6808	2.42
MATCH	18.1	906.5161	906.5176	1.60	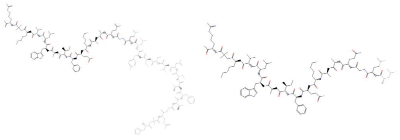	604.6794	604.6808	2.42
MATCH	14.5	915.5203	915.5229	2.76	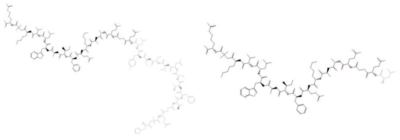	915.5203	915.5229	2.85
MATCH	19.7	969.6332	969.6356	2.54	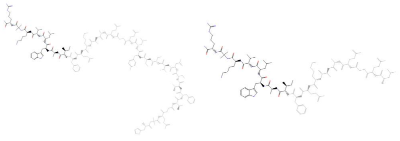	969.6353	969.6356	0.30
MATCH	74.4	1113.5749	1113.5771	2.03	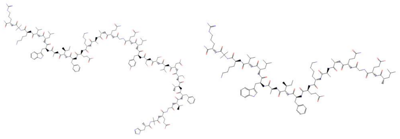	648.3782	648.3790	1.32
MATCH	56.2	1113.5749	1113.5771	2.03	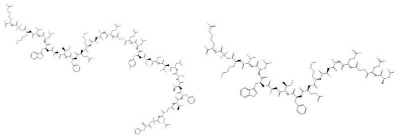	972.0611	972.0649	3.92
MATCH	21.5	1116.7004	1116.7040	3.23	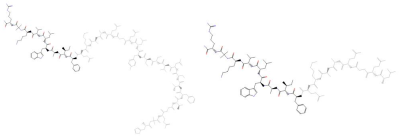	1116.7021	1116.7040	1.71
MATCH	19.3	1245.7435	1245.7466	2.52	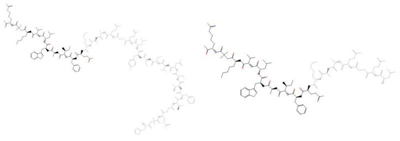	1245.7470	1245.7466	-0.31

Metabolite: M10 -1396 RT=5.27

Type	score	sub. m/z observed	sub. m/z calculated	sub ppm			met. m/z observed	met. m/z calculated	met. ppm
MATCH	40.7	1638.7199	1638.7282	5.06	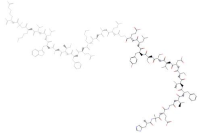	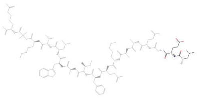	243.1337	243.1339	0.99
						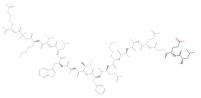	243.1337	243.1339	0.99
MISMATCH	-15.5	102.0552	102.0550	-2.07	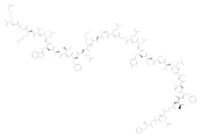		102.0553	102.0553	0.00
MISMATCH	-31.9	186.0864	186.0855	-4.92	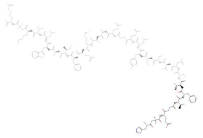		186.0872	186.0872	0.00
MISMATCH	-3.4	249.1229	249.1234	2.01	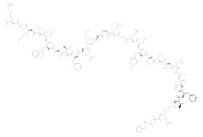		249.1229	249.1229	0.00
MISMATCH	-5.3	315.1288	315.1313	7.71	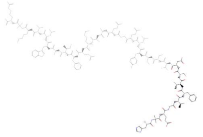		315.1294	315.1294	0.00
MISMATCH	-5.1	393.2598	393.2609	2.83	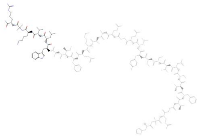		197.1285	197.1285	0.00
MISMATCH	-15.7	486.3500	486.3511	2.28	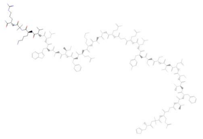		243.6787	243.6787	0.00
MISMATCH	-26.0	599.4338	599.4351	2.24	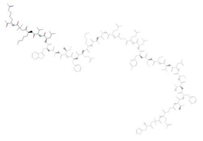		300.2208	300.2208	0.00

Metabolite: M10 -1396 RT=5.27

Type	score	sub. m/z observed	sub. m/z calculated	sub ppm		met. m/z observed	met. m/z calculated	met. ppm
MISMATCH	-4.6	722.9408	722.9430	2.97	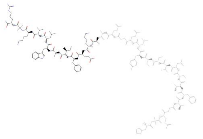	482.2979	482.2979	0.00
MISMATCH	-9.7	758.4573	758.4616	5.67	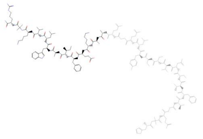	505.9764	505.9764	0.00
MISMATCH	-13.8	785.5122	785.5145	2.93	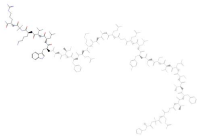	197.1285	197.1285	0.00
MISMATCH	-2.4	947.9247	947.9263	1.74	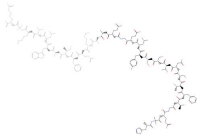	499.2517	499.2517	0.00
MISMATCH	-7.5	969.6332	969.6356	2.54	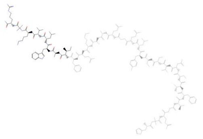	485.3222	485.3222	0.00
MISMATCH	-5.0	1112.0134	1112.0137	0.21	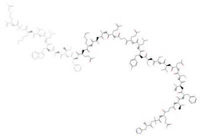	827.4252	827.4252	0.00
MISMATCH	-14.2	1116.7004	1116.7040	3.23	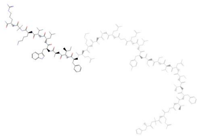	558.8547	558.8547	0.00
MISMATCH	-18.3	1245.7435	1245.7466	2.52	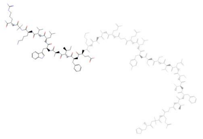	623.3757	623.3757	0.00
MISMATCH	-5.9	1444.8767	1444.8787	1.42	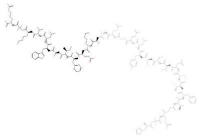	482.2979	482.2979	0.00

Metabolite: M10 -1396 RT=5.27

Type	score	sub. m/z observed	sub. m/z calculated	sub ppm		met. m/z observed	met. m/z calculated	met. ppm
MISMATCH	-10.5	1515.9113	1515.9158	2.97	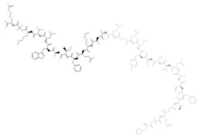	505.9764	505.9764	0.00
MET_MATCH					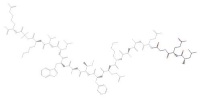	159.0916	159.0946	19.21
MET_MATCH					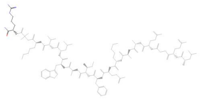	174.1346	174.1349	1.87
MET_MATCH					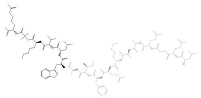	197.1285	197.1341	28.13
MET_MATCH					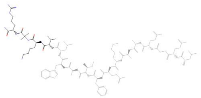	243.6787	243.6792	1.97
MET_MATCH					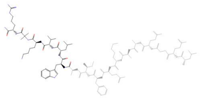	271.1763	271.1694	-25.2
MET_MATCH					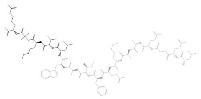	300.2208	300.2212	1.20
MET_MATCH					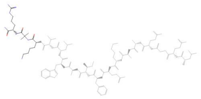	370.2557	370.2561	1.12
MET_MATCH					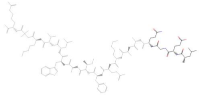	400.2185	400.2191	1.29

Metabolite: M10 -1396 RT=5.27

Type	score	sub. m/z observed	sub. m/z calculated	sub ppm		met. m/z observed	met. m/z calculated	met. ppm
MET_MATCH					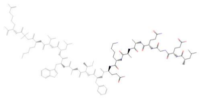	400.2185	400.2191	1.29
MET_MATCH					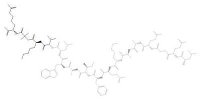	469.3232	469.3245	2.78
MET_MATCH					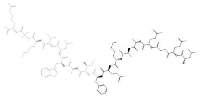	473.7533	473.7533	-0.16
MET_MATCH					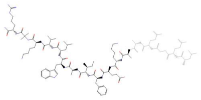	482.2979	482.2978	-0.39
MET_MATCH					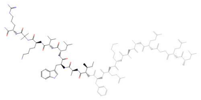	485.3222	485.3215	-1.51
MET_MATCH					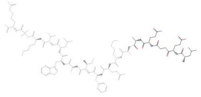	499.2517	499.2511	-1.30
MET_MATCH					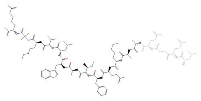	505.9764	505.9768	0.75
MET_MATCH					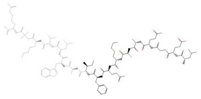	530.2948	530.2953	0.94
MET_MATCH					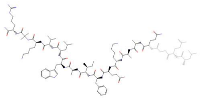	542.9876	542.9875	-0.26

Metabolite: M10 -1396 RT=5.27

Type	score	sub. m/z observed	sub. m/z calculated	sub ppm		met. m/z observed	met. m/z calculated	met. ppm
MET_MATCH					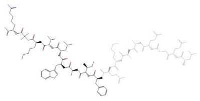	558.8547	558.8557	1.77
MET_MATCH					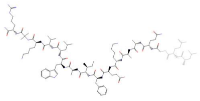	561.9946	561.9946	0.11
MET_MATCH					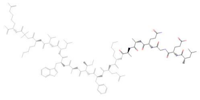	570.2864	570.2882	3.20
MET_MATCH					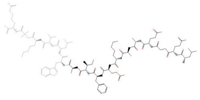	579.8083	579.8113	5.22
MET_MATCH					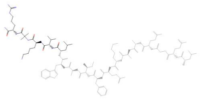	582.4071	582.4086	2.55
MET_MATCH					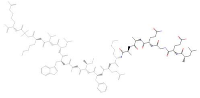	585.2988	585.2991	0.47
MET_MATCH					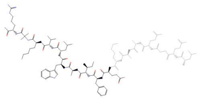	623.3757	623.3770	2.09
MET_MATCH					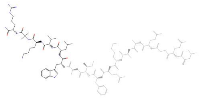	768.4878	768.4879	0.11
MET_MATCH					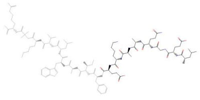	827.4252	827.4258	0.62

Metabolite: M10 -1396 RT=5.27

Type	score	sub. m/z observed	sub. m/z calculated	sub ppm		met. m/z observed	met. m/z calculated	met. ppm
MET_MATCH					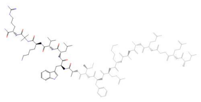	839.5229	839.5250	2.54
MET_MATCH					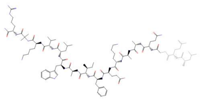	842.4868	842.4883	1.83
MET_MATCH					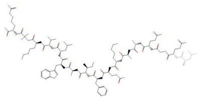	907.0153	907.0096	-6.24
MET_MATCH					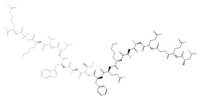	974.4913	974.4942	2.98
MET_MATCH					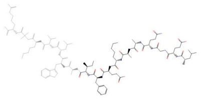	1087.5771	1087.5782	1.00
MET_MATCH					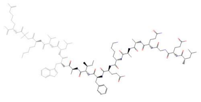	1158.6090	1158.6154	5.44

MS (+) FT

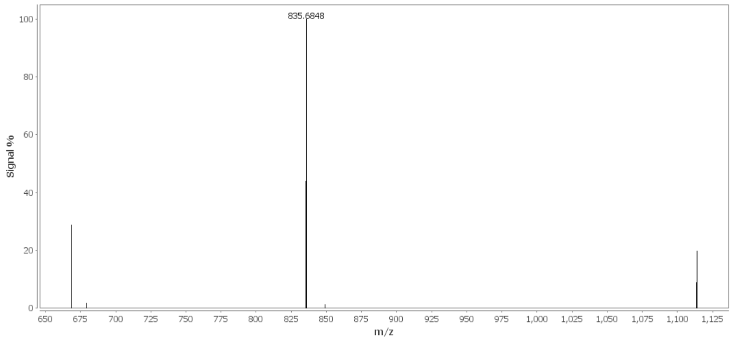

MS (+) FT

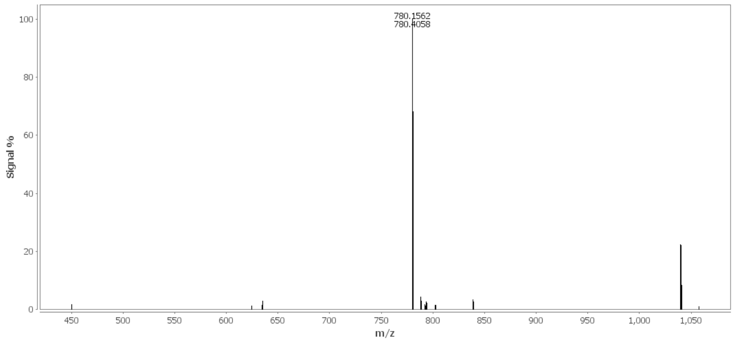

MS2 (+) FT activ = HCD:ce =

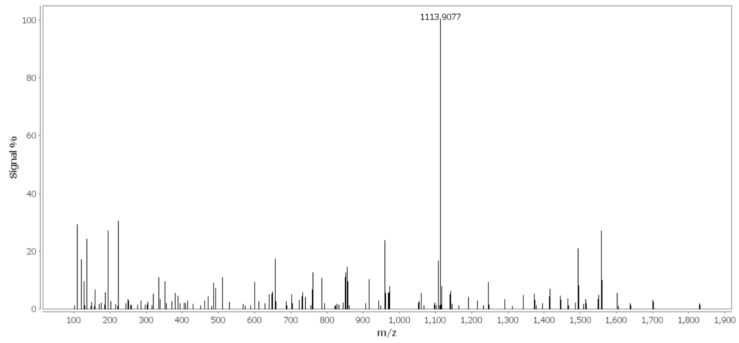

MS2 (+) FT activ = HCD:ce =

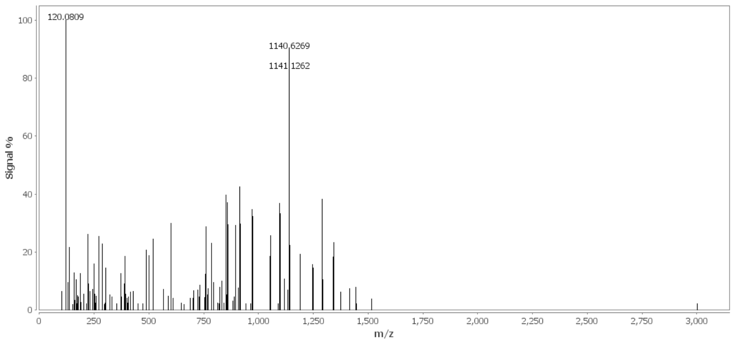

Metabolite: M13 -222 RT=6.77

Type	score	sub. m/z observed	sub. m/z calculated	sub ppm		met. m/z observed	met. m/z calculated	met. ppm
MATCH	63.2	668.5496	668.5492	-0.61	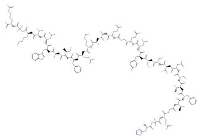 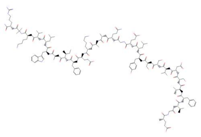	779.9066	779.9067	0.14
MATCH	63.2	668.5496	668.5492	-0.61	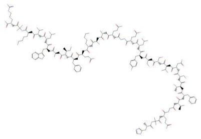 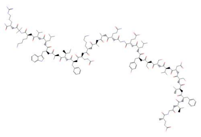	779.9066	779.9067	0.14
					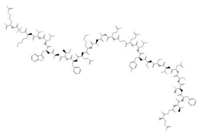	779.9066	779.9067	0.14
MATCH	23.4	668.5496	668.5492	-0.61	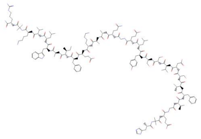 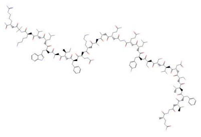	1039.5383	1039.5399	1.54
MATCH	23.4	668.5496	668.5492	-0.61	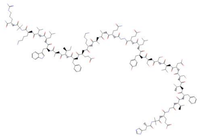 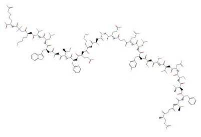	1039.5383	1039.5399	1.54
					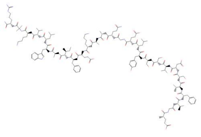	1039.5383	1039.5399	1.54
MATCH	94.7	835.4371	835.4347	-2.91	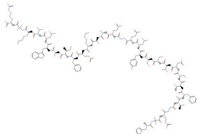 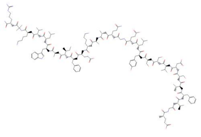	779.9066	779.9067	0.14

Metabolite: M13 -222 RT=6.77

Type	score	sub. m/z observed	sub. m/z calculated	sub ppm			met. m/z observed	met. m/z calculated	met. ppm
MATCH	94.7	835.4371	835.4347	-2.91	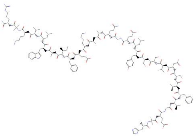	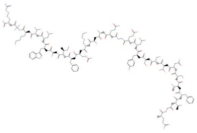	779.9066	779.9067	0.14
						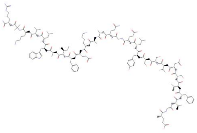	779.9066	779.9067	0.14
MATCH	54.9	835.4371	835.4347	-2.91	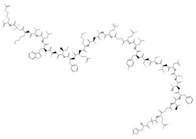	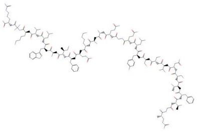	1039.5383	1039.5399	1.54
MATCH	54.9	835.4371	835.4347	-2.91	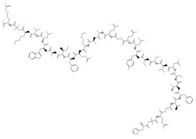	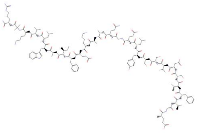	1039.5383	1039.5399	1.54
						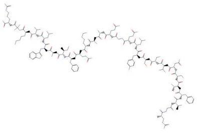	1039.5383	1039.5399	1.54
MATCH	59.6	1113.5734	1113.5771	3.31	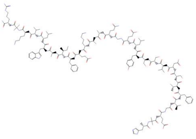	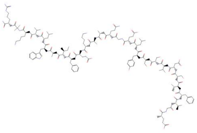	779.9066	779.9067	0.14
MATCH	59.6	1113.5734	1113.5771	3.31	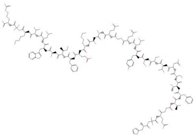	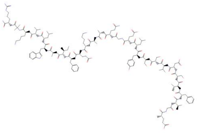	779.9066	779.9067	0.14
						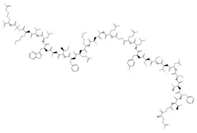	779.9066	779.9067	0.14
MATCH	19.8	1113.5734	1113.5771	3.31	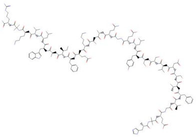	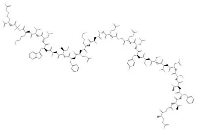	1039.5383	1039.5399	1.54

Metabolite: M13 -222 RT=6.77

Type	score	sub. m/z observed	sub. m/z calculated	sub ppm		met. m/z observed	met. m/z calculated	met. ppm
MATCH	19.8	1113.5734	1113.5771	3.31	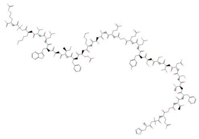 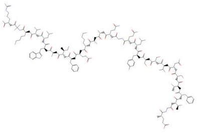	1039.5383	1039.5399	1.54
					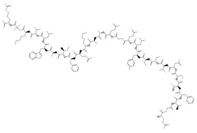	1039.5383	1039.5399	1.54
MATCH	7.7	102.0552	102.0550	-2.07	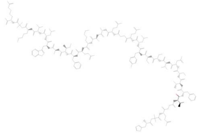 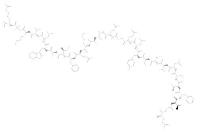	102.0552	102.0550	-2.39
MATCH	7.7	102.0552	102.0550	-2.07	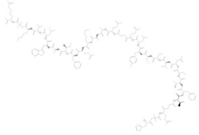 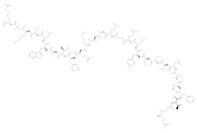	102.0552	102.0550	-2.39
MATCH	7.7	102.0552	102.0550	-2.07	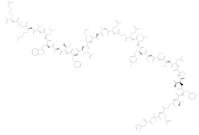 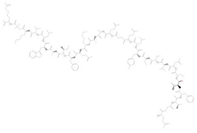	102.0552	102.0550	-2.39
MATCH	7.7	102.0552	102.0550	-2.07	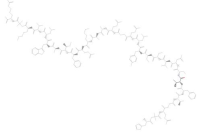 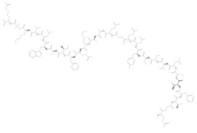	102.0552	102.0550	-2.39
MATCH	7.7	102.0552	102.0588	35.30	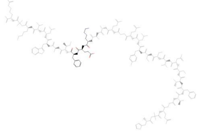 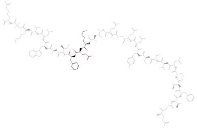	102.0552	102.0588	34.98
MATCH	7.7	102.0552	102.0588	35.30	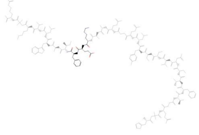 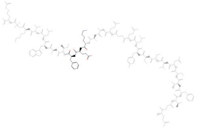	102.0552	102.0588	34.98
MATCH	7.7	102.0552	102.0606	52.97	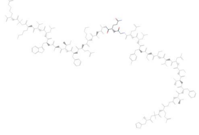 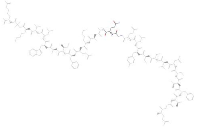	102.0552	102.0606	52.65

Metabolite: M13 -222 RT=6.77

Type	score	sub. m/z observed	sub. m/z calculated	sub ppm			met. m/z observed	met. m/z calculated	met. ppm
MATCH	117.2	120.0808	120.0731	-63.5	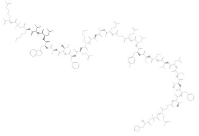	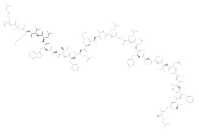	120.0809	120.0731	-64.7
MATCH	117.2	120.0808	120.0808	-0.05	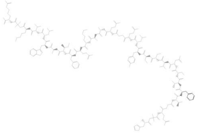	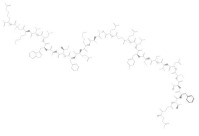	120.0809	120.0808	-1.21
MATCH	117.2	120.0808	120.0808	-0.05	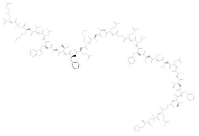	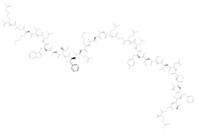	120.0809	120.0808	-1.21
MATCH	117.2	120.0808	120.0865	47.43	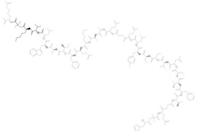	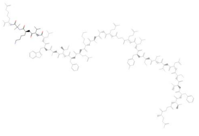	120.0809	120.0865	46.27
MATCH	19.1	129.1021	129.1022	0.79	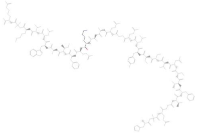	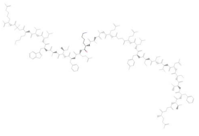	129.1023	129.1022	-0.77
MATCH	19.1	129.1021	129.1022	0.79	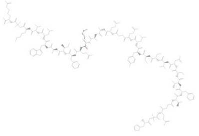	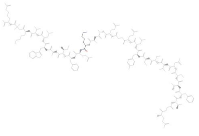	129.1023	129.1022	-0.77
MATCH	19.1	129.1021	129.1022	0.79	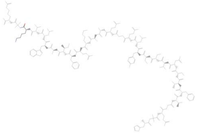	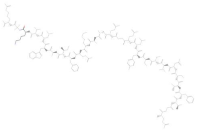	129.1023	129.1022	-0.77
MATCH	19.1	129.1021	129.1022	0.79	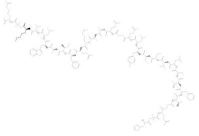	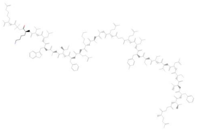	129.1023	129.1022	-0.77
MATCH	45.9	136.0755	136.0693	-45.4	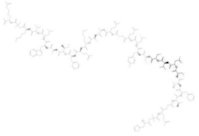	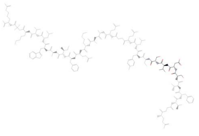	136.0756	136.0693	-46.4

Metabolite: M13 -222 RT=6.77

Type	score	sub. m/z observed	sub. m/z calculated	sub ppm		met. m/z observed	met. m/z calculated	met. ppm
MATCH	45.9	136.0755	136.0693	-45.4	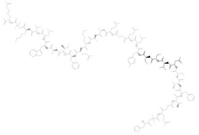 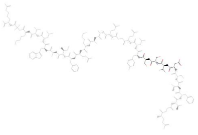	136.0756	136.0693	-46.4
MATCH	45.9	136.0755	136.0706	-35.6	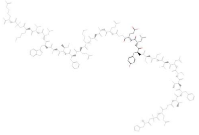 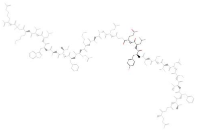	136.0756	136.0706	-36.5
MATCH	45.9	136.0755	136.0706	-35.6	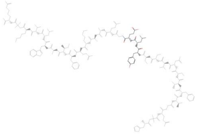 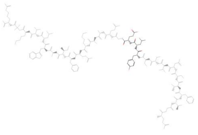	136.0756	136.0706	-36.5
MATCH	45.9	136.0755	136.0737	-13.0	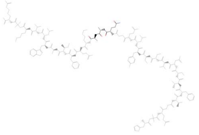 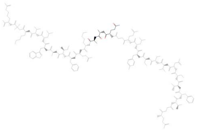	136.0756	136.0737	-13.9
MATCH	45.9	136.0755	136.0737	-13.0	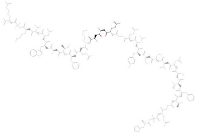 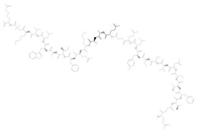	136.0756	136.0737	-13.9
MATCH	2.9	157.0604	157.0608	2.08	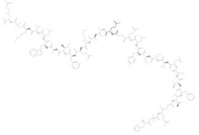 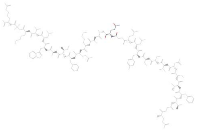	157.0604	157.0608	2.19
MATCH	19.6	159.0913	159.0946	20.67	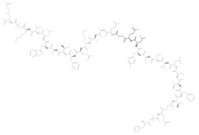 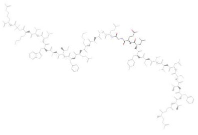	159.0916	159.0946	19.13
MATCH	3.4	186.0864	186.0873	4.77	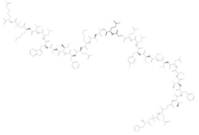 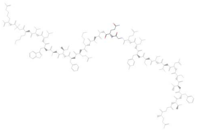	186.0869	186.0873	2.09
MATCH	3.4	186.0864	186.0873	4.77	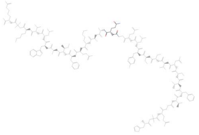 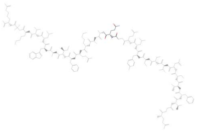	186.0869	186.0873	2.09

Metabolite: M13 -222 RT=6.77

Type	score	sub. m/z observed	sub. m/z calculated	sub ppm		met. m/z observed	met. m/z calculated	met. ppm
MATCH	3.4	186.0864	186.0908	23.47	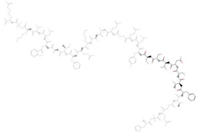 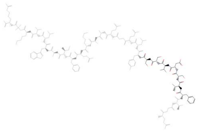	186.0869	186.0908	20.79
MATCH	17.7	187.1073	187.1077	2.11	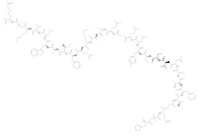 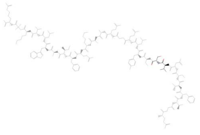	187.1077	187.1077	0.28
MATCH	17.7	187.1073	187.1077	2.11	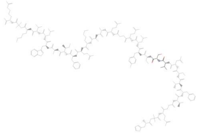 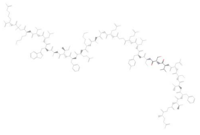	187.1077	187.1077	0.28
MATCH	8.0	203.0657	203.0662	2.70	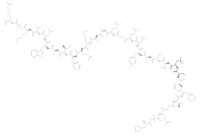 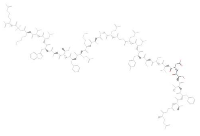	203.0660	203.0662	1.10
MATCH	8.0	203.0657	203.0662	2.70	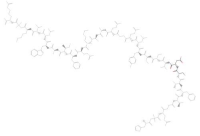 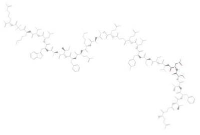	203.0660	203.0662	1.10
MATCH	3.8	215.1021	215.1026	2.41	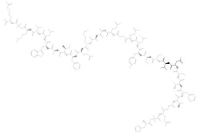 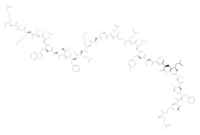	215.1021	215.1026	2.49
MATCH	3.8	215.1021	215.1026	2.41	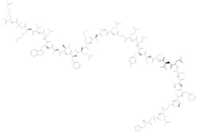 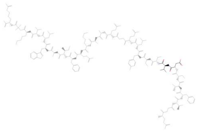	215.1021	215.1026	2.49
MATCH	27.1	221.1278	221.1285	2.74	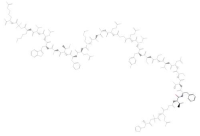 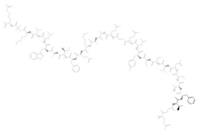	221.1283	221.1285	0.51
MATCH	27.1	221.1278	221.1285	2.74	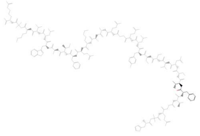 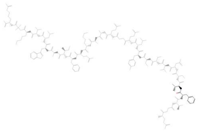	221.1283	221.1285	0.51

Metabolite: M13 -222 RT=6.77

Type	score	sub. m/z observed	sub. m/z calculated	sub ppm			met. m/z observed	met. m/z calculated	met. ppm
MATCH	17.3	249.1229	249.1234	2.01	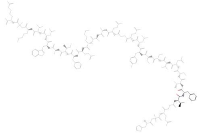	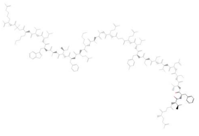	249.1229	249.1234	1.69
MATCH	17.3	249.1229	249.1234	2.01	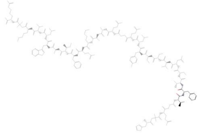	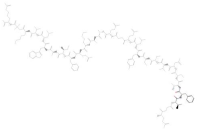	249.1229	249.1234	1.69
MATCH	17.3	249.1229	249.1234	2.01	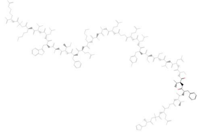	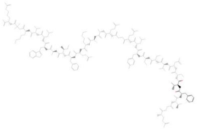	249.1229	249.1234	1.69
MATCH	17.3	249.1229	249.1234	2.01	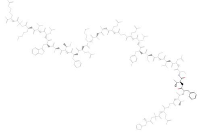	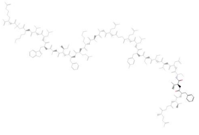	249.1229	249.1234	1.69
MATCH	3.8	257.1239	257.1244	2.20	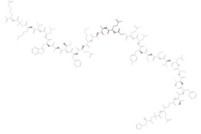	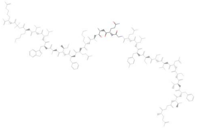	257.1233	257.1244	4.55
MATCH	3.8	257.1239	257.1244	2.20	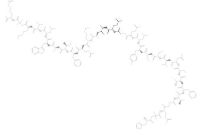	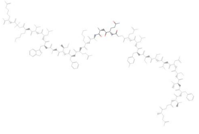	257.1233	257.1244	4.55
MATCH	3.8	257.1239	257.1291	20.25	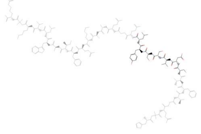	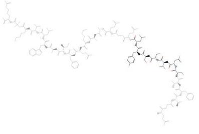	257.1233	257.1291	22.60
MATCH	6.0	259.1870	259.1877	2.62	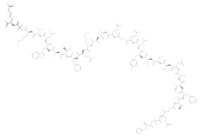	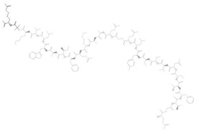	259.1868	259.1877	3.36
MATCH	9.7	286.1027	286.1034	2.41	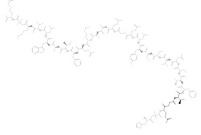	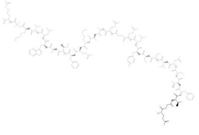	286.1028	286.1034	1.81

Metabolite: M13 -222 RT=6.77

Type	score	sub. m/z observed	sub. m/z calculated	sub ppm			met. m/z observed	met. m/z calculated	met. ppm
MATCH	3.5	297.1187	297.1193	2.22	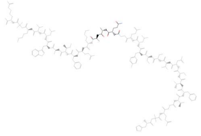	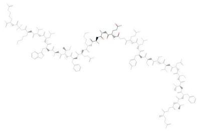	297.1183	297.1193	3.46
MATCH	10.5	302.1338	302.1347	2.91	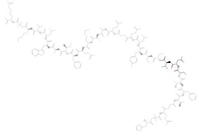	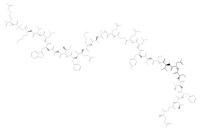	302.1343	302.1347	1.31
MATCH	10.5	302.1338	302.1347	2.91	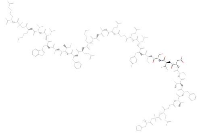	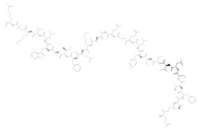	302.1343	302.1347	1.31
MATCH	10.5	302.1338	302.1347	2.91	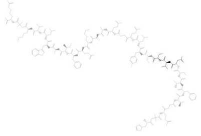	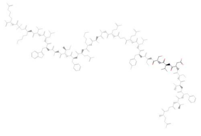	302.1343	302.1347	1.31
MATCH	10.5	302.1338	302.1397	19.74	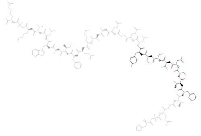	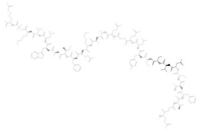	302.1343	302.1347	1.31
MATCH	10.5	302.1338	302.1347	2.91	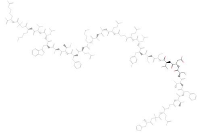	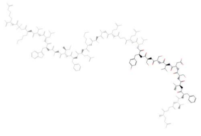	302.1343	302.1397	18.14
MATCH	16.9	304.1131	304.1139	2.82	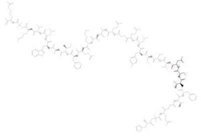	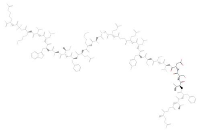	304.1135	304.1139	1.38
MATCH	16.9	304.1131	304.1139	2.82	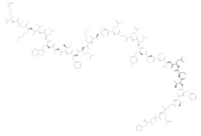	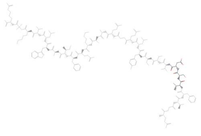	304.1135	304.1139	1.38
MATCH	10.7	320.1233	320.1241	2.47	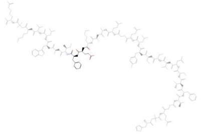	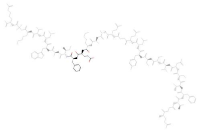	320.1234	320.1241	2.04

Metabolite: M13 -222 RT=6.77

Type	score	sub. m/z observed	sub. m/z calculated	sub ppm			met. m/z observed	met. m/z calculated	met. ppm
MATCH	13.6	387.2815	387.2827	3.02	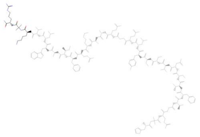	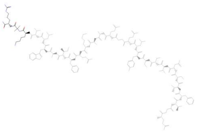	387.2820	387.2827	1.68
MATCH	7.5	393.2598	393.2609	2.83	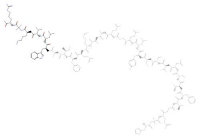	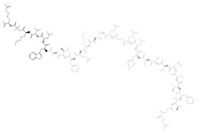	393.2603	393.2609	1.40
MATCH	14.6	409.1821	409.1830	2.21	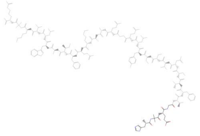	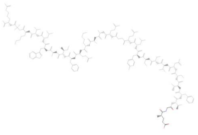	187.0710	187.0713	1.57
MATCH	8.0	428.7782	428.7794	2.80	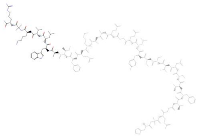	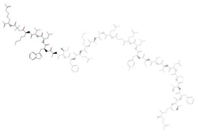	428.7784	428.7794	2.36
MATCH	29.7	486.3500	486.3511	2.28	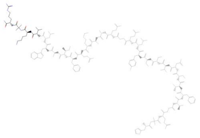	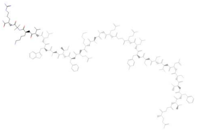	486.3510	486.3511	0.19
MATCH	33.7	510.2295	510.2307	2.28	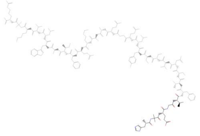	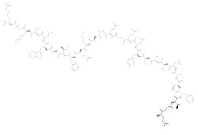	288.1190	288.1190	0.07
MATCH	8.5	567.6678	567.6701	4.15	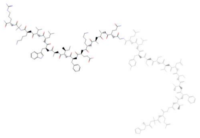	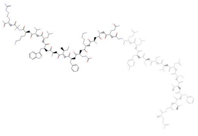	567.6684	567.6701	3.07
MATCH	3.2	589.3329	589.3344	2.62	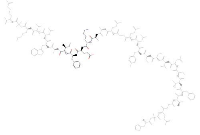	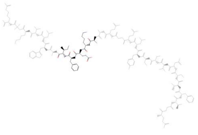	589.3329	589.3344	2.61
MATCH	3.2	589.3329	589.3344	2.62	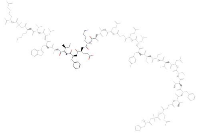	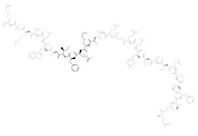	589.3329	589.3344	2.61

Metabolite: M13 -222 RT=6.77

Type	score	sub. m/z observed	sub. m/z calculated	sub ppm			met. m/z observed	met. m/z calculated	met. ppm
MATCH	3.2	589.3329	589.3344	2.62	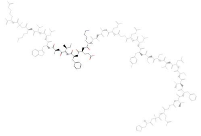	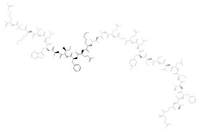	589.3329	589.3344	2.61
MATCH	3.2	589.3329	589.3344	2.62	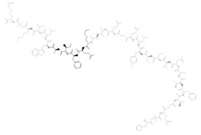	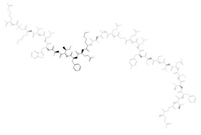	589.3329	589.3344	2.61
MATCH	39.4	599.4338	599.4351	2.24	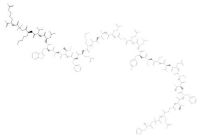	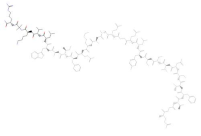	599.4342	599.4351	1.57
MATCH	6.6	610.6825	610.6843	2.98	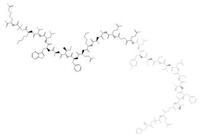	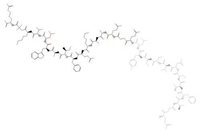	610.6838	610.6843	0.82
MATCH	6.6	629.3021	629.3042	3.35	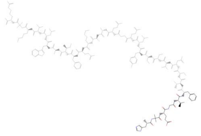	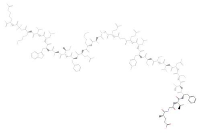	407.1914	407.1925	2.64
MATCH	11.3	639.2876	639.2885	1.49	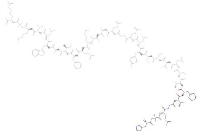	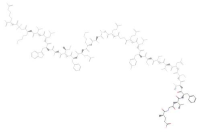	417.1761	417.1769	1.71
MATCH	7.7	648.3771	648.3790	3.00	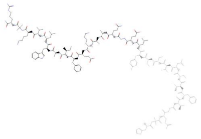	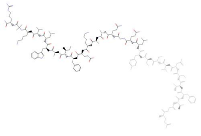	648.3780	648.3790	1.60
MATCH	6.9	687.4217	687.4244	3.99	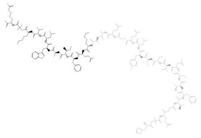	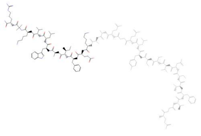	687.4242	687.4244	0.36
MATCH	8.3	702.7311	702.7335	3.31	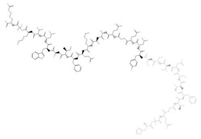	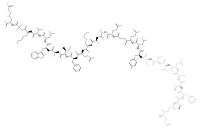	702.7327	702.7335	1.14

Metabolite: M13 -222 RT=6.77

Type	score	sub. m/z observed	sub. m/z calculated	sub ppm			met. m/z observed	met. m/z calculated	met. ppm
MATCH	10.2	722.9408	722.9430	2.97	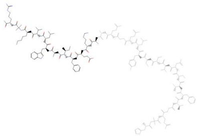	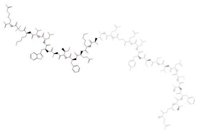	722.9406	722.9430	3.38
MATCH	10.0	731.7417	731.7441	3.31	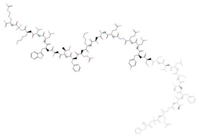	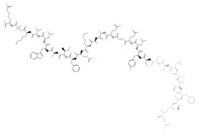	731.7432	731.7441	1.34
MATCH	28.4	740.3344	740.3362	2.49	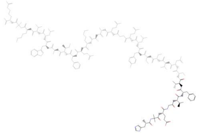	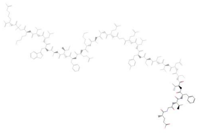	518.2245	518.2245	0.13
MATCH	28.4	740.3344	740.3362	2.49	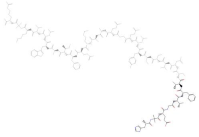	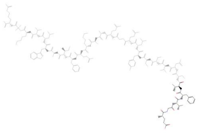	518.2245	518.2245	0.13
MATCH	5.6	755.0814	755.0793	-2.78	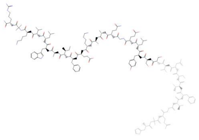	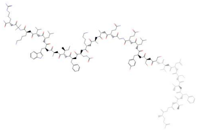	755.0822	755.0793	-3.77
MATCH	15.0	758.4573	758.4616	5.67	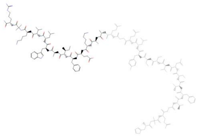	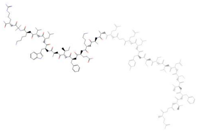	758.4609	758.4616	0.81
MATCH	28.0	760.7524	760.7548	3.17	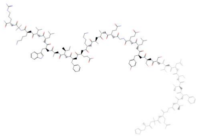	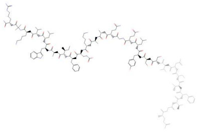	760.7535	760.7548	1.77
MATCH	33.7	785.5122	785.5145	2.93	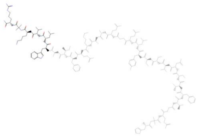	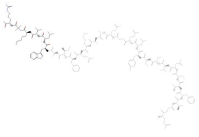	785.5128	785.5145	2.06
MATCH	10.7	793.7750	793.7776	3.28	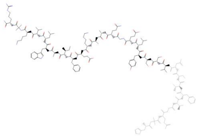	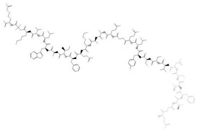	793.7767	793.7776	1.13

Metabolite: M13 -222 RT=6.77

Type	score	sub. m/z observed	sub. m/z calculated	sub ppm		met. m/z observed	met. m/z calculated	met. ppm
MATCH	8.9	822.4887	822.4908	2.58	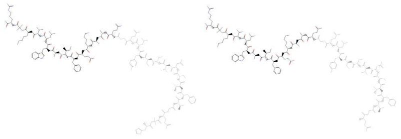	822.4910	822.4908	-0.23
MATCH	50.4	850.9987	851.0016	3.36	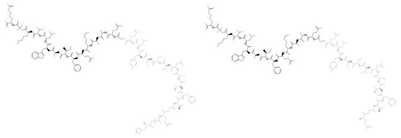	850.9999	851.0016	1.98
MATCH	51.4	856.5492	856.5516	2.77	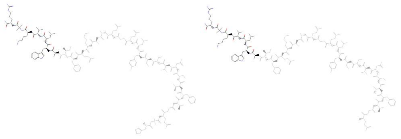	856.5510	856.5516	0.64
MATCH	7.2	906.5161	906.5176	1.60	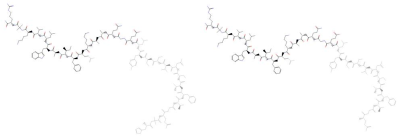	906.5143	906.5176	3.65
MATCH	7.2	906.5161	906.5176	1.60	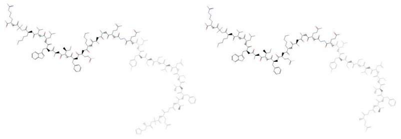	906.5143	906.5176	3.65
MATCH	7.2	906.5161	906.5176	1.60	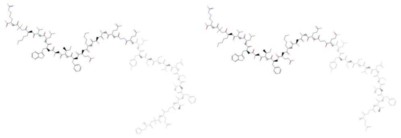	906.5143	906.5176	3.65
MATCH	7.2	906.5161	906.5176	1.60	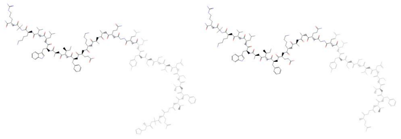	906.5143	906.5176	3.65
MATCH	52.7	915.5203	915.5229	2.76	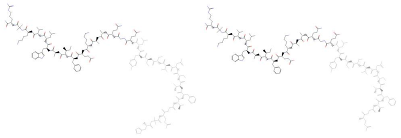	915.5218	915.5229	1.14
MATCH	19.0	969.6332	969.6356	2.54	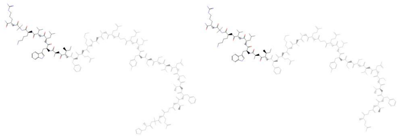	969.6371	969.6356	-1.53

Metabolite: M13 -222 RT=6.77

Type	score	sub. m/z observed	sub. m/z calculated	sub ppm		met. m/z observed	met. m/z calculated	met. ppm
MATCH	40.3	972.0625	972.0649	2.44	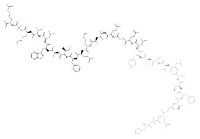 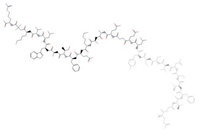	972.0622	972.0649	2.76
MATCH	20.8	1053.5927	1053.5966	3.63	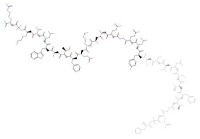 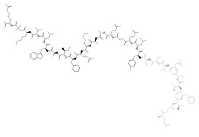	1053.5950	1053.5966	1.51
MATCH	38.2	1097.1096	1097.1126	2.69	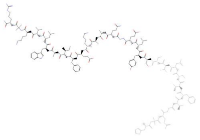 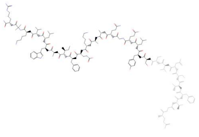	1097.1108	1097.1126	1.60
MATCH	18.5	1116.7004	1116.7040	3.23	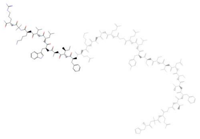 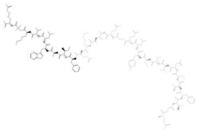	1116.7041	1116.7040	-0.09
MATCH	95.0	1140.6251	1140.6286	3.06	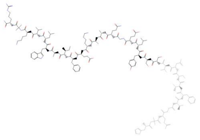 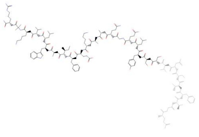	1140.6269	1140.6286	1.52
MATCH	22.8	1190.1592	1190.1628	3.04	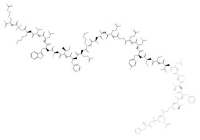 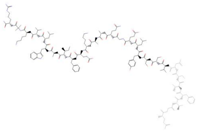	1190.1566	1190.1628	5.25
MATCH	25.0	1245.7435	1245.7466	2.52	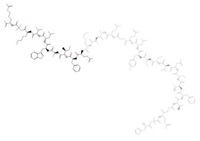 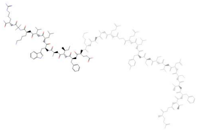	1245.7448	1245.7466	1.45
MATCH	24.8	1291.1879	1291.1923	3.43	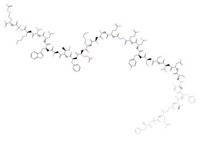 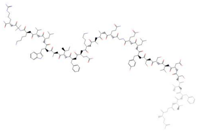	1291.1894	1291.1923	2.26
MATCH	22.7	1341.7122	1341.7161	2.93	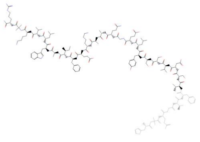 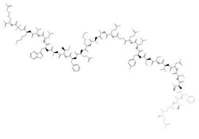	1341.7148	1341.7161	0.96

Metabolite: M13 -222 RT=6.77

Type	score	sub. m/z observed	sub. m/z calculated	sub ppm		met. m/z observed	met. m/z calculated	met. ppm
MATCH	11.4	1373.8392	1373.8416	1.75	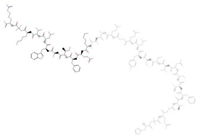 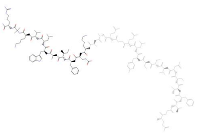	1373.8379	1373.8416	2.70
MATCH	12.4	1444.8767	1444.8787	1.42	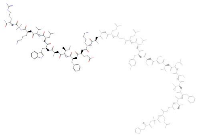 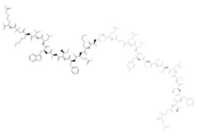	1444.8712	1444.8787	5.19
MATCH	7.3	1515.9113	1515.9158	2.97	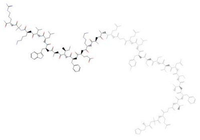 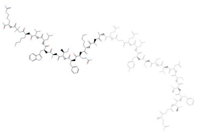	1515.9138	1515.9158	1.33
MISMATCH	-14.0	186.0864	186.0908	23.47	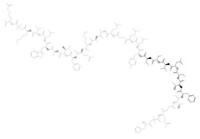	371.1733	371.1733	0.00
MISMATCH	-17.2	223.1071	223.1039	-14.3	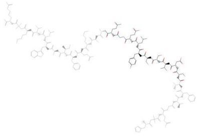	371.1733	371.1733	0.00
MISMATCH	-10.5	302.1338	302.1413	24.82	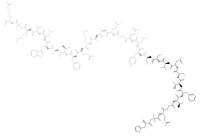	302.1343	302.1343	0.00
MISMATCH	-7.0	462.6949	462.7009	12.93	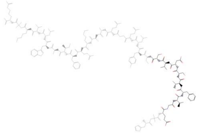	702.2702	702.2702	0.00
MISMATCH	-11.8	599.4338	599.4351	2.24	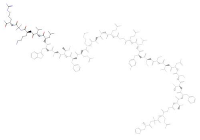	300.2209	300.2209	0.00
MISMATCH	-8.0	755.0814	755.0793	-2.78	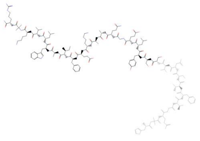	1132.1177	1132.1177	0.00

Metabolite: M13 -222 RT=6.77

Type	score	sub. m/z observed	sub. m/z calculated	sub ppm		met. m/z observed	met. m/z calculated	met. ppm
MISMATCH	-8.5	1101.8988	1101.8981	-0.68	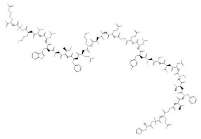	771.1490	771.1490	0.00
MISMATCH	-9.2	1247.6634	1247.6686	4.21	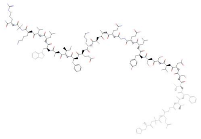	832.1195	832.1195	0.00
MISMATCH	-21.8	1291.1879	1291.1923	3.43	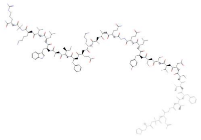	861.1274	861.1274	0.00
MISMATCH	-22.2	1341.7122	1341.7161	2.93	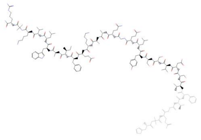	894.8111	894.8111	0.00
MISMATCH	-6.5	1415.2481	1415.2503	1.57	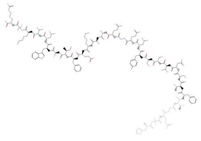	943.8321	943.8321	0.00
MET_MATCH					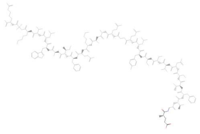	159.0763	159.0764	0.88
MET_MATCH					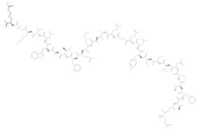	174.1343	174.1349	3.88
MET_MATCH					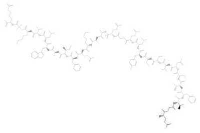	260.1293	260.1241	-20.1
MET_MATCH					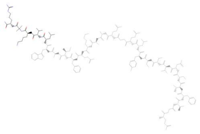	300.2209	300.2212	1.18

Metabolite: M13 -222 RT=6.77

Type	score	sub. m/z observed	sub. m/z calculated	sub ppm		met. m/z observed	met. m/z calculated	met. ppm
MET_MATCH					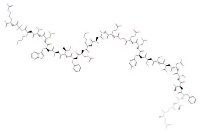	703.8759	703.8722	-5.32
MET_MATCH					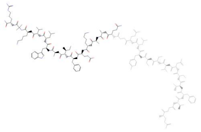	813.9789	813.9776	-1.62
MET_MATCH					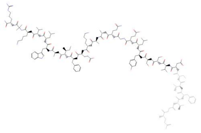	832.1195	832.1199	0.52
MET_MATCH					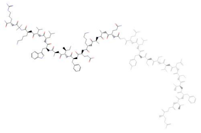	842.4910	842.4883	-3.24
MET_MATCH					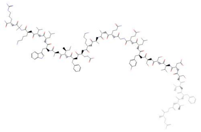	861.1274	861.1306	3.76
MET_MATCH					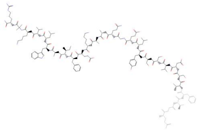	889.1430	889.1377	-6.01
MET_MATCH					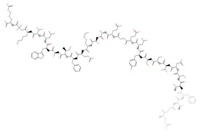	894.8111	894.8132	2.33
MET_MATCH					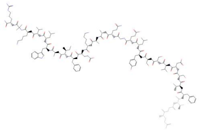	943.8321	943.8360	4.12
MET_MATCH					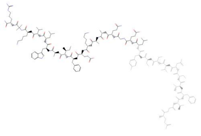	963.5535	963.5516	-1.91

Metabolite: M13 -222 RT=6.77

Type	score	sub. m/z observed	sub. m/z calculated	sub ppm		met. m/z observed	met. m/z calculated	met. ppm
MET_MATCH					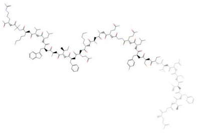	1132.1177	1132.1153	-2.08
MET_MATCH					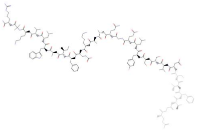	1247.6744	1247.6763	1.53

MS (+) FT

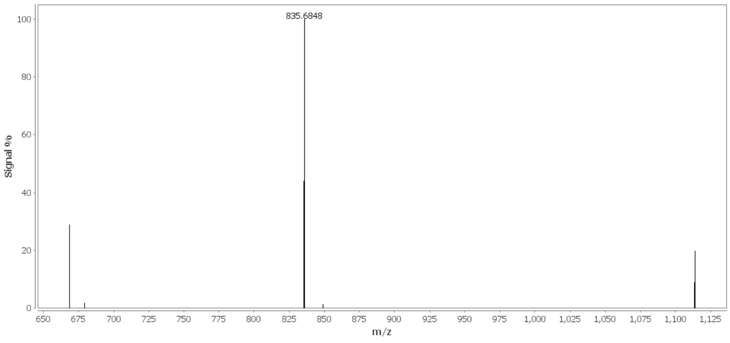

MS (+) FT

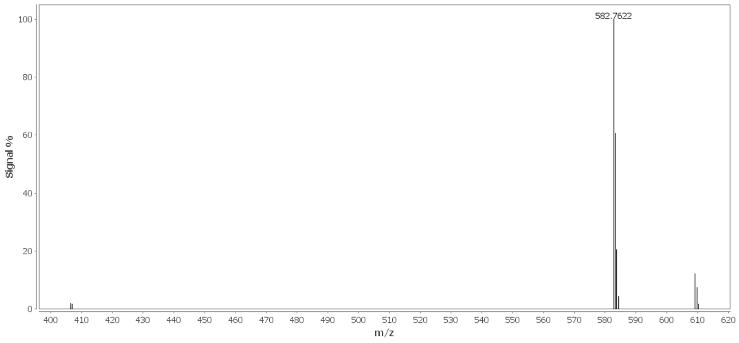

MS2 (+) FT activ = HCD:ce =

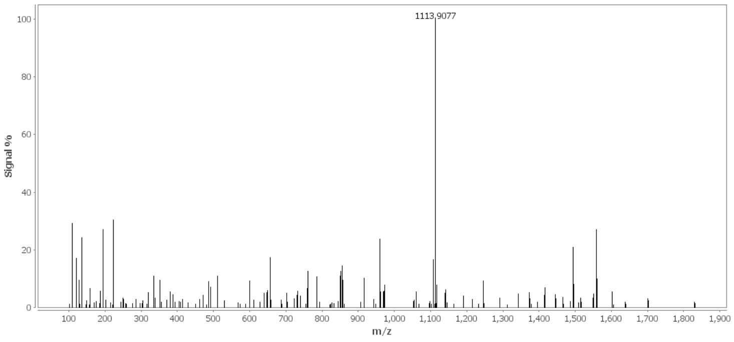

MS2 (+) FT activ = HCD:ce =

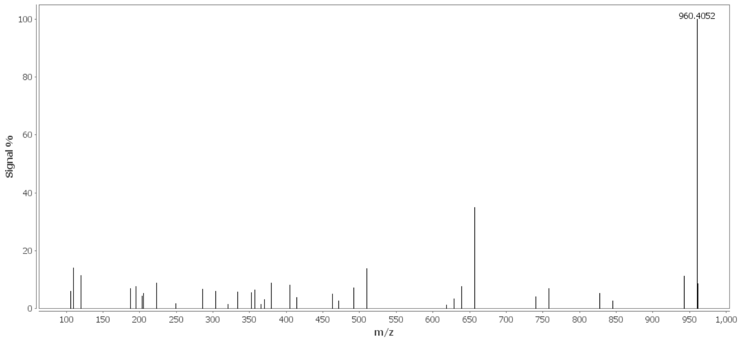

Metabolite: M1 -2174 RT=2.69

Type	score	sub. m/z observed	sub. m/z calculated	sub ppm		met. m/z observed	met. m/z calculated	met. ppm
MATCH	112.4	668.5496	668.5492	-0.61	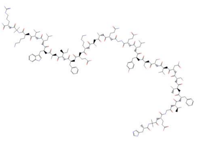 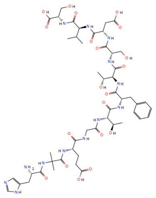	582.7622	582.7620	-0.29
					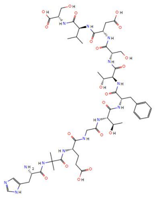	582.7622	582.7620	-0.29

Metabolite: M1 -2174 RT=2.69

Type	score	sub. m/z observed	sub. m/z calculated	sub ppm			met. m/z observed	met. m/z calculated	met. ppm
						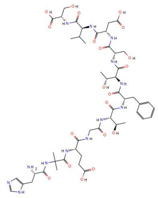	582.7622	582.7620	-0.29
MATCH	143.9	835.4371	835.4347	-2.91	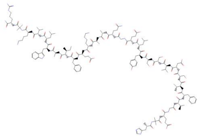	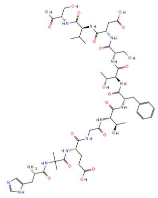	582.7622	582.7620	-0.29
						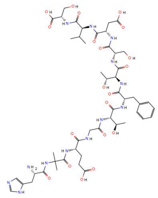	582.7622	582.7620	-0.29
						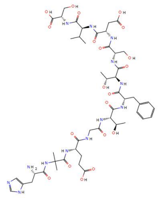	582.7622	582.7620	-0.29
MATCH	108.8	1113.5734	1113.5771	3.31	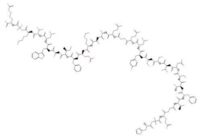	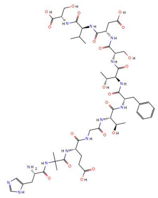	582.7622	582.7620	-0.29
						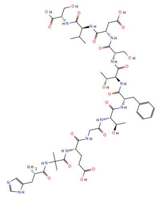	582.7622	582.7620	-0.29
						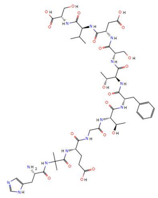	582.7622	582.7620	-0.29
MATCH	43.1	110.0714	110.0713	-1.24	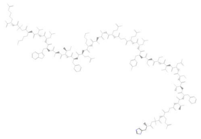	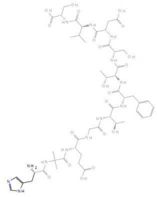	110.0716	110.0713	-3.32
MATCH	28.6	120.0808	120.0808	-0.05	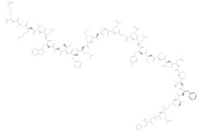	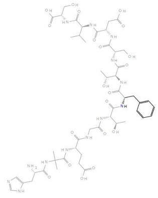	120.0811	120.0808	-2.36

Metabolite: M1 -2174 RT=2.69

Type	score	sub. m/z observed	sub. m/z calculated	sub ppm		met. m/z observed	met. m/z calculated	met. ppm
MATCH	34.6	195.1237	195.1240	1.96	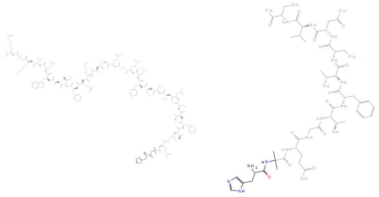	195.1240	195.1240	-0.03
MATCH	6.8	203.0657	203.0662	2.70	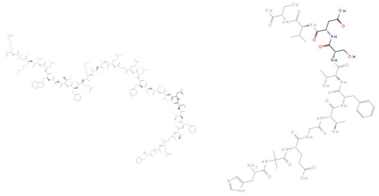	203.0660	203.0662	0.99
MATCH	6.8	203.0657	203.0662	2.70	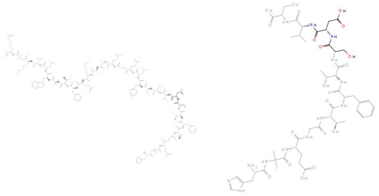	203.0660	203.0662	0.99
MATCH	39.2	223.1184	223.1190	2.48	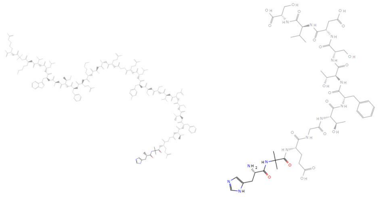	223.1188	223.1190	0.68
MATCH	2.9	249.1229	249.1234	2.01	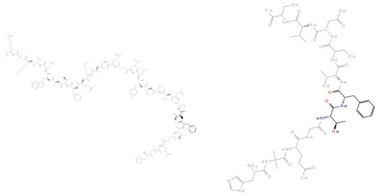	249.1230	249.1234	1.30
MATCH	2.9	249.1229	249.1234	2.01	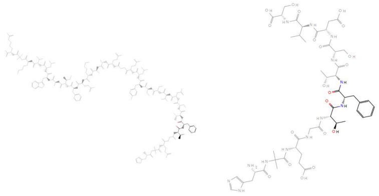	249.1230	249.1234	1.30
MATCH	2.9	249.1229	249.1234	2.01	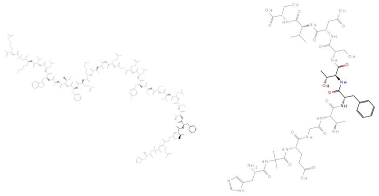	249.1230	249.1234	1.30
MATCH	2.9	249.1229	249.1234	2.01	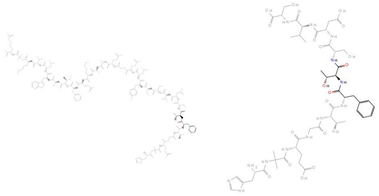	249.1230	249.1234	1.30
MATCH	9.5	286.1027	286.1034	2.41	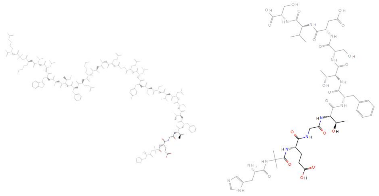	286.1028	286.1034	1.92

Metabolite: M1 -2174 RT=2.69

Type	score	sub. m/z observed	sub. m/z calculated	sub ppm			met. m/z observed	met. m/z calculated	met. ppm
MATCH	9.5	286.1027	286.1034	2.41	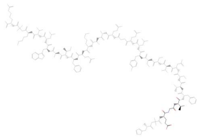	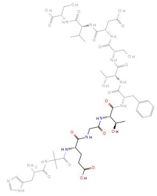	286.1028	286.1034	1.92
MATCH	9.5	286.1027	286.1034	2.41	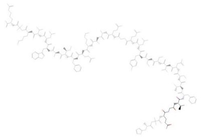	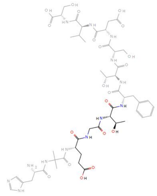	286.1028	286.1034	1.92
MATCH	8.5	304.1131	304.1139	2.82	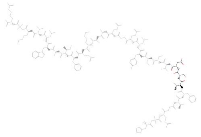	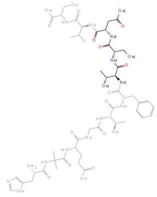	304.1138	304.1139	0.51
MATCH	8.5	304.1131	304.1139	2.82	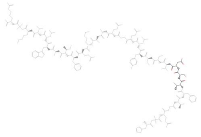	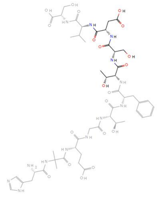	304.1138	304.1139	0.51
MATCH	15.1	352.1605	352.1615	3.00	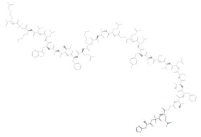	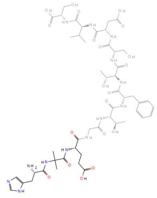	352.1614	352.1615	0.42
MATCH	8.5	356.6731	356.6743	3.31	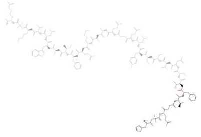	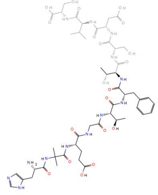	356.6735	356.6743	2.30
MATCH	8.5	356.6731	356.6743	3.31	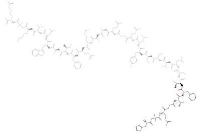	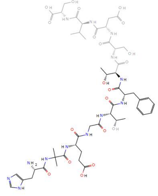	356.6735	356.6743	2.30
MATCH	8.5	356.6731	356.6743	3.31	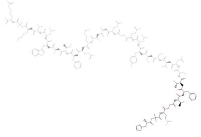	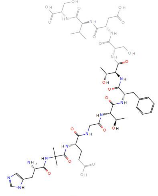	356.6735	356.6743	2.30
MATCH	8.5	356.6731	356.6792	17.18	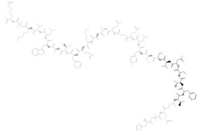	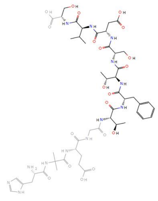	356.6735	356.6792	16.17

Metabolite: M1 -2174 RT=2.69

Type	score	sub. m/z observed	sub. m/z calculated	sub ppm		met. m/z observed	met. m/z calculated	met. ppm
MATCH	5.7	370.6705	370.6717	3.48	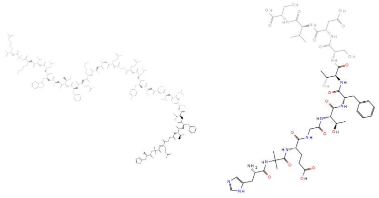	370.6709	370.6717	2.39
MATCH	5.7	370.6705	370.6717	3.48	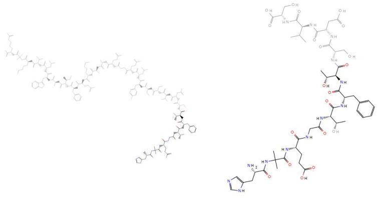	370.6709	370.6717	2.39
MATCH	14.3	379.6760	379.6770	2.78	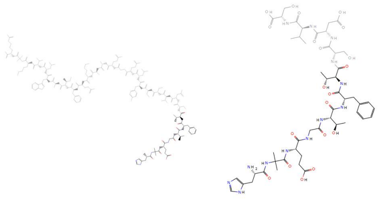	379.6765	379.6770	1.46
MATCH	6.7	414.1869	414.1878	2.02	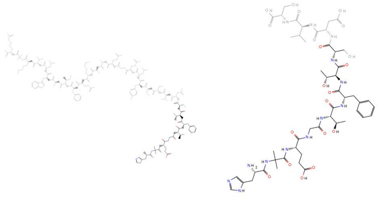	414.1872	414.1878	1.33
MATCH	6.7	414.1869	414.1878	2.02	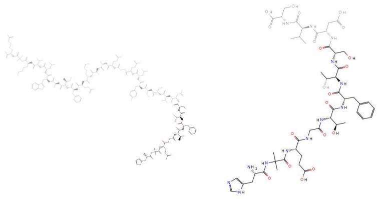	414.1872	414.1878	1.33
MATCH	6.7	414.1869	414.1878	2.02	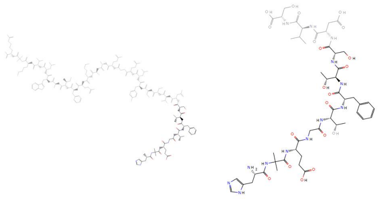	414.1872	414.1878	1.33
MATCH	6.8	471.7005	471.7012	1.61	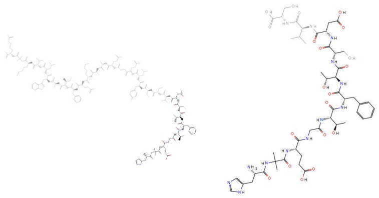	471.7009	471.7012	0.74
MATCH	6.8	471.7005	471.7012	1.61	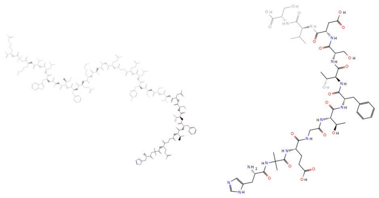	471.7009	471.7012	0.74
MATCH	6.8	471.7005	471.7012	1.61	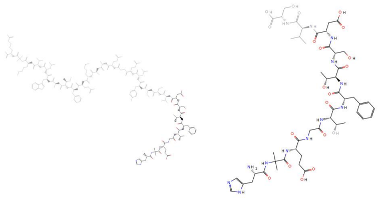	471.7009	471.7012	0.74

Metabolite: M1 -2174 RT=2.69

Type	score	sub. m/z observed	sub. m/z calculated	sub ppm			met. m/z observed	met. m/z calculated	met. ppm
						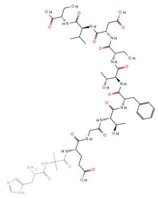	471.7009	471.7062	11.23
MATCH	24.7	510.2295	510.2307	2.28	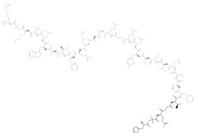	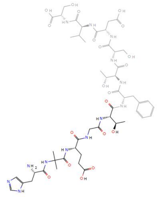	510.2303	510.2307	0.73
MATCH	5.4	629.3021	629.3042	3.35	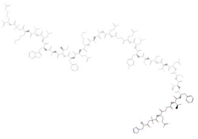	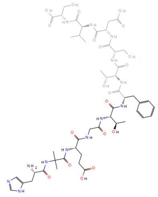	629.3038	629.3042	0.61
MATCH	12.7	639.2876	639.2885	1.49	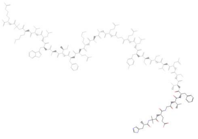	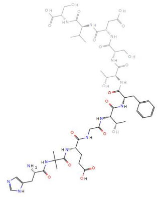	639.2874	639.2885	1.73
MATCH	52.2	657.2973	657.2991	2.75	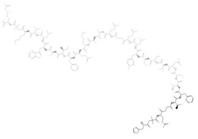	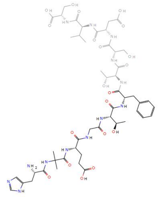	657.2986	657.2991	0.81
MATCH	8.0	740.3344	740.3362	2.49	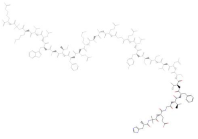	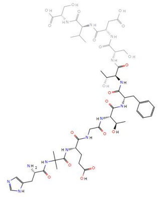	740.3351	740.3362	1.47
MATCH	8.0	740.3344	740.3362	2.49	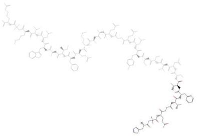	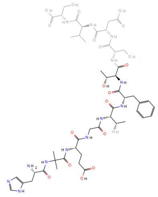	740.3351	740.3362	1.47
MATCH	13.5	758.3455	758.3468	1.71	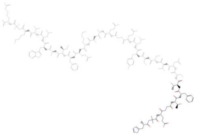	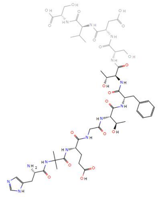	758.3457	758.3468	1.37
MATCH	7.1	827.3615	827.3682	8.18	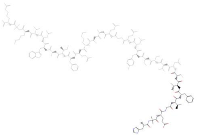	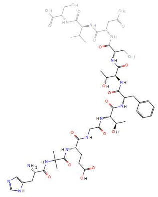	827.3674	827.3682	0.96

Metabolite: M1 -2174 RT=2.69

Type	score	sub. m/z observed	sub. m/z calculated	sub ppm		met. m/z observed	met. m/z calculated	met. ppm
MATCH	7.1	827.3615	827.3682	8.18	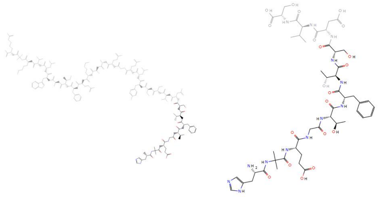	827.3674	827.3682	0.96
MATCH	7.1	827.3615	827.3682	8.18	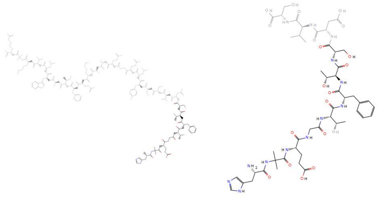	827.3674	827.3682	0.96
MATCH	4.7	845.3747	845.3788	4.90	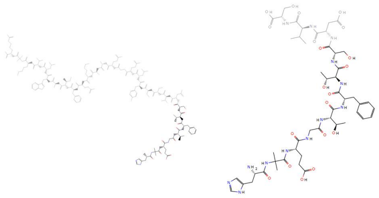	845.3780	845.3788	0.97
MATCH	14.1	942.3926	942.3952	2.80	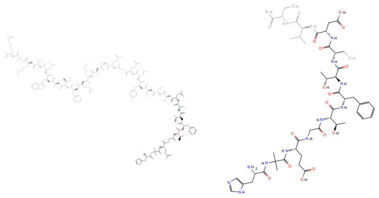	942.3943	942.3952	0.94
MATCH	14.1	942.3926	942.3952	2.80	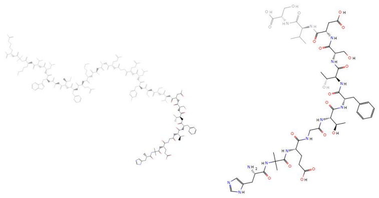	942.3943	942.3952	0.94
MATCH	14.1	942.3926	942.3952	2.80	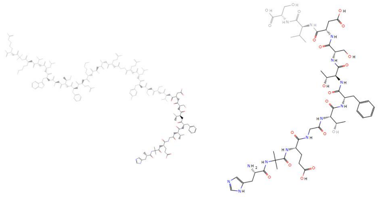	942.3943	942.3952	0.94
MATCH	123.9	960.4032	960.4058	2.64	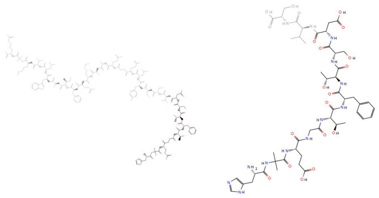	960.4052	960.4058	0.61
MATCH	8.3	1549.7979	1549.8009	1.95	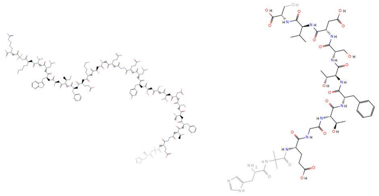	462.6951	462.7009	12.54
MATCH	8.3	1549.7979	1549.8009	1.95	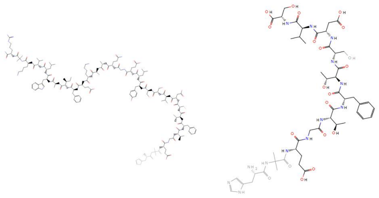	462.6951	462.7009	12.54

Metabolite: M1 -2174 RT=2.69

Type	score	sub. m/z observed	sub. m/z calculated	sub ppm			met. m/z observed	met. m/z calculated	met. ppm
MATCH	8.3	1549.7979	1549.8009	1.95	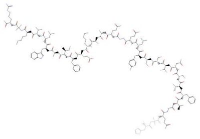	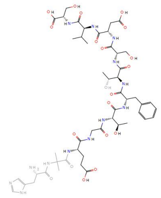	462.6951	462.7009	12.54
MATCH	8.3	1549.7979	1549.8009	1.95	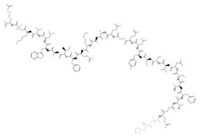	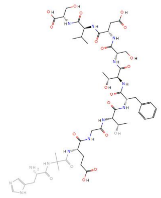	462.6951	462.7009	12.54
MATCH	18.8	1558.8038	1558.8062	1.56	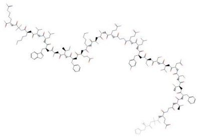	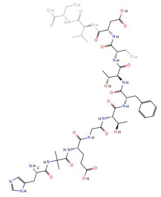	471.7009	471.7012	0.74
						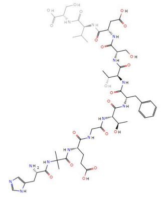	471.7009	471.7012	0.74
						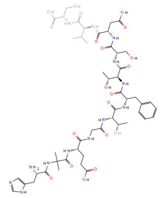	471.7009	471.7012	0.74
						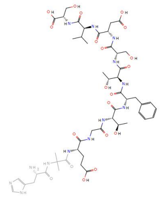	471.7009	471.7062	11.23
MISMATCH	-28.6	120.0808	120.0731	-63.5	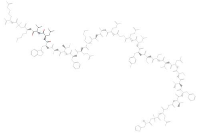		120.0811	120.0811	0.00
MISMATCH	-6.5	639.2876	639.2885	1.49	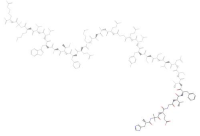		320.1467	320.1467	0.00
MISMATCH	-15.4	760.7524	760.7548	3.17	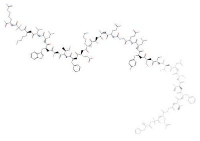		106.0502	106.0502	0.00

Metabolite: M1 -2174 RT=2.69

Type	score	sub. m/z observed	sub. m/z calculated	sub ppm		met. m/z observed	met. m/z calculated	met. ppm
MISMATCH	-6.4	793.7750	793.7776	3.28	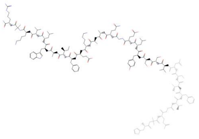	205.1180	205.1180	0.00
MISMATCH	-11.0	1140.6251	1140.6286	3.06	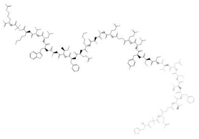	106.0502	106.0502	0.00
MISMATCH	-8.8	1190.1592	1190.1628	3.04	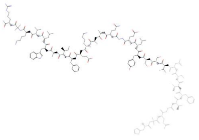	205.1180	205.1180	0.00
MET_MATCH					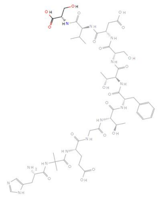	106.0502	106.0499	-3.39
MET_MATCH					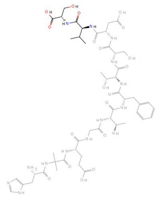	205.1180	205.1183	1.14
MET_MATCH					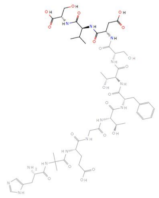	320.1467	320.1452	-4.63
MET_MATCH					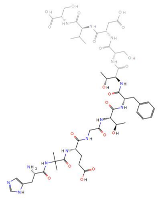	365.6792	365.6796	1.14

MS (+) FT

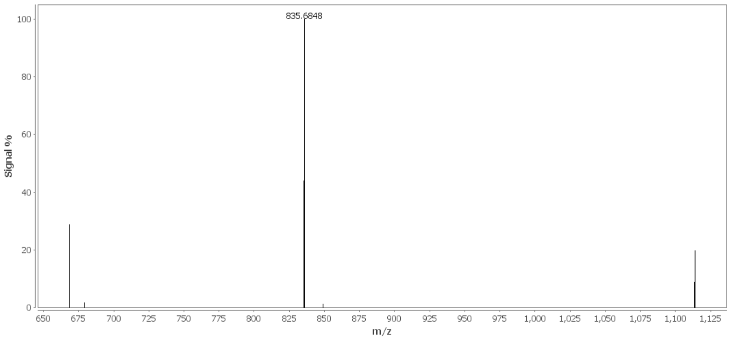

MS (+) FT

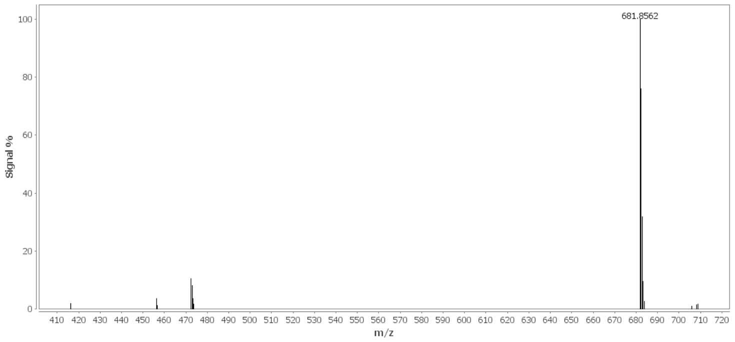

MS2 (+) FT activ = HCD:ce =

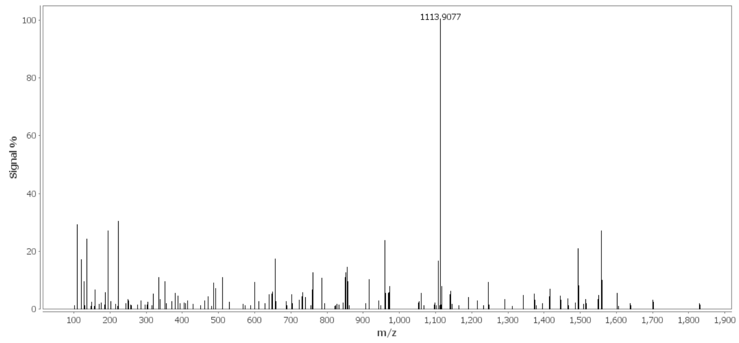

MS2 (+) FT activ = HCD:ce =

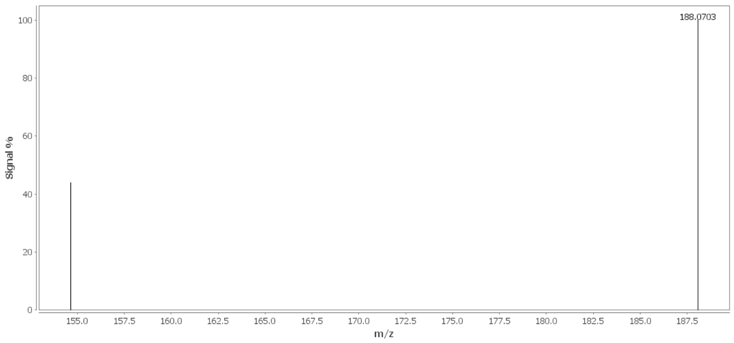

Metabolite: M9 -1976 RT=4.92

Type	score	sub. m/z observed	sub. m/z calculated	sub ppm		met. m/z observed	met. m/z calculated	met. ppm
MATCH	112.4	668.5496	668.5492	-0.61	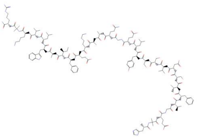 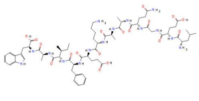	681.8562	681.8563	0.05
					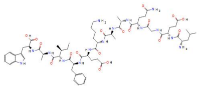	681.8562	681.8563	0.05
					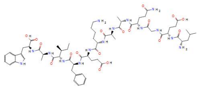	681.8562	681.8563	0.05
MATCH	143.9	835.4371	835.4347	-2.91	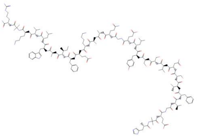 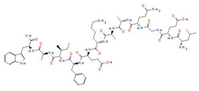	681.8562	681.8563	0.05
					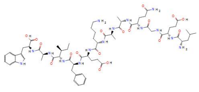	681.8562	681.8563	0.05
					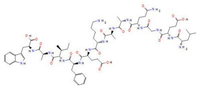	681.8562	681.8563	0.05
MATCH	108.8	1113.5734	1113.5771	3.31	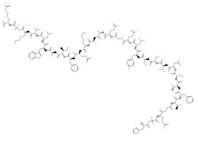 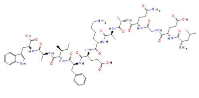	681.8562	681.8563	0.05

Metabolite: M9 -1976 RT=4.92

Type	score	sub. m/z observed	sub. m/z calculated	sub ppm		met. m/z observed	met. m/z calculated	met. ppm
					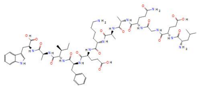	681.8562	681.8563	0.05
					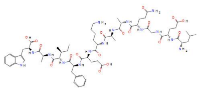	681.8562	681.8563	0.05
MET_MATCH					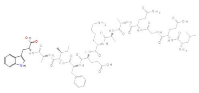	188.0703	188.0706	1.47

MS (+) FT

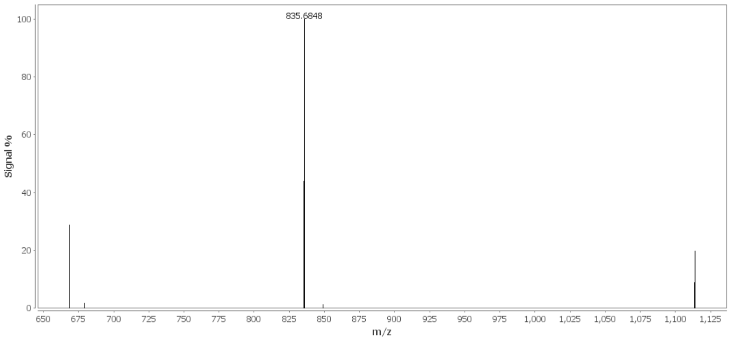

MS (+) FT

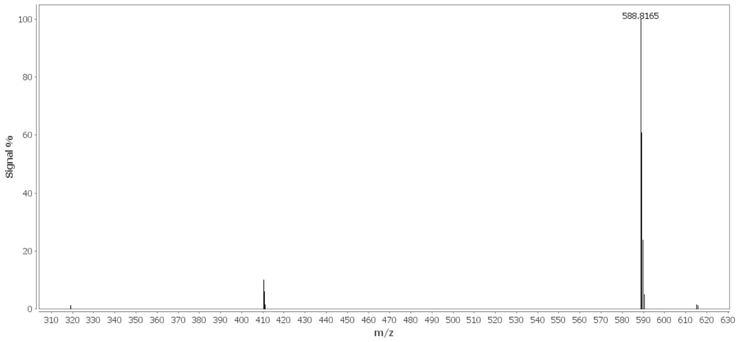

MS2 (+) FT activ = HCD:ce =

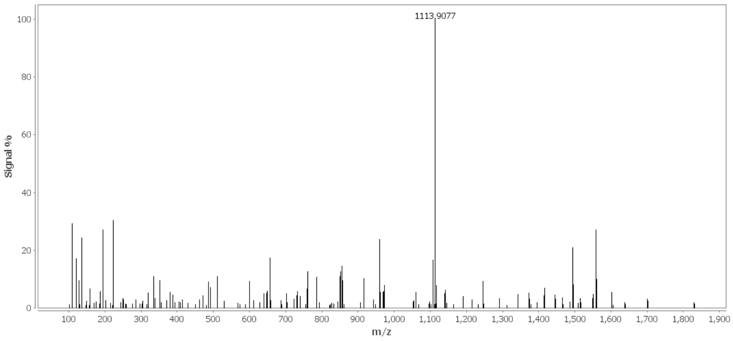

MS2 (+) FT activ = HCD:ce =

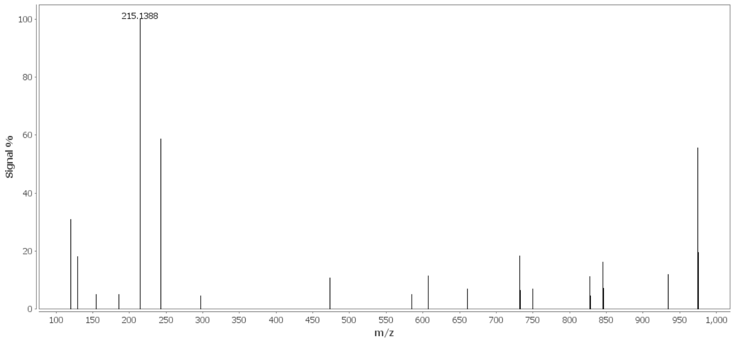

Metabolite: M2 -2162 RT=3.54

Type	score	sub. m/z observed	sub. m/z calculated	sub ppm		met. m/z observed	met. m/z calculated	met. ppm
MATCH	112.4	668.5496	668.5492	-0.61	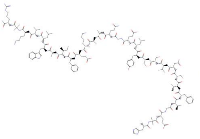 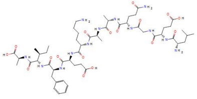	588.8165	588.8166	0.17

Metabolite: M2 -2162 RT=3.54

Type	score	sub. m/z observed	sub. m/z calculated	sub ppm			met. m/z observed	met. m/z calculated	met. ppm
						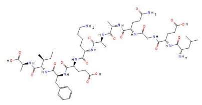	588.8165	588.8166	0.17
						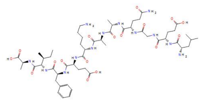	588.8165	588.8166	0.17
MATCH	143.9	835.4371	835.4347	-2.91	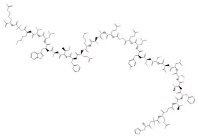	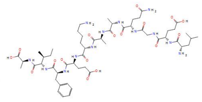	588.8165	588.8166	0.17
						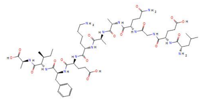	588.8165	588.8166	0.17
						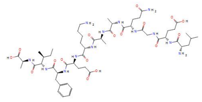	588.8165	588.8166	0.17
MATCH	108.8	1113.5734	1113.5771	3.31	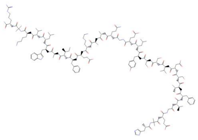	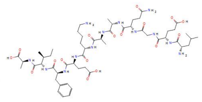	588.8165	588.8166	0.17
						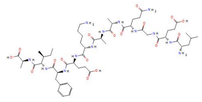	588.8165	588.8166	0.17
						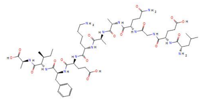	588.8165	588.8166	0.17
MATCH	48.2	120.0808	120.0808	-0.05	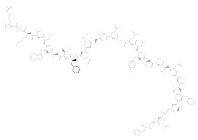	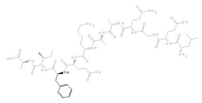	120.0808	120.0808	0.05

Metabolite: M2 -2162 RT=3.54

Type	score	sub. m/z observed	sub. m/z calculated	sub ppm		met. m/z observed	met. m/z calculated	met. ppm
MATCH	27.5	129.1021	129.1022	0.79	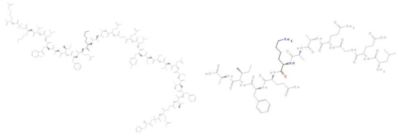	129.1023	129.1022	-0.15
MATCH	27.5	129.1021	129.1022	0.79	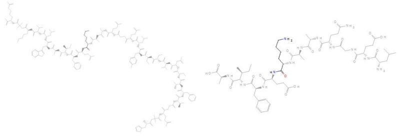	129.1023	129.1022	-0.15
MATCH	6.4	186.0864	186.0873	4.77	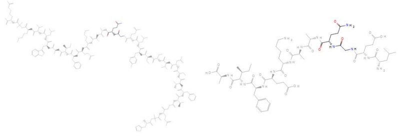	186.0871	186.0873	1.35
MATCH	6.4	186.0864	186.0873	4.77	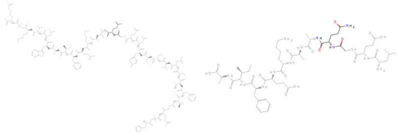	186.0871	186.0873	1.35
MATCH	101.3	215.1383	215.1390	3.14	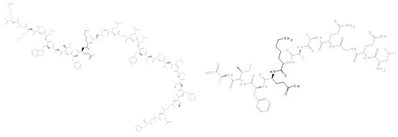	215.1388	215.1390	0.97
MATCH	60.5	243.1333	243.1339	2.72	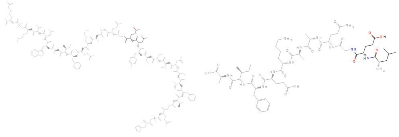	243.1338	243.1339	0.68
					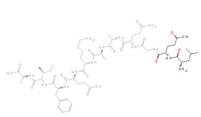	243.1338	243.1339	0.68
MATCH	6.0	297.1187	297.1193	2.22	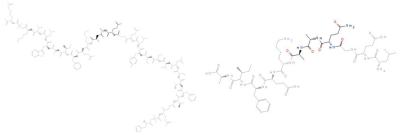	297.1193	297.1193	0.18
MATCH	16.8	1373.8392	1373.8416	1.75	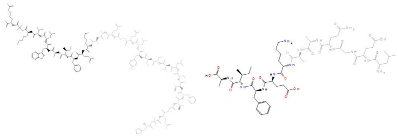	607.3444	607.3450	0.91

Metabolite: M2 -2162 RT=3.54

Type	score	sub. m/z observed	sub. m/z calculated	sub ppm		met. m/z observed	met. m/z calculated	met. ppm
MATCH	10.2	1515.9113	1515.9158	2.97	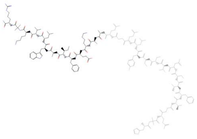 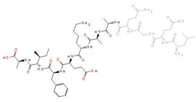	749.4194	749.4192	-0.24
MATCH	60.5	1638.7199	1638.7282	5.06	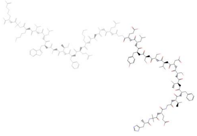 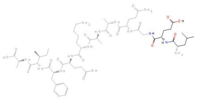	243.1338	243.1339	0.68
					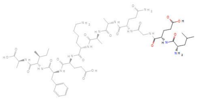	243.1338	243.1339	0.68
MATCH	14.8	1700.9942	1700.9959	0.96	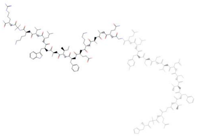 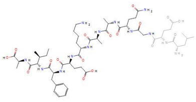	934.4936	934.4993	6.00
MISMATCH	-48.2	120.0808	120.0731	-63.5	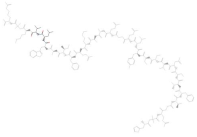	120.0808	120.0808	0.00
MISMATCH	-12.3	1112.0134	1112.0137	0.21	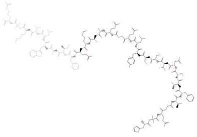	827.4239	827.4239	0.00
MET_MATCH					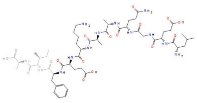	473.7531	473.7533	0.40
MET_MATCH					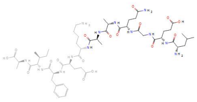	585.2968	585.2991	4.01
MET_MATCH					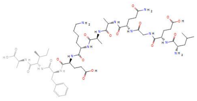	827.4239	827.4258	2.19

Metabolite: M2 -2162 RT=3.54

Type	score	sub. m/z observed	sub. m/z calculated	sub ppm		met. m/z observed	met. m/z calculated	met. ppm
MET_MATCH					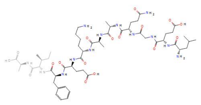	974.4938	974.4942	0.38

MS (+) FT

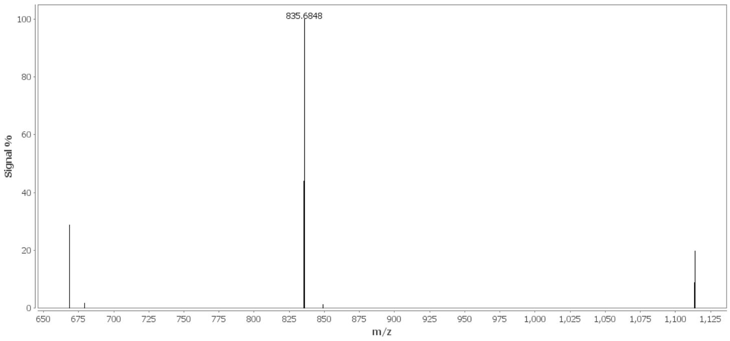

MS (+) FT

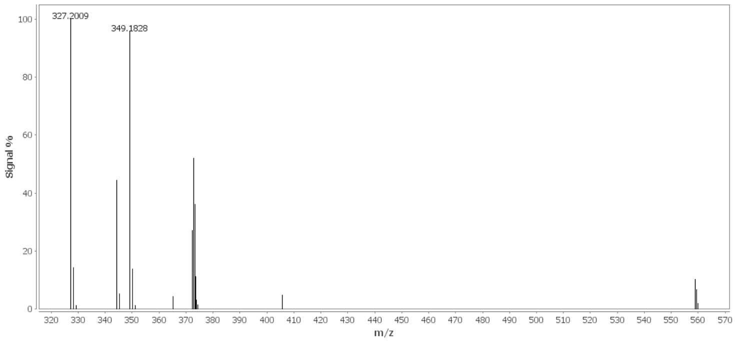

MS2 (+) FT activ = HCD:ce =

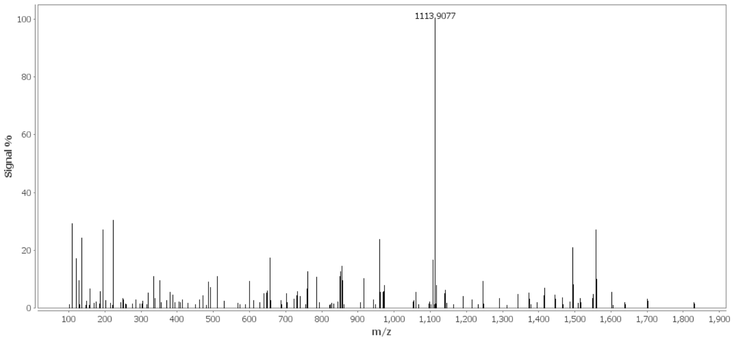

MS2 (+) FT activ = HCD:ce =

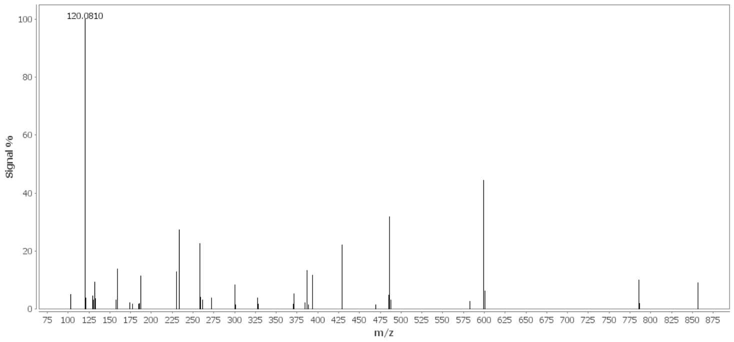

Metabolite: M4 -2222 RT=3.88

Type	score	sub. m/z observed	sub. m/z calculated	sub ppm			met. m/z observed	met. m/z calculated	met. ppm
MATCH	64.4	668.5496	668.5492	-0.61	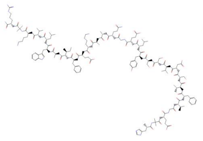	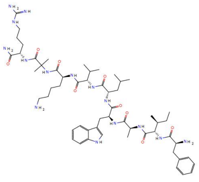	372.9061	372.9062	0.14
MATCH	64.4	668.5496	668.5492	-0.61	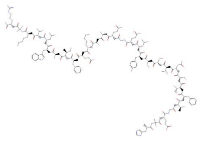	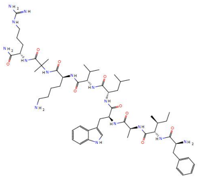	372.9061	372.9062	0.14
						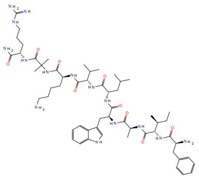	372.9061	372.9062	0.14

Metabolite: M4 -2222 RT=3.88

Type	score	sub. m/z observed	sub. m/z calculated	sub ppm			met. m/z observed	met. m/z calculated	met. ppm
MATCH	22.5	668.5496	668.5492	-0.61	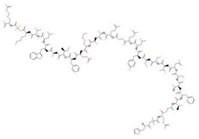	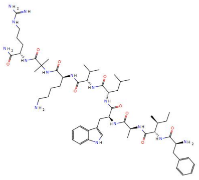	558.8552	558.8557	0.77
MATCH	22.5	668.5496	668.5492	-0.61	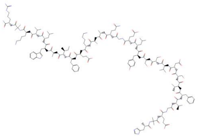	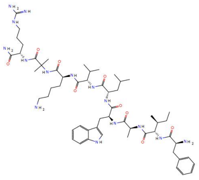	558.8552	558.8557	0.77
						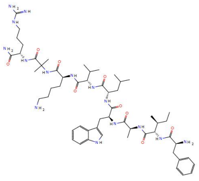	558.8552	558.8557	0.77
MATCH	95.9	835.4371	835.4347	-2.91	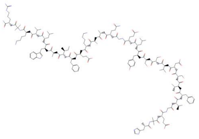	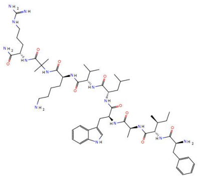	372.9061	372.9062	0.14
MATCH	95.9	835.4371	835.4347	-2.91	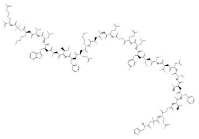	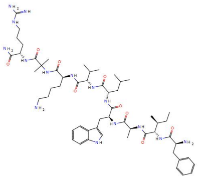	372.9061	372.9062	0.14
						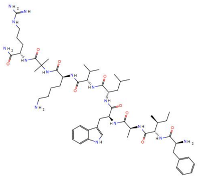	372.9061	372.9062	0.14
MATCH	54.0	835.4371	835.4347	-2.91	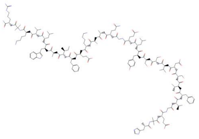	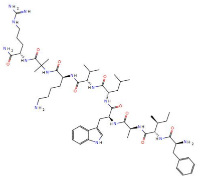	558.8552	558.8557	0.77
MATCH	54.0	835.4371	835.4347	-2.91	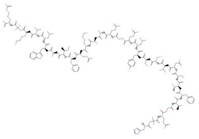	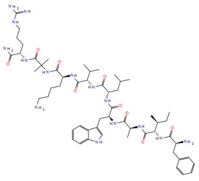	558.8552	558.8557	0.77
						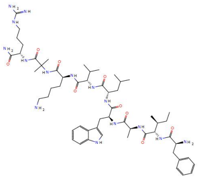	558.8552	558.8557	0.77

Metabolite: M4 -2222 RT=3.88

Type	score	sub. m/z observed	sub. m/z calculated	sub ppm		met. m/z observed	met. m/z calculated	met. ppm
MATCH	60.8	1113.5734	1113.5771	3.31	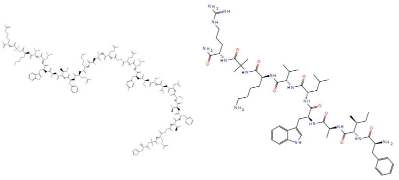	372.9061	372.9062	0.14
MATCH	60.8	1113.5734	1113.5771	3.31	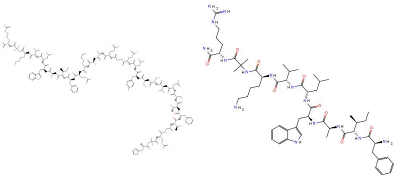	372.9061	372.9062	0.14
					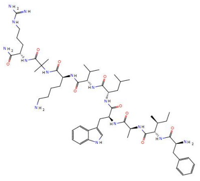	372.9061	372.9062	0.14
MATCH	18.9	1113.5734	1113.5771	3.31	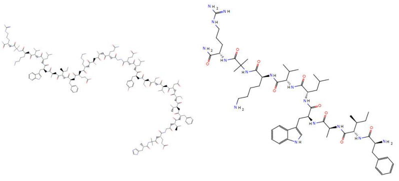	558.8552	558.8557	0.77
MATCH	18.9	1113.5734	1113.5771	3.31	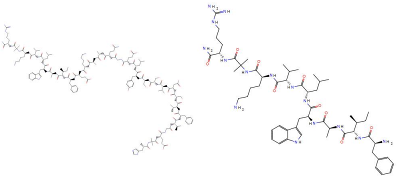	558.8552	558.8557	0.77
					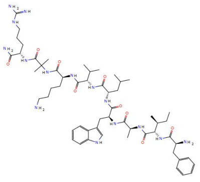	558.8552	558.8557	0.77
MATCH	117.2	120.0808	120.0731	-63.5	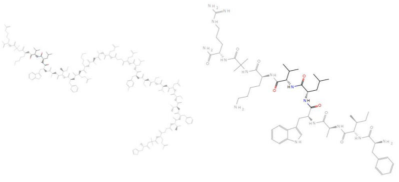	120.0810	120.0731	-65.5
MATCH	117.2	120.0808	120.0865	47.43	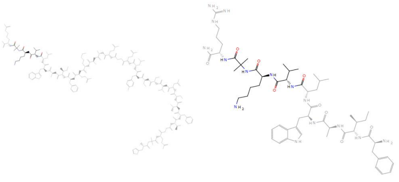	120.0810	120.0865	45.47
MATCH	14.1	129.1021	129.1022	0.79	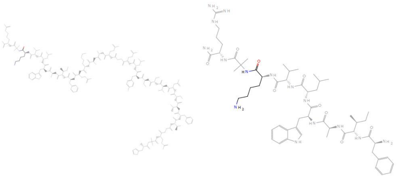	129.1022	129.1022	0.01

Metabolite: M4 -2222 RT=3.88

Type	score	sub. m/z observed	sub. m/z calculated	sub ppm		met. m/z observed	met. m/z calculated	met. ppm
MATCH	14.1	129.1021	129.1022	0.79	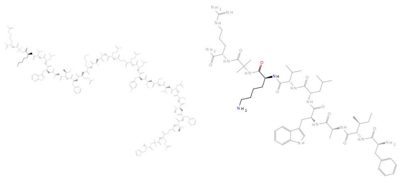	129.1022	129.1022	0.01
MATCH	5.2	259.1870	259.1877	2.62	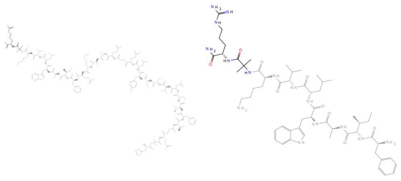	259.1877	259.1877	-0.17
MATCH	17.9	387.2815	387.2827	3.02	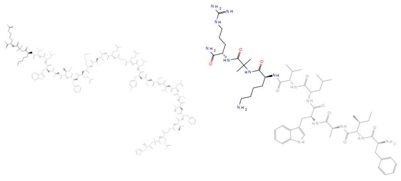	387.2822	387.2827	1.13
MATCH	13.7	393.2598	393.2609	2.83	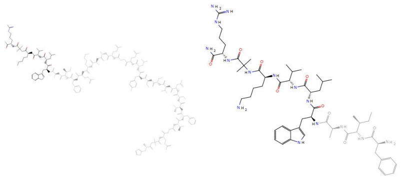	393.2604	393.2609	1.08
MATCH	23.8	428.7782	428.7794	2.80	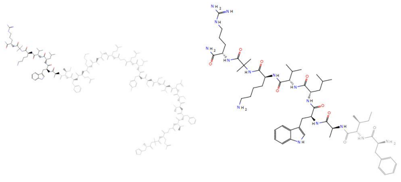	428.7791	428.7794	0.74
MATCH	40.9	486.3500	486.3511	2.28	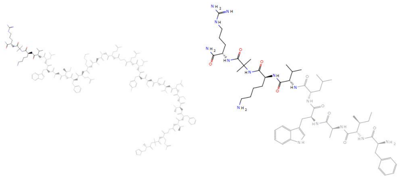	486.3506	486.3511	0.97
MATCH	53.8	599.4338	599.4351	2.24	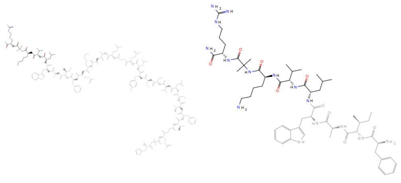	599.4343	599.4351	1.33
MATCH	20.7	785.5122	785.5145	2.93	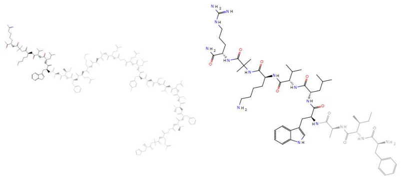	785.5131	785.5145	1.69
MATCH	23.6	856.5492	856.5516	2.77	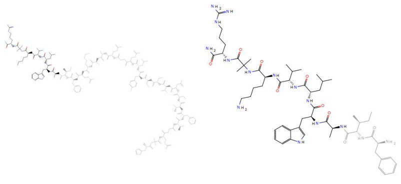	856.5499	856.5516	2.00

Metabolite: M4 -2222 RT=3.88

Type	score	sub. m/z observed	sub. m/z calculated	sub ppm		met. m/z observed	met. m/z calculated	met. ppm
MISMATCH	-117.2	120.0808	120.0808	-0.05	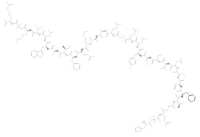	120.0810	120.0810	0.00
MISMATCH	-20.6	159.0913	159.0946	20.67	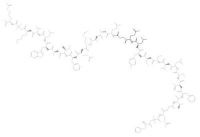	159.0916	159.0916	0.00
MISMATCH	-17.6	599.4338	599.4351	2.24	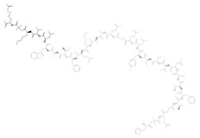	300.2209	300.2209	0.00
MISMATCH	-10.3	969.6332	969.6356	2.54	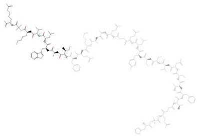	485.3216	485.3216	0.00
MET_MATCH					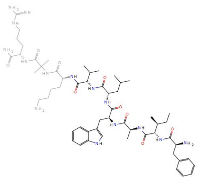	374.2362	374.2312	-13.3
MET_MATCH					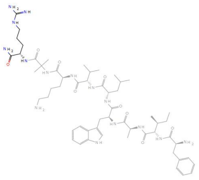	174.1350	174.1349	-0.24
MET_MATCH					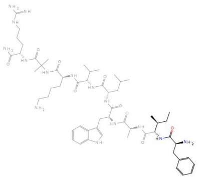	233.1646	233.1648	1.15
MET_MATCH					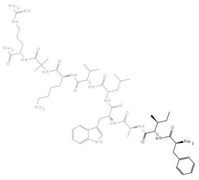	261.1595	261.1598	0.83
MET_MATCH					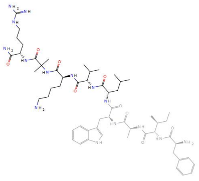	300.2209	300.2212	1.08

Metabolite: M4 -2222 RT=3.88

Type	score	sub. m/z observed	sub. m/z calculated	sub ppm		met. m/z observed	met. m/z calculated	met. ppm
MET_MATCH					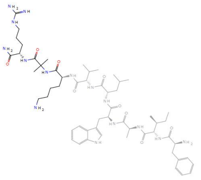	370.2563	370.2561	-0.63
MET_MATCH					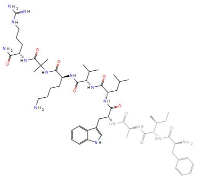	384.7471	384.7476	1.27
MET_MATCH					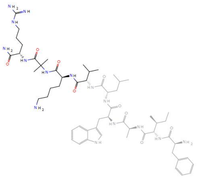	469.3250	469.3245	-0.90
MET_MATCH					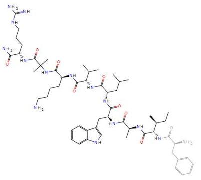	485.3216	485.3215	-0.29
MET_MATCH					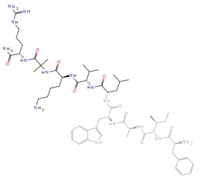	582.4096	582.4086	-1.74

MS (+) FT

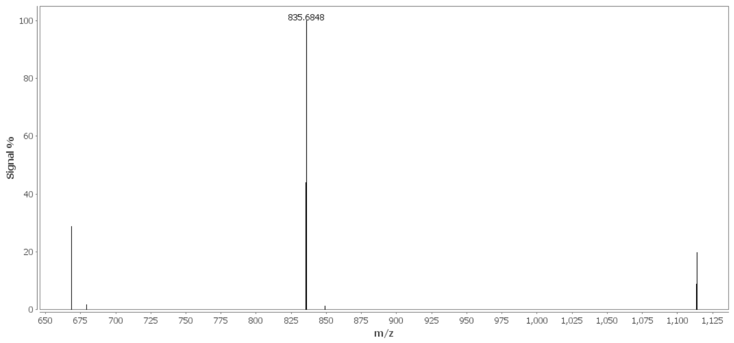

MS (+) FT

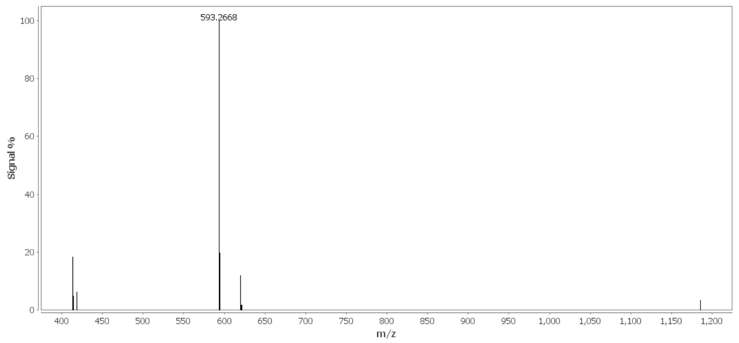

MS2 (+) FT activ = HCD:ce =

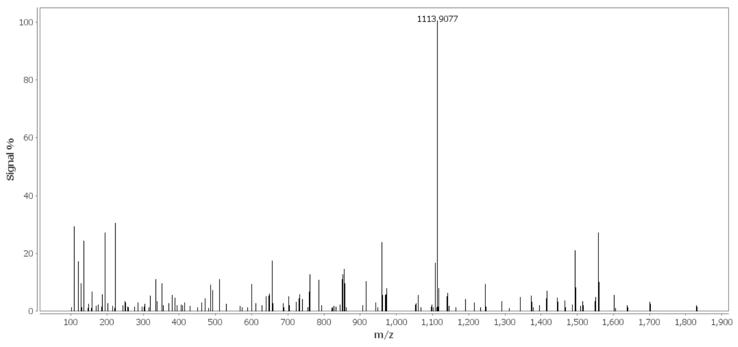

MS2 (+) FT activ = HCD:ce =

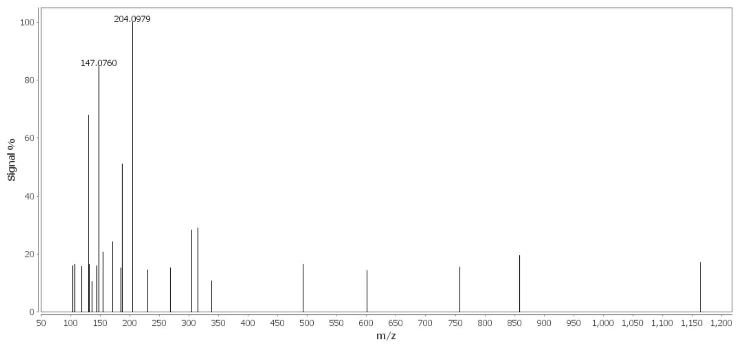

Metabolite: M7 -2153 RT=4.47

Type	score	sub. m/z observed	sub. m/z calculated	sub ppm		met. m/z observed	met. m/z calculated	met. ppm
MATCH	112.4	668.5496	668.5492	-0.61	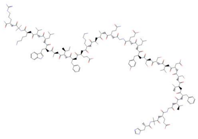 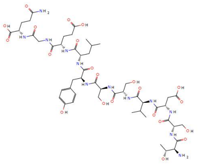	593.2668	593.2671	0.50
MATCH	112.4	668.5496	668.5492	-0.61	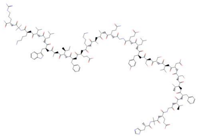 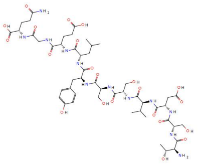	593.2668	593.2671	0.50
					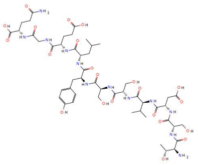	593.2668	593.2671	0.50
MATCH	15.7	668.5496	668.5492	-0.61	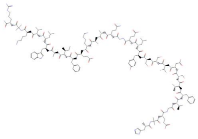 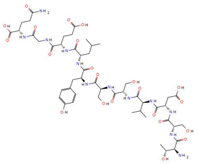	1185.5263	1185.5270	0.61
MATCH	143.9	835.4371	835.4347	-2.91	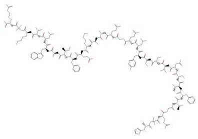 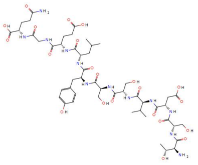	593.2668	593.2671	0.50
					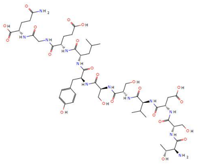	593.2668	593.2671	0.50
					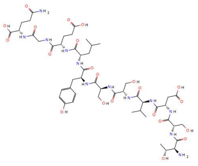	593.2668	593.2671	0.50
MATCH	108.8	1113.5734	1113.5771	3.31	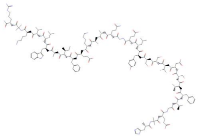 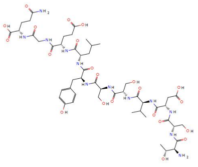	593.2668	593.2671	0.50
					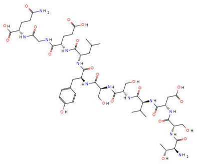	593.2668	593.2671	0.50

Metabolite: M7 -2153 RT=4.47

Type	score	sub. m/z observed	sub. m/z calculated	sub ppm			met. m/z observed	met. m/z calculated	met. ppm
						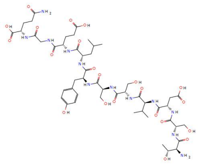	593.2668	593.2671	0.50
MATCH	85.9	147.0760	147.0764	2.67	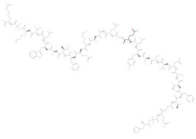	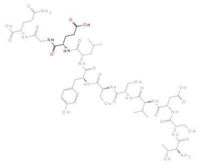	147.0760	147.0764	2.52
MATCH	30.9	304.1131	304.1139	2.82	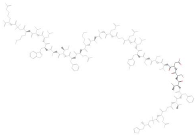	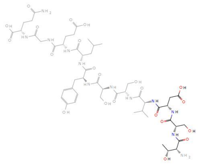	304.1137	304.1139	0.85
						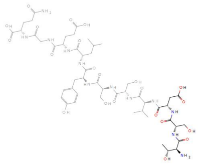	304.1137	304.1139	0.85
MATCH	14.0	338.1337	338.1347	2.83	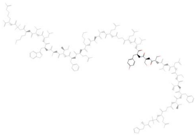	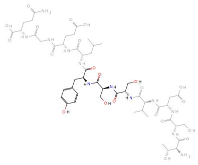	338.1336	338.1347	3.12
MATCH	14.0	338.1337	338.1347	2.83	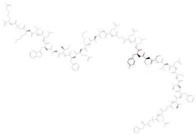	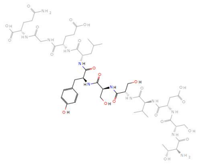	338.1336	338.1347	3.12
MATCH	30.2	906.5161	906.5176	1.60	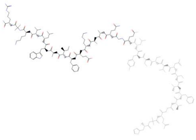	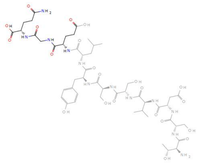	315.1290	315.1299	2.80
MATCH	30.2	906.5161	906.5176	1.60	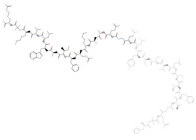	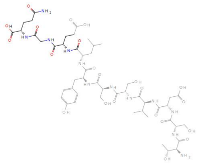	315.1290	315.1299	2.80
MATCH	52.2	960.4032	960.4058	2.64	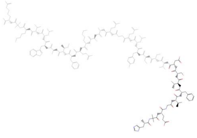	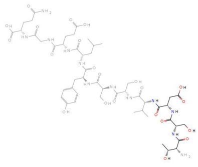	304.1137	304.1139	0.85

Metabolite: M7 -2153 RT=4.47

Type	score	sub. m/z observed	sub. m/z calculated	sub ppm			met. m/z observed	met. m/z calculated	met. ppm
						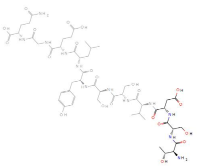	304.1137	304.1139	0.85
MATCH	103.0	1700.9942	1700.9959	0.96	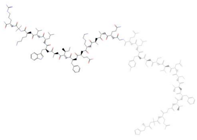	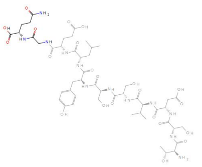	204.0979	204.0979	-0.26
MET_MATCH						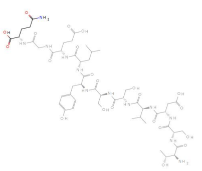	130.0498	130.0499	0.38
MET_MATCH						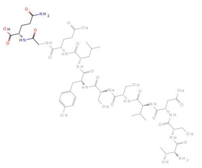	187.0710	187.0713	1.85
MET_MATCH						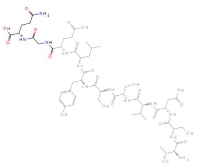	230.0840	230.0771	-29.5

MS (+) FT

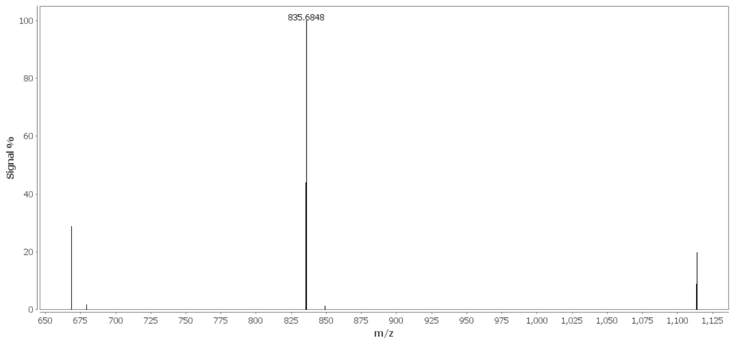

MS (+) FT

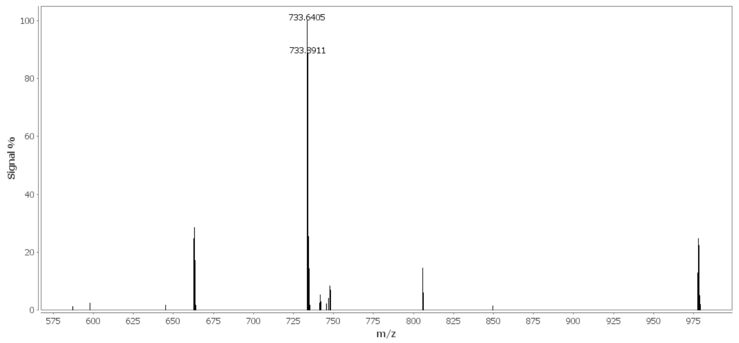

MS2 (+) FT activ = HCD:ce =

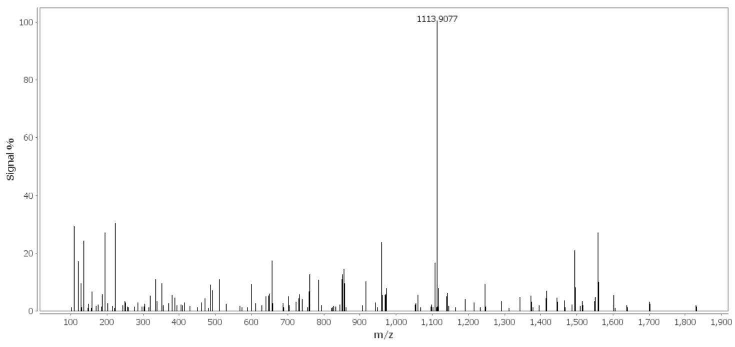

MS2 (+) FT activ = HCD:ce =

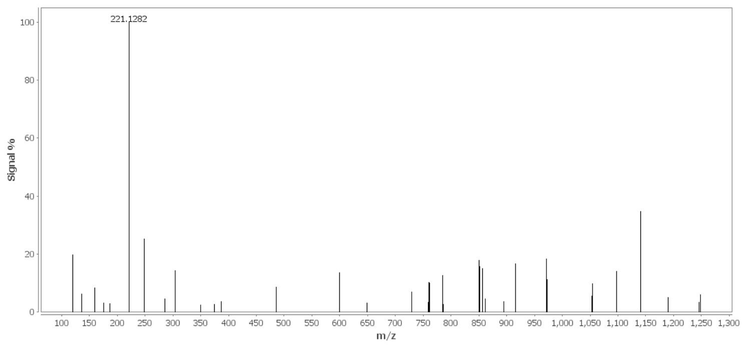

Metabolite: M12 -408 RT=6.72

Type	score	sub. m/z observed	sub. m/z calculated	sub ppm			met. m/z observed	met. m/z calculated	met. ppm
MATCH	73.8	668.5496	668.5492	-0.61	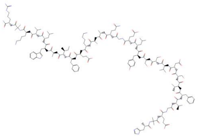	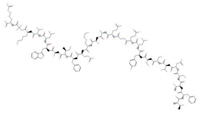	733.3900	733.3907	0.98
MATCH	73.8	668.5496	668.5492	-0.61	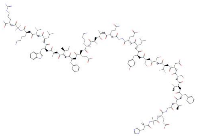	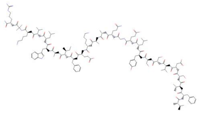	733.3900	733.3907	0.98
						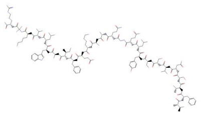	733.3900	733.3907	0.98
MATCH	25.2	668.5496	668.5492	-0.61	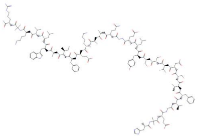	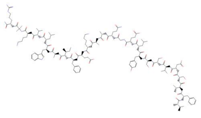	977.5172	977.5185	1.41
MATCH	25.2	668.5496	668.5492	-0.61	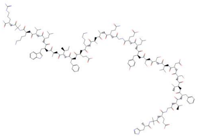	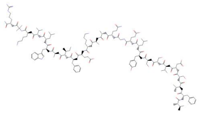	977.5172	977.5185	1.41
						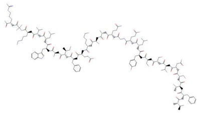	977.5172	977.5185	1.41
MATCH	105.3	835.4371	835.4347	-2.91	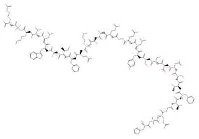	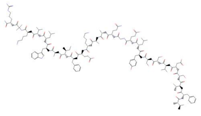	733.3900	733.3907	0.98
MATCH	105.3	835.4371	835.4347	-2.91	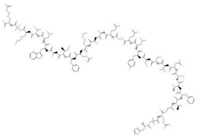	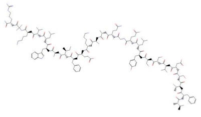	733.3900	733.3907	0.98
						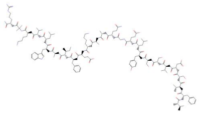	733.3900	733.3907	0.98

Metabolite: M12 -408 RT=6.72

Type	score	sub. m/z observed	sub. m/z calculated	sub ppm			met. m/z observed	met. m/z calculated	met. ppm
MATCH	56.7	835.4371	835.4347	-2.91	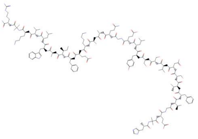	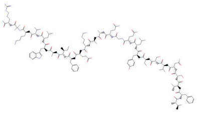	977.5172	977.5185	1.41
MATCH	56.7	835.4371	835.4347	-2.91	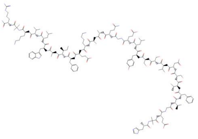	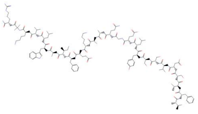	977.5172	977.5185	1.41
						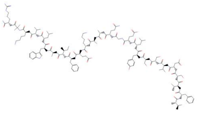	977.5172	977.5185	1.41
MATCH	70.2	1113.5734	1113.5771	3.31	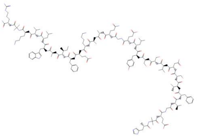	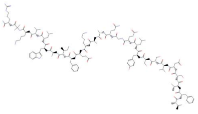	733.3900	733.3907	0.98
MATCH	70.2	1113.5734	1113.5771	3.31	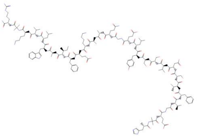	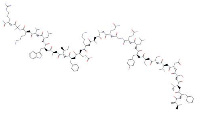	733.3900	733.3907	0.98
						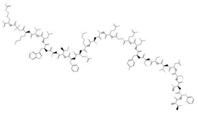	733.3900	733.3907	0.98
MATCH	21.6	1113.5734	1113.5771	3.31	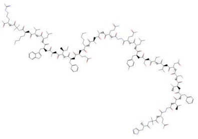	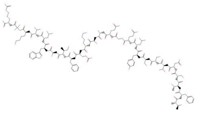	977.5172	977.5185	1.41
MATCH	21.6	1113.5734	1113.5771	3.31	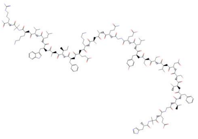	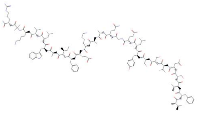	977.5172	977.5185	1.41
						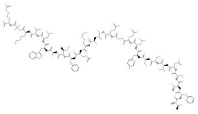	977.5172	977.5185	1.41

Metabolite: M12 -408 RT=6.72

Type	score	sub. m/z observed	sub. m/z calculated	sub ppm		met. m/z observed	met. m/z calculated	met. ppm
MATCH	36.8	120.0808	120.0731	-63.5	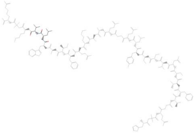 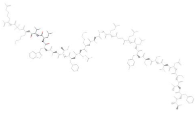	120.0808	120.0731	-63.5
MATCH	36.8	120.0808	120.0808	-0.05	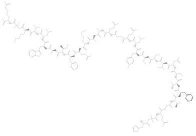 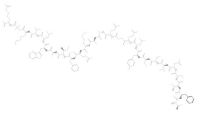	120.0808	120.0808	-0.04
MATCH	36.8	120.0808	120.0808	-0.05	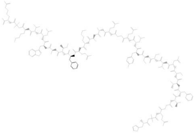 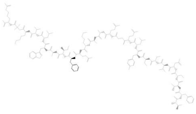	120.0808	120.0808	-0.04
MATCH	36.8	120.0808	120.0865	47.43	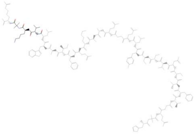 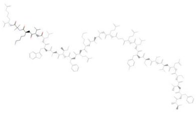	120.0808	120.0865	47.44
MATCH	30.5	136.0755	136.0693	-45.4	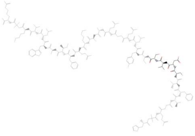 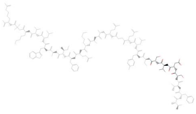	136.0755	136.0693	-45.8
MATCH	30.5	136.0755	136.0693	-45.4	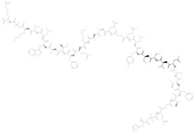 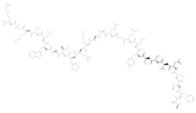	136.0755	136.0693	-45.8
MATCH	30.5	136.0755	136.0706	-35.6	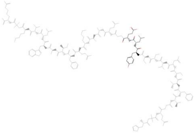 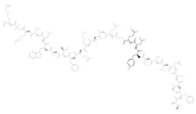	136.0755	136.0706	-35.9
MATCH	30.5	136.0755	136.0706	-35.6	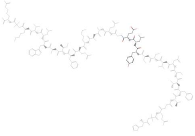 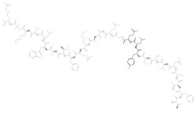	136.0755	136.0706	-35.9
MATCH	30.5	136.0755	136.0737	-13.0	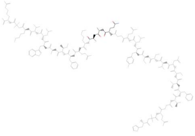 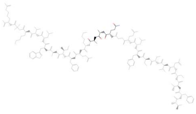	136.0755	136.0737	-13.3

Metabolite: M12 -408 RT=6.72

Type	score	sub. m/z observed	sub. m/z calculated	sub ppm			met. m/z observed	met. m/z calculated	met. ppm
MATCH	30.5	136.0755	136.0737	-13.0	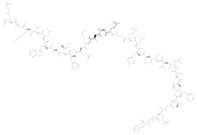	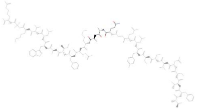	136.0755	136.0737	-13.3
MATCH	14.9	159.0913	159.0946	20.67	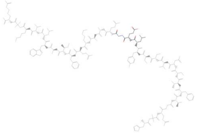	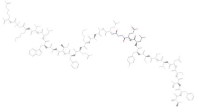	159.0914	159.0946	19.94
MATCH	8.8	187.1073	187.1077	2.11	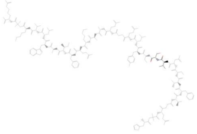	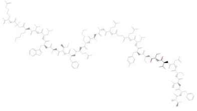	187.1073	187.1077	2.49
MATCH	8.8	187.1073	187.1077	2.11	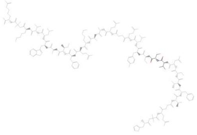	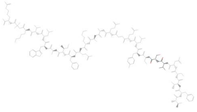	187.1073	187.1077	2.49
MATCH	101.1	221.1278	221.1285	2.74	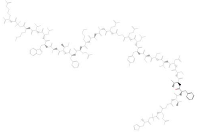	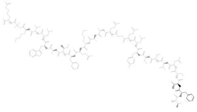	221.1282	221.1285	1.05
						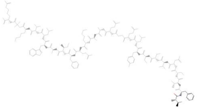	221.1282	221.1285	1.05
MATCH	26.5	249.1229	249.1234	2.01	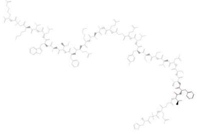	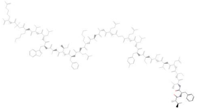	249.1230	249.1234	1.61
MATCH	26.5	249.1229	249.1234	2.01	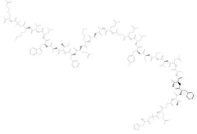	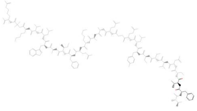	249.1230	249.1234	1.61
MATCH	26.5	249.1229	249.1234	2.01	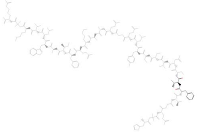	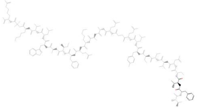	249.1230	249.1234	1.61

Metabolite: M12 -408 RT=6.72

Type	score	sub. m/z observed	sub. m/z calculated	sub ppm			met. m/z observed	met. m/z calculated	met. ppm
						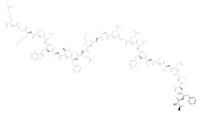	249.1230	249.1234	1.61
MATCH	16.7	304.1131	304.1139	2.82	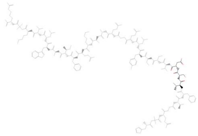	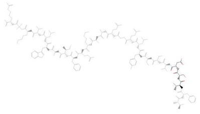	304.1135	304.1139	1.47
MATCH	16.7	304.1131	304.1139	2.82	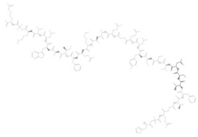	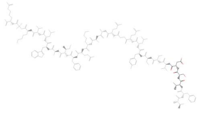	304.1135	304.1139	1.47
MATCH	8.1	387.2815	387.2827	3.02	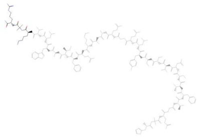	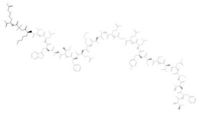	387.2821	387.2827	1.36
MATCH	17.7	486.3500	486.3511	2.28	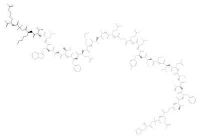	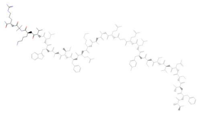	486.3495	486.3511	3.32
MATCH	23.0	599.4338	599.4351	2.24	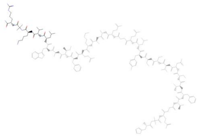	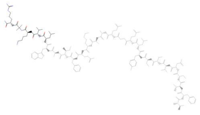	599.4337	599.4351	2.45
MATCH	102.0	629.3021	629.3042	3.35	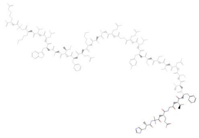	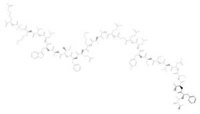	221.1282	221.1285	1.05
						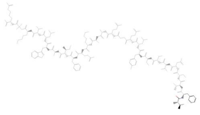	221.1282	221.1285	1.05
MATCH	42.5	657.2973	657.2991	2.75	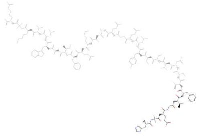	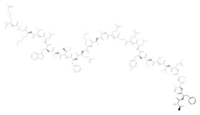	249.1230	249.1234	1.61

Metabolite: M12 -408 RT=6.72

Type	score	sub. m/z observed	sub. m/z calculated	sub ppm			met. m/z observed	met. m/z calculated	met. ppm
						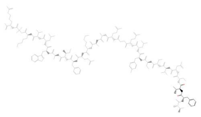	249.1230	249.1234	1.61
						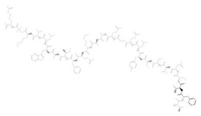	249.1230	249.1234	1.61
						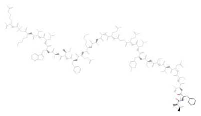	249.1230	249.1234	1.61
MATCH	9.1	758.3455	758.3468	1.71	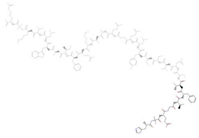	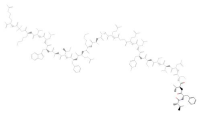	350.1714	350.1710	-0.97
MATCH	5.9	758.4573	758.4616	5.67	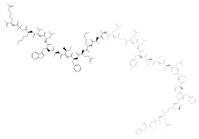	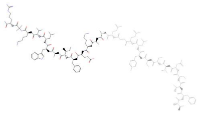	758.4591	758.4616	3.21
MATCH	19.7	760.7524	760.7548	3.17	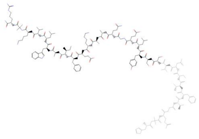	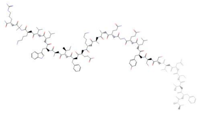	760.7534	760.7548	1.91
MATCH	23.3	785.5122	785.5145	2.93	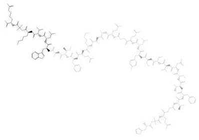	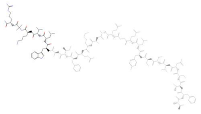	785.5115	785.5145	3.70
MATCH	28.7	850.9987	851.0016	3.36	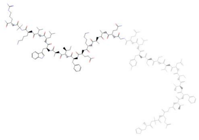	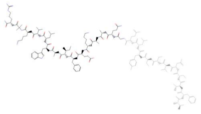	851.0003	851.0016	1.51
MATCH	29.4	856.5492	856.5516	2.77	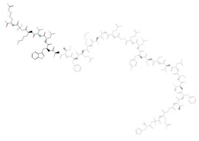	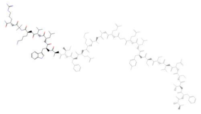	856.5503	856.5516	1.51

Metabolite: M12 -408 RT=6.72

Type	score	sub. m/z observed	sub. m/z calculated	sub ppm			met. m/z observed	met. m/z calculated	met. ppm
MATCH	5.8	861.4701	861.4618	-9.65	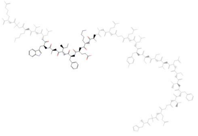	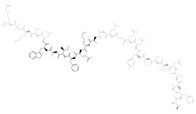	861.4674	861.4618	-6.58
MATCH	5.8	861.4701	861.4667	-3.90	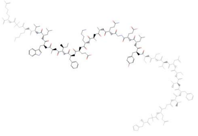	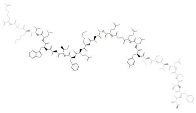	861.4674	861.4667	-0.84
MATCH	5.8	861.4701	861.4667	-3.90	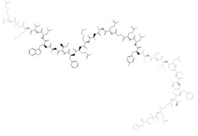	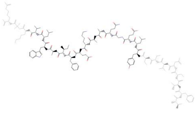	861.4674	861.4667	-0.84
MATCH	5.8	861.4701	861.4667	-3.90	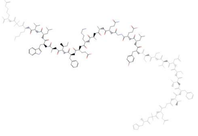	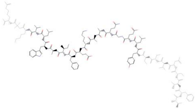	861.4674	861.4667	-0.84
MATCH	26.7	915.5203	915.5229	2.76	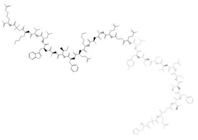	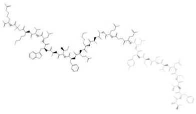	915.5225	915.5229	0.40
MATCH	24.1	972.0625	972.0649	2.44	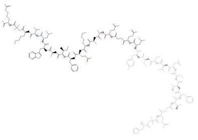	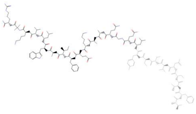	972.0627	972.0649	2.22
MATCH	7.7	1053.5927	1053.5966	3.63	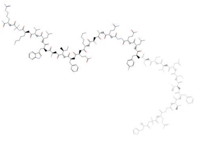	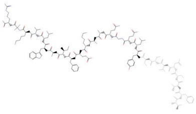	1053.5937	1053.5966	2.74
MATCH	15.5	1097.1096	1097.1126	2.69	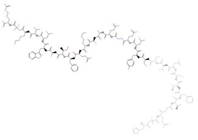	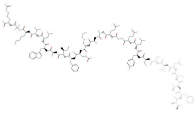	1097.1081	1097.1126	4.07
MATCH	39.7	1140.6251	1140.6286	3.06	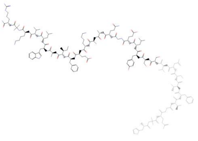	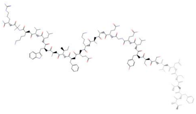	1140.6276	1140.6286	0.90

Metabolite: M12 -408 RT=6.72

Type	score	sub. m/z observed	sub. m/z calculated	sub ppm			met. m/z observed	met. m/z calculated	met. ppm
MATCH	8.6	1190.1592	1190.1628	3.04	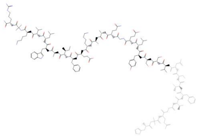	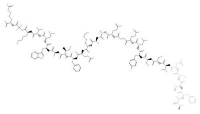	1190.1646	1190.1628	-1.53
MATCH	12.6	1245.7435	1245.7466	2.52	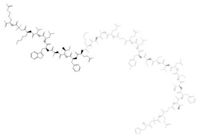	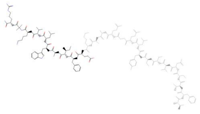	1245.7456	1245.7466	0.83
MISMATCH	-8.2	1341.7122	1341.7161	2.93	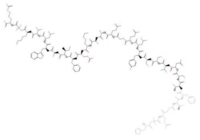		894.8113	894.8113	0.00
MET_MATCH						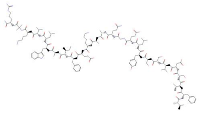	729.1353	729.1341	-1.68
MET_MATCH						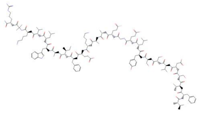	729.1353	729.1341	-1.68
MET_MATCH						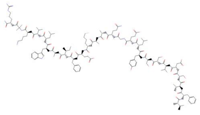	729.1353	729.1341	-1.68
MET_MATCH						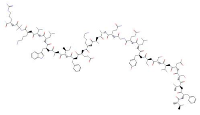	729.1353	729.1341	-1.68
MET_MATCH						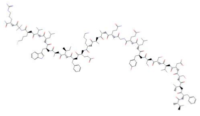	729.1353	729.1341	-1.68
MET_MATCH						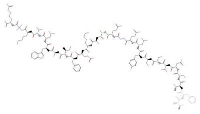	894.8113	894.8132	2.12

MS (+) FT

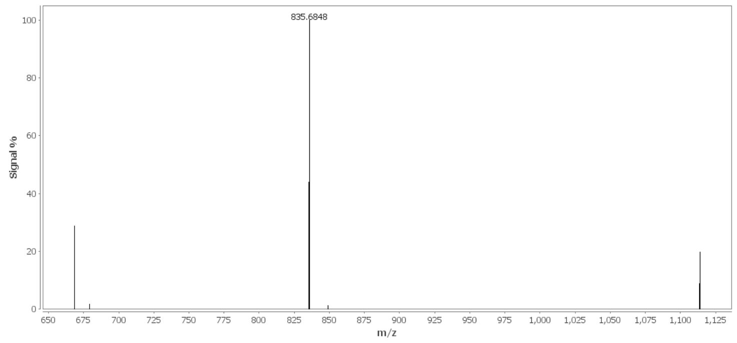

MS (+) FT

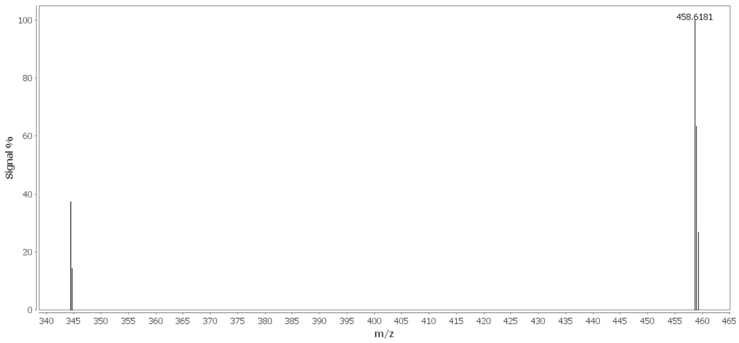

MS2 (+) FT activ = HCD:ce =

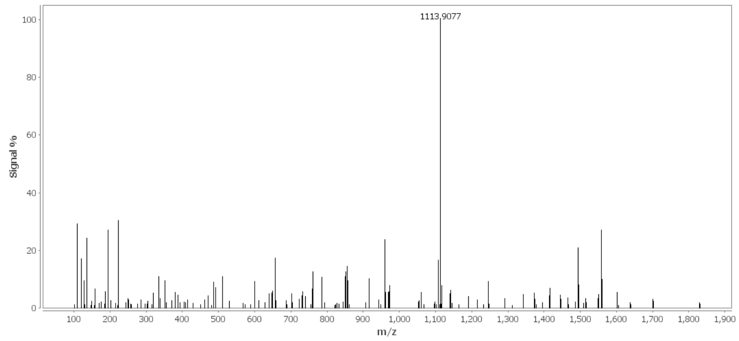

MS2 (+) FT activ = HCD:ce =

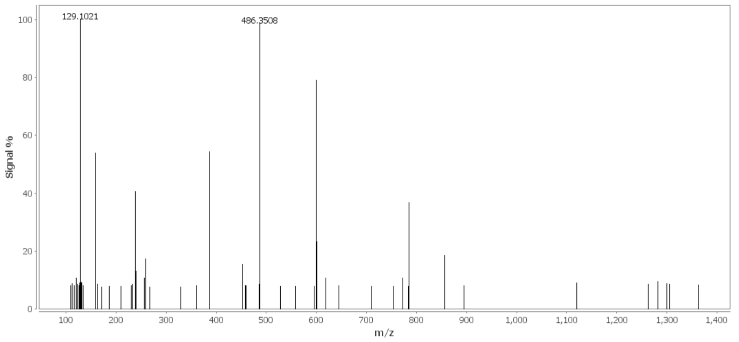

Metabolite: M3 -1965 RT=3.74

Type	score	sub. m/z observed	sub. m/z calculated	sub ppm			met. m/z observed	met. m/z calculated	met. ppm
MATCH	112.4	668.5496	668.5492	-0.61	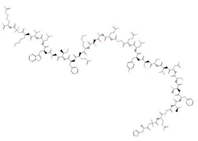	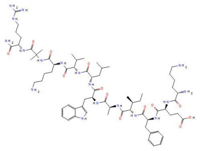	458.6181	458.6187	1.33
						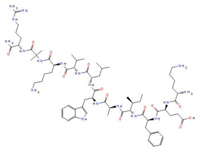	458.6181	458.6187	1.33
						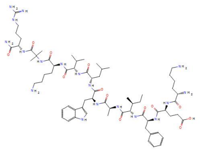	458.6181	458.6187	1.33
MATCH	143.9	835.4371	835.4347	-2.91	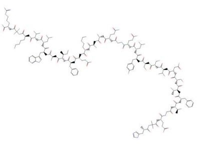	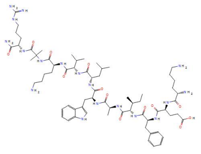	458.6181	458.6187	1.33
						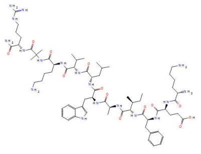	458.6181	458.6187	1.33

Metabolite: M3 -1965 RT=3.74

Type	score	sub. m/z observed	sub. m/z calculated	sub ppm			met. m/z observed	met. m/z calculated	met. ppm
						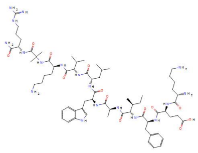	458.6181	458.6187	1.33
MATCH	108.8	1113.5734	1113.5771	3.31	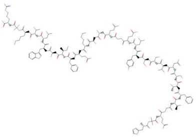	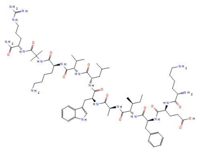	458.6181	458.6187	1.33
						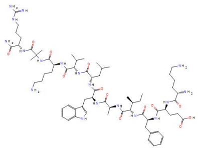	458.6181	458.6187	1.33
						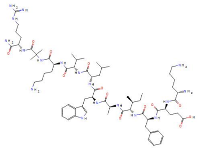	458.6181	458.6187	1.33
MATCH	27.9	120.0808	120.0731	-63.5	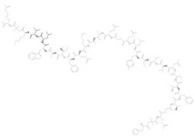	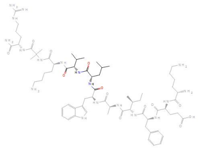	120.0810	120.0731	-65.4
MATCH	27.9	120.0808	120.0808	-0.05	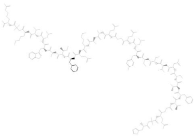	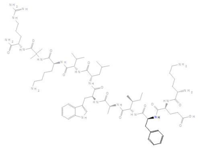	120.0810	120.0808	-1.87
MATCH	27.9	120.0808	120.0865	47.43	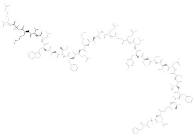	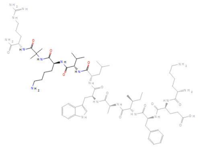	120.0810	120.0865	45.61
MATCH	109.5	129.1021	129.1022	0.79	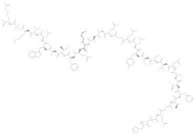	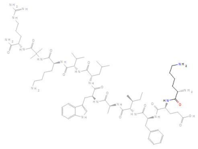	129.1021	129.1022	1.06
MATCH	109.5	129.1021	129.1022	0.79	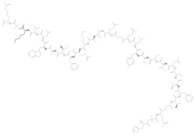	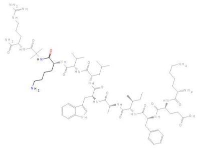	129.1021	129.1022	1.06

Metabolite: M3 -1965 RT=3.74

Type	score	sub. m/z observed	sub. m/z calculated	sub ppm		met. m/z observed	met. m/z calculated	met. ppm
MATCH	109.5	129.1021	129.1022	0.79	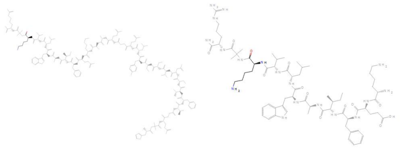	129.1021	129.1022	1.06
MATCH	10.3	130.0649	130.0671	16.63	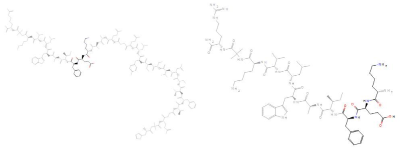	130.0649	130.0671	16.47
MATCH	18.6	259.1870	259.1877	2.62	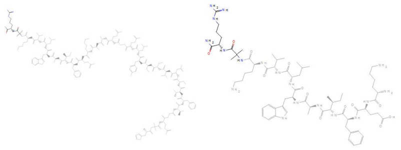	259.1873	259.1877	1.60
MATCH	59.0	387.2815	387.2827	3.02	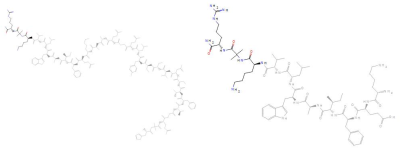	387.2825	387.2827	0.48
MATCH	107.8	486.3500	486.3511	2.28	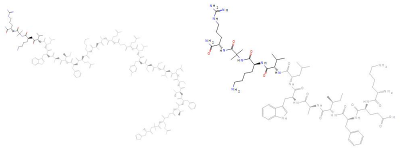	486.3508	486.3511	0.59
MATCH	88.3	599.4338	599.4351	2.24	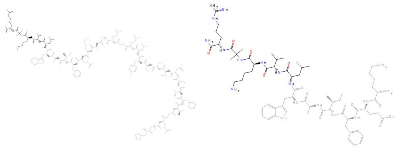	599.4350	599.4351	0.29
MATCH	47.4	785.5122	785.5145	2.93	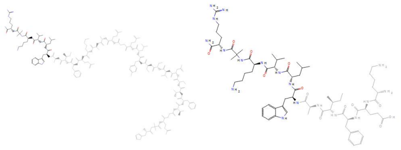	785.5114	785.5145	3.95
MATCH	33.0	856.5492	856.5516	2.77	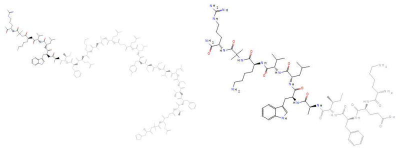	856.5506	856.5516	1.13
MISMATCH	-60.6	159.0913	159.0946	20.67	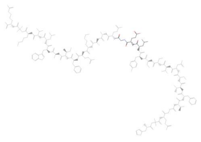	159.0914	159.0914	0.00

Metabolite: M3 -1965 RT=3.74

Type	score	sub. m/z observed	sub. m/z calculated	sub ppm		met. m/z observed	met. m/z calculated	met. ppm
MISMATCH	-14.2	969.6332	969.6356	2.54	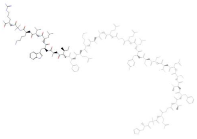	485.3208	485.3208	0.00
MISMATCH	-15.8	1116.7004	1116.7040	3.23	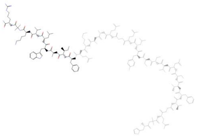	558.8549	558.8549	0.00
MET_MATCH					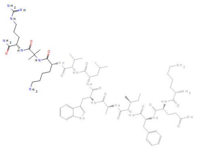	186.6373	186.6395	11.99
MET_MATCH					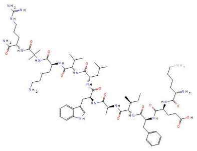	452.9427	452.9432	1.00
MET_MATCH					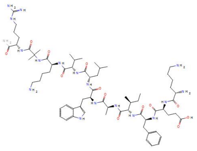	452.9427	452.9432	1.00
MET_MATCH					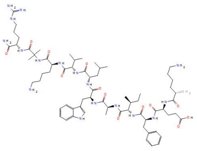	452.9427	452.9432	1.00
MET_MATCH					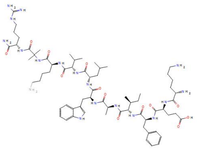	452.9427	452.9432	1.00
MET_MATCH					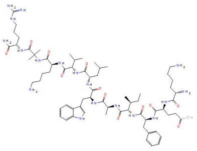	453.2776	453.2871	20.87
MET_MATCH					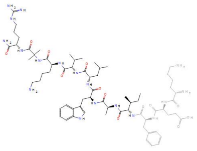	485.3208	485.3215	1.28

Metabolite: M3 -1965 RT=3.74

Type	score	sub. m/z observed	sub. m/z calculated	sub ppm		met. m/z observed	met. m/z calculated	met. ppm
MET_MATCH					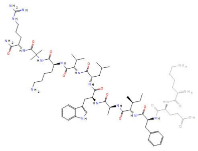	558.8549	558.8557	1.33
MET_MATCH					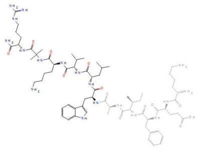	783.5004	783.4988	-2.07

MS (+) FT

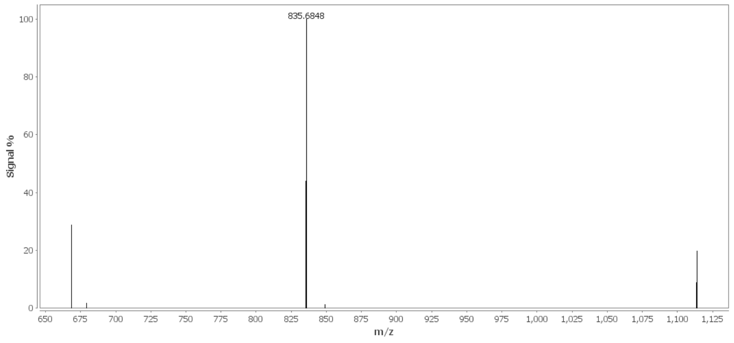

MS (+) FT

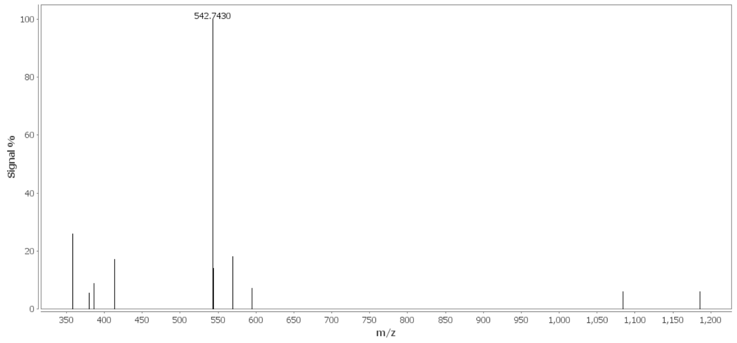

MS2 (+) FT activ = HCD:ce =

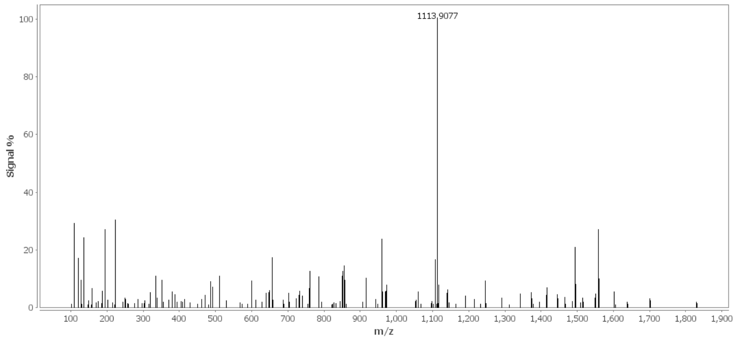

MS2 (+) FT activ = HCD:ce =

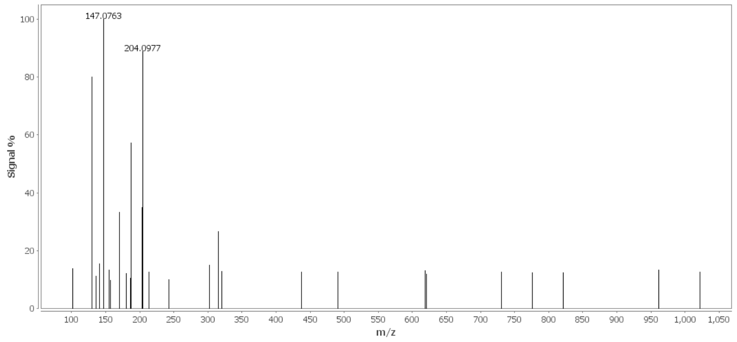

Metabolite: M8 -2254 RT=4.48

Type	score	sub. m/z observed	sub. m/z calculated	sub ppm		met. m/z observed	met. m/z calculated	met. ppm
MATCH	112.4	668.5496	668.5492	-0.61	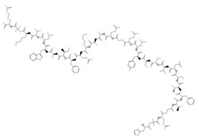 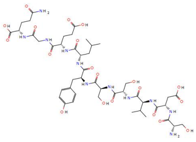	542.7430	542.7433	0.54
MATCH	112.4	668.5496	668.5492	-0.61	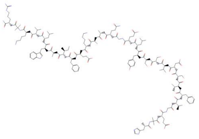 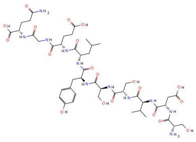	542.7430	542.7433	0.54

Metabolite: M8 -2254 RT=4.48

Type	score	sub. m/z observed	sub. m/z calculated	sub ppm		met. m/z observed	met. m/z calculated	met. ppm
					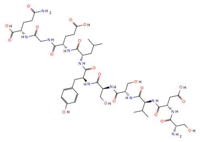	542.7430	542.7433	0.54
MATCH	18.4	668.5496	668.5492	-0.61	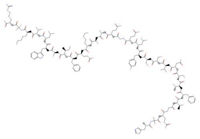 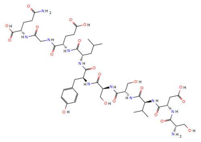	1084.4791	1084.4793	0.24
MATCH	18.4	668.5496	668.5492	-0.61	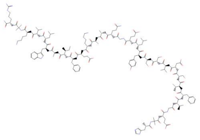 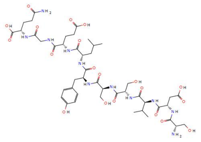	1084.4791	1084.4793	0.24
MATCH	143.9	835.4371	835.4347	-2.91	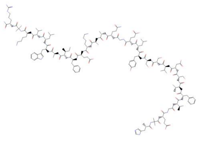 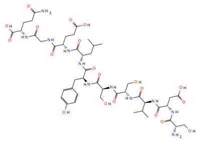	542.7430	542.7433	0.54
MATCH	143.9	835.4371	835.4347	-2.91	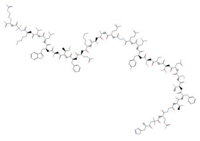 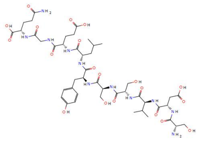	542.7430	542.7433	0.54
					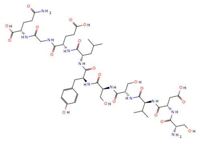	542.7430	542.7433	0.54
MATCH	49.9	835.4371	835.4347	-2.91	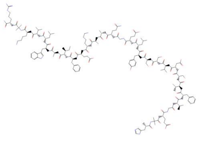 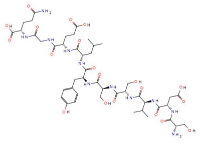	1084.4791	1084.4793	0.24
MATCH	49.9	835.4371	835.4347	-2.91	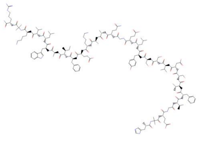 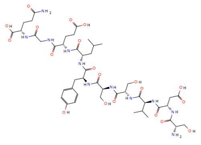	1084.4791	1084.4793	0.24
MATCH	108.8	1113.5734	1113.5771	3.31	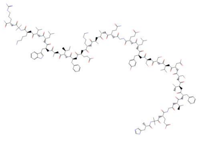 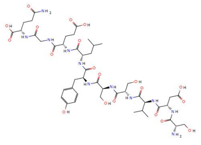	542.7430	542.7433	0.54

Metabolite: M8 -2254 RT=4.48

Type	score	sub. m/z observed	sub. m/z calculated	sub ppm			met. m/z observed	met. m/z calculated	met. ppm
						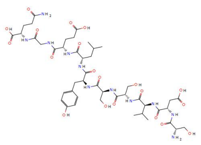	542.7430	542.7433	0.54
						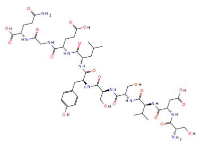	542.7430	542.7433	0.54
MATCH	101.0	147.0760	147.0764	2.67	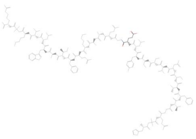	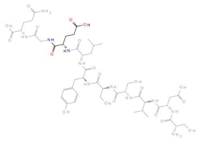	147.0763	147.0764	0.70
MATCH	37.5	203.0657	203.0662	2.70	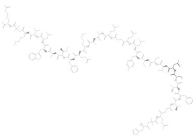	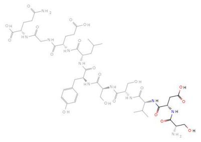	203.0655	203.0662	3.88
						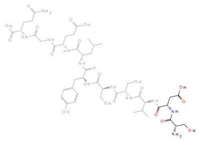	203.0655	203.0662	3.88
MATCH	11.8	243.1333	243.1339	2.72	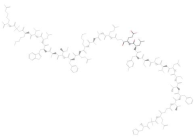	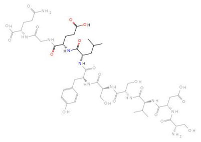	243.1336	243.1339	1.54
MATCH	11.8	243.1333	243.1339	2.72	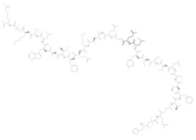	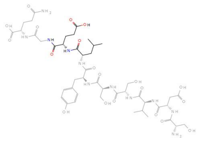	243.1336	243.1339	1.54
MATCH	16.3	302.1338	302.1347	2.91	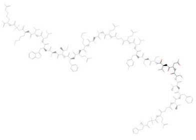	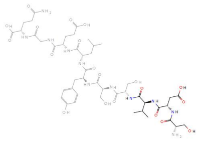	302.1336	302.1347	3.40
MATCH	16.3	302.1338	302.1347	2.91	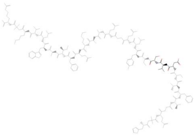	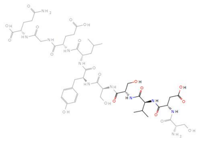	302.1336	302.1347	3.40

Metabolite: M8 -2254 RT=4.48

Type	score	sub. m/z observed	sub. m/z calculated	sub ppm		met. m/z observed	met. m/z calculated	met. ppm
MATCH	16.3	302.1338	302.1347	2.91	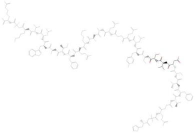 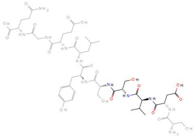	302.1336	302.1347	3.40
					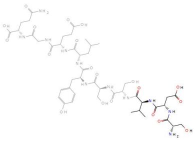	302.1336	302.1347	3.40
MATCH	27.6	906.5161	906.5176	1.60	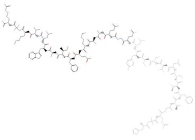 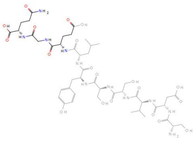	315.1299	315.1299	0.19
MATCH	27.6	906.5161	906.5176	1.60	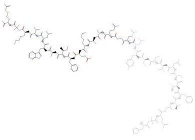 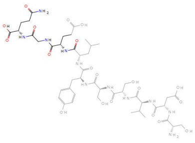	315.1299	315.1299	0.19
MATCH	58.8	960.4032	960.4058	2.64	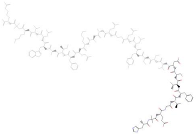 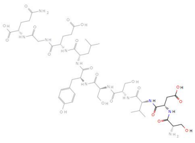	203.0655	203.0662	3.88
					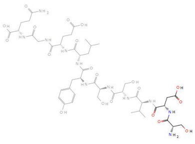	203.0655	203.0662	3.88
MATCH	20.3	1059.4718	1059.4742	2.23	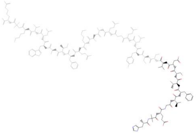 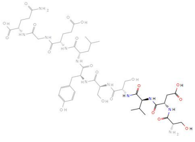	302.1336	302.1347	3.40
					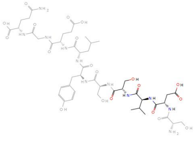	302.1336	302.1347	3.40
					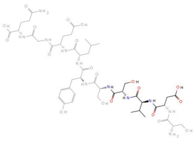	302.1336	302.1347	3.40

Metabolite: M8 -2254 RT=4.48

Type	score	sub. m/z observed	sub. m/z calculated	sub ppm			met. m/z observed	met. m/z calculated	met. ppm
						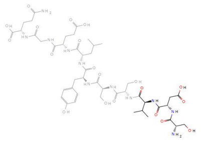	302.1336	302.1347	3.40
MATCH	13.0	1378.5812	1378.5910	7.09	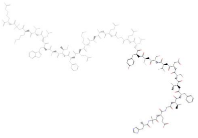	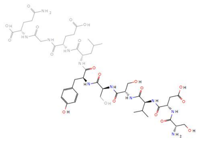	621.2510	621.2515	0.81
MATCH	13.0	1378.5812	1378.5910	7.09	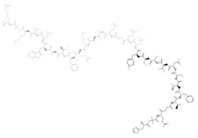	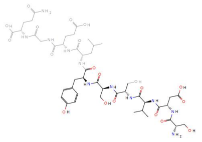	621.2510	621.2515	0.81
MATCH	13.0	1378.5812	1378.5910	7.09	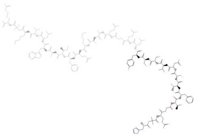	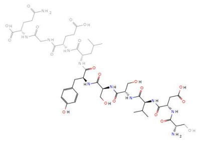	621.2510	621.2515	0.81
MATCH	91.8	1700.9942	1700.9959	0.96	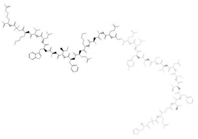	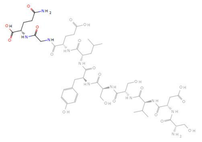	204.0977	204.0979	0.73
MISMATCH	-12.0	102.0552	102.0550	-2.07	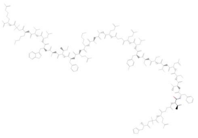		102.0552	102.0552	0.00
MISMATCH	-18.2	320.1233	320.1241	2.47	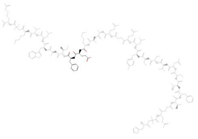		320.1230	320.1230	0.00
MISMATCH	-14.8	1396.5975	1396.6016	2.89	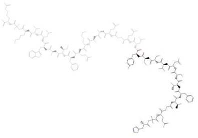		320.1230	320.1230	0.00
MET_MATCH						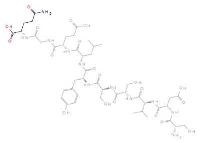	130.0499	130.0499	-0.05

Metabolite: M8 -2254 RT=4.48

Type	score	sub. m/z observed	sub. m/z calculated	sub ppm		met. m/z observed	met. m/z calculated	met. ppm
MET_MATCH					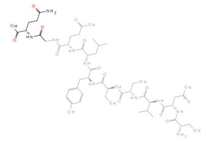	187.0713	187.0713	-0.01

MS (+) FT

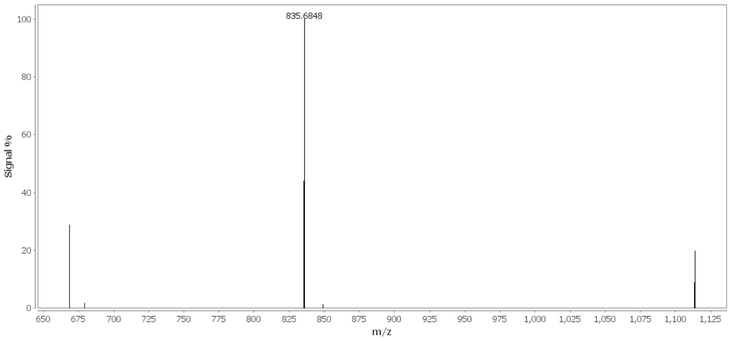

MS (+) FT

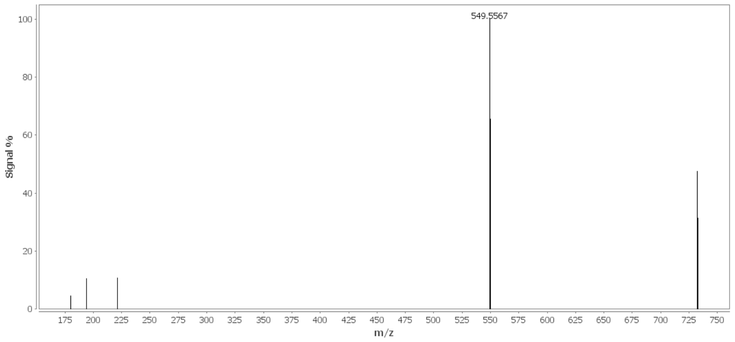

MS2 (+) FT activ = HCD:ce =

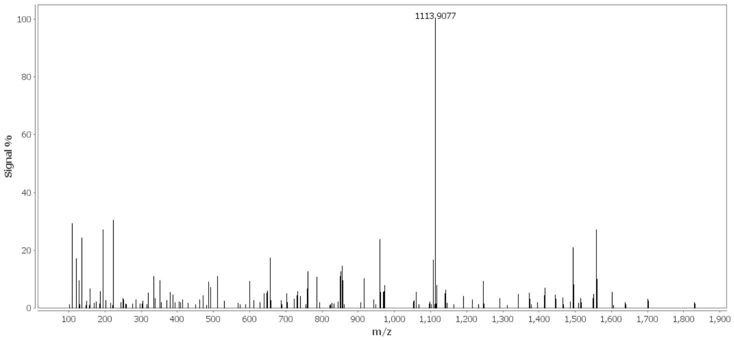

MS2 (+) FT activ = HCD:ce =

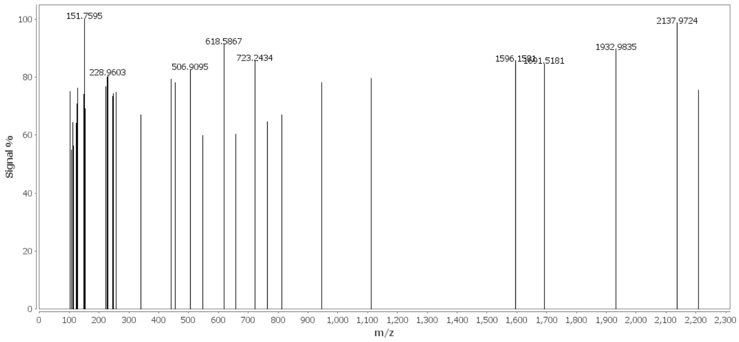

Metabolite: M11 -1145 RT=5.90

Type	score	sub. m/z observed	sub. m/z calculated	sub ppm			met. m/z observed	met. m/z calculated	met. ppm
MATCH	87.2	668.5496	668.5492	-0.61	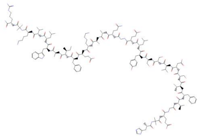	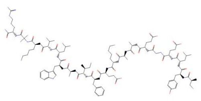	549.3060	549.3059	-0.18
MATCH	87.2	668.5496	668.5492	-0.61	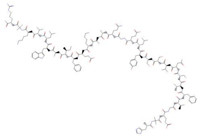	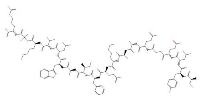	549.3060	549.3059	-0.18
						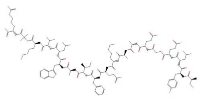	549.3060	549.3059	-0.18

Metabolite: M11 -1145 RT=5.90

Type	score	sub. m/z observed	sub. m/z calculated	sub ppm			met. m/z observed	met. m/z calculated	met. ppm
MATCH	46.3	668.5496	668.5492	-0.61	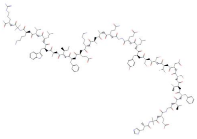	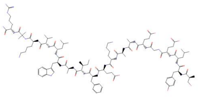	732.0716	732.0722	0.75
MATCH	46.3	668.5496	668.5492	-0.61	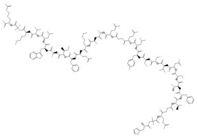	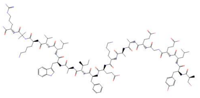	732.0716	732.0722	0.75
						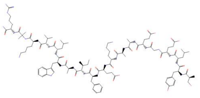	732.0716	732.0722	0.75
MATCH	118.8	835.4371	835.4347	-2.91	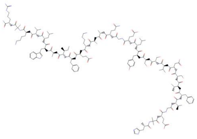	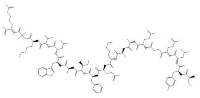	549.3060	549.3059	-0.18
MATCH	118.8	835.4371	835.4347	-2.91	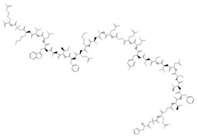	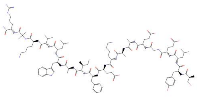	549.3060	549.3059	-0.18
						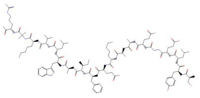	549.3060	549.3059	-0.18
MATCH	77.8	835.4371	835.4347	-2.91	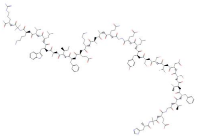	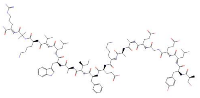	732.0716	732.0722	0.75
MATCH	77.8	835.4371	835.4347	-2.91	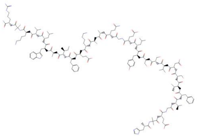	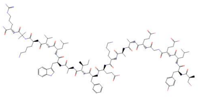	732.0716	732.0722	0.75
						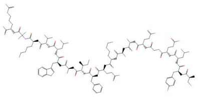	732.0716	732.0722	0.75

Metabolite: M11 -1145 RT=5.90

Type	score	sub. m/z observed	sub. m/z calculated	sub ppm			met. m/z observed	met. m/z calculated	met. ppm
MATCH	83.6	1113.5734	1113.5771	3.31	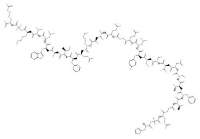	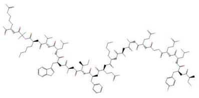	549.3060	549.3059	-0.18
MATCH	83.6	1113.5734	1113.5771	3.31	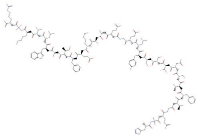	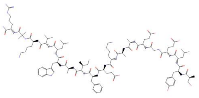	549.3060	549.3059	-0.18
						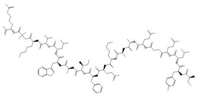	549.3060	549.3059	-0.18
MATCH	42.7	1113.5734	1113.5771	3.31	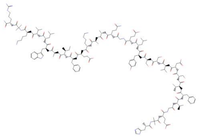	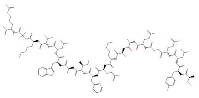	732.0716	732.0722	0.75
MATCH	42.7	1113.5734	1113.5771	3.31	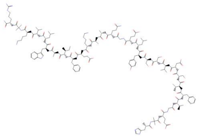	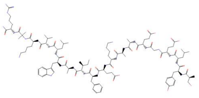	732.0716	732.0722	0.75
						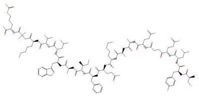	732.0716	732.0722	0.75
MISMATCH	-65.8	1509.6797	1509.6856	3.95	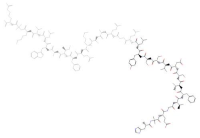		122.3850	122.3850	0.00
MET_MATCH						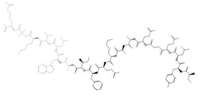	339.6689	339.6765	22.50

MS (+) FT

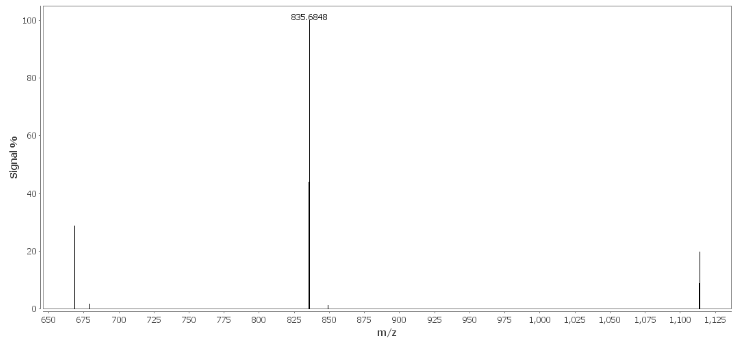

MS (+) FT

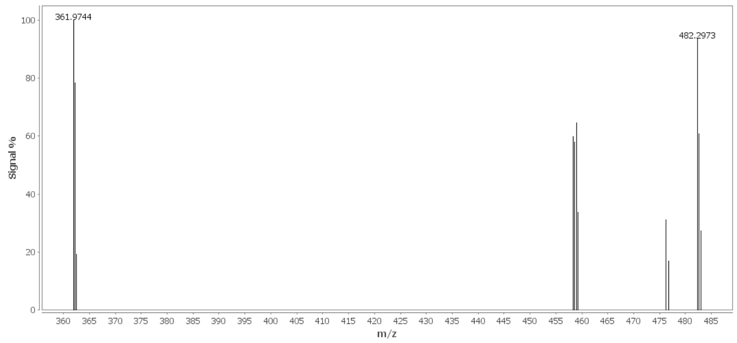

MS2 (+) FT activ = HCD:ce =

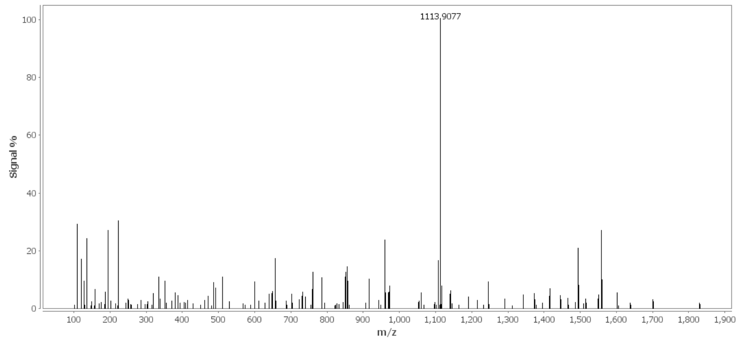

MS2 (+) FT activ = HCD:ce =

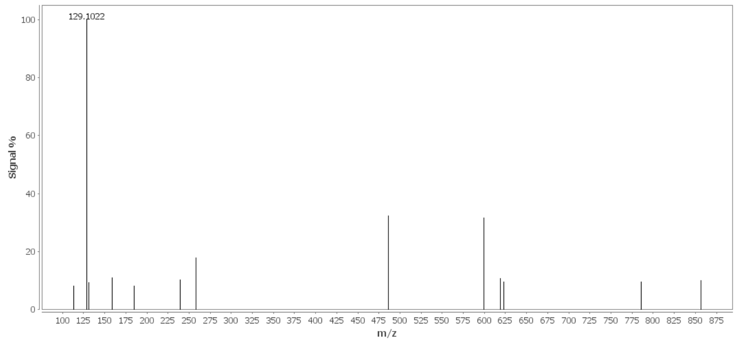

Metabolite: M6 -1894 RT=4.00

Type	score	sub. m/z observed	sub. m/z calculated	sub ppm		met. m/z observed	met. m/z calculated	met. ppm
MATCH	112.4	668.5496	668.5492	-0.61	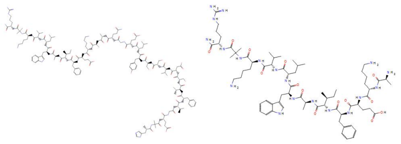	361.9744	361.9751	1.92
MATCH	112.4	668.5496	668.5492	-0.61	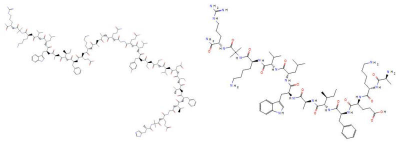	361.9744	361.9751	1.92
					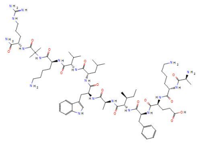	361.9744	361.9751	1.92
MATCH	105.9	668.5496	668.5492	-0.61	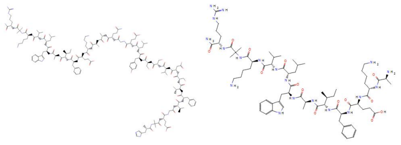	482.2973	482.2978	0.85
MATCH	105.9	668.5496	668.5492	-0.61	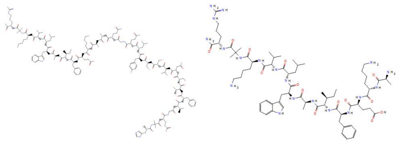	482.2973	482.2978	0.85

Metabolite: M6 -1894 RT=4.00

Type	score	sub. m/z observed	sub. m/z calculated	sub ppm			met. m/z observed	met. m/z calculated	met. ppm
						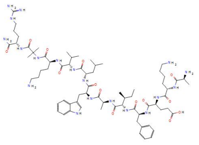	482.2973	482.2978	0.85
MATCH	143.9	835.4371	835.4347	-2.91	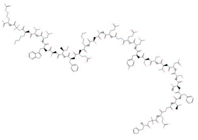	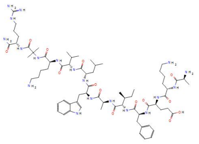	361.9744	361.9751	1.92
MATCH	143.9	835.4371	835.4347	-2.91	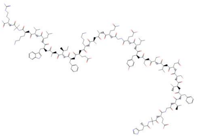	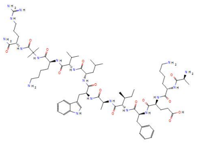	361.9744	361.9751	1.92
						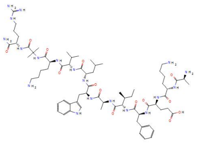	361.9744	361.9751	1.92
MATCH	137.4	835.4371	835.4347	-2.91	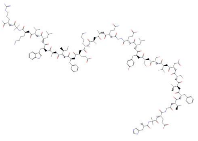	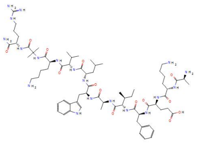	482.2973	482.2978	0.85
MATCH	137.4	835.4371	835.4347	-2.91	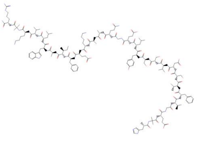	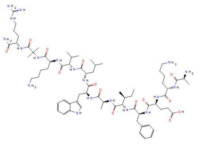	482.2973	482.2978	0.85
						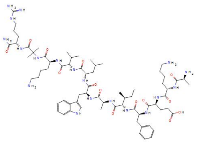	482.2973	482.2978	0.85
MATCH	108.8	1113.5734	1113.5771	3.31	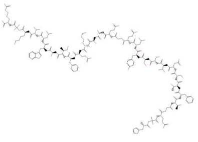	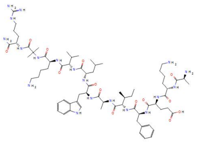	361.9744	361.9751	1.92
MATCH	108.8	1113.5734	1113.5771	3.31	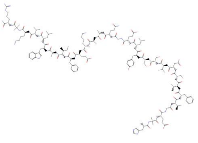	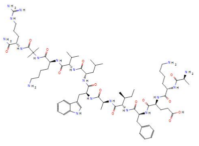	361.9744	361.9751	1.92

Metabolite: M6 -1894 RT=4.00

Type	score	sub. m/z observed	sub. m/z calculated	sub ppm			met. m/z observed	met. m/z calculated	met. ppm
						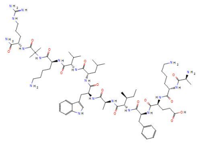	361.9744	361.9751	1.92
MATCH	102.3	1113.5734	1113.5771	3.31	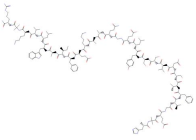	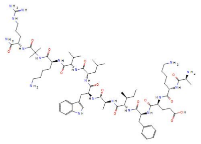	482.2973	482.2978	0.85
MATCH	102.3	1113.5734	1113.5771	3.31	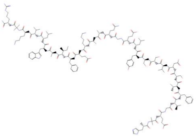	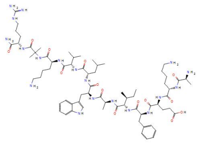	482.2973	482.2978	0.85
						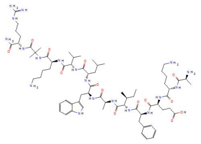	482.2973	482.2978	0.85
MATCH	109.5	129.1021	129.1022	0.79	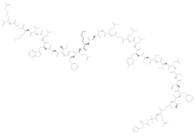	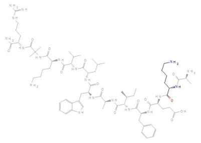	129.1022	129.1022	0.12
MATCH	109.5	129.1021	129.1022	0.79	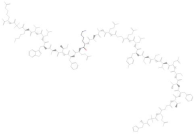	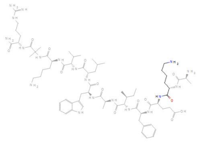	129.1022	129.1022	0.12
MATCH	109.5	129.1021	129.1022	0.79	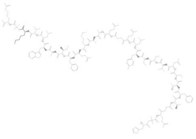	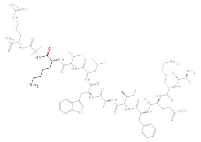	129.1022	129.1022	0.12
MATCH	109.5	129.1021	129.1022	0.79	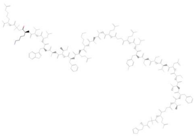	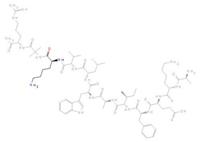	129.1022	129.1022	0.12
MATCH	41.3	486.3500	486.3511	2.28	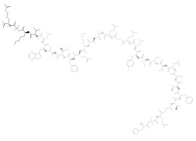	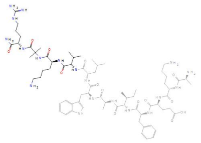	486.3507	486.3511	0.82

Metabolite: M6 -1894 RT=4.00

Type	score	sub. m/z observed	sub. m/z calculated	sub ppm		met. m/z observed	met. m/z calculated	met. ppm
MATCH	41.0	599.4338	599.4351	2.24	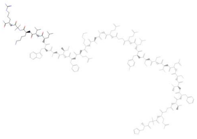 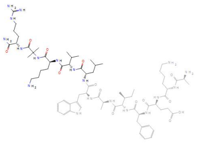	599.4345	599.4351	1.13
MATCH	20.1	785.5122	785.5145	2.93	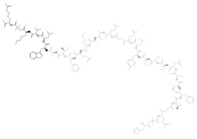 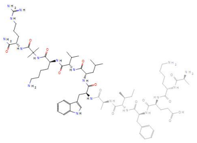	785.5140	785.5145	0.53
MATCH	24.4	856.5492	856.5516	2.77	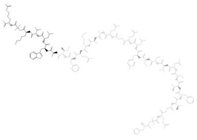 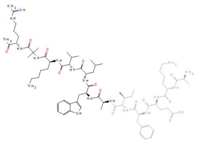	856.5515	856.5516	0.06
MISMATCH	-17.7	159.0913	159.0946	20.67	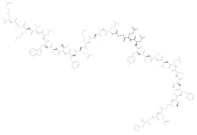	159.0917	159.0917	0.00
MISMATCH	-18.7	1245.7435	1245.7466	2.52	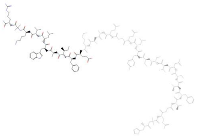	623.3763	623.3763	0.00
MET_MATCH					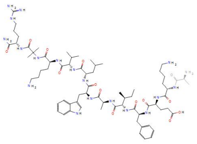	458.6088	458.6187	21.59
MET_MATCH					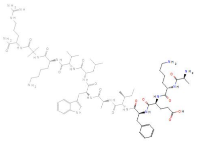	476.2499	476.2504	0.93
MET_MATCH					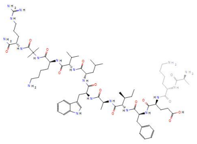	623.3763	623.3770	0.99
