# Supplementary material for: Software-aided approach to investigate peptide structure and metabolic susceptibility of amide bonds in peptide drugs based on high resolution mass spectrometry
Source: PLoS One. 2017 Nov 1;12(11):e0186461. doi: 10.1371/journal.pone.0186461 (PMC5665424; doi:10.1371/journal.pone.0186461)
Supplement: S1 File — (ZIP) [file pone.0186461.s007.zip › SFiles/S47_File.pdf]

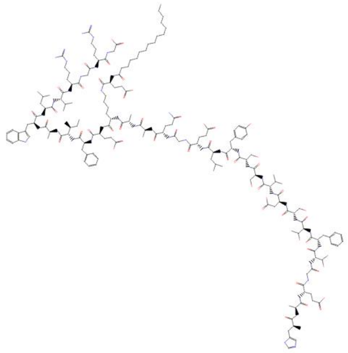

Liraglutide

Property name	Property value
Time	0min, 2min, 4min, 24min
Instrument	ddMS2
Matrix	DPP4

Chromatograms

Time=0min

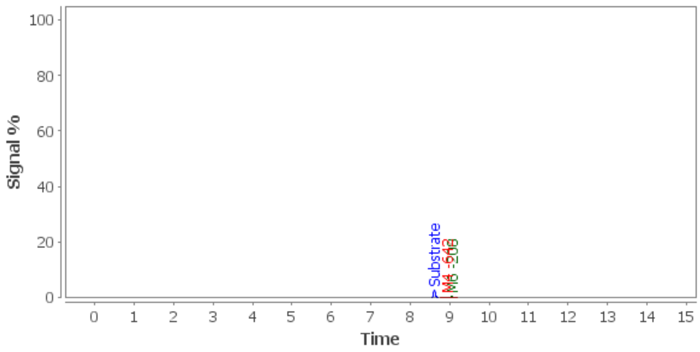

Time=2min

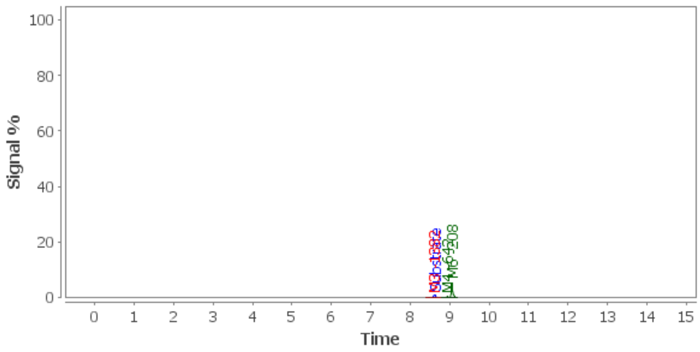

Time=4min

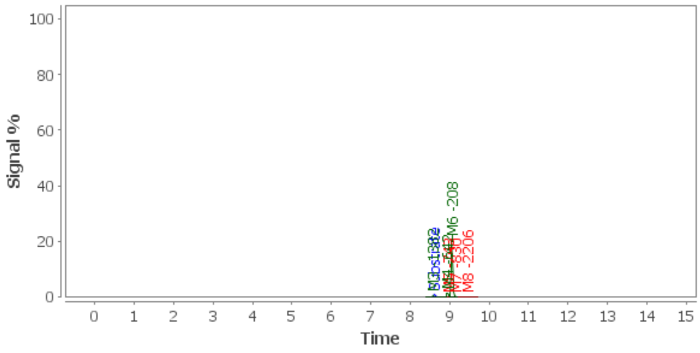

Time=24min

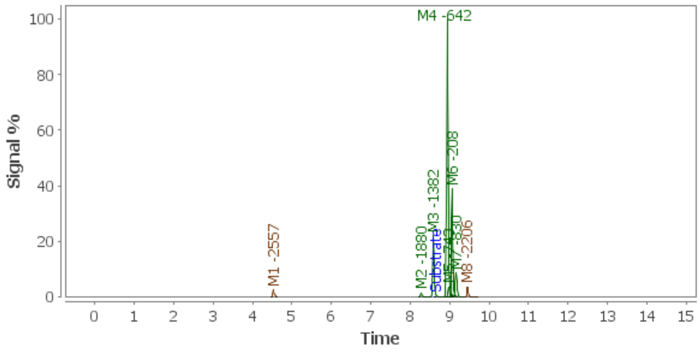

Custom Charts

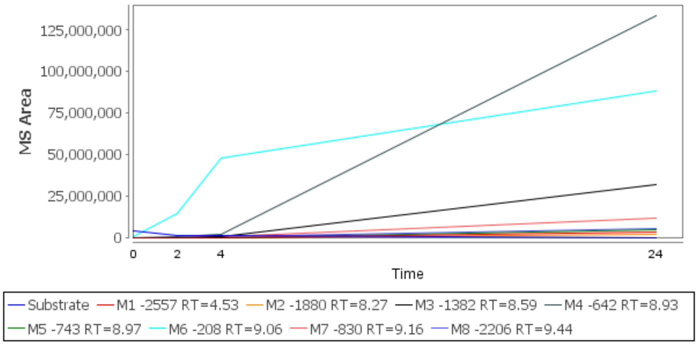
125,000,000
100,000,000
75,000,000
50,000,000
25,000,000
0
MS Area
0
2
4
24
Time
Substrate
M1 -2557 RT=4.53
M2 -1880 RT=8.27
M3 -1382 RT=8.59
M4 -642 RT=8.93
M5 -743 RT=8.97
M6 -208 RT=9.06
M7 -830 RT=9.16
M8 -2206 RT=9.44

Fragmentation

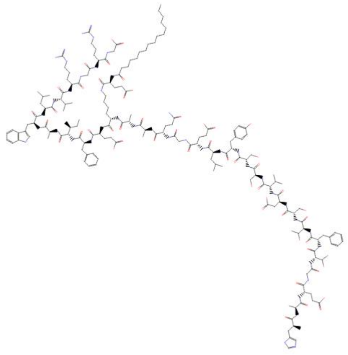

Liraglutide

MS (+) FT

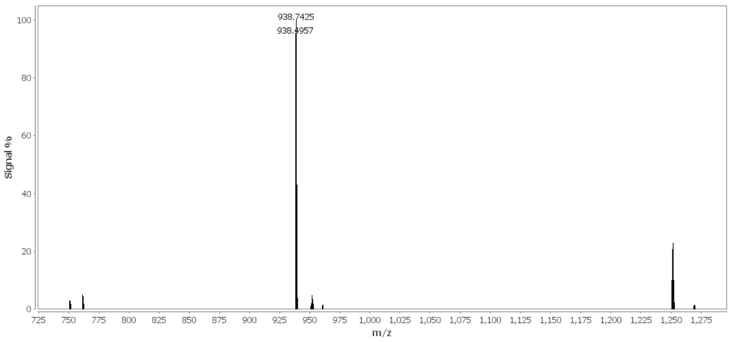

MS (+) FT

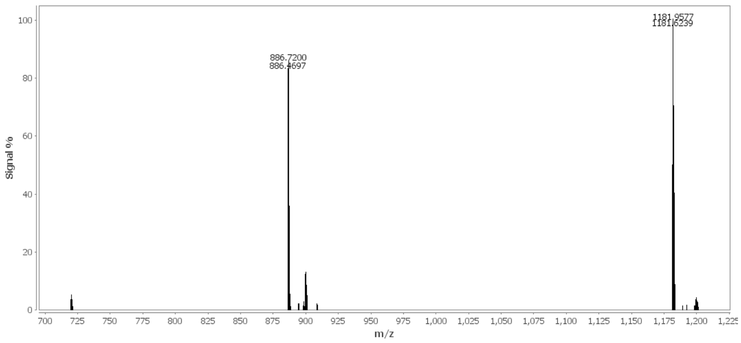

MS2 (+) FT activ = HCD:ce =

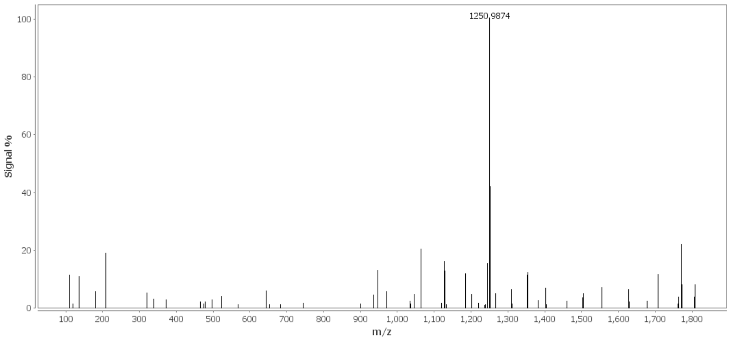

MS2 (+) FT activ = HCD:ce =

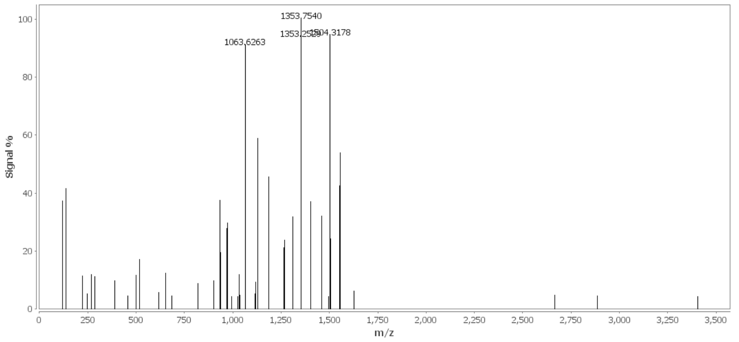

Metabolite: M6 -208 RT=9.06

Type	score	sub. m/z observed	sub. m/z calculated	sub ppm			met. m/z observed	met. m/z calculated	met. ppm
MATCH	43.0	750.7942	750.7966	3.21	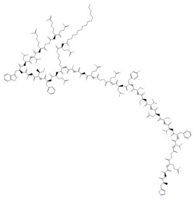	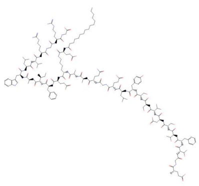	886.2192	886.2199	0.79
MATCH	43.0	750.7942	750.7966	3.21	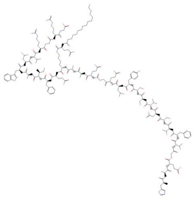	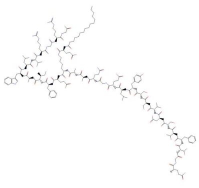	886.2192	886.2199	0.79
						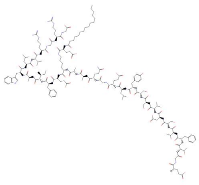	886.2192	886.2199	0.79

Metabolite: M6 -208 RT=9.06

Type	score	sub. m/z observed	sub. m/z calculated	sub ppm			met. m/z observed	met. m/z calculated	met. ppm
MATCH	51.3	750.7942	750.7966	3.21	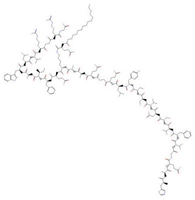	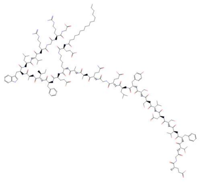	1181.2898	1181.2908	0.82
MATCH	51.3	750.7942	750.7966	3.21	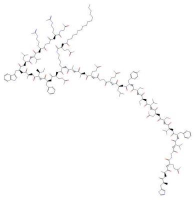	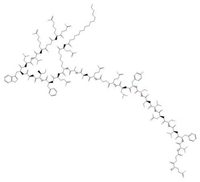	1181.2898	1181.2908	0.82
						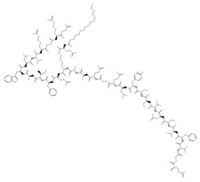	1181.2898	1181.2908	0.82
MATCH	88.5	938.2475	938.2439	-3.87	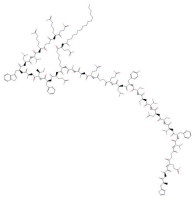	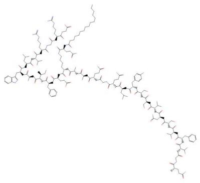	886.2192	886.2199	0.79
MATCH	88.5	938.2475	938.2439	-3.87	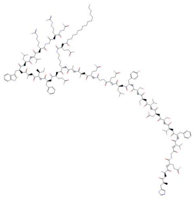	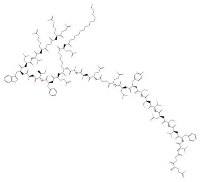	886.2192	886.2199	0.79
						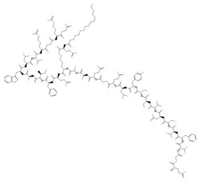	886.2192	886.2199	0.79
MATCH	96.8	938.2475	938.2439	-3.87	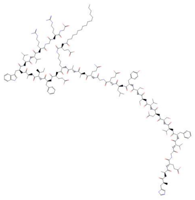	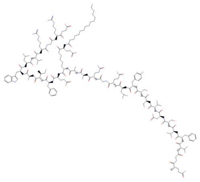	1181.2898	1181.2908	0.82
MATCH	96.8	938.2475	938.2439	-3.87	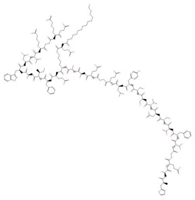	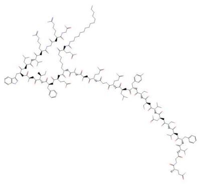	1181.2898	1181.2908	0.82
						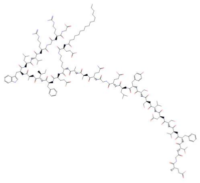	1181.2898	1181.2908	0.82

Metabolite: M6 -208 RT=9.06

Type	score	sub. m/z observed	sub. m/z calculated	sub ppm			met. m/z observed	met. m/z calculated	met. ppm
MATCH	51.8	1250.6532	1250.6561	2.29	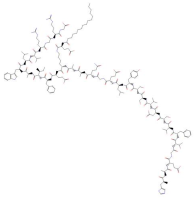	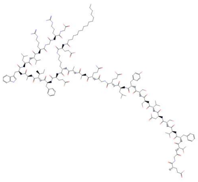	886.2192	886.2199	0.79
MATCH	51.8	1250.6532	1250.6561	2.29	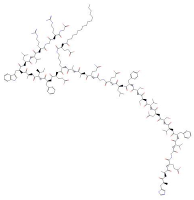	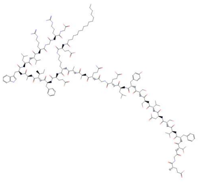	886.2192	886.2199	0.79
						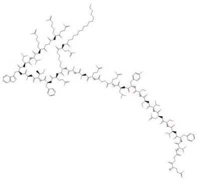	886.2192	886.2199	0.79
MATCH	60.1	1250.6532	1250.6561	2.29	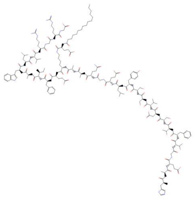	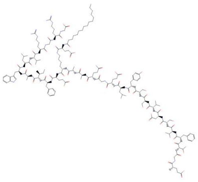	1181.2898	1181.2908	0.82
MATCH	60.1	1250.6532	1250.6561	2.29	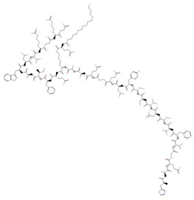	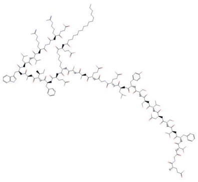	1181.2898	1181.2908	0.82
						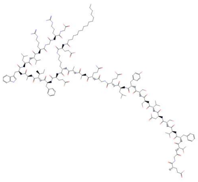	1181.2898	1181.2908	0.82
MATCH	38.8	120.0807	120.0788	-16.4	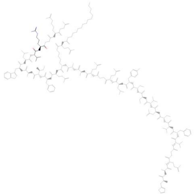	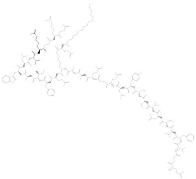	120.0808	120.0788	-17.0
MATCH	52.5	136.0765	136.0693	-53.2	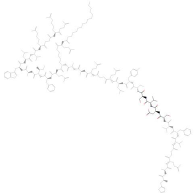	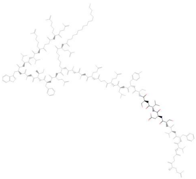	136.0756	136.0693	-46.3
MATCH	52.5	136.0765	136.0693	-53.2	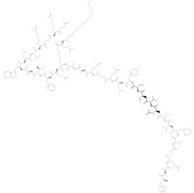	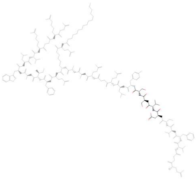	136.0756	136.0693	-46.3

Metabolite: M6 -208 RT=9.06

Type	score	sub. m/z observed	sub. m/z calculated	sub ppm			met. m/z observed	met. m/z calculated	met. ppm
MATCH	52.5	136.0765	136.0706	-43.3	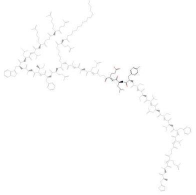	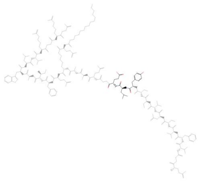	136.0756	136.0706	-36.4
MATCH	52.5	136.0765	136.0706	-43.3	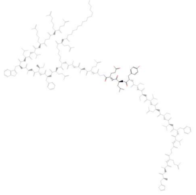	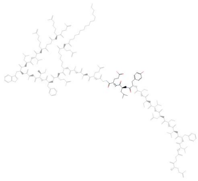	136.0756	136.0706	-36.4
MATCH	52.5	136.0765	136.0737	-20.7	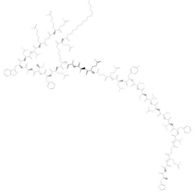	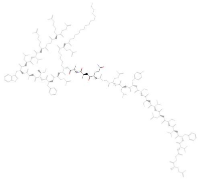	136.0756	136.0737	-13.8
MATCH	52.5	136.0765	136.0737	-20.7	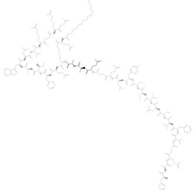	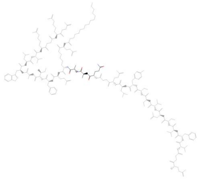	136.0756	136.0737	-13.8
MATCH	14.0	478.2032	478.2045	2.68	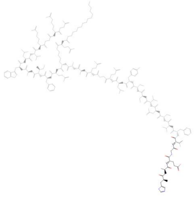	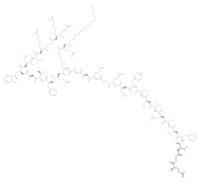	270.1075	270.1084	3.57
MATCH	14.1	496.2139	496.2150	2.32	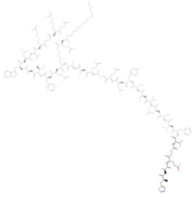	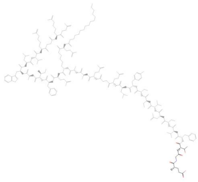	288.1177	288.1190	4.64
MATCH	13.7	652.3655	652.3671	2.43	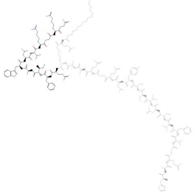	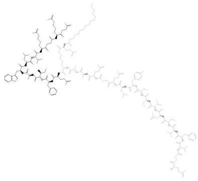	652.3646	652.3671	3.83
MATCH	11.4	900.0483	900.0507	2.75	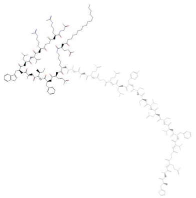	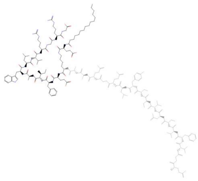	900.0477	900.0507	3.34
MATCH	42.0	935.5676	935.5693	1.80	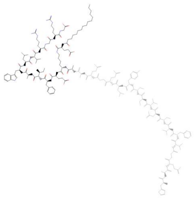	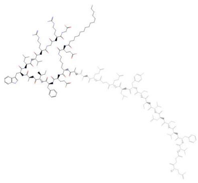	935.5675	935.5693	1.94

Metabolite: M6 -208 RT=9.06

Type	score	sub. m/z observed	sub. m/z calculated	sub ppm			met. m/z observed	met. m/z calculated	met. ppm
MATCH	33.4	971.0854	971.0878	2.54	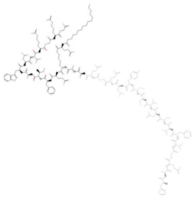	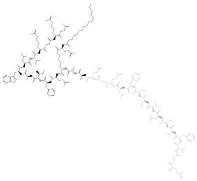	971.0868	971.0878	1.03
MATCH	14.3	1035.1141	1035.1171	2.96	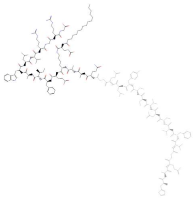	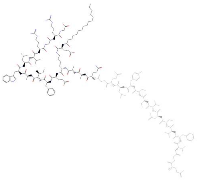	1035.1139	1035.1171	3.13
MATCH	111.6	1063.6253	1063.6279	2.43	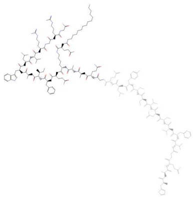	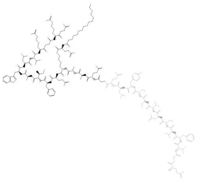	1063.6263	1063.6279	1.46
MATCH	10.9	1119.1410	1119.1439	2.54	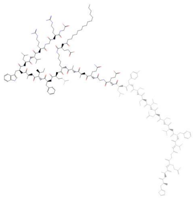	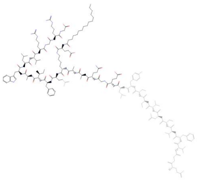	1119.1335	1119.1439	9.24
MATCH	10.9	1119.1410	1119.1439	2.54	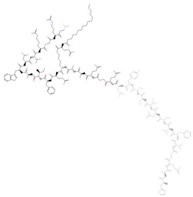	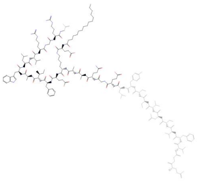	1119.1335	1119.1439	9.24
MATCH	10.9	1119.1410	1119.1439	2.54	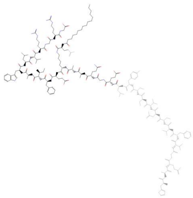	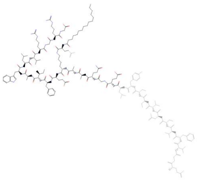	1119.1335	1119.1439	9.24
MATCH	10.9	1119.1410	1119.1439	2.54	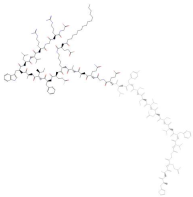	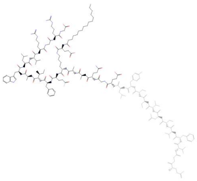	1119.1335	1119.1439	9.24
MATCH	10.9	1119.1410	1119.1439	2.54	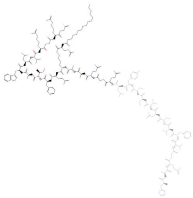	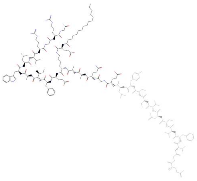	1119.1335	1119.1439	9.24
MATCH	10.9	1119.1410	1119.1439	2.54	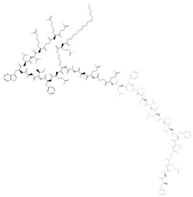	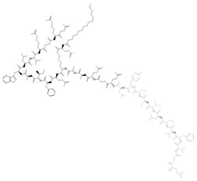	1119.1335	1119.1439	9.24

Metabolite: M6 -208 RT=9.06

Type	score	sub. m/z observed	sub. m/z calculated	sub ppm			met. m/z observed	met. m/z calculated	met. ppm
MATCH	10.9	1119.1410	1119.1439	2.54	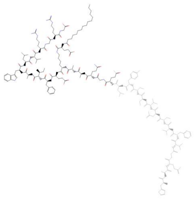	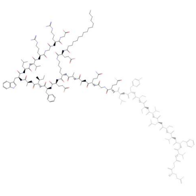	1119.1335	1119.1439	9.24
MATCH	10.9	1119.1410	1119.1439	2.54	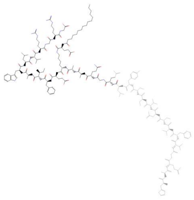	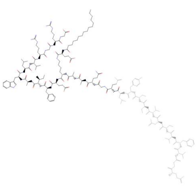	1119.1335	1119.1439	9.24
MATCH	75.0	1128.1470	1128.1492	1.88	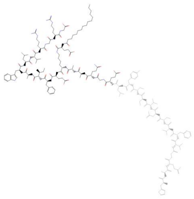	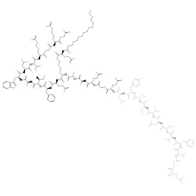	1128.1467	1128.1492	2.15
MATCH	57.3	1184.6888	1184.6912	2.06	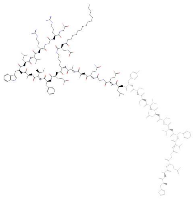	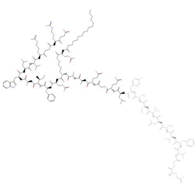	1184.6924	1184.6912	-0.99
MATCH	9.3	1201.5091	1201.5120	2.42	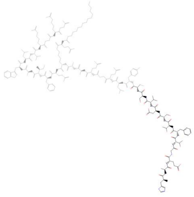	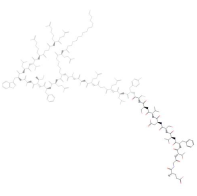	993.4117	993.4160	4.33
MATCH	9.3	1201.5091	1201.5120	2.42	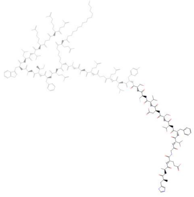	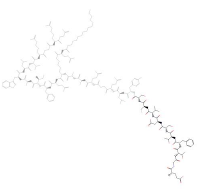	993.4117	993.4160	4.33
MATCH	9.3	1201.5091	1201.5120	2.42	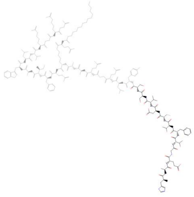	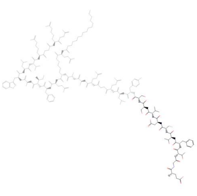	993.4117	993.4160	4.33
MATCH	9.3	1201.5091	1201.5120	2.42	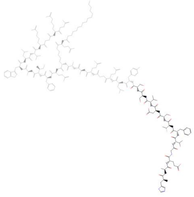	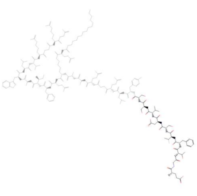	993.4117	993.4160	4.33
MATCH	9.3	1201.5091	1201.5120	2.42	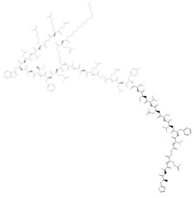	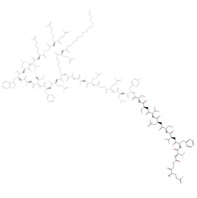	993.4117	993.4160	4.33

Metabolite: M6 -208 RT=9.06

Type	score	sub. m/z observed	sub. m/z calculated	sub ppm			met. m/z observed	met. m/z calculated	met. ppm
MATCH	25.3	1266.2207	1266.2229	1.70	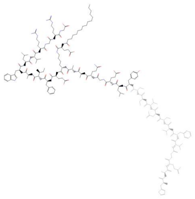	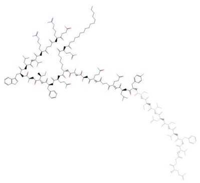	1266.2191	1266.2229	2.98
MATCH	36.2	1309.7364	1309.7389	1.93	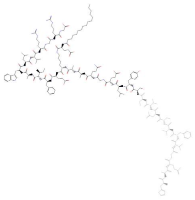	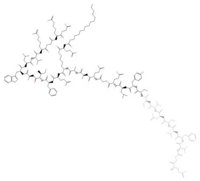	1309.7452	1309.7389	-4.79
MATCH	105.2	1353.2522	1353.2549	2.01	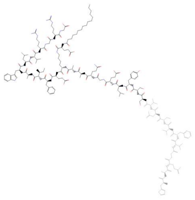	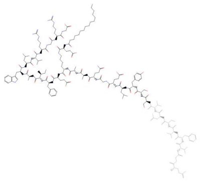	1353.2529	1353.2549	1.46
MATCH	35.7	1402.7850	1402.7891	2.90	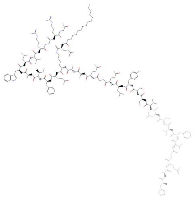	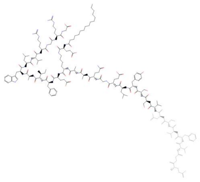	1402.7916	1402.7891	-1.80
MATCH	25.9	1460.2991	1460.3026	2.38	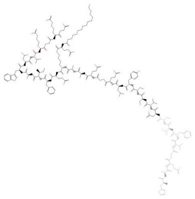	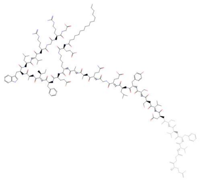	1460.2966	1460.3026	4.06
MATCH	55.3	1503.8175	1503.8186	0.72	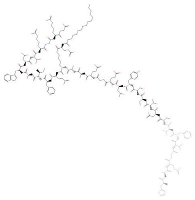	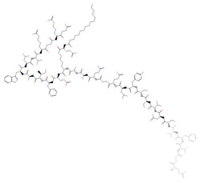	1503.8138	1503.8186	3.20
MATCH	47.4	1554.3420	1554.3424	0.26	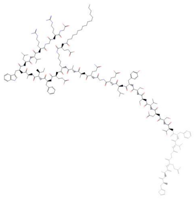	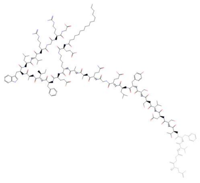	1554.3401	1554.3424	1.50
MET_MATCH						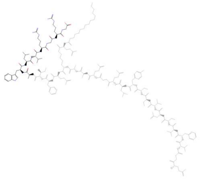	457.7677	457.7696	4.12
MET_MATCH						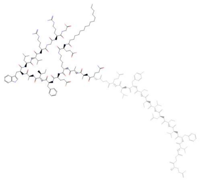	1026.6047	1026.6039	-0.85

Metabolite: M6 -208 RT=9.06

Type	score	sub. m/z observed	sub. m/z calculated	sub ppm			met. m/z observed	met. m/z calculated	met. ppm
MET_MATCH						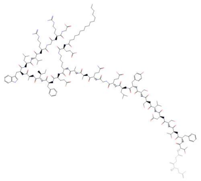	1113.5980	1113.5939	-3.71
MET_MATCH						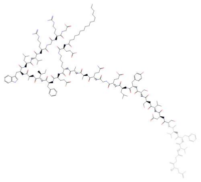	1495.3068	1495.3053	-0.97

MS (+) FT

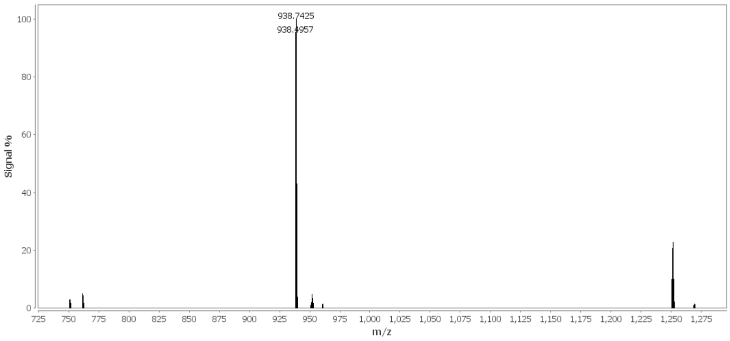

MS (+) FT

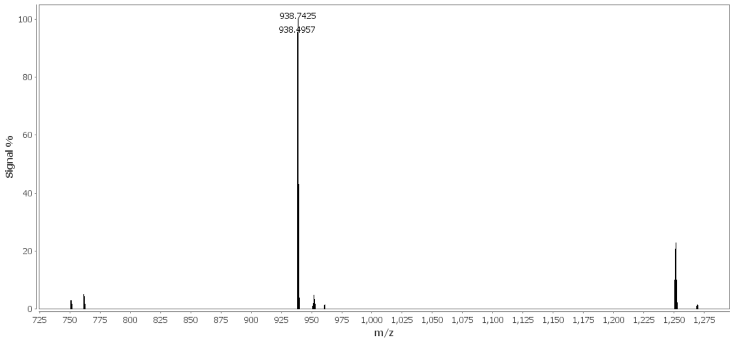

MS2 (+) FT activ = HCD:ce =

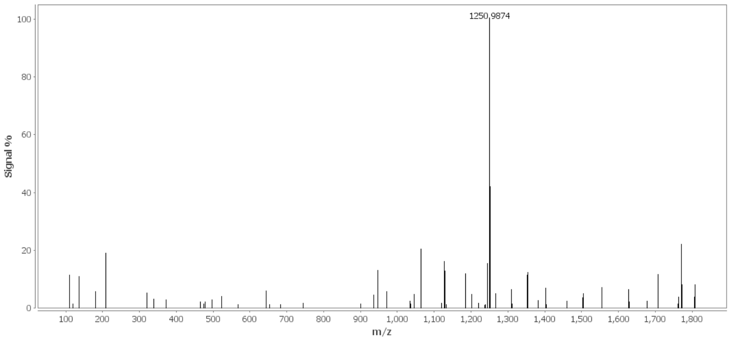

MS2 (+) FT activ = HCD:ce =

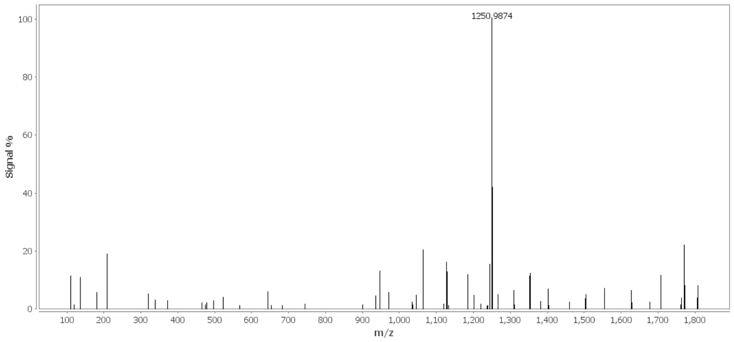

Metabolite: Substrate

Type	score	sub. m/z observed	sub. m/z calculated	sub ppm			met. m/z observed	met. m/z calculated	met. ppm
MATCH	47.4	1554.3420	1554.3424	0.26	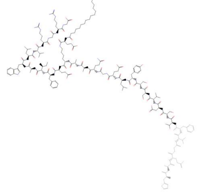	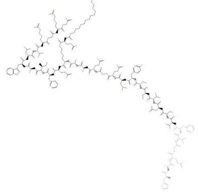	1554.3420	1554.3424	0.26
MATCH	55.3	1503.8175	1503.8186	0.72	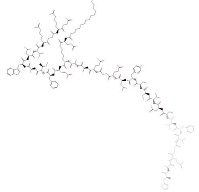	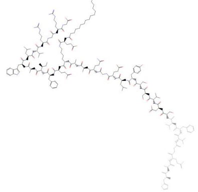	1503.8175	1503.8186	0.72

Metabolite: Substrate

Type	score	sub. m/z observed	sub. m/z calculated	sub ppm			met. m/z observed	met. m/z calculated	met. ppm
MATCH	25.9	1460.2991	1460.3026	2.38	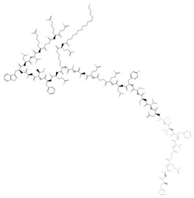	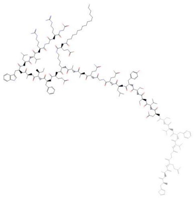	1460.2991	1460.3026	2.38
MATCH	35.7	1402.7850	1402.7891	2.90	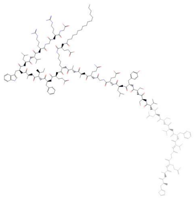	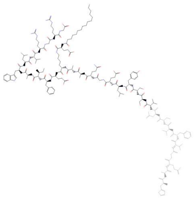	1402.7850	1402.7891	2.90
MISMATCH	4.7	1382.5827	1382.5859	2.33	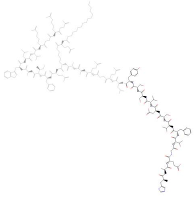	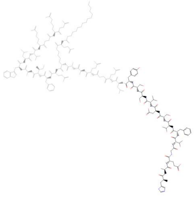	1382.5827	1382.5859	2.33
MATCH	105.2	1353.2522	1353.2549	2.01	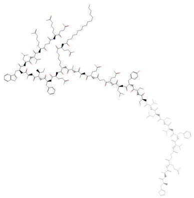	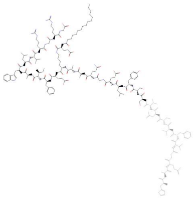	1353.2522	1353.2549	2.01
MATCH	42.1	1309.7364	1309.7389	1.93	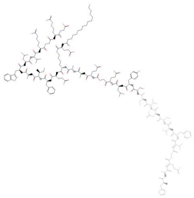	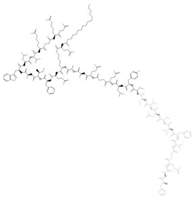	1309.7364	1309.7389	1.93
MATCH	37.5	1266.2207	1266.2229	1.70	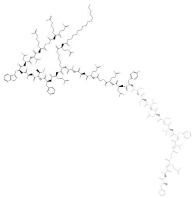	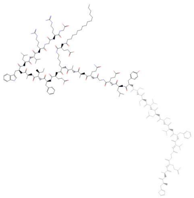	1266.2207	1266.2229	1.70
MATCH	151.1	1250.6556	1250.6561	0.38	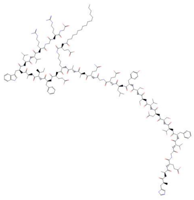	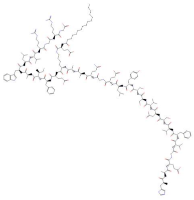	1250.6556	1250.6561	0.38
MATCH	110.1	1250.6532	1250.6561	2.29	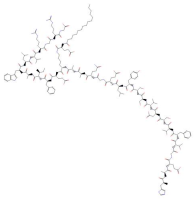	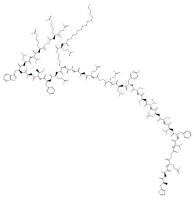	1250.6532	1250.6561	2.29
MATCH	3.1	1219.5188	1219.5226	3.07	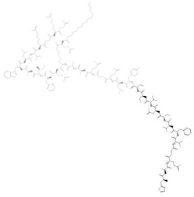	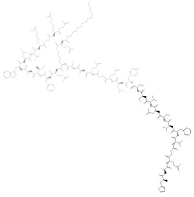	1219.5188	1219.5226	3.07

Metabolite: Substrate

Type	score	sub. m/z observed	sub. m/z calculated	sub ppm			met. m/z observed	met. m/z calculated	met. ppm
MATCH	9.3	1201.5091	1201.5120	2.42	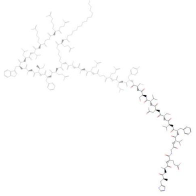	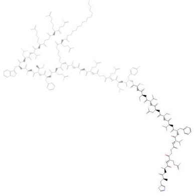	1201.5091	1201.5120	2.42
MATCH	9.3	1201.5091	1201.5120	2.42	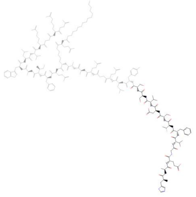	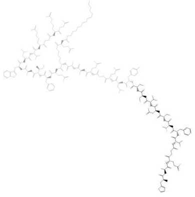	1201.5091	1201.5120	2.42
MATCH	9.3	1201.5091	1201.5120	2.42	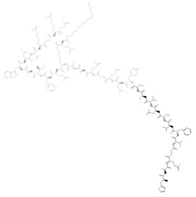	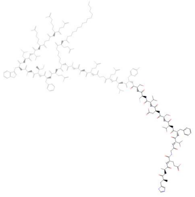	1201.5091	1201.5120	2.42
MATCH	9.3	1201.5091	1201.5120	2.42	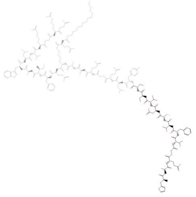	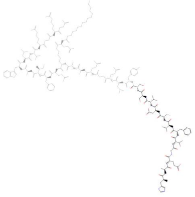	1201.5091	1201.5120	2.42
MATCH	9.3	1201.5091	1201.5120	2.42	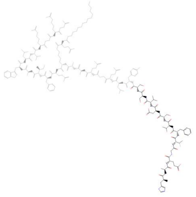	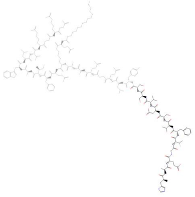	1201.5091	1201.5120	2.42
MATCH	103.4	1184.6888	1184.6912	2.06	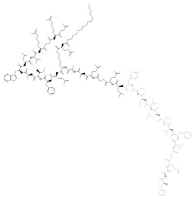	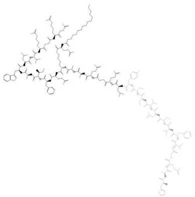	1184.6888	1184.6912	2.06
MATCH	10.9	1132.4870	1132.4905	3.12	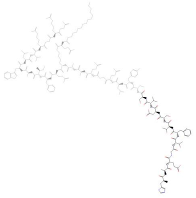	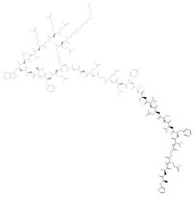	1132.4870	1132.4905	3.12
MATCH	5.0	1128.2393	1128.2320	-6.48	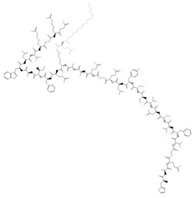	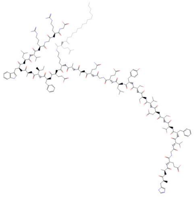	1128.2393	1128.2320	-6.48
MATCH	92.0	1128.1470	1128.1492	1.88	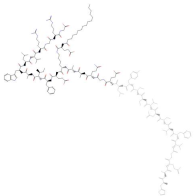	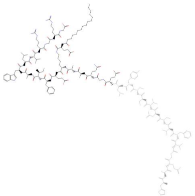	1128.1470	1128.1492	1.88

Metabolite: Substrate

Type	score	sub. m/z observed	sub. m/z calculated	sub ppm			met. m/z observed	met. m/z calculated	met. ppm
MATCH	10.9	1119.1410	1119.1439	2.54	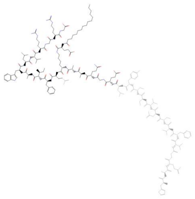	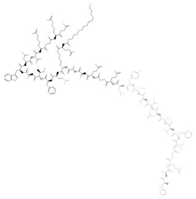	1119.1410	1119.1439	2.54
MATCH	10.9	1119.1410	1119.1439	2.54	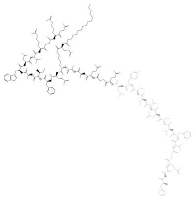	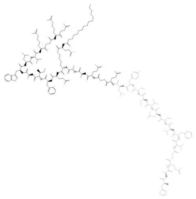	1119.1410	1119.1439	2.54
MATCH	10.9	1119.1410	1119.1439	2.54	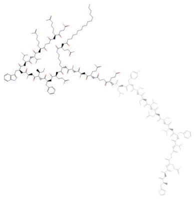	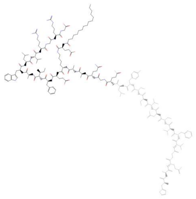	1119.1410	1119.1439	2.54
MATCH	10.9	1119.1410	1119.1439	2.54	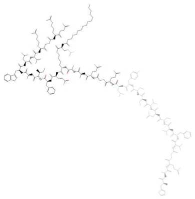	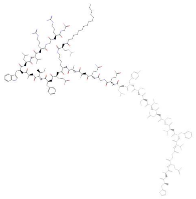	1119.1410	1119.1439	2.54
MATCH	10.9	1119.1410	1119.1439	2.54	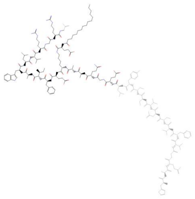	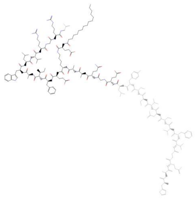	1119.1410	1119.1439	2.54
MATCH	10.9	1119.1410	1119.1439	2.54	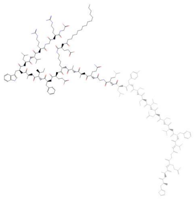	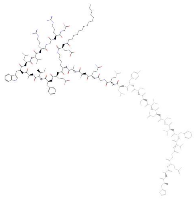	1119.1410	1119.1439	2.54
MATCH	10.9	1119.1410	1119.1439	2.54	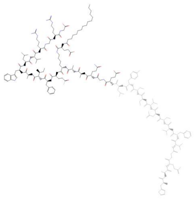	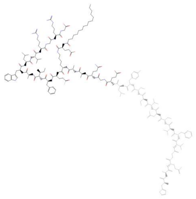	1119.1410	1119.1439	2.54
MATCH	10.9	1119.1410	1119.1439	2.54	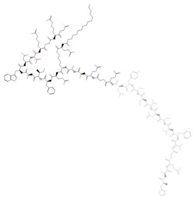	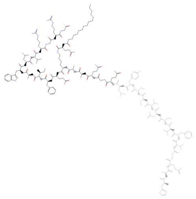	1119.1410	1119.1439	2.54
MATCH	120.5	1063.6253	1063.6279	2.43	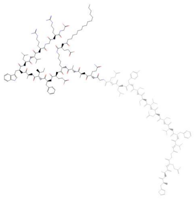	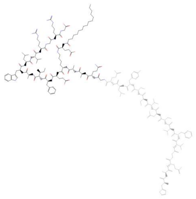	1063.6253	1063.6279	2.43

Metabolite: Substrate

Type	score	sub. m/z observed	sub. m/z calculated	sub ppm			met. m/z observed	met. m/z calculated	met. ppm
MATCH	24.4	1045.4564	1045.4585	1.99	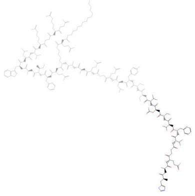	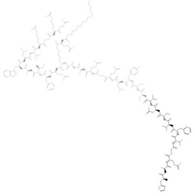	1045.4564	1045.4585	1.99
MATCH	14.3	1035.1141	1035.1171	2.96	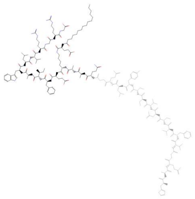	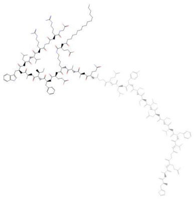	1035.1141	1035.1171	2.96
MATCH	77.9	971.0854	971.0878	2.54	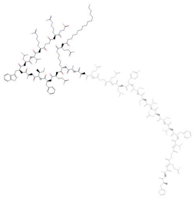	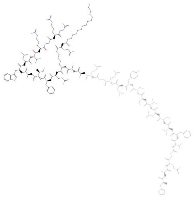	971.0854	971.0878	2.54
MATCH	57.7	946.3881	946.3901	2.12	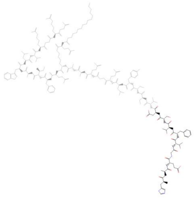	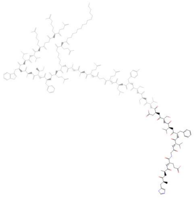	946.3881	946.3901	2.12
MATCH	146.8	938.2475	938.2439	-3.87	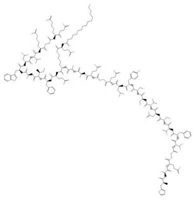	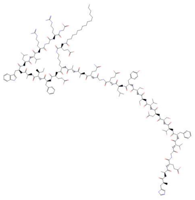	938.2475	938.2439	-3.87
MATCH	50.0	935.5676	935.5693	1.80	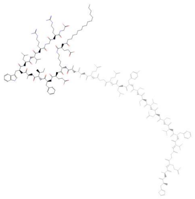	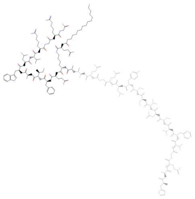	935.5676	935.5693	1.80
MATCH	30.1	900.0483	900.0507	2.75	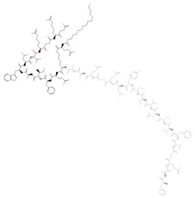	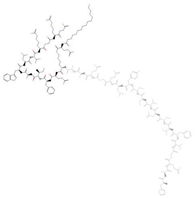	900.0483	900.0507	2.75
MATCH	101.3	750.7942	750.7966	3.21	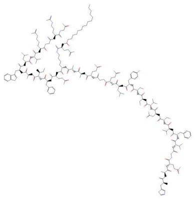	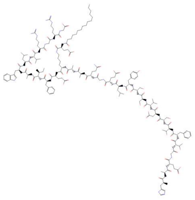	750.7942	750.7966	3.21
MISMATCH	-5.7	682.7895	682.7913	2.67	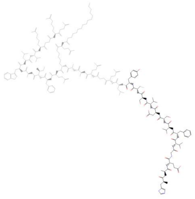	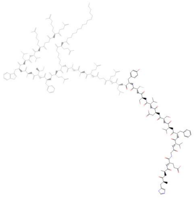	682.7895	682.7913	2.67

Metabolite: Substrate

Type	score	sub. m/z observed	sub. m/z calculated	sub ppm			met. m/z observed	met. m/z calculated	met. ppm
MISMATCH	-5.7	682.7895	682.7913	2.67	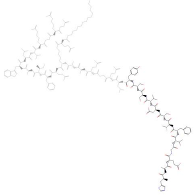	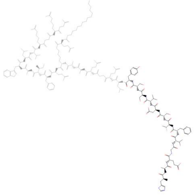	682.7895	682.7913	2.67
MISMATCH	-5.7	682.7895	682.7913	2.67	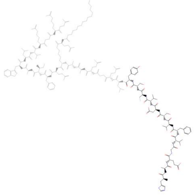	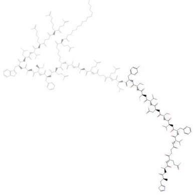	682.7895	682.7913	2.67
MISMATCH	-5.7	682.7895	682.7913	2.67	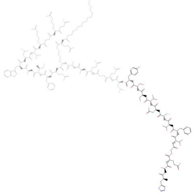	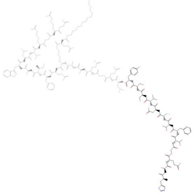	682.7895	682.7913	2.67
MATCH	101.3	652.3655	652.3671	2.43	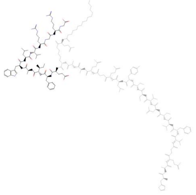	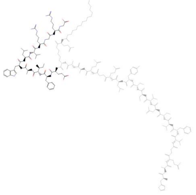	652.3655	652.3671	2.43
MISMATCH	-25.5	643.2822	643.2835	2.01	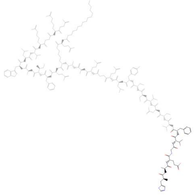	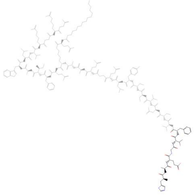	643.2822	643.2835	2.01
MATCH	14.1	496.2139	496.2150	2.32	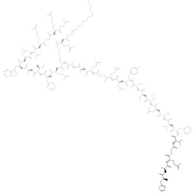	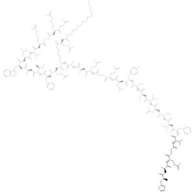	496.2139	496.2150	2.32
MATCH	14.0	478.2032	478.2045	2.68	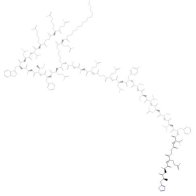	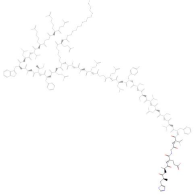	478.2032	478.2045	2.68
MISMATCH	-9.1	464.6927	464.6934	1.54	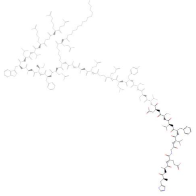	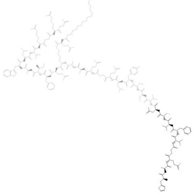	464.6927	464.6934	1.54
MISMATCH	-9.1	464.6927	464.6934	1.54	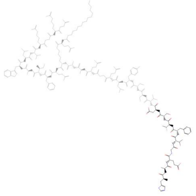	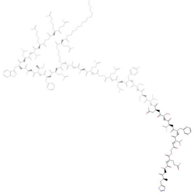	464.6927	464.6934	1.54

Metabolite: Substrate

Type	score	sub. m/z observed	sub. m/z calculated	sub ppm			met. m/z observed	met. m/z calculated	met. ppm
MISMATCH	-9.1	464.6927	464.6934	1.54	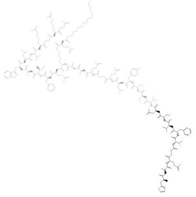	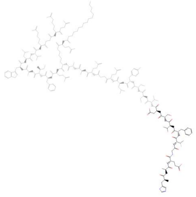	464.6927	464.6934	1.54
MATCH	52.5	136.0765	136.0706	-43.3	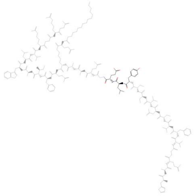	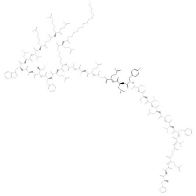	136.0765	136.0706	-43.3
MATCH	52.5	136.0765	136.0693	-53.2	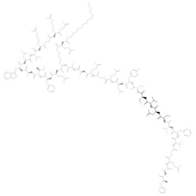	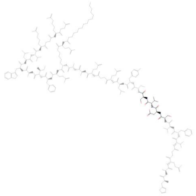	136.0765	136.0693	-53.2
MATCH	52.5	136.0765	136.0693	-53.2	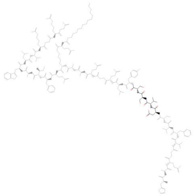	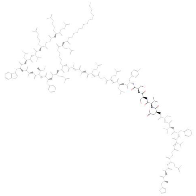	136.0765	136.0693	-53.2
MATCH	52.5	136.0765	136.0737	-20.7	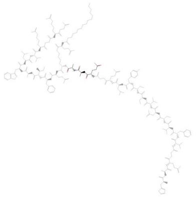	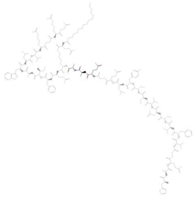	136.0765	136.0737	-20.7
MATCH	52.5	136.0765	136.0737	-20.7	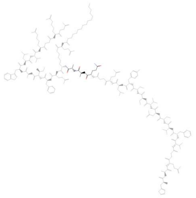	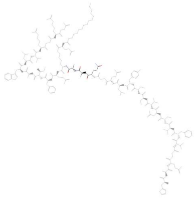	136.0765	136.0737	-20.7
MATCH	52.5	136.0765	136.0706	-43.3	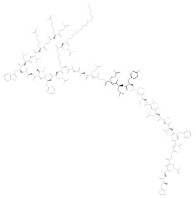	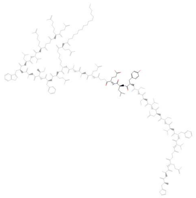	136.0765	136.0706	-43.3
MISMATCH	38.8	120.0807	120.0788	-16.4	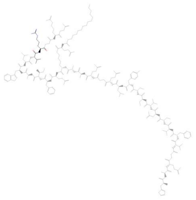	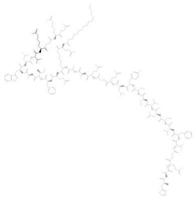	120.0807	120.0788	-16.4

MS (+) FT

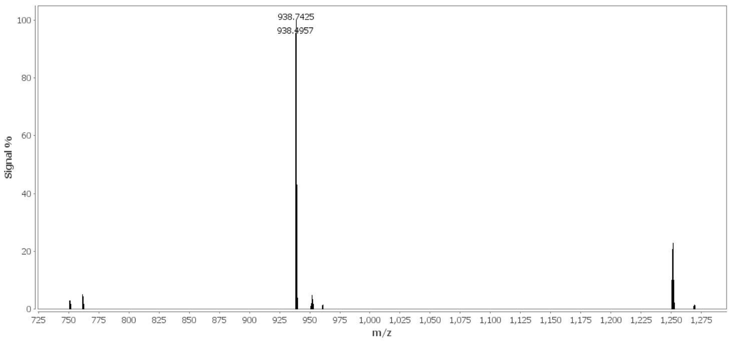

MS (+) FT

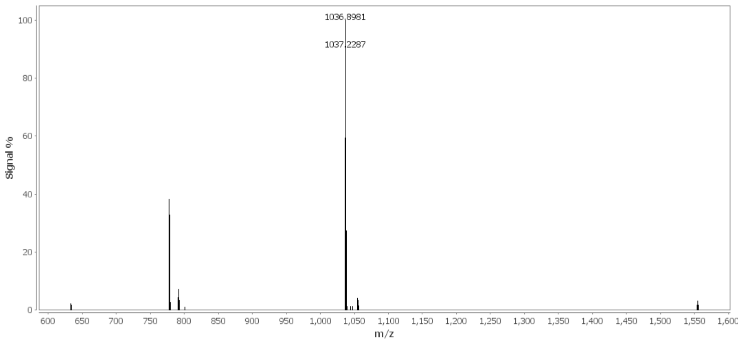

MS2 (+) FT activ = HCD:ce =

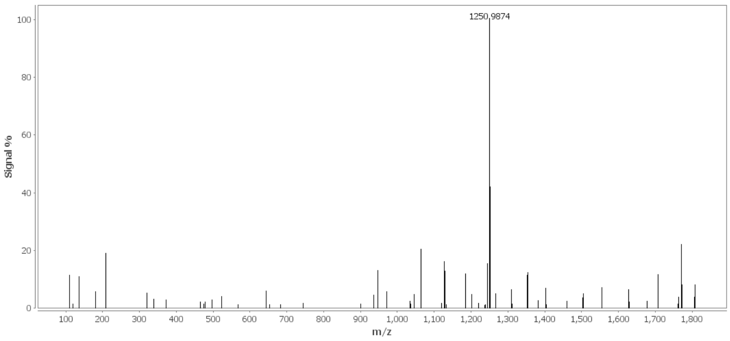

MS2 (+) FT activ = HCD:ce =

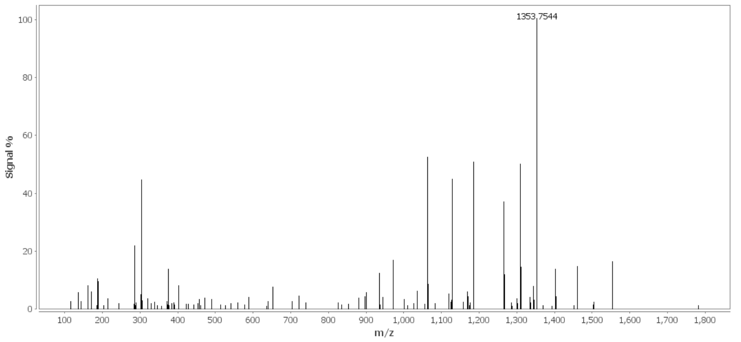

Metabolite: M4 -642 RT=8.93

Type	score	sub. m/z observed	sub. m/z calculated	sub ppm		met. m/z observed	met. m/z calculated	met. ppm
MATCH	23.4	750.7942	750.7966	3.21	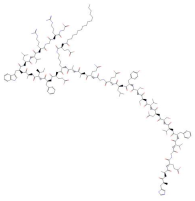 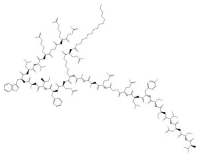	777.6761	777.6748	-1.57
MATCH	23.4	750.7942	750.7966	3.21	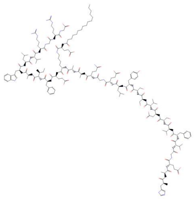 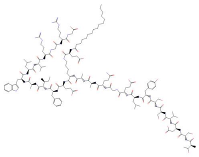	777.6761	777.6748	-1.57
MATCH	23.4	750.7942	750.7966	3.21	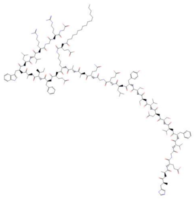 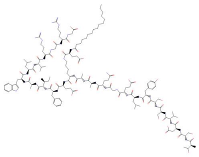	777.6761	777.6748	-1.57
MATCH	60.6	750.7942	750.7966	3.21	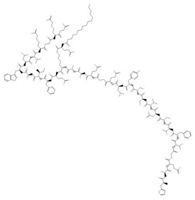 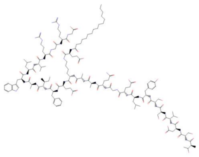	1036.5666	1036.5640	-2.52
MATCH	60.6	750.7942	750.7966	3.21	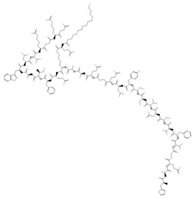 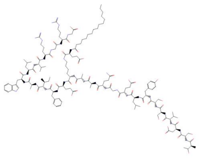	1036.5666	1036.5640	-2.52

Metabolite: M4 -642 RT=8.93

Type	score	sub. m/z observed	sub. m/z calculated	sub ppm		met. m/z observed	met. m/z calculated	met. ppm
MATCH	60.6	750.7942	750.7966	3.21	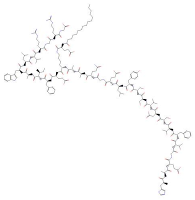 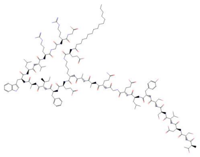	1036.5666	1036.5640	-2.52
MATCH	2.9	750.7942	750.7966	3.21	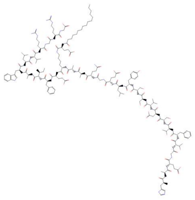 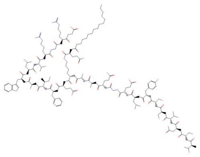	1554.3389	1554.3424	2.25
MATCH	2.9	750.7942	750.7966	3.21	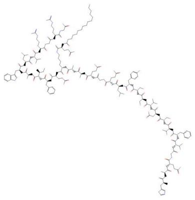 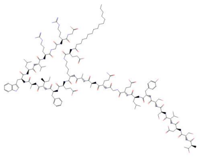	1554.3389	1554.3424	2.25
MATCH	69.0	938.2475	938.2439	-3.87	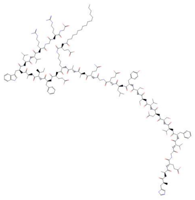 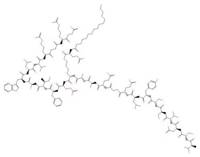	777.6761	777.6748	-1.57
MATCH	69.0	938.2475	938.2439	-3.87	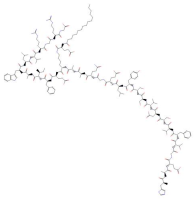 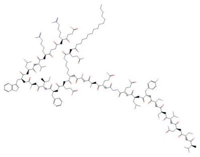	777.6761	777.6748	-1.57
					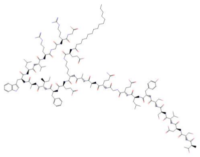	777.6761	777.6748	-1.57
MATCH	106.1	938.2475	938.2439	-3.87	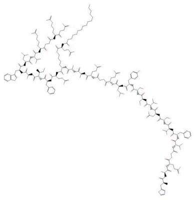 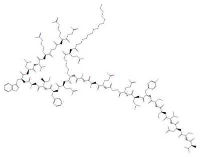	1036.5666	1036.5640	-2.52
MATCH	106.1	938.2475	938.2439	-3.87	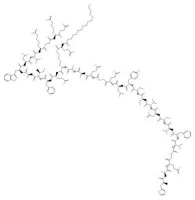 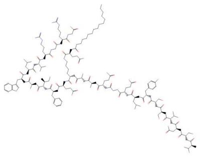	1036.5666	1036.5640	-2.52
					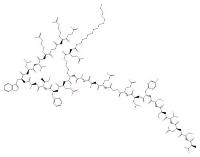	1036.5666	1036.5640	-2.52

Metabolite: M4 -642 RT=8.93

Type	score	sub. m/z observed	sub. m/z calculated	sub ppm		met. m/z observed	met. m/z calculated	met. ppm
MATCH	32.2	1250.6532	1250.6561	2.29	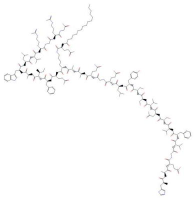 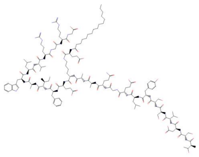	777.6761	777.6748	-1.57
MATCH	32.2	1250.6532	1250.6561	2.29	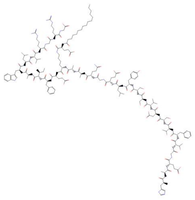 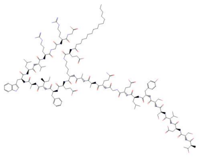	777.6761	777.6748	-1.57
MATCH	32.2	1250.6532	1250.6561	2.29	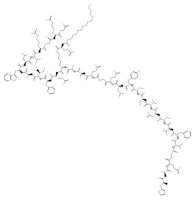 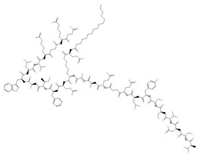	777.6761	777.6748	-1.57
MATCH	69.4	1250.6532	1250.6561	2.29	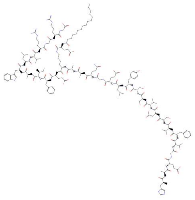 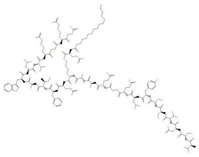	1036.5666	1036.5640	-2.52
MATCH	69.4	1250.6532	1250.6561	2.29	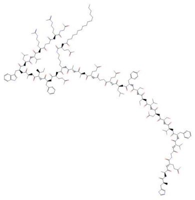 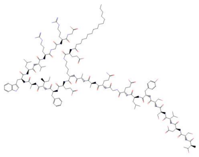	1036.5666	1036.5640	-2.52
MATCH	69.4	1250.6532	1250.6561	2.29	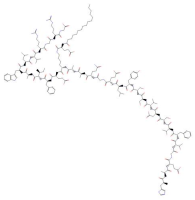 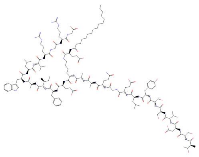	1036.5666	1036.5640	-2.52
MATCH	11.7	1250.6532	1250.6561	2.29	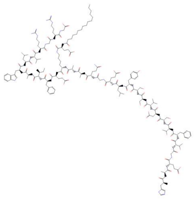 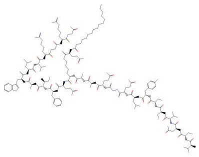	1554.3389	1554.3424	2.25
MATCH	11.7	1250.6532	1250.6561	2.29	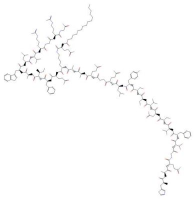 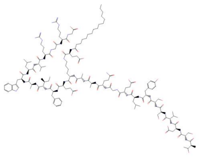	1554.3389	1554.3424	2.25
MATCH	16.6	136.0765	136.0693	-53.2	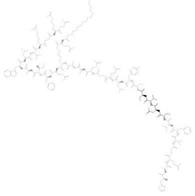 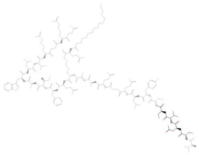	136.0756	136.0693	-46.2

Metabolite: M4 -642 RT=8.93

Type	score	sub. m/z observed	sub. m/z calculated	sub ppm			met. m/z observed	met. m/z calculated	met. ppm
MATCH	16.6	136.0765	136.0693	-53.2	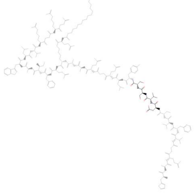	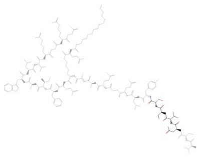	136.0756	136.0693	-46.2
MATCH	16.6	136.0765	136.0706	-43.3	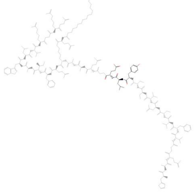	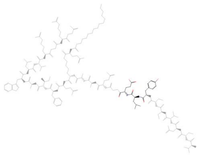	136.0756	136.0706	-36.3
MATCH	16.6	136.0765	136.0706	-43.3	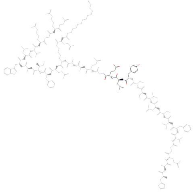	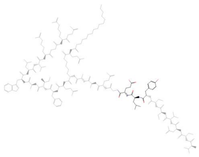	136.0756	136.0706	-36.3
MATCH	16.6	136.0765	136.0737	-20.7	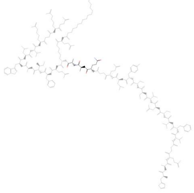	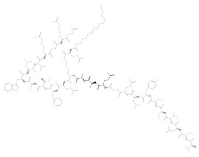	136.0756	136.0737	-13.7
MATCH	16.6	136.0765	136.0737	-20.7	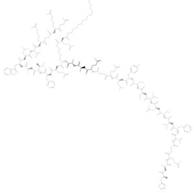	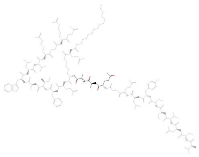	136.0756	136.0737	-13.7
MATCH	8.9	652.3655	652.3671	2.43	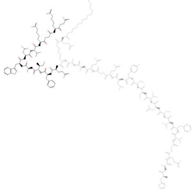	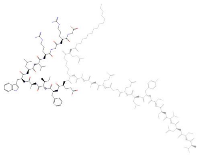	652.3655	652.3671	2.41
MATCH	7.3	900.0483	900.0507	2.75	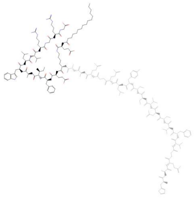	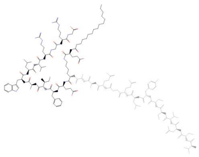	900.0488	900.0507	2.16
MATCH	17.1	935.5676	935.5693	1.80	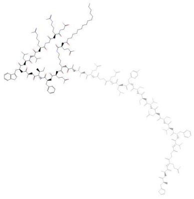	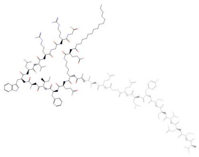	935.5692	935.5693	0.15
MATCH	57.7	946.3881	946.3901	2.12	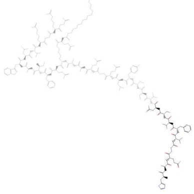	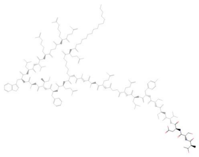	304.1134	304.1139	1.78

Metabolite: M4 -642 RT=8.93

Type	score	sub. m/z observed	sub. m/z calculated	sub ppm		met. m/z observed	met. m/z calculated	met. ppm
MATCH	22.5	971.0854	971.0878	2.54	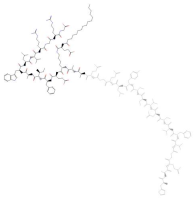 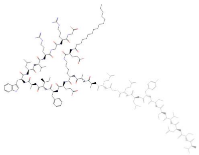	971.0861	971.0878	1.76
MATCH	7.3	1035.1141	1035.1171	2.96	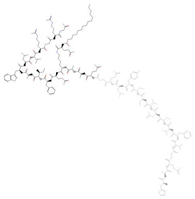 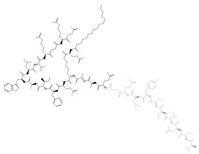	1035.1159	1035.1171	1.21
MATCH	12.9	1045.4564	1045.4585	1.99	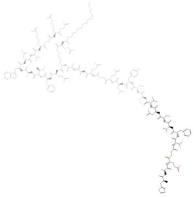 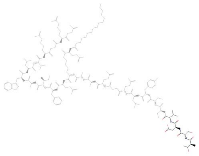	403.1817	403.1823	1.71
MATCH	72.9	1063.6253	1063.6279	2.43	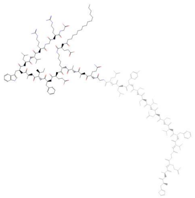 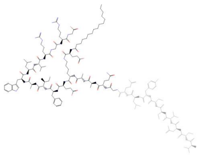	1063.6261	1063.6279	1.65
MATCH	6.2	1119.1410	1119.1439	2.54	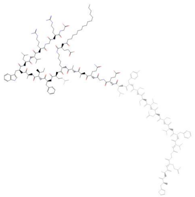 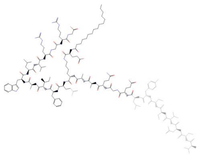	1119.1459	1119.1439	-1.80
MATCH	6.2	1119.1410	1119.1439	2.54	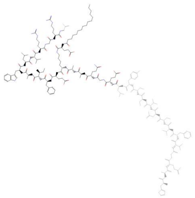 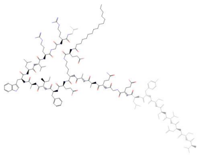	1119.1459	1119.1439	-1.80
MATCH	6.2	1119.1410	1119.1439	2.54	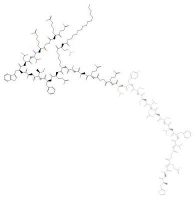 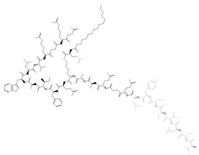	1119.1459	1119.1439	-1.80
MATCH	6.2	1119.1410	1119.1439	2.54	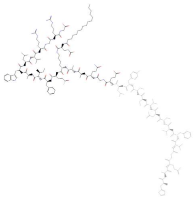 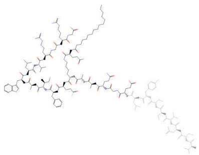	1119.1459	1119.1439	-1.80
MATCH	6.2	1119.1410	1119.1439	2.54	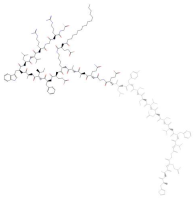 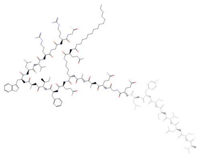	1119.1459	1119.1439	-1.80

Metabolite: M4 -642 RT=8.93

Type	score	sub. m/z observed	sub. m/z calculated	sub ppm			met. m/z observed	met. m/z calculated	met. ppm
MATCH	6.2	1119.1410	1119.1439	2.54	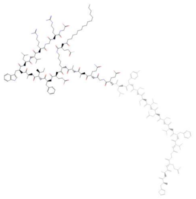	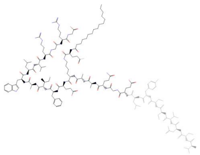	1119.1459	1119.1439	-1.80
MATCH	6.2	1119.1410	1119.1439	2.54	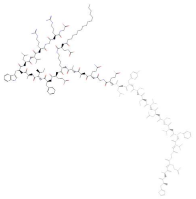	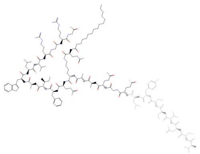	1119.1459	1119.1439	-1.80
MATCH	6.2	1119.1410	1119.1439	2.54	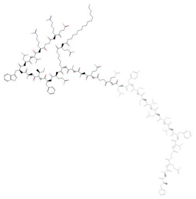	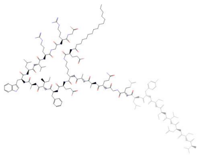	1119.1459	1119.1439	-1.80
MATCH	59.3	1128.1470	1128.1492	1.88	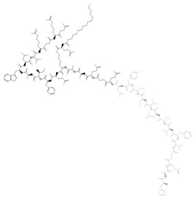	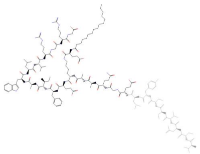	1128.1477	1128.1492	1.33
MATCH	4.7	1132.4870	1132.4905	3.12	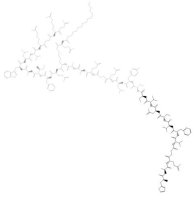	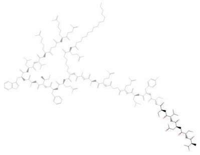	490.2136	490.2144	1.62
MATCH	59.5	1184.6888	1184.6912	2.06	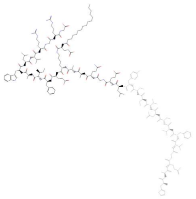	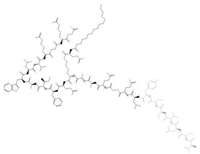	1184.6888	1184.6912	1.99
MATCH	7.1	1201.5091	1201.5120	2.42	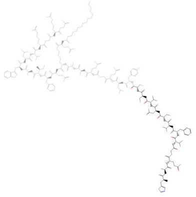	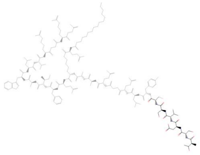	559.2337	559.2358	3.80
MATCH	7.1	1201.5091	1201.5120	2.42	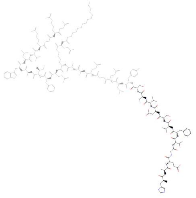	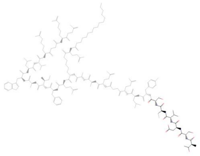	559.2337	559.2358	3.80
MATCH	7.1	1201.5091	1201.5120	2.42	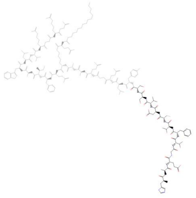	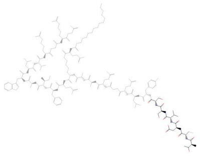	559.2337	559.2358	3.80

Metabolite: M4 -642 RT=8.93

Type	score	sub. m/z observed	sub. m/z calculated	sub ppm			met. m/z observed	met. m/z calculated	met. ppm
MATCH	7.1	1201.5091	1201.5120	2.42	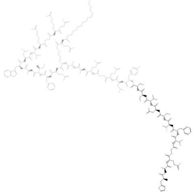	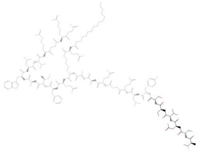	559.2337	559.2358	3.80
MATCH	3.1	1219.5188	1219.5226	3.07	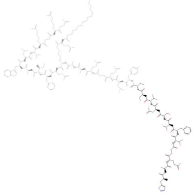	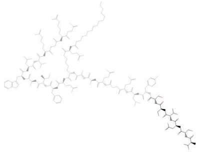	577.2472	577.2464	-1.39
MATCH	67.5	1250.6556	1250.6561	0.38	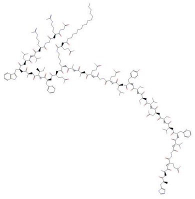	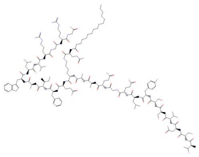	1554.3410	1554.3424	0.88
MATCH	33.3	1266.2207	1266.2229	1.70	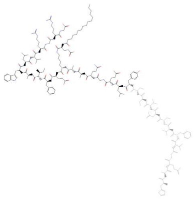	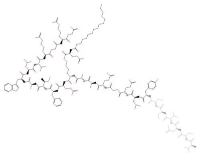	1266.2216	1266.2229	0.98
MATCH	42.1	1309.7364	1309.7389	1.93	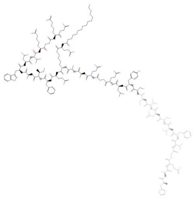	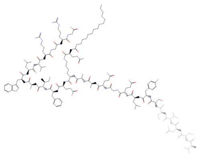	1309.7380	1309.7389	0.67
MATCH	85.2	1353.2522	1353.2549	2.01	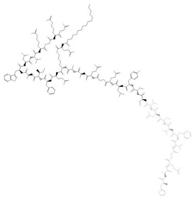	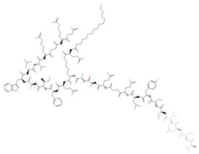	1353.2535	1353.2549	1.02
MATCH	4.7	1382.5827	1382.5859	2.33	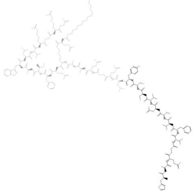	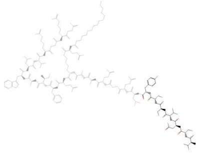	740.3076	740.3097	2.87
MATCH	15.3	1402.7850	1402.7891	2.90	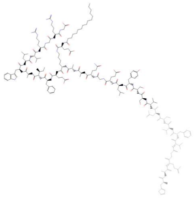	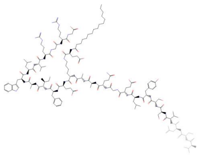	1402.7882	1402.7891	0.63
MATCH	11.2	1460.2991	1460.3026	2.38	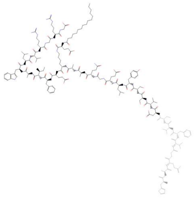	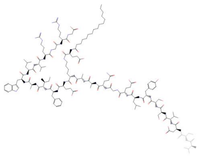	1460.3006	1460.3026	1.36

Metabolite: M4 -642 RT=8.93

Type	score	sub. m/z observed	sub. m/z calculated	sub ppm			met. m/z observed	met. m/z calculated	met. ppm
MATCH	5.1	1503.8175	1503.8186	0.72	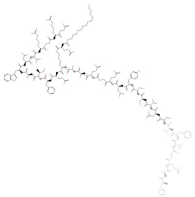	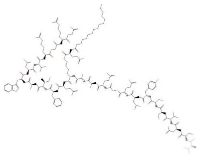	1503.8150	1503.8186	2.39
MISMATCH	-24.1	464.6927	464.6934	1.54	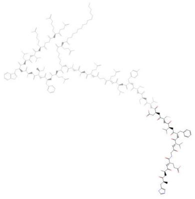		286.1029	286.1029	0.00
MISMATCH	-5.7	682.7895	682.7913	2.67	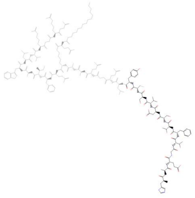		722.2973	722.2973	0.00
MET_MATCH						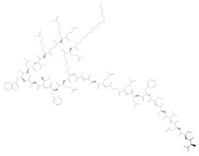	161.0918	161.0921	1.48
MET_MATCH						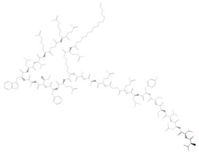	189.0867	189.0870	1.35
MET_MATCH						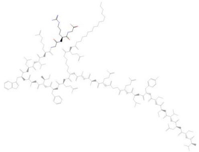	289.1611	289.1619	2.85
MET_MATCH						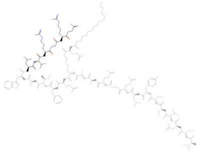	329.2110	329.2114	1.20
MET_MATCH						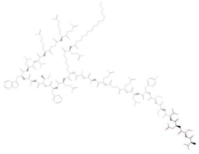	375.1868	375.1874	1.80
MET_MATCH						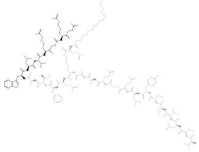	422.2496	422.2510	3.45

Metabolite: M4 -642 RT=8.93

Type	score	sub. m/z observed	sub. m/z calculated	sub ppm		met. m/z observed	met. m/z calculated	met. ppm
MET_MATCH					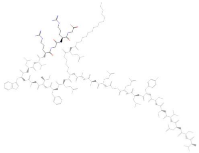	428.2368	428.2364	-0.94
MET_MATCH					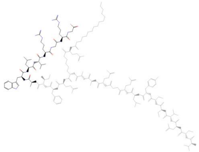	457.7692	457.7696	0.93
MET_MATCH					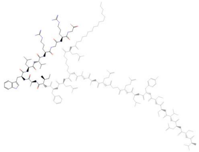	514.3110	514.3116	1.22
MET_MATCH					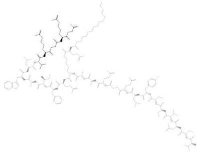	527.3061	527.3049	-2.34
MET_MATCH					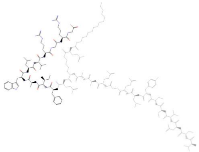	587.8454	587.8458	0.78
MET_MATCH					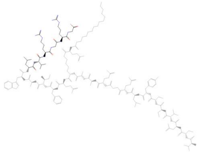	640.3870	640.3889	2.96
MET_MATCH					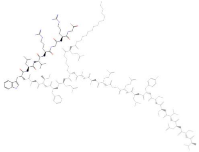	826.4663	826.4682	2.35
MET_MATCH					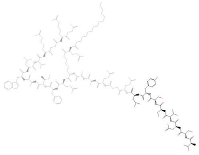	853.3899	853.3938	4.53
MET_MATCH					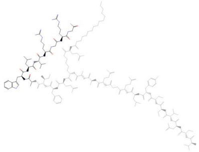	897.5022	897.5053	3.48

Metabolite: M4 -642 RT=8.93

Type	score	sub. m/z observed	sub. m/z calculated	sub ppm		met. m/z observed	met. m/z calculated	met. ppm
MET_MATCH					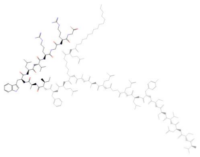	1010.5868	1010.5894	2.54
MET_MATCH					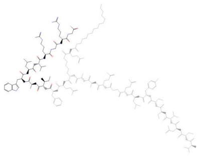	1055.6154	1055.6109	-4.30
MET_MATCH					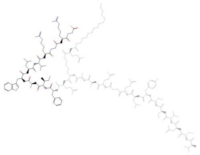	1157.6564	1157.6578	1.20
MET_MATCH					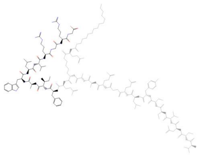	1174.6882	1174.6844	-3.29
MET_MATCH					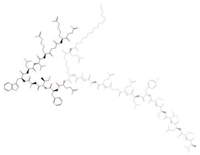	1286.6983	1286.7004	1.61
MET_MATCH					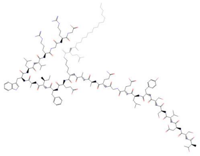	1370.7051	1370.7063	0.88
MET_MATCH					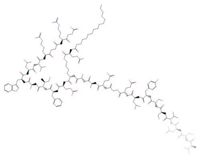	1394.2811	1394.2758	-3.81
MET_MATCH					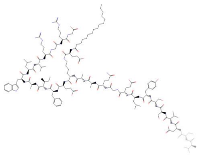	1451.7949	1451.7893	-3.86
MET_MATCH					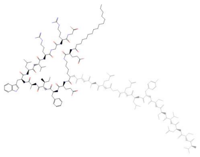	1782.0609	1782.0676	3.77

MS (+) FT

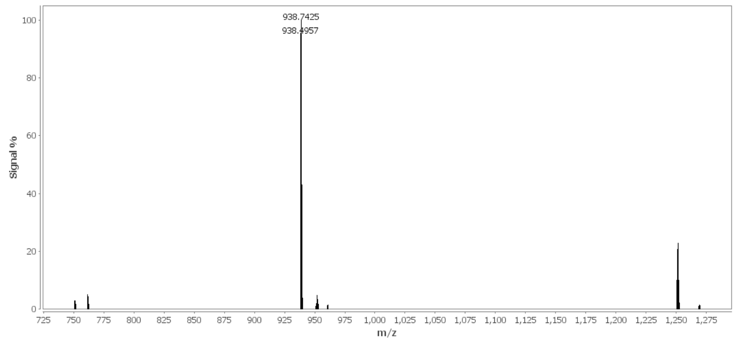

MS (+) FT

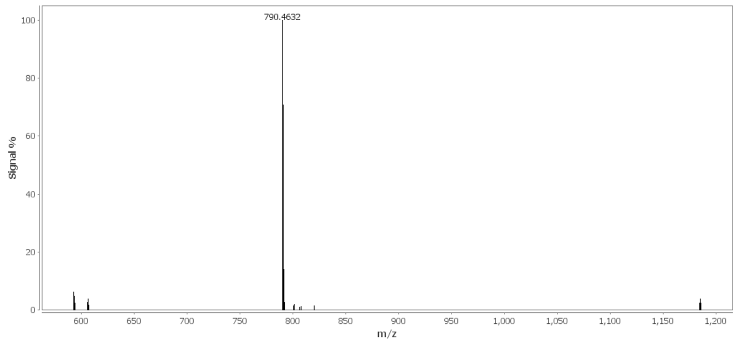

MS2 (+) FT activ = HCD:ce =

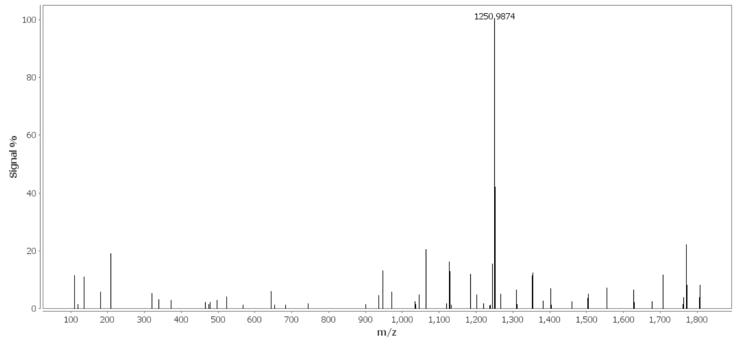

MS2 (+) FT activ = HCD:ce =

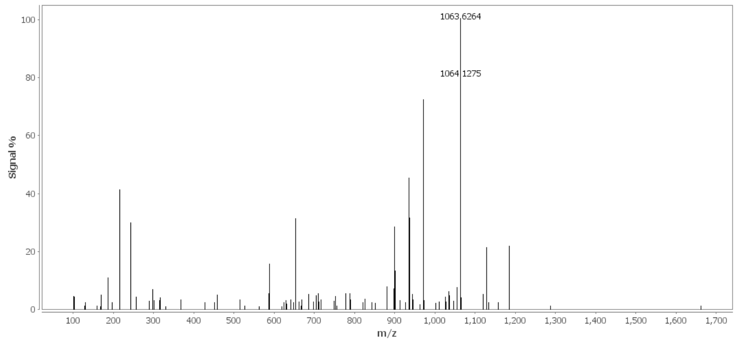

Metabolite: M3 -1382 RT=8.59

Type	score	sub. m/z observed	sub. m/z calculated	sub ppm		met. m/z observed	met. m/z calculated	met. ppm
MATCH	5.9	750.7942	750.7966	3.21	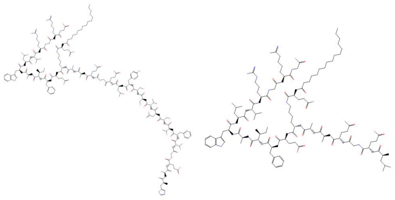	592.8485	592.8492	1.25
MATCH	5.9	750.7942	750.7966	3.21	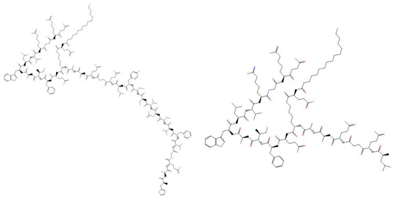	592.8485	592.8492	1.25
MATCH	5.9	750.7942	750.7966	3.21	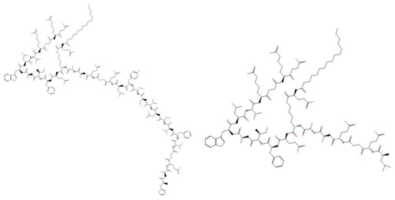	592.8485	592.8492	1.25
MATCH	75.4	750.7942	750.7966	3.21	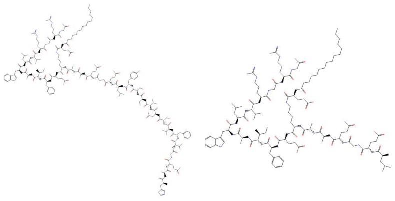	790.1301	790.1299	-0.26
MATCH	75.4	750.7942	750.7966	3.21	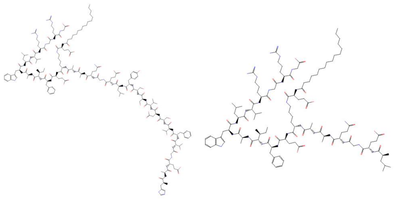	790.1301	790.1299	-0.26

Metabolite: M3 -1382 RT=8.59

Type	score	sub. m/z observed	sub. m/z calculated	sub ppm		met. m/z observed	met. m/z calculated	met. ppm
MATCH	75.4	750.7942	750.7966	3.21	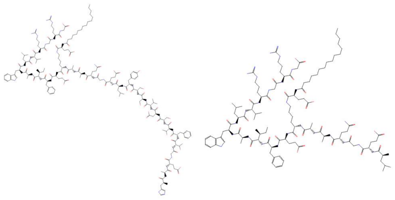	790.1301	790.1299	-0.26
MATCH	3.7	750.7942	750.7966	3.21	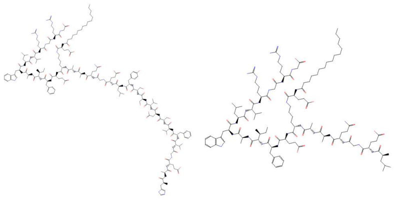	1184.6894	1184.6912	1.51
MATCH	3.7	750.7942	750.7966	3.21	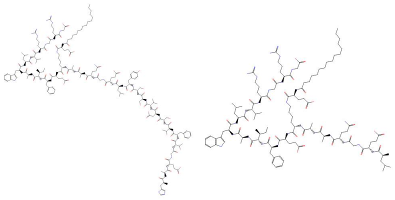	1184.6894	1184.6912	1.51
MATCH	3.7	750.7942	750.7966	3.21	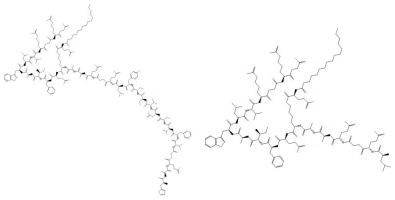	1184.6894	1184.6912	1.51
MATCH	51.4	938.2475	938.2439	-3.87	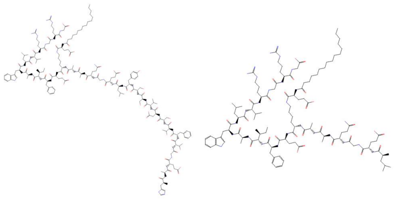	592.8485	592.8492	1.25
MATCH	51.4	938.2475	938.2439	-3.87	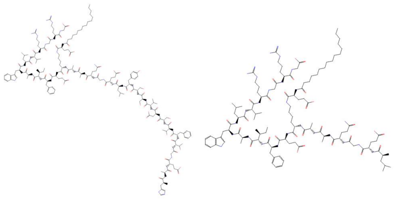	592.8485	592.8492	1.25
MATCH	51.4	938.2475	938.2439	-3.87	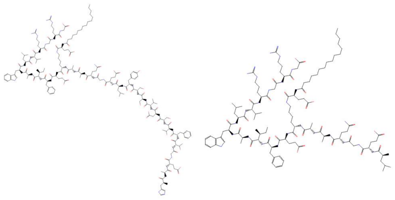	592.8485	592.8492	1.25
MATCH	120.9	938.2475	938.2439	-3.87	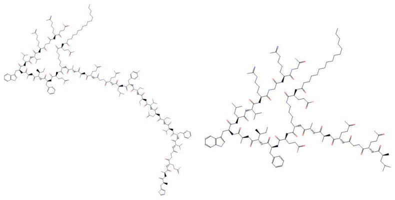	790.1301	790.1299	-0.26
MATCH	120.9	938.2475	938.2439	-3.87	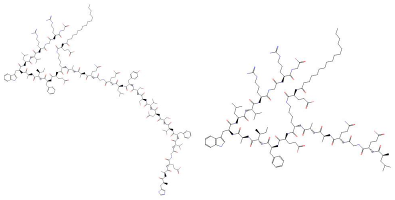	790.1301	790.1299	-0.26

Metabolite: M3 -1382 RT=8.59

Type	score	sub. m/z observed	sub. m/z calculated	sub ppm		met. m/z observed	met. m/z calculated	met. ppm
MATCH	120.9	938.2475	938.2439	-3.87	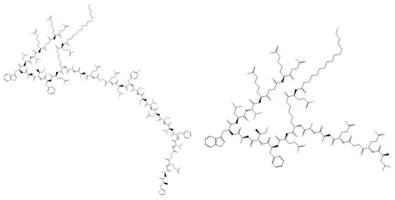	790.1301	790.1299	-0.26
MATCH	49.2	938.2475	938.2439	-3.87	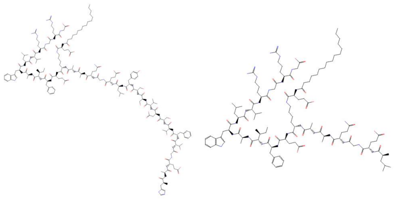	1184.6894	1184.6912	1.51
MATCH	49.2	938.2475	938.2439	-3.87	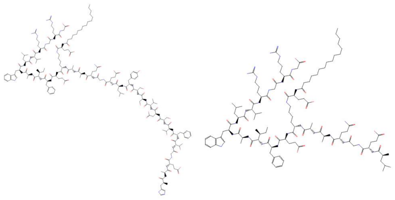	1184.6894	1184.6912	1.51
MATCH	49.2	938.2475	938.2439	-3.87	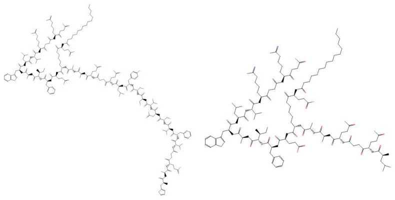	1184.6894	1184.6912	1.51
MATCH	14.7	1250.6532	1250.6561	2.29	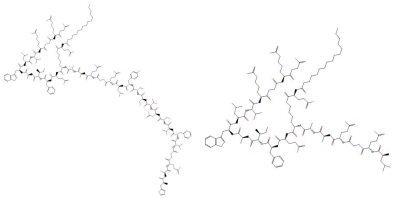	592.8485	592.8492	1.25
MATCH	14.7	1250.6532	1250.6561	2.29	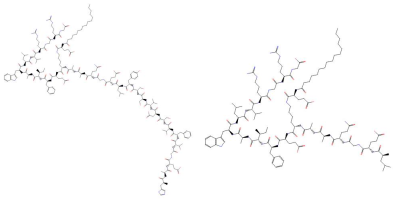	592.8485	592.8492	1.25
MATCH	14.7	1250.6532	1250.6561	2.29	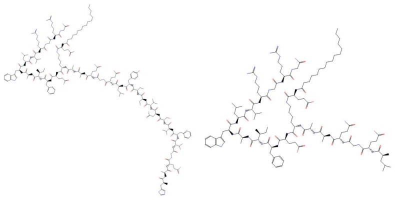	592.8485	592.8492	1.25
MATCH	84.2	1250.6532	1250.6561	2.29	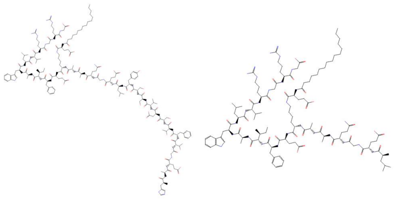	790.1301	790.1299	-0.26
MATCH	84.2	1250.6532	1250.6561	2.29	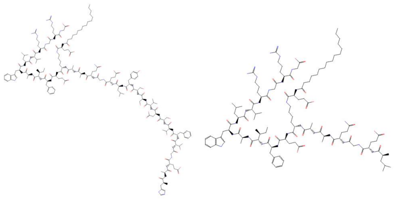	790.1301	790.1299	-0.26

Metabolite: M3 -1382 RT=8.59

Type	score	sub. m/z observed	sub. m/z calculated	sub ppm		met. m/z observed	met. m/z calculated	met. ppm
MATCH	84.2	1250.6532	1250.6561	2.29	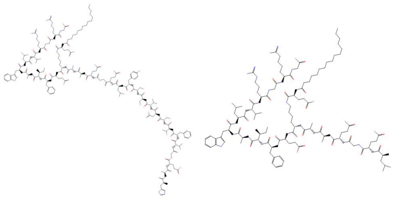	790.1301	790.1299	-0.26
MATCH	12.5	1250.6532	1250.6561	2.29	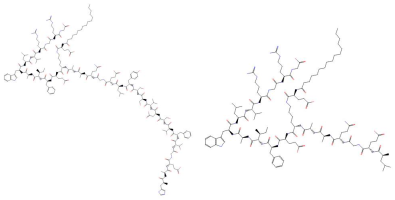	1184.6894	1184.6912	1.51
MATCH	12.5	1250.6532	1250.6561	2.29	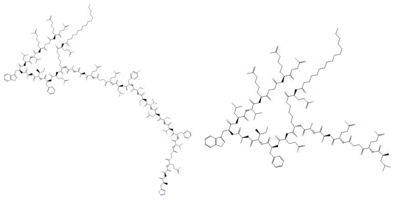	1184.6894	1184.6912	1.51
MATCH	12.5	1250.6532	1250.6561	2.29	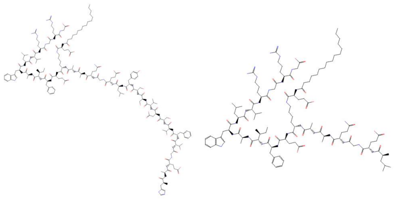	1184.6894	1184.6912	1.51
MATCH	32.7	652.3655	652.3671	2.43	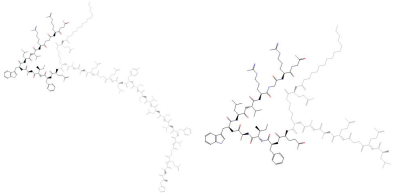	652.3664	652.3671	1.08
MATCH	30.1	900.0483	900.0507	2.75	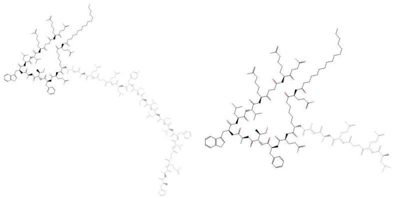	900.0495	900.0507	1.33
MATCH	50.0	935.5676	935.5693	1.80	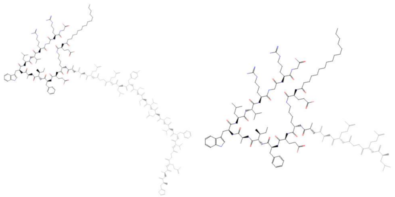	935.5691	935.5693	0.22
MATCH	77.9	971.0854	971.0878	2.54	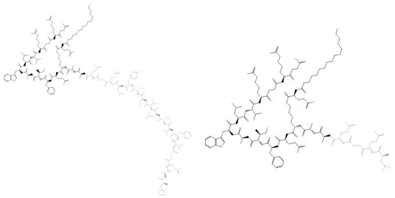	971.0866	971.0878	1.27
MATCH	8.7	1035.1141	1035.1171	2.96	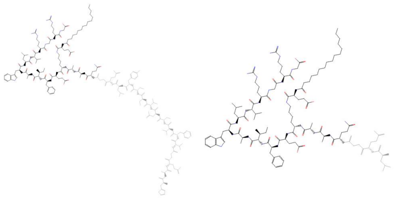	1035.1140	1035.1171	3.02

Metabolite: M3 -1382 RT=8.59

Type	score	sub. m/z observed	sub. m/z calculated	sub ppm			met. m/z observed	met. m/z calculated	met. ppm
MATCH	120.5	1063.6253	1063.6279	2.43	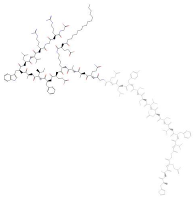	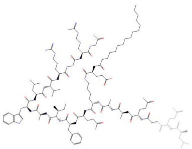	1063.6264	1063.6279	1.39
MATCH	5.3	1119.1410	1119.1439	2.54	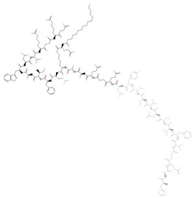	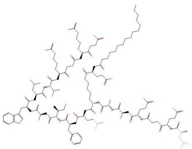	1119.1427	1119.1439	1.06
MATCH	5.3	1119.1410	1119.1439	2.54	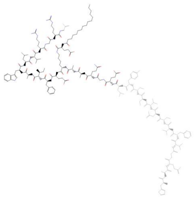	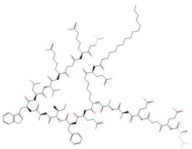	1119.1427	1119.1439	1.06
MATCH	5.3	1119.1410	1119.1439	2.54	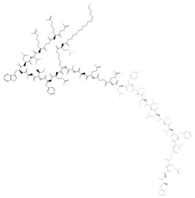	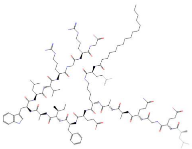	1119.1427	1119.1439	1.06
MATCH	5.3	1119.1410	1119.1439	2.54	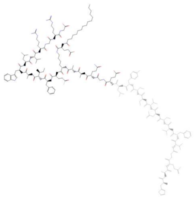	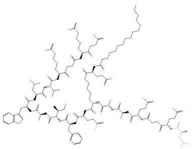	1119.1427	1119.1439	1.06
MATCH	5.3	1119.1410	1119.1439	2.54	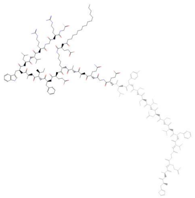	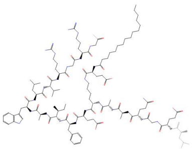	1119.1427	1119.1439	1.06
MATCH	5.3	1119.1410	1119.1439	2.54	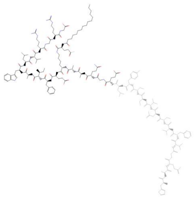	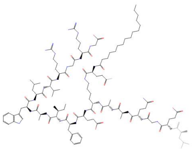	1119.1427	1119.1439	1.06
MATCH	5.3	1119.1410	1119.1439	2.54	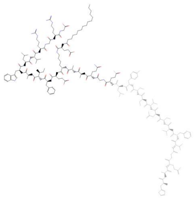	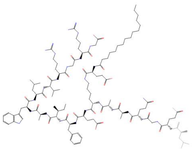	1119.1427	1119.1439	1.06
MATCH	5.3	1119.1410	1119.1439	2.54	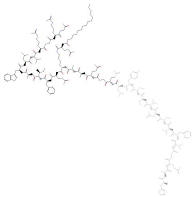	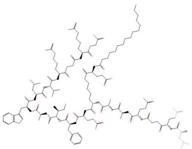	1119.1427	1119.1439	1.06

Metabolite: M3 -1382 RT=8.59

Type	score	sub. m/z observed	sub. m/z calculated	sub ppm		met. m/z observed	met. m/z calculated	met. ppm
MATCH	37.5	1128.1470	1128.1492	1.88	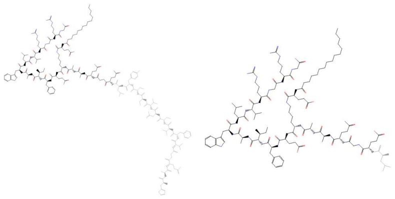	1128.1464	1128.1492	2.46
MATCH	5.0	1128.2393	1128.2320	-6.48	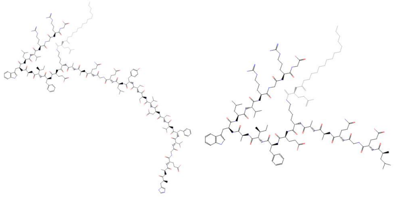	667.7039	667.7058	2.91
MATCH	54.6	1250.6556	1250.6561	0.38	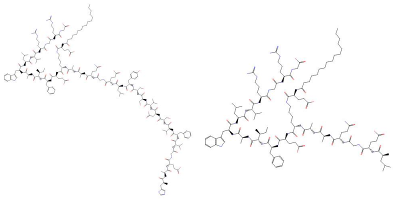	790.1277	790.1299	2.71
MATCH	73.0	1250.6556	1250.6561	0.38	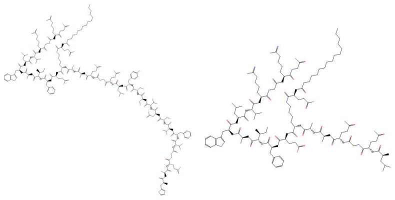	1184.6898	1184.6912	1.21
MISMATCH	-8.1	971.0854	971.0878	2.54	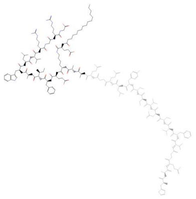	647.7271	647.7271	0.00
MISMATCH	-26.0	1063.6253	1063.6279	2.43	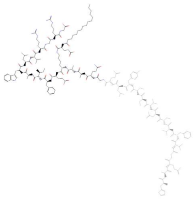	709.4205	709.4205	0.00
MET_MATCH					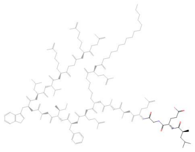	159.0912	159.0946	21.33
MET_MATCH					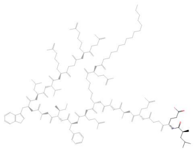	215.1389	215.1390	0.58
MET_MATCH					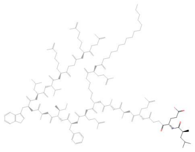	243.1337	243.1339	1.06

Metabolite: M3 -1382 RT=8.59

Type	score	sub. m/z observed	sub. m/z calculated	sub ppm		met. m/z observed	met. m/z calculated	met. ppm
MET_MATCH					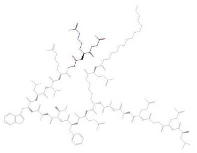	289.1613	289.1619	2.16
MET_MATCH					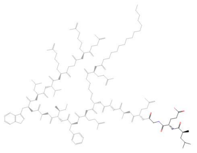	300.1545	300.1554	2.96
MET_MATCH					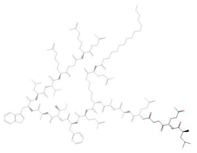	317.1823	317.1819	-1.15
MET_MATCH					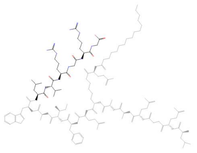	329.2110	329.2114	1.04
MET_MATCH					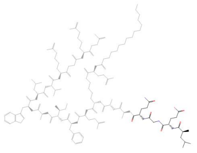	428.2129	428.2140	2.46
MET_MATCH					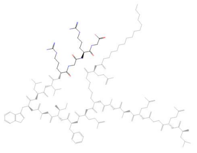	428.2379	428.2364	-3.30
MET_MATCH					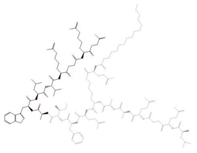	457.7693	457.7696	0.69
MET_MATCH					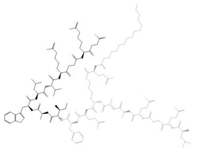	514.3110	514.3116	1.24
MET_MATCH					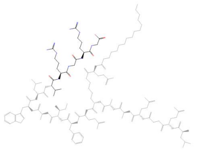	527.3035	527.3049	2.63

Metabolite: M3 -1382 RT=8.59

Type	score	sub. m/z observed	sub. m/z calculated	sub ppm		met. m/z observed	met. m/z calculated	met. ppm
MET_MATCH					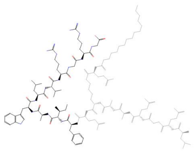	587.8450	587.8458	1.46
MET_MATCH					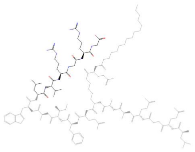	640.3885	640.3889	0.67
MET_MATCH					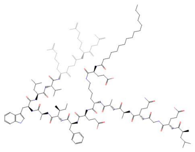	647.7271	647.7202	-10.7
MET_MATCH					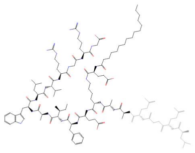	647.7271	647.7277	0.86
MET_MATCH					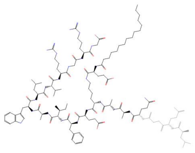	684.7407	684.7383	-3.51
MET_MATCH					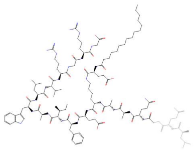	703.7452	703.7455	0.35
MET_MATCH					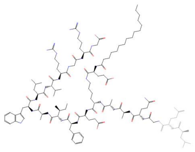	709.4205	709.4210	0.78
MET_MATCH					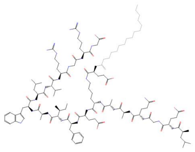	710.7189	710.7200	1.59
MET_MATCH					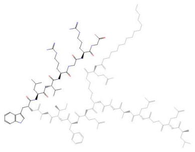	826.4659	826.4682	2.77

Metabolite: M3 -1382 RT=8.59

Type	score	sub. m/z observed	sub. m/z calculated	sub ppm		met. m/z observed	met. m/z calculated	met. ppm
MET_MATCH					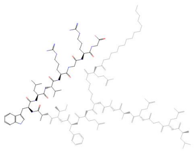	897.5027	897.5053	2.98
MET_MATCH					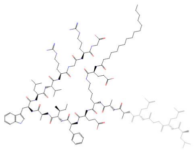	962.5781	962.5746	-3.62
MET_MATCH					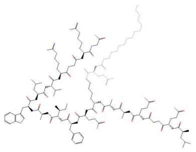	1001.0522	1001.0551	2.91
MET_MATCH					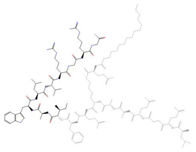	1010.5927	1010.5894	-3.21
MET_MATCH					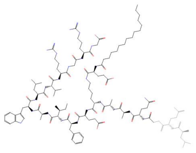	1055.1151	1055.1146	-0.44
MET_MATCH					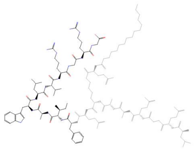	1157.6538	1157.6578	3.47

MS (+) FT

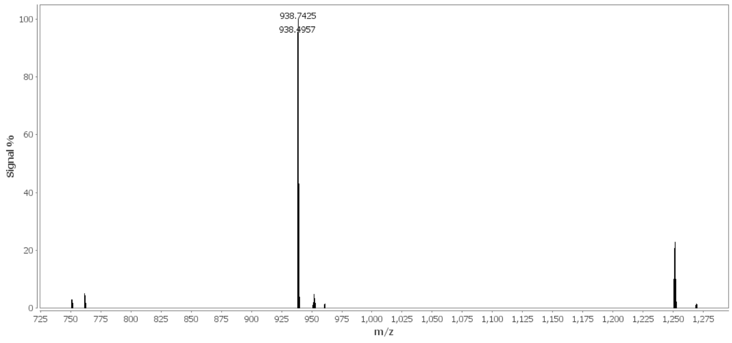

MS (+) FT

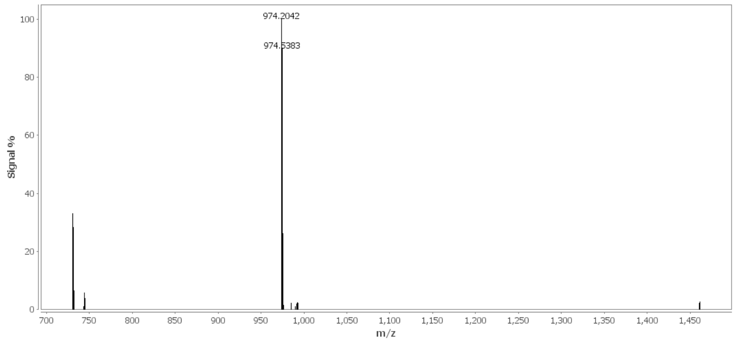

MS2 (+) FT activ = HCD:ce =

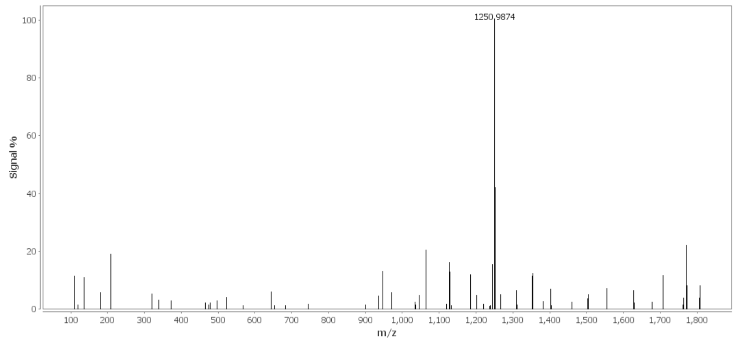

MS2 (+) FT activ = HCD:ce =

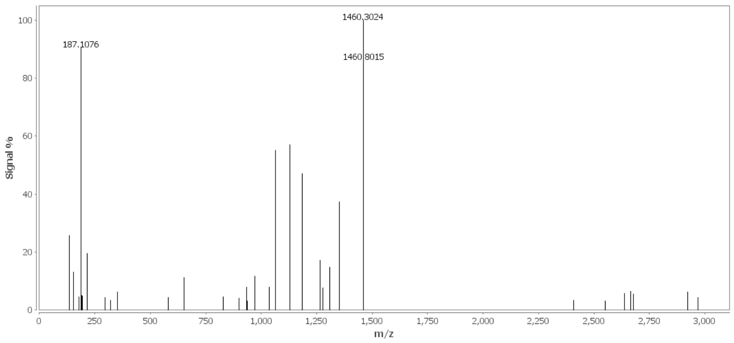

Metabolite: M7 -830 RT=9.16

Type	score	sub. m/z observed	sub. m/z calculated	sub ppm		met. m/z observed	met. m/z calculated	met. ppm
MATCH	20.3	750.7942	750.7966	3.21	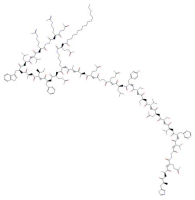 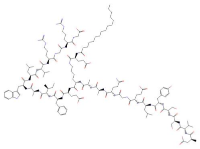	730.6542	730.6549	1.00
MATCH	20.3	750.7942	750.7966	3.21	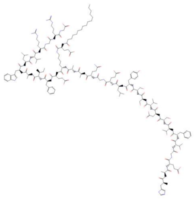 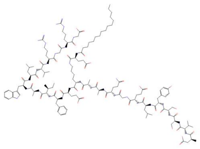	730.6542	730.6549	1.00
MATCH	20.3	750.7942	750.7966	3.21	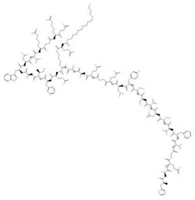 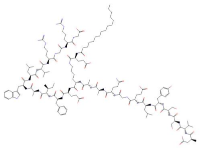	730.6542	730.6549	1.00
MATCH	69.6	750.7942	750.7966	3.21	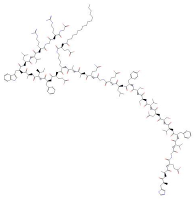 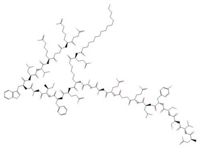	973.8700	973.8708	0.83
MATCH	69.6	750.7942	750.7966	3.21	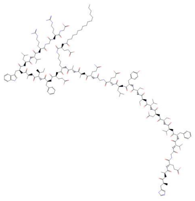 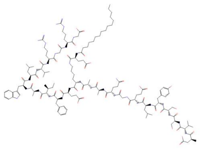	973.8700	973.8708	0.83
MATCH	69.6	750.7942	750.7966	3.21	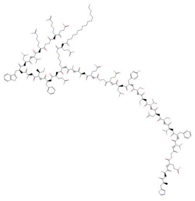 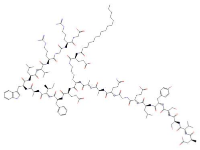	973.8700	973.8708	0.83
MATCH	3.5	750.7942	750.7966	3.21	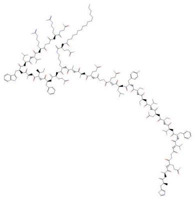 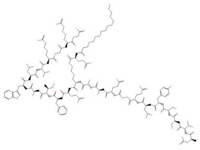	1460.3043	1460.3026	-1.20

Metabolite: M7 -830 RT=9.16

Type	score	sub. m/z observed	sub. m/z calculated	sub ppm		met. m/z observed	met. m/z calculated	met. ppm
MATCH	3.5	750.7942	750.7966	3.21	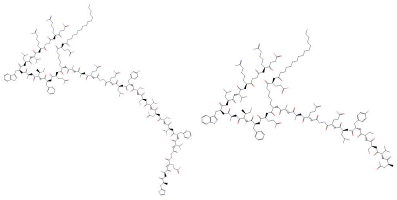	1460.3043	1460.3026	-1.20
MATCH	3.5	750.7942	750.7966	3.21	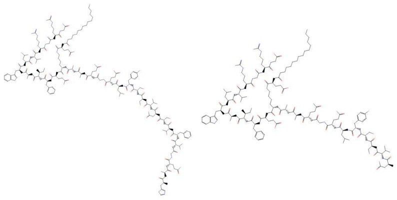	1460.3043	1460.3026	-1.20
MATCH	65.8	938.2475	938.2439	-3.87	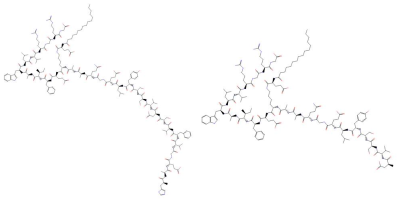	730.6542	730.6549	1.00
MATCH	65.8	938.2475	938.2439	-3.87	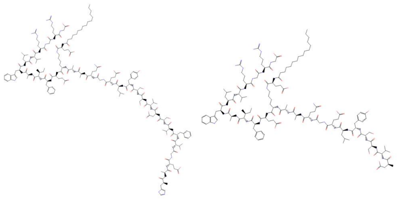	730.6542	730.6549	1.00
MATCH	65.8	938.2475	938.2439	-3.87	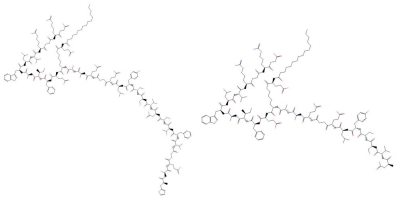	730.6542	730.6549	1.00
MATCH	115.1	938.2475	938.2439	-3.87	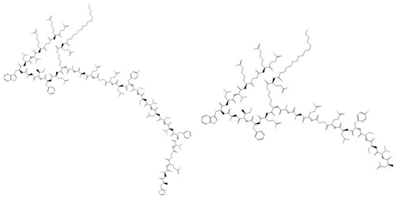	973.8700	973.8708	0.83
MATCH	115.1	938.2475	938.2439	-3.87	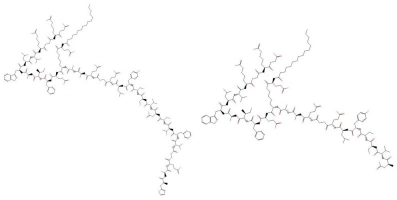	973.8700	973.8708	0.83
MATCH	115.1	938.2475	938.2439	-3.87	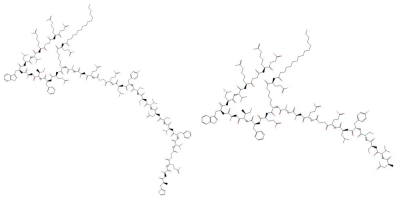	973.8700	973.8708	0.83
MATCH	49.0	938.2475	938.2439	-3.87	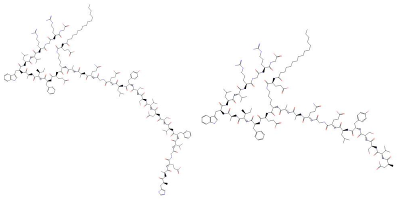	1460.3043	1460.3026	-1.20

Metabolite: M7 -830 RT=9.16

Type	score	sub. m/z observed	sub. m/z calculated	sub ppm		met. m/z observed	met. m/z calculated	met. ppm
MATCH	49.0	938.2475	938.2439	-3.87	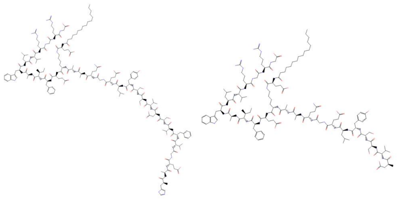	1460.3043	1460.3026	-1.20
MATCH	49.0	938.2475	938.2439	-3.87	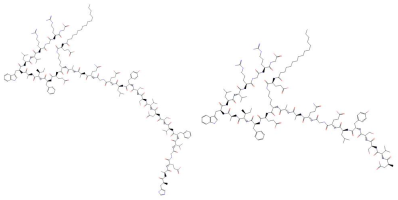	1460.3043	1460.3026	-1.20
MATCH	29.1	1250.6532	1250.6561	2.29	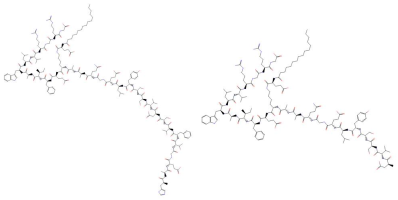	730.6542	730.6549	1.00
MATCH	29.1	1250.6532	1250.6561	2.29	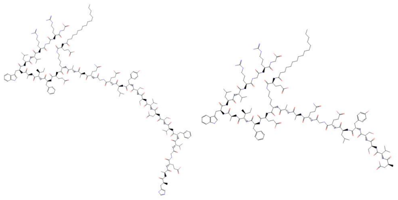	730.6542	730.6549	1.00
MATCH	29.1	1250.6532	1250.6561	2.29	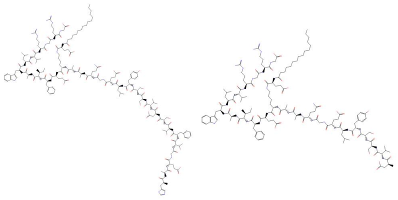	730.6542	730.6549	1.00
MATCH	78.4	1250.6532	1250.6561	2.29	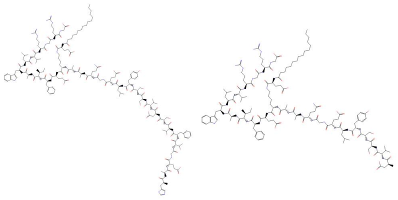	973.8700	973.8708	0.83
MATCH	78.4	1250.6532	1250.6561	2.29	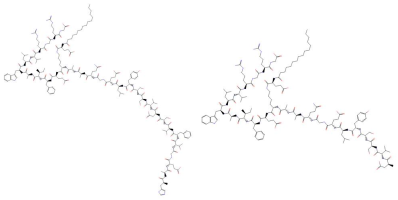	973.8700	973.8708	0.83
MATCH	78.4	1250.6532	1250.6561	2.29	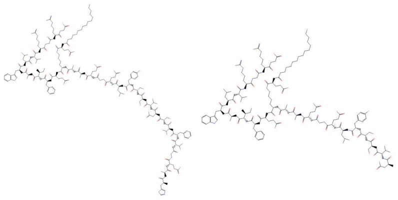	973.8700	973.8708	0.83
MATCH	12.2	1250.6532	1250.6561	2.29	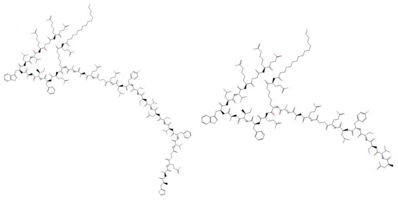	1460.3043	1460.3026	-1.20

Metabolite: M7 -830 RT=9.16

Type	score	sub. m/z observed	sub. m/z calculated	sub ppm		met. m/z observed	met. m/z calculated	met. ppm
MATCH	12.2	1250.6532	1250.6561	2.29	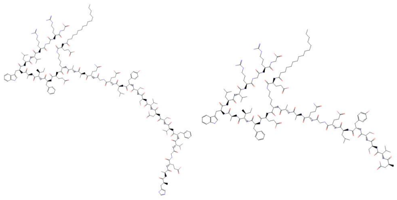	1460.3043	1460.3026	-1.20
MATCH	12.2	1250.6532	1250.6561	2.29	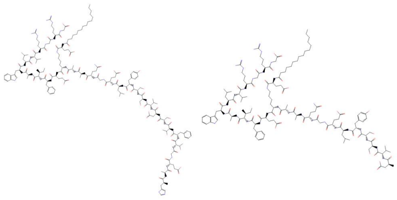	1460.3043	1460.3026	-1.20
MATCH	36.5	136.0765	136.0706	-43.3	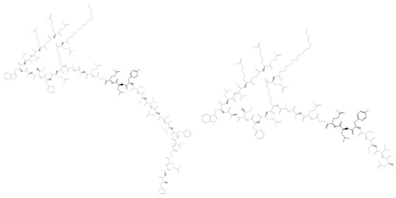	136.0755	136.0706	-36.2
MATCH	36.5	136.0765	136.0706	-43.3	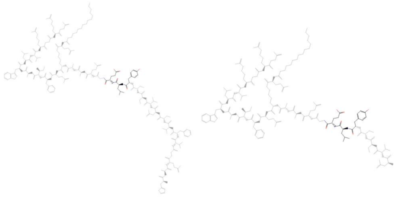	136.0755	136.0706	-36.2
MATCH	36.5	136.0765	136.0737	-20.7	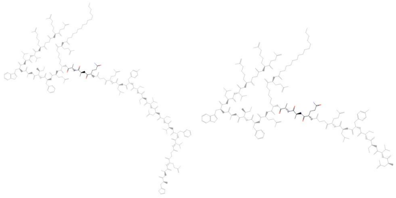	136.0755	136.0737	-13.6
MATCH	36.5	136.0765	136.0737	-20.7	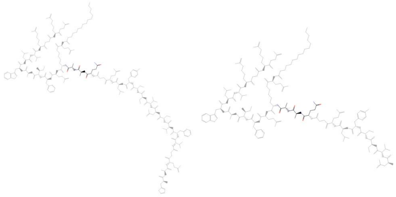	136.0755	136.0737	-13.6
MATCH	12.6	652.3655	652.3671	2.43	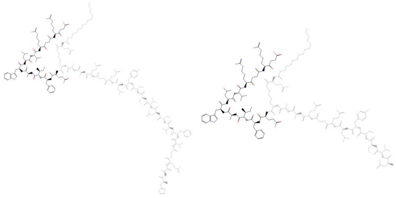	652.3679	652.3671	-1.23
MATCH	5.7	900.0483	900.0507	2.75	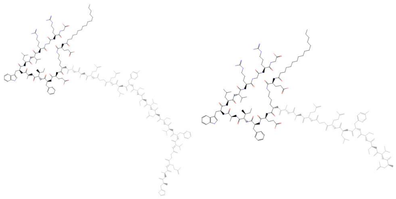	900.0524	900.0507	-1.89
MATCH	12.4	935.5676	935.5693	1.80	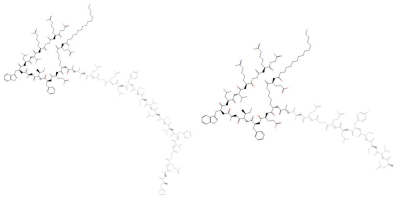	935.5667	935.5693	2.76

Metabolite: M7 -830 RT=9.16

Type	score	sub. m/z observed	sub. m/z calculated	sub ppm		met. m/z observed	met. m/z calculated	met. ppm
MATCH	17.3	971.0854	971.0878	2.54	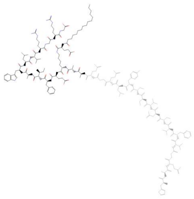 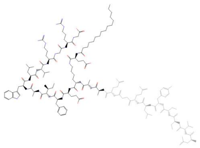	971.0872	971.0878	0.65
MATCH	10.5	1035.1141	1035.1171	2.96	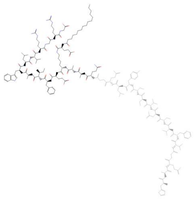 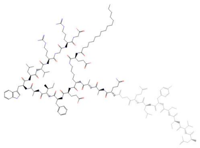	1035.1158	1035.1171	1.28
MATCH	24.4	1045.4564	1045.4585	1.99	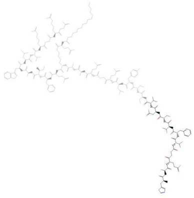 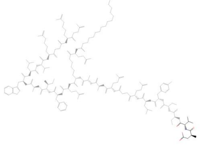	215.1023	215.1026	1.70
MATCH	75.5	1063.6253	1063.6279	2.43	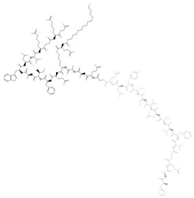 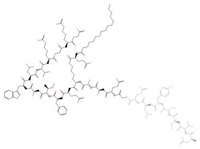	1063.6251	1063.6279	2.61
MATCH	68.7	1128.1470	1128.1492	1.88	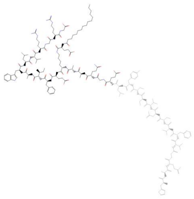 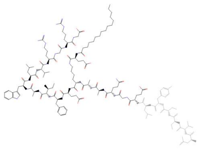	1128.1478	1128.1492	1.23
MATCH	58.8	1184.6888	1184.6912	2.06	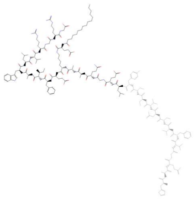 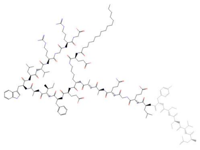	1184.6878	1184.6912	2.87
MATCH	151.1	1250.6556	1250.6561	0.38	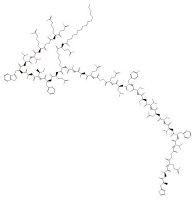 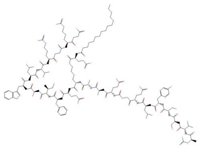	1460.3024	1460.3026	0.14
MATCH	10.9	1266.2207	1266.2229	1.70	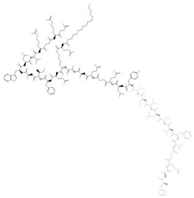 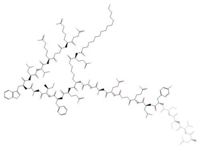	1266.2113	1266.2229	9.13
MATCH	36.7	1353.2522	1353.2549	2.01	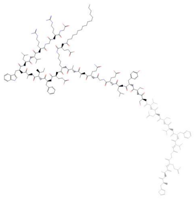 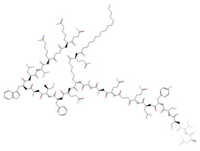	1353.2482	1353.2549	4.92

Metabolite: M7 -830 RT=9.16

Type	score	sub. m/z observed	sub. m/z calculated	sub ppm		met. m/z observed	met. m/z calculated	met. ppm
MISMATCH	-25.5	643.2822	643.2835	2.01	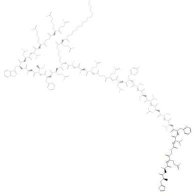	215.1023	215.1023	0.00
MET_MATCH					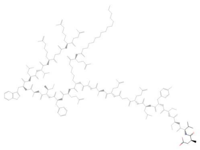	187.1076	187.1077	0.55

MS (+) FT

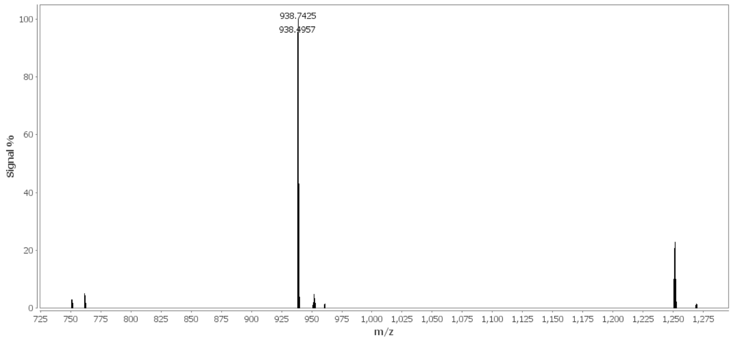

MS (+) FT

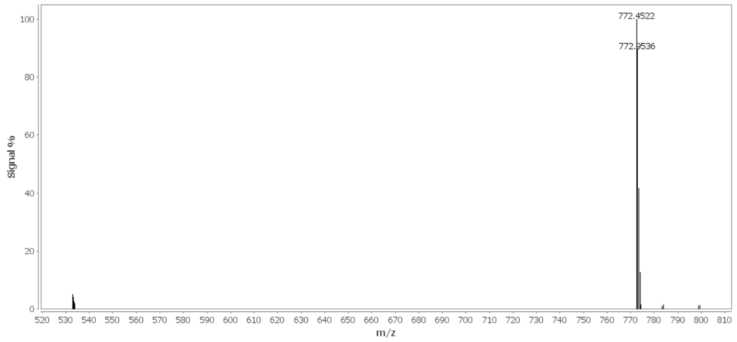

MS2 (+) FT activ = HCD:ce =

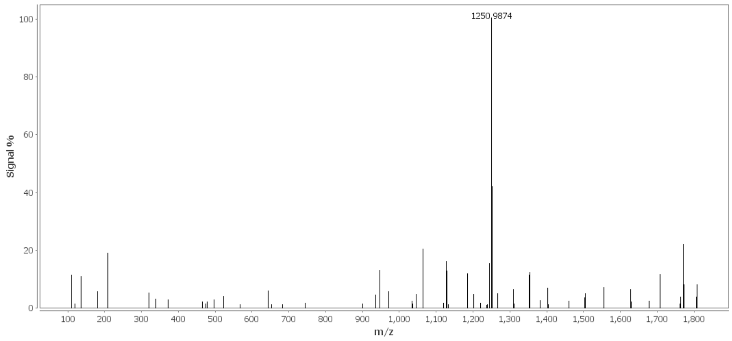

MS2 (+) FT activ = HCD:ce =

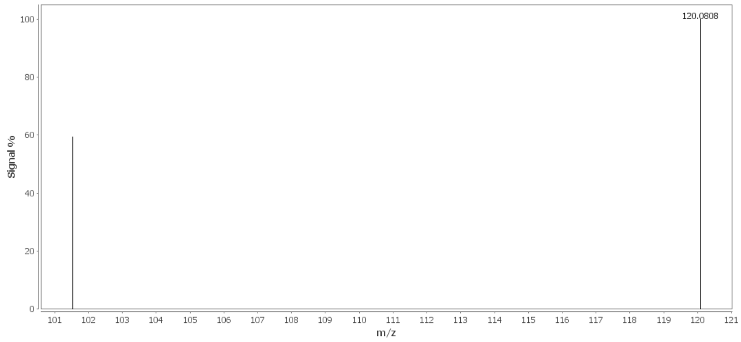

Metabolite: M8 -2206 RT=9.44

Type	score	sub. m/z observed	sub. m/z calculated	sub ppm		met. m/z observed	met. m/z calculated	met. ppm
MATCH	101.3	750.7942	750.7966	3.21	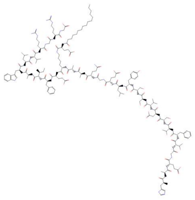 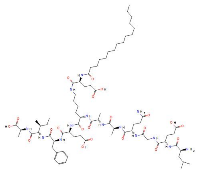	772.4522	772.4527	0.73
					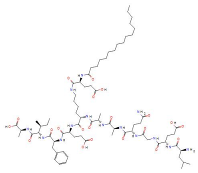	772.4522	772.4527	0.73

Metabolite: M8 -2206 RT=9.44

Type	score	sub. m/z observed	sub. m/z calculated	sub ppm			met. m/z observed	met. m/z calculated	met. ppm
						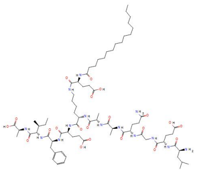	772.4522	772.4527	0.73
MATCH	146.8	938.2475	938.2439	-3.87	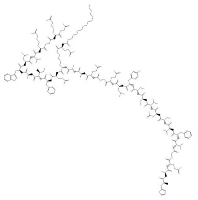	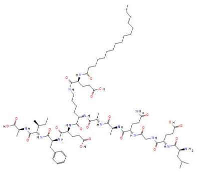	772.4522	772.4527	0.73
						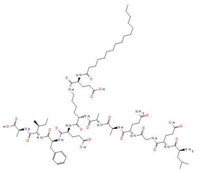	772.4522	772.4527	0.73
						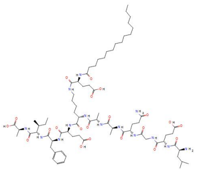	772.4522	772.4527	0.73
MATCH	110.1	1250.6532	1250.6561	2.29	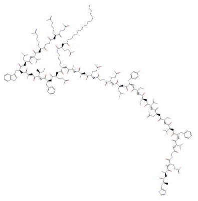	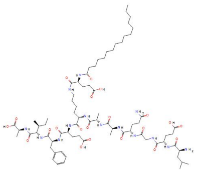	772.4522	772.4527	0.73
						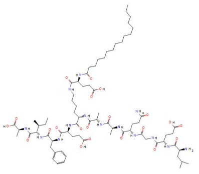	772.4522	772.4527	0.73
						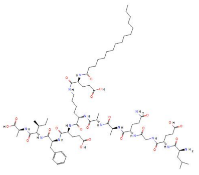	772.4522	772.4527	0.73
MISMATCH	-101.4	120.0807	120.0788	-16.4	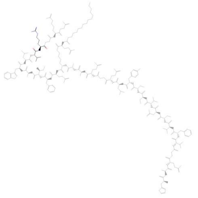		120.0808	120.0808	0.00

MS (+) FT

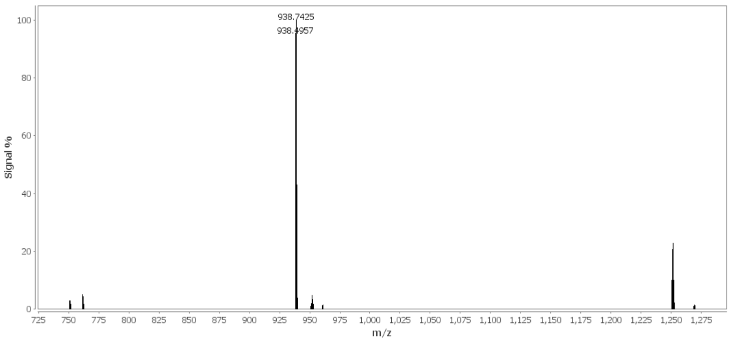

MS (+) FT

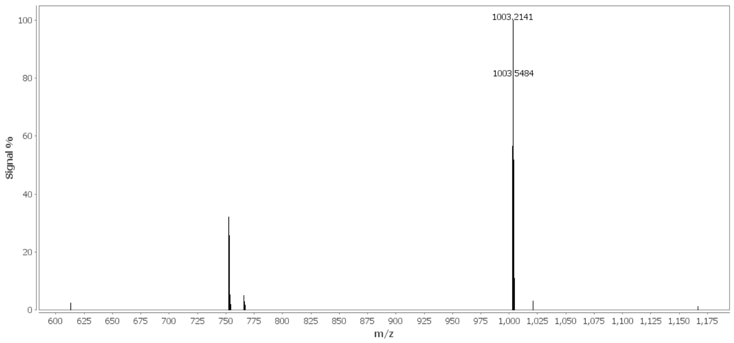

MS2 (+) FT activ = HCD:ce =

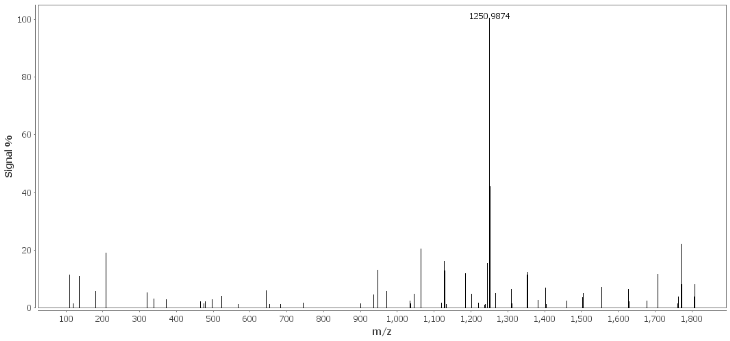

MS2 (+) FT activ = HCD:ce =

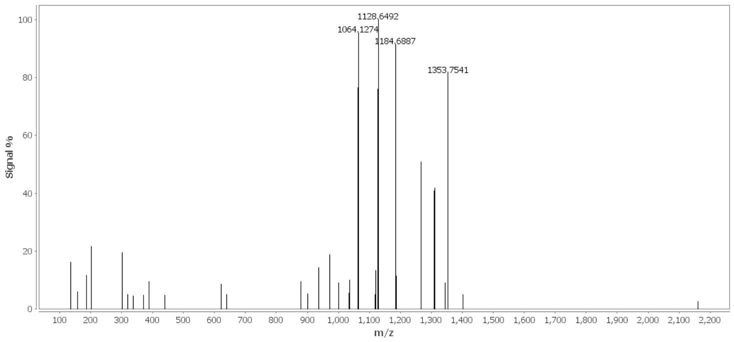

Metabolite: M5 -743 RT=8.97

Type	score	sub. m/z observed	sub. m/z calculated	sub ppm			met. m/z observed	met. m/z calculated	met. ppm
MATCH	22.5	750.7942	750.7966	3.21	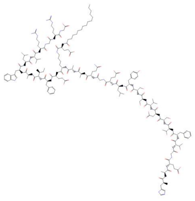	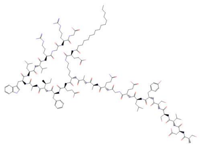	752.4114	752.4129	2.04
MATCH	22.5	750.7942	750.7966	3.21	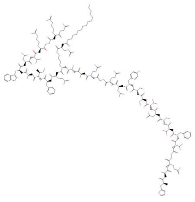	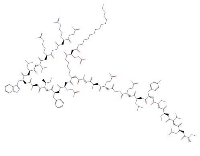	752.4114	752.4129	2.04
						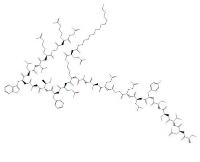	752.4114	752.4129	2.04
MATCH	57.8	750.7942	750.7966	3.21	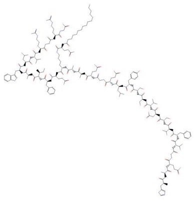	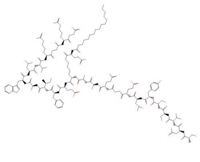	1002.8799	1002.8815	1.53
MATCH	57.8	750.7942	750.7966	3.21	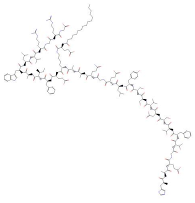	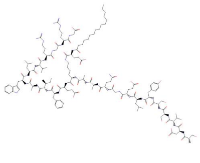	1002.8799	1002.8815	1.53

Metabolite: M5 -743 RT=8.97

Type	score	sub. m/z observed	sub. m/z calculated	sub ppm			met. m/z observed	met. m/z calculated	met. ppm
						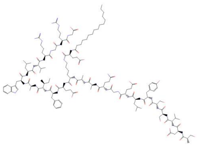	1002.8799	1002.8815	1.53
MATCH	68.0	938.2475	938.2439	-3.87	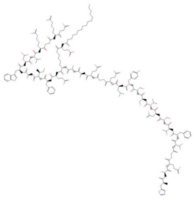	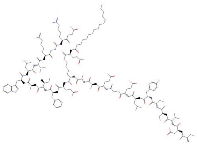	752.4114	752.4129	2.04
MATCH	68.0	938.2475	938.2439	-3.87	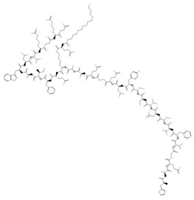	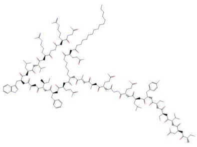	752.4114	752.4129	2.04
						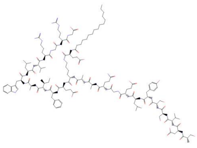	752.4114	752.4129	2.04
MATCH	103.3	938.2475	938.2439	-3.87	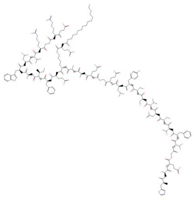	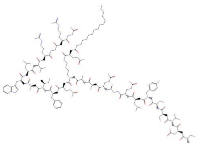	1002.8799	1002.8815	1.53
MATCH	103.3	938.2475	938.2439	-3.87	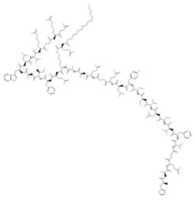	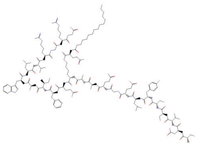	1002.8799	1002.8815	1.53
						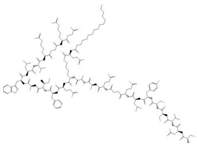	1002.8799	1002.8815	1.53
MATCH	31.3	1250.6532	1250.6561	2.29	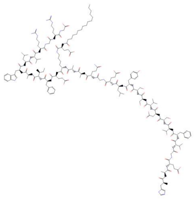	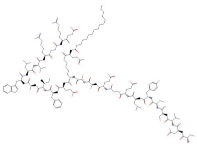	752.4114	752.4129	2.04
MATCH	31.3	1250.6532	1250.6561	2.29	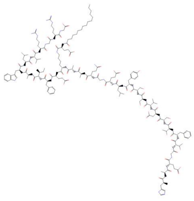	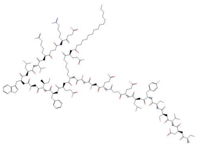	752.4114	752.4129	2.04

Metabolite: M5 -743 RT=8.97

Type	score	sub. m/z observed	sub. m/z calculated	sub ppm			met. m/z observed	met. m/z calculated	met. ppm
						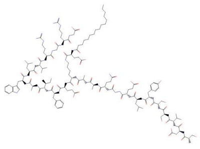	752.4114	752.4129	2.04
MATCH	66.6	1250.6532	1250.6561	2.29	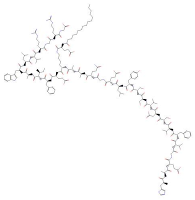	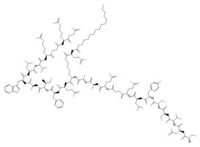	1002.8799	1002.8815	1.53
MATCH	66.6	1250.6532	1250.6561	2.29	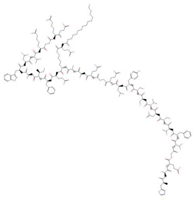	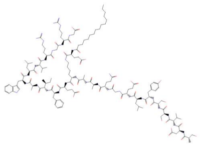	1002.8799	1002.8815	1.53
						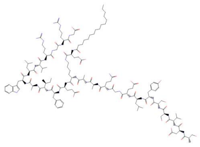	1002.8799	1002.8815	1.53
MATCH	27.1	136.0765	136.0693	-53.2	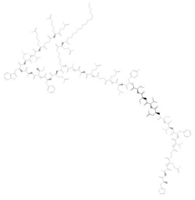	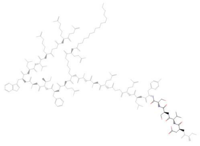	136.0755	136.0693	-46.0
MATCH	27.1	136.0765	136.0706	-43.3	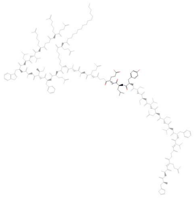	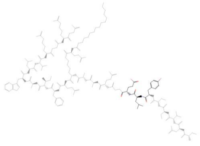	136.0755	136.0706	-36.2
MATCH	27.1	136.0765	136.0706	-43.3	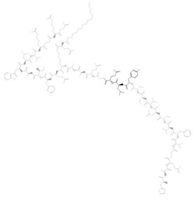	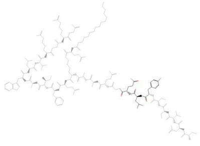	136.0755	136.0706	-36.2
MATCH	27.1	136.0765	136.0737	-20.7	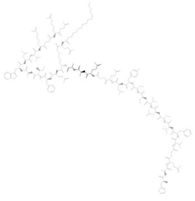	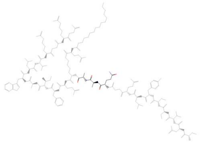	136.0755	136.0737	-13.6
MATCH	27.1	136.0765	136.0737	-20.7	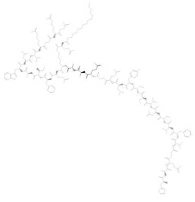	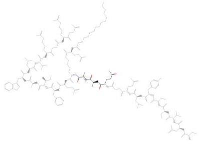	136.0755	136.0737	-13.6

Metabolite: M5 -743 RT=8.97

Type	score	sub. m/z observed	sub. m/z calculated	sub ppm		met. m/z observed	met. m/z calculated	met. ppm
MATCH	6.8	900.0483	900.0507	2.75	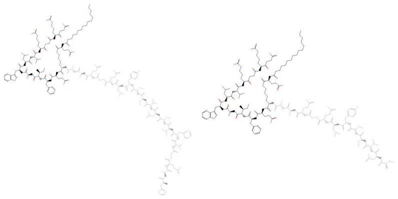	900.0498	900.0507	1.03
MATCH	10.0	935.5676	935.5693	1.80	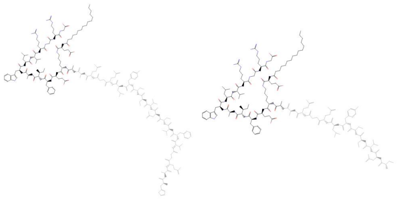	935.5656	935.5693	3.99
MATCH	34.8	946.3881	946.3901	2.12	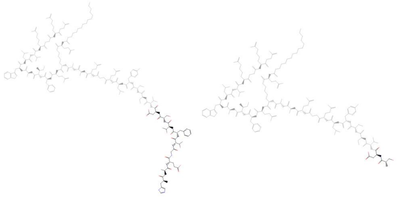	203.0659	203.0662	1.70
MATCH	20.4	971.0854	971.0878	2.54	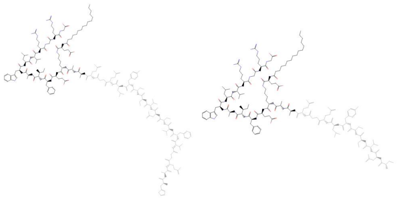	971.0837	971.0878	4.29
MATCH	8.1	1035.1141	1035.1171	2.96	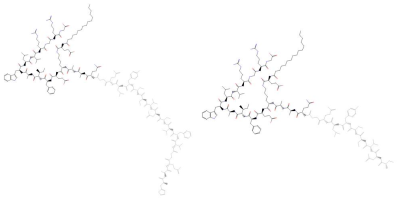	1035.1111	1035.1171	5.85
MATCH	24.4	1045.4564	1045.4585	1.99	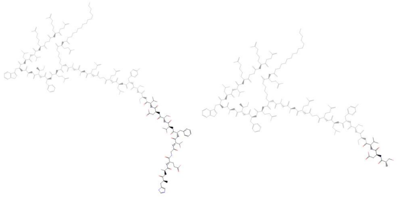	302.1337	302.1347	3.29
MATCH	97.0	1063.6253	1063.6279	2.43	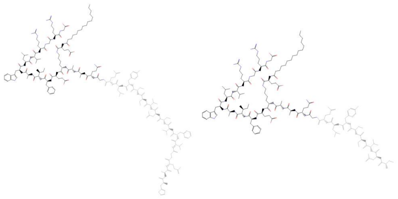	1063.6266	1063.6279	1.24
MATCH	6.5	1119.1410	1119.1439	2.54	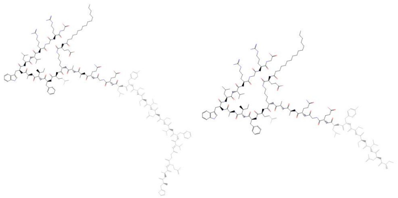	1119.1417	1119.1439	1.93
MATCH	6.5	1119.1410	1119.1439	2.54	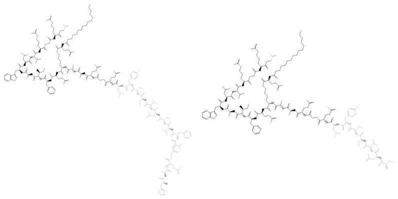	1119.1417	1119.1439	1.93

Metabolite: M5 -743 RT=8.97

Type	score	sub. m/z observed	sub. m/z calculated	sub ppm			met. m/z observed	met. m/z calculated	met. ppm
MATCH	6.5	1119.1410	1119.1439	2.54	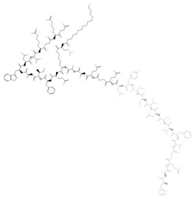	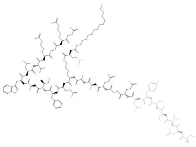	1119.1417	1119.1439	1.93
MATCH	6.5	1119.1410	1119.1439	2.54	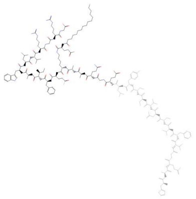	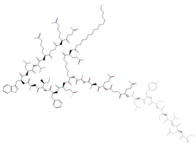	1119.1417	1119.1439	1.93
MATCH	6.5	1119.1410	1119.1439	2.54	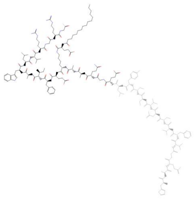	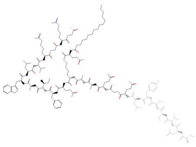	1119.1417	1119.1439	1.93
MATCH	6.5	1119.1410	1119.1439	2.54	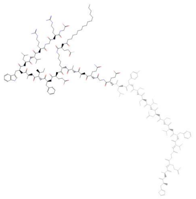	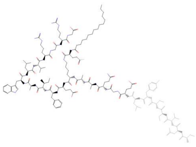	1119.1417	1119.1439	1.93
MATCH	6.5	1119.1410	1119.1439	2.54	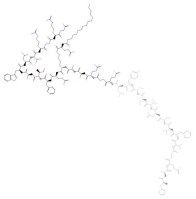	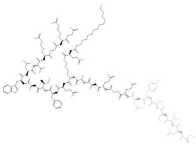	1119.1417	1119.1439	1.93
MATCH	6.5	1119.1410	1119.1439	2.54	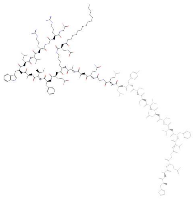	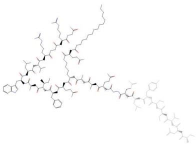	1119.1417	1119.1439	1.93
MATCH	92.0	1128.1470	1128.1492	1.88	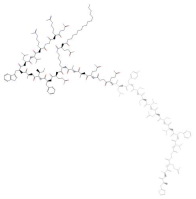	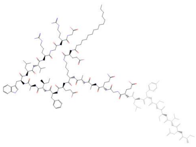	1128.1468	1128.1492	2.14
MATCH	10.9	1132.4870	1132.4905	3.12	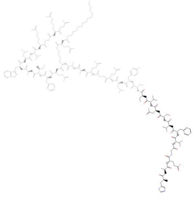	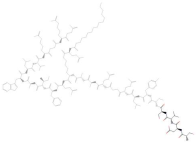	389.1656	389.1667	2.83
MATCH	103.4	1184.6888	1184.6912	2.06	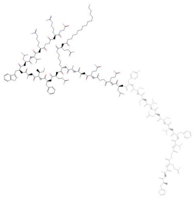	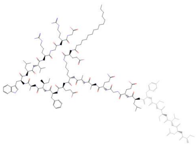	1184.6887	1184.6912	2.08

Metabolite: M5 -743 RT=8.97

Type	score	sub. m/z observed	sub. m/z calculated	sub ppm			met. m/z observed	met. m/z calculated	met. ppm
MATCH	37.5	1266.2207	1266.2229	1.70	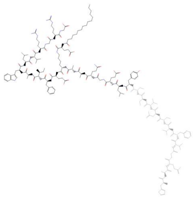	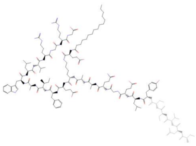	1266.2186	1266.2229	3.32
MATCH	41.3	1309.7364	1309.7389	1.93	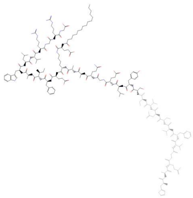	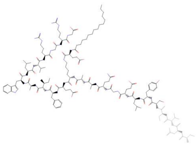	1309.7388	1309.7389	0.06
MATCH	49.8	1353.2522	1353.2549	2.01	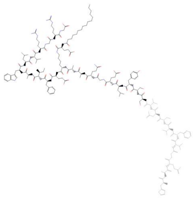	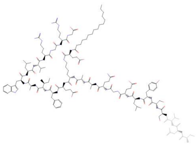	1353.2522	1353.2549	1.99
MISMATCH	-9.7	682.7895	682.7913	2.67	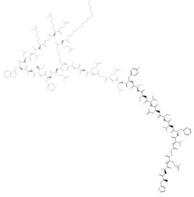		621.2515	621.2515	0.00
MISMATCH	-7.5	1382.5827	1382.5859	2.33	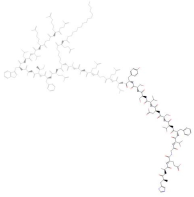		320.1235	320.1235	0.00
MET_MATCH						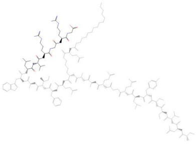	640.3879	640.3889	1.63
MET_MATCH						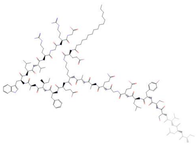	1344.7400	1344.7416	1.21

MS (+) FT

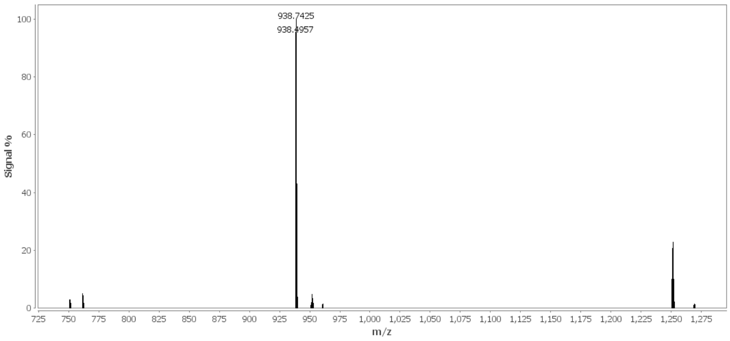

MS (+) FT

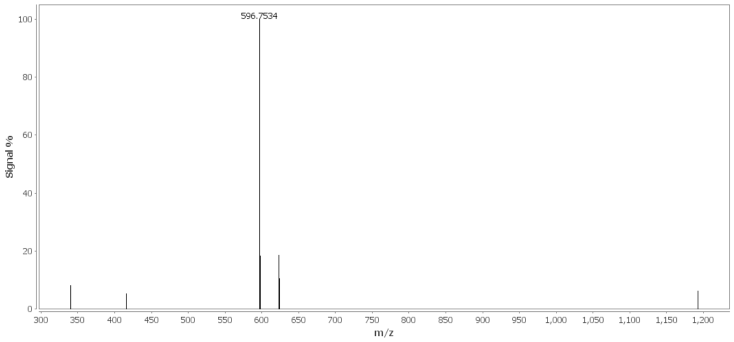

MS2 (+) FT activ = HCD:ce =

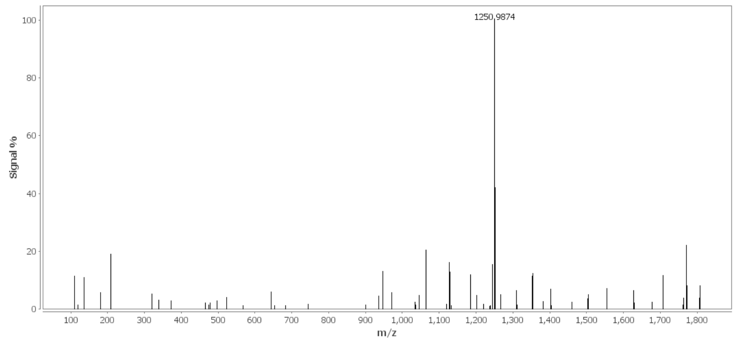

MS2 (+) FT activ = HCD:ce =

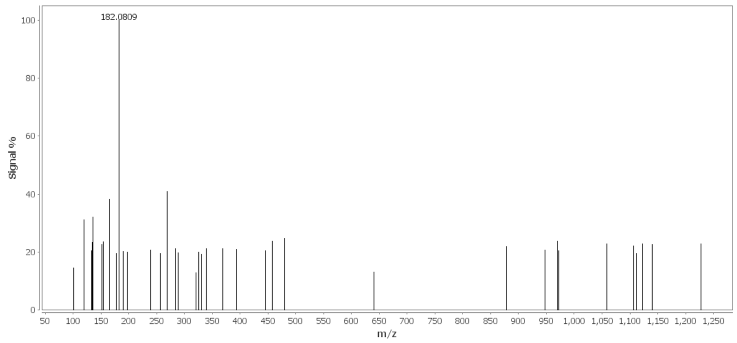

Metabolite: M1 -2557 RT=4.53

Type	score	sub. m/z observed	sub. m/z calculated	sub ppm			met. m/z observed	met. m/z calculated	met. ppm
MATCH	101.3	750.7942	750.7966	3.21	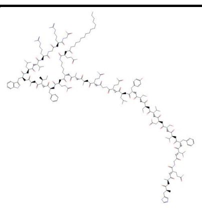	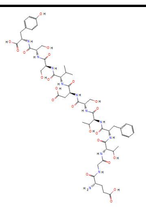	596.7534	596.7539	0.72
						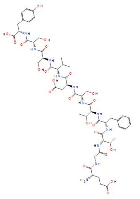	596.7534	596.7539	0.72
						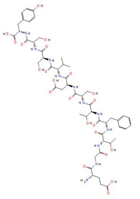	596.7534	596.7539	0.72
MATCH	146.8	938.2475	938.2439	-3.87	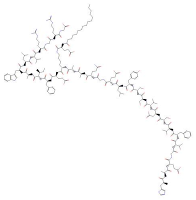	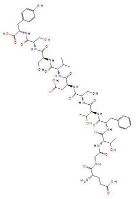	596.7534	596.7539	0.72
						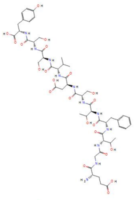	596.7534	596.7539	0.72
						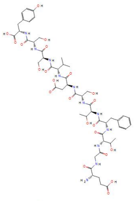	596.7534	596.7539	0.72
MATCH	110.1	1250.6532	1250.6561	2.29	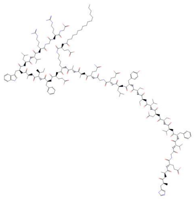	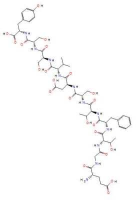	596.7534	596.7539	0.72

Metabolite: M1 -2557 RT=4.53

Type	score	sub. m/z observed	sub. m/z calculated	sub ppm			met. m/z observed	met. m/z calculated	met. ppm
MATCH	110.1	1250.6532	1250.6561	2.29	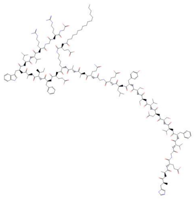	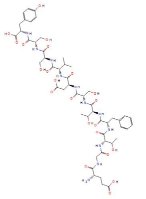	596.7534	596.7539	0.72
						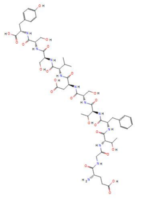	596.7534	596.7539	0.72
MATCH	16.3	1250.6532	1250.6561	2.29	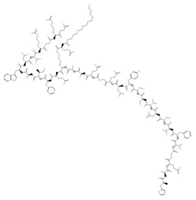	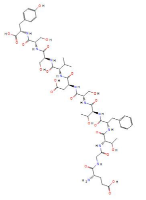	1192.5002	1192.5004	0.21
MISMATCH	-32.6	120.0807	120.0788	-16.4	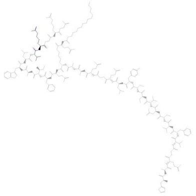		120.0808	120.0808	0.00
MISMATCH	-43.0	136.0765	136.0737	-20.7	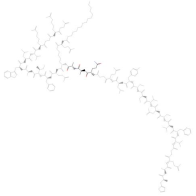		136.0754	136.0754	0.00
MISMATCH	-104.2	1266.2207	1266.2229	1.70	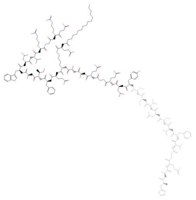		182.0809	182.0809	0.00
MISMATCH	-45.2	1309.7364	1309.7389	1.93	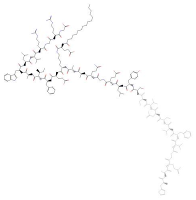		269.1126	269.1126	0.00
MET_MATCH						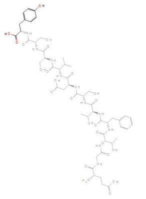	165.0542	165.0546	2.51
MET_MATCH						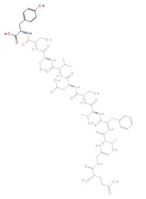	182.0809	182.0812	1.75

Metabolite: M1 -2557 RT=4.53

Type	score	sub. m/z observed	sub. m/z calculated	sub ppm		met. m/z observed	met. m/z calculated	met. ppm
MET_MATCH					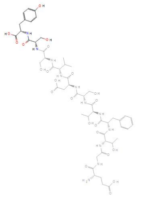	269.1126	269.1132	2.36
MET_MATCH					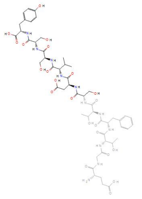	640.2554	640.2461	-14.6

MS (+) FT

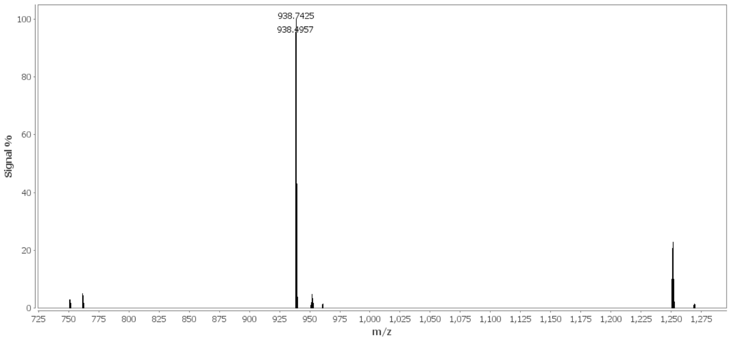

MS (+) FT

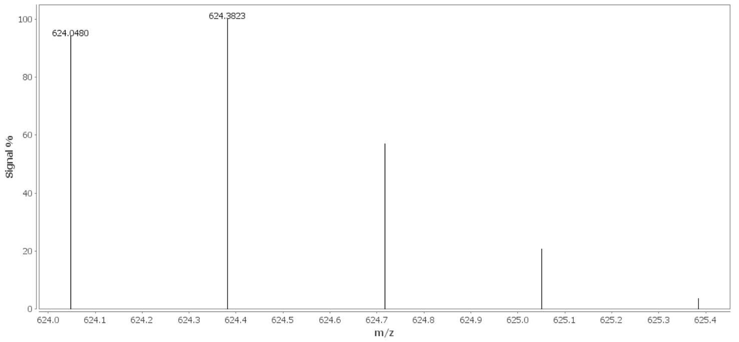

MS2 (+) FT activ = HCD:ce =

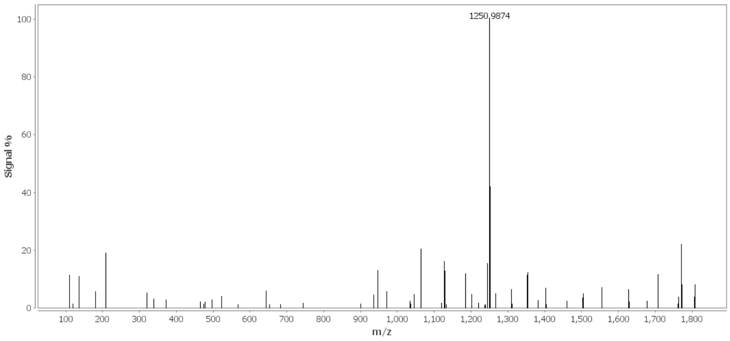

MS2 (+) FT activ = HCD:ce =

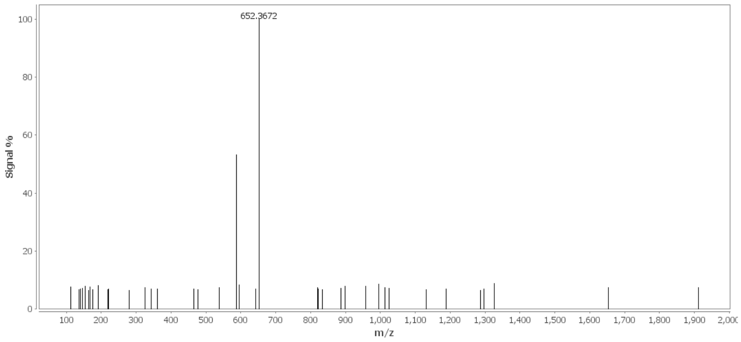

Metabolite: M2 -1880 RT=8.27

Type	score	sub. m/z observed	sub. m/z calculated	sub ppm		met. m/z observed	met. m/z calculated	met. ppm
MATCH	95.4	750.7942	750.7966	3.21	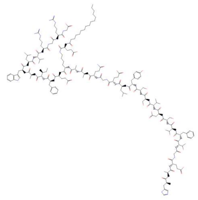 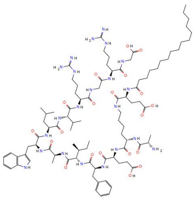	624.0480	624.0486	0.93
					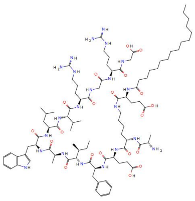	624.0480	624.0486	0.93

Metabolite: M2 -1880 RT=8.27

Type	score	sub. m/z observed	sub. m/z calculated	sub ppm			met. m/z observed	met. m/z calculated	met. ppm
						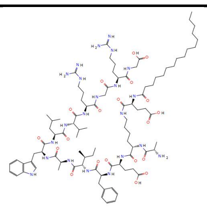	624.0480	624.0486	0.93
MATCH	140.9	938.2475	938.2439	-3.87	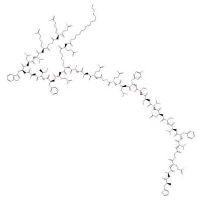	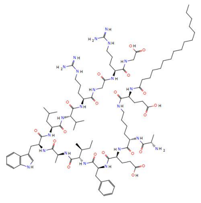	624.0480	624.0486	0.93
						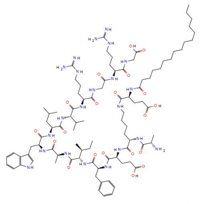	624.0480	624.0486	0.93
						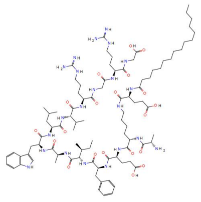	624.0480	624.0486	0.93
MATCH	104.2	1250.6532	1250.6561	2.29	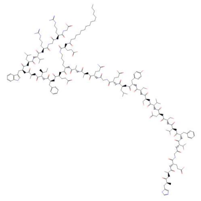	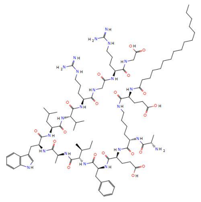	624.0480	624.0486	0.93
						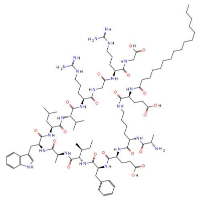	624.0480	624.0486	0.93
						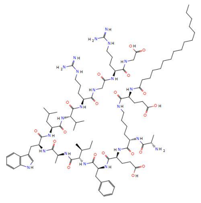	624.0480	624.0486	0.93
MATCH	101.3	652.3655	652.3671	2.43	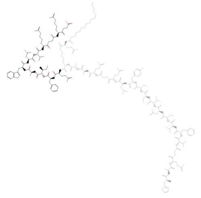	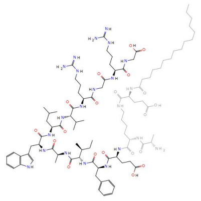	652.3672	652.3671	-0.07
MISMATCH	-9.1	464.6927	464.6934	1.54	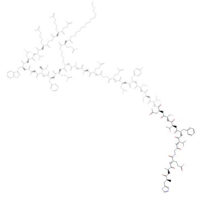		464.6956	464.6956	0.00

Metabolite: M2 -1880 RT=8.27

Type	score	sub. m/z observed	sub. m/z calculated	sub ppm		met. m/z observed	met. m/z calculated	met. ppm
MET_MATCH					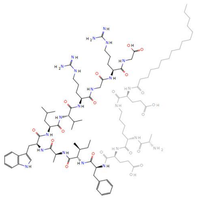	587.8452	587.8458	1.11
