# Supplementary material for: Software-aided approach to investigate peptide structure and metabolic susceptibility of amide bonds in peptide drugs based on high resolution mass spectrometry
Source: PLoS One. 2017 Nov 1;12(11):e0186461. doi: 10.1371/journal.pone.0186461 (PMC5665424; doi:10.1371/journal.pone.0186461)
Supplement: S1 File — (ZIP) [file pone.0186461.s007.zip › SFiles/S48_File.pdf]

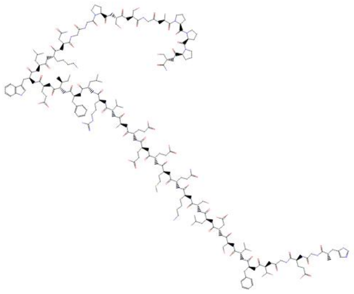

Exenatide

Property name	Property value
Time	0min, 2min, 4min, 24min
Instrument	ddMS2
Matrix	DPP4

Chromatograms

Time=0min

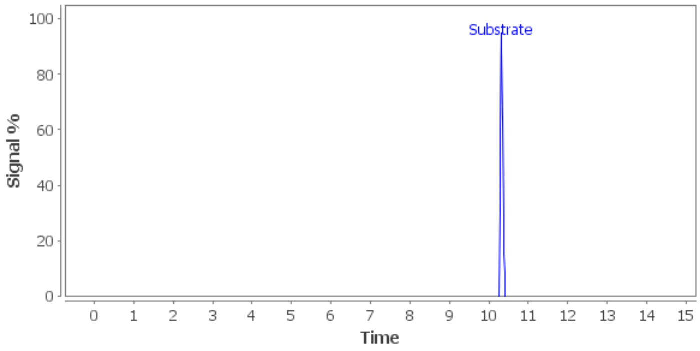

Time=2min

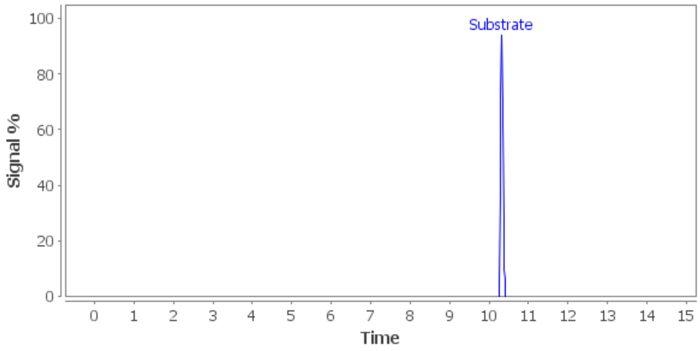

Time=4min

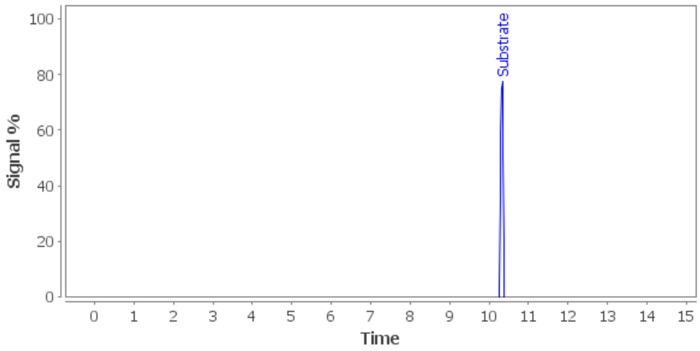

Time=24min

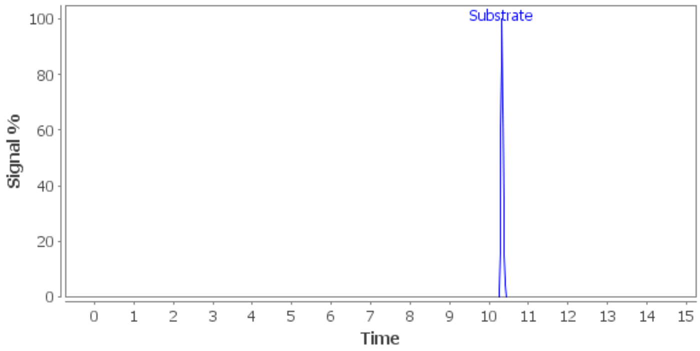

Custom Charts

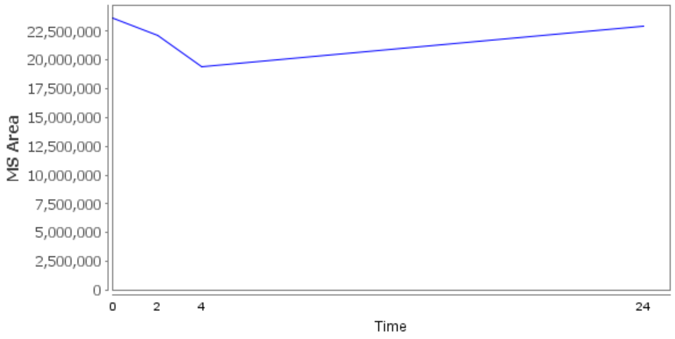

Fragmentation

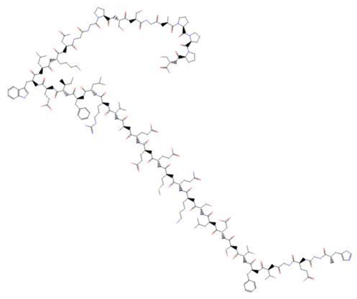

Exenatide
