# Supplementary material for: Software-aided approach to investigate peptide structure and metabolic susceptibility of amide bonds in peptide drugs based on high resolution mass spectrometry
Source: PLoS One. 2017 Nov 1;12(11):e0186461. doi: 10.1371/journal.pone.0186461 (PMC5665424; doi:10.1371/journal.pone.0186461)
Supplement: S1 File — (ZIP) [file pone.0186461.s007.zip › SFiles/S49_File.pdf]

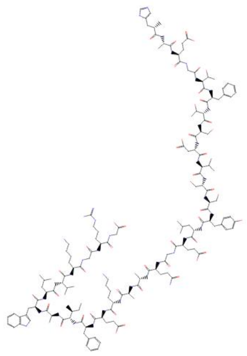

GLP1

Property name	Property value
Time	0min, 5min, 15min, 30min, 60min
Instrument	ddMS2
Matrix	NEP

Chromatograms

Time=0min

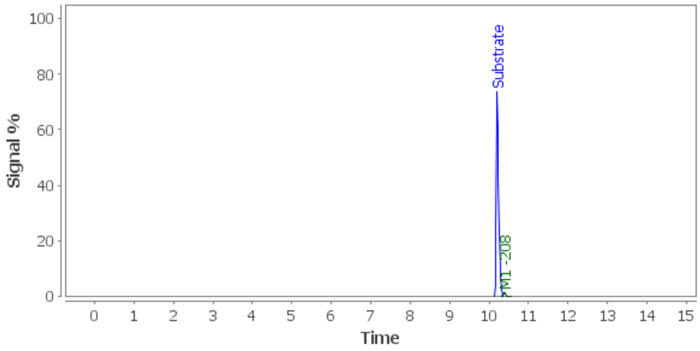

Time=5min

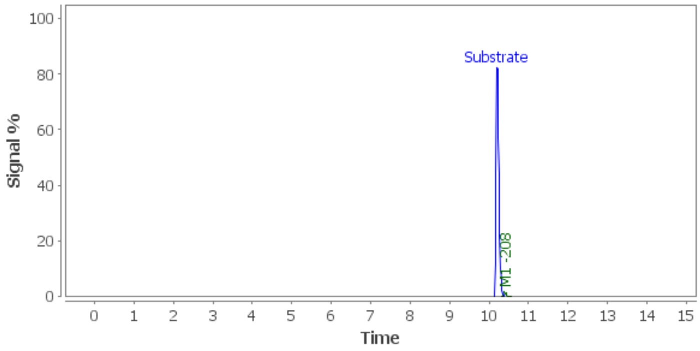

Time=15min

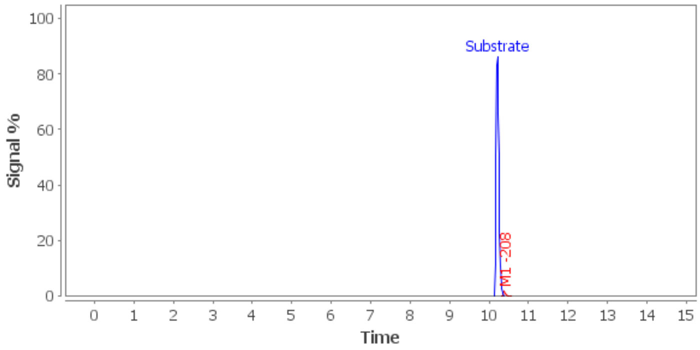

Time=30min

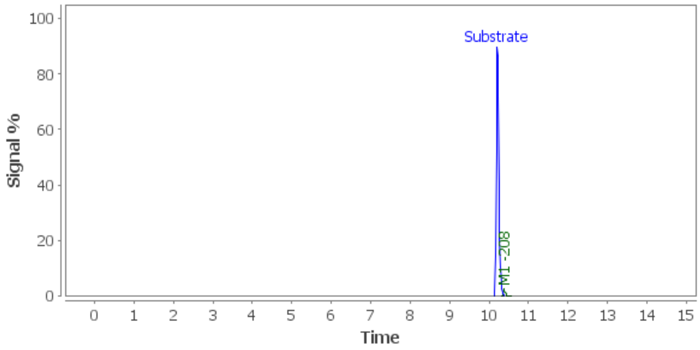

Time=60min

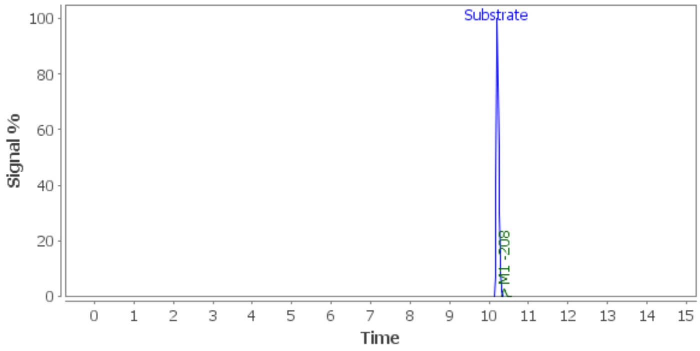

Custom Charts

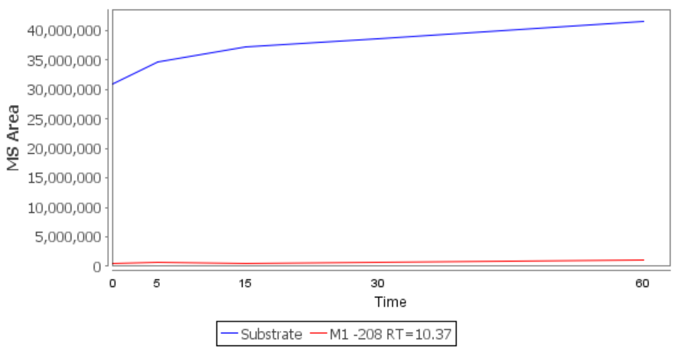

Fragmentation

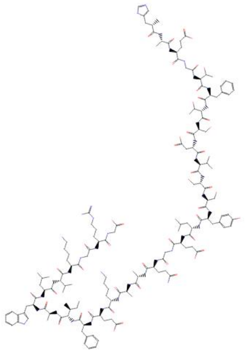

GLP1

MS (+) FT

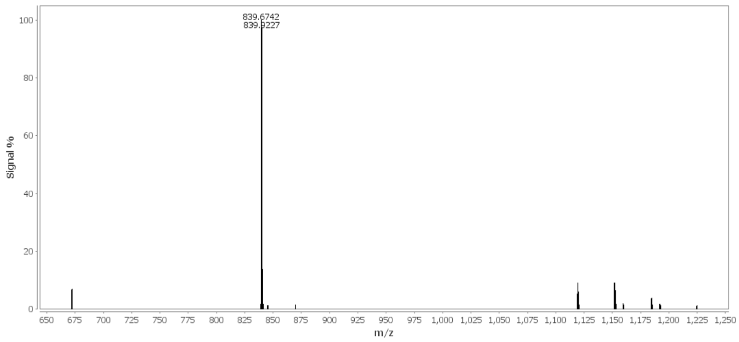

MS (+) FT

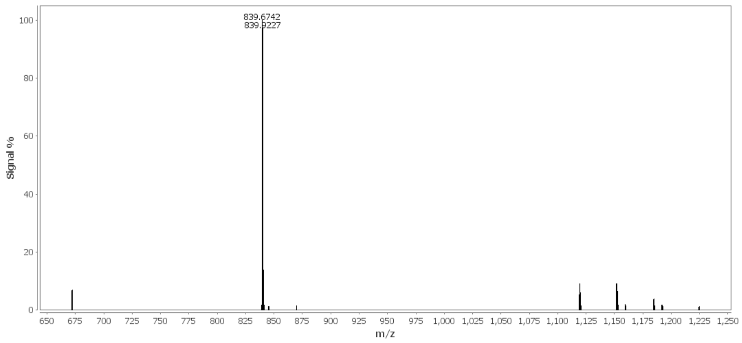

MS2 (+) FT activ = HCD:ce =

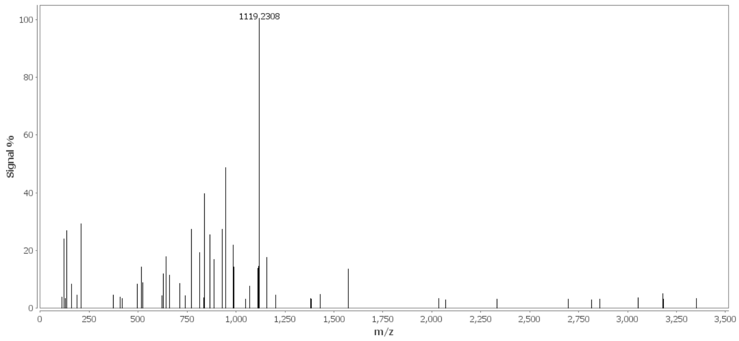

MS2 (+) FT activ = HCD:ce =

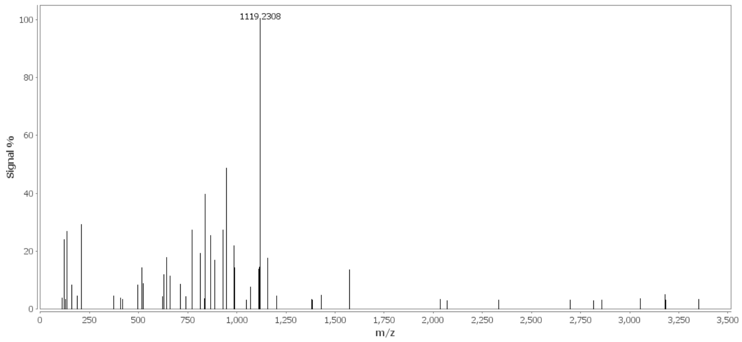

Metabolite: Substrate

Type	score	sub. m/z observed	sub. m/z calculated	sub ppm			met. m/z observed	met. m/z calculated	met. ppm
MATCH	60.3	1118.8936	1118.8966	2.72	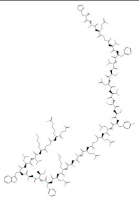	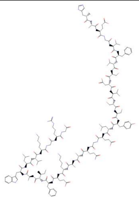	1118.8936	1118.8966	2.72
MATCH	111.8	839.4250	839.4243	-0.89	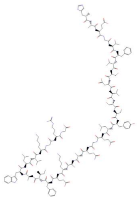	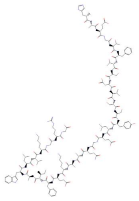	839.4250	839.4243	-0.89
MATCH	59.1	671.7389	671.7409	3.00	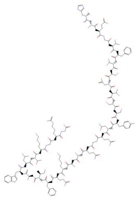	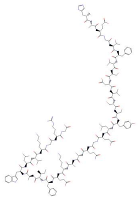	671.7389	671.7409	3.00

MS (+) FT

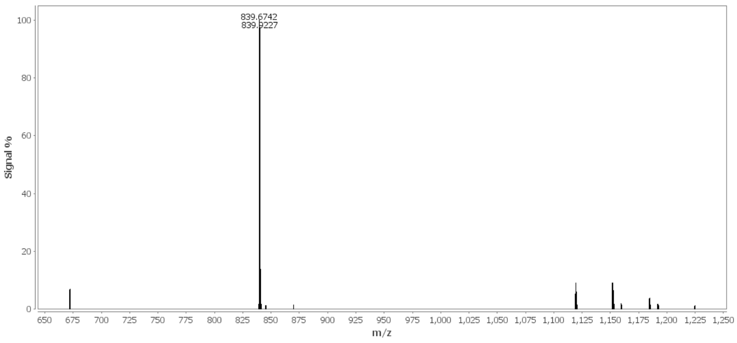

MS (+) FT

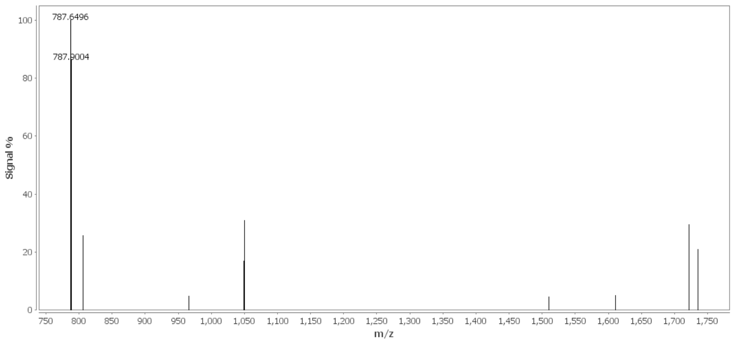

MS2 (+) FT activ = HCD:ce =

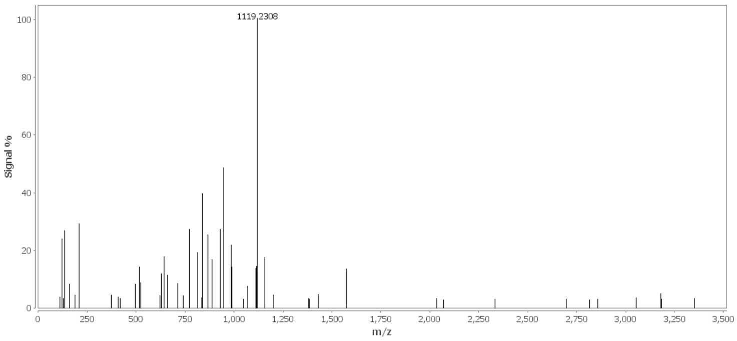

MS2 (+) FT activ = HCD:ce =

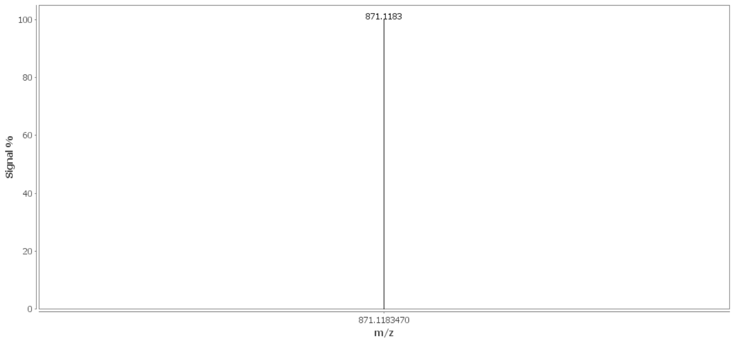

Metabolite: M1 -208 RT=10.37

Type	score	sub. m/z observed	sub. m/z calculated	sub ppm			met. m/z observed	met. m/z calculated	met. ppm
MATCH	59.1	671.7389	671.7409	3.00	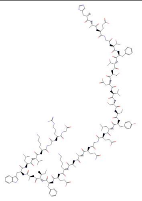	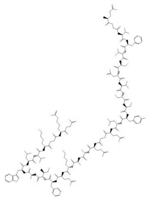	787.3984	787.4003	2.34
MATCH	59.1	671.7389	671.7409	3.00	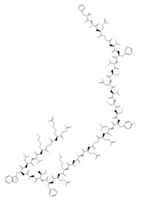	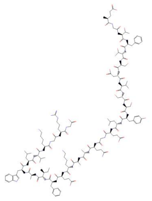	787.3984	787.4003	2.34
						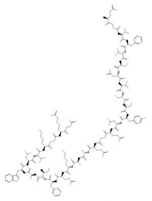	787.3984	787.4003	2.34
MATCH	20.8	671.7389	671.7409	3.00	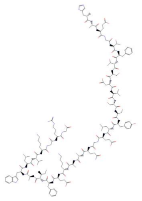	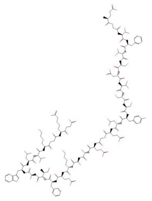	1049.5302	1049.5313	1.05
MATCH	20.8	671.7389	671.7409	3.00	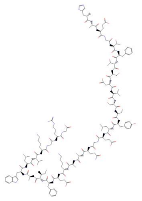	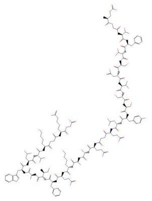	1049.5302	1049.5313	1.05

Metabolite: M1 -208 RT=10.37

Type	score	sub. m/z observed	sub. m/z calculated	sub ppm			met. m/z observed	met. m/z calculated	met. ppm
						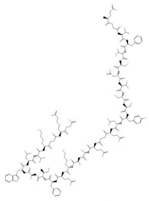	1049.5302	1049.5313	1.05
MATCH	111.8	839.4250	839.4243	-0.89	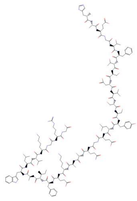	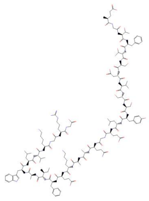	787.3984	787.4003	2.34
MATCH	111.8	839.4250	839.4243	-0.89	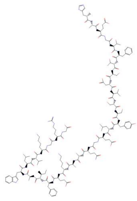	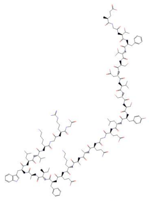	787.3984	787.4003	2.34
						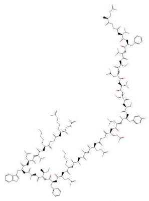	787.3984	787.4003	2.34
MATCH	73.5	839.4250	839.4243	-0.89	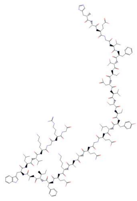	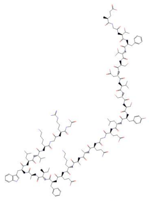	1049.5302	1049.5313	1.05
MATCH	73.5	839.4250	839.4243	-0.89	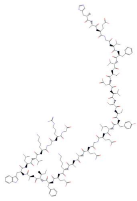	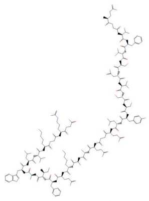	1049.5302	1049.5313	1.05
						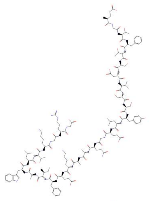	1049.5302	1049.5313	1.05
MATCH	60.3	1118.8936	1118.8966	2.72	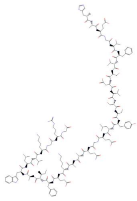	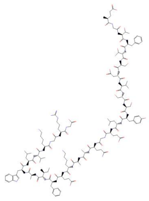	787.3984	787.4003	2.34
MATCH	60.3	1118.8936	1118.8966	2.72	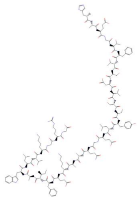	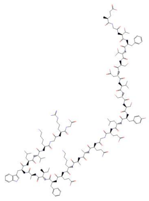	787.3984	787.4003	2.34

Metabolite: M1 -208 RT=10.37

Type	score	sub. m/z observed	sub. m/z calculated	sub ppm			met. m/z observed	met. m/z calculated	met. ppm
						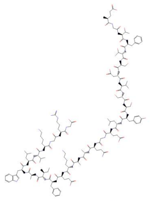	787.3984	787.4003	2.34
MATCH	22.1	1118.8936	1118.8966	2.72	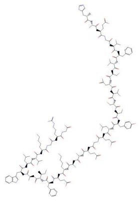	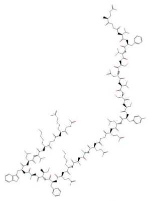	1049.5302	1049.5313	1.05
MATCH	22.1	1118.8936	1118.8966	2.72	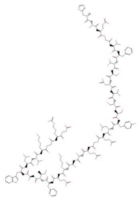	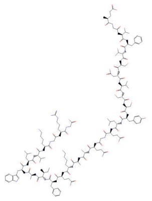	1049.5302	1049.5313	1.05
						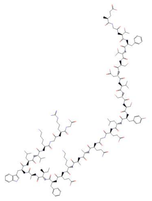	1049.5302	1049.5313	1.05
MET_MATCH						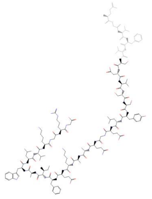	871.1183	871.1220	4.20
