# Supplementary material for: Software-aided approach to investigate peptide structure and metabolic susceptibility of amide bonds in peptide drugs based on high resolution mass spectrometry
Source: PLoS One. 2017 Nov 1;12(11):e0186461. doi: 10.1371/journal.pone.0186461 (PMC5665424; doi:10.1371/journal.pone.0186461)
Supplement: S1 File — (ZIP) [file pone.0186461.s007.zip › SFiles/S4_File.pdf]

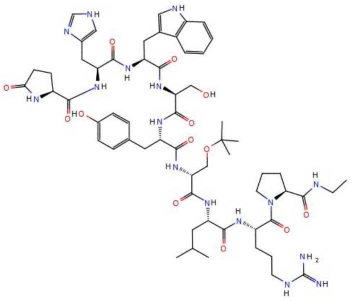

Buserelin

Property name	Property value
Time	0min, 5min, 15min, 45min, 120min
Instrument	ThermoQAPlus
Acquisition Mode	ddMS2
Matrix	pepsin

Chromatograms

Time=0min

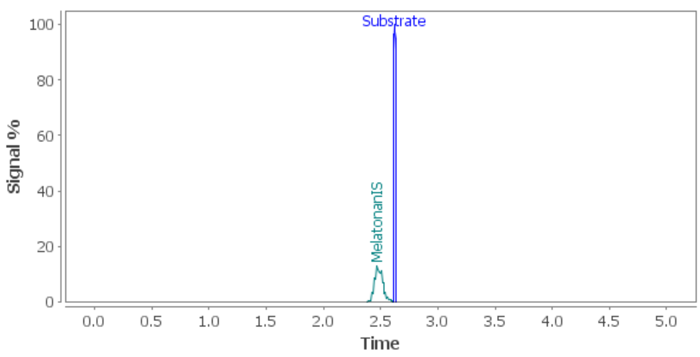

Time=5min

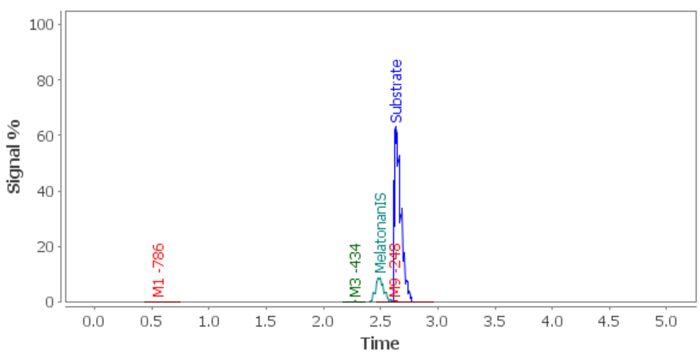

Time=15min

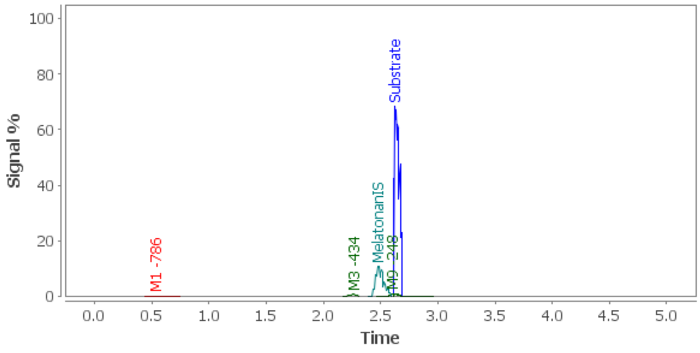

Time=45min

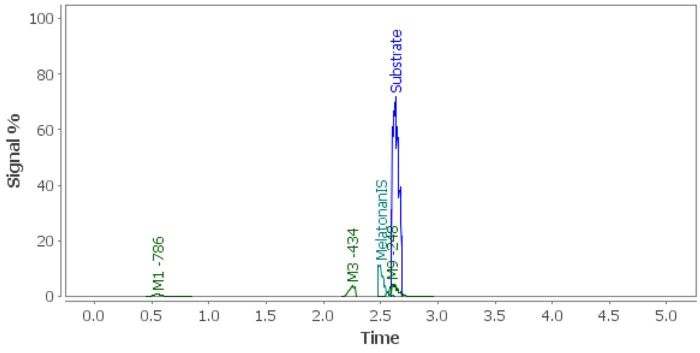

Time=120min

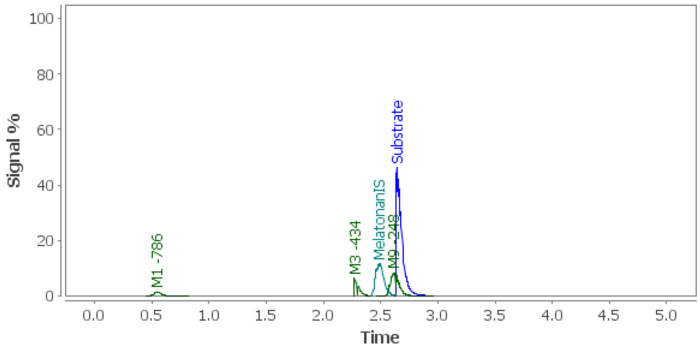

Custom Charts

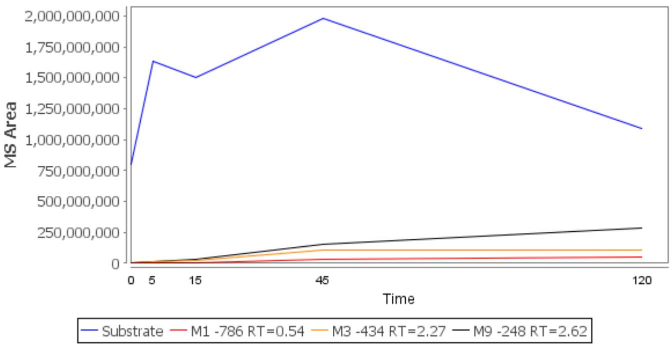

Fragmentation

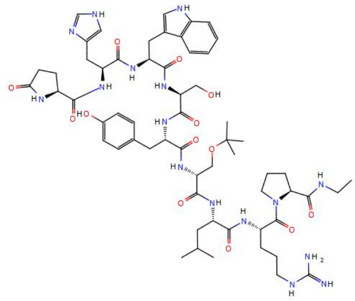

Buserelin

MS (+) FT

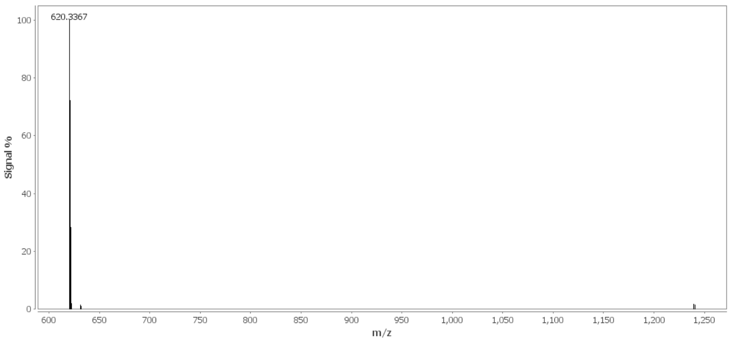

MS (+) FT

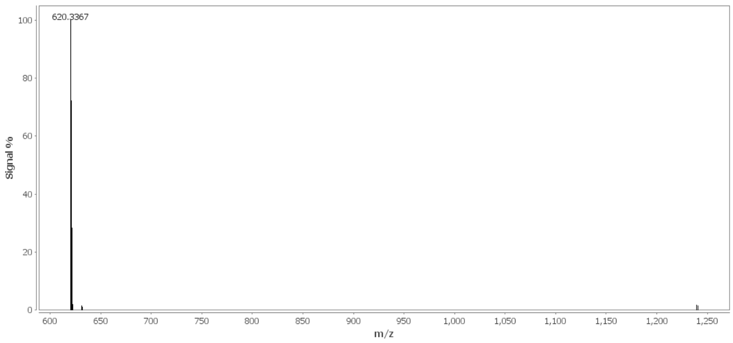

MS2 (+) FT activ = HCD:ce =

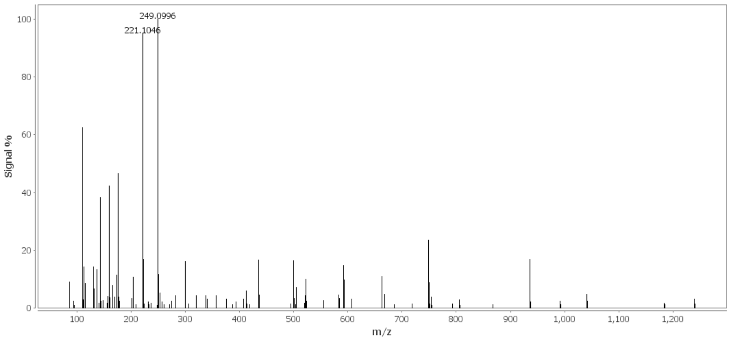

MS2 (+) FT activ = HCD:ce =

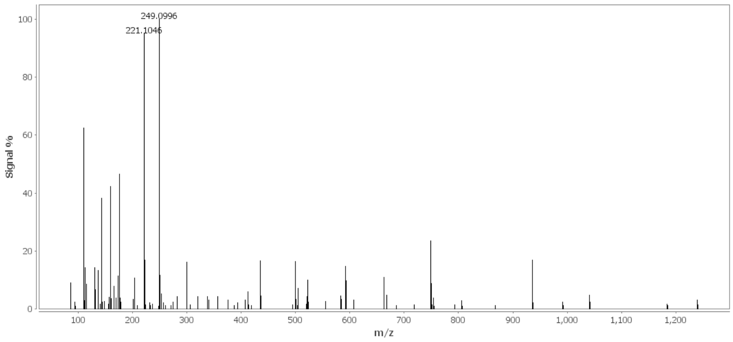

Metabolite: Substrate

Type	score	sub. m/z observed	sub. m/z calculated	sub ppm			met. m/z observed	met. m/z calculated	met. ppm
MATCH	12.4	1239.6673	1239.6633	-3.24	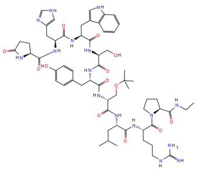	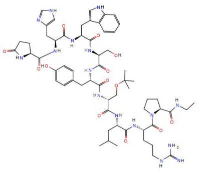	1239.6673	1239.6633	-3.24
MISMATCH	101.8	1239.6668	1239.6633	-2.84	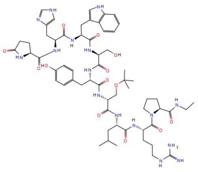	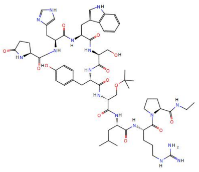	1239.6668	1239.6633	-2.84
MATCH	3.0	1183.5919	1183.6007	7.43	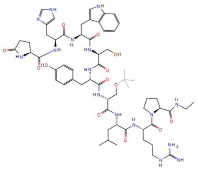	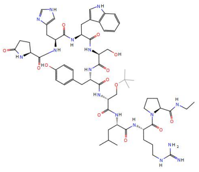	1183.5919	1183.6007	7.43

Metabolite: Substrate

Type	score	sub. m/z observed	sub. m/z calculated	sub ppm			met. m/z observed	met. m/z calculated	met. ppm
MATCH	6.8	754.2985	754.2944	-5.47	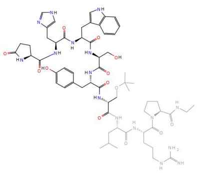	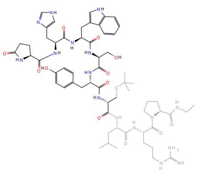	754.2985	754.2944	-5.47
MATCH	40.7	749.4348	749.4304	-5.77	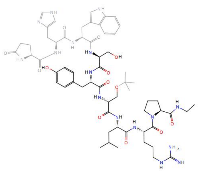	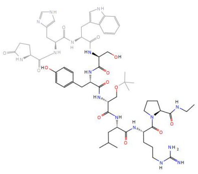	749.4348	749.4304	-5.77
MATCH	3.4	718.4613	718.4610	-0.32	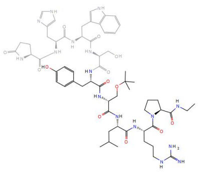	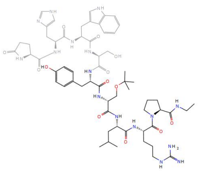	718.4613	718.4610	-0.32
MATCH	15.3	685.2747	685.2729	-2.63	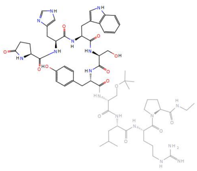	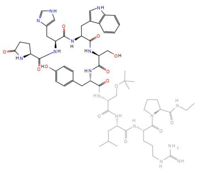	685.2747	685.2729	-2.63
MATCH	7.0	667.2648	667.2623	-3.69	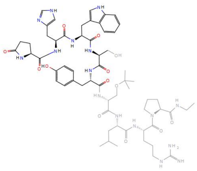	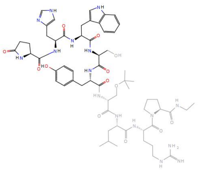	667.2648	667.2623	-3.69
MISMATCH	31.6	662.4030	662.3984	-6.99	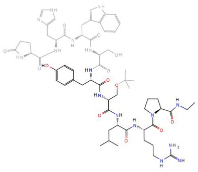	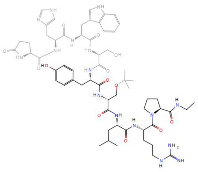	662.4030	662.3984	-6.99
MISMATCH	200.0	620.3367	620.3353	-2.21	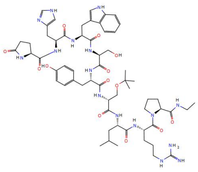	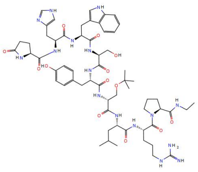	620.3367	620.3353	-2.21
MATCH	8.0	583.3020	583.2987	-5.68	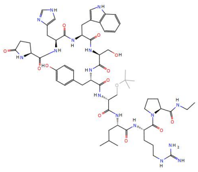	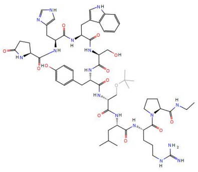	583.3020	583.2987	-5.68
MATCH	4.7	555.4016	555.3977	-7.06	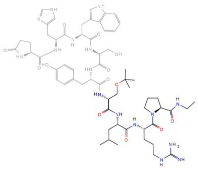	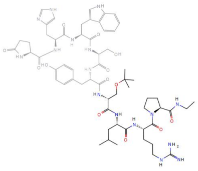	555.4016	555.3977	-7.06

Metabolite: Substrate

Type	score	sub. m/z observed	sub. m/z calculated	sub ppm			met. m/z observed	met. m/z calculated	met. ppm
MATCH	17.3	522.2114	522.2096	-3.59	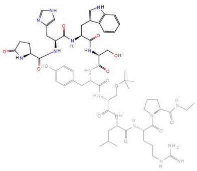	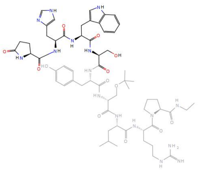	522.2114	522.2096	-3.59
MATCH	10.6	504.2021	504.1990	-6.21	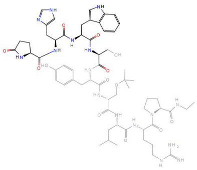	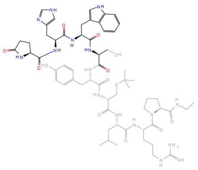	504.2021	504.1990	-6.21
MATCH	55.2	499.3384	499.3351	-6.61	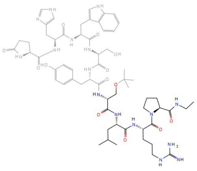	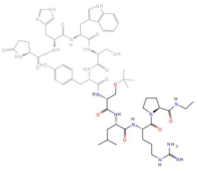	499.3384	499.3351	-6.61
MATCH	21.9	494.2170	494.2146	-4.83	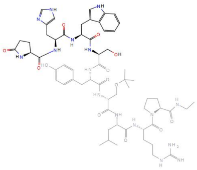	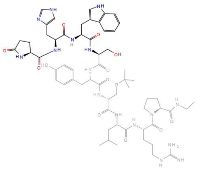	494.2170	494.2146	-4.83
MATCH	20.7	435.1801	435.1775	-5.81	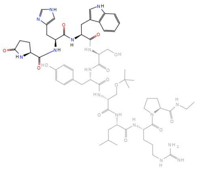	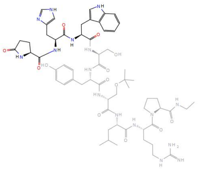	435.1801	435.1775	-5.81
MATCH	14.2	412.3056	412.3031	-6.05	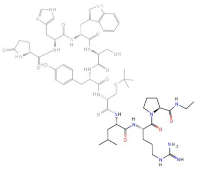	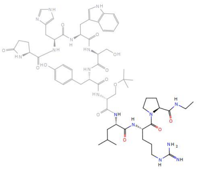	412.3056	412.3031	-6.05
MATCH	103.0	407.1846	407.1826	-4.83	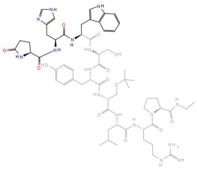	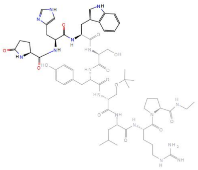	407.1846	407.1826	-4.83
MISMATCH	-7.3	320.1270	320.1353	26.04	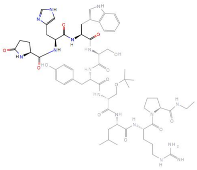	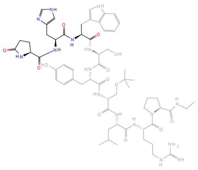	320.1270	320.1353	26.04
MATCH	31.2	299.2211	299.2190	-6.85	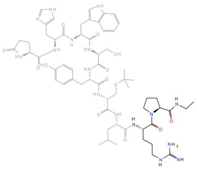	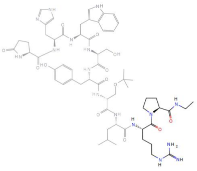	299.2211	299.2190	-6.85

Metabolite: Substrate

Type	score	sub. m/z observed	sub. m/z calculated	sub ppm			met. m/z observed	met. m/z calculated	met. ppm
MATCH	8.9	282.1940	282.1925	-5.44	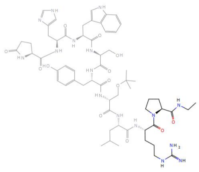	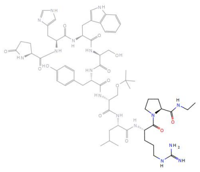	282.1940	282.1925	-5.44
MATCH	9.8	274.1199	274.1186	-4.53	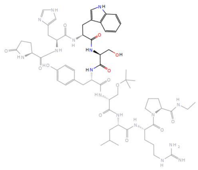	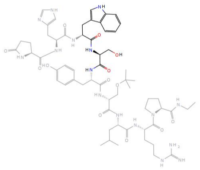	274.1199	274.1186	-4.53
MATCH	3.1	270.1935	270.1925	-4.03	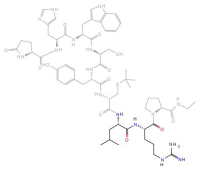	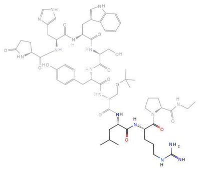	270.1935	270.1925	-4.03
MATCH	19.7	261.1156	261.1164	3.24	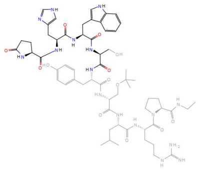	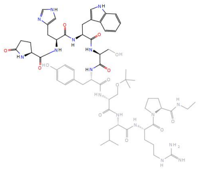	261.1156	261.1164	3.24
MATCH	13.2	257.0924	257.0921	-1.15	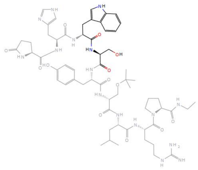	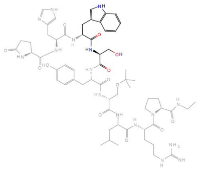	257.0924	257.0921	-1.15
MATCH	13.5	253.1674	253.1659	-5.92	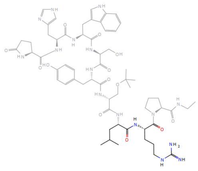	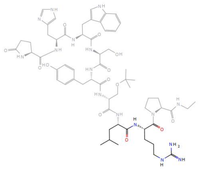	253.1674	253.1659	-5.92
MATCH	173.7	249.0996	249.0982	-5.62	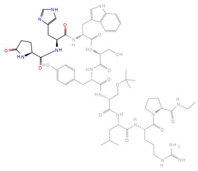	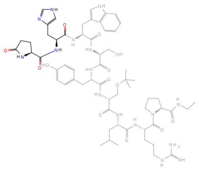	249.0996	249.0982	-5.62
MATCH	3.6	237.1357	237.1346	-4.59	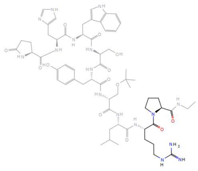	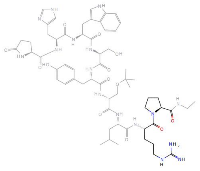	237.1357	237.1346	-4.59
MATCH	177.4	221.1046	221.1033	-5.85	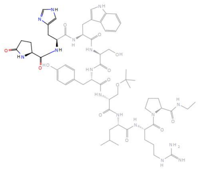	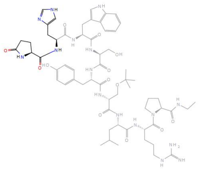	221.1046	221.1033	-5.85

Metabolite: Substrate

Type	score	sub. m/z observed	sub. m/z calculated	sub ppm			met. m/z observed	met. m/z calculated	met. ppm
MATCH	2.3	209.1413	209.1397	-7.49	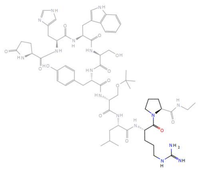	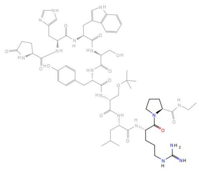	209.1413	209.1397	-7.49
MATCH	41.5	170.0609	170.0600	-5.22	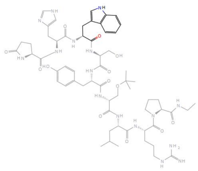	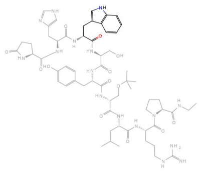	170.0609	170.0600	-5.22
MATCH	15.6	166.0622	166.0611	-6.61	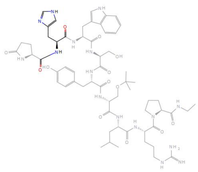	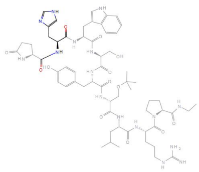	166.0622	166.0611	-6.61
MATCH	54.4	159.0926	159.0917	-5.97	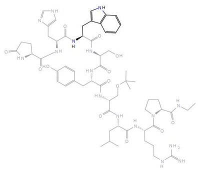	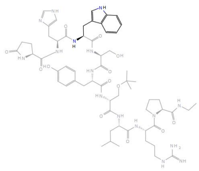	159.0926	159.0917	-5.97
MATCH	9.0	157.1093	157.1084	-5.94	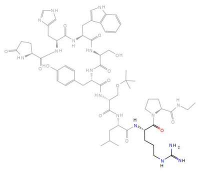	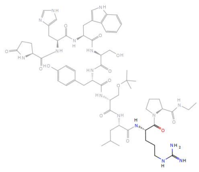	157.1093	157.1084	-5.94
MATCH	76.5	143.1188	143.1179	-6.54	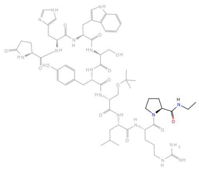	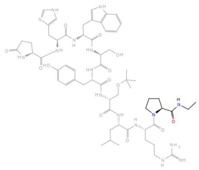	143.1188	143.1179	-6.54
MATCH	3.2	140.0827	140.0818	-5.85	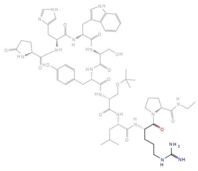	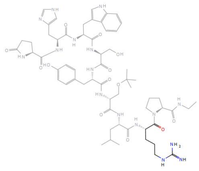	140.0827	140.0818	-5.85
MATCH	86.1	136.0766	136.0757	-6.56	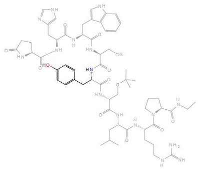	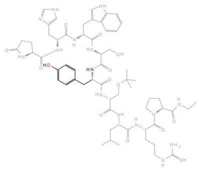	136.0766	136.0757	-6.56
MATCH	75.1	130.0660	130.0575	-65.5	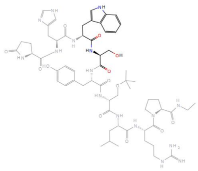	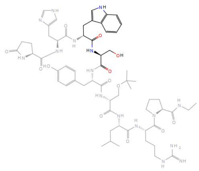	130.0660	130.0575	-65.5

Metabolite: Substrate

Type	score	sub. m/z observed	sub. m/z calculated	sub ppm			met. m/z observed	met. m/z calculated	met. ppm
MATCH	19.9	115.0876	115.0866	-8.54	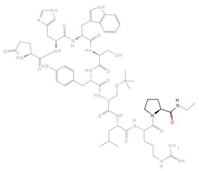	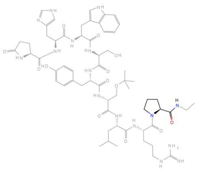	115.0876	115.0866	-8.54
MATCH	32.4	112.0880	112.0869	-9.33	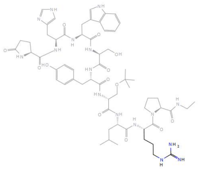	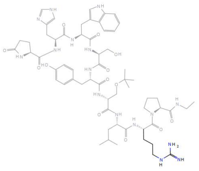	112.0880	112.0869	-9.33
MATCH	162.4	110.0723	110.0713	-9.43	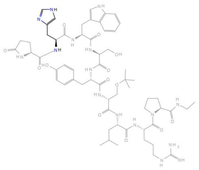	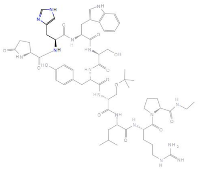	110.0723	110.0713	-9.43
MATCH	4.1	93.0457	93.0447	-10.5	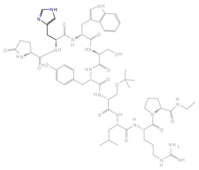	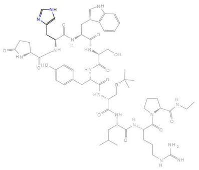	93.0457	93.0447	-10.5
MATCH	25.8	86.0976	86.0964	-13.6	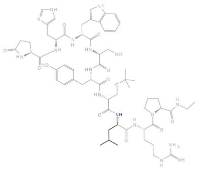	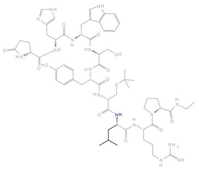	86.0976	86.0964	-13.6

MS (+) FT

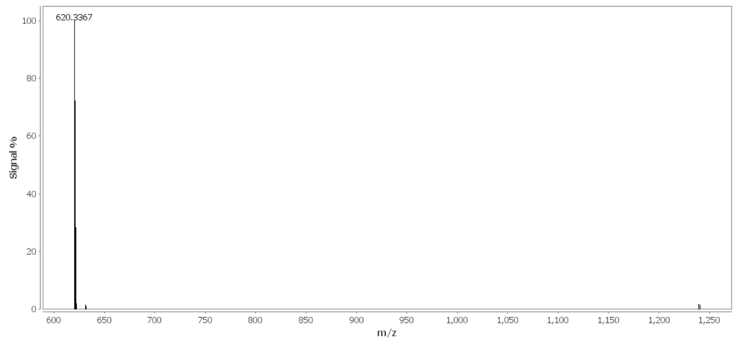

MS (+) FT

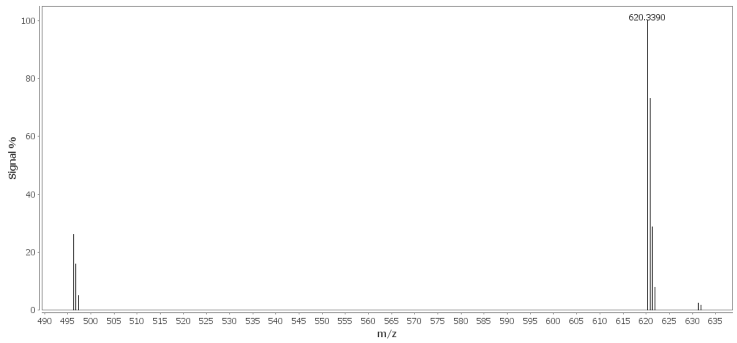

MS2 (+) FT activ = HCD:ce =

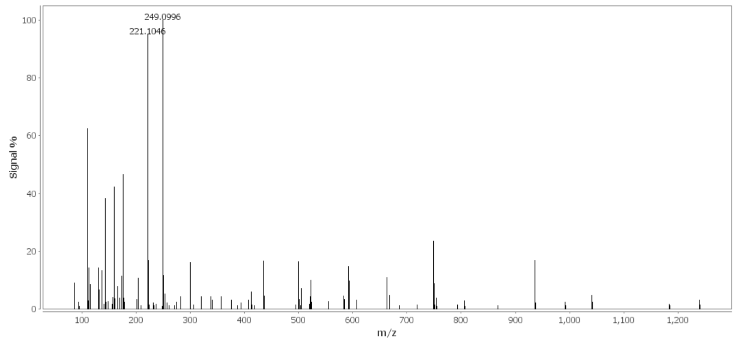

MS2 (+) FT activ = HCD:ce =

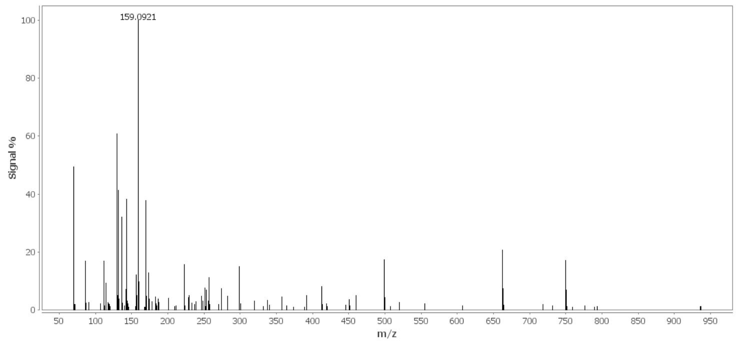

Metabolite: M9 -248 RT=2.62

Type	score	sub. m/z observed	sub. m/z calculated	sub ppm			met. m/z observed	met. m/z calculated	met. ppm
MATCH	126.2	620.3367	620.3353	-2.21	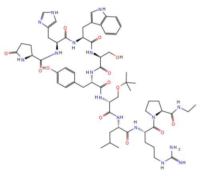	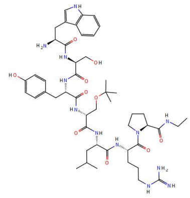	496.2927	496.2898	-5.82
						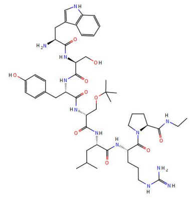	496.2927	496.2898	-5.82
MATCH	28.0	1239.6668	1239.6633	-2.84	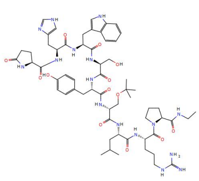	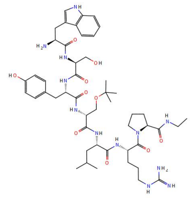	496.2927	496.2898	-5.82
						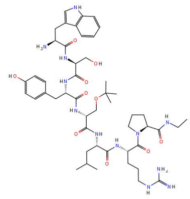	496.2927	496.2898	-5.82
MATCH	25.8	86.0976	86.0964	-13.6	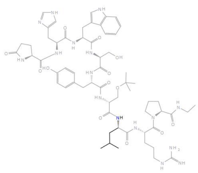	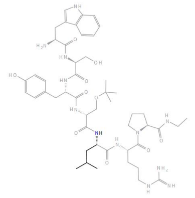	86.0973	86.0964	-9.94
MATCH	31.3	112.0880	112.0869	-9.33	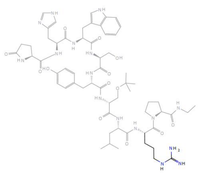	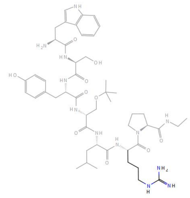	112.0876	112.0869	-5.97
MATCH	17.8	115.0876	115.0866	-8.54	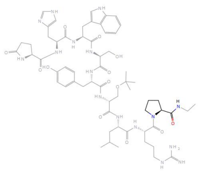	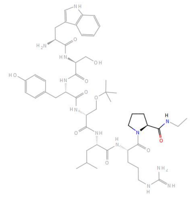	115.0872	115.0866	-5.01
MATCH	75.1	130.0660	130.0575	-65.5	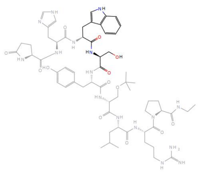	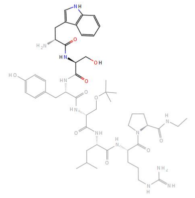	130.0656	130.0575	-61.9
MATCH	45.5	136.0766	136.0757	-6.56	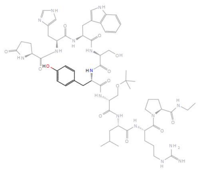	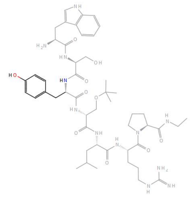	136.0761	136.0757	-3.09

Metabolite: M9 -248 RT=2.62

Type	score	sub. m/z observed	sub. m/z calculated	sub ppm			met. m/z observed	met. m/z calculated	met. ppm
MATCH	3.2	140.0827	140.0818	-5.85	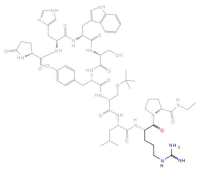	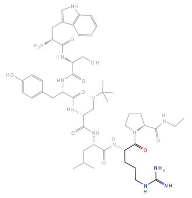	140.0821	140.0818	-1.60
MATCH	76.5	143.1188	143.1179	-6.54	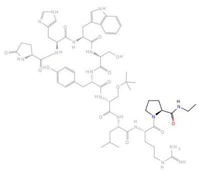	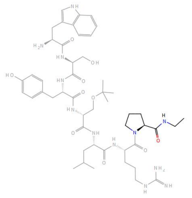	143.1183	143.1179	-2.98
MATCH	9.0	157.1093	157.1084	-5.94	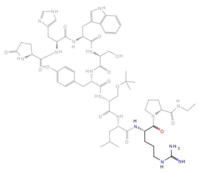	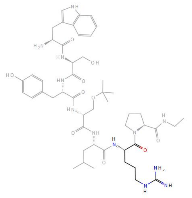	157.1087	157.1084	-2.30
MATCH	41.5	170.0609	170.0600	-5.22	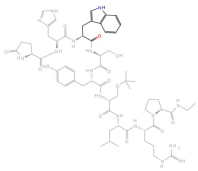	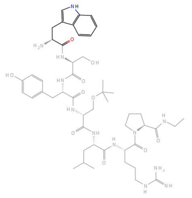	170.0604	170.0600	-1.99
MATCH	2.3	209.1413	209.1397	-7.49	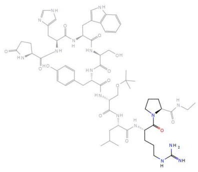	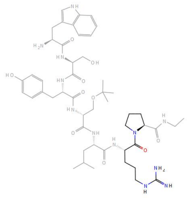	209.1403	209.1397	-3.09
MATCH	3.6	237.1357	237.1346	-4.59	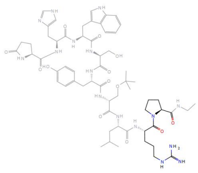	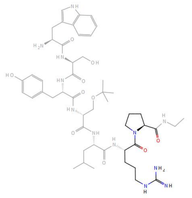	237.1352	237.1346	-2.56
MATCH	12.2	253.1674	253.1659	-5.92	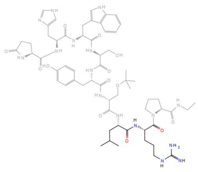	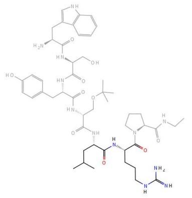	253.1663	253.1659	-1.71
MATCH	13.2	257.0924	257.0921	-1.15	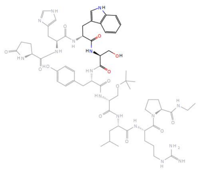	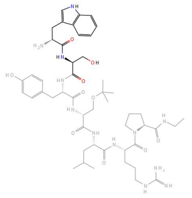	257.0922	257.0921	-0.60
MATCH	3.1	270.1935	270.1925	-4.03	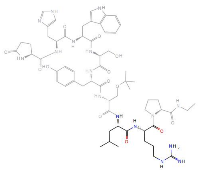	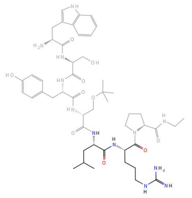	270.1935	270.1925	-3.81

Metabolite: M9 -248 RT=2.62

Type	score	sub. m/z observed	sub. m/z calculated	sub ppm			met. m/z observed	met. m/z calculated	met. ppm
MATCH	9.8	274.1199	274.1186	-4.53	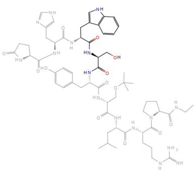	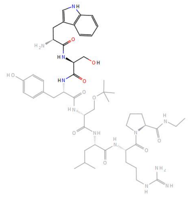	274.1189	274.1186	-1.13
						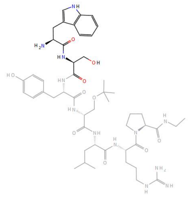	274.1189	274.1186	-1.13
MATCH	8.9	282.1940	282.1925	-5.44	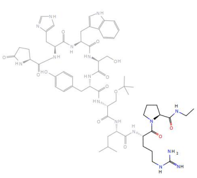	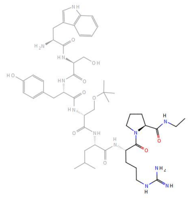	282.1929	282.1925	-1.60
MATCH	31.2	299.2211	299.2190	-6.85	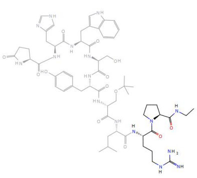	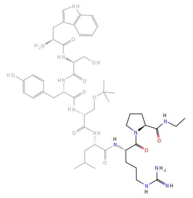	299.2196	299.2190	-2.12
MATCH	103.0	407.1846	407.1826	-4.83	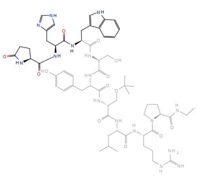	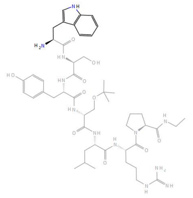	159.0921	159.0917	-2.50
MATCH	14.2	412.3056	412.3031	-6.05	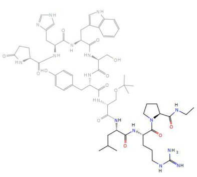	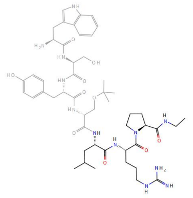	412.3043	412.3031	-3.05
MATCH	20.7	435.1801	435.1775	-5.81	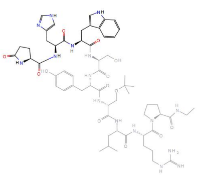	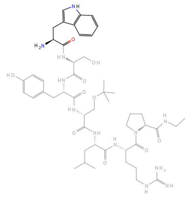	187.0867	187.0866	-0.68
MATCH	6.3	494.2170	494.2146	-4.83	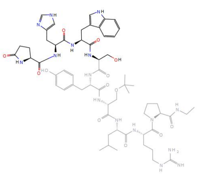	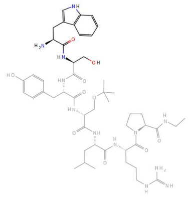	246.1244	246.1237	-2.73
MATCH	33.9	499.3384	499.3351	-6.61	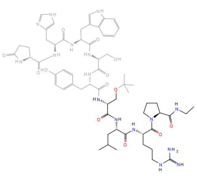	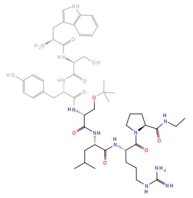	499.3363	499.3351	-2.36

Metabolite: M9 -248 RT=2.62

Type	score	sub. m/z observed	sub. m/z calculated	sub ppm			met. m/z observed	met. m/z calculated	met. ppm
MATCH	10.1	504.2021	504.1990	-6.21	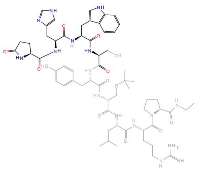	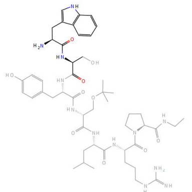	256.1088	256.1081	-3.02
MATCH	17.3	522.2114	522.2096	-3.59	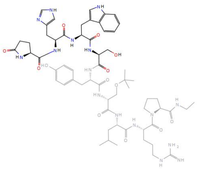	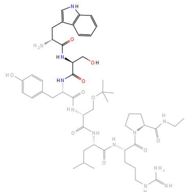	274.1189	274.1186	-1.13
						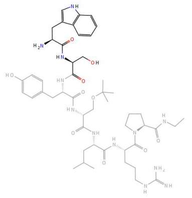	274.1189	274.1186	-1.13
MATCH	4.7	555.4016	555.3977	-7.06	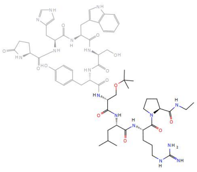	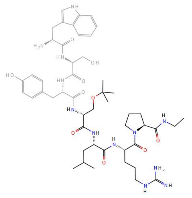	555.4023	555.3977	-8.37
MATCH	31.6	662.4030	662.3984	-6.99	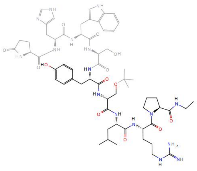	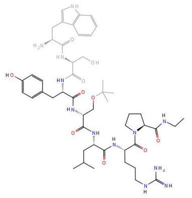	662.4001	662.3984	-2.60
MATCH	7.0	667.2648	667.2623	-3.69	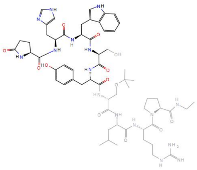	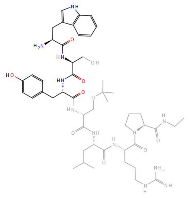	419.1735	419.1714	-5.03
MATCH	3.4	718.4613	718.4610	-0.32	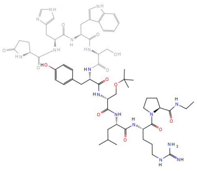	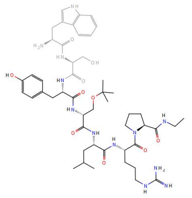	718.4691	718.4610	-11.2
MATCH	40.7	749.4348	749.4304	-5.77	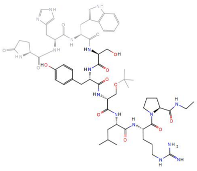	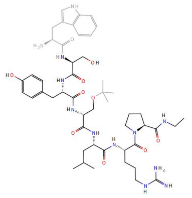	749.4320	749.4304	-2.08
MATCH	3.0	1183.5919	1183.6007	7.43	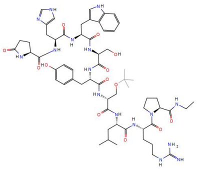	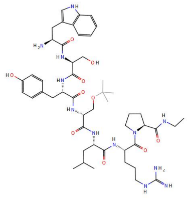	935.5015	935.5098	8.80

Metabolite: M9 -248 RT=2.62

Type	score	sub. m/z observed	sub. m/z calculated	sub ppm		met. m/z observed	met. m/z calculated	met. ppm
MISMATCH	-200.0	620.3367	620.3353	-2.21	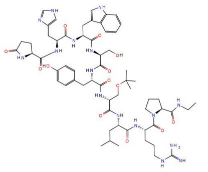	620.3390	620.3390	0.00
MISMATCH	-101.8	1239.6668	1239.6633	-2.84	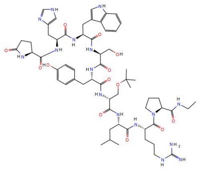	620.3390	620.3390	0.00
MISMATCH	-7.3	320.1270	320.1353	26.04	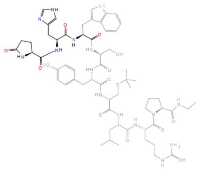	320.1243	320.1243	0.00
MISMATCH	-12.1	662.4030	662.3984	-6.99	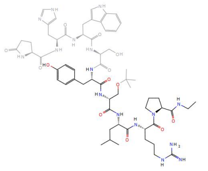	331.7030	331.7030	0.00

MS (+) FT

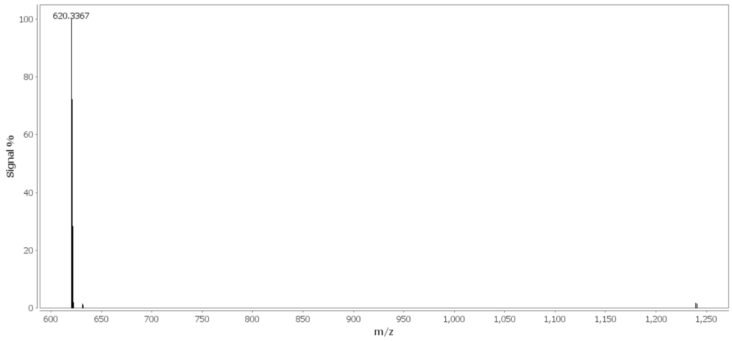

MS (+) FT

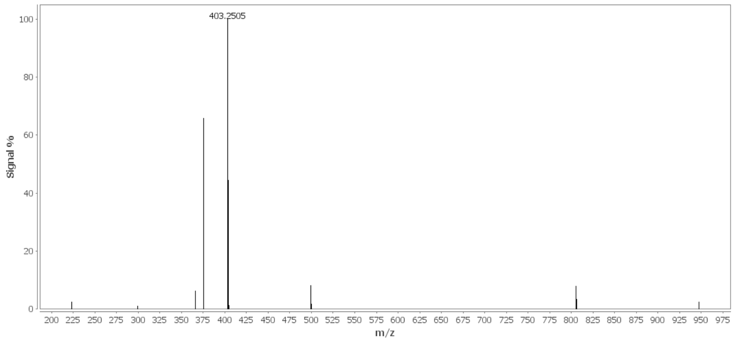

MS2 (+) FT activ = HCD:ce =

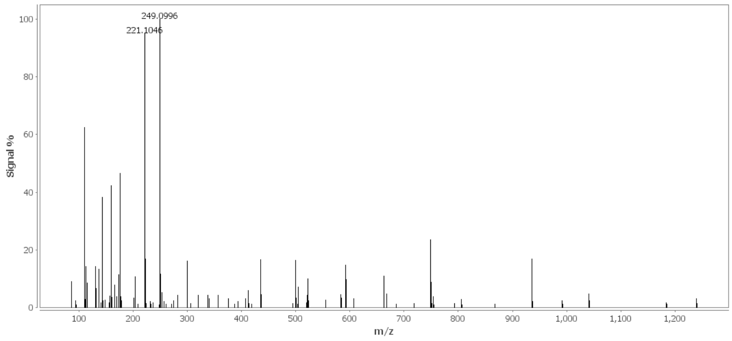

MS2 (+) FT activ = HCD:ce =

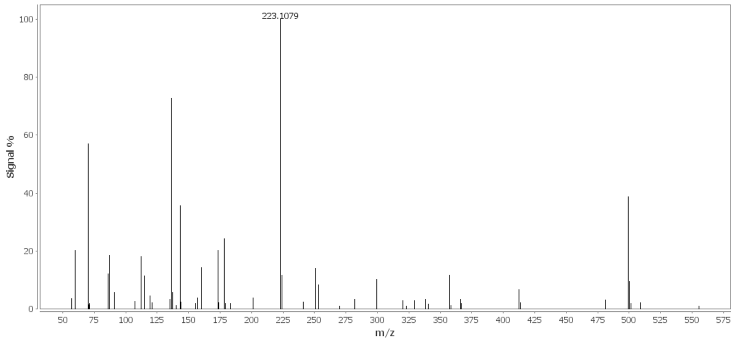

Metabolite: M3 -434 RT=2.27

Type	score	sub. m/z observed	sub. m/z calculated	sub ppm		met. m/z observed	met. m/z calculated	met. ppm

Metabolite: M3 -434 RT=2.27

Type	score	sub. m/z observed	sub. m/z calculated	sub ppm		met. m/z observed	met. m/z calculated	met. ppm
MATCH	200.0	620.3367	620.3353	-2.21	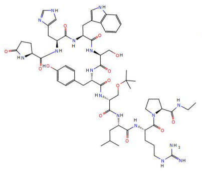 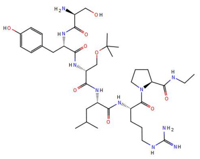	403.2505	403.2502	-0.75
MATCH	200.0	620.3367	620.3353	-2.21	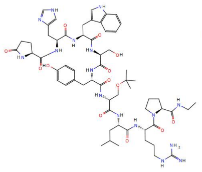 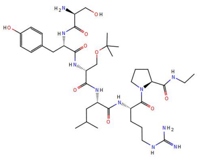	403.2505	403.2502	-0.75
MATCH	107.8	620.3367	620.3353	-2.21	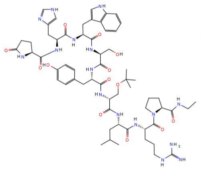 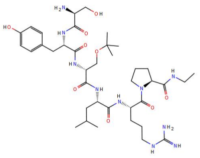	805.4929	805.4930	0.20
MATCH	107.8	620.3367	620.3353	-2.21	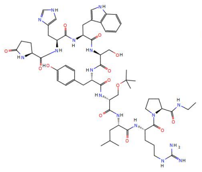 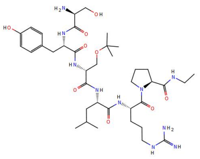	805.4929	805.4930	0.20
MATCH	101.8	1239.6668	1239.6633	-2.84	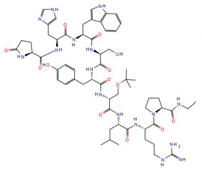 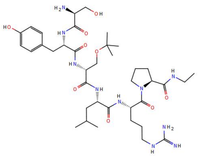	403.2505	403.2502	-0.75
MATCH	101.8	1239.6668	1239.6633	-2.84	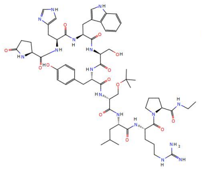 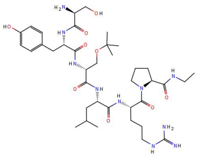	403.2505	403.2502	-0.75
MATCH	9.6	1239.6668	1239.6633	-2.84	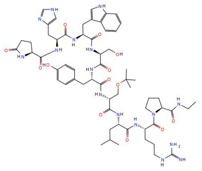 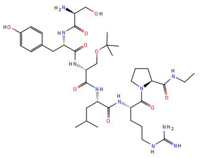	805.4929	805.4930	0.20
MATCH	9.6	1239.6668	1239.6633	-2.84	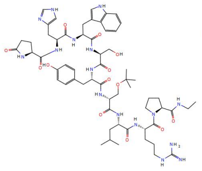 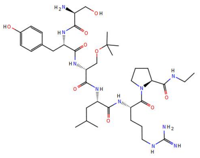	805.4929	805.4930	0.20
MATCH	21.2	86.0976	86.0964	-13.6	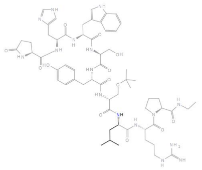 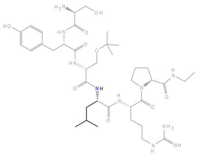	86.0972	86.0964	-8.79

Metabolite: M3 -434 RT=2.27

Type	score	sub. m/z observed	sub. m/z calculated	sub ppm			met. m/z observed	met. m/z calculated	met. ppm
MATCH	32.4	112.0880	112.0869	-9.33	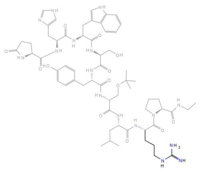	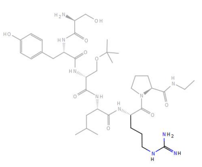	112.0874	112.0869	-4.20
MATCH	19.9	115.0876	115.0866	-8.54	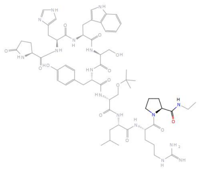	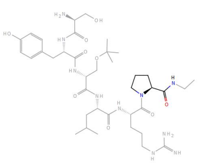	115.0872	115.0866	-4.89
MATCH	86.1	136.0766	136.0757	-6.56	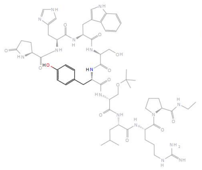	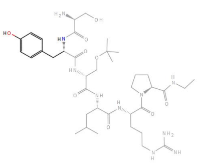	136.0760	136.0757	-1.92
MATCH	2.7	140.0827	140.0818	-5.85	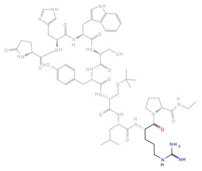	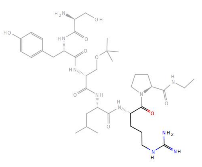	140.0821	140.0818	-1.71
MATCH	73.8	143.1188	143.1179	-6.54	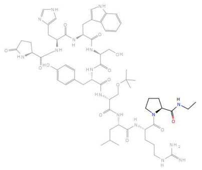	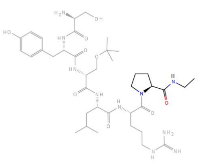	143.1182	143.1179	-1.97
MATCH	7.8	157.1093	157.1084	-5.94	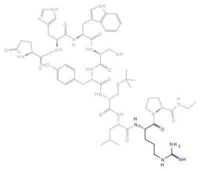	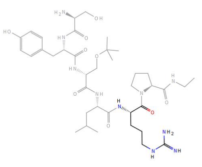	157.1088	157.1084	-2.33
MATCH	13.5	253.1674	253.1659	-5.92	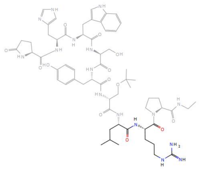	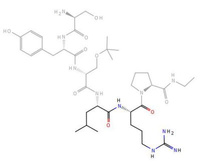	253.1667	253.1659	-3.31
MATCH	19.7	261.1156	261.1164	3.24	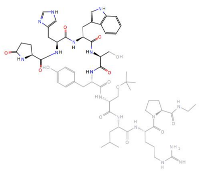	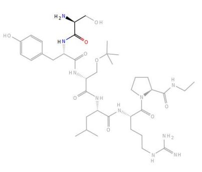	87.0560	87.0553	-8.59
MATCH	2.3	270.1935	270.1925	-4.03	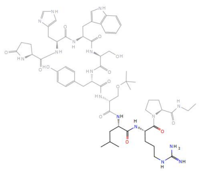	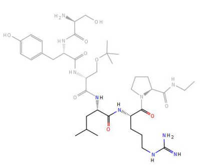	270.1922	270.1925	1.04

Metabolite: M3 -434 RT=2.27

Type	score	sub. m/z observed	sub. m/z calculated	sub ppm			met. m/z observed	met. m/z calculated	met. ppm
MATCH	7.5	282.1940	282.1925	-5.44	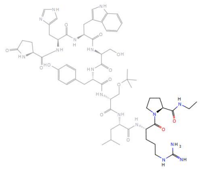	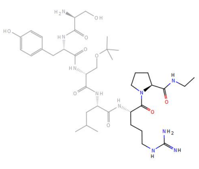	282.1926	282.1925	-0.44
MATCH	26.5	299.2211	299.2190	-6.85	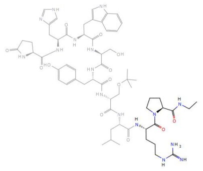	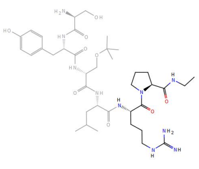	299.2191	299.2190	-0.18
MATCH	12.6	412.3056	412.3031	-6.05	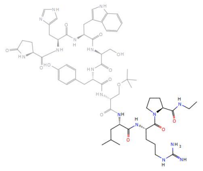	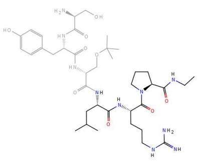	412.3034	412.3031	-0.90
MATCH	21.9	494.2170	494.2146	-4.83	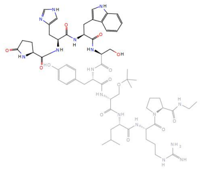	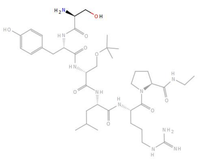	60.0453	60.0444	-15.8
MATCH	55.2	499.3384	499.3351	-6.61	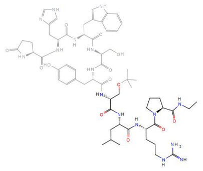	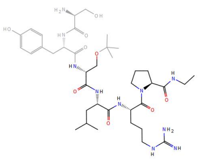	499.3361	499.3351	-2.06
MATCH	10.6	504.2021	504.1990	-6.21	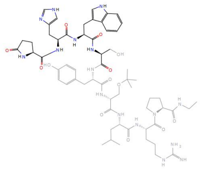	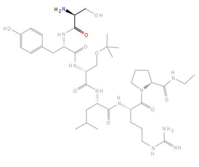	70.0297	70.0287	-13.3
MATCH	3.5	555.4016	555.3977	-7.06	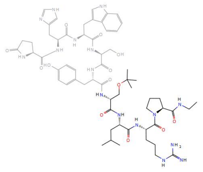	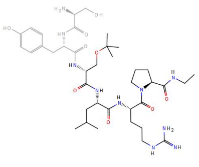	555.3928	555.3977	8.77
MATCH	8.0	583.3020	583.2987	-5.68	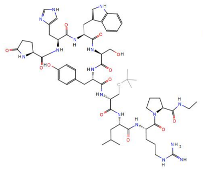	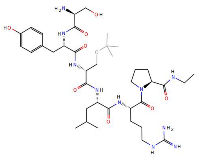	366.2137	366.2136	-0.37
MATCH	15.3	685.2747	685.2729	-2.63	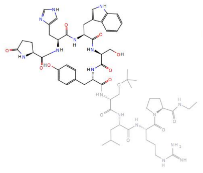	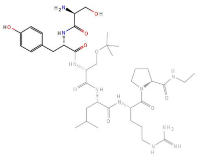	251.1030	251.1026	-1.33

Metabolite: M3 -434 RT=2.27

Type	score	sub. m/z observed	sub. m/z calculated	sub ppm		met. m/z observed	met. m/z calculated	met. ppm
MATCH	6.8	754.2985	754.2944	-5.47	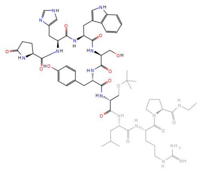 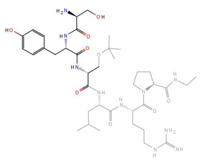	320.1250	320.1241	-2.68
MET_MATCH					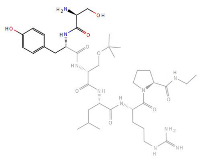	223.1080	223.1077	-1.46
MET_MATCH					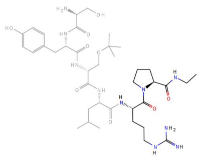	299.2200	299.2190	-3.49
MET_MATCH					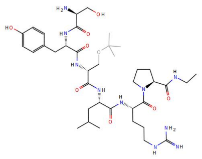	366.2146	366.2136	-2.71
MET_MATCH					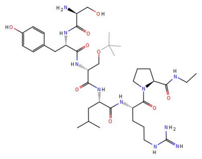	375.2192	375.2189	-0.88
MET_MATCH					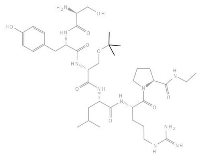	57.0708	57.0699	-15.5
MET_MATCH					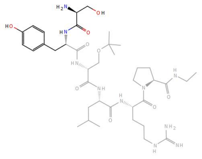	223.1079	223.1077	-0.67

MS (+) FT

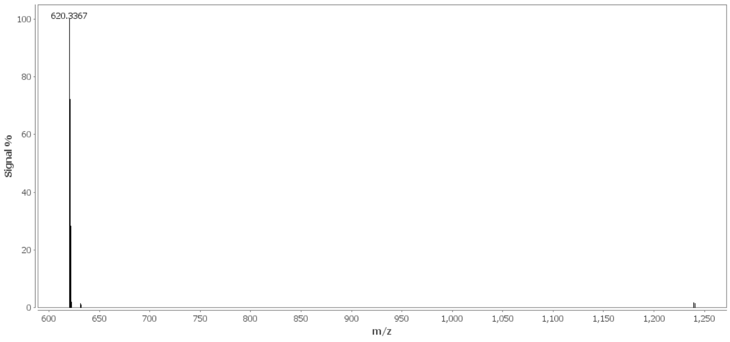

MS (+) FT

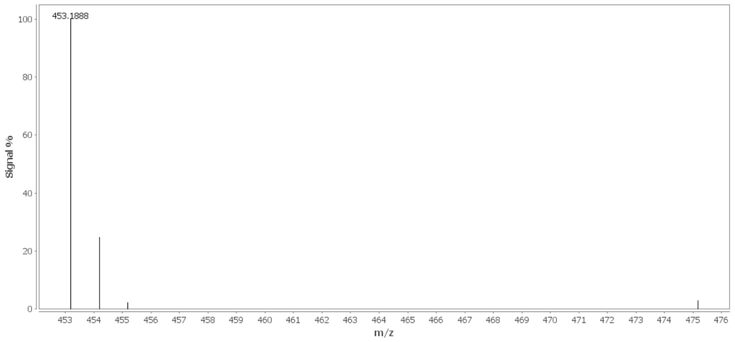

MS2 (+) FT activ = HCD:ce =

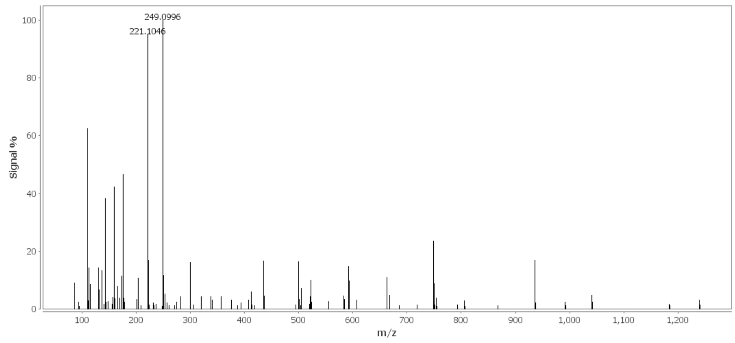

MS2 (+) FT activ = HCD:ce =

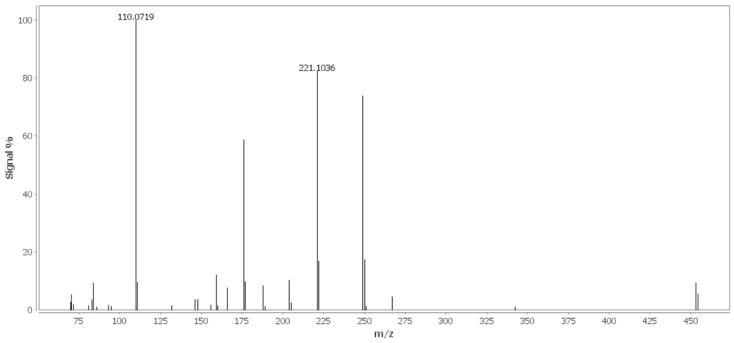

Metabolite: M1 -786 RT=0.54

Type	score	sub. m/z observed	sub. m/z calculated	sub ppm		met. m/z observed	met. m/z calculated	met. ppm
MATCH	200.0	620.3367	620.3353	-2.21	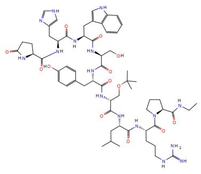 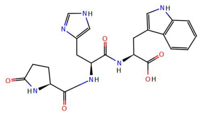	453.1888	453.1881	-1.51
					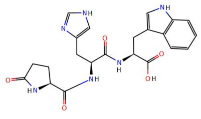	453.1888	453.1881	-1.51
MATCH	101.8	1239.6668	1239.6633	-2.84	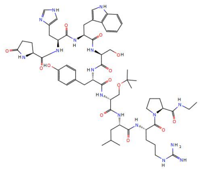 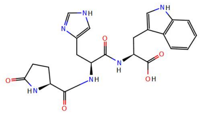	453.1888	453.1881	-1.51
					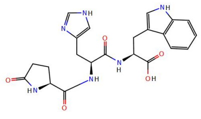	453.1888	453.1881	-1.51
MATCH	4.1	93.0457	93.0447	-10.5	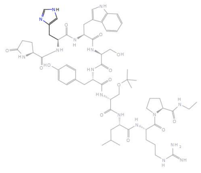 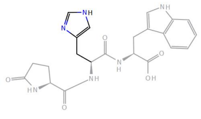	93.0454	93.0447	-7.60
MATCH	2.3	95.0616	95.0604	-12.8	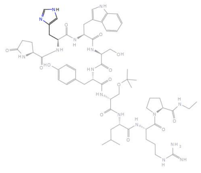 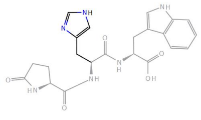	95.0610	95.0604	-6.91
MATCH	162.4	110.0723	110.0713	-9.43	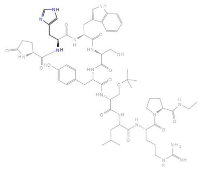 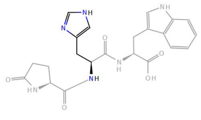	110.0719	110.0713	-5.41

Metabolite: M1 -786 RT=0.54

Type	score	sub. m/z observed	sub. m/z calculated	sub ppm			met. m/z observed	met. m/z calculated	met. ppm
MATCH	54.4	159.0926	159.0917	-5.97	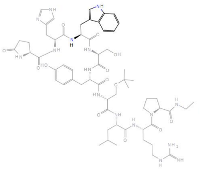	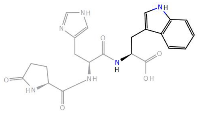	159.0919	159.0917	-1.19
MATCH	15.6	166.0622	166.0611	-6.61	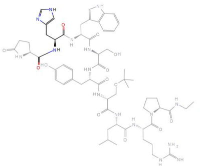	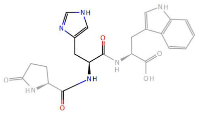	166.0614	166.0611	-1.55
MATCH	177.4	221.1046	221.1033	-5.85	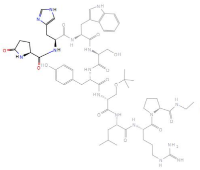	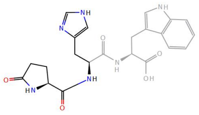	221.1036	221.1033	-1.42
MATCH	173.7	249.0996	249.0982	-5.62	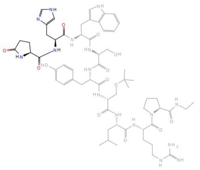	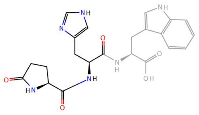	249.0986	249.0982	-1.47
MATCH	12.4	1239.6673	1239.6633	-3.24	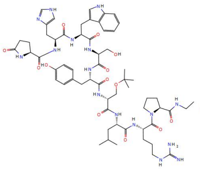	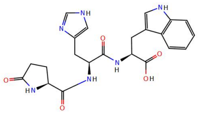	453.1887	453.1881	-1.26
MET_MATCH						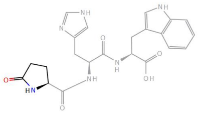	84.0453	84.0444	-10.4
MET_MATCH						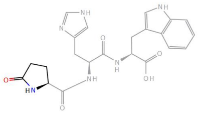	86.0608	86.0600	-8.83
MET_MATCH						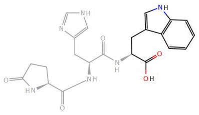	188.0708	188.0706	-0.96
MET_MATCH						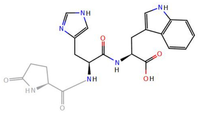	342.1552	342.1561	2.40
