# Supplementary material for: Software-aided approach to investigate peptide structure and metabolic susceptibility of amide bonds in peptide drugs based on high resolution mass spectrometry
Source: PLoS One. 2017 Nov 1;12(11):e0186461. doi: 10.1371/journal.pone.0186461 (PMC5665424; doi:10.1371/journal.pone.0186461)
Supplement: S1 File — (ZIP) [file pone.0186461.s007.zip › SFiles/S50_File.pdf]

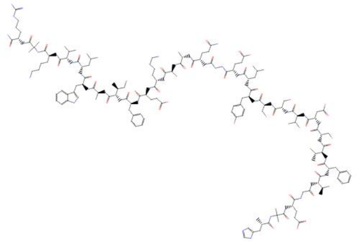

Taspoglutide

Property name	Property value
Time	0min, 2min, 4min, 24min
Instrument	ddMS2
Matrix	NEP

Chromatograms

Time=0min

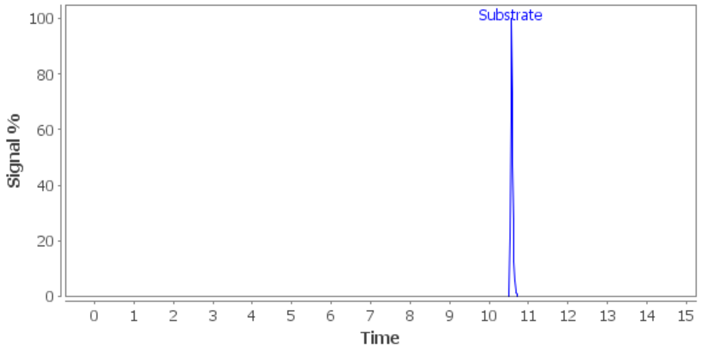

Time=2min

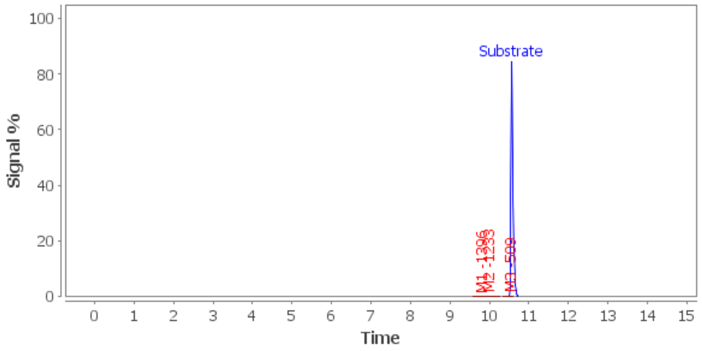

Time=4min

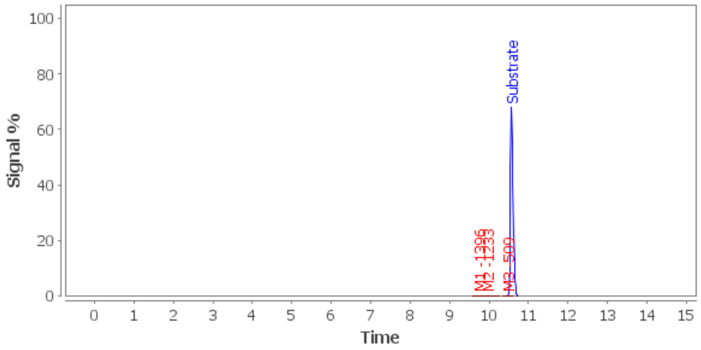

Time=24min

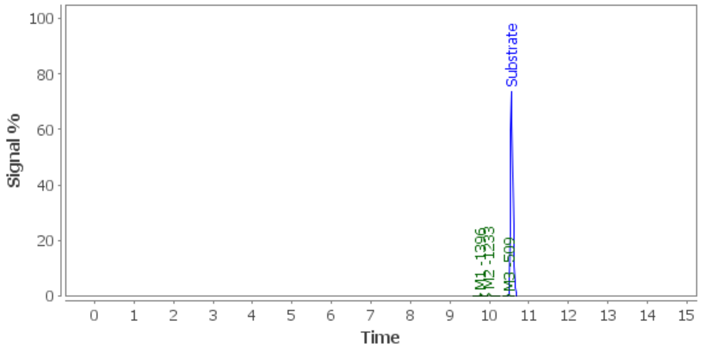

Custom Charts

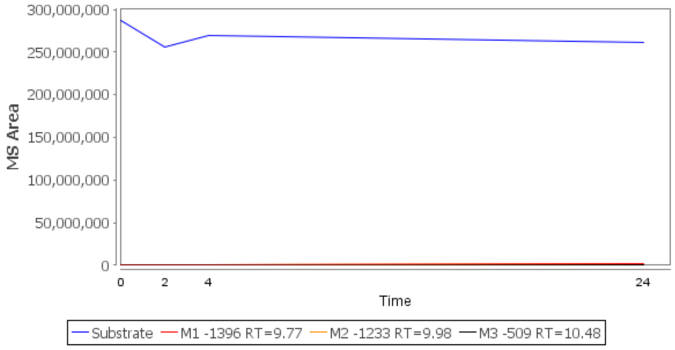

Fragmentation

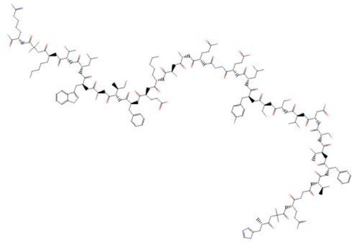

Taspoglutide

MS (+) FT

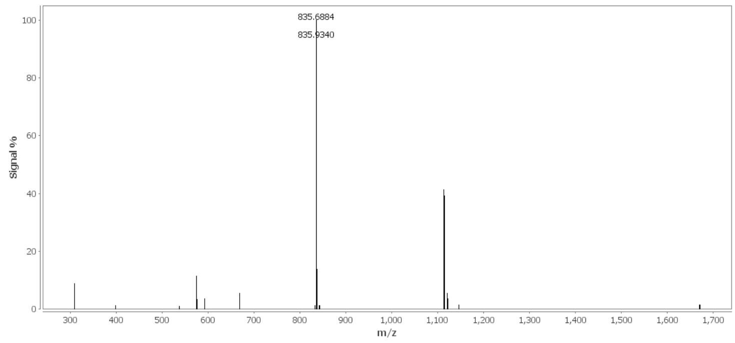

MS (+) FT

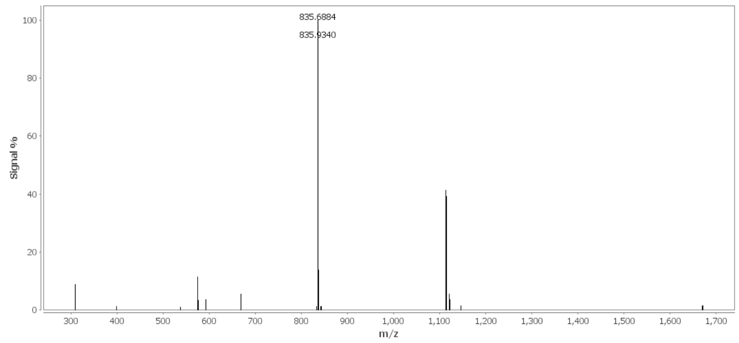

MS2 (+) FT activ = HCD:ce =

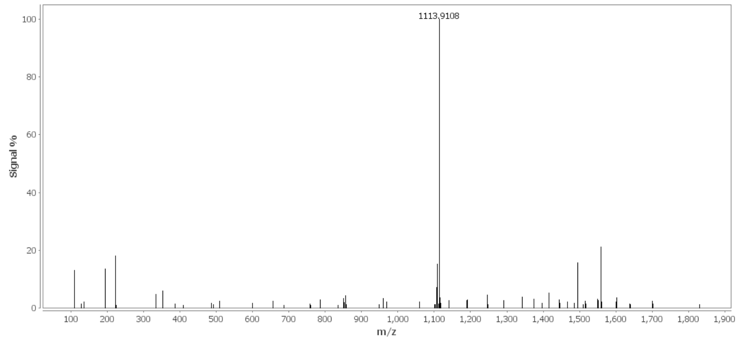

MS2 (+) FT activ = HCD:ce =

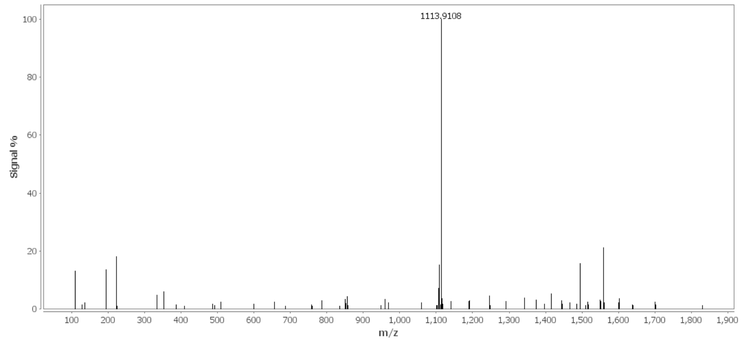

Metabolite: Substrate

Type	score	sub. m/z observed	sub. m/z calculated	sub ppm			met. m/z observed	met. m/z calculated	met. ppm
MATCH	20.2	1638.7260	1638.7282	1.36	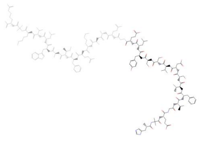	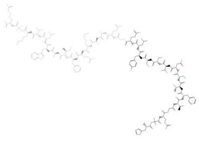	1638.7260	1638.7282	1.36
MATCH	158.7	1113.5781	1113.5771	-0.85	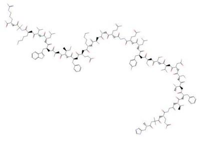	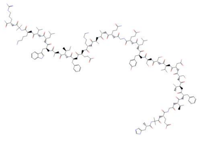	1113.5781	1113.5771	-0.85
MATCH	111.7	1113.5778	1113.5771	-0.63	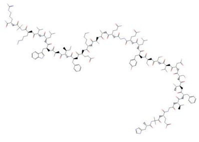	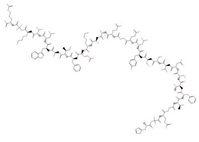	1113.5778	1113.5771	-0.63

Metabolite: Substrate

Type	score	sub. m/z observed	sub. m/z calculated	sub ppm			met. m/z observed	met. m/z calculated	met. ppm
MATCH	48.4	851.0007	851.0016	1.08	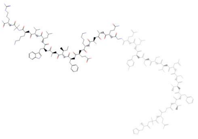	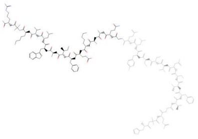	851.0007	851.0016	1.08
MATCH	144.5	835.4415	835.4347	-8.23	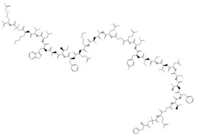	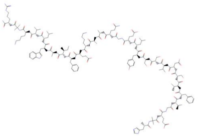	835.4415	835.4347	-8.23
MATCH	18.6	785.5137	785.5145	0.99	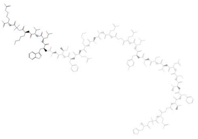	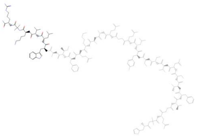	785.5137	785.5145	0.99
MATCH	92.5	668.5489	668.5492	0.36	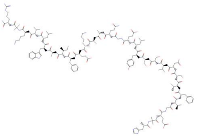	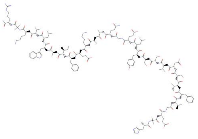	668.5489	668.5492	0.36
MATCH	42.6	599.4353	599.4351	-0.27	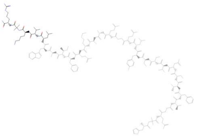	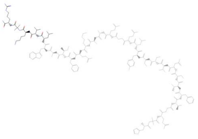	599.4353	599.4351	-0.27
MATCH	43.0	486.3514	486.3511	-0.71	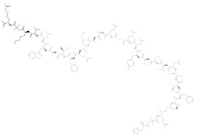	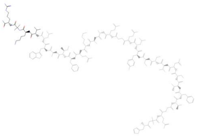	486.3514	486.3511	-0.71

MS (+) FT

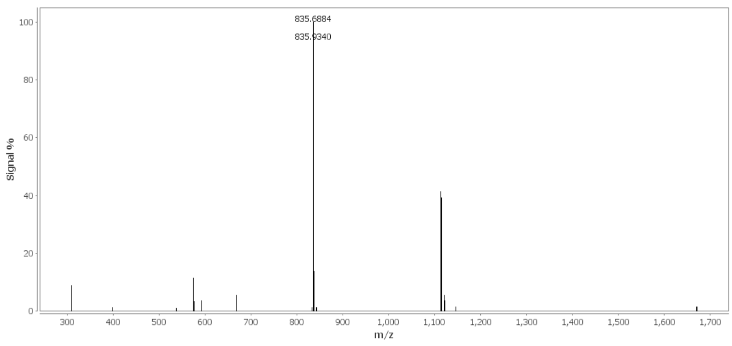

MS (+) FT

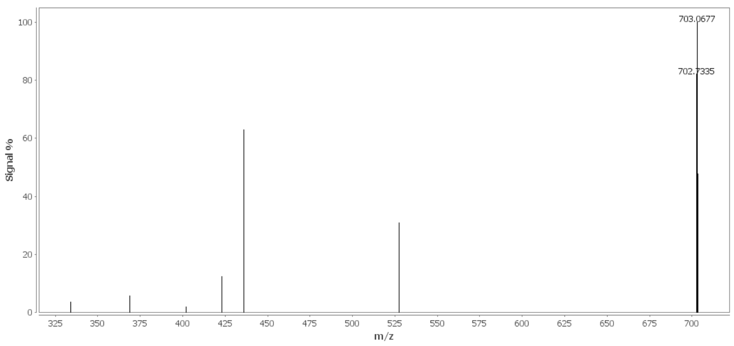

MS2 (+) FT activ = HCD:ce =

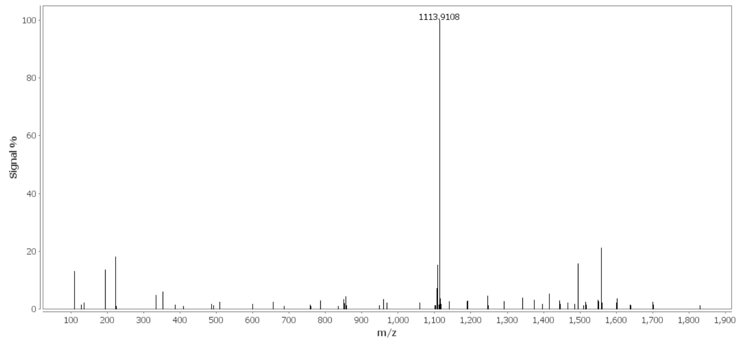

MS2 (+) FT activ = HCD:ce =

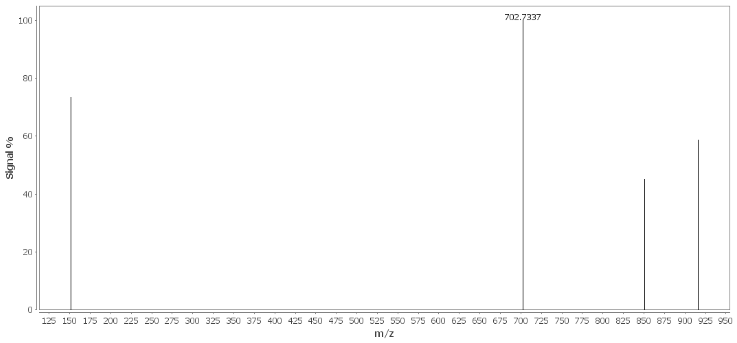

Metabolite: M2 -1233 RT=9.98

Type	score	sub. m/z observed	sub. m/z calculated	sub ppm		met. m/z observed	met. m/z calculated	met. ppm
MATCH	33.7	668.5489	668.5492	0.36	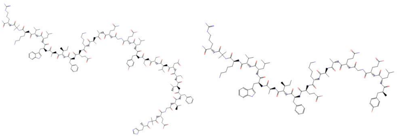	527.3022	527.3019	-0.57
MATCH	33.7	668.5489	668.5492	0.36	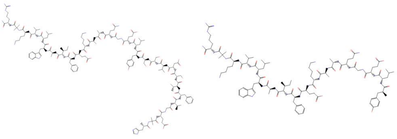	527.3022	527.3019	-0.57
					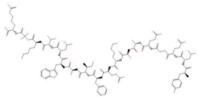	527.3022	527.3019	-0.57
MATCH	84.8	668.5489	668.5492	0.36	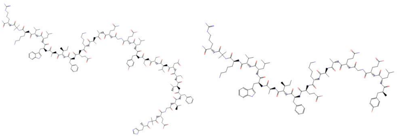	702.7335	702.7335	0.00
MATCH	84.8	668.5489	668.5492	0.36	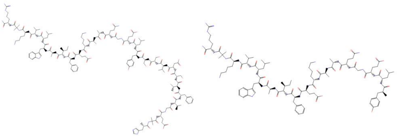	702.7335	702.7335	0.00
					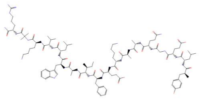	702.7335	702.7335	0.00
MATCH	85.7	835.4415	835.4347	-8.23	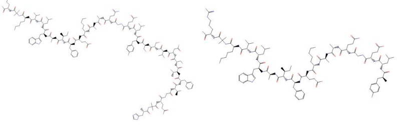	527.3022	527.3019	-0.57

Metabolite: M2 -1233 RT=9.98

Type	score	sub. m/z observed	sub. m/z calculated	sub ppm		met. m/z observed	met. m/z calculated	met. ppm
MATCH	85.7	835.4415	835.4347	-8.23	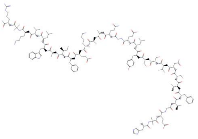 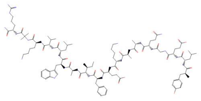	527.3022	527.3019	-0.57
					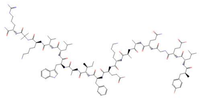	527.3022	527.3019	-0.57
MATCH	136.8	835.4415	835.4347	-8.23	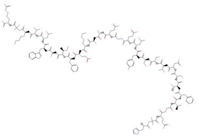 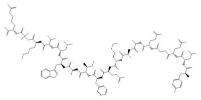	702.7335	702.7335	0.00
MATCH	136.8	835.4415	835.4347	-8.23	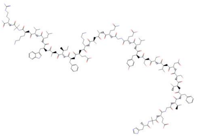 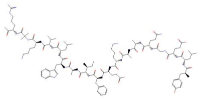	702.7335	702.7335	0.00
					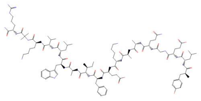	702.7335	702.7335	0.00
MATCH	52.9	1113.5778	1113.5771	-0.63	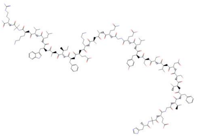 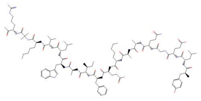	527.3022	527.3019	-0.57
MATCH	52.9	1113.5778	1113.5771	-0.63	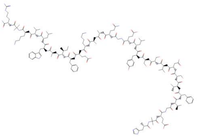 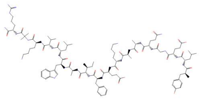	527.3022	527.3019	-0.57
					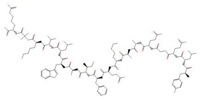	527.3022	527.3019	-0.57
MATCH	104.0	1113.5778	1113.5771	-0.63	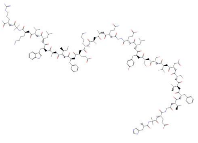 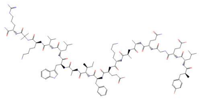	702.7335	702.7335	0.00

Metabolite: M2 -1233 RT=9.98

Type	score	sub. m/z observed	sub. m/z calculated	sub ppm			met. m/z observed	met. m/z calculated	met. ppm
MATCH	104.0	1113.5778	1113.5771	-0.63	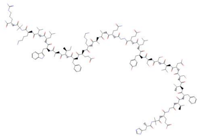	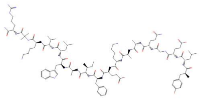	702.7335	702.7335	0.00
						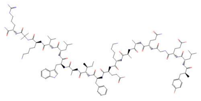	702.7335	702.7335	0.00
MATCH	48.4	851.0007	851.0016	1.08	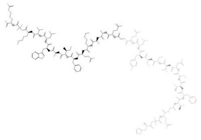	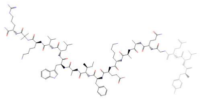	850.9999	851.0016	1.99
MATCH	158.7	1113.5781	1113.5771	-0.85	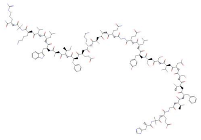	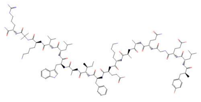	702.7337	702.7335	-0.34

MS (+) FT

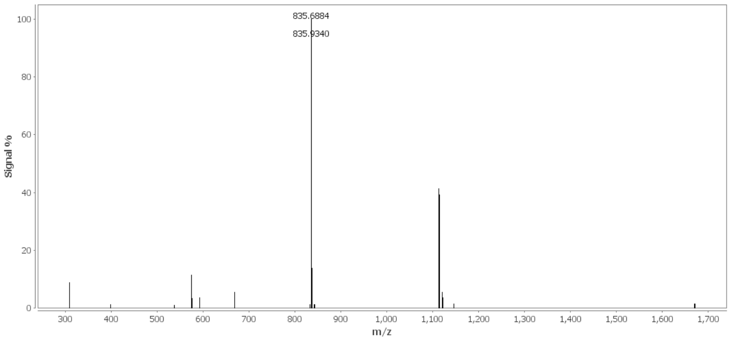

MS (+) FT

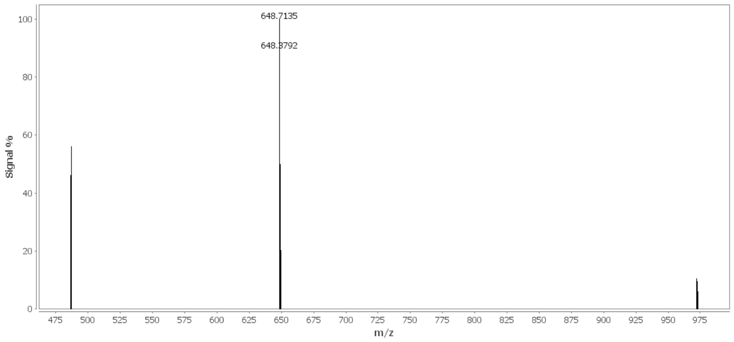

MS2 (+) FT activ = HCD:ce =

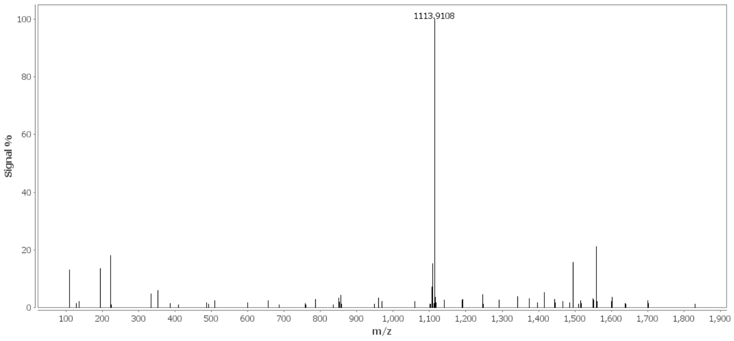

MS2 (+) FT activ = HCD:ce =

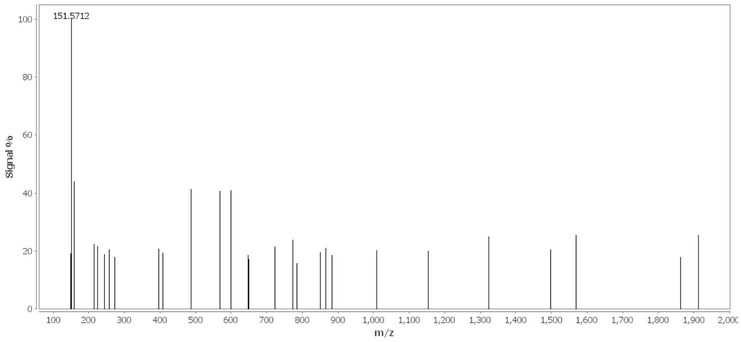

Metabolite: M1 -1396 RT=9.77

Type	score	sub. m/z observed	sub. m/z calculated	sub ppm	met. m/z observed	met. m/z calculated	met. ppm

Metabolite: M1 -1396 RT=9.77

Type	score	sub. m/z observed	sub. m/z calculated	sub ppm		met. m/z observed	met. m/z calculated	met. ppm
MATCH	48.8	668.5489	668.5492	0.36	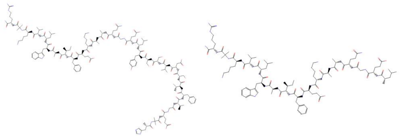	486.5365	486.5361	-0.80
MATCH	48.8	668.5489	668.5492	0.36	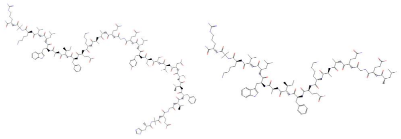	486.5365	486.5361	-0.80
MATCH	48.8	668.5489	668.5492	0.36	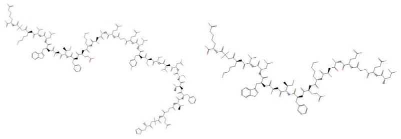	486.5365	486.5361	-0.80
MATCH	92.5	668.5489	668.5492	0.36	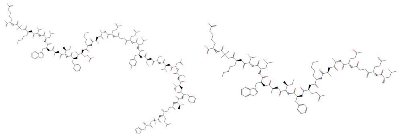	648.3792	648.3790	-0.34
MATCH	92.5	668.5489	668.5492	0.36	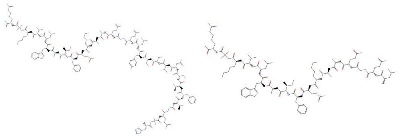	648.3792	648.3790	-0.34
MATCH	92.5	668.5489	668.5492	0.36	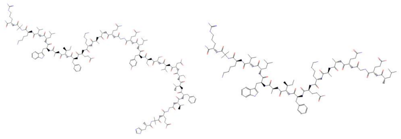	648.3792	648.3790	-0.34
MATCH	13.3	668.5489	668.5492	0.36	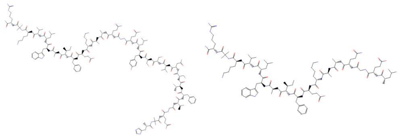	972.0647	972.0649	0.18
MATCH	13.3	668.5489	668.5492	0.36	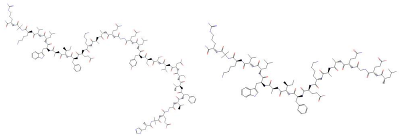	972.0647	972.0649	0.18
MATCH	100.9	835.4415	835.4347	-8.23	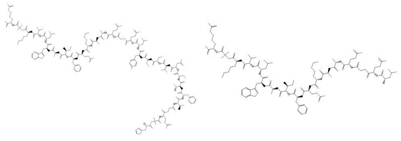	486.5365	486.5361	-0.80

Metabolite: M1 -1396 RT=9.77

Type	score	sub. m/z observed	sub. m/z calculated	sub ppm		met. m/z observed	met. m/z calculated	met. ppm
MATCH	100.9	835.4415	835.4347	-8.23	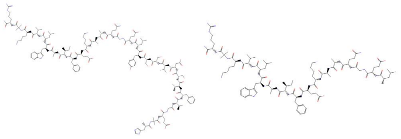	486.5365	486.5361	-0.80
					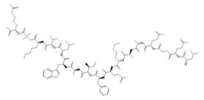	486.5365	486.5361	-0.80
MATCH	144.5	835.4415	835.4347	-8.23	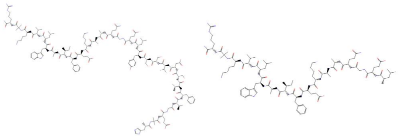	648.3792	648.3790	-0.34
MATCH	144.5	835.4415	835.4347	-8.23	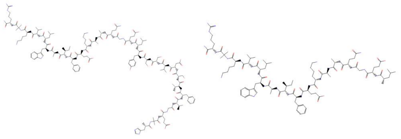	648.3792	648.3790	-0.34
					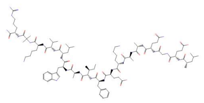	648.3792	648.3790	-0.34
MATCH	68.0	1113.5778	1113.5771	-0.63	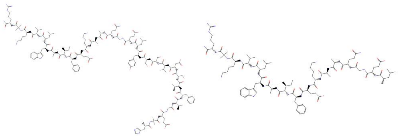	486.5365	486.5361	-0.80
MATCH	68.0	1113.5778	1113.5771	-0.63	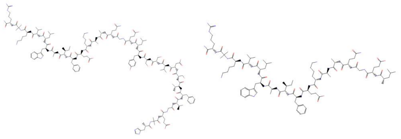	486.5365	486.5361	-0.80
MATCH	68.0	1113.5778	1113.5771	-0.63	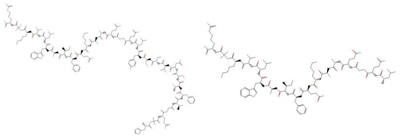	486.5365	486.5361	-0.80
MATCH	111.7	1113.5778	1113.5771	-0.63	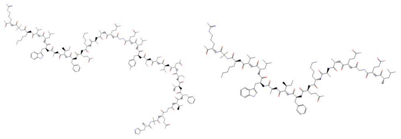	648.3792	648.3790	-0.34

Metabolite: M1 -1396 RT=9.77

Type	score	sub. m/z observed	sub. m/z calculated	sub ppm		met. m/z observed	met. m/z calculated	met. ppm
MATCH	111.7	1113.5778	1113.5771	-0.63	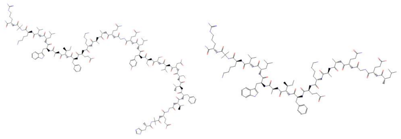	648.3792	648.3790	-0.34
MATCH	111.7	1113.5778	1113.5771	-0.63	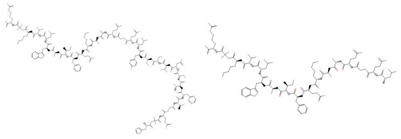	648.3792	648.3790	-0.34
MATCH	32.5	1113.5778	1113.5771	-0.63	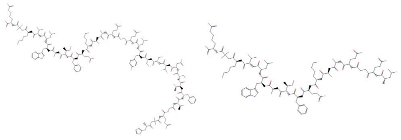	972.0647	972.0649	0.18
MATCH	32.5	1113.5778	1113.5771	-0.63	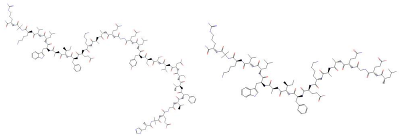	972.0647	972.0649	0.18
MATCH	43.0	486.3514	486.3511	-0.71	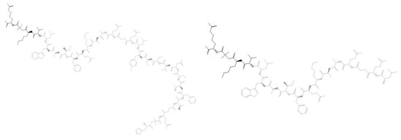	486.3520	486.3511	-1.83
MATCH	42.6	599.4353	599.4351	-0.27	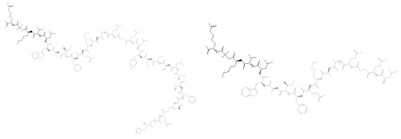	599.4326	599.4351	4.30
MATCH	18.6	785.5137	785.5145	0.99	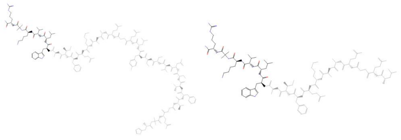	785.5115	785.5145	3.71
MATCH	22.8	851.0007	851.0016	1.08	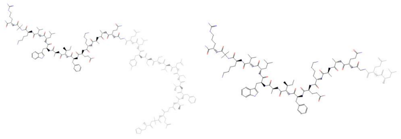	850.9989	851.0016	3.14
MATCH	77.1	1113.5781	1113.5771	-0.85	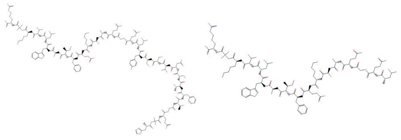	648.3777	648.3790	1.97

Metabolite: M1 -1396 RT=9.77

Type	score	sub. m/z observed	sub. m/z calculated	sub ppm		met. m/z observed	met. m/z calculated	met. ppm
MATCH	20.2	1638.7260	1638.7282	1.36	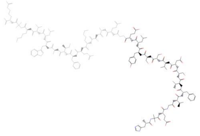 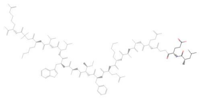	243.1335	243.1339	1.79
MET_MATCH					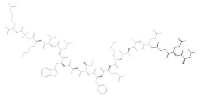	159.0916	159.0946	18.84
MET_MATCH					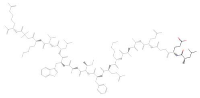	215.1394	215.1390	-1.93

MS (+) FT

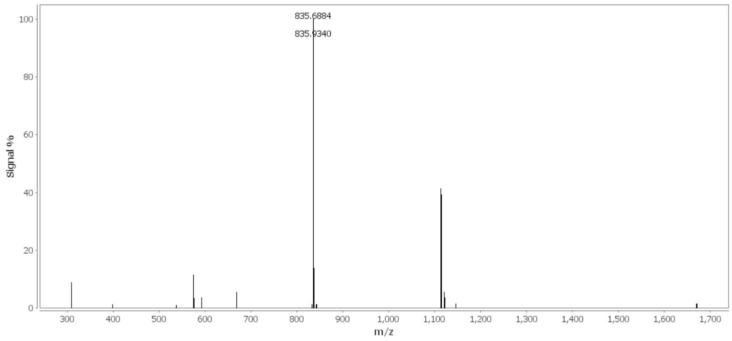

MS (+) FT

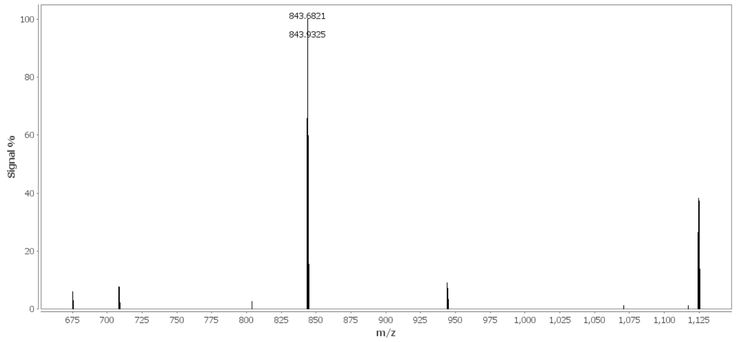

MS2 (+) FT activ = HCD:ce =

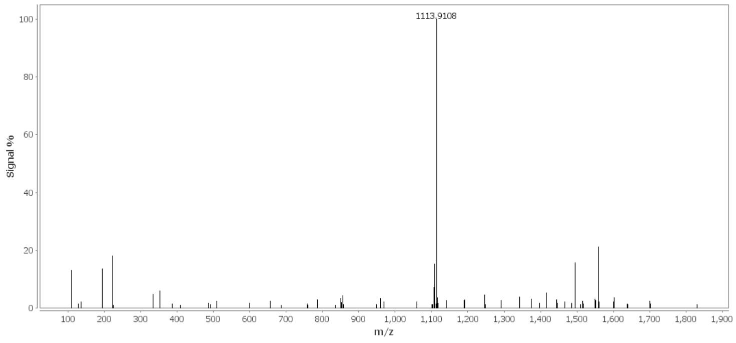

MS2 (+) FT activ = HCD:ce =

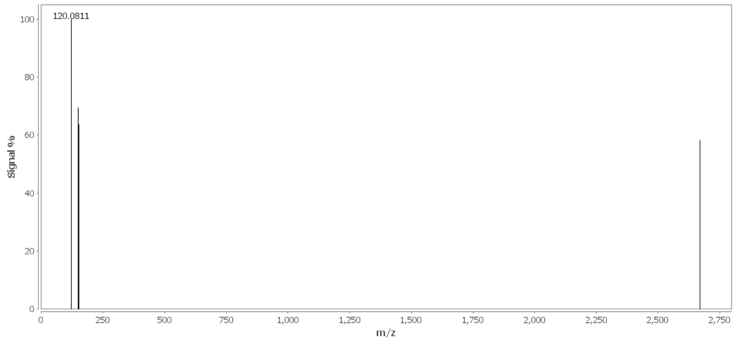

Metabolite: M3 -509 RT=10.48

Type	score	sub. m/z observed	sub. m/z calculated	sub ppm		met. m/z observed	met. m/z calculated	met. ppm
MATCH	8.2	668.5489	668.5492	0.36	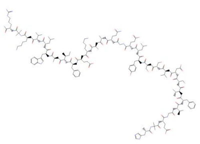 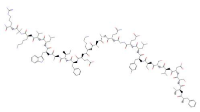	708.1292	708.1288	-0.59

Metabolite: M3 -509 RT=10.48

Type	score	sub. m/z observed	sub. m/z calculated	sub ppm		met. m/z observed	met. m/z calculated	met. ppm
MATCH	8.2	668.5489	668.5492	0.36	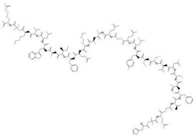 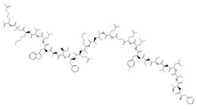	708.1292	708.1288	-0.59
					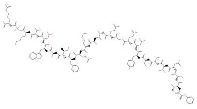	708.1292	708.1288	-0.59
MATCH	8.3	668.5489	668.5492	0.36	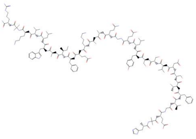 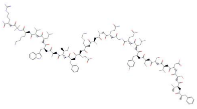	943.8367	943.8360	-0.72
MATCH	8.3	668.5489	668.5492	0.36	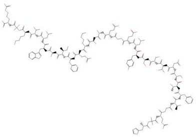 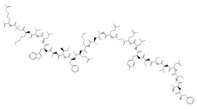	943.8367	943.8360	-0.72
					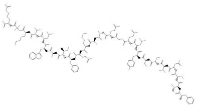	943.8367	943.8360	-0.72
MATCH	60.2	835.4415	835.4347	-8.23	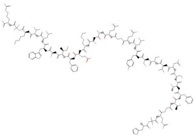 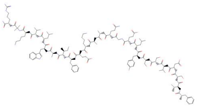	708.1292	708.1288	-0.59
MATCH	60.2	835.4415	835.4347	-8.23	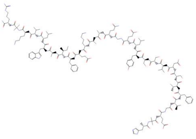 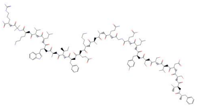	708.1292	708.1288	-0.59
					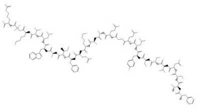	708.1292	708.1288	-0.59
MATCH	60.3	835.4415	835.4347	-8.23	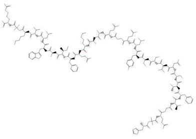 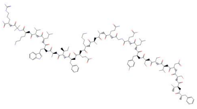	943.8367	943.8360	-0.72

Metabolite: M3 -509 RT=10.48

Type	score	sub. m/z observed	sub. m/z calculated	sub ppm		met. m/z observed	met. m/z calculated	met. ppm
MATCH	60.3	835.4415	835.4347	-8.23	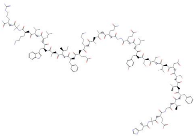 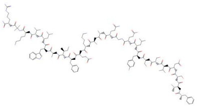	943.8367	943.8360	-0.72
					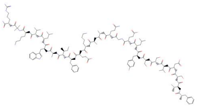	943.8367	943.8360	-0.72
MATCH	27.4	1113.5778	1113.5771	-0.63	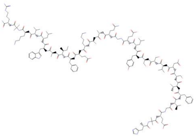 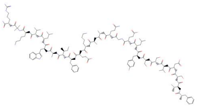	708.1292	708.1288	-0.59
MATCH	27.4	1113.5778	1113.5771	-0.63	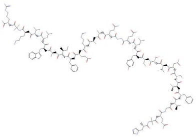 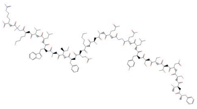	708.1292	708.1288	-0.59
					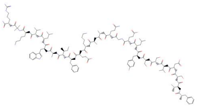	708.1292	708.1288	-0.59
MATCH	27.5	1113.5778	1113.5771	-0.63	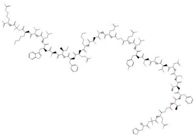 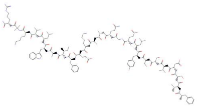	943.8367	943.8360	-0.72
MATCH	27.5	1113.5778	1113.5771	-0.63	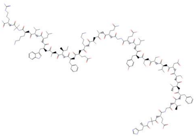 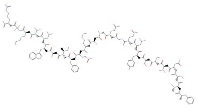	943.8367	943.8360	-0.72
					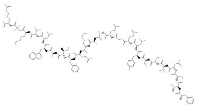	943.8367	943.8360	-0.72
MET_MATCH					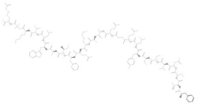	120.0811	120.0808	-2.61
