# Supplementary material for: Software-aided approach to investigate peptide structure and metabolic susceptibility of amide bonds in peptide drugs based on high resolution mass spectrometry
Source: PLoS One. 2017 Nov 1;12(11):e0186461. doi: 10.1371/journal.pone.0186461 (PMC5665424; doi:10.1371/journal.pone.0186461)
Supplement: S1 File — (ZIP) [file pone.0186461.s007.zip › SFiles/S6_File.pdf]

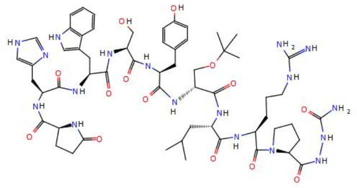

Goserelin

Property name	Property value
Time	0min, 5min, 15min, 45min, 120min
Instrument	ThermoQAPlus
Matrix	trypsin
Acquisition Mode	ddMS2

Chromatograms

Time=0min

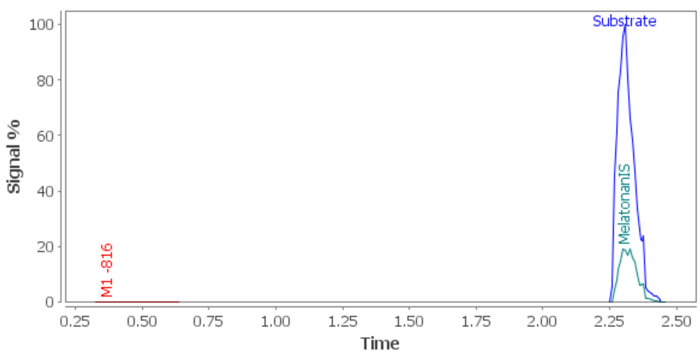

Time=5min

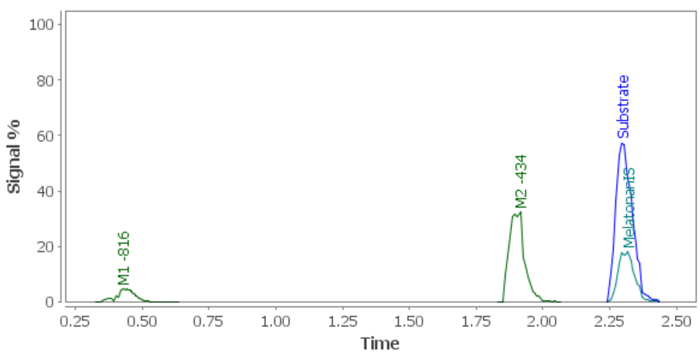

Time=15min

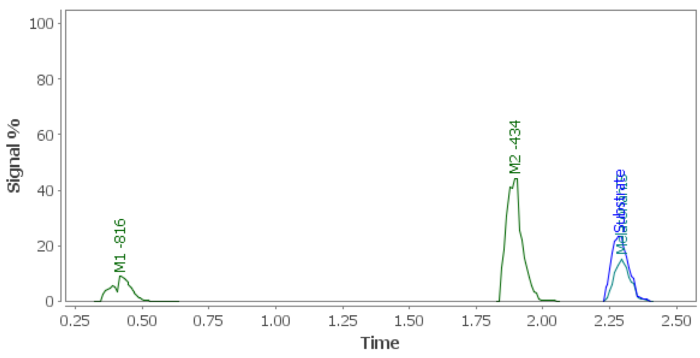

Time=45min

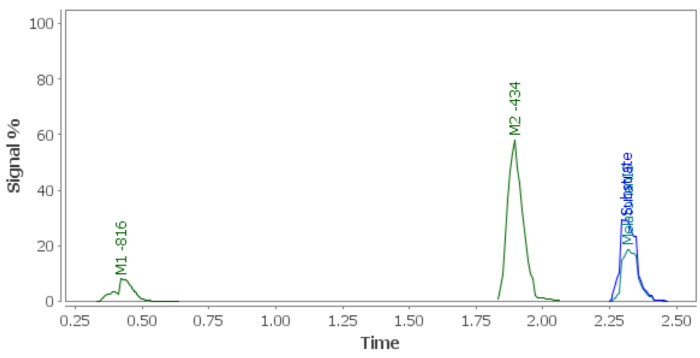

Time=120min

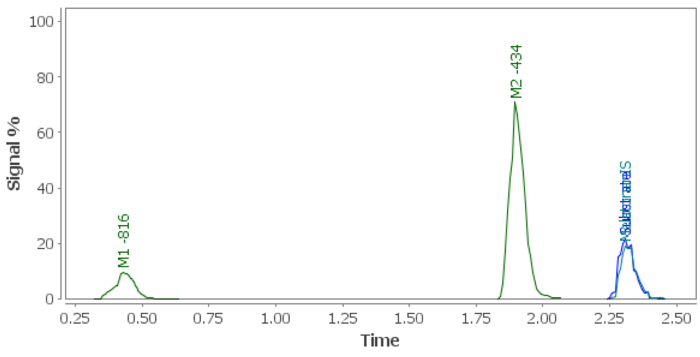

Custom Charts

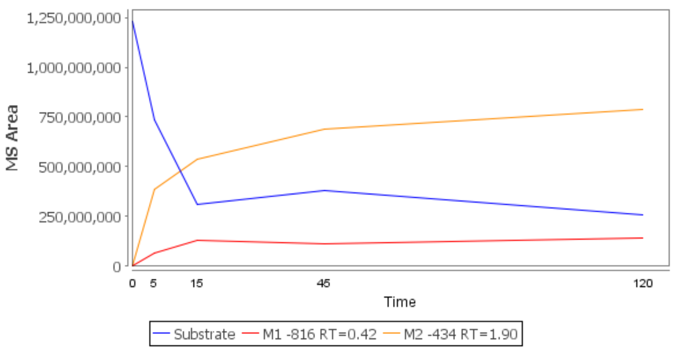

Fragmentation

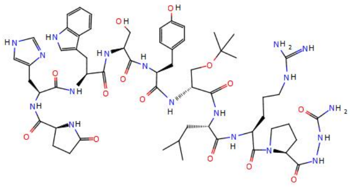

Goserelin

MS (+) FT

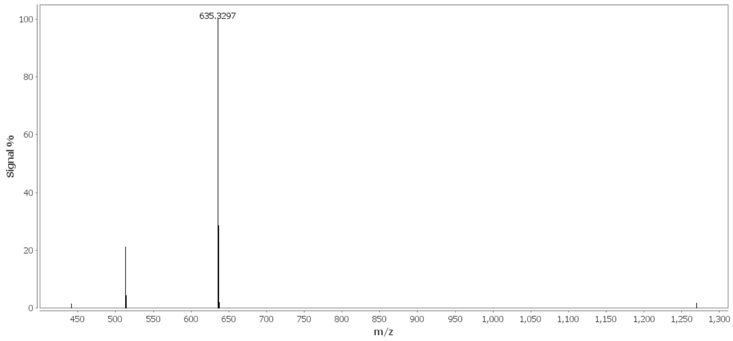

MS (+) FT

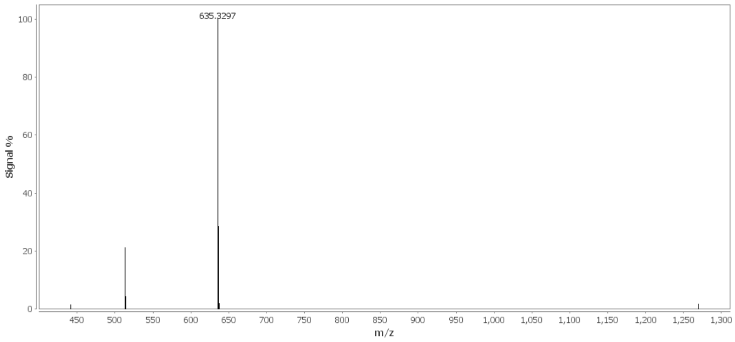

MS2 (+) FT activ = HCD:ce =

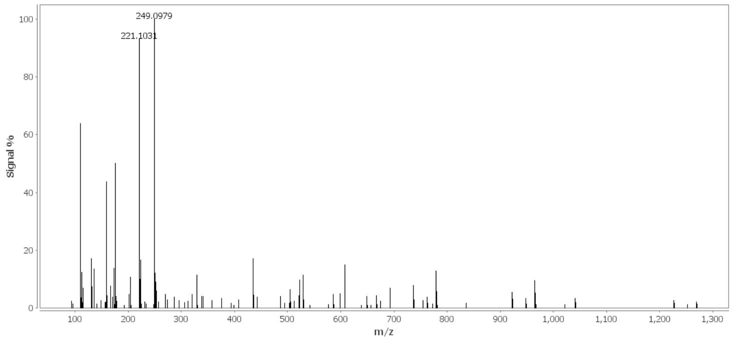

MS2 (+) FT activ = HCD:ce =

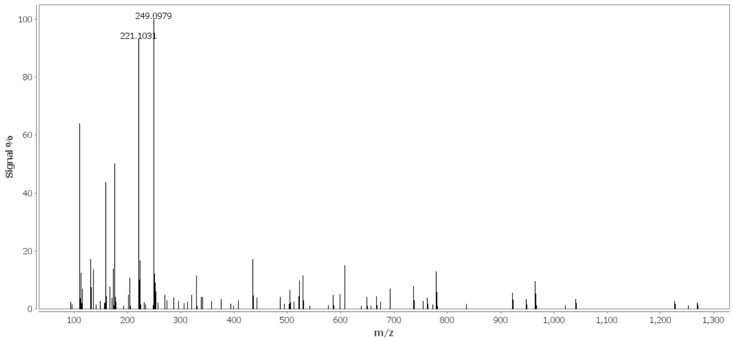

Metabolite: Substrate

Type	score	sub. m/z observed	sub. m/z calculated	sub ppm			met. m/z observed	met. m/z calculated	met. ppm
MATCH	13.5	1269.6517	1269.6487	-2.38	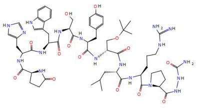	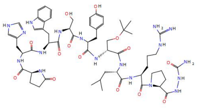	1269.6517	1269.6487	-2.38
MATCH	101.7	1269.6503	1269.6487	-1.27	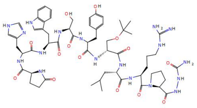	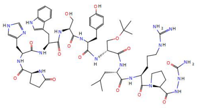	1269.6503	1269.6487	-1.27
MATCH	4.5	772.2981	772.3049	8.84	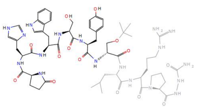	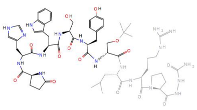	772.2981	772.3049	8.84

Metabolite: Substrate

Type	score	sub. m/z observed	sub. m/z calculated	sub ppm		met. m/z observed	met. m/z calculated	met. ppm
MATCH	5.2	754.2922	754.2944	2.89	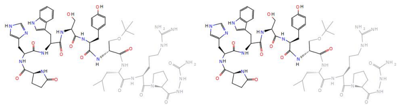	754.2922	754.2944	2.89
MISMATCH	-8.3	692.3831	692.3838	1.02	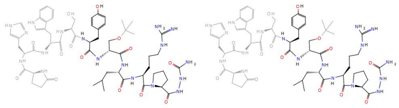	692.3831	692.3838	1.02
MATCH	101.1	657.2731	657.2780	7.40	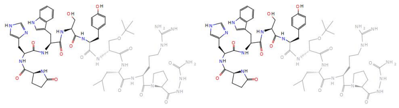	657.2731	657.2780	7.40
MATCH	200.0	635.3297	635.3280	-2.64	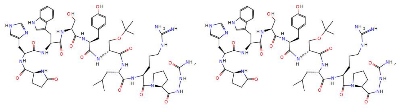	635.3297	635.3280	-2.64
MATCH	8.6	598.2916	598.2914	-0.38	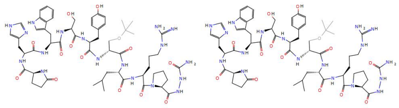	598.2916	598.2914	-0.38
MATCH	44.7	529.3205	529.3205	0.06	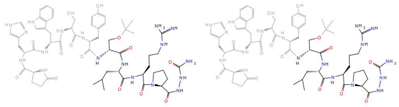	529.3205	529.3205	0.06
MATCH	8.3	512.2960	512.2940	-3.97	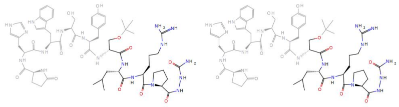	512.2960	512.2940	-3.97
MATCH	9.4	504.1990	504.1990	-0.04	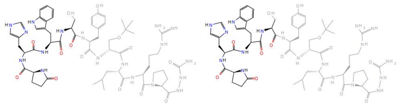	504.1990	504.1990	-0.04
MATCH	22.3	494.2126	494.2146	4.12	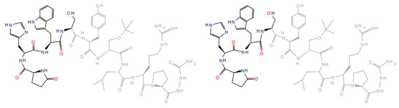	494.2126	494.2146	4.12

Metabolite: Substrate

Type	score	sub. m/z observed	sub. m/z calculated	sub ppm		met. m/z observed	met. m/z calculated	met. ppm
MATCH	9.7	442.2888	442.2885	-0.80	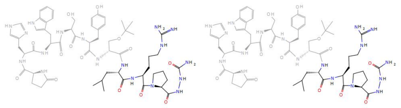	442.2888	442.2885	-0.80
MATCH	2.4	399.2827	399.2827	-0.04	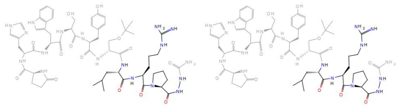	399.2827	399.2827	-0.04
MATCH	16.9	329.2033	329.2044	3.51	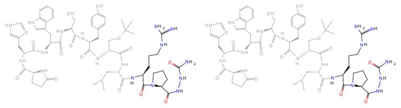	329.2033	329.2044	3.51
MATCH	4.2	312.1758	312.1779	6.65	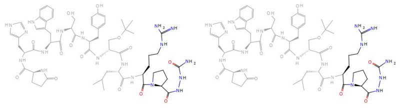	312.1758	312.1779	6.65
MATCH	4.5	295.1504	295.1513	3.02	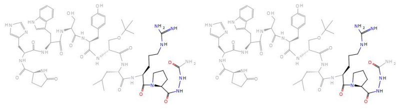	295.1504	295.1513	3.02
MATCH	6.4	286.1990	286.1986	-1.37	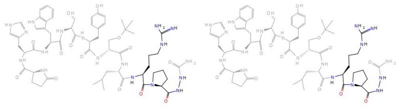	286.1990	286.1986	-1.37
MATCH	9.6	269.1713	269.1720	2.65	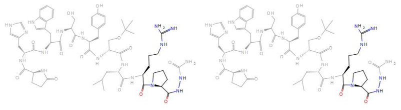	269.1713	269.1720	2.65
MATCH	12.9	253.1654	253.1659	1.84	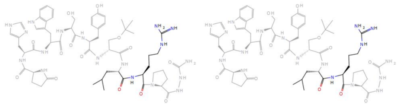	253.1654	253.1659	1.84
MATCH	174.6	249.0979	249.0982	1.11	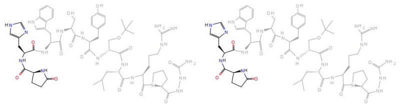	249.0979	249.0982	1.11

Metabolite: Substrate

Type	score	sub. m/z observed	sub. m/z calculated	sub ppm			met. m/z observed	met. m/z calculated	met. ppm
MATCH	173.0	221.1031	221.1033	0.90	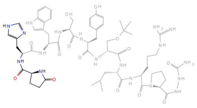	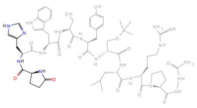	221.1031	221.1033	0.90
MISMATCH	17.9	173.1037	173.1033	-2.12	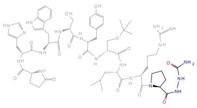	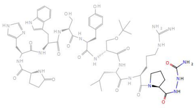	173.1037	173.1033	-2.12
MATCH	15.0	166.0610	166.0611	0.40	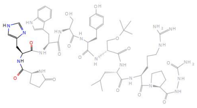	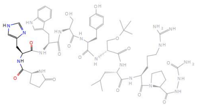	166.0610	166.0611	0.40
MATCH	56.4	159.0915	159.0917	0.87	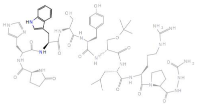	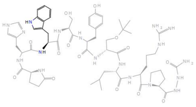	159.0915	159.0917	0.87
MATCH	4.5	157.1079	157.1084	2.95	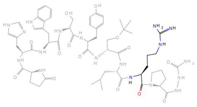	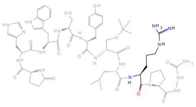	157.1079	157.1084	2.95
MISMATCH	-3.5	156.0797	156.0768	-18.9	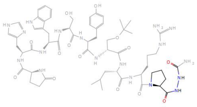	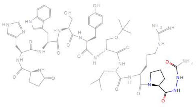	156.0797	156.0768	-18.9
MISMATCH	-3.5	156.0797	156.0768	-18.9	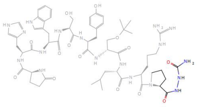	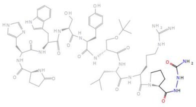	156.0797	156.0768	-18.9
MATCH	76.9	136.0757	136.0757	-0.20	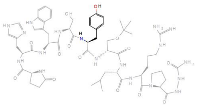	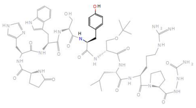	136.0757	136.0757	-0.20
MATCH	34.5	130.0975	130.0975	-0.01	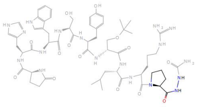	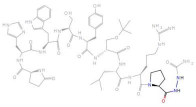	130.0975	130.0975	-0.01

Metabolite: Substrate

Type	score	sub. m/z observed	sub. m/z calculated	sub ppm		met. m/z observed	met. m/z calculated	met. ppm
MATCH	17.5	115.0869	115.0866	-2.52	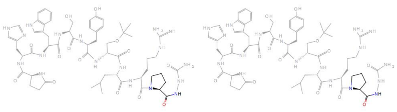	115.0869	115.0866	-2.52
MATCH	26.1	112.0872	112.0869	-2.40	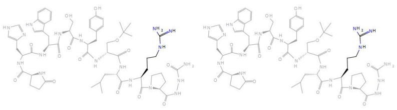	112.0872	112.0869	-2.40
MATCH	163.8	110.0716	110.0713	-2.78	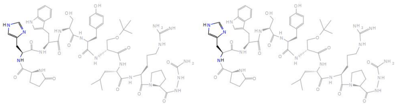	110.0716	110.0713	-2.78
MATCH	4.8	95.0608	95.0604	-4.90	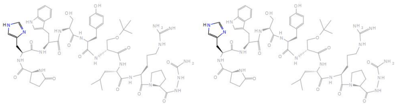	95.0608	95.0604	-4.90

MS (+) FT

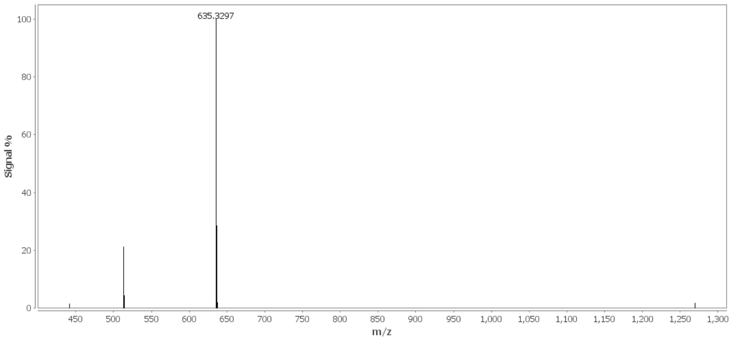

MS (+) FT

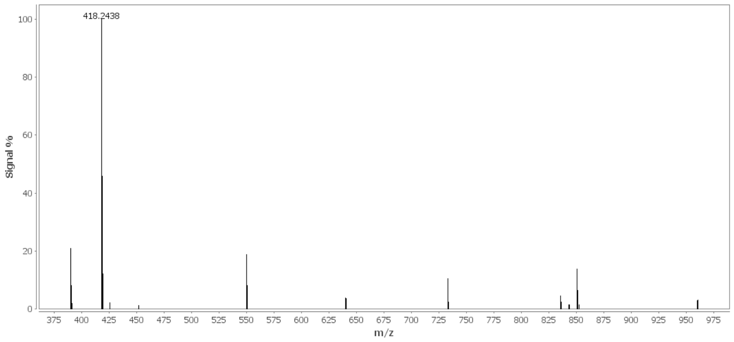

MS2 (+) FT activ = HCD:ce =

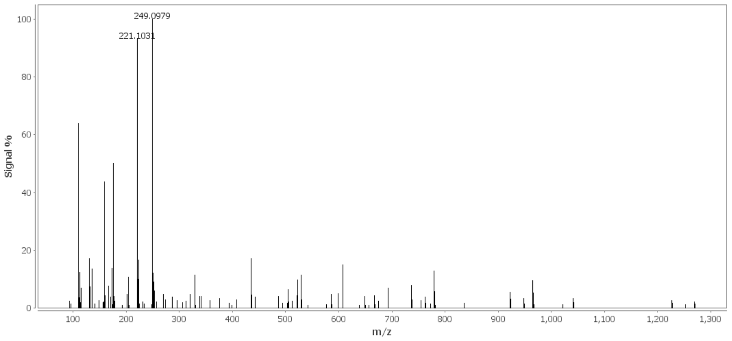

MS2 (+) FT activ = HCD:ce =

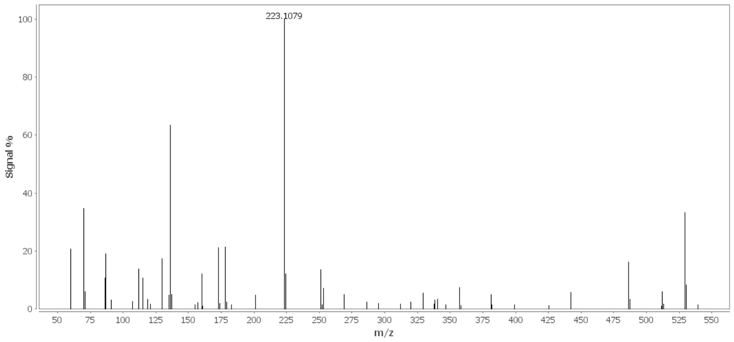

Metabolite: M2 -434 RT=1.90

Type	score	sub. m/z observed	sub. m/z calculated	sub ppm		met. m/z observed	met. m/z calculated	met. ppm

Metabolite: M2 -434 RT=1.90

Type	score	sub. m/z observed	sub. m/z calculated	sub ppm		met. m/z observed	met. m/z calculated	met. ppm
MATCH	200.0	635.3297	635.3280	-2.64	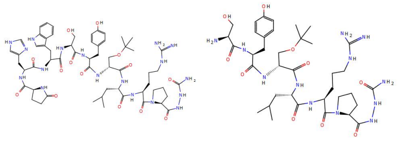	418.2438	418.2429	-2.27
MATCH	200.0	635.3297	635.3280	-2.64	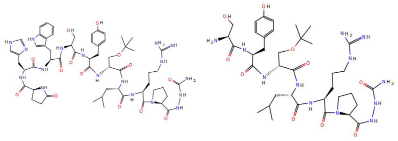	418.2438	418.2429	-2.27
MATCH	104.6	635.3297	635.3280	-2.64	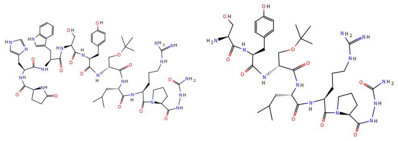	835.4804	835.4785	-2.30
MATCH	104.6	635.3297	635.3280	-2.64	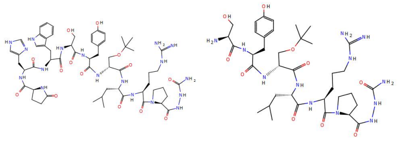	835.4804	835.4785	-2.30
MATCH	101.7	1269.6503	1269.6487	-1.27	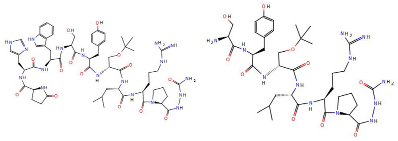	418.2438	418.2429	-2.27
MATCH	101.7	1269.6503	1269.6487	-1.27	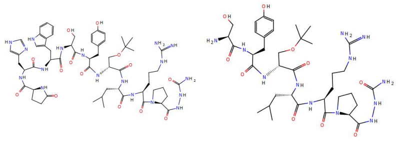	418.2438	418.2429	-2.27
MATCH	6.3	1269.6503	1269.6487	-1.27	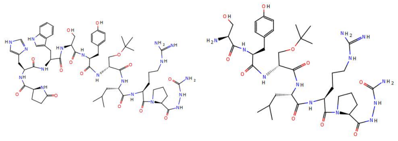	835.4804	835.4785	-2.30
MATCH	6.3	1269.6503	1269.6487	-1.27	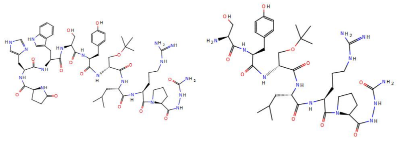	835.4804	835.4785	-2.30
MATCH	26.1	112.0872	112.0869	-2.40	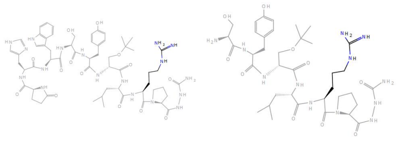	112.0875	112.0869	-4.90

Metabolite: M2 -434 RT=1.90

Type	score	sub. m/z observed	sub. m/z calculated	sub ppm		met. m/z observed	met. m/z calculated	met. ppm
MATCH	17.5	115.0869	115.0866	-2.52	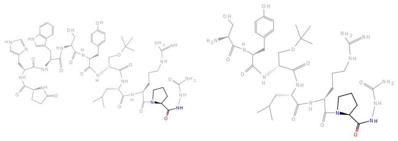	115.0871	115.0866	-4.49
MATCH	34.5	130.0975	130.0975	-0.01	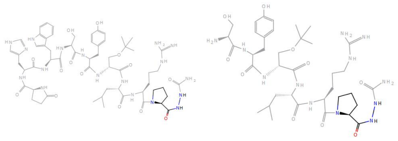	130.0978	130.0975	-2.61
MATCH	76.9	136.0757	136.0757	-0.20	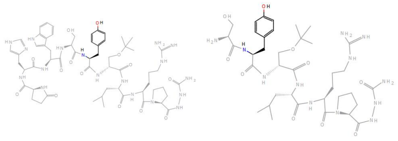	136.0760	136.0757	-2.28
MATCH	4.5	157.1079	157.1084	2.95	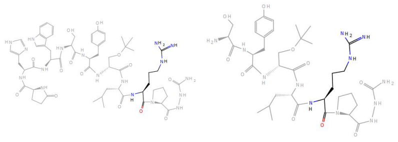	157.1086	157.1084	-1.44
MATCH	17.9	173.1037	173.1033	-2.12	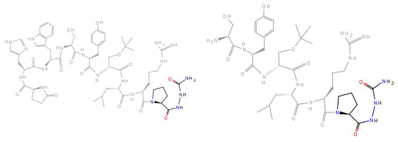	173.1047	173.1033	-7.85
MATCH	12.9	253.1654	253.1659	1.84	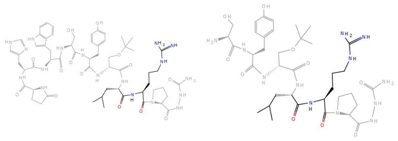	253.1661	253.1659	-0.75
MATCH	9.6	269.1713	269.1720	2.65	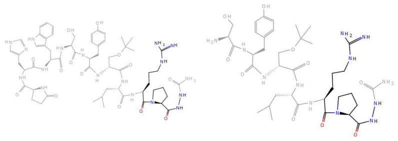	269.1723	269.1720	-1.01
MATCH	6.4	286.1990	286.1986	-1.37	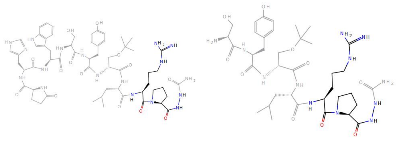	286.1997	286.1986	-3.81
MATCH	4.5	295.1504	295.1513	3.02	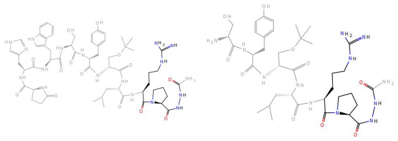	295.1525	295.1513	-4.02

Metabolite: M2 -434 RT=1.90

Type	score	sub. m/z observed	sub. m/z calculated	sub ppm		met. m/z observed	met. m/z calculated	met. ppm
MATCH	4.2	312.1758	312.1779	6.65	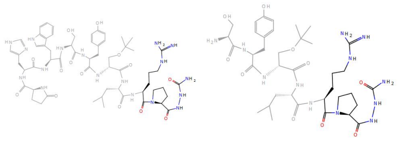	312.1781	312.1779	-0.67
MATCH	16.9	329.2033	329.2044	3.51	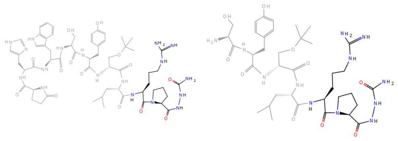	329.2018	329.2044	7.86
MATCH	2.4	399.2827	399.2827	-0.04	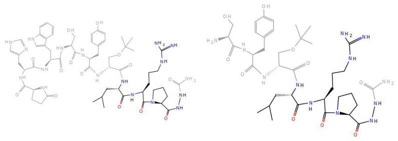	399.2825	399.2827	0.32
MATCH	9.7	442.2888	442.2885	-0.80	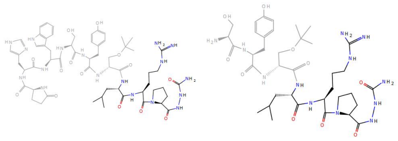	442.2888	442.2885	-0.82
MATCH	22.3	494.2126	494.2146	4.12	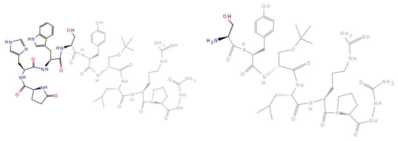	60.0454	60.0444	-16.3
MATCH	9.4	504.1990	504.1990	-0.04	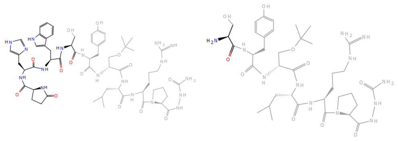	70.0296	70.0287	-11.9
MATCH	8.3	512.2960	512.2940	-3.97	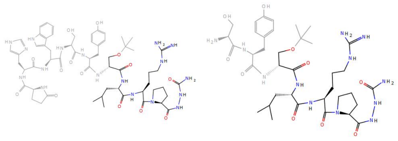	512.2957	512.2940	-3.33
MATCH	44.7	529.3205	529.3205	0.06	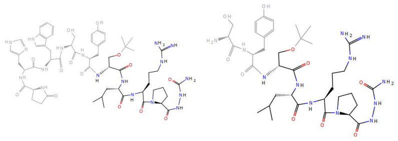	529.3211	529.3205	-1.19
MATCH	8.6	598.2916	598.2914	-0.38	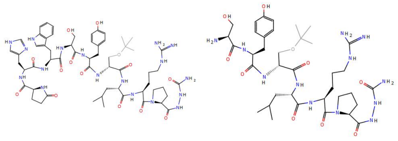	381.2061	381.2063	0.49

Metabolite: M2 -434 RT=1.90

Type	score	sub. m/z observed	sub. m/z calculated	sub ppm			met. m/z observed	met. m/z calculated	met. ppm
MATCH	101.1	657.2731	657.2780	7.40	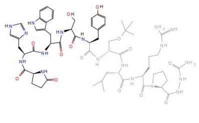	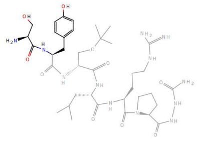	223.1079	223.1077	-0.97
MATCH	5.2	754.2922	754.2944	2.89	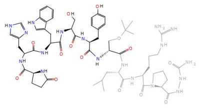	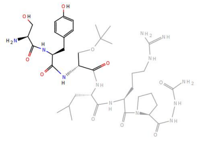	320.1249	320.1241	-2.54
MATCH	4.5	772.2981	772.3049	8.84	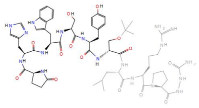	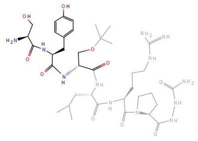	338.1345	338.1347	0.38
MISMATCH	-28.7	173.1037	173.1033	-2.12	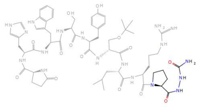		87.0561	87.0561	0.00
MISMATCH	-8.3	692.3831	692.3838	1.02	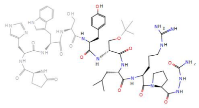		346.6954	346.6954	0.00
MET_MATCH						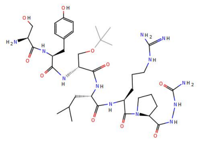	390.2124	390.2116	-2.03
MET_MATCH						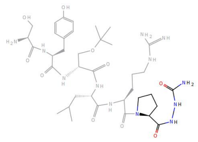	87.0561	87.0553	-8.86
MET_MATCH						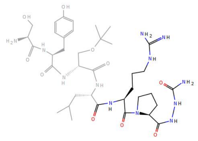	178.0864	178.0955	50.79
MET_MATCH						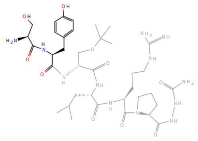	251.1028	251.1026	-0.77

Metabolite: M2 -434 RT=1.90

Type	score	sub. m/z observed	sub. m/z calculated	sub ppm		met. m/z observed	met. m/z calculated	met. ppm
MET_MATCH					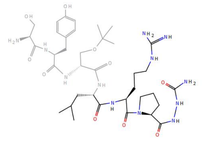	425.2626	425.2619	-1.66

MS (+) FT

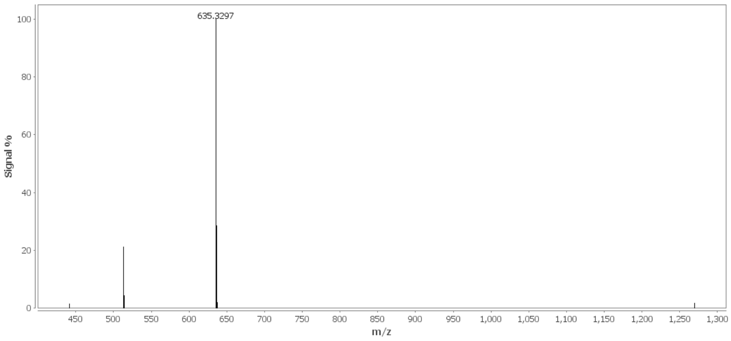

MS (+) FT

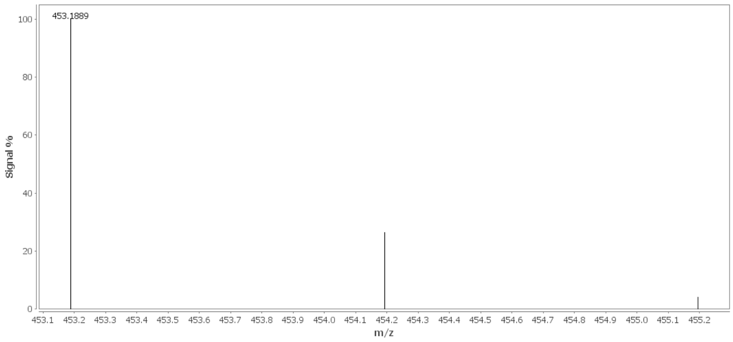

MS2 (+) FT activ = HCD:ce =

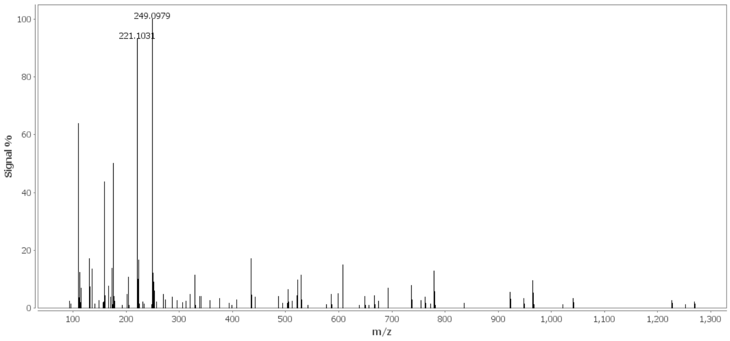

MS2 (+) FT activ = HCD:ce =

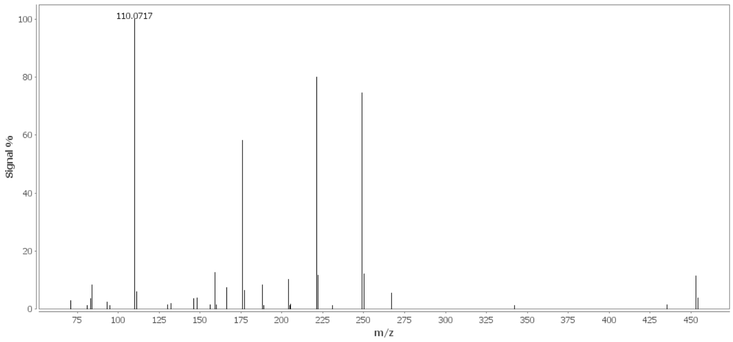

Metabolite: M1 -816 RT=0.42

Type	score	sub. m/z observed	sub. m/z calculated	sub ppm			met. m/z observed	met. m/z calculated	met. ppm
MATCH	200.0	635.3297	635.3280	-2.64	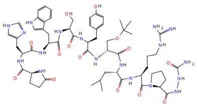	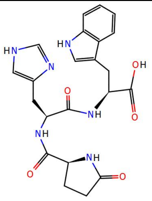	453.1889	453.1881	-1.69
						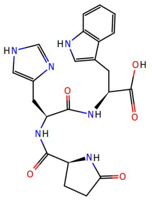	453.1889	453.1881	-1.69
MATCH	101.7	1269.6503	1269.6487	-1.27	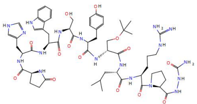	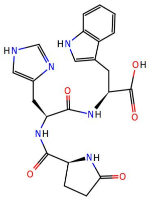	453.1889	453.1881	-1.69

Metabolite: M1 -816 RT=0.42

Type	score	sub. m/z observed	sub. m/z calculated	sub ppm			met. m/z observed	met. m/z calculated	met. ppm
						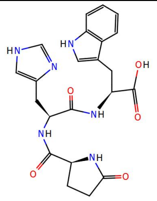	453.1889	453.1881	-1.69
MATCH	4.8	93.0454	93.0447	-6.91	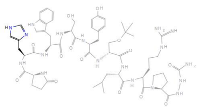	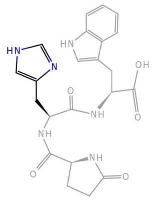	93.0454	93.0447	-6.77
MATCH	2.6	95.0608	95.0604	-4.90	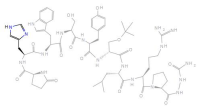	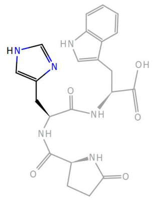	95.0609	95.0604	-5.70
MATCH	163.8	110.0716	110.0713	-2.78	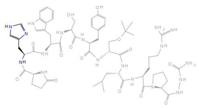	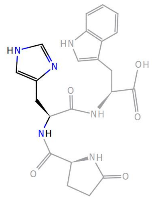	110.0717	110.0713	-3.98
MATCH	56.4	159.0915	159.0917	0.87	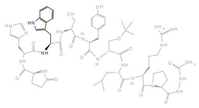	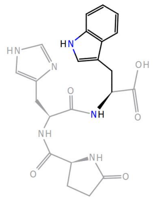	159.0917	159.0917	-0.38
MATCH	15.0	166.0610	166.0611	0.40	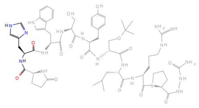	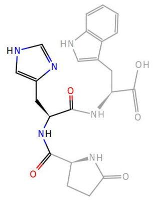	166.0612	166.0611	-0.53
MATCH	173.0	221.1031	221.1033	0.90	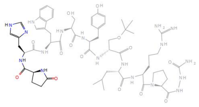	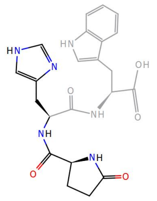	221.1033	221.1033	-0.05
MATCH	174.6	249.0979	249.0982	1.11	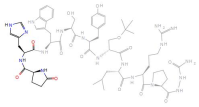	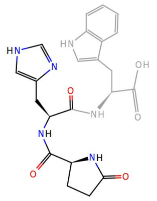	249.0982	249.0982	-0.04
MATCH	13.5	1269.6517	1269.6487	-2.38	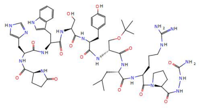	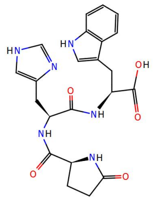	453.1880	453.1881	0.23

Metabolite: M1 -816 RT=0.42

Type	score	sub. m/z observed	sub. m/z calculated	sub ppm		met. m/z observed	met. m/z calculated	met. ppm
MISMATCH	-3.5	156.0797	156.0768	-18.9	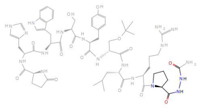	156.0767	156.0767	0.00
MET_MATCH					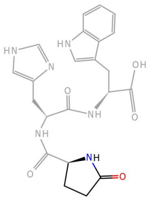	84.0451	84.0444	-8.75
MET_MATCH					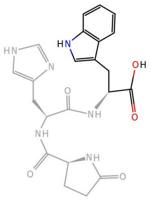	188.0705	188.0706	0.55
MET_MATCH					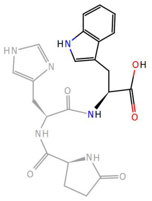	205.0971	205.0972	0.26
MET_MATCH					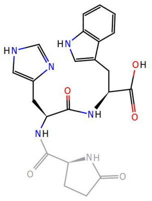	342.1562	342.1561	-0.32
MET_MATCH					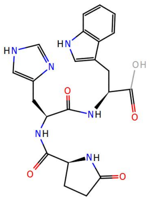	435.1764	435.1775	2.57
