# Supplementary material for: Software-aided approach to investigate peptide structure and metabolic susceptibility of amide bonds in peptide drugs based on high resolution mass spectrometry
Source: PLoS One. 2017 Nov 1;12(11):e0186461. doi: 10.1371/journal.pone.0186461 (PMC5665424; doi:10.1371/journal.pone.0186461)
Supplement: S1 File — (ZIP) [file pone.0186461.s007.zip › SFiles/S7_File.pdf]

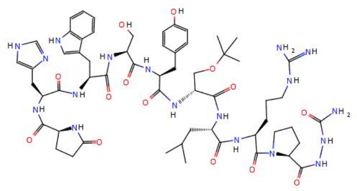

Goserelin

Property name	Property value
Time	0min, 5min, 15min, 45min, 120min
Instrument	ThermoQAPlus
Matrix	elastase
Acquisition Mode	ddMS2

Chromatograms

Time=0min

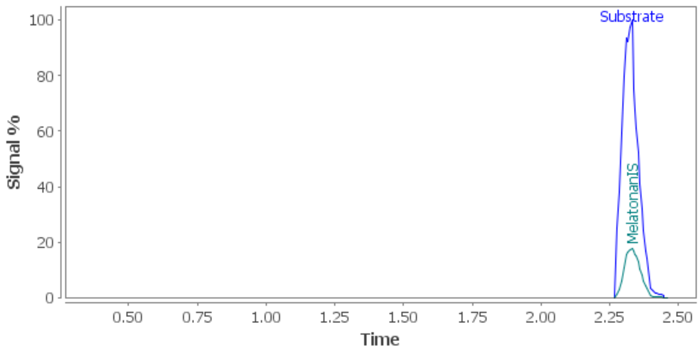

Time=5min

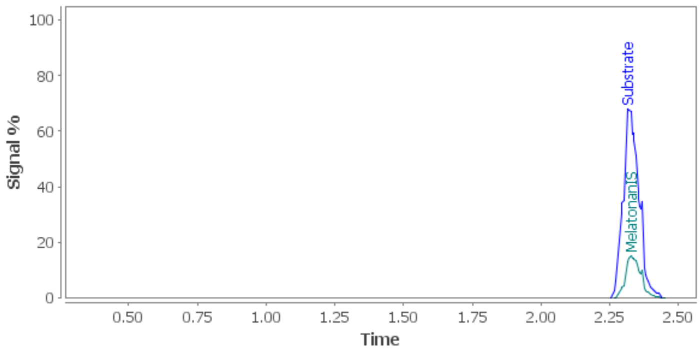

Time=15min

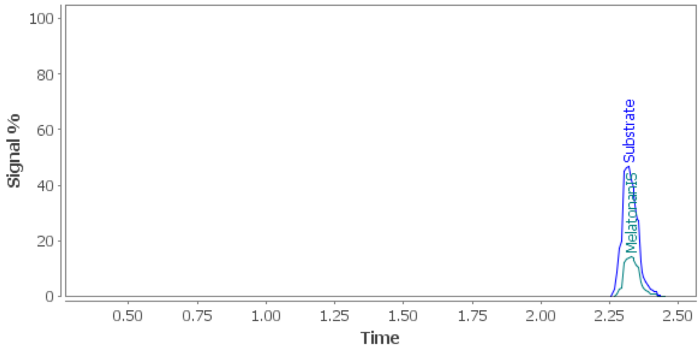

Time=45min

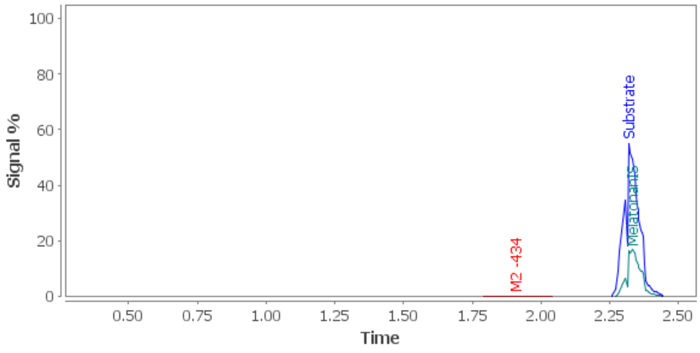

Time=120min

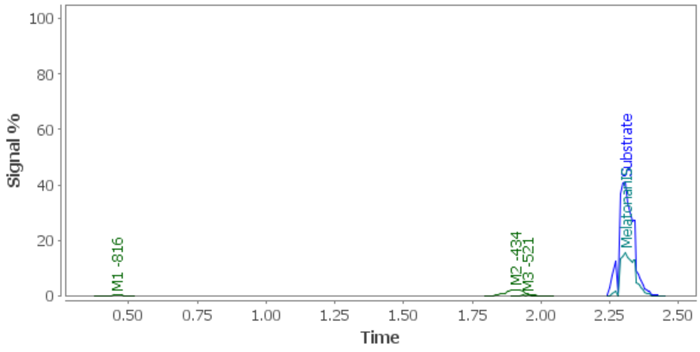

Custom Charts

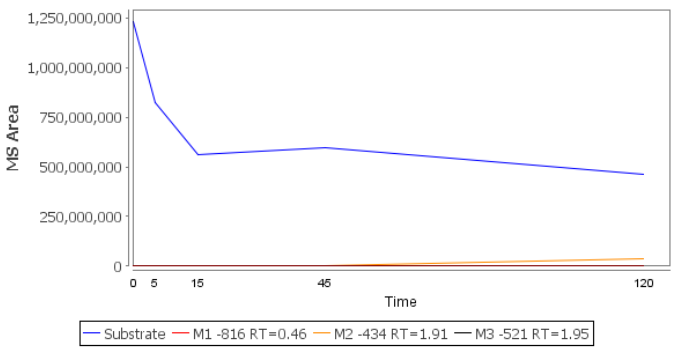

Fragmentation

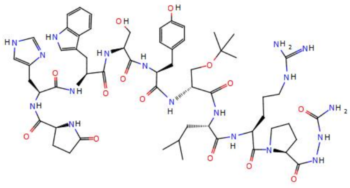

Goserelin

MS (+) FT

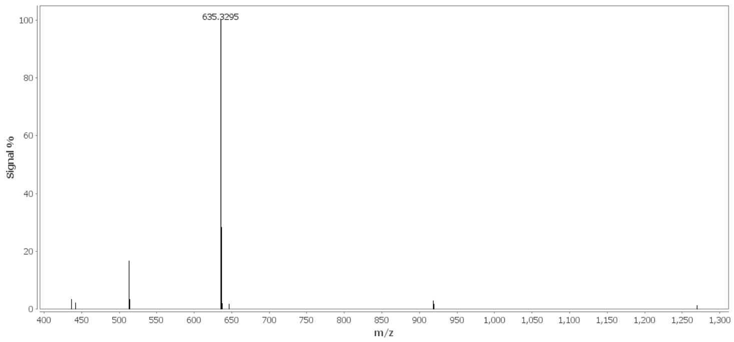

MS (+) FT

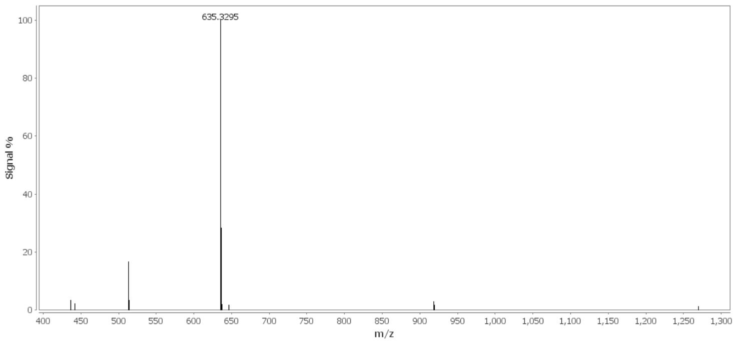

MS2 (+) FT activ = HCD:ce =

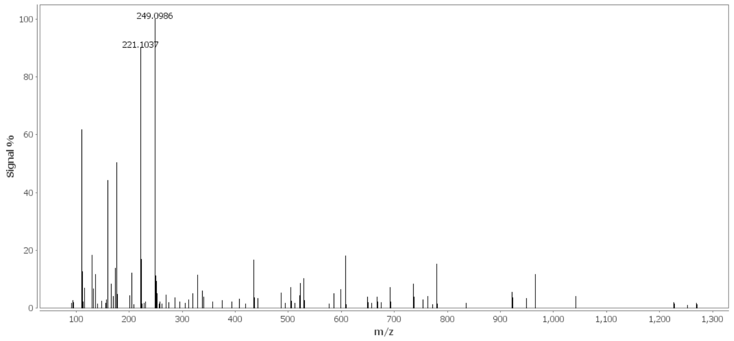

MS2 (+) FT activ = HCD:ce =

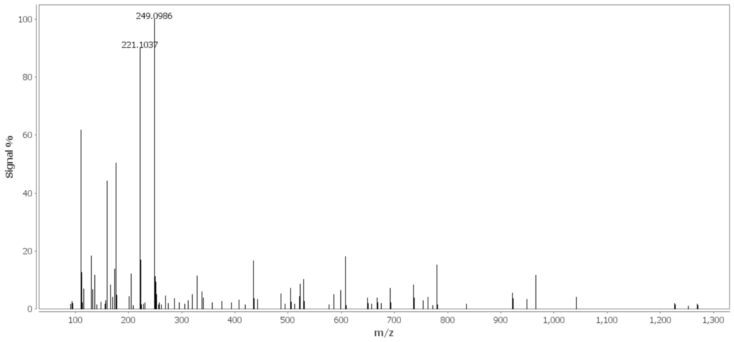

Metabolite: Substrate

Type	score	sub. m/z observed	sub. m/z calculated	sub ppm		met. m/z observed	met. m/z calculated	met. ppm
MATCH	4.8	772.3004	772.3049	5.83	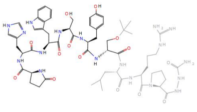 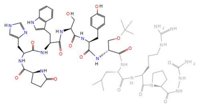	772.3004	772.3049	5.83
MATCH	4.5	754.2917	754.2944	3.50	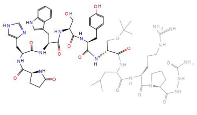 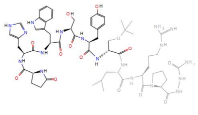	754.2917	754.2944	3.50
MISMATCH	-9.6	692.3850	692.3838	-1.75	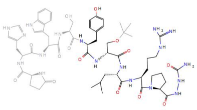 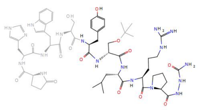	692.3850	692.3838	-1.75

Metabolite: Substrate

Type	score	sub. m/z observed	sub. m/z calculated	sub ppm		met. m/z observed	met. m/z calculated	met. ppm
MATCH	200.0	635.3294	635.3280	-2.16	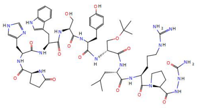 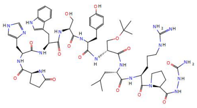	635.3294	635.3280	-2.16
MATCH	19.3	607.2989	607.2967	-3.68	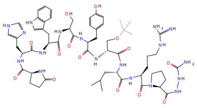 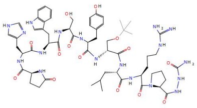	607.2989	607.2967	-3.68
MATCH	7.4	598.2934	598.2914	-3.37	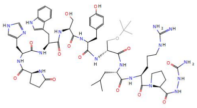 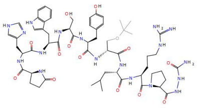	598.2934	598.2914	-3.37
MATCH	43.6	529.3216	529.3205	-2.14	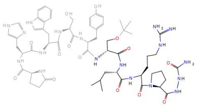 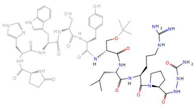	529.3216	529.3205	-2.14
MATCH	8.2	512.2925	512.2940	2.83	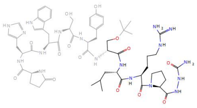 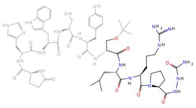	512.2925	512.2940	2.83
MATCH	9.0	504.2000	504.1990	-2.08	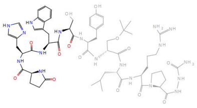 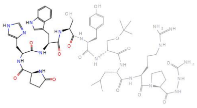	504.2000	504.1990	-2.08
MATCH	22.4	494.2139	494.2146	1.56	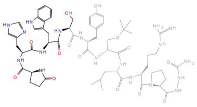 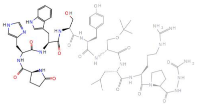	494.2139	494.2146	1.56
MATCH	10.3	442.2882	442.2885	0.57	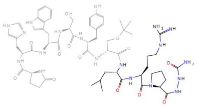 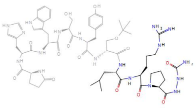	442.2882	442.2885	0.57
MATCH	4.6	399.2820	399.2827	1.57	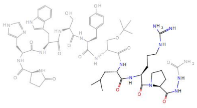 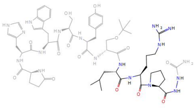	399.2820	399.2827	1.57

Metabolite: Substrate

Type	score	sub. m/z observed	sub. m/z calculated	sub ppm			met. m/z observed	met. m/z calculated	met. ppm
MATCH	15.0	329.2040	329.2044	1.40	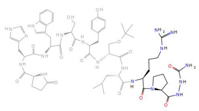	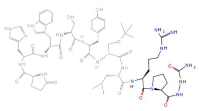	329.2040	329.2044	1.40
MATCH	4.2	312.1797	312.1779	-5.95	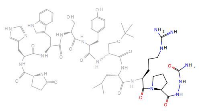	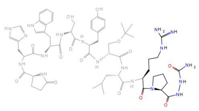	312.1797	312.1779	-5.95
MATCH	4.5	295.1519	295.1513	-2.03	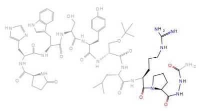	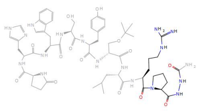	295.1519	295.1513	-2.03
MATCH	5.1	286.1994	286.1986	-2.95	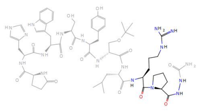	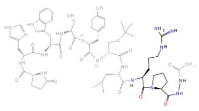	286.1994	286.1986	-2.95
MATCH	7.1	269.1718	269.1720	0.80	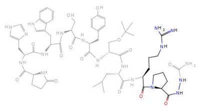	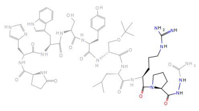	269.1718	269.1720	0.80
MATCH	11.1	253.1668	253.1659	-3.55	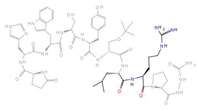	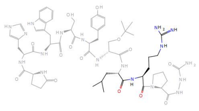	253.1668	253.1659	-3.55
MATCH	176.2	249.0987	249.0982	-1.88	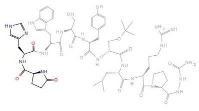	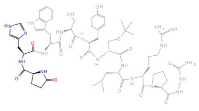	249.0987	249.0982	-1.88
MATCH	174.5	221.1038	221.1033	-2.17	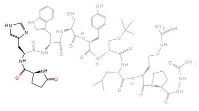	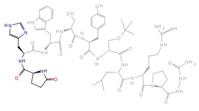	221.1038	221.1033	-2.17
MATCH	17.7	173.1043	173.1033	-6.02	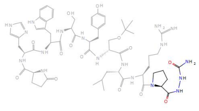	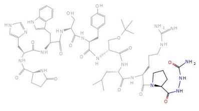	173.1043	173.1033	-6.02

Metabolite: Substrate

Type	score	sub. m/z observed	sub. m/z calculated	sub ppm			met. m/z observed	met. m/z calculated	met. ppm
MATCH	14.7	166.0615	166.0611	-2.32	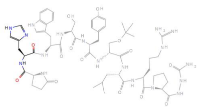	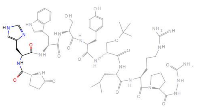	166.0615	166.0611	-2.32
MATCH	53.2	159.0920	159.0917	-2.36	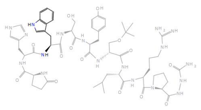	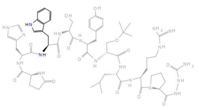	159.0920	159.0917	-2.36
MATCH	5.6	157.1085	157.1084	-0.52	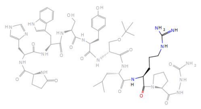	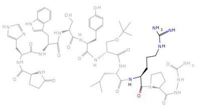	157.1085	157.1084	-0.52
MATCH	77.8	136.0762	136.0757	-3.51	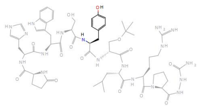	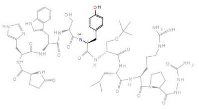	136.0762	136.0757	-3.51
MATCH	33.4	130.0980	130.0975	-3.70	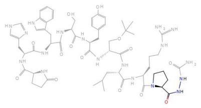	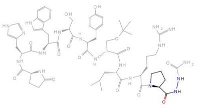	130.0980	130.0975	-3.70
MATCH	17.0	115.0873	115.0866	-5.96	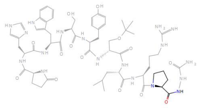	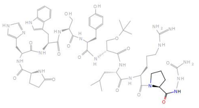	115.0873	115.0866	-5.96
MATCH	26.3	112.0875	112.0869	-5.45	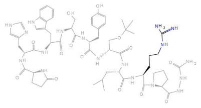	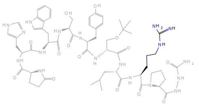	112.0875	112.0869	-5.45
MATCH	160.2	110.0719	110.0713	-5.85	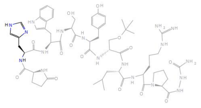	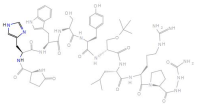	110.0719	110.0713	-5.85
MATCH	3.0	95.0612	95.0604	-9.12	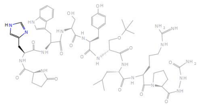	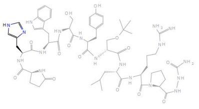	95.0612	95.0604	-9.12

Metabolite: Substrate

Type	score	sub. m/z observed	sub. m/z calculated	sub ppm		met. m/z observed	met. m/z calculated	met. ppm
MATCH	4.4	91.0551	91.0522	-32.0	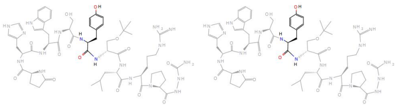	91.0551	91.0522	-32.0

MS (+) FT

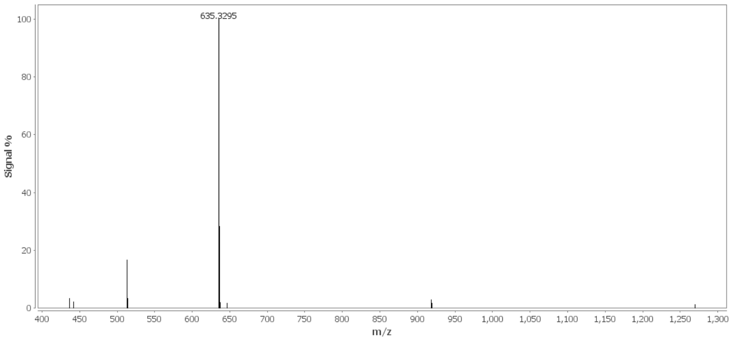

MS (+) FT

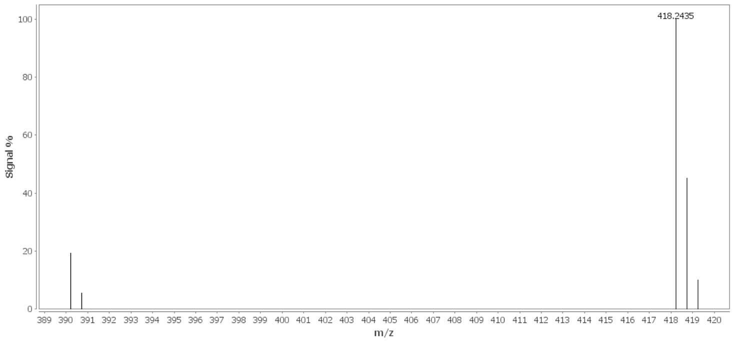

MS2 (+) FT activ = HCD:ce =

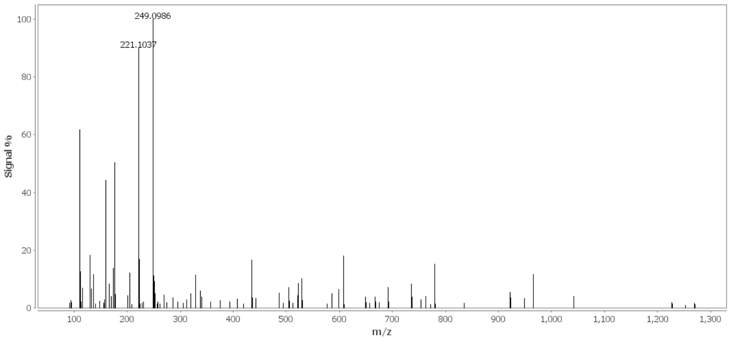

MS2 (+) FT activ = HCD:ce =

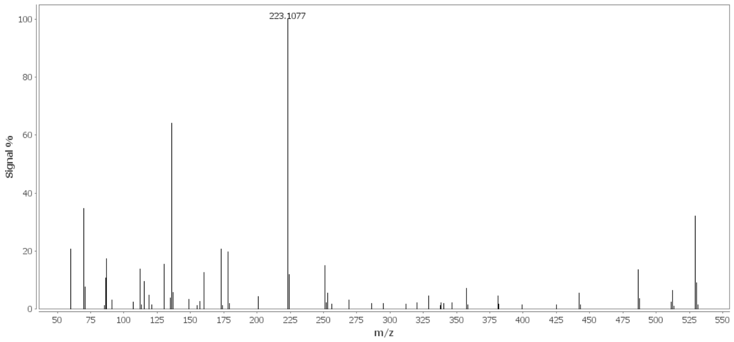

Metabolite: M2 -434 RT=1.91

Type	score	sub. m/z observed	sub. m/z calculated	sub ppm		met. m/z observed	met. m/z calculated	met. ppm
MATCH	200.0	635.3294	635.3280	-2.16	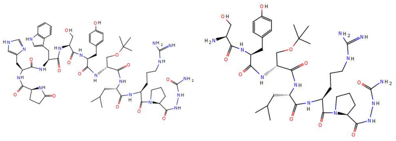	418.2435	418.2429	-1.58
MATCH	4.4	91.0551	91.0522	-32.0	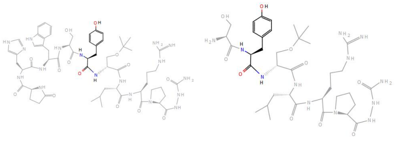	91.0547	91.0522	-27.2
MATCH	26.3	112.0875	112.0869	-5.45	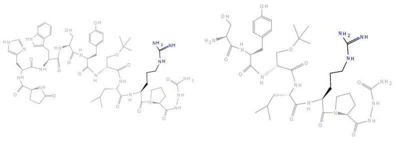	112.0874	112.0869	-3.89

Metabolite: M2 -434 RT=1.91

Type	score	sub. m/z observed	sub. m/z calculated	sub ppm		met. m/z observed	met. m/z calculated	met. ppm
MATCH	17.0	115.0873	115.0866	-5.96	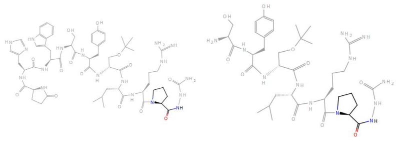	115.0869	115.0866	-2.98
MATCH	33.4	130.0980	130.0975	-3.70	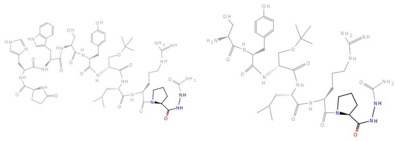	130.0976	130.0975	-1.19
MATCH	77.8	136.0762	136.0757	-3.51	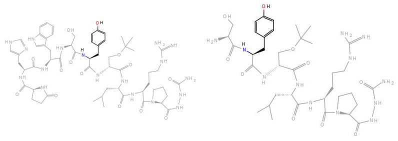	136.0759	136.0757	-1.30
MATCH	5.6	157.1085	157.1084	-0.52	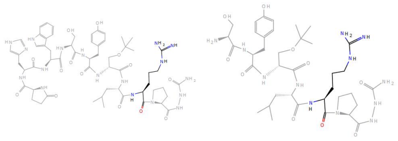	157.1086	157.1084	-1.24
MATCH	17.7	173.1043	173.1033	-6.02	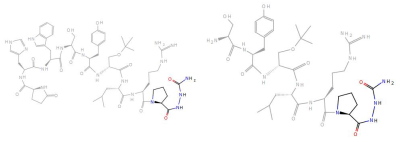	173.1044	173.1033	-6.11
MATCH	11.1	253.1668	253.1659	-3.55	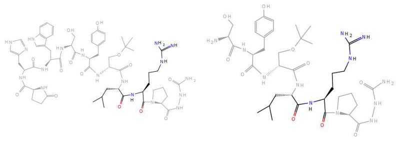	253.1659	253.1659	0.20
MATCH	7.1	269.1718	269.1720	0.80	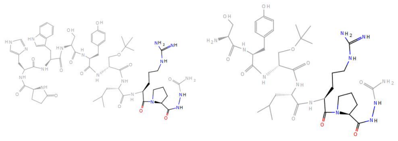	269.1716	269.1720	1.84
MATCH	5.1	286.1994	286.1986	-2.95	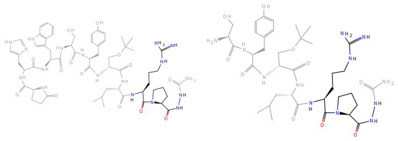	286.1988	286.1986	-0.56
MATCH	4.5	295.1519	295.1513	-2.03	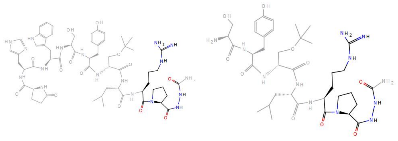	295.1508	295.1513	1.62

Metabolite: M2 -434 RT=1.91

Type	score	sub. m/z observed	sub. m/z calculated	sub ppm		met. m/z observed	met. m/z calculated	met. ppm
MATCH	4.2	312.1797	312.1779	-5.95	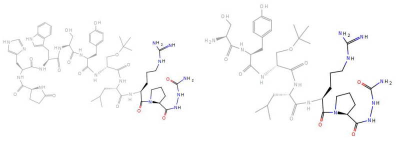	312.1772	312.1779	2.11
MATCH	15.0	329.2040	329.2044	1.40	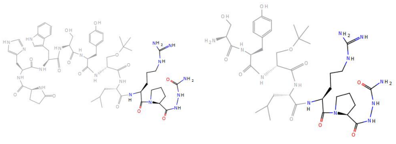	329.2013	329.2044	9.51
MATCH	3.4	399.2820	399.2827	1.57	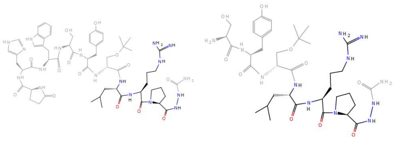	399.2828	399.2827	-0.43
MATCH	9.4	442.2882	442.2885	0.57	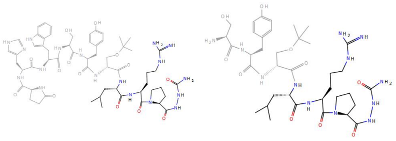	442.2885	442.2885	-0.15
MATCH	22.4	494.2139	494.2146	1.56	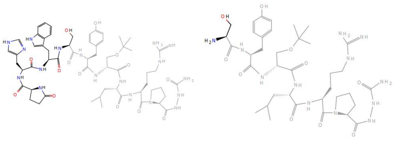	60.0453	60.0444	-15.4
MATCH	9.0	504.2000	504.1990	-2.08	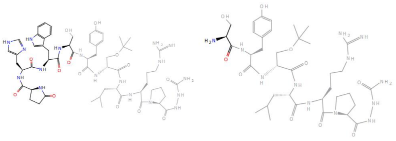	70.0295	70.0287	-11.2
MATCH	8.2	512.2925	512.2940	2.83	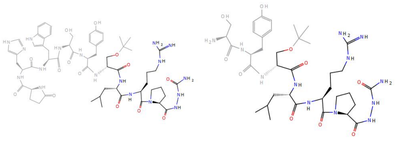	512.2946	512.2940	-1.25
MATCH	43.6	529.3216	529.3205	-2.14	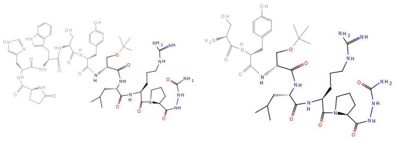	529.3206	529.3205	-0.14
MATCH	7.4	598.2934	598.2914	-3.37	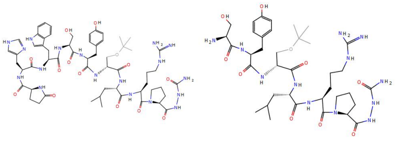	381.2062	381.2063	0.31

Metabolite: M2 -434 RT=1.91

Type	score	sub. m/z observed	sub. m/z calculated	sub ppm		met. m/z observed	met. m/z calculated	met. ppm
MATCH	4.5	754.2917	754.2944	3.50	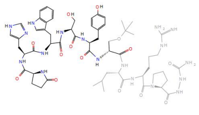 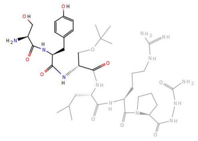	320.1236	320.1241	1.64
MATCH	4.0	772.3004	772.3049	5.83	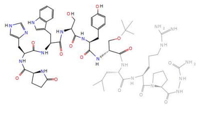 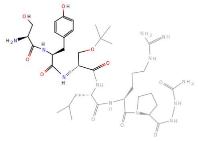	338.1344	338.1347	0.77
MISMATCH	-26.8	173.1043	173.1033	-6.02	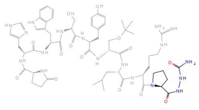	87.0560	87.0560	0.00
MISMATCH	-9.6	692.3850	692.3838	-1.75	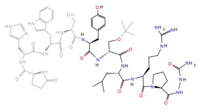	346.6959	346.6959	0.00
MET_MATCH					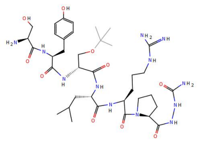	390.2121	390.2116	-1.46
MET_MATCH					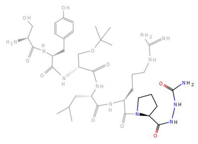	87.0560	87.0553	-8.20
MET_MATCH					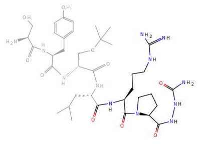	178.0863	178.0955	51.53
MET_MATCH					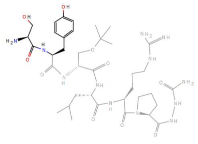	223.1077	223.1077	-0.07
MET_MATCH					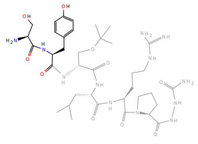	251.1025	251.1026	0.46

Metabolite: M2 -434 RT=1.91

Type	score	sub. m/z observed	sub. m/z calculated	sub ppm			met. m/z observed	met. m/z calculated	met. ppm
MET_MATCH						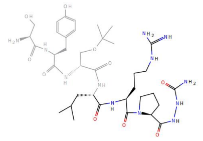	425.2614	425.2619	1.15

MS (+) FT

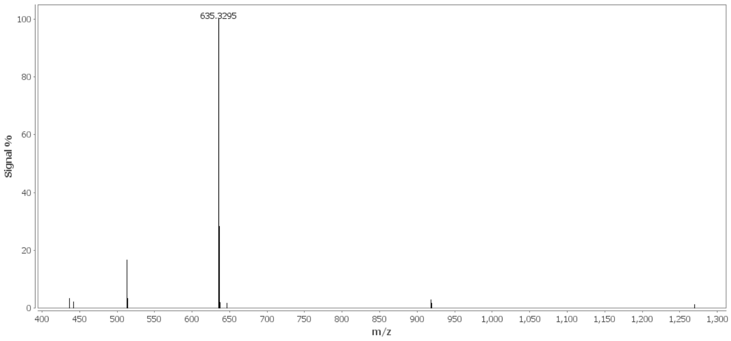

MS (+) FT

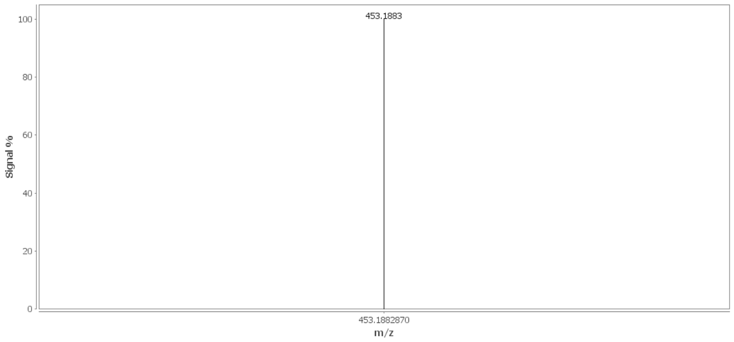

MS2 (+) FT activ = HCD:ce =

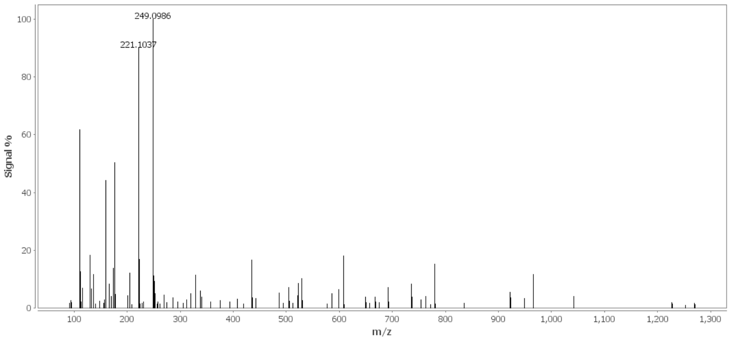

MS2 (+) FT activ = HCD:ce =

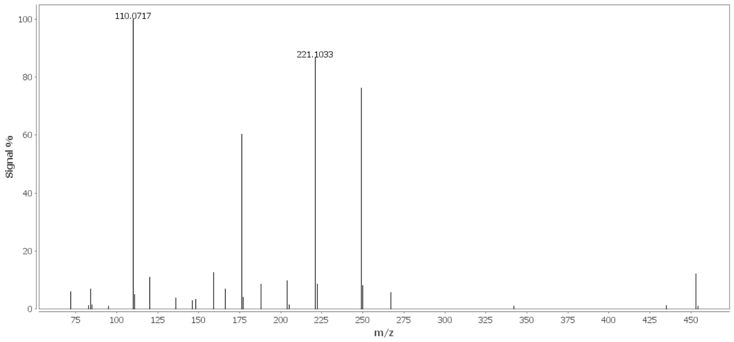

Metabolite: M1 -816 RT=0.46

Type	score	sub. m/z observed	sub. m/z calculated	sub ppm			met. m/z observed	met. m/z calculated	met. ppm
MATCH	200.0	635.3294	635.3280	-2.16	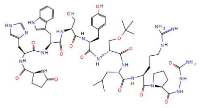	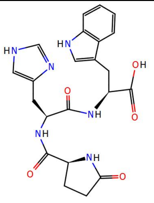	453.1883	453.1881	-0.43
MATCH	3.0	95.0612	95.0604	-9.12	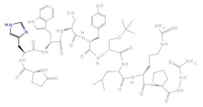	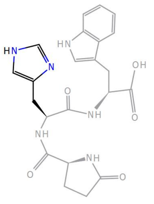	95.0610	95.0604	-6.63
MATCH	160.2	110.0719	110.0713	-5.85	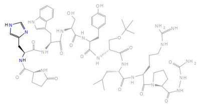	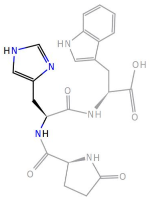	110.0717	110.0713	-3.72

Metabolite: M1 -816 RT=0.46

Type	score	sub. m/z observed	sub. m/z calculated	sub ppm			met. m/z observed	met. m/z calculated	met. ppm
MATCH	53.2	159.0920	159.0917	-2.36	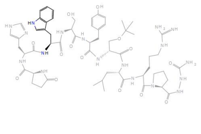	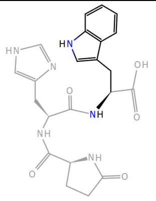	159.0920	159.0917	-2.02
MATCH	14.7	166.0615	166.0611	-2.32	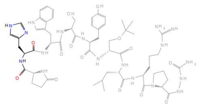	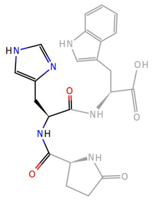	166.0607	166.0611	2.62
MATCH	174.5	221.1038	221.1033	-2.17	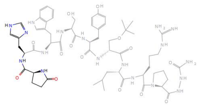	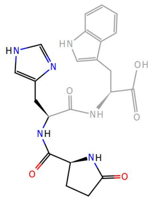	221.1033	221.1033	-0.14
MATCH	176.2	249.0987	249.0982	-1.88	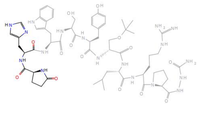	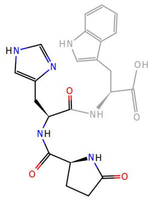	249.0981	249.0982	0.59
MISMATCH	-17.5	136.0762	136.0757	-3.51	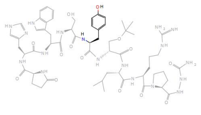		136.0758	136.0758	0.00
MET_MATCH						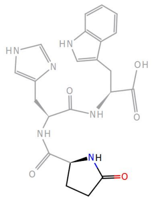	84.0451	84.0444	-8.27
MET_MATCH						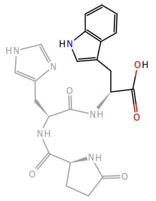	188.0702	188.0706	2.40
MET_MATCH						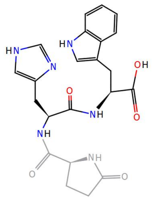	342.1590	342.1561	-8.48
MET_MATCH						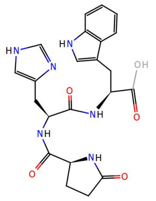	435.1789	435.1775	-3.21

Metabolite: M1 -816 RT=0.46

Type	score	sub. m/z observed	sub. m/z calculated	sub ppm		met. m/z observed	met. m/z calculated	met. ppm
MET_MATCH					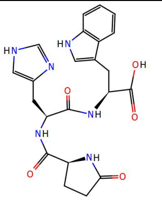	453.1879	453.1881	0.50

MS (+) FT

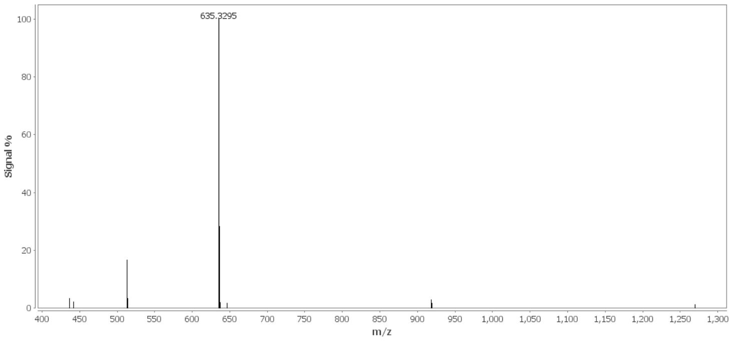

MS (+) FT

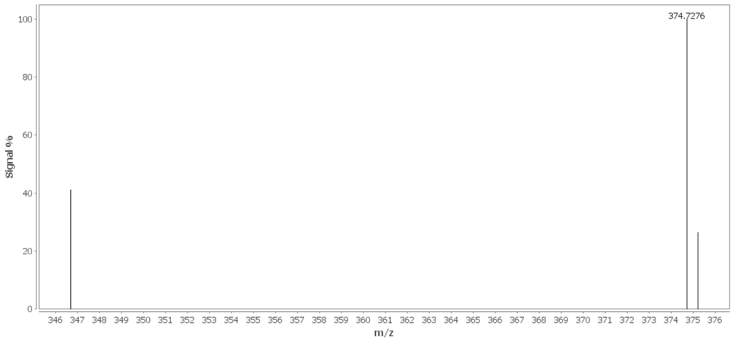

MS2 (+) FT activ = HCD:ce =

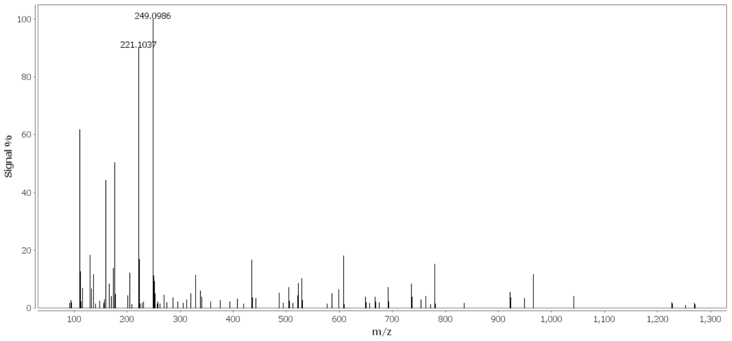

MS2 (+) FT activ = HCD:ce =

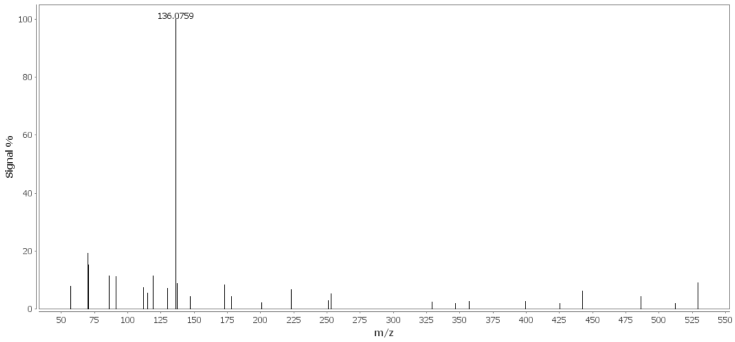

Metabolite: M3 -521 RT=1.95

Type	score	sub. m/z observed	sub. m/z calculated	sub ppm		met. m/z observed	met. m/z calculated	met. ppm
MATCH	200.0	635.3294	635.3280	-2.16	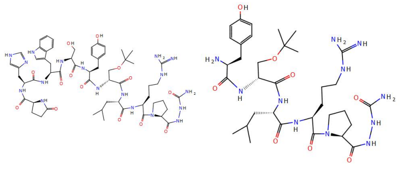	374.7276	374.7269	-2.07
MATCH	20.0	112.0875	112.0869	-5.45	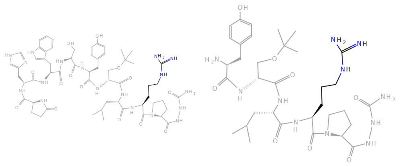	112.0874	112.0869	-4.60
MATCH	13.0	115.0873	115.0866	-5.96	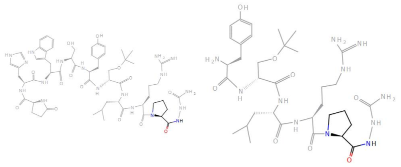	115.0871	115.0866	-4.68

Metabolite: M3 -521 RT=1.95

Type	score	sub. m/z observed	sub. m/z calculated	sub ppm		met. m/z observed	met. m/z calculated	met. ppm
MATCH	25.2	130.0980	130.0975	-3.70	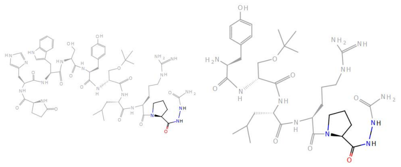	130.0978	130.0975	-2.11
MATCH	14.5	173.1043	173.1033	-6.02	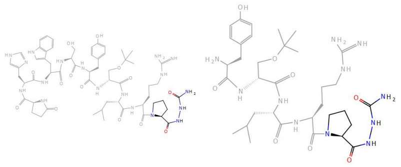	173.1038	173.1033	-3.00
MATCH	10.7	253.1668	253.1659	-3.55	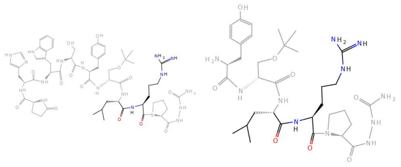	253.1666	253.1659	-2.56
MATCH	12.9	329.2040	329.2044	1.40	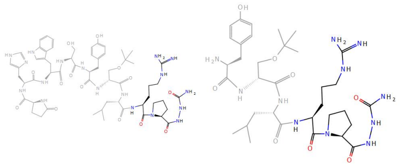	329.2034	329.2044	3.17
MATCH	4.6	399.2820	399.2827	1.57	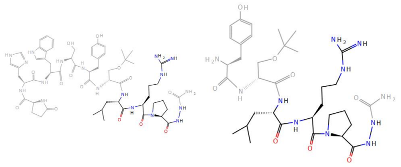	399.2811	399.2827	3.92
MATCH	10.3	442.2882	442.2885	0.57	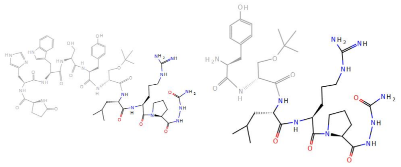	442.2877	442.2885	1.78
MATCH	3.7	512.2925	512.2940	2.83	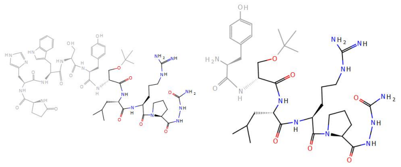	512.2923	512.2940	3.24
MATCH	20.6	529.3216	529.3205	-2.14	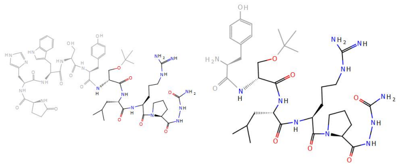	529.3215	529.3205	-1.94
MATCH	19.3	607.2989	607.2967	-3.68	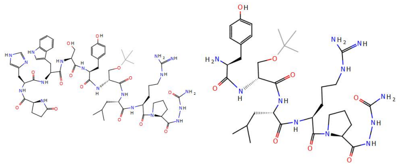	346.6948	346.6956	2.11

Metabolite: M3 -521 RT=1.95

Type	score	sub. m/z observed	sub. m/z calculated	sub ppm		met. m/z observed	met. m/z calculated	met. ppm
MATCH	4.8	772.3004	772.3049	5.83	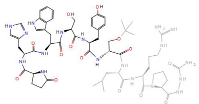 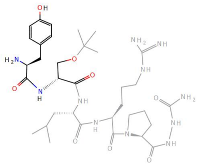	251.1027	251.1026	-0.23
MET_MATCH					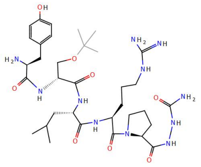	346.6961	346.6956	-1.61
MET_MATCH					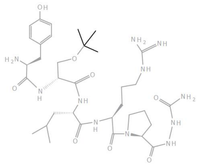	57.0708	57.0699	-17.0
MET_MATCH					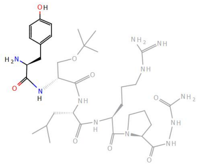	91.0547	91.0522	-27.8
MET_MATCH					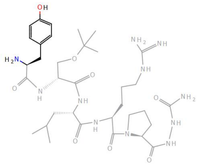	136.0759	136.0757	-1.52
MET_MATCH					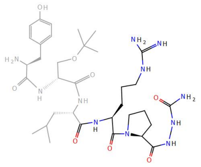	178.0864	178.0955	50.91
MET_MATCH					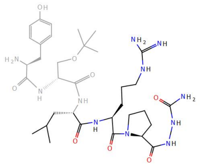	425.2649	425.2619	-7.04
