# Supplementary material for: Software-aided approach to investigate peptide structure and metabolic susceptibility of amide bonds in peptide drugs based on high resolution mass spectrometry
Source: PLoS One. 2017 Nov 1;12(11):e0186461. doi: 10.1371/journal.pone.0186461 (PMC5665424; doi:10.1371/journal.pone.0186461)
Supplement: S1 File — (ZIP) [file pone.0186461.s007.zip › SFiles/S8_File.pdf]

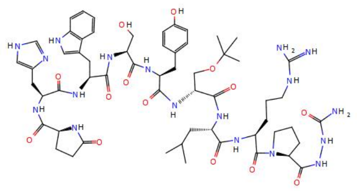

Goserelin

Property name	Property value
Time	0min, 5min, 15min, 45min, 120min
Instrument	ThermoQAPlus
Acquisition Mode	ddMS2
Matrix	pepsin

Chromatograms

Time=0min

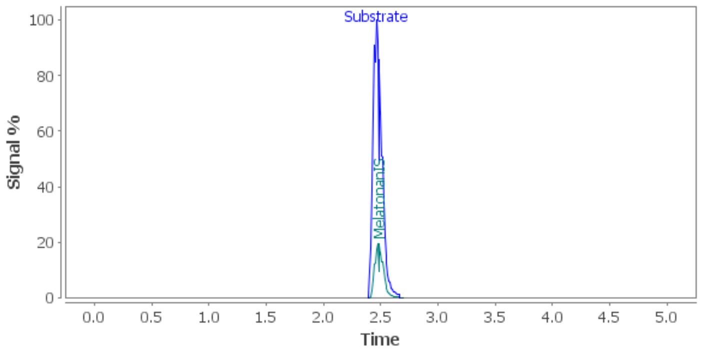

Time=5min

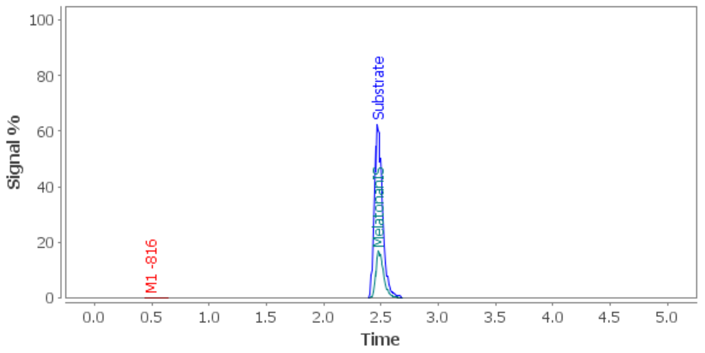

Time=15min

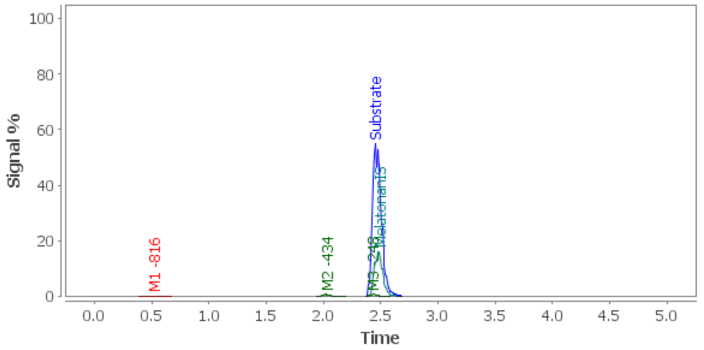

Time=45min

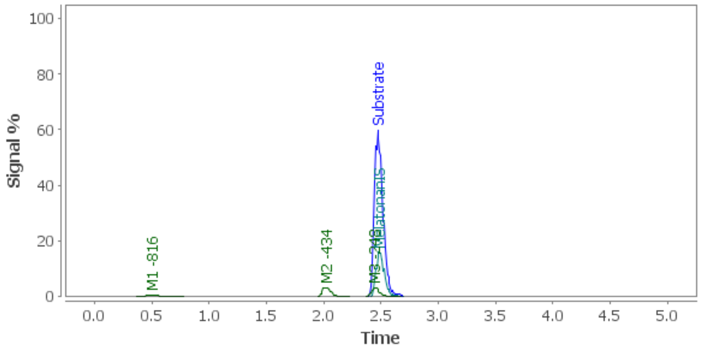

Time=120min

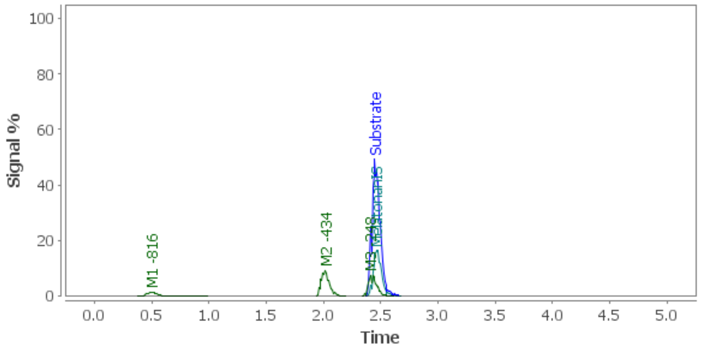

Custom Charts

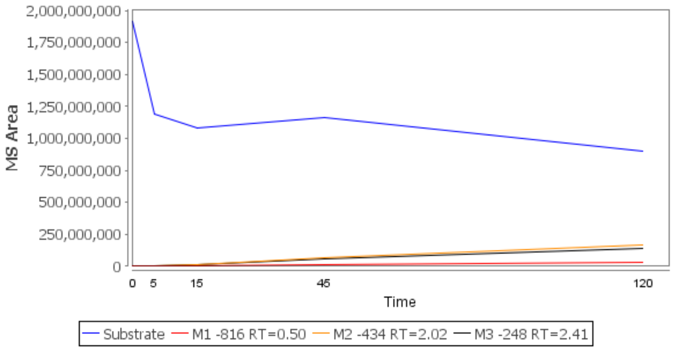

Fragmentation

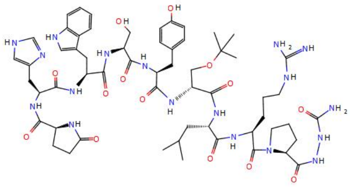

Goserelin

MS (+) FT

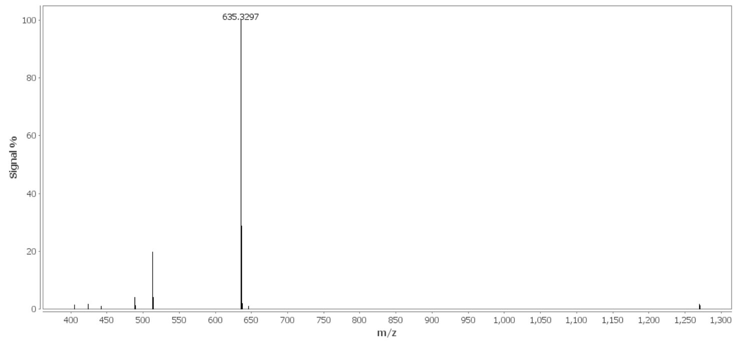

MS (+) FT

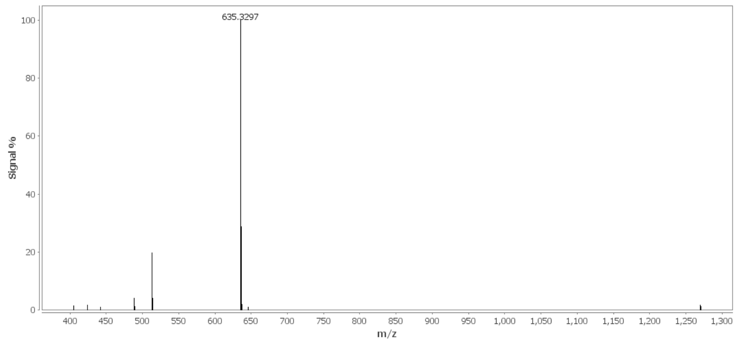

MS2 (+) FT activ = HCD:ce =

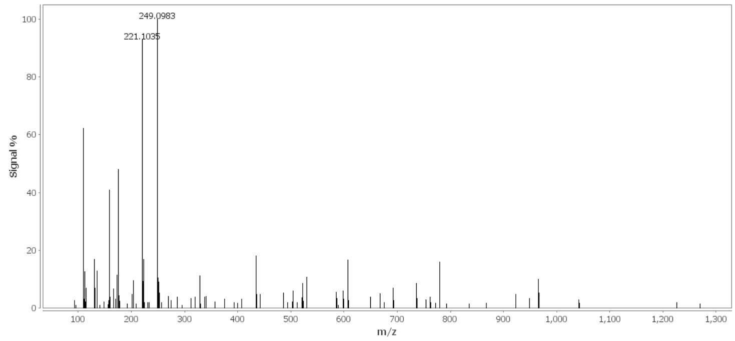

MS2 (+) FT activ = HCD:ce =

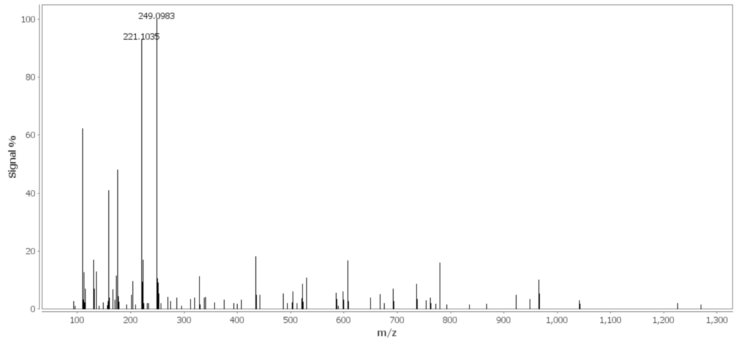

Metabolite: Substrate

Type	score	sub. m/z observed	sub. m/z calculated	sub ppm		met. m/z observed	met. m/z calculated	met. ppm
MATCH	11.0	1269.6524	1269.6487	-2.87	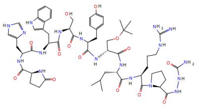 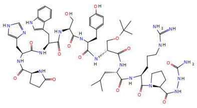	1269.6524	1269.6487	-2.87
MATCH	101.6	1269.6513	1269.6487	-2.00	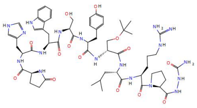 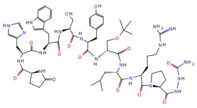	1269.6513	1269.6487	-2.00
MATCH	6.7	1226.6461	1226.6429	-2.64	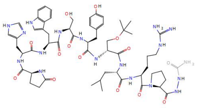 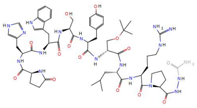	1226.6461	1226.6429	-2.64

Metabolite: Substrate

Type	score	sub. m/z observed	sub. m/z calculated	sub ppm			met. m/z observed	met. m/z calculated	met. ppm
MATCH	8.8	948.4637	948.4686	5.21	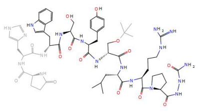	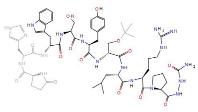	948.4637	948.4686	5.21
MATCH	28.0	779.4166	779.4159	-0.91	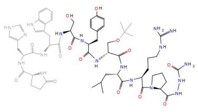	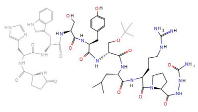	779.4166	779.4159	-0.91
MATCH	4.2	772.3000	772.3049	6.33	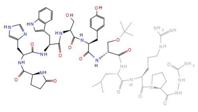	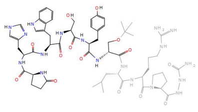	772.3000	772.3049	6.33
MATCH	6.7	762.3868	762.3893	3.34	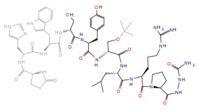	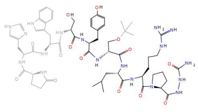	762.3868	762.3893	3.34
MATCH	6.0	754.2944	754.2944	-0.06	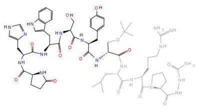	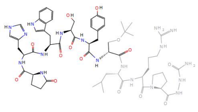	754.2944	754.2944	-0.06
MISMATCH	22.4	692.3824	692.3838	2.14	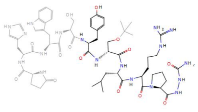	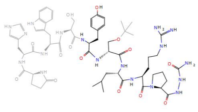	692.3824	692.3838	2.14
MATCH	4.6	675.3529	675.3573	6.54	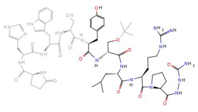	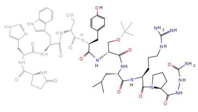	675.3529	675.3573	6.54
MATCH	7.2	667.2636	667.2623	-1.89	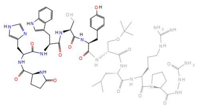	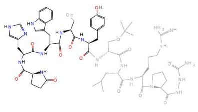	667.2636	667.2623	-1.89
MATCH	200.0	635.3297	635.3280	-2.63	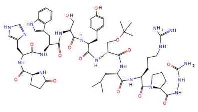	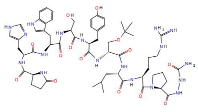	635.3297	635.3280	-2.63

Metabolite: Substrate

Type	score	sub. m/z observed	sub. m/z calculated	sub ppm		met. m/z observed	met. m/z calculated	met. ppm
MISMATCH	-18.3	607.2987	607.2967	-3.36	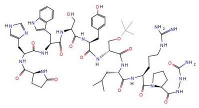 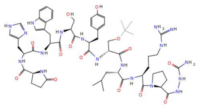	607.2987	607.2967	-3.36
MATCH	11.4	598.7893	598.7834	-9.83	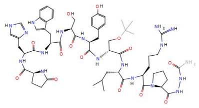 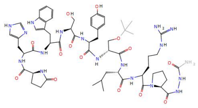	598.7893	598.7834	-9.83
MATCH	7.2	598.2875	598.2914	6.62	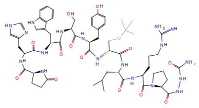 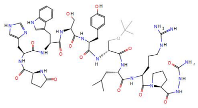	598.2875	598.2914	6.62
MATCH	3.1	589.7732	589.7781	8.32	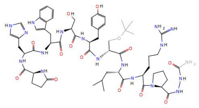 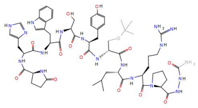	589.7732	589.7781	8.32
MATCH	2.1	585.3806	585.3831	4.28	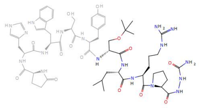 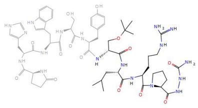	585.3806	585.3831	4.28
MATCH	45.2	529.3213	529.3205	-1.52	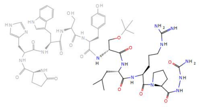 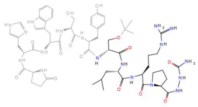	529.3213	529.3205	-1.52
MATCH	16.3	522.2089	522.2096	1.29	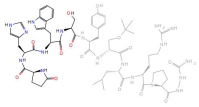 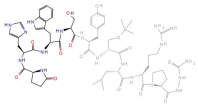	522.2089	522.2096	1.29
MATCH	8.7	512.2909	512.2940	6.03	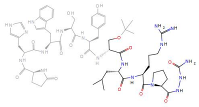 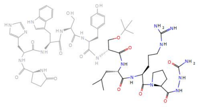	512.2909	512.2940	6.03
MATCH	8.9	504.1999	504.1990	-1.84	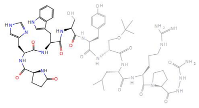 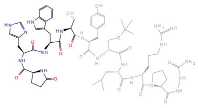	504.1999	504.1990	-1.84

Metabolite: Substrate

Type	score	sub. m/z observed	sub. m/z calculated	sub ppm			met. m/z observed	met. m/z calculated	met. ppm
MISMATCH	-3.6	503.2591	503.2669	15.48	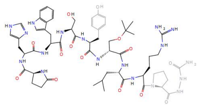	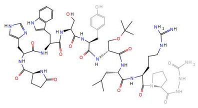	503.2591	503.2669	15.48
MATCH	22.0	494.2138	494.2146	1.76	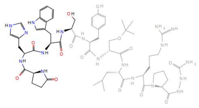	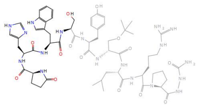	494.2138	494.2146	1.76
MATCH	11.8	442.2890	442.2885	-1.13	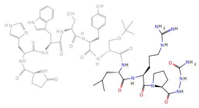	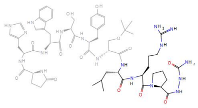	442.2890	442.2885	-1.13
MATCH	21.6	435.1780	435.1775	-1.08	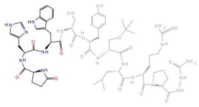	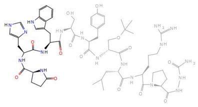	435.1780	435.1775	-1.08
MATCH	101.7	423.8885	423.8878	-1.81	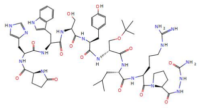	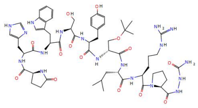	423.8885	423.8878	-1.81
MATCH	103.0	407.1827	407.1826	-0.32	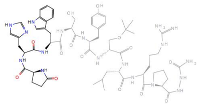	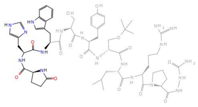	407.1827	407.1826	-0.32
MISMATCH	-32.5	405.2009	405.2002	-1.64	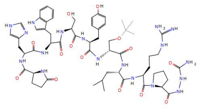	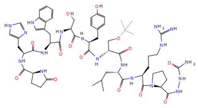	405.2009	405.2002	-1.64
MATCH	3.9	399.2825	399.2827	0.29	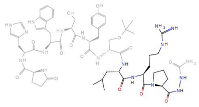	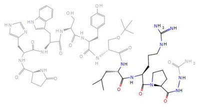	399.2825	399.2827	0.29
MATCH	21.1	329.2042	329.2044	0.51	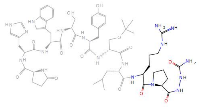	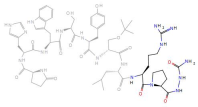	329.2042	329.2044	0.51

Metabolite: Substrate

Type	score	sub. m/z observed	sub. m/z calculated	sub ppm		met. m/z observed	met. m/z calculated	met. ppm
MATCH	5.9	312.1764	312.1779	4.69	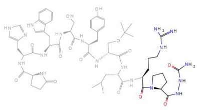 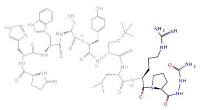	312.1764	312.1779	4.69
MATCH	2.6	295.1522	295.1513	-3.05	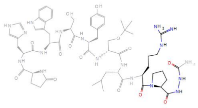 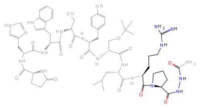	295.1522	295.1513	-3.05
MATCH	7.3	286.1988	286.1986	-0.83	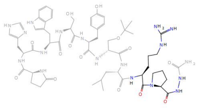 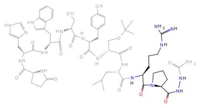	286.1988	286.1986	-0.83
MATCH	10.4	274.1190	274.1186	-1.58	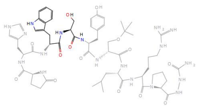 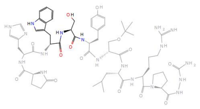	274.1190	274.1186	-1.58
MATCH	8.9	269.1716	269.1720	1.60	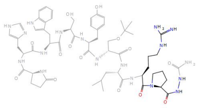 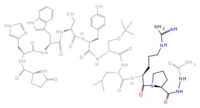	269.1716	269.1720	1.60
MATCH	12.1	253.1659	253.1659	-0.17	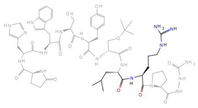 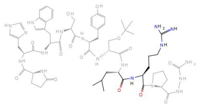	253.1659	253.1659	-0.17
MATCH	173.6	249.0983	249.0982	-0.52	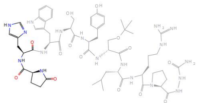 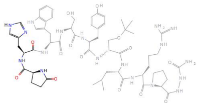	249.0983	249.0982	-0.52
MATCH	172.7	221.1035	221.1033	-0.73	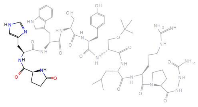 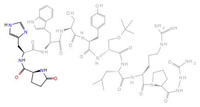	221.1035	221.1033	-0.73
MISMATCH	19.5	173.1039	173.1033	-3.57	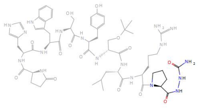 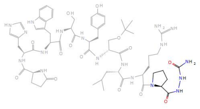	173.1039	173.1033	-3.57

Metabolite: Substrate

Type	score	sub. m/z observed	sub. m/z calculated	sub ppm		met. m/z observed	met. m/z calculated	met. ppm
MATCH	42.5	170.0602	170.0600	-0.93	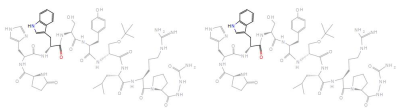	170.0602	170.0600	-0.93
MATCH	13.5	166.0614	166.0611	-1.60	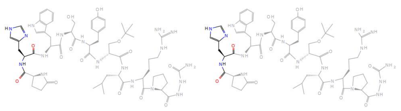	166.0614	166.0611	-1.60
MATCH	52.1	159.0918	159.0917	-1.01	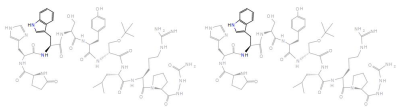	159.0918	159.0917	-1.01
MATCH	5.2	157.1086	157.1084	-1.45	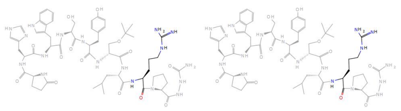	157.1086	157.1084	-1.45
MATCH	16.2	156.0806	156.0768	-24.6	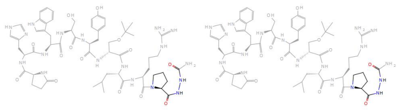	156.0806	156.0768	-24.6
MATCH	16.2	156.0806	156.0768	-24.6	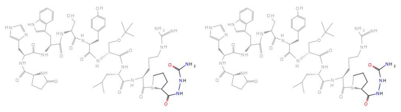	156.0806	156.0768	-24.6
MATCH	2.4	140.0823	140.0818	-3.36	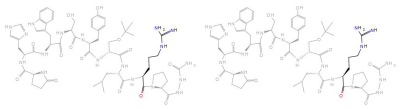	140.0823	140.0818	-3.36
MATCH	84.9	136.0759	136.0757	-1.87	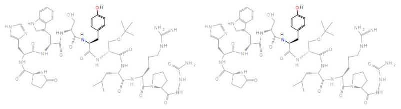	136.0759	136.0757	-1.87
MATCH	35.4	130.0977	130.0975	-1.82	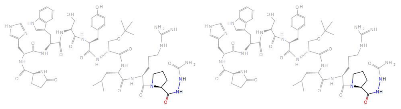	130.0977	130.0975	-1.82

Metabolite: Substrate

Type	score	sub. m/z observed	sub. m/z calculated	sub ppm			met. m/z observed	met. m/z calculated	met. ppm
MATCH	73.0	130.0653	130.0575	-59.9	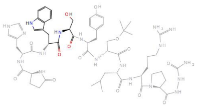	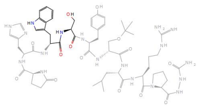	130.0653	130.0575	-59.9
MATCH	17.3	115.0871	115.0866	-4.19	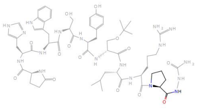	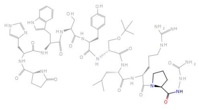	115.0871	115.0866	-4.19
MATCH	28.0	112.0874	112.0869	-4.54	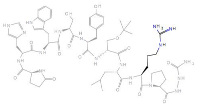	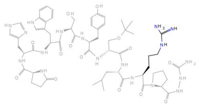	112.0874	112.0869	-4.54
MATCH	162.2	110.0718	110.0713	-4.42	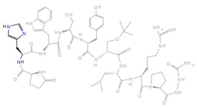	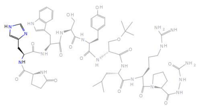	110.0718	110.0713	-4.42
MATCH	4.8	95.0612	95.0604	-9.15	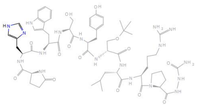	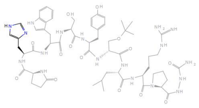	95.0612	95.0604	-9.15

MS (+) FT

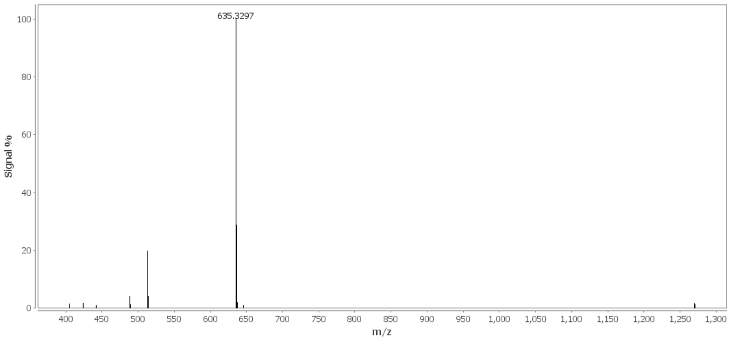

MS (+) FT

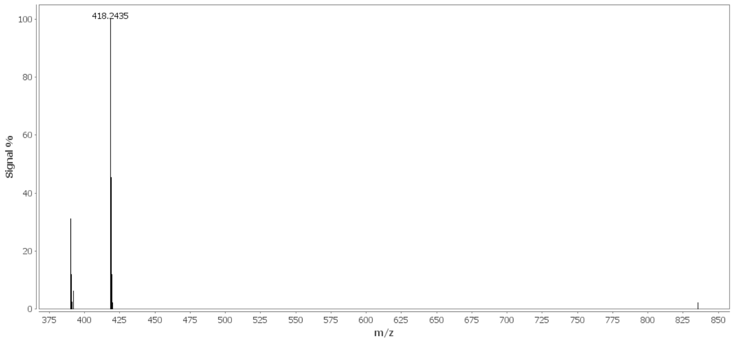

MS2 (+) FT activ = HCD:ce =

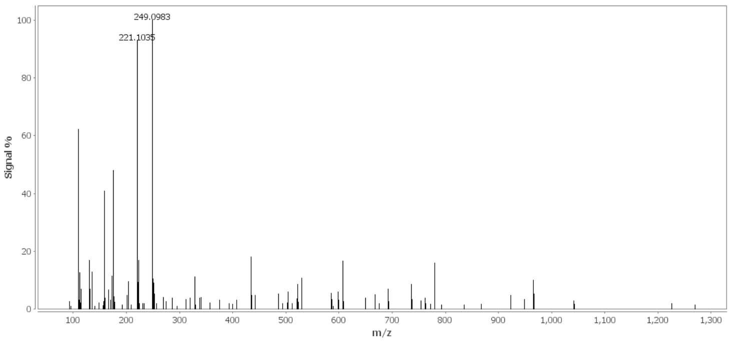

MS2 (+) FT activ = HCD:ce =

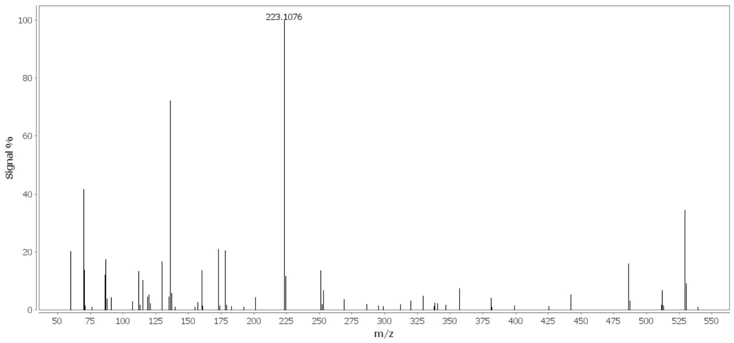

Metabolite: M2 -434 RT=2.02

Type	score	sub. m/z observed	sub. m/z calculated	sub ppm			met. m/z observed	met. m/z calculated	met. ppm
MATCH	101.7	423.8885	423.8878	-1.81	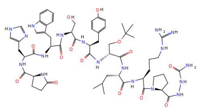	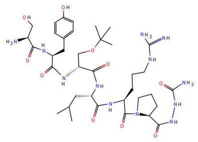	418.2435	418.2429	-1.55
MATCH	101.7	423.8885	423.8878	-1.81	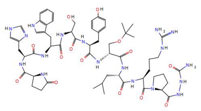	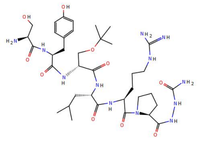	418.2435	418.2429	-1.55
						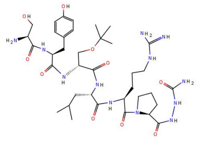	418.2435	418.2429	-1.55
MATCH	3.8	423.8885	423.8878	-1.81	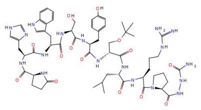	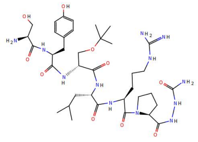	835.4807	835.4785	-2.65
MATCH	3.8	423.8885	423.8878	-1.81	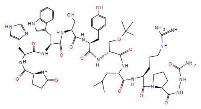	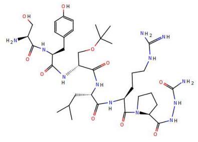	835.4807	835.4785	-2.65
						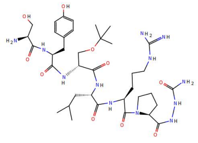	835.4807	835.4785	-2.65
MATCH	200.0	635.3297	635.3280	-2.63	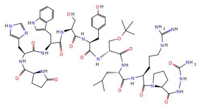	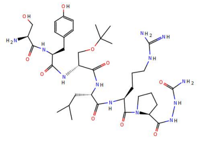	418.2435	418.2429	-1.55
MATCH	200.0	635.3297	635.3280	-2.63	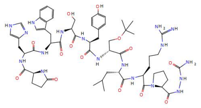	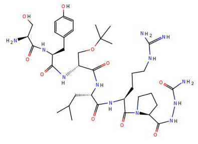	418.2435	418.2429	-1.55
						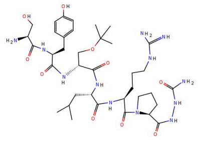	418.2435	418.2429	-1.55

Metabolite: M2 -434 RT=2.02

Type	score	sub. m/z observed	sub. m/z calculated	sub ppm		met. m/z observed	met. m/z calculated	met. ppm
MATCH	102.2	635.3297	635.3280	-2.63	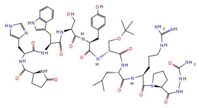 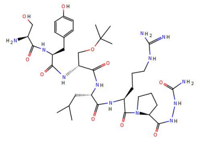	835.4807	835.4785	-2.65
MATCH	102.2	635.3297	635.3280	-2.63	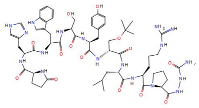 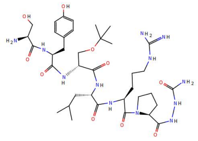	835.4807	835.4785	-2.65
					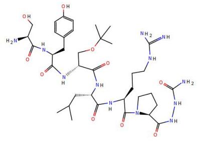	835.4807	835.4785	-2.65
MATCH	101.6	1269.6513	1269.6487	-2.00	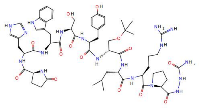 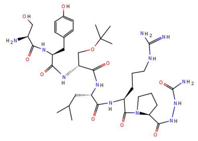	418.2435	418.2429	-1.55
MATCH	101.6	1269.6513	1269.6487	-2.00	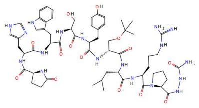 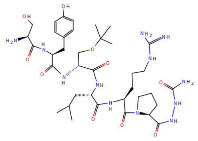	418.2435	418.2429	-1.55
					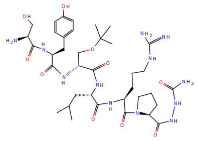	418.2435	418.2429	-1.55
MATCH	3.8	1269.6513	1269.6487	-2.00	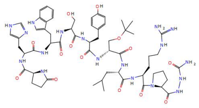 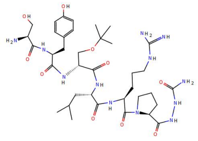	835.4807	835.4785	-2.65
MATCH	3.8	1269.6513	1269.6487	-2.00	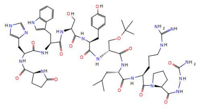 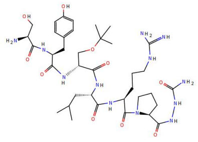	835.4807	835.4785	-2.65
					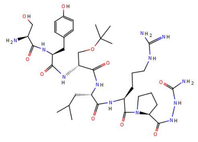	835.4807	835.4785	-2.65

Metabolite: M2 -434 RT=2.02

Type	score	sub. m/z observed	sub. m/z calculated	sub ppm		met. m/z observed	met. m/z calculated	met. ppm
MATCH	25.9	112.0874	112.0869	-4.54	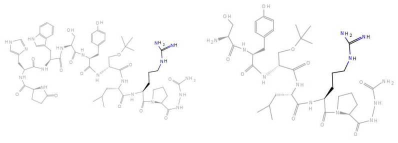	112.0874	112.0869	-3.85
MATCH	17.3	115.0871	115.0866	-4.19	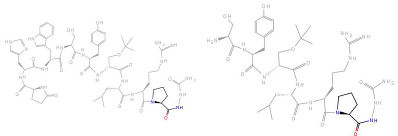	115.0869	115.0866	-2.69
MATCH	33.3	130.0977	130.0975	-1.82	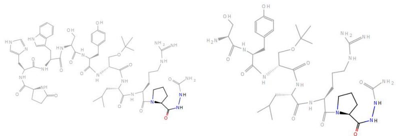	130.0976	130.0975	-1.04
MATCH	84.9	136.0759	136.0757	-1.87	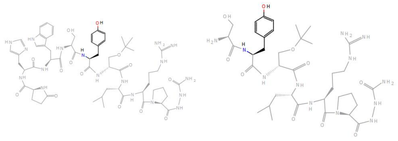	136.0758	136.0757	-1.14
MATCH	2.1	140.0823	140.0818	-3.36	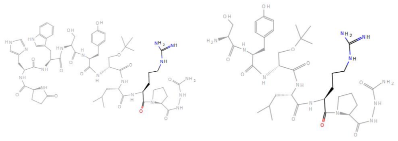	140.0819	140.0818	-0.66
MATCH	5.2	157.1086	157.1084	-1.45	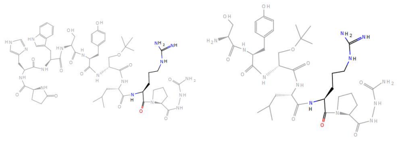	157.1085	157.1084	-0.45
MATCH	16.7	173.1039	173.1033	-3.57	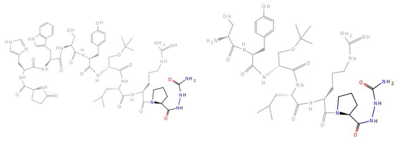	173.1043	173.1033	-5.88
MATCH	11.9	253.1659	253.1659	-0.17	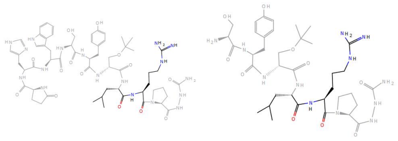	253.1657	253.1659	0.71
MATCH	7.7	269.1716	269.1720	1.60	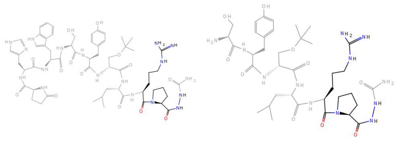	269.1718	269.1720	0.96

Metabolite: M2 -434 RT=2.02

Type	score	sub. m/z observed	sub. m/z calculated	sub ppm		met. m/z observed	met. m/z calculated	met. ppm
MATCH	5.6	286.1988	286.1986	-0.83	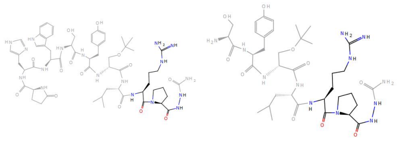	286.1980	286.1986	2.06
MATCH	2.6	295.1522	295.1513	-3.05	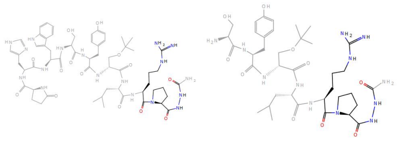	295.1508	295.1513	1.72
MATCH	5.1	312.1764	312.1779	4.69	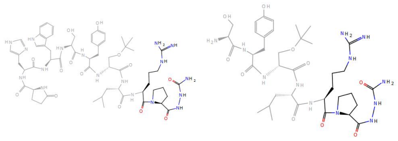	312.1763	312.1779	5.01
MATCH	16.0	329.2042	329.2044	0.51	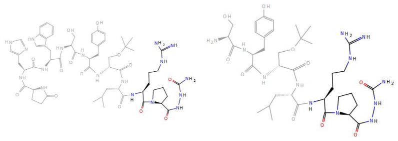	329.2015	329.2044	8.78
MATCH	3.1	399.2825	399.2827	0.29	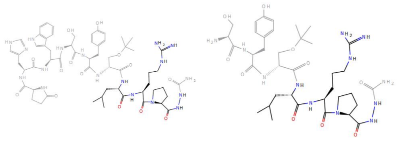	399.2819	399.2827	1.92
MATCH	10.0	442.2890	442.2885	-1.13	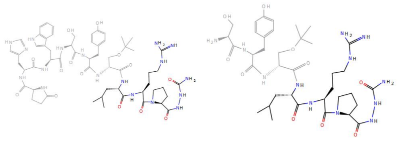	442.2882	442.2885	0.72
MATCH	22.0	494.2138	494.2146	1.76	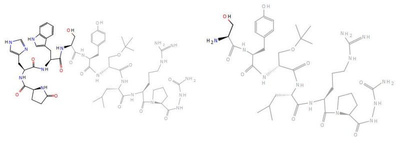	60.0453	60.0444	-15.3
MATCH	8.1	504.1999	504.1990	-1.84	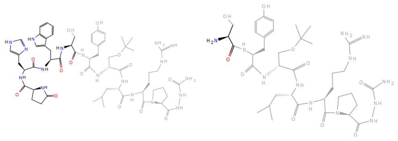	70.0296	70.0287	-11.7
MATCH	8.7	512.2909	512.2940	6.03	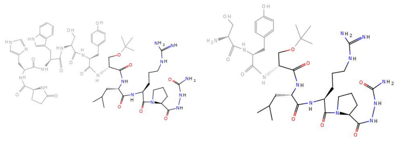	512.2951	512.2940	-2.19

Metabolite: M2 -434 RT=2.02

Type	score	sub. m/z observed	sub. m/z calculated	sub ppm		met. m/z observed	met. m/z calculated	met. ppm
MATCH	45.2	529.3213	529.3205	-1.52	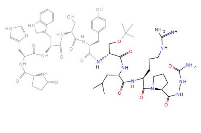 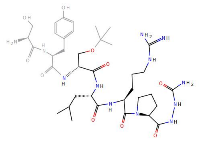	529.3208	529.3205	-0.52
MATCH	7.2	598.2875	598.2914	6.62	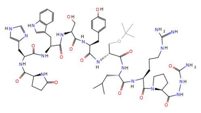 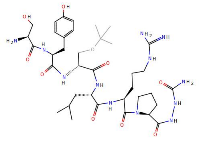	381.2062	381.2063	0.20
MATCH	6.0	754.2944	754.2944	-0.06	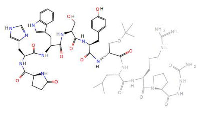 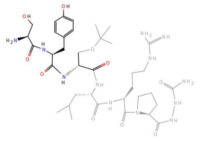	320.1239	320.1241	0.62
MATCH	4.2	772.3000	772.3049	6.33	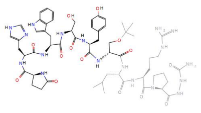 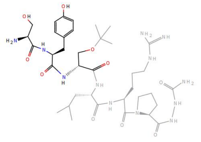	338.1343	338.1347	1.06
MISMATCH	-32.5	405.2009	405.2002	-1.64	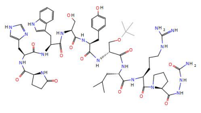	390.2122	390.2122	0.00
MISMATCH	-26.6	173.1039	173.1033	-3.57	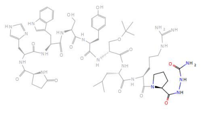	87.0560	87.0560	0.00
MISMATCH	-8.7	692.3824	692.3838	2.14	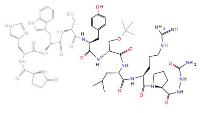	346.6950	346.6950	0.00
MET_MATCH					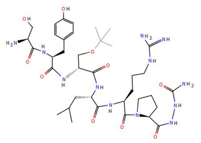	390.2122	390.2116	-1.58
MET_MATCH					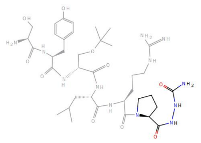	87.0560	87.0553	-7.79

Metabolite: M2 -434 RT=2.02

Type	score	sub. m/z observed	sub. m/z calculated	sub ppm		met. m/z observed	met. m/z calculated	met. ppm
MET_MATCH					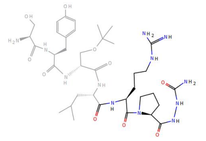	178.0863	178.0955	51.66
MET_MATCH					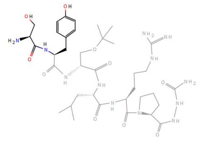	223.1076	223.1077	0.31
MET_MATCH					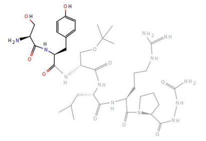	251.1027	251.1026	-0.10
MET_MATCH					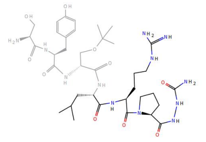	425.2609	425.2619	2.52

MS (+) FT

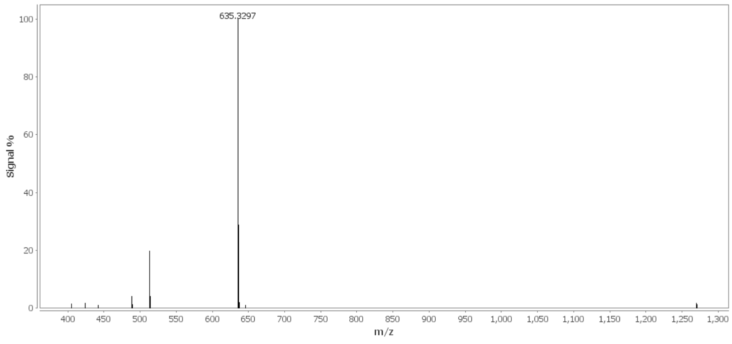

MS (+) FT

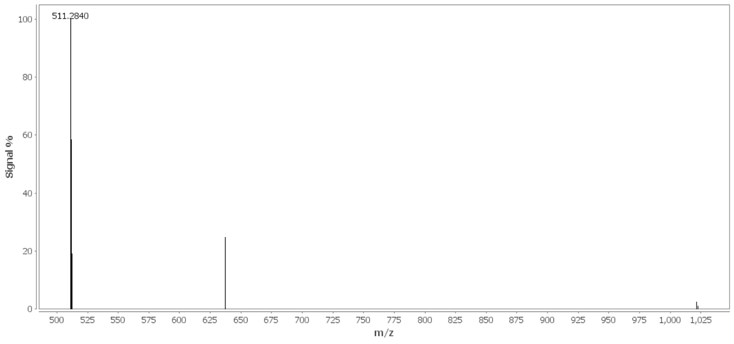

MS2 (+) FT activ = HCD:ce =

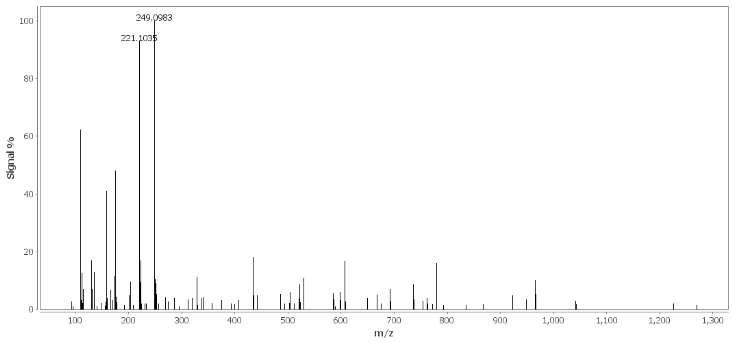

MS2 (+) FT activ = HCD:ce =

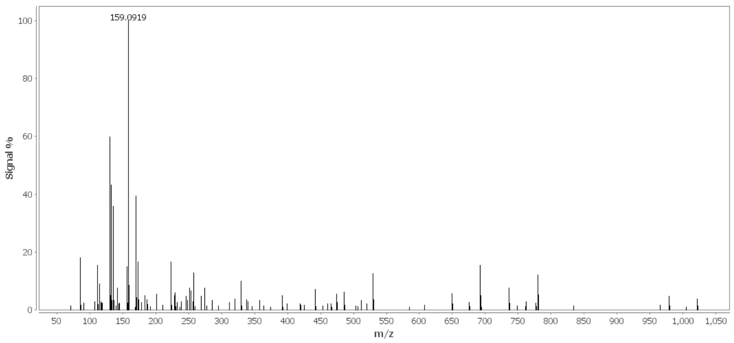

Metabolite: M3 -248 RT=2.41

Type	score	sub. m/z observed	sub. m/z calculated	sub ppm	met. m/z observed	met. m/z calculated	met. ppm

Metabolite: M3 -248 RT=2.41

Type	score	sub. m/z observed	sub. m/z calculated	sub ppm			met. m/z observed	met. m/z calculated	met. ppm
MATCH	101.7	423.8885	423.8878	-1.81	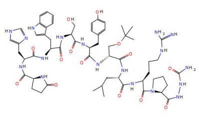	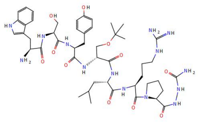	511.2840	511.2825	-2.89
MATCH	101.7	423.8885	423.8878	-1.81	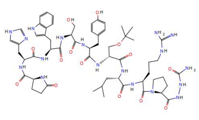	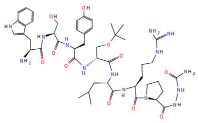	511.2840	511.2825	-2.89
						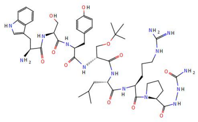	511.2840	511.2825	-2.89
MATCH	4.0	423.8885	423.8878	-1.81	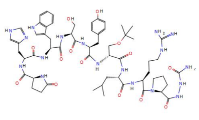	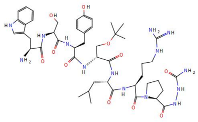	1021.5621	1021.5578	-4.24
MATCH	4.0	423.8885	423.8878	-1.81	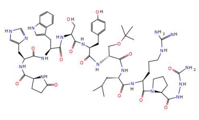	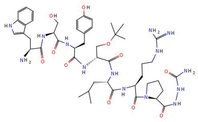	1021.5621	1021.5578	-4.24
						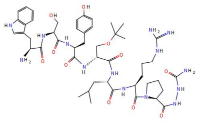	1021.5621	1021.5578	-4.24
MATCH	200.0	635.3297	635.3280	-2.63	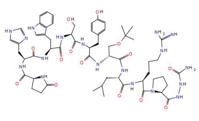	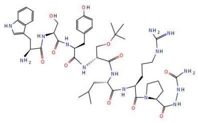	511.2840	511.2825	-2.89
MATCH	200.0	635.3297	635.3280	-2.63	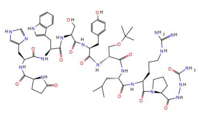	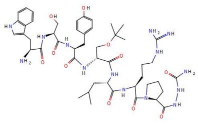	511.2840	511.2825	-2.89
						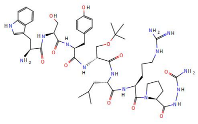	511.2840	511.2825	-2.89

Metabolite: M3 -248 RT=2.41

Type	score	sub. m/z observed	sub. m/z calculated	sub ppm		met. m/z observed	met. m/z calculated	met. ppm
MATCH	102.3	635.3297	635.3280	-2.63	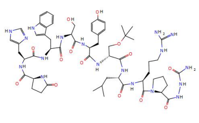 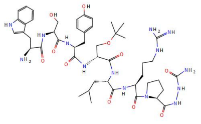	1021.5621	1021.5578	-4.24
MATCH	102.3	635.3297	635.3280	-2.63	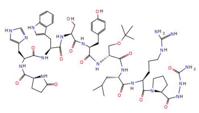 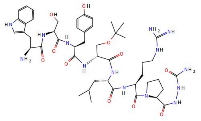	1021.5621	1021.5578	-4.24
					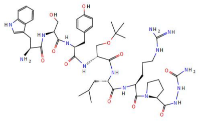	1021.5621	1021.5578	-4.24
MATCH	101.6	1269.6513	1269.6487	-2.00	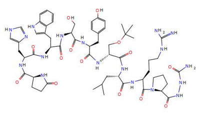 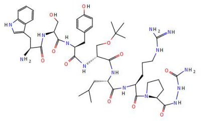	511.2840	511.2825	-2.89
MATCH	101.6	1269.6513	1269.6487	-2.00	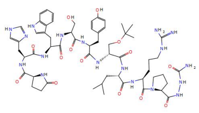 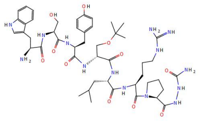	511.2840	511.2825	-2.89
					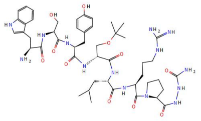	511.2840	511.2825	-2.89
MATCH	3.9	1269.6513	1269.6487	-2.00	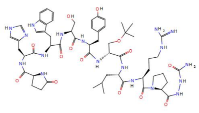 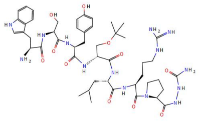	1021.5621	1021.5578	-4.24
MATCH	3.9	1269.6513	1269.6487	-2.00	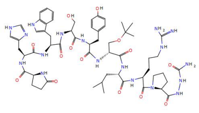 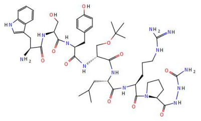	1021.5621	1021.5578	-4.24
					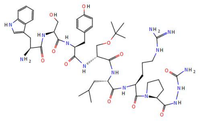	1021.5621	1021.5578	-4.24

Metabolite: M3 -248 RT=2.41

Type	score	sub. m/z observed	sub. m/z calculated	sub ppm		met. m/z observed	met. m/z calculated	met. ppm
MATCH	28.0	112.0874	112.0869	-4.54	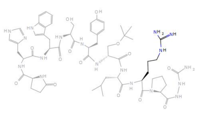 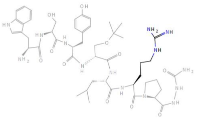	112.0874	112.0869	-4.42
MATCH	15.9	115.0871	115.0866	-4.19	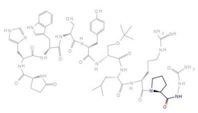 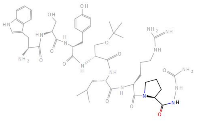	115.0870	115.0866	-3.85
MATCH	73.0	130.0653	130.0575	-59.9	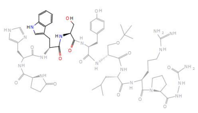 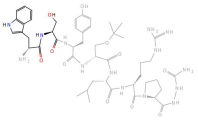	130.0654	130.0575	-60.6
MATCH	35.4	130.0977	130.0975	-1.82	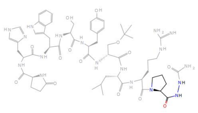 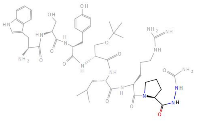	130.0977	130.0975	-1.75
MATCH	48.6	136.0759	136.0757	-1.87	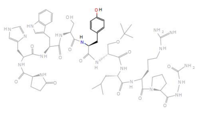 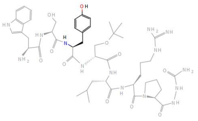	136.0759	136.0757	-1.73
MATCH	2.4	140.0823	140.0818	-3.36	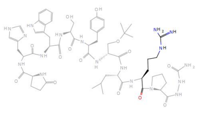 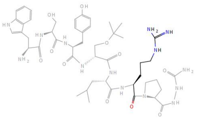	140.0820	140.0818	-0.84
MATCH	16.2	156.0806	156.0768	-24.6	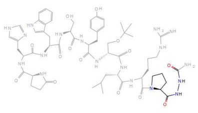 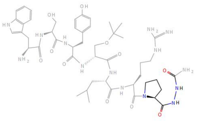	156.0810	156.0768	-27.0
MATCH	16.2	156.0806	156.0768	-24.6	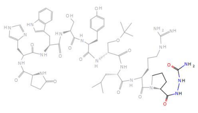 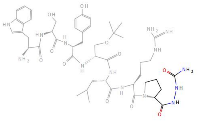	156.0810	156.0768	-27.0
MATCH	5.0	157.1086	157.1084	-1.45	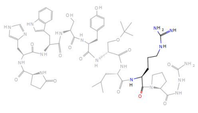 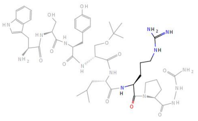	157.1084	157.1084	0.03

Metabolite: M3 -248 RT=2.41

Type	score	sub. m/z observed	sub. m/z calculated	sub ppm			met. m/z observed	met. m/z calculated	met. ppm
MATCH	42.5	170.0602	170.0600	-0.93	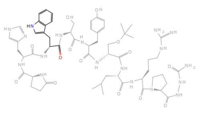	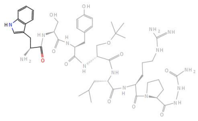	170.0601	170.0600	-0.46
MATCH	19.5	173.1039	173.1033	-3.57	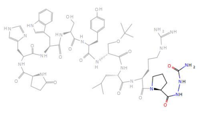	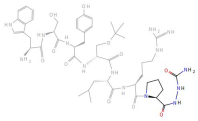	173.1040	173.1033	-4.06
MATCH	12.1	253.1659	253.1659	-0.17	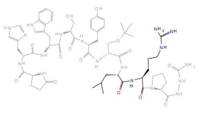	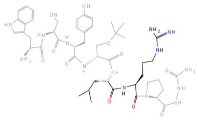	253.1661	253.1659	-0.75
MATCH	8.9	269.1716	269.1720	1.60	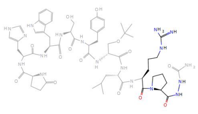	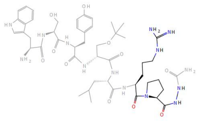	269.1719	269.1720	0.52
MATCH	10.4	274.1190	274.1186	-1.58	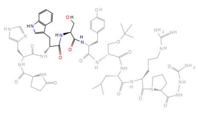	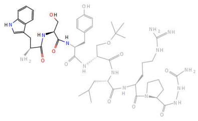	274.1187	274.1186	-0.20
						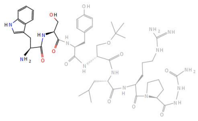	274.1187	274.1186	-0.20
MATCH	7.3	286.1988	286.1986	-0.83	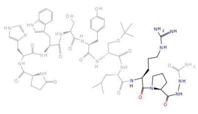	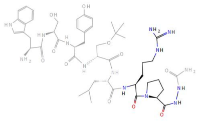	286.1991	286.1986	-1.63
MATCH	2.6	295.1522	295.1513	-3.05	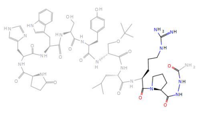	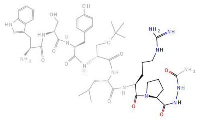	295.1513	295.1513	0.13
MATCH	5.9	312.1764	312.1779	4.69	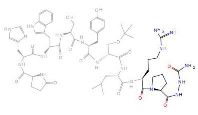	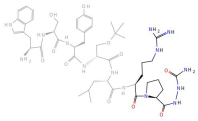	312.1770	312.1779	2.76

Metabolite: M3 -248 RT=2.41

Type	score	sub. m/z observed	sub. m/z calculated	sub ppm			met. m/z observed	met. m/z calculated	met. ppm
MATCH	21.1	329.2042	329.2044	0.51	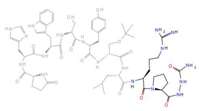	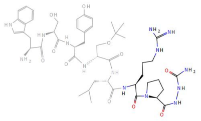	329.2034	329.2044	3.17
MATCH	3.9	399.2825	399.2827	0.29	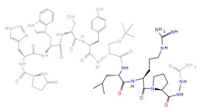	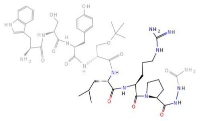	399.2834	399.2827	-1.81
MATCH	103.0	407.1827	407.1826	-0.32	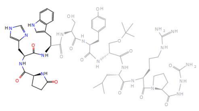	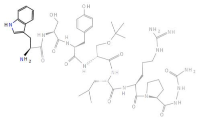	159.0919	159.0917	-1.14
MATCH	21.6	435.1780	435.1775	-1.08	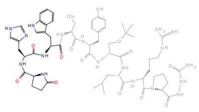	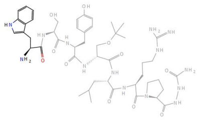	187.0870	187.0866	-2.23
MATCH	11.8	442.2890	442.2885	-1.13	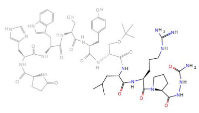	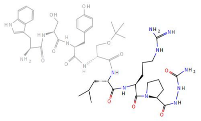	442.2889	442.2885	-1.05
MATCH	6.7	494.2138	494.2146	1.76	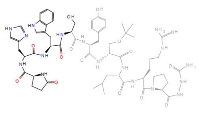	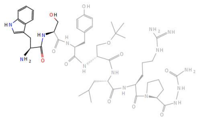	246.1237	246.1237	-0.06
MATCH	8.9	504.1999	504.1990	-1.84	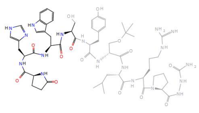	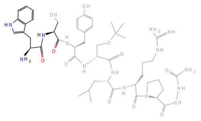	256.1084	256.1081	-1.47
MATCH	5.2	512.2909	512.2940	6.03	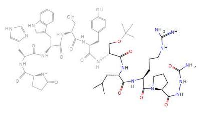	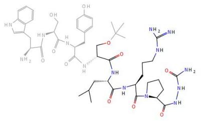	512.2950	512.2940	-2.00
MATCH	16.3	522.2089	522.2096	1.29	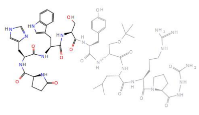	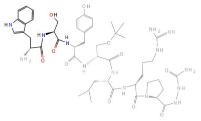	274.1187	274.1186	-0.20

Metabolite: M3 -248 RT=2.41

Type	score	sub. m/z observed	sub. m/z calculated	sub ppm			met. m/z observed	met. m/z calculated	met. ppm
						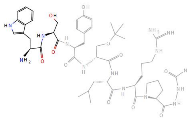	274.1187	274.1186	-0.20
MATCH	23.5	529.3213	529.3205	-1.52	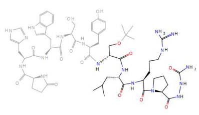	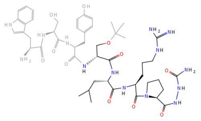	529.3213	529.3205	-1.48
MATCH	2.1	585.3806	585.3831	4.28	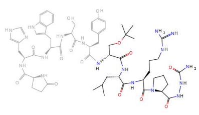	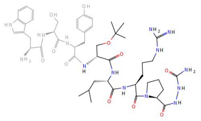	585.3820	585.3831	1.96
MATCH	3.1	589.7732	589.7781	8.32	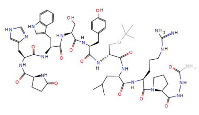	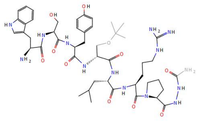	465.7333	465.7327	-1.37
MATCH	11.4	598.7893	598.7834	-9.83	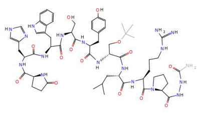	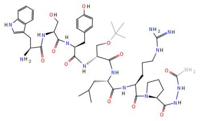	474.7388	474.7380	-1.73
						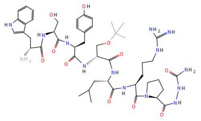	474.7388	474.7380	-1.73
MATCH	7.2	667.2636	667.2623	-1.89	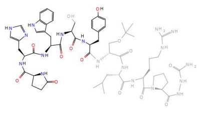	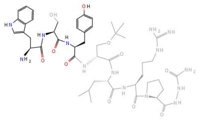	419.1708	419.1714	1.27
MATCH	4.6	675.3529	675.3573	6.54	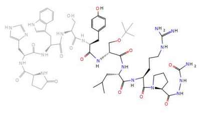	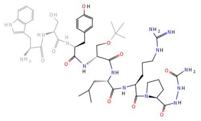	675.3569	675.3573	0.52
MATCH	22.4	692.3824	692.3838	2.14	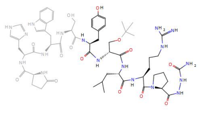	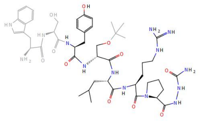	692.3845	692.3838	-0.99

Metabolite: M3 -248 RT=2.41

Type	score	sub. m/z observed	sub. m/z calculated	sub ppm		met. m/z observed	met. m/z calculated	met. ppm
MATCH	6.7	762.3868	762.3893	3.34	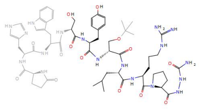 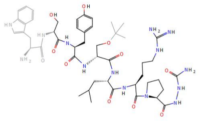	762.3885	762.3893	1.08
MATCH	28.0	779.4166	779.4159	-0.91	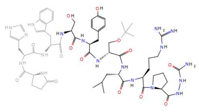 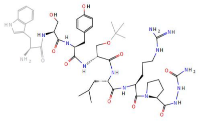	779.4167	779.4159	-1.14
MATCH	8.8	948.4637	948.4686	5.21	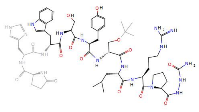 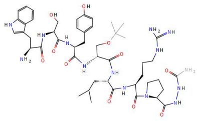	474.7388	474.7380	-1.73
					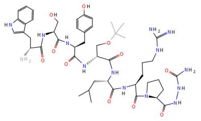	474.7388	474.7380	-1.73
MATCH	6.7	1226.6461	1226.6429	-2.64	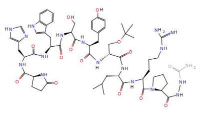 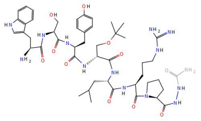	978.5494	978.5520	2.58
MATCH	5.3	1269.6524	1269.6487	-2.87	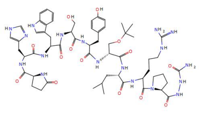 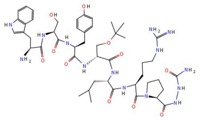	1021.5583	1021.5578	-0.50
MISMATCH	-10.9	173.1039	173.1033	-3.57	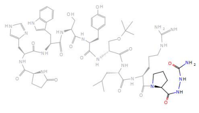	87.0563	87.0563	0.00
MISMATCH	-3.6	503.2591	503.2669	15.48	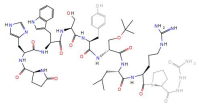	503.2610	503.2610	0.00
MISMATCH	-18.3	607.2987	607.2967	-3.36	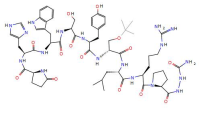	965.4964	965.4964	0.00

Metabolite: M3 -248 RT=2.41

Type	score	sub. m/z observed	sub. m/z calculated	sub ppm		met. m/z observed	met. m/z calculated	met. ppm
MISMATCH	-8.2	692.3824	692.3838	2.14	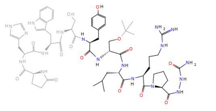	346.6956	346.6956	0.00
MET_MATCH					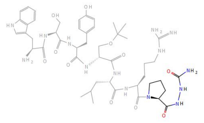	87.0563	87.0553	-11.0
MET_MATCH					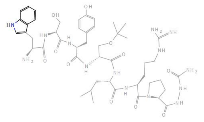	118.0658	118.0651	-5.61
MET_MATCH					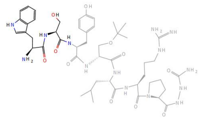	145.0759	145.0684	-51.3
MET_MATCH					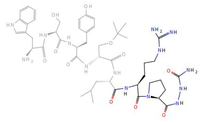	178.0867	178.0955	49.02
MET_MATCH					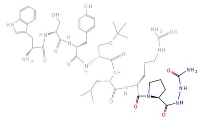	201.1025	201.0982	-21.4
MET_MATCH					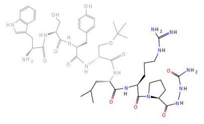	425.2610	425.2619	2.07
MET_MATCH					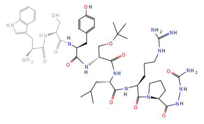	748.4431	748.4464	4.43
MET_MATCH					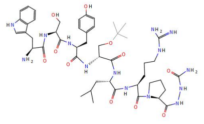	965.4964	965.4952	-1.27

MS (+) FT

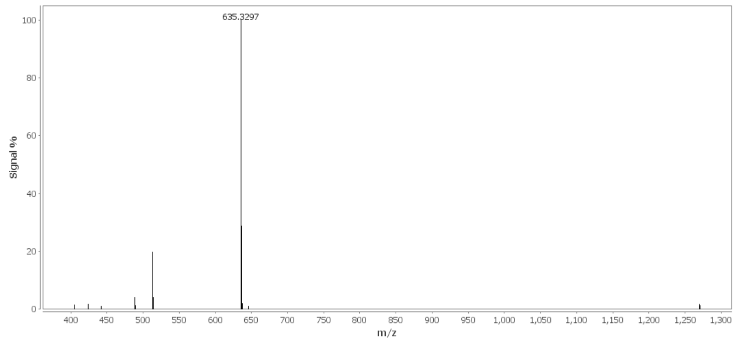

MS (+) FT

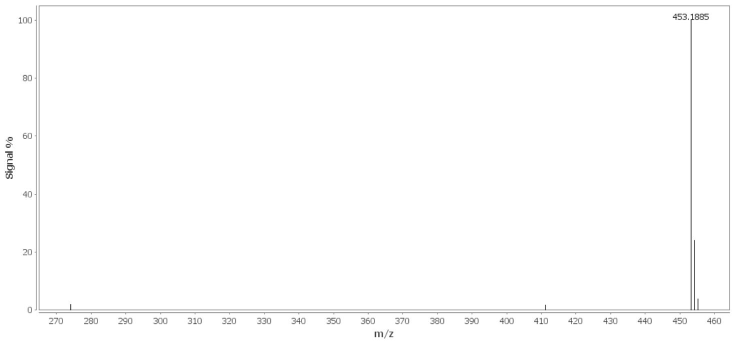

MS2 (+) FT activ = HCD:ce =

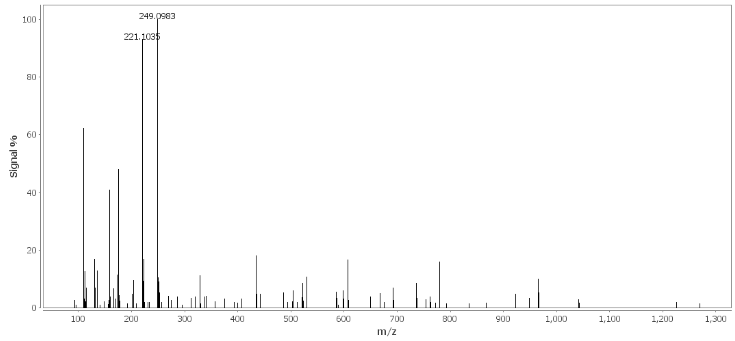

MS2 (+) FT activ = HCD:ce =

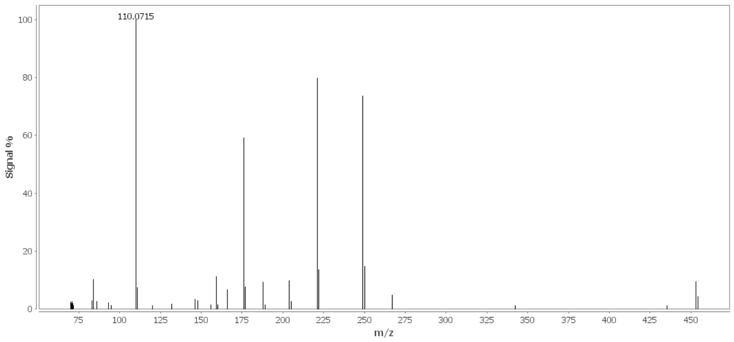

Metabolite: M1 -816 RT=0.50

Type	score	sub. m/z observed	sub. m/z calculated	sub ppm			met. m/z observed	met. m/z calculated	met. ppm
MATCH	101.7	423.8885	423.8878	-1.81	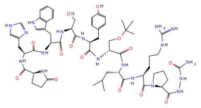	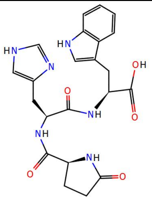	453.1885	453.1881	-0.99
						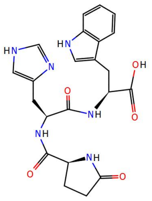	453.1885	453.1881	-0.99
						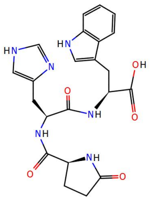	453.1885	453.1881	-0.99
MATCH	200.0	635.3297	635.3280	-2.63	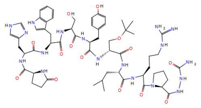	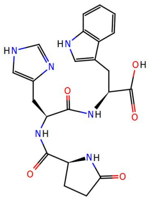	453.1885	453.1881	-0.99
						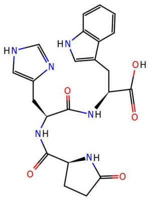	453.1885	453.1881	-0.99

Metabolite: M1 -816 RT=0.50

Type	score	sub. m/z observed	sub. m/z calculated	sub ppm			met. m/z observed	met. m/z calculated	met. ppm
						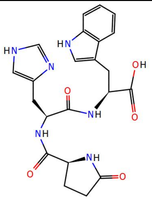	453.1885	453.1881	-0.99
MATCH	101.6	1269.6513	1269.6487	-2.00	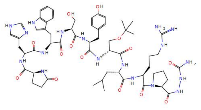	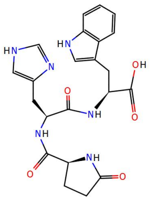	453.1885	453.1881	-0.99
						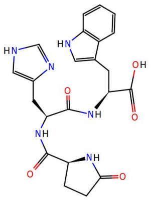	453.1885	453.1881	-0.99
						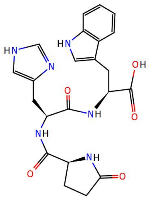	453.1885	453.1881	-0.99
MATCH	4.8	93.0457	93.0447	-10.0	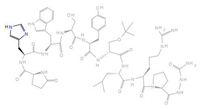	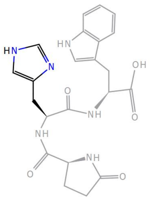	93.0451	93.0447	-4.26
MATCH	2.3	95.0612	95.0604	-9.15	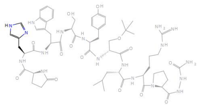	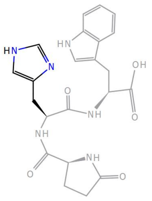	95.0608	95.0604	-4.50
MATCH	162.2	110.0718	110.0713	-4.42	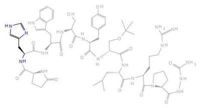	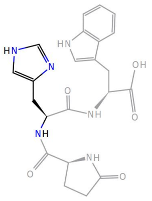	110.0715	110.0713	-2.21
MATCH	52.1	159.0918	159.0917	-1.01	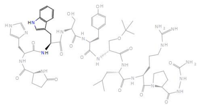	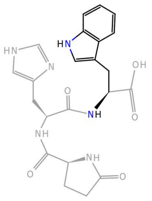	159.0914	159.0917	1.82
MATCH	13.5	166.0614	166.0611	-1.60	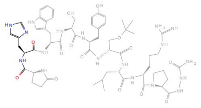	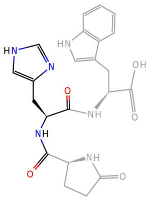	166.0608	166.0611	1.69

Metabolite: M1 -816 RT=0.50

Type	score	sub. m/z observed	sub. m/z calculated	sub ppm			met. m/z observed	met. m/z calculated	met. ppm
MATCH	172.7	221.1035	221.1033	-0.73	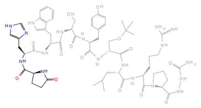	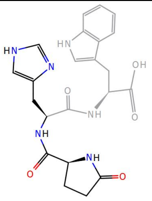	221.1029	221.1033	1.78
MATCH	173.6	249.0983	249.0982	-0.52	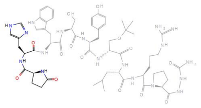	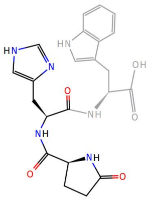	249.0978	249.0982	1.71
MATCH	11.0	1269.6524	1269.6487	-2.87	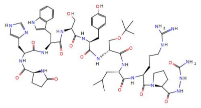	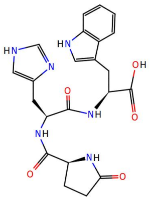	453.1858	453.1881	5.15
MISMATCH	-2.6	156.0806	156.0768	-24.6	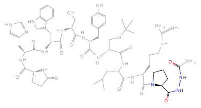		156.0767	156.0767	0.00
MET_MATCH						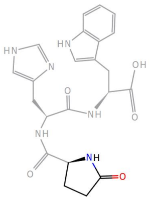	84.0450	84.0444	-6.84
MET_MATCH						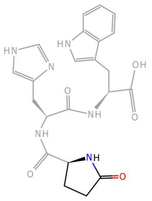	86.0605	86.0600	-5.71
MET_MATCH						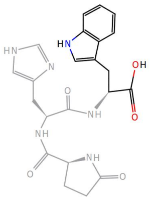	188.0701	188.0706	2.83
MET_MATCH						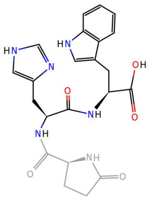	342.1552	342.1561	2.46
MET_MATCH						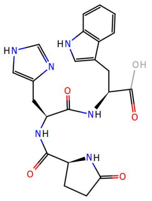	435.1759	435.1775	3.66
