# Supplementary material for: Software-aided approach to investigate peptide structure and metabolic susceptibility of amide bonds in peptide drugs based on high resolution mass spectrometry
Source: PLoS One. 2017 Nov 1;12(11):e0186461. doi: 10.1371/journal.pone.0186461 (PMC5665424; doi:10.1371/journal.pone.0186461)
Supplement: S1 File — (ZIP) [file pone.0186461.s007.zip › SFiles/S9_File.pdf]

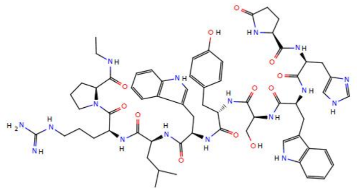

Deslorelin

Property name	Property value
Time	0min, 5min, 15min, 45min, 120min
Instrument	ThermoQAPlus
Matrix	chymotrypsin
Acquisition Mode	ddMS2

Chromatograms

Time=0min

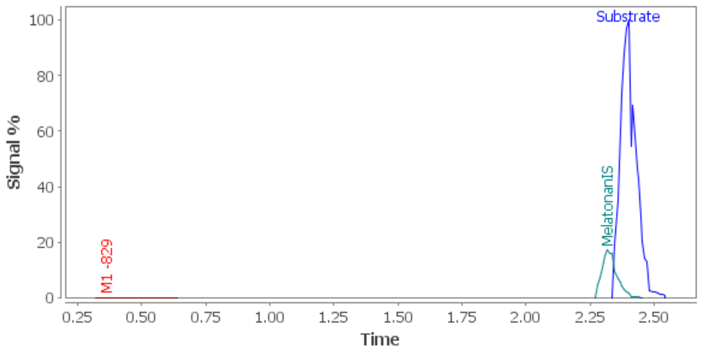

Time=5min

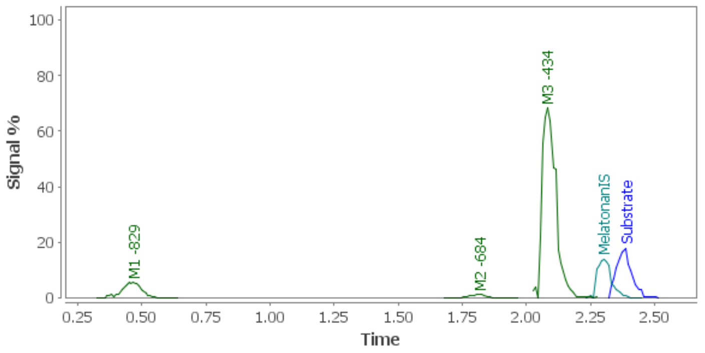

Time=15min

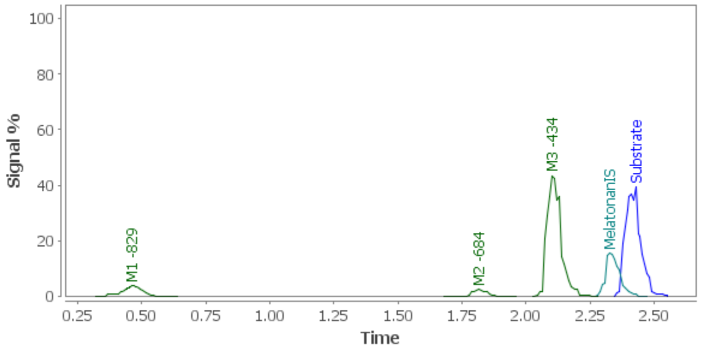

Time=45min

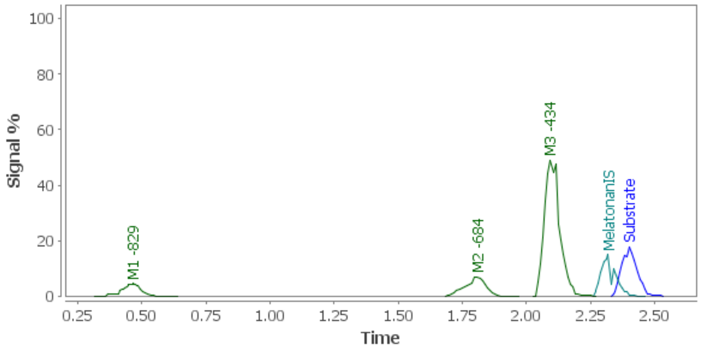

Time=120min

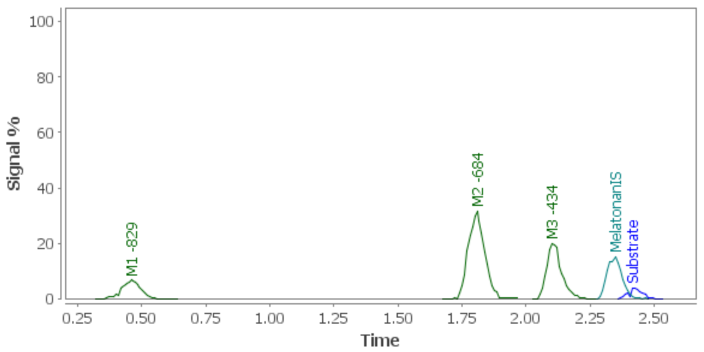

Custom Charts

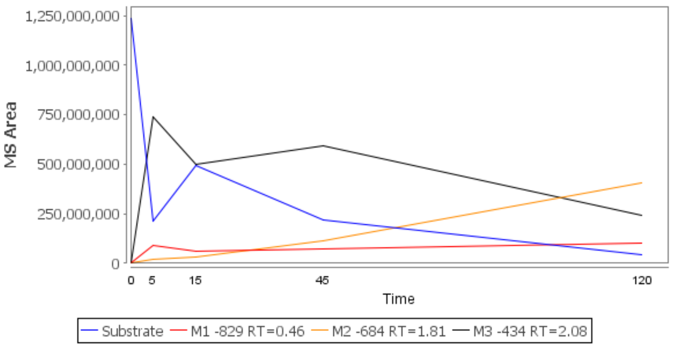

Fragmentation

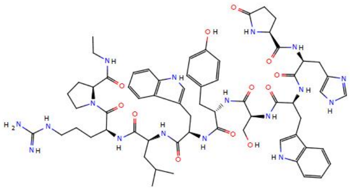

Deslorelin

MS (+) FT

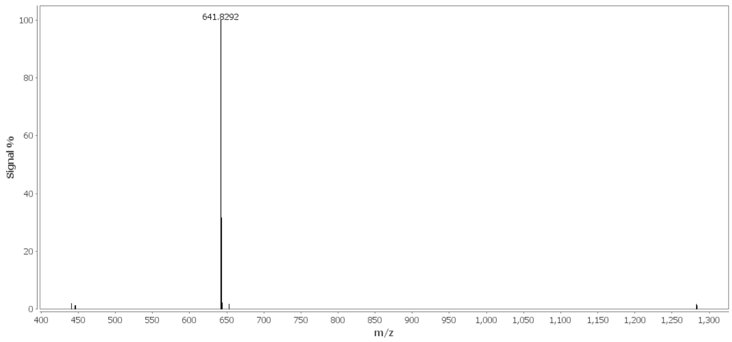

MS (+) FT

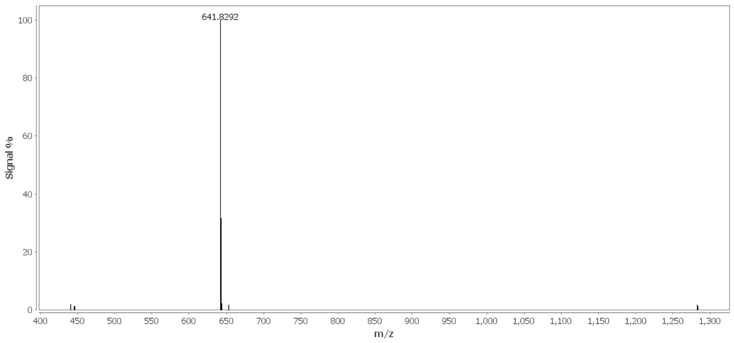

MS2 (+) FT activ = HCD:ce =

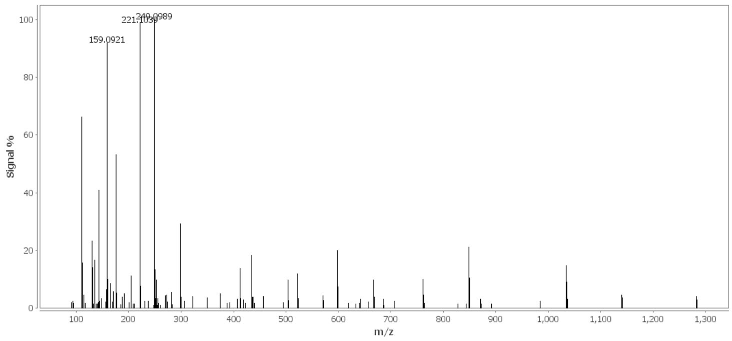

MS2 (+) FT activ = HCD:ce =

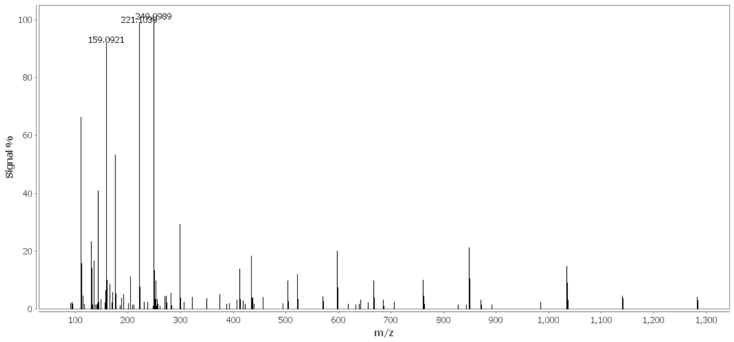

Metabolite: Substrate

Type	score	sub. m/z observed	sub. m/z calculated	sub ppm			met. m/z observed	met. m/z calculated	met. ppm
MATCH	16.5	1282.6515	1282.6480	-2.70	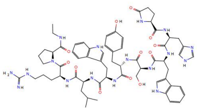	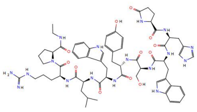	1282.6515	1282.6480	-2.70
MATCH	101.7	1282.6508	1282.6480	-2.23	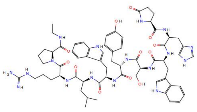	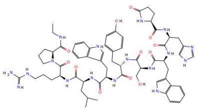	1282.6508	1282.6480	-2.23
MATCH	7.4	1140.5401	1140.5374	-2.39	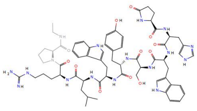	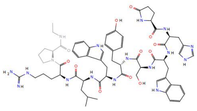	1140.5401	1140.5374	-2.39

Metabolite: Substrate

Type	score	sub. m/z observed	sub. m/z calculated	sub ppm		met. m/z observed	met. m/z calculated	met. ppm
MATCH	17.0	1034.5597	1034.5570	-2.56	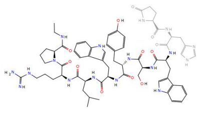 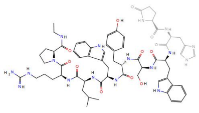	1034.5597	1034.5570	-2.56
MATCH	4.1	871.3582	871.3522	-6.91	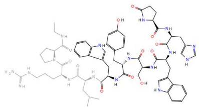 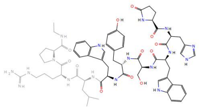	871.3582	871.3522	-6.91
MATCH	35.6	843.3510	843.3573	7.43	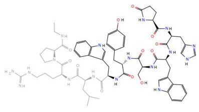 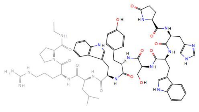	843.3510	843.3573	7.43
MATCH	9.0	685.2756	685.2729	-3.90	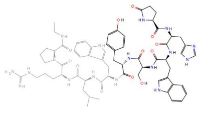 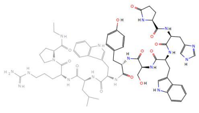	685.2756	685.2729	-3.90
MATCH	102.1	657.2769	657.2780	1.65	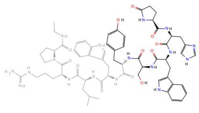 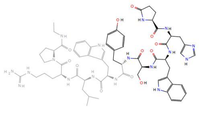	657.2769	657.2780	1.65
MATCH	15.4	641.8312	641.8276	-5.50	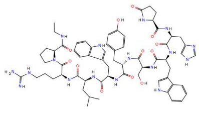 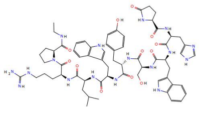	641.8312	641.8276	-5.50
MATCH	200.0	641.8292	641.8276	-2.45	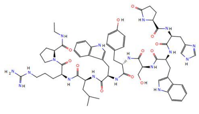 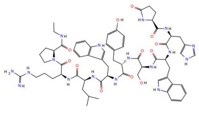	641.8292	641.8276	-2.45
MATCH	51.9	598.3834	598.3824	-1.78	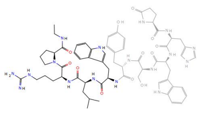 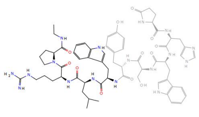	598.3834	598.3824	-1.78
MATCH	12.4	504.1998	504.1990	-1.70	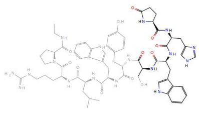 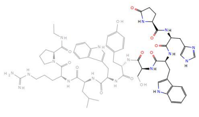	504.1998	504.1990	-1.70

Metabolite: Substrate

Type	score	sub. m/z observed	sub. m/z calculated	sub ppm			met. m/z observed	met. m/z calculated	met. ppm
MATCH	14.0	494.2148	494.2146	-0.36	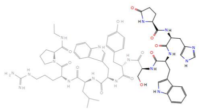	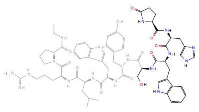	494.2148	494.2146	-0.36
MATCH	12.5	456.2734	456.2718	-3.49	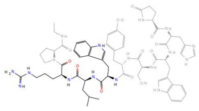	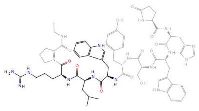	456.2734	456.2718	-3.49
MATCH	4.9	439.2458	439.2452	-1.31	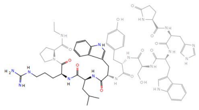	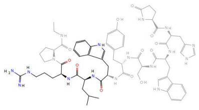	439.2458	439.2452	-1.31
MATCH	38.4	412.3040	412.3031	-2.28	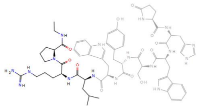	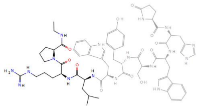	412.3040	412.3031	-2.28
MATCH	3.9	300.1711	300.1707	-1.56	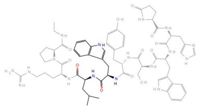	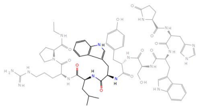	300.1711	300.1707	-1.56
MATCH	3.9	300.1711	300.1707	-1.56	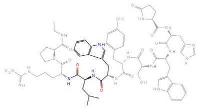	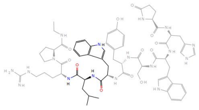	300.1711	300.1707	-1.56
MATCH	42.4	299.2199	299.2190	-2.87	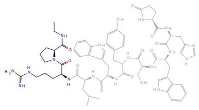	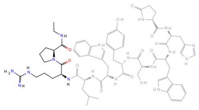	299.2199	299.2190	-2.87
MATCH	10.3	282.1924	282.1925	0.05	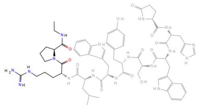	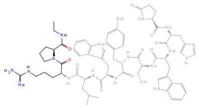	282.1924	282.1925	0.05
MATCH	11.8	272.1767	272.1757	-3.68	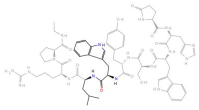	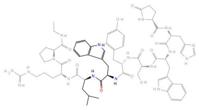	272.1767	272.1757	-3.68

Metabolite: Substrate

Type	score	sub. m/z observed	sub. m/z calculated	sub ppm		met. m/z observed	met. m/z calculated	met. ppm
MATCH	11.8	270.1934	270.1925	-3.67	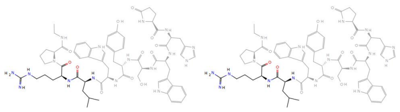	270.1934	270.1925	-3.67
MATCH	21.0	261.1139	261.1164	9.48	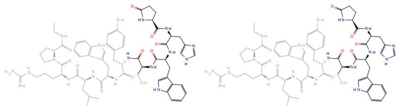	261.1139	261.1164	9.48
MATCH	8.8	255.1491	255.1492	0.46	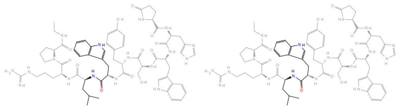	255.1491	255.1492	0.46
MATCH	33.9	253.1665	253.1659	-2.34	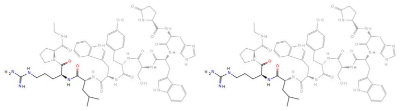	253.1665	253.1659	-2.34
MATCH	175.7	249.0988	249.0982	-2.54	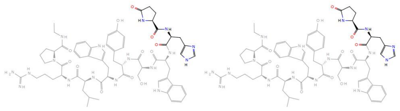	249.0988	249.0982	-2.54
MATCH	4.4	237.1351	237.1346	-2.06	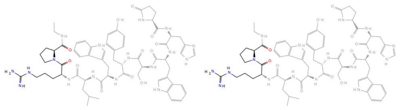	237.1351	237.1346	-2.06
MATCH	176.9	221.1039	221.1033	-2.78	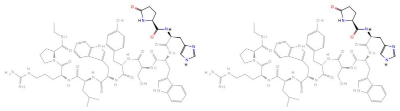	221.1039	221.1033	-2.78
MATCH	3.4	209.1402	209.1397	-2.29	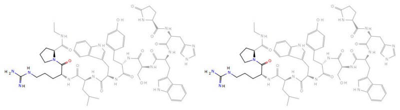	209.1402	209.1397	-2.29
MATCH	8.7	187.0870	187.0866	-1.95	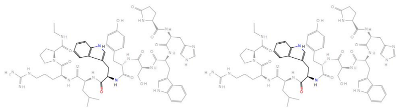	187.0870	187.0866	-1.95

Metabolite: Substrate

Type	score	sub. m/z observed	sub. m/z calculated	sub ppm			met. m/z observed	met. m/z calculated	met. ppm
MATCH	8.7	187.0870	187.0866	-1.95	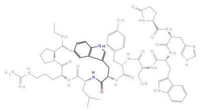	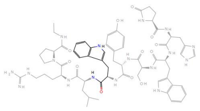	187.0870	187.0866	-1.95
MATCH	3.9	185.1040	185.1033	-3.52	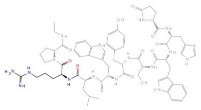	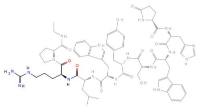	185.1040	185.1033	-3.52
MATCH	105.6	170.0604	170.0600	-2.09	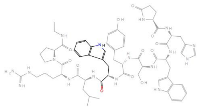	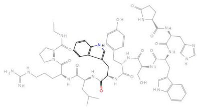	170.0604	170.0600	-2.09
MATCH	15.6	166.0617	166.0611	-3.81	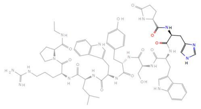	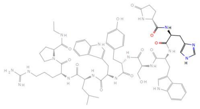	166.0617	166.0611	-3.81
MISMATCH	-3.2	160.0764	160.0737	-16.6	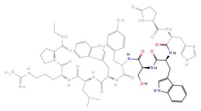	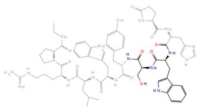	160.0764	160.0737	-16.6
MATCH	103.3	159.0921	159.0917	-2.93	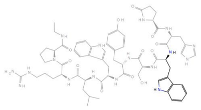	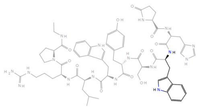	159.0921	159.0917	-2.93
MATCH	184.6	159.0921	159.0917	-2.93	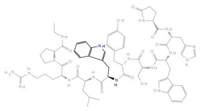	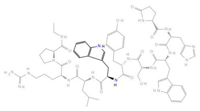	159.0921	159.0917	-2.93
MATCH	12.9	157.1087	157.1084	-2.23	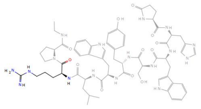	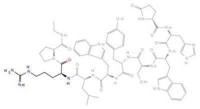	157.1087	157.1084	-2.23
MATCH	12.8	144.0811	144.0808	-2.35	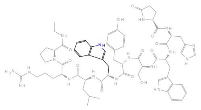	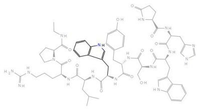	144.0811	144.0808	-2.35

Metabolite: Substrate

Type	score	sub. m/z observed	sub. m/z calculated	sub ppm		met. m/z observed	met. m/z calculated	met. ppm
MATCH	76.5	143.1184	143.1179	-3.38	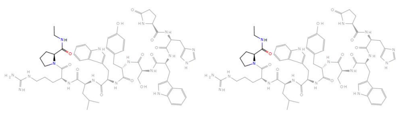	143.1184	143.1179	-3.38
MISMATCH	2.5	138.0666	138.0662	-2.71	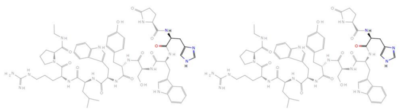	138.0666	138.0662	-2.71
MISMATCH	2.5	138.0666	138.0662	-2.71	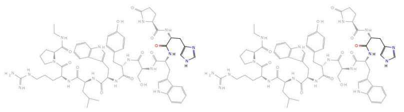	138.0666	138.0662	-2.71
MATCH	84.4	136.0762	136.0757	-3.45	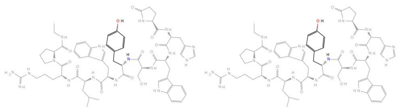	136.0762	136.0757	-3.45
MISMATCH	-33.0	130.0656	130.0575	-62.4	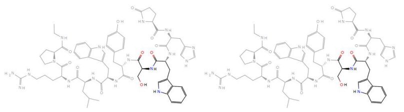	130.0656	130.0575	-62.4
MISMATCH	-3.3	117.0580	117.0679	84.28	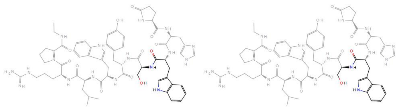	117.0580	117.0679	84.28
MATCH	10.4	115.0874	115.0866	-7.26	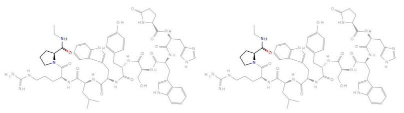	115.0874	115.0866	-7.26
MATCH	35.2	112.0876	112.0869	-6.00	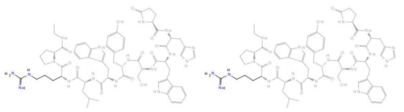	112.0876	112.0869	-6.00
MATCH	166.3	110.0720	110.0713	-6.37	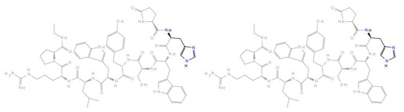	110.0720	110.0713	-6.37

Metabolite: Substrate

Type	score	sub. m/z observed	sub. m/z calculated	sub ppm			met. m/z observed	met. m/z calculated	met. ppm
MISMATCH	4.4	95.0612	95.0604	-8.62	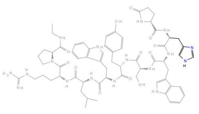	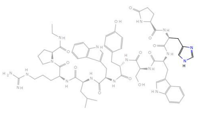	95.0612	95.0604	-8.62
MATCH	5.7	91.0550	91.0522	-30.9	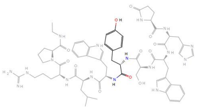	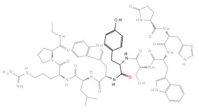	91.0550	91.0522	-30.9

MS (+) FT

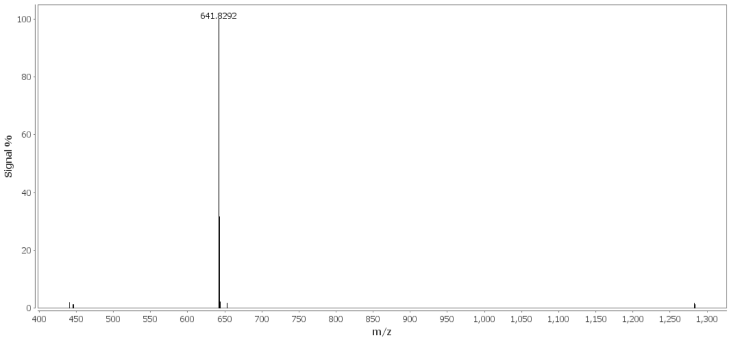

MS (+) FT

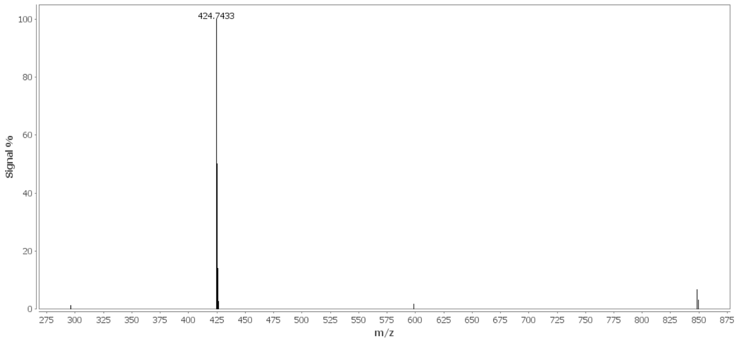

MS2 (+) FT activ = HCD:ce =

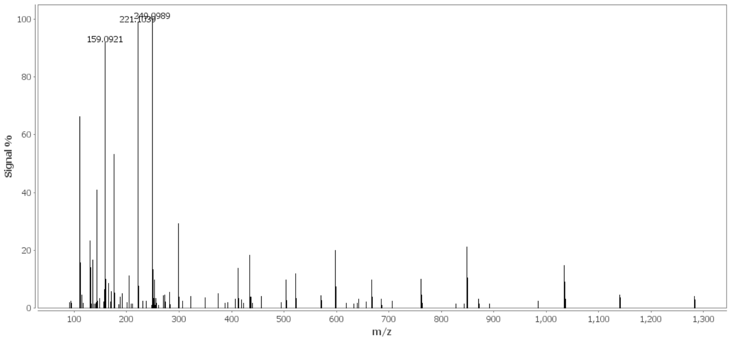

MS2 (+) FT activ = HCD:ce =

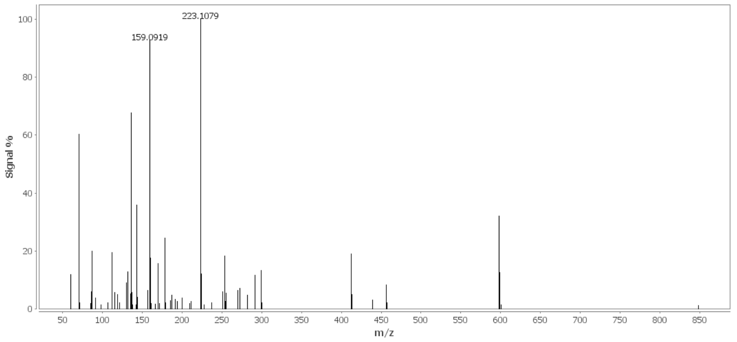

Metabolite: M3 -434 RT=2.08

Type	score	sub. m/z observed	sub. m/z calculated	sub ppm			met. m/z observed	met. m/z calculated	met. ppm
MATCH	200.0	641.8292	641.8276	-2.45	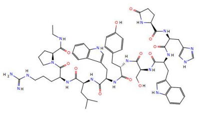	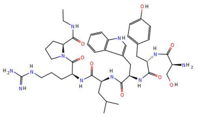	424.7433	424.7425	-1.77
MATCH	200.0	641.8292	641.8276	-2.45	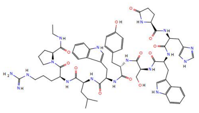	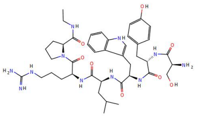	424.7433	424.7425	-1.77

Metabolite: M3 -434 RT=2.08

Type	score	sub. m/z observed	sub. m/z calculated	sub ppm		met. m/z observed	met. m/z calculated	met. ppm
MATCH	106.6	641.8292	641.8276	-2.45	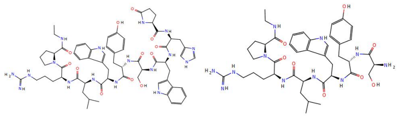	848.4793	848.4777	-1.88
MATCH	106.6	641.8292	641.8276	-2.45	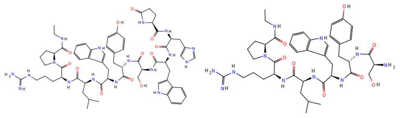	848.4793	848.4777	-1.88
MATCH	101.7	1282.6508	1282.6480	-2.23	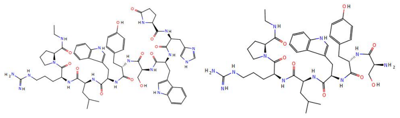	424.7433	424.7425	-1.77
MATCH	101.7	1282.6508	1282.6480	-2.23	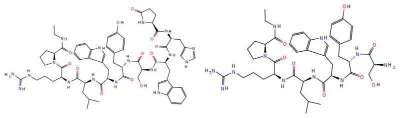	424.7433	424.7425	-1.77
MATCH	8.3	1282.6508	1282.6480	-2.23	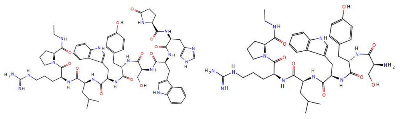	848.4793	848.4777	-1.88
MATCH	8.3	1282.6508	1282.6480	-2.23	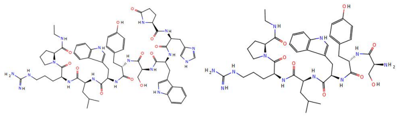	848.4793	848.4777	-1.88
MATCH	5.7	91.0550	91.0522	-30.9	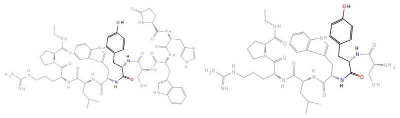	91.0549	91.0522	-29.2
MATCH	35.2	112.0876	112.0869	-6.00	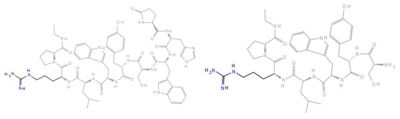	112.0874	112.0869	-4.39
MATCH	10.4	115.0874	115.0866	-7.26	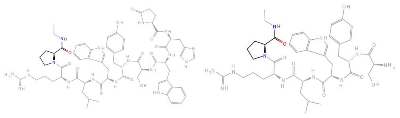	115.0870	115.0866	-3.55

Metabolite: M3 -434 RT=2.08

Type	score	sub. m/z observed	sub. m/z calculated	sub ppm		met. m/z observed	met. m/z calculated	met. ppm
MATCH	84.4	136.0762	136.0757	-3.45	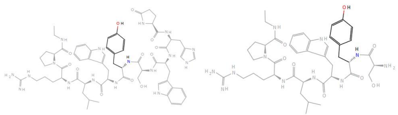	136.0760	136.0757	-2.06
MATCH	3.3	142.0655	142.0651	-2.72	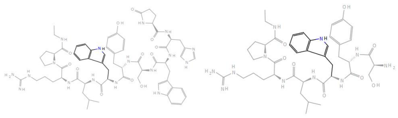	142.0652	142.0651	-0.87
MATCH	76.5	143.1184	143.1179	-3.38	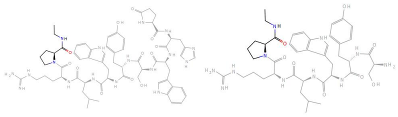	143.1181	143.1179	-1.75
MATCH	6.1	144.0811	144.0808	-2.35	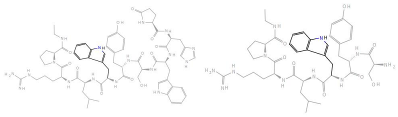	144.0810	144.0808	-1.77
MATCH	12.9	157.1087	157.1084	-2.23	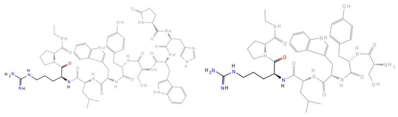	157.1084	157.1084	-0.26
MATCH	184.6	159.0921	159.0917	-2.93	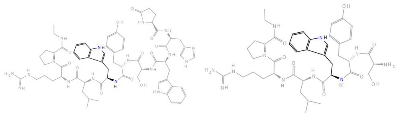	159.0919	159.0917	-1.29
MATCH	21.4	170.0604	170.0600	-2.09	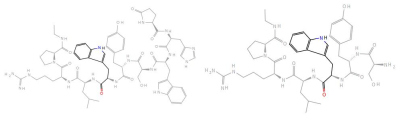	170.0602	170.0600	-1.21
MATCH	3.9	185.1040	185.1033	-3.52	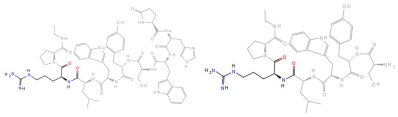	185.1037	185.1033	-2.19
MATCH	8.7	187.0870	187.0866	-1.95	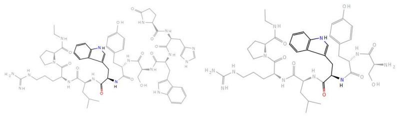	187.0870	187.0866	-2.08

Metabolite: M3 -434 RT=2.08

Type	score	sub. m/z observed	sub. m/z calculated	sub ppm		met. m/z observed	met. m/z calculated	met. ppm
MATCH	8.7	187.0870	187.0866	-1.95	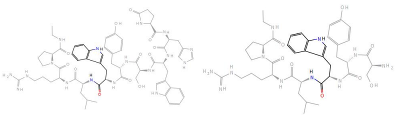	187.0870	187.0866	-2.08
MATCH	3.4	209.1402	209.1397	-2.29	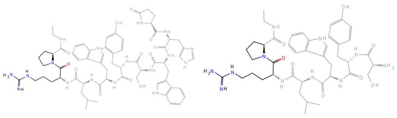	209.1398	209.1397	-0.68
MATCH	4.4	237.1351	237.1346	-2.06	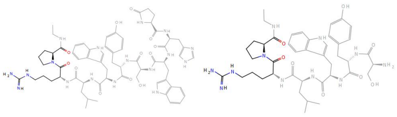	237.1347	237.1346	-0.60
MATCH	28.0	253.1665	253.1659	-2.34	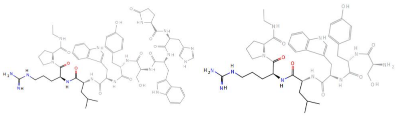	253.1661	253.1659	-0.62
MATCH	8.8	255.1491	255.1492	0.46	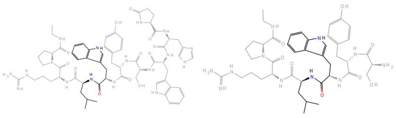	255.1497	255.1492	-2.01
MATCH	21.0	261.1139	261.1164	9.48	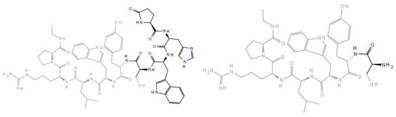	87.0561	87.0553	-8.78
MATCH	10.6	270.1934	270.1925	-3.67	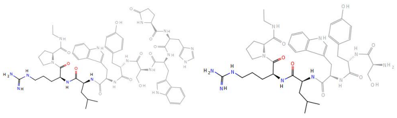	270.1928	270.1925	-1.29
MATCH	11.8	272.1767	272.1757	-3.68	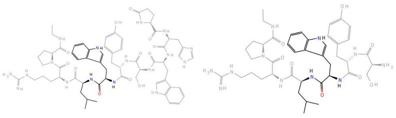	272.1760	272.1757	-0.78
MATCH	10.3	282.1924	282.1925	0.05	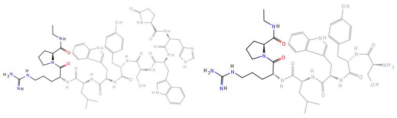	282.1920	282.1925	1.60

Metabolite: M3 -434 RT=2.08

Type	score	sub. m/z observed	sub. m/z calculated	sub ppm			met. m/z observed	met. m/z calculated	met. ppm
MATCH	42.4	299.2199	299.2190	-2.87	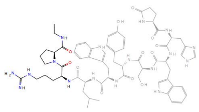	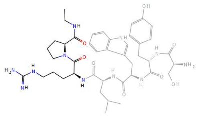	299.2195	299.2190	-1.52
MATCH	3.9	300.1711	300.1707	-1.56	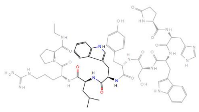	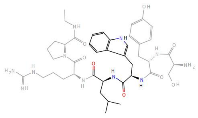	300.1708	300.1707	-0.46
MATCH	3.9	300.1711	300.1707	-1.56	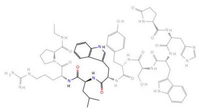	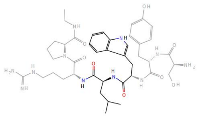	300.1708	300.1707	-0.46
MATCH	32.9	412.3040	412.3031	-2.28	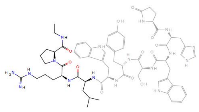	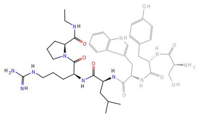	412.3030	412.3031	0.08
MATCH	4.9	439.2458	439.2452	-1.31	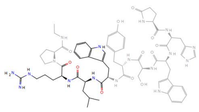	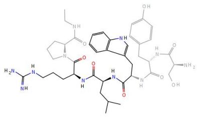	439.2458	439.2452	-1.41
MATCH	12.5	456.2734	456.2718	-3.49	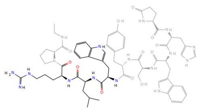	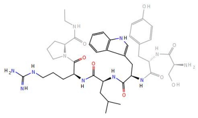	456.2724	456.2718	-1.42
MATCH	14.0	494.2148	494.2146	-0.36	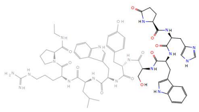	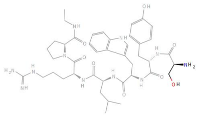	60.0454	60.0444	-16.2
MATCH	12.4	504.1998	504.1990	-1.70	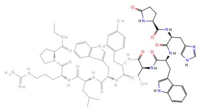	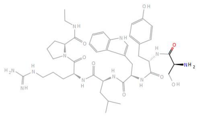	70.0297	70.0287	-13.0
MATCH	51.9	598.3834	598.3824	-1.78	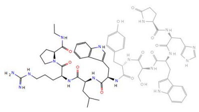	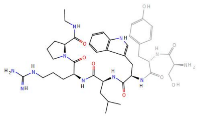	598.3829	598.3824	-0.91

Metabolite: M3 -434 RT=2.08

Type	score	sub. m/z observed	sub. m/z calculated	sub ppm			met. m/z observed	met. m/z calculated	met. ppm
MATCH	4.2	641.8312	641.8276	-5.50	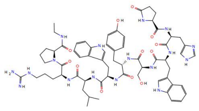	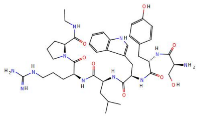	848.4788	848.4777	-1.27
						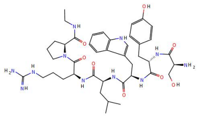	848.4788	848.4777	-1.27
MATCH	102.1	657.2769	657.2780	1.65	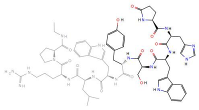	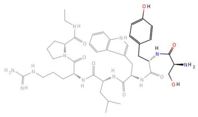	223.1079	223.1077	-0.82
MATCH	9.0	685.2756	685.2729	-3.90	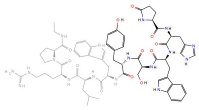	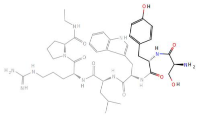	251.1029	251.1026	-0.96
MATCH	5.3	1282.6515	1282.6480	-2.70	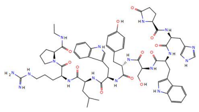	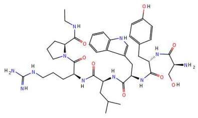	848.4788	848.4777	-1.27
						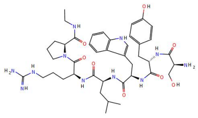	848.4788	848.4777	-1.27
MET_MATCH						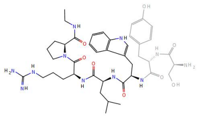	598.3839	598.3824	-2.48
MET_MATCH						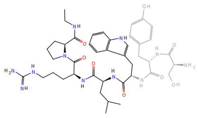	291.1819	291.1816	-1.36

MS (+) FT

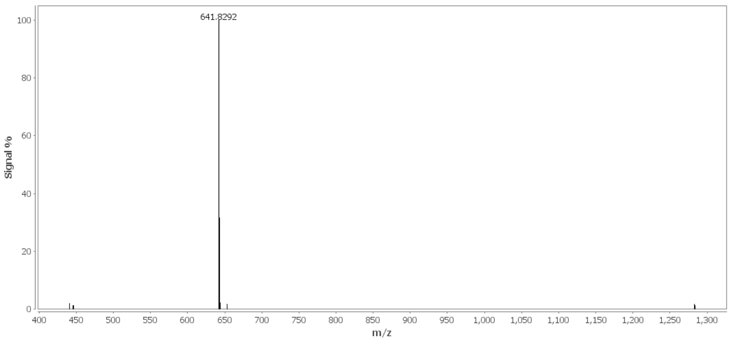

MS (+) FT

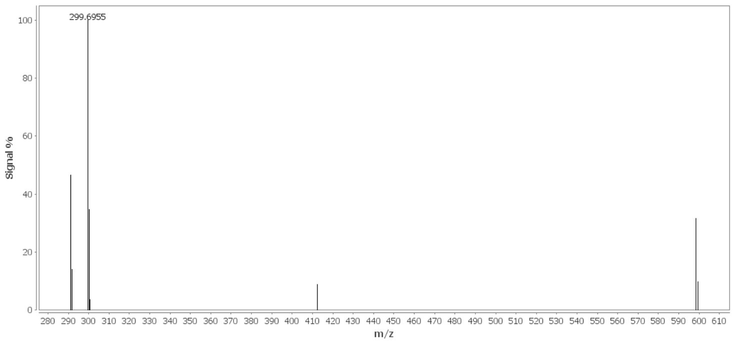

MS2 (+) FT activ = HCD:ce =

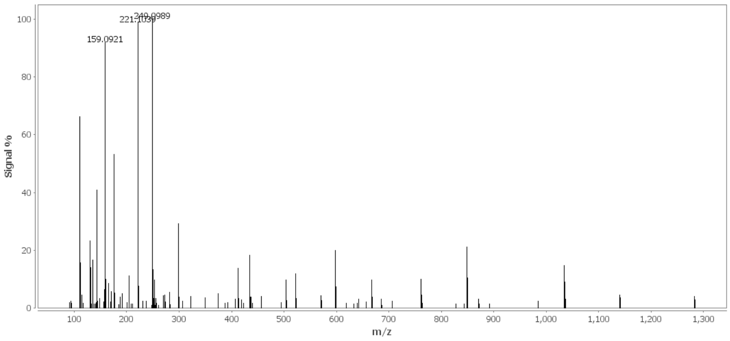

MS2 (+) FT activ = HCD:ce =

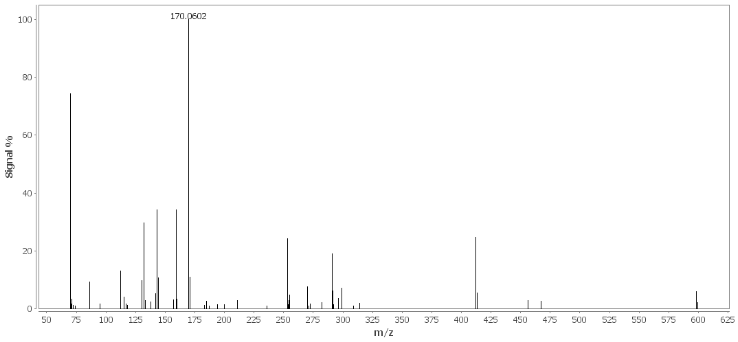

Metabolite: M2 -684 RT=1.81

Type	score	sub. m/z observed	sub. m/z calculated	sub ppm		met. m/z observed	met. m/z calculated	met. ppm
MATCH	200.0	641.8292	641.8276	-2.45	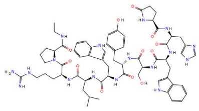 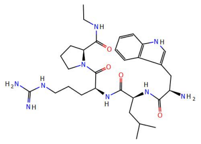	299.6955	299.6948	-2.17
MATCH	200.0	641.8292	641.8276	-2.45	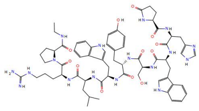 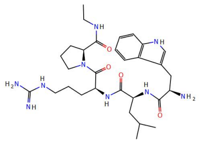	299.6955	299.6948	-2.17
MATCH	131.6	641.8292	641.8276	-2.45	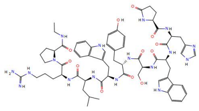 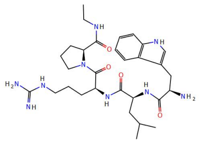	598.3834	598.3824	-1.73
MATCH	131.6	641.8292	641.8276	-2.45	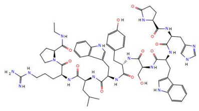 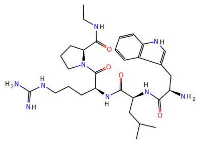	598.3834	598.3824	-1.73
MATCH	101.7	1282.6508	1282.6480	-2.23	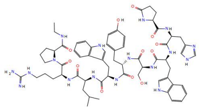 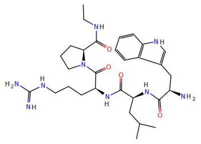	299.6955	299.6948	-2.17

Metabolite: M2 -684 RT=1.81

Type	score	sub. m/z observed	sub. m/z calculated	sub ppm		met. m/z observed	met. m/z calculated	met. ppm
MATCH	101.7	1282.6508	1282.6480	-2.23	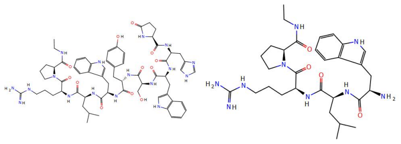	299.6955	299.6948	-2.17
MATCH	33.3	1282.6508	1282.6480	-2.23	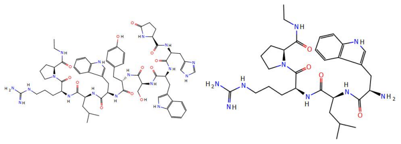	598.3834	598.3824	-1.73
MATCH	33.3	1282.6508	1282.6480	-2.23	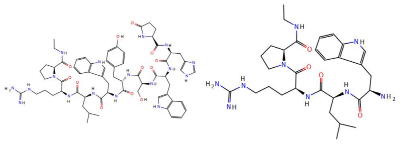	598.3834	598.3824	-1.73
MATCH	28.8	112.0876	112.0869	-6.00	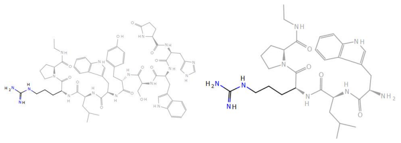	112.0874	112.0869	-4.25
MATCH	8.6	115.0874	115.0866	-7.26	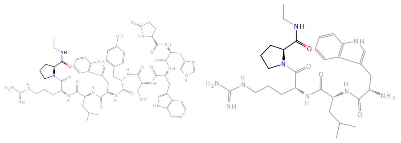	115.0871	115.0866	-4.05
MATCH	7.2	142.0655	142.0651	-2.72	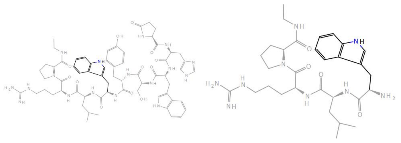	142.0653	142.0651	-1.52
MATCH	75.0	143.1184	143.1179	-3.38	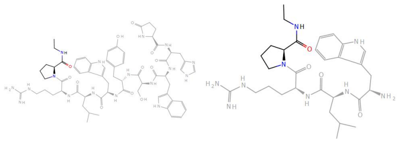	143.1181	143.1179	-1.73
MATCH	12.8	144.0811	144.0808	-2.35	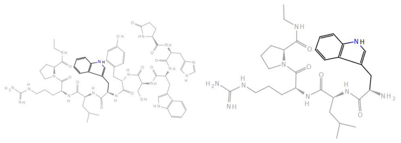	144.0810	144.0808	-1.63
MATCH	9.4	157.1087	157.1084	-2.23	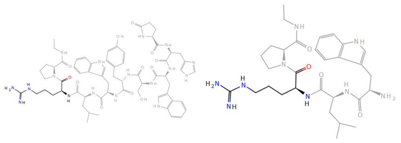	157.1082	157.1084	1.26

Metabolite: M2 -684 RT=1.81

Type	score	sub. m/z observed	sub. m/z calculated	sub ppm		met. m/z observed	met. m/z calculated	met. ppm
MATCH	105.6	170.0604	170.0600	-2.09	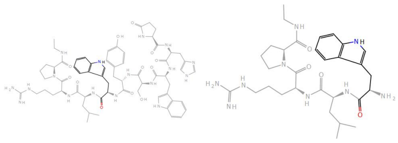	170.0602	170.0600	-0.92
MATCH	3.8	185.1040	185.1033	-3.52	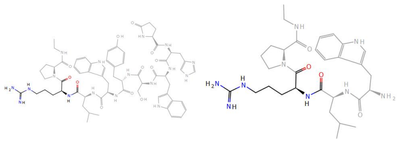	185.1036	185.1033	-1.65
MATCH	4.9	187.0870	187.0866	-1.95	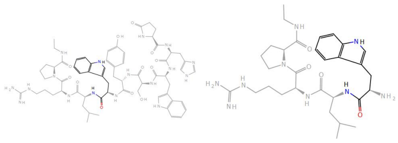	187.0867	187.0866	-0.59
					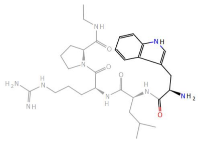	187.0867	187.0866	-0.59
MATCH	33.9	253.1665	253.1659	-2.34	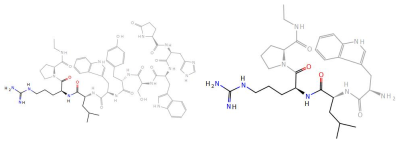	253.1660	253.1659	-0.45
MATCH	8.1	255.1491	255.1492	0.46	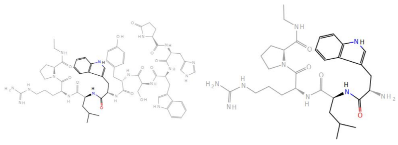	255.1493	255.1492	-0.58
MATCH	11.8	270.1934	270.1925	-3.67	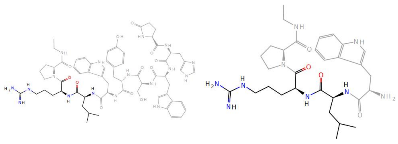	270.1933	270.1925	-3.22
MATCH	7.7	282.1924	282.1925	0.05	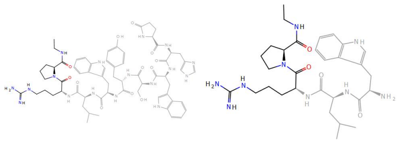	282.1932	282.1925	-2.65
MATCH	36.2	299.2199	299.2190	-2.87	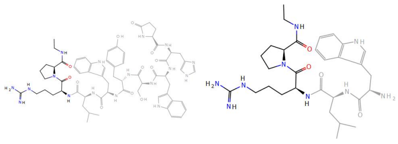	299.2190	299.2190	0.10

Metabolite: M2 -684 RT=1.81

Type	score	sub. m/z observed	sub. m/z calculated	sub ppm			met. m/z observed	met. m/z calculated	met. ppm
MATCH	38.4	412.3040	412.3031	-2.28	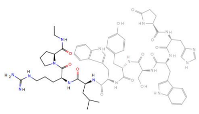	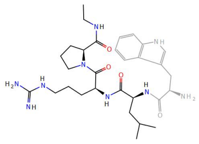	412.3034	412.3031	-0.69
MATCH	9.1	641.8312	641.8276	-5.50	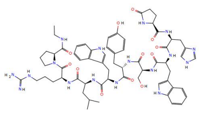	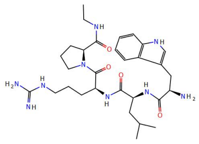	598.3818	598.3824	0.89
						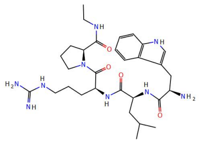	598.3818	598.3824	0.89
MATCH	35.6	843.3510	843.3573	7.43	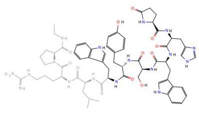	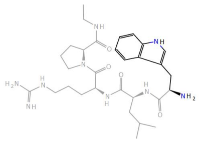	159.0919	159.0917	-1.22
MATCH	4.1	871.3582	871.3522	-6.91	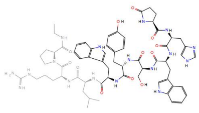	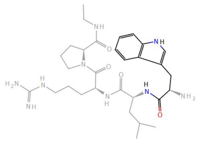	187.0867	187.0866	-0.59
						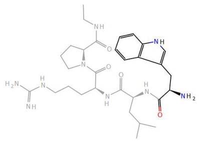	187.0867	187.0866	-0.59
MATCH	7.4	1140.5401	1140.5374	-2.39	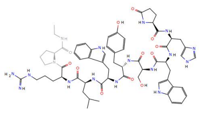	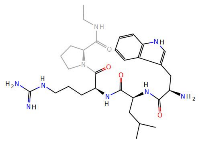	456.2719	456.2718	-0.37
MATCH	10.2	1282.6515	1282.6480	-2.70	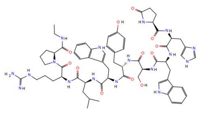	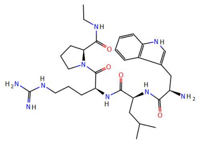	598.3818	598.3824	0.89
						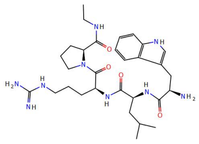	598.3818	598.3824	0.89

Metabolite: M2 -684 RT=1.81

Type	score	sub. m/z observed	sub. m/z calculated	sub ppm		met. m/z observed	met. m/z calculated	met. ppm
MISMATCH	-3.5	95.0612	95.0604	-8.62	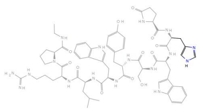	95.0609	95.0609	0.00
MISMATCH	-3.3	117.0580	117.0679	84.28	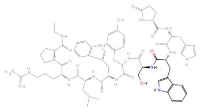	117.0576	117.0576	0.00
MISMATCH	-33.0	130.0656	130.0575	-62.4	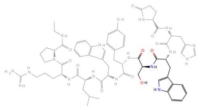	130.0654	130.0654	0.00
MISMATCH	-3.8	138.0666	138.0662	-2.71	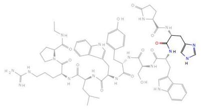	138.0663	138.0663	0.00
MISMATCH	-3.2	160.0764	160.0737	-16.6	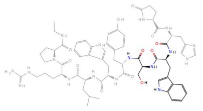	160.0762	160.0762	0.00
MET_MATCH					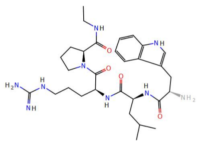	291.1821	291.1816	-1.72
MET_MATCH					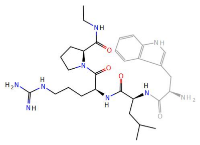	412.3036	412.3031	-1.20
MET_MATCH					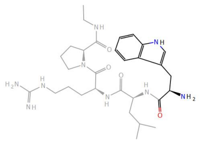	95.0609	95.0548	-64.4
MET_MATCH					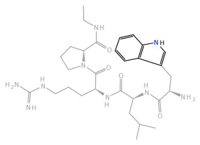	118.0654	118.0651	-2.44

Metabolite: M2 -684 RT=1.81

Type	score	sub. m/z observed	sub. m/z calculated	sub ppm		met. m/z observed	met. m/z calculated	met. ppm
MET_MATCH					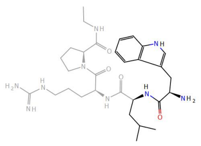	272.1757	272.1757	0.18
MET_MATCH					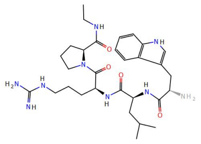	291.1818	291.1816	-0.79

MS (+) FT

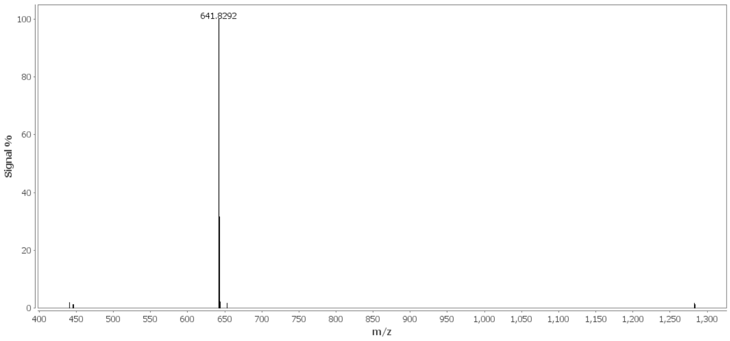

MS (+) FT

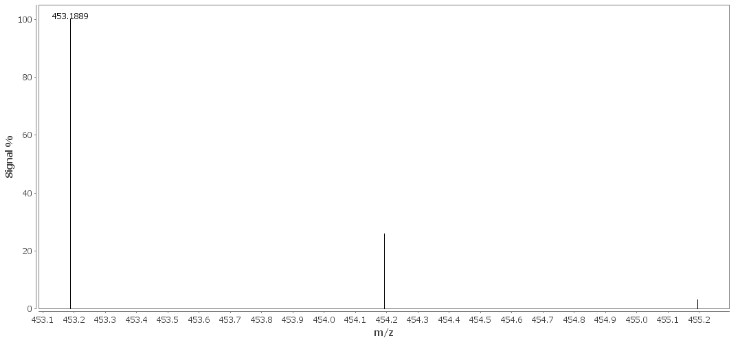

MS2 (+) FT activ = HCD:ce =

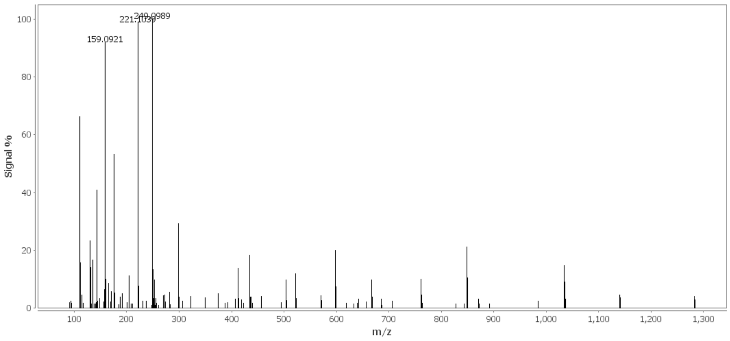

MS2 (+) FT activ = HCD:ce =

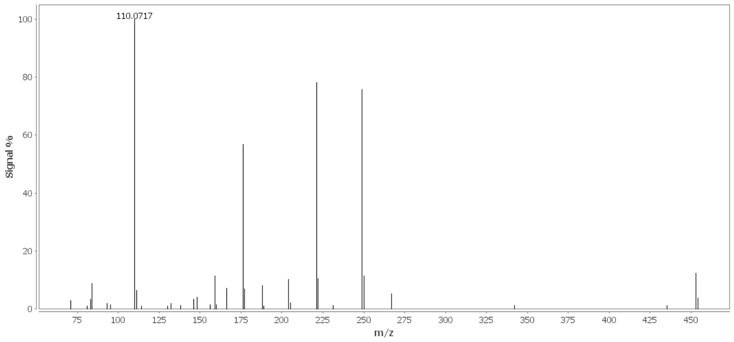

Metabolite: M1 -829 RT=0.46

Type	score	sub. m/z observed	sub. m/z calculated	sub ppm			met. m/z observed	met. m/z calculated	met. ppm
MATCH	200.0	641.8292	641.8276	-2.45	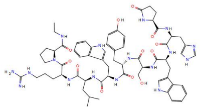	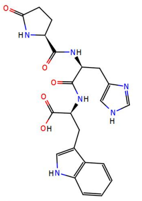	453.1889	453.1881	-1.73
						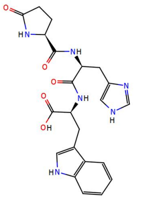	453.1889	453.1881	-1.73

Metabolite: M1 -829 RT=0.46

Type	score	sub. m/z observed	sub. m/z calculated	sub ppm			met. m/z observed	met. m/z calculated	met. ppm
MATCH	101.7	1282.6508	1282.6480	-2.23	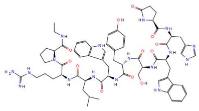	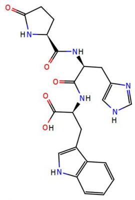	453.1889	453.1881	-1.73
						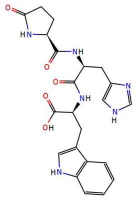	453.1889	453.1881	-1.73
MATCH	4.4	93.0456	93.0447	-9.50	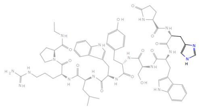	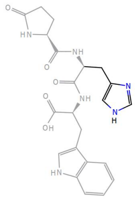	93.0454	93.0447	-6.92
MATCH	3.1	95.0612	95.0604	-8.62	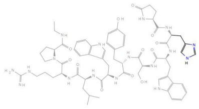	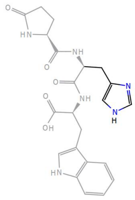	95.0609	95.0604	-5.25
MATCH	166.3	110.0720	110.0713	-6.37	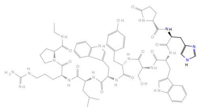	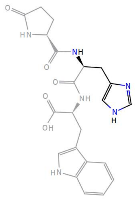	110.0717	110.0713	-3.78
MATCH	2.5	138.0666	138.0662	-2.71	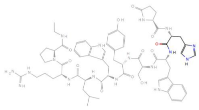	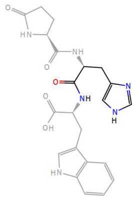	138.0664	138.0662	-1.21
MATCH	2.5	138.0666	138.0662	-2.71	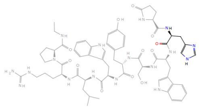	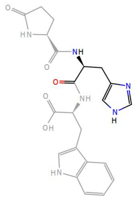	138.0664	138.0662	-1.21
MATCH	103.3	159.0921	159.0917	-2.93	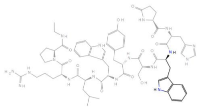	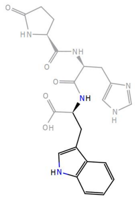	159.0917	159.0917	-0.14
MATCH	15.6	166.0617	166.0611	-3.81	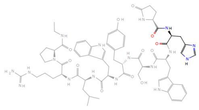	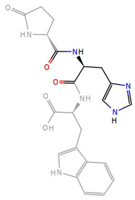	166.0612	166.0611	-0.40

Metabolite: M1 -829 RT=0.46

Type	score	sub. m/z observed	sub. m/z calculated	sub ppm			met. m/z observed	met. m/z calculated	met. ppm
MATCH	176.9	221.1039	221.1033	-2.78	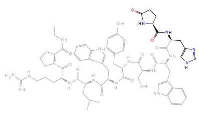	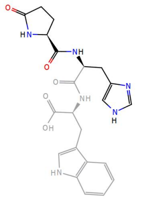	221.1033	221.1033	0.07
MATCH	175.7	249.0988	249.0982	-2.54	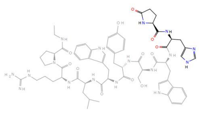	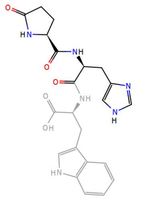	249.0982	249.0982	0.14
MATCH	15.4	641.8312	641.8276	-5.50	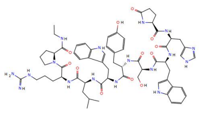	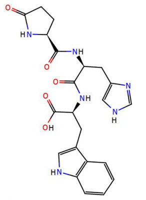	453.1880	453.1881	0.16
						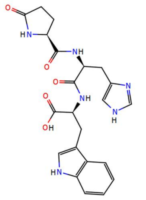	453.1880	453.1881	0.16
MATCH	17.0	1034.5597	1034.5570	-2.56	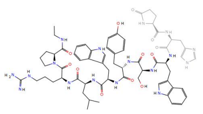	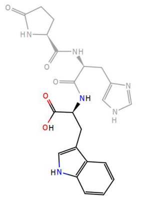	205.0969	205.0972	1.05
MATCH	16.5	1282.6515	1282.6480	-2.70	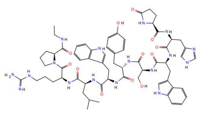	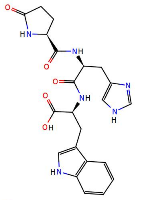	453.1880	453.1881	0.16
						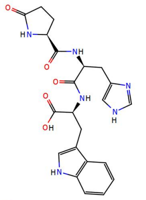	453.1880	453.1881	0.16
MET_MATCH						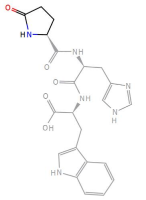	84.0451	84.0444	-8.86
MET_MATCH						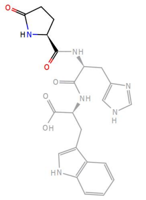	114.0554	114.0550	-3.67

Metabolite: M1 -829 RT=0.46

Type	score	sub. m/z observed	sub. m/z calculated	sub ppm		met. m/z observed	met. m/z calculated	met. ppm
MET_MATCH					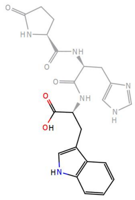	188.0705	188.0706	0.76
MET_MATCH					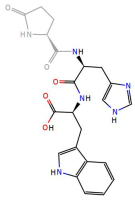	342.1564	342.1561	-1.06
MET_MATCH					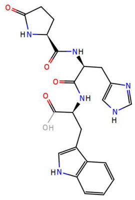	435.1765	435.1775	2.36
