# Supplementary figures and images for: Software-aided approach to investigate peptide structure and metabolic susceptibility of amide bonds in peptide drugs based on high resolution mass spectrometry
Source: PLoS One. 2017 Nov 1;12(11):e0186461. doi: 10.1371/journal.pone.0186461 (PMC5665424; doi:10.1371/journal.pone.0186461)

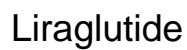

## Chromatograms

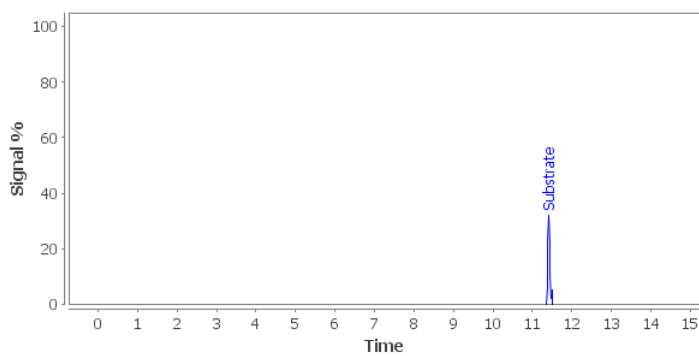

## Custom Charts

Fragmentation

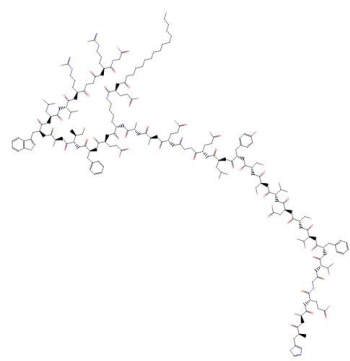

Liraglutide

Supplement: S1 File — (ZIP) [file pone.0186461.s007.zip › SFiles/S51_File.pdf]
